# Worldwide trends in underweight and obesity from 1990 to 2022: a pooled analysis of 3663 population-representative studies with 222 million children, adolescents, and adults

**DOI:** 10.1016/S0140-6736(23)02750-2

**Published:** 2024-02-29

**Authors:** NCD Risk Factor Collaboration (NCD-RisC), Pooled analysis and writing, Nowell H Phelps, Rosie K Singleton, Bin Zhou, Rachel A Heap, Anu Mishra, James E Bennett, Christopher J Paciorek, Victor PF Lhoste, Rodrigo M Carrillo-Larco, Gretchen A Stevens, Andrea Rodriguez-Martinez, Honor Bixby, James Bentham, Mariachiara Di Cesare, Goodarz Danaei, Archie W Rayner, Ana Barradas-Pires, Melanie J Cowan, Stefan Savin, Leanne M Riley, Carlos A Aguilar-Salinas, Jennifer L Baker, Amina Barkat, Zulfiqar A Bhutta, Francesco Branca, Roberta B Caixeta, Sarah Cuschieri, Farshad Farzadfar, Shubash Ganapathy, Nayu Ikeda, Violeta Iotova, Andre P Kengne, Young-Ho Khang, Avula Laxmaiah, Hsien-Ho Lin, Jun Ma, Jean Claude N Mbanya, J Jaime Miranda, Rajendra Pradeepa, Fernando Rodríguez-Artalejo, Maroje Sorić, Maria Turley, Limin Wang, Karen Webster-Kerr, Majid Ezzati, Country and regional data, Julie Aarestrup, Leandra Abarca-Gómez, Mohsen Abbasi-Kangevari, Ziad A Abdeen, Shynar Abdrakhmanova, Suhaila Abdul Ghaffar, Hanan F Abdul Rahim, Zulfiya Abdurrahmonova, Niveen M Abu-Rmeileh, Jamila Abubakar Garba, Benjamin Acosta-Cazares, Ishag Adam, Marzena Adamczyk, Robert J Adams, Seth Adu-Afarwuah, Wichai Aekplakorn, Kaosar Afsana, Shoaib Afzal, Valirie N Agbor, Imelda A Agdeppa, Javad Aghazadeh-Attari, Åsa Ågren, Hassan Aguenaou, Carlos A Aguilar-Salinas, Charles Agyemang, Mohamad Hasnan Ahmad, Noor Ani Ahmad, Ali Ahmadi, Naser Ahmadi, Nastaran Ahmadi, Imran Ahmed, Soheir H Ahmed, Wolfgang Ahrens, Gulmira Aitmurzaeva, Kamel Ajlouni, Hazzaa M Al-Hazzaa, Halima Al-Hinai, Badreya Al-Lahou, Jawad A Al-Lawati, Rajaa Al-Raddadi, Deena Al Asfoor, Huda M Al Hourani, Nawal M Al Qaoud, Monira Alarouj, Fadia AlBuhairan, Shahla AlDhukair, Maryam A Aldwairji, Sylvia Alexius, Mohamed M Ali, Anna V Alieva, Abdullah Alkandari, Ala’a Alkerwi, Buthaina M Alkhatib, Kristine Allin, Shaker A Alomary, Husam F Alomirah, Arwa M Alshangiti, Mar Alvarez-Pedrerol, Eman Aly, Deepak N Amarapurkar, Pilar Amiano Etxezarreta, John Amoah, Norbert Amougou, Philippe Amouyel, Lars Bo Andersen, Sigmund A Anderssen, Odysseas Androutsos, Lars Ängquist, Ranjit Mohan Anjana, Alireza Ansari-Moghaddam, Elena Anufrieva, Hajer Aounallah-Skhiri, Joana Araújo, Inger Ariansen, Tahir Aris, Raphael E Arku, Nimmathota Arlappa, Krishna K Aryal, Nega Assefa, Thor Aspelund, Felix K Assah, Batyrbek Assembekov, Maria Cecília F Assunção, May Soe Aung, Correia Júnior Marco Aurélio de Valois, Juha Auvinen, Mária Avdičová, Shina Avi, Kishwar Azad, Ana Azevedo, Mohsen Azimi-Nezhad, Fereidoun Azizi, Bontha V Babu, Flora Bacopoulou, Maja Bæksgaard Jørgensen, Azli Baharudin, Suhad Bahijri, Izet Bajramovic, Marta Bakacs, Jennifer L Baker, Nagalla Balakrishna, Yulia Balanova, Mohamed Bamoshmoosh, Maciej Banach, José R Banegas, Joanna Baran, Rafał Baran, Carlo M Barbagallo, Valter Barbosa Filho, Alberto Barceló, Maja Baretić, Amina Barkat, Joaquin Barnoya, Lena Barrera, Marta Barreto, Aluisio JD Barros, Mauro Virgílio Gomes Barros, Anna Bartosiewicz, Abdul Basit, Joao Luiz Bastos, Iqbal Bata, Anwar M Batieha, Aline P Batista, Rosangela L Batista, Zhamilya Battakova, Louise A Baur, Pascal M Bayauli, Robert Beaglehole, Silvia Bel-Serrat, Antonisamy Belavendra, Habiba Ben Romdhane, Theodora Benedek, Judith Benedics, Mikhail Benet, Gilda Estela Benitez Rolandi, James E Bennett, Michaela Benzeval, Elling Bere, Nicolas Berger, Ingunn Holden Bergh, Yemane Berhane, Salim Berkinbayev, Antonio Bernabe-Ortiz, Gailute Bernotiene, Ximena Berrios Carrasola, Heloísa Bettiol, Manfred E Beutel, Augustin F Beybey, Jorge Bezerra, Aroor Bhagyalaxmi, Sumit Bharadwaj, Santosh K Bhargava, Zulfiqar A Bhutta, Hongsheng Bi, Yufang Bi, Daniel Bia, Katia Biasch, Elysée Claude Bika Lele, Mukharram M Bikbov, Bihungum Bista, Dusko J Bjelica, Anne A Bjerregaard, Peter Bjerregaard, Espen Bjertness, Marius B Bjertness, Cecilia Björkelund, Katia V Bloch, Anneke Blokstra, Moran Blychfeld Magnazu, Simona Bo, Martin Bobak, Lynne M Boddy, Bernhard O Boehm, Jolanda MA Boer, Jose G Boggia, Elena Bogova, Carlos P Boissonnet, Stig E Bojesen, Marialaura Bonaccio, Vanina Bongard, Alice Bonilla-Vargas, Matthias Bopp, Herman Borghs, Steve Botomba, Rupert RA Bourne, Pascal Bovet, Khadichamo Boymatova, Lien Braeckevelt, Lutgart Braeckman, Marjolijn CE Bragt, Tasanee Braithwaite, Imperia Brajkovich, Francesco Branca, Juergen Breckenkamp, João Breda, Hermann Brenner, Lizzy M Brewster, Garry R Brian, Yajaira Briceño, Lacramioara Brinduse, Bettina Bringolf-Isler, Miguel Brito, Sinead Brophy, Johannes Brug, Graziella Bruno, Anna Bugge, Marta Buoncristiano, Genc Burazeri, Con Burns, Antonio Cabrera de León, Joseph Cacciottolo, Hui Cai, Roberta B Caixeta, Tilema Cama, Christine Cameron, José Camolas, Günay Can, Ana Paula C Cândido, Felicia Cañete, Mario V Capanzana, Naděžda Čapková, Eduardo Capuano, Rocco Capuano, Vincenzo Capuano, Marloes Cardol, Viviane C Cardoso, Axel C Carlsson, Esteban Carmuega, Rodrigo M Carrillo-Larco, Joana Carvalho, José A Casajús, Felipe F Casanueva, Maribel Casas, Ertugrul Celikcan, Laura Censi, Marvin Cervantes-Loaiza, Juraci A Cesar, Parinya Chamnan, Snehalatha Chamukuttan, Angelique Chan, Queenie Chan, Fadi J Charchar, Marie-Aline Charles, Himanshu K Chaturvedi, Nish Chaturvedi, Norsyamlina Che Abdul Rahim, Miao Li Chee, Chien-Jen Chen, Fangfang Chen, Huashuai Chen, Long-Sheng Chen, Shuohua Chen, Zhengming Chen, Ching-Yu Cheng, Yiling J Cheng, Bahman Cheraghian, Angela Chetrit, Ekaterina Chikova-Iscener, Mai JM Chinapaw, Anne Chinnock, Arnaud Chiolero, Shu-Ti Chiou, Adela Chirita-Emandi, María-Dolores Chirlaque, Belong Cho, Kaare Christensen, Diego G Christofaro, Jerzy Chudek, Renata Cifkova, Michelle Cilia, Eliza Cinteza, Massimo Cirillo, Frank Claessens, Philip Clare, Janine Clarke, Els Clays, Emmanuel Cohen, Cosmin R Cojocaru, Sandra Colorado-Yohar, Laura-María Compañ-Gabucio, Hans Concin, Susana C Confortin, Cyrus Cooper, Tara C Coppinger, Eva Corpeleijn, Lilia Yadira Cortés, Simona Costanzo, Dominique Cottel, Chris Cowell, Cora L Craig, Amelia C Crampin, Amanda J Cross, Ana B Crujeiras, Juan J Cruz, Tamás Csányi, Semánová Csilla, Alexandra M Cucu, Liufu Cui, Felipe V Cureau, Sarah Cuschieri, Ewelina Czenczek-Lewandowska, Graziella D’Arrigo, Eleonora d’Orsi, Alanna G da Silva, Liliana Dacica, Christina C Dahm, Jean Dallongeville, Albertino Damasceno, Camilla T Damsgaard, Goodarz Danaei, Rachel Dankner, Thomas M Dantoft, Parasmani Dasgupta, Saeed Dastgiri, Luc Dauchet, Kairat Davletov, Francisco de Assis Guedes de Vasconcelos, Maria Alice Altenburg de Assis, Guy De Backer, Dirk De Bacquer, Jaco De Bacquer, Jeroen de Bont, Amalia De Curtis, Patrícia de Fragas Hinnig, Giovanni de Gaetano, Stefaan De Henauw, Pilar De Miguel-Etayo, Jan-Walter De Neve, Paula Duarte de Oliveira, David De Ridder, Karin De Ridder, Susanne R de Rooij, Ana Carolina MGN de Sá, Delphine De Smedt, Mohan Deepa, Alexander D Deev, Vincent DeGennaro, Hélène Delisle, Francis Delpeuch, Stefaan Demarest, Elaine Dennison, Katarzyna Dereń, Valérie Deschamps, Ruslan D Devrishov, Meghnath Dhimal, Augusto Di Castelnuovo, Juvenal Soares Dias-da-Costa, María Elena Díaz-Sánchez, Alejandro Diaz, Pedro Díaz Fernández, María Pilar Díez Ripollés, Zivka Dika, Shirin Djalalinia, Visnja Djordjic, Ha TP Do, Annette J Dobson, Liria Dominguez, Maria Benedetta Donati, Chiara Donfrancesco, Guanghui Dong, Yanhui Dong, Silvana P Donoso, Angela Döring, Maria Dorobantu, Ahmad Reza Dorosty, Marcus Dörr, Kouamelan Doua, Nico Dragano, Wojciech Drygas, Shufa Du, Jia Li Duan, Charmaine A Duante, Priscilla Duboz, Vesselka L Duleva, Virginija Dulskiene, Samuel C Dumith, Anar Dushpanova, Terence Dwyer, Azhar Dyussupova, Vilnis Dzerve, Elzbieta Dziankowska-Zaborszczyk, Narges Ebrahimi, Guadalupe Echeverría, Ricky Eddie, Ebrahim Eftekhar, Vasiliki Efthymiou, Eruke E Egbagbe, Robert Eggertsen, Sareh Eghtesad, Gabriele Eiben, Ulf Ekelund, Mohammad El-Khateeb, Laila El Ammari, Jalila El Ati, Denise Eldemire-Shearer, Paul Elliott, Ofem Enang, Ronit Endevelt, Reina Engle-Stone, Rajiv T Erasmus, Cihangir Erem, Gul Ergor, Louise Eriksen, Johan G Eriksson, Jorge Escobedo-de la Peña, Saeid Eslami, Ali Esmaeili, Alun Evans, Roger G Evans, David Faeh, Guy Fagherazzi, Ildar Fakhradiyev, Albina A Fakhretdinova, Caroline H Fall, Elnaz Faramarzi, Mojtaba Farjam, Victoria Farrugia Sant’Angelo, Farshad Farzadfar, Yosef Farzi, Mohammad Reza Fattahi, Asher Fawwad, Wafaie W Fawzi, Francisco J Felix-Redondo, Trevor S Ferguson, Romulo A Fernandes, Daniel Fernández-Bergés, Daniel Ferrante, Thomas Ferrao, Gerson Ferrari, Marika Ferrari, Marco M Ferrario, Catterina Ferreccio, Haroldo S Ferreira, Eldridge Ferrer, Jean Ferrieres, Thamara Hubler Figueiró, Anna Fijalkowska, Günther Fink, Mauro Fisberg, Krista Fischer, Leng Huat Foo, Maria Forsner, Edward F Fottrell, Heba M Fouad, Damian K Francis, Maria do Carmo Franco, Zlatko Fras, Brooklyn Fraser, Guillermo Frontera, Flavio D Fuchs, Sandra C Fuchs, Isti I Fujiati, Yuki Fujita, Matsuda Fumihiko, Viktoriya Furdela, Takuro Furusawa, Stefan Adela Gabriela, Zbigniew Gaciong, Mihai Gafencu, Manuel Galán Cuesta, Andrzej Galbarczyk, Sonya V Galcheva, Henrike Galenkamp, Daniela Galeone, Myriam Galfo, Fabio Galvano, Jingli Gao, Pei Gao, Manoli Garcia-de-la-Hera, María José García Mérida, Marta García Solano, Dickman Gareta, Sarah P Garnett, Jean-Michel Gaspoz, Magda Gasull, Adroaldo Cesar Araujo Gaya, Anelise Reis Gaya, Andrea Gazzinelli, Ulrike Gehring, Harald Geiger, Johanna M Geleijnse, Ronnie George, Eva Gerdts, Ebrahim Ghaderi, Seyyed-Hadi Ghamari, Ali Ghanbari, Erfan Ghasemi, Oana-Florentina Gheorghe-Fronea, Alessandro Gialluisi, Simona Giampaoli, Francesco Gianfagna, Christian Gieger, Tiffany K Gill, Jonathan Giovannelli, Glen Gironella, Aleksander Giwercman, Konstantinos Gkiouras, Natalya Glushkova, Ramesh Godara, Justyna Godos, Sibel Gogen, Marcel Goldberg, David Goltzman, Georgina Gómez, Jesús Humberto Gómez Gómez, Luis F Gomez, Santiago F Gómez, Aleksandra Gomula, Bruna Gonçalves Cordeiro da Silva, Helen Gonçalves, Mauer Gonçalves, Ana D González-Alvarez, David A Gonzalez-Chica, Esther M González-Gil, Marcela Gonzalez-Gross, Margot González-Leon, Juan P González-Rivas, Clicerio González-Villalpando, María-Elena González-Villalpando, Angel R Gonzalez, Frederic Gottrand, Antonio Pedro Graça, Dušan Grafnetter, Aneta Grajda, Maria G Grammatikopoulou, Edward W Gregg, Ronald D Gregor, Maria João Gregório, Else Karin Grøholt, Anders Grøntved, Giuseppe Grosso, Gabriella Gruden, Dongfeng Gu, Viviana Guajardo, Emanuela Gualdi-Russo, Pilar Guallar-Castillón, Andrea Gualtieri, Elias F Gudmundsson, Vilmundur Gudnason, Maëlenn Guerchet, Ramiro Guerrero, Idris Guessous, Andre L Guimaraes, Unjali P Gujral, Martin C Gulliford, Johanna Gunnlaugsdottir, Marc J Gunter, Xiu-Hua Guo, Yin Guo, Prakash C Gupta, Rajeev Gupta, Oye Gureje, Mirjana A Gurinović, Enrique Gutiérrez González, Laura Gutierrez, Felix Gutzwiller, Xinyi Gwee, Seongjun Ha, Farzad Hadaegh, Charalambos A Hadjigeorgiou, Rosa Haghshenas, Hamid Hakimi, Jytte Halkjær, Ian R Hambleton, Behrooz Hamzeh, Willem A Hanekom, Dominique Hange, Abu AM Hanif, Sari Hantunen, Jie Hao, Carla Menêses Hardman, Louise Hardy, Rachakulla Hari Kumar, Tina Harmer Lassen, Javad Harooni, Seyed Mohammad Hashemi-Shahri, Maria Hassapidou, Jun Hata, Teresa Haugsgjerd, Alison J Hayes, Jiang He, Yuan He, Yuna He, Regina Heidinger-Felső, Margit Heier, Mirjam Heinen, Tatjana Hejgaard, Marleen Elisabeth Hendriks, Rafael dos Santos Henrique, Ana Henriques, Leticia Hernandez Cadena, Sauli Herrala, Marianella Herrera-Cuenca, Victor M Herrera, Isabelle Herter-Aeberli, Karl-Heinz Herzig, Ramin Heshmat, Barbara Heude, Allan G Hill, Sai Yin Ho, Suzanne C Ho, Michael Hobbs, Doroteia A Höfelmann, Michelle Holdsworth, Reza Homayounfar, Clara Homs, Emiel Hoogendijk, Wilma M Hopman, Andrea RVR Horimoto, Claudia M Hormiga, Bernardo L Horta, Leila Houti, Christina Howitt, Thein Thein Htay, Aung Soe Htet, Maung Maung Than Htike, Yonghua Hu, José María Huerta, Ilpo Tapani Huhtaniemi, Laetitia Huiart, Constanta Huidumac Petrescu, Abdullatif Husseini, Chinh Nguyen Huu, Inge Huybrechts, Nahla Hwalla, Jolanda Hyska, Licia Iacoviello, Ellina M Iakupova, Jesús Ibarluzea, Mohsen M Ibrahim, Norazizah Ibrahim Wong, Jannicke Igland, Chinwuba Ijoma, Nayu Ikeda, M Arfan Ikram, Carmen Iñiguez, Violeta Iotova, Vilma E Irazola, Takafumi Ishida, Godsent C Isiguzo, Muhammad Islam, Sheikh Mohammed Shariful Islam, Duygu Islek, Till Ittermann, Ivaila Y Ivanova-Pandourska, Masanori Iwasaki, Tuija Jääskeläinen, Rod T Jackson, Jeremy M Jacobs, Michel Jadoul, Tazeen Jafar, Bakary Jallow, Kenneth James, Kazi M Jamil, Konrad Jamrozik, Nataša Jan, Anna Jansson, Imre Janszky, Edward Janus, Juel Jarani, Gerald Jarnig, Marjo-Riitta Jarvelin, Grazyna Jasienska, Ana Jelaković, Bojan Jelaković, Garry Jennings, Chao Qiang Jiang, Ramon O Jimenez, Karl-Heinz Jöckel, Michel Joffres, Jari J Jokelainen, Jost B Jonas, Jitendra Jonnagaddala, Lars Jøran Kjerpeseth, Torben Jørgensen, Pradeep Joshi, Rohina Joshi, Josipa Josipović, Farahnaz Joukar, Jacek J Jóźwiak, Debra S Judge, Anne Juolevi, Gregor Jurak, Iulia Jurca Simina, Vesna Juresa, Rudolf Kaaks, Felix O Kaducu, Agnes L Kadvan, Anthony Kafatos, Mónika Kaj, Eero O Kajantie, Natia Kakutia, Daniela Kállayová, Zhanna Kalmatayeva, Ofra Kalter-Leibovici, Yves Kameli, Kodanda R Kanala, Srinivasan Kannan, Efthymios Kapantais, Eva Karaglani, Argyro Karakosta, Line L Kårhus, Khem B Karki, Omat Karlsson, Adoubi Kassi Anicet, Philippe B Katchunga, Marzieh Katibeh, Joanne Katz, Peter T Katzmarzyk, Jussi Kauhanen, Prabhdeep Kaur, Maryam Kavousi, Gyulli M Kazakbaeva, François F Kaze, Benson M Kazembe, Calvin Ke, Ulrich Keil, Lital Keinan Boker, Sirkka Keinänen-Kiukaanniemi, Roya Kelishadi, Cecily Kelleher, Han CG Kemper, Andre P Kengne, Maryam Keramati, Alina Kerimkulova, Mathilde Kersting, Timothy Key, Yousef Saleh Khader, Arsalan Khaledifar, Davood Khalili, Young-Ho Khang, Bahareh Kheiri, Motahareh Kheradmand, Alireza Khosravi, Ilse MSL Khouw, Ursula Kiechl-Kohlendorfer, Sophia J Kiechl, Stefan Kiechl, Japhet Killewo, Hyeon Chang Kim, Jeongseon Kim, Jenny M Kindblom, Andrew Kingston, Heidi Klakk, Suntara Klanarong, Jana Klanova, Magdalena Klimek, Jeannette Klimont, Jurate Klumbiene, Michael Knoflach, Susanne Kobel, Bhawesh Koirala, Elin Kolle, Sanda M Kolo, Patrick Kolsteren, Jürgen König, Raija Korpelainen, Paul Korrovits, Magdalena Korzycka, Jelena Kos, Seppo Koskinen, Katsuyasu Kouda, Malik Koussoh Simone, Éva Kovács, Viktoria Anna Kovacs, Irina Kovalskys, Sudhir Kowlessur, Slawomir Koziel, Jana Kratenova, Wolfgang Kratzer, Vilma Kriaucioniene, Susi Kriemler, Peter Lund Kristensen, Helena Krizan, Maria F Kroker-Lobos, Steinar Krokstad, Daan Kromhout, Herculina S Kruger, Ruan Kruger, Łukasz Kryst, Ruzena Kubinova, Renata Kuciene, Urho M Kujala, Enisa Kujundzic, Zbigniew Kulaga, Mukhtar Kulimbet, Vaitheeswaran Kulothungan, R Krishna Kumar, Meena Kumari, Marie Kunešová, Pawel Kurjata, Yadlapalli S Kusuma, Vladimir Kutsenko, Kari Kuulasmaa, Catherine Kyobutungi, Quang Ngoc La, Fatima Zahra Laamiri, Tiina Laatikainen, Demetre Labadarios, Carl Lachat, Karl J Lackner, Daphne Lai, Youcef Laid, Lachmie Lall, Tai Hing Lam, Maritza Landaeta Jimenez, Edwige Landais, Tiina Lankila, Vera Lanska, Georg Lappas, Bagher Larijani, Simo Pone Larissa, Mina P Lateva, Tint Swe Latt, Martino Laurenzi, Laura Lauria, Avula Laxmaiah, Maria Lazo-Porras, Gwenaëlle Le Coroller, Khanh Le Nguyen Bao, Agnès Le Port, Tuyen D Le, Jeannette Lee, Jeonghee Lee, Paul H Lee, Terho Lehtimäki, Daniel Lemogoum, Elvynna Leong, Branimir Leskošek, Justyna Leszczak, Katja B Leth-Møller, Gabriel M Leung, Naomi S Levitt, Yanping Li, Merike Liivak, Christa L Lilly, Charlie Lim, Wei-Yen Lim, M Fernanda Lima-Costa, Hsien-Ho Lin, Xu Lin, Lars Lind, Vijaya Lingam, Birgit Linkohr, Allan Linneberg, Lauren Lissner, Mieczyslaw Litwin, Jing Liu, Lijuan Liu, Liping Liu, Xiaotian Liu, Wei-Cheng Lo, Helle-Mai Loit, Khuong Quynh Long, Guadalupe Longo Abril, Luis Lopes, Marcus SS Lopes, Oscar Lopes, Esther Lopez-Garcia, Tania Lopez, Paulo A Lotufo, José Eugenio Lozano, Janice L Lukrafka, Dalia Luksiene, Annamari Lundqvist, Nuno Lunet, Charles Lunogelo, Michala Lustigová, Edyta Łuszczki, Jean-René M’Buyamba-Kabangu, Guansheng Ma, Jun Ma, Xu Ma, George LL Machado-Coelho, Aristides M Machado-Rodrigues, Enguerran Macia, Luisa M Macieira, Ahmed A Madar, Sherilynn Madraisau, Anja L Madsen, Gladys E Maestre, Stefania Maggi, Dianna J Magliano, Sara Magnacca, Emmanuella Magriplis, Gowri Mahasampath, Bernard Maire, Marjeta Majer, Marcia Makdisse, Päivi Mäki, Mohammad-Reza Malekpour, Fatemeh Malekzadeh, Reza Malekzadeh, Rahul Malhotra, Kodavanti Mallikharjuna Rao, Deborah C Malta, Sofia K Malyutina, Lynell V Maniego, Yannis Manios, Jim I Mann, Masimango Imani Mannix, Fariborz Mansour-Ghanaei, Taru Manyanga, Enzo Manzato, Mala Ali Mapatano, Anie Marcil, Paula Margozzini, Rosu Maria-Magdalena, Joany Mariño, Anastasia Markaki, Oonagh Markey, Eliza Markidou Ioannidou, Pedro Marques-Vidal, Larissa Pruner Marques, Jaume Marrugat, Yves Martin-Prevel, Rosemarie Martin, Reynaldo Martorell, Eva Martos, Fatai A Maruf, Katharina Maruszczak, Stefano Marventano, Giovanna Masala, Luis P Mascarenhas, Masoud Masinaei, Shariq R Masoodi, Ellisiv B Mathiesen, Prashant Mathur, Alicia Matijasevich, Piotr Matłosz, Tandi E Matsha, Victor Matsudo, Giletta Matteo, Pallab K Maulik, Christina Mavrogianni, Artur Mazur, Jean Claude N Mbanya, Shelly R McFarlane, Stephen T McGarvey, Martin McKee, Rachael M McLean, Scott B McLean, Margaret L McNairy, Breige A McNulty, Sounnia Mediene Benchekor, Jurate Medzioniene, Kirsten Mehlig, Amir Houshang Mehrparvar, Aline Meirhaeghe, Jørgen Meisfjord, Christa Meisinger, Jesus D Melgarejo, Marina Melkumova, Júlio Mello, Fabián Méndez, Carlos O Mendivil, Ana Maria B Menezes, Geetha R Menon, Gert BM Mensink, Maria Teresa Menzano, Indrapal I Meshram, Diane T Meto, Haakon E Meyer, Jie Mi, Kim F Michaelsen, Nathalie Michels, Kairit Mikkel, Karolina Miłkowska, Jody C Miller, Olga Milushkina, Cláudia S Minderico, GK Mini, Juan Francisco Miquel, J Jaime Miranda, Mohammad Reza Mirjalili, Daphne Mirkopoulou, Erkin Mirrakhimov, Marjeta Mišigoj-Duraković, Antonio Mistretta, Veronica Mocanu, Pietro A Modesti, Sahar Saeedi Moghaddam, Shukri F Mohamed, Kazem Mohammad, Mohammad Reza Mohammadi, Zahra Mohammadi, Noushin Mohammadifard, Reza Mohammadpourhodki, Viswanathan Mohan, Salim Mohanna, Muhammad Fadhli Mohd Yusoff, Iraj Mohebbi, Marie Moitry, Line T Møllehave, Niels C Møller, Dénes Molnár, Amirabbas Momenan, Charles K Mondo, Michele Monroy-Valle, Roger A Montenegro Mendoza, Eric Monterrubio-Flores, Kotsedi Daniel K Monyeki, Jin Soo Moon, Mahmood Moosazadeh, Hermine T Mopa, Farhad Moradpour, Leila B Moreira, Alain Morejon, Luis A Moreno, Francis Morey, Karen Morgan, Suzanne N Morin, Erik Lykke Mortensen, George Moschonis, Alireza Moslem, Mildrey Mosquera, Malgorzata Mossakowska, Aya Mostafa, Seyed-Ali Mostafavi, Anabela Mota-Pinto, Jorge Mota, Mohammad Esmaeel Motlagh, Jorge Motta, Marcos André Moura-dos-Santos, Yeva Movsesyan, Malay K Mridha, Kelias P Msyamboza, Thet Thet Mu, Magdalena Muc, Florian Muca, Boban Mugoša, Maria L Muiesan, Martina Müller-Nurasyid, Thomas Münzel, Jaakko Mursu, Elaine M Murtagh, Kamarul Imran Musa, Sanja Musić Milanović, Vera Musil, Geofrey Musinguzi, Muel Telo Muyer, Iraj Nabipour, Gabriele Nagel, Farid Najafi, Harunobu Nakamura, Hanna Nalecz, Jana Námešná, Ei Ei K Nang, Vinay B Nangia, Martin Nankap, Sameer Narake, KM Venkat Narayan, Paola Nardone, Take Naseri, Michels Nathalie, William A Neal, Nareemarn Neelapaichit, Azim Nejatizadeh, Chandini Nekkantti, Keiu Nelis, Ilona Nenko, Martin Neovius, Flavio Nervi, Tze Pin Ng, Chung T Nguyen, Nguyen D Nguyen, Quang Ngoc Nguyen, Michael Y Ni, Rodica Nicolescu, Peng Nie, Ramfis E Nieto-Martínez, Yury P Nikitin, Guang Ning, Toshiharu Ninomiya, Nobuo Nishi, Sania Nishtar, Marianna Noale, Oscar A Noboa, Helena Nogueira, Maria Nordendahl, Børge G Nordestgaard, Kevin I Norton, Davide Noto, Natalia Nowak-Szczepanska, Mohannad Al Nsour, Irfan Nuhoğlu, Baltazar Nunes, Eha Nurk, Fred Nuwaha, Moffat Nyirenda, Terence W O’Neill, Dermot O’Reilly, Galina Obreja, Caleb Ochimana, Angélica M Ochoa-Avilés, Eiji Oda, Augustine N Odili, Kyungwon Oh, Kumiko Ohara, Claes Ohlsson, Ryutaro Ohtsuka, Örn Olafsson, Brian Oldenburg, Maria Teresa A Olinto, Isabel O Oliveira, Mohd Azahadi Omar, Saeed M Omar, Altan Onat, Sok King Ong, N Charlotte Onland-Moret, Lariane M Ono, Obinna Onodugo, Pedro Ordunez, Rui Ornelas, Ana P Ortiz, Pedro J Ortiz, Merete Osler, Clive Osmond, Sergej M Ostojic, Afshin Ostovar, Johanna A Otero, Charlotte B Ottendahl, Akaninyene Otu, Kim Overvad, Ellis Owusu-Dabo, Adetoyeje Y Oyeyemi, Adewale L Oyeyemi, Fred Michel Paccaud, Cristina P Padez, Ioannis Pagkalos, Elena Pahomova, Karina Mary de Paiva, Andrzej Pająk, Natalja Pajula, Alberto Palloni, Luigi Palmieri, Wen-Harn Pan, Songhomitra Panda-Jonas, Arvind Pandey, Zengchang Pang, Francesco Panza, Mariela Paoli, Sousana K Papadopoulou, Dimitrios Papandreou, Rossina G Pareja, Soon-Woo Park, Suyeon Park, Winsome R Parnell, Mahboubeh Parsaeian, Ionela M Pascanu, Patrick Pasquet, Nikhil D Patel, Marcos Pattussi, Halyna Pavlyshyn, Raimund Pechlaner, Ivan Pećin, Mangesh S Pednekar, João M Pedro, Nasheeta Peer, Sergio Viana Peixoto, Markku Peltonen, Alexandre C Pereira, Marco A Peres, Agustín Perez-Londoño, Cynthia M Pérez, Valentina Peterkova, Annette Peters, Janina Petkeviciene, Ausra Petrauskiene, Olga Petrovna Kovtun, Emanuela Pettenuzzo, Niloofar Peykari, Norbert Pfeiffer, Modou Cheyassin Phall, Son Thai Pham, Felix P Phiri, Rafael N Pichardo, Daniela Pierannunzio, Preux Pierre-Marie, Iris Pigeot, Hynek Pikhart, Aida Pilav, Pavel Piler, Lorenza Pilotto, Francesco Pistelli, Freda Pitakaka, Aleksandra Piwonska, Andreia N Pizarro, Pedro Plans-Rubió, Alina G Platonova, Bee Koon Poh, Hermann Pohlabeln, Nadija S Polka, Raluca M Pop, Barry M Popkin, Stevo R Popovic, Miquel Porta, Georg Posch, Anil Poudyal, Dimitrios Poulimeneas, Hamed Pouraram, Farhad Pourfarzi, Akram Pourshams, Hossein Poustchi, Rajendra Pradeepa, Alison J Price, Jacqueline F Price, Antonio Prista, Rui Providencia, Jardena J Puder, Iveta Pudule, Solie Puhakka, Maria Puiu, Margus Punab, Muhammed S Qadir, Radwan F Qasrawi, Qing Qiao, Mostafa Qorbani, Hedley K Quintana, Pedro J Quiroga-Padilla, Tran Quoc Bao, Stefan Rach, Ivana Radic, Ricardas Radisauskas, Salar Rahimikazerooni, Mahfuzar Rahman, Mahmudur Rahman, Olli Raitakari, Manu Raj, Tamerlan Rajabov, Sherali Rakhmatulloev, Ivo Rakovac, Sudha Ramachandra Rao, Ambady Ramachandran, Otim PC Ramadan, Virgílio V Ramires, Manuel Ramirez-Zea, Jacqueline Ramke, Elisabete Ramos, Rafel Ramos, Lekhraj Rampal, Sanjay Rampal, Sheena E Ramsay, Lalka S Rangelova, Vayia Rarra, Ramon A Rascon-Pacheco, Mohammad-Mahdi Rashidi, Cassiano Ricardo Rech, Josep Redon, Paul Ferdinand M Reganit, Valéria Regecová, Jane DP Renner, Judit A Repasy, Cézane P Reuter, Luis Revilla, Andrew Reynolds, Negar Rezaei, Abbas Rezaianzadeh, Yeunsook Rho, Lourdes Ribas-Barba, Robespierre Ribeiro, Elio Riboli, Fernando Rigo, Attilio Rigotti, Natascia Rinaldo, Tobias F Rinke de Wit, Ulf Risérus, Ana I Rito, Raphael M Ritti-Dias, Juan A Rivera, Reina G Roa, Louise Robinson, Romana Roccaldo, Daniela Rodrigues, Fernando Rodríguez-Artalejo, María del Cristo Rodriguez-Perez, Laura A Rodríguez-Villamizar, Andrea Y Rodríguez, Ulla Roggenbuck, Peter Rohloff, Fabian Rohner, Rosalba Rojas-Martinez, Nipa Rojroongwasinkul, Dora Romaguera, Elisabetta L Romeo, Rafaela V Rosario, Annika Rosengren, Ian Rouse, Vanessa Rouzier, Joel GR Roy, Maira H Ruano, Adolfo Rubinstein, Frank J Rühli, Jean-Bernard Ruidavets, Blanca Sandra Ruiz-Betancourt, Maria Ruiz-Castell, Emma Ruiz Moreno, Iuliia A Rusakova, Wojciech Rusek, Kenisha Russell Jonsson, Paola Russo, Petra Rust, Marcin Rutkowski, Marge Saamel, Crizian G Saar, Charumathi Sabanayagam, Hamideh Sabbaghi, Elena Sacchini, Harshpal S Sachdev, Alireza Sadjadi, Ali Reza Safarpour, Sare Safi, Saeid Safiri, Mohammad Hossien Saghi, Olfa Saidi, Calogero Saieva, Satoko Sakata, Nader Saki, Sanja Šalaj, Benoit Salanave, Eduardo Salazar Martinez, Akkumis Salhanova, Diego Salmerón, Veikko Salomaa, Jukka T Salonen, Massimo Salvetti, Margarita Samoutian, Jose Sánchez-Abanto, Inés Sánchez Rodríguez, Sandjaja, Susana Sans, Loreto Santa-Marina, Ethel Santacruz, Diana A Santos, Ina S Santos, Lèlita C Santos, Maria Paula Santos, Osvaldo Santos, Rute Santos, Tamara R Santos, Jouko L Saramies, Luis B Sardinha, Nizal Sarrafzadegan, Thirunavukkarasu Sathish, Kai-Uwe Saum, Savvas Savva, Mathilde Savy, Norie Sawada, Mariana Sbaraini, Marcia Scazufca, Beatriz D Schaan, Angelika Schaffrath Rosario, Herman Schargrodsky, Anja Schienkiewitz, Karin Schindler, Sabine Schipf, Börge Schmidt, Carsten O Schmidt, Ida Maria Schmidt, Andrea Schneider, Peter Schnohr, Ben Schöttker, Sara Schramm, Stine Schramm, Helmut Schröder, Constance Schultsz, Gry Schultz, Matthias B Schulze, Aletta E Schutte, Sylvain Sebert, Moslem Sedaghattalab, Rusidah Selamat, Vedrana Sember, Abhijit Sen, Idowu O Senbanjo, Sadaf G Sepanlou, Guillermo Sequera, Luis Serra-Majem, Jennifer Servais, Ľudmila Ševčíková, Ronel Sewpaul, Svetlana Shalnova, Teresa Shamah-Levy, Seyed Morteza Shamshirgaran, Coimbatore Subramaniam Shanthirani, Maryam Sharafkhah, Sanjib K Sharma, Almaz Sharman, Jonathan E Shaw, Amaneh Shayanrad, Ali Akbar Shayesteh, Lela Shengelia, Zumin Shi, Kenji Shibuya, Hana Shimizu-Furusawa, Tal Shimony, Rahman Shiri, Namuna Shrestha, Khairil Si-Ramlee, Alfonso Siani, Rosalynn Siantar, Abla M Sibai, Labros S Sidossis, Natalia Silitrari, Antonio M Silva, Caroline Ramos de Moura Silva, Diego Augusto Santos Silva, Kelly Samara Silva, Xueling Sim, Mary Simon, Judith Simons, Leon A Simons, Agneta Sjöberg, Michael Sjöström, Elena V Skoblina, Natalia A Skoblina, Tatyana Slazhnyova, Jolanta Slowikowska-Hilczer, Przemysław Slusarczyk, Liam Smeeth, Hung-Kwan So, Fernanda Cunha Soares, Grzegorz Sobek, Eugène Sobngwi, Morten Sodemann, Stefan Söderberg, Moesijanti YE Soekatri, Agustinus Soemantri, Reecha Sofat, Vincenzo Solfrizzi, Yuliya V Solovieva, Mohammad Hossein Somi, Emily Sonestedt, Yi Song, Sajid Soofi, Thorkild IA Sørensen, Elin P Sørgjerd, Maroje Sorić, Charles Sossa Jérome, Victoria E Soto-Rojas, Aïcha Soumaré, Alfonso Sousa-Poza, Slavica Sovic, Bente Sparboe-Nilsen, Karen Sparrenberger, Phoebe R Spencer, Angela Spinelli, Igor Spiroski, Jan A Staessen, Hanspeter Stamm, Andreas Stang, Gregor Starc, Kaspar Staub, Bill Stavreski, Jostein Steene-Johannessen, Peter Stehle, Aryeh D Stein, Silje Steinsbekk, George S Stergiou, Jochanan Stessman, Ranko Stevanović, Jutta Stieber, Doris Stöckl, Jakub Stokwiszewski, Ekaterina Stoyanova, Gareth Stratton, Karien Stronks, Maria Wany Strufaldi, Lela Sturua, Ramón Suárez-Medina, Milton F Suarez-Ortegón, Phalakorn Suebsamran, Mindy Sugiyama, Machi Suka, Gerhard Sulo, Chien-An Sun, Liang Sun, Malin Sund, Johan Sundström, Yn-Tz Sung, Jordi Sunyer, Paibul Suriyawongpaisal, Nabil William G Sweis, Boyd A Swinburn, Rody G Sy, René Charles Sylva, Lucjan Szponar, Lorraine Tabone, E Shyong Tai, Furusawa Takuro, Konstantinos D Tambalis, Mari-Liis Tammesoo, Abdonas Tamosiunas, Eng Joo Tan, Xun Tang, Maya Tanrygulyyeva, Frank Tanser, Yong Tao, Mohammed Rasoul Tarawneh, Jakob Tarp, Carolina B Tarqui-Mamani, Radka Taxová Braunerová, Anne Taylor, Julie Taylor, Félicité Tchibindat, Saskia Te Velde, William R Tebar, Grethe S Tell, Tania Tello, Masresha Tessema, Yih Chung Tham, KR Thankappan, Holger Theobald, Xenophon Theodoridis, Nihal Thomas, Barbara Thorand, Amanda G Thrift, Ľubica Tichá, Erik J Timmermans, Dwi Hapsari Tjandrarini, Anne Tjonneland, Hanna K Tolonen, Janne S Tolstrup, Maciej Tomaszewski, Murat Topbas, Roman Topór-Mądry, Liv Elin Torheim, Michael J Tornaritis, Maties Torrent, Laura Torres-Collado, Stefania Toselli, Giota Touloumi, Pierre Traissac, Thi Tuyet-Hanh Tran, Mark S Tremblay, Areti Triantafyllou, Dimitrios Trichopoulos, Antonia Trichopoulou, Oanh TH Trinh, Atul Trivedi, Lechaba Tshepo, Maria Tsigga, Panagiotis Tsintavis, Shoichiro Tsugane, John Tuitele, Azaliia M Tuliakova, Marshall K Tulloch-Reid, Fikru Tullu, Tomi-Pekka Tuomainen, Jaakko Tuomilehto, Maria L Turley, Gilad Twig, Per Tynelius, Evangelia Tzala, Themistoklis Tzotzas, Christophe Tzourio, Nwannedimma Udoji, Peter Ueda, Eunice Ugel, Flora AM Ukoli, Hanno Ulmer, Belgin Unal, Zhamyila Usupova, Hannu MT Uusitalo, Nalan Uysal, Justina Vaitkeviciute, Gonzalo Valdivia, Susana Vale, Damaskini Valvi, Rob M van Dam, Bert-Jan van den Born, Johan Van der Heyden, Yvonne T van der Schouw, Koen Van Herck, Wendy Van Lippevelde, Hoang Van Minh, Natasja M Van Schoor, Irene GM van Valkengoed, Dirk Vanderschueren, Diego Vanuzzo, Anette Varbo, Gregorio Varela-Moreiras, Luz Nayibe Vargas, Patricia Varona-Pérez, Senthil K Vasan, Daniel G Vasques, Radu Vatasescu, Tomas Vega, Toomas Veidebaum, Gustavo Velasquez-Melendez, Biruta Velika, Maïté Verloigne, Giovanni Veronesi, WM Monique Verschuren, Cesar G Victora, Giovanni Viegi, Lucie Viet, Frøydis N Vik, Monica Vilar, Salvador Villalpando, Jesus Vioque, Napaphan Viriyautsahakul, Jyrki K Virtanen, Marjolein Visser, Sophie Visvikis-Siest, Bharathi Viswanathan, Mihaela Vladulescu, Tiina Vlasoff, Dorja Vocanec, Peter Vollenweider, Henry Völzke, Georgia Vourli, Ari Voutilainen, Martine Vrijheid, Tanja GM Vrijkotte, Silvije Vuletić, Alisha N Wade, Thomas Waldhör, Janette Walton, Elvis OA Wambiya, Wan Mohamad Wan Bebakar, Wan Nazaimoon Wan Mohamud, Rildo de Souza Wanderley, Chongjian Wang, Huijun Wang, Limin Wang, Ming-Dong Wang, Ningli Wang, Qian Wang, Xiangjun Wang, Ya Xing Wang, Ying-Wei Wang, S Goya Wannamethee, Nicholas Wareham, Olivia Wartha, Adelheid Weber, Karen Webster-Kerr, Niels Wedderkopp, Daniel Weghuber, Wenbin Wei, Aneta Weres, Bo Werner, Leo D Westbury, Peter H Whincup, Lars Wichstrøm, Kremlin Wickramasinghe, Kurt Widhalm, Indah S Widyahening, Andrzej Więcek, Philipp S Wild, Rainford J Wilks, Johann Willeit, Peter Willeit, Julianne Williams, Tom Wilsgaard, James P Wirth, Bogdan Wojtyniak, Meseret Woldeyohannes, Kathrin Wolf, Roy A Wong-McClure, Andrew Wong, Emily B Wong, Jyh Eiin Wong, Tien Yin Wong, Jean Woo, Mark Woodward, Frederick C Wu, Hon-Yen Wu, Jianfeng Wu, Li Juan Wu, Shouling Wu, Justyna Wyszyńska, Haiquan Xu, Liang Xu, Nor Azwany Yaacob, Uruwan Yamborisut, Li Yan, Weili Yan, Ling Yang, Xiaoguang Yang, Yang Yang, Nazan Yardim, Tabara Yasuharu, Martha Yépez García, Panayiotis K Yiallouros, Agneta Yngve, Moein Yoosefi, Akihiro Yoshihara, Yoto Yotov, Qi Sheng You, San-Lin You, Novie O Younger-Coleman, Yu-Ling Yu, Yunjiang Yu, Safiah Md Yusof, Ahmad Faudzi Yusoff, Luciana Zaccagni, Vassilis Zafiropulos, Ahmad A Zainuddin, Seyed Rasoul Zakavi, Farhad Zamani, Sabina Zambon, Antonis Zampelas, Hana Zamrazilová, Maria Elisa Zapata, Abdul Hamid Zargar, Ko Ko Zaw, Ayman A Zayed, Tomasz Zdrojewski, Magdalena Żegleń, Kristyna Zejglicova, Tajana Zeljkovic Vrkic, Yi Zeng, Andrea Zentai, Bing Zhang, Luxia Zhang, Zhen-Yu Zhang, Dong Zhao, Ming-Hui Zhao, Wenhua Zhao, Yanitsa V Zhecheva, Shiqi Zhen, Wei Zheng, Yingfeng Zheng, Bekbolat Zholdin, Maigeng Zhou, Dan Zhu, Paul Zimmet, Marie Zins, Emanuel Zitt, Yanina Zocalo, Nada Zoghlami, Julio Zuñiga Cisneros, Monika Zuziak

## Abstract

**Background:**

Underweight and obesity are associated with adverse health outcomes throughout the life course. We estimated the individual and combined prevalence of underweight or thinness and obesity, and their changes, from 1990 to 2022 for adults and school-aged children and adolescents in 200 countries and territories.

**Methods:**

We used data from 3663 population-based studies with 222 million participants that measured height and weight in representative samples of the general population. We used a Bayesian hierarchical model to estimate trends in the prevalence of different BMI categories, separately for adults (age ≥20 years) and school-aged children and adolescents (age 5–19 years), from 1990 to 2022 for 200 countries and territories. For adults, we report the individual and combined prevalence of underweight (BMI <18·5 kg/m^2^) and obesity (BMI ≥30 kg/m^2^). For school-aged children and adolescents, we report thinness (BMI <2 SD below the median of the WHO growth reference) and obesity (BMI >2 SD above the median).

**Findings:**

From 1990 to 2022, the combined prevalence of underweight and obesity in adults decreased in 11 countries (6%) for women and 17 (9%) for men with a posterior probability of at least 0·80 that the observed changes were true decreases. The combined prevalence increased in 162 countries (81%) for women and 140 countries (70%) for men with a posterior probability of at least 0·80. In 2022, the combined prevalence of underweight and obesity was highest in island nations in the Caribbean and Polynesia and Micronesia, and countries in the Middle East and north Africa. Obesity prevalence was higher than underweight with posterior probability of at least 0·80 in 177 countries (89%) for women and 145 (73%) for men in 2022, whereas the converse was true in 16 countries (8%) for women, and 39 (20%) for men. From 1990 to 2022, the combined prevalence of thinness and obesity decreased among girls in five countries (3%) and among boys in 15 countries (8%) with a posterior probability of at least 0·80, and increased among girls in 140 countries (70%) and boys in 137 countries (69%) with a posterior probability of at least 0·80. The countries with highest combined prevalence of thinness and obesity in school-aged children and adolescents in 2022 were in Polynesia and Micronesia and the Caribbean for both sexes, and Chile and Qatar for boys. Combined prevalence was also high in some countries in south Asia, such as India and Pakistan, where thinness remained prevalent despite having declined. In 2022, obesity in school-aged children and adolescents was more prevalent than thinness with a posterior probability of at least 0·80 among girls in 133 countries (67%) and boys in 125 countries (63%), whereas the converse was true in 35 countries (18%) and 42 countries (21%), respectively. In almost all countries for both adults and school-aged children and adolescents, the increases in double burden were driven by increases in obesity, and decreases in double burden by declining underweight or thinness.

**Interpretation:**

The combined burden of underweight and obesity has increased in most countries, driven by an increase in obesity, while underweight and thinness remain prevalent in south Asia and parts of Africa. A healthy nutrition transition that enhances access to nutritious foods is needed to address the remaining burden of underweight while curbing and reversing the increase in obesity.

**Funding:**

UK Medical Research Council, UK Research and Innovation (Research England), UK Research and Innovation (Innovate UK), and European Union.

## Introduction

Underweight and obesity are associated with adverse health outcomes throughout the life course. Therefore, optimal nutrition and health policies should address both forms of malnutrition, as indicated by Sustainable Development Goal Target 2.2, which calls for ending “all forms of malnutrition”. Trends in underweight and obesity have varied substantially across countries and age groups.^1–4^ Furthermore, underweight and obesity have changed independently of each other in some regions.^2^ Despite these heterogeneities, global data on how the combined (double) burden of underweight and obesity has changed in terms of magnitude and composition are scarce, and the latest data on their individual prevalence are from 2016.^1^ This lack of consistent evidence hinders optimal resource allocation and policy formulation to address both forms of malnutrition.

We estimated individual and combined prevalence of underweight and obesity among adults and school-aged children and adolescents from 1990 to 2022. After 1990, the focus on obesity as an epidemic increasingly matched that on undernutrition and the phrase nutrition transition was introduced to refer to changes in the type and quantity of food available in different countries.^5,6^ The consistent analysis of these outcomes helps to evaluate similarities and variations in the transition from underweight to obesity, termed the obesity transition,^7^ across countries and age groups, and helps to bridge the gap between knowledge and policies focused on undernutrition and obesity.

## Methods

### Study design

To estimate trends in underweight and obesity in national populations from 1990 to 2022, we pooled population-based studies with measurements of height and weight. Pooled data were analysed using a Bayesian hierarchical meta-regression model. Our primary outcome was the individual and combined prevalence of underweight (adults; age ≥20 years) or thinness (school-aged children and adolescents; age 5–19 years) and obesity. On the basis of previous work,^1^ we conducted separate analyses for adults and for children and adolescents because cutoffs for underweight and obesity differ between them.^8^ Under-weight was defined as a BMI of less than 18·5 kg/m^2^ and thinness as BMI less than two SD below the median of the WHO growth reference.^8^ Obesity was defined as a BMI of 30 kg/m^2^ or higher for adults and a BMI of more than two SD above the median of the WHO growth reference for children and adolescents.^8,9^ We estimated trends in these outcomes for 200 countries and territories.

### Data

We pooled population-based studies with measurements of height and weight in samples of the general population from a database collated by the NCD Risk Factor Collaboration (NCD-RisC). Details of the data sources are provided in previous publications^1–4,10,11^ and in the appendix (pp 47–51).

### Statistical methods

Data were analysed using a Bayesian hierarchical meta-regression model. The statistical methods are detailed in previous publications^4,11,12^ and in the appendix (pp 52–64). In summary, the model had a hierarchical structure in which estimates for each country and year were informed by its own data, if available, and by data from other years in the same country and from other countries, especially those in the same region with data for similar time periods. The extent to which estimates for each country-year were influenced by data from other years and other countries depended on whether the country had data, the sample size of data, whether the data were at the national, subnational, or community level, and the within-country and within-region variability of the available data. The model incorporated non-linear time trends and age associations. The model accounted for the possibility that BMI in subnational and community studies might systematically differ from, and have larger variation than, nationally representative samples, as well as for urban-rural differences in BMI. We fitted the statistical model with the Markov chain Monte Carlo (MCMC) algorithm. Posterior estimates were made in 1-year age groups for ages 5–19 years and in 5-year age groups for ages 20 years and older.

We calculated the prevalence of double burden as the sum of the prevalence of underweight or thinness and obesity. We also calculated the proportion (share) of the combined prevalence that was from obesity. We report age-standardised prevalence and proportions. Estimates were age-standardised using age weights from the WHO standard population.^13^ The number of people who were affected by underweight, thinness, and obesity was calculated by multiplying the corresponding age-specific prevalence by the age-specific population by sex, country, and year. We report Pearson correlation coefficients (*r*) among specific quantities of interest as a measure of their association.

All calculations were done at the posterior draw level. The reported credible intervals (CrIs) represent the 2·5th–97·5th percentiles of the posterior distributions, which contains the true estimates with 95% probability. We obtained the posterior probability that an estimated change represented a true increase as the proportion of draws from the posterior distribution that indicated an increase. We obtained the posterior probability that obesity was more prevalent than underweight as the proportion of posterior draws for which obesity prevalence was greater than underweight prevalence; we did the equivalent for underweight being more prevalent than obesity. Analyses were performed in R (version 4.2.0).

### Role of the funding source

The funders of the study had no role in study design, data collection, data analysis, data interpretation, or writing of the report.

## Results

The results of this study can be explored using visualisations and downloaded from the NCD-RisC website. Results presented in this report are age-standardised as described in the Methods.

A list of data sources and their characteristics is provided in the appendix (pp 66–134), along with visualisations of data availability (appendix pp 137–140). We used 3663 studies with height and weight measurements from 222 million participants aged 5 years and older, including 63 million aged 5–19 years. We had at least one study for 197 (99%) of the 200 countries for which estimates were made; 196 (98%) had data for adults and 190 (95%) for children and adolescents. Data were most scarce in Oceania (average of 5·3 studies per country) and sub-Saharan Africa (7·6 per country), with all other regions having more than ten studies per country on average (appendix pp 139–140). The high-income western region (consisting of high-income English-speaking countries, northwestern Europe, and southwestern Europe; appendix p 135) had the most data (49·0 studies per country).

**Figure 1:**
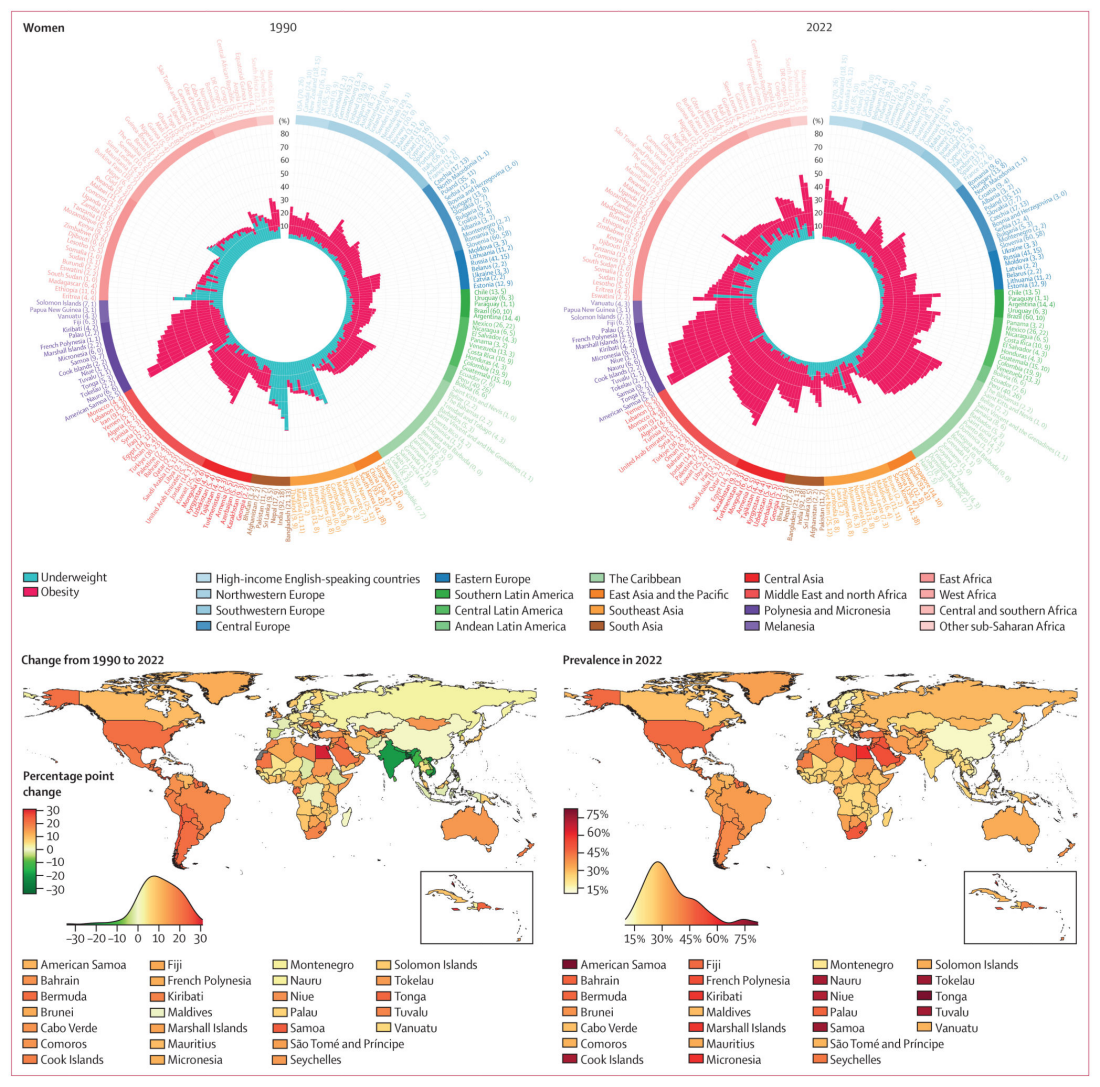

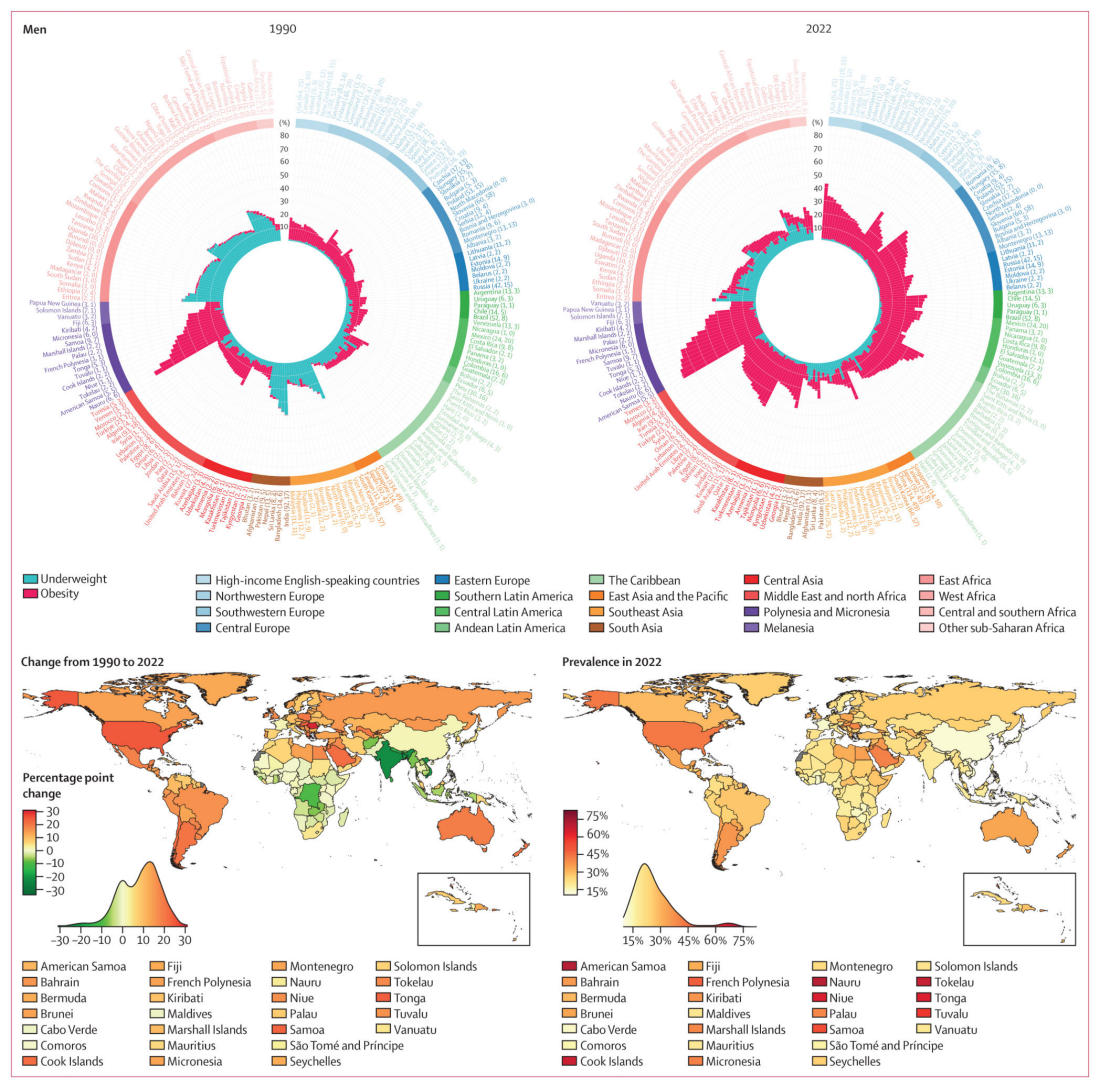
Age-standardised combined prevalence of underweight and obesity by country, for adults (age ≥20 years) The circular bar plots show the burden of underweight and obesity in 1990 and 2022. The lengths of bars show the age-standardised prevalence of underweight (blue) and obesity (red), and their sum shows the age-standardised combined prevalence. Country names are coloured by region. The numbers in brackets after each country’s name show the total number of data sources and the number of nationally representative data sources, respectively. Countries are ordered by decreasing posterior mean combined prevalence within each region. The maps show the change in combined prevalence of underweight and obesity from 1990 to 2022, and its level in 2022. The density plot alongside each map shows the smoothed distribution of estimates across countries.

In 1990, the combined age-standardised prevalence of underweight and obesity in adults (age ≥20 years) was lowest in South Korea for both women (8·0%, 95% CrI 6·6–9·6) and men (5·2%, 4·1–6·5; figure 1). The combined prevalence was less than 10% in 19 other countries (10% of countries) for men, but surpassed 10% in all other countries for women. Combined age-standardised prevalence was more than 40% in 15 countries (8%) for women and seven countries (4%) for men. These countries were the island nations in Polynesia and Micronesia, where the dominant component of the double burden was obesity, as well as India (both sexes) and Bangladesh (women), where double burden was dominated by underweight. Combined prevalence was highest in American Samoa for women (70·6%, 67·8–73·4) and Nauru for men (66·3%, 63·0–69·5).

**Figure 2:**
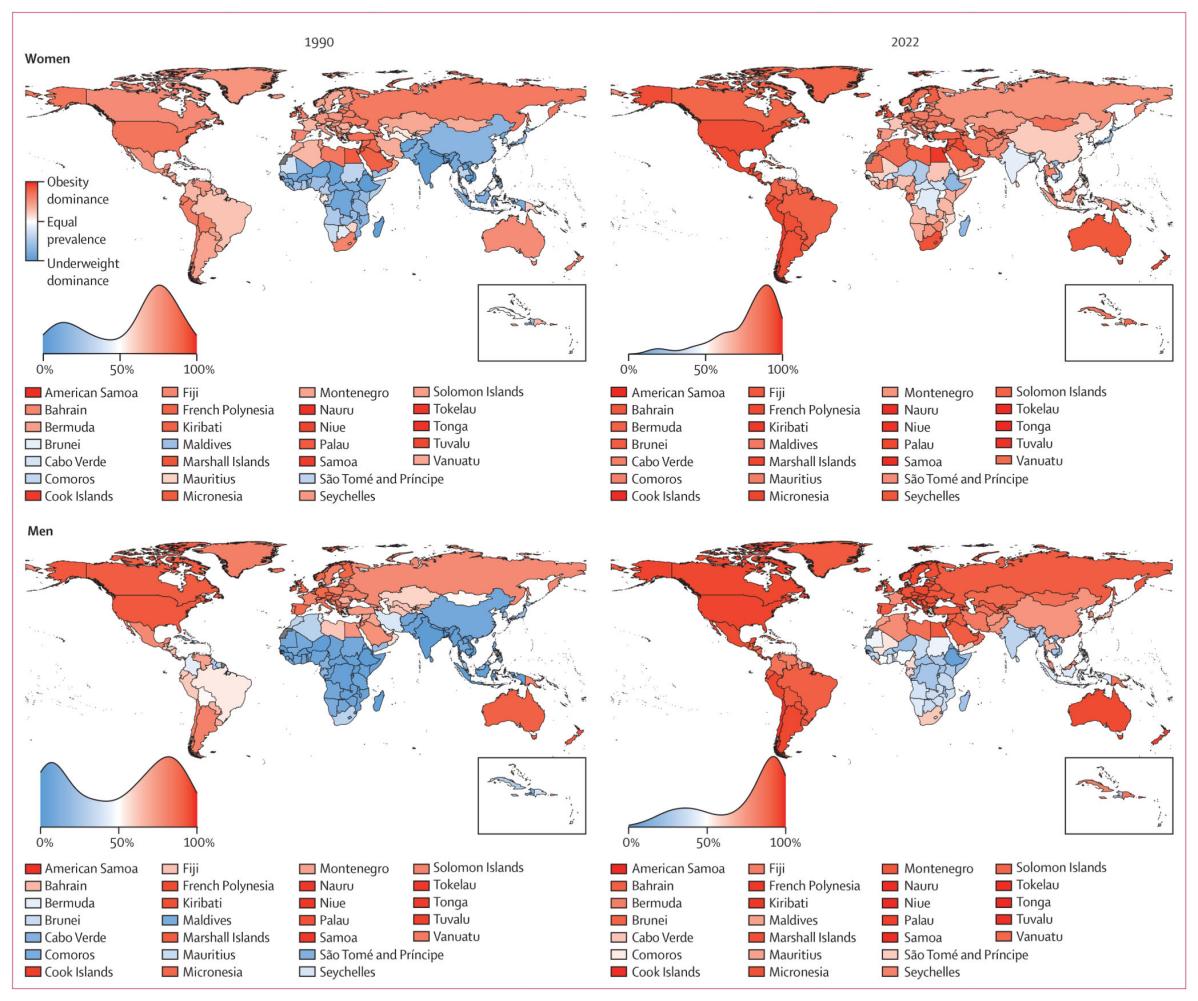
Proportion of the double burden from obesity, for adults (age ≥20 years) Age-standardised proportion of double burden that was from obesity in 1990 and 2022. The density plot alongside each map shows the smoothed distribution of estimates across countries.

In 1990, underweight was more prevalent than obesity in adults, with a posterior probability of at least 0·80, in 65 countries (33%) for women and 89 (45%) for men (figure 2, appendix pp 163–164). Conversely, obesity was more prevalent than underweight in 128 countries (64%) for women and 104 (52%) for men. In the other seven countries each for women and men, the prevalence of the two conditions was indistinguishable at a posterior probability of 0·80. In 21 countries (11%) for women and 55 (28%) for men, more than 90% of the double burden was from underweight, whereas the share of double burden from obesity surpassed 90% in 18 countries (9%) for women and 21 (11%) for men. In 1990, the double burden was most dominated by underweight in countries in southeast Asia (Viet Nam, Timor-Leste, and Cambodia), south Asia (Bangladesh, India, and Nepal), and east Africa (Ethiopia and Eritrea) for both sexes. The double burden was most dominated by obesity in countries in Polynesia and Micronesia for both sexes, and in Kuwait for women.

**Figure 3:**
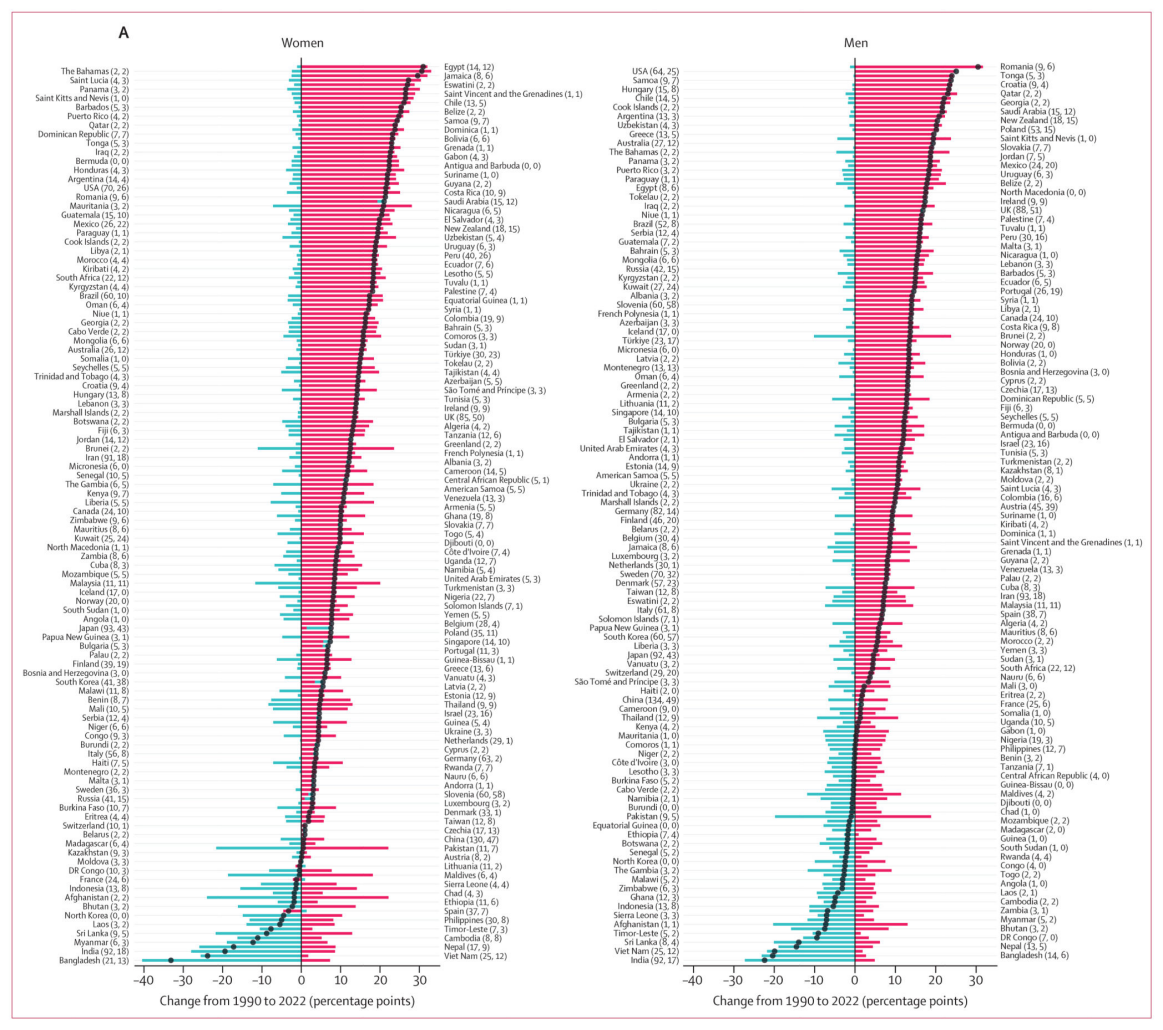

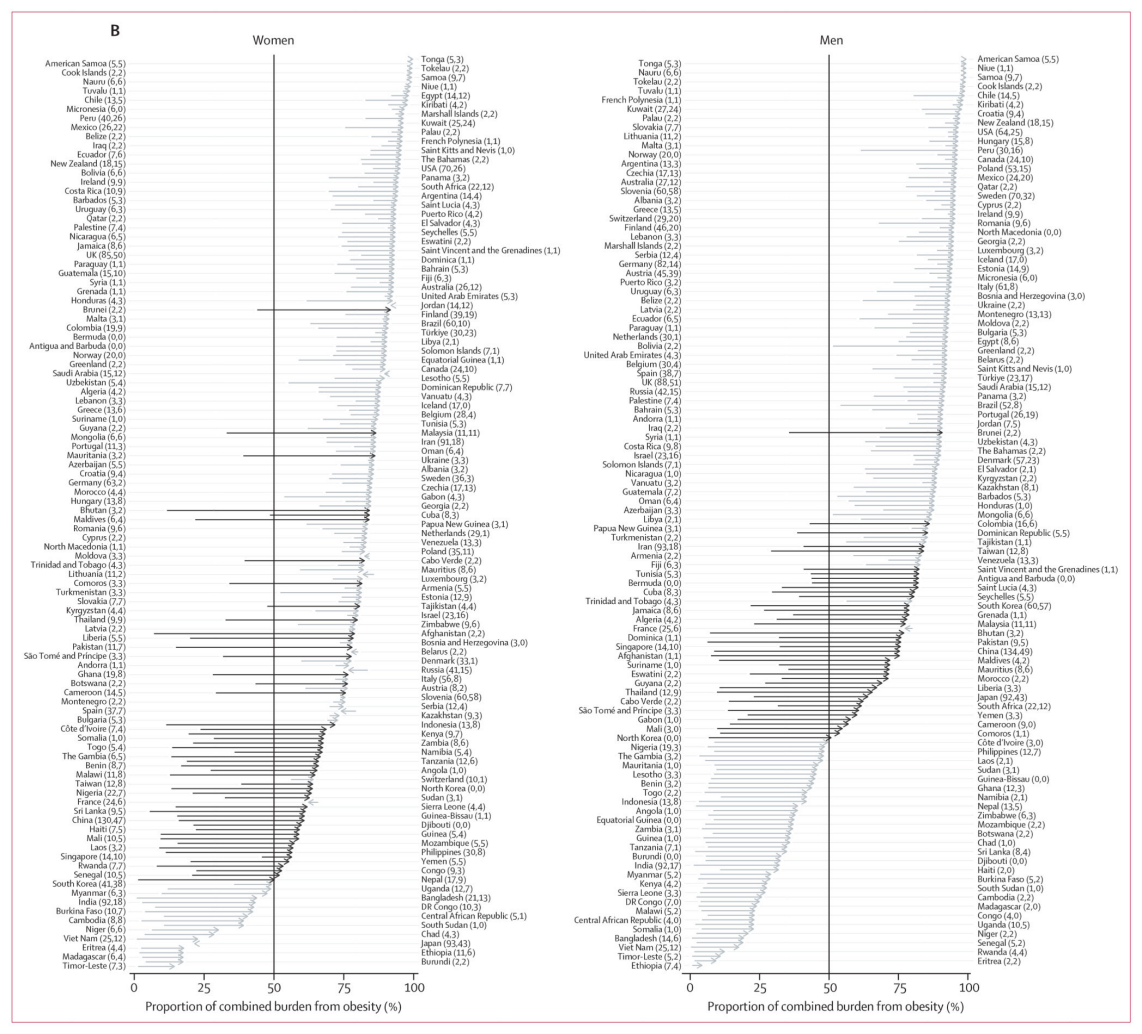
Change in the individual and combined prevalence of underweight and obesity from 1990 to 2022, for adults (age ≥20 years) (A) Contributions of change in underweight and obesity prevalence to the change in their combined prevalence from 1990 to 2022. The blue and red bars show the change in age-standardised prevalence of underweight and obesity, respectively, and the points show the change in the age-standardised combined prevalence. (B) Change in the composition of double burden from 1990 to 2022. The arrows start from the age-standardised proportion of double burden that was from obesity in 1990 and end at the age-standardised proportion from obesity in 2022; they are ordered by the posterior mean proportion in 2022. The arrows in a darker shade show countries where double burden shifted from underweight dominance to obesity dominance. In A and B, the numbers in brackets after each country’s name show the total number of data sources and the number of nationally representative data sources, respectively.

From 1990 to 2022, age-standardised prevalence of underweight among adults decreased in 129 countries (65%) for women and 149 (75%) for men with a posterior probability of at least 0·80 (figure 3, appendix pp 146–148). The largest absolute decreases were in countries in south Asia (eg, Bangladesh and India) and southeast Asia (eg, Viet Nam and Myanmar), with decreases of as much as 40·5 percentage points (95% CrI 35·0 to 45·9, posterior probability of being a true decrease >0·999) in women in Bangladesh and 27·3 percentage points (23·4 to 31·1, posterior probability >0·999) in men in India. The only groups that experienced an epidemiologically relevant increase in underweight—by more than two percentage points and with a posterior probability of at least 0·80—were women in Japan and South Korea. In 63 (32%) countries for women and 49 (25%) for men, neither an increase nor a decrease in underweight was detected at a posterior probability of 0·80; in the majority of these, age-standardised prevalence of underweight was already low (<10%) in 1990. The exceptions were men in Eritrea, Ethiopia, Haiti, Somalia, and Uganda, and women in 11 countries in sub-Saharan Africa, for whom age-standardised underweight prevalence was more than 10% in 1990 but did not show a detectable decrease at a posterior probability of 0·80. Change in underweight was negatively correlated with its starting level (*r*= –0·83 [–0·88 to –0·77] for women and –0·80 [–0·89 to –0·68] for men; appendix pp 141–142), indicating that countries with higher underweight prevalence in 1990 experienced a larger decline on average.

Age-standardised prevalence of obesity in adults increased from 1990 to 2022 in 188 countries (94%) for women and in all except one country for men with a posterior probability of at least 0·80 (figure 3, appendix pp 149–151). The largest increases were in some countries in sub-Saharan Africa for women; the USA, Brunei, and some countries in central Europe and Polynesia and Micronesia for men; and some countries in the Caribbean and in the Middle East and north Africa for both sexes. Age-standardised prevalence of obesity increased by more than 20 percentage points in 49 countries (25%) for women and 24 countries (12%) for men, and by as much as 33·0 percentage points (95% CrI 23·0 to 42·3, posterior probability >0·999) in The Bahamas for women and 31·7 percentage points (25·3 to 38·1, posterior probability >0·999) in Romania for men. It decreased only among women in Spain (by 4·6 percentage points [1·1 to 7·8, posterior probability 0·995]) and France (2·2 percentage points [–0·8 to 5·1, posterior probability 0·927]). In women in the remaining ten countries, mostly in Europe, and in men in France, neither an increase nor a decrease was detected at a posterior probability of 0·80. Unlike underweight, the correlation between change in obesity and its 1990 level was weak (*r*=0·13 [0·01 to 0·24] for women and 0·31 [0·13 to 0·47] for men). Furthermore, at 1990 baseline obesity levels of 5% or higher (appendix pp 141–142), the correlation was almost non-existent (*r*= –0·04 [–0·16 to 0·09] for women and –0·08 [–0·31 to 0·15] for men), indicating that the extent of increase in obesity was largely unrelated to how prevalent it was in 1990. There was no correlation between change in obesity and change in underweight for women (*r*= –0·02 [–0·10 to 0·05]) but a weak positive correlation was found for men (*r*= 0·29 [0·14 to 0·43]).

In 2022, the prevalence of obesity was less than 5% among women in six countries (3%; Viet Nam, Timor-Leste, Japan, Burundi, Madagascar, and Ethiopia) and among men in 17 countries (9%) in south and southeast Asia and sub-Saharan Africa. It surpassed 60% among women in eight countries (4%) and men in six countries (3%), all in Polynesia and Micronesia. The range of underweight prevalence across countries in 2022 was smaller than the range of obesity prevalence, and prevalence surpassed 20% only among women in Timor-Leste and four countries in sub-Saharan Africa, and among men in four countries in sub-Saharan Africa (figure 1).

The net effect of changes in underweight and obesity in adults was that their combined age-standardised prevalence decreased in 11 countries (6%) for women and 17 countries (9%) for men with a posterior probability of at least 0·80, and increased in 162 countries (81%) for women and 140 countries (70%) for men with a posterior probability of at least 0·80, from 1990 to 2022 (figure 3, appendix pp 152–153). Countries where the double burden decreased were mostly in south and southeast Asia for both sexes, and in sub-Saharan Africa for men. Among women in sub-Saharan Africa, however, the decline in underweight was counteracted by a rise in obesity, such that their combined prevalence either changed little or increased (figures 1, 3). Decreases in the double burden of underweight and obesity were driven by decreases in underweight, with the exception of women in Spain, for whom the decrease was driven by a decline in obesity.

Bangladesh, India, and Viet Nam experienced the largest decreases in double burden among adults for both sexes, with decreases by 33·1 percentage points (95% CrI 27·4–38·7, posterior probability >0·999) among women in Bangladesh and 22·4 percentage points (18·5–26·2, posterior probability >0·999) among men in India (figures 1, 3). Mirroring the declines, the increase in combined prevalence of underweight and obesity in most countries was due to the rise in obesity exceeding a decline in underweight. As a result, countries that experienced a large increase in double burden were the same as those that had a large increase in obesity. The largest increases in double burden among women were in Egypt (31·0 percentage points, 25·4–36·6, posterior probability >0·999), followed by The Bahamas and Jamaica, and among men in Romania (30·5 percentage points, 24·0–37·0, posterior probability >0·999), followed by the USA and Tonga.

In 2022, the combined prevalence of underweight and obesity in adults was more than 10% in all countries (figure 1). It was between 10% and 15% for women in four countries (South Korea, China, Viet Nam, and Denmark) and men in 17 countries (9%). South Korea and China had the lowest combined prevalence for women, and Sierra Leone, South Korea, and China had the lowest combined prevalence for men. At the other extreme, combined underweight and obesity prevalence was 40% or higher in 55 countries (28%) for women and 16 (8%) for men. The prevalence was highest in American Samoa (81·7%, 73·6–88·8 for women; 70·6%, 59·8–79·8 for men), followed by other island nations in Polynesia and Micronesia and the Caribbean (eg, The Bahamas and Saint Kitts and Nevis), and countries in the Middle East and north Africa (Egypt for women, Qatar for men). Outside of these regions, men in the USA also had a high prevalence of double burden (43·0%, 39·1–47·1), as did women in South Africa (49·6%, 45·1–54·2). The number of countries where underweight was more prevalent than obesity among adults with a posterior probability of at least 0·80 decreased from 65 (33%) in 1990 to 16 (8%) in 2022 for women, and from 89 (45%) to 39 (20%) for men (figure 2). In 2022, all such countries were in sub-Saharan Africa, southeast Asia, and south Asia, with the exceptions of Haiti (for men) and Japan (for women).^14^ Obesity prevalence was higher than underweight with a posterior probability of at least 0·80 in 177 countries (89%) for women and 145 countries (73%) for men in 2022. The largest absolute increases in the proportion of double burden from obesity were in countries in south Asia (eg, Bhutan, Pakistan, and Afghanistan), southeast Asia (eg, Maldives and Malaysia), and west Africa (eg, Liberia) for both sexes, and in east Asia and the Pacific (eg, China) for men. In 50 countries (25%) for women and 41 countries (21%) for men, double burden shifted from underweight dominance to obesity dominance (figure 3). By 2022, more than 90% of double burden consisted of obesity in 67 countries (34%) for women and 88 (44%) for men. These countries were predominantly in Oceania, Latin America and the Caribbean, the Middle East and north Africa, and the high-income western region for both sexes, and in central and eastern Europe for men. The share of double burden from underweight surpassed 90% only among men in Ethiopia and Eritrea.

Further results for adults are provided in the appendix, including separate results for ages 20–39 years, 40–64 years, and 65 years and older (appendix pp 156–162, 165–168, 172–179); results on the levels of all BMI categories (appendix pp 143–145); maps of the age-standardised prevalence of underweight in 1990 and 2022, change from 1990 to 2022, and posterior probability that underweight increased from 1990 to 2022 (appendix pp 146–148); maps of the age-standardised prevalence of obesity in 1990 and 2022, change from 1990 to 2022, and posterior probability that obesity increased from 1990 to 2022 (appendix pp 149–151); maps of the posterior probability that the age-standardised combined prevalence increased from 1990 to 2022 (appendix pp 152–153); trends in underweight, obesity, and their combined prevalence from 1990 to 2022, together with their 95% CrIs, for all countries (appendix pp 204–404); maps of change in the proportion of double burden that was from obesity from 1990 to 2022, and posterior probability that the proportion of double burden that was from obesity increased from 1990 to 2022 (appendix pp 154–155); the uncertainty (posterior SD) of the proportion of double burden that was from obesity (appendix pp 163–164); and the uncertainty (posterior SD) of the changes in underweight, obesity, their combined prevalence, and the proportion of the combined prevalence that was from obesity, from 1990 to 2022 (appendix pp 169–171).

The global age-standardised prevalence of underweight in adults decreased from 14·5% (95% CrI 14·0–15·0) in 1990 to 7·0% (6·5–7·5) in 2022 in women and from 13·7% (13·0–14·4) to 6·2% (5·6–6·9) in men. 183 million (169–197) women and 164 million (148–180) men were underweight in 2022, a decrease of 44·9 million (28·5–61·0) and 47·6 million (27·1–66·7), respectively, from 1990, despite global population growth. The countries with the largest number of adults with underweight in 2022 were India, China, Japan (for women only), Indonesia, Ethiopia, and Bangladesh. Age-standardised prevalence of underweight in Ethiopia, Bangladesh, India, and Japan (for women) ranged from 11% to 26% and ranked in the 25 highest countries, which, together with large populations, led to large numbers of people with under-weight. The relatively high prevalence of underweight among women (but not men) in Japan, the only high-income country in this group, has been attributed to perceived weight being higher than actual weight, and higher than desired weight.^14^ Underweight prevalence was lower in Indonesia (6·6% [4·7–8·8] and 10·6% [7·1–14·6] for women and men, respectively) and especially in China (5·9% [4·6–7·4] and 2·9% [1·9–4·0], ranking 61st-highest and 97th-highest globally). Nonetheless, when combined with the large populations in these countries, even this low or moderate prevalence led to large absolute numbers. The global age-standardised prevalence of obesity increased from 8·8% (8·5–9·1) in 1990 to 18·5% (17·9–19·1) in 2022 in women and from 4·8% (4·6–5·0) to 14·0% (13·4–14·6) in men. The global age-standardised prevalence of obesity overtook that of underweight in 2003 (2003–2004) for women and 2009 (2008–2010) for men. The number of women and men with obesity in 2022 was 504 million (489–520) and 374 million (358–391), respectively, which was an increase of 377 million (360–393) and 307 million (290–324), respectively, from 1990. The countries with the largest absolute numbers of adults with obesity in 2022 were the USA, China, and India.

**Figure 4:**
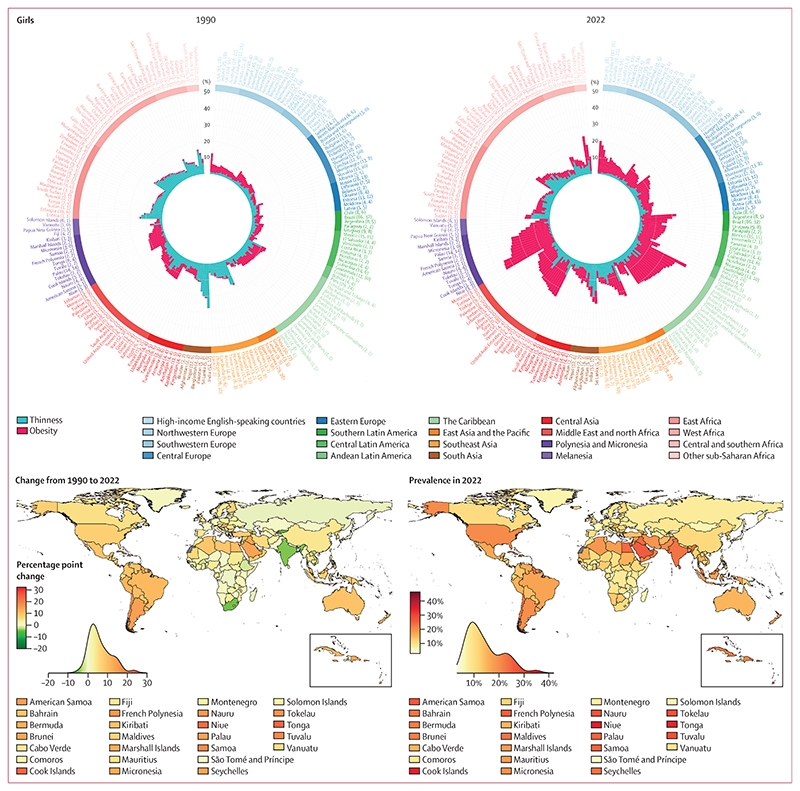

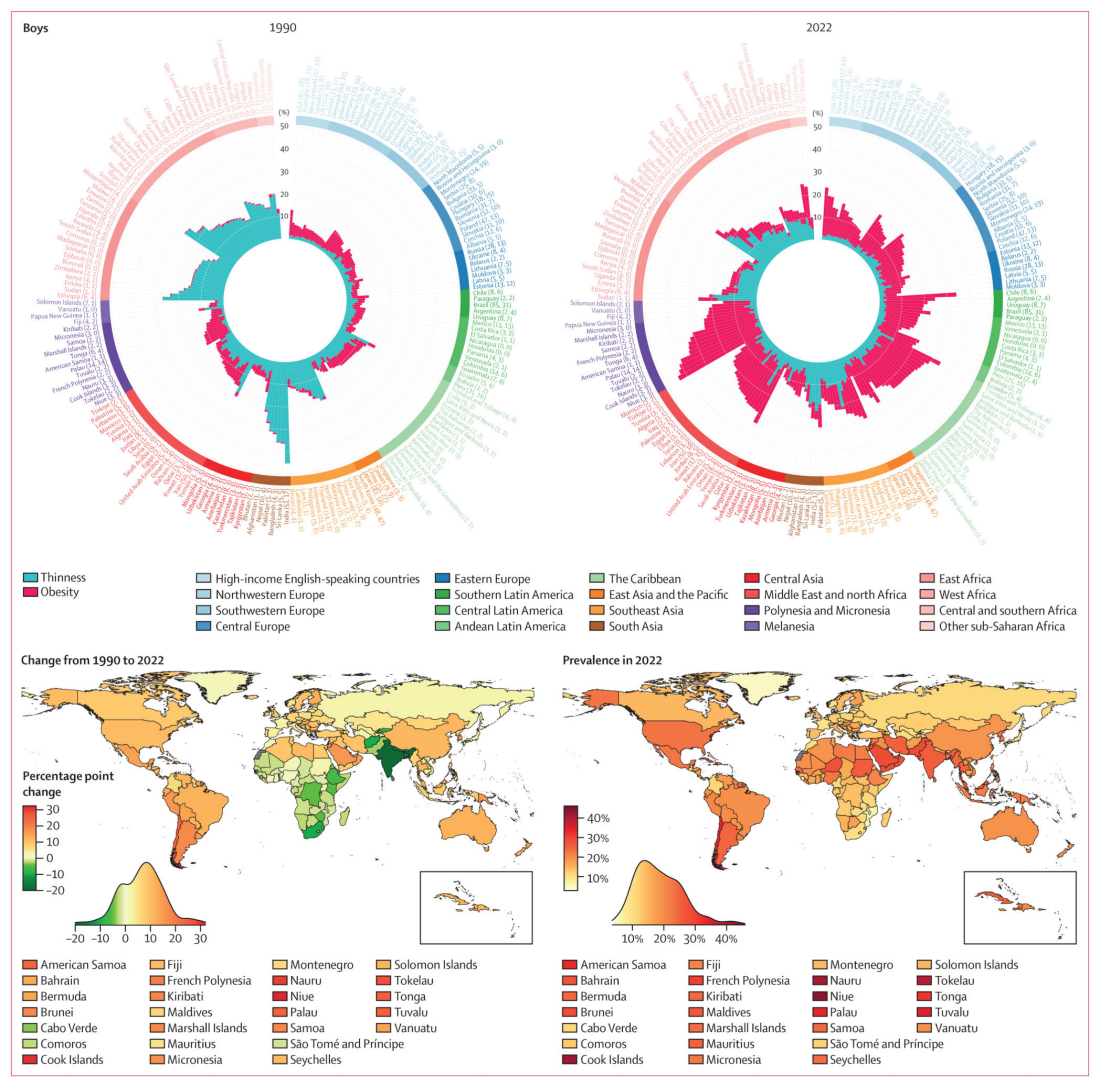
Age-standardised combined prevalence of thinness and obesity by country, for school-aged children and adolescents (age 5–19 years) The circular bar plots show the burden of thinness and obesity in 1990 and 2022. The lengths of the bars show the age-standardised prevalence of thinness (blue) and obesity (red), and their sum shows the age-standardised combined prevalence. Country names are coloured by region. The numbers in brackets after each country’s name show the total number of data sources and the number of nationally representative data sources, respectively. Countries are ordered by decreasing posterior mean combined prevalence within each region. The maps show the change in combined prevalence of thinness and obesity from 1990 to 2022 and its level in 2022. The density plot alongside each map shows the smoothed distribution of estimates across countries.

**Figure 5:**
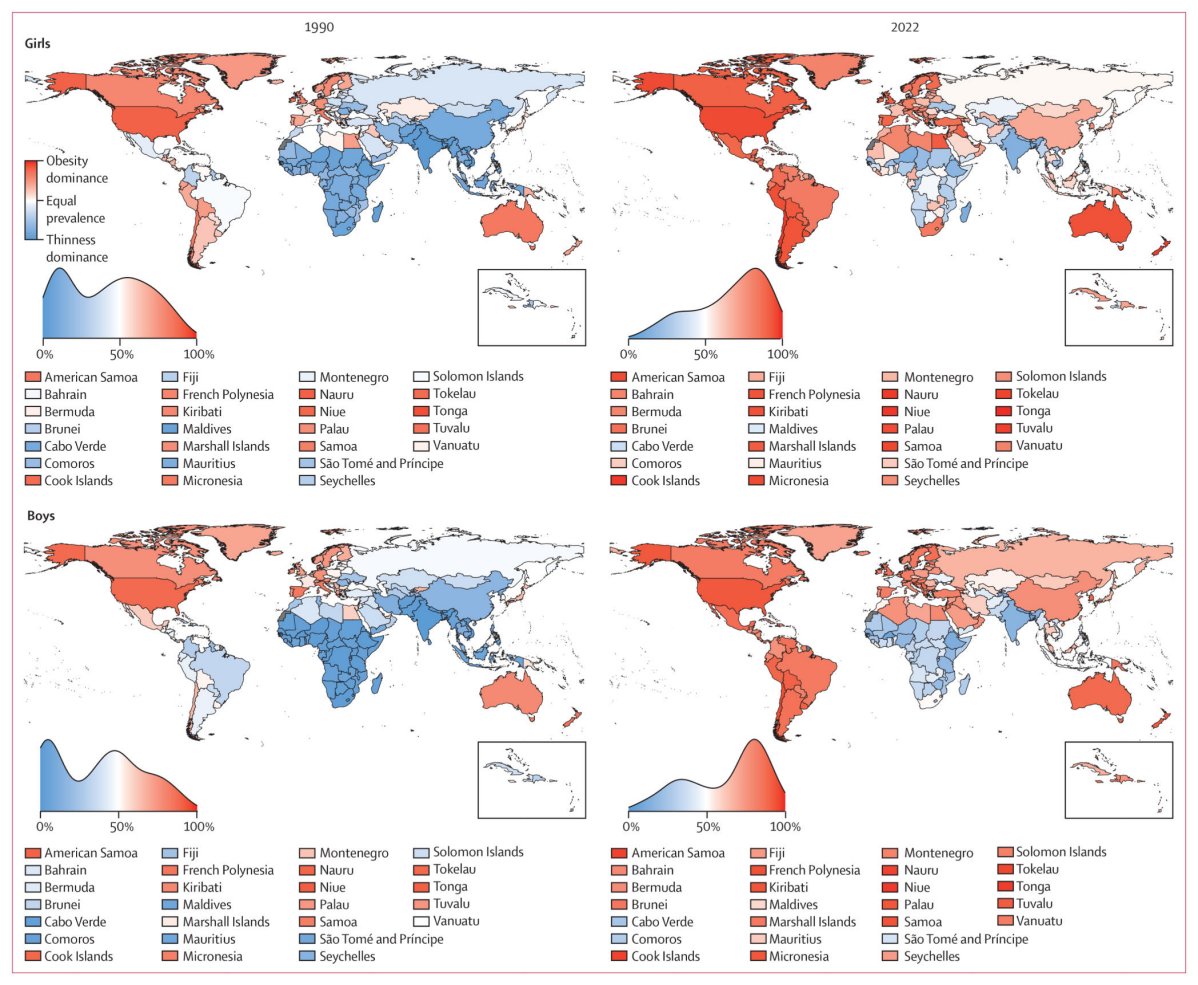
Proportion of double burden from obesity for school-aged children and adolescents (age 5–19 years) Age-standardised proportion of double burden that was from obesity in 1990 and 2022. The density plot alongside each map shows the smoothed distribution of estimates across countries.

In 1990, the contribution of thinness to the double burden of thinness and obesity in school-aged children and adolescents (age 5–19 years) was larger than the corresponding share of underweight in adults, especially for boys. Specifically, in 93 countries (47%) for each sex, the age-standardised prevalence of thinness was greater than that of obesity with a posterior probability of at least 0·80; conversely, the prevalence of obesity was greater than that of thinness in 52 countries (26%) for girls and 47 countries (24%) for boys (figures 4, 5; appendix pp 195–196). In the other 55 countries (28%) for girls and 60 countries (30%) for boys, the prevalence of the two forms of malnutrition were indistinguishable at a posterior probability of 0·80. In 34 countries (17%) for girls and in 64 (32%) for boys, mostly in south and southeast Asia and sub-Saharan Africa, more than 90% of double burden consisted of thinness, whereas the opposite (>90% of double burden from obesity) occurred in some countries in Polynesia and Micronesia for both sexes, and in the USA for girls.

India experienced the highest age-standardised prevalence of double burden in school-aged children and adolescents in 1990 (27·4% [95% CrI 24·3–30·7] for girls and 45·3% [42·1–48·5] for boys, with 99·6% [99·3–99·8] and 99·7% [99·5–99·9], respectively, consisting of thinness). Age-standardised prevalence was between 30% and 40% for boys in Sri Lanka, Bangladesh, Pakistan, and Nepal, with 98–99% of double burden coming from thinness (figures 4, 5). Kuwait and Egypt were also among the top ten countries for girls in terms of double burden, but unlike the aforementioned countries where thinness was dominant, more than 70% of double burden came from obesity. Age-standardised prevalence of obesity in 1990 surpassed 10% in only seven countries (4%) for girls and six (3%) for boys, including the USA and some countries in Polynesia and Micronesia and the Middle East and north Africa, whereas thinness surpassed 10% in 20 countries (10%) for girls and 67 countries (34%) for boys.

**Figure 6:**
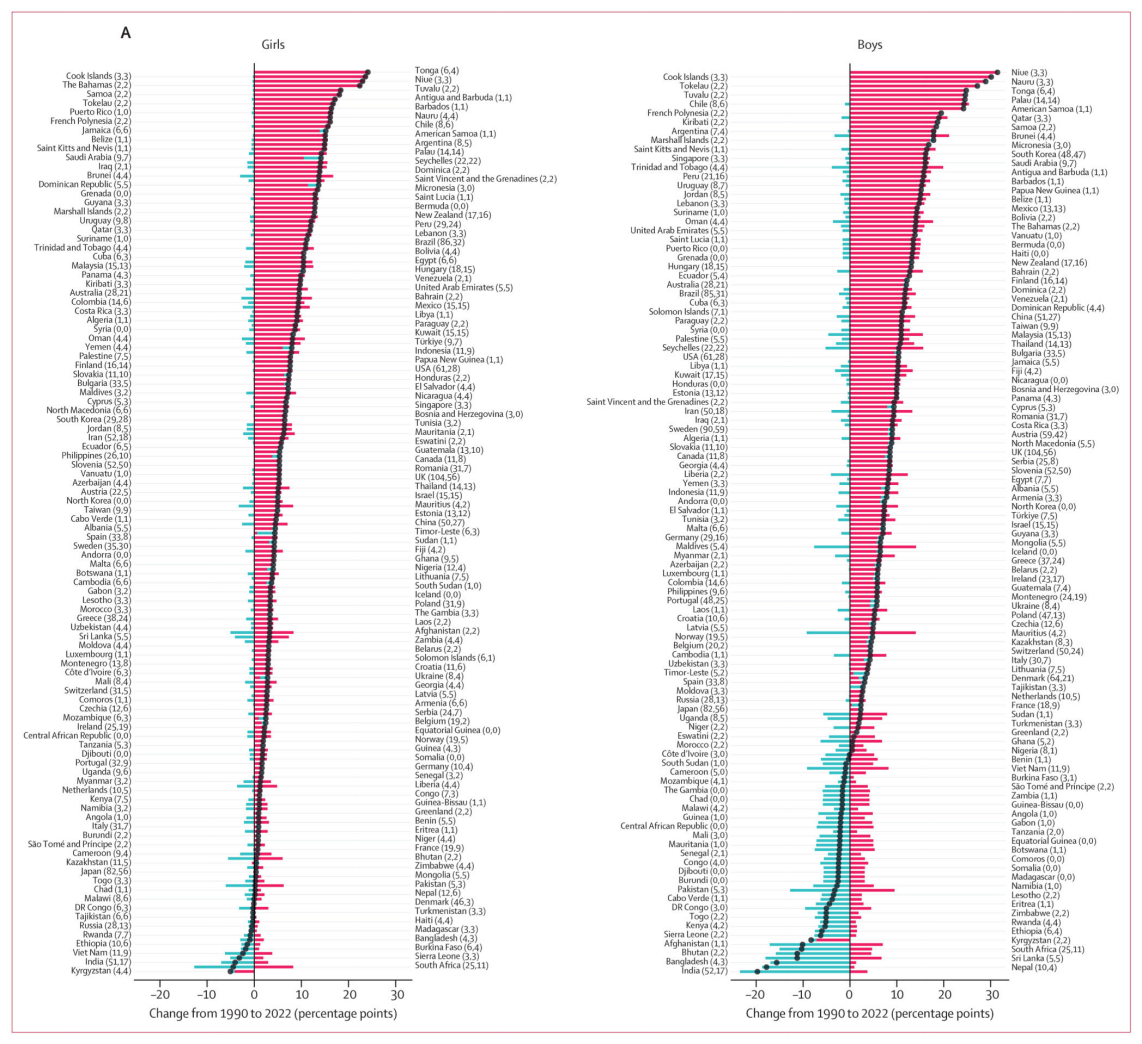

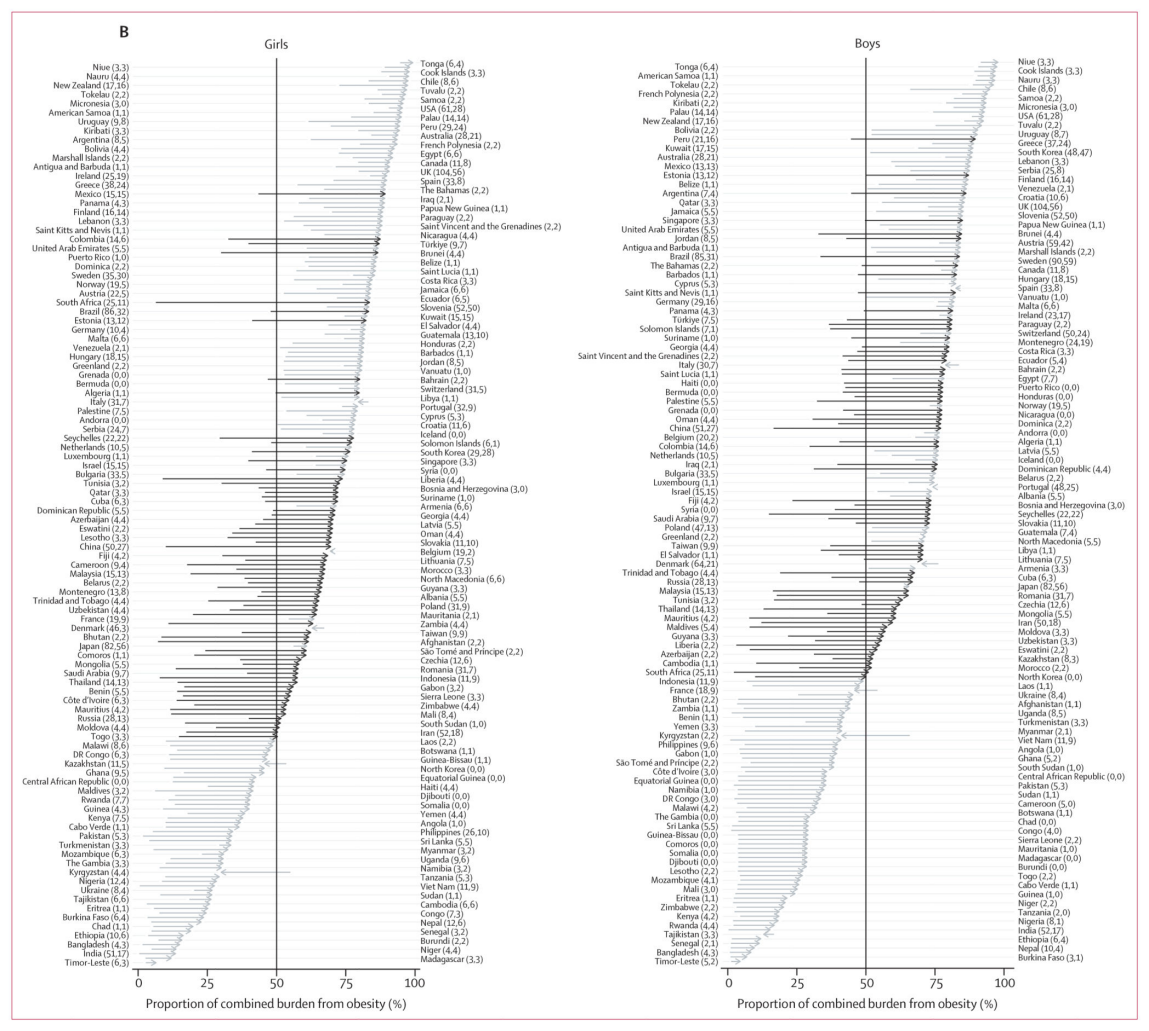
Change in the individual and combined prevalence of thinness and obesity from 1990 to 2022, for school-aged children and adolescents (age 5–19 years) (A) Contributions of change in thinness and obesity to the change in their combined prevalence from 1990 to 2022. The blue and red bars show the change in age-standardised prevalence of thinness and obesity, respectively, and the points show the change in the age-standardised combined prevalence. (B) Change in the composition of double burden from 1990 to 2022. The arrows start from the age-standardised proportion of double burden that was from obesity in 1990 and end at the age-standardised proportion in 2022; they are ordered by the posterior mean proportion in 2022. The arrows in a darker shade show countries where double burden shifted from thinness dominance to obesity dominance. In A and B, the numbers in brackets after each country’s name show the total number of data sources and the number of nationally representative data sources, respectively.

From 1990 to 2022, age-standardised prevalence of thinness decreased in girls in 44 countries (22%) and boys in 80 countries (40%) with a posterior probability of at least 0·80 (figure 6). The largest decreases took place in countries in south and southeast Asia and sub-Saharan Africa (figures 4, 5), with the greatest decreases in South Africa (decrease of 12·7 percentage points [95% CrI 6·8 to 19·8]) and India (7·0 [2·2 to 11·9]) for girls, and India (23·5 [18·5 to 28·4]) and Nepal (18·7 [9·1 to 28·7]) for boys. Like in adults, change in thinness was negatively correlated with the starting level of thinness (*r*= –0·53 [–0·70 to –0·33] for girls and –0·79 [–0·90 to –0·62] for boys; appendix pp 180–181), indicating that larger declines occurred where thinness was more common in 1990. However, in some countries, such as Yemen, Niger, and Myanmar, age-standardised thinness prevalence was at least 10% in 1990 but did not change from 1990 to 2022 with a posterior probability of at least 0·80 (appendix pp 185–187). Thinness increased by at least two percentage points and with a posterior probability of at least 0·80 in three countries for girls and one country for boys.

Over the same period, age-standardised prevalence of obesity increased in girls in 186 countries (93%) and in boys in 195 countries (98%) with a posterior probability of at least 0·80 (figure 6, appendix pp 188–190). In most countries, obesity more than doubled (appendix pp 180–181). Obesity decreased with a posterior probability of at least 0·80 only in Kyrgyzstan, by 4·1 percentage points (95% CrI 1·3 to 7·3) for girls and 7·2 percentage points (2·8 to 11·6) for boys. In the remaining 13 countries (7%) for girls and four countries (2%) for boys, mostly in Europe and central Asia, neither an increase nor a decrease was detected at a posterior probability of 0·80. The largest increases in child and adolescent obesity were in the island nations of Polynesia and Micronesia and the Caribbean, Brunei, and Chile. The size of the increase surpassed 20 percentage points among girls in Tonga, Cook Islands, Niue, and The Bahamas, and surpassed 25 percentage points among boys in Niue, Cook Islands, Nauru, Tokelau, and Chile. Like in adults, there was weak correlation between obesity in 1990 and change from 1990 to 2022 (*r*=0·37 [0·19 to 0·54] for girls and 0·30 [0·11 to 0·47] for boys; appendix pp 180–181). In particular, in many countries in Europe, Japan, the USA, and some countries in central Asia, an increase in child and adolescent obesity was either not detectable at a posterior probability of 0·80 or was smaller than in countries elsewhere that had a similar prevalence in 1990. There was no or weak correlation between change in thinness and change in obesity (*r*= –0·02 [–0·17 to 0·12] for girls and 0·14 [–0·04 to 0·30] for boys).

In 2022, age-standardised prevalence of thinness was less than 15% for girls in all countries except India and Sri Lanka, and below 20% among boys in all countries except Niger, India, Senegal, and Timor-Leste (figure 4). By contrast, age-standardised prevalence of obesity was more than 20% in girls in 21 countries (11%) and boys in 35 countries (18%), with the highest prevalence in Niue for both girls (34·3%, 95% CrI 23·5–44·7) and boys (42·9%, 31·6–53·4). These countries were mostly in Polynesia and Micronesia, Latin America and the Caribbean, and the Middle East and north Africa.

As a result of changes in thinness and obesity, by 2022, double burden in school-aged children and adolescents began to mirror the obesity dominance that was seen in adults throughout these three decades. Specifically, in 2022, obesity in school-aged children and adolescents was more prevalent than thinness with a posterior probability of at least 0·80 among girls in 133 countries (67%) and boys in 125 countries (63%), whereas the opposite was true in 35 (18%) and 42 countries (21%), respectively (figure 5, appendix pp 195–196). In 25 countries (13%) for girls and 17 countries (9%) for boys, more than 90% of double burden consisted of obesity in 2022, whereas the share of thinness surpassed 90% only among girls and boys in Timor-Leste, and boys in Burkina Faso, Bangladesh, and Nepal. The largest shifts in the thinness–obesity share of double burden in school-aged children and adolescents were in South Africa for girls, with the share of obesity increasing by 77·2 percentage points (95% CrI 64·3–86·7, posterior probability >0·999), and in China for boys, with an increase of 60·5 percentage points (50·4–69·4, posterior probability >0·999; figure 6).

The combined prevalence of thinness and obesity declined with posterior probability of at least 0·80 among girls in India, Kyrgyzstan, Sierra Leone, South Africa, and Viet Nam, and among boys in 15 countries (8%), all of which were in central Asia, south Asia, and sub-Saharan Africa (figure 6, appendix pp 191–192). With the exception of Kyrgyzstan, the declines in the double burden were driven by declines in the prevalence of thinness. The largest declines were in Kyrgyzstan (5·1 percentage points, 95% CrI 1·0–9·2, posterior probability 0·991) for girls and in India (19·7 percentage points, 14·7–24·8, posterior probability >0·999) for boys. The combined burden of thinness and obesity increased with posterior probability of at least 0·80 among girls in 140 countries (70%) and boys in 137 countries (69%). The largest increases were in some countries in Polynesia and Micronesia and the Caribbean for both sexes, and in Chile for boys, with the size of the increase reaching 24·1 percentage points (12·7–34·6, posterior probability >0·999) in girls in Tonga and 31·1 percentage points (16·4–44·6, posterior probability >0·999) in boys in Niue.

In 2022, the combined age-standardised prevalence of thinness and obesity in school-aged children and adolescents was less than 10% in 70 countries (35%) for girls and 16 countries (8%) for boys (figure 4). In these countries, thinness either remained low throughout the analysis period or declined, and obesity did not rise noticeably. These countries were mostly in central Asia, Europe, and sub-Saharan Africa. At the other extreme, the countries with highest combined prevalence of thinness and obesity in 2022 were in Polynesia and Micronesia and the Caribbean for both sexes, plus Chile and Qatar for boys. Double burden prevalence was also high in some countries in south Asia, notably Sri Lanka and India, where thinness remained prevalent despite its decline.

Further results for children and adolescents are shown in the appendix, including levels of all BMI categories (appendix pp 182–184); maps of the age-standardised prevalence of thinness in 1990 and 2022, change from 1990 to 2022, and posterior probability that thinness increased from 1990 to 2022 (appendix pp 185–187); maps of the age-standardised prevalence of obesity in 1990 and 2022, change from 1990 to 2022, and posterior probability that obesity increased from 1990 to 2022 (appendix pp 188–190); maps of the posterior probability that the age-standardised combined prevalence increased from 1990 to 2022 (appendix pp 191–192); trends in thinness, obesity, and their combined prevalence from 1990 to 2022, together with their 95% CrIs, for all countries (appendix pp 204–404); maps of change in the proportion of double burden from obesity from 1990 to 2022, and posterior probability that the proportion of double burden from obesity increased from 1990 to 2022 (appendix pp 193–194); the uncertainty (posterior SD) of the proportion of double burden from obesity (appendix pp 195–196); and the uncertainty (posterior SD) of the changes in thinness, obesity, their combined prevalence, and the proportion of the double burden from obesity, from 1990 to 2022 (appendix pp 197–199).

The global age-standardised prevalence of thinness among school-aged children and adolescents decreased from 10·3% (95% CrI 9·5–11·1) in 1990 to 8·2% (7·3–9·0) in 2022 in girls and from 16·7% (15·6–17·8) to 10·8% (9·7–11·9) in boys (posterior probability >0·999). A total of 77·0 million (69·1–84·9) girls and 108 million (98–119) boys were affected by thinness in 2022, a decrease of 4·4 million (95% CrI ranging from an increase of 5·6 million to a decrease of 14·6 million) for girls and 30·1 million (15·6–44·3) for boys from 1990. The global age-standardised prevalence of obesity in school-aged children and adolescents increased from 1·7% (1·5–2·0) in 1990 to 6·9% (6·3–7·6) in 2022 in girls and from 2·1% (1·9–2·3) to 9·3% (8·5–10·2) in boys (posterior probability >0·999). The number of girls and boys with obesity in 2022 was 65·1 million (59·4–71·7) and 94·2 million (85·3–103·0), respectively, an increase of 51·2 million (45·2–57·8) and 76·7 million (67·6–85·7), respectively, from 1990.

## Discussion

Our results show three important global transitions in underweight and obesity since 1990. First, the combined prevalence of these forms of malnutrition has increased in most countries, with the notable exception of countries in south and southeast Asia and, for some age-sex groups, in sub-Saharan Africa. Second, decreases in the double burden were largely driven by declining prevalence of underweight, whereas increases were driven by increasing obesity, leading to a transition from underweight dominance to obesity dominance in many countries. The rise in double burden has been largest in some low-income and middle-income countries, notably those in Polynesia and Micronesia, the Caribbean, and the Middle East and north Africa; newly high-income countries such as Chile; and, for men, in central Europe. These countries now have higher obesity prevalence than industrialised high-income countries. Finally, the transition to obesity was already apparent in adults in 1990 in much of the world, as demonstrated by the large number of countries in which adult obesity exceeded underweight at that time, and has since followed in school-aged children and adolescents.^7^

Our study has strengths related to its scope, data, and methods. We present consistent estimates of underweight and obesity, and their combined prevalence, over a period of substantial change in food and nutrition. We used a large amount of population-based data, from 197 countries covering more than 99% of the world’s population. We maintained a high standard of data quality through repeated checks of study sample and characteristics, and did not use self-reported data to avoid bias. Data were analysed according to a consistent protocol. We used a statistical model that accounted for the age patterns of BMI during childhood, adolescence, and adulthood. We used all available data while giving more weight to national data than to subnational and community data.

As with all global analyses, our study has limitations. Some countries had fewer data and three had none; their estimates were informed to a stronger degree by data from other countries through geographical hierarchy. There were also differences in data availability by age group, with less data available for ages 5–9 years, and in older adults (≥65 years), which increased the uncertainty of estimates in these age groups. Despite our systematic and rigorous process of evaluating study representativeness, data from health surveys are subject to error if sample weights do not fully adjust for non-response. We did not report on height, a marker of the quality of nutrition and the living environment, and predictive of health throughout the life course,^15^ as reported previously.^4,11^ BMI is an imperfect measure of the extent and distribution of body fat, but is widely available in population-based surveys, and is used in clinical practice; it is also correlated with the more complex and costly dual-energy x-ray absorptiometry.^16^ Cutoffs for thinness and obesity for school-aged children and adolescents are based on BMI distributions in a reference population, and were not selected to represent optimal BMI in epidemiological studies, as was done for adults, or optimal nutritional status, as for children younger than 5 years.^8,9^ Finally, various hypotheses exist about the impact of the COVID-19 pandemic on BMI. Data on the impact of the pandemic on obesity are scarce,^17^ and there are even fewer data on the impact on underweight. The available data on obesity, mostly from high-income countries, indicate a small rise in prevalence, with large heterogeneity across studies;^17^ it is unclear whether these effects are transitory or permanent. Studies from low-income and middle-income countries have indicated worsening food security and diet quality during and after the pandemic, but did not measure underweight prevalence.^18^ We used 103 studies from 2020 and later, but additional data are needed to evaluate the population-level effects. Finally, although our statistical model has been shown to be unbiased and have small deviation (ie, random error) in cross-validation analyses,^3,10^ fitting to data that vary in relation to age, country, and year has the potential for model misspecification.

Height and weight are affected by the quantity and quality of nutrition, energy expenditure, and some infections.^19,20^ For decades before the COVID-19 pandemic, higher incomes allowed more spending on food, especially in poor countries and households where a large share of income is spent on food.^21^ Meanwhile, as food production, distribution, and storage changed, there was a shift from subsistence and local foods to transported commercial foods, and the time spent obtaining and preparing food was reduced.^22–24^ These economic and technological changes have affected both the amount and types of food that are consumed, including higher total calories, and higher consumption of animal-source foods, sugar, vegetables, and oil crops in many low- and middle-income countries, whereas animal-source foods and possibly sugar consumption have declined in high-income countries.^25–29^ Furthermore, changes in food processing technology and increasing commercialisation and industrialisation of food^30^ have increased the consumption of processed foods, which leads to higher caloric intake and weight gain than fibre-rich foods such as whole grains and fruits.^31,32^ In some countries, equitable economic and agricultural policies and food programmes have improved the quality of nutrition, especially for the poor,^33–36^ resulting in gains in height, whereas elsewhere weight gain occurred without substantial height gain, leading to increasing obesity.^4,11,19,37^ These height gains were similar to those in Europe and north America during industrialisation and economic development.^38^ Researchers have also detected a so-far unexplained decline in adult basal energy expenditure,^39^ which might have contributed to the rise in obesity.

The finding that obesity increased, and double burden shifted from underweight-dominated to obesity-dominated, earlier in adults than in children and adolescents^40^ might be due to two phenomena: first, adults began to eat away from home earlier than children and adolescents,^41–43^ and second, the mechanisation of work and transport, while providing many health benefits, also reduced energy expenditure, and hence contributed to weight gain among adults.^44^ The shift in onset of obesity to younger ages over these three decades could be because eating away from home and access to commercial and processed foods in school-aged children and adolescents followed that of adults over this period.^45^ It has also been hypothesised that some leisure-time play and sports have been replaced by sedentary activities, but data on trends are scarce.^46^ Breastfeeding, which improves child survival and development, has also been associated with a lower risk of obesity in observational studies but the findings from randomised trials are mixed, possibly because of reverse causality.^47,48^ In all regions except the Middle East and north Africa, optimal breastfeeding has increased slightly.^49^ However, any benefits of these improvements in reducing obesity are likely to have been overwhelmed by much larger changes in other aspects of nutrition. The reasons for the small decline in obesity among women in France and Spain, which was also seen in middle-age and older ages (approximately age ≥60 years) in other studies that used measurement data,^50–52^ are not known but could be related to changes in eating and exercise, following changes in social norms and roles.^53^

Our findings have two implications. First, there is an urgent need for obesity prevention, supporting weight loss and reducing disease risk (via treatment of the mediators of its hazards, such as hypertension and hypercholesterolaemia) in those with obesity. Prevention and management are especially important because the age of onset of obesity has decreased, which increases the duration of exposure. Most efforts to prevent obesity have focused on individual behaviours or isolated changes to the built or food environment.^54^ These have had little impact on obesity prevalence, in part because healthy foods and participating in sports and other active lifestyles are not accessible or affordable for people with low income and autonomy.^55–59^ In the past decade, some countries have regulated marketing and taxed items such as sugar-sweetened beverages;^60^ the impacts on obesity prevalence are currently being evaluated.^61^ There are fewer policies that make healthy foods accessible and affordable, especially for people with low income and in communities where such foods are scarce. Unaffordability and inaccessibility of healthy foods and opportunities for play and sports leads to inequalities in obesity, and could limit the impact of policies that target unhealthy foods.^58,59,62,63^ New pharmacological treatment of obesity, although promising, is likely to have a low impact globally in the short-term, due to high cost and the absence of generalisable clinical guidelines.

Second, the remaining burden of underweight should be tackled, especially in south and southeast Asia, and parts of Africa, where food insecurity persists.^64,65^ Our results show that this need cannot be considered in isolation, because the underweight–obesity transition can occur rapidly, leaving their combined burden unchanged or higher. A healthy transition away from high underweight prevalence while limiting the rise in obesity, consistent with the Sustainable Development Goal Target 2.2, requires improving access to healthy and nutritious foods. This is particularly important because both poverty and the cost of food, especially nutrient-rich foods, have increased since the COVID-19 pandemic and the war in Ukraine.^65–67^ Together with the adverse impact of climate change on food production and supply, these factors risk worsening both underweight and obesity through a combination of underconsumption in some countries and households, and a switch to less healthy foods in others.^68,69^ To engender a healthy transition, economic and agricultural policies are needed that tackle poverty and improve food security.^70^ As these macro policies are implemented, there is an urgent need for programmes that enhance healthy nutrition, such as targeted cash transfers, food assistance as subsidies or vouchers for healthy foods, free healthy school meals, and primary care-based nutritional interventions.^71–75^

## Supplementary Material

Appendix

## Data Availability

Age-standardised and age-specific results of this study, as well as the computer code and input data for the analyses, can be downloaded from https://www.ncdrisc.org. Computer code and input data are also available from the Zenodo repository (https://doi.org/10.5281/zenodo.10534960). Input data are provided where permitted by data governance and sharing arrangements; contact information is provided for other data sources.

## Research in context

### Evidence before this study

We searched MEDLINE (via PubMed) for articles published from database inception up to Sept 25, 2023, with no language restrictions, using the following search terms: (((“obesity”[mh:noexp] OR “overweight”[mh:noexp] OR “overnutrition”[mh:noexp]) AND (“thinness”[mh:noexp] OR “malnutrition”[mh:noexp])) OR (“double burden” AND “malnutrition”)) AND (“Health Surveys”[mh] OR “Epidemiological Monitoring”[mh] OR “Prevalence”[mh]) AND (“global^*^” OR “worldwide”) NOT “patient^*^”[Title] NOT Comment[ptyp] NOT Case Reports[ptyp]. Articles were screened to include measured data on height and weight, collected from probabilistic samples of national, subnational, or community populations aged 5 years and older. We found two studies that reported the prevalence of underweight and obesity in adults, one of which also reported the prevalence of thinness and obesity in school-aged children and adolescents, for all countries in the world. These outcomes were reported separately, and neither their combination nor their relative sizes were evaluated. The most recent data for both age groups were from 2016. We found eight studies on the double burden of malnutrition in low-income and middle-income countries, using data mostly from Demographic and Health Surveys or Global School-based Student Health Surveys. These studies covered only women of reproductive age or school-aged children and adolescents, and few assessed change over time. We found two other studies that covered multiple world regions but they did not report country-level prevalence or assess change over time. These studies defined double burden of malnutrition at the level of the individual (eg, combination of stunting and obesity), household (eg, presence of underweight and obesity in different members of the same household), or country, depending on the study. None reported for all countries in the world.

### Added value of this study

This study uses thousands of high-quality population-based studies, and presents consistent and up-to-date estimates of underweight, thinness, and obesity from late childhood through to adulthood, separately and in combination, for all countries in the world. It also analyses how the two components have contributed to the change in their combined burden over a period of more than three decades, 1990–2022, during which major changes occurred in food policies and programmes and nutrition.

### Implications of all the available evidence

The combined prevalence of underweight and obesity has increased in most countries since 1990, due to the rise in obesity surpassing the decline in underweight. Exceptions were most countries in south Asia, and some in southeast Asia and sub-Saharan Africa, where a decrease in the prevalence of underweight led to a decrease in the combined burden. This transition to obesity dominance was already apparent in adults in 1990 in much of the world, and has followed in school-aged children and adolescents. There is a need throughout the world for social and agricultural policies and food programmes that address the remaining burden of underweight while curbing and reversing the rise in obesity by enhancing access to healthy and nutritious foods.
