# Supplementary material for: Worldwide trends in underweight and obesity from 1990 to 2022: a pooled analysis of 3663 population-representative studies with 222 million children, adolescents, and adults
Source: Lancet. Author manuscript; Available in PMC 2024 Mar 26. (PMC7615769; doi:10.1016/S0140-6736(23)02750-2)
Supplement: Appendix [file EMS194840-supplement-Appendix.pdf]

THE LANCET

## Supplementary appendix

This appendix formed part of the original submission and has been peer reviewed. We post it as supplied by the authors.

Supplement to: NCD Risk Factor Collaboration (NCD-RisC). Worldwide trends in underweight and obesity from 1990 to 2022: a pooled analysis of 3663 population-representative studies with 222 million children, adolescents, and adults. *Lancet* 2024; published online Feb 29. https://doi.org/10.1016/S0140-6736(23)02750-2.

# Table of Contents

**Appendix Text 1.** NCD Risk Factor Collaboration (NCD-RisC) .......... 1

**Appendix Text 2.** Data sources .......... 47

**Appendix Text 3.** Statistical methods .......... 52

**Appendix Text 4.** Comparison with previous studies .......... 65

**Appendix Table 1.** Data sources used in the analysis. .......... 66

**Appendix Table 2.** List of analysis regions and "super-regions", and countries in each region. .......... 135

**Appendix Figure 1.** Number of data sources used in this analysis, by country. .......... 137

**Appendix Figure 2.** Number of data sources used in this analysis, by region and year. .......... 139

**Appendix Figure 3**. Change in age-standardised prevalence of underweight and obesity, for adults. .......... 141

**Appendix Figure 4.** Age-standardised prevalence of all BMI categories in 1990 and 2022, for adults. .......... 143

**Appendix Figure 5.** Age-standardised prevalence of underweight in 1990 and 2022, its change from 1990 to 2022, and posterior probability that the prevalence increased from 1990 to 2022, for adults. .......... 146

**Appendix Figure 6.** Age-standardised prevalence of obesity in 1990 and 2022, its change from 1990 to 2022, and posterior probability that the prevalence increased from 1990 to 2022, for adults. .......... 149

**Appendix Figure 7.** Posterior probability that age-standardised combined prevalence of underweight and obesity increased from 1990 to 2022, for adults. .......... 152

**Appendix Figure 8.** Change in age-standardised proportion of double burden composed of obesity and posterior probability that the proportion increased from 1990 to 2022, for adults. .......... 154

**Appendix Figure 9.** Combined prevalence of underweight and obesity for adults, by age group. .......... 156

**Appendix Figure 10.** Age-standardised proportion of the combined burden of underweight and obesity composed of obesity in 1990 and 2022, in relation to the uncertainty of the proportion measured by posterior standard deviation, for adults. .......... 163

**Appendix Figure 11.** Proportion of double burden composed of obesity for adults, by age group .......... 165

**Appendix Figure 12.** Change in age-standardised underweight, obesity, combined burden, and the proportion of combined burden composed of obesity from 1990 to 2022, in relation to the uncertainty of the change measured by posterior standard deviation, for adults. .......... 169

**Appendix Figure 13.** Contributions of the change in age-standardised underweight and obesity prevalence to their combined prevalence for adults, by age group. .......... 172

**Appendix Figure 14.** Change in composition of double burden from 1990 to 2022 for adults, by age group. .......... 176

**Appendix Figure 15.** Change in age-standardised prevalence of thinness and obesity, for school-aged children and adolescents. .......... 180

**Appendix Figure 16.** Age-standardised prevalence of all BMI categories in 1990 and 2022, for school-aged children and adolescents. .......... 182

**Appendix Figure 17.** Age-standardised prevalence of thinness in 1990 and 2022, its change from 1990 to 2022, and posterior probability that the prevalence increased from 1990 to 2022, for school-aged children and adolescents. .......... 185

**Appendix Figure 18.** Age-standardised prevalence of obesity in 1990 and 2022, its change from 1990 to 2022, and posterior probability that the prevalence increased from 1990 to 2022, for school-aged children and adolescents. .......... 188

**Appendix Figure 19.** Posterior probability that the age-standardised combined prevalence of thinness and obesity increased from 1990 to 2022, for school-aged children and adolescents. .......191

**Appendix Figure 20.** Change in age-standardised proportion of combined burden composed of obesity and posterior probability that the proportion increased from 1990 to 2022, for school-aged children and adolescents. ....................................................................................................193

**Appendix Figure 21.** Age-standardised proportion of the combined burden of thinness and obesity composed of obesity in 1990 and 2022, in relation to the uncertainty of the proportion measured by posterior standard deviation, for school-aged children and adolescents. ..........................................195

**Appendix Figure 22.** Change in age-standardised thinness, obesity, combined burden, and the proportion of combined burden composed of obesity from 1990 to 2022, in relation to the uncertainty of the change measured by posterior standard deviation, for school-aged children and adolescents. ....................................................................................................................................197

**Appendix Figure 23.** Comparison of age-standardised prevalence of thinness in school-aged children and adolescents and of underweight in adults. ....................................................................200

**Appendix Figure 24.** Comparison of age-standardised prevalence of obesity in school-aged children and adolescents and in adults.....................................................................................................202

**Appendix Figure 25.** Age standardised prevalence of thinness, obesity and their combined burden for school-aged children and adolescents, and underweight, obesity and their combined burden for adults, from 1990 to 2022, by country.....................................................................................204

**References**.............................................................................................................................405

**Appendix Text 1.** NCD Risk Factor Collaboration (NCD-RisC)

Pooled Analysis and Writing (* equal contribution; joint first authors)

Nowell H Phelps (MMath; Imperial College London, UK)*; Rosie K Singleton (MSc; Imperial College London, UK)*; Bin Zhou (PhD; Imperial College London, UK)*; Rachel A Heap (MPH; Imperial College London, UK); Anu Mishra (PhD; Bill & Melinda Gates Foundation, USA); James E Bennett (PhD; Imperial College London, UK); Prof Christopher J Paciorek (PhD; University of California Berkeley, USA); Victor PF Lhoste (MSc; Imperial College London, UK); Rodrigo M Carrillo-Larco (MD; Emory University, USA); Gretchen A Stevens (ScD; World Health Organization, Switzerland); Andrea Rodriguez-Martinez (PhD; Imperial College London, UK); Honor Bixby (PhD; University of Essex, UK); James Bentham (PhD; University of Kent, UK); Prof Mariachiara Di Cesare (PhD; University of Essex, UK); Prof Goodarz Danaei (ScD; Harvard TH Chan School of Public Health, USA); Archie W Rayner (MSci; Imperial College London, UK); Ana Barradas-Pires (MD; Imperial College London, UK); Melanie J Cowan (MPH; World Health Organization, Switzerland); Stefan Savin (MPH; World Health Organization, Switzerland); Leanne M Riley (MSc; World Health Organization, Switzerland); Prof Carlos A Aguilar-Salinas (DSc; Instituto Nacional de Ciencias Médicas y Nutrición, Mexico); Jennifer L Baker (PhD; Copenhagen University Hospital - Bispebjerg and Frederiksberg, Denmark); Prof Amina Barkat (PhD; Mohammed V University, Morocco); Prof Zulfiqar A Bhutta (PhD; The Hospital for Sick Children, Canada; The Aga Khan University, Pakistan); Francesco Branca (PhD; World Health Organization, Switzerland); Roberta B Caixeta (MHS; Pan American Health Organization, USA); Sarah Cuschieri (PhD; University of Malta, Malta); Prof Farshad Farzadfar (MD; World Health Organization, Switzerland); Shubash Ganapathy (PhD; National Institute of Health, Malaysia); Nayu Ikeda (PhD; National Institutes of Biomedical Innovation, Health and Nutrition, Japan); Prof Violeta Iotova (PhD; Medical University - Varna, Bulgaria); Prof Andre P Kengne (PhD; South African Medical Research Council, South Africa); Prof Young-Ho Khang (MD; Seoul National University College of Medicine, Republic of Korea); Prof Avula Laxmaiah (PhD; ICMR - National Institute of Nutrition, India); Prof Hsien-Ho Lin (MD; National Taiwan University, Taiwan); Prof Jun Ma

(PhD; Peking University, China); Prof Jean Claude N Mbanya (PhD; University of Yaoundé 1, Cameroon); Prof J Jaime Miranda (PhD; University of Sydney, Australia); Rajendra Pradeepa (PhD; Madras Diabetes Research Foundation, India); Prof Fernando Rodríguez-Artalejo (MD; Universidad Autónoma de Madrid CIBERESP, Spain); Maroje Sorić (PhD; University of Zagreb, Croatia; University of Ljubljana, Slovenia); Maria Turley (MSc; Ministry of Health, New Zealand); Limin Wang (MPH; Chinese Center for Disease Control and Prevention, China); Karen Webster-Kerr (PhD; Ministry of Health and Wellness, Jamaica); Prof Majid Ezzati (FMedSci; Imperial College London, UK; University of Ghana, Ghana)

Country and Regional Data (* equal contribution; listed alphabetically)

Julie Aarestrup (Copenhagen University Hospital - Bispebjerg and Frederiksberg, Denmark)*; Leandra Abarca-Gómez (Caja Costarricense de Seguro Social, Costa Rica)*; Mohsen Abbasi-Kangevari (Non-Communicable Diseases Research Center, Iran)*; Ziad A Abdeen (Al-Quds University, State of Palestine)*; Shynar Abdrakhmanova (National Center of Public Health, Kazakhstan)*; Suhaila Abdul Ghaffar (Ministry of Health, Malaysia)*; Hanan F Abdul Rahim (Qatar University, Qatar)*; Zulfiya Abdurrahmonova (Ministry of Health and Social Protection, Tajikistan)*; Niveen M Abu-Rmeileh (Birzeit University, State of Palestine)*; Jamila Abubakar Garba (Usmanu Danfodiyo University Teaching Hospital, Nigeria)*; Benjamin Acosta-Cazares (Instituto Mexicano del Seguro Social, Mexico)*; Ishag Adam (Qassim University, Saudi Arabia)*; Marzena Adamczyk (RehaKlinika, Poland)*; Robert J Adams (Flinders University, Australia)*; Seth Adu-Afarwuah (University of Ghana, Ghana)*; Wichai Aekplakorn (Mahidol University, Thailand)*; Kaosar Afsana (BRAC James P Grant School of Public Health, Bangladesh)*; Shoaib Afzal (University of Copenhagen, Denmark; Copenhagen University Hospital, Denmark)*; Valirie N Agbor (University of Oxford, UK)*; Imelda A Agdeppa (Food and Nutrition Research Institute, Philippines)*; Javad Aghazadeh-Attari (Urmia University of Medical Sciences, Iran)*; Åsa Ågren (Umeå University, Sweden)*; Hassan Aguenaou (Ibn Tofail University, Morocco)*; Carlos A Aguilar-Salinas (Instituto Nacional de Ciencias Médicas y Nutrición, Mexico)*; Charles Agyemang (University of Amsterdam, The Netherlands)*;

Mohamad Hasnan Ahmad (Ministry of Health, Malaysia)*; Noor Ani Ahmad (Ministry of Health, Malaysia)*; Ali Ahmadi (Shahrekord University of Medical Sciences, Iran)*; Naser Ahmadi (Non-Communicable Diseases Research Center, Iran)*; Nastaran Ahmadi (Shahid Sadoughi University of Medical Sciences, Iran)*; Imran Ahmed (The Aga Khan University, Pakistan)*; Soheir H Ahmed (University of Hargeisa, Somaliland)*; Wolfgang Ahrens (Leibniz Institute for Prevention Research and Epidemiology - BIPS, Germany)*; Gulmira Aitmurzaeva (Republican Center for Health Promotion, Kyrgyzstan)*; Kamel Ajlouni (The National Center for Diabetes, Endocrinology and Genetics, Jordan)*; Hazzaa M Al-Hazzaa (Princess Nourah bint Abdulrahman University, Saudi Arabia)*; Halima Al-Hinai (Ministry of Health, Oman)*; Badreya Al-Lahou (Kuwait Institute for Scientific Research, Kuwait)*; Jawad A Al-Lawati (Ministry of Health, Oman)*; Rajaa Al-Raddadi (King Abdulaziz University, Saudi Arabia)*; Deena Al Asfoor (World Health Organization Regional Office for the Eastern Mediterranean, Egypt)*; Huda M Al Hourani (The Hashemite University, Jordan)*; Nawal M Al Qaoud (Ministry of Health, Kuwait)*; Monira Alarouj (Dasman Diabetes Institute, Kuwait)*; Fadia AlBuhairan (Aldara Hospital and Medical Center, Saudi Arabia)*; Shahla AlDhukair (King Abdullah International Medical Research Center, Saudi Arabia)*; Maryam A Aldwairji (Ministry of Health, Kuwait)*; Sylvia Alexius (Universidade Federal da Integração Latino-Americana, Brazil)*; Mohamed M Ali (World Health Organization, Switzerland)*; Anna V Alieva (Ministry of Health, Uzbekistan)*; Abdullah Alkandari (Dasman Diabetes Institute, Kuwait)*; Ala'a Alkerwi (Luxembourg Institute of Health, Luxembourg)*; Buthaina M Alkhatib (The Hashemite University, Jordan)*; Kristine Allin (Copenhagen University Hospital - Bispebjerg and Frederiksberg, Denmark)*; Shaker A Alomary (Ministry of Health, Saudi Arabia)*; Husam F Alomirah (Kuwait Institute for Scientific Research, Kuwait)*; Arwa M Alshangiti (Ministry of Health, Saudi Arabia)*; Mar Alvarez-Pedrerol (Barcelona Institute for Global Health CIBERESP, Spain)*; Eman Aly (World Health Organization Regional Office for the Eastern Mediterranean, Egypt)*; Deepak N Amarapurkar (Bombay Hospital and Medical Research Centre, India)*; Pilar Amiano Etxezarreta (Departamento de Salud del Gobierno Vasco, Spain)*; John Amoah (Ghana Health Service, Ghana)*; Norbert Amougou (UMR CNRS-

MNHN 7206, France)*; Philippe Amouyel (University of Lille, France; Lille University Hospital, France)*; Lars Bo Andersen (Western Norway University of Applied Sciences, Norway)*; Sigmund A Anderssen (Norwegian School of Sport Sciences, Norway)*; Odysseas Androutsos (University of Thessaly, Greece)*; Lars Ängquist (University of Copenhagen, Denmark)*; Ranjit Mohan Anjana (Madras Diabetes Research Foundation, India)*; Alireza Ansari-Moghaddam (Health Promotion Research Center, Iran)*; Elena Anufrieva (Yekaterinburg State Medical Academy, Russia)*; Hajer Aounallah-Skhiri (National Institute of Public Health, Tunisia)*; Joana Araújo (Institute of Public Health of the University of Porto, Portugal)*; Inger Ariansen (Norwegian Institute of Public Health, Norway)*; Tahir Aris (Ministry of Health, Malaysia)*; Raphael E Arku (University of Massachusetts Amherst, USA)*; Nimmathota Arlappa (ICMR - National Institute of Nutrition, India)*; Krishna K Aryal (University of Bergen, Norway)*; Nega Assefa (Haramaya University, Ethiopia)*; Thor Aspelund (University of Iceland, Iceland)*; Felix K Assah (University of Yaoundé 1, Cameroon)*; Batyrbek Assembekov (Asfendiyarov Kazakh National Medical University, Kazakhstan)*; Maria Cecília F Assunção (Federal University of Pelotas, Brazil)*; May Soe Aung (University of Medicine 1, Myanmar)*; Correia Júnior Marco Aurélio de Valois (University of Pernambuco, Brazil)*; Juha Auvinen (Oulu University Hospital, Finland; University of Oulu, Finland)*; Mária Avdičová (Regional Authority of Public Health, Slovakia)*; Shina Avi (Tel Aviv University, Israel; Hebrew University of Jerusalem, Israel)*; Kishwar Azad (Diabetic Association of Bangladesh, Bangladesh)*; Ana Azevedo (University of Porto Medical School, Portugal)*; Mohsen Azimi-Nezhad (Neyshabur University of Medical Sciences, Iran)*; Fereidoun Azizi (Research Institute for Endocrine Sciences, Iran)*; Bontha V Babu (Indian Council of Medical Research, India)*; Flora Bacopoulou (National and Kapodistrian University of Athens, Greece)*; Maja Bæksgaard Jørgensen (National Institute of Public Health, Denmark)*; Azli Baharudin (Ministry of Health, Malaysia)*; Suhad Bahijri (King Abdulaziz University, Saudi Arabia)*; Izet Bajramovic (University of Sarajevo, Bosnia and Herzegovina)*; Marta Bakacs (National Centre for Public Health and Pharmacy, Hungary)*; Jennifer L Baker (Copenhagen University Hospital - Bispebjerg and Frederiksberg, Denmark)*; Nagalla Balakrishna (ICMR - National

Institute of Nutrition, India)*; Yulia Balanova (National Medical Research Centre for Therapy and Preventive Medicine, Russia)*; Mohamed Bamoshmoosh (University of Science and Technology, Yemen)*; Maciej Banach (Medical University of Lodz, Poland)*; José R Banegas (Universidad Autónoma de Madrid CIBERESP, Spain)*; Joanna Baran (University of Rzeszów, Poland)*; Rafał Baran (University of Rzeszów, Poland)*; Carlo M Barbagallo (University of Palermo, Italy)*; Valter Barbosa Filho (Ceara State University, Brazil)*; Alberto Barceló (University of Miami, USA)*; Maja Baretić (University Hospital Centre Zagreb, Croatia)*; Amina Barkat (Mohammed V University, Morocco)*; Joaquin Barnoya (Unidad de Cirugia Cardiovascular, Guatemala)*; Lena Barrera (Universidad del Valle, Colombia)*; Marta Barreto (National Institute of Health Doutor Ricardo Jorge, Portugal; NOVA University Lisbon, Portugal)*; Aluisio JD Barros (Federal University of Pelotas, Brazil)*; Mauro Virgílio Gomes Barros (University of Pernambuco, Brazil)*; Anna Bartosiewicz (University of Rzeszów, Poland)*; Abdul Basit (Baqai Institute of Diabetology and Endocrinology, Pakistan)*; Joao Luiz Bastos (Simon Fraser University, Canada)*; Iqbal Bata (Dalhousie University, Canada)*; Anwar M Batieha (Jordan University of Science and Technology, Jordan)*; Aline P Batista (Universidade Federal de Ouro Preto, Brazil)*; Rosangela L Batista (Federal University of Maranhão, Brazil)*; Zhamilya Battakova (National Center of Public Health, Kazakhstan)*; Louise A Baur (University of Sydney, Australia)*; Pascal M Bayauli (Cliniques Universitaires de Kinshasa, DR Congo)*; Robert Beaglehole (University of Auckland, New Zealand)*; Silvia Bel-Serrat (University College Dublin, Ireland)*; Antonisamy Belavendra (Christian Medical College Vellore, India)*; Habiba Ben Romdhane (University Tunis El Manar, Tunisia)*; Theodora Benedek (University of Medicine, Pharmacy, Sciences and Technology of Târgu-Mureș, Romania)*; Judith Benedics (Federal Ministry of Social Affairs, Health, Care and Consumer Protection, Austria)*; Mikhail Benet (Cafam University Foundation, Colombia)*; Gilda Estela Benitez Rolandi (Ministerio de Salud Pública y Bienestar Social, Paraguay)*; James E Bennett (Imperial College London, UK)*; Michaela Benzeval (University of Essex, UK)*; Elling Bere (University of Agder, Norway)*; Nicolas Berger (Sciensano, Belgium)*; Ingunn Holden Bergh (Norwegian Institute of Public Health, Norway)*; Yemane Berhane

(Addis Continental Institute of Public Health, Ethiopia)*; Salim Berkinbayev (Kazakh National Medical University, Kazakhstan)*; Antonio Bernabe-Ortiz (Universidad Cientifica del Sur, Peru)*; Gailute Bernotiene (Lithuanian University of Health Sciences, Lithuania)*; Ximena Berrios Carrasola (Pontificia Universidad Católica de Chile, Chile)*; Heloísa Bettiol (University of São Paulo, Brazil)*; Manfred E Beutel (Johannes Gutenberg University, Germany)*; Augustin F Beybey (University of Yaoundé 1, Cameroon)*; Jorge Bezerra (University of Pernambuco, Brazil)*; Aroor Bhagyalaxmi (B J Medical College, India)*; Sumit Bharadwaj (Chirayu Medical College, India)*; Santosh K Bhargava (Sunder Lal Jain Hospital, India)*; Zulfiqar A Bhutta (The Hospital for Sick Children, Canada; The Aga Khan University, Pakistan)*; Hongsheng Bi (Shandong University of Traditional Chinese Medicine, China)*; Yufang Bi (Shanghai Jiao-Tong University School of Medicine, China)*; Daniel Bia (Universidad de la República, Uruguay)*; Katia Biasch (University of Strasbourg, France)*; Elysée Claude Bika Lele (Institute of Medical Research and Medicinal Plant Studies, Cameroon)*; Mukharram M Bikbov (Ufa Eye Research Institute, Russia)*; Bihungum Bista (Nepal Health Research Council, Nepal)*; Dusko J Bjelica (University of Montenegro, Montenegro)*; Anne A Bjerregaard (Copenhagen University Hospital - Bispebjerg and Frederiksberg, Denmark)*; Peter Bjerregaard (University of Southern Denmark, Denmark)*; Espen Bjertness (University of Oslo, Norway)*; Marius B Bjertness (University of Oslo, Norway)*; Cecilia Björkelund (University of Gothenburg, Sweden)*; Katia V Bloch (Universidade Federal do Rio de Janeiro, Brazil)*; Anneke Blokstra (National Institute for Public Health and the Environment, The Netherlands)*; Moran Blychfeld Magnazu (University of Haifa, Israel; Ministry of Health, Israel)*; Simona Bo (University of Turin, Italy)*; Martin Bobak (University College London, UK)*; Lynne M Boddy (Liverpool John Moores University, UK)*; Bernhard O Boehm (Nanyang Technological University, Singapore)*; Jolanda MA Boer (National Institute for Public Health and the Environment, The Netherlands)*; Jose G Boggia (Universidad de la República, Uruguay)*; Elena Bogova (National Medical Research Center for Endocrinology, Russia)*; Carlos P Boissonnet (Centro de Educación Médica e Investigaciones Clínicas, Argentina)*; Stig E Bojesen (Copenhagen University Hospital,

Denmark; University of Copenhagen, Denmark)*; Marialaura Bonaccio (IRCCS Neuromed, Italy)*; Vanina Bongard (Toulouse University School of Medicine, France)*; Alice Bonilla-Vargas (Caja Costarricense de Seguro Social, Costa Rica)*; Matthias Bopp (University of Zurich, Switzerland)*; Herman Borghs (KU Leuven, Belgium)*; Steve Botomba (Epidemiology Center of Diabetes, DR Congo)*; Rupert RA Bourne (Cambridge University Hospitals, UK)*; Pascal Bovet (Ministry of Health, Seychelles; Unisanté, Switzerland)*; Khadichamo Boymatova (World Health Organization Country Office, Tajikistan)*; Lien Braeckevelt (Flemish Agency for Care and Health, Belgium)*; Lutgart Braeckman (Ghent University, Belgium)*; Marjolijn CE Bragt (FrieslandCampina, The Netherlands)*; Tasanee Braithwaite (King's College London, UK)*; Imperia Brajkovich (Universidad Central de Venezuela, Venezuela)*; Francesco Branca (World Health Organization, Switzerland)*; Juergen Breckenkamp (Bielefeld University, Germany)*; João Breda (World Health Organization Athens Quality of Care Office, Greece)*; Hermann Brenner (German Cancer Research Center, Germany)*; Lizzy M Brewster (University of Amsterdam, The Netherlands)*; Garry R Brian (The Fred Hollows Foundation, New Zealand)*; Yajaira Briceño (University of the Andes, Venezuela)*; Lacramioara Brinduse (Carol Davila University of Medicine and Pharmacy, Romania)*; Bettina Bringolf-Isler (Swiss Tropical and Public Health Institute, Switzerland)*; Miguel Brito (Instituto Politécnico de Lisboa, Portugal)*; Sinead Brophy (Swansea University, UK)*; Johannes Brug (National Institute for Public Health and the Environment, The Netherlands)*; Graziella Bruno (University of Turin, Italy)*; Anna Bugge (University College Copenhagen, Denmark)*; Marta Buoncristiano (World Health Organization Regional Office for Europe, Denmark)*; Genc Burazeri (University of Medicine, Albania)*; Con Burns (Munster Technological University, Ireland)*; Antonio Cabrera de León (Universidad de La Laguna, Spain)*; Joseph Cacciottolo (University of Malta, Malta)*; Hui Cai (Vanderbilt University, USA)*; Roberta B Caixeta (Pan American Health Organization, USA)*; Tilema Cama (Ministry of Health, Tonga)*; Christine Cameron (Canadian Fitness and Lifestyle Research Institute, Canada)*; José Camolas (Hospital Santa Maria, Portugal)*; Günay Can (Istanbul University - Cerrahpasa, Türkiye)*; Ana Paula C Cândido (Universidade Federal de Juiz de Fora, Brazil)*; Felicia Cañete (Ministry

of Public Health, Paraguay)*; Mario V Capanzana (Food and Nutrition Research Institute, Philippines)*; Naděžda Čapková (National Institute of Public Health, Czech Republic)*; Eduardo Capuano (Canopo Study Center - Salerno, Italy)*; Rocco Capuano (Canopo Study Center - Salerno, Italy)*; Vincenzo Capuano (Canopo Study Center - Salerno, Italy)*; Marloes Cardol (University of Groningen, The Netherlands)*; Viviane C Cardoso (University of São Paulo, Brazil)*; Axel C Carlsson (Karolinska Institutet, Sweden)*; Esteban Carmuega (Centro de Estudios Sobre Nutrición Infantil, Argentina)*; Rodrigo M Carrillo-Larco (Emory University, USA)*; Joana Carvalho (University of Porto, Portugal)*; José A Casajús (University of Zaragoza, Spain)*; Felipe F Casanueva (Santiago de Compostela University, Spain)*; Maribel Casas (Barcelona Institute for Global Health CIBERESP, Spain)*; Ertugrul Celikcan (Ministry of Health, Türkiye)*; Laura Censi (Council for Agricultural Research and Economics, Italy)*; Marvin Cervantes-Loaiza (Caja Costarricense de Seguro Social, Costa Rica)*; Juraci A Cesar (Federal University of Rio Grande, Brazil)*; Parinya Chamnan (Sanpasitthiprasong Regional Hospital, Thailand)*; Snehalatha Chamukuttan (India Diabetes Research Foundation, India)*; Angelique Chan (Duke-NUS Medical School, Singapore)*; Queenie Chan (Imperial College London, UK)*; Fadi J Charchar (Federation University Australia, Australia)*; Marie-Aline Charles (Paris Cité University, France)*; Himanshu K Chaturvedi (ICMR - National Institute of Medical Statistics, India)*; Nish Chaturvedi (University College London, UK)*; Norsyamlina Che Abdul Rahim (Ministry of Health, Malaysia)*; Miao Li Chee (Singapore Eye Research Institute, Singapore)*; Chien-Jen Chen (Academia Sinica, Taiwan)*; Fangfang Chen (Capital Institute of Pediatrics, China)*; Huashuai Chen (Xiangtan University, China)*; Long-Sheng Chen (Ministry of Health and Welfare, Taiwan)*; Shuohua Chen (Kailuan General Hospital, China)*; Zhengming Chen (University of Oxford, UK)*; Ching-Yu Cheng (National University of Singapore, Singapore)*; Yiling J Cheng (US Centers for Disease Control and Prevention, USA)*; Bahman Cheraghian (Ahvaz Jundishapur University of Medical Sciences, Iran)*; Angela Chetrit (The Gertner Institute for Epidemiology and Health Policy Research, Israel)*; Ekaterina Chikova-Iscener (National Centre of Public Health and Analyses, Bulgaria)*; Mai JM Chinapaw (VU University Medical Center, The Netherlands)*; Anne Chinnock (Universidad

de Costa Rica, Costa Rica)*; Arnaud Chiolero (University of Fribourg, Switzerland)*; Shu-Ti Chiou (National Taiwan University, Taiwan)*; Adela Chirita-Emandi (Victor Babes University of Medicine and Pharmacy, Romania)*; María-Dolores Chirlaque (CIBERESP, Spain)*; Belong Cho (Seoul National University, Republic of Korea)*; Kaare Christensen (University of Southern Denmark, Denmark)*; Diego G Christofaro (Universidade Estadual Paulista, Brazil)*; Jerzy Chudek (Medical University of Silesia, Poland)*; Renata Cifkova (Charles University, Czech Republic; Thomayer University Hospital, Czech Republic)*; Michelle Cilia (Primary Health Care, Malta)*; Eliza Cinteza (Carol Davila University of Medicine and Pharmacy, Romania)*; Massimo Cirillo (University of Salerno, Italy)*; Frank Claessens (KU Leuven, Belgium)*; Philip Clare (University of Sydney, Australia)*; Janine Clarke (Statistics Canada, Canada)*; Els Clays (Ghent University, Belgium)*; Emmanuel Cohen (UMR CNRS-MNHN 7206, France)*; Cosmin R Cojocaru (Carol Davila University of Medicine and Pharmacy, Romania)*; Sandra Colorado-Yohar (CIBERSP, Spain)*; Laura-María Compañ-Gabucio (Alicante Institute for Health and Biomedical Research, Spain)*; Hans Concin (Agency for Preventive and Social Medicine, Austria)*; Susana C Confortin (University of the Extreme South of Santa Catarina, Brazil)*; Cyrus Cooper (University of Southampton, UK)*; Tara C Coppinger (Munster Technological University, Ireland)*; Eva Corpeleijn (University of Groningen, The Netherlands)*; Lilia Yadira Cortés (Pontificia Universidad Javeriana, Colombia)*; Simona Costanzo (IRCCS Neuromed, Italy)*; Dominique Cottel (Institut Pasteur de Lille, France)*; Chris Cowell (University of Sydney, Australia)*; Cora L Craig (Canadian Fitness and Lifestyle Research Institute, Canada)*; Amelia C Crampin (Malawi Epidemiology and Intervention Research Unit, Malawi)*; Amanda J Cross (Imperial College London, UK)*; Ana B Crujeiras (CIBEROBN, Spain)*; Juan J Cruz (Universidad Autónoma de Madrid CIBERESP, Spain)*; Tamás Csányi (Hungarian University of Sports Science, Hungary)*; Semánová Csilla (University of Debrecen, Hungary)*; Alexandra M Cucu (National Institute of Public Health, Romania; University of Medicine and Pharmacy, Romania)*; Liufu Cui (Kailuan General Hospital, China)*; Felipe V Cureau (Universidade Federal do Rio Grande do Norte, Brazil)*; Sarah Cuschieri (University of Malta, Malta)*; Ewelina Czenczek-Lewandowska

(University of Rzeszów, Poland)*; Graziella D'Arrigo (National Research Council, Italy)*; Eleonora d'Orsi (Federal University of Santa Catarina, Brazil)*; Alanna G da Silva (Universidade Federal de Minas Gerais, Brazil)*; Liliana Dacica (Eftimie Murgu University Resita, Romania)*; Christina C Dahm (Aarhus University, Denmark)*; Jean Dallongeville (Institut Pasteur de Lille, France)*; Albertino Damasceno (Eduardo Mondlane University, Mozambique)*; Camilla T Damsgaard (University of Copenhagen, Denmark)*; Goodarz Danaei (Harvard TH Chan School of Public Health, USA)*; Rachel Dankner (The Gertner Institute for Epidemiology and Health Policy Research, Israel)*; Thomas M Dantoft (Copenhagen University Hospital - Bispebjerg and Frederiksberg, Denmark)*; Parasmani Dasgupta (Indian Statistical Institute, India)*; Saeed Dastgiri (Tabriz Health Services Management Research Center, Iran)*; Luc Dauchet (University of Lille, France; Lille University Hospital, France)*; Kairat Davletov (Asfendiyarov Kazakh National Medical University, Kazakhstan)*; Francisco de Assis Guedes de Vasconcelos (Federal University of Santa Catarina, Brazil)*; Maria Alice Altenburg de Assis (Federal University of Santa Catarina, Brazil)*; Guy De Backer (Ghent University, Belgium)*; Dirk De Bacquer (Ghent University, Belgium)*; Jaco De Bacquer (Ghent University, Belgium)*; Jeroen de Bont (Karolinska Institutet, Sweden)*; Amalia De Curtis (IRCCS Neuromed, Italy)*; Patrícia de Fragas Hinnig (Federal University of Santa Catarina, Brazil)*; Giovanni de Gaetano (IRCCS Neuromed, Italy)*; Stefaan De Henauw (Ghent University, Belgium)*; Pilar De Miguel-Etayo (CIBEROBN, Spain; University of Zaragoza, Spain)*; Jan-Walter De Neve (University of Heidelberg, Germany)*; Paula Duarte de Oliveira (Federal University of Pelotas, Brazil)*; David De Ridder (Geneva University Hospitals, Switzerland)*; Karin De Ridder (Sciensano, Belgium)*; Susanne R de Rooij (University of Amsterdam, The Netherlands)*; Ana Carolina MGN de Sá (Universidade Federal de Minas Gerais, Brazil)*; Delphine De Smedt (Ghent University, Belgium)*; Mohan Deepa (Madras Diabetes Research Foundation, India)*; Alexander D Deev (National Medical Research Centre for Therapy and Preventive Medicine, Russia)*; Vincent Jr DeGennaro (Innovating Health International, Haiti)*; Hélène Delisle (University of Montreal, Canada)*; Francis Delpeuch (French National Research Institute for Sustainable

Development, France)*; Stefaan Demarest (Sciensano, Belgium)*; Elaine Dennison (University of Southampton, UK)*; Katarzyna Dereń (University of Rzeszów, Poland)*; Valérie Deschamps (French Public Health Agency, France)*; Ruslan D Devrishov (Astrakhan State Medical University, Russia)*; Meghnath Dhimal (Nepal Health Research Council, Nepal)*; Augusto Di Castelnuovo (Mediterranea Cardiocentro, Italy)*; Juvenal Soares Dias-da-Costa (Universidade do Vale do Rio dos Sinos, Brazil)*; María Elena Díaz-Sánchez (National Institute of Hygiene, Epidemiology and Microbiology, Cuba)*; Alejandro Diaz (National Council of Scientific and Technical Research, Argentina)*; Pedro Díaz Fernández (Servicio Canario de la Salud del Gobierno de Canarias, Spain)*; María Pilar Díez Ripollés (Consejería de Salud del Gobierno de La Rioja, Spain)*; Zivka Dika (University of Zagreb, Croatia)*; Shirin Djalalinia (Ministry of Health and Medical Education, Iran)*; Visnja Djordjic (University of Novi Sad, Serbia)*; Ha TP Do (National Institute of Nutrition, Vietnam)*; Annette J Dobson (University of Queensland, Australia)*; Liria Dominguez (Instituto de Investigación Nutricional, Peru)*; Maria Benedetta Donati (IRCCS Neuromed, Italy)*; Chiara Donfrancesco (Istituto Superiore di Sanità, Italy)*; Guanghui Dong (Sun Yat-sen University, China)*; Yanhui Dong (Peking University, China)*; Silvana P Donoso (Universidad de Cuenca, Ecuador)*; Angela Döring (Helmholtz Zentrum München, Germany)*; Maria Dorobantu (Carol Davila University of Medicine and Pharmacy, Romania)*; Ahmad Reza Dorosty (Tehran University of Medical Sciences, Iran)*; Marcus Dörr (University Medicine Greifswald, Germany)*; Kouamelan Doua (Ministère de la Santé et de l'Hygiène Publique, Côte d'Ivoire)*; Nico Dragano (University Hospital Düsseldorf, Germany)*; Wojciech Drygas (Lazarski University, Poland)*; Shufa Du (University of North Carolina at Chapel Hill, USA)*; Jia Li Duan (Beijing Center for Disease Prevention and Control, China)*; Charmaine A Duante (Food and Nutrition Research Institute, Philippines)*; Priscilla Duboz (IRL 3189 ESS, France)*; Vesselka L Duleva (National Centre of Public Health and Analyses, Bulgaria)*; Virginija Dulskiene (Lithuanian University of Health Sciences, Lithuania)*; Samuel C Dumith (Federal University of Rio Grande, Brazil)*; Anar Dushpanova (Scuola Superiore Sant'Anna, Italy; Al-Farabi Kazakh National University, Kazakhstan)*; Terence Dwyer (Murdoch Children's Research Institute, Australia)*; Azhar

Dyussupova (Semey Medical University, Kazakhstan)*; Vilnis Dzerve (University of Latvia, Latvia)*; Elzbieta Dziankowska-Zaborszczyk (Medical University of Lodz, Poland)*; Narges Ebrahimi (Non-Communicable Diseases Research Center, Iran)*; Guadalupe Echeverría (Pontificia Universidad Católica de Chile, Chile)*; Ricky Eddie (Ministry of Health and Medical Services, Solomon Islands)*; Ebrahim Eftekhar (Hormozgan University of Medical Sciences, Iran)*; Vasiliki Efthymiou (National and Kapodistrian University of Athens, Greece)*; Eruke E Egbagbe (University of Benin, Nigeria)*; Robert Eggertsen (University of Gothenburg, Sweden)*; Sareh Eghtesad (Tehran University of Medical Sciences, Iran)*; Gabriele Eiben (University of Skövde, Sweden)*; Ulf Ekelund (Norwegian School of Sport Sciences, Norway)*; Mohammad El-Khateeb (The National Center for Diabetes, Endocrinology and Genetics, Jordan)*; Laila El Ammari (Ministry of Health, Morocco)*; Jalila El Ati (National Institute of Nutrition and Food Technology, Tunisia)*; Denise Eldemire-Shearer (The University of the West Indies, Jamaica)*; Paul Elliott (Imperial College London, UK)*; Ofem Enang (University of Calabar, Nigeria)*; Ronit Endevelt (University of Haifa, Israel; Ministry of Health, Israel)*; Reina Engle-Stone (University of California Davis, USA)*; Rajiv T Erasmus (University of Stellenbosch, South Africa)*; Cihangir Erem (Karadeniz Technical University, Türkiye)*; Gul Ergor (Dokuz Eylul University, Türkiye)*; Louise Eriksen (University of Southern Denmark, Denmark)*; Johan G Eriksson (University of Helsinki, Finland)*; Jorge Escobedo-de la Peña (Instituto Mexicano del Seguro Social, Mexico)*; Saeid Eslami (Mashhad University of Medical Sciences, Iran)*; Ali Esmaeili (Rafsanjan University of Medical Sciences, Iran)*; Alun Evans (Queen’s University of Belfast, UK)*; Roger G Evans (Monash University, Australia)*; David Faeh (University of Zurich, Switzerland)*; Guy Fagherazzi (Luxembourg Institute of Health, Luxembourg)*; Ildar Fakhradiyev (Kazakh National Medical University, Kazakhstan)*; Albina A Fakhretdinova (Ufa Eye Research Institute, Russia)*; Caroline H Fall (University of Southampton, UK)*; Elnaz Faramarzi (Tabriz University of Medical Sciences, Iran)*; Mojtaba Farjam (Fasa University of Medical Sciences, Iran)*; Victoria Farrugia Sant’Angelo (Primary Health Care, Malta)*; Farshad Farzadfar (World Health Organization, Switzerland)*; Yosef Farzi (Non-Communicable Diseases Research Center, Iran)*; Mohammad Reza Fattahi

(Shiraz University of Medical Sciences, Iran)*; Asher Fawwad (Baqai Medical University, Pakistan)*; Wafaie W Fawzi (Harvard TH Chan School of Public Health, USA)*; Francisco J Felix-Redondo (Centro de Salud Villanueva Norte, Spain)*; Trevor S Ferguson (The University of the West Indies, Jamaica)*; Romulo A Fernandes (Universidade Estadual Paulista, Brazil)*; Daniel Fernández-Bergés (Hospital Don Benito-Villanueva de la Serena, Spain)*; Daniel Ferrante (Ministry of Health, Argentina)*; Thomas Ferrao (Statistics Canada, Canada)*; Gerson Ferrari (Universidad de Santiago de Chile, Chile)*; Marika Ferrari (Council for Agricultural Research and Economics, Italy)*; Marco M Ferrario (University of Insubria, Italy)*; Catterina Ferreccio (Pontificia Universidad Católica de Chile, Chile)*; Haroldo S Ferreira (Federal University of Alagoas, Brazil)*; Eldridge Ferrer (Food and Nutrition Research Institute, Philippines)*; Jean Ferrieres (Toulouse University School of Medicine, France)*; Thamara Hubler Figueiró (Federal University of Santa Catarina, Brazil)*; Anna Fijalkowska (Institute of Mother and Child, Poland)*; Günther Fink (Swiss Tropical and Public Health Institute, Switzerland; University of Basel, Switzerland)*; Mauro Fisberg (Hospital Infantil Sabará, Brazil)*; Krista Fischer (University of Tartu, Estonia)*; Leng Huat Foo (Universiti Sains Malaysia, Malaysia)*; Maria Forsner (Umeå University, Sweden)*; Edward F Fottrell (University College London, UK)*; Heba M Fouad (World Health Organization Regional Office for the Eastern Mediterranean, Egypt)*; Damian K Francis (The University of the West Indies, Jamaica)*; Maria do Carmo Franco (Federal University of São Paulo, Brazil)*; Zlatko Fras (University Clinical Centre Ljubljana, Slovenia)*; Brooklyn Fraser (University of Tasmania, Australia)*; Guillermo Frontera (Hospital Universitario Son Espases, Spain)*; Flavio D Fuchs (Hospital de Clinicas de Porto Alegre, Brazil)*; Sandra C Fuchs (Universidade Federal do Rio Grande do Sul, Brazil)*; Isti I Fujiati (Universitas Sumatera Utara, Indonesia)*; Yuki Fujita (Kindai University, Japan)*; Matsuda Fumihiko (Kyoto University, Japan)*; Viktoriya Furdela (I. Horbachevsky Ternopil National Medical University, Ukraine)*; Takuro Furusawa (Kyoto University, Japan)*; Stefan Adela Gabriela (Calafat Municipal Hospital, Romania)*; Zbigniew Gaciong (Medical University of Warsaw, Poland)*; Mihai Gafencu (Victor Babes University of Medicine and Pharmacy, Romania)*; Manuel Galán Cuesta (Consejería de Sanidad del

Gobierno de Cantabria, Spain)*; Andrzej Galbarczyk (Jagiellonian University Medical College, Poland)*; Sonya V Galcheva (Medical University - Varna, Bulgaria)*; Henrike Galenkamp (University of Amsterdam, The Netherlands)*; Daniela Galeone (Ministero della Salute DG Prevenzione Sanitaria, Italy)*; Myriam Galfo (Council for Agricultural Research and Economics, Italy)*; Fabio Galvano (University of Catania, Italy)*; Jingli Gao (Kailuan General Hospital, China)*; Pei Gao (Peking University, China)*; Manoli Garcia-de-la-Hera (CIBERESP, Spain)*; María José García Mérida (Servicio Canario de la Salud del Gobierno de Canarias, Spain)*; Marta García Solano (Agencia Española de Seguridad Alimentaria y Nutrición, Spain)*; Dickman Gareta (Africa Health Research Institute, South Africa)*; Sarah P Garnett (University of Sydney, Australia)*; Jean-Michel Gaspoz (Geneva University Medical School, Switzerland)*; Magda Gasull (CIBERESP, Spain)*; Adroaldo Cesar Araujo Gaya (Universidade Federal do Rio Grande do Sul, Brazil)*; Anelise Reis Gaya (Universidade Federal do Rio Grande do Sul, Brazil)*; Andrea Gazzinelli (Universidade Federal de Minas Gerais, Brazil)*; Ulrike Gehring (Utrecht University, The Netherlands)*; Harald Geiger (Agency for Preventive and Social Medicine, Austria)*; Johanna M Geleijnse (Wageningen University, The Netherlands)*; Ronnie George (Medical Research Foundation, India)*; Eva Gerdts (University of Bergen, Norway)*; Ebrahim Ghaderi (Kurdistan University of Medical Sciences, Iran)*; Seyyed-Hadi Ghamari (Non-Communicable Diseases Research Center, Iran)*; Ali Ghanbari (Non-Communicable Diseases Research Center, Iran)*; Erfan Ghasemi (Non-Communicable Diseases Research Center, Iran)*; Oana-Florentina Gheorghe-Fronea (Carol Davila University of Medicine and Pharmacy, Romania)*; Alessandro Gialluisi (LUM University, Italy)*; Simona Giampaoli (Istituto Superiore di Sanità, Italy)*; Francesco Gianfagna (University of Insubria, Italy; Mediterranea Cardiocentro, Italy)*; Christian Gieger (Helmholtz Zentrum München, Germany)*; Tiffany K Gill (University of Adelaide, Australia)*; Jonathan Giovannelli (University of Lille, France; Lille University Hospital, France)*; Glen Gironella (Food and Nutrition Research Institute, Philippines)*; Aleksander Giwercman (Lund University, Sweden)*; Konstantinos Gkiouras (Aristotle University of Thessaloniki, Greece)*; Natalya Glushkova (Al-Farabi Kazakh National University, Kazakhstan; Asfendiyarov Kazakh National

Medical University, Kazakhstan)*; Ramesh Godara (Central University of Kerala, India)*; Justyna Godos (University of Catania, Italy)*; Sibel Gogen (Ministry of Health, Türkiye)*; Marcel Goldberg (Institut National de la Santé et de la Recherche Médicale, France; Paris Cité University, France)*; David Goltzman (McGill University, Canada)*; Georgina Gómez (Universidad de Costa Rica, Costa Rica)*; Jesús Humberto Gómez Gómez (Instituto Murciano de Investigación Biosanitaria Virgen de la Arrixaca, Spain)*; Luis F Gomez (Pontificia Universidad Javeriana, Colombia)*; Santiago F Gómez (Gasol Foundation, Spain; University of Lleida, Spain)*; Aleksandra Gomula (PASs Hirszfeld Institute of Immunology and Experimental Therapy, Poland)*; Bruna Gonçalves Cordeiro da Silva (Federal University of Pelotas, Brazil)*; Helen Gonçalves (Federal University of Pelotas, Brazil)*; Mauer Gonçalves (University Agostinho Neto, Angola)*; Ana D González-Alvarez (Kansas State University, USA)*; David A Gonzalez-Chica (University of Adelaide, Australia)*; Esther M González-Gil (University of Zaragoza, Spain)*; Marcela Gonzalez-Gross (Universidad Politécnica de Madrid, Spain)*; Margot González-Leon (Instituto Mexicano del Seguro Social, Mexico)*; Juan P González-Rivas (International Clinical Research Center, Czech Republic)*; Clicerio González-Villalpando (National Institute of Public Health, Mexico)*; María-Elena González-Villalpando (Centro de Estudios en Diabetes A.C., Mexico)*; Angel R Gonzalez (Universidad Autónoma de Santo Domingo, Dominican Republic)*; Frederic Gottrand (University of Lille, France)*; Antonio Pedro Graça (Ministry of Health, Portugal)*; Dušan Grafnetter (Institute for Clinical and Experimental Medicine, Czech Republic)*; Aneta Grajda (Children's Memorial Health Institute, Poland)*; Maria G Grammatikopoulou (University of Thessaly, Greece)*; Edward W Gregg (Imperial College London, UK; RCSI University of Medicine and Health Sciences, Ireland)*; Ronald D Gregor (Dalhousie University, Canada)*; Maria João Gregório (Ministry of Health, Portugal)*; Else Karin Grøholt (Norwegian Institute of Public Health, Norway)*; Anders Grøntved (University of Southern Denmark, Denmark)*; Giuseppe Grosso (University of Catania, Italy)*; Gabriella Gruden (University of Turin, Italy)*; Dongfeng Gu (National Center of Cardiovascular Diseases, China)*; Viviana Guajardo (International Life Science Institute, Argentina)*; Emanuela Gualdi-Russo (University of Ferrara, Italy)*; Pilar Guallar-Castillón

(Universidad Autónoma de Madrid CIBERESP, Spain)*; Andrea Gualtieri (Authority Sanitaria San Marino, San Marino)*; Elias F Gudmundsson (Icelandic Heart Association, Iceland)*; Vilmundur Gudnason (University of Iceland, Iceland)*; Maëlenn Guerchet (French National Research Institute for Sustainable Development, France)*; Ramiro Guerrero (Universidad Icesi, Colombia)*; Idris Guessous (Geneva University Hospitals, Switzerland)*; Andre L Guimaraes (State University of Montes Claros, Brazil)*; Unjali P Gujral (Emory University, USA)*; Martin C Gulliford (King's College London, UK)*; Johanna Gunnlaugsdottir (Icelandic Heart Association, Iceland)*; Marc J Gunter (International Agency for Research on Cancer, France)*; Xiu-Hua Guo (Capital Medical University, China)*; Yin Guo (Capital Medical University Beijing Tongren Hospital, China)*; Prakash C Gupta (Healis-Sekhsaria Institute for Public Health, India)*; Rajeev Gupta (Eternal Heart Care Centre and Research Institute, India)*; Oye Gureje (University of Ibadan, Nigeria)*; Mirjana A Gurinović (Capacity Development Network in Nutrition in Central and Eastern Europe, Serbia)*; Enrique Gutiérrez González (Agencia Española de Seguridad Alimentaria y Nutrición, Spain)*; Laura Gutierrez (Institute for Clinical Effectiveness and Health Policy, Argentina)*; Felix Gutzwiller (University of Zurich, Switzerland)*; Xinyi Gwee (National University of Singapore, Singapore)*; Seongjun Ha (National Health Insurance Service, Republic of Korea)*; Farzad Hadaegh (Prevention of Metabolic Disorders Research Center, Iran)*; Charalambos A Hadjigeorgiou (Research and Education Institute of Child Health, Cyprus)*; Rosa Haghshenas (Non-Communicable Diseases Research Center, Iran)*; Hamid Hakimi (Rafsanjan University of Medical Sciences, Iran)*; Jytte Halkjær (Danish Cancer Institute, Denmark)*; Ian R Hambleton (The University of the West Indies, Barbados)*; Behrooz Hamzeh (Kermanshah University of Medical Sciences, Iran)*; Willem A Hanekom (Africa Health Research Institute, South Africa)*; Dominique Hange (University of Gothenburg, Sweden)*; Abu AM Hanif (BRAC James P Grant School of Public Health, Bangladesh)*; Sari Hantunen (University of Eastern Finland, Finland)*; Jie Hao (Capital Medical University, China)*; Carla Menêses Hardman (Federal University of Pernambuco, Brazil)*; Louise Hardy (University of Sydney, Australia)*; Rachakulla Hari Kumar (ICMR - National Institute of Nutrition, India)*; Tina Harmer Lassen (National Institute of Public

Health, Denmark)*; Javad Harooni (Yasuj University of Medical Sciences, Iran)*; Seyed Mohammad Hashemi-Shahri (Zahedan University Of Medical Sciences, Iran)*; Maria Hassapidou (International Hellenic University, Greece)*; Jun Hata (Kyushu University, Japan)*; Teresa Haugsgjerd (University of Bergen, Norway)*; Alison J Hayes (University of Sydney, Australia)*; Jiang He (Tulane University, USA)*; Yuan He (National Research Institute for Health and Family Planning, China)*; Yuna He (Chinese Center for Disease Control and Prevention, China)*; Regina Heidinger-Felső (University of Pécs, Hungary)*; Margit Heier (Helmholtz Zentrum München, Germany)*; Mirjam Heinen (University College Dublin, Denmark)*; Tatjana Hejgaard (Danish Health Authority, Denmark)*; Marleen Elisabeth Hendriks (Joep Lange Institute, The Netherlands)*; Rafael dos Santos Henrique (Federal University of Pernambuco, Brazil)*; Ana Henriques (Institute of Public Health of the University of Porto, Portugal)*; Leticia Hernandez Cadena (National Institute of Public Health, Mexico)*; Sauli Herrala (Oulu University Hospital, Finland)*; Marianella Herrera-Cuenca (Universidad Central de Venezuela, Venezuela)*; Victor M Herrera (Universidad Autónoma de Bucaramanga, Colombia)*; Isabelle Herter-Aeberli (ETH Zurich, Switzerland)*; Karl-Heinz Herzig (University of Oulu, Finland; Oulu University Hospital, Finland)*; Ramin Heshmat (Chronic Diseases Research Center, Iran)*; Barbara Heude (Paris Cité University, France)*; Allan G Hill (University of Southampton, UK)*; Sai Yin Ho (University of Hong Kong, China)*; Suzanne C Ho (The Chinese University of Hong Kong, China)*; Michael Hobbs (University of Western Australia, Australia)*; Doroteia A Höfelmann (Universidade Federal do Paraná, Brazil)*; Michelle Holdsworth (French National Research Institute for Sustainable Development, France)*; Reza Homayounfar (Shahid Beheshti University of Medical Sciences, Iran)*; Clara Homs (Gasol Foundation, Spain; University Ramon Llull, Spain)*; Emiel Hoogendijk (VU University Medical Center, The Netherlands)*; Wilma M Hopman (Kingston Health Sciences Centre, Canada)*; Andrea RVR Horimoto (University of São Paulo, Brazil)*; Claudia M Hormiga (Fundación Oftalmológica de Santander, Colombia)*; Bernardo L Horta (Federal University of Pelotas, Brazil)*; Leila Houti (University Oran 1, Algeria)*; Christina Howitt (The University of the West Indies, Barbados)*; Thein Thein Htay (Independent Public

Health Specialist, Myanmar)*; Aung Soe Htet (University of Oslo, Norway)*; Maung Maung Than Htike (Ministry of Health and Sports, Myanmar)*; Yonghua Hu (Peking University, China)*; José María Huerta (CIBERESP, Spain)*; Ilpo Tapani Huhtaniemi (Imperial College London, UK)*; Laetitia Huiart (Santé publique France, France)*; Constanta Huidumac Petrescu (National Institute of Public Health, Romania)*; Abdullatif Husseini (Birzeit University, State of Palestine)*; Chinh Nguyen Huu (National Institute of Nutrition, Vietnam)*; Inge Huybrechts (International Agency for Research on Cancer, France)*; Nahla Hwalla (American University of Beirut, Lebanon)*; Jolanda Hyska (Institute of Public Health, Albania)*; Licia Iacoviello (IRCCS Neuromed, Italy; University of Insubria, Italy)*; Ellina M Iakupova (Ufa Eye Research Institute, Russia)*; Jesús Ibarluzea (CIBERESP, Spain)*; Mohsen M Ibrahim (Cairo University, Egypt)*; Norazizah Ibrahim Wong (Ministry of Health, Malaysia)*; Jannicke Igland (University of Bergen, Norway)*; Chinwuba Ijoma (University of Nigeria, Nigeria)*; Nayu Ikeda (National Institutes of Biomedical Innovation, Health and Nutrition, Japan)*; M Arfan Ikram (Erasmus Medical Center Rotterdam, The Netherlands)*; Carmen Iñiguez (Universitat de València, Spain)*; Violeta Iotova (Medical University - Varna, Bulgaria)*; Vilma E Irazola (Institute for Clinical Effectiveness and Health Policy, Argentina)*; Takafumi Ishida (The University of Tokyo, Japan)*; Godsent C Isiguzo (Alex Ekwueme Federal University Teaching Hospital, Nigeria)*; Muhammad Islam (The Hospital for Sick Children, Canada)*; Sheikh Mohammed Shariful Islam (Deakin University, Australia)*; Duygu Islek (Emory University, USA)*; Till Ittermann (University Medicine Greifswald, Germany)*; Ivaila Y Ivanova-Pandourska (Bulgarian Academy of Sciences, Bulgaria)*; Masanori Iwasaki (Hokkaido University, Japan)*; Tuija Jääskeläinen (Finnish Institute for Health and Welfare, Finland)*; Rod T Jackson (University of Auckland, New Zealand)*; Jeremy M Jacobs (Hadassah University Medical Center, Israel)*; Michel Jadoul (Université Catholique de Louvain, Belgium)*; Tazeen Jafar (Duke-NUS Medical School, Singapore)*; Bakary Jallow (Gambia National Nutrition Agency, The Gambia)*; Kenneth James (The University of the West Indies, Jamaica)*; Kazi M Jamil (Kuwait Institute for Scientific Research, Kuwait)*; Konrad Jamrozik (University of Adelaide, Australia; deceased)*; Nataša Jan (Slovenian Heart Foundation,

Slovenia)*; Anna Jansson (Public Health Agency of Sweden, Sweden)*; Imre Janszky (Norwegian University of Science and Technology, Norway)*; Edward Janus (University of Melbourne, Australia)*; Juel Jarani (Sports University of Tirana, Albania)*; Gerald Jarnig (University of Graz, Austria)*; Marjo-Riitta Jarvelin (Imperial College London, UK; University of Oulu, Finland)*; Grazyna Jasienska (Jagiellonian University Medical College, Poland)*; Ana Jelaković (University Hospital Centre Zagreb, Croatia)*; Bojan Jelaković (University of Zagreb, Croatia)*; Garry Jennings (Heart Foundation, Australia)*; Chao Qiang Jiang (Guangzhou 12th Hospital, China)*; Ramon O Jimenez (Universidad Eugenio Maria de Hostos, Dominican Republic)*; Karl-Heinz Jöckel (University of Duisburg-Essen, Germany)*; Michel Joffres (Simon Fraser University, Canada)*; Jari J Jokelainen (Oulu University Hospital, Finland)*; Jost B Jonas (Institute of Molecular and Clinical Ophthalmology Basel, Switzerland)*; Jitendra Jonnagaddala (University of New South Wales, Australia)*; Lars Jøran Kjerpeseth (Norwegian Institute of Public Health, Norway)*; Torben Jørgensen (Copenhagen University Hospital - Bispebjerg and Frederiksberg, Denmark)*; Pradeep Joshi (World Health Organization Country Office, India)*; Rohina Joshi (University of New South Wales, Australia)*; Josipa Josipović (University Hospital Centre Zagreb, Croatia)*; Farahnaz Joukar (Guilan University of Medical Sciences, Iran)*; Jacek J Jóźwiak (University of Opole, Poland)*; Debra S Judge (University of Western Australia, Australia)*; Anne Juolevi (Finnish Institute for Health and Welfare, Finland)*; Gregor Jurak (University of Ljubljana, Slovenia)*; Iulia Jurca Simina (Victor Babes University of Medicine and Pharmacy, Romania)*; Vesna Juresa (University of Zagreb, Croatia)*; Rudolf Kaaks (German Cancer Research Center, Germany)*; Felix O Kaducu (Gulu University, Uganda)*; Agnes L Kadvan (Capacity Development Network in Nutrition in Central and Eastern Europe, Serbia)*; Anthony Kafatos (University of Crete, Greece)*; Mónika Kaj (Hungarian School Sport Federation, Hungary)*; Eero O Kajantie (Finnish Institute for Health and Welfare, Finland)*; Natia Kakutia (National Center for Disease Control and Public Health, Georgia)*; Daniela Kállayová (Ministry of Health, Slovakia)*; Zhanna Kalmatayeva (Al-Farabi Kazakh National University, Kazakhstan)*; Ofra Kalter-Leibovici (The Gertner Institute for Epidemiology and Health Policy Research, Israel)*; Yves Kameli (French National Research

Institute for Sustainable Development, France)*; Kodanda R Kanala (Sri Venkateswara University, India)*; Srinivasan Kannan (Sree Chitra Tirunal Institute for Medical Sciences and Technology, India)*; Efthymios Kapantais (Hellenic Medical Association for Obesity, Greece)*; Eva Karaglani (Harokopio University, Greece)*; Argyro Karakosta (National and Kapodistrian University of Athens, Greece)*; Line L Kårhus (Copenhagen University Hospital - Bispebjerg and Frederiksberg, Denmark)*; Khem B Karki (Maharajgunj Medical Campus, Nepal)*; Omat Karlsson (Duke University, USA)*; Adoubi Kassi Anicet (University of Bouaké, Côte d'Ivoire)*; Philippe B Katchunga (Université Officielle de Bukavu, DR Congo)*; Marzieh Katibeh (Aarhus University, Denmark)*; Joanne Katz (Johns Hopkins Bloomberg School of Public Health, USA)*; Peter T Katzmarzyk (Pennington Biomedical Research Center, USA)*; Jussi Kauhanen (University of Eastern Finland, Finland)*; Prabhdeep Kaur (National Institute of Epidemiology, India)*; Maryam Kavousi (Erasmus Medical Center Rotterdam, The Netherlands)*; Gyulli M Kazakbaeva (Ufa Eye Research Institute, Russia)*; François F Kaze (University of Yaoundé 1, Cameroon)*; Benson M Kazembe (UNICEF, Malawi)*; Calvin Ke (University of Toronto, Canada)*; Ulrich Keil (University of Münster, Germany)*; Lital Keinan Boker (Israel Center for Disease Control, Israel)*; Sirkka Keinänen-Kiukaanniemi (Oulu University Hospital, Finland)*; Roya Kelishadi (Child Growth and Development Research Center, Iran)*; Cecily Kelleher (University College Dublin, Ireland)*; Han CG Kemper (VU University Medical Center, The Netherlands)*; Andre P Kengne (South African Medical Research Council, South Africa)*; Maryam Keramati (Mashhad University of Medical Sciences, Iran)*; Alina Kerimkulova (Kyrgyz State Medical Academy, Kyrgyzstan)*; Mathilde Kersting (Research Institute of Child Nutrition, Germany)*; Timothy Key (University of Oxford, UK)*; Yousef Saleh Khader (Jordan University of Science and Technology, Jordan)*; Arsalan Khaledifar (Shahrekord University of Medical Sciences, Iran)*; Davood Khalili (Shahid Beheshti University of Medical Sciences, Iran)*; Young-Ho Khang (Seoul National University College of Medicine, Republic of Korea)*; Bahareh Kheiri (Shahid Beheshti University of Medical Sciences, Iran)*; Motahareh Kheradmand (Mazandaran University of Medical Sciences, Iran)*; Alireza Khosravi (Hypertension Research Center, Iran)*; Ilse MSL Khouw

(FrieslandCampina, The Netherlands)*; Ursula Kiechl-Kohlendorfer (Medical University of Innsbruck, Austria)*; Sophia J Kiechl (VASCage – Research Centre on Vascular Ageing and Stroke, Austria; Hochzirl Hospital, Austria)*; Stefan Kiechl (Medical University of Innsbruck, Austria; VASCage – Research Centre on Vascular Ageing and Stroke, Austria)*; Japhet Killewo (Muhimbili University of Health and Allied Sciences, Tanzania)*; Hyeon Chang Kim (Yonsei University College of Medicine, Republic of Korea)*; Jeongseon Kim (National Cancer Center, Republic of Korea)*; Jenny M Kindblom (University of Gothenburg, Sweden; Sahlgrenska University Hospital, Sweden)*; Andrew Kingston (Newcastle University, UK)*; Heidi Klakk (University College South Denmark, Denmark)*; Suntara Klanarong (Thaksin University, Thailand; deceased)*; Jana Klanova (Masaryk University, Czechia)*; Magdalena Klimek (Jagiellonian University Medical College, Poland)*; Jeannette Klimont (Statistics Austria, Austria)*; Jurate Klumbiene (Lithuanian University of Health Sciences, Lithuania)*; Michael Knoflach (Medical University of Innsbruck, Austria)*; Susanne Kobel (Ulm University Hospital, Germany)*; Bhawesh Koirala (B P Koirala Institute of Health Sciences, Nepal)*; Elin Kolle (Norwegian School of Sport Sciences, Norway)*; Sanda M Kolo (International committee of Red Cross, Nigeria)*; Patrick Kolsteren (Ghent University, Belgium)*; Jürgen König (University of Vienna, Austria)*; Raija Korpelainen (University of Oulu, Finland)*; Paul Korrovits (Tartu University Clinics, Estonia)*; Magdalena Korzycka (Institute of Mother and Child, Poland)*; Jelena Kos (University Hospital Centre Zagreb, Croatia)*; Seppo Koskinen (Finnish Institute for Health and Welfare, Finland)*; Katsuyasu Kouda (Kansai Medical University, Japan)*; Malik Koussoh Simone (National Institute of Public Health, Côte d'Ivoire)*; Éva Kovács (Hildburghausen District Department of State Public Health Service, Germany)*; Viktoria Anna Kovacs (Hungarian School Sport Federation, Hungary)*; Irina Kovalskys (Pontificia Universidad Católica Argentina, Argentina)*; Sudhir Kowlessur (Ministry of Health and Wellness, Mauritius)*; Slawomir Koziel (PASs Hirszfeld Institute of Immunology and Experimental Therapy, Poland)*; Jana Kratenova (National Institute of Public Health, Czech Republic)*; Wolfgang Kratzer (University Hospital Ulm, Germany)*; Vilma Kriaucioniene (Lithuanian University of Health Sciences, Lithuania)*; Susi Kriemler (University of Zurich,

Switzerland)*; Peter Lund Kristensen (University of Southern Denmark, Denmark)*; Helena Krizan (Croatian Institute of Public Health, Croatia)*; Maria F Kroker-Lobos (Institute of Nutrition of Central America and Panama, Guatemala)*; Steinar Krokstad (Norwegian University of Science and Technology, Norway)*; Daan Kromhout (University of Groningen, The Netherlands)*; Herculina S Kruger (North-West University, South Africa; South African Medical Research Council, South Africa)*; Ruan Kruger (North-West University, South Africa)*; Łukasz Kryst (University of Physical Education, Poland)*; Ruzena Kubinova (National Institute of Public Health, Czech Republic)*; Renata Kuciene (Lithuanian University of Health Sciences, Lithuania)*; Urho M Kujala (University of Jyväskylä, Finland)*; Enisa Kujundzic (Institute of Public Health, Montenegro)*; Zbigniew Kulaga (Children's Memorial Health Institute, Poland)*; Mukhtar Kulimbet (Al-Farabi Kazakh National University, Kazakhstan; Asfendiyarov Kazakh National Medical University, Kazakhstan)*; Vaitheeswaran Kulothungan (ICMR - National Centre for Disease Informatics and Research, India)*; R Krishna Kumar (Amrita Institute of Medical Sciences, India)*; Meena Kumari (University of Essex, UK)*; Marie Kunešová (Institute of Endocrinology, Czech Republic)*; Pawel Kurjata (National Institute of Cardiology, Poland)*; Yadlapalli S Kusuma (All India Institute of Medical Sciences, India)*; Vladimir Kutsenko (National Medical Research Centre for Therapy and Preventive Medicine, Russia)*; Kari Kuulasmaa (Finnish Institute for Health and Welfare, Finland)*; Catherine Kyobutungi (African Population and Health Research Center, Kenya)*; Quang Ngoc La (Hanoi University of Public Health, Vietnam)*; Fatima Zahra Laamiri (Hassan First University of Settat, Morocco)*; Tiina Laatikainen (University of Eastern Finland, Finland; Finnish Institute for Health and Welfare, Finland)*; Demetre Labadarios (University of Limpopo, South Africa; Stellenbosch University, South Africa)*; Carl Lachat (Ghent University, Belgium)*; Karl J Lackner (Johannes Gutenberg University, Germany)*; Daphne Lai (Universiti Brunei Darussalam, Brunei)*; Youcef Laid (Ministry of Health, Algeria)*; Lachmie Lall (Ministry of Health, Guyana)*; Tai Hing Lam (University of Hong Kong, China)*; Maritza Landaeta Jimenez (Universidad Central de Venezuela, Venezuela)*; Edwige Landais (French National Research Institute for Sustainable Development, France)*; Tiina Lankila (Oulu Deaconess Institute

Foundation, Finland)*; Vera Lanska (Institute for Clinical and Experimental Medicine, Czech Republic)*; Georg Lappas (Sahlgrenska Academy, Sweden)*; Bagher Larijani (Endocrinology and Metabolism Research Center, Iran)*; Simo Pone Larissa (Clinical Research Education, Networking & Consultancy, Cameroon)*; Mina P Lateva (Medical University - Varna, Bulgaria)*; Tint Swe Latt (University of Public Health, Myanmar)*; Martino Laurenzi (Centro Studi Epidemiologici di Gubbio, Italy)*; Laura Lauria (Istituto Superiore di Sanità, Italy)*; Avula Laxmaiah (ICMR - National Institute of Nutrition, India)*; Maria Lazo-Porras (Universidad Peruana Cayetano Heredia, Peru)*; Gwenaëlle Le Coroller (Luxembourg Institute of Health, Luxembourg)*; Khanh Le Nguyen Bao (National Institute of Nutrition, Vietnam)*; Agnès Le Port (French National Research Institute for Sustainable Development, France)*; Tuyen D Le (National Institute of Nutrition, Vietnam)*; Jeannette Lee (National University of Singapore, Singapore; National University Health System, Singapore)*; Jeonghee Lee (National Cancer Center, Republic of Korea)*; Paul H Lee (University of Southampton, UK)*; Terho Lehtimäki (Tampere University Hospital, Finland; Tampere University, Finland)*; Daniel Lemogoum (University of Douala, Cameroon)*; Elvynna Leong (Universiti Brunei Darussalam, Brunei)*; Branimir Leskošek (University of Ljubljana, Slovenia)*; Justyna Leszczak (University of Rzeszów, Poland)*; Katja B Leth-Møller (Copenhagen University Hospital - Bispebjerg and Frederiksberg, Denmark)*; Gabriel M Leung (University of Hong Kong, China)*; Naomi S Levitt (University of Cape Town, South Africa)*; Yanping Li (Harvard TH Chan School of Public Health, USA)*; Merike Liivak (National Institute for Health Development, Estonia)*; Christa L Lilly (West Virginia University, USA)*; Charlie Lim (National University of Singapore, Singapore; National University Health System, Singapore)*; Wei-Yen Lim (National University of Singapore, Singapore; National University Health System, Singapore)*; M Fernanda Lima-Costa (Oswaldo Cruz Foundation Rene Rachou Research Institute, Brazil)*; Hsien-Ho Lin (National Taiwan University, Taiwan)*; Xu Lin (Shanghai Institute of Nutrition and Health of Chinese Academy of Sciences, China)*; Lars Lind (Uppsala University, Sweden)*; Vijaya Lingam (Medical Research Foundation, India)*; Birgit Linkohr (Helmholtz Zentrum München, Germany)*; Allan Linneberg (Copenhagen University Hospital - Bispebjerg and Frederiksberg,

Denmark)*; Lauren Lissner (University of Gothenburg, Sweden)*; Mieczyslaw Litwin (Children's Memorial Health Institute, Poland)*; Jing Liu (Capital Medical University Beijing An Zhen Hospital, China)*; Lijuan Liu (Capital Medical University Beijing Tongren Hospital, China)*; Liping Liu (Beijing Center for Disease Prevention and Control, China)*; Xiaotian Liu (Zhengzhou University, China)*; Wei-Cheng Lo (Taipei Medical University, Taiwan)*; Helle-Mai Loit (National Institute for Health Development, Estonia)*; Khuong Quynh Long (Hanoi University of Public Health, Vietnam)*; Guadalupe Longo Abril (Servicio Andaluz de Salud, Spain)*; Luis Lopes (University of Porto, Portugal)*; Marcus SS Lopes (Federal University of Santa Catarina, Brazil)*; Oscar Lopes (Sports Medical Center of Minho, Portugal)*; Esther Lopez-Garcia (Universidad Autónoma de Madrid CIBERESP, Spain)*; Tania Lopez (Universidad San Martín de Porres, Peru)*; Paulo A Lotufo (University of São Paulo, Brazil)*; José Eugenio Lozano (Consejería de Sanidad Junta de Castilla y León, Spain)*; Janice L Lukrafka (Universidade Federal de Ciências da Saúde de Porto Alegre, Brazil)*; Dalia Luksiene (Lithuanian University of Health Sciences, Lithuania)*; Annamari Lundqvist (Finnish Institute for Health and Welfare, Finland)*; Nuno Lunet (University of Porto, Portugal)*; Charles Lunogelo (Ilembula Lutheran Hospital, Tanzania)*; Michala Lustigová (Charles University, Czech Republic; National Institute of Public Health, Czech Republic)*; Edyta Łuszczki (University of Rzeszów, Poland)*; Jean-René M'Buyamba-Kabangu (University of Kinshasa Hospital, DR Congo)*; Guansheng Ma (Peking University, China)*; Jun Ma (Peking University, China)*; Xu Ma (National Research Institute for Health and Family Planning, China)*; George LL Machado-Coelho (Universidade Federal de Ouro Preto, Brazil)*; Aristides M Machado-Rodrigues (University of Coimbra, Portugal)*; Enguerran Macia (UMR 7268 ADES, France)*; Luisa M Macieira (Coimbra University Hospital Center, Portugal)*; Ahmed A Madar (University of Oslo, Norway)*; Sherilynn Madraisau (Ministry of Health and Human Services, Palau)*; Anja L Madsen (Copenhagen University Hospital - Bispebjerg and Frederiksberg, Denmark)*; Gladys E Maestre (University of Texas Rio Grande Valley, USA)*; Stefania Maggi (Institute of Neuroscience of the National Research Council, Italy)*; Dianna J Magliano (Baker Heart and Diabetes Institute, Australia)*; Sara Magnacca (Mediterranea

Cardiocentro, Italy)*; Emmanuella Magriplis (Agricultural University of Athens, Greece)*; Gowri Mahasampath (Christian Medical College Vellore, India)*; Bernard Maire (French National Research Institute for Sustainable Development, France)*; Marjeta Majer (University of Zagreb, Croatia)*; Marcia Makdisse (Academia VBHC, Brazil)*; Päivi Mäki (Finnish Institute for Health and Welfare, Finland)*; Mohammad-Reza Malekpour (Non-Communicable Diseases Research Center, Iran)*; Fatemeh Malekzadeh (Tehran University of Medical Sciences, Iran)*; Reza Malekzadeh (Shiraz University of Medical Sciences, Iran; Tehran University of Medical Sciences, Iran)*; Rahul Malhotra (Duke-NUS Medical School, Singapore)*; Kodavanti Mallikharjuna Rao (ICMR - National Institute of Nutrition, India)*; Deborah C Malta (Universidade Federal de Minas Gerais, Brazil)*; Sofia K Malyutina (SB RAS Federal Research Center Institute of Cytology and Genetics, Russia)*; Lynell V Maniego (Food and Nutrition Research Institute, Philippines)*; Yannis Manios (Harokopio University, Greece)*; Jim I Mann (University of Otago, New Zealand)*; Masimango Imani Mannix (Université Catholique de Bukavu, DR Congo)*; Fariborz Mansour-Ghanaei (Guilan University of Medical Sciences, Iran)*; Taru Manyanga (University of Northern British Columbia, Canada)*; Enzo Manzato (University of Padua, Italy)*; Mala Ali Mapatano (University of Kinshasa, DR Congo)*; Anie Marcil (Statistics Canada, Canada)*; Paula Margozzini (Pontificia Universidad Católica de Chile, Chile)*; Rosu Maria-Magdalena (Clinical County Emergency Hospital Craiova, Romania)*; Joany Mariño (University Medicine Greifswald, Germany)*; Anastasia Markaki (Hellenic Mediterranean University, Greece)*; Oonagh Markey (Loughborough University, UK)*; Eliza Markidou Ioannidou (Ministry of Health, Cyprus)*; Pedro Marques-Vidal (Lausanne University Hospital, Switzerland; University of Lausanne, Switzerland)*; Larissa Pruner Marques (Fundação Oswaldo Cruz, Brazil)*; Jaume Marrugat (CIBERCV, Spain; Hospital del Mar Research Institute, Spain)*; Yves Martin-Prevel (French National Research Institute for Sustainable Development, France)*; Rosemarie Martin (Mary Immaculate College, Ireland)*; Reynaldo Martorell (Emory University, USA)*; Eva Martos (Hungarian Society of Sports Medicine, Hungary)*; Fatai A Maruf (Naamdi Azikiwe University, Nigeria)*; Katharina Maruszczak (Paracelsus Medical University, Austria)*; Stefano

Marventano (University of Catania, Italy)*; Giovanna Masala (Institute for Cancer Reseach, Prevention and Clinical Network, Italy)*; Luis P Mascarenhas (Universidade Estadual do Centro-Oeste, Brazil)*; Masoud Masinaei (Non-Communicable Diseases Research Center, Iran)*; Shariq R Masoodi (Sher-i-Kashmir Institute of Medical Sciences, India)*; Ellisiv B Mathiesen (UiT The Arctic University of Norway, Norway)*; Prashant Mathur (ICMR - National Centre for Disease Informatics and Research, India)*; Alicia Matijasevich (University of São Paulo, Brazil)*; Piotr Matłosz (University of Rzeszów, Poland)*; Tandi E Matsha (Sefako Makgatho Health Sciences University, South Africa)*; Victor Matsudo (Centro de Estudos do Laboratório de Aptidão Física de São Caetano do Sul, Brazil)*; Giletta Matteo (Ghent University, Belgium)*; Pallab K Maulik (George Institute for Global Health, India)*; Christina Mavrogianni (Harokopio University, Greece)*; Artur Mazur (University of Rzeszów, Poland)*; Jean Claude N Mbanya (University of Yaoundé 1, Cameroon)*; Shelly R McFarlane (The University of the West Indies, Jamaica)*; Stephen T McGarvey (Brown University, USA)*; Martin McKee (London School of Hygiene & Tropical Medicine, UK)*; Rachael M McLean (University of Otago, New Zealand)*; Scott B McLean (Statistics Canada, Canada)*; Margaret L McNairy (Weill Cornell Medicine, USA)*; Breige A McNulty (University College Dublin, Ireland)*; Sounnia Mediene Benchekor (University Oran 1, Algeria)*; Jurate Medzioniene (Lithuanian University of Health Sciences, Lithuania)*; Kirsten Mehlig (University of Gothenburg, Sweden)*; Amir Houshang Mehrparvar (Shahid Sadoughi University of Medical Sciences, Iran)*; Aline Meirhaeghe (Institut National de la Santé et de la Recherche Médicale, France)*; Jørgen Meisfjord (Norwegian Institute of Public Health, Norway)*; Christa Meisinger (Helmholtz Zentrum München, Germany)*; Jesus D Melgarejo (University of Texas Rio Grande Valley, USA)*; Marina Melkumova (Arabkir Medical Centre Institute of Child and Adolescent Health, Armenia)*; Júlio Mello (Pontificia Universidad Católica de Valparaíso, Chile)*; Fabián Méndez (Universidad del Valle, Colombia)*; Carlos O Mendivil (Universidad de los Andes, Colombia)*; Ana Maria B Menezes (Federal University of Pelotas, Brazil)*; Geetha R Menon (ICMR - National Institute of Medical Statistics, India)*; Gert BM Mensink (Robert Koch Institute, Germany)*; Maria Teresa Menzano (Ministero della Salute DG

Prevenzione Sanitaria, Italy)*; Indrapal I Meshram (ICMR - National Institute of Nutrition, India)*; Diane T Meto (University of Abidjan, Côte d'Ivoire)*; Haakon E Meyer (University of Oslo, Norway)*; Jie Mi (Capital Institute of Pediatrics, China)*; Kim F Michaelsen (University of Copenhagen, Denmark)*; Nathalie Michels (Ghent University, Belgium)*; Kairit Mikkel (University of Tartu, Estonia)*; Karolina Miłkowska (Jagiellonian University Medical College, Poland)*; Jody C Miller (University of Otago, New Zealand)*; Olga Milushkina (Pirogov Russian National Research Medical University, Russia)*; Cláudia S Minderico (Universidade de Lisboa, Portugal)*; GK Mini (Saveetha Institute of Medical and Technical Sciences, India)*; Juan Francisco Miquel (Pontificia Universidad Católica de Chile, Chile)*; J Jaime Miranda (University of Sydney, Australia)*; Mohammad Reza Mirjalili (Shahid Sadoughi University of Medical Sciences, Iran)*; Daphne Mirkopoulou (Democritus University, Greece)*; Erkin Mirrakhimov (Kyrgyz State Medical Academy, Kyrgyzstan)*; Marjeta Mišigoj-Duraković (University of Zagreb, Croatia)*; Antonio Mistretta (University of Catania, Italy)*; Veronica Mocanu (Grigore T Popa University of Medicine and Pharmacy, Romania)*; Pietro A Modesti (Università degli Studi di Firenze, Italy)*; Sahar Saeedi Moghaddam (Non-Communicable Diseases Research Center, Iran)*; Shukri F Mohamed (African Population and Health Research Center, Kenya)*; Kazem Mohammad (Tehran University of Medical Sciences, Iran)*; Mohammad Reza Mohammadi (Psychiatry and Psychology Research Center, Iran)*; Zahra Mohammadi (Tehran University of Medical Sciences, Iran)*; Noushin Mohammadifard (Isfahan Cardiovascular Research Center, Iran)*; Reza Mohammadpourhodki (Mashhad University of Medical Sciences, Iran)*; Viswanathan Mohan (Madras Diabetes Research Foundation, India)*; Salim Mohanna (Universidad Peruana Cayetano Heredia, Peru)*; Muhammad Fadhli Mohd Yusoff (Ministry of Health, Malaysia)*; Iraj Mohebbi (Urmia University of Medical Sciences, Iran)*; Marie Moitry (University of Strasbourg, France; Strasbourg University Hospital, France)*; Line T Møllehave (Copenhagen University Hospital - Bispebjerg and Frederiksberg, Denmark)*; Niels C Møller (University of Southern Denmark, Denmark)*; Dénes Molnár (University Medical School of Pécs, Hungary)*; Amirabbas Momenan (Shahid Beheshti University of Medical Sciences, Iran)*; Charles K Mondo (Mulago Hospital, Uganda)*;

Michele Monroy-Valle (University of San Carlos of Guatemala, Guatemala)*; Roger A Montenegro Mendoza (Gorgas Memorial Institute for Studies of Health, Panama)*; Eric Monterrubio-Flores (National Institute of Public Health, Mexico)*; Kotsedi Daniel K Monyeki (University of Limpopo, South Africa)*; Jin Soo Moon (Seoul National University, Republic of Korea)*; Mahmood Moosazadeh (Mazandaran University of Medical Sciences, Iran)*; Hermine T Mopa (University of Yaoundé 1, Cameroon)*; Farhad Moradpour (Kurdistan University of Medical Sciences, Iran)*; Leila B Moreira (Universidade Federal do Rio Grande do Sul, Brazil)*; Alain Morejon (University of Medical Sciences of Cienfuegos, Cuba)*; Luis A Moreno (University of Zaragoza, Spain; CIBEROBN, Spain)*; Francis Morey (Ministry of Health and Wellness, Belize)*; Karen Morgan (RCSI University of Medicine and Health Sciences, Ireland)*; Suzanne N Morin (McGill University, Canada)*; Erik Lykke Mortensen (University of Copenhagen, Denmark)*; George Moschonis (La Trobe University, Australia)*; Alireza Moslem (Sabzevar University Of Medical Sciences, Iran)*; Mildrey Mosquera (Universidad del Valle, Colombia)*; Malgorzata Mossakowska (International Institute of Molecular and Cell Biology, Poland)*; Aya Mostafa (Ain Shams University, Egypt)*; Seyed-Ali Mostafavi (Tehran University of Medical Sciences, Iran)*; Anabela Mota-Pinto (University of Coimbra, Portugal)*; Jorge Mota (University of Porto, Portugal)*; Mohammad Esmaeel Motlagh (Ahvaz Jundishapur University of Medical Sciences, Iran)*; Jorge Motta (Instituto Conmemorativo Gorgas de Estudios de la Salud, Panama)*; Marcos André Moura-dos-Santos (University of Pernambuco, Brazil)*; Yeva Movsesyan (Arabkir Medical Centre Institute of Child and Adolescent Health, Armenia)*; Malay K Mridha (BRAC James P Grant School of Public Health, Bangladesh)*; Kelias P Msyamboza (World Health Organization Country Office, Malawi)*; Thet Thet Mu (Department of Public Health, Myanmar)*; Magdalena Muc (University of Coimbra, Portugal)*; Florian Muca (Albanian Sports Science Association, Albania)*; Boban Mugoša (Institute of Public Health, Montenegro)*; Maria L Muiesan (University of Brescia, Italy)*; Martina Müller-Nurasyid (Johannes Gutenberg University, Germany)*; Thomas Münzel (Johannes Gutenberg University, Germany)*; Jaakko Mursu (University of Eastern Finland, Finland)*; Elaine M Murtagh (University of Limerick, Ireland)*; Kamarul Imran Musa (Universiti

Sains Malaysia, Malaysia)*; Sanja Musić Milanović (Croatian Institute of Public Health, Croatia; University of Zagreb, Croatia)*; Vera Musil (University of Zagreb, Croatia)*; Geofrey Musinguzi (Makerere University School of Public Health, Uganda)*; Muel Telo Muyer (University de Kinshasa, DR Congo)*; Iraj Nabipour (Bushehr University of Medical Sciences, Iran)*; Gabriele Nagel (Ulm University, Germany)*; Farid Najafi (Kermanshah University of Medical Sciences, Iran)*; Harunobu Nakamura (Kansai Medical University, Japan)*; Hanna Nalecz (Institute of Mother and Child, Poland)*; Jana Námešná (Regional Authority of Public Health, Slovakia)*; Ei Ei K Nang (National University of Singapore, Singapore; National University Health System, Singapore)*; Vinay B Nangia (Suraj Eye Institute, India)*; Martin Nankap (UNICEF, Cameroon)*; Sameer Narake (Healis-Sekhsaria Institute for Public Health, India)*; KM Venkat Narayan (Emory University, USA)*; Paola Nardone (Istituto Superiore di Sanità, Italy)*; Take Naseri (Ministry of Health, Samoa)*; Michels Nathalie (Ghent University, Belgium)*; William A Neal (West Virginia University, USA)*; Nareemarn Neelapaichit (Mahidol University, Thailand)*; Azim Nejatizadeh (Hormozgan University of Medical Sciences, Iran)*; Chandini Nekkantti (University of New South Wales, Australia)*; Keiu Nelis (National Institute for Health Development, Estonia)*; Ilona Nenko (Jagiellonian University Medical College, Poland)*; Martin Neovius (Karolinska Institutet, Sweden)*; Flavio Nervi (Pontificia Universidad Católica de Chile, Chile)*; Tze Pin Ng (National University of Singapore, Singapore)*; Chung T Nguyen (National Institute of Hygiene and Epidemiology, Vietnam)*; Nguyen D Nguyen (University of Medicine and Pharmacy at Ho Chi Minh City, Vietnam)*; Quang Ngoc Nguyen (Hanoi Medical University, Vietnam)*; Michael Y Ni (University of Hong Kong, China)*; Rodica Nicolescu (National Institute of Public Health, Romania)*; Peng Nie (Xi'an Jiaotong University, China)*; Ramfis E Nieto-Martínez (Precision Care Clinic Corp, USA)*; Yury P Nikitin (SB RAS Federal Research Center Institute of Cytology and Genetics, Russia)*; Guang Ning (Shanghai Jiao-Tong University School of Medicine, China)*; Toshiharu Ninomiya (Kyushu University, Japan)*; Nobuo Nishi (National Institutes of Biomedical Innovation, Health and Nutrition, Japan)*; Sania Nishtar (Heartfile, Pakistan)*; Marianna Noale (Institute of Neuroscience of the National Research Council, Italy)*; Oscar A Noboa (Universidad de la República, Uruguay)*;

Helena Nogueira (University of Coimbra, Portugal)*; Maria Nordendahl (Umeå University, Sweden)*; Børge G Nordestgaard (Copenhagen University Hospital, Denmark; University of Copenhagen, Denmark)*; Kevin I Norton (University of South Australia, Australia)*; Davide Noto (University of Palermo, Italy)*; Natalia Nowak-Szczepanska (PASs Hirszfeld Institute of Immunology and Experimental Therapy, Poland)*; Mohannad Al Nsour (Eastern Mediterranean Public Health Network, Jordan)*; Irfan Nuhoğlu (Karadeniz Technical University, Türkiye)*; Baltazar Nunes (National Institute of Health Doutor Ricardo Jorge, Portugal; NOVA University Lisbon, Portugal)*; Eha Nurk (National Institute for Health Development, Estonia)*; Fred Nuwaha (Makerere University School of Public Health, Uganda)*; Moffat Nyirenda (London School of Hygiene & Tropical Medicine, UK)*; Terence W O'Neill (University of Manchester, UK)*; Dermot O'Reilly (Queen's University of Belfast, UK)*; Galina Obreja (State University of Medicine and Pharmacy, Moldova)*; Caleb Ochimana (Harvard TH Chan School of Public Health, USA)*; Angélica M Ochoa-Avilés (Universidad de Cuenca, Ecuador)*; Eiji Oda (Tachikawa General Hospital, Japan)*; Augustine N Odili (University of Abuja College of Health Sciences, Nigeria)*; Kyungwon Oh (Korea Disease Control and Prevention Agency, Republic of Korea)*; Kumiko Ohara (Kansai Medical University, Japan)*; Claes Ohlsson (University of Gothenburg, Sweden; Sahlgrenska University Hospital, Sweden)*; Ryutaro Ohtsuka (Japan Wildlife Research Center, Japan; deceased)*; Örn Olafsson (Icelandic Heart Association, Iceland)*; Brian Oldenburg (Baker Heart & Diabetes Institute, Australia)*; Maria Teresa A Olinto (Universidade Federal do Rio Grande do Sul, Brazil)*; Isabel O Oliveira (Federal University of Pelotas, Brazil)*; Mohd Azahadi Omar (Ministry of Health, Malaysia)*; Saeed M Omar (Gadarif University, Sudan)*; Altan Onat (Istanbul University, Türkiye; deceased)*; Sok King Ong (Ministry of Health, Brunei)*; N Charlotte Onland-Moret (Utrecht University, The Netherlands)*; Lariane M Ono (Universidade Federal do Paraná, Brazil)*; Obinna Onodugo (University of Nigeria, Nigeria)*; Pedro Ordunez (Pan American Health Organization, USA)*; Rui Ornelas (University of Madeira, Portugal)*; Ana P Ortiz (University of Puerto Rico, Puerto Rico)*; Pedro J Ortiz (Universidad Peruana Cayetano Heredia, Peru)*; Merete Osler (Copenhagen University

Hospital - Bispebjerg and Frederiksberg, Denmark)*; Clive Osmond (University of Southampton, UK)*; Sergej M Ostojic (University of Novi Sad, Serbia)*; Afshin Ostovar (Osteoporosis Research Center, Iran)*; Johanna A Otero (Universidad de Santander, Colombia)*; Charlotte B Ottendahl (University of Southern Denmark, Denmark)*; Akaninyene Otu (University of Calabar, Nigeria)*; Kim Overvad (Aarhus University, Denmark)*; Ellis Owusu-Dabo (Kwame Nkrumah University of Science and Technology, Ghana)*; Adetoyeje Y Oyeyemi (University of Maiduguri, Nigeria)*; Adewale L Oyeyemi (Arizona State University, USA)*; Fred Michel Paccaud (Unisanté, Switzerland)*; Cristina P Padez (University of Coimbra, Portugal)*; Ioannis Pagkalos (International Hellenic University, Greece)*; Elena Pahomova (University of Latvia, Latvia)*; Karina Mary de Paiva (Federal University of Santa Catarina, Brazil)*; Andrzej Pająk (Jagiellonian University Medical College, Poland)*; Natalja Pajula (National Institute for Health Development, Estonia)*; Alberto Palloni (University of Wisconsin-Madison, USA)*; Luigi Palmieri (Istituto Superiore di Sanità, Italy)*; Wen-Harn Pan (Academia Sinica, Taiwan)*; Songhomitra Panda-Jonas (University Clinic Heidelberg, Germany)*; Arvind Pandey (ICMR - National Institute of Medical Statistics, India)*; Zengchang Pang (Qingdao Centers for Disease Control and Prevention, China)*; Francesco Panza (University of Bari Aldo Moro, Italy)*; Mariela Paoli (University of the Andes, Venezuela)*; Sousana K Papadopoulou (International Hellenic University, Greece)*; Dimitrios Papandreou (University of Sharjah, United Arab Emirates)*; Rossina G Pareja (Instituto de Investigación Nutricional, Peru)*; Soon-Woo Park (Catholic University of Daegu, Republic of Korea)*; Suyeon Park (Korea Disease Control and Prevention Agency, Republic of Korea)*; Winsome R Parnell (University of Otago, New Zealand)*; Mahboubeh Parsaeian (Tehran University of Medical Sciences, Iran)*; Ionela M Pascanu (University of Medicine, Pharmacy, Science and Technology of Târgu Mures, Romania)*; Patrick Pasquet (UMR CNRS-MNHN 7206, France)*; Nikhil D Patel (Jivandeep Hospital, India)*; Marcos Pattussi (Universidade do Vale do Rio dos Sinos, Brazil)*; Halyna Pavlyshyn (I. Horbachevsky Ternopil National Medical University, Ukraine)*; Raimund Pechlaner (Medical University of Innsbruck, Austria)*; Ivan Pećin (University Hospital Centre Zagreb, Croatia)*; Mangesh S Pednekar (Healis-Sekhsaria

Institute for Public Health, India)*; João M Pedro (Centro de Investigação em Saúde de Angola, Angola)*; Nasheeta Peer (South African Medical Research Council, South Africa)*; Sergio Viana Peixoto (Oswaldo Cruz Foundation Rene Rachou Research Institute, Brazil)*; Markku Peltonen (Finnish Institute for Health and Welfare, Finland)*; Alexandre C Pereira (University of São Paulo, Brazil)*; Marco A Peres (National Dental Care Centre Singapore, Singapore)*; Agustín Perez-Londoño (Universidad de los Andes, Colombia)*; Cynthia M Pérez (University of Puerto Rico, Puerto Rico)*; Valentina Peterkova (National Medical Research Center for Endocrinology, Russia)*; Annette Peters (Helmholtz Zentrum München, Germany)*; Janina Petkeviciene (Lithuanian University of Health Sciences, Lithuania)*; Ausra Petrauskiene (Lithuanian University of Health Sciences, Lithuania)*; Olga Petrovna Kovtun (Yekaterinburg State Medical Academy, Russia)*; Emanuela Pettenuzzo (University Hospital of Varese, Italy)*; Niloofar Peykari (Ministry of Health and Medical Education, Iran)*; Norbert Pfeiffer (Johannes Gutenberg University, Germany)*; Modou Cheyassin Phall (Gambia National Nutrition Agency, The Gambia)*; Son Thai Pham (Vietnam National Heart Institute, Vietnam)*; Felix P Phiri (Ministry of Health Malawi, Malawi)*; Rafael N Pichardo (Clínica de Medicina Avanzada Dr. Abel González, Dominican Republic)*; Daniela Pierannunzio (Istituto Superiore di Sanità, Italy)*; Preux Pierre-Marie (Université de Limoges, France)*; Iris Pigeot (Leibniz Institute for Prevention Research and Epidemiology - BIPS, Germany)*; Hynek Pikhart (University College London, UK)*; Aida Pilav (University of Sarajevo, Bosnia and Herzegovina)*; Pavel Piler (Masaryk University, Czechia)*; Lorenza Pilotto (Cardiovascular Prevention Centre Udine, Italy)*; Francesco Pistelli (University of Pisa, Italy)*; Freda Pitakaka (Ministry of Health and Medical Services, Solomon Islands)*; Aleksandra Piwonska (National Institute of Cardiology, Poland)*; Andreia N Pizarro (University of Porto, Portugal)*; Pedro Plans-Rubió (Public Health Agency of Catalonia, Spain)*; Alina G Platonova (O. M. Marzeyev Institute for Public Health of the National Academy of the Medical Sciences of Ukraine, Ukraine)*; Bee Koon Poh (Universiti Kebangsaan Malaysia, Malaysia)*; Hermann Pohlabeln (Leibniz Institute for Prevention Research and Epidemiology - BIPS, Germany)*; Nadija S Polka (O. M. Marzeyev Institute for Public Health of the National Academy of the Medical

Sciences of Ukraine, Ukraine)*; Raluca M Pop (University of Medicine, Pharmacy, Science and Technology of Târgu Mures, Romania)*; Barry M Popkin (University of North Carolina at Chapel Hill, USA)*; Stevo R Popovic (University of Montenegro, Montenegro)*; Miquel Porta (Institut Hospital del Mar d'Investigacions Mèdiques, Spain)*; Georg Posch (Agency for Preventive and Social Medicine, Austria)*; Anil Poudyal (Nepal Health Research Council, Nepal)*; Dimitrios Poulimeneas (International Hellenic University, Greece)*; Hamed Pouraram (Tehran University of Medical Sciences, Iran)*; Farhad Pourfarzi (Ardabil University of Medical Sciences, Iran)*; Akram Pourshams (Tehran University of Medical Sciences, Iran)*; Hossein Poustchi (Tehran University of Medical Sciences, Iran)*; Rajendra Pradeepa (Madras Diabetes Research Foundation, India)*; Alison J Price (London School of Hygiene & Tropical Medicine, UK)*; Jacqueline F Price (University of Edinburgh, UK)*; Antonio Prista (Universidade Pedagógica, Mozambique)*; Rui Providencia (University College London, UK)*; Jardena J Puder (Lausanne University Hospital, Switzerland)*; Iveta Pudule (Centre for Disease Prevention and Control, Latvia)*; Solie Puhakka (Oulu Deaconess Institute Foundation, Finland)*; Maria Puiu (Victor Babes University of Medicine and Pharmacy, Romania)*; Margus Punab (Tartu University Clinics, Estonia)*; Muhammed S Qadir (Sulaimani Polytechnic University, Iraq)*; Radwan F Qasrawi (Al-Quds University, State of Palestine)*; Qing Qiao (Boehringer Ingelheim, Germany)*; Mostafa Qorbani (Alborz University of Medical Sciences, Iran)*; Hedley K Quintana (Gorgas Memorial Institute for Studies of Health, Panama)*; Pedro J Quiroga-Padilla (Universidad de los Andes, Colombia)*; Tran Quoc Bao (Ministry of Health, Vietnam)*; Stefan Rach (Leibniz Institute for Prevention Research and Epidemiology - BIPS, Germany)*; Ivana Radic (University of Novi Sad, Serbia)*; Ricardas Radisauskas (Lithuanian University of Health Sciences, Lithuania)*; Salar Rahimikazerooni (Shiraz University of Medical Sciences, Iran)*; Mahfuzar Rahman (Pure Earth, Bangladesh)*; Mahmudur Rahman (Institute of Epidemiology Disease Control and Research, Bangladesh)*; Olli Raitakari (University of Turku, Finland)*; Manu Raj (Amrita Institute of Medical Sciences, India)*; Tamerlan Rajabov (UNICEF, Azerbaijan)*; Sherali Rakhmatulloev (Ministry of Health and Social Protection, Tajikistan)*; Ivo Rakovac (World

Health Organization Regional Office for Europe, Denmark)*; Sudha Ramachandra Rao (National Institute of Epidemiology, India)*; Ambady Ramachandran (India Diabetes Research Foundation, India)*; Otim PC Ramadan (World Health Organization Country Office, South Sudan)*; Virgílio V Ramires (Instituto Federal Riograndense, Brazil)*; Manuel Ramirez-Zea (Institute of Nutrition of Central America and Panama, Guatemala)*; Jacqueline Ramke (University of Auckland, New Zealand)*; Elisabete Ramos (University of Porto Medical School, Portugal)*; Rafel Ramos (Institut Universitari d'Investigació en Atenció Primària Jordi Gol, Spain)*; Lekhraj Rampal (Universiti Putra Malaysia, Malaysia)*; Sanjay Rampal (University of Malaya, Malaysia)*; Sheena E Ramsay (Newcastle University, UK)*; Lalka S Rangelova (National Centre of Public Health and Analyses, Bulgaria)*; Vayia Rarra (Sotiria Hospital, Greece)*; Ramon A Rascon-Pacheco (Instituto Mexicano del Seguro Social, Mexico)*; Mohammad-Mahdi Rashidi (Non-Communicable Diseases Research Center, Iran)*; Cassiano Ricardo Rech (Federal University of Santa Catarina, Brazil)*; Josep Redon (University of Valencia, Spain)*; Paul Ferdinand M Reganit (University of the Philippines, Philippines)*; Valéria Regecová (Slovak Academy of Sciences, Slovakia)*; Jane DP Renner (University of Santa Cruz do Sul, Brazil)*; Judit A Repasy (University of Pécs, Hungary)*; Cézane P Reuter (University of Santa Cruz do Sul, Brazil)*; Luis Revilla (Universidad San Martín de Porres, Peru)*; Andrew Reynolds (University of Otago, New Zealand)*; Negar Rezaei (Tehran University of Medical Sciences, Iran)*; Abbas Rezaianzadeh (Shiraz University of Medical Sciences, Iran)*; Yeunsook Rho (National Health Insurance Service, Republic of Korea)*; Lourdes Ribas-Barba (Nutrition Research Foundation, Spain)*; Robespierre Ribeiro (Minas Gerais State Secretariat for Health, Brazil; deceased)*; Elio Riboli (Imperial College London, UK)*; Fernando Rigo (CS S. Agustín Ibsalut, Spain)*; Attilio Rigotti (Pontificia Universidad Católica de Chile, Chile)*; Natascia Rinaldo (University of Ferrara, Italy)*; Tobias F Rinke de Wit (Amsterdam Institute for Global Health and Development, The Netherlands)*; Ulf Risérus (Uppsala University, Sweden)*; Ana I Rito (National Institute of Health Doutor Ricardo Jorge, Portugal)*; Raphael M Ritti-Dias (Universidade Nove de Julho, Brazil)*; Juan A Rivera (National Institute of Public Health, Mexico)*; Reina G Roa (Ministerio de Salud, Panama)*;

Louise Robinson (Newcastle University, UK)*; Romana Roccaldo (Council for Agricultural Research and Economics, Italy)*; Daniela Rodrigues (University of Coimbra, Portugal)*; Fernando Rodríguez-Artalejo (Universidad Autónoma de Madrid CIBERESP, Spain)*; María del Cristo Rodriguez-Perez (Canarian Health Service, Spain)*; Laura A Rodríguez-Villamizar (Universidad Industrial de Santander, Colombia)*; Andrea Y Rodríguez (Ministery of Health and Social Protection, Colombia)*; Ulla Roggenbuck (University of Duisburg-Essen, Germany)*; Peter Rohloff (Wuqu' Kawoq, Guatemala)*; Fabian Rohner (GroundWork, Switzerland)*; Rosalba Rojas-Martinez (National Institute of Public Health, Mexico)*; Nipa Rojroongwasinkul (Mahidol University, Thailand)*; Dora Romaguera (CIBEROBN, Spain)*; Elisabetta L Romeo (Associazione Calabrese di Epatologia, Italy)*; Rafaela V Rosario (University of Minho, Portugal)*; Annika Rosengren (University of Gothenburg, Sweden; Sahlgrenska University Hospital, Sweden)*; Ian Rouse (Fiji National University, Fiji)*; Vanessa Rouzier (GHESKIO Clinics, Haiti)*; Joel GR Roy (Statistics Canada, Canada)*; Maira H Ruano (Universidad de San Carlos, Guatemala)*; Adolfo Rubinstein (Institute for Clinical Effectiveness and Health Policy, Argentina)*; Frank J Rühli (University of Zurich, Switzerland)*; Jean-Bernard Ruidavets (Toulouse University School of Medicine, France)*; Blanca Sandra Ruiz-Betancourt (Instituto Mexicano del Seguro Social, Mexico)*; Maria Ruiz-Castell (Luxembourg Institute of Health, Luxembourg)*; Emma Ruiz Moreno (National Center for Epidemiology CIBERESP, Spain)*; Iuliia A Rusakova (Ufa Eye Research Institute, Russia)*; Wojciech Rusek (Rehamed-Center, Poland)*; Kenisha Russell Jonsson (Public Health Agency of Sweden, Sweden)*; Paola Russo (Institute of Food Sciences of the National Research Council, Italy)*; Petra Rust (University of Vienna, Austria)*; Marcin Rutkowski (Medical University of Gdansk, Poland)*; Marge Saamel (National Institute for Health Development, Estonia)*; Crizian G Saar (Universidade Federal de Minas Gerais, Brazil)*; Charumathi Sabanayagam (Singapore Eye Research Institute, Singapore)*; Hamideh Sabbaghi (Shahid Beheshti University of Medical Sciences, Iran)*; Elena Sacchini (Authority Sanitaria San Marino, San Marino)*; Harshpal S Sachdev (Sitaram Bhartia Institute of Science and Research, India)*; Alireza Sadjadi (Tehran University of Medical Sciences, Iran)*; Ali Reza

Safarpour (Shiraz University of Medical Sciences, Iran)*; Sare Safi (Shahid Beheshti University of Medical Sciences, Iran)*; Saeid Safiri (Tabriz University of Medical Sciences, Iran)*; Mohammad Hossien Saghi (Sabzevar University of Medical Sciences, Iran)*; Olfa Saidi (University Tunis El Manar, Tunisia)*; Calogero Saieva (Institute for Cancer Research, Prevention and Clinical Network, Italy)*; Satoko Sakata (Kyushu University, Japan)*; Nader Saki (Ahvaz Jundishapur University of Medical Sciences, Iran)*; Sanja Šalaj (University of Zagreb, Croatia)*; Benoit Salanave (French Public Health Agency, France)*; Eduardo Salazar Martinez (National Institute of Public Health, Mexico)*; Akkumis Salhanova (Academy of Preventive Medicine, Kazakhstan)*; Diego Salmerón (CIBERESP, Spain)*; Veikko Salomaa (Finnish Institute for Health and Welfare, Finland)*; Jukka T Salonen (University of Helsinki, Finland)*; Massimo Salvetti (University of Brescia, Italy)*; Margarita Samoutian (Kindergarten of Avlonari, Greece)*; Jose Sánchez-Abanto (National Institute of Health, Peru)*; Inés Sánchez Rodríguez (Instituto Murciano de Investigación Biosanitaria Virgen de la Arrixaca, Spain)*; Sandjaja (Ministry of Health, Indonesia)*; Susana Sans (Catalan Department of Health, Spain)*; Loreto Santa-Marina (Biogipuzkoa Health Research Institute, Spain)*; Ethel Santacruz (Ministerio de Salud Pública y Bienestar Social, Paraguay)*; Diana A Santos (Universidade de Lisboa, Portugal)*; Ina S Santos (Federal University of Pelotas, Brazil)*; Lèlita C Santos (Coimbra University Hospital Center, Portugal)*; Maria Paula Santos (University of Porto, Portugal)*; Osvaldo Santos (Instituto de Saúde Ambiental, Portugal)*; Rute Santos (University of Minho, Portugal)*; Tamara R Santos (Federal University of Alagoas, Brazil)*; Jouko L Saramies (Wellbeing Services County of South Karelia, Finland)*; Luis B Sardinha (Universidade de Lisboa, Portugal)*; Nizal Sarrafzadegan (Isfahan Cardiovascular Research Center, Iran)*; Thirunavukkarasu Sathish (Emory University, USA)*; Kai-Uwe Saum (German Cancer Research Center, Germany)*; Savvas Savva (Research and Education Institute of Child Health, Cyprus)*; Mathilde Savy (French National Research Institute for Sustainable Development, France)*; Norie Sawada (National Cancer Center, Japan)*; Mariana Sbaraini (Universidade Federal do Rio Grande do Sul, Brazil)*; Marcia Scazufca (University of São Paulo Clinics Hospital, Brazil)*; Beatriz D Schaan (Universidade Federal do

Rio Grande do Sul, Brazil)*; Angelika Schaffrath Rosario (Robert Koch Institute, Germany)*; Herman Schargrodsky (Hospital Italiano de Buenos Aires, Argentina)*; Anja Schienkiewitz (Robert Koch Institute, Germany)*; Karin Schindler (Medical University of Vienna, Austria)*; Sabine Schipf (University Medicine Greifswald, Germany)*; Börge Schmidt (University of Duisburg-Essen, Germany)*; Carsten O Schmidt (University Medicine Greifswald, Germany)*; Ida Maria Schmidt (Rigshospitalet, Denmark)*; Andrea Schneider (Helmholtz Zentrum München, Germany)*; Peter Schnohr (Copenhagen University Hospital - Bispebjerg and Frederiksberg, Denmark)*; Ben Schöttker (German Cancer Research Center, Germany)*; Sara Schramm (University of Duisburg-Essen, Germany)*; Stine Schramm (University of Southern Denmark, Denmark)*; Helmut Schröder (CIBERESP, Spain)*; Constance Schultsz (University of Amsterdam, The Netherlands)*; Gry Schultz (Nes Municipality, Norway)*; Matthias B Schulze (German Institute of Human Nutrition Potsdam-Rehbruecke, Germany)*; Aletta E Schutte (University of New South Wales, Australia; The George Institute for Global Health, Australia)*; Sylvain Sebert (University of Oulu, Finland)*; Moslem Sedaghattalab (Yasuj University of Medical Sciences, Iran)*; Rusidah Selamat (Ministry of Health, Malaysia)*; Vedrana Sember (University of Ljubljana, Slovenia)*; Abhijit Sen (Center for Oral Health Services and Research Mid-Norway, Norway)*; Idowu O Senbanjo (Lagos State University College of Medicine, Nigeria)*; Sadaf G Sepanlou (Tehran University of Medical Sciences, Iran)*; Guillermo Sequera (Ministerio de Salud Pública y Bienestar Social, Paraguay)*; Luis Serra-Majem (University of Las Palmas de Gran Canaria, Spain)*; Jennifer Servais (Statistics Canada, Canada)*; Ľudmila Ševčíková (Comenius University, Slovakia)*; Ronel Sewpaul (Human Sciences Research Council, South Africa)*; Svetlana Shalnova (National Medical Research Centre for Therapy and Preventive Medicine, Russia)*; Teresa Shamah-Levy (National Institute of Public Health, Mexico)*; Seyed Morteza Shamshirgaran (Neyshabur University of Medical Sciences, Iran)*; Coimbatore Subramaniam Shanthirani (Madras Diabetes Research Foundation, India)*; Maryam Sharafkhah (Tehran University of Medical Sciences, Iran)*; Sanjib K Sharma (B P Koirala Institute of Health Sciences, Nepal)*; Almaz Sharman (Academy of Preventive Medicine, Kazakhstan)*; Jonathan E Shaw (Baker Heart

and Diabetes Institute, Australia)*; Amaneh Shayanrad (Tehran University of Medical Sciences, Iran)*; Ali Akbar Shayesteh (Ahvaz Jundishapur University of Medical Sciences, Iran)*; Lela Shengelia (National Center for Disease Control and Public Health, Georgia)*; Zumin Shi (Qatar University, Qatar)*; Kenji Shibuya (King's College London, UK)*; Hana Shimizu-Furusawa (Teikyo University, Japan)*; Tal Shimony (Israel Center for Disease Control, Israel)*; Rahman Shiri (Finnish Institute of Occupational Health, Finland)*; Namuna Shrestha (Public Health Promotion and Development Organization, Nepal)*; Khairil Si-Ramlee (Ministry of Health, Brunei)*; Alfonso Siani (Institute of Food Sciences of the National Research Council, Italy)*; Rosalynn Siantar (Singapore Eye Research Institute, Singapore)*; Abla M Sibai (American University of Beirut, Lebanon)*; Labros S Sidossis (Rutgers University, USA)*; Natalia Silitrari (National Agency for Public Health, Moldova)*; Antonio M Silva (Federal University of Maranhão, Brazil)*; Caroline Ramos de Moura Silva (University of Pernambuco, Brazil)*; Diego Augusto Santos Silva (Federal University of Santa Catarina, Brazil)*; Kelly Samara Silva (Federal University of Santa Catarina, Brazil)*; Xueling Sim (National University of Singapore, Singapore; National University Health System, Singapore)*; Mary Simon (India Diabetes Research Foundation, India)*; Judith Simons (St Vincent's Hospital, Australia)*; Leon A Simons (University of New South Wales, Australia)*; Agneta Sjöberg (University of Gothenburg, Sweden)*; Michael Sjöström (Karolinska Institutet, Sweden; deceased)*; Elena V Skoblina (Institute for Demographic Research FCTAS RAS, Russia)*; Natalia A Skoblina (Pirogov Russian National Research Medical University, Russia)*; Tatyana Slazhnyova (National Center of Public Health, Kazakhstan)*; Jolanta Slowikowska-Hilczer (Medical University of Lodz, Poland)*; Przemysław Slusarczyk (International Institute of Molecular and Cell Biology, Poland)*; Liam Smeeth (London School of Hygiene & Tropical Medicine, UK)*; Hung-Kwan So (University of Hong Kong, China)*; Fernanda Cunha Soares (University of Pernambuco, Brazil)*; Grzegorz Sobek (University of Rzeszów, Poland)*; Eugène Sobngwi (University of Yaoundé 1, Cameroon)*; Morten Sodemann (University of Southern Denmark, Denmark)*; Stefan Söderberg (Umeå University, Sweden)*; Moesijanti YE Soekatri (Health Polytechnic Jakarta II Institute, Indonesia)*; Agustinus Soemantri

(Diponegoro University, Indonesia; deceased)*; Reecha Sofat (University College London, UK)*; Vincenzo Solfrizzi (University of Bari Aldo Moro, Italy)*; Yuliya V Solovieva (National Medical Research Center for Children's Health, Russia)*; Mohammad Hossein Somi (Tabriz University of Medical Sciences, Iran)*; Emily Sonestedt (Lund University, Sweden)*; Yi Song (Peking University, China)*; Sajid Soofi (The Aga Khan University, Pakistan)*; Thorkild IA Sørensen (University of Copenhagen, Denmark)*; Elin P Sørgjerd (Norwegian University of Science and Technology, Norway)*; Maroje Sorić (University of Zagreb, Croatia; University of Ljubljana, Slovenia)*; Charles Sossa Jérome (Institut Régional de Santé Publique, Benin)*; Victoria E Soto-Rojas (Universidad Icesi, Colombia)*; Aïcha Soumaré (University of Bordeaux, France)*; Alfonso Sousa-Poza (University of Hohenheim, Germany)*; Slavica Sovic (University of Zagreb, Croatia)*; Bente Sparboe-Nilsen (Oslo Metropolitan University, Norway)*; Karen Sparrenberger (Universidade Federal do Rio Grande do Sul, Brazil)*; Phoebe R Spencer (University of Western Australia, Australia)*; Angela Spinelli (Istituto Superiore di Sanità, Italy)*; Igor Spiroski (Institute of Public Health, North Macedonia; Ss. Cyril and Methodius University, North Macedonia)*; Jan A Staessen (KU Leuven, Belgium)*; Hanspeter Stamm (Lamprecht und Stamm Sozialforschung und Beratung AG, Switzerland)*; Andreas Stang (University of Duisburg-Essen, Germany)*; Gregor Starc (University of Ljubljana, Slovenia)*; Kaspar Staub (University of Zurich, Switzerland)*; Bill Stavreski (Heart Foundation, Australia)*; Jostein Steene-Johannessen (Norwegian School of Sport Sciences, Norway)*; Peter Stehle (Bonn University, Germany)*; Aryeh D Stein (Emory University, USA)*; Silje Steinsbekk (Norwegian University of Science and Technology, Norway)*; George S Stergiou (National and Kapodistrian University of Athens, Greece)*; Jochanan Stessman (Hadassah University Medical Center, Israel)*; Ranko Stevanović (Croatian Institute of Public Health, Croatia)*; Jutta Stieber (Helmholtz Zentrum München, Germany; deceased)*; Doris Stöckl (Helmholtz Zentrum München, Germany)*; Jakub Stokwiszewski (National Institute of Public Health - National Institute of Hygiene, Poland)*; Ekaterina Stoyanova (Kalina Malina Kindergarten, Bulgaria)*; Gareth Stratton (Swansea University, UK)*; Karien Stronks (University of Amsterdam, The Netherlands)*; Maria Wany Strufaldi (Federal University of São

Paulo, Brazil)*; Lela Sturua (National Center for Disease Control and Public Health, Georgia)*; Ramón Suárez-Medina (National Institute of Hygiene, Epidemiology and Microbiology, Cuba)*; Milton F Suarez-Ortegón (Pontificia Universidad Javeriana Seccional Cali, Colombia)*; Phalakorn Suebsamran (Ubon Ratchathani University, Thailand)*; Mindy Sugiyama (Ministry of Health and Human Services, Palau)*; Machi Suka (The Jikei University School of Medicine, Japan)*; Gerhard Sulo (University of Bergen, Norway)*; Chien-An Sun (Fu Jen Catholic University, Taiwan)*; Liang Sun (Shanghai Institute of Nutrition and Health of Chinese Academy of Sciences, China)*; Malin Sund (Umeå University, Sweden)*; Johan Sundström (Uppsala University, Sweden)*; Yn-Tz Sung (The Chinese University of Hong Kong, China)*; Jordi Sunyer (Barcelona Institute for Global Health, Spain)*; Paibul Suriyawongpaisal (Mahidol University, Thailand)*; Nabil William G Sweis (University of Jordan, Jordan)*; Boyd A Swinburn (University of Auckland, New Zealand)*; Rody G Sy (University of the Philippines, Philippines)*; René Charles Sylva (National Statistical Office, Cabo Verde)*; Lucjan Szponar (National Institute of Public Health - National Institute of Hygiene, Poland)*; Lorraine Tabone (Primary Health Care, Malta)*; E Shyong Tai (National University of Singapore, Singapore; National University Health System, Singapore)*; Furusawa Takuro (Kyoto University, Japan)*; Konstantinos D Tambalis (National and Kapodistrian University of Athens, Greece)*; Mari-Liis Tammesoo (University of Tartu, Estonia)*; Abdonas Tamosiunas (Lithuanian University of Health Sciences, Lithuania)*; Eng Joo Tan (Monash University, Australia)*; Xun Tang (Peking University, China)*; Maya Tanrygulyyeva (Scientific Research Institute of Maternal and Child Health, Turkmenistan)*; Frank Tanser (Stellenbosch University, South Africa)*; Yong Tao (Peking University, China)*; Mohammed Rasoul Tarawneh (Ministry of Health, Jordan)*; Jakob Tarp (Aarhus University, Denmark)*; Carolina B Tarqui-Mamani (National Institute of Health, Peru)*; Radka Taxová Braunerová (Institute of Endocrinology, Czech Republic)*; Anne Taylor (University of Adelaide, Australia)*; Julie Taylor (University College London, UK)*; Félicité Tchibindat (UNICEF, Niger)*; Saskia Te Velde (University of Applied Sciences Utrecht, The Netherlands)*; William R Tebar (Universidade Estadual Paulista, Brazil)*; Grethe S Tell (University of Bergen, Norway)*; Tania Tello (Universidad Peruana Cayetano Heredia, Peru)*;

Masresha Tessema (Ethiopian Public Health Institute, Ethiopia)*; Yih Chung Tham (Singapore Eye Research Institute, Singapore)*; KR Thankappan (Amrita Institute of Medical Sciences, India)*; Holger Theobald (Karolinska Institutet, Sweden)*; Xenophon Theodoridis (Aristotle University of Thessaloniki, Greece)*; Nihal Thomas (Christian Medical College Vellore, India)*; Barbara Thorand (Helmholtz Zentrum München, Germany)*; Amanda G Thrift (Monash University, Australia)*; Ľubica Tichá (Comenius University, Slovakia)*; Erik J Timmermans (University Medical Center Utrecht, The Netherlands)*; Dwi Hapsari Tjandrarini (National Research and Innovation Agency, Indonesia)*; Anne Tjonneland (Danish Cancer Institute, Denmark)*; Hanna K Tolonen (Finnish Institute for Health and Welfare, Finland)*; Janne S Tolstrup (University of Southern Denmark, Denmark)*; Maciej Tomaszewski (University of Manchester, UK)*; Murat Topbas (Karadeniz Technical University, Türkiye)*; Roman Topór-Mądry (Jagiellonian University Medical College, Poland)*; Liv Elin Torheim (Oslo Metropolitan University, Norway)*; Michael J Tornaritis (Research and Education Institute of Child Health, Cyprus)*; Maties Torrent (Institut d'Investigacio Sanitaria Illes Balears, Spain)*; Laura Torres-Collado (Universidad Miguel Hernandez, Spain)*; Stefania Toselli (University of Bologna, Italy)*; Giota Touloumi (National and Kapodistrian University of Athens, Greece)*; Pierre Traissac (French National Research Institute for Sustainable Development, France)*; Thi Tuyet-Hanh Tran (Hanoi University of Public Health, Vietnam)*; Mark S Tremblay (Children's Hospital of Eastern Ontario Research Institute, Canada)*; Areti Triantafyllou (Aristotle University of Thessaloniki, Greece)*; Dimitrios Trichopoulos (Harvard TH Chan School of Public Health, USA; deceased)*; Antonia Trichopoulou (Hellenic Health Foundation, Greece)*; Oanh TH Trinh (University of Medicine and Pharmacy at Ho Chi Minh City, Vietnam)*; Atul Trivedi (Government Medical College, India)*; Lechaba Tshepo (Sefako Makgatho Health Science University, South Africa)*; Maria Tsigga (International Hellenic University, Greece)*; Panagiotis Tsintavis (International Hellenic University, Greece)*; Shoichiro Tsugane (National Institutes of Biomedical Innovation, Health and Nutrition, Japan)*; John Tuitele (Department of Health, American Samoa; LBJ Hospital, American Samoa)*; Azaliia M Tuliakova (Ufa Eye Research Institute, Russia)*; Marshall K Tulloch-Reid (The University of the West Indies,

Jamaica)*; Fikru Tullu (Addis Ababa University, Ethiopia)*; Tomi-Pekka Tuomainen (University of Eastern Finland, Finland)*; Jaakko Tuomilehto (Finnish Institute for Health and Welfare, Finland)*; Maria L Turley (Ministry of Health, New Zealand)*; Gilad Twig (The Gertner Institute for Epidemiology and Health Policy Research, Israel; Israel Defense Forces Medical Corps, Israel)*; Per Tynelius (Karolinska Institutet, Sweden)*; Evangelia Tzala (Imperial College London, UK)*; Themistoklis Tzotzas (Hellenic Medical Association for Obesity, Greece)*; Christophe Tzourio (University of Bordeaux, France)*; Nwannedimma Udoji (Everyage Carolina Senior Care, USA)*; Peter Ueda (Karolinska Institutet, Sweden)*; Eunice Ugel (Universidad Centro-Occidental Lisandro Alvarado, Venezuela)*; Flora AM Ukoli (Meharry Medical College, USA)*; Hanno Ulmer (Medical University of Innsbruck, Austria)*; Belgin Unal (Dokuz Eylul University, Türkiye)*; Zhamyila Usupova (Republican Center for Health Promotion, Kyrgyzstan)*; Hannu MT Uusitalo (University of Tampere Tays Eye Center, Finland)*; Nalan Uysal (Sabiha Gokcen Ilkokulu, Türkiye)*; Justina Vaitkeviciute (Lithuanian University of Health Sciences, Lithuania)*; Gonzalo Valdivia (Pontificia Universidad Católica de Chile, Chile)*; Susana Vale (Polytechnic Institute of Porto, Portugal)*; Damaskini Valvi (Icahn School of Medicine at Mount Sinai, USA)*; Rob M van Dam (George Washington University, USA)*; Bert-Jan van den Born (University of Amsterdam, The Netherlands)*; Johan Van der Heyden (Sciensano, Belgium)*; Yvonne T van der Schouw (Utrecht University, The Netherlands)*; Koen Van Herck (Ghent University, Belgium)*; Wendy Van Lippevelde (Ghent University, Belgium)*; Hoang Van Minh (Hanoi University of Public Health, Vietnam)*; Natasja M Van Schoor (Vrije Universiteit Amsterdam, The Netherlands)*; Irene GM van Valkengoed (University of Amsterdam, The Netherlands)*; Dirk Vanderschueren (KU Leuven, Belgium)*; Diego Vanuzzo (MONICA-FRIULI Study Group, Italy)*; Anette Varbo (Copenhagen University Hospital, Denmark; University of Copenhagen, Denmark)*; Gregorio Varela-Moreiras (Universidad CEU San Pablo, Spain)*; Luz Nayibe Vargas (Pontificia Universidad Javeriana, Colombia)*; Patricia Varona-Pérez (National Institute of Hygiene, Epidemiology and Microbiology, Cuba)*; Senthil K Vasan (University of Southampton, UK)*; Daniel G Vasques (Universidade Federal do Rio Grande do Sul, Brazil)*; Radu Vatasescu (Carol Davila

University of Medicine and Pharmacy, Romania)*; Tomas Vega (Consejería de Sanidad Junta de Castilla y León, Spain)*; Toomas Veidebaum (National Institute for Health Development, Estonia)*; Gustavo Velasquez-Melendez (Universidade Federal de Minas Gerais, Brazil)*; Biruta Velika (Centre for Disease Prevention and Control, Latvia)*; Maïté Verloigne (Ghent University, Belgium)*; Giovanni Veronesi (University of Insubria, Italy)*; WM Monique Verschuren (National Institute for Public Health and the Environment, The Netherlands)*; Cesar G Victora (Federal University of Pelotas, Brazil)*; Giovanni Viegi (Institute of Clinical Physiology of National Research Council, Italy)*; Lucie Viet (National Institute for Public Health and the Environment, The Netherlands)*; Frøydis N Vik (University of Agder, Norway)*; Monica Vilar (Universidad San Francisco de Quito, Ecuador)*; Salvador Villalpando (National Institute of Public Health, Mexico)*; Jesus Vioque (CIBERESP, Spain)*; Napaphan Viriyautsahakul (Ministry of Public Health, Thailand)*; Jyrki K Virtanen (University of Eastern Finland, Finland)*; Marjolein Visser (Vrije Universiteit Amsterdam, The Netherlands)*; Sophie Visvikis-Siest (Université de Lorraine, France)*; Bharathi Viswanathan (Ministry of Health, Seychelles)*; Mihaela Vladulescu (Sunflower Nursery School, Romania)*; Tiina Vlasoff (North Karelia Center for Public Health, Finland)*; Dorja Vocanec (University of Zagreb, Croatia)*; Peter Vollenweider (Lausanne University Hospital, Switzerland; University of Lausanne, Switzerland)*; Henry Völzke (University Medicine Greifswald, Germany)*; Georgia Vourli (Academy of Athens, Greece)*; Ari Voutilainen (University of Eastern Finland, Finland)*; Martine Vrijheid (Barcelona Institute for Global Health CIBERESP, Spain)*; Tanja GM Vrijkotte (University of Amsterdam, The Netherlands)*; Silvije Vuletić (University of Zagreb, Croatia)*; Alisha N Wade (University of the Witwatersrand, South Africa)*; Thomas Waldhör (Medical University of Vienna, Austria)*; Janette Walton (Munster Technological University, Ireland)*; Elvis OA Wambiya (African Population and Health Research Center, Kenya)*; Wan Mohamad Wan Bebakar (Universiti Sains Malaysia, Malaysia)*; Wan Nazaimoon Wan Mohamud (Institute for Medical Research, Malaysia)*; Rildo de Souza Wanderley Júnior (University of Pernambuco, Brazil)*; Chongjian Wang (Zhengzhou University, China)*; Huijun Wang (Chinese Center for Disease Control and Prevention, China)*; Limin Wang (Chinese Center

of Disease Control and Prevention, China)*; Ming-Dong Wang (Public Health Agency of Canada, Canada)*; Ningli Wang (Capital Medical University Beijing Tongren Hospital, China)*; Qian Wang (Xinjiang Medical University, China)*; Xiangjun Wang (Shanghai Educational Development Co. Ltd, China)*; Ya Xing Wang (Capital Medical University, China)*; Ying-Wei Wang (Ministry of Health and Welfare, Taiwan)*; S Goya Wannamethee (University College London, UK)*; Nicholas Wareham (University of Cambridge, UK)*; Olivia Wartha (Ulm University Hospital, Germany)*; Adelheid Weber (Federal Ministry of Social Affairs, Health, Care and Consumer Protection, Austria)*; Karen Webster-Kerr (Ministry of Health and Wellness, Jamaica)*; Niels Wedderkopp (University of Southern Denmark, Denmark)*; Daniel Weghuber (Paracelsus Medical University, Austria)*; Wenbin Wei (Capital Medical University, China)*; Aneta Weres (University of Rzeszów, Poland)*; Bo Werner (Örebro University, Sweden)*; Leo D Westbury (University of Southampton, UK)*; Peter H Whincup (St George's, University of London, UK)*; Lars Wichstrøm (Norwegian University of Science and Technology, Norway)*; Kremlin Wickramasinghe (World Health Organization Regional Office for Europe, Denmark)*; Kurt Widhalm (Medical University of Vienna, Austria)*; Indah S Widyahening (Universitas Indonesia, Indonesia)*; Andrzej Więcek (Medical University of Silesia, Poland)*; Philipp S Wild (Johannes Gutenberg University, Germany)*; Rainford J Wilks (The University of the West Indies, Jamaica)*; Johann Willeit (Medical University of Innsbruck, Austria)*; Peter Willeit (Medical University of Innsbruck, Austria)*; Julianne Williams (World Health Organization Regional Office for Europe, Denmark)*; Tom Wilsgaard (UiT The Arctic University of Norway, Norway)*; James P Wirth (GroundWork, Switzerland)*; Bogdan Wojtyniak (National Institute of Public Health - National Institute of Hygiene, Poland)*; Meseret Woldeyohannes (Ethiopian Public Health Institute, Ethiopia)*; Kathrin Wolf (Helmholtz Zentrum München, Germany)*; Roy A Wong-McClure (Caja Costarricense de Seguro Social, Costa Rica)*; Andrew Wong (University College London, UK)*; Emily B Wong (Africa Health Research Institute, South Africa)*; Jyh Eiin Wong (Universiti Kebangsaan Malaysia, Malaysia)*; Tien Yin Wong (Duke-NUS Medical School, Singapore)*; Jean Woo (The Chinese University of Hong Kong, China)*; Mark Woodward (University of New South Wales, Australia;

Imperial College London, UK)*; Frederick C Wu (University of Manchester, UK)*; Hon-Yen Wu (National Yang Ming Chiao Tung University, Taiwan)*; Jianfeng Wu (Shandong University of Traditional Chinese Medicine, China)*; Li Juan Wu (Capital Medical University, China)*; Shouling Wu (Kailuan General Hospital, China)*; Justyna Wyszyńska (University of Rzeszów, Poland)*; Haiquan Xu (Institute of Food and Nutrition Development of Ministry of Agriculture and Rural Affairs, China)*; Liang Xu (Beijing Institute of Ophthalmology, China)*; Nor Azwany Yaacob (Universiti Sains Malaysia, Malaysia)*; Uruwan Yamborisut (Mahidol University, Thailand)*; Li Yan (Imperial College London, UK)*; Weili Yan (Children's Hospital of Fudan University, China)*; Ling Yang (University of Oxford, UK)*; Xiaoguang Yang (Chinese Center for Disease Control and Prevention, China)*; Yang Yang (Shanghai Educational Development Co. Ltd, China)*; Nazan Yardim (Ministry of Health, Türkiye)*; Tabara Yasuharu (Kyoto University, Japan)*; Martha Yépez García (Universidad San Francisco de Quito, Ecuador)*; Panayiotis K Yiallouros (University of Cyprus, Cyprus)*; Agneta Yngve (Uppsala University, Sweden)*; Moein Yoosefi (Non-Communicable Diseases Research Center, Iran)*; Akihiro Yoshihara (Niigata University, Japan)*; Yoto Yotov (Medical University - Varna, Bulgaria)*; Qi Sheng You (Capital Medical University, China)*; San-Lin You (Fu Jen Catholic University, Taiwan)*; Novie O Younger-Coleman (The University of the West Indies, Jamaica)*; Yu-Ling Yu (KU Leuven, Belgium)*; Yunjiang Yu (South China Institute of Environmental Sciences, China)*; Safiah Md Yusof (International Medical University, Malaysia)*; Ahmad Faudzi Yusoff (Ministry of Health, Malaysia)*; Luciana Zaccagni (University of Ferrara, Italy)*; Vassilis Zafiropulos (Hellenic Mediterranean University, Greece)*; Ahmad A Zainuddin (Ministry of Health, Malaysia)*; Seyed Rasoul Zakavi (Mashhad University of Medical Sciences, Iran)*; Farhad Zamani (Iran University of Medical Sciences, Iran)*; Sabina Zambon (University of Padua, Italy)*; Antonis Zampelas (Agricultural University of Athens, Greece)*; Hana Zamrazilová (Institute of Endocrinology, Czech Republic)*; Maria Elisa Zapata (Centro de Estudios Sobre Nutrición Infantil, Argentina)*; Abdul Hamid Zargar (Center for Diabetes and Endocrine Care, India)*; Ko Ko Zaw (University of Public Health, Myanmar)*; Ayman A Zayed (University of Jordan, Jordan)*; Tomasz Zdrojewski (Medical University of Gdańsk, Poland)*;

Magdalena Żegleń (Jagiellonian University, Poland)*; Kristyna Zejglicova (National Institute of Public Health, Czech Republic)*; Tajana Zeljkovic Vrkic (University Hospital Centre Zagreb, Croatia)*; Yi Zeng (Peking University, China; Duke University, USA)*; Andrea Zentai (National Centre for Public Health and Pharmacy, Hungary)*; Bing Zhang (Chinese Center for Disease Control and Prevention, China)*; Luxia Zhang (Peking University First Hospital, China)*; Zhen-Yu Zhang (KU Leuven, Belgium)*; Dong Zhao (Capital Medical University Beijing An Zhen Hospital, China)*; Ming-Hui Zhao (Peking University First Hospital, China)*; Wenhua Zhao (Chinese Center for Disease Control and Prevention, China)*; Yanitsa V Zhecheva (Bulgarian Academy of Sciences, Bulgaria)*; Shiqi Zhen (Jiangsu Provincial Center for Disease Control and Prevention, China)*; Wei Zheng (Vanderbilt University, USA)*; Yingfeng Zheng (Sun Yat-sen University, China)*; Bekbolat Zholdin (West Kazakhstan Medical University, Kazakhstan)*; Maigeng Zhou (Chinese Center for Disease Control and Prevention, China)*; Dan Zhu (Inner Mongolia Medical University, China)*; Paul Zimmet (Monash University, Australia)*; Marie Zins (Institut National de la Santé et de la Recherche Médicale, France; Paris Cité University, France)*; Emanuel Zitt (Agency for Preventive and Social Medicine, Austria)*; Yanina Zocalo (Universidad de la República, Uruguay)*; Nada Zoghlami (National Institute of Public Health, Tunisia)*; Julio Zuñiga Cisneros (Instituto Conmemorativo Gorgas de Estudios de la Salud, Panama)*; Monika Zuziak (Przedszkole No. 81, Poland)*

**Appendix Text 2.** Data sources

*Data access*

We used data from a population-based database on cardiometabolic risk factors collated by the Non-Communicable Disease Risk Factor Collaboration (NCD-RisC), as detailed previously.[1-5] Data were obtained from publicly available multi-country and national measurement surveys (e.g., Demographic and Health Surveys, WHO STEPwise approach to Surveillance (STEPS) surveys, and those identified via the Inter-University Consortium for Political and Social Research, European Health Interview and Health Examination Surveys Database, and the UK Data Service). With the help of the World Health Organization (WHO) regional and country offices, we identified and accessed population-based survey data from national health and statistical agencies. We searched and reviewed published studies as detailed previously and invited eligible studies to join NCD-RisC,[1-5] as we did with data holders from a previous pooled analyses of cardiometabolic risk factors.[6-9] The NCD-RisC database is continuously updated through all the above routes as well as through periodic requests to NCD-RisC members, which consist of representatives of participating studies who are knowledgeable with the study's methods and measurements and often with other studies in their countries and regions, to suggest additional sources in their countries.

*Data inclusion criteria and characteristics of included studies*

We carefully checked that each data source met our inclusion criteria, described below. Potential duplicate data sources were first identified by comparing studies from the same country and year, followed by checking with NCD-RisC members who had provided data whether sources from the same country and year, and with similar sample sizes and age ranges, were the same or distinct. If two sources were confirmed as duplicates, only one was maintained and used. All NCD-RisC members were also periodically asked to review the list of sources from their countries, to verify that the included data met the inclusion criteria, were not duplicates, and were appropriately classified in terms of the population that they had sampled from.

For each data source, we recorded the study population, the sampling approach, the years of measurement and the measurement methods. This information was provided by participating studies together with available documentation on study design and methods. Using this information, data sources were classified in terms of whether they covered the whole country, one or more subnational regions (that is one or more provinces or states, more than three cities, or more than five rural communities), or one or a small number of communities (limited geographical scope not meeting the above national or subnational criteria). As stated in Appendix Text 3, these study-level attributes were used in the Bayesian hierarchical meta-regression model that was used to estimate prevalence of body-mass index (BMI) categories, while taking into account differences in the populations from which different studies had sampled. All submitted data were checked by at least two persons independently. Questions and clarifications were discussed with NCD-RisC members and resolved before data were incorporated into the database.

Data were included if the following criteria were met: measured data on height and weight were available; study participants were five years of age and older; data were collected using a probabilistic sampling method with a defined sampling frame; data were from population samples at the national, subnational, or community level as defined above; and data were from the countries and territories listed in Appendix Table 2.

We excluded all data sources that were solely based on self-reported height and weight without a measurement component because these data are subject to biases that vary by geography, time, age, sex and socioeconomic characteristics.[10-12] Due to these variations, approaches to correcting self-reported data may leave residual bias. We also excluded data sources on population subgroups whose anthropometric status may differ systematically from the general population, including the following: studies that had included or excluded people based on their health status; studies whose participants were only specific educational,

occupational, socioeconomic or ethnic subgroups of the general population; studies that recruited participants or collected data through educational or health facilities, with the exceptions noted below; and females aged 15-19 years in surveys which sampled only ever-married women or measured height and weight only among mothers.

We used school-based data in countries and age-sex groups with school enrolment of 70% or higher. We used studies whose sampling frame was health insurance schemes in countries where at least 80% of the population had insurance coverage, financed publicly, privately or through employment. Finally, we used studies that had recruited participants or data collected through general practice and primary care systems in high-income and central European countries with universal insurance because contact with the primary care systems tends to be as good as or better than response rates for population-based surveys.[13]

*Data cleaning and management*

We excluded participants whose age was <18 years if their data were not reported by single year of age (<0.01% of all participants), because height and weight may have non-linear age associations in these ages, especially during growth spurts. We excluded BMI data for females who were pregnant at the time of measurement (0.19% of all participants), because weight changes during pregnancy. We excluded 0.23% of participants with recorded values outside of predefined ranges, which were likely to reflect measurement or data recording errors: those who had recorded height <60 cm or >180 cm for ages <10 years; <80 cm or >200 cm for ages 10-14 years; <100 cm or >250 cm for ages ≥15 years, recorded weight <5 kg or >90 kg for age <10 years; <8 kg or >150 kg for ages 10-14 years; <12 kg or >300 kg for ages ≥15 years, or recorded BMI <6 kg/m$^2$ or >40 kg/m$^2$ for ages <10 years; <8 kg/m$^2$ or >60 kg/m$^2$ for ages 10-14 years; <10 kg/m$^2$ or >80 kg/m$^2$ for ages ≥15 years.

Anonymised individual data from the studies from 1980 to 2022 in the NCD-RisC database were reanalysed according to a common protocol. We calculated prevalence in the following

BMI ranges: for children and adolescents, prevalence of BMI <-2SD, -2SD to <-1SD, -1SD to 1SD, >1 SD to 2SD, and >2SD;[14] for adults, prevalence of BMI <18.5 kg/m$^2$, 18.5 kg/m$^2$ to <20 kg/m$^2$, 20 kg/m$^2$ to <25 kg/m$^2$, 25 kg/m$^2$ to <30 kg/m$^2$, 30 kg/m$^2$ to <35 kg/m$^2$, 35 kg/m$^2$ to <40 kg/m$^2$, and ≥40 kg/m$^2$.

76% of the studies with BMI data were included in the NCD-RisC database as individual participant data; another 16% were provided as summary statistics, i.e., age–sex specific prevalence of relevant BMI categories. When summary statistics were prepared by study investigators, detailed instructions were provided, as was computer code when requested, to ensure analysis was conducted according to the study protocol. The cut-offs for calculating prevalence in the BMI categories for school-aged children and adolescents were all age-specific and sex-specific and were applied to data in single years of age. All analyses incorporated sample weights and complex survey design, when applicable, in calculating summary statistics. Information on survey design and sample weights were provided by participating studies. For studies which used multistage (stratified) sampling, we accounted for survey design features when calculating standard errors, including clusters, strata and sample weights, using Taylor series linearisation as implemented in the R package ‘survey’ v4.2.1.[15]

We used two additional types of data sources, accounting for 8% of studies. First, we had some data from a previous pooling analysis.[6] We invited these studies to join NCD-RisC, as stated above. However, data from some studies were no longer available, for example because the authors had retired or moved, or because data had been permanently archived or stored using older storage technologies that could not be easily retrieved. Second, summary statistics for nationally representative data from sources that were identified but not accessed via the above routes were extracted from published reports. Data were also extracted for two STEPS surveys that were not publicly available.[16,17] These data made up 2.4% of our data points for children and adolescents and 9.6% for adults (a data point is an

age-sex-study-specific prevalence in a BMI category which is used in the Bayesian meta-regression model as described below to make estimates for all age groups, countries and years). These studies had information on mean BMI and/or on a subset of BMI categories that were analysed in this work. In order to use these data, we used previously validated conversion regressions to estimate the missing primary outcome from the available BMI metric(s). Additional details on conversion regression model specifications and the model coefficients are reported at https://github.com/NCD-RisC/ncdrisc-methods/.

**Appendix Text 3.** Statistical methods

*Overview*

We used a Bayesian hierarchical meta-regression model, fitted using a Markov chain Monte Carlo (MCMC) sampler, with inference made using posterior MCMC samples, to estimate trends in the prevalence of different BMI categories by sex, age, country, and year from 1980 to 2022. We used data from 1980 so that estimates for the early part of the reporting period, when data were more sparse, were also informed by data in preceding years. The categories analysed were: for children and adolescents, prevalence of BMI <-2SD, -2SD to <-1SD, -1SD to 1SD, >1 SD to 2SD, and >2SD;[14] for adults, prevalence of BMI <18.5 $kg/m^2$, 18.5 $kg/m^2$ to <20 $kg/m^2$, 20 $kg/m^2$ to <25 $kg/m^2$, 25 $kg/m^2$ to <30 $kg/m^2$, 30 $kg/m^2$ to <35 $kg/m^2$, 35 $kg/m^2$ to <40 $kg/m^2$, and ≥40 $kg/m^2$. As stated in the main paper, we report trends from 1990.

The original version of the model is described in detail in a statistical paper[18] and related substantive papers.[4,9,19] The model used in this paper included adaptations that better capture the variations over age and time of BMI, described in previous publications.[3,20] Model specification is summarised here and described using statistical notation below together with details of its implementation and computation.

In summary, we organised countries into 20 regions, mostly based on geography and national income, which were further grouped into eight super-regions (Appendix Table 2). The super-regions were: Central and eastern Europe, Central Asia, Middle East and north Africa, High-income western, Latin America and the Caribbean, Oceania, South Asia, East and southeast Asia and the Pacific, sub-Saharan Africa. The model had a hierarchical structure in which estimates for each country and year were informed by its own data, if available, and by data from other years in the same country and from other countries, especially those in the same region and super-region with data for similar time periods. The extent to which estimates for each country-year were influenced by data from other years and other countries depended on whether the country had data, the sample size of data, whether the sources were at national,

subnational or community level, and the within-country and within-region variability of the available data.

The model included non-linear time trends through a combination of linear and second-order random walk terms, all modelled hierarchically. The age association of BMI was modelled using a cubic spline to allow non-linear age patterns, which might vary across countries. The coefficients of the splines were modelled hierarchically.[3,20] As detailed below, for adults, we allowed the coefficients to vary over time to reflect changing age associations,[20] whereas for children and adolescents, a simpler model without age-time interaction had better performance.[3]

The model accounted for the possibility that BMI in subnational and community samples might systematically differ from, and have larger variation than, nationally representative surveys through the inclusion of fixed-effect and random-effect terms. The fixed effects adjusted for systematic differences between subnational or community studies and national studies and allowed these differences to vary over time. The random effects allowed national data to have a larger influence on the estimates than subnational or community data with similar sample sizes. The model also accounted for urban-rural differences in the prevalence of a BMI category through data-driven fixed effects for urban-only and rural-only studies. These urban and rural effects were weighted by the difference between study-level and country-level urbanisation in the year when the study was conducted and were also permitted to vary across time.

We performed all analyses separately for each of the prevalence categories given above and by sex because there were differences in their levels and trends in relation to category and sex.[1,5] We also conducted separate analyses for adults and for school-aged children and adolescents. Data on participants aged 5-19 years were included in the analysis of trends in children and adolescents, and data on participants aged 18 years and older were included in

the analysis of trends in adults. Data on participants aged 18 and 19 years were included in both sets of models because these groups form a transitional age from adolescence to adulthood, and hence inform the estimates in both groups.

*Statistical model specification*

This section, and the following sections, describe the specification of the Bayesian meta-regression model used to estimate the prevalence of each of the BMI categories listed above.

Each study contributed up to 15 data points for each BMI category and sex, with the exact number depending on the age groups represented in the study. In the model specification an observation $y_{h,i}$, that is the number of people in the prevalence category from age group $h$ of study $i$, carried out in country $j$ at time $t$, was specified to have a binomial distribution with sample size $n_{h,i}$. The distribution of $y_{h,i}$ given prevalence $p_{h,i}$ was

$$y_{h,i} \sim Bin\left(n_{h,i},\ p_{h,i}\right).$$

We modelled the prevalence $p_{h,i}$ via a latent variable $\alpha_{h,i} = \Phi^{-1}\left(p_{h,i}\right)$, representing probit-transformed prevalence, which follows a Gaussian distribution:

$$\alpha_{h,i} \sim N\left(a_{j[i]} + b_{j[i]}t[i] + u_{j[i],t[i]} + \gamma_i(z_h) + \boldsymbol{X_i\beta} + e_i, \tau^2\right)$$

where $j$, the country in which a study was carried out, and $t$, the study year, are uniquely determined by the study index $i$; we denote this determination of $j$ and $t$ on $i$ by $j[i]$ and $t[i]$ respectively. The country-specific intercept and linear time slope from country $j$ are denoted $a_j$ and $b_j$ respectively, with $j \in \{1,..J\}$, where $J$ = 200 is the total number of countries and territories in our analysis. We describe the hierarchical model used for the $a$'s and the $b$'s in the section *Linear components of country time trends*. Letting $T$ = 43 be the total number of years from 1980 to 2022, the $T$-length vector $u_j$ captures smooth non-linear change over time in country $j$, as described in the section *Nonlinear change*. The contribution of the age term for age group $h$ (with mid-age $z_h$) in study $i$ is denoted by $\gamma_i(z_h)$; these are described in detail in the section *Age model*. The matrix $\boldsymbol{X}$ contains terms describing whether studies were

representative at the national, subnational or community level, and whether they were urban-only, rural-only, or covered both areas, and $\boldsymbol{\beta}$ contains the associated fixed effects. In addition, a random effect $e_i$ was estimated for each study. These study-specific terms are described in the section *Study-level terms and study-specific random effects*. The variance term $\tau^2$ captures variability not accounted for by the study-specific random effects, described in the section *Residual age-by-study variability*. Details on model fitting and convergence are given in the section *Model implementation.* Finally, details on how country-level inference was performed are given in the section *Model inference.*

*Linear components of country time trends*

The model had a hierarchical structure, whereby studies were nested in countries, which were nested in regions (indexed by $l$), which were nested in super-regions (indexed by $m$), which were all nested in the globe (see Appendix Table 2 for a list of countries and territories in each region, and regions in each super-region). This structure allowed the model to share information across units to a greater degree when data were non-existent or weakly informative (for example, had a small sample size or were not nationally representative) and, to a lesser extent, in data-rich countries and regions.[21]

The $a$ and $b$ terms are country-specific linear intercepts and time slopes with terms at each level of the hierarchy, denoted by the superscripts *c*, *r*, *s* and *g*, respectively:

$$a_j = a_j^c + a_{l[j]}^r + a_{m[j]}^s + a^g,$$

$$b_j = b_j^c + b_{l[j]}^r + b_{m[j]}^s + b^g,$$

$$a^x \sim N(0, \kappa_a^x),$$

$$b^x \sim N(0, \kappa_b^x),$$

where $x \in \{c, r, s\}$. The $\kappa$ terms were each assigned a flat prior on the standard deviation scale.[21] We also assigned flat priors to $a^g$ and $b^g$.

*Nonlinear change*

The prevalence of a BMI category may change nonlinearly over time.[1,4] We captured smooth nonlinear change in time in country $j$ using the vector $u_j$. Just as $a_j$ and $b_j$ are each defined as the sum of country, region, super-region and global components, we defined

$$u_j = u_j^c + u_{l[j]}^r + u_{m[j]}^s + u^g.$$

To allow the model to differentiate between the degrees of nonlinearity that exist at the country, region, super-region and global levels, we assigned the four components of each $u$ a discrete second-order Gaussian autoregressive prior.[22,23] In particular, the vectors $u_j^c, j \in \{1, \dots, J\}$, $u_l^r, l \in \{1, \dots, L\}$, $u_m^s, m \in \{1, \dots, M\}$, and $u^g$, all of length $T$, are each given a Gaussian prior with mean zero and precision $\lambda_c P$, $\lambda_r P$, $\lambda_s P$ and $\lambda_g P$ respectively, where the scaled precision matrix *P* in the Gaussian autoregressive prior penalizes first and second differences as follows:

$$P = \begin{bmatrix} 1 & 0 & 0 & \cdots & 0 \\ -2 & 1 & 0 & \cdots & 0 \\ 1 & -2 & 1 & \cdots & 0 \\ 0 & 1 & -2 & \cdots & 0 \\ 0 & 0 & 1 & \cdots & 0 \\ \vdots & \vdots & \vdots & \ddots & \vdots \\ 0 & 0 & 0 & \cdots & 1 \end{bmatrix} \begin{bmatrix} 1 & -2 & 1 & 0 & 0 & \cdots & 0 \\ 0 & 1 & -2 & 1 & 0 & \cdots & 0 \\ 0 & 0 & 1 & -2 & 1 & \cdots & 0 \\ \vdots & \vdots & \vdots & \vdots & \vdots & \ddots & \vdots \\ 0 & 0 & 0 & 0 & 0 & \cdots & 1 \end{bmatrix}$$

$$= \begin{bmatrix} 1 & -2 & 1 & 0 & 0 & \cdots & 0 \\ -2 & 5 & -4 & 1 & 0 & \cdots & 0 \\ 1 & -4 & 6 & -4 & 1 & \cdots & 0 \\ 0 & 1 & -4 & 6 & -4 & \cdots & 0 \\ 0 & 0 & 1 & -4 & 6 & \cdots & 0 \\ \vdots & \vdots & \vdots & \vdots & \vdots & \ddots & \vdots \\ 0 & 0 & 0 & 0 & 0 & \cdots & 1 \end{bmatrix}.$$

*P* is multiplied by the estimated precision parameters $\lambda_c$, $\lambda_r$, $\lambda_s$, and $\lambda_g$, thus upweighting or downweighting the strength of its penalties and ultimately determining the degree of smoothing at each level. For each of the four precision parameters, we used a truncated flat prior on the standard deviation scale ($1/\sqrt{\lambda}$).[21] We truncated these priors such that $\log\lambda \leq 20$ for each of the four $\lambda's$. This upper bound is enforced as a computational convenience, whereby models with $\log\lambda > 20$ are treated as equivalent to a model with $\log\lambda = 20$, as they

essentially have no extra-linear variability in time. In practice, this upper bound had little effect on the parameter estimates. Furthermore, we ordered the $\lambda's$ a priori as follows: $\lambda_c < \lambda_r < \lambda_s < \lambda_g$. This prior constraint conveys the expectation that the global trend in the prevalence of a BMI category has less extra-linear variability than the trend of any given super-region, which has less than those of constituent regions, which in turn has less variability than the trends of constituent countries.

The matrix $P$ has rank $T-2$, corresponding to a flat, improper prior on the mean and the slope of the $u_j^c$'s, the $u_l^r$'s, the $u_m^s$'s and $u^g$, and is not invertible.[24] Thus, we had a proper prior in a reduced-dimension space,[25] with the prior expressed as follows:

$$P\left(u_j^c \middle| \lambda_c\right) \propto \lambda_c^{\frac{T-2}{2}} \exp\left\{-\frac{\lambda_c}{2} u_j^{c\prime} P u_j^c\right\}.$$

Note that if $u_j^c$ had a non-zero mean, this would introduce non-identifiability with respect to $a_j^c$. By the same token, $b_j^c$ would not be identifiable if $u_j^c$ had a non-zero time slope, and similarly for the other means and slopes. Thus, to achieve identifiability of the $a$'s, $b$'s, and $u$'s, we constrained the mean and slope of $u^g$ and each of $u^s$, $u^r$, and $u^c$ to be zero. Enforcing orthogonality between the linear and nonlinear portions of the time trends meant that each can be interpreted independently.

For the countries in which there are observations for at least two different time points, this improper prior will not lead to an improper posterior because the data will provide information about the mean and slope. In order to enforce the desired orthogonality between the linear and nonlinear portions of the model, we used the Rue and Held correction.[25] For the countries without data (for adults, four for women and eight for men; for children and adolescents, ten for girls and 20 for boys), we took the Moore-Penrose pseudoinverse of $P$,[26] setting to infinity those eigenvalues that correspond to the non-identifiability. This effectively constrained the non-identified portions of the model to zero, as the corresponding variances are set to zero;[23]

in this case the Rue and Held correction[25] is not needed. An intermediate case occurs when data are observed for only one time point in a country. In this case, the full conditional precision has rank $T-1$ because the mean but not the linear trend of $u_j^c$ is identified by the data. We therefore constrained the linear trend of $u_j^c$ to zero in this case, by taking the generalized inverse of the full conditional precision. We then constrained the mean of $u_j^c$ to zero using the one-dimensional version of the Rue and Held correction.[25] Computational details are given in a previous paper.[9]

*Age model*

We sought a smooth function that could characterise gradual changes in BMI over age, as seen in the data, with parsimonious number of parameters to allow robust estimation. To achieve this, we modelled age using cubic splines, with the number and position of the knots of the spines selected based on epidemiological and physiological knowledge about changes in body shape[14,27] and statistical considerations, as previously described.[1,5] Statistically, we used age stratified residuals to confirm the number and position of knots.

For age group $h$ with mid-age $z_h$, in study $i$, the age term is given by

$$\gamma_i(z_h) = \gamma_{1,i} z_h + \gamma_{2,i} z_h^2 + \gamma_{3,i} z_h^3 + \gamma_{4,i}(z_h - k_1)_+^3 + \gamma_{5,i}(z_h - k_2)_+^3,$$

where for children and adolescents the two knots were placed at ages $(k_1, k_2) = (10, 15)$ and for adults at $(k_1, k_2) = (45, 60)$ years. To reduce dependence among model parameters, we centred the age variable.

We used different age models for children and adolescents and for adults, as explained below, following previous analyses,[3,5,20] visual inspection of results as well as formal model testing carried out using the Watanabe-Akaike information criterion (WAIC).[28,29]

For adults, each of the spline coefficients was allowed to vary across countries and was modelled hierarchically, and was further allowed to vary across time, in order to reflect different trends in prevalence across age groups. We modelled spline coefficients consistently with previous analysis,[20] with the $k^{\text{th}}$ age term coefficients for study $i$ given as follows:

$$\gamma_{k,i} = \psi_k^g + \psi_{k,j[i]}^c + \psi_{k,l[i]}^r + \psi_{k,m[i]}^s + \left(\phi_k^g + \phi_{k,j[i]}^c + \phi_{k,l[i]}^r + \phi_{k,m[i]}^s\right) t[i],$$

$$\psi_{k,j[i]}^c \sim N\left(0, \sigma_{\psi,k,c}^2\right),$$

$$\psi_{k,l[i]}^r \sim N\left(0, \sigma_{\psi,k,r}^2\right),$$

$$\psi_{k,m[i]}^s \sim N\left(0, \sigma_{\psi,k,s}^2\right),$$

$$\phi_{k,j[i]}^c \sim N\left(0, \sigma_{\phi,k,c}^2\right),$$

$$\phi_{k,l[i]}^r \sim N\left(0, \sigma_{\phi,k,r}^2\right),$$

$$\phi_{k,m[i]}^s \sim N\left(0, \sigma_{\phi,k,s}^2\right).$$

Here $\psi^g$, $\psi^c$, $\psi^r$, and $\psi^s$ are global, country, region, and super-region intercepts, and $\phi^g$, $\phi^c$, $\phi^r$, and $\phi^s$ are global, country, region and super-region time slope parameters. A flat improper prior was placed on each of the $\sigma_\psi$'s and $\sigma_\phi$'s.

For children and adolescents, use of the model comparison criteria WAIC showed that the age-time interaction terms, $\phi$, did not improve model fit. Therefore, each of the spline coefficients was still allowed to vary across countries and was modelled hierarchically but was held constant over time. The $k^{\text{th}}$ age term coefficients for study $i$ were given as follows:

$$\gamma_{k,i} = \psi_k^g + \psi_{k,j[i]}^c + \psi_{k,l[i]}^r + \psi_{k,m[i]}^s,$$

$$\psi_{k,j[i]}^c \sim N\left(0, \sigma_{\psi,k,c}^2\right),$$

$$\psi_{k,l[i]}^r \sim N\left(0, \sigma_{\psi,k,r}^2\right),$$

$$\psi_{k,m[i]}^s \sim N\left(0, \sigma_{\psi,k,s}^2\right),$$

with flat improper priors placed on each of the $\sigma_\psi$'s.

*Study-level terms and study-specific random effects*

The prevalence of a BMI category as measured in individual studies may vary from the true unobserved country-year prevalence due to study implementation factors such as those associated with sampling, participation and response, and measurement. We included time-varying offsets (referred to above as fixed effects) to help account for potential systematic differences associated with data sources that are representative of subnational or community populations, and data sources that are representative of urban-only or rural-only populations, through the terms in $\boldsymbol{X_i\beta}$:

$$\boldsymbol{X_i\beta} = \beta_1 \mathbf{I}\{\boldsymbol{X}_i^{cvrg} = subnational\} + \beta_2 \mathbf{I}\{\boldsymbol{X}_i^{cvrg} = subnational\} t[i]$$

$$+ \beta_3 \mathbf{I}\{\boldsymbol{X}_i^{cvrg} = community\} + \beta_4 \mathbf{I}\{\boldsymbol{X}_i^{cvrg} = community\} t[i]$$

$$+ \beta_5 \boldsymbol{X}_{j[i],t[i]}^{c.urb} \mathbf{I}\{\boldsymbol{X}_i^{s.urb} = rural\} + \beta_6 \mathrm{X}_{\mathrm{j[i]},t[i]}^{\mathrm{c.urb}} \mathbf{I}\{\boldsymbol{X}_i^{s.urb} = rural\} t[i]$$

$$+ \beta_7 (1 - \boldsymbol{X}_{j[i],t[i]}^{c.urb}) \mathbf{I}\{\boldsymbol{X}_i^{s.urb} = urban\} + \beta_8 (1 - \boldsymbol{X}_{j[i],t[i]}^{c.urb}) \mathbf{I}\{\boldsymbol{X}_i^{s.urb} = urban\} t[i],$$

where $\boldsymbol{X}_i^{cvrg}$ is the indicator for whether the coverage of study $i$, in country $j$ and year $t$, is subnational or community, $\boldsymbol{X}_i^{s.urb}$ is the indicator for whether the study $i$ covered rural-only or urban-only populations, and $\boldsymbol{X}_{j[i],t[i]}^{c.urb}$ is the percentage of the national population of country $j$ in year $t$ living in urban areas, as obtained from the 2018 revision to the United Nation's World Urbanization Prospects.[30] We note that $\beta_5$ through $\beta_8$ are all multiplied by zero for studies which are urban-only in countries where all residents lived in urban areas (e.g., Singapore) and for studies which are rural-only in countries where all residents lived in rural areas (e.g., Tokelau), i.e., in such cases the model does not consider studies classified as urban (respectively rural) to have potential systematic differences from the true underlying prevalence in the country.

Even after accounting for sampling variability, national studies may still not reflect the true prevalence of a BMI category in a country with perfect accuracy, and subnational and community studies have even larger variability. We include the study-specific random effect $e_i$ to allow all age groups from the same study to have an unusually high or an unusually low prevalence, after conditioning on the other terms in the model. Each $e_i$ is assigned a Gaussian

prior with variance dependent on whether study $i$ is representative at the national, subnational or community level. Random effects from national studies were constrained to have smaller variance ($v_n$) than random effects of subnational studies ($v_s$), which were in turn constrained to have smaller variance than community studies ($v_c$).

*Residual age-by-study variability*

The age patterns across communities within a given country may differ from the overall age pattern of that country. This within-study variability cannot be captured by the $e_i$ terms, which are equal across age-specific observations in each study, so we included an additional variance component for each study, $\tau^2$.

*Model implementation*

The model was fitted through a bespoke MCMC sampler coded in R, which uses a combination of Metropolis-Hastings and Gibbs updates.[31] To generate starting values for the model runs we ran an initial set of eight MCMC chains. We generated the starting values of each initial chain by first randomly generating log variance parameter values from diffuse Gaussian distributions centred on estimates from previous analyses, and then generating all other starting values conditional on these variance parameters. We ran each of the initial chains for 50,000 iterations after burn-in, thinned, and combined across chains to obtain 5000 posterior draws. In order to estimate a distribution from which to sample initial values for the final model runs, we fitted a multivariate Gaussian distribution to the posterior distribution of all non-study-specific parameters obtained from the initial chains, scaling the variance-covariance matrix by a factor of 1.5; this equates to an increase in the variance of the multivariate Gaussian distribution of approximately 50% relative to the target posterior distribution. This is a conservative overdispersion compared to that of 10%, which is considered sufficient for the Rhat convergence diagnostic.[32] To obtain initial values for study-specific parameters, we first sampled a study-specific random effect $e_i$, for each study $i$, from

a Gaussian distribution with mean zero and variance given by the sampled initial values of $v_n$, $v_s$, or $v_c$, dependent on whether study $i$ was representative at national, subnational or community level. We then sampled initial values of the latent variable $\alpha_{h,i}$ for each age group $h$ and study $i$ from its Gaussian distribution, conditional on all other sampled parameter values, including the study-specific random effect $e_i$.

We had a target of eight converged MCMC chains for generating our estimates, which is twice the recommended minimum number to assess convergence using the Rhat diagnostic.[29,33] The exact numbers of chains used for the model runs are not critical so long as at least four chains are run in order to estimate between chain variation, which is necessary for the Rhat convergence diagnostic,[33] and so long as there are sufficient computational resources to run chains to convergence and subsequently to collect samples. We ran ten chains for each BMI category sex combination, with chains ordered by their seeds. The additional two chains were run to allow for a small number of the first eight chains to be discarded if mixing was slow. In practice, only one chain, for the prevalence of BMI -1SD to 1SD in girls, was slow to converge and was replaced. We did not run more chains because the computational and time cost outweighed the gains, if any, in results. We identified, through visual inspection of hyperparameter trace plots, a burn-in period of 20,000 iterations for adult prevalence categories, and 30,000 for child and adolescent categories. We took 50,000 post-burn-in iterations from each of the eight target chains, and combined and thinned to obtain a final sample of 5000 posterior draws for each outcome.

Convergence was confirmed through visual inspection as well as through calculated split-Rhat diagnostic for country-year-age outcomes as implemented in the R package 'rstan' v2.26.15.[33,34] The 97.5$^{th}$ quantile of split-Rhat ranged across BMI categories and sexes from 1.002 to 1.014 for adults, and from 1.005 to 1.019 for children and adolescents. Over 99.97%

of country-year-age outcomes across all categories and sexes for adults and for children and adolescents had split-Rhat < 1.05.

*Inference and post-processing*

All inference was done for country-year-age combinations, through combining the $a$, $b$, $u$, and $\gamma$ terms, and setting $\beta = e_i = 0$. We set $\beta = 0$ as fixed effects associated with study design are not relevant for country-level inference. We set $e_i = 0$ as random effects arising from imperfections and variations in study design and implementation, and from within-country variability of the prevalence of a BMI category, are also not relevant for country-level inference.

As in previous work,[1] we re-scaled the estimated prevalence of different categories so that their sum was 1 in each sex, age, country, and year. The average scaling factors across samples ranged from 0.99 to 1.02 for children and adolescents and 0.99 to 1.03 for adults, i.e., the sum of the separately estimated prevalence categories was close to 1. We calculated the prevalence of double burden at the posterior draw level, as the sum of the prevalence of underweight/thinness and obesity. We also calculated the proportion of the combined burden which was composed of obesity at the draw level.

Posterior estimates were made in one-year age groups for ages 5-19 years, because BMI changes rapidly in relation to age in these ages, and in five-year age groups for those aged 20 years and older. For presentation, we summarised results for ages 5-19 years for children and adolescents, and for ages 20 years and older for adults, as age-standardised results. Age-standardisation puts the population for each country-year on the same (standard) age distribution, and hence enables comparisons to be made over time and across countries. Age-standardisation was performed by taking the weighted means of age-sex-specific estimates, separately for children and adolescents and for adults, using age weights from the WHO standard population.[35] Estimates for regions and the world were calculated as population-weighted averages of the constituent country estimates by sex and age group. The number of

adults who were affected by underweight, the number of children and adolescents who were in the thinness category, and the number of adults or adolescents with obesity were calculated by multiplying the corresponding age-specific prevalence by the age-specific population by sex, country, and year.

Throughout results, we report Pearson correlation coefficients among specific quantities of interest as a measure of their association, with correlation coefficients and their credible intervals calculated at the posterior draw level.

The uncertainties of our estimates, represented by their posterior distributions, include the following sources: uncertainty due to sampling in each data source; uncertainty associated with the variability of national data beyond what is accounted for by sampling; uncertainty associated with subnational and community data, which are more variable than national data; and uncertainty due to making estimates by country, year, and age when data were missing, scarce or weakly informative. The reported credible intervals (CrI) represent the $2.5^{th}$ to $97.5^{th}$ percentiles of the posterior distributions, which contain the true estimates with 95% probability. We obtained the posterior probability (PP) that an estimated change in underweight/thinness or in obesity represented a true increase as the proportion of draws from the posterior distribution that indicated an increase, i.e., a positive change. We obtained the PP that obesity was more prevalent than underweight/thinness as the proportion of posterior draws for which obesity prevalence was greater than underweight/thinness prevalence. Similarly, we obtained the PP that underweight/thinness was more prevalent as the proportion of posterior draws for which underweight/thinness prevalence was greater than obesity prevalence.

**Appendix Text 4.** Comparison with previous studies

To our knowledge, there are no previous reports of the combined prevalence of underweight and obesity for all countries in the world, and the latest data on the individual outcomes are from 2016.[1] The estimated prevalence for both underweight or thinness and obesity for 2016 from this study were correlated with the estimates in the earlier work, but there were differences in some countries and years, because the current estimates benefitted from 48% more data. The additional six years of data covered a period of significant shift in both the character of double burden, i.e., towards obesity dominance, and in its geographical epicentre, which is increasingly in middle-income nations. A report on double burden of malnutrition[36] used a mix of indicators (stunting, thinness and overweight) and age groups and hence is not directly comparable to our results; nonetheless, the study reached similar conclusions about the role of obesity in increasing double burden of malnutrition, especially in low- and middle-income countries. A few studies used data from Demographic Health Surveys or Global School-Based Health Surveys to report double burden of malnutrition in low- and middle-income countries, but they were restricted to women of reproductive age or school-aged children and adolescents.[37-44] Only two studies[37,38] reported change over time, but these studies did not use data after 2012 as we did; their findings were consistent with ours about obesity making up an increasing share of the overall burden of malnutrition in low- and middle-income countries. Two additional studies reviewed published data and reported double burden of malnutrition in multiple world regions.[45,46] They did not provide country-level results, and hence are not comparable with our results. Our results are consistent with trends in underweight or obesity reported in specific countries. In particular, the decline in female obesity in Spain and France, the only two countries where we estimated a decline in obesity in adults, has been noted in some age groups in other studies that used measurement data.[47-49] Spain and France also have self-reported data on height and weight which are subject to bias and hence differ from measurement surveys[50,51] used for our estimates.

**Appendix Table 1.** Data sources used in the analysis.

| | Country | Study years | Survey/Study name/Citation | Level of representative-ness | Rural, urban, or both | Age range as in NCD-RisC database | | Sample size | | Notes |
|---|---|---|---|---|---|---|---|---|---|---|
| | | | | | | Male | Female | Male | Female | |
| 1 | Afghanistan | 2013 | National Nutrition Survey | National | both | | 10-49 | | 18,433 | |
| 2 | Afghanistan | 2018 | STEPS | National | both | 18-69 | 18-69 | 1,984 | 1,703 | |
| 3 | Albania | 2001 | Shapo et al., Public Health Nutr 6:471-77, 2003 | Community | urban | 24+ | 24+ | 535 | 585 | |
| 4 | Albania | 2008-2009 | DHS | National | both | 15-49 | 15-49 | 2,978 | 7,386 | |
| 5 | Albania | 2013 | Childhood Obesity Surveillance Initiative 3 | National | both | 7-9 | 7-9 | 2,971 | 2,794 | |
| 6 | Albania | 2013-2015 | Balkan Survey of Inactivity in Children (BASIC) | National | both | 6-16 | 6-16 | 4,978 | 4,972 | |
| 7 | Albania | 2015-2016 | Childhood Obesity Surveillance Initiative 4 | National | both | 7-9 | 7-9 | 3,358 | 3,062 | |
| 8 | Albania | 2017-2018 | DHS | National | both | 15-59 | 15-59 | 5,953 | 14,447 | |
| 9 | Algeria | 2003 | STEPS | Subnational | both | 25-64 | 25-64 | 1,612 | 2,437 | |
| 10 | Algeria | 2005 | Transition and Health Impact in North Africa | National | both | 35-70 | 35-70 | 2,004 | 2,741 | |
| 11 | Algeria | 2007-2009 | The ISOR (InSulino-resistance in ORan) Study | Community | urban | 30-64 | 30-64 | 376 | 408 | |
| 12 | Algeria | 2016-2017 | STEPS | National | both | 18-69 | 18-69 | 2,991 | 3,636 | |
| 13 | American Samoa | 1976-1978 | McGarvey, Am J Clin Nutr 53(6 Suppl):1586S-1594S, 1991 | National | both | 5+ | 5+ | 1,017 | 1,329 | 1 |
| 14 | American Samoa | 1990 | McGarvey, Pac Health Dialog 8(1):157-62, 2001 | National | both | 25+ | 25+ | 359 | 484 | |
| 15 | American Samoa | 1992 | McGarvey, Pac Health Dialog 8(1):157-62, 2001 | National | both | 27+ | 27+ | 232 | 337 | |
| 16 | American Samoa | 1994 | McGarvey, Pac Health Dialog 8(1):157-62, 2001 | National | both | 29+ | 29+ | 165 | 245 | |
| 17 | American Samoa | 2004 | STEPS | National | both | 25-64 | 25-64 | 949 | 1,060 | |
| 18 | Andorra | 2004-2005 | Enquesta Nutricional D'Andora | National | both | 18-75 | 18-75 | 400 | 447 | |
| 19 | Angola | 2013-2014 | CardioBengo - Population based cardiovascular longitudinal study in Bengo Province, Angola | Community | both | 14-65 | 14-65 | 875 | 1,486 | |
| 20 | Antigua and Barbuda | 2009 | Global School-based Student Health Survey | National | both | 13-17 | 13-17 | 70 | 122 | |
| 21 | Argentina | 1981-1985 | Hernandez et al., Diabetes Res Clin Pract 3:277-83, 1987 | Community | urban | 20-74 | 20-74 | 395 | 414 | |
| 22 | Argentina | 1985-1986 | INTERSALT | Community | urban | 20-59 | 20-59 | 100 | 100 | |
| 23 | Argentina | 1995-1998 | de Sereday et al., Diabetes Metab 30:335-9, 2004 | Subnational | urban | 15-74 | 15-74 | 924 | 1,246 | |
| 24 | Argentina | 2003 | CEDES-Programa VIGI+A-Banco Mundial, 2004 | Community | urban | 15-74 | 15-74 | 151 | 176 | |
| 25 | Argentina | 2004-2005 | CArdiovascular Risk factors Multiple Evaluation in Latin America (CARMELA) | Community | urban | 25-64 | 25-64 | 733 | 742 | |
| 26 | Argentina | 2005 | Encuesta Nacional de Nutrición y Salud 2005 | National | both | | 10-49 | | 6,581 | |
| 27 | Argentina | 2006 | Virasoro Survey | Community | urban | 15-84 | 15-84 | 261 | 306 | |
| 28 | Argentina | 2008-2011 | The VELA Project | Community | rural | 5+ | 5+ | 380 | 543 | |
| 29 | Argentina | 2011 | Primera Encuesta Alimentaria y Nutricional de la Ciudad Autónoma de Buenos Aires - EANCABA | Community | urban | 5-18; 60+ | 5-49; 60+ | 1,173 | 2,229 | |
| 30 | Argentina | 2011-2012 | CESCAS Study | Community | urban | 35-74 | 35-74 | 1,571 | 2,359 | |
| 31 | Argentina | 2012 | Global School-based Student Health Survey | National | both | 13-17 | 13-17 | 8,333 | 8,904 | |
| 32 | Argentina | 2012-2013 | Primer estudio sobre el estado nutricional y los hábitos alimentarios de la población adulta de Rosario | Community | urban | 18-70 | 18-70 | 371 | 823 | |
| 33 | Argentina | 2014-2015 | Latin American Study of Nutrition and Health (ELANS) | National | urban | 15-65 | 15-65 | 573 | 693 | |
| 34 | Argentina | 2018 | Encuesta Nacional de Factores de Riesgo 2018 | National | both | 18+ | 18+ | 6,960 | 9,449 | |
| 35 | Argentina | 2018-2019 | Encuesta Nacional de Nutricion y Salud | National | urban | 5+ | 5+ | 6,482 | 7,332 | |
| 36 | Armenia | 1998 | The health and nutritional status of children and women in Armenia | National | both | | 18-45 | | 2,420 | |
| 37 | Armenia | 2000 | DHS | National | both | | 15-49 | | 5,982 | |
| 38 | Armenia | 2005 | DHS | National | both | 15-49 | 15-49 | 1,160 | 6,123 | |
| 39 | Armenia | 2015-2016 | DHS | National | both | | 15-49 | | 5,731 | |
| 40 | Armenia | 2016 | STEPS | National | both | 18-69 | 18-69 | 604 | 1,447 | |
| 41 | Armenia | 2019 | Childhood Obesity Surveillance Initiative 5 | National | both | 7-8 | 7-8 | 1,860 | 1,703 | |
| 42 | Australia | 1980 | Risk Factor Prevalence Study | National | urban | 25-64 | 25-64 | 2,756 | 2,781 | |
| 43 | Australia | 1981 | Busselton Health Study | Community | urban | 18+ | 18+ | 225 | 290 | |
| 44 | Australia | 1983 | MONICA, Newcastle | Subnational | urban | 35-64 | 35-64 | 1,215 | 1,244 | |
| 45 | Australia | 1983 | Risk Factor Prevalence Study | National | urban | 25-64 | 25-64 | 3,731 | 3,813 | |
| 46 | Australia | 1985 | Australian Schools Health and Fitness Survey (ASHFS) | National | both | 7-15 | 7-15 | 4,302 | 4,189 | |
| 47 | Australia | 1988-1989 | Dubbo Study of Australian Elderly | Community | urban | 59+ | 59+ | 877 | 1,219 | |
| 48 | Australia | 1988-1989 | MONICA, Newcastle | Subnational | urban | 35-64 | 35-64 | 672 | 671 | |
| 49 | Australia | 1988-1989 | MONICA, Newcastle | Community | urban | 25-34 | 25-34 | 70 | 84 | |
| 50 | Australia | 1989 | Risk Factor Prevalence Study | National | urban | 20-69 | 20-69 | 4,497 | 4,678 | |
| 51 | Australia | 1992-1993 | Australia Longitudinal Study of Ageing | Community | urban | 65+ | 65+ | 814 | 746 | |
| 52 | Australia | 1994 | MONICA, Newcastle | Subnational | urban | 35-64 | 35-64 | 637 | 688 | |
| 53 | Australia | 1994 | MONICA, Perth inner | Community | urban | 25-64 | 25-64 | 363 | 349 | |
| 54 | Australia | 1994 | MONICA, Perth outer | Community | urban | 25-64 | 25-64 | 373 | 387 | |
| 55 | Australia | 1995 | National Nutrition Survey 1995 | National | both | 5+ | 5+ | 5,983 | 6,390 | |
| 56 | Australia | 1996 | The Nepean Longitudinal Cohort Study | Community | urban | 7-8 | 7-8 | 221 | 215 | |
| 57 | Australia | 1996-1998 | Western Australian AAA Screening Program | Community | urban | 65-84 | | 12,194 | | |

| | Country | Study years | Survey/Study name/Citation | Level of representative-ness | Rural, urban, or both | Age range as in NCD-RisC database | | Sample size | | Notes |
|---|---|---|---|---|---|---|---|---|---|---|
| | | | | | | Male | Female | Male | Female | |
| 58 | Australia | 1997 | South Australia Schools Fitness and Physical Activity Survey | Subnational | both | 10-12 | 10-12 | 921 | 765 | |
| 59 | Australia | 1999-2000 | The Australian Diabetes, Obesity and Lifestyle Study 1999-2000 | National | both | 25+ | 25+ | 4,991 | 6,070 | |
| 60 | Australia | 1999-2003 | North West Adelaide Health Study | Community | urban | 18+ | 18+ | 1,932 | 2,122 | |
| 61 | Australia | 2000 | Perth children | Community | both | 25 | 25 | 266 | 334 | |
| 62 | Australia | 2004 | The Longitudinal Study of Australian Children, K cohort (child) | National | both | 5 | 5 | 425 | 431 | |
| 63 | Australia | 2004 | The Nepean Longitudinal Cohort Study | Community | urban | 14-15 | 14-15 | 143 | 149 | |
| 64 | Australia | 2004-2005 | Janus et al., Med J Aust 187:147-52, 2007 | Community | rural | 25-74 | 25-74 | 383 | 422 | |
| 65 | Australia | 2004-2005 | The Australian Diabetes, Obesity and Lifestyle Study 2004-2005 | National | both | 30+ | 30+ | 2,874 | 3,472 | |
| 66 | Australia | 2004-2006 | North West Adelaide Health Study | Community | urban | 20+ | 20+ | 1,523 | 1,679 | |
| 67 | Australia | 2006 | The Longitudinal Study of Australian Children, K cohort (child) | National | both | 6-7 | 6-7 | 2,245 | 2,156 | |
| 68 | Australia | 2007 | Children's Nutrition and Physical Activity Survey | National | both | 5-16 | 5-16 | 1,649 | 1,669 | |
| 69 | Australia | 2007-2008 | National Health Survey | National | both | 18+ | 18+ | 5,279 | 5,655 | |
| 70 | Australia | 2008 | The Longitudinal Study of Australian Children, B cohort (infant) | National | both | 5 | 5 | 508 | 513 | |
| 71 | Australia | 2008 | The Longitudinal Study of Australian Children, K cohort (child) | National | both | 8-9 | 8-9 | 2,121 | 2,023 | |
| 72 | Australia | 2008-2010 | North West Adelaide Health Study | Community | urban | 24+ | 24+ | 1,168 | 1,318 | |
| 73 | Australia | 2010 | The Longitudinal Study of Australian Children, B cohort (infant) | National | both | 6-7 | 6-7 | 2,148 | 2,014 | |
| 74 | Australia | 2010 | The Longitudinal Study of Australian Children, K cohort (child) | National | both | 10-11 | 10-11 | 2,052 | 1,945 | |
| 75 | Australia | 2010 | NSW School physical activity and nutrition survey | Subnational | both | 5-16 | 5-16 | 4,076 | 3,764 | |
| 76 | Australia | 2011-2013 | Australian Health Survey | National | both | 5+ | 5+ | 12,190 | 13,011 | 2 |
| 77 | Australia | 2011-2013 | International Study of Childhood Obesity, Lifestyle and the Environment (ISCOLE) | Community | urban | 9-11 | 9-11 | 243 | 285 | |
| 78 | Australia | 2012 | The Australian Diabetes, Obesity and Lifestyle Study 2012 | National | both | 37+ | 37+ | 2,048 | 2,530 | |
| 79 | Australia | 2012 | The Longitudinal Study of Australian Children, B cohort (infant) | National | both | 8-9 | 8-9 | 2,041 | 1,947 | |
| 80 | Australia | 2012 | The Longitudinal Study of Australian Children, K cohort (child) | National | both | 12-13 | 12-13 | 1,948 | 1,851 | |
| 81 | Australia | 2014 | The Longitudinal Study of Australian Children, B cohort (infant) | National | both | 10-11 | 10-11 | 1,829 | 1,742 | |
| 82 | Australia | 2014 | The Longitudinal Study of Australian Children, K cohort (child) | National | both | 14-15 | 14-15 | 1,694 | 1,581 | |
| 83 | Australia | 2014-2015 | National Health Survey | National | both | 5+ | 5+ | 8,331 | 9,424 | |
| 84 | Australia | 2015 | NSW School physical activity and nutrition survey | Subnational | both | 5-16 | 5-16 | 3,571 | 3,791 | |
| 85 | Australia | 2016 | The Longitudinal Study of Australian Children, B cohort (infant) | National | both | 12-13 | 12-13 | 1,639 | 1,529 | |
| 86 | Australia | 2016 | The Longitudinal Study of Australian Children, K cohort (child) | National | both | 16-17 | 16-17 | 1,477 | 1,380 | |
| 87 | Australia | 2017-2018 | National Health Survey | National | both | 18+ | 18+ | 7,576 | 8,729 | |
| 88 | Australia | 2018 | The Longitudinal Study of Australian Children, B cohort (infant) | National | both | 14-15 | 14-15 | 1,523 | 1,403 | |
| 89 | Australia | 2018 | The Longitudinal Study of Australian Children, K cohort (child) | National | both | 18-19 | 18-19 | 1,304 | 1,233 | |
| 90 | Austria | 1983 | The Austrian Conscription Database | National | both | 17-18 | | 58,517 | | |
| 91 | Austria | 1984 | The Austrian Conscription Database | National | both | 17-18 | | 58,830 | | |
| 92 | Austria | 1985 | The Austrian Conscription Database | National | both | 17-18 | | 58,638 | | |
| 93 | Austria | 1986 | CINDI | Community | both | 25-64 | 25-64 | 657 | 715 | |
| 94 | Austria | 1986 | The Austrian Conscription Database | National | both | 17-18 | | 57,870 | | |
| 95 | Austria | 1987 | The Austrian Conscription Database | National | both | 17-18 | | 55,292 | | |
| 96 | Austria | 1988 | The Austrian Conscription Database | National | both | 17-18 | | 51,050 | | |
| 97 | Austria | 1989 | The Austrian Conscription Database | National | both | 17-18 | | 49,380 | | |
| 98 | Austria | 1990 | The Austrian Conscription Database | National | both | 17-18 | | 47,102 | | |
| 99 | Austria | 1991 | CINDI survey Vorarlberg/Austria | Subnational | both | 25-64 | 25-64 | 698 | 738 | |
| 100 | Austria | 1991 | The Austrian Conscription Database | National | both | 17-18 | | 44,288 | | |
| 101 | Austria | 1992 | The Austrian Conscription Database | National | both | 17-18 | | 43,553 | | |
| 102 | Austria | 1992 | Vorarlberg Health Monitoring and Promotion Programme (VHM&PP) | Subnational | both | 18+ | 18+ | 14,161 | 18,835 | |
| 103 | Austria | 1993 | The Austrian Conscription Database | National | both | 17-18 | | 42,356 | | |
| 104 | Austria | 1994 | The Austrian Conscription Database | National | both | 17-18 | | 40,401 | | |
| 105 | Austria | 1995 | The Austrian Conscription Database | National | both | 17-18 | | 39,809 | | |
| 106 | Austria | 1996 | The Austrian Conscription Database | National | both | 17-18 | | 39,587 | | |
| 107 | Austria | 1997 | The Austrian Conscription Database | National | both | 17-18 | | 40,408 | | |
| 108 | Austria | 1998 | The Austrian Conscription Database | National | both | 17-18 | | 43,131 | | |
| 109 | Austria | 1998 | Vorarlberg Health Monitoring and Promotion Programme (VHM&PP) | Subnational | both | 18+ | 18+ | 16,153 | 20,915 | |
| 110 | Austria | 1998-1999 | CINDI survey Vorarlberg/Austria | Subnational | both | 25-64 | 25-64 | 409 | 414 | |
| 111 | Austria | 1999 | The Austrian Conscription Database | National | both | 17-18 | | 44,163 | | |
| 112 | Austria | 2000 | The Austrian Conscription Database | National | both | 17-18 | | 44,275 | | |
| 113 | Austria | 2001 | The Austrian Conscription Database | National | both | 17-18 | | 43,315 | | |
| 114 | Austria | 2002 | The Austrian Conscription Database | National | both | 17-18 | | 42,899 | | |

| | Country | Study years | Survey/Study name/Citation | Level of representative-ness | Rural, urban, or both | Age range as in NCD-RisC database | | Sample size | | Notes |
|---|---|---|---|---|---|---|---|---|---|---|
| | | | | | | Male | Female | Male | Female | |
| 115 | Austria | 2003 | The Austrian Conscription Database | National | both | 17-18 | | 42,389 | | |
| 116 | Austria | 2004 | The Austrian Conscription Database | National | both | 17-18 | | 42,774 | | |
| 117 | Austria | 2004 | Vorarlberg Health Monitoring and Promotion Programme (VHM&PP) | Subnational | both | 18+ | 18+ | 20,160 | 23,893 | |
| 118 | Austria | 2004-2005 | Vorarlberg Health Monitoring and Promotion Programme (VHM&PP) | Subnational | both | 6-17 | 6-17 | 17,504 | 15,823 | |
| 119 | Austria | 2005 | The Austrian Conscription Database | National | both | 17-18 | | 42,906 | | |
| 120 | Austria | 2006 | The Austrian Conscription Database | National | both | 17-18 | | 44,571 | | |
| 121 | Austria | 2006-2007 | HELENA | Community | urban | 12-17 | 12-17 | 191 | 211 | |
| 122 | Austria | 2007 | The Austrian Conscription Database | National | both | 17-18 | | 44,755 | | |
| 123 | Austria | 2008 | The Austrian Conscription Database | National | both | 17-18 | | 44,880 | | |
| 124 | Austria | 2008-2009 | Vorarlberg Health Monitoring and Promotion Programme (VHM&PP) | Subnational | both | 6-17 | 6-17 | 16,847 | 15,295 | |
| 125 | Austria | 2009 | The Austrian Conscription Database | National | both | 17-18 | | 45,594 | | |
| 126 | Austria | 2009-2011 | Mayer et al., Ann Hum Biol 42(1):45-55, 2015 | National | both | 5-17 | 5-17 | 6,634 | 6,340 | |
| 127 | Austria | 2010 | The Austrian Conscription Database | National | both | 17-18 | | 44,968 | | |
| 128 | Austria | 2010-2012 | Austrian Study on Nutritional Status 2012 | National | both | 6-80 | 6-80 | 363 | 446 | |
| 129 | Austria | 2011 | The Austrian Conscription Database | National | both | 17-18 | | 44,076 | | |
| 130 | Austria | 2011-2012 | BMI in Upper Austrian children and adolescents | Subnational | both | 6-17 | 6-17 | 7,808 | 6,853 | |
| 131 | Austria | 2012 | The Austrian Conscription Database | National | both | 17-18 | | 42,768 | | |
| 132 | Austria | 2012-2013 | Vorarlberg Health Monitoring and Promotion Programme (VHM&PP) | Subnational | both | 6-17 | 6-17 | 14,760 | 13,648 | |
| 133 | Austria | 2013 | The Austrian Conscription Database | National | both | 17-18 | | 41,574 | | |
| 134 | Austria | 2013-2014 | Prevalence of obesity and motor performance in Tyrolean preschool children | Subnational | both | 5 | 5 | 550 | 513 | |
| 135 | Austria | 2013-2017 | Austrian Study on Nutrition (ASN) | National | both | 19-64 | 19-64 | 774 | 1,332 | |
| 136 | Austria | 2014 | The Austrian Conscription Database | National | both | 17-18 | | 41,740 | | |
| 137 | Austria | 2014-2015 | Influence of selected risk factors on the motor performance of 10 to 11-year-old schoolchildren | Subnational | both | 10-11 | 10-11 | 197 | 129 | |
| 138 | Austria | 2015 | The Austrian Conscription Database | National | both | 17-18 | | 39,154 | | |
| 139 | Austria | 2015-2016 | Childhood Obesity Surveillance Initiative 4 | National | both | 8-9 | 8-9 | 1,220 | 1,175 | |
| 140 | Austria | 2015-2016 | Vorarlberg Health Monitoring and Promotion Programme (VHM&PP) | Subnational | both | 6-17 | 6-17 | 13,037 | 12,048 | |
| 141 | Austria | 2015-2017 | The Tyrolean Early Vascular Ageing-study (EVA-Tyrol) - East-Tyrol | Subnational | both | 14-17 | 14-17 | 680 | 831 | |
| 142 | Austria | 2016 | The Austrian Conscription Database | National | both | 17-19 | | 35,944 | | |
| 143 | Austria | 2017 | The Austrian Conscription Database | National | both | 17-19 | | 34,399 | | |
| 144 | Austria | 2018 | The Austrian Conscription Database | National | both | 17-19 | | 34,188 | | |
| 145 | Austria | 2019 | Fitness and health status of primary school children - before and during the COVID-19 pandemic | Community | both | 7-10 | 7-10 | 381 | 382 | |
| 146 | Austria | 2019 | The Austrian Conscription Database | National | both | 17-19 | | 32,185 | | |
| 147 | Austria | 2019 | Childhood Obesity Surveillance Initiative 5 | National | both | 8-9 | 8-9 | 1,223 | 1,137 | |
| 148 | Austria | 2020 | Fitness and health status of primary school children - before and during the COVID-19 pandemic - June | Community | both | 7-10 | 7-10 | 381 | 383 | |
| 149 | Austria | 2020 | Fitness and health status of primary school children - before and during the COVID-19 pandemic - September | Community | both | 8-11 | 8-11 | 380 | 382 | |
| 150 | Austria | 2021 | Fitness and health status of primary school children - before and during the COVID-19 pandemic | Community | both | 8-11 | 8-11 | 374 | 372 | |
| 151 | Austria | 2021-2023 | Early Vascular Ageing in the YOUth (EVA4YOU) | Community | both | 14-17 | 14-17 | 373 | 665 | |
| 152 | Azerbaijan | 1996 | Health and Nutrition Survey | National | both | 19-59 | 19-59 | 121 | 295 | |
| 153 | Azerbaijan | 2001 | Reproductive Health Survey | National | both | | 15-44 | | 1,726 | |
| 154 | Azerbaijan | 2006 | DHS | National | both | 15-59 | 15-49 | 2,388 | 7,868 | |
| 155 | Azerbaijan | 2013 | Azerbaijan Nutrition Survey | National | both | | 15-49 | | 2,839 | |
| 156 | Azerbaijan | 2017 | STEPS | National | both | 18-69 | 18-69 | 1,117 | 1,577 | |
| 157 | Bahamas | 2011-2012 | STEPS | National | both | 25-64 | 25-64 | 586 | 938 | |
| 158 | Bahamas | 2013 | Global School-based Student Health Survey | National | both | 13-17 | 13-17 | 460 | 533 | |
| 159 | Bahamas | 2019 | STEPS | National | both | 18-69 | 18-69 | 862 | 1,317 | |
| 160 | Bahrain | 1991-1992 | Al-Mannai et al., J R Soc Health 116:30-2, 7-40, 1996 | Community | both | 20+ | 20+ | 137 | 153 | |
| 161 | Bahrain | 1995 | Musaiger et al., Ann Hum Biol 28:346-50, 2001 | Community | both | 30+ | 30+ | 298 | 216 | |
| 162 | Bahrain | 1998-1999 | National Nutrition Survey | National | both | 19+ | 19+ | 1,120 | 1,181 | |
| 163 | Bahrain | 2001-2004 | Global database on growth and malnutrition of school children and adolescents, WHO | National | both | 6-19 | 6-20 | 1,268 | 1,326 | |
| 164 | Bahrain | 2007 | STEPS | National | both | 20-64 | 20-64 | 854 | 858 | |
| 165 | Bahrain | 2016 | Global School-based Student Health Survey | National | both | 12-17 | 12-17 | 3,416 | 3,262 | |
| 166 | Bangladesh | 1992 | Rahman et al., Hypertension 33:74-8, 1999 | Community | rural | 30+ | 30+ | 965 | 643 | |
| 167 | Bangladesh | 1996-1997 | DHS | National | both | | 20-49 | | 3,384 | |
| 168 | Bangladesh | 1998 | Zaman et al., J Health Popul Nutr 21:162-63, 2003 | Community | rural | 20+ | 20+ | 290 | 379 | |
| 169 | Bangladesh | 1999-2000 | Hussain et al., Eur J Public Health 17:291-96, 2007 | Community | rural | 20-59 | 20-59 | 2,037 | 2,720 | |
| 170 | Bangladesh | 1999-2000 | DHS | National | both | | 20-49 | | 3,887 | |

| | Country | Study years | Survey/Study name/Citation | Level of representative-ness | Rural, urban, or both | Age range as in NCD-RisC database | | Sample size | | Notes |
|---|---|---|---|---|---|---|---|---|---|---|
| | | | | | | Male | Female | Male | Female | |
| 171 | Bangladesh | 2000-2004 | Nutritional Surveillance Project | National | rural | | 15-45 | | 224,251 | |
| 172 | Bangladesh | 2002 | STEPS | Community | rural | 25-64 | 25-64 | 2,086 | 2,038 | |
| 173 | Bangladesh | 2002 | STEPS | Community | urban | 25-64 | 25-64 | 3,533 | 3,737 | |
| 174 | Bangladesh | 2004 | DHS | National | both | | 20-49 | | 9,165 | |
| 175 | Bangladesh | 2006 | Urban Health Survey | Subnational | urban | 20-59 | 20-59 | 6,109 | 5,898 | |
| 176 | Bangladesh | 2007 | DHS | National | both | | 20-49 | | 9,037 | |
| 177 | Bangladesh | 2009-2010 | STEPS | National | both | 25+ | 25+ | 4,310 | 4,849 | |
| 178 | Bangladesh | 2011 | DHS | National | both | 15+ | 20+ | 5,254 | 16,679 | |
| 179 | Bangladesh | 2011-2012 | Chronic Disease Risk Factor Study | Community | rural | 14+ | 14+ | 327 | 470 | |
| 180 | Bangladesh | 2013 | STEPS | National | both | 25+ | 25+ | 1,812 | 2,261 | |
| 181 | Bangladesh | 2014 | DHS | National | both | | 20-49 | | 14,963 | |
| 182 | Bangladesh | 2015-2016 | An Assessment of BRAC Health Nutrition and Population Programme and Benchmark Survey of Sustainable Development Goal - 2015 | National | rural | 35+ | 11+ | 5,432 | 18,378 | |
| 183 | Bangladesh | 2016 | Diabetes Mellitus: Action through community Groups or Health Information for better Control of population blood glucose, risk factors, knowledge and care seeking (DMagic) | Subnational | rural | 30+ | 30+ | 5,630 | 6,414 | |
| 184 | Bangladesh | 2017-2018 | DHS | National | both | | 15-49 | | 17,073 | |
| 185 | Bangladesh | 2018 | STEPS | National | both | 18-69 | 18-69 | 3,784 | 4,229 | |
| 186 | Bangladesh | 2018-2019 | National Nutrition Surveillence | National | both | 10+ | 10+ | 12,212 | 12,104 | |
| 187 | Barbados | 1987-1992 | Barbados Eye Study | National | both | 40-84 | 40-84 | 1,980 | 2,627 | |
| 188 | Barbados | 1991-1994 | Cooper et al., Am J Public Health 87(2):160-68, 1997 | Community | urban | 25-100 | 25-100 | 329 | 482 | |
| 189 | Barbados | 1997-2002 | The Barbados Incidence Studies of Eye Diseases II | National | both | 40-84 | 40-84 | 1,004 | 1,441 | |
| 190 | Barbados | 1999-2000 | The Survey on Health, Well-Being, and Aging in Latin America and the Caribbean (SABE) | Community | urban | 60+ | 60+ | 559 | 866 | 3 |
| 191 | Barbados | 2011 | Global School-based Student Health Survey | National | both | 13-17 | 13-17 | 627 | 708 | |
| 192 | Barbados | 2011-2013 | Health of the Nation (HotN) | National | both | 25+ | 25+ | 455 | 703 | |
| 193 | Belarus | 2016-2017 | STEPS | National | both | 18-69 | 18-69 | 2,085 | 2,894 | |
| 194 | Belarus | 2020 | STEPS | National | both | 18-69 | 18-69 | 2,271 | 2,978 | |
| 195 | Belgium | 1983-1985 | MONICA, Luxembourg | Community | urban | 35-64 | 35-64 | 944 | 936 | |
| 196 | Belgium | 1984-1985 | Belgian Interuniversity Research on Nutrition and Health | National | both | 25-74 | 25-74 | 5,837 | 5,242 | |
| 197 | Belgium | 1985-1986 | INTERSALT, Ghent | Community | urban | 20-59 | 20-59 | 100 | 100 | |
| 198 | Belgium | 1985-1987 | INTERSALT, Charleroi | Community | urban | 20-59 | 20-59 | 82 | 75 | |
| 199 | Belgium | 1985-1987 | MONICA, Charleroi | Community | urban | 25-64 | 25-64 | 347 | 327 | |
| 200 | Belgium | 1985-1987 | MONICA, Ghent | Community | urban | 25-64 | 25-64 | 549 | 459 | |
| 201 | Belgium | 1985-1990 | Flemish Study on Environment, Genes and Health Outcomes | Community | rural | 20+ | 20+ | 656 | 692 | |
| 202 | Belgium | 1987-1990 | MONICA, Charleroi | Community | urban | 25-64 | 25-64 | 325 | 301 | |
| 203 | Belgium | 1988-1990 | MONICA, Ghent | Community | urban | 25-64 | 25-64 | 456 | 449 | |
| 204 | Belgium | 1990-1992 | MONICA, Ghent | Community | urban | 25-64 | 25-64 | 507 | 475 | |
| 205 | Belgium | 1990-1993 | MONICA, Charleroi | Community | urban | 25-64 | 25-64 | 337 | 332 | |
| 206 | Belgium | 1991-1994 | Flemish Study on Environment, Genes and Health Outcomes | Community | rural | 26+ | 26+ | 393 | 416 | |
| 207 | Belgium | 1992-1995 | Flemish Study on Environment, Genes and Health Outcomes | Community | rural | 27+ | 27+ | 298 | 312 | |
| 208 | Belgium | 1994-1996 | BIRNH Elderly: Belgian Interuniversity Research on Nutrition and Health in the Elderly | National | both | 65-89 | 65-89 | 1,147 | 959 | |
| 209 | Belgium | 1996-1998 | Flemish Study on Environment, Genes and Health Outcomes | Community | rural | 10+ | 10+ | 404 | 403 | |
| 210 | Belgium | 1998 | Flemish Study on Environment, Genes and Health Outcomes | Community | rural | 32+ | 32+ | 320 | 359 | |
| 211 | Belgium | 1998-2000 | Flemish Study on Environment, Genes and Health Outcomes | Community | rural | 10+ | 10+ | 220 | 217 | |
| 212 | Belgium | 1999-2001 | Flemish Study on Environment, Genes and Health Outcomes | Community | rural | 10+ | 10+ | 232 | 254 | |
| 213 | Belgium | 2001 | Flemish Study on Environment, Genes and Health Outcomes | Community | rural | 10+ | 10+ | 242 | 222 | |
| 214 | Belgium | 2002-2003 | Flemish Study on Environment, Genes and Health Outcomes | Community | rural | 10+ | 10+ | 174 | 197 | |
| 215 | Belgium | 2002-2004 | SPAH | Subnational | both | 18-75 | 18-75 | 2,594 | 2,308 | |
| 216 | Belgium | 2002-2005 | Flemish Study on Environment, Genes and Health Outcomes | Community | rural | 10+ | 10+ | 447 | 462 | |
| 217 | Belgium | 2003 | The European Male Ageing Study | Community | both | 40+ | | 433 | | |
| 218 | Belgium | 2005-2008 | Flemish Study on Environment, Genes and Health Outcomes | Community | rural | 10+ | 10+ | 348 | 357 | |
| 219 | Belgium | 2006-2007 | HELENA | Community | urban | 12-17 | 12-17 | 156 | 180 | |
| 220 | Belgium | 2006-2008 | Flemish Study on Environment, Genes and Health Outcomes | Community | rural | 10+ | 10+ | 111 | 110 | |
| 221 | Belgium | 2007-2008 | Childhood Obesity Surveillance Initiative 1 | Subnational | both | 6-9 | 6-9 | 64,322 | 61,754 | |
| 222 | Belgium | 2007-2010 | Identification and prevention of Dietary- and lifestyle-induced health EFfects In Children and infantS (IDEFICS) | Community | urban | 5-9 | 5-9 | 822 | 834 | |
| 223 | Belgium | 2008 | The European Male Ageing Study | Community | both | 40+ | | 383 | | |
| 224 | Belgium | 2009-2010 | Childhood Obesity Surveillance Initiative 2 | Subnational | both | 6-9 | 6-9 | 67,775 | 65,365 | |

| | Country | Study years | Survey/Study name/Citation | Level of representative-ness | Rural, urban, or both | Age range as in NCD-RisC database | | Sample size | | Notes |
|---|---|---|---|---|---|---|---|---|---|---|
| | | | | | | Male | Female | Male | Female | |
| 225 | Belgium | 2009-2013 | Flemish Study on Environment, Genes and Health Outcomes | Community | rural | 20+ | 20+ | 330 | 335 | |
| 226 | Belgium | 2010 | EuropeaN Energy balance Research to prevent excessive weight Gain among Youth - The ENERGY-project | Subnational | urban | 10-12 | 10-12 | 460 | 497 | |
| 227 | Belgium | 2010-2015 | Flemish Study on Environment, Genes and Health Outcomes | Community | rural | 15+ | 15+ | 388 | 410 | |
| 228 | Belgium | 2012-2013 | Childhood Obesity Surveillance Initiative 3 | Subnational | both | 6-9 | 6-9 | 70,441 | 67,874 | |
| 229 | Belgium | 2014-2015 | Food Consumption Survey | National | both | 5-64 | 5-64 | 1,477 | 1,485 | |
| 230 | Belgium | 2018-2019 | Belgian Health Examination Survey | National | both | 18+ | 18+ | 558 | 614 | 4 |
| 231 | Belgium | 2021-2022 | OUTSIDE-IN | Community | both | 11-12 | 11-12 | 212 | 184 | |
| 232 | Belgium | 2022 | OUTSIDE-IN | Community | both | 12-13 | 12-13 | 210 | 172 | |
| 233 | Belgium | 2023 | OUTSIDE-IN, spring | Community | both | 11-13 | 11-13 | 218 | 188 | 5 |
| 234 | Belize | 2005-2006 | CAMDI | National | both | 20+ | 20+ | 599 | 1,018 | |
| 235 | Belize | 2011 | Global School-based Student Health Survey | National | both | 13 | 13 | 163 | 188 | |
| 236 | Belize | 2017 | Survey of Risk Factors for Chronic Kidney Disease (SRFCKD) | National | both | 20-55 | 20-55 | 3,019 | 4,481 | |
| 237 | Benin | 1996 | DHS | National | both | | 20-49 | | 2,137 | |
| 238 | Benin | 2001 | DHS | National | both | | 15-49 | | 5,449 | |
| 239 | Benin | 2006 | DHS | National | both | | 15-49 | | 14,891 | |
| 240 | Benin | 2007 | STEPS | Community | urban | 25-64 | 25-64 | 955 | 1,508 | |
| 241 | Benin | 2008 | STEPS | National | both | 25-64 | 25-64 | 3,430 | 3,365 | |
| 242 | Benin | 2011-2012 | DHS | National | both | | 15-49 | | 14,589 | |
| 243 | Benin | 2015 | STEPS | National | both | 18-69 | 18-69 | 2,304 | 2,543 | |
| 244 | Benin | 2017-2018 | DHS | National | both | | 15-49 | | 7,180 | |
| 245 | Bhutan | 2007 | STEPS | Community | urban | 25-74 | 25-74 | 1,125 | 1,322 | |
| 246 | Bhutan | 2014 | STEPS | National | both | 18-69 | 18-69 | 1,069 | 1,674 | |
| 247 | Bhutan | 2019 | STEPS | National | both | 15-69 | 15-69 | 2,159 | 3,340 | |
| 248 | Bolivia | 1994 | DHS | National | both | | 20-49 | | 2,128 | |
| 249 | Bolivia | 1998 | DHS | National | both | | 20-49 | | 3,939 | |
| 250 | Bolivia | 2003 | DHS | National | both | | 15-49 | | 16,349 | |
| 251 | Bolivia | 2005-2007 | Cardiovascular and metabolic syndrome risk assessment of Bolivian school children and adolescents - Relationships to obesity, diabetes, income, food intake and physical activity | National | both | 12-18 | 12-18 | 1,499 | 1,841 | |
| 252 | Bolivia | 2008 | DHS | National | both | | 15-49 | | 15,543 | |
| 253 | Bolivia | 2019 | STEPS | National | both | 18-69 | 18-69 | 1,733 | 2,371 | |
| 254 | Bosnia and Herzegovina | 2002 | Non-communicable disease risk factor survey, Federation of B&H | Subnational | both | 25-64 | 25-64 | 1,118 | 1,613 | |
| 255 | Bosnia and Herzegovina | 2012 | Non-communicable disease risk factor survey, Federation of B&H | Subnational | rural | 18+ | 18+ | 1,191 | 1,274 | |
| 256 | Bosnia and Herzegovina | 2012 | Non-communicable disease risk factor survey, Federation of B&H | Subnational | urban | 18+ | 18+ | 591 | 697 | |
| 257 | Bosnia and Herzegovina | 2018-2019 | Determining anthropometric measurements of students aged seven to eight from Sarajevo Canton, Bosnia and Herzegovina | Community | both | 6-8 | 6-8 | 42 | 43 | |
| 258 | Botswana | 2007 | STEPS | National | both | 25-64 | 25-64 | 1,243 | 2,577 | |
| 259 | Botswana | 2014 | STEPS | National | both | 15-69 | 15-69 | 1,298 | 2,602 | |
| 260 | Brazil | 1989 | Pesquisa Nacional sobre Saude e Nutricao | National | both | 5+ | 5+ | 26,642 | 27,504 | |
| 261 | Brazil | 1990-1991 | Fornes et al., Rev Saude Publica 36:12-8, 2002 | Community | urban | 20+ | 20+ | 432 | 613 | |
| 262 | Brazil | 1991-1993 | EPIDOSO | Community | urban | 65+ | 65+ | 269 | 473 | |
| 263 | Brazil | 1992-1998 | Moraes et al., Int J Cardiol 90:205-11, 2003 | Community | urban | 18+ | 18+ | 438 | 543 | |
| 264 | Brazil | 1995 | Health and Nutrition Survey of Rio de Janeiro | Community | urban | 60+ | 60+ | 248 | 385 | |
| 265 | Brazil | 1995 | The 1982 Pelotas (Brazil) Birth Cohort: 13 years follow-up | Community | urban | 13 | 13 | 352 | 363 | |
| 266 | Brazil | 1995-1996 | Cohort study from Porto Alegre | Community | urban | 18+ | 18+ | 489 | 596 | |
| 267 | Brazil | 1996 | DHS | National | both | | 20-49 | | 2,884 | |
| 268 | Brazil | 1996-1997 | Pesquisa sobre Padrões de Vida (PPV) | Subnational | both | 5+ | 5+ | 7,451 | 8,466 | |
| 269 | Brazil | 1996-1997 | The Bambui Cohort Study of Ageing | Community | urban | 18+ | 18+ | 931 | 1,335 | |
| 270 | Brazil | 1997 | The 1982 Pelotas (Brazil) Birth Cohort: 15 years follow-up | Community | urban | 15 | 15 | 559 | 513 | |
| 271 | Brazil | 1998 | Belo Horizonte Heart Study | Community | urban | 6-18 | 6-18 | 658 | 738 | |
| 272 | Brazil | 1999-2000 | Pelotas cross-sectional survey | Community | urban | 20-69 | 20-69 | 839 | 1,096 | |
| 273 | Brazil | 1999-2000 | Projeto Esporte Brasil | National | urban | 6-11 | 6-11 | 107 | 102 | |
| 274 | Brazil | 1999-2000 | Prevalence of Risk Factors for Coronary Artery Disease in the State of Rio Grande do Sul | Subnational | urban | 20+ | 20+ | 504 | 548 | |
| 275 | Brazil | 1999-2000 | The Survey on Health, Well-Being, and Aging in Latin America and the Caribbean (SABE) | Community | urban | 60+ | 60+ | 732 | 1,064 | 3 |
| 276 | Brazil | 2000 | The 1982 Pelotas (Brazil) Birth Cohort: 18 years follow-up | Community | urban | 18 | | 2,228 | | |
| 277 | Brazil | 2001 | Freitas et al., Arq Bras Cardiol 88:191-99, 2007 | Community | urban | 15+ | 15+ | 310 | 331 | |
| 278 | Brazil | 2001 | The 1982 Pelotas (Brazil) Birth Cohort: 19 years follow-up | Community | urban | | 19 | | 919 | |
| 279 | Brazil | 2001 | Projeto Esporte Brasil | National | urban | 6-14 | 6-17 | 249 | 225 | |

| | Country | Study years | Survey/Study name/Citation | Level of representative-ness | Rural, urban, or both | Age range as in NCD-RisC database | | Sample size | | Notes |
|---|---|---|---|---|---|---|---|---|---|---|
| | | | | | | Male | Female | Male | Female | |
| 280 | Brazil | 2001-2003 | Bustos et al., Nutr Metab Cardiovasc Dis 17:581-89, 2007 | Community | both | 22-28 | 22-28 | 992 | 1,064 | |
| 281 | Brazil | 2002 | Study of the prevalence of obesity in children and adolescents (Estudo da prevalência da obesidade em crianças e adolescentes (EPOCA)) | Community | urban | 7-10 | 7-10 | 1,504 | 1,430 | |
| 282 | Brazil | 2002-2003 | Pesquisa de Orcamentos Familiares | National | both | 5+ | 5+ | 81,152 | 80,163 | |
| 283 | Brazil | 2002-2004 | 1978-1979 Ribeira Preto Birth Cohort | Community | urban | 22-25 | 22-25 | 1,012 | 1,082 | |
| 284 | Brazil | 2003 | Nutrition, Physical Activity, and Health Survey (PNAFS) | Community | urban | 20+ | 20+ | 1,155 | 1,941 | |
| 285 | Brazil | 2003 | Projeto Esporte Brasil | National | both | 6-14 | 6-17 | 2,016 | 2,073 | |
| 286 | Brazil | 2003 | Women health in Southern Brazil | Community | urban | | 20-60 | | 986 | |
| 287 | Brazil | 2003-2005 | Sao Paulo Health and Ageing Study | Community | urban | 65+ | 65+ | 783 | 1,198 | |
| 288 | Brazil | 2004 | Caju & Virgen das Gracas | Community | rural | 18+ | 18+ | 291 | 286 | |
| 289 | Brazil | 2004 | Projeto Esporte Brasil | National | both | 6-14 | 6-17 | 14,447 | 12,254 | |
| 290 | Brazil | 2004-2005 | The 1982 Pelotas (Brazil) Birth Cohort: 23 years follow-up | Community | urban | 23 | 23 | 2,173 | 1,935 | |
| 291 | Brazil | 2004-2005 | The 1993 Pelotas (Brazil) Birth Cohort: 11 years follow-up | Community | urban | 10-12 | 10-12 | 2,184 | 2,257 | |
| 292 | Brazil | 2004-2005 | 1994 Ribeira Preto Birth Cohort | Community | urban | 10-11 | 10-11 | 400 | 388 | |
| 293 | Brazil | 2004-2006 | Hearts of Brazil | National | urban | 18+ | 18+ | 550 | 626 | |
| 294 | Brazil | 2005 | Prevalência e Fatores de Risco Cardiovascular em Crianças | Community | urban | 7-12 | 7-12 | 776 | 719 | |
| 295 | Brazil | 2005 | Projeto Esporte Brasil | National | both | 6-14 | 6-17 | 3,615 | 3,287 | |
| 296 | Brazil | 2005 | Syndrome of Obesity and Risk Factors for Cardiovascular Disease Study | Community | urban | 18-90 | 18-90 | 739 | 1,093 | |
| 297 | Brazil | 2005-2006 | Sao Luis Birth Cohort | Community | urban | 7-8 | 7-8 | 347 | 325 | |
| 298 | Brazil | 2006 | Krause et al., J Aging Phys Act 17:387-97, 2009 | Community | urban | 60+ | 60+ | 93 | 1,069 | |
| 299 | Brazil | 2006 | ATITUDE | Subnational | both | 14 | 14-20 | 51 | 2,665 | |
| 300 | Brazil | 2006 | The Ouro Preto Study | Community | urban | 7-14 | 7-14 | 364 | 399 | |
| 301 | Brazil | 2006 | Pesquisa Nacional de Demografia e Saude 2006 | National | both | | 15-49 | | 14,783 | |
| 302 | Brazil | 2006 | Projeto Esporte Brasil | National | both | 6-14 | 6-17 | 7,390 | 6,211 | |
| 303 | Brazil | 2006-2007 | Syndrome of Obesity and Risk Factors for Cardiovascular Disease Study among Teenagers | Community | urban | 11-18 | 11-18 | 230 | 236 | |
| 304 | Brazil | 2007 | Cardiovascular Disease Risk Factors in Caxias do Sul-RS, Brazil Adolescents | Community | urban | 11-17 | 11-17 | 774 | 886 | |
| 305 | Brazil | 2007 | Study of the prevalence of obesity in children and adolescents (Estudo da prevalência da obesidade em crianças e adolescentes (EPOCA)) | Community | urban | 7-14 | 7-14 | 1,354 | 1,463 | |
| 306 | Brazil | 2007 | Prevalence of overweight and obesity in children from Medianeira, Paraná, Brazil | Community | urban | 5-12 | 5-12 | 529 | 515 | |
| 307 | Brazil | 2007 | Projeto Esporte Brasil | National | both | 6-14 | 6-17 | 4,444 | 4,215 | |
| 308 | Brazil | 2007-2008 | Christofaro et al., Scand J Med Sci Sports 23(3):317-22, 2013 | Community | urban | 10-16 | 10-16 | 493 | 528 | |
| 309 | Brazil | 2007-2008 | Nutritional status of children in daycare center | Community | urban | 5-7 | 5-7 | 83 | 81 | |
| 310 | Brazil | 2007-2009 | Schoolchildren's Health | Community | both | 6-14 | 6-17 | 690 | 742 | |
| 311 | Brazil | 2008 | The Bambui Cohort Study of Ageing | Community | urban | 71+ | 71+ | 248 | 456 | |
| 312 | Brazil | 2008 | Caju & Virgen das Gracas | Community | rural | 18+ | 18+ | 273 | 287 | |
| 313 | Brazil | 2008 | The 1993 Pelotas (Brazil) Birth Cohort: 15 years follow-up | Community | urban | 14-15 | 14-15 | 2,001 | 2,095 | |
| 314 | Brazil | 2008 | Projeto Esporte Brasil | National | both | 6-14 | 6-17 | 2,979 | 2,386 | |
| 315 | Brazil | 2008-2009 | Pesquisa de Orcamentos Familiares | National | both | 5+ | 5+ | 85,725 | 88,156 | |
| 316 | Brazil | 2008-2010 | Machado-Rodrigues et al., Ann Hum Biol 41(3): 271-6, 2013 | Community | urban | 10-17 | 10-17 | 376 | 507 | |
| 317 | Brazil | 2009 | Pesquisa Nacional de Saude do Escolar (PeNSE) | National | both | 13-14 | 13-16 | 17,945 | 30,060 | |
| 318 | Brazil | 2009 | Projeto Esporte Brasil | National | both | 6-14 | 6-17 | 652 | 767 | |
| 319 | Brazil | 2009-2010 | EpiFloripa Cohort Study of Ageing - Wave 1 | Community | urban | 60+ | 60+ | 592 | 1,047 | |
| 320 | Brazil | 2009-2010 | EpiFloripa Adults Cohort Study | Community | urban | 20-59 | 20-59 | 755 | 940 | |
| 321 | Brazil | 2010 | Longitudinal Study of Health and Wellbeing in Preschool Age (Project ELOS-Pré) | Community | urban | 5 | 5 | 255 | 247 | |
| 322 | Brazil | 2010 | Projeto Esporte Brasil | National | both | 6-14 | 6-17 | 871 | 1,047 | |
| 323 | Brazil | 2010 | San Pedro | Community | rural | 18+ | 18+ | 153 | 214 | |
| 324 | Brazil | 2010-2011 | The 2004 Pelotas (Brazil) Birth Cohort: 6 years follow-up | Community | urban | 6-7 | 6-7 | 1,721 | 1,631 | |
| 325 | Brazil | 2010-2015 | Baependi Heart Study | Community | rural | 18+ | 18+ | 1,002 | 1,357 | |
| 326 | Brazil | 2011 | ATITUDE | Subnational | both | 14 | 14-19 | 79 | 3,658 | |
| 327 | Brazil | 2011 | Pregnancy in adolescence in municipalities of small size in the Northeast of Brazil | Community | both | 13-19 | 13-19 | 512 | 563 | |
| 328 | Brazil | 2011 | Projeto Esporte Brasil | National | both | 6-14 | 6-17 | 904 | 929 | |
| 329 | Brazil | 2011-2012 | The 1993 Pelotas (Brazil) Birth Cohort: 18 years follow-up | Community | urban | 17-19 | 17-19 | 1,970 | 2,004 | |
| 330 | Brazil | 2011-2012 | Schoolchildren's Health | Community | both | 6-14 | 6-17 | 708 | 1,052 | |
| 331 | Brazil | 2011-2013 | International Study of Childhood Obesity, Lifestyle and the Environment (ISCOLE) | Community | urban | 9-11 | 9-11 | 277 | 287 | |
| 332 | Brazil | 2011-2014 | Profile of Risk Factors for Coronary Arterial Disease in Rio Grade do Sul - Revaluation After 10 Years | Subnational | urban | 20+ | 20+ | 364 | 466 | |
| 333 | Brazil | 2012 | Anthropometric indices in Brazilian children: Colombo, Parana, Brazil | Community | both | 6-11 | 6-11 | 1,022 | 1,013 | |
| 334 | Brazil | 2012 | Longitudinal Study of Health and Wellbeing in Preschool Age (Project ELOS-Pré) | Community | urban | 5-7 | 5-7 | 388 | 348 | |

| | Country | Study years | Survey/Study name/Citation | Level of representative-ness | Rural, urban, or both | Age range as in NCD-RisC database | | Sample size | | Notes |
|---|---|---|---|---|---|---|---|---|---|---|
| | | | | | | Male | Female | Male | Female | |
| 335 | Brazil | 2012 | EpiFloripa Adults Cohort Study | Community | urban | 22-62 | 22-62 | 486 | 655 | |
| 336 | Brazil | 2012 | Projeto Esporte Brasil | National | both | 6-14 | 6-17 | 2,610 | 1,976 | |
| 337 | Brazil | 2012-2013 | Study of the prevalence of obesity in children and adolescents (Estudo da prevalência da obesidade em crianças e adolescentes (EPOCA)) | Community | urban | 7-14 | 7-14 | 1,162 | 1,322 | |
| 338 | Brazil | 2012-2013 | Evaluation of the realization of the human right to adequate food among public and private elementary school students in Maceió | Subnational | urban | 9-11 | 9-11 | 639 | 701 | |
| 339 | Brazil | 2012-2013 | The 1982 Pelotas (Brazil) Birth Cohort: 30 years follow-up | Community | urban | 30 | 30 | 1,753 | 1,798 | |
| 340 | Brazil | 2012-2013 | Prevalence of Leptin Polymorphism Gln223Arg | Community | urban | 18+ | 18+ | 282 | 523 | |
| 341 | Brazil | 2013 | Pesquisas Nacional de Saude | National | both | 18+ | 18+ | 24,918 | 32,351 | |
| 342 | Brazil | 2013 | Projeto Esporte Brasil | National | urban | 6-14 | 6-17 | 1,026 | 960 | |
| 343 | Brazil | 2013-2014 | EpiFloripa Cohort Study of Ageing - Wave 2 | Community | urban | 63+ | 63+ | 404 | 744 | |
| 344 | Brazil | 2013-2014 | Estudo de Riscos Cardiovasculares em Adolescentes (ERICA) | National | both | 12-17 | 12-17 | 32,723 | 40,675 | |
| 345 | Brazil | 2014 | Brazilian Guide to the Physical Fitnees related to Health Assessment and Lifestyle Habits | Community | urban | 14-19 | 14-19 | 473 | 535 | |
| 346 | Brazil | 2014 | Longitudinal Study of Health and Wellbeing in Preschool Age (Project ELOS-Pré) | Community | urban | 7-9 | 7-9 | 223 | 200 | |
| 347 | Brazil | 2014 | Projeto Esporte Brasil | National | both | 6-14 | 6-17 | 219 | 212 | |
| 348 | Brazil | 2014-2015 | II Diagnóstico de Saúde da População Materno-Infantil do Estado de Alagoas | Subnational | both | | 19-49 | | 3,135 | |
| 349 | Brazil | 2014-2015 | Latin American Study of Nutrition and Health (ELANS) | National | urban | 15-65 | 15-65 | 942 | 1,058 | |
| 350 | Brazil | 2014-2015 | EpiFloripa Adults Cohort Study | Community | urban | 25-65 | 25-65 | 353 | 476 | |
| 351 | Brazil | 2014-2015 | Schoolchildren's Health | Community | both | 6-14 | 6-17 | 923 | 1,398 | |
| 352 | Brazil | 2015 | The 2004 Pelotas (Brazil) Birth Cohort: 11 years follow-up | Community | urban | 10-11 | 10-11 | 1,736 | 1,632 | |
| 353 | Brazil | 2015 | Pesquisa Nacional de Saude do Escolar (PeNSE) | National | both | 13-14 | 13-17 | 2,219 | 5,054 | |
| 354 | Brazil | 2015 | Projeto Esporte Brasil | National | urban | 6-14 | 6-17 | 383 | 496 | |
| 355 | Brazil | 2015-2016 | The Ouro Preto Study | Community | rural | 18+ | 18+ | 184 | 329 | |
| 356 | Brazil | 2015-2016 | Brazilian Longitudinal Study of the Elderly Health and Wellness | National | both | 50+ | 50+ | 3,937 | 5,064 | |
| 357 | Brazil | 2015-2016 | The 1993 Pelotas (Brazil) Birth Cohort: 22 years follow-up | Community | urban | 21-23 | 21-23 | 1,687 | 1,872 | |
| 358 | Brazil | 2016 | ATITUDE | Subnational | both | 14 | 14-19 | 64 | 3,198 | |
| 359 | Brazil | 2016 | Projeto Esporte Brasil | National | urban | 6-14 | 6-17 | 169 | 110 | |
| 360 | Brazil | 2016-2017 | Schoolchildren's Health | Community | both | 6-14 | 6-17 | 904 | 1,424 | |
| 361 | Brazil | 2016-2017 | Study in Presidente Prudente | Community | urban | 18+ | 18+ | 304 | 481 | |
| 362 | Brazil | 2017 | Effectiveness of interventions for health promotion in frail older adults with chronic non-communicable diseases in primary healthcare in Recife: a randomized community trial | Community | urban | 60+ | 60+ | 155 | 528 | |
| 363 | Brazil | 2017 | HealthRise Evaluation | Subnational | both | 30+ | 30+ | 599 | 1,169 | |
| 364 | Brazil | 2017 | Projeto Esporte Brasil | National | both | 6-14 | 6-17 | 2,226 | 1,350 | |
| 365 | Brazil | 2017 | Intervention in physical education classes to reduce sedentary behavior and improve cognitive function: SACODE | Community | both | 14-19 | 14-19 | 501 | 631 | |
| 366 | Brazil | 2017-2018 | EpiFloripa Cohort Study of Ageing - Wave 3 | Community | urban | 60+ | 60+ | 361 | 635 | |
| 367 | Brazil | 2018 | Healthy Living study in Lagoa do Carro | Community | both | 5-15 | 5-15 | 704 | 657 | |
| 368 | Brazil | 2018 | Projeto Esporte Brasil | National | both | 6-14 | 6-17 | 956 | 1,151 | |
| 369 | Brazil | 2018-2019 | Study of the prevalence of obesity in children and adolescents (Estudo da prevalência da obesidade em crianças e adolescentes (EPOCA)) | Community | urban | 7-14 | 7-14 | 680 | 871 | |
| 370 | Brazil | 2018-2019 | BP-SAMPA Project | Community | urban | 10-17 | 10-17 | 1,328 | 1,853 | |
| 371 | Brazil | 2018-2019 | Epidemiology in the health (Santo Anastácio Edition) | Community | urban | 18+ | 18+ | 105 | 145 | |
| 372 | Brazil | 2019 | Pesquisas Nacional de Saude | National | both | 15+ | 15+ | 3,304 | 3,243 | |
| 373 | Brazil | 2019 | Projeto Esporte Brasil | National | both | 6-14 | 6-17 | 1,126 | 1,356 | |
| 374 | Brazil | 2020 | Projeto Esporte Brasil | National | urban | 6-14 | 6-17 | 276 | 153 | |
| 375 | Brazil | 2020-2022 | ATITUDE | Subnational | both | 14 | 14-19 | 36 | 2,271 | |
| 376 | Brazil | 2021 | Projeto Esporte Brasil | National | urban | 6-14 | 6-17 | 131 | 170 | |
| 377 | Brunei Darussalam | 2010-2011 | National Health And Nutritional Status Survey (NHANSS) | National | both | 5-75 | 5-75 | 1,027 | 1,157 | |
| 378 | Brunei Darussalam | 2014 | Global School-based Student Health Survey | National | both | 12-17 | 12-17 | 1,145 | 1,326 | |
| 379 | Brunei Darussalam | 2015-2016 | National Non-Communicable Diseases Survey (NNCDS) | National | both | 18-69 | 18-69 | 814 | 1,075 | |
| 380 | Brunei Darussalam | 2019 | Global School-based Student Health Survey | National | both | 12-17 | 12-17 | 1,061 | 1,152 | |
| 381 | Bulgaria | 1984-1988 | Postnatal growth of SGA and normal born high school graduates | Community | urban | 5 | 5 | 753 | 952 | |
| 382 | Bulgaria | 1985-1989 | Postnatal growth of SGA and normal born high school graduates | Community | urban | 6 | 6 | 788 | 993 | |
| 383 | Bulgaria | 1986-1990 | Postnatal growth of SGA and normal born high school graduates | Community | urban | 7 | 7 | 817 | 1,049 | |
| 384 | Bulgaria | 1987-1991 | Postnatal growth of SGA and normal born high school graduates | Community | urban | 8 | 8 | 767 | 989 | |
| 385 | Bulgaria | 1988-1992 | Postnatal growth of SGA and normal born high school graduates | Community | urban | 9 | 9 | 732 | 940 | |
| 386 | Bulgaria | 1989-1993 | Postnatal growth of SGA and normal born high school graduates | Community | urban | 10 | 10 | 719 | 906 | |

| | Country | Study years | Survey/Study name/Citation | Level of representative-ness | Rural, urban, or both | Age range as in NCD-RisC database | | Sample size | | Notes |
|---|---|---|---|---|---|---|---|---|---|---|
| | | | | | | Male | Female | Male | Female | |
| 387 | Bulgaria | 1990-1994 | Postnatal growth of SGA and normal born high school graduates | Community | urban | 11 | 11 | 729 | 927 | |
| 388 | Bulgaria | 1991-1995 | Postnatal growth of SGA and normal born high school graduates | Community | urban | 12 | 12 | 740 | 972 | |
| 389 | Bulgaria | 1992-1996 | Postnatal growth of SGA and normal born high school graduates | Community | urban | 13 | 13 | 752 | 947 | |
| 390 | Bulgaria | 1993 | Anthropometric characterization of growth and development in children aged 7 to 13 years from Sofia at the beginning of the 21st century | Community | urban | 6-7 | 6-7 | 109 | 119 | |
| 391 | Bulgaria | 1993-1997 | Postnatal growth of SGA and normal born high school graduates | Community | urban | 14 | 14 | 750 | 893 | |
| 392 | Bulgaria | 1994 | Anthropometric characterization of growth and development in children aged 7 to 13 years from Sofia at the beginning of the 21st century | Community | urban | 7-8 | 7-8 | 108 | 118 | |
| 393 | Bulgaria | 1994-1998 | Postnatal growth of SGA and normal born high school graduates | Community | urban | 15 | 15 | 816 | 972 | |
| 394 | Bulgaria | 1995 | Anthropometric characterization of growth and development in children aged 7 to 13 years from Sofia at the beginning of the 21st century | Community | urban | 8-9 | 8-9 | 109 | 120 | |
| 395 | Bulgaria | 1995-1999 | Postnatal growth of SGA and normal born high school graduates | Community | urban | 16 | 16 | 896 | 1,012 | |
| 396 | Bulgaria | 1995-2001 | Anthropometric characterization of growth and development in children aged 7 to17 years from Sofia at the beginning of the 21st century | Community | urban | 7-17 | 7-17 | 1,127 | 1,132 | |
| 397 | Bulgaria | 1996 | Anthropometric characterization of growth and development in children aged 7 to 13 years from Sofia at the beginning of the 21st century | Community | urban | 9-10 | 9-10 | 109 | 119 | |
| 398 | Bulgaria | 1996-2000 | Postnatal growth of SGA and normal born high school graduates | Community | urban | 17 | 17 | 816 | 872 | |
| 399 | Bulgaria | 1997 | Anthropometric characterization of growth and development in children aged 7 to 13 years from Sofia at the beginning of the 21st century | Community | urban | 10-11 | 10-11 | 109 | 119 | |
| 400 | Bulgaria | 1997-2000 | Postnatal growth of SGA and normal born high school graduates | Community | urban | 18 | 18 | 485 | 322 | |
| 401 | Bulgaria | 1998 | Anthropometric characterization of growth and development in children aged 7 to 13 years from Sofia at the beginning of the 21st century | Community | urban | 11-12 | 11-12 | 109 | 118 | |
| 402 | Bulgaria | 1999 | Anthropometric characterization of growth and development in children aged 7 to 13 years from Sofia at the beginning of the 21st century | Community | urban | 12-13 | 12-13 | 109 | 118 | |
| 403 | Bulgaria | 2004 | National Nutrition Survey | National | both | 15+ | 15+ | 515 | 515 | |
| 404 | Bulgaria | 2004-2005 | Anthropometric characterization of growth and development in children aged 3 to 6 years from Sofia at the beginning of the 21st century | Community | urban | 5-6 | 5-6 | 160 | 160 | |
| 405 | Bulgaria | 2006-2007 | Waist circumference percentile curves for Bulgarian children and adolescents aged 6-18 years | Community | urban | 5-18 | 5-18 | 2,052 | 1,758 | |
| 406 | Bulgaria | 2008 | Childhood Obesity Surveillance Initiative 1 | National | both | 7-8 | 7-8 | 1,657 | 1,661 | |
| 407 | Bulgaria | 2009 | Survey of children in Varna kindergartens | Community | urban | 5-6 | 5-6 | 188 | 157 | |
| 408 | Bulgaria | 2013 | Childhood Obesity Surveillance Initiative 3 | National | both | 7 | 7 | 1,671 | 1,677 | |
| 409 | Bulgaria | 2014 | Bulgarian National Monitoring of Dietary Intake | National | both | 20+ | 20+ | 1,421 | 1,552 | |
| 410 | Bulgaria | 2015-2016 | Childhood Obesity Surveillance Initiative 4 | National | both | 7 | 7 | 1,702 | 1,698 | |
| 411 | Bulgaria | 2016 | Feel4Diabetes | Community | urban | 6-10 | 6-10 | 1,447 | 1,522 | |
| 412 | Bulgaria | 2016-2017 | Erasmus plus KA2, Healthyland | Community | urban | 5 | 5 | 26 | 24 | |
| 413 | Bulgaria | 2017-2018 | Erasmus plus KA2, Healthyland | Community | urban | 5-6 | 5-6 | 49 | 51 | |
| 414 | Bulgaria | 2019 | Childhood Obesity Surveillance Initiative 5 | National | both | 7 | 7 | 1,536 | 1,531 | |
| 415 | Bulgaria | 2020 | National Survey on Risk Factors for Population's Health in Bulgaria 2020 | National | both | 5+ | 5+ | 1,686 | 2,297 | |
| 416 | Burkina Faso | 1992-1993 | DHS | National | both | | 20-49 | | 3,190 | |
| 417 | Burkina Faso | 1998-1999 | DHS | National | both | | 20-49 | | 3,114 | |
| 418 | Burkina Faso | 2002 | Vulnérabilité Alimentaire et Sécurité Nutritionnelle dans la Gnagna (VASN-Gnagna) | Subnational | rural | 5+ | 5+ | 1,471 | 3,522 | |
| 419 | Burkina Faso | 2003 | DHS | National | both | | 15-49 | | 11,001 | |
| 420 | Burkina Faso | 2004 | Ouedraogo et al., Public Health Nutr 11:1280-87, 2008 | Community | urban | 35+ | 35+ | 956 | 1,066 | |
| 421 | Burkina Faso | 2010 | DHS | National | both | | 15-49 | | 7,755 | |
| 422 | Burkina Faso | 2013 | STEPS | National | both | 25-64 | 25-64 | 2,223 | 2,250 | |
| 423 | Burkina Faso | 2017 | ARISE Network Adolescent Health Study (Nouna) | Community | both | 12-19 | 12-19 | 915 | 680 | |
| 424 | Burkina Faso | 2021 | DHS | National | both | | 15-49 | | 8,005 | |
| 425 | Burkina Faso | 2021 | STEPS | National | both | 18-69 | 18-69 | 1,467 | 2,002 | |
| 426 | Burundi | 2010 | DHS | National | both | | 15-49 | | 4,188 | |
| 427 | Burundi | 2016-2017 | DHS | National | both | | 15-49 | | 7,909 | |
| 428 | Cabo Verde | 2007 | STEPS | National | both | 25-64 | 25-64 | 658 | 1,066 | |
| 429 | Cabo Verde | 2020 | STEPS | National | both | 18-69 | 18-69 | 1,824 | 2,636 | |
| 430 | Cambodia | 2000 | DHS | National | both | | 15-49 | | 6,915 | |
| 431 | Cambodia | 2005 | DHS | National | both | | 15-49 | | 8,130 | |
| 432 | Cambodia | 2008 | Anthropometrics Survey | National | both | | 15-49 | | 5,955 | |
| 433 | Cambodia | 2010 | DHS | National | both | | 15-49 | | 8,856 | |
| 434 | Cambodia | 2010 | STEPS | National | both | 25-64 | 25-64 | 1,881 | 3,344 | |

| | Country | Study years | Survey/Study name/Citation | Level of representative-ness | Rural, urban, or both | Age range as in NCD-RisC database | | Sample size | | Notes |
|---|---|---|---|---|---|---|---|---|---|---|
| | | | | | | Male | Female | Male | Female | |
| 435 | Cambodia | 2014 | DHS | National | both | | 15-49 | | 10,821 | |
| 436 | Cambodia | 2021-2022 | DHS | National | both | | 15-49 | | 9,332 | |
| 437 | Cambodia | 2023 | STEPS | National | both | 18-69 | 18-69 | 1,504 | 2,669 | 5 |
| 438 | Cameroon | 1998 | DHS | National | both | | 20-49 | | 1,429 | |
| 439 | Cameroon | 1998-1999 | Essential Non-communicable disease Health Intervention Project (ENHIP) | Community | rural | 15+ | 15+ | 523 | 738 | |
| 440 | Cameroon | 1998-1999 | Essential Non-communicable disease Health Intervention Project (ENHIP) | Community | urban | 15+ | 15+ | 523 | 640 | |
| 441 | Cameroon | 2003 | STEPS | Subnational | urban | 15+ | 15+ | 3,672 | 5,490 | |
| 442 | Cameroon | 2004 | DHS | National | both | | 15-49 | | 4,646 | |
| 443 | Cameroon | 2007 | Cameroon Burden of Diabetes - Second Survey | Subnational | urban | 18+ | 18+ | 3,345 | 4,633 | |
| 444 | Cameroon | 2009 | National Survey of Micronutrient Status and Consumption of Fortifiable Foods | National | both | | 15-49 | | 816 | |
| 445 | Cameroon | 2009-2012 | Anthropologie nutritionnelle des migrants d'Afrique centrale à la ville et en France | Subnational | both | 18-76 | 18-76 | 528 | 584 | |
| 446 | Cameroon | 2011 | DHS | National | both | | 15-49 | | 7,343 | |
| 447 | Cameroon | 2013 | Prevalence and risk factors of chronic kidney disease in urban adult Cameroonians according to three common estimators of the glomerular filtration rate: a cross-sectional study | Community | urban | 19+ | 19+ | 265 | 232 | |
| 448 | Cameroon | 2014 | Prevalence and determinants of chronic kidney disease in rural and urban Cameroonians: A cross-sectional study | Community | both | 20+ | 20+ | 177 | 246 | |
| 449 | Cameroon | 2014-2015 | Cardiovascular risk factors screening in urban and rural areas in the Far-North Region Cameroon | Subnational | both | 20+ | 20+ | 520 | 369 | |
| 450 | Cameroon | 2018 | Prevalence and determinants of chronic kidney disease in urban adults' populations of northern Cameroon | Community | urban | 20+ | 20+ | 210 | 221 | |
| 451 | Cameroon | 2018-2019 | DHS | National | both | | 15-64 | | 6,255 | |
| 452 | Canada | 1981 | Canada Fitness Survey | National | both | 7-64 | 7-64 | 7,432 | 7,940 | |
| 453 | Canada | 1985-1986 | INTERSALT, St Johns | Community | urban | 20-59 | 20-59 | 100 | 100 | |
| 454 | Canada | 1985-1988 | MONICA, Halifax | Community | both | 25-64 | 25-64 | 438 | 420 | |
| 455 | Canada | 1986-1992 | Canada Heart Health Survey | National | both | 18-74 | 18-74 | 9,644 | 9,777 | |
| 456 | Canada | 1991-1992 | Canadian Study of Health and Aging | Community | both | 70+ | 70+ | 236 | 348 | |
| 457 | Canada | 1993 | Chen et al., Int J Obes Relat Metab Disord 22:771-77, 1998 | Community | rural | 18-74 | 18-74 | 803 | 988 | |
| 458 | Canada | 1995 | MONICA, Halifax | Community | both | 25-64 | 25-64 | 274 | 287 | |
| 459 | Canada | 1995-1997 | Canadian Multicentre Osteoporosis Study (CaMos) - Adult Baseline | Subnational | both | 25+ | 25+ | 2,803 | 6,343 | |
| 460 | Canada | 1996 | Canadian Study of Health and Aging | Community | both | 70+ | 70+ | 236 | 348 | |
| 461 | Canada | 1997 | PEI Nutrition Survey | Subnational | both | 18-74 | 18-74 | 999 | 995 | |
| 462 | Canada | 1998-2000 | Canadian Multicentre Osteoporosis Study (CaMos) - Adult Year 3 follow-up | Subnational | both | 42-64 | 42-64 | 782 | 1,670 | |
| 463 | Canada | 2000-2003 | Canadian Multicentre Osteoporosis Study (CaMos) - Adult Year 5 follow-up | Subnational | both | 30+ | 30+ | 1,992 | 4,820 | |
| 464 | Canada | 2004-2005 | Canadian Multicentre Osteoporosis Study (CaMos) - Youth baseline | Subnational | both | 16-24 | 16-24 | 471 | 521 | |
| 465 | Canada | 2005 | Canadian Community Health Survey | National | both | 15+ | 15+ | 1,684 | 2,030 | |
| 466 | Canada | 2005-2008 | Canadian Multicentre Osteoporosis Study (CaMos) - Adult Year 10 follow-up | Subnational | both | 35+ | 35+ | 1,486 | 3,661 | |
| 467 | Canada | 2006-2008 | Canadian Multicentre Osteoporosis Study (CaMos) - Youth Year 2 follow-up | Subnational | both | 17-27 | 17-27 | 331 | 383 | |
| 468 | Canada | 2007-2009 | Canadian Health Measures Survey, Cycle 1 | National | both | 6-79 | 6-79 | 2,703 | 2,864 | |
| 469 | Canada | 2008 | Canadian Community Health Survey | National | both | 15+ | 15+ | 1,689 | 1,987 | |
| 470 | Canada | 2009-2011 | Canadian Health Measures Survey, Cycle 2 | National | both | 5-79 | 5-79 | 2,870 | 3,086 | |
| 471 | Canada | 2011-2013 | International Study of Childhood Obesity, Lifestyle and the Environment (ISCOLE) | Community | urban | 9-11 | 9-11 | 238 | 327 | |
| 472 | Canada | 2012-2013 | Canadian Health Measures Survey, Cycle 3 | National | both | 5-79 | 5-79 | 2,670 | 2,676 | |
| 473 | Canada | 2012-2014 | Canadian Multicentre Osteoporosis Study (CaMos) - Adult Year 16 follow-up | Subnational | both | 60+ | 60+ | 445 | 1,307 | |
| 474 | Canada | 2014-2015 | Canadian Health Measures Survey, Cycle 4 | National | both | 5-79 | 5-79 | 2,697 | 2,674 | |
| 475 | Canada | 2016-2017 | Canadian Health Measures Survey, Cycle 5 | National | both | 5-79 | 5-79 | 2,677 | 2,660 | |
| 476 | Canada | 2018-2019 | Canadian Health Measures Survey, Cycle 6 | National | both | 5-79 | 5-79 | 2,685 | 2,680 | |
| 477 | Central African Republic | 1994-1995 | DHS | National | both | | 20-49 | | 1,760 | |
| 478 | Central African Republic | 2010 | STEPS | Subnational | both | 25-64 | 25-64 | 1,846 | 1,967 | |
| 479 | Central African Republic | 2011-2012 | Epidemiology of dementia in Central Africa: Bangui | Community | urban | 65+ | 65+ | 157 | 300 | |
| 480 | Central African Republic | 2011-2012 | Epidemiology of dementia in Central Africa: Nola | Community | rural | 65+ | 65+ | 184 | 264 | |
| 481 | Central African Republic | 2017 | STEPS | Subnational | both | 25-64 | 25-64 | 1,076 | 1,834 | |
| 482 | Chad | 1996-1997 | DHS | National | both | | 20-49 | | 3,262 | |
| 483 | Chad | 2004 | DHS | National | both | | 20-49 | | 2,618 | |
| 484 | Chad | 2008 | STEPS | Community | urban | 25-64 | 25-64 | 995 | 845 | |
| 485 | Chad | 2014-2015 | DHS | National | both | | 15-49 | | 9,733 | |
| 486 | Chile | 1988 | Chilean Health Study | Subnational | urban | 15+ | 15+ | 471 | 741 | |
| 487 | Chile | 1989 | INCLEN | Community | urban | 35-65 | | 199 | | |
| 488 | Chile | 1992-1993 | Miquel et al., Gastroenterology 115(4):937-46, 1998 | Community | urban | 18+ | 18+ | 657 | 1,031 | |
| 489 | Chile | 1999-2000 | The Survey on Health, Well-Being, and Aging in Latin America and the Caribbean (SABE) | Community | urban | 60+ | 60+ | 410 | 806 | 3 |

| | Country | Study years | Survey/Study name/Citation | Level of representative-ness | Rural, urban, or both | Age range as in NCD-RisC database | | Sample size | | Notes |
|---|---|---|---|---|---|---|---|---|---|---|
| | | | | | | Male | Female | Male | Female | |
| 490 | Chile | 2000 | Nervi et al., J Hepatol 45(2):299-305, 2006 | Community | urban | 18+ | 18+ | 335 | 624 | |
| 491 | Chile | 2001-2003 | Bustos et al., Nutr Metab Cardiovasc Dis 17:581-89, 2007 | Community | both | 22-28 | 22-28 | 436 | 562 | |
| 492 | Chile | 2003 | Encuesta Nacional de Salud | National | both | 17+ | 17+ | 1,557 | 1,867 | |
| 493 | Chile | 2004-2005 | CArdiovascular Risk factors Multiple Evaluation in Latin America (CARMELA) | Community | urban | 25-64 | 25-64 | 783 | 865 | |
| 494 | Chile | 2005 | Palomo et al., Rev Med Chil 135:904-12, 2007 | Community | urban | 18-74 | 18-74 | 339 | 668 | |
| 495 | Chile | 2009-2010 | Encuesta Nacional de Salud | National | both | 15+ | 15+ | 1,935 | 2,869 | |
| 496 | Chile | 2010-2011 | Encuesta Nacional de Consumo Alimentario | National | both | 5+ | 5+ | 1,840 | 2,902 | |
| 497 | Chile | 2011-2012 | CESCAS Study | Community | urban | 35-74 | 35-74 | 917 | 1,000 | |
| 498 | Chile | 2013 | Global School-based Student Health Survey | National | both | 13-17 | 13-17 | 799 | 793 | |
| 499 | Chile | 2014-2015 | Latin American Study of Nutrition and Health (ELANS) | National | urban | 15-65 | 15-65 | 425 | 454 | |
| 500 | Chile | 2016-2017 | Encuesta Nacional de Salud | National | both | 15+ | 15+ | 1,977 | 3,420 | |
| 501 | China | 1979-1982 | East Beijing Study 1 | Community | urban | 20-84 | 20-84 | 361 | 380 | |
| 502 | China | 1982 | China National Nutrition Survey | National | both | 7+ | 7+ | 14,418 | 13,683 | |
| 503 | China | 1983 | Sino-MONICA Shanghai | Community | rural | 30-64 | 30-64 | 624 | 630 | |
| 504 | China | 1984-1985 | Sino-MONICA Beijing | Community | both | 25-64 | 25-64 | 813 | 857 | |
| 505 | China | 1985 | Chinese National Surveys on Students Constitution and Health | National | both | 7-18 | 7-18 | 205,041 | 204,796 | |
| 506 | China | 1985-1986 | Shatin New Town Study | Community | urban | 70+ | 70+ | 276 | 669 | |
| 507 | China | 1986 | INTERSALT, Beijing | Community | urban | 20-59 | 20-59 | 100 | 100 | |
| 508 | China | 1986 | INTERSALT, Nanning | Community | both | 20-59 | 20-59 | 100 | 100 | |
| 509 | China | 1986 | INTERSALT, Tianjin | Community | urban | 20-59 | 20-59 | 100 | 100 | |
| 510 | China | 1986-1989 | Ewang et al., Zhonghua Liu Xing Bing Xue Za Zhi 26:394-9, 2005 | Community | both | 45-64 | | 18,244 | | |
| 511 | China | 1986-1989 | Sino-MONICA Shanghai | Community | rural | 25-64 | 25-64 | 675 | 753 | |
| 512 | China | 1987 | INCLEN | Community | urban | 35-65 | | 989 | | |
| 513 | China | 1988 | Sino-MONICA Hebei | Community | both | 25-64 | | 800 | | |
| 514 | China | 1988 | Sino-MONICA Heilongjiang | Community | urban | 25-64 | 25-64 | 800 | 800 | |
| 515 | China | 1988 | Sino-MONICA Henan | Community | urban | 25-64 | 25-64 | 345 | 427 | |
| 516 | China | 1988 | Sino-MONICA Neimenggu | Community | urban | 25-64 | 25-64 | 396 | 400 | |
| 517 | China | 1988 | Sino-MONICA Sichuan | Community | both | 25-64 | 25-64 | 312 | 334 | |
| 518 | China | 1988 | Sino-MONICA Shandong | Community | urban | 25-64 | 25-64 | 211 | 225 | |
| 519 | China | 1988-1989 | Sino-MONICA Beijing | Community | both | 25-64 | 25-64 | 701 | 862 | |
| 520 | China | 1988-1989 | Sino-MONICA Jilin | Community | urban | 25-64 | 25-64 | 380 | 400 | |
| 521 | China | 1988-1989 | Sino-MONICA Jiangxi | Community | urban | 25-64 | 25-64 | 379 | 386 | |
| 522 | China | 1988-1989 | Sino-MONICA Liaoning | Community | both | 25-64 | 25-64 | 728 | 734 | |
| 523 | China | 1988-1990 | East Beijing Study 2 | Community | urban | 20-84 | 20-84 | 135 | 148 | |
| 524 | China | 1989 | The Tianjin Project | Community | urban | 15-64 | 15-64 | 3,894 | 3,971 | |
| 525 | China | 1989 | China Health and Nutrition Study | National | both | 5-45 | 5-45 | 2,556 | 2,715 | 6 |
| 526 | China | 1989 | Sino-MONICA Fujian | Community | urban | 25-64 | 25-64 | 179 | 191 | |
| 527 | China | 1989 | Sino-MONICA Jiangsu | Community | rural | 25-64 | 25-64 | 398 | 399 | |
| 528 | China | 1990-1991 | China Prospective Study | National | both | 40-79 | | 230,676 | | |
| 529 | China | 1991 | Hua et al., Zhonghua Nei Ke Za Zhi 36:18-20, 1997 | Community | rural | 60+ | 60+ | 288 | 335 | |
| 530 | China | 1991 | The Hong Kong study on health, health risk and quality of life in the Chinese elderly cohort | Community | both | 70+ | 70+ | 943 | 944 | |
| 531 | China | 1991 | China National Hypertension Survey Epidemiology Follow-up Study | National | both | 40+ | 40+ | 75,696 | 79,040 | |
| 532 | China | 1991 | China Health and Nutrition Study | National | both | 5+ | 5+ | 5,586 | 5,920 | 6 |
| 533 | China | 1991 | Sino-MONICA Shanghai | Community | rural | 30-64 | 30-64 | 564 | 624 | |
| 534 | China | 1991-1992 | Fangshan Cohort Study | Community | urban | 34-86 | 34-86 | 871 | 1,736 | |
| 535 | China | 1992 | Huashan Study | Community | urban | 35-75 | 35-75 | 892 | 965 | |
| 536 | China | 1992 | China National Nutrition Survey | National | both | 5+ | 5+ | 33,714 | 36,271 | |
| 537 | China | 1992 | Sino-MONICA Sichuan | Community | both | 25-64 | 25-64 | 608 | 526 | |
| 538 | China | 1992-1993 | Anzhen 02 Cohort Study | Community | urban | 34-65 | 34-65 | 2,032 | 2,120 | |
| 539 | China | 1993 | China Health and Nutrition Study | National | both | 5+ | 5+ | 5,371 | 5,563 | 6 |
| 540 | China | 1993 | Sino-MONICA Anhui | Community | urban | 25-64 | 25-64 | 193 | 195 | |
| 541 | China | 1993 | Sino-MONICA Beijing | Community | both | 25-64 | 25-64 | 613 | 816 | |
| 542 | China | 1993 | Sino-MONICA Jiangsu | Community | urban | 25-64 | 25-64 | 462 | 365 | |
| 543 | China | 1993 | Sino-MONICA Liaoning | Community | both | 25-64 | 25-64 | 493 | 500 | |
| 544 | China | 1995 | Chinese National Surveys on Students Constitution and Health | National | both | 7-18 | 7-18 | 103,009 | 101,772 | |
| 545 | China | 1995-1996 | Hong Kong Cardiovascular Risk Factor Prevalence Study 1995-1996 | Community | urban | 25-74 | 25-74 | 1,412 | 1,478 | |
| 546 | China | 1996 | Wang et al., Zhonghua Liu Xing Bing Xue Za Zhi 24:272-75, 2003 | Community | both | 25-64 | 25-64 | 735 | 720 | |

| | Country | Study years | Survey/Study name/Citation | Level of representative-ness | Rural, urban, or both | Age range as in NCD-RisC database | | Sample size | | Notes |
|---|---|---|---|---|---|---|---|---|---|---|
| | | | | | | Male | Female | Male | Female | |
| 547 | China | 1996 | The Tianjin Project | Community | urban | 15-64 | 15-64 | 722 | 717 | |
| 548 | China | 1996-2000 | Shanghai Women's Health Study | Community | urban | | 40-70 | | 74,915 | |
| 549 | China | 1996-2003 | Wu et al., Osteoporos Int 15:751-59, 2004 | Community | urban | | 18+ | | 3,418 | |
| 550 | China | 1997 | China Health and Nutrition Study | National | both | 5+ | 5+ | 5,550 | 5,648 | 6 |
| 551 | China | 1997 | INTERMAP, Beijing | Community | rural | 40-59 | 40-59 | 133 | 139 | |
| 552 | China | 1997 | INTERMAP, Guangxi | Community | rural | 40-59 | 40-59 | 140 | 138 | |
| 553 | China | 1997 | INTERMAP, Shanxi | Community | rural | 40-59 | 40-59 | 143 | 146 | |
| 554 | China | 1998 | Shanghai Diabetes Study | Community | urban | 25+ | 25+ | 1,264 | 1,768 | |
| 555 | China | 1998-2000 | Jia et al., Obes Rev 3:157-65, 2002 | Community | urban | 20+ | 20+ | 1,106 | 1,670 | |
| 556 | China | 1999 | Chen et al., Zhonghua Yi Xue Za Zhi 85(40):2830-4, 2005 | Subnational | both | 35-85 | 35-85 | 13,549 | 10,315 | |
| 557 | China | 1999 | Wang et al., Zhonghua Liu Xing Bing Xue Za Zhi 24:272-75, 2003 | Community | both | 25-64 | 25-64 | 817 | 684 | |
| 558 | China | 1999-2000 | Xu et al., Public Health Nutr 8:47-51, 2005 | Community | both | 35+ | 35+ | 18,194 | 18,902 | |
| 559 | China | 2000 | China Health and Nutrition Study | National | both | 5+ | 5+ | 5,829 | 6,099 | 6 |
| 560 | China | 2000 | Chinese National Surveys on Students Constitution and Health | National | both | 7-18 | 7-18 | 107,997 | 108,096 | |
| 561 | China | 2000-2001 | The International Collaborative Study of Cardiovascular Disease in Asia | National | both | 35-74 | 35-74 | 7,512 | 8,006 | |
| 562 | China | 2001 | Shanghai Diabetes Study | Community | urban | 25+ | 25+ | 1,264 | 1,768 | |
| 563 | China | 2002 | Ma et al., Zhonghua Liu Xing Bing Xue Za Zhi 25:1035-8, 2004 | Subnational | both | 18+ | 18+ | 7,352 | 7,352 | |
| 564 | China | 2002 | China National Nutrition and Health Survey | National | both | 5+ | 5+ | 84,194 | 92,687 | |
| 565 | China | 2002-2003 | Fan et al., J Gastroenterol Hepatol 20:1825-32, 2005 | Community | urban | 15+ | 15-74 | 5,502 | 7,767 | |
| 566 | China | 2002-2006 | Shanghai Men's Health Study | Community | urban | 40-74 | | 61,445 | | |
| 567 | China | 2004 | Tian et al., Prev Med 48:59-63, 2009 | Community | rural | 15+ | 15+ | 1,022,669 | 1,163,313 | |
| 568 | China | 2004 | Beijing Child and Adolescent Metabolic Syndrome study | Community | both | 5-18 | 5-18 | 10,562 | 10,412 | |
| 569 | China | 2004 | China Chronic Disease and Risk Factors Surveillance (CCDRFS) | National | rural | 18-69 | 18-69 | 9,561 | 10,937 | |
| 570 | China | 2004 | China Chronic Disease and Risk Factors Surveillance (CCDRFS) | National | urban | 18-69 | 18-69 | 4,978 | 7,317 | |
| 571 | China | 2004 | China Health and Nutrition Study | National | both | 5+ | 5+ | 5,229 | 5,516 | 6 |
| 572 | China | 2004-2005 | Xinjiang Children and Adolescent Survey | Community | urban | 7-18 | 7-18 | 2,030 | 2,231 | |
| 573 | China | 2004-2006 | Pang et al., Intern Med 47:893-97, 2008 | Community | rural | 35+ | 35+ | 22,962 | 22,962 | |
| 574 | China | 2004-2006 | Shanghai Women's Health Study | Community | urban | | 45-80 | | 64,545 | |
| 575 | China | 2004-2008 | China Kadoorie Biobank baseline survey | Subnational | rural | 35-74 | 35-74 | 115,792 | 162,848 | |
| 576 | China | 2004-2008 | China Kadoorie Biobank baseline survey | Subnational | urban | 35-74 | 35-74 | 89,219 | 132,860 | |
| 577 | China | 2004-2008 | Shanghai Men's Health Study | Community | urban | 41-80 | | 54,800 | | |
| 578 | China | 2005 | Ye et al., J Am Coll Cardiol 49:1798-805, 2007 | Community | urban | 50-70 | 50-70 | 743 | 906 | |
| 579 | China | 2005 | Chinese National Surveys on Students Constitution and Health | National | both | 7-18 | 7-18 | 117,598 | 116,704 | |
| 580 | China | 2005-2006 | Zhou et al., World J Gastroenterol 13:6419-24, 2007 | Community | urban | 18-79 | 18-79 | 1,101 | 2,063 | |
| 581 | China | 2005-2006 | Hong Kong Growth Survey | Community | urban | 7-19 | 7-19 | 7,472 | 7,370 | |
| 582 | China | 2006 | Beijing Eye Study | Community | both | 45+ | 45+ | 1,394 | 1,820 | |
| 583 | China | 2006 | China Health and Nutrition Study | National | both | 5+ | 5+ | 4,950 | 5,397 | 6 |
| 584 | China | 2006-2007 | Handan Eye Study | Community | rural | 30+ | 30+ | 2,995 | 3,456 | |
| 585 | China | 2006-2012 | Qingdao Diabetes Cohort Study | Subnational | both | 35-74 | 35-74 | 4,047 | 6,346 | |
| 586 | China | 2007 | China Chronic Disease and Risk Factors Surveillance (CCDRFS) | National | rural | 18-69 | 18-69 | 14,212 | 15,006 | |
| 587 | China | 2007 | China Chronic Disease and Risk Factors Surveillance (CCDRFS) | National | urban | 18-69 | 18-69 | 8,461 | 10,160 | |
| 588 | China | 2007-2008 | China National Diabetes & Metabolic Disorders Study | National | both | 20+ | 20+ | 18,419 | 27,820 | |
| 589 | China | 2007-2010 | WHO Study on global AGEing and adult health (SAGE) | National | both | 50+ | 50+ | 5,759 | 6,616 | |
| 590 | China | 2007-2011 | Shanghai Women's Health Study | Community | urban | | 47-83 | | 52,116 | |
| 591 | China | 2008 | China Health and Retirement Longitudinal Study (CHARLS), pilot survey | Subnational | both | 45+ | 45+ | 919 | 920 | |
| 592 | China | 2008-2009 | Chinese Longitudinal Healthy Longevity Survey | National | both | 65+ | 65+ | 6,827 | 8,976 | 7 |
| 593 | China | 2008-2010 | Fangshan Family-based Ischemic Stroke Study in China (FISSIC) program | Community | rural | | 40+ | | 36,449 | |
| 594 | China | 2008-2011 | Shanghai Men's Health Study | Community | urban | 43-84 | | 51,948 | | |
| 595 | China | 2009 | The 33 Chinese Communities Health Study (33CCHS) | Subnational | urban | 18-74 | 18-74 | 8,156 | 7,321 | |
| 596 | China | 2009 | China Health and Nutrition Study | National | both | 5+ | 5+ | 5,176 | 5,489 | 6 |
| 597 | China | 2009 | The nutrition-based comprehensive intervention study on childhood obesity in China | Subnational | urban | 6-11 | 6-11 | 4,495 | 4,269 | |
| 598 | China | 2009-2010 | China National Survey of Chronic Kidney Disease | National | both | 18+ | 18+ | 20,003 | 26,854 | |
| 599 | China | 2009-2011 | The FAMILY Cohort | Community | urban | 15+ | 15+ | 8,318 | 9,557 | |
| 600 | China | 2010 | China Chronic Disease and Risk Factors Surveillance (CCDRFS) | National | rural | 18+ | 18+ | 27,827 | 31,844 | |
| 601 | China | 2010 | China Chronic Disease and Risk Factors Surveillance (CCDRFS) | National | urban | 18+ | 18+ | 17,239 | 21,608 | |
| 602 | China | 2010 | Chinese National Surveys on Students Constitution and Health | National | both | 7-18 | 7-18 | 107,611 | 107,611 | |
| 603 | China | 2010-2013 | China Nutrition and Health Surveillance | National | both | 5+ | 5+ | 72,494 | 87,092 | |

| | Country | Study years | Survey/Study name/Citation | Level of representative-ness | Rural, urban, or both | Age range as in NCD-RisC database | | Sample size | | Notes |
|---|---|---|---|---|---|---|---|---|---|---|
| | | | | | | Male | Female | Male | Female | |
| 604 | China | 2010-2014 | National Free Preconception Health Examination Project | National | rural | 20-64 | | 16,166,534 | | |
| 605 | China | 2011 | Beijing Childhood Eye Study | Community | both | 7-18 | 7-18 | 6,686 | 6,967 | |
| 606 | China | 2011 | Beijing Children Eye Study | Community | both | 5-13 | 5-13 | 291 | 261 | |
| 607 | China | 2011 | Beijing Eye Study | Community | both | 50+ | 50+ | 1,467 | 1,895 | |
| 608 | China | 2011 | China Health and Nutrition Study | National | both | 5+ | 5+ | 6,770 | 7,478 | 6 |
| 609 | China | 2011-2012 | China Health and Retirement Longitudinal Study (CHARLS), baseline survey | National | both | 45+ | 45+ | 6,337 | 7,003 | |
| 610 | China | 2011-2012 | Chinese Longitudinal Healthy Longevity Survey | National | both | 65+ | 65+ | 4,035 | 4,620 | 7 |
| 611 | China | 2011-2013 | International Study of Childhood Obesity, Lifestyle and the Environment (ISCOLE) | Community | urban | 9-11 | 9-11 | 293 | 258 | |
| 612 | China | 2011-2014 | The FAMILY Cohort | Community | urban | 15+ | 15+ | 4,593 | 5,557 | |
| 613 | China | 2012 | Beijing Children Eye Study | Community | both | 5-13 | 5-13 | 283 | 251 | |
| 614 | China | 2012 | Beijing Eye High School Students Study | Community | both | 16-18 | 16-18 | 2,088 | 2,340 | |
| 615 | China | 2012 | China Health and Retirement Longitudinal Study (CHARLS), wave 2 pilot survey | Subnational | both | 45+ | 45+ | 856 | 934 | |
| 616 | China | 2012 | Shandong Children Study | Community | rural | 5-18 | 5-18 | 1,663 | 1,385 | |
| 617 | China | 2012 | Shandong Children Study | Community | urban | 5-18 | 5-18 | 1,423 | 1,381 | |
| 618 | China | 2012-2013 | The Kailuan Study | Community | urban | 18+ | 18+ | 80,921 | 21,385 | |
| 619 | China | 2012-2013 | The Seven Northeastern Cities (SNEC) Study | Subnational | urban | 5-17 | 5-17 | 4,769 | 4,577 | |
| 620 | China | 2012-2015 | Shanghai Men's Health Study | Community | urban | 47-87 | | 40,921 | | |
| 621 | China | 2012-2015 | Shanghai Women's Health Study | Community | urban | | 52-88 | | 49,592 | |
| 622 | China | 2013 | China Health and Retirement Longitudinal Study (CHARLS), wave 2 survey | National | both | 45+ | 45+ | 5,898 | 6,582 | |
| 623 | China | 2013 | Gobi Desert Children Eye Study | Community | urban | 6-21 | 6-21 | 800 | 761 | |
| 624 | China | 2013-2014 | China Chronic Disease and Risk Factors Surveillance (CCDRFS) | National | rural | 18-69 | 18-69 | 39,987 | 51,871 | |
| 625 | China | 2013-2014 | China Chronic Disease and Risk Factors Surveillance (CCDRFS) | National | urban | 18-69 | 18-69 | 26,374 | 38,695 | |
| 626 | China | 2014 | Chinese Longitudinal Healthy Longevity Survey | National | both | 65+ | 65+ | 2,978 | 3,172 | 7 |
| 627 | China | 2014 | Chinese National Surveys on Students Constitution and Health | National | both | 7-18 | 7-18 | 107,216 | 107,138 | |
| 628 | China | 2014 | Shanghai Municipal Surveys on Students Constitution and Health | Community | both | 7-18 | 7-18 | 7,758 | 7,665 | |
| 629 | China | 2014-2015 | The Kailuan Study | Community | urban | 18+ | 18+ | 73,161 | 18,280 | |
| 630 | China | 2015 | China Nutrition and Health Surveillance (Adult Chronic Disease and Nutrition Surveillance) | National | both | 18+ | 18+ | 84,991 | 97,136 | |
| 631 | China | 2015 | China Health and Retirement Longitudinal Study (CHARLS), wave 3 survey | National | both | 45+ | 45+ | 7,032 | 7,719 | |
| 632 | China | 2015 | China Health and Nutrition Study | National | both | 5+ | 5+ | 6,538 | 7,222 | 6 |
| 633 | China | 2015-2016 | China Chronic Disease and Risk Factors Surveillance (CCDRFS) | National | rural | 18-69 | 18-69 | 43,616 | 49,395 | |
| 634 | China | 2015-2016 | China Chronic Disease and Risk Factors Surveillance (CCDRFS) | National | urban | 18-69 | 18-69 | 30,987 | 38,057 | |
| 635 | China | 2015-2016 | INTERMAP China Prospective (ICP) | Subnational | rural | 40-79 | 40-79 | 334 | 413 | |
| 636 | China | 2015-2017 | Henan Rural Cohort | Subnational | rural | 18-79 | 18-79 | 15,432 | 23,652 | |
| 637 | China | 2016 | Greater Beijing School Children Myopia Study | Subnational | rural | 6-18 | 6-18 | 12,873 | 12,866 | |
| 638 | China | 2016 | Greater Beijing School Children Myopia Study | Subnational | urban | 6-18 | 6-18 | 4,384 | 3,650 | |
| 639 | China | 2016-2017 | China Nutrition and Health Surveillance (Children and Lactating Women) | National | both | 5-17 | 5-17 | 42,215 | 42,301 | |
| 640 | China | 2016-2018 | The FAMILY Cohort | Community | urban | 18+ | 18+ | 839 | 1,122 | |
| 641 | China | 2016-2018 | Smart device usage, lifestyles behaviors, physical fitness, and eye problems: A prospective study in Hong Kong adolescents | Community | urban | 7-15 | 7-15 | 703 | 741 | |
| 642 | China | 2018 | China Health and Nutrition Survey | National | both | 5+ | 5+ | 7,870 | 8,845 | 6 |
| 643 | China | 2018 | Chinese Longitudinal Healthy Longevity Survey | National | both | 65+ | 65+ | 6,430 | 7,781 | 7 |
| 644 | China | 2018-2019 | China Chronic Disease and Risk Factors Surveillance (CCDRFS) | National | rural | 18-69 | 18-69 | 37,707 | 46,845 | |
| 645 | China | 2018-2019 | China Chronic Disease and Risk Factors Surveillance (CCDRFS) | National | urban | 18-69 | 18-69 | 29,780 | 41,081 | |
| 646 | China | 2019 | Chinese National Surveys on Students Constitution and Health | National | both | 6-18 | 6-18 | 114,825 | 113,992 | |
| 647 | Colombia | 1986 | INTERSALT | Community | rural | 20-59 | 20-59 | 96 | 95 | |
| 648 | Colombia | 1995 | DHS | National | both | | 20-49 | | 3,068 | |
| 649 | Colombia | 1997-1998 | Identification of Risk Factors of Non-Transmissible Adult Chronic Diseases in School-age Populations in the City of de Cali | Community | urban | 6-17 | 6-17 | 1,084 | 1,077 | |
| 650 | Colombia | 2000 | DHS | National | both | | 20-49 | | 2,929 | |
| 651 | Colombia | 2001 | CINDI/CARMEN - Bucaramaga | Community | urban | 15-74 | 15-74 | 627 | 1,218 | |
| 652 | Colombia | 2002 | CINDI/CARMEN - Bogota | Community | urban | 15-74 | 15-74 | 322 | 570 | |
| 653 | Colombia | 2002 | The Santa Fe Study (Santa Fe) | Community | urban | 15-69 | 15-69 | 394 | 684 | |
| 654 | Colombia | 2002 | The Santa Fe Study (Tunjuelito) | Community | urban | 15-29 | 15-29 | 208 | 312 | |
| 655 | Colombia | 2004-2005 | CArdiovascular Risk factors Multiple Evaluation in Latin America (CARMELA) | Community | urban | 25-64 | 25-64 | 738 | 812 | |
| 656 | Colombia | 2005 | DHS | National | both | 5-64 | 5-64 | 43,436 | 57,778 | |
| 657 | Colombia | 2005 | Encuesta Nacional de Situacion Nutricional | National | both | 5-12 | 5-49 | 2,644 | 6,088 | |
| 658 | Colombia | 2007 | Encuesta Nacional de Salud | National | both | 18-69 | 18-69 | 5,462 | 7,686 | |

| | Country | Study years | Survey/Study name/Citation | Level of representative-ness | Rural, urban, or both | Age range as in NCD-RisC database | | Sample size | | Notes |
|---|---|---|---|---|---|---|---|---|---|---|
| | | | | | | Male | Female | Male | Female | |
| 659 | Colombia | 2010 | DHS | National | both | 5-64 | 5-64 | 65,086 | 76,792 | |
| 660 | Colombia | 2010 | STEPS | Subnational | both | 15-64 | 15-64 | 1,034 | 1,356 | |
| 661 | Colombia | 2011-2013 | International Study of Childhood Obesity, Lifestyle and the Environment (ISCOLE) | Community | urban | 9-11 | 9-11 | 454 | 462 | |
| 662 | Colombia | 2014-2015 | Latin American Study of Nutrition and Health (ELANS) | National | urban | 15-65 | 15-65 | 394 | 404 | |
| 663 | Colombia | 2015 | Encuesta Nacional de Situacion Nutricional | National | both | 5-64 | 5-64 | 51,180 | 58,896 | |
| 664 | Colombia | 2015 | STEPS | Subnational | both | 15-64 | 15-64 | 979 | 1,181 | |
| 665 | Colombia | 2016 | The Survey on Health, Well-Being, and Aging in Latin America and the Caribbean (SABE) | National | both | 60+ | 60+ | 9,041 | 11,467 | |
| 666 | Colombia | 2018 | COPEN: Estudio Colombiano de Perfiles Nutricionales | Subnational | urban | 5-75 | 5-75 | 916 | 919 | |
| 667 | Colombia | 2022 | COPEN: Estudio Colombiano de Perfiles Nutricionales | Subnational | urban | 5-80 | 5-80 | 959 | 1,029 | |
| 668 | Comoros | 1996 | DHS | National | both | | 20-49 | | 744 | |
| 669 | Comoros | 2011 | STEPS | National | both | 25-64 | 25-64 | 1,541 | 3,505 | |
| 670 | Comoros | 2012 | DHS | National | both | | 15-49 | | 4,845 | |
| 671 | Congo | 1986 | Enquête Brazzaville 1986 | Community | urban | 5-50 | 5-50 | 129 | 1,005 | |
| 672 | Congo | 1987 | Maire et al., Rev Epidemiol Sante Publique 40:252-58, 1992 | Community | rural | | 16-45 | | 750 | |
| 673 | Congo | 1987 | Enquête Nationale Congo 1987 | National | rural | | 13-49 | | 1,356 | |
| 674 | Congo | 1991 | Enquête Brazzaville 1991 | Community | urban | 5-90 | 5-90 | 2,393 | 3,149 | |
| 675 | Congo | 1996 | Enquête Brazzaville 1996 | Community | urban | 5-90 | 5-90 | 2,496 | 3,073 | |
| 676 | Congo | 2004 | STEPS | Community | urban | 25-64 | 25-64 | 1,013 | 956 | |
| 677 | Congo | 2005 | DHS | National | both | | 15-49 | | 6,266 | |
| 678 | Congo | 2011-2012 | DHS | National | both | | 15-49 | | 5,060 | |
| 679 | Congo | 2019 | Diabetes prevalence and risk factors | Community | both | 19+ | 19+ | 721 | 807 | |
| 680 | Cook Islands | 2003 | STEPS | National | both | 25-64 | 25-64 | 925 | 958 | |
| 681 | Cook Islands | 2011 | Global School-based Student Health Survey | National | both | 13-17 | 13-17 | 530 | 543 | |
| 682 | Cook Islands | 2013-2015 | STEPS | National | both | 18-64 | 18-64 | 456 | 469 | |
| 683 | Cook Islands | 2015 | Global School-based Student Health Survey | National | both | 13-17 | 13-17 | 304 | 313 | |
| 684 | Costa Rica | 2004 | CAMDI | Community | urban | 20+ | 20+ | 304 | 624 | |
| 685 | Costa Rica | 2004-2006 | Costa Rican Longevity and Healthy Aging Study Pre-1945 Cohort Wave 1 | National | both | 60+ | 60+ | 1,163 | 1,346 | |
| 686 | Costa Rica | 2006-2008 | Costa Rican Longevity and Healthy Aging Study Pre-1945 Cohort Wave 2 | National | both | 62+ | 62+ | 944 | 1,102 | |
| 687 | Costa Rica | 2008-2009 | Encuesta Nacional de Nutricion 2008-2009 | National | both | | 45-64 | | 661 | |
| 688 | Costa Rica | 2009 | Global School-based Student Health Survey | National | both | 13 | 13-17 | 356 | 1,308 | |
| 689 | Costa Rica | 2009-2010 | Costa Rican Longevity and Healthy Aging Study Pre-1945 Cohort Wave 3 | National | both | 64+ | 64+ | 737 | 887 | |
| 690 | Costa Rica | 2010 | Costa Rican National Cardiovascular Risk Factors Survey, 2010 | National | both | 20+ | 20+ | 778 | 1,958 | |
| 691 | Costa Rica | 2010-2011 | Costa Rican Longevity and Healthy Aging Study 1945-1955 Cohort Wave 1 | National | both | 54-66 | 54-66 | 1,058 | 1,676 | |
| 692 | Costa Rica | 2012-2014 | Costa Rican Longevity and Healthy Aging Study 1945-1955 Cohort Wave 2 | National | both | 56-68 | 56-68 | 867 | 1,470 | |
| 693 | Costa Rica | 2014 | Costa Rican National Cardiovascular Risk Factors Survey, 2014 | National | both | 20+ | 20+ | 1,003 | 2,196 | |
| 694 | Costa Rica | 2014-2015 | Latin American Study of Nutrition and Health (ELANS) | National | urban | 15-65 | 15-65 | 397 | 403 | |
| 695 | Costa Rica | 2016 | Censo Escolar peso y talla | National | both | 6-12 | 6-12 | 178,416 | 168,958 | |
| 696 | Cote d'Ivoire | 1994 | DHS | National | both | | 20-49 | | 2,682 | |
| 697 | Cote d'Ivoire | 1998-1999 | DHS | National | both | | 15-49 | | 2,740 | |
| 698 | Cote d'Ivoire | 2005 | STEPS | Subnational | rural | 15-64 | 15-64 | 894 | 1,022 | |
| 699 | Cote d'Ivoire | 2005 | STEPS | Subnational | urban | 15-64 | 15-64 | 1,071 | 1,437 | |
| 700 | Cote d'Ivoire | 2011-2012 | DHS | National | both | | 15-49 | | 4,601 | |
| 701 | Cote d'Ivoire | 2014 | Prevalence and factors associated with obesity in a periurban west African population | Community | urban | 18+ | 18+ | 153 | 317 | |
| 702 | Cote d'Ivoire | 2021 | DHS | National | both | | 15-49 | | 6,767 | |
| 703 | Croatia | 1997-1999 | Croatian Health Survey | National | both | 18+ | 18+ | 1,972 | 2,983 | |
| 704 | Croatia | 2002-2007 | Epidemiology of arterial hypertension in Croatia (EH-UH) | National | both | 18+ | 18+ | 446 | 600 | |
| 705 | Croatia | 2003-2004 | School Health Survey | National | both | 7-17 | 7-17 | 1,302 | 1,153 | |
| 706 | Croatia | 2005 | Endemic Nephropathy and Arterial hypertension (ENAH) | Subnational | rural | 18+ | 18+ | 264 | 367 | |
| 707 | Croatia | 2006-2008 | The cardiovascular risk factors in school age - intervention model development | National | both | 6-20 | 6-20 | 6,011 | 5,625 | |
| 708 | Croatia | 2008 | Endemic Nephropathy and Arterial hypertension (ENAH) | Subnational | rural | 18+ | 18+ | 331 | 527 | |
| 709 | Croatia | 2010 | Endemic Nephropathy and Arterial hypertension (ENAH) | Subnational | rural | 18+ | 18+ | 252 | 393 | |
| 710 | Croatia | 2014 | Croatian Physical Activity in Adolesence Longitudinal Study (CRO-PALS) | Community | both | 14-17 | 14-17 | 428 | 410 | |
| 711 | Croatia | 2015 | Endemic Nephropathy and Arterial hypertension (ENAH) Follow-up Study | Subnational | rural | 18+ | 18+ | 224 | 460 | |
| 712 | Croatia | 2015-2016 | Childhood Obesity Surveillance Initiative 4 | National | both | 8 | 8 | 1,364 | 1,364 | |
| 713 | Croatia | 2015-2019 | Motor skills in preschool children | National | both | 5-7 | 5-7 | 405 | 382 | |
| 714 | Croatia | 2016-2017 | Croatian Physical Activity in Adolesence Longitudinal Study (CRO-PALS) | Community | both | 17-20 | 17-20 | 383 | 384 | |
| 715 | Croatia | 2018-2021 | Epidemiology of arterial hypertension in Croatia (EH-UH) | National | both | 18+ | 18+ | 381 | 622 | |

| | Country | Study years | Survey/Study name/Citation | Level of representative-ness | Rural, urban, or both | Age range as in NCD-RisC database | | Sample size | | Notes |
|---|---|---|---|---|---|---|---|---|---|---|
| | | | | | | Male | Female | Male | Female | |
| 716 | Croatia | 2019 | Childhood Obesity Surveillance Initiative 5 | National | both | 8-9 | 8-9 | 2,778 | 2,677 | |
| 717 | Cuba | 1981-1982 | Berdasco, Eur J Clin Nutr 1994; 48 Suppl 3:S155-63; discussion S64, 1994 | Subnational | both | 20-59 | 20-59 | 11,355 | 18,708 | |
| 718 | Cuba | 1991 | Non communicable disease risk factors in Cienfuegos | Community | urban | 15+ | 15+ | 527 | 600 | |
| 719 | Cuba | 1999-2000 | The Survey on Health, Well-Being, and Aging in Latin America and the Caribbean (SABE) | Community | urban | 60+ | 60+ | 630 | 1,044 | 3 |
| 720 | Cuba | 2001 | Non communicable disease risk factors in Cienfuegos | Community | urban | 15-74 | 15-74 | 727 | 888 | |
| 721 | Cuba | 2001 | National Survey on Risk Factors and Chronic Diseases (NSRFCD) | National | urban | 15+ | 15+ | 10,163 | 11,376 | |
| 722 | Cuba | 2010 | National Survey on Risk Factors and Chronic Diseases (NSRFCD) | National | both | 15+ | 15+ | 3,344 | 3,868 | |
| 723 | Cuba | 2010-2011 | Non communicable disease risk factors in Cienfuegos | Community | urban | 15-74 | 15-74 | 611 | 873 | |
| 724 | Cuba | 2018-2020 | Encuesta nacional de salud Cuba | National | both | 6+ | 6+ | 5,991 | 7,479 | |
| 725 | Cyprus | 1999-2000 | Countrywide Integrated Noncommunicable Diseases Intervention Programme Cyprus | National | both | 25-65 | 25-65 | 457 | 546 | |
| 726 | Cyprus | 2007-2008 | Asthma Study Cyprus | National | both | 15-18 | 15-18 | 368 | 490 | |
| 727 | Cyprus | 2007-2008 | Childhood asthma and atopy in Cyprus | Subnational | both | 7-9, 13-15 | 7-9, 13-15 | 566 | 590 | |
| 728 | Cyprus | 2007-2010 | Identification and prevention of Dietary- and lifestyle-induced health EFfects In Children and infantS (IDEFICS) | Community | urban | 5-9 | 5-9 | 1,129 | 1,106 | |
| 729 | Cyprus | 2015-2016 | Childhood Obesity Surveillance Initiative 4 | National | urban | 6-9 | 6-9 | 685 | 623 | |
| 730 | Cyprus | 2019 | Childhood Obesity Surveillance Initiative 5 | National | both | 6-10 | 6-10 | 1,001 | 994 | |
| 731 | Czechia | 1985 | Czech-MONICA | National | both | 25-64 | 25-64 | 1,243 | 1,303 | |
| 732 | Czechia | 1988 | Czech-MONICA | National | both | 25-64 | 25-64 | 1,357 | 1,408 | |
| 733 | Czechia | 1992 | Czech-MONICA | National | both | 25-64 | 25-64 | 1,131 | 1,207 | |
| 734 | Czechia | 1997-1998 | Czech post-MONICA | National | both | 25-64 | 25-64 | 1,527 | 1,665 | |
| 735 | Czechia | 1998-2002 | Health, Lifestyle and the Environment | National | urban | 45-54 | 45-54 | 1,539 | 2,044 | |
| 736 | Czechia | 1999-2000 | ELSPAC (The European Longitudinal Study of Pregnancy and Childhood) | Community | both | 8 | 8 | 445 | 444 | |
| 737 | Czechia | 2000-2001 | Czech post-MONICA | National | both | 25-64 | 25-64 | 1,628 | 1,690 | |
| 738 | Czechia | 2001 | 6th Nationwide Anthropometric Survey of Children and Adolescents 2001 | National | both | 5-19 | 5-20 | 18,960 | 22,523 | |
| 739 | Czechia | 2002-2003 | ELSPAC (The European Longitudinal Study of Pregnancy and Childhood) | Community | both | 11 | 11 | 544 | 521 | |
| 740 | Czechia | 2002-2005 | Health, Alcohol and Psychosocial Factors In Eastern Europe | Subnational | urban | 45-70 | 45-70 | 3,289 | 3,901 | |
| 741 | Czechia | 2004-2005 | ELSPAC (The European Longitudinal Study of Pregnancy and Childhood) | Community | both | 13 | 13 | 440 | 366 | |
| 742 | Czechia | 2004-2005 | Health, Lifestyle and the Environment | National | urban | 45-54 | 45-54 | 775 | 1,072 | |
| 743 | Czechia | 2006-2007 | ELSPAC (The European Longitudinal Study of Pregnancy and Childhood) | Community | both | 15 | 15 | 517 | 456 | |
| 744 | Czechia | 2006-2009 | Czech post-MONICA | National | both | 25-64 | 25-64 | 1,717 | 1,861 | |
| 745 | Czechia | 2008 | Childhood Obesity Surveillance Initiative 1 | National | both | 6-7 | 6-7 | 834 | 838 | |
| 746 | Czechia | 2009 | Health, Lifestyle and the Environment | National | urban | 45-54 | 45-54 | 307 | 447 | |
| 747 | Czechia | 2009-2010 | ELSPAC (The European Longitudinal Study of Pregnancy and Childhood) | Community | both | 18 | 18 | 262 | 280 | |
| 748 | Czechia | 2010 | Childhood Obesity Surveillance Initiative 2 | National | both | 6-7 | 6-7 | 1,203 | 1,239 | |
| 749 | Czechia | 2010-2011 | ELSPAC (The European Longitudinal Study of Pregnancy and Childhood) | Community | both | 19 | 19 | 127 | 147 | |
| 750 | Czechia | 2013 | Childhood Obesity Surveillance Initiative 3 | National | both | 6-7 | 6-7 | 1,267 | 1,200 | |
| 751 | Czechia | 2014-2015 | European Heath Examination Survey | National | both | 25-64 | 25-64 | 473 | 691 | |
| 752 | Czechia | 2015-2016 | Childhood Obesity Surveillance Initiative 4 | National | both | 6-7 | 6-7 | 809 | 883 | |
| 753 | Czechia | 2015-2018 | Czech post-MONICA | National | both | 25-64 | 25-64 | 1,220 | 1,345 | |
| 754 | Czechia | 2019-2020 | European Heath Examination Survey | National | both | 25-64 | 25-64 | 425 | 627 | |
| 755 | Czechia | 2019-2022 | CELSPAC: YA (The Central European Longitudinal Studies of Parents and Children: Young Adults) | Community | both | 27-30 | 27-30 | 140 | 152 | |
| 756 | Czechia | 2020 | Childhood Obesity Surveillance Initiative 5 | National | both | 6-7 | 6-7 | 1,149 | 1,112 | |
| 757 | Denmark | 1977 | The Danish Conscription Database | National | both | 17-26 | | 25,710 | | 1 |
| 758 | Denmark | 1978 | The Danish Conscription Database | National | both | 17-26 | | 13,580 | | 1 |
| 759 | Denmark | 1979 | The Danish Conscription Database | National | both | 17-26 | | 6,661 | | 1 |
| 760 | Denmark | 1980 | The Danish Conscription Database | National | both | 17-26 | | 2,897 | | |
| 761 | Denmark | 1980 | Copenhagen School Health Records Register | Community | urban | 6-13 | 6-13 | 11,234 | 11,194 | |
| 762 | Denmark | 1981 | The Danish Conscription Database | National | both | 17-26 | | 1,752 | | |
| 763 | Denmark | 1981 | Copenhagen School Health Records Register | Community | urban | 6-13 | 6-13 | 11,433 | 11,349 | |
| 764 | Denmark | 1981-1983 | Copenhagen City Heart Study | Subnational | urban | 20+ | 20+ | 5,633 | 6,937 | |
| 765 | Denmark | 1982 | The Danish Conscription Database | National | both | 17-26 | | 1,186 | | |
| 766 | Denmark | 1982 | Copenhagen School Health Records Register | Community | urban | 6-13 | 6-13 | 10,971 | 10,746 | |
| 767 | Denmark | 1982-1984 | MONICA I (baseline), The Glostrup Population Studies | Subnational | urban | 30-61 | 30-61 | 1,940 | 1,844 | |
| 768 | Denmark | 1983 | The Danish Conscription Database | National | both | 17-26 | | 762 | | |
| 769 | Denmark | 1983 | Copenhagen School Health Records Register | Community | urban | 6-13 | 6-13 | 7,956 | 7,945 | |
| 770 | Denmark | 1984 | The Danish Conscription Database | National | both | 17-26 | | 379 | | |
| 771 | Denmark | 1984 | Copenhagen School Health Records Register | Community | urban | 6-13 | 6-13 | 4,886 | 4,947 | |

| | Country | Study years | Survey/Study name/Citation | Level of representative-ness | Rural, urban, or both | Age range as in NCD-RisC database | | Sample size | | Notes |
|---|---|---|---|---|---|---|---|---|---|---|
| | | | | | | Male | Female | Male | Female | |
| 772 | Denmark | 1984-1985 | The Epidemiology of Gallstones in a 70 Year-Old Danish Population | Community | both | 70 | 70 | 187 | 157 | |
| 773 | Denmark | 1985 | Copenhagen School Health Records Register | Community | urban | 6-13 | 6-13 | 4,266 | 4,275 | |
| 774 | Denmark | 1985 | INTERSALT | Community | urban | 20-59 | 20-59 | 99 | 100 | |
| 775 | Denmark | 1986 | Copenhagen School Health Records Register | Community | urban | 6-13 | 6-13 | 4,533 | 4,462 | |
| 776 | Denmark | 1986-1987 | MONICA II, The Glostrup Population Studies | Subnational | urban | 29-61 | 29-61 | 746 | 753 | |
| 777 | Denmark | 1987 | Nilsson et al., J Intern Med 237:479-86, 1995 | Community | urban | 51 | | 439 | | |
| 778 | Denmark | 1987 | Copenhagen School Health Records Register | Community | urban | 6-13 | 6-13 | 4,686 | 4,544 | |
| 779 | Denmark | 1987-1988 | MONICA I (5-year follow-up), The Glostrup Population Studies | Subnational | urban | 35-66 | 35-66 | 1,524 | 1,463 | |
| 780 | Denmark | 1988 | Copenhagen School Health Records Register | Community | urban | 6-13 | 6-13 | 4,738 | 4,501 | |
| 781 | Denmark | 1989 | Copenhagen School Health Records Register | Community | urban | 6-13 | 6-13 | 4,845 | 4,598 | |
| 782 | Denmark | 1990 | Copenhagen School Health Records Register | Community | urban | 6-13 | 6-13 | 4,669 | 4,449 | |
| 783 | Denmark | 1991 | Copenhagen School Health Records Register | Community | urban | 6-13 | 6-13 | 4,844 | 4,811 | |
| 784 | Denmark | 1991-1992 | Monica III, the Glostrup population studies | Subnational | both | 29-71 | 29-71 | 1,009 | 998 | |
| 785 | Denmark | 1991-1994 | Copenhagen City Heart Study | Subnational | urban | 20+ | 20+ | 4,274 | 5,416 | |
| 786 | Denmark | 1992 | Copenhagen School Health Records Register | Community | urban | 6-13 | 6-13 | 5,243 | 5,104 | |
| 787 | Denmark | 1993 | Copenhagen School Health Records Register | Community | urban | 6-13 | 6-13 | 4,968 | 4,890 | |
| 788 | Denmark | 1993-1994 | MONICA I (10-year follow-up), The Glostrup Population Studies | Subnational | urban | 41-72 | 41-72 | 1,333 | 1,323 | |
| 789 | Denmark | 1993-1997 | EPIC Aarhus | Community | urban | 50-65 | 50-65 | 8,430 | 8,717 | |
| 790 | Denmark | 1993-1997 | EPIC Copenhagen | Community | urban | 50-65 | 50-65 | 18,729 | 21,133 | |
| 791 | Denmark | 1994 | Copenhagen School Health Records Register | Community | urban | 6-13 | 6-13 | 4,065 | 4,005 | |
| 792 | Denmark | 1995 | Copenhagen School Health Records Register | Community | urban | 6-13 | 6-13 | 5,437 | 5,379 | |
| 793 | Denmark | 1996 | Copenhagen School Health Records Register | Community | urban | 6-13 | 6-13 | 4,674 | 4,670 | |
| 794 | Denmark | 1996-1997 | Drivsholm et al., Diabet Med 18:126-32, 2001 | Subnational | urban | 60 | 60 | 325 | 370 | |
| 795 | Denmark | 1997 | Copenhagen School Health Records Register | Community | urban | 7-13 | 7-13 | 4,105 | 4,025 | |
| 796 | Denmark | 1997-1998 | European Youth Heart Study | Community | urban | 8-18 | 8-18 | 485 | 532 | |
| 797 | Denmark | 1998 | Copenhagen School Health Records Register | Community | urban | 8-13 | 8-13 | 3,203 | 3,253 | |
| 798 | Denmark | 1999 | Copenhagen School Health Records Register | Community | urban | 9-13 | 9-13 | 2,860 | 2,777 | |
| 799 | Denmark | 2000 | Copenhagen School Health Records Register | Community | urban | 10-13 | 10-13 | 1,911 | 1,923 | |
| 800 | Denmark | 2001 | Copenhagen School Health Records Register | Community | urban | 11-13 | 11-13 | 1,594 | 1,595 | |
| 801 | Denmark | 2001-2002 | The Copenhagen School Child Intervention Study | Community | urban | 5-8 | 5-8 | 362 | 329 | |
| 802 | Denmark | 2001-2003 | Copenhagen City Heart Study | Subnational | urban | 20+ | 20+ | 2,567 | 3,446 | |
| 803 | Denmark | 2002 | Copenhagen School Health Records Register | Community | urban | 12-13 | 12-13 | 860 | 895 | |
| 804 | Denmark | 2002-2003 | Odense Androgen Study | Community | urban | 20-29 | | 783 | | |
| 805 | Denmark | 2003 | Copenhagen School Health Records Register | Community | urban | 13 | 13 | 322 | 369 | |
| 806 | Denmark | 2003-2004 | Copenhagen General Population Study 1 | Subnational | urban | 20+ | 20+ | 4,828 | 5,397 | |
| 807 | Denmark | 2003-2004 | European Youth Heart Study | Community | urban | 8-17 | 8-17 | 392 | 509 | |
| 808 | Denmark | 2004-2005 | The Copenhagen School Child Intervention Study | Community | urban | 8-11 | 8-11 | 121 | 130 | |
| 809 | Denmark | 2005 | Copenhagen General Population Study 1 | Subnational | urban | 20+ | 20+ | 5,171 | 6,023 | |
| 810 | Denmark | 2006 | Copenhagen General Population Study 1 | Subnational | urban | 20+ | 20+ | 5,055 | 4,861 | |
| 811 | Denmark | 2006 | The Danish Conscription Database | National | both | 17-26 | | 25,063 | | |
| 812 | Denmark | 2006-2008 | The Health2006 Cohort (baseline), The Glostrup Population Studies | Subnational | urban | 18-71 | 18-71 | 1,553 | 1,916 | |
| 813 | Denmark | 2007 | Copenhagen General Population Study 1 | Subnational | urban | 20+ | 20+ | 4,027 | 6,913 | |
| 814 | Denmark | 2007 | The Danish Conscription Database | National | both | 17-26 | | 27,194 | | |
| 815 | Denmark | 2007-2008 | The Danish Health Examination Survey 2007-2008 | National | both | 18+ | 18+ | 7,349 | 10,651 | |
| 816 | Denmark | 2008 | Copenhagen General Population Study 1 | Subnational | urban | 20+ | 20+ | 4,735 | 6,467 | |
| 817 | Denmark | 2008 | The Childhood Health Activity and Motor Performance School Study | Community | both | 5-10 | 5-10 | 544 | 626 | |
| 818 | Denmark | 2008 | The Danish Conscription Database | National | both | 17-26 | | 24,538 | | |
| 819 | Denmark | 2008 | The Copenhagen School Child Intervention Study | Community | urban | 12-14 | 12-14 | 99 | 111 | |
| 820 | Denmark | 2009 | Copenhagen General Population Study 1 | Subnational | urban | 20+ | 20+ | 4,214 | 5,244 | |
| 821 | Denmark | 2009 | The Childhood Health Activity and Motor Performance School Study - Fall | Community | both | 6-12 | 6-12 | 240 | 246 | |
| 822 | Denmark | 2009 | The Childhood Health Activity and Motor Performance School Study - Spring | Community | both | 6-11 | 6-11 | 231 | 243 | |
| 823 | Denmark | 2009 | The Danish Conscription Database | National | both | 17-26 | | 27,093 | | |
| 824 | Denmark | 2009-2010 | European Youth Heart Studyy | Community | both | 14-28 | 14-28 | 481 | 553 | |
| 825 | Denmark | 2010 | Copenhagen General Population Study 1 | Subnational | urban | 20+ | 20+ | 3,967 | 4,891 | |
| 826 | Denmark | 2010 | The Childhood Health Activity and Motor Performance School Study - Fall | Community | both | 7-13 | 7-13 | 254 | 248 | |
| 827 | Denmark | 2010 | The Childhood Health Activity and Motor Performance School Study - Spring | Community | both | 7-12 | 7-12 | 234 | 235 | |
| 828 | Denmark | 2010 | The Danish Conscription Database | National | both | 17-26 | | 30,814 | | |

| | Country | Study years | Survey/Study name/Citation | Level of representative-ness | Rural, urban, or both | Age range as in NCD-RisC database | | Sample size | | Notes |
|---|---|---|---|---|---|---|---|---|---|---|
| | | | | | | Male | Female | Male | Female | |
| 829 | Denmark | 2011 | Copenhagen General Population Study 1 | Subnational | urban | 20+ | 20+ | 4,935 | 5,824 | |
| 830 | Denmark | 2011 | The Childhood Health Activity and Motor Performance School Study | Community | both | 8-13 | 8-13 | 245 | 241 | |
| 831 | Denmark | 2011 | The Danish Conscription Database | National | both | 17-26 | | 30,719 | | |
| 832 | Denmark | 2011-2012 | The Health2006 Cohort (5-year follow-up), The Glostrup Population Studies | Subnational | urban | 24-76 | 24-76 | 1,057 | 1,249 | |
| 833 | Denmark | 2011-2012 | The OPUS School Meal Study | Subnational | both | 8-11 | 8-11 | 427 | 388 | |
| 834 | Denmark | 2012 | Copenhagen General Population Study 1 | Subnational | urban | 20+ | 20+ | 4,516 | 5,423 | |
| 835 | Denmark | 2012 | The Childhood Health Activity and Motor Performance School Study | Community | both | 9-14 | 9-14 | 273 | 249 | |
| 836 | Denmark | 2012 | The Danish Conscription Database | National | both | 17-26 | | 29,651 | | |
| 837 | Denmark | 2012-2015 | DanFunD (baseline), The Glostrup Population Studies | Subnational | urban | 18-72 | 18-72 | 3,451 | 4,034 | |
| 838 | Denmark | 2013 | Copenhagen General Population Study 1 | Subnational | urban | 20+ | 20+ | 4,001 | 4,703 | |
| 839 | Denmark | 2013 | The Childhood Health Activity and Motor Performance School Study | Community | both | 10-15 | 10-15 | 225 | 208 | |
| 840 | Denmark | 2013 | The Danish Conscription Database | National | both | 17-26 | | 30,565 | | |
| 841 | Denmark | 2013 | Learning, Cognition and Motion (LCoMotion) | Subnational | both | 11-14 | 11-14 | 353 | 365 | |
| 842 | Denmark | 2014 | Copenhagen General Population Study 2 | Subnational | urban | 20+ | 20+ | 1,391 | 1,782 | |
| 843 | Denmark | 2014 | The Danish Conscription Database | National | both | 17-26 | | 32,397 | | |
| 844 | Denmark | 2014-2015 | Copenhagen General Population Study 1 | Subnational | urban | 20+ | 20+ | 2,245 | 2,837 | |
| 845 | Denmark | 2015 | Copenhagen General Population Study 2 | Subnational | urban | 20+ | 20+ | 4,103 | 5,107 | |
| 846 | Denmark | 2015 | The Childhood Health Activity and Motor Performance School Study | Community | both | 12-17 | 12-17 | 122 | 120 | |
| 847 | Denmark | 2015 | The Danish Conscription Database | National | both | 17-26 | | 28,907 | | |
| 848 | Denmark | 2015-2016 | Childhood Obesity Surveillance Initiative 4 | National | both | 6-7 | 6-7 | 1,339 | 1,281 | |
| 849 | Denmark | 2016 | Copenhagen General Population Study 2 | Subnational | urban | 20+ | 20+ | 4,493 | 5,495 | |
| 850 | Denmark | 2016 | The Danish Conscription Database | National | both | 17-29 | | 29,057 | | |
| 851 | Denmark | 2017 | Copenhagen General Population Study 2 | Subnational | urban | 20+ | 20+ | 3,182 | 4,395 | |
| 852 | Denmark | 2017 | The Danish Conscription Database | National | both | 17-29 | | 31,057 | | |
| 853 | Denmark | 2018 | The Danish Conscription Database | National | both | 17-29 | | 27,597 | | |
| 854 | Denmark | 2019 | The Danish Conscription Database | National | both | 17-29 | | 25,412 | | |
| 855 | Denmark | 2019 | Childhood Obesity Surveillance Initiative 5 | National | both | 6-7 | 6-7 | 1,204 | 1,291 | |
| 856 | Dominica | 2007-2008 | STEPS | National | both | 15-64 | 15-64 | 459 | 568 | |
| 857 | Dominica | 2009 | Global School-based Student Health Survey | National | both | 13-17 | 13-17 | 508 | 542 | |
| 858 | Dominican Republic | 1991 | DHS | National | both | | 20-49 | | 1,965 | |
| 859 | Dominican Republic | 1993 | Aono et al., J Epidemiol 7(4):238-43, 1997 | National | both | 20-70 | 20-70 | 767 | 1,149 | |
| 860 | Dominican Republic | 1996 | DHS | National | both | | 15-49 | | 7,441 | |
| 861 | Dominican Republic | 1996-1998 | Estudio factores de riesgo cardiovascular y sindrome metabolico en la Republica Dominicana I (EFRICARD I) | National | both | 18-75 | 18-75 | 2,087 | 4,095 | |
| 862 | Dominican Republic | 2010-2012 | Estudio factores de riesgo cardiovascular y sindrome metabolico en la Republica Dominicana II (EFRICARD II) | National | both | 18-75 | 18-75 | 1,599 | 3,180 | |
| 863 | Dominican Republic | 2013 | DHS | National | both | 15-59 | 15-49 | 10,433 | 8,960 | |
| 864 | Dominican Republic | 2017 | Prevalencia de hta y factores de riesgo en La Republica Dominicana al 2017 (ENPREFAR HAS 17) | National | both | 18+ | 18+ | 1,001 | 1,008 | |
| 865 | DR Congo | 2001 | Multiple Indicator Cluster Survey 2 | National | both | | 15-49 | | 5,520 | |
| 866 | DR Congo | 2005 | STEPS | Subnational | urban | 15+ | 15+ | 761 | 1,152 | |
| 867 | DR Congo | 2007 | DHS | National | both | | 15-49 | | 4,137 | |
| 868 | DR Congo | 2007 | Diabetes and intermediate hyperglycaemia in Kisantu, DR Congo: a cross-sectional prevalence study | Community | urban | 20+ | 20+ | 653 | 1,199 | |
| 869 | DR Congo | 2008 | Visite de la Tension Artérielle et des Facteurs de Risque Associés en Afrique subsaharienne (VITARAA) - Sud-Kivu, RD Congo | Community | both | 20+ | 20+ | 246 | 408 | |
| 870 | DR Congo | 2008 | Visite de la Tension Artérielle et des Facteurs de Risque Associés en Afrique subsaharienne (VITARAA) - Kinshasa, RD Congo | Community | urban | 10+ | 10+ | 810 | 979 | |
| 871 | DR Congo | 2011-2012 | Epidemiology of dementia in Central Africa: Brazzaville | Community | urban | 65+ | 65+ | 171 | 262 | |
| 872 | DR Congo | 2011-2012 | Epidemiology of dementia in Central Africa: Gamboma | Community | rural | 65+ | 65+ | 187 | 299 | |
| 873 | DR Congo | 2013-2014 | DHS | National | both | | 15-49 | | 8,163 | |
| 874 | DR Congo | 2016-2017 | Prevalence and Risk Factors of CKD in South Kivu, Democratic Republic of Congo: A Large-Scale Population Study | Subnational | both | 18+ | 18+ | 499 | 791 | |
| 875 | Ecuador | 2004 | Encuesta Demografica y de Salud Materno e Infantil/ Reproductive Health Survey | National | both | | 15-49 | | 3,850 | |
| 876 | Ecuador | 2004-2005 | CArdiovascular Risk factors Multiple Evaluation in Latin America (CARMELA) | Community | urban | 25-64 | 25-64 | 813 | 814 | |
| 877 | Ecuador | 2008-2009 | Food Nutrition and Health | Community | both | 10-16 | 10-16 | 379 | 375 | |
| 878 | Ecuador | 2009-2010 | The Survey on Health, Well-Being, and Aging in Latin America and the Caribbean (SABE) | National | both | 60+ | 60+ | 2,341 | 2,595 | |
| 879 | Ecuador | 2011-2013 | Encuesta Nacional de Salud y Nutrición (ENSANUT) | National | both | 5-59 | 5-59 | 22,919 | 25,767 | |
| 880 | Ecuador | 2014-2015 | Latin American Study of Nutrition and Health (ELANS) | National | urban | 15-65 | 15-65 | 517 | 586 | |

| | Country | Study years | Survey/Study name/Citation | Level of representative-ness | Rural, urban, or both | Age range as in NCD-RisC database | | Sample size | | Notes |
|---|---|---|---|---|---|---|---|---|---|---|
| | | | | | | Male | Female | Male | Female | |
| 881 | Ecuador | 2018 | Encuesta Nacional de Salud y Nutrición (ENSANUT) | National | both | 5+ | 5+ | 63,176 | 65,597 | |
| 882 | Ecuador | 2018 | STEPS | National | both | 18-69 | 18-69 | 1,893 | 2,579 | |
| 883 | Egypt | 1992 | DHS | National | both | | 20-49 | | 4,654 | |
| 884 | Egypt | 1995 | DHS | National | both | | 20-49 | | 6,499 | |
| 885 | Egypt | 2000 | DHS | National | both | | 20-49 | | 13,602 | |
| 886 | Egypt | 2002 | National Survey of Smoking, Obesity, Blood Pressure and Blood Glucose | National | both | 6+ | 6+ | 4,397 | 5,161 | |
| 887 | Egypt | 2003 | DHS | National | both | | 20-49 | | 7,930 | |
| 888 | Egypt | 2003-2004 | Marzouk et al., Gut 56(8):1105-10, 2007 | Community | rural | 25+ | 25+ | 322 | 456 | |
| 889 | Egypt | 2005 | DHS | National | both | | 20-49 | | 16,864 | |
| 890 | Egypt | 2005 | STEPS | National | both | 15-65 | 15-65 | 4,757 | 4,428 | |
| 891 | Egypt | 2007-2009 | Mostafa et al., Gut 59(8):1135-40, 2010 | Community | rural | 35+ | 35+ | 642 | 843 | |
| 892 | Egypt | 2008 | DHS | National | both | 10-59 | 20-49 | 14,261 | 15,242 | |
| 893 | Egypt | 2011 | Global School-based Student Health Survey | National | both | 13-17 | 13-17 | 325 | 454 | |
| 894 | Egypt | 2011 | STEPS | National | both | 15-65 | 15-65 | 1,761 | 2,977 | |
| 895 | Egypt | 2014 | DHS | National | both | | 20-49 | | 18,891 | |
| 896 | Egypt | 2015 | DHS | National | both | 15-59 | 15-59 | 7,235 | 8,471 | |
| 897 | Egypt | 2017 | STEPS | National | both | 15-69 | 15-69 | 2,273 | 3,692 | |
| 898 | El Salvador | 2002-2003 | Encuesta Nacional de Salud Familiar | National | both | | 15-49 | | 3,885 | |
| 899 | El Salvador | 2004 | CAMDI | Community | urban | 20+ | 20+ | 396 | 811 | |
| 900 | El Salvador | 2008 | Encuesta Nacional de Salud Familiar | National | both | | 15-49 | | 6,808 | |
| 901 | El Salvador | 2013 | Global School-based Student Health Survey | National | both | | 13-15 | | 675 | |
| 902 | El Salvador | 2014-2015 | Encuesta Nacional de Enfermedades Crónicas (ENECA-ELS) | National | both | 20+ | 20+ | 1,684 | 2,945 | |
| 903 | El Salvador | 2016 | National height and weight census | National | both | 6-8 | 6-8 | 56,034 | 53,321 | |
| 904 | Equatorial Guinea | 2011 | DHS | National | both | | 15-49 | | 1,074 | |
| 905 | Eritrea | 1995 | DHS | National | both | | 15-49 | | 1,621 | |
| 906 | Eritrea | 2002 | DHS | National | both | | 15-49 | | 3,223 | |
| 907 | Eritrea | 2004 | STEPS | National | both | 15-64 | 15-64 | 1,113 | 1,089 | |
| 908 | Eritrea | 2010 | STEPS | National | both | 25-74 | 25-74 | 1,712 | 4,285 | |
| 909 | Estonia | 1984-1986 | Abina et al., Blood Press 12:111-21, 2003 | Community | urban | 20-54 | 30-54 | 2,477 | 851 | |
| 910 | Estonia | 1992-1994 | Abina et al., Blood Press 12:111-21, 2003 | Community | urban | 20-54 | 20-54 | 921 | 678 | |
| 911 | Estonia | 1997 | Pomerleau et al., Public Health Nutrition 3(1):3-10, 2000 | National | both | 19-64 | 19-64 | 525 | 629 | |
| 912 | Estonia | 1999-2001 | Abina et al., Blood Press 12:111-21, 2003 | Community | urban | 20-54 | 20-54 | 635 | 692 | |
| 913 | Estonia | 2002 | Estonian Biobank | National | both | 18+ | 18+ | 89 | 217 | |
| 914 | Estonia | 2003 | Estonian Biobank | National | both | 18+ | 18+ | 2,695 | 5,688 | |
| 915 | Estonia | 2003 | The European Male Ageing Study | Community | both | 40+ | | 416 | | |
| 916 | Estonia | 2004 | Estonian Biobank | National | both | 18+ | 18+ | 527 | 947 | |
| 917 | Estonia | 2007 | Estonian Biobank | National | both | 18+ | 18+ | 1,000 | 2,187 | |
| 918 | Estonia | 2007-2010 | Identification and prevention of Dietary- and lifestyle-induced health EFfects In Children and infantS (IDEFICS) | Community | urban | 5-9 | 5-9 | 558 | 634 | |
| 919 | Estonia | 2008 | Estonian Biobank | National | both | 18+ | 18+ | 5,147 | 10,990 | |
| 920 | Estonia | 2008 | The European Male Ageing Study | Community | both | 40+ | | 305 | | |
| 921 | Estonia | 2009 | Estonian Biobank | National | both | 18+ | 18+ | 3,963 | 6,493 | |
| 922 | Estonia | 2010 | Estonian Biobank | National | both | 18+ | 18+ | 4,052 | 7,045 | |
| 923 | Estonia | 2013-2015 | National Dietary Survey (RTU) 2014 | National | both | 5-74 | 5-74 | 1,260 | 2,202 | |
| 924 | Estonia | 2015-2016 | Childhood Obesity Surveillance Initiative 4 | National | both | 7-8 | 7-8 | 6,502 | 6,198 | |
| 925 | Estonia | 2018-2019 | Childhood Obesity Surveillance Initiative 5 | National | both | 7-11 | 7-11 | 6,066 | 6,038 | |
| 926 | Estonia | 2022 | Childhood Obesity Surveillance Initiative 6 | National | both | 7-14 | 7-14 | 9,177 | 8,792 | |
| 927 | Eswatini | 2006-2007 | DHS | National | both | 15-49 | 15-49 | 4,074 | 4,714 | |
| 928 | Eswatini | 2014 | STEPS | National | both | 15-69 | 15-69 | 1,102 | 1,976 | |
| 929 | Ethiopia | 2000 | DHS | National | both | | 15-49 | | 13,912 | |
| 930 | Ethiopia | 2005 | DHS | National | both | | 15-49 | | 6,133 | |
| 931 | Ethiopia | 2006 | STEPS | Subnational | urban | 25-64 | 25-64 | 1,642 | 2,295 | |
| 932 | Ethiopia | 2011 | DHS | National | both | 15-59 | 15-49 | 14,329 | 15,111 | |
| 933 | Ethiopia | 2015 | National Micronutrient Survey | National | both | 5-54 | 5-49 | 1,187 | 2,459 | |
| 934 | Ethiopia | 2015 | STEPS | National | both | 15-69 | 15-69 | 3,912 | 5,369 | |
| 935 | Ethiopia | 2016 | ARISE Network Adolescent Health Study (Harar) | Community | urban | 10-19 | 10-19 | 499 | 556 | |
| 936 | Ethiopia | 2016 | ARISE Network Adolescent Health Study (Kersa) | Community | both | 10-19 | 10-19 | 526 | 418 | |

| | Country | Study years | Survey/Study name/Citation | Level of representative-ness | Rural, urban, or both | Age range as in NCD-RisC database | | Sample size | | Notes |
|---|---|---|---|---|---|---|---|---|---|---|
| | | | | | | Male | Female | Male | Female | |
| 937 | Ethiopia | 2016 | DHS | National | both | 15-59 | 15-49 | 12,380 | 14,104 | |
| 938 | Ethiopia | 2018 | Sustainable Urban Diets (SUDS) Addis Ababa | Community | urban | | 18-49 | | 994 | |
| 939 | Ethiopia | 2018 | Sustainable Urban Diets (SUDS) Kersa | Community | rural | | 18-49 | | 1,015 | |
| 940 | Fiji | 1980 | National Cardiovascular and Diabetes Survey (NCVDS) | Subnational | both | 20+ | 20+ | 1,448 | 1,522 | |
| 941 | Fiji | 2002 | STEPS | National | both | 15-64 | 15-64 | 2,684 | 3,820 | |
| 942 | Fiji | 2005-2007 | Pacific Obesity Prevention in Communities - Healthy Youth Health Communities Study | Subnational | urban | 11-19 | 11-19 | 3,730 | 4,109 | |
| 943 | Fiji | 2007-2008 | Pacific Obesity Prevention in Communities - Healthy Youth Health Communities Study | Subnational | urban | 13-22 | 13-22 | 1,492 | 1,832 | |
| 944 | Fiji | 2009 | Fiji Eye Health Survey 2009 | National | both | 40+ | 40+ | 582 | 776 | |
| 945 | Fiji | 2011 | STEPS | National | both | 25-64 | 25-64 | 1,123 | 1,417 | |
| 946 | Fiji | 2016 | Global School-based Student Health Survey | National | both | 13-17 | 13-17 | 1,394 | 1,469 | |
| 947 | Finland | 1980 | Young Finns Study 1980 | National | rural | 5-18 | 5-18 | 752 | 814 | |
| 948 | Finland | 1980 | Young Finns Study 1980 | National | urban | 5-18 | 5-18 | 704 | 727 | |
| 949 | Finland | 1982 | MONICA, North Karelia/Kuopio/Turku/Loimaa | Subnational | both | 25-64 | 25-64 | 4,550 | 4,659 | |
| 950 | Finland | 1983 | Young Finns Study 1983 | National | rural | 6-21 | 6-21 | 727 | 773 | |
| 951 | Finland | 1983 | Young Finns Study 1983 | National | urban | 6-21 | 6-21 | 656 | 685 | |
| 952 | Finland | 1984 | Finland, Italy, Netherlands, Elderly (Fine-Finland) | Community | rural | 65-84 | | 673 | | |
| 953 | Finland | 1984-1989 | Kuopio Ischaemic Heart Disease Risk Factor Study | Subnational | both | 42-61 | | 2,672 | | |
| 954 | Finland | 1985 | INTERSALT, Turku | Community | urban | 20-59 | 20-59 | 100 | 100 | |
| 955 | Finland | 1985-1986 | INTERSALT, Joensuu | Community | urban | 20-59 | 20-59 | 100 | 100 | |
| 956 | Finland | 1986 | Young Finns Study 1986 | National | rural | 9-24 | 9-24 | 594 | 631 | |
| 957 | Finland | 1986 | Young Finns Study 1986 | National | urban | 9-24 | 9-24 | 587 | 666 | |
| 958 | Finland | 1987 | MONICA, North Karelia/Kuopio/Turku/Loimaa | Subnational | both | 25-64 | 25-64 | 2,896 | 3,151 | |
| 959 | Finland | 1989 | Finland, Italy, Netherlands, Elderly (Fine-Finland) | Community | rural | 70-89 | | 446 | | |
| 960 | Finland | 1990-1992 | Oulu 35 Study | Community | urban | 56-57 | 56-57 | 231 | 326 | |
| 961 | Finland | 1991-1993 | Kuopio Ischaemic Heart Disease Risk Factor Study | Subnational | both | 46-65 | | 1,037 | | |
| 962 | Finland | 1992 | The National FINRISK Study | Subnational | both | 25-64 | 25-64 | 2,849 | 3,201 | |
| 963 | Finland | 1994 | Finland, Italy, Netherlands, Elderly (Fine-Finland) | Community | rural | 75-94 | | 266 | | |
| 964 | Finland | 1996-1998 | Oulu 35 Study | Community | urban | 60-63 | 60-63 | 242 | 345 | |
| 965 | Finland | 1996-1998 | Savitaipale Study, Baseline | Community | rural | 40-66 | 40-66 | 574 | 574 | |
| 966 | Finland | 1997 | The National FINRISK Study | National | both | 25-74 | 25-74 | 4,128 | 4,131 | |
| 967 | Finland | 1997 | Northern Finland Birth Cohort 1966 | Community | both | 30-31 | 30-31 | 2,614 | 141 | |
| 968 | Finland | 1998-2001 | Kuopio Ischaemic Heart Disease Risk Factor Study | Subnational | both | 53-73 | 53-73 | 834 | 919 | |
| 969 | Finland | 2000 | Finland, Italy, Netherlands, Elderly (Fine-Finland) | Community | rural | 81-96 | | 92 | | |
| 970 | Finland | 2000-2001 | Health 2000 Survey | National | both | 30+ | 30+ | 2,663 | 3,222 | |
| 971 | Finland | 2001 | Young Finns Study 2001 | National | rural | 24-39 | 24-39 | 346 | 393 | |
| 972 | Finland | 2001 | Young Finns Study 2001 | National | urban | 24-39 | 24-39 | 658 | 769 | |
| 973 | Finland | 2001-2002 | Northern Finland Birth Cohort 1986 | Community | both | 15-17 | 15-17 | 3,126 | 3,194 | |
| 974 | Finland | 2001-2003 | Oulu 45 Study | Community | urban | 55-58 | 55-58 | 426 | 550 | |
| 975 | Finland | 2001-2004 | Helsinki Birth Cohort Study | Community | urban | 56-69 | 56-69 | 927 | 1,074 | |
| 976 | Finland | 2002 | The National FINRISK Study | National | both | 25-74 | 25-74 | 3,299 | 3,826 | |
| 977 | Finland | 2004-2005 | FIN-D2D | Subnational | both | 45-74 | 45-74 | 1,364 | 1,461 | |
| 978 | Finland | 2005 | Mantyselka et al., Rheumatology (Oxford) 47(8):1235-38, 2008 | Community | rural | 30-65 | 30-65 | 230 | 241 | |
| 979 | Finland | 2005-2008 | Kuopio Ischaemic Heart Disease Risk Factor Study | Subnational | both | 60-81 | 60-81 | 1,241 | 634 | |
| 980 | Finland | 2007 | The National FINRISK Study | National | both | 25-74 | 25-74 | 2,934 | 3,323 | |
| 981 | Finland | 2007 | Oulu 35 Study | Community | urban | 71-73 | 71-73 | 182 | 271 | |
| 982 | Finland | 2007 | Young Finns Study 2007 | National | rural | 30-45 | 30-45 | 374 | 431 | |
| 983 | Finland | 2007 | Young Finns Study 2007 | National | urban | 30-45 | 30-45 | 602 | 714 | |
| 984 | Finland | 2007-2008 | Savitaipale Study, 10-year Follow-up | Community | rural | 51-75 | 51-75 | 430 | 483 | |
| 985 | Finland | 2008 | Control group for Finnish male former elite athletes | National | both | 61+ | | 206 | | |
| 986 | Finland | 2011 | Young Finns Study 2011 | National | rural | 34-49 | 34-49 | 364 | 424 | |
| 987 | Finland | 2011 | Young Finns Study 2011 | National | urban | 34-49 | 34-49 | 506 | 636 | |
| 988 | Finland | 2011-2012 | Health 2011 Survey | National | both | 30+ | 30+ | 2,041 | 2,535 | |
| 989 | Finland | 2011-2013 | International Study of Childhood Obesity, Lifestyle and the Environment (ISCOLE) | Community | urban | 9-11 | 9-11 | 253 | 282 | |
| 990 | Finland | 2012 | The National FINRISK Study | National | both | 25-74 | 25-74 | 2,774 | 3,052 | |
| 991 | Finland | 2012 | Northern Finland Birth Cohort 1966 | Community | both | 45-47 | 45-47 | 2,362 | 2,966 | |
| 992 | Finland | 2016 | Register of Primary Health Care visits | National | rural | 5-17 | 5-17 | 37,441 | 35,048 | |
| 993 | Finland | 2016 | Register of Primary Health Care visits | National | urban | 5-17 | 5-17 | 92,177 | 87,911 | |

| | Country | Study years | Survey/Study name/Citation | Level of representative-ness | Rural, urban, or both | Age range as in NCD-RisC database | | Sample size | | Notes |
|---|---|---|---|---|---|---|---|---|---|---|
| | | | | | | Male | Female | Male | Female | |
| 994 | Finland | 2017 | The FinHealth Survey | National | both | 18+ | 18+ | 2,700 | 3,136 | |
| 995 | Finland | 2017 | Register of Primary Health Care visits | National | rural | 5-17 | 5-17 | 41,744 | 38,890 | |
| 996 | Finland | 2017 | Register of Primary Health Care visits | National | urban | 5-17 | 5-17 | 106,968 | 100,998 | |
| 997 | Finland | 2018 | Register of Primary Health Care visits | National | rural | 5-17 | 5-17 | 44,828 | 41,905 | |
| 998 | Finland | 2018 | Register of Primary Health Care visits | National | urban | 5-17 | 5-17 | 112,753 | 106,296 | |
| 999 | Finland | 2018-2019 | Savitaipale Study, 22-year Follow-up | Community | rural | 62-86 | 62-86 | 263 | 341 | |
| 1000 | Finland | 2019 | Childhood Obesity Surveillance Initiative 5 | National | both | 7-9 | 7-9 | 5,749 | 5,096 | |
| 1001 | Finland | 2019-2020 | Northern Finland Birth Cohort 1986 | Community | both | 33-35 | 33-35 | 579 | 916 | |
| 1002 | France | 1985-1987 | MONICA, Strasbourg | Subnational | both | 35-64 | 35-64 | 664 | 713 | |
| 1003 | France | 1985-1987 | MONICA, Strasbourg | Subnational | both | 25-34 | 25-34 | 65 | 78 | |
| 1004 | France | 1985-1987 | MONICA, Toulouse | Subnational | both | 35-64 | 35-64 | 675 | 644 | |
| 1005 | France | 1986-1989 | MONICA, Lille | Community | urban | 25-64 | 25-64 | 878 | 732 | |
| 1006 | France | 1988-1991 | MONICA, Toulouse | Subnational | both | 35-64 | | 586 | | |
| 1007 | France | 1994-1996 | MONICA, Toulouse | Subnational | both | 35-64 | 35-64 | 608 | 566 | |
| 1008 | France | 1995-1997 | MONICA, Lille | Community | urban | 36-67 | 36-67 | 598 | 590 | |
| 1009 | France | 1995-1997 | MONICA, Strasbourg | Subnational | both | 35-64 | 35-64 | 526 | 523 | |
| 1010 | France | 1996-2003 | Jaquet et al., Diabetologia 48(5):849-55, 2005 | Community | urban | 15-34 | 15-34 | 173 | 164 | |
| 1011 | France | 1999-2001 | The Three City Study | Community | urban | 65+ | 65+ | 2,423 | 3,778 | |
| 1012 | France | 2000 | Corpulence 7-9 ans | Subnational | both | 7-9 | 7-9 | 786 | 796 | |
| 1013 | France | 2004-2006 | National Monitoring of Arterial Risk in Lille (MONA LISA Lille) | Subnational | urban | 35-75 | 35-75 | 783 | 795 | |
| 1014 | France | 2005-2007 | Etude individuelle nationale des consommations alimentaires 2 (INCA2), adults | National | both | 18-79 | 18-79 | 1,001 | 1,368 | |
| 1015 | France | 2005-2007 | Etude individuelle nationale des consommations alimentaires 2 (INCA2), children and adolescents | National | both | 5-17 | 5-17 | 607 | 684 | |
| 1016 | France | 2005-2007 | National Monitoring of Arterial Risk in Bas-Rhin (MONA LISA Bas-Rhin) | Subnational | both | 35-74 | 35-74 | 780 | 787 | |
| 1017 | France | 2005-2007 | National Monitoring of Arterial Risk in Toulouse (MONA LISA Toulouse) | Subnational | both | 35-74 | 35-74 | 829 | 796 | |
| 1018 | France | 2006-2007 | Etude Nationale Nutrition Santé | National | both | 5-74 | 5-74 | 1,505 | 2,030 | 8 |
| 1019 | France | 2006-2007 | HELENA | Community | urban | 12-17 | 12-17 | 122 | 165 | |
| 1020 | France | 2006-2008 | The Three City Study | Community | urban | 72+ | 72+ | 768 | 1,217 | |
| 1021 | France | 2007 | Corpulence 7-9 ans | National | both | 7-9 | 7-9 | 1,281 | 1,244 | |
| 1022 | France | 2008-2011 | Study of pre- and early postnatal determinants of child health and development (EDEN) | Community | both | 5 | 5 | 639 | 569 | |
| 1023 | France | 2009-2012 | Study of pre- and early postnatal determinants of child health and development (EDEN) | Community | both | 6 | 6 | 306 | 283 | |
| 1024 | France | 2010-2013 | Study of pre- and early postnatal determinants of child health and development (EDEN) | Community | both | 7 | 7 | 339 | 320 | |
| 1025 | France | 2011-2013 | Enquête LIttorale Souffle Air Biologie EnvironnemenT (ELISABET) Dunkerque | Community | urban | 40-64 | 40-64 | 750 | 779 | |
| 1026 | France | 2011-2013 | Enquête LIttorale Souffle Air Biologie EnvironnemenT (ELISABET) Lille | Community | urban | 40-64 | 40-64 | 753 | 838 | |
| 1027 | France | 2011-2014 | Study of pre- and early postnatal determinants of child health and development (EDEN) | Community | both | 8 | 8 | 269 | 236 | |
| 1028 | France | 2012-2014 | Cohorte des consultants des Centres d'examens de santé (CONSTANCES) | National | urban | 18-69 | 18-69 | 22,515 | 26,317 | |
| 1029 | France | 2012-2015 | Study of pre- and early postnatal determinants of child health and development (EDEN) | Community | both | 9 | 9 | 86 | 79 | |
| 1030 | France | 2013-2016 | Study of pre- and early postnatal determinants of child health and development (EDEN) | Community | both | 10 | 10 | 193 | 178 | |
| 1031 | France | 2014-2016 | L'Etude de Sante sur l'Environnement, la Biosurveillance, l'Activite physique et la Nutrition (Etude Esteban) | National | both | 6-74 | 6-74 | 1,662 | 1,913 | 9 |
| 1032 | France | 2014-2017 | Study of pre- and early postnatal determinants of child health and development (EDEN) | Community | both | 11 | 11 | 246 | 230 | |
| 1033 | France | 2015-2016 | Childhood Obesity Surveillance Initiative 4 | National | both | 7-9 | 7-9 | 2,510 | 2,561 | |
| 1034 | France | 2015-2017 | Cohorte des consultants des Centres d'examens de santé (CONSTANCES) | National | urban | 18-69 | 18-69 | 45,102 | 51,410 | |
| 1035 | France | 2017-2019 | Cohorte des consultants des Centres d'examens de santé (CONSTANCES ), follow up | Subnational | urban | 22-76 | 22-76 | 10,443 | 11,334 | |
| 1036 | France | 2018-2019 | Cohorte des consultants des Centres d'examens de santé (CONSTANCES) | National | urban | 18-69 | 18-69 | 23,891 | 27,546 | |
| 1037 | France | 2020-2021 | Cohorte des consultants des Centres d'examens de santé (CONSTANCES) | Subnational | urban | 18-69 | 18-69 | 1,467 | 1,813 | |
| 1038 | France | 2020-2023 | Cohorte des consultants des Centres d'examens de santé (CONSTANCES), follow up | Subnational | urban | 22-80 | 22-80 | 22,053 | 23,119 | |
| 1039 | French Polynesia | 2010 | STEPS | National | both | 18-64 | 18-64 | 1,458 | 1,916 | |
| 1040 | French Polynesia | 2015 | Global School-based Student Health Survey | National | both | 12-17 | 12-17 | 1,310 | 1,438 | |
| 1041 | Gabon | 2000 | DHS | National | both | | 20-49 | | 2,082 | |
| 1042 | Gabon | 2009 | STEPS | Subnational | urban | 15-64 | 15-64 | 1,051 | 1,515 | |
| 1043 | Gabon | 2012 | DHS | National | both | | 15-49 | | 5,066 | |
| 1044 | Gabon | 2019-2021 | DHS | National | both | | 15-64 | | 5,663 | |
| 1045 | Gambia | 1996-1997 | National Survey of Blindness and Low Vision | National | both | 16+ | 16+ | 1,733 | 2,071 | |
| 1046 | Gambia | 2003 | Siervo et al., Eur J Clin Nutr 60(4):455-63, 2006 | Community | urban | 14-50 | 14-50 | 50 | 50 | |
| 1047 | Gambia | 2010 | STEPS | National | both | 25-64 | 25-64 | 1,610 | 1,919 | |
| 1048 | Gambia | 2013 | DHS | National | both | | 15-49 | | 4,180 | |
| 1049 | Gambia | 2018 | The Gambia Micronutrient Survey (GMNS) | National | both | | 15-49 | | 1,640 | |
| 1050 | Gambia | 2019-2020 | DHS | National | both | | 15-49 | | 5,474 | |

| | Country | Study years | Survey/Study name/Citation | Level of representative-ness | Rural, urban, or both | Age range as in NCD-RisC database | | Sample size | | Notes |
|---|---|---|---|---|---|---|---|---|---|---|
| | | | | | | Male | Female | Male | Female | |
| 1051 | Georgia | 2010 | STEPS | National | both | 18-64 | 18-64 | 1,842 | 4,460 | |
| 1052 | Georgia | 2015-2016 | Childhood Obesity Surveillance Initiative 4 | National | both | 7-8 | 7-8 | 1,685 | 1,585 | |
| 1053 | Georgia | 2016 | STEPS | National | both | 18-69 | 18-69 | 1,188 | 2,784 | |
| 1054 | Georgia | 2019 | Childhood Obesity Surveillance Initiative 5 | National | both | 7-8 | 7-8 | 1,777 | 1,646 | |
| 1055 | Germany | 1982 | MONICA, Erfurt | Community | urban | 25-64 | 25-64 | 106 | 103 | |
| 1056 | Germany | 1982-1984 | MONICA, Chemnitz | Community | urban | 25-64 | 25-64 | 267 | 295 | |
| 1057 | Germany | 1982-1984 | MONICA, Zwickau | Community | urban | 25-64 | 25-64 | 246 | 276 | |
| 1058 | Germany | 1982-1985 | MONICA, Rest of Karl-Marx-Stadt County | Subnational | urban | 25-64 | 25-64 | 592 | 657 | |
| 1059 | Germany | 1982-1985 | MONICA, Rest of DDR-MONICA | Subnational | urban | 25-64 | 25-64 | 235 | 232 | |
| 1060 | Germany | 1983-1984 | MONICA, Halle County | Subnational | urban | 25-64 | 25-64 | 1,110 | 1,172 | |
| 1061 | Germany | 1983-1987 | MONICA, Rhein-Neckar Region | Community | urban | 25-64 | 25-64 | 1,489 | 1,609 | |
| 1062 | Germany | 1984 | The German Conscription Database | Subnational | both | 19 | | 419,719 | | |
| 1063 | Germany | 1984 | German Cardiovascular Prevention Study (GCP) - National Health Survey 1984 | Subnational | both | 25-69 | 25-69 | 2,415 | 2,366 | |
| 1064 | Germany | 1984 | MONICA, Bremen North/West | Community | urban | 25-64 | 25-64 | 813 | 852 | |
| 1065 | Germany | 1984-1985 | MONICA, Berlin-Lichtenberg | Community | urban | 25-64 | 25-64 | 593 | 635 | |
| 1066 | Germany | 1984-1985 | KORA-S1 (Cooperative Health Research in the Augsburg Region) - formerly MONICA Augsburg | Community | both | 24-65 | 24-65 | 2,006 | 1,963 | |
| 1067 | Germany | 1984-1986 | MONICA, Cottbus County | Community | urban | 25-64 | 25-64 | 657 | 739 | |
| 1068 | Germany | 1985 | The German Conscription Database | Subnational | both | 19 | | 402,487 | | |
| 1069 | Germany | 1985-1986 | INTERSALT, Cottbus | Community | urban | 20-59 | 20-59 | 99 | 99 | |
| 1070 | Germany | 1985-1986 | INTERSALT, Heidelberg | Community | urban | 20-59 | 20-59 | 97 | 99 | |
| 1071 | Germany | 1985-1986 | CINDI | Subnational | both | 25-64 | 25-64 | 1,875 | 1,990 | |
| 1072 | Germany | 1985-1986 | INTERSALT, Bernried | Community | urban | 20-59 | 20-59 | 99 | 98 | |
| 1073 | Germany | 1986 | The German Conscription Database | Subnational | both | 19 | | 382,632 | | |
| 1074 | Germany | 1987 | The German Conscription Database | Subnational | both | 19 | | 349,083 | | |
| 1075 | Germany | 1987-1988 | MONICA, Erfurt | Community | urban | 25-64 | 25-64 | 871 | 909 | |
| 1076 | Germany | 1988 | The German Conscription Database | Subnational | both | 19 | | 303,265 | | |
| 1077 | Germany | 1988 | German Cardiovascular Prevention Study (GCP) - National Health Survey 1988 | Subnational | both | 25-69 | 25-69 | 2,642 | 2,678 | |
| 1078 | Germany | 1988 | MONICA, Berlin-Lichtenberg | Community | urban | 25-64 | 25-64 | 690 | 728 | |
| 1079 | Germany | 1988 | MONICA, Bremen North/West | Community | urban | 25-69 | 25-69 | 619 | 632 | |
| 1080 | Germany | 1988 | MONICA, Bremen Center/South/East | Community | urban | 25-69 | 25-69 | 499 | 582 | |
| 1081 | Germany | 1988 | MONICA, Chemnitz | Community | urban | 25-64 | 25-64 | 288 | 382 | |
| 1082 | Germany | 1988 | MONICA, Zwickau | Community | urban | 25-64 | 25-64 | 193 | 250 | |
| 1083 | Germany | 1988-1989 | CINDI | Subnational | both | 25-64 | 25-64 | 1,361 | 1,435 | |
| 1084 | Germany | 1988-1989 | MONICA, Halle County | Subnational | urban | 25-64 | 25-64 | 959 | 1,201 | |
| 1085 | Germany | 1988-1989 | MONICA, Rest of Karl-Marx-Stadt County | Subnational | urban | 25-64 | 25-64 | 541 | 626 | |
| 1086 | Germany | 1989 | The German Conscription Database | Subnational | both | 19 | | 245,740 | | |
| 1087 | Germany | 1989-1990 | MONICA, Cottbus County | Community | urban | 25-64 | 25-64 | 539 | 529 | |
| 1088 | Germany | 1989-1990 | KORA-S2 (Cooperative Health Research in the Augsburg Region) - formerly MONICA Augsburg | Community | both | 24-75 | 24-75 | 2,439 | 2,374 | |
| 1089 | Germany | 1990 | The German Conscription Database | Subnational | both | 19 | | 206,599 | | |
| 1090 | Germany | 1990-1992 | European Community Respiratory Health Survey, Hamburg | Community | urban | 20-47 | 20-47 | 146 | 138 | |
| 1091 | Germany | 1990-1992 | European Community Respiratory Health Survey, Erfurt | Community | urban | 20-47 | 20-47 | 146 | 124 | |
| 1092 | Germany | 1991 | The German Conscription Database | National | both | 19 | | 138,195 | | |
| 1093 | Germany | 1991-1992 | MONICA, Bremen North/West | Community | urban | 25-69 | 25-69 | 599 | 671 | |
| 1094 | Germany | 1991-1992 | MONICA, Bremen Center/South/East | Community | urban | 25-69 | 25-69 | 524 | 546 | |
| 1095 | Germany | 1991-1992 | CINDI | Subnational | both | 25-64 | 25-64 | 1,326 | 1,400 | |
| 1096 | Germany | 1991-1992 | German Cardiovascular Prevention Study (GCP) - National Health Survey 1991 | Subnational | both | 25-69 | 25-69 | 2,599 | 2,670 | |
| 1097 | Germany | 1991-1992 | First National Examination of life conditions, Environment and Health in East Germany 1991/92 | Subnational | both | 25-69 | 25-69 | 1,042 | 1,155 | |
| 1098 | Germany | 1991-1992 | MONICA, Erfurt | Community | urban | 25-64 | 25-64 | 587 | 572 | |
| 1099 | Germany | 1992 | The German Conscription Database | National | both | 19 | | 220,956 | | |
| 1100 | Germany | 1993 | The German Conscription Database | National | both | 19 | | 188,655 | | |
| 1101 | Germany | 1993-1994 | MONICA, Chemnitz | Community | urban | 25-64 | 25-64 | 408 | 424 | |
| 1102 | Germany | 1993-1994 | MONICA, Zwickau | Community | urban | 25-64 | 25-64 | 139 | 186 | |
| 1103 | Germany | 1994 | The German Conscription Database | National | both | 19 | | 155,426 | | |
| 1104 | Germany | 1994-1995 | KORA-S3 (Cooperative Health Research in the Augsburg Region) - formerly MONICA Augsburg | Community | both | 24-75 | 24-75 | 2,358 | 2,372 | |
| 1105 | Germany | 1994-1998 | EPIC Heidelberg | Community | urban | 40-64 | 35-64 | 11,680 | 13,458 | |
| 1106 | Germany | 1994-1998 | EPIC Potsdam | Community | urban | 40-64 | 35-64 | 10,224 | 15,995 | |
| 1107 | Germany | 1995 | The German Conscription Database | National | both | 19 | | 185,762 | | |

| | Country | Study years | Survey/Study name/Citation | Level of representative-ness | Rural, urban, or both | Age range as in NCD-RisC database | | Sample size | | Notes |
|---|---|---|---|---|---|---|---|---|---|---|
| | | | | | | Male | Female | Male | Female | |
| 1108 | Germany | 1996 | The German Conscription Database | National | both | 19 | | 191,260 | | |
| 1109 | Germany | 1997 | The German Conscription Database | National | both | 19 | | 148,738 | | |
| 1110 | Germany | 1997-1999 | German National Health Interview and Examination Survey (GNHIES98) | National | both | 18-79 | 18-79 | 3,406 | 3,580 | |
| 1111 | Germany | 1997-2001 | Study of Health in Pomerania (SHIP-START-0) baseline study | Subnational | both | 20-80 | 20-80 | 2,109 | 2,168 | 10 |
| 1112 | Germany | 1998 | The German Conscription Database | National | both | 19 | | 146,528 | | |
| 1113 | Germany | 1999 | The German Conscription Database | National | both | 19 | | 292,732 | | |
| 1114 | Germany | 1999-2001 | KORA-S4 (Cooperative Health Research in the Augsburg Region) | Community | both | 24-75 | 24-75 | 2,071 | 2,138 | |
| 1115 | Germany | 2000-2001 | European Community Respiratory Health Survey, Hamburg | Community | urban | 30-57 | 30-57 | 146 | 138 | |
| 1116 | Germany | 2000-2001 | European Community Respiratory Health Survey, Erfurt | Community | urban | 30-57 | 30-57 | 146 | 124 | |
| 1117 | Germany | 2000-2002 | Epidemiological study of the chances of prevention, early recognition and optimal treatment of chronic diseases in an elderly population (ESTHER) | Subnational | both | 50-75 | 50-75 | 4,344 | 5,334 | |
| 1118 | Germany | 2000-2003 | Heinz Nixdorf Recall Study | Subnational | urban | 45-75 | 45-75 | 2,380 | 2,401 | 11 |
| 1119 | Germany | 2002 | Echinoccoccus Multilocularis and Internal Diseases in Leutkirch | Community | urban | 12-65 | 12-65 | 1,172 | 1,257 | |
| 1120 | Germany | 2002-2006 | Study of Health in Pomerania (SHIP-START-1) 5-year follow-up | Subnational | both | 25-85 | 25-85 | 1,578 | 1,691 | 10 |
| 1121 | Germany | 2003-2006 | German Health Interview and Examination Survey for Children and Adolescents (KiGGS) | National | both | 5-17 | 5-17 | 6,602 | 6,260 | |
| 1122 | Germany | 2005-2008 | Heinz Nixdorf Recall Study | Subnational | both | 50-80 | 50-80 | 2,044 | 2,099 | 11 |
| 1123 | Germany | 2006-2007 | HELENA | Community | urban | 12-17 | 12-17 | 282 | 194 | |
| 1124 | Germany | 2006-2008 | KORA-F4 (Cooperative Health Research in the Augsburg Region) - first follow up of KORA-S4 | Community | both | 31-82 | 31-82 | 1,478 | 1,580 | |
| 1125 | Germany | 2007-2010 | Identification and prevention of Dietary- and lifestyle-induced health EFfects In Children and infantS (IDEFICS) | Community | urban | 5-9 | 5-9 | 772 | 762 | |
| 1126 | Germany | 2007-2012 | Gutenberg Health Study | Community | urban | 35-74 | 35-74 | 7,577 | 7,419 | |
| 1127 | Germany | 2008 | The German Conscription Database | National | both | 19 | | 98,926 | | |
| 1128 | Germany | 2008-2011 | Epidemiological study of the chances of prevention, early recognition and optimal treatment of chronic diseases in an elderly population (ESTHER) | Subnational | both | 58-84 | 58-84 | 1,468 | 1,622 | |
| 1129 | Germany | 2008-2011 | German Health Interview and Examination Survey for adults 2008-11 (DEGS1) | National | both | 18-79 | 18-79 | 3,389 | 3,650 | |
| 1130 | Germany | 2008-2012 | Study of Health in Pomerania (SHIP-START-2) 11-year follow-up | Subnational | both | 31-81 | 31-81 | 1,049 | 1,201 | 10 |
| 1131 | Germany | 2008-2012 | Study of Health in Pomerania, second cohort (SHIP-TREND-0) | Subnational | both | 20-79 | 20-79 | 2,099 | 2,229 | 10 |
| 1132 | Germany | 2009 | The German Conscription Database | National | both | 19 | | 111,455 | | |
| 1133 | Germany | 2009-2013 | Join the Healthy Boat | Subnational | both | 5-9 | 5-9 | 837 | 827 | |
| 1134 | Germany | 2010 | The German Conscription Database | National | both | 19 | | 101,911 | | |
| 1135 | Germany | 2011-2014 | Heinz Nixdorf Recall Study | Subnational | both | 56-85 | 56-85 | 1,493 | 1,560 | 11 |
| 1136 | Germany | 2012-2017 | Gutenberg Health Study | Community | urban | 40-80 | 40-80 | 6,356 | 6,062 | |
| 1137 | Germany | 2013-2014 | KORA-FF4 (Cooperative Health Research in the Augsburg Region) - second follow up of KORA-S4 | Community | both | 38-88 | 38-88 | 1,099 | 1,176 | |
| 1138 | Germany | 2014-2016 | Study of Health in Pomerania (SHIP-START-3) 16-year follow-up | Subnational | both | 37-87 | 37-87 | 778 | 907 | 10 |
| 1139 | Germany | 2014-2017 | German Health Interview and Examination Survey for Children and Adolescents (KiGGS) - Wave 2 | National | both | 5-17 | 5-17 | 1,541 | 1,584 | |
| 1140 | Germany | 2016-2017 | Join the Healthy Boat | Subnational | both | 5-6 | 5-6 | 124 | 129 | |
| 1141 | Germany | 2016-2019 | Study of Health in Pomerania, second cohort (SHIP-TREND-1) 8-year follow-up | Subnational | both | 28-90 | 28-90 | 1,200 | 1,273 | 10 |
| 1142 | Germany | 2017-2021 | Gutenberg Health Study | Community | urban | 45+ | 45+ | 4,209 | 3,927 | |
| 1143 | Germany | 2018-2019 | Childhood Obesity Surveillance Initiative 5 - Bremen | Community | urban | 7-9 | 7-9 | 1,110 | 1,111 | |
| 1144 | Ghana | 1993 | DHS | National | both | | 20-49 | | 1,650 | |
| 1145 | Ghana | 1997 | Amoah et al., Ethn Dis 13(2 Suppl 2):S97-101, 2003 | Community | both | 25+ | 25+ | 1,857 | 2,875 | |
| 1146 | Ghana | 1998 | DHS | National | both | | 20-49 | | 1,979 | |
| 1147 | Ghana | 2001 | Addo et al., Ethn Dis 16(4):894-99, 2006 | Community | rural | 15+ | 15+ | 89 | 206 | |
| 1148 | Ghana | 2001-2002 | Cappuccio et al., Hypertension 43(5):1017-22, 2004 | Community | both | 35-84 | 35-84 | 194 | 338 | |
| 1149 | Ghana | 2002 | Amoah et al., Ethn Dis 13(2 Suppl 2):S97-101, 2003 | Community | both | 25+ | 25+ | 1,859 | 2,947 | |
| 1150 | Ghana | 2003 | Women's Health Study of Accra (WHSA-I) | Community | urban | | 18+ | | 1,184 | |
| 1151 | Ghana | 2003 | DHS | National | both | | 15-49 | | 4,935 | |
| 1152 | Ghana | 2006 | STEPS | Community | urban | 25+ | 25+ | 841 | 1,635 | |
| 1153 | Ghana | 2007-2008 | WHO Study on global AGEing and adult health (SAGE) | National | both | 50+ | 50+ | 2,192 | 1,987 | |
| 1154 | Ghana | 2008 | DHS | National | both | | 15-49 | | 4,455 | |
| 1155 | Ghana | 2008-2010 | Women's Health Study of Accra (WHSA-II) | Community | urban | | 18+ | | 2,677 | |
| 1156 | Ghana | 2012-2014 | Research on Obesity and Diabetes among African Migrants (RODAM), control group | Subnational | rural | 25+ | 25+ | 431 | 676 | |
| 1157 | Ghana | 2012-2014 | Research on Obesity and Diabetes among African Migrants (RODAM), control group | Subnational | urban | 25+ | 25+ | 418 | 1,034 | |
| 1158 | Ghana | 2014 | DHS | National | both | 15-59 | 15-49 | 4,293 | 4,393 | |
| 1159 | Ghana | 2016-2017 | Ghana Living Standards Survey | National | both | 5+ | 5+ | 18,888 | 20,977 | |
| 1160 | Ghana | 2017 | Ghana Micronutrient Survey | National | both | | 15-49 | | 1,001 | |

| | Country | Study years | Survey/Study name/Citation | Level of representative-ness | Rural, urban, or both | Age range as in NCD-RisC database | | Sample size | | Notes |
|---|---|---|---|---|---|---|---|---|---|---|
| | | | | | | Male | Female | Male | Female | |
| 1161 | Ghana | 2018 | Intervention to reduce cardiovascular disease risk factors among secondary school students: Randomised controlled trial - Baseline | Subnational | both | 14-19 | 14-19 | 411 | 437 | |
| 1162 | Ghana | 2018 | Intervention to reduce cardiovascular disease risk factors among secondary school students: Randomised controlled trial - Post-intervention study | Subnational | both | 14-19 | 14-19 | 199 | 218 | |
| 1163 | Ghana | 2022 | Global School-based Student Health Survey (Sekondi-Takoradi) | Community | urban | 13-16 | 13-16 | 1,429 | 1,700 | |
| 1164 | Greece | 1991 | Seven Countries Study | Subnational | both | 70-89 | | 177 | | |
| 1165 | Greece | 1997 | The Didima Study | Community | rural | 18+ | 18+ | 265 | 373 | |
| 1166 | Greece | 1997 | Greece Physical Fitness Study | National | both | 8 | 8 | 31,619 | 29,980 | |
| 1167 | Greece | 1997-2000 | Dietary criteria of adolescents: The role of physical activity, anthropometrics, dietetic, psychological and other factors | Community | urban | 12-18 | 12-18 | 494 | 505 | |
| 1168 | Greece | 1998 | Greece Physical Fitness Study | National | both | 8-10 | 8-10 | 33,370 | 32,057 | |
| 1169 | Greece | 1999 | Greece Physical Fitness Study | National | both | 8 | 8 | 30,090 | 28,570 | |
| 1170 | Greece | 2000 | Greece Physical Fitness Study | National | both | 8 | 8 | 32,466 | 30,109 | |
| 1171 | Greece | 2000-2001 | Karalis et al., BMC Public Health7:351, 2007 | Community | rural | 5+ | 5+ | 73 | 87 | |
| 1172 | Greece | 2001 | Greece Physical Fitness Study | National | both | 8 | 8 | 31,504 | 29,956 | |
| 1173 | Greece | 2003 | National Epidemiological Survey | National | both | 13-19 | 13-19 | 6,675 | 7,778 | |
| 1174 | Greece | 2003 | Greece Physical Fitness Study | National | both | 8 | 8 | 33,575 | 31,495 | |
| 1175 | Greece | 2004 | Greece Physical Fitness Study | National | both | 8 | 8 | 33,239 | 31,989 | |
| 1176 | Greece | 2004-2005 | Arsakeion School Study | Community | urban | 6-18 | 6-18 | 358 | 420 | |
| 1177 | Greece | 2005 | Daphne | Community | rural | 17-18 | 17-18 | 41 | 57 | |
| 1178 | Greece | 2005 | Greece Physical Fitness Study | National | both | 9 | 9 | 33,206 | 31,950 | |
| 1179 | Greece | 2005-2006 | Prevalence of hypertension and association of dietary mineral intake with blood pressure in healthy school children from Northern Greece aged 7-15years | Community | urban | 7-15 | 7-15 | 308 | 297 | |
| 1180 | Greece | 2006 | Greece Physical Fitness Study | National | both | 9 | 9 | 33,545 | 31,944 | |
| 1181 | Greece | 2006 | Paliouri Study | Community | rural | 65-94 | 65-94 | 92 | 66 | |
| 1182 | Greece | 2006 | Samos | Community | both | 5-13 | 5-13 | 55 | 65 | |
| 1183 | Greece | 2006-2007 | HELENA, Athens | Community | urban | 12-17 | 12-17 | 158 | 162 | |
| 1184 | Greece | 2006-2007 | HELENA, Heraklion | Community | urban | 12-17 | 12-17 | 135 | 149 | |
| 1185 | Greece | 2007 | Greece Physical Fitness Study | National | both | 9 | 9 | 33,501 | 31,870 | |
| 1186 | Greece | 2007-2009 | Healthy Growth Study | Subnational | both | 9-13 | 9-13 | 1,317 | 1,300 | |
| 1187 | Greece | 2008 | Greece Physical Fitness Study | National | rural | 8 | 8 | 8,411 | 8,077 | |
| 1188 | Greece | 2008 | Greece Physical Fitness Study | National | urban | 8 | 8 | 28,189 | 27,247 | |
| 1189 | Greece | 2008-2009 | Greek Childhood Obesity Study (GRECO) | National | both | 10-12 | 10-12 | 2,033 | 2,160 | |
| 1190 | Greece | 2010 | EuropeaN Energy balance Research to prevent excessive weight Gain among Youth - The ENERGY-project | National | both | 10-12 | 10-12 | 493 | 577 | |
| 1191 | Greece | 2010-2011 | Childhood Obesity Surveillance Initiative 2 | National | both | 7-9 | 7-9 | 2,581 | 2,688 | |
| 1192 | Greece | 2010-2012 | ADONUT | National | both | 12-19 | 12-19 | 18,668 | 18,675 | |
| 1193 | Greece | 2013 | Telemedicine screening adolescent metabolic syndrome in Greek schools | Community | urban | 12-17 | 12-17 | 669 | 905 | |
| 1194 | Greece | 2013 | Childhood Obesity Surveillance Initiative 3 | National | both | 7-10 | 7-10 | 3,965 | 3,908 | |
| 1195 | Greece | 2013-2014 | Greek National Health and Nutrition Survey - HYDRIA | National | both | 18+ | 18+ | 1,798 | 2,082 | |
| 1196 | Greece | 2013-2015 | Hellenic National Nutrition and Health Survey (HNNHS) | Subnational | urban | 5+ | 5+ | 476 | 757 | |
| 1197 | Greece | 2013-2016 | National Survey of Morbidity and Risk Factors (EMENO) | National | both | 18+ | 18+ | 2,039 | 2,726 | |
| 1198 | Greece | 2014-2015 | Evaluation of a web-based dietary intervention among primary school children (NUTRI-WEB Children project) | Community | both | 7-12 | 7-12 | 387 | 433 | |
| 1199 | Greece | 2014-2017 | National Survey of Morbidity and Risk Factors, Children substudy (EMENO-Children) | National | both | 5-18 | 5-18 | 187 | 164 | |
| 1200 | Greece | 2015-2016 | Childhood Obesity Surveillance Initiative 4 | National | both | 7-9 | 7-9 | 1,907 | 1,872 | |
| 1201 | Greece | 2016 | SKG-Elderly | Community | urban | 60+ | 60+ | 51 | 63 | |
| 1202 | Greece | 2016-2017 | Erasmus plus KA2, Healthyland | Community | rural | 5 | 5 | 13 | 9 | |
| 1203 | Greece | 2017-2018 | Erasmus plus KA2, Healthyland | Community | rural | 5-6 | 5-6 | 33 | 12 | |
| 1204 | Greece | 2018 | Greece Physical Fitness Study | National | both | 5-19 | 5-19 | 165,298 | 156,790 | |
| 1205 | Greece | 2018 | STEPS-Thessaloniki | Community | both | 60+ | 60+ | 353 | 141 | |
| 1206 | Greece | 2019 | Childhood Obesity Surveillance Initiative 5 | National | both | 7-10 | 7-10 | 2,013 | 2,014 | |
| 1207 | Greenland | 2005-2010 | Population Health Survey in Greenland | National | both | 18+ | 18+ | 1,336 | 1,714 | |
| 1208 | Greenland | 2016-2019 | Population Health Survey in Greenland | National | both | 15+ | 15+ | 1,117 | 1,308 | |
| 1209 | Grenada | 2011 | STEPS | National | both | 25-64 | 25-64 | 438 | 637 | |
| 1210 | Guatemala | 1995 | DHS | National | both | | 20-49 | | 4,547 | |
| 1211 | Guatemala | 1998-1999 | DHS | National | both | | 20-49 | | 2,172 | |
| 1212 | Guatemala | 2001-2002 | CAMDI | Community | urban | 20+ | 20+ | 293 | 638 | |

| | Country | Study years | Survey/Study name/Citation | Level of representative-ness | Rural, urban, or both | Age range as in NCD-RisC database | | Sample size | | Notes |
|---|---|---|---|---|---|---|---|---|---|---|
| | | | | | | Male | Female | Male | Female | |
| 1213 | Guatemala | 2002 | Reproducive Health Survey | National | both | 15-59 | 15-49 | 2,164 | 7,374 | |
| 1214 | Guatemala | 2003-2005 | The Institute of Nutrition of Central America and Panama Nutrition Supplementation Trial Cohort | Community | both | 25-41 | 25-41 | 268 | 288 | |
| 1215 | Guatemala | 2008-2009 | Encuesta Nacional de Salud Materno Infantil | National | both | 15-59 | 15-49 | 6,636 | 15,271 | |
| 1216 | Guatemala | 2013 | Sistema de vigilancia Epidemiológica de Salud y Nutrición (SIVESNU) | National | both | | 15-49 | | 1,545 | |
| 1217 | Guatemala | 2014-2015 | DHS | National | both | | 15-49 | | 24,195 | |
| 1218 | Guatemala | 2015 | Sistema de vigilancia Epidemiológica de Salud y Nutrición (SIVESNU) | National | both | | 15-49 | | 1,542 | |
| 1219 | Guatemala | 2015 | STEPS | Subnational | urban | 18+ | 18+ | 432 | 1,444 | |
| 1220 | Guatemala | 2015-2017 | Nutrition on early childhood and metabolomic and cardiometabolic profile on adulthood (META) | Community | both | 37-55 | 37-55 | 213 | 311 | |
| 1221 | Guatemala | 2016 | Sistema de vigilancia Epidemiológica de Salud y Nutrición (SIVESNU) | National | both | | 15-49 | | 1,560 | |
| 1222 | Guatemala | 2017-2018 | Sistema de vigilancia Epidemiológica de Salud y Nutrición (SIVESNU) | National | both | | 15-49 | | 1,557 | |
| 1223 | Guatemala | 2018 | Assessing the food environment inside and around rural public schools in three villages of El Progreso, Guatemala. A pilot study. | Community | rural | 6-15 | 6-15 | 202 | 192 | |
| 1224 | Guatemala | 2018-2019 | Population-Based Survey of Chronic Kidney Disease in Guatemala | Community | rural | 18+ | 18+ | 265 | 509 | |
| 1225 | Guatemala | 2018-2019 | Sistema de vigilancia Epidemiológica de Salud y Nutrición (SIVESNU) | National | both | | 15-49 | | 1,588 | |
| 1226 | Guatemala | 2019 | Evaluación del estado nutricional de escolares de nivel preprimario y primario en el marco de la ley de alimentación escolar | National | both | 5-14 | 5-14 | 9,645 | 8,979 | |
| 1227 | Guatemala | 2021 | Evaluación del estado nutricional de escolares de nivel preprimario y primario en el marco de la ley de alimentación escolar | National | both | 5-14 | 5-14 | 11,919 | 11,376 | |
| 1228 | Guinea | 1999 | DHS | National | both | | 20-49 | | 2,984 | |
| 1229 | Guinea | 2005 | DHS | National | both | | 15-49 | | 3,574 | |
| 1230 | Guinea | 2009 | STEPS | Subnational | both | 15-64 | 15-64 | 1,124 | 1,232 | |
| 1231 | Guinea | 2012 | DHS | National | both | | 15-49 | | 4,229 | |
| 1232 | Guinea | 2018 | DHS | National | both | | 15-49 | | 4,905 | |
| 1233 | Guinea Bissau | 2010 | Multiple Indicator Cluster Survey | National | both | | 15-49 | | 7,676 | |
| 1234 | Guyana | 2009 | DHS | National | both | 15-49 | 15-49 | 3,412 | 4,575 | |
| 1235 | Guyana | 2010 | Global School-based Student Health Survey | National | both | 13-17 | 13-17 | 987 | 1,261 | |
| 1236 | Guyana | 2016 | STEPS | National | both | 18-69 | 18-69 | 1,060 | 1,571 | |
| 1237 | Haiti | 1994-1995 | DHS | National | both | | 20-49 | | 1,788 | |
| 1238 | Haiti | 2000 | DHS | National | both | | 15-49 | | 9,163 | |
| 1239 | Haiti | 2005-2006 | DHS | National | both | | 15-49 | | 5,011 | |
| 1240 | Haiti | 2012 | DHS | National | both | | 15-49 | | 8,993 | |
| 1241 | Haiti | 2015-2016 | Haiti Health Study (Carrefour) | Community | urban | 25-65 | 25-65 | 557 | 835 | |
| 1242 | Haiti | 2015-2016 | Haiti Health Study (Thomonde) | Community | rural | 25-65 | 25-65 | 257 | 420 | |
| 1243 | Haiti | 2016-2017 | DHS | National | both | | 15-49 | | 9,049 | |
| 1244 | Honduras | 1996 | Honduras National Micronutrient Survey | National | both | | 20-40 | | 722 | |
| 1245 | Honduras | 2003-2004 | CAMDI | Community | urban | 20+ | 20+ | 428 | 764 | |
| 1246 | Honduras | 2005-2006 | DHS | National | both | | 15-49 | | 18,125 | |
| 1247 | Honduras | 2011-2012 | DHS | National | both | | 15-49 | | 21,097 | |
| 1248 | Hungary | 1982-1983 | MONICA, Pecs | Community | urban | 25-64 | 25-64 | 823 | 861 | |
| 1249 | Hungary | 1982-1984 | MONICA, Budapest | Community | urban | 25-64 | 25-64 | 774 | 737 | |
| 1250 | Hungary | 1985 | INTERSALT | Community | rural | 20-59 | 20-59 | 100 | 100 | |
| 1251 | Hungary | 1985-1988 | First Hungarian Representative Nutrition Survey | National | both | 15+ | 15+ | 3,079 | 8,916 | |
| 1252 | Hungary | 1987-1988 | MONICA, Budapest | Community | urban | 25-64 | 25-64 | 1,413 | 1,594 | |
| 1253 | Hungary | 1987-1988 | MONICA, Pecs | Community | urban | 35-64 | 35-64 | 1,573 | 1,510 | |
| 1254 | Hungary | 2003 | The European Male Ageing Study | Community | both | 40+ | | 428 | | |
| 1255 | Hungary | 2006-2007 | HELENA | Community | urban | 12-17 | 12-17 | 197 | 197 | |
| 1256 | Hungary | 2007-2010 | Identification and prevention of Dietary- and lifestyle-induced health EFfects In Children and infantS (IDEFICS) | Community | urban | 5-9 | 5-9 | 1,128 | 1,169 | |
| 1257 | Hungary | 2008 | The European Male Ageing Study | Community | both | 40+ | | 349 | | |
| 1258 | Hungary | 2009 | Hungarian diet and nutritional status survey | National | both | 18+ | 18+ | 463 | 666 | |
| 1259 | Hungary | 2010 | Childhood Obesity Surveillance Initiative 2 | National | both | 7 | 7 | 553 | 682 | |
| 1260 | Hungary | 2010 | EuropeaN Energy balance Research to prevent excessive weight Gain among Youth - The ENERGY-project | National | both | 11-13 | 11-13 | 452 | 557 | |
| 1261 | Hungary | 2014 | Hungarian diet and nutritional status survey | National | both | 18+ | 18+ | 369 | 478 | |
| 1262 | Hungary | 2015-2016 | Childhood Obesity Surveillance Initiative 4 | National | both | 6-8 | 6-8 | 2,750 | 2,572 | |
| 1263 | Hungary | 2016 | Feel4Diabetes | Community | both | 6-10 | 6-10 | 1,447 | 1,522 | |
| 1264 | Hungary | 2016 | Hungarian National Student Fitness Test (NETFIT) - Primary | National | both | 10-15 | 10-15 | 164,897 | 158,087 | |
| 1265 | Hungary | 2016 | Hungarian National Student Fitness Test (NETFIT) - Secondary | National | both | 14-19 | 14-19 | 134,129 | 132,326 | |

| | Country | Study years | Survey/Study name/Citation | Level of representative-ness | Rural, urban, or both | Age range as in NCD-RisC database | | Sample size | | Notes |
|---|---|---|---|---|---|---|---|---|---|---|
| | | | | | | Male | Female | Male | Female | |
| 1266 | Hungary | 2017 | Hungarian National Student Fitness Test (NETFIT) - Primary | National | both | 10-15 | 10-15 | 170,978 | 163,599 | |
| 1267 | Hungary | 2017 | Hungarian National Student Fitness Test (NETFIT) - Secondary | National | both | 14-19 | 14-19 | 136,278 | 134,646 | |
| 1268 | Hungary | 2018 | Hungarian National Student Fitness Test (NETFIT) - Primary | National | both | 10-15 | 10-15 | 169,915 | 162,664 | |
| 1269 | Hungary | 2018 | Hungarian National Student Fitness Test (NETFIT) - Secondary | National | both | 14-19 | 14-19 | 128,717 | 127,821 | |
| 1270 | Hungary | 2019 | Childhood Obesity Surveillance Initiative 5 | National | both | 6-8 | 6-8 | 3,173 | 2,996 | |
| 1271 | Hungary | 2019 | Hungarian National Student Fitness Test (NETFIT) - Primary | National | both | 10-15 | 10-15 | 171,701 | 165,199 | |
| 1272 | Hungary | 2019 | Hungarian National Student Fitness Test (NETFIT) - Secondary | National | both | 14-19 | 14-19 | 132,647 | 130,182 | |
| 1273 | Hungary | 2019 | Hungarian diet and nutritional status survey | National | both | 18+ | 18+ | 419 | 493 | |
| 1274 | Iceland | 1979-1981 | The Reykjavik Study (Men) | Subnational | urban | 45-74 | | 3,235 | | |
| 1275 | Iceland | 1981-1984 | The Reykjavik Study (Women) | Subnational | urban | | 46-75 | | 3,567 | |
| 1276 | Iceland | 1983 | MONICA, Arnes County | Community | rural | 25-64 | 25-64 | 388 | 450 | |
| 1277 | Iceland | 1983 | MONICA, Reykjavik | Subnational | urban | 25-64 | 25-64 | 434 | 461 | |
| 1278 | Iceland | 1983-1985 | The Reykjavik Study for the young | Subnational | urban | 29-45 | 29-45 | 823 | 895 | |
| 1279 | Iceland | 1985-1986 | INTERSALT | Community | urban | 20-59 | 20-59 | 100 | 100 | |
| 1280 | Iceland | 1985-1987 | The Reykjavik Study (Men) | Subnational | urban | 51-79 | | 2,584 | | |
| 1281 | Iceland | 1987-1991 | The Reykjavik Study (Women) | Subnational | urban | | 52-82 | | 2,993 | |
| 1282 | Iceland | 1988-1989 | MONICA, Arnes County | Community | rural | 25-64 | 25-64 | 385 | 435 | |
| 1283 | Iceland | 1988-1989 | MONICA, Reykjavik | Subnational | urban | 25-64 | 25-64 | 414 | 443 | |
| 1284 | Iceland | 1991-1994 | The Reykjavik Study (Men) | Subnational | urban | 70-86 | | 797 | | |
| 1285 | Iceland | 1993-1994 | MONICA, Arnes County | Community | rural | 25-64 | 25-64 | 422 | 484 | |
| 1286 | Iceland | 1993-1994 | MONICA, Reykjavik | Subnational | urban | 25-64 | 25-64 | 441 | 448 | |
| 1287 | Iceland | 1994-1996 | The Reykjavik Study (Women) | Subnational | urban | | 69-88 | | 1,101 | |
| 1288 | Iceland | 2001-2003 | The Reykjavik Study for the young | Subnational | urban | 47-62 | 47-62 | 626 | 705 | |
| 1289 | Iceland | 2002-2006 | AGES | Subnational | urban | 66-96 | 66-96 | 2,414 | 3,282 | |
| 1290 | Iceland | 2005-2011 | Risk Evaluation For INfarct Estimates (REFINE) | Subnational | urban | 20-73 | 20-73 | 3,402 | 3,525 | |
| 1291 | Iceland | 2007-2011 | AGESII | Subnational | urban | 71-98 | 71-98 | 1,374 | 1,929 | |
| 1292 | Iceland | 2010-2012 | Risk Evaluation For INfarct Estimates (REFINE) follow-up visit (REFINELO) | Subnational | urban | 26-74 | 26-74 | 653 | 667 | |
| 1293 | Iceland | 2012-2013 | Risk Evaluation For INfarct Estimates (REFINE) follow-up visit (REFLOCT) | Subnational | urban | 55-73 | 55-73 | 516 | 561 | |
| 1294 | India | 1975-1979 | National Nutrition Monitoring Bureau rural survey | National | rural | 5+ | 5+ | 52,704 | 37,750 | 1 |
| 1295 | India | 1982-1983 | Bengali School Children | Community | urban | 7-21 | | 808 | | |
| 1296 | India | 1986 | INTERSALT | Community | urban | 20-59 | 20-59 | 100 | 99 | |
| 1297 | India | 1988-1989 | Ramachandran et al., Diabetes Res Clin Pract 58(1):55-60, 2002 | Community | urban | 20-74 | 20-74 | 455 | 437 | |
| 1298 | India | 1990 | National Nutrition Monitoring Bureau rural survey | National | rural | 5+ | 5+ | 7,607 | 9,649 | |
| 1299 | India | 1991-1994 | Prabhakaran et al., Chronic Illn 3(1):8-19, 2007 | Community | rural | 35-64 | 35-64 | 542 | 630 | |
| 1300 | India | 1991-1994 | Prabhakaran et al., Chronic Illn 3(1):8-19, 2007 | Community | urban | 35-64 | 35-64 | 1,388 | 1,455 | |
| 1301 | India | 1991-1995 | Reddy et al., Obes Rev 3(3):197-202, 2002 | Community | rural | 35-64 | 35-64 | 1,070 | 1,332 | |
| 1302 | India | 1991-1995 | Reddy et al., Obes Rev 3(3):197-202, 2002 | Community | urban | 35-64 | 35-64 | 1,456 | 1,594 | |
| 1303 | India | 1991-1997 | Mumbai Cohort Study | Community | urban | 35+ | 35+ | 88,658 | 59,515 | |
| 1304 | India | 1992-1994 | Jaipur Heart Watch 1 | Community | urban | 20-80 | 20-80 | 1,385 | 782 | |
| 1305 | India | 1992-1994 | Jaipur Heart Watch 1 | Community | rural | 20-80 | 20-80 | 1,946 | 1,147 | |
| 1306 | India | 1993-1994 | Khongsdier, Eur J Clin Nutr 56(6):484-89, 2002 | Community | both | 18-59 | | 575 | | |
| 1307 | India | 1995 | Shobana et al., Diabetes Res Clin Pract 42(3):18186, 1998 | Community | urban | 20-74 | 20-74 | 1,061 | 1,093 | |
| 1308 | India | 1995-1996 | Kusuma et al., Ann Hum Biol 29(5):502-12, 2002 | Community | both | 15-84 | 15-84 | 747 | 737 | |
| 1309 | India | 1995-1996 | Epidemiology of blood pressure across cross-cultural populations of Visakhapatnam district, Andhra Pradesh, India | Community | rural | 19-76 | 19-76 | 209 | 228 | |
| 1310 | India | 1995-1997 | Aravind Comprehensive Eye Survey | Community | rural | 40+ | 40+ | 2,308 | 2,830 | |
| 1311 | India | 1995-1997 | Kashmiri Adults | Subnational | both | 40+ | 40+ | 2,496 | 2,587 | |
| 1312 | India | 1996-1997 | National Nutrition Monitoring Bureau rural survey | National | rural | 5+ | 5+ | 22,155 | 27,802 | |
| 1313 | India | 1996-1999 | Chennai Urban Population Study | Community | urban | 20+ | 20+ | 557 | 705 | |
| 1314 | India | 1997 | Ramachandran et al., Diabetes Res Clin Pract 44(3):207-13, 1999 | Community | rural | 20-74 | 20-74 | 738 | 879 | |
| 1315 | India | 1998-1999 | DHS | National | both | | 20-49 | | 72,536 | |
| 1316 | India | 1998-2001 | Chennai Prospective Study | Community | urban | 35+ | 35+ | 264,848 | 235,968 | |
| 1317 | India | 1998-2002 | Vellore Birth Cohort | Subnational | both | 25-31 | 25-31 | 1,160 | 1,050 | |
| 1318 | India | 1999-2001 | Jaipur Heart Watch 2 | Community | urban | 20-75 | 20-75 | 534 | 569 | |
| 1319 | India | 1999-2002 | Bengali School Children | Community | urban | 7-21 | | 1,152 | | |
| 1320 | India | 1999-2002 | New Delhi Birth Cohort | Community | urban | 26-33 | 26-33 | 886 | 638 | |
| 1321 | India | 2000 | Ramachandran et al., Diabet Med 20(3):220-24, 2003 | Subnational | urban | 20-75 | 20-75 | 4,640 | 5,257 | |

| | Country | Study years | Survey/Study name/Citation | Level of representative-ness | Rural, urban, or both | Age range as in NCD-RisC database | | Sample size | | Notes |
|---|---|---|---|---|---|---|---|---|---|---|
| | | | | | | Male | Female | Male | Female | |
| 1322 | India | 2000-2001 | National Nutrition Monitoring Bureau rural survey | National | rural | 5+ | 5+ | 18,046 | 24,844 | |
| 1323 | India | 2001-2004 | Chennai Urban Rural Epidemiology Study | Community | urban | 20+ | 20+ | 1,094 | 1,254 | |
| 1324 | India | 2002-2003 | Blood pressure epidemiology in tribal, rural and urban communities of Orissa with special reference to physical and social parameters | Community | rural | 18-80 | 18-80 | 200 | 186 | |
| 1325 | India | 2002-2005 | The Chennai Glaucoma Study | Subnational | urban | 40+ | 40+ | 1,577 | 1,972 | |
| 1326 | India | 2003-2004 | Jaipur Heart Watch 3 | Community | urban | 20-75 | 20-75 | 211 | 228 | |
| 1327 | India | 2003-2004 | ICMR RF RHD Registry, Jai Vigyan Mission Mode, Kochi | Subnational | both | 5-16 | 5-16 | 11,327 | 13,515 | |
| 1328 | India | 2003-2005 | STEPS, Ballabgarh | Subnational | rural | 15-69 | 15-69 | 1,360 | 1,468 | |
| 1329 | India | 2003-2005 | STEPS, Ballabgarh | Subnational | urban | 15-69 | 15-69 | 1,263 | 1,294 | |
| 1330 | India | 2003-2005 | STEPS, Chennai | Subnational | rural | 15-69 | 15-69 | 1,372 | 1,338 | |
| 1331 | India | 2003-2005 | STEPS, Chennai | Subnational | urban | 15-69 | 15-69 | 1,282 | 1,282 | |
| 1332 | India | 2003-2005 | STEPS, Delhi | Subnational | urban | 15-69 | 15-69 | 1,250 | 1,265 | |
| 1333 | India | 2003-2005 | STEPS, Dibrugarh | Subnational | rural | 15-69 | 15-69 | 1,460 | 1,410 | |
| 1334 | India | 2003-2005 | STEPS, Dibrugarh | Subnational | urban | 15-69 | 15-69 | 1,243 | 1,254 | |
| 1335 | India | 2003-2005 | STEPS, Nagpur | Subnational | rural | 15-69 | 15-69 | 1,252 | 1,256 | |
| 1336 | India | 2003-2005 | STEPS, Nagpur | Subnational | urban | 15-69 | 15-69 | 1,252 | 1,261 | |
| 1337 | India | 2003-2005 | STEPS, Trivandrum | Subnational | rural | 15-69 | 15-69 | 1,199 | 1,324 | |
| 1338 | India | 2003-2005 | STEPS, Trivandrum | Subnational | urban | 15-69 | 15-69 | 1,250 | 1,252 | |
| 1339 | India | 2004-2005 | India Human Development Survey | National | both | 8-11 | 8-11 | 6,959 | 6,406 | 12 |
| 1340 | India | 2005-2006 | DHS | National | both | 15-54 | 15-49 | 71,463 | 115,828 | |
| 1341 | India | 2005-2006 | Risk factor profile for chronic non-communicable diseases: Results of a community-based study in Kerala, India | Community | both | 15-64 | 15-64 | 2,795 | 2,926 | |
| 1342 | India | 2005-2006 | ICMR RF RHD Registry, Jai Vigyan Mission Mode, Kochi | Subnational | both | 5-16 | 5-16 | 9,754 | 10,509 | |
| 1343 | India | 2005-2006 | National Nutrition Monitoring Bureau rural survey | National | rural | 5+ | 5+ | 20,448 | 25,272 | |
| 1344 | India | 2005-2007 | Prevalence of cardiovascular risk factors in rural Tamil Nadu | Community | rural | 25-64 | 25-64 | 4,927 | 5,573 | |
| 1345 | India | 2005-2011 | Bengali School Children | Community | urban | 7-21 | 7-21 | 847 | 2,180 | |
| 1346 | India | 2006 | Ramachandran et al., Diabetes Care 31(5):893-98, 2008 | Community | both | 20+ | 20+ | 3,321 | 3,745 | |
| 1347 | India | 2006-2007 | Kusuma et al., Asia Pac J Public Health 21(4):497-507, 2009 | Community | urban | 15-74 | 15-74 | 182 | 192 | |
| 1348 | India | 2006-2007 | Jaipur Heart Watch 4 | Community | urban | 20-75 | 20-75 | 533 | 558 | |
| 1349 | India | 2006-2008 | Central India Eye and Medical Study | Community | rural | 30+ | 30+ | 2,190 | 2,518 | |
| 1350 | India | 2006-2008 | Kashmiri Young Adults | Subnational | both | 20-40 | 20-40 | 2,119 | 905 | |
| 1351 | India | 2006-2009 | New Delhi Birth Cohort | Community | urban | 33-38 | 33-38 | 650 | 445 | |
| 1352 | India | 2007-2008 | Urban population in Hyderabad | Community | urban | 20-60 | 20-60 | 1,519 | 1,560 | |
| 1353 | India | 2007-2008 | Integrated Disease Surveillance Project Non-communicable Disease Risk Factors Survey, Andhra | Subnational | both | 15-64 | 15-64 | 2,674 | 3,390 | |
| 1354 | India | 2007-2008 | Integrated Disease Surveillance Project Non-communicable Disease Risk Factors Survey, Kerala | Subnational | both | 15-64 | 15-64 | 1,672 | 2,403 | |
| 1355 | India | 2007-2008 | Integrated Disease Surveillance Project Non-communicable Disease Risk Factors Survey, Madhya | Subnational | both | 15-64 | 15-64 | 2,797 | 2,862 | |
| 1356 | India | 2007-2008 | Integrated Disease Surveillance Project Non-communicable Disease Risk Factors Survey, Maharashtra | Subnational | both | 15-64 | 15-64 | 3,025 | 2,921 | |
| 1357 | India | 2007-2008 | Integrated Disease Surveillance Project Non-communicable Disease Risk Factors Survey, Mizoram | Subnational | both | 15-64 | 15-64 | 2,232 | 2,108 | |
| 1358 | India | 2007-2008 | Integrated Disease Surveillance Project Non-communicable Disease Risk Factors Survey, Tamil | Subnational | both | 15-64 | 15-64 | 2,039 | 2,928 | |
| 1359 | India | 2007-2008 | Integrated Disease Surveillance Project Non-communicable Disease Risk Factors Survey, Uttrakhand | Subnational | both | 15-64 | 15-64 | 2,094 | 3,110 | |
| 1360 | India | 2007-2008 | WHO Study on global AGEing and adult health (SAGE) | National | both | 50+ | 50+ | 3,213 | 3,147 | |
| 1361 | India | 2007-2009 | Prevalence of NCD risk factor in people above 15 year in rural area Nagpur using WHO STEP approach | Community | rural | 15+ | 15+ | 1,984 | 1,828 | |
| 1362 | India | 2008-2010 | ICMR-India Diabetes (INDIAB) Study, Phase I | National | both | 20+ | 20+ | 6,953 | 6,850 | |
| 1363 | India | 2009-2010 | Jaipur Heart Watch 5 | Community | urban | 20-75 | 20-75 | 425 | 275 | |
| 1364 | India | 2009-2010 | Baseline Survey for the assessment of prevalence of risk factors of NCDs in Gandhinagar District | Community | rural | 15-64 | 15-64 | 875 | 774 | |
| 1365 | India | 2009-2010 | Baseline Survey for the assessment of prevalence of risk factors of NCDs in Gandhinagar District | Community | urban | 15-64 | 15-64 | 895 | 890 | |
| 1366 | India | 2010 | Kerala 2010 follow-up | Community | rural | 21-70 | 21-70 | 214 | 237 | |
| 1367 | India | 2010-2011 | Longitudinal Aging Study in India | Subnational | both | 45+ | 45+ | 630 | 678 | |
| 1368 | India | 2010-2012 | Centre for cArdiometabolic Risk Reduction in South-Asia (CARRS) - Surveillance Study | Community | urban | 20+ | 20+ | 4,320 | 5,138 | 13 |
| 1369 | India | 2011-2012 | Body Mass Index, Social Conditions and Environmental Effect on High Blood Pressure among the Adolescent School Children | National | both | 12-16 | 12-16 | 1,096 | 1,161 | |
| 1370 | India | 2011-2012 | India Human Development Survey | National | both | 8-11 | 8-11 | 6,285 | 5,758 | 14 |
| 1371 | India | 2011-2012 | National Nutrition Monitoring Bureau rural survey | National | rural | 5+ | 5+ | 34,447 | 43,063 | |
| 1372 | India | 2011-2013 | International Study of Childhood Obesity, Lifestyle and the Environment (ISCOLE) | Community | urban | 9-11 | 9-11 | 292 | 328 | |
| 1373 | India | 2012-2013 | Health Survey in Anand School Children | Community | both | 5-13 | 5-13 | 1,628 | 959 | |
| 1374 | India | 2012-2013 | District Level Household and Facility Survey (DLHS) 4 | National | both | 5+ | 5+ | 538,808 | 602,926 | |
| 1375 | India | 2012-2013 | ICMR-India Diabetes (INDIAB) Study, Phase II | Subnational | both | 20+ | 20+ | 8,165 | 10,738 | |

| | Country | Study years | Survey/Study name/Citation | Level of representative-ness | Rural, urban, or both | Age range as in NCD-RisC database | | Sample size | | Notes |
|---|---|---|---|---|---|---|---|---|---|---|
| | | | | | | Male | Female | Male | Female | |
| 1376 | India | 2012-2013 | Processed and non-processed foods | National | both | 5+ | 5+ | 7,161 | 7,800 | |
| 1377 | India | 2012-2014 | Jaipur Heart Watch 6 | Community | urban | 20-75 | 20-75 | 1,013 | 722 | |
| 1378 | India | 2012-2015 | ICMR-India Diabetes (INDIAB) Study, North East Phase | Subnational | both | 20+ | 20+ | 14,071 | 16,456 | |
| 1379 | India | 2013-2014 | Vellore Birth Cohort | Subnational | both | 39-44 | 39-44 | 580 | 499 | |
| 1380 | India | 2014 | Annual Health Survey: Clinical, Anthropometric and Bio-chemical | National | both | 5+ | 5+ | 658,441 | 680,611 | |
| 1381 | India | 2014-2015 | Control of Hypertension In Rural India (CHIRI) - Rishi Valley | Community | rural | 18+ | 18+ | 2,591 | 3,518 | |
| 1382 | India | 2014-2015 | Control of Hypertension In Rural India (CHIRI) - Trivandrum | Community | rural | 18+ | 18+ | 1,848 | 1,898 | |
| 1383 | India | 2014-2015 | Control of Hypertension In Rural India (CHIRI) - West Godavari | Community | rural | 18+ | 18+ | 2,230 | 2,233 | |
| 1384 | India | 2015-2016 | DHS | National | both | 15-54 | 15-49 | 108,751 | 655,681 | |
| 1385 | India | 2015-2016 | Diet and nutritional status of urban population and prevalence of hypertension | National | urban | 5+ | 5+ | 68,893 | 81,999 | |
| 1386 | India | 2016-2017 | Secular TRends in DiabEtes in India (STRiDE-I) -Change in Prevalence in Ten Years among Urban and Rural Populations in Tamil Nadu | Community | both | 20+ | 20+ | 4,527 | 5,321 | |
| 1387 | India | 2016-2019 | Vellore Birth Cohort | Subnational | both | 43-48 | 43-48 | 843 | 758 | |
| 1388 | India | 2017-2018 | ICMR-India Diabetes (INDIAB) Study, Phase III | Subnational | both | 20+ | 20+ | 7,146 | 7,550 | |
| 1389 | India | 2017-2018 | National Noncommunicable Disease Monitoring Survey (NNMS) | National | both | 15-69 | 15-69 | 6,223 | 5,657 | |
| 1390 | India | 2017-2019 | Longitudinal Aging Study in India | National | both | 45+ | 45+ | 27,341 | 31,725 | |
| 1391 | India | 2018-2019 | ICMR-India Diabetes (INDIAB) Study, Phase IV | Subnational | both | 20+ | 20+ | 8,850 | 10,154 | |
| 1392 | India | 2019-2020 | Prevalence, awareness, treatment and control of hypertension among adults aged 30 years and above in Barmer district, Rajasthan, India | Community | rural | 30+ | 30+ | 153 | 146 | |
| 1393 | India | 2019-2020 | ICMR-India Diabetes (INDIAB) Study, Phase V | Subnational | both | 20+ | 20+ | 6,597 | 6,656 | |
| 1394 | India | 2019-2021 | DHS | National | both | 15-54 | 15-49 | 95,959 | 672,286 | |
| 1395 | India | 2021 | STEPS, Mumbai | Community | urban | 18-69 | 18-69 | 2,551 | 2,530 | |
| 1396 | India | 2022 | Global School-based Student Health Survey (Jaipur) | Community | urban | 12-15 | 12-15 | 1,524 | 1,355 | |
| 1397 | Indonesia | 1983-1987 | Strickland et al., Eur J Clin Nutr 48 Suppl 3: S98-108; discussion S-9, 1994 | Community | both | 18+ | 18+ | 447 | 564 | |
| 1398 | Indonesia | 1993-1994 | Indonesian Family Life Surveys | National | both | 5+ | 5+ | 8,211 | 9,480 | |
| 1399 | Indonesia | 1997-1998 | Indonesian Family Life Surveys | National | both | 5+ | 5+ | 12,154 | 13,897 | |
| 1400 | Indonesia | 2000-2001 | Indonesian Family Life Surveys | National | both | 5+ | 5+ | 15,442 | 16,225 | |
| 1401 | Indonesia | 2001 | Ng et al., Bull World Health Organ 84(4):305-13, 2006 | Community | both | 15-74 | 15-74 | 1,261 | 1,234 | |
| 1402 | Indonesia | 2001 | STEPS/SURKESNAS | National | both | 15-64 | 15-64 | 4,100 | 4,775 | |
| 1403 | Indonesia | 2003 | A genetic-ecological study of the risk factors for lifestyle-related diseases in Oceanian populations, Study A | Community | rural | 18-79 | 18-79 | 99 | 103 | |
| 1404 | Indonesia | 2003 | A genetic-ecological study of the risk factors for lifestyle-related diseases in Oceanian populations, Study B | Community | rural | 18-79 | 18-79 | 100 | 140 | |
| 1405 | Indonesia | 2006 | Jakarta Non Communicable Disease Risk Factor Surveillance | Community | urban | 25-64 | 25-64 | 641 | 950 | |
| 1406 | Indonesia | 2007-2008 | Indonesian Family Life Surveys | National | both | 5+ | 5+ | 17,869 | 19,157 | |
| 1407 | Indonesia | 2011 | SEANUTS | National | both | 5-12 | 5-12 | 1,363 | 1,380 | |
| 1408 | Indonesia | 2013 | Population Health Basic Health Research 2013 (Riskesdas 2013) | National | both | 5+ | 5+ | 454,771 | 474,011 | |
| 1409 | Indonesia | 2014-2015 | Indonesian Family Life Surveys | National | both | 5+ | 5+ | 20,328 | 21,540 | |
| 1410 | Indonesia | 2015 | Global School-based Student Health Survey | National | both | 13-17 | 13-17 | 3,845 | 4,688 | |
| 1411 | Indonesia | 2018 | Indonesian Basic Health Survey 2018 | National | both | 15+ | 15+ | 8,677 | 14,460 | |
| 1412 | Iran | 1990-1991 | National Health Survey I | National | both | 5-18 | 5-18 | 8,883 | 9,038 | |
| 1413 | Iran | 1997-1998 | Khadivzadeh, East Mediterr Health J 8(4-5):612-18, 2002 | Community | urban | | 15-49 | | 1,513 | |
| 1414 | Iran | 1999-2000 | National Health Survey II | National | both | 5+ | 5+ | 23,727 | 26,636 | |
| 1415 | Iran | 1999-2001 | Tehran Lipid and Glucose Study | Community | urban | 5+ | 5+ | 6,339 | 7,988 | |
| 1416 | Iran | 2000 | ASADABADI Study | Community | urban | 18+ | 18+ | 131 | 168 | |
| 1417 | Iran | 2001 | Isfahan Healthy Heart Programme (IHHP), Arak | Community | rural | 19+ | 19+ | 1,023 | 1,080 | |
| 1418 | Iran | 2001 | Isfahan Healthy Heart Programme (IHHP), Arak | Community | urban | 19+ | 19+ | 2,084 | 2,124 | |
| 1419 | Iran | 2001 | Isfahan Healthy Heart Programme (IHHP), Isfahan | Community | rural | 19+ | 19+ | 232 | 233 | |
| 1420 | Iran | 2001 | Isfahan Healthy Heart Programme (IHHP), Isfahan | Community | urban | 19+ | 19+ | 1,760 | 1,912 | |
| 1421 | Iran | 2001 | Isfahan Healthy Heart Programme (IHHP), Najaf Abad | Community | rural | 19+ | 19+ | 405 | 416 | |
| 1422 | Iran | 2001 | Isfahan Healthy Heart Programme (IHHP), Najaf Abad | Community | urban | 19+ | 19+ | 573 | 571 | |
| 1423 | Iran | 2001 | Isfahan Healthy Heart Programme (IHHP) Students, Arak | Community | rural | 11-18 | 11-18 | 144 | 192 | |
| 1424 | Iran | 2001 | Isfahan Healthy Heart Programme (IHHP) Students, Arak | Community | urban | 11-18 | 11-18 | 323 | 303 | |
| 1425 | Iran | 2001 | Isfahan Healthy Heart Programme (IHHP) Students, Isfahan | Community | rural | 11-18 | 11-18 | 89 | 118 | |
| 1426 | Iran | 2001 | Isfahan Healthy Heart Programme (IHHP) Students, Isfahan | Community | urban | 11-18 | 11-18 | 242 | 225 | |
| 1427 | Iran | 2001 | Isfahan Healthy Heart Programme (IHHP) Students, Najaf Abad | Community | rural | 11-18 | 11-18 | 61 | 74 | |
| 1428 | Iran | 2001 | Isfahan Healthy Heart Programme (IHHP) Students, Najaf Abad | Community | urban | 11-18 | 11-18 | 62 | 72 | |
| 1429 | Iran | 2002-2005 | Tehran Lipid and Glucose Study | Community | urban | 5+ | 5+ | 2,736 | 3,388 | |
| 1430 | Iran | 2003-2004 | Childhood and Adolescence Surveillance and Prevention of Adult Noncommunicable Disease (CASPIAN) | National | both | 6-18 | 6-18 | 10,791 | 10,170 | |

| | Country | Study years | Survey/Study name/Citation | Level of representative-ness | Rural, urban, or both | Age range as in NCD-RisC database | | Sample size | | Notes |
|---|---|---|---|---|---|---|---|---|---|---|
| | | | | | | Male | Female | Male | Female | |
| 1431 | Iran | 2003-2004 | The Persian Gulf Healthy Heart Study | Subnational | urban | 25-75 | 25-75 | 1,736 | 1,973 | |
| 1432 | Iran | 2004 | Hajian-Tilaki et al., Obes Rev 8(1):3-10, 2007 | Community | urban | 20-70 | 20-70 | 1,800 | 1,800 | |
| 1433 | Iran | 2004-2008 | Golestan Cohort Study Main Phase | Subnational | rural | 40-75 | 40-75 | 17,298 | 22,708 | |
| 1434 | Iran | 2004-2008 | Golestan Cohort Study Main Phase | Community | urban | 40-75 | 40-75 | 3,931 | 6,100 | |
| 1435 | Iran | 2005 | Dastgiri et al., Public Health Nutr 9(8): 996-1000, 2006 | Subnational | urban | 18-70 | 18-70 | 116 | 151 | |
| 1436 | Iran | 2005 | STEPS | National | both | 15-64 | 15-64 | 40,722 | 39,748 | |
| 1437 | Iran | 2005-2006 | Rashidy-Pour, Obes Rev (1):2-6, 2009 | Subnational | both | 30-70 | 30-70 | 1,695 | 2,104 | |
| 1438 | Iran | 2005-2008 | Tehran Lipid and Glucose Study | Community | urban | 5+ | 5+ | 3,104 | 3,808 | |
| 1439 | Iran | 2006 | STEPS | National | both | 16-65 | 16-65 | 14,885 | 14,617 | |
| 1440 | Iran | 2007 | Isfahan Healthy Heart Programme (IHHP), Arak | Community | rural | 19+ | 19+ | 1,028 | 1,024 | |
| 1441 | Iran | 2007 | Isfahan Healthy Heart Programme (IHHP), Arak | Community | urban | 19+ | 19+ | 1,424 | 1,359 | |
| 1442 | Iran | 2007 | Isfahan Healthy Heart Programme (IHHP), Isfahan | Community | rural | 19+ | 19+ | 155 | 151 | |
| 1443 | Iran | 2007 | Isfahan Healthy Heart Programme (IHHP), Isfahan | Community | urban | 19+ | 19+ | 1,309 | 1,301 | |
| 1444 | Iran | 2007 | Isfahan Healthy Heart Programme (IHHP), Najaf Abad | Community | rural | 19+ | 19+ | 253 | 253 | |
| 1445 | Iran | 2007 | Isfahan Healthy Heart Programme (IHHP), Najaf Abad | Community | urban | 19+ | 19+ | 494 | 542 | |
| 1446 | Iran | 2007 | Isfahan Healthy Heart Programme (IHHP) Students, Arak | Community | rural | 11-18 | 11-18 | 177 | 164 | |
| 1447 | Iran | 2007 | Isfahan Healthy Heart Programme (IHHP) Students, Arak | Community | urban | 11-18 | 11-18 | 327 | 341 | |
| 1448 | Iran | 2007 | Isfahan Healthy Heart Programme (IHHP) Students, Isfahan | Community | rural | 11-18 | 11-18 | 16 | 19 | |
| 1449 | Iran | 2007 | Isfahan Healthy Heart Programme (IHHP) Students, Isfahan | Community | urban | 11-18 | 11-18 | 396 | 335 | |
| 1450 | Iran | 2007 | Isfahan Healthy Heart Programme (IHHP) Students, Najaf Abad | Community | rural | 11-18 | 11-18 | 38 | 39 | |
| 1451 | Iran | 2007 | Isfahan Healthy Heart Programme (IHHP) Students, Najaf Abad | Community | urban | 11-18 | 11-18 | 44 | 67 | |
| 1452 | Iran | 2007 | STEPS - National | National | both | 15-64 | 15-64 | 2,372 | 2,312 | |
| 1453 | Iran | 2007 | STEPS - Provincial | National | both | 15-64 | 15-64 | 14,867 | 14,550 | |
| 1454 | Iran | 2008 | STEPS | National | both | 15-64 | 15-64 | 14,757 | 14,353 | |
| 1455 | Iran | 2008-2011 | Tehran Lipid and Glucose Study | Community | urban | 20+ | 20+ | 4,622 | 5,884 | |
| 1456 | Iran | 2009 | STEPS | National | both | 15-64 | 15-64 | 14,834 | 14,495 | |
| 1457 | Iran | 2009-2010 | Childhood and Adolescence Surveillance and Prevention of Adult Noncommunicable Disease (CASPIAN) | National | both | 10-18 | 10-18 | 2,799 | 2,814 | |
| 1458 | Iran | 2009-2010 | The Persian Gulf Healthy Heart Study | Subnational | urban | 31-79 | 31-79 | 834 | 1,016 | |
| 1459 | Iran | 2010-2011 | The Yazd Eye Study | Subnational | both | 40-80 | 40-80 | 876 | 1,012 | |
| 1460 | Iran | 2010-2012 | Golestan Cohort Study Second Phase | Subnational | rural | 43-82 | 43-82 | 4,325 | 4,919 | |
| 1461 | Iran | 2010-2012 | Golestan Cohort Study Second Phase | Community | urban | 43-82 | 43-82 | 1,091 | 1,061 | |
| 1462 | Iran | 2011 | STEPS | National | both | 6-69 | 6-69 | 4,903 | 6,548 | |
| 1463 | Iran | 2011-2012 | Amol county study | Community | rural | 10+ | 10+ | 1,862 | 1,098 | |
| 1464 | Iran | 2011-2012 | Amol county study | Community | urban | 10+ | 10+ | 1,624 | 1,548 | |
| 1465 | Iran | 2011-2012 | Childhood and Adolescence Surveillance and Prevention of Adult Noncommunicable Disease (CASPIAN) | National | both | 6-18 | 6-18 | 6,649 | 6,443 | |
| 1466 | Iran | 2012 | National Integrated Micronutrient Survey (NIMS) 2012 | National | both | 6-60 | 6-60 | 10,526 | 11,087 | |
| 1467 | Iran | 2012-2013 | Tehran City | Community | urban | 10-90 | 10-90 | 419 | 537 | |
| 1468 | Iran | 2012-2013 | Zahedan City | Community | urban | 10-90 | 10-90 | 1,377 | 1,205 | |
| 1469 | Iran | 2012-2014 | Pars Cohort Study | Community | rural | 40-90 | 40-90 | 4,272 | 4,987 | |
| 1470 | Iran | 2013-2014 | Bushehr Elderly Health Program (BEH) | Community | urban | 60+ | 60+ | 1,437 | 1,514 | |
| 1471 | Iran | 2013-2014 | Gilan Eye Study | Subnational | both | 50+ | 50+ | 1,059 | 1,439 | |
| 1472 | Iran | 2013-2014 | Isfahan Salt Study (ISS) | Community | urban | 6-18 | 6-18 | 400 | 383 | |
| 1473 | Iran | 2014-2015 | Childhood and Adolescence Surveillance and Prevention of Adult Noncommunicable Disease (CASPIAN) | National | both | 7-18 | 7-18 | 7,164 | 6,966 | |
| 1474 | Iran | 2014-2016 | The PERSIAN Fasa Cohort Study | Community | rural | 35-70 | 35-70 | 3,742 | 4,578 | |
| 1475 | Iran | 2014-2016 | The PERSIAN Fasa Cohort Study | Community | urban | 35-70 | 35-70 | 721 | 806 | |
| 1476 | Iran | 2014-2016 | The PERSIAN Guilan Cohort Study | Community | rural | 35-70 | 35-70 | 2,645 | 3,261 | |
| 1477 | Iran | 2014-2016 | The PERSIAN Guilan Cohort Study | Community | urban | 35-70 | 35-70 | 2,236 | 2,349 | |
| 1478 | Iran | 2014-2016 | The PERSIAN Kermanshah Cohort Study | Community | rural | 35-70 | 35-70 | 1,806 | 2,194 | |
| 1479 | Iran | 2014-2016 | The PERSIAN Kermanshah Cohort Study | Community | urban | 35-70 | 35-70 | 2,940 | 2,973 | |
| 1480 | Iran | 2014-2016 | The PERSIAN Kharameh Cohort Study | Community | rural | 35-70 | 35-70 | 2,913 | 3,860 | |
| 1481 | Iran | 2014-2016 | The PERSIAN Kharameh Cohort Study | Community | urban | 35-70 | 35-70 | 1,794 | 2,000 | |
| 1482 | Iran | 2014-2016 | The PERSIAN Tabriz Cohort Study | Community | rural | 35-70 | 35-70 | 1,974 | 2,548 | |
| 1483 | Iran | 2014-2016 | The PERSIAN Tabriz Cohort Study | Community | urban | 35-70 | 35-70 | 4,670 | 5,592 | |
| 1484 | Iran | 2015 | Iranian School Measurement Database | National | both | 6-18 | 6-18 | 911,584 | 912,687 | |
| 1485 | Iran | 2015-2017 | The PERSIAN Mazandaran Cohort Study | Community | rural | 35-70 | 35-70 | 936 | 1,608 | |
| 1486 | Iran | 2015-2017 | The PERSIAN Mazandaran Cohort Study | Community | urban | 35-70 | 35-70 | 3,179 | 4,421 | |
| 1487 | Iran | 2015-2017 | The PERSIAN Rafsanjan Cohort Study | Community | rural | 35-70 | 35-70 | 1,560 | 1,030 | |

| | Country | Study years | Survey/Study name/Citation | Level of representative-ness | Rural, urban, or both | Age range as in NCD-RisC database | | Sample size | | Notes |
|---|---|---|---|---|---|---|---|---|---|---|
| | | | | | | Male | Female | Male | Female | |
| 1488 | Iran | 2015-2017 | The PERSIAN Rafsanjan Cohort Study | Community | urban | 35-70 | 35-70 | 3,591 | 4,270 | |
| 1489 | Iran | 2015-2017 | The PERSIAN Yazd Cohort Study | Community | urban | 30-70 | 30-70 | 4,947 | 4,818 | |
| 1490 | Iran | 2015-2018 | The PERSIAN Zahedan Cohort Study | Community | urban | 35-70 | 35-70 | 3,890 | 6,013 | |
| 1491 | Iran | 2016 | STEPS | National | both | 18+ | 18+ | 14,080 | 15,036 | |
| 1492 | Iran | 2016-2017 | Iranian Children and Adolescents Psychiatric Disorders (IRCAP) Survey | National | both | 6-18 | 6-18 | 13,049 | 13,476 | |
| 1493 | Iran | 2016-2018 | The PERSIAN Ahvaz Cohort Study | Community | rural | 35-70 | 35-70 | 1,475 | 2,282 | |
| 1494 | Iran | 2016-2018 | The PERSIAN Ahvaz Cohort Study | Community | urban | 35-70 | 35-70 | 2,507 | 3,551 | |
| 1495 | Iran | 2016-2018 | The PERSIAN BandarKong Cohort Study | Community | rural | 35-70 | 35-70 | 232 | 366 | |
| 1496 | Iran | 2016-2018 | The PERSIAN BandarKong Cohort Study | Community | urban | 35-70 | 35-70 | 1,470 | 1,893 | |
| 1497 | Iran | 2016-2018 | The PERSIAN Urmia Cohort Study | Community | rural | 35-70 | 35-70 | 1,750 | 2,323 | |
| 1498 | Iran | 2016-2018 | The PERSIAN Urmia Cohort Study | Community | urban | 35-70 | 35-70 | 411 | 473 | |
| 1499 | Iran | 2016-2019 | The Khuzestan comprehensive health study: A platform for NCDs, blood borne and mental diseases research | Subnational | both | 20-65 | 20-65 | 10,846 | 19,427 | |
| 1500 | Iran | 2016-2019 | The PERSIAN Shahrekord Cohort Study | Community | rural | 35-70 | 35-70 | 1,136 | 1,686 | |
| 1501 | Iran | 2016-2019 | The PERSIAN Shahrekord Cohort Study | Community | urban | 35-70 | 35-70 | 3,450 | 3,474 | |
| 1502 | Iran | 2016-2020 | The PERSIAN Ardabil Cohort Study | Community | urban | 35-70 | 35-70 | 9,501 | 11,200 | |
| 1503 | Iran | 2017-2018 | The PERSIAN Kavar Cohort Study | Community | urban | 35-70 | 35-70 | 2,417 | 2,539 | |
| 1504 | Iran | 2017-2018 | PERSIAN Elderly Component-Iranian Longitudinal Study on Ageing | Subnational | urban | 50-95 | 50-95 | 3,357 | 3,839 | |
| 1505 | Iran | 2017-2019 | The PERSIAN Dena (Yasouj) Cohort Study | Community | rural | 35-70 | 35-70 | 524 | 809 | |
| 1506 | Iran | 2017-2019 | The PERSIAN Dena (Yasouj) Cohort Study | Community | urban | 35-70 | 35-70 | 918 | 1,083 | |
| 1507 | Iran | 2017-2019 | The PERSIAN Sabzevar Cohort Study | Community | urban | 35-70 | 35-70 | 1,874 | 2,320 | |
| 1508 | Iran | 2018-2019 | Prevalence of risk factors for cardiovascular disease among a rural population in eastern Iran | Community | rural | 18-69 | 18-69 | 148 | 146 | |
| 1509 | Iran | 2018-2019 | The PERSIAN Dehgolan (Kordistan) Cohort Study | Community | urban | 35-70 | 35-70 | 1,741 | 2,197 | |
| 1510 | Iran | 2020-2021 | STEPS | National | both | 18+ | 18+ | 12,402 | 15,333 | |
| 1511 | Iraq | 2006 | STEPS | National | both | 25-64 | 25-64 | 2,251 | 2,252 | |
| 1512 | Iraq | 2013-2014 | Qadir et al., Malays J Med Health Sci, 10(2):27-38, 2014 | Community | urban | 13-17 | 13-17 | 832 | 748 | |
| 1513 | Iraq | 2015 | STEPS | National | both | 18+ | 18+ | 1,589 | 2,312 | |
| 1514 | Ireland | 1997-1999 | North/South Ireland Food Consumption Survey | National | both | 18-64 | 18-64 | 613 | 698 | |
| 1515 | Ireland | 1998 | Survey of Lifestyle, Attitudes and Nutritional in Ireland 1998 | National | both | 18+ | 18+ | 123 | 296 | |
| 1516 | Ireland | 2002 | Survey of Lifestyle, Attitudes and Nutritional in Ireland 2002 | National | both | 18+ | 18+ | 164 | 216 | |
| 1517 | Ireland | 2003-2004 | National Children's Food Survey | National | both | 5-12 | 5-12 | 293 | 301 | |
| 1518 | Ireland | 2005-2006 | National Teens Food Survey | National | both | 13-17 | 13-17 | 224 | 216 | |
| 1519 | Ireland | 2006-2007 | Survey of Lifestyle, Attitudes and Nutritional in Ireland 2006-2007 | National | both | 18+ | 18+ | 945 | 1,225 | |
| 1520 | Ireland | 2007-2008 | Growing Up in Ireland - Child Cohort | National | both | 9 | 9 | 3,899 | 4,107 | 15 |
| 1521 | Ireland | 2008 | Childhood Obesity Surveillance Initiative 1 | National | both | 7 | 7 | 1,098 | 1,285 | |
| 1522 | Ireland | 2008-2010 | National Adult Nutrition Survey | National | both | 18+ | 18+ | 658 | 696 | |
| 1523 | Ireland | 2009-2011 | The Irish Longitudinal Study on Ageing | National | both | 50+ | 50+ | 2,693 | 3,170 | |
| 1524 | Ireland | 2010 | Murtagh et al., Pediatr Exerc Sci 25(2):300-7, 2013 | Community | rural | 7-12 | 7-12 | 20 | 11 | |
| 1525 | Ireland | 2010 | Murtagh et al., Pediatr Exerc Sci 25(2):300-7, 2013 | Community | rural | 7-12 | 7-12 | 19 | 19 | |
| 1526 | Ireland | 2010 | Murtagh et al., Pediatr Exerc Sci 25(2):300-7, 2013 | Community | rural | 7-12 | 7-12 | 14 | 12 | |
| 1527 | Ireland | 2010 | Murtagh et al., Pediatr Exerc Sci 25(2):300-7, 2013 | Community | rural | 7-12 | 7-12 | 16 | 21 | |
| 1528 | Ireland | 2010 | Childhood Obesity Surveillance Initiative 2 | National | both | 6-9 | 6-9 | 1,452 | 1,533 | |
| 1529 | Ireland | 2011-2012 | Growing Up in Ireland - Child Cohort | National | both | 13 | 13 | 3,537 | 3,661 | 15 |
| 1530 | Ireland | 2012-2013 | Childhood Obesity Surveillance Initiative 3 | National | both | 6-9 | 6-9 | 1,087 | 1,054 | |
| 1531 | Ireland | 2013 | Growing Up in Ireland - Infant Cohort | National | both | 5 | 5 | 4,498 | 4,382 | 15 |
| 1532 | Ireland | 2013-2016 | Project Spraoi | Community | both | 5-11 | 5-11 | 474 | 429 | |
| 1533 | Ireland | 2015 | Active Classrooms Study | Community | both | 8-11 | 8-11 | 124 | 120 | |
| 1534 | Ireland | 2015-2016 | Childhood Obesity Surveillance Initiative 4 | National | both | 6-10 | 6-10 | 1,441 | 1,679 | |
| 1535 | Ireland | 2015-2016 | Growing Up in Ireland - Child Cohort | National | both | 18 | 18 | 551 | 583 | 15 |
| 1536 | Ireland | 2016 | Growing Up in Ireland - Infant Cohort | National | both | 7-8 | 7-8 | 2,016 | 1,921 | 15 |
| 1537 | Ireland | 2017-2018 | Growing Up in Ireland - Infant Cohort | National | both | 9 | 9 | 3,943 | 3,892 | 15 |
| 1538 | Ireland | 2017-2018 | National Children's Food Survey II | National | both | 5-12 | 5-12 | 298 | 298 | |
| 1539 | Ireland | 2018-2019 | Childhood Obesity Surveillance Initiative 5 | National | both | 6-12 | 6-12 | 2,793 | 2,768 | |
| 1540 | Ireland | 2018-2019 | Growing Up in Ireland - Child Cohort | National | both | 20 | 20 | 2,202 | 2,352 | 15 |
| 1541 | Ireland | 2019-2020 | National Teens' Food Survey II | National | both | 13-18 | 13-18 | 211 | 214 | |
| 1542 | Israel | 1975-1979 | Israeli Conscripts | National | both | 16-19 | 16-19 | 125,532 | 73,424 | 1 |
| 1543 | Israel | 1980-1984 | Israeli Conscripts | National | both | 16-19 | 16-19 | 133,146 | 85,931 | |

| | Country | Study years | Survey/Study name/Citation | Level of representative-ness | Rural, urban, or both | Age range as in NCD-RisC database | | Sample size | | Notes |
|---|---|---|---|---|---|---|---|---|---|---|
| | | | | | | Male | Female | Male | Female | |
| 1544 | Israel | 1985-1986 | MONICA, Tel Aviv | Community | urban | 25-64 | 25-64 | 653 | 685 | |
| 1545 | Israel | 1985-1989 | Israeli Conscripts | National | both | 16-19 | 16-19 | 148,878 | 105,833 | |
| 1546 | Israel | 1990-1991 | The Jerusalem Longitudinal Cohort Study | Community | urban | 69-70 | 69-70 | 245 | 199 | |
| 1547 | Israel | 1990-1994 | Israeli Conscripts | National | both | 16-19 | 16-19 | 191,052 | 139,555 | |
| 1548 | Israel | 1995-1999 | Israeli Conscripts | National | both | 16-19 | 16-19 | 202,608 | 146,585 | |
| 1549 | Israel | 1997-1998 | The Jerusalem Longitudinal Cohort Study | Community | urban | 76-77 | 76-77 | 422 | 429 | |
| 1550 | Israel | 1999-2001 | Mabat First Israeli National Health and Nutrition Survey | National | both | 25-64 | 25-64 | 1,371 | 1,410 | |
| 1551 | Israel | 1999-2005 | The Israel Glucose Intolerance, Obesity and Hypertension Study (GOH) | National | urban | 58-93 | 58-93 | 511 | 527 | |
| 1552 | Israel | 2000-2004 | Israeli Conscripts | National | both | 16-19 | 16-19 | 202,713 | 152,674 | |
| 1553 | Israel | 2002-2008 | The Hadera District Study (HDS) | Subnational | urban | 25-78 | 25-78 | 385 | 369 | |
| 1554 | Israel | 2003-2004 | Mabat Youth First Israeli National Health and Nutrition Survey in 7th-12th grade students | National | both | 12-18 | 12-18 | 2,553 | 3,073 | |
| 1555 | Israel | 2005-2006 | The Jerusalem Longitudinal Cohort Study | Community | urban | 83-85 | 83-85 | 490 | 584 | |
| 1556 | Israel | 2005-2006 | Mabat Zahav First National Health and Nutrition Survey in ages 65 and over | National | urban | 65+ | 65+ | 743 | 819 | |
| 1557 | Israel | 2005-2009 | Israeli Conscripts | National | both | 16-19 | 16-19 | 192,835 | 146,308 | |
| 1558 | Israel | 2010-2011 | The Jerusalem Longitudinal Cohort Study | Community | urban | 89-92 | 89-92 | 185 | 201 | |
| 1559 | Israel | 2010-2014 | Israeli Conscripts | National | both | 16-19 | 16-19 | 213,094 | 148,908 | |
| 1560 | Israel | 2014-2015 | Mabat Zahav Second National Health and Nutrition Survey ages in 65 and over | National | both | 65+ | 65+ | 307 | 318 | |
| 1561 | Israel | 2014-2016 | Mabat Second Israeli National Health and Nutrition Survey | National | both | 18-64 | 18-64 | 1,061 | 1,073 | |
| 1562 | Israel | 2015-2016 | The Jerusalem Longitudinal Cohort Study | Community | urban | 94-96 | 94-96 | 50 | 65 | |
| 1563 | Israel | 2015-2016 | Rav Mabat Kids - First National Health and Nutrition Survey in 2-11 year olds | National | rural | 2-11 | 2-11 | 73 | 88 | |
| 1564 | Israel | 2015-2016 | Rav Mabat Kids - First National Health and Nutrition Survey in 2-11 year olds | National | urban | 2-11 | 2-11 | 515 | 463 | |
| 1565 | Israel | 2015-2016 | Mabat Youth Second Israeli National Health and Nutrition Survey in 7th-12th grade students | National | both | 12-18 | 12-18 | 2,221 | 2,415 | |
| 1566 | Israel | 2015-2019 | Israeli Conscripts | National | both | 16-19 | 16-19 | 199,488 | 152,571 | |
| 1567 | Israel | 2018-2019 | Childhood Obesity Surveillance Initiative 5 | National | both | 6-7 | 6-7 | 73,724 | 73,561 | |
| 1568 | Italy | 1980-1982 | Po river delta epidemiological study - first survey | Community | rural | 8-64 | 8-64 | 1,573 | 1,710 | |
| 1569 | Italy | 1982-1987 | MONICA, Latina | Community | both | 24-66 | 24-66 | 852 | 868 | |
| 1570 | Italy | 1983-1984 | Malattie cardiovascolari ATerosclerotiche Istituto Superiore di Sanità (MATISS) | Community | rural | 19-69 | 19-69 | 1,709 | 1,921 | |
| 1571 | Italy | 1983-1985 | Gubbio Study | Community | both | 5+ | 5+ | 2,431 | 2,749 | |
| 1572 | Italy | 1985 | Finland, Italy, Netherlands, Elderly (Fine-Italy) | Community | rural | 65-84 | | 650 | | |
| 1573 | Italy | 1985 | INTERSALT, Naples | Community | urban | 20-59 | 20-59 | 100 | 100 | |
| 1574 | Italy | 1985-1988 | Pisa epidemiological study - first survey | Community | urban | 5-90 | 5-90 | 1,834 | 2,019 | |
| 1575 | Italy | 1986 | INTERSALT, Bassiano | Community | urban | 20-59 | 20-59 | 99 | 100 | |
| 1576 | Italy | 1986 | INTERSALT, Mirano | Community | urban | 20-59 | 20-59 | 100 | 100 | |
| 1577 | Italy | 1986 | MONICA, Friuli | Subnational | both | 25-64 | 25-64 | 921 | 918 | |
| 1578 | Italy | 1986-1987 | Malattie cardiovascolari ATerosclerotiche Istituto Superiore di Sanità (MATISS) | Community | rural | 19-72 | 19-72 | 1,273 | 1,568 | |
| 1579 | Italy | 1986-1987 | MONICA, Brianza | Subnational | urban | 25-64 | 25-64 | 814 | 832 | |
| 1580 | Italy | 1988-1991 | Po river delta epidemiological study - second survey | Community | rural | 8-73 | 8-73 | 1,341 | 1,497 | |
| 1581 | Italy | 1989 | MONICA, Friuli | Subnational | both | 25-64 | 25-64 | 902 | 900 | |
| 1582 | Italy | 1989 | Ventimiglia Heart Study | Community | rural | 5+ | 5+ | 603 | 701 | |
| 1583 | Italy | 1989-1990 | MONICA, Brianza | Subnational | urban | 25-64 | 25-64 | 787 | 786 | |
| 1584 | Italy | 1989-1992 | Gubbio Study | Community | both | 10+ | 10+ | 1,677 | 1,958 | |
| 1585 | Italy | 1990 | Bruneck Study | Community | rural | 40-79 | 40-79 | 469 | 450 | |
| 1586 | Italy | 1991 | Finland, Italy, Netherlands, Elderly (Fine-Italy) | Community | rural | 70-90 | | 389 | | |
| 1587 | Italy | 1991-1993 | Pisa epidemiological study - second survey | Community | urban | 8-97 | 8-97 | 1,288 | 1,553 | |
| 1588 | Italy | 1992-1993 | Italian Longitudinal Study on Aging | National | both | 65-84 | 65-84 | 1,666 | 1,455 | |
| 1589 | Italy | 1992-1998 | Vobarno Study | Community | both | 25-64 | 35-64 | 265 | 309 | |
| 1590 | Italy | 1993-1994 | MONICA, Brianza | Subnational | urban | 25-64 | 25-64 | 801 | 856 | |
| 1591 | Italy | 1993-1996 | Malattie cardiovascolari ATerosclerotiche Istituto Superiore di Sanità (MATISS) | Community | rural | 20-77 | 20-77 | 965 | 999 | |
| 1592 | Italy | 1993-1998 | EPIC Florence | Community | urban | 24-72 | 24-72 | 3,498 | 9,968 | |
| 1593 | Italy | 1994 | MONICA, Friuli | Subnational | both | 25-64 | 25-64 | 882 | 888 | |
| 1594 | Italy | 1995 | Bruneck Study | Community | rural | 45-84 | 45-84 | 411 | 408 | |
| 1595 | Italy | 1995-1996 | Friuli Studio Emostatico | Community | urban | 45-64 | 45-64 | 198 | 198 | |
| 1596 | Italy | 1995-1996 | Italian Longitudinal Study on Aging | National | both | 68-90 | 68-90 | 1,011 | 808 | |
| 1597 | Italy | 1995-1999 | PROgetto Veneto Anziani (PROVA) | Subnational | both | 65+ | 65+ | 1,187 | 1,722 | |
| 1598 | Italy | 1997-1999 | Lucca CUORE Study | Community | urban | 15-84 | 15-84 | 897 | 1,123 | |
| 1599 | Italy | 1998-1999 | Progetto VIP | Community | both | 25-74 | 25-74 | 599 | 600 | |
| 1600 | Italy | 1998-2000 | InCHIANTI study | Community | both | 15+ | 15+ | 560 | 681 | |

| | Country | Study years | Survey/Study name/Citation | Level of representative-ness | Rural, urban, or both | Age range as in NCD-RisC database | | Sample size | | Notes |
|---|---|---|---|---|---|---|---|---|---|---|
| | | | | | | Male | Female | Male | Female | |
| 1601 | Italy | 1998-2002 | Osservatorio Epidemiologico Cardiovascolare (OEC) | National | both | 35-74 | 35-74 | 4,870 | 4,752 | |
| 1602 | Italy | 2000 | Bruneck Study | Community | rural | 50-89 | 50-89 | 331 | 361 | |
| 1603 | Italy | 2000 | Sorveglianza Nutrizionale Infanzia e Adolescenza (SoNIA) | Subnational | both | 13-15 | 13-15 | 236 | 244 | |
| 1604 | Italy | 2000-2001 | Italian Longitudinal Study on Aging | National | both | 73-93 | 73-93 | 557 | 473 | |
| 1605 | Italy | 2000-2001 | Sorveglianza Nutrizionale Infanzia e Adolescenza (SoNIA) | Subnational | both | 8-9 | 8-9 | 444 | 413 | |
| 1606 | Italy | 2000-2003 | PROgetto Veneto Anziani (PROVA) | Subnational | both | 67+ | 67+ | 795 | 1,331 | |
| 1607 | Italy | 2001-2003 | The Study of Asti | Community | both | 45-64 | 45-64 | 780 | 878 | |
| 1608 | Italy | 2001-2007 | Gubbio Study | Community | both | 26+ | 26+ | 1,187 | 1,454 | |
| 1609 | Italy | 2002-2005 | PROgetto Veneto Anziani (PROVA) | Subnational | both | 68+ | 68+ | 621 | 1,138 | |
| 1610 | Italy | 2003 | The European Male Ageing Study | Community | both | 40+ | | 433 | | |
| 1611 | Italy | 2003 | Sorveglianza Nutrizionale Infanzia e Adolescenza (SoNIA) | Subnational | both | 5-6 | 5-6 | 1,355 | 1,327 | |
| 1612 | Italy | 2004-2005 | Italian Project on the Epidemiology of Alzheimer's Disease | National | both | 65-84 | 65-84 | 1,569 | 1,421 | |
| 1613 | Italy | 2004-2005 | Vobarno study | Community | rural | 55-74 | 55-74 | 99 | 113 | |
| 1614 | Italy | 2004-2008 | Cardiolab project | National | urban | 40+ | 40+ | 19,022 | 14,526 | |
| 1615 | Italy | 2005 | Bruneck Study | Community | rural | 55-93 | 55-93 | 264 | 307 | |
| 1616 | Italy | 2005-2007 | Moli-family Study | Subnational | both | 14+ | 14+ | 243 | 301 | |
| 1617 | Italy | 2005-2010 | Moli-sani Study | Subnational | both | 35+ | 35+ | 11,693 | 12,614 | |
| 1618 | Italy | 2006-2007 | HELENA | Community | urban | 12-17 | 12-17 | 119 | 185 | |
| 1619 | Italy | 2007-2010 | Identification and prevention of Dietary- and lifestyle-induced health EFfects In Children and infantS (IDEFICS) | Community | urban | 5-9 | 5-9 | 893 | 847 | |
| 1620 | Italy | 2008 | Childhood Obesity Surveillance Initiative 1 | National | both | 8-9 | 8-9 | 4,100 | 3,896 | |
| 1621 | Italy | 2008 | The European Male Ageing Study | Community | both | 40+ | | 346 | | |
| 1622 | Italy | 2008-2009 | Progetto VIP | Community | both | 25-74 | 25-74 | 597 | 598 | |
| 1623 | Italy | 2008-2012 | Osservatorio Epidemiologico Cardiovascolare/Health Examination Survey (OEC/HES) | National | both | 35-80 | 35-80 | 4,368 | 4,332 | |
| 1624 | Italy | 2009 | The ZOOM8 study: nutrition and physical activity of primary school children | National | both | 6-11 | 6-11 | 1,083 | 1,045 | |
| 1625 | Italy | 2009-2010 | Grosso et al., J Epidemiol 24(4):327-33, 2014 | Community | both | 19+ | 19+ | 760 | 1,129 | |
| 1626 | Italy | 2009-2011 | Pisa epidemiological study - third survey | Community | urban | 6+ | 6+ | 496 | 574 | |
| 1627 | Italy | 2010 | Bruneck Study | Community | rural | 60+ | 60+ | 225 | 259 | |
| 1628 | Italy | 2010 | Childhood Obesity Surveillance Initiative 2 | National | both | 8-9 | 8-9 | 21,474 | 20,190 | |
| 1629 | Italy | 2010-2012 | CArdiovascular risk MEtabolic syndrome LIver and Autoimmunity diseases (CA.ME.LI.A) | Community | both | 18-75 | 18-75 | 477 | 515 | |
| 1630 | Italy | 2011 | Grosso et al., Nutrients 5(12):4908-23, 2013 | Community | rural | 13-16 | 13-16 | 115 | 89 | |
| 1631 | Italy | 2011 | Grosso et al., Nutrients 5(12):4908-23, 2013 | Community | urban | 13-16 | 13-16 | 512 | 419 | |
| 1632 | Italy | 2011 | CONVERGI Study | Community | urban | 13-19 | 13-19 | 159 | 269 | |
| 1633 | Italy | 2011-2012 | ALImentazione e stile di vita negli ADOlescenti (ALIADO) | Subnational | both | 15-16 | 15-16 | 149 | 194 | |
| 1634 | Italy | 2011-2012 | Vobarno study | Community | rural | 49-62 | 49-62 | 107 | 143 | |
| 1635 | Italy | 2012 | Childhood Obesity Surveillance Initiative 3 | National | both | 8-9 | 8-9 | 23,137 | 22,405 | |
| 1636 | Italy | 2012-2014 | Mistretta et al., Obes Res Clin Pract 11(2):215-226, 2017 | Community | urban | 11-16 | 11-16 | 878 | 753 | |
| 1637 | Italy | 2014 | OKkio alla SALUTE | National | both | 8-9 | 8-9 | 24,457 | 22,855 | |
| 1638 | Italy | 2014-2016 | Mediterranean healthy Eating, Aging and Lifestyles (MEAL) study | Subnational | both | 20+ | 20+ | 762 | 1,130 | |
| 1639 | Italy | 2015 | Bruneck Study | Community | rural | 65+ | 65+ | 171 | 169 | |
| 1640 | Italy | 2016 | Childhood Obesity Surveillance Initiative 4 | National | both | 8-9 | 8-9 | 22,732 | 21,454 | |
| 1641 | Italy | 2016 | The Tyrolean Early Vascular Ageing-study (EVA-Tyrol) - South-Tyrol | Subnational | both | 14-18 | 14-18 | 108 | 200 | |
| 1642 | Italy | 2017-2020 | Moli-sani Study | Subnational | both | 47-94 | 47-94 | 1,160 | 1,422 | |
| 1643 | Italy | 2018-2019 | Progetto VIP | Community | both | 25-74 | 25-74 | 600 | 598 | |
| 1644 | Italy | 2018-2019 | Health Examination Survey 2018-2019 - CUORE Project | National | both | 35-74 | 35-74 | 1,035 | 1,060 | |
| 1645 | Italy | 2019 | Childhood Obesity Surveillance Initiative 5 | National | both | 8-9 | 8-9 | 23,432 | 22,462 | |
| 1646 | Jamaica | 1993 | Zohoori et al., West Indian Med J 52(2):111-17, 2003 | Community | urban | 25-74 | 25-74 | 845 | 1,245 | |
| 1647 | Jamaica | 1994-1995 | Cooper et al., Am J Public Health 87(2):160-68, 1997 | Community | urban | 25-100 | 25-100 | 597 | 833 | |
| 1648 | Jamaica | 1998 | Ragoobirsingh et al., Diabetes Obes Metab 6(1):23-27, 2004 | National | both | 15+ | 15+ | 552 | 945 | |
| 1649 | Jamaica | 2000-2001 | Jamaica Health and Lifestyle Survey | National | both | 15-74 | 15-74 | 653 | 1,281 | |
| 1650 | Jamaica | 2005 | Jamaica Youth Risk and Resiliency Behaviour Survey 2005 | National | both | 10-15 | 10-15 | 1,328 | 1,386 | |
| 1651 | Jamaica | 2006-2007 | Jamaica Youth Risk and Resiliency Behaviour Survey 2006 | National | both | 15-19 | 15-19 | 585 | 701 | |
| 1652 | Jamaica | 2007-2008 | Jamaica Health and Lifestyle Survey | National | both | 15-74 | 15-74 | 862 | 1,904 | |
| 1653 | Jamaica | 2010 | Global School-based Student Health Survey | National | both | | 13-17 | | 787 | |
| 1654 | Jamaica | 2012 | Older Persons in Jamaica 2012 | National | both | 60+ | 60+ | 158 | 205 | 16 |
| 1655 | Jamaica | 2016-2017 | Jamaica Health and Lifestyle Survey | National | both | 15+ | 15+ | 939 | 1,491 | |
| 1656 | Japan | 1977 | National Nutrition Survey | National | both | 5+ | 5+ | 6,455 | 8,094 | 1 |

| | Country | Study years | Survey/Study name/Citation | Level of representative-ness | Rural, urban, or both | Age range as in NCD-RisC database | | Sample size | | Notes |
|---|---|---|---|---|---|---|---|---|---|---|
| | | | | | | Male | Female | Male | Female | |
| 1657 | Japan | 1978 | National Nutrition Survey | National | both | 5+ | 5+ | 6,979 | 8,694 | 1 |
| 1658 | Japan | 1979 | National Nutrition Survey | National | both | 5+ | 5+ | 7,249 | 9,031 | 1 |
| 1659 | Japan | 1980 | National Cardiovascular Survey | National | both | 5+ | 5+ | 7,955 | 9,437 | |
| 1660 | Japan | 1980-1983 | Aito Town Study | Community | rural | 20-77 | 20-77 | 741 | 970 | |
| 1661 | Japan | 1981 | National Nutrition Survey | National | both | 5+ | 5+ | 5,922 | 7,828 | |
| 1662 | Japan | 1982 | National Nutrition Survey | National | both | 5+ | 5+ | 6,845 | 8,831 | |
| 1663 | Japan | 1983 | National Nutrition Survey | National | both | 5+ | 5+ | 6,609 | 8,478 | |
| 1664 | Japan | 1984 | National Nutrition Survey | National | both | 5+ | 5+ | 6,389 | 8,072 | |
| 1665 | Japan | 1985 | INTERSALT, Osaka | Community | urban | 20-59 | 20-59 | 100 | 97 | |
| 1666 | Japan | 1985 | INTERSALT, Tochigi | Community | urban | 20-59 | 20-59 | 95 | 99 | |
| 1667 | Japan | 1985 | INTERSALT, Toyama | Community | urban | 20-59 | 20-59 | 100 | 100 | |
| 1668 | Japan | 1985 | National Nutrition Survey | National | both | 5+ | 5+ | 7,461 | 8,865 | |
| 1669 | Japan | 1985-1986 | Akabane Study | Community | urban | 40-69 | 40-69 | 812 | 1,022 | |
| 1670 | Japan | 1986 | National Nutrition Survey | National | both | 5+ | 5+ | 7,280 | 8,635 | |
| 1671 | Japan | 1987 | Konan Town Study | Community | rural | 20-79 | 20-79 | 70 | 88 | |
| 1672 | Japan | 1987 | National Nutrition Survey | National | both | 5+ | 5+ | 6,427 | 8,160 | |
| 1673 | Japan | 1988 | Konan Town Study | Community | rural | 20-79 | 20-79 | 76 | 85 | |
| 1674 | Japan | 1988 | The Hisayama Study | Community | rural | 40+ | 40+ | 1,165 | 1,573 | |
| 1675 | Japan | 1988 | National Nutrition Survey | National | both | 5+ | 5+ | 6,885 | 8,045 | |
| 1676 | Japan | 1989 | Aito Town Study | Community | rural | 5-74 | 5-84 | 529 | 525 | |
| 1677 | Japan | 1989 | Konan Town Study | Community | rural | 20-79 | 20-79 | 58 | 63 | |
| 1678 | Japan | 1989 | National Nutrition Survey | National | both | 5+ | 5+ | 5,767 | 6,882 | |
| 1679 | Japan | 1990 | Konan Town Study | Community | rural | 20-79 | 20-79 | 27 | 51 | |
| 1680 | Japan | 1990 | National Nutrition Survey and National Cardiovascular Survey | National | both | 5+ | 5+ | 6,080 | 7,291 | |
| 1681 | Japan | 1990-1994 | Japan Public Health Center-based prospective Study (JPHC Study), Cohort I | Subnational | both | 40-59 | 40-59 | 8,749 | 14,481 | |
| 1682 | Japan | 1991 | Konan Town Study | Community | rural | 20-79 | 20-79 | 93 | 116 | |
| 1683 | Japan | 1991 | Shigaraki Town Study | Community | rural | 30-89 | 30-89 | 230 | 319 | |
| 1684 | Japan | 1991 | National Nutrition Survey | National | both | 5+ | 5+ | 6,036 | 7,098 | |
| 1685 | Japan | 1992 | Konan Town Study | Community | rural | 20-79 | 20-79 | 45 | 47 | |
| 1686 | Japan | 1992 | Shigaraki Town Study | Community | rural | 30-89 | 30-89 | 288 | 385 | |
| 1687 | Japan | 1992 | National Nutrition Survey | National | both | 5+ | 5+ | 5,635 | 6,656 | |
| 1688 | Japan | 1993 | Konan Town Study | Community | rural | 20-79 | 20-79 | 54 | 65 | |
| 1689 | Japan | 1993 | Shigaraki Town Study | Community | rural | 30-89 | 30-89 | 301 | 452 | |
| 1690 | Japan | 1993 | Iwata kids health study | Community | urban | 10 | 10 | 513 | 485 | |
| 1691 | Japan | 1993 | National Nutrition Survey | National | both | 5+ | 5+ | 5,708 | 6,740 | |
| 1692 | Japan | 1993-1994 | Japan Public Health Center-based prospective Study (JPHC Study), Cohort II | Subnational | both | 40-69 | 40-69 | 8,534 | 16,190 | |
| 1693 | Japan | 1994 | Japanese Population-Based Osteoporosis Study | Subnational | both | | 15-79 | | 3,222 | |
| 1694 | Japan | 1994 | Konan Town Study | Community | rural | 20-79 | 20-79 | 43 | 59 | |
| 1695 | Japan | 1994 | Shigaraki Town Study | Community | rural | 30-89 | 30-89 | 251 | 336 | |
| 1696 | Japan | 1994 | Iwata kids health study | Community | urban | 10 | 10 | 569 | 567 | |
| 1697 | Japan | 1994 | National Nutrition Survey | National | both | 5+ | 5+ | 5,439 | 6,386 | |
| 1698 | Japan | 1995 | Konan Town Study | Community | rural | 20-79 | 20-79 | 45 | 61 | |
| 1699 | Japan | 1995 | Shigaraki Town Study | Community | rural | 30-89 | 30-89 | 300 | 470 | |
| 1700 | Japan | 1995 | Iwata kids health study | Community | urban | 10 | 10 | 524 | 567 | |
| 1701 | Japan | 1995 | National Nutrition Survey | National | both | 5+ | 5+ | 5,480 | 6,365 | |
| 1702 | Japan | 1996 | Shigaraki Town Study | Community | rural | 30-89 | 30-89 | 86 | 152 | |
| 1703 | Japan | 1996 | Iwata kids health study | Community | urban | 10 | 10 | 552 | 480 | |
| 1704 | Japan | 1996 | National Nutrition Survey | National | both | 5+ | 5+ | 5,277 | 6,185 | |
| 1705 | Japan | 1996-1997 | INTERMAP, AitoTown | Community | rural | 40-59 | 40-59 | 130 | 129 | |
| 1706 | Japan | 1997 | Shigaraki Town Study | Community | rural | 30-89 | 30-89 | 61 | 100 | |
| 1707 | Japan | 1997 | Iwata kids health study | Community | urban | 10 | 10 | 506 | 537 | |
| 1708 | Japan | 1997 | National Nutrition Survey | National | both | 5+ | 5+ | 5,104 | 6,068 | |
| 1709 | Japan | 1997-1998 | INTERMAP, Sapporo | Community | urban | 40-59 | 40-59 | 149 | 148 | |
| 1710 | Japan | 1997-1998 | INTERMAP, Toyama | Community | urban | 40-59 | 40-59 | 149 | 150 | |
| 1711 | Japan | 1997-1998 | INTERMAP, Wakayama | Community | urban | 40-59 | 40-59 | 146 | 144 | |
| 1712 | Japan | 1997-2000 | Sudo et al., J Orthop Sci 13(5):413-18, 2008 | Community | rural | 55+ | 45+ | 261 | 785 | |
| 1713 | Japan | 1998 | Iwata kids health study | Community | urban | 10 | 10 | 527 | 464 | |

| | Country | Study years | Survey/Study name/Citation | Level of representative-ness | Rural, urban, or both | Age range as in NCD-RisC database | | Sample size | | Notes |
|---|---|---|---|---|---|---|---|---|---|---|
| | | | | | | Male | Female | Male | Female | |
| 1714 | Japan | 1998 | Niigata Study | Community | urban | 70 | 70 | 287 | 284 | |
| 1715 | Japan | 1998 | National Nutrition Survey | National | both | 5+ | 5+ | 5,381 | 6,249 | |
| 1716 | Japan | 1999 | Iwata kids health study | Community | urban | 10 | 10 | 468 | 463 | |
| 1717 | Japan | 1999 | Niigata Study | Community | urban | 71 | 71 | 245 | 216 | |
| 1718 | Japan | 1999 | National Nutrition Survey | National | both | 5+ | 5+ | 4,367 | 5,333 | |
| 1719 | Japan | 2000 | Iwata kids health study | Community | urban | 10 | 10 | 440 | 401 | |
| 1720 | Japan | 2000 | Niigata Study | Community | urban | 72 | 72 | 233 | 202 | |
| 1721 | Japan | 2000 | National Nutrition Survey and National Cardiovascular Survey | National | both | 5+ | 5+ | 4,665 | 5,430 | |
| 1722 | Japan | 2001 | Iwata kids health study | Community | urban | 10 | 10 | 452 | 414 | |
| 1723 | Japan | 2001 | The Japan Association of Health Service Database | Subnational | both | 20+ | 20+ | 1,471,868 | 1,231,378 | |
| 1724 | Japan | 2001 | Niigata Study | Community | urban | 73 | 73 | 235 | 201 | |
| 1725 | Japan | 2001 | National Nutrition Survey | National | both | 5+ | 5+ | 4,527 | 5,448 | |
| 1726 | Japan | 2002 | Iwata kids health study | Community | urban | 10 | 10 | 496 | 398 | |
| 1727 | Japan | 2002 | Niigata Study | Community | urban | 74 | 74 | 228 | 202 | |
| 1728 | Japan | 2002 | National Nutrition Survey | National | both | 5+ | 5+ | 4,104 | 4,941 | |
| 1729 | Japan | 2002-2003 | The Hisayama Study | Community | rural | 40+ | 40+ | 1,414 | 1,884 | |
| 1730 | Japan | 2003 | Iwata kids health study | Community | urban | 10 | 10 | 415 | 399 | |
| 1731 | Japan | 2003 | National Health and Nutrition Survey | National | both | 5+ | 5+ | 4,035 | 4,920 | |
| 1732 | Japan | 2003 | Niigata Study | Community | urban | 75 | 75 | 215 | 189 | |
| 1733 | Japan | 2004 | Iwata kids health study | Community | urban | 10 | 10 | 463 | 412 | |
| 1734 | Japan | 2004 | National Health and Nutrition Survey | National | both | 5+ | 5+ | 3,384 | 3,952 | |
| 1735 | Japan | 2004 | Niigata Study | Community | urban | 76 | 76 | 215 | 185 | |
| 1736 | Japan | 2005 | Iwata kids health study | Community | urban | 10 | 10 | 476 | 420 | |
| 1737 | Japan | 2005 | National Health and Nutrition Survey | National | both | 5+ | 5+ | 3,154 | 3,802 | |
| 1738 | Japan | 2005 | Niigata Study | Community | urban | 77 | 77 | 203 | 184 | |
| 1739 | Japan | 2006 | Iwata kids health study | Community | urban | 10 | 10 | 417 | 391 | |
| 1740 | Japan | 2006 | National Health and Nutrition Survey | National | both | 5+ | 5+ | 3,522 | 4,165 | |
| 1741 | Japan | 2006 | Niigata Study | Community | urban | 78 | 78 | 199 | 194 | |
| 1742 | Japan | 2007 | Fukuroi kids health study | Community | urban | 13-14 | 13-14 | 395 | 372 | |
| 1743 | Japan | 2007 | Iwata kids health study | Community | urban | 10 | 10 | 439 | 394 | |
| 1744 | Japan | 2007 | National Health and Nutrition Survey | National | both | 5+ | 5+ | 3,520 | 4,154 | |
| 1745 | Japan | 2007 | Niigata Study | Community | urban | 79 | 79 | 183 | 192 | |
| 1746 | Japan | 2008 | Resident in Kanazawa City age 40+ | Community | urban | 40+ | 40+ | 6,562 | 11,944 | |
| 1747 | Japan | 2008 | Fukuroi kids health study | Community | urban | 13-14 | 13-14 | 381 | 346 | |
| 1748 | Japan | 2008 | Iwata kids health study | Community | urban | 10 | 10 | 406 | 417 | |
| 1749 | Japan | 2008 | MEXT School Health Statistics | National | both | 5-17 | 5-17 | 326,401 | 326,957 | |
| 1750 | Japan | 2008 | National Health and Nutrition Survey | National | both | 5+ | 5+ | 3,518 | 4,190 | |
| 1751 | Japan | 2008 | Niigata Study | Community | urban | 80 | 80 | 174 | 180 | |
| 1752 | Japan | 2009 | Fukuroi kids health study | Community | urban | 13-14 | 13-14 | 388 | 357 | |
| 1753 | Japan | 2009 | MEXT School Health Statistics | National | both | 5-17 | 5-17 | 326,523 | 327,097 | |
| 1754 | Japan | 2009 | National Health and Nutrition Survey | National | both | 5+ | 5+ | 3,486 | 4,197 | |
| 1755 | Japan | 2010 | Fukuroi kids health study | Community | urban | 13-14 | 13-14 | 360 | 387 | |
| 1756 | Japan | 2010 | MEXT School Health Statistics | National | both | 5-17 | 5-17 | 326,508 | 326,401 | |
| 1757 | Japan | 2010 | National Health and Nutrition Survey | National | both | 5+ | 5+ | 3,218 | 3,822 | |
| 1758 | Japan | 2011 | Fukuroi kids health study | Community | urban | 13-14 | 13-14 | 402 | 369 | |
| 1759 | Japan | 2011 | MEXT School Health Statistics | National | both | 5-17 | 5-17 | 305,267 | 306,382 | |
| 1760 | Japan | 2011 | National Health and Nutrition Survey | National | both | 5+ | 5+ | 3,020 | 3,586 | |
| 1761 | Japan | 2011 | The Tokyo Health Service Association Database | Community | urban | 20+ | 20+ | 82,453 | 54,028 | |
| 1762 | Japan | 2012 | Fukuroi kids health study | Community | urban | 13-14 | 13-14 | 432 | 353 | |
| 1763 | Japan | 2012 | MEXT School Health Statistics | National | both | 5-17 | 5-17 | 326,524 | 326,572 | |
| 1764 | Japan | 2012 | National Health and Nutrition Survey | National | both | 5+ | 5+ | 11,298 | 13,674 | |
| 1765 | Japan | 2012-2016 | The Nagahama study | Community | rural | 35-80 | 35-80 | 3,206 | 6,620 | |
| 1766 | Japan | 2013 | Awaji Child Health Study | Community | urban | 10-14 | 10-14 | 198 | 203 | |
| 1767 | Japan | 2013 | Fukuroi kids health study | Community | urban | 13-14 | 13-14 | 387 | 404 | |
| 1768 | Japan | 2013 | MEXT School Health Statistics | National | both | 5-17 | 5-17 | 327,923 | 327,577 | |
| 1769 | Japan | 2013 | National Health and Nutrition Survey | National | both | 5+ | 5+ | 3,198 | 3,637 | |
| 1770 | Japan | 2014 | Awaji Child Health Study | Community | urban | 10-14 | 10-14 | 229 | 218 | |

| | Country | Study years | Survey/Study name/Citation | Level of representative-ness | Rural, urban, or both | Age range as in NCD-RisC database | | Sample size | | Notes |
|---|---|---|---|---|---|---|---|---|---|---|
| | | | | | | Male | Female | Male | Female | |
| 1771 | Japan | 2014 | MEXT School Health Statistics | National | both | 5-17 | 5-17 | 327,062 | 326,884 | |
| 1772 | Japan | 2014 | National Health and Nutrition Survey | National | both | 5+ | 5+ | 3,208 | 3,657 | |
| 1773 | Japan | 2014-2015 | Nagaoka Health Screening | Community | both | 20-89 | 20-89 | 4,938 | 4,298 | |
| 1774 | Japan | 2015 | Awaji Child Health Study | Community | urban | 10-14 | 10-14 | 230 | 228 | |
| 1775 | Japan | 2015 | MEXT School Health Statistics | National | both | 5-17 | 5-17 | 326,382 | 327,210 | |
| 1776 | Japan | 2015 | National Health and Nutrition Survey | National | both | 5+ | 5+ | 2,914 | 3,457 | |
| 1777 | Japan | 2016 | MEXT School Health Statistics | National | both | 5-17 | 5-17 | 334,731 | 334,444 | |
| 1778 | Japan | 2016 | National Health and Nutrition Survey | National | both | 5+ | 5+ | 9,814 | 11,638 | |
| 1779 | Japan | 2017 | MEXT School Health Statistics | National | both | 5-17 | 5-17 | 333,182 | 333,722 | |
| 1780 | Japan | 2017 | National Health and Nutrition Survey | National | both | 5+ | 5+ | 2,662 | 3,057 | |
| 1781 | Japan | 2017 | The Tokyo Health Service Association Database | Community | urban | 20+ | 20+ | 63,713 | 47,577 | |
| 1782 | Japan | 2017-2018 | The Hisayama Study | Community | rural | 40+ | 40+ | 1,510 | 1,947 | |
| 1783 | Japan | 2018 | MEXT School Health Statistics | National | both | 5-17 | 5-17 | 337,022 | 336,953 | |
| 1784 | Japan | 2018 | National Health and Nutrition Survey | National | both | 5+ | 5+ | 2,790 | 3,144 | |
| 1785 | Japan | 2019 | MEXT School Health Statistics | National | both | 5-17 | 5-17 | 332,231 | 333,546 | |
| 1786 | Japan | 2019 | National Health and Nutrition Survey | National | both | 5+ | 5+ | 2,277 | 2,606 | |
| 1787 | Japan | 2020 | MEXT School Health Statistics | National | both | 5-17 | 5-17 | 333,405 | 333,857 | |
| 1788 | Jordan | 1994-1996 | Ajlouni, Int J Obes Relat Metab Disord 22(7), 1998 | Subnational | both | 25+ | 25+ | 1,047 | 1,787 | |
| 1789 | Jordan | 1997 | DHS | National | both | | 20-49 | | 3,002 | |
| 1790 | Jordan | 2002 | DHS | National | both | | 20-49 | | 4,839 | |
| 1791 | Jordan | 2004 | Khader et al., Metab Syndr Relat Disord 6(2):113-20, 2008 | Community | both | 25+ | 25-59 | 394 | 548 | |
| 1792 | Jordan | 2004 | Behavioural Risk Factor Surveillence Survey | National | rural | 18+ | 18+ | 236 | 473 | |
| 1793 | Jordan | 2007 | Behavioural Risk Factor Surveillence Survey | National | both | 18+ | 18+ | 332 | 433 | |
| 1794 | Jordan | 2007 | DHS | National | both | | 20-49 | | 4,451 | |
| 1795 | Jordan | 2009 | DHS | National | both | | 20-49 | | 4,054 | |
| 1796 | Jordan | 2009 | Metablic abnomalities and vitamin D study | National | both | 7+ | 7+ | 1,601 | 3,863 | |
| 1797 | Jordan | 2012 | DHS | National | both | | 20-49 | | 6,357 | |
| 1798 | Jordan | 2015-2016 | Zayed et al., BMC Public Health 16(1):1040, 2016 | Subnational | rural | 6-15 | 6-17 | 407 | 288 | |
| 1799 | Jordan | 2015-2016 | Zayed et al., BMC Public Health 16(1):1040, 2016 | Subnational | urban | 6-15 | 6-17 | 565 | 974 | |
| 1800 | Jordan | 2016-2017 | National Cardiovascular Diseases and Diabetes Study (NCDDS) | National | both | 18+ | 18+ | 1,187 | 2,745 | |
| 1801 | Jordan | 2017-2018 | DHS | National | both | | 15-49 | | 6,261 | |
| 1802 | Jordan | 2018-2019 | Anthropometric Indices of Obesity as Predictors of High Blood Pressure among School Children | Community | urban | 10-14 | 10-14 | 284 | 504 | |
| 1803 | Jordan | 2019 | Jordan National Micronutrient and Nutrition Survey (15-49 women) | National | both | | 15-49 | | 621 | |
| 1804 | Jordan | 2019 | STEPS | National | both | 18-69 | 18-69 | 2,009 | 3,084 | |
| 1805 | Kazakhstan | 1985 | Balakhmetova et l., Ter Arkh 63(1):17-20, 1991 | Community | urban | 20-54 | | 2,886 | | |
| 1806 | Kazakhstan | 1995 | DHS | National | both | | 15-49 | | 3,542 | |
| 1807 | Kazakhstan | 1999 | DHS | National | both | | 15-49 | | 2,227 | |
| 1808 | Kazakhstan | 2011-2012 | Household Health Survey | National | both | 15+ | 15+ | 4,578 | 6,044 | |
| 1809 | Kazakhstan | 2015 | Almaty STEPS | Subnational | both | 18-69 | 18-69 | 381 | 1,136 | |
| 1810 | Kazakhstan | 2015 | Shymkent STEPS | Subnational | both | 18-69 | 18-69 | 400 | 793 | |
| 1811 | Kazakhstan | 2015-2016 | Aktobe STEPS | Subnational | both | 18-69 | 18-69 | 348 | 1,144 | |
| 1812 | Kazakhstan | 2015-2016 | Childhood Obesity Surveillance Initiative 4 | National | both | 8-10 | 8-10 | 2,755 | 2,683 | |
| 1813 | Kazakhstan | 2018-2019 | Early diagnosis of metabolic syndrome in children and adolescents of Semey Region | Subnational | both | 6-18 | 6-18 | 1,008 | 963 | |
| 1814 | Kazakhstan | 2019 | A health status assessment of a population of Karaganda urban region | Community | urban | 18+ | 18+ | 324 | 670 | |
| 1815 | Kazakhstan | 2020 | Childhood Obesity Surveillance Initiative 5 | National | both | 6-9 | 6-9 | 3,396 | 3,379 | |
| 1816 | Kazakhstan | 2021 | Prevalence of NCD Risk Factors in Kazakhstan | Subnational | both | 18-69 | 18-69 | 800 | 809 | 17 |
| 1817 | Kenya | 1985 | INTERSALT | Community | rural | 20-59 | 20-59 | 90 | 86 | |
| 1818 | Kenya | 1993 | DHS | National | both | | 20-49 | | 3,113 | |
| 1819 | Kenya | 1998 | DHS | National | both | | 20-49 | | 3,009 | |
| 1820 | Kenya | 2003 | DHS | National | both | | 15-49 | | 7,189 | |
| 1821 | Kenya | 2008-2009 | DHS | National | both | | 15-49 | | 7,827 | |
| 1822 | Kenya | 2011-2013 | International Study of Childhood Obesity, Lifestyle and the Environment (ISCOLE) | Community | urban | 9-11 | 9-11 | 262 | 301 | |
| 1823 | Kenya | 2014 | DHS | National | both | | 15-49 | | 13,469 | |
| 1824 | Kenya | 2015 | STEPS | National | both | 18-69 | 18-69 | 1,750 | 2,511 | |
| 1825 | Kenya | 2018 | Assessing the gaps in healthcare and determining the feasibility for the setup of a social enterprise - Viwandani Lown Community Health Center, Kenya | Community | urban | 19-73 | 19-73 | 153 | 143 | |
| 1826 | Kenya | 2022 | DHS | National | both | | 15-49 | | 15,656 | |

| | Country | Study years | Survey/Study name/Citation | Level of representative-ness | Rural, urban, or both | Age range as in NCD-RisC database | | Sample size | | Notes |
|---|---|---|---|---|---|---|---|---|---|---|
| | | | | | | Male | Female | Male | Female | |
| 1827 | Kiribati | 1981 | Epidemiological survey of Kiribati | Subnational | rural | 20+ | 20+ | 473 | 532 | |
| 1828 | Kiribati | 1981 | Epidemiological survey of Kiribati | Subnational | urban | 20+ | 20+ | 939 | 906 | |
| 1829 | Kiribati | 2004 | STEPS | National | both | 15-64 | 15-64 | 779 | 939 | |
| 1830 | Kiribati | 2011 | Global School-based Student Health Survey | National | both | | 13 | | 156 | |
| 1831 | Kiribati | 2015-2016 | STEPS | National | both | 18-69 | 18-69 | 557 | 694 | |
| 1832 | Kuwait | 1980-1981 | al-Isa, Ann Nutr Metab 41(5):307-14, 1997 | Community | both | 18+ | | 959 | | |
| 1833 | Kuwait | 1993-1994 | al-Isa, Ann Nutr Metab 41(5):307-14, 1997 | Community | both | 18+ | | 1,730 | | |
| 1834 | Kuwait | 1995-1996 | Abdella et al., Diabetes Res and Clin Pract 42(3):187-196, 1998 | Subnational | both | 20-84 | 20-84 | 1,099 | 1,892 | |
| 1835 | Kuwait | 1998 | Abiaka et al., Biol Trace Elem Res 91(1):33-43, 2003 | National | both | 15-80 | 15-80 | 178 | 233 | |
| 1836 | Kuwait | 2001 | Kuwait Nutrition Surveillance System | National | urban | 20+ | 20+ | 822 | 1,686 | |
| 1837 | Kuwait | 2001 | Kuwait Nutrition Surveillance System | National | urban | 5-19 | 5-19 | 4,520 | 5,389 | |
| 1838 | Kuwait | 2002 | Kuwait Nutrition Surveillance System | National | both | 20+ | 20+ | 1,694 | 2,368 | |
| 1839 | Kuwait | 2002 | Kuwait Nutrition Surveillance System | National | both | 5-19 | 5-19 | 5,517 | 5,239 | |
| 1840 | Kuwait | 2003 | Kuwait Nutrition Surveillance System | National | both | 20+ | 20+ | 1,285 | 1,192 | |
| 1841 | Kuwait | 2003 | Kuwait Nutrition Surveillance System | National | both | 5-19 | 5-19 | 5,179 | 5,132 | |
| 1842 | Kuwait | 2004 | Kuwait Nutrition Surveillance System | National | both | 20+ | 20+ | 1,209 | 1,748 | |
| 1843 | Kuwait | 2004 | Kuwait Nutrition Surveillance System | National | both | 5-19 | 5-19 | 5,021 | 4,880 | |
| 1844 | Kuwait | 2005 | Kuwait Nutrition Surveillance System | National | both | 20+ | 20+ | 1,493 | 1,642 | |
| 1845 | Kuwait | 2005 | Kuwait Nutrition Surveillance System | National | both | 5-19 | 5-19 | 4,520 | 5,389 | |
| 1846 | Kuwait | 2006 | Kuwait Nutrition Surveillance System | National | both | 20+ | 20+ | 1,290 | 1,661 | |
| 1847 | Kuwait | 2006 | Kuwait Nutrition Surveillance System | National | both | 5-19 | 5-19 | 5,259 | 5,066 | |
| 1848 | Kuwait | 2006 | STEPS | National | both | 20-64 | 20-64 | 918 | 1,298 | |
| 1849 | Kuwait | 2007 | Kuwait Nutrition Surveillance System | National | both | 20+ | 20+ | 1,711 | 1,706 | |
| 1850 | Kuwait | 2007 | Kuwait Nutrition Surveillance System | National | both | 5-19 | 5-19 | 5,481 | 5,502 | |
| 1851 | Kuwait | 2008 | Kuwait Nutrition Surveillance System | National | both | 20+ | 20+ | 1,604 | 1,669 | |
| 1852 | Kuwait | 2008 | Kuwait Nutrition Surveillance System | National | both | 5-19 | 5-19 | 5,757 | 5,595 | |
| 1853 | Kuwait | 2008-2009 | National Nutrition Program for the State of Kuwait | National | both | 5+ | 5+ | 772 | 830 | |
| 1854 | Kuwait | 2008-2010 | Gulf Cooperation Council World Health Survey | National | both | 18+ | 18+ | 1,598 | 1,782 | |
| 1855 | Kuwait | 2009 | Kuwait Nutrition Surveillance System | National | both | 20+ | 20+ | 1,331 | 1,496 | |
| 1856 | Kuwait | 2009 | Kuwait Nutrition Surveillance System | National | both | 5-19 | 5-19 | 5,358 | 5,574 | |
| 1857 | Kuwait | 2011 | Global School-based Student Health Survey | National | both | 13-17 | 13-17 | 1,265 | 1,274 | |
| 1858 | Kuwait | 2011-2014 | Kuwait Diabetes Epidemiology Program | National | both | 18-82 | 18-82 | 3,007 | 2,242 | |
| 1859 | Kuwait | 2014 | STEPS | National | both | 18-69 | 18-69 | 1,382 | 2,212 | |
| 1860 | Kuwait | 2015 | Global School-based Student Health Survey | National | both | 13-17 | 13-17 | 1,363 | 1,553 | |
| 1861 | Kyrgyzstan | 1993 | Kyrgyzstan Multipurpose Poverty Survey | National | both | 5+ | 5+ | 3,512 | 3,786 | |
| 1862 | Kyrgyzstan | 1997 | DHS | National | both | | 15-49 | | 3,570 | |
| 1863 | Kyrgyzstan | 2012 | DHS | National | both | | 15-49 | | 7,516 | |
| 1864 | Kyrgyzstan | 2013 | STEPS | National | both | 25-64 | 25-64 | 942 | 1,600 | |
| 1865 | Kyrgyzstan | 2015-2016 | Childhood Obesity Surveillance Initiative 4 | National | both | 6-9 | 6-9 | 3,945 | 3,905 | |
| 1866 | Lao PDR | 2006 | Multiple Indicator Cluster Survey 3 | National | both | | 15-49 | | 807 | |
| 1867 | Lao PDR | 2008 | STEPS | Community | both | 25-64 | 25-64 | 1,568 | 2,353 | |
| 1868 | Lao PDR | 2013 | STEPS | National | both | 18-64 | 18-64 | 984 | 1,461 | |
| 1869 | Latvia | 1997 | Nutrition and Lifestyle in the Baltic Republics, WHO, 1997 | National | both | 19-50 | 19-50 | 703 | 732 | |
| 1870 | Latvia | 2008 | Childhood Obesity Surveillance Initiative 1 | National | both | 7-8 | 7-8 | 2,283 | 2,101 | |
| 1871 | Latvia | 2008-2009 | Cardiovascular risk factor study | National | both | 25-74 | 25-74 | 1,362 | 2,398 | |
| 1872 | Latvia | 2010 | Childhood Obesity Surveillance Initiative 2 | National | both | 7-8 | 7-8 | 2,093 | 2,053 | |
| 1873 | Latvia | 2012 | Childhood Obesity Surveillance Initiative 3 | National | both | 6-7 | 6-7 | 1,804 | 1,677 | |
| 1874 | Latvia | 2015-2016 | Childhood Obesity Surveillance Initiative 4 | National | both | 7-9 | 7-9 | 2,952 | 2,991 | |
| 1875 | Latvia | 2019 | Childhood Obesity Surveillance Initiative 5 | National | both | 6-9 | 6-9 | 3,420 | 3,441 | |
| 1876 | Lebanon | 1997 | Obesity in Lebanon: National Survey | National | both | 5+ | 5+ | 871 | 1,164 | |
| 1877 | Lebanon | 2008-2009 | STEPS | National | both | 5+ | 5+ | 1,721 | 1,886 | |
| 1878 | Lebanon | 2017 | STEPS | National | both | 18-69 | 18-69 | 729 | 983 | |
| 1879 | Lesotho | 1993 | National survey on iodine, vitamin A and iron status of women and children in Lesotho | National | both | | 20-65 | | 792 | |
| 1880 | Lesotho | 2004-2005 | DHS | National | both | | 15-49 | | 3,206 | |
| 1881 | Lesotho | 2009-2010 | DHS | National | both | 15-59 | 15-49 | 3,216 | 3,781 | |
| 1882 | Lesotho | 2012 | STEPS | National | both | 25-64 | 25-64 | 726 | 1,442 | |
| 1883 | Lesotho | 2014 | DHS | National | both | 15-59 | 15-49 | 2,860 | 3,244 | |

| | Country | Study years | Survey/Study name/Citation | Level of representative-ness | Rural, urban, or both | Age range as in NCD-RisC database | | Sample size | | Notes |
|---|---|---|---|---|---|---|---|---|---|---|
| | | | | | | Male | Female | Male | Female | |
| 1884 | Liberia | 2006-2007 | DHS | National | both | | 15-49 | | 6,419 | |
| 1885 | Liberia | 2011 | STEPS | National | both | 25-64 | 25-64 | 998 | 1,254 | |
| 1886 | Liberia | 2013 | DHS | National | both | 15-49 | 15-49 | 4,235 | 4,718 | |
| 1887 | Liberia | 2019 | DHS | National | both | | 15-49 | | 3,793 | |
| 1888 | Liberia | 2022 | STEPS | National | both | 18-69 | 18-69 | 1,528 | 2,361 | |
| 1889 | Libya | 1998-1999 | Kadiki et al., Diabetes Metab 27(6):647-54, 2001 | Community | both | 15+ | 15+ | 228 | 398 | |
| 1890 | Libya | 2007 | Global School-based Student Health Survey | National | both | 12-15 | 12-15 | 697 | 995 | |
| 1891 | Libya | 2009 | STEPS | National | both | 25-64 | 25-64 | 1,678 | 1,564 | |
| 1892 | Lithuania | 1983-1985 | MONICA, Kaunas | Community | urban | 35-64 | 35-64 | 728 | 735 | |
| 1893 | Lithuania | 1986-1987 | MONICA, Kaunas | Community | urban | 35-64 | 35-64 | 894 | 868 | |
| 1894 | Lithuania | 1987 | Countrywide Integrated Noncommunicable Diseases Intervention Programme survey | Subnational | rural | 25-64 | 25-64 | 1,220 | 1,434 | |
| 1895 | Lithuania | 1992-1993 | MONICA, Kaunas | Community | urban | 35-64 | 35-64 | 610 | 621 | |
| 1896 | Lithuania | 1992-1993 | Countrywide Integrated Noncommunicable Diseases Intervention Programme survey | Subnational | rural | 25-64 | 25-64 | 617 | 798 | |
| 1897 | Lithuania | 1997 | Pomerleau et al., Public Health Nutrition 3(1):3-10, 2000 | National | both | 19+ | 19+ | 966 | 1,130 | |
| 1898 | Lithuania | 1998-1999 | Countrywide Integrated Noncommunicable Diseases Intervention Programme survey | Subnational | rural | 25-64 | 25-64 | 816 | 1,021 | |
| 1899 | Lithuania | 2001-2002 | MONICA4 | Community | urban | 35-64 | 35-64 | 625 | 776 | |
| 1900 | Lithuania | 2002 | Pomerleau, 2002 | National | both | 24-70 | 24-70 | 977 | 928 | |
| 1901 | Lithuania | 2006-2007 | Countrywide Integrated Noncommunicable Diseases Intervention Programme survey | Subnational | rural | 25-64 | 25-64 | 718 | 972 | |
| 1902 | Lithuania | 2006-2008 | Health, Alcohol and Psychosocial Factors In Eastern Europe | Community | urban | 45-72 | 45-72 | 3,214 | 3,857 | |
| 1903 | Lithuania | 2008 | Childhood Obesity Surveillance Initiative 1 | National | both | 7-8 | 7-8 | 2,532 | 2,344 | |
| 1904 | Lithuania | 2010 | Childhood Obesity Surveillance Initiative 2 | National | both | 7-9 | 7-9 | 3,306 | 3,414 | |
| 1905 | Lithuania | 2010-2012 | Prevalence and risk factors of high blood pressure in 12-15-year-old Lithuanian children and adolescents (Study 1, 2010-2012) | Community | both | 12-15 | 12-15 | 3,494 | 3,963 | |
| 1906 | Lithuania | 2012-2013 | Prevalence and risk factors of high blood pressure in 12-15-year-old Lithuanian children and adolescents (Study 2, 2012-2013) | Community | both | 12-15 | 12-15 | 962 | 985 | |
| 1907 | Lithuania | 2013 | Childhood Obesity Surveillance Initiative 3 | National | both | 7-8 | 7-8 | 1,890 | 1,895 | |
| 1908 | Lithuania | 2015-2016 | Childhood Obesity Surveillance Initiative 4 | National | both | 7-8 | 7-8 | 1,924 | 1,876 | |
| 1909 | Lithuania | 2019 | Childhood Obesity Surveillance Initiative 5 | National | both | 7-8 | 7-8 | 1,649 | 1,585 | |
| 1910 | Luxembourg | 2007-2009 | Observation of cardiovascular risk factors in Luxembourg (ORISCAV-LUX) | National | both | 18-69 | 18-69 | 696 | 735 | |
| 1911 | Luxembourg | 2013-2015 | European Health Examination Survey in Luxembourg | National | both | 25-64 | 25-64 | 721 | 785 | |
| 1912 | Luxembourg | 2016-2018 | Observation of cardiovascular risk factors in Luxembourg (ORISCAV-LUX2) | Community | both | 25-79 | 25-79 | 670 | 763 | |
| 1913 | Madagascar | 1997 | Mauny et al., Ann Trop Med Parasitol 97(6):645-54, 2003 | Community | both | 15+ | 15+ | 248 | 283 | |
| 1914 | Madagascar | 1997 | DHS | National | both | | 20-49 | | 2,253 | |
| 1915 | Madagascar | 2003-2004 | DHS | National | both | | 15-49 | | 7,155 | |
| 1916 | Madagascar | 2005 | STEPS | Subnational | both | 25-64 | 25-64 | 2,596 | 2,494 | |
| 1917 | Madagascar | 2008-2009 | DHS | National | both | | 15-49 | | 7,869 | |
| 1918 | Madagascar | 2021 | DHS | National | both | | 15-49 | | 8,888 | |
| 1919 | Malawi | 1992 | DHS | National | both | | 20-49 | | 2,102 | |
| 1920 | Malawi | 1996 | Chilima et al., Eur J Clin Nutr 52(9):643-9, 1998 | Community | rural | 55-94 | 55-94 | 86 | 185 | |
| 1921 | Malawi | 2000 | DHS | National | both | | 15-49 | | 11,491 | |
| 1922 | Malawi | 2004 | DHS | National | both | | 15-49 | | 9,751 | |
| 1923 | Malawi | 2009 | National Micronutrient Survey | National | both | 6-12 | 6-49 | 344 | 932 | |
| 1924 | Malawi | 2009 | STEPS | National | both | 25-64 | 25-64 | 1,666 | 3,189 | |
| 1925 | Malawi | 2010 | DHS | National | both | | 15-49 | | 7,118 | |
| 1926 | Malawi | 2013-2017 | NCD Survey Malawi Epidemiology and Intervention Research Unit | Community | rural | 18+ | 18+ | 5,849 | 7,507 | |
| 1927 | Malawi | 2013-2017 | NCD Survey Malawi Epidemiology and Intervention Research Unit | Community | urban | 18+ | 18+ | 5,802 | 10,291 | |
| 1928 | Malawi | 2015-2016 | DHS | National | both | | 15-49 | | 7,415 | |
| 1929 | Malawi | 2017 | STEPS | National | both | 18-69 | 18-69 | 1,478 | 2,534 | |
| 1930 | Malaysia | 1996 | National Health and Morbidity Survey (NHMS) | National | both | 18+ | 18+ | 14,520 | 16,244 | |
| 1931 | Malaysia | 2002-2003 | Malaysian Adult Nutrition Survey | National | both | 18-59 | 18-59 | 3,303 | 3,395 | |
| 1932 | Malaysia | 2004 | Rampal et al., Public Health 122(1):11-8, 2008 | National | both | 15+ | 15+ | 7,028 | 9,527 | |
| 1933 | Malaysia | 2005 | STEPS | National | both | 25-64 | 25-64 | 1,286 | 1,286 | |
| 1934 | Malaysia | 2006 | National Health and Morbidity Survey (NHMS) | National | both | 5+ | 5+ | 22,970 | 25,508 | |
| 1935 | Malaysia | 2008 | National Iodine Deficiency Disorder (IDD) Survey | National | both | 7-10 | 7-10 | 9,388 | 8,659 | |
| 1936 | Malaysia | 2008 | Metabolic Syndrome Study in Malaysia | National | rural | 18+ | 18+ | 753 | 1,368 | |
| 1937 | Malaysia | 2008 | Metabolic Syndrome Study in Malaysia | National | urban | 18+ | 18+ | 769 | 1,446 | |
| 1938 | Malaysia | 2010-2011 | SEANUTS | National | both | 5-12 | 5-12 | 1,306 | 1,352 | |

| | Country | Study years | Survey/Study name/Citation | Level of representative-ness | Rural, urban, or both | Age range as in NCD-RisC database | | Sample size | | Notes |
|---|---|---|---|---|---|---|---|---|---|---|
| | | | | | | Male | Female | Male | Female | |
| 1939 | Malaysia | 2011 | National Health and Morbidity Survey (NHMS) | National | both | 5+ | 5+ | 8,033 | 8,780 | |
| 1940 | Malaysia | 2012 | Malaysian School-Based Health Survey | National | both | 9-17 | 9-17 | 20,254 | 19,652 | |
| 1941 | Malaysia | 2012-2013 | Petaling District | Subnational | urban | 12-17 | 12-17 | 882 | 1,364 | |
| 1942 | Malaysia | 2013-2014 | Batang Padang District | Subnational | both | 12-17 | 12-17 | 2,928 | 3,319 | |
| 1943 | Malaysia | 2014 | Malaysian Adult Nutrition Survey | National | both | 18-59 | 18-59 | 1,328 | 1,495 | |
| 1944 | Malaysia | 2015 | National Health and Morbidity Survey (NHMS) | National | both | 5+ | 5+ | 12,144 | 12,871 | |
| 1945 | Malaysia | 2019 | National Health and Morbidity Survey (NHMS) | National | both | 5+ | 5+ | 6,155 | 6,848 | |
| 1946 | Maldives | 2001 | Multiple Indicator Cluster Survey | National | both | | 15-50 | | 1,145 | |
| 1947 | Maldives | 2004 | STEPS | Subnational | urban | 25-64 | 25-64 | 933 | 1,086 | |
| 1948 | Maldives | 2009 | DHS | National | both | | 20-49 | | 5,139 | |
| 1949 | Maldives | 2009 | Global School-based Student Health Survey | National | both | 13-17 | | 806 | | |
| 1950 | Maldives | 2011 | STEPS | Subnational | urban | 15-64 | 15-64 | 660 | 1,060 | |
| 1951 | Maldives | 2014 | Global School-based Student Health Survey | National | both | 13-17 | | 931 | | |
| 1952 | Maldives | 2016-2017 | DHS | National | both | 15-49 | 15-49 | 3,831 | 6,839 | |
| 1953 | Maldives | 2020-2021 | STEPS | National | both | 15-69 | 15-69 | 790 | 1,884 | |
| 1954 | Mali | 1995-1996 | DHS | National | both | | 20-49 | | 3,789 | |
| 1955 | Mali | 1997 | Programme Intégré de Développement de Bafoulabé | Community | rural | 15-45 | 15-45 | 425 | 716 | |
| 1956 | Mali | 1999-2000 | Bafoulabe Iodine Study | Community | rural | | 15-45 | | 365 | |
| 1957 | Mali | 2001 | DHS | National | both | | 15-49 | | 10,526 | |
| 1958 | Mali | 2006 | DHS | National | both | | 15-49 | | 12,512 | |
| 1959 | Mali | 2007 | STEPS | Subnational | both | 15-64 | 15-64 | 1,036 | 1,494 | |
| 1960 | Mali | 2012-2013 | DHS | National | both | | 15-49 | | 4,646 | |
| 1961 | Mali | 2013 | Santé Nutritionnelle à Assise Comunautaire dans la région de Kayes (SNACK) | Subnational | rural | | 20-68 | | 4,595 | |
| 1962 | Mali | 2013 | STEPS | Subnational | both | 15-65 | 15-65 | 525 | 949 | |
| 1963 | Mali | 2018 | DHS | National | both | | 15-49 | | 4,576 | |
| 1964 | Malta | 1984 | MONICA, Malta | Community | both | 25-64 | 25-64 | 948 | 929 | |
| 1965 | Malta | 1986 | INTERSALT | Community | rural | 20-59 | 20-59 | 100 | 100 | |
| 1966 | Malta | 2008 | Childhood Obesity Surveillance Initiative 1 | National | both | 6 | 6 | 1,084 | 1,031 | |
| 1967 | Malta | 2010 | Childhood Obesity Surveillance Initiative 2 | National | both | 6 | 6 | 1,151 | 1,170 | |
| 1968 | Malta | 2013 | Childhood Obesity Surveillance Initiative 3 | National | both | 7-8 | 7-8 | 1,757 | 1,693 | |
| 1969 | Malta | 2014-2016 | SAHHTEK - The University of Malta Health and Wellbeing Study | National | both | 18-70 | 18-70 | 834 | 1,024 | |
| 1970 | Malta | 2015-2016 | Childhood Obesity Surveillance Initiative 4 | National | both | 7-8 | 7-8 | 2,056 | 1,905 | |
| 1971 | Malta | 2019 | Childhood Obesity Surveillance Initiative 5 | National | both | 7-8 | 7-8 | 2,085 | 1,999 | |
| 1972 | Marshall Islands | 2002 | STEPS | National | both | 15-64 | 15-64 | 772 | 1,195 | |
| 1973 | Marshall Islands | 2017-2018 | STEPS | National | both | 18+ | 18+ | 1,317 | 1,490 | |
| 1974 | Mauritania | 2000-2001 | DHS | National | both | | 15-49 | | 2,635 | |
| 1975 | Mauritania | 2006 | STEPS | Community | urban | 15-64 | 15-64 | 1,132 | 1,300 | |
| 1976 | Mauritania | 2019-2021 | DHS | National | both | | 15-49 | | 6,820 | |
| 1977 | Mauritius | 1987 | Mauritius Noncommunicable Disease Survey | National | both | 25-74 | 25-74 | 2,347 | 2,653 | |
| 1978 | Mauritius | 1992 | Mauritius Noncommunicable Disease Survey | National | both | 25-74 | 25-74 | 2,985 | 3,477 | |
| 1979 | Mauritius | 1992 | Rodrigues, Mauritius (1992) | Community | rural | 25-64 | 25-64 | 736 | 770 | |
| 1980 | Mauritius | 1998 | Mauritius Noncommunicable Disease Survey | National | both | 25-74 | 25-74 | 2,566 | 3,248 | |
| 1981 | Mauritius | 1999 | Rodrigues, Mauritius (1999) | Community | rural | 20+ | 20+ | 974 | 1,292 | |
| 1982 | Mauritius | 2009 | Mauritius Noncommunicable Disease Survey | National | both | 19-74 | 19-74 | 2,859 | 3,391 | |
| 1983 | Mauritius | 2011 | Global School-based Student Health Survey | National | both | 13-17 | 13-17 | 859 | 1,043 | |
| 1984 | Mauritius | 2011 | Global School-based Student Health Survey-Rodrigues | Subnational | both | 13-17 | 13-17 | 425 | 546 | |
| 1985 | Mauritius | 2015 | Mauritius Noncommunicable Disease Survey | National | both | 20-74 | 20-74 | 1,615 | 1,944 | |
| 1986 | Mauritius | 2015 | Mauritius Noncommunicable Disease Survey 1998 Follow Up | National | both | 20+ | 20+ | 873 | 1,164 | |
| 1987 | Mauritius | 2017 | Global School-based Student Health Survey | National | both | 13-17 | 13-17 | 1,358 | 1,525 | |
| 1988 | Mauritius | 2019 | Global School-based Student Health Survey | Subnational | rural | 12-17 | 12-17 | 1,116 | 1,289 | |
| 1989 | Mexico | 1988-1989 | Encuesta Nacional de Nutricion | National | both | | 12-49 | | 16,434 | |
| 1990 | Mexico | 1992-1993 | Encuesta Nacional de Enfermedades Cronicas | National | urban | 20-69 | 20-69 | 6,040 | 8,298 | |
| 1991 | Mexico | 1996 | Sanchez-Castillo et al., Eur J Clin Nutr 55(10):833-40, 2001 | Community | rural | 18+ | 18+ | 104 | 149 | |
| 1992 | Mexico | 1998-1999 | Encuesta Nacional de Nutricion | National | both | | 12-49 | | 17,892 | |
| 1993 | Mexico | 1998-2004 | Mexico City Prospective Study | Community | urban | 35-84 | 35-84 | 51,768 | 105,313 | |
| 1994 | Mexico | 1999 | National Survey on School Children | National | both | 5-10 | 5-10 | 4,898 | 5,031 | |
| 1995 | Mexico | 1999-2000 | The Survey on Health, Well-Being, and Aging in Latin America and the Caribbean (SABE) | Community | urban | 60+ | 60+ | 359 | 548 | 3 |

| | Country | Study years | Survey/Study name/Citation | Level of representative-ness | Rural, urban, or both | Age range as in NCD-RisC database | | Sample size | | Notes |
|---|---|---|---|---|---|---|---|---|---|---|
| | | | | | | Male | Female | Male | Female | |
| 1996 | Mexico | 2000 | Encuesta Nacional de Salud | National | both | 10+ | 10+ | 22,554 | 39,204 | |
| 1997 | Mexico | 2001 | The Mexican Health and Aging Study | National | both | 50+ | 50+ | 1,030 | 1,224 | |
| 1998 | Mexico | 2002 | Encuesta Nacional Sobre Niveles de vida de los Hogares | National | both | 5+ | 5+ | 11,606 | 13,614 | |
| 1999 | Mexico | 2003 | The Mexican Health and Aging Study | National | both | 50+ | 50+ | 893 | 1,162 | |
| 2000 | Mexico | 2004-2005 | CArdiovascular Risk factors Multiple Evaluation in Latin America (CARMELA) | Community | urban | 25-64 | 25-64 | 833 | 894 | |
| 2001 | Mexico | 2005 | Encuesta Nacional Sobre Niveles de vida de los Hogares | National | both | 5+ | 5+ | 11,696 | 13,211 | |
| 2002 | Mexico | 2006 | Encuesta Nacional de Salud y Nutrición | National | both | 5+ | 5+ | 27,848 | 34,909 | |
| 2003 | Mexico | 2006 | PREVENIMSS National Coverage Surveys | National | urban | 20+ | 20+ | 8,727 | 11,335 | |
| 2004 | Mexico | 2009-2010 | WHO Study on global AGEing and adult health (SAGE) | National | both | 50+ | 50+ | 793 | 1,234 | |
| 2005 | Mexico | 2009-2012 | Encuesta Nacional Sobre Niveles de vida de los Hogares | National | both | 5+ | 5+ | 12,408 | 14,385 | |
| 2006 | Mexico | 2010 | PREVENIMSS National Coverage Surveys | National | urban | 20+ | 20+ | 6,238 | 6,003 | |
| 2007 | Mexico | 2011-2012 | Encuesta Nacional de Salud y Nutrición | National | both | 5+ | 5+ | 31,293 | 36,834 | |
| 2008 | Mexico | 2012 | The Mexican Health and Aging Study | National | both | 50+ | 50+ | 786 | 1,106 | |
| 2009 | Mexico | 2014 | WHO Study on global AGEing and adult health (SAGE), Wave 2 | National | both | 18+ | 18+ | 1,658 | 2,417 | |
| 2010 | Mexico | 2016 | Encuesta Nacional de Salud y Nutrición | National | both | 5+ | 5+ | 5,659 | 8,424 | |
| 2011 | Mexico | 2016 | Cognitive Aging Linked to MHAS (Mex-Cog) | National | both | 55+ | 55+ | 786 | 1,141 | 18 |
| 2012 | Mexico | 2018-2019 | Encuesta Nacional de Salud y Nutrición | National | both | 5+ | 5+ | 11,581 | 13,420 | |
| 2013 | Mexico | 2020 | Encuesta Nacional de Salud y Nutrición | National | both | 5+ | 5+ | 5,593 | 7,459 | |
| 2014 | Mexico | 2021 | Encuesta Nacional de Salud y Nutrición | National | both | 5+ | 5+ | 5,798 | 7,949 | |
| 2015 | Mexico | 2022 | Encuesta Nacional de Salud y Nutrición | National | both | 5+ | 5+ | 5,441 | 7,550 | |
| 2016 | Micronesia | 2002 | STEPS | Subnational | both | 25-64 | 25-64 | 591 | 893 | |
| 2017 | Micronesia | 2006 | STEPS | Subnational | both | 15-64 | 15-64 | 918 | 1,553 | |
| 2018 | Micronesia | 2008 | STEPS | Subnational | both | 25-64 | 25-64 | 875 | 1,266 | |
| 2019 | Micronesia | 2009 | STEPS, Kosrae | Subnational | both | 15-64 | 15-64 | 208 | 413 | |
| 2020 | Micronesia | 2009 | STEPS, Yap | Subnational | both | 15-64 | 15-64 | 405 | 521 | |
| 2021 | Micronesia | 2016 | STEPS | Subnational | both | 18-69 | 18-69 | 516 | 818 | |
| 2022 | Moldova | 2005 | DHS | National | both | | 15-49 | | 7,076 | |
| 2023 | Moldova | 2013 | Childhood Obesity Surveillance Initiative 3 | National | both | 7-8 | 7-8 | 1,931 | 1,751 | |
| 2024 | Moldova | 2013 | STEPS | National | both | 18-69 | 18-69 | 1,712 | 2,777 | |
| 2025 | Moldova | 2021 | STEPS | National | both | 18-69 | 18-69 | 1,721 | 2,252 | |
| 2026 | Mongolia | 1999 | National Nutrition Survey | National | both | 35-65 | 35-65 | 907 | 1,317 | |
| 2027 | Mongolia | 2004 | National Nutrition Survey | National | both | 15-74 | 15-74 | 248 | 360 | |
| 2028 | Mongolia | 2005 | STEPS | National | both | 15-64 | 15-64 | 1,669 | 1,717 | |
| 2029 | Mongolia | 2009 | STEPS | National | both | 15-64 | 15-64 | 2,197 | 3,117 | |
| 2030 | Mongolia | 2013 | Global School-based Student Health Survey | National | both | 13-17 | 13-17 | 2,095 | 2,360 | |
| 2031 | Mongolia | 2013 | STEPS | National | both | 15-64 | 15-64 | 2,698 | 3,167 | |
| 2032 | Mongolia | 2019 | STEPS | National | both | 15-69 | 15-69 | 2,926 | 3,543 | |
| 2033 | Montenegro | 1978 | Anthropometric Characteristics of Montenegrin Recruiters | National | both | 17-28 | | 86 | | 1 |
| 2034 | Montenegro | 1979 | Anthropometric Characteristics of Montenegrin Recruiters | National | both | 17-28 | | 7,229 | | 1 |
| 2035 | Montenegro | 1980 | Anthropometric Characteristics of Montenegrin Recruiters | National | both | 17-28 | | 11,208 | | |
| 2036 | Montenegro | 1981 | Anthropometric Characteristics of Montenegrin Recruiters | National | both | 17-28 | | 11,372 | | |
| 2037 | Montenegro | 1982 | Anthropometric Characteristics of Montenegrin Recruiters | National | both | 17-28 | | 5,502 | | |
| 2038 | Montenegro | 1983 | Anthropometric Characteristics of Montenegrin Recruiters | National | both | 17-28 | | 9,448 | | |
| 2039 | Montenegro | 1984 | Anthropometric Characteristics of Montenegrin Recruiters | National | both | 17-28 | | 10,320 | | |
| 2040 | Montenegro | 1985 | Anthropometric Characteristics of Montenegrin Recruiters | National | both | 17-28 | | 9,722 | | |
| 2041 | Montenegro | 1986 | Anthropometric Characteristics of Montenegrin Recruiters | National | both | 17-28 | | 9,961 | | |
| 2042 | Montenegro | 1987 | Anthropometric Characteristics of Montenegrin Recruiters | National | both | 17-28 | | 10,230 | | |
| 2043 | Montenegro | 1988 | Anthropometric Characteristics of Montenegrin Recruiters | National | both | 17-28 | | 86 | | |
| 2044 | Montenegro | 2015-2016 | Childhood Obesity Surveillance Initiative 4 | National | both | 6-8 | 6-8 | 1,802 | 1,623 | |
| 2045 | Montenegro | 2016 | Anthropometric parameters as an indicator of obesity at adolescents in Montenegro | National | both | 14-18 | 14-18 | 678 | 771 | |
| 2046 | Montenegro | 2018-2019 | Initiative for monitoring obesity of children aged 6 to 9 in Montenegro and Slovenia | National | both | 6-9 | 6-9 | 1,111 | 999 | |
| 2047 | Montenegro | 2019 | Body composition of high school students in Montenegro and its relationship with their eating habits | National | both | 18-20 | 18-20 | 504 | 497 | |
| 2048 | Montenegro | 2019 | Childhood Obesity Surveillance Initiative 5 | National | both | 6-8 | 6-8 | 1,729 | 1,626 | |
| 2049 | Montenegro | 2019 | Initiative for monitoring obesity of children aged 11 to 12 in Montenegro | National | both | 11-12 | 11-12 | 666 | 619 | |
| 2050 | Montenegro | 2019 | Initiative for monitoring obesity of children aged 5 to 6 in Montenegro and Slovenia | National | both | 5-6 | 5-6 | 231 | 214 | |
| 2051 | Montenegro | 2020-2021 | Initiative for monitoring obesity of school children aged 9 to 10 in Montenegro and Slovenia | National | both | 9-10 | 9-10 | 694 | 666 | |

| | Country | Study years | Survey/Study name/Citation | Level of representative-ness | Rural, urban, or both | Age range as in NCD-RisC database | | Sample size | | Notes |
|---|---|---|---|---|---|---|---|---|---|---|
| | | | | | | Male | Female | Male | Female | |
| 2052 | Montenegro | 2021-2022 | Determining the differences in the levels of anthropometric characteristics of students in the first grades of elementary schools in Podgorica and Niksic | Subnational | urban | 6-8 | 6-8 | 397 | 346 | |
| 2053 | Montenegro | 2022 | Determining the anthropometric characteristics of final grade elementary school students in Montenegro - Bijelo Polje | Community | both | 14-15 | 14-15 | 15 | 9 | |
| 2054 | Montenegro | 2022 | Determining the anthropometric characteristics of final grade elementary school students in Montenegro - Herceg Novi | Community | both | 14-15 | 14-15 | 25 | 23 | |
| 2055 | Montenegro | 2022 | Determining the anthropometric characteristics of final grade elementary school students in Montenegro - Nikšić | Community | both | 14-15 | 14-15 | 97 | 52 | |
| 2056 | Montenegro | 2022 | Determining the anthropometric characteristics of final grade elementary school students in Montenegro - Podgorica | Community | both | 14-15 | 14-15 | 66 | 59 | |
| 2057 | Morocco | 1992 | DHS | National | both | | 20-49 | | 2,804 | |
| 2058 | Morocco | 2000 | National Survey 2000 | National | both | 20+ | 20+ | 755 | 1,047 | |
| 2059 | Morocco | 2003-2004 | DHS | National | both | | 15-49 | | 15,944 | |
| 2060 | Morocco | 2017 | STEPS | National | both | 18+ | 18+ | 1,871 | 3,390 | |
| 2061 | Morocco | 2019-2020 | National Nutrition Survey | National | both | 6-12 | 6-12 | 574 | 591 | |
| 2062 | Mozambique | 1997 | DHS | National | both | | 20-49 | | 2,824 | |
| 2063 | Mozambique | 2000 | Growth of adolescents in Mozambique | Community | urban | 9-17 | 9-17 | 690 | 727 | |
| 2064 | Mozambique | 2003 | DHS | National | both | | 15-49 | | 10,535 | |
| 2065 | Mozambique | 2005 | STEPS | National | both | 25-64 | 25-64 | 1,276 | 1,689 | |
| 2066 | Mozambique | 2011 | DHS | National | both | | 15-49 | | 12,201 | |
| 2067 | Mozambique | 2014-2015 | STEPS | National | both | 15-64 | 15-64 | 1,147 | 1,684 | |
| 2068 | Mozambique | 2017-2018 | Examining lifestyle behaviours and weight status of primary schoolchildren in Mozambique | Community | rural | 9-11 | 9-11 | 146 | 158 | |
| 2069 | Mozambique | 2017-2018 | Examining lifestyle behaviours and weight status of primary schoolchildren in Mozambique | Community | urban | 9-11 | 9-11 | 140 | 170 | |
| 2070 | Myanmar | 2003-2004 | STEPS | Subnational | both | 25-74 | 25-74 | 1,990 | 2,449 | |
| 2071 | Myanmar | 2009 | STEPS | National | both | 15-64 | 15-64 | 2,826 | 4,421 | |
| 2072 | Myanmar | 2011 | Underweight prevalence among young adults from rural areas, Salin Township, Magwe Region | Community | rural | 15-35 | 15-35 | 156 | 233 | |
| 2073 | Myanmar | 2013-2014 | STEPS, Yangon | Subnational | both | 25-74 | 25-74 | 745 | 740 | |
| 2074 | Myanmar | 2014 | STEPS | National | both | 25-64 | 25-64 | 2,947 | 5,444 | |
| 2075 | Myanmar | 2015-2016 | DHS | National | both | | 15-49 | | 12,163 | |
| 2076 | Namibia | 1992 | DHS | National | both | | 20-49 | | 2,062 | |
| 2077 | Namibia | 2005 | STEPS | National | both | 25-64 | 25-64 | 1,390 | 1,778 | |
| 2078 | Namibia | 2006-2007 | DHS | National | both | | 15-49 | | 8,968 | |
| 2079 | Namibia | 2009 | Okambilimbili Survey | Community | urban | 5+ | 5+ | 962 | 1,167 | |
| 2080 | Namibia | 2013 | DHS | National | both | | 15-64 | | 5,111 | |
| 2081 | Nauru | 1982 | Trends in the prevalence and incidence of non-insulin-dependent diabetes mellitus and impaired glucose tolerance | National | both | 20+ | 20+ | 701 | 773 | |
| 2082 | Nauru | 1987 | Trends in the prevalence and incidence of non-insulin-dependent diabetes mellitus and impaired glucose tolerance | National | both | 20+ | 20+ | 555 | 667 | |
| 2083 | Nauru | 1994 | Trends in the prevalence and incidence of non-insulin-dependent diabetes mellitus and impaired glucose tolerance | National | both | 25+ | 25+ | 647 | 731 | |
| 2084 | Nauru | 2004 | STEPS | National | both | 15-64 | 15-64 | 1,082 | 1,149 | |
| 2085 | Nauru | 2006 | STEPS | National | both | 16-65 | 16-65 | 255 | 236 | |
| 2086 | Nauru | 2011 | Global School-based Student Health Survey | National | both | | 13-17 | | 259 | |
| 2087 | Nauru | 2015 | STEPS | National | both | 18-69 | 18-69 | 505 | 540 | |
| 2088 | Nepal | 1996 | DHS | National | both | | 20-49 | | 3,068 | |
| 2089 | Nepal | 1997 | Ohno et al., Asia Pac J Public Health 18(3):20-9, 2006 | Community | rural | 17-75 | 17-75 | 36 | 41 | |
| 2090 | Nepal | 2001 | DHS | National | both | | 20-49 | | 7,216 | |
| 2091 | Nepal | 2003 | STEPS | Subnational | both | 25-64 | 25-64 | 1,010 | 996 | |
| 2092 | Nepal | 2005 | STEPS | Subnational | both | 15-64 | 15-64 | 3,634 | 3,998 | |
| 2093 | Nepal | 2006 | DHS | National | both | | 15-49 | | 10,117 | |
| 2094 | Nepal | 2006-2011 | Early detection and management of Kidney disease, Hypertension, Diabetes and Cardiovascular disease (KHDC Nepal), Tarahara | Community | rural | 18+ | 18+ | 1,175 | 2,350 | |
| 2095 | Nepal | 2006-2011 | Early detection and management of Kidney disease, Hypertension, Diabetes and Cardiovascular disease (KHDC Nepal), Damak | Community | urban | 18+ | 18+ | 1,095 | 1,576 | |
| 2096 | Nepal | 2006-2011 | Early detection and management of Kidney disease, Hypertension, Diabetes and Cardiovascular disease (KHDC Nepal), Dharan | Community | urban | 18+ | 18+ | 4,130 | 6,126 | |
| 2097 | Nepal | 2007-2008 | STEPS | National | both | 15-64 | 15-64 | 1,889 | 2,348 | |

| | Country | Study years | Survey/Study name/Citation | Level of representative-ness | Rural, urban, or both | Age range as in NCD-RisC database | | Sample size | | Notes |
|---|---|---|---|---|---|---|---|---|---|---|
| | | | | | | Male | Female | Male | Female | |
| 2098 | Nepal | 2011 | DHS | National | both | | 15-49 | | 5,848 | |
| 2099 | Nepal | 2012-2013 | STEPS | National | both | 15-69 | 15-69 | 1,316 | 2,753 | |
| 2100 | Nepal | 2015 | Community based intervention for prevention and control of non-communicable diseases risk factors (CIPCON) baseline survey, Dhankuta | Subnational | rural | 15-69 | 15-69 | 555 | 781 | |
| 2101 | Nepal | 2015 | Community based intervention for prevention and control of non-communicable diseases risk factors (CIPCON) baseline survey, Ilam | Subnational | rural | 15-69 | 15-69 | 546 | 721 | |
| 2102 | Nepal | 2016 | DHS | National | both | 15-49 | 15-49 | 4,035 | 6,165 | |
| 2103 | Nepal | 2016-2018 | The Population Based Prevalence of Selected Non-Communicable Diseases In Nepal | National | both | 20+ | 20+ | 4,907 | 7,529 | |
| 2104 | Nepal | 2019 | STEPS | National | both | 15-69 | 15-69 | 1,996 | 3,521 | |
| 2105 | Netherlands | 1985 | INTERSALT | Community | urban | 20-59 | 20-59 | 100 | 99 | |
| 2106 | Netherlands | 1985 | Zutphen Elderly Study | Community | urban | 65-85 | | 886 | | |
| 2107 | Netherlands | 1987-1991 | Doetinchem Cohort Study | Subnational | urban | 20-59 | 20-59 | 2,995 | 3,317 | |
| 2108 | Netherlands | 1989-1993 | The Rotterdam Study, first subcohort | Community | urban | 55+ | 55+ | 2,807 | 4,103 | |
| 2109 | Netherlands | 1990 | Zutphen Elderly Study | Community | urban | 69-90 | | 552 | | |
| 2110 | Netherlands | 1992-1993 | The Longitudinal Aging Study Amsterdam (LASA) | Subnational | both | 55-85 | 55-85 | 1,266 | 1,308 | 19 |
| 2111 | Netherlands | 1993-1995 | The Rotterdam Study, first subcohort | Community | urban | 56+ | 56+ | 2,214 | 3,105 | |
| 2112 | Netherlands | 1993-1997 | EPIC Bilthoven | Community | urban | 20-59 | 20-59 | 9,941 | 12,021 | |
| 2113 | Netherlands | 1993-1997 | EPIC Utrecht | Community | both | | 49-70 | | 17,335 | |
| 2114 | Netherlands | 1995-1996 | The Longitudinal Aging Study Amsterdam (LASA) | Subnational | both | 65-88 | 65-88 | 714 | 764 | 19 |
| 2115 | Netherlands | 1997-1999 | The Rotterdam Study, first subcohort | Community | urban | 61+ | 61+ | 1,718 | 2,361 | |
| 2116 | Netherlands | 1998-1999 | The Longitudinal Aging Study Amsterdam (LASA) | Subnational | both | 61-91 | 61-91 | 604 | 743 | 19 |
| 2117 | Netherlands | 1998-2001 | Regenboog Project | National | both | 12-89 | 12-89 | 2,714 | 2,643 | |
| 2118 | Netherlands | 2000-2001 | The Rotterdam Study, second subcohort | Community | urban | 55+ | 55+ | 1,210 | 1,468 | |
| 2119 | Netherlands | 2001-2002 | The Longitudinal Aging Study Amsterdam (LASA) | Subnational | both | 64-94 | 64-94 | 577 | 690 | 19 |
| 2120 | Netherlands | 2001-2003 | Surinamese in the Netherlands: Study on Ethnicity and Health (SUNSET) | Community | urban | 35-60 | 35-60 | 251 | 257 | |
| 2121 | Netherlands | 2002-2003 | The Longitudinal Aging Study Amsterdam (LASA) | Subnational | both | 54-65 | 54-65 | 431 | 482 | 19 |
| 2122 | Netherlands | 2002-2004 | The Rotterdam Study, first subcohort | Community | urban | 64+ | 64+ | 1,206 | 1,708 | |
| 2123 | Netherlands | 2003-2007 | Doetinchem Cohort Study | Subnational | urban | 36-75 | 36-75 | 2,135 | 2,368 | |
| 2124 | Netherlands | 2004-2005 | The Rotterdam Study, second subcohort | Community | urban | 58+ | 58+ | 964 | 1,244 | |
| 2125 | Netherlands | 2004-2006 | Prevention and Incidence of Asthma and Mite Allergy (PIAMA) | National | both | 7-9 | 7-9 | 1,110 | 1,104 | |
| 2126 | Netherlands | 2005-2006 | The Longitudinal Aging Study Amsterdam (LASA) | Subnational | both | 57-97 | 57-97 | 789 | 958 | 19 |
| 2127 | Netherlands | 2006-2008 | The Rotterdam Study, third subcohort | Community | urban | 45+ | 45+ | 1,547 | 2,029 | |
| 2128 | Netherlands | 2008-2009 | The Longitudinal Aging Study Amsterdam (LASA) | Subnational | both | 60-100 | 60-100 | 642 | 789 | 19 |
| 2129 | Netherlands | 2008-2011 | Prevention and Incidence of Asthma and Mite Allergy (PIAMA) | National | both | 12-13 | 12-13 | 739 | 769 | |
| 2130 | Netherlands | 2008-2011 | Amsterdam Born Children and their Development Study (ABCD) | Community | both | 5-7 | 5-7 | 1,540 | 1,528 | |
| 2131 | Netherlands | 2009-2010 | Measuring the Netherlands (NL de Maat) | Subnational | both | 30-70 | 30-70 | 1,781 | 2,014 | |
| 2132 | Netherlands | 2009-2011 | The Rotterdam Study, first subcohort | Community | urban | 72+ | 72+ | 690 | 1,006 | |
| 2133 | Netherlands | 2010 | EuropeaN Energy balance Research to prevent excessive weight Gain among Youth - The ENERGY-project | National | both | 10-12 | 10-12 | 404 | 399 | |
| 2134 | Netherlands | 2011-2012 | The Rotterdam Study, second subcohort | Community | urban | 65+ | 65+ | 735 | 934 | |
| 2135 | Netherlands | 2011-2012 | The Longitudinal Aging Study Amsterdam (LASA) | Subnational | both | 63-104 | 63-104 | 532 | 653 | 19 |
| 2136 | Netherlands | 2011-2013 | GECKO Drenthe Onderzoek | Subnational | rural | 5-7 | 5-7 | 1,139 | 1,133 | |
| 2137 | Netherlands | 2011-2015 | Healthy Life in an Urban Setting (HELIUS) | Community | urban | 18-71 | 18-71 | 2,088 | 2,473 | |
| 2138 | Netherlands | 2012-2013 | The Longitudinal Aging Study Amsterdam (LASA) | Subnational | both | 55-65 | 55-65 | 426 | 448 | 19, 20 |
| 2139 | Netherlands | 2012-2014 | Prevention and Incidence of Asthma and Mite Allergy (PIAMA) | National | both | 15-17 | 15-17 | 386 | 414 | |
| 2140 | Netherlands | 2012-2014 | The Rotterdam Study, third subcohort | Community | urban | 52+ | 52+ | 1,256 | 1,639 | |
| 2141 | Netherlands | 2012-2016 | Amsterdam Born Children and their Development Study (ABCD) | Community | both | 9-12 | 9-12 | 1,120 | 1,136 | |
| 2142 | Netherlands | 2015-2016 | The Longitudinal Aging Study Amsterdam (LASA) | Subnational | both | 58+ | 58+ | 759 | 857 | 19 |
| 2143 | Netherlands | 2016-2018 | GECKO Drenthe Onderzoek | Subnational | rural | 8-12 | 8-12 | 1,083 | 1,114 | |
| 2144 | New Zealand | 1982 | MONICA, Auckland | Community | urban | 35-64 | 35-64 | 1,019 | 568 | |
| 2145 | New Zealand | 1989 | The Life in New Zealand Survey | National | both | 15+ | 15+ | 1,418 | 1,571 | |
| 2146 | New Zealand | 1990-1993 | Williams, N Z Med J 113(1114):308-11, 2000 | Community | both | 18-21 | 18-21 | 932 | 859 | |
| 2147 | New Zealand | 1993-1994 | MONICA, Auckland | Community | urban | 35-64 | 35-64 | 723 | 674 | |
| 2148 | New Zealand | 1996-1997 | National Nutrition Survey | National | both | 15+ | 15+ | 1,857 | 2,522 | |
| 2149 | New Zealand | 2002 | National Children’s Nutrition Survey | National | both | 5-14 | 5-14 | 1,564 | 1,485 | |
| 2150 | New Zealand | 2002-2003 | New Zealand Health Survey | National | both | 15+ | 15+ | 4,594 | 6,729 | |
| 2151 | New Zealand | 2006-2007 | New Zealand Health Survey | National | both | 5+ | 5+ | 6,766 | 8,029 | |
| 2152 | New Zealand | 2008-2009 | New Zealand Adult Nutrition Survey | National | both | 15+ | 15+ | 2,003 | 2,500 | |

| | Country | Study years | Survey/Study name/Citation | Level of representative-ness | Rural, urban, or both | Age range as in NCD-RisC database | | Sample size | | Notes |
|---|---|---|---|---|---|---|---|---|---|---|
| | | | | | | Male | Female | Male | Female | |
| 2153 | New Zealand | 2011-2012 | New Zealand Health Survey | National | both | 5+ | 5+ | 5,782 | 7,220 | |
| 2154 | New Zealand | 2012-2013 | New Zealand Health Survey | National | both | 5+ | 5+ | 6,412 | 7,932 | |
| 2155 | New Zealand | 2013-2014 | New Zealand Health Survey | National | both | 5+ | 5+ | 6,966 | 8,309 | |
| 2156 | New Zealand | 2014-2015 | New Zealand Health Survey | National | both | 5+ | 5+ | 7,124 | 8,437 | |
| 2157 | New Zealand | 2015-2016 | New Zealand Health Survey | National | both | 5+ | 5+ | 7,204 | 8,401 | |
| 2158 | New Zealand | 2016-2017 | New Zealand Health Survey | National | both | 5+ | 5+ | 6,944 | 8,275 | |
| 2159 | New Zealand | 2017-2018 | New Zealand Health Survey | National | both | 5+ | 5+ | 7,116 | 8,399 | |
| 2160 | New Zealand | 2018-2019 | New Zealand Health Survey | National | both | 5+ | 5+ | 6,809 | 8,461 | |
| 2161 | New Zealand | 2019-2020 | New Zealand Health Survey | National | both | 5+ | 5+ | 4,842 | 5,975 | |
| 2162 | New Zealand | 2020-2021 | New Zealand Health Survey | National | both | 5+ | 5+ | 4,595 | 5,488 | |
| 2163 | Nicaragua | 1997-1998 | DHS | National | both | | 15-49 | | 12,257 | |
| 2164 | Nicaragua | 2001 | DHS | National | both | | 15-49 | | 11,940 | |
| 2165 | Nicaragua | 2003-2004 | CAMDI | Community | urban | 20+ | 20+ | 773 | 916 | |
| 2166 | Nicaragua | 2003-2005 | Sistema Integrado de Vigilancia de Intervenciones Nutricionales (SIVIN) | National | both | | 15-50 | | 1,115 | |
| 2167 | Nicaragua | 2006-2007 | Encuesta Nicaraguense de Demografia y Salud | National | both | | 15-49 | | 13,216 | |
| 2168 | Nicaragua | 2011-2012 | Encuesta Nicaraguense de Demografia y Salud | National | both | | 15-49 | | 14,318 | |
| 2169 | Niger | 1992 | DHS | National | both | | 20-49 | | 2,993 | |
| 2170 | Niger | 1998 | DHS | National | both | | 20-49 | | 2,958 | |
| 2171 | Niger | 2006 | DHS | National | both | | 15-49 | | 4,151 | |
| 2172 | Niger | 2007 | STEPS | National | both | 15-64 | 15-64 | 1,430 | 1,215 | |
| 2173 | Niger | 2012 | DHS | National | both | | 15-49 | | 4,429 | |
| 2174 | Niger | 2021 | STEPS | National | both | 18-69 | 18-69 | 2,270 | 3,059 | |
| 2175 | Nigeria | 1990 | Non-communicable diseases National Survey | National | rural | 15+ | 15+ | 3,618 | 3,681 | |
| 2176 | Nigeria | 1990 | Non-communicable diseases National Survey | National | urban | 15+ | 15+ | 1,616 | 1,644 | |
| 2177 | Nigeria | 1991-1994 | Cooper et al., Am J Public Health 87(2):160-68, 1997 | Community | both | 20-100 | 20-100 | 910 | 1,080 | |
| 2178 | Nigeria | 1999 | DHS | National | both | | 20-49 | | 2,004 | |
| 2179 | Nigeria | 1999-2009 | Prostate cancer dietary risk factors study | Subnational | both | 35+ | | 627 | | |
| 2180 | Nigeria | 2003 | DHS | National | both | | 15-49 | | 6,605 | |
| 2181 | Nigeria | 2006 | Senbanjo et al., West Afr J Med 30(6):425-31, 2011 | Community | urban | 5-19 | 5-19 | 296 | 274 | |
| 2182 | Nigeria | 2007 | Ibadan Study of Ageing | Subnational | both | 60+ | 60+ | 642 | 914 | |
| 2183 | Nigeria | 2007 | Southeast Nigeria kidney disease study | Community | rural | 25-64 | 25-64 | 168 | 442 | |
| 2184 | Nigeria | 2008 | DHS | National | both | | 15-49 | | 28,973 | |
| 2185 | Nigeria | 2008 | Ibadan Study of Ageing | Subnational | both | 61+ | 61+ | 453 | 656 | |
| 2186 | Nigeria | 2009 | Ibadan Study of Ageing | Subnational | both | 62+ | 62+ | 420 | 619 | |
| 2187 | Nigeria | 2009 | Community Health Plan - Kwara Central Survey | Community | rural | 5+ | 5+ | 2,264 | 2,354 | |
| 2188 | Nigeria | 2009-2011 | Anthropometric indices in Calabar | Community | urban | 15-79 | 15-79 | 383 | 333 | |
| 2189 | Nigeria | 2010 | Nnewi obesity study | Community | urban | 19-85 | 19-85 | 774 | 746 | |
| 2190 | Nigeria | 2010-2011 | Nigeria built environment | Subnational | urban | 20-65 | 20-65 | 725 | 1,093 | |
| 2191 | Nigeria | 2011 | Community Health Plan - Kwara Central Survey | Community | rural | 5+ | 5+ | 791 | 853 | |
| 2192 | Nigeria | 2012 | Neighbourhood walkability and sedentary lifesytle | Community | urban | 60+ | 60+ | 212 | 141 | |
| 2193 | Nigeria | 2013 | DHS | National | both | | 15-49 | | 33,943 | |
| 2194 | Nigeria | 2013 | Community Health Plan - Kwara Central Survey | Community | rural | 5+ | 5+ | 714 | 754 | |
| 2195 | Nigeria | 2017 | ARISE Network Adolescent Health Study (Ibadan) | Community | urban | 10-19 | 10-19 | 336 | 407 | |
| 2196 | Nigeria | 2017-2019 | Removing the Mask on Hypertension (REMAH) | National | both | 18+ | 18+ | 1,806 | 2,353 | |
| 2197 | Nigeria | 2018 | Hypertension Prevalence, Awareness, Treatment and Control in Rural Area, Nigeria | Community | rural | 18+ | 18+ | 189 | 202 | |
| 2198 | Niue | 2010 | Global School-based Student Health Survey | National | both | 13-17 | 13-17 | 63 | 38 | |
| 2199 | Niue | 2011 | STEPS | National | both | 15+ | 15+ | 407 | 478 | |
| 2200 | Niue | 2019 | Global School-based Student Health Survey | National | both | 12-17 | 12-17 | 51 | 64 | |
| 2201 | North Macedonia | 1999 | Multiple Indicator Cluster Survey | National | both | | 15-45 | | 1,038 | |
| 2202 | North Macedonia | 2009 | Annual assessment of nutritional status of school children aged 7 years | National | both | 7 | 7 | 1,087 | 982 | |
| 2203 | North Macedonia | 2010 | Childhood Obesity Surveillance Initiative 2 | National | both | 7 | 7 | 1,427 | 1,311 | |
| 2204 | North Macedonia | 2013 | Childhood Obesity Surveillance Initiative 3 | National | both | 6-7 | 6-7 | 1,655 | 1,511 | |
| 2205 | North Macedonia | 2015-2016 | Childhood Obesity Surveillance Initiative 4 | National | both | 7-8 | 7-8 | 1,809 | 1,727 | |
| 2206 | North Macedonia | 2019 | Childhood Obesity Surveillance Initiative 5 | National | both | 7-8 | 7-8 | 1,579 | 1,611 | |
| 2207 | Norway | 1979-1980 | The Tromsø Study: Tromsø 2 | Community | both | 20-54 | 20-49 | 8,312 | 7,823 | |
| 2208 | Norway | 1984-1986 | HUNT1 study | Subnational | rural | 20+ | 20+ | 36,514 | 37,811 | |
| 2209 | Norway | 1986-1987 | The Tromsø Study: Tromsø 3 | Community | both | 20-61 | 20-56 | 10,374 | 9,804 | |

| | Country | Study years | Survey/Study name/Citation | Level of representative-ness | Rural, urban, or both | Age range as in NCD-RisC database | | Sample size | | Notes |
|---|---|---|---|---|---|---|---|---|---|---|
| | | | | | | Male | Female | Male | Female | |
| 2210 | Norway | 1992-1993 | The Hordaland Homocysteine Study 1925-1927 birth cohort | Subnational | both | 65-67 | 65-67 | 2,123 | 2,630 | |
| 2211 | Norway | 1992-1993 | The Hordaland Homocysteine Study 1928-1949 birth cohort | Subnational | urban | 43-64 | 43-64 | 335 | 348 | |
| 2212 | Norway | 1992-1993 | The Hordaland Homocysteine Study 1950-1952 birth cohort | Subnational | both | 40-42 | 40-42 | 6,103 | 6,475 | |
| 2213 | Norway | 1994-1995 | The Tromsø Study: Tromsø 4 | Community | both | 25+ | 25+ | 12,782 | 13,836 | |
| 2214 | Norway | 1995-1997 | HUNT2 study | Subnational | rural | 20+ | 20+ | 30,184 | 34,058 | |
| 2215 | Norway | 1995-1997 | YoungHUNT1 Study | Subnational | rural | 13-19 | 13-19 | 4,144 | 4,202 | |
| 2216 | Norway | 1997-1998 | The Hordaland Health Study (HUSK) 1925-1927 birth cohort | Subnational | both | 70-74 | 70-74 | 1,467 | 1,848 | |
| 2217 | Norway | 1997-1998 | The Hordaland Health Study (HUSK) 1950-1951 birth cohort | Subnational | both | 46-49 | 46-49 | 1,658 | 2,058 | |
| 2218 | Norway | 1997-1998 | The Hordaland Health Study (HUSK) 1953-1957 birth cohort | Subnational | both | 39-46 | 39-46 | 8,526 | 9,878 | |
| 2219 | Norway | 1999-2000 | European Youth Heart Study | Community | urban | 9-15 | 9-15 | 364 | 369 | |
| 2220 | Norway | 2000-2001 | YoungHUNT2 Study | Subnational | rural | 13-19 | 13-19 | 769 | 902 | |
| 2221 | Norway | 2000-2003 | The Oslo cohort (HUBRO), the Oppland and Hedmark cohort (OPPHED), and the Troms and Finnmark cohort (TROFINN) of Cohort of Norway (CONOR) | Subnational | both | 30-76 | 30-76 | 16,825 | 20,592 | |
| 2222 | Norway | 2001-2002 | The Tromsø Study: Tromsø 5, Tromsø Study Panel | Community | both | 30-89 | 30-89 | 2,525 | 3,579 | |
| 2223 | Norway | 2005-2006 | Physical Activity among Norwegian Children and Adolescents | National | both | 8-16 | 8-16 | 1,186 | 1,055 | |
| 2224 | Norway | 2006-2008 | HUNT3 study | Subnational | rural | 19+ | 19+ | 22,856 | 27,553 | |
| 2225 | Norway | 2006-2008 | YoungHUNT3 Study | Subnational | rural | 13-18 | 13-18 | 3,750 | 3,700 | |
| 2226 | Norway | 2007-2008 | The Tromsø Study: Tromsø 6 | Community | both | 30-87 | 30-87 | 6,048 | 6,889 | |
| 2227 | Norway | 2008 | Childhood Obesity Surveillance Initiative 1 | National | both | 8 | 8 | 1,435 | 1,399 | |
| 2228 | Norway | 2009-2010 | Trondheim Early Secure Study (TESS) | Community | urban | 6 | 6 | 328 | 322 | |
| 2229 | Norway | 2010 | Childhood Obesity Surveillance Initiative 2 | National | both | 8 | 8 | 1,335 | 1,286 | |
| 2230 | Norway | 2010 | EuropeaN Energy balance Research to prevent excessive weight Gain among Youth - The ENERGY-project | Subnational | urban | 10-13 | 10-13 | 458 | 491 | |
| 2231 | Norway | 2011-2012 | Trondheim Early Secure Study (TESS) | Community | urban | 8 | 8 | 331 | 344 | |
| 2232 | Norway | 2012 | Childhood Obesity Surveillance Initiative 3 | National | both | 8 | 8 | 1,492 | 1,381 | |
| 2233 | Norway | 2013-2014 | Trondheim Early Secure Study (TESS) | Community | urban | 10 | 10 | 332 | 360 | |
| 2234 | Norway | 2015-2016 | Childhood Obesity Surveillance Initiative 4 | National | both | 7-8 | 7-8 | 1,690 | 1,645 | |
| 2235 | Norway | 2015-2016 | Trondheim Early Secure Study (TESS) | Community | urban | 12 | 12 | 310 | 333 | |
| 2236 | Norway | 2017-2018 | Trondheim Early Secure Study (TESS) | Community | urban | 14 | 14 | 289 | 322 | |
| 2237 | Norway | 2017-2019 | HUNT4 study | Community | rural | 19+ | 19+ | 24,270 | 29,256 | |
| 2238 | Norway | 2017-2019 | YoungHUNT4 Study | Community | rural | 13-18 | 13-18 | 3,452 | 3,747 | |
| 2239 | Norway | 2018-2020 | The Hordaland Health Study (HUSK) 1950-1951 birth cohort | Subnational | both | 67-70 | 67-70 | 986 | 1,192 | |
| 2240 | Norway | 2019-2021 | Trondheim Early Secure Study (TESS) | Community | urban | 16 | 16 | 287 | 341 | |
| 2241 | Oman | 1991 | The 1991 National Diabetes Survey of Oman | National | both | 20+ | 20+ | 2,024 | 2,868 | |
| 2242 | Oman | 2000 | Oman National Health survey | National | both | 20+ | 20+ | 3,069 | 3,331 | |
| 2243 | Oman | 2001 | Nizwa Healthy Lifestyle Project | Community | urban | 20+ | 20+ | 707 | 726 | |
| 2244 | Oman | 2006 | STEPS | Community | urban | 20-59 | 20-59 | 540 | 732 | |
| 2245 | Oman | 2008 | Gulf Cooperation Council World Health Survey | National | both | 18+ | 18+ | 2,389 | 2,112 | |
| 2246 | Oman | 2010 | Global School-based Student Health Survey | National | both | 13-17 | 13-17 | 251 | 300 | |
| 2247 | Oman | 2015 | Global School-based Student Health Survey | National | both | 13-17 | 13-17 | 1,330 | 1,551 | |
| 2248 | Oman | 2017 | STEPS | National | both | 18+ | 18+ | 3,334 | 2,948 | |
| 2249 | Pakistan | 1990-1994 | MHS | Community | urban | 18+ | 18+ | 432 | 478 | |
| 2250 | Pakistan | 1990-1994 | National Health Survey of Pakistan 1990-1994 | National | both | 5+ | 5+ | 7,110 | 7,405 | |
| 2251 | Pakistan | 1999 | Shah et al., Trop Med Int Health 9(4):526-32, 2004 | Community | both | 18+ | 18+ | 1,391 | 2,754 | |
| 2252 | Pakistan | 2004-2005 | COBRA-1 | Community | urban | 40+ | 40+ | 1,500 | 1,635 | |
| 2253 | Pakistan | 2005 | STEPS | National | both | 25-65 | 25-65 | 787 | 1,071 | |
| 2254 | Pakistan | 2011 | National Nutrition Survey | National | both | 5-49 | 5-49 | 21,461 | 48,503 | |
| 2255 | Pakistan | 2012-2013 | DHS | National | both | | 20-49 | | 3,968 | |
| 2256 | Pakistan | 2014 | STEPS | Subnational | both | 18-69 | 18-69 | 2,964 | 3,674 | |
| 2257 | Pakistan | 2016-2017 | National Diabetes Survey of Pakistan | National | both | 20+ | 20+ | 3,771 | 4,647 | |
| 2258 | Pakistan | 2017-2018 | DHS | National | both | | 15-49 | | 3,637 | |
| 2259 | Pakistan | 2018-2019 | National Nutrition Survey | National | both | 5-19 | 5-49 | 11,888 | 127,307 | |
| 2260 | Palau | 2010 | School Health Screening | National | both | 5-17 | 5-17 | 537 | 511 | |
| 2261 | Palau | 2011 | School Health Screening | National | both | 5-17 | 5-17 | 613 | 599 | |
| 2262 | Palau | 2011-2013 | STEPS | National | both | 25-64 | 25-64 | 1,031 | 1,124 | |
| 2263 | Palau | 2012 | School Health Screening | National | both | 5-17 | 5-17 | 548 | 596 | |
| 2264 | Palau | 2013 | School Health Screening | National | both | 5-17 | 5-17 | 570 | 576 | |
| 2265 | Palau | 2014 | School Health Screening | National | both | 5-17 | 5-17 | 576 | 558 | |

| | Country | Study years | Survey/Study name/Citation | Level of representative-ness | Rural, urban, or both | Age range as in NCD-RisC database | | Sample size | | Notes |
|---|---|---|---|---|---|---|---|---|---|---|
| | | | | | | Male | Female | Male | Female | |
| 2266 | Palau | 2015 | School Health Screening | National | both | 5-17 | 5-17 | 522 | 565 | |
| 2267 | Palau | 2016 | School Health Screening | National | both | 5-17 | 5-17 | 569 | 563 | |
| 2268 | Palau | 2016 | STEPS | National | both | 18+ | 18+ | 713 | 717 | |
| 2269 | Palau | 2017 | School Health Screening | National | both | 5-17 | 5-17 | 484 | 522 | |
| 2270 | Palau | 2018 | School Health Screening | National | both | 5-17 | 5-17 | 598 | 554 | |
| 2271 | Palau | 2019 | School Health Screening | National | both | 5-17 | 5-17 | 504 | 561 | |
| 2272 | Palau | 2020 | School Health Screening | National | both | 5-17 | 5-17 | 591 | 568 | |
| 2273 | Palau | 2021 | School Health Screening | National | both | 5-17 | 5-17 | 600 | 598 | |
| 2274 | Palau | 2022 | School Health Screening | National | both | 5-17 | 5-17 | 525 | 515 | |
| 2275 | Panama | 2003 | Encuesta de Niveles de Vida | National | both | 5+ | 5+ | 10,808 | 11,133 | |
| 2276 | Panama | 2010-2011 | Prevalencia de factores de riesgo asociados a enfermedad cardiovascular 2010-2011 | Subnational | both | 18+ | 18+ | 1,067 | 2,469 | |
| 2277 | Panama | 2018 | Global School-based Student Health Survey | National | both | 13-17 | 13-17 | 1,121 | 1,384 | |
| 2278 | Panama | 2019 | Encuesta Nacional de Salud de Panama (ENSPA) | National | both | 15+ | 15+ | 4,288 | 10,851 | |
| 2279 | Papua New Guinea | 1985-1986 | INTERSALT | Community | rural | 20-59 | 20-59 | 88 | 74 | |
| 2280 | Papua New Guinea | 1991 | Dowse et al., Med J Aust 160:767-74, 1994 | Subnational | both | 25-88 | 25-88 | 836 | 1,012 | |
| 2281 | Papua New Guinea | 2007 | STEPS | National | both | 15-64 | 15-64 | 1,401 | 1,440 | |
| 2282 | Paraguay | 2011 | Primera Encuesta Nacional de Factores de Riesgo de Enfermedades No Transmisibles en la Poblacion General | National | both | 15-75 | 15-75 | 931 | 1,574 | |
| 2283 | Paraguay | 2017 | Global School-based Student Health Survey | National | both | 12-16 | 12-16 | 1,119 | 1,231 | |
| 2284 | Peru | 1991-1992 | DHS | National | both | | 15-49 | | 4,887 | |
| 2285 | Peru | 1996 | DHS | National | both | | 20-49 | | 10,125 | |
| 2286 | Peru | 2000 | DHS | National | both | | 15-49 | | 25,508 | |
| 2287 | Peru | 2003 | Factores de Riesgo de Enfermedades No Transmisibles | Community | urban | 16+ | 16+ | 327 | 503 | |
| 2288 | Peru | 2004 | Factores de Riesgo de Enfermedades No Transmisibles | Community | urban | 15+ | 15+ | 218 | 445 | |
| 2289 | Peru | 2004-2005 | CArdiovascular Risk factors Multiple Evaluation in Latin America (CARMELA) | Community | urban | 25-64 | 25-64 | 769 | 876 | |
| 2290 | Peru | 2004-2005 | Encuesta Nacional de Indicadores Nutricionales, Bioquímicos, Socioeconómicos y Culturales Relacionados con las Enfermedades Crónicas Degenerativas | National | both | 20+ | 20+ | 2,087 | 2,095 | |
| 2291 | Peru | 2004-2006 | DHS | National | both | | 15-49 | | 5,798 | |
| 2292 | Peru | 2005 | Factores de Riesgo de Enfermedades No Transmisibles | Community | urban | 15+ | 15+ | 209 | 550 | |
| 2293 | Peru | 2006 | Factores de Riesgo de Enfermedades No Transmisibles | Community | urban | 15+ | 15+ | 662 | 1,101 | |
| 2294 | Peru | 2007-2008 | Monitoreo de Indicadores Nutricionales en la ENAHO 2007-2008 | National | both | 5+ | 5+ | 15,041 | 16,282 | |
| 2295 | Peru | 2007-2008 | DHS | National | both | | 15-49 | | 20,918 | |
| 2296 | Peru | 2007-2008 | PERU MIGRANT Study | Community | both | 30+ | 30+ | 464 | 522 | |
| 2297 | Peru | 2007-2010 | Monitoreo Nacional de Indicadores Nutricionales | National | both | | 12-49 | | 3,804 | |
| 2298 | Peru | 2009 | DHS | National | both | | 15-49 | | 23,034 | |
| 2299 | Peru | 2009-2011 | Monitoreo de Indicadores Nutricionales en la ENAHO 2009-2010 | National | both | 5+ | 5+ | 27,753 | 31,269 | |
| 2300 | Peru | 2009-2012 | CRONICAS Cohort Study | Subnational | both | 35+ | 35+ | 1,557 | 1,660 | |
| 2301 | Peru | 2010 | DHS | National | both | | 15-49 | | 22,425 | |
| 2302 | Peru | 2010 | Global School-based Student Health Survey | National | both | 13-17 | 13-17 | 1,267 | 1,306 | |
| 2303 | Peru | 2010-2013 | CRONICAS Cohort Study | Subnational | both | 35+ | 35+ | 1,379 | 1,468 | |
| 2304 | Peru | 2011 | DHS | National | both | | 15-49 | | 22,215 | |
| 2305 | Peru | 2011-2012 | Monitoreo de Indicadores Nutricionales en la ENAHO 2011 | National | both | 5+ | 5+ | 7,424 | 8,424 | |
| 2306 | Peru | 2012 | DHS | National | both | | 15-49 | | 23,724 | |
| 2307 | Peru | 2012-2013 | PERU MIGRANT Study | Community | both | 35+ | 35+ | 339 | 427 | |
| 2308 | Peru | 2013 | DHS | National | both | 15+ | 15+ | 2,932 | 23,784 | |
| 2309 | Peru | 2013 | Clinical functional and sociofamilial profiles of the elderly from a community in a district of Lima, Peru | Community | urban | 60+ | 60+ | 185 | 309 | |
| 2310 | Peru | 2013-2014 | CRONICAS Cohort Study | Subnational | both | 36+ | 36+ | 1,292 | 1,361 | |
| 2311 | Peru | 2014 | DHS | National | both | 15+ | 15+ | 12,670 | 28,554 | |
| 2312 | Peru | 2014 | Launching a salt substitute to reduce blood pressure at the population level: a cluster randomized stepped wedge trial in Peru | Community | both | 18+ | 18+ | 1,149 | 1,166 | |
| 2313 | Peru | 2014-2015 | Latin American Study of Nutrition and Health (ELANS) | National | urban | 15-65 | 15-65 | 603 | 627 | |
| 2314 | Peru | 2015 | DHS | National | both | 15+ | 15+ | 14,744 | 38,415 | |
| 2315 | Peru | 2015-2016 | PERU MIGRANT Study | Community | both | 38+ | 38+ | 324 | 414 | |
| 2316 | Peru | 2016 | DHS | National | both | 15+ | 15+ | 14,035 | 35,998 | |
| 2317 | Peru | 2016-2017 | Screening of T2DM | Community | urban | 30-70 | 30-70 | 798 | 809 | |
| 2318 | Peru | 2017 | DHS | National | both | 15+ | 15+ | 14,340 | 37,386 | |
| 2319 | Peru | 2017-2018 | Vigilancia Alimentario Nutricional por Etapas de Vida (VIANEV) 2017-2018 | National | both | 18-59 | 18-59 | 465 | 618 | |

| | Country | Study years | Survey/Study name/Citation | Level of representative-ness | Rural, urban, or both | Age range as in NCD-RisC database | | Sample size | | Notes |
|---|---|---|---|---|---|---|---|---|---|---|
| | | | | | | Male | Female | Male | Female | |
| 2320 | Peru | 2018 | DHS | National | both | 15+ | 15+ | 14,600 | 39,575 | |
| 2321 | Peru | 2019 | DHS | National | both | 15+ | 15+ | 14,200 | 37,943 | |
| 2322 | Peru | 2020 | DHS | National | both | 15+ | 15+ | 9,926 | 25,781 | |
| 2323 | Peru | 2021 | DHS | National | both | 15+ | 15+ | 13,515 | 36,250 | |
| 2324 | Peru | 2022 | DHS | National | both | 15+ | 15+ | 13,673 | 35,914 | |
| 2325 | Philippines | 1983-1984 | Cebu Longitudinal Health and Nutrition Survey Baseline 2-Month Follow-up | Community | both | | 15-50 | | 2,866 | |
| 2326 | Philippines | 1983-1984 | Cebu Longitudinal Health and Nutrition Survey Baseline 4-Month Follow-up | Community | both | | 15-50 | | 2,728 | |
| 2327 | Philippines | 1983-1984 | Cebu Longitudinal Health and Nutrition Survey Baseline 6-Month Follow-up | Community | both | | 15-50 | | 2,601 | |
| 2328 | Philippines | 1984-1985 | Cebu Longitudinal Health and Nutrition Survey Baseline 8-Month Follow-up | Community | both | | 15-50 | | 2,473 | |
| 2329 | Philippines | 1984-1985 | Cebu Longitudinal Health and Nutrition Survey Baseline 10-Month Follow-up | Community | both | | 15-50 | | 2,348 | |
| 2330 | Philippines | 1984-1985 | Cebu Longitudinal Health and Nutrition Survey Baseline 12-Month Follow-up | Community | both | | 15-50 | | 2,263 | |
| 2331 | Philippines | 1984-1985 | Cebu Longitudinal Health and Nutrition Survey Baseline 14-Month Follow-up | Community | both | | 15-50 | | 2,193 | |
| 2332 | Philippines | 1984-1985 | Cebu Longitudinal Health and Nutrition Survey Baseline 16-Month Follow-up | Community | both | | 15-50 | | 2,129 | |
| 2333 | Philippines | 1984-1985 | Cebu Longitudinal Health and Nutrition Survey Baseline 18-Month Follow-up | Community | both | | 15-50 | | 2,079 | |
| 2334 | Philippines | 1985-1986 | Cebu Longitudinal Health and Nutrition Survey Baseline 20-Month Follow-up | Community | both | | 15-50 | | 2,047 | |
| 2335 | Philippines | 1985-1986 | Cebu Longitudinal Health and Nutrition Survey Baseline 22-Month Follow-up | Community | both | | 15-50 | | 2,017 | |
| 2336 | Philippines | 1985-1986 | Cebu Longitudinal Health and Nutrition Survey Baseline 24-Month Follow-up | Community | both | | 15-50 | | 2,022 | |
| 2337 | Philippines | 1988 | INCLEN | Community | rural | 35-65 | | 274 | | |
| 2338 | Philippines | 1991-1992 | Cebu Longitudinal Health and Nutrition Survey 1991 Child Follow-up | Community | both | 8 | 8 | 1,202 | 1,076 | |
| 2339 | Philippines | 1991-1992 | Cebu Longitudinal Health and Nutrition Survey 1991 Mother Follow-up | Community | both | | 22-55 | | 2,195 | |
| 2340 | Philippines | 1993 | 4th National Nutrition Survey | National | both | 20-70 | 20-70 | 4,383 | 4,754 | |
| 2341 | Philippines | 1993 | National Safe Motherhood Survey | National | both | | 15-49 | | 7,181 | |
| 2342 | Philippines | 1994-1995 | Cebu Longitudinal Health and Nutrition Survey 1994-1995 Mother Follow-up | Community | both | | 15-59 | | 2,692 | |
| 2343 | Philippines | 1998 | 5th National Nutrition Survey | National | both | 20-60 | 20-60 | 1,323 | 1,340 | |
| 2344 | Philippines | 1998-1999 | Cebu Longitudinal Health and Nutrition Survey 1998-1999 Child Follow-up | Community | both | 14-16 | 14-16 | 1,102 | 999 | |
| 2345 | Philippines | 1998-1999 | Cebu Longitudinal Health and Nutrition Survey 1998-1999 Mother Follow-up | Community | both | | 15-59 | | 1,911 | |
| 2346 | Philippines | 2002 | Cebu Longitudinal Health and Nutrition Survey 2002 Child Follow-up | Community | both | 17-19 | 17-19 | 1,087 | 907 | |
| 2347 | Philippines | 2002 | Cebu Longitudinal Health and Nutrition Survey 2002 Mother Follow-up | Community | both | | 32-66 | | 2,080 | |
| 2348 | Philippines | 2003 | Global School-based Student Health Survey | National | both | | 13 | | 291 | |
| 2349 | Philippines | 2003 | 6th National Nutrition Survey | National | both | 5+ | 5+ | 10,686 | 11,131 | |
| 2350 | Philippines | 2005 | Cebu Longitudinal Health and Nutrition Survey 2005 Child Follow-up | Community | both | 20-22 | 20-22 | 1,006 | 831 | |
| 2351 | Philippines | 2005 | Cebu Longitudinal Health and Nutrition Survey 2005 Mother Follow-up | Community | both | | 35-69 | | 2,001 | |
| 2352 | Philippines | 2007 | Cebu Longitudinal Health and Nutrition Survey 2007 Child Follow-up | Community | both | 23-24 | 23-24 | 937 | 751 | |
| 2353 | Philippines | 2007 | Cebu Longitudinal Health and Nutrition Survey 2007 Mother Follow-up | Community | both | | 38-71 | | 1,925 | |
| 2354 | Philippines | 2007 | Global School-based Student Health Survey | National | both | | 13 | | 254 | |
| 2355 | Philippines | 2008 | 7th National Nutrition Survey | National | both | 5+ | 5+ | 64,001 | 63,616 | |
| 2356 | Philippines | 2009 | Cebu Longitudinal Health and Nutrition Survey 2009 Child Follow-up | Community | both | 24-26 | 24-26 | 864 | 718 | |
| 2357 | Philippines | 2011 | Global School-based Student Health Survey | National | both | | 13 | | 540 | |
| 2358 | Philippines | 2011 | 2011 Updating of Nutritional Status of Filipino Children and Other Population Groups | National | both | 5+ | 5+ | 63,654 | 66,866 | |
| 2359 | Philippines | 2013-2014 | 8th National Nutrition Survey | National | both | 5+ | 5+ | 57,432 | 61,620 | |
| 2360 | Philippines | 2015 | Global School-based Student Health Survey | National | both | | 13-17 | | 3,730 | |
| 2361 | Philippines | 2015 | 2015 Updating of Nutritional Status of Filipino Children and Other Population Groups | National | both | 5+ | 5+ | 69,309 | 73,250 | |
| 2362 | Philippines | 2019 | Global School-based Student Health Survey | National | both | 12-16 | 12-16 | 3,571 | 4,286 | |
| 2363 | Poland | 1983-1984 | MONICA, Tarnobrzeg Voivodship | Community | rural | 35-64 | 35-64 | 1,236 | 1,441 | |
| 2364 | Poland | 1983-1985 | MONICA, Warsaw | Community | urban | 35-64 | 35-64 | 1,297 | 1,327 | |
| 2365 | Poland | 1986 | Poland Conscripts 10% Sample Cohort | National | both | 18-19 | | 29,421 | | |
| 2366 | Poland | 1986 | INTERSALT, Krakow | Community | urban | 20-59 | 20-59 | 100 | 100 | |
| 2367 | Poland | 1986 | INTERSALT, Warsaw | Community | urban | 20-59 | 20-59 | 100 | 100 | |
| 2368 | Poland | 1987-1988 | MONICA, Tarnobrzeg Voivodship | Community | rural | 35-64 | 35-64 | 616 | 672 | |
| 2369 | Poland | 1988 | Fourth National Survey | Subnational | both | 7-19 | 7-19 | 12,345 | 10,371 | |
| 2370 | Poland | 1988-1989 | MONICA, Warsaw | Community | urban | 35-64 | 35-64 | 705 | 713 | |
| 2371 | Poland | 1989-1990 | Polish Program CINDI (CINDI Lodz 1989-1990) | Community | urban | 25-64 | 25-64 | 831 | 957 | |
| 2372 | Poland | 1992-1993 | MONICA, Tarnobrzeg Voivodship | Community | rural | 35-64 | 35-64 | 618 | 692 | |
| 2373 | Poland | 1993 | MONICA, Warsaw | Community | urban | 35-64 | 35-64 | 751 | 763 | |
| 2374 | Poland | 1995 | Poland Conscripts 10% Sample Cohort | National | both | 18-19 | | 31,043 | | |
| 2375 | Poland | 1995-1996 | Polish Program CINDI (CINDI Lodz 1995) | Community | urban | 17-64 | 17-64 | 997 | 1,459 | |
| 2376 | Poland | 1997 | Wroclaw survey in adolescents | Community | both | 12-17 | 12-17 | 2,198 | 2,141 | |

| | Country | Study years | Survey/Study name/Citation | Level of representative-ness | Rural, urban, or both | Age range as in NCD-RisC database | | Sample size | | Notes |
|---|---|---|---|---|---|---|---|---|---|---|
| | | | | | | Male | Female | Male | Female | |
| 2377 | Poland | 2000 | The health status, risk factors of chronic diseases and health behaviors of residents of Torun (CINDI Torun 2000) | Community | urban | 16-83 | 16-83 | 989 | 1,054 | |
| 2378 | Poland | 2000 | Binkowska-Bury et al., Neuro Endocrinol Lett 34(8):814-20, 2013 | Subnational | both | 19-20 | | 3,003 | | |
| 2379 | Poland | 2000-2001 | Household Food Consumption and Anthropometric Survey | National | both | 5+ | 5+ | 1,766 | 2,107 | |
| 2380 | Poland | 2001 | Binkowska-Bury et al., Neuro Endocrinol Lett 34(8):814-20, 2013 | Subnational | both | 19-20 | | 3,420 | | |
| 2381 | Poland | 2001 | Poland Conscripts 10% Sample Cohort | National | both | 18-19 | | 31,213 | | |
| 2382 | Poland | 2001-2002 | The health status, risk factors of chronic diseases and health behaviors of residents of Lodz (CINDI Lodz 2001) | Community | urban | 18-64 | 18-64 | 1,000 | 840 | |
| 2383 | Poland | 2001-2002 | Young Men Cardiovascular Association Study | Community | urban | 16+ | | 1,156 | | 21 |
| 2384 | Poland | 2002 | The health status, risk factors of chronic diseases and health behaviors of residents of Lodz - seniors (CINDI Lodz 2002) | Community | urban | 65+ | 65+ | 285 | 532 | |
| 2385 | Poland | 2002 | Binkowska-Bury et al., Neuro Endocrinol Lett 34(8):814-20, 2013 | Subnational | both | 19-20 | | 3,544 | | |
| 2386 | Poland | 2002 | NATPOL | National | both | 18+ | 18+ | 1,018 | 1,301 | |
| 2387 | Poland | 2002-2005 | Health, Alcohol and Psychosocial Factors In Eastern Europe | Community | urban | 45-70 | 45-70 | 4,502 | 4,752 | |
| 2388 | Poland | 2003 | Binkowska-Bury et al., Neuro Endocrinol Lett 34(8):814-20, 2013 | Subnational | both | 19-20 | | 3,633 | | |
| 2389 | Poland | 2003 | The European Male Ageing Study | Community | both | 40+ | | 406 | | |
| 2390 | Poland | 2003-2005 | National Multicenter Health Survey in Poland. Project WOBASZ | National | both | 20-74 | 20-74 | 6,245 | 6,910 | |
| 2391 | Poland | 2003-2006 | Mogielica Human Ecology Study Site | Community | rural | 18+ | 18+ | 119 | 321 | |
| 2392 | Poland | 2004 | Binkowska-Bury et al., Neuro Endocrinol Lett 34(8):814-20, 2013 | Subnational | both | 19-20 | | 3,538 | | |
| 2393 | Poland | 2004 | LIPIDOGRAM2004 Study - National epidemiological study of lipid disorders and selected risk factors of cardiovascular disease in primary health care in Poland | National | both | 30+ | 30+ | 6,673 | 9,920 | |
| 2394 | Poland | 2005 | Binkowska-Bury et al., Neuro Endocrinol Lett 34(8):814-20, 2013 | Subnational | both | 19-20 | | 3,308 | | |
| 2395 | Poland | 2006 | The health, risk factors for chronic diseases, attitudes and behaviors of health residents of Torun (CINDI Torun 2006) | Community | urban | 15-65 | 15-65 | 790 | 1,147 | |
| 2396 | Poland | 2006 | Binkowska-Bury et al., Neuro Endocrinol Lett 34(8):814-20, 2013 | Subnational | both | 19-20 | | 3,701 | | |
| 2397 | Poland | 2006 | LIPIDOGRAM2006 Study - National epidemiological study of lipid disorders and selected risk factors of cardiovascular disease in primary health care in Poland | National | both | 32+ | 32+ | 6,441 | 10,640 | |
| 2398 | Poland | 2006-2007 | National Multicenter Health Survey in Poland. Project WOBASZ Senior | National | both | 75+ | 75+ | 541 | 533 | |
| 2399 | Poland | 2007 | Binkowska-Bury et al., Neuro Endocrinol Lett 34(8):814-20, 2013 | Subnational | both | 19-20 | | 3,612 | | |
| 2400 | Poland | 2007-2009 | Elaboration of the reference range of arterial blood pressure for the population of children and adolescents in Poland - PL0080 OLAF - Primary | National | both | 6-16 | 6-16 | 6,497 | 6,603 | |
| 2401 | Poland | 2007-2009 | Elaboration of the reference range of arterial blood pressure for the population of children and adolescents in Poland - PL0080 OLAF - Secondary | National | both | 16-18 | 16-18 | 1,852 | 2,520 | |
| 2402 | Poland | 2007-2010 | Mogielica Human Ecology Study Site | Community | rural | 18+ | 18+ | 133 | 290 | |
| 2403 | Poland | 2007-2011 | Medical, psychological and socioeconomic aspects of aging in Poland | National | both | 55+ | 55+ | 2,682 | 2,507 | |
| 2404 | Poland | 2008 | Binkowska-Bury et al., Neuro Endocrinol Lett 34(8):814-20, 2013 | Subnational | both | 19-20 | | 3,435 | | |
| 2405 | Poland | 2008 | The European Male Ageing Study | Community | both | 40+ | | 310 | | |
| 2406 | Poland | 2009 | Binkowska-Bury et al., Neuro Endocrinol Lett 34(8):814-20, 2013 | Subnational | both | 19-20 | | 3,405 | | |
| 2407 | Poland | 2009-2010 | Poland Conscripts 10% Sample Cohort | National | both | 18-19 | | 9,208 | | |
| 2408 | Poland | 2010 | Binkowska-Bury et al., Neuro Endocrinol Lett 34(8):814-20, 2013 | Subnational | both | 19-20 | | 3,317 | | |
| 2409 | Poland | 2010-2012 | Blood pressure references for Polish preschool children - the OLA study | National | both | 5-6 | 5-6 | 926 | 904 | |
| 2410 | Poland | 2011 | NATPOL | National | both | 18-79 | 18-79 | 1,158 | 1,235 | |
| 2411 | Poland | 2011-2014 | Mogielica Human Ecology Study Site | Community | rural | 18+ | 18+ | 142 | 418 | |
| 2412 | Poland | 2012-2013 | Fifth National Survey | Subnational | both | 6-19 | 6-19 | 3,091 | 2,686 | |
| 2413 | Poland | 2013-2014 | National Multicenter Health Survey in Poland. Project WOBASZ II | National | both | 20+ | 20+ | 2,626 | 3,198 | |
| 2414 | Poland | 2014 | Prevalence of risk factors for obesity and hypertension among Polish children and adolescents | Subnational | both | 7-18 | 7-18 | 284 | 283 | |
| 2415 | Poland | 2014-2017 | The impact of physical activity and selected perinatal risk factors on the occurrence of overweight and obesity and hypertension in children | Subnational | both | 5-15 | 5-15 | 516 | 454 | |
| 2416 | Poland | 2015-2016 | Childhood Obesity Surveillance Initiative 4 | National | both | 8 | 8 | 1,675 | 1,664 | |
| 2417 | Poland | 2015-2016 | LIPIDOGRAM2015 & LIPIDOGEN2015 Study - National epidemiological study of lipid disorders and selected risk factors of cardiovascular disease in primary health care in Poland | National | both | 18+ | 18+ | 5,034 | 8,690 | |
| 2418 | Poland | 2016 | Body fat in Polish adolescents | Community | urban | 11-13 | 11-13 | 76 | 82 | |
| 2419 | Poland | 2016-2017 | Erasmus plus KA2, Healthyland | Community | urban | 6 | 6 | 25 | 25 | |
| 2420 | Poland | 2016-2017 | The occurrence of overweight and obesity in children from different place of residence | Subnational | both | 7-13 | 7-13 | 164 | 151 | |
| 2421 | Poland | 2016-2020 | The prevalence of overweight and obesity and the assessment of body balance among children in a rural area in Poland | Subnational | rural | 7-15 | 7-15 | 580 | 557 | |

| | Country | Study years | Survey/Study name/Citation | Level of representative-ness | Rural, urban, or both | Age range as in NCD-RisC database | | Sample size | | Notes |
|---|---|---|---|---|---|---|---|---|---|---|
| | | | | | | Male | Female | Male | Female | |
| 2422 | Poland | 2017 | Multivariate assessment of the occurrence of noncommunicable diseases and their risk factors among preschool children | Community | urban | 5-7 | 5-7 | 226 | 206 | |
| 2423 | Poland | 2017 | Preferences for sweet and fatty taste in children and their mothers in association with weight status | Subnational | both | 8-15 | 8-15 | 138 | 150 | |
| 2424 | Poland | 2017 | Risk factors for obesity among Polish adolescents | Community | both | 14-19 | 14-19 | 40 | 38 | |
| 2425 | Poland | 2017-2018 | Erasmus plus KA2, Healthyland | Community | urban | 5-6 | 5-6 | 24 | 26 | |
| 2426 | Poland | 2018 | Childhood Obesity Surveillance Initiative 5 | National | both | 8 | 8 | 1,387 | 1,303 | |
| 2427 | Poland | 2018 | Mogielica Human Ecology Study Site | Community | rural | 18+ | 18+ | 30 | 95 | |
| 2428 | Poland | 2018 | Multivariate assessment of the occurrence of noncommunicable diseases and their risk factors among preschool children | Community | urban | 5-7 | 5-7 | 393 | 385 | |
| 2429 | Poland | 2018-2020 | Child of Kraków 2020 | Community | urban | 5-18 | 5-18 | 1,382 | 1,487 | |
| 2430 | Poland | 2019 | Multivariate assessment of the occurrence of noncommunicable diseases and their risk factors among preschool children | Community | urban | 5-7 | 5-7 | 395 | 375 | |
| 2431 | Poland | 2019-2020 | Assessment of the olfactory and taste functions of children with type 1 diabetes | Community | rural | 10-15 | 10-15 | 54 | 46 | |
| 2432 | Poland | 2019-2020 | Child health status and risk factors | Subnational | both | 6-17 | 6-17 | 371 | 329 | |
| 2433 | Portugal | 1980-1982 | Growth of children from the countryside of Portugal | Community | both | 6-15 | 6-15 | 1,349 | 1,316 | |
| 2434 | Portugal | 1985 | Body-Mass Index of Portuguese Conscripts | National | both | 18-20 | | 29,420 | | |
| 2435 | Portugal | 1986 | Body-Mass Index of Portuguese Conscripts | National | both | 18-20 | | 70,504 | | |
| 2436 | Portugal | 1986 | INTERSALT | Community | rural | 20-59 | 20-59 | 99 | 99 | |
| 2437 | Portugal | 1987 | Body-Mass Index of Portuguese Conscripts | National | both | 18-20 | | 68,079 | | |
| 2438 | Portugal | 1988 | Body-Mass Index of Portuguese Conscripts | National | both | 18-20 | | 67,573 | | |
| 2439 | Portugal | 1989 | Body-Mass Index of Portuguese Conscripts | National | both | 18-20 | | 68,827 | | |
| 2440 | Portugal | 1990 | Body-Mass Index of Portuguese Conscripts | National | both | 18-20 | | 44,359 | | |
| 2441 | Portugal | 1991 | Body-Mass Index of Portuguese Conscripts | National | both | 18-20 | | 19,552 | | |
| 2442 | Portugal | 1992 | Body-Mass Index of Portuguese Conscripts | National | both | 18-20 | | 52,393 | | |
| 2443 | Portugal | 1993 | Body-Mass Index of Portuguese Conscripts | National | both | 18-20 | | 59,780 | | |
| 2444 | Portugal | 1994 | Body-Mass Index of Portuguese Conscripts | National | both | 18-20 | | 55,511 | | |
| 2445 | Portugal | 1995 | Body-Mass Index of Portuguese Conscripts | National | both | 18-20 | | 68,221 | | |
| 2446 | Portugal | 1996 | Body-Mass Index of Portuguese Conscripts | National | both | 18-21 | | 106,097 | | |
| 2447 | Portugal | 1997 | Body-Mass Index of Portuguese Conscripts | National | both | 18-21 | | 61,215 | | |
| 2448 | Portugal | 1998 | Body-Mass Index of Portuguese Conscripts | National | both | 18-21 | | 41,027 | | |
| 2449 | Portugal | 1998-2000 | European Youth Heart Study | Community | both | 9-16 | 9-16 | 554 | 535 | |
| 2450 | Portugal | 1999 | Body-Mass Index of Portuguese Conscripts | National | both | 18-21 | | 54,187 | | |
| 2451 | Portugal | 1999-2003 | EPIPorto study | Community | urban | 18+ | 18+ | 932 | 1,507 | |
| 2452 | Portugal | 2000 | Body-Mass Index of Portuguese Conscripts | National | both | 18-21 | | 53,326 | | |
| 2453 | Portugal | 2003-2004 | EPITeen - Epidemiological Health Investigation of Teenagers in Porto | Community | urban | 13-14 | 13-14 | 981 | 1,048 | |
| 2454 | Portugal | 2003-2005 | Estudo de Prevalência da Obesidade e Consumos Alimentares em Portugal | National | both | 18-64 | 18-64 | 3,796 | 4,320 | |
| 2455 | Portugal | 2004 | Growth of adolescents in Coimbra | Community | both | 9-16 | 9-16 | 265 | 408 | |
| 2456 | Portugal | 2004 | Growth of adolescents in Gouveia | Community | rural | 10-19 | 10-19 | 238 | 246 | |
| 2457 | Portugal | 2007 | Growth of adolescents in Tondela | Community | rural | 6-19 | 6-19 | 314 | 312 | |
| 2458 | Portugal | 2007-2008 | EPITeen - Epidemiological Health Investigation of Teenagers in Porto | Community | urban | 16-17 | 16-17 | 1,186 | 1,251 | |
| 2459 | Portugal | 2007-2008 | European Youth Heart Study | Community | both | 8-17 | 8-17 | 315 | 311 | |
| 2460 | Portugal | 2007-2008 | Primary schools health promotion | Community | urban | 6-12 | 6-12 | 224 | 238 | |
| 2461 | Portugal | 2007-2009 | Portuguese National Survey of Physical Activity and Physical Fitness | National | both | 9+ | 9+ | 14,914 | 18,025 | |
| 2462 | Portugal | 2007-2010 | Promoção do Exercício e Saúde no Sedentarismo e Obesidade da Adolescência (PESSOA Program) | Community | urban | 9-16 | 9-16 | 1,931 | 1,813 | |
| 2463 | Portugal | 2007-2010 | The Midland Adolescent Lifestyle Study | Subnational | both | 12-16 | 12-16 | 196 | 235 | |
| 2464 | Portugal | 2008 | Azorean Physical Activity and Health Study II | Subnational | urban | 15-18 | 15-18 | 608 | 893 | |
| 2465 | Portugal | 2008 | Childhood Obesity Surveillance Initiative 1 | National | both | 6-8 | 6-8 | 1,801 | 1,792 | |
| 2466 | Portugal | 2008-2013 | Preschool Physical Activity, Body Composition and Lifestyle Study (PRESTYLE) | Community | urban | 5-6 | 5-6 | 651 | 607 | |
| 2467 | Portugal | 2009 | Bracara Study | Community | urban | 8-14 | 8-14 | 398 | 336 | |
| 2468 | Portugal | 2009-2010 | Portuguese Prevelance Study of Obesity in Childhood | National | both | 5-10 | 5-10 | 6,804 | 7,099 | |
| 2469 | Portugal | 2010 | Childhood Obesity Surveillance Initiative 2 | National | both | 6-8 | 6-8 | 1,830 | 1,826 | |
| 2470 | Portugal | 2010-2012 | Exercise for Elderly | Community | urban | 60-84 | 60-84 | 48 | 104 | |
| 2471 | Portugal | 2010-2012 | Promoção do Exercício e Saúde no Sedentarismo e Obesidade da Adolescência (PESSOA Program) | Community | urban | 9-14 | 9-14 | 287 | 275 | |
| 2472 | Portugal | 2011-2012 | The association of childhood obesity with asthma and rhinitis symptoms in 6-8 years old children living in the Coimbra district, Portugal: the role of environmental, family and socioeconomic factors | Community | both | 6-8 | 6-8 | 480 | 504 | |
| 2473 | Portugal | 2011-2013 | EPITeen - Epidemiological Health Investigation of Teenagers in Porto | Community | urban | 20-23 | 20-23 | 854 | 895 | |
| 2474 | Portugal | 2011-2013 | International Study of Childhood Obesity, Lifestyle and the Environment (ISCOLE) | Community | urban | 9-11 | 9-11 | 358 | 419 | |

| | Country | Study years | Survey/Study name/Citation | Level of representative-ness | Rural, urban, or both | Age range as in NCD-RisC database | | Sample size | | Notes |
|---|---|---|---|---|---|---|---|---|---|---|
| | | | | | | Male | Female | Male | Female | |
| 2475 | Portugal | 2011-2013 | Environmental Support for Leisure and Active Transport | Subnational | urban | 10-15 | 10-15 | 294 | 340 | |
| 2476 | Portugal | 2011-2014 | Longitudinal Analysis of Biomarkers and Environmental Determinants of Physical activity (LABMED Study) | Subnational | urban | 12-18 | 12-18 | 531 | 460 | |
| 2477 | Portugal | 2013 | Childhood Obesity Surveillance Initiative 3 | National | both | 6-8 | 6-8 | 2,951 | 2,982 | |
| 2478 | Portugal | 2013 | Childhood obesity in Lousao | Community | rural | 5-14 | 5-14 | 444 | 418 | |
| 2479 | Portugal | 2013-2014 | Cultural, social, economic, and environmental factors that can influence children's sport participation and obesity levels | Community | urban | 6-10 | 6-10 | 385 | 408 | |
| 2480 | Portugal | 2015 | Inquérito Nacional de Saúde com Exame Físico (INSEF) | National | both | 25-74 | 25-74 | 2,248 | 2,639 | |
| 2481 | Portugal | 2015-2016 | Childhood Obesity Surveillance Initiative 4 | National | both | 6-8 | 6-8 | 3,346 | 3,399 | |
| 2482 | Portugal | 2016-2017 | Overweight and obesity and their associated factors among early adolescence school children in urban and rural Portugal | National | rural | 10-12 | 10-12 | 38 | 33 | |
| 2483 | Portugal | 2016-2017 | Portuguese Prevalence Study of Obesity in Childhood | Subnational | both | 5-10 | 5-10 | 3,572 | 3,522 | |
| 2484 | Portugal | 2018-2019 | Childhood Obesity Surveillance Initiative 5 | National | both | 6-8 | 6-8 | 3,626 | 3,429 | |
| 2485 | Puerto Rico | 2002-2003 | Puerto Rican Elderly: Health Conditions | National | both | 60+ | 60+ | 1,914 | 2,850 | 22 |
| 2486 | Puerto Rico | 2005-2007 | Perez et al., Ethn Dis 18(4):434-41, 2008 | Community | urban | 15-84 | 15-84 | 275 | 529 | |
| 2487 | Puerto Rico | 2006-2007 | Puerto Rican Elderly: Health Conditions | National | both | 60+ | 60+ | 1,056 | 1,669 | 22 |
| 2488 | Puerto Rico | 2010-2013 | HPV Infection in a Population-Based Sample of Puerto Rican Women | Subnational | both | | 16-64 | | 563 | |
| 2489 | Qatar | 2006 | World Health Survey | National | both | 18+ | 18+ | 1,859 | 2,018 | |
| 2490 | Qatar | 2011 | Global School-based Student Health Survey | National | both | 13 | 13 | 102 | 126 | |
| 2491 | Qatar | 2012 | STEPS | National | both | 18-64 | 18-64 | 1,034 | 1,353 | |
| 2492 | Romania | 1986-1987 | MONICA, Bucharest | Community | urban | 25-64 | 25-64 | 702 | 873 | |
| 2493 | Romania | 1997 | Somatometria | National | both | 25-75 | 25-75 | 3,142 | 4,063 | |
| 2494 | Romania | 1999 | Romania physical development rural data | National | rural | 5-18 | 5-18 | 20,452 | 20,043 | |
| 2495 | Romania | 1999 | Romania physical development urban data | National | urban | 5-18 | 5-18 | 21,423 | 23,605 | |
| 2496 | Romania | 2006-2008 | Hypertension in Romanian Children and Adolescents: A Cross-Sectional Survey | Subnational | both | 5-17 | 5-17 | 2,313 | 2,339 | |
| 2497 | Romania | 2008 | Healthy traditions for healthy children | Community | rural | 5-11 | 5-11 | 74 | 69 | |
| 2498 | Romania | 2008-2009 | Healthy traditions for healthy children | Community | urban | 7-11 | 7-11 | 562 | 525 | |
| 2499 | Romania | 2009-2011 | Study on children in Dolj County, South Romania | Subnational | both | 5-21 | 5-21 | 746 | 672 | |
| 2500 | Romania | 2010-2013 | Healthy traditions for healthy children | Community | urban | 5-11 | 5-11 | 1,306 | 1,362 | |
| 2501 | Romania | 2011-2012 | SEPHAR II (Study for the Evaluation of Prevalence of Hypertension and Cardiovascular Risk in Romania - 2nd edition | National | both | 18-80 | 18-80 | 927 | 1,023 | |
| 2502 | Romania | 2012 | Healthy traditions for healthy children | Community | rural | 5-10 | 5-10 | 98 | 75 | |
| 2503 | Romania | 2012-2014 | PREDATORR | National | both | 20-79 | 20-79 | 1,284 | 1,431 | |
| 2504 | Romania | 2013 | Childhood Obesity Surveillance Initiative 3 | National | both | 8 | 8 | 2,175 | 2,173 | |
| 2505 | Romania | 2013 | Healthy traditions for healthy children | Subnational | rural | 6-10 | 6-10 | 121 | 107 | |
| 2506 | Romania | 2013-2014 | Auxological evaluation of school children in Mures County | Subnational | both | 6-14 | 6-14 | 936 | 957 | |
| 2507 | Romania | 2014 | Timis County Study | Community | urban | 6-19 | 6-19 | 237 | 205 | |
| 2508 | Romania | 2014-2015 | Healthy traditions for healthy children | Subnational | rural | 5-11 | 5-11 | 599 | 554 | |
| 2509 | Romania | 2014-2015 | Healthy traditions for healthy children | Community | urban | 5-10 | 5-10 | 393 | 427 | |
| 2510 | Romania | 2015-2016 | Childhood Obesity Surveillance Initiative 4 | National | both | 7-9 | 7-9 | 3,699 | 3,595 | |
| 2511 | Romania | 2015-2016 | SEPHAR III (Study for the Evaluation of Prevalence of Hypertension and Cardiovascular Risk in Romania - 3rd edition | National | both | 18-80 | 18-80 | 936 | 1,034 | |
| 2512 | Romania | 2016 | Healthy traditions for healthy children | Community | rural | 5-11 | 5-11 | 352 | 313 | |
| 2513 | Romania | 2016 | Healthy traditions for healthy children | Community | urban | 5-11 | 5-11 | 259 | 255 | |
| 2514 | Romania | 2016-2017 | Erasmus plus KA2, Healthyland | Community | urban | 5-6 | 5-6 | 14 | 16 | |
| 2515 | Romania | 2017 | Healthy traditions for healthy children | Community | rural | 5-11 | 5-11 | 226 | 231 | |
| 2516 | Romania | 2017 | Healthy traditions for healthy children | Community | urban | 5-11 | 5-11 | 582 | 588 | |
| 2517 | Romania | 2017-2018 | Erasmus plus KA2, Healthyland | Community | urban | 5-7 | 5-7 | 31 | 34 | |
| 2518 | Romania | 2018 | Healthy traditions for healthy children | Community | rural | 5-11 | 5-11 | 456 | 404 | |
| 2519 | Romania | 2018 | Healthy traditions for healthy children | Community | urban | 5-11 | 5-11 | 679 | 681 | |
| 2520 | Romania | 2019 | Childhood Obesity Surveillance Initiative 5 | National | both | 7-9 | 7-9 | 5,234 | 5,142 | |
| 2521 | Romania | 2019 | Healthy traditions for healthy children | Community | rural | 5-11 | 5-11 | 78 | 92 | |
| 2522 | Romania | 2019 | Healthy traditions for healthy children | Community | urban | 5-11 | 5-11 | 353 | 340 | |
| 2523 | Romania | 2019 | Resita preschool measurements | Community | urban | 5-8 | 5-8 | 232 | 236 | |
| 2524 | Romania | 2022 | Healthy traditions for healthy children | Community | rural | 5-11 | 5-11 | 232 | 210 | |
| 2525 | Romania | 2022 | Healthy traditions for healthy children | Community | urban | 5-11 | 5-11 | 1,163 | 982 | |
| 2526 | Russian Federation | 1984-1985 | Novosibirsk cohort semi-MONICA | Community | urban | 23-63 | | 1,603 | | |
| 2527 | Russian Federation | 1984-1986 | MONICA, Moscow (control) | Community | urban | 35-64 | 35-64 | 774 | 642 | |

| | Country | Study years | Survey/Study name/Citation | Level of representative-ness | Rural, urban, or both | Age range as in NCD-RisC database | | Sample size | | Notes |
|---|---|---|---|---|---|---|---|---|---|---|
| | | | | | | Male | Female | Male | Female | |
| 2528 | Russian Federation | 1984-1986 | MONICA, Moscow, Leninsky district | Community | urban | 35-64 | 35-64 | 553 | 622 | |
| 2529 | Russian Federation | 1984-1986 | MONICA, Moscow, Cheremushkinsky district | Community | urban | 35-64 | 35-64 | 580 | 579 | |
| 2530 | Russian Federation | 1985 | MONICA, Novosibirsk (intervention) | Community | urban | 25-64 | 25-64 | 797 | 818 | |
| 2531 | Russian Federation | 1985-1986 | MONICA, Novosibirsk, Kirowsky district | Community | urban | 25-64 | 25-64 | 758 | 774 | |
| 2532 | Russian Federation | 1985-1986 | MONICA, Novosibirsk, Leninsky district | Community | urban | 25-64 | 25-64 | 624 | 624 | |
| 2533 | Russian Federation | 1986 | INTERSALT | Community | urban | 20-59 | 20-59 | 97 | 97 | |
| 2534 | Russian Federation | 1988 | MONICA, Novosibirsk (intervention) | Community | urban | 25-64 | 25-64 | 837 | 852 | |
| 2535 | Russian Federation | 1988-1989 | MONICA, Moscow (control) | Community | urban | 35-64 | 35-64 | 620 | 581 | |
| 2536 | Russian Federation | 1988-1989 | MONICA, Moscow, Leninsky district | Community | urban | 35-64 | 35-64 | 597 | 612 | |
| 2537 | Russian Federation | 1988-1989 | MONICA, Novosibirsk, Kirowsky district | Community | urban | 25-64 | 25-64 | 871 | 705 | |
| 2538 | Russian Federation | 1992 | CINDI | Community | rural | 25-64 | 25-64 | 377 | 453 | |
| 2539 | Russian Federation | 1992 | Russian Karelia Survey in Pitkaranta | Community | both | 25-64 | 25-64 | 380 | 455 | |
| 2540 | Russian Federation | 1992-1993 | Russia Longitudinal Monitoring Survey- Higher School of Economics Round II | National | both | 5+ | 5+ | 4,764 | 6,348 | |
| 2541 | Russian Federation | 1992-1995 | MONICA, Moscow (control) | Community | urban | 35-64 | 35-64 | 556 | 527 | |
| 2542 | Russian Federation | 1992-1995 | MONICA, Moscow, Leninsky district | Community | urban | 35-64 | 35-64 | 538 | 858 | |
| 2543 | Russian Federation | 1993 | Russia Longitudinal Monitoring Survey- Higher School of Economics Round III | National | both | 5+ | 5+ | 6,009 | 7,685 | |
| 2544 | Russian Federation | 1993-1994 | Russia Longitudinal Monitoring Survey- Higher School of Economics Round IV | National | both | 5+ | 5+ | 5,519 | 7,094 | |
| 2545 | Russian Federation | 1994 | Russia Longitudinal Monitoring Survey- Higher School of Economics Round V | National | both | 5+ | 5+ | 4,726 | 5,788 | |
| 2546 | Russian Federation | 1994-1995 | MONICA, Novosibirsk (intervention) | Community | urban | 25-64 | 25-64 | 820 | 860 | |
| 2547 | Russian Federation | 1995 | MONICA, Novosibirsk, Kirowsky district | Community | urban | 25-64 | 25-64 | 771 | 787 | |
| 2548 | Russian Federation | 1995 | Russia Longitudinal Monitoring Survey- Higher School of Economics Round VI | National | both | 5+ | 5+ | 4,463 | 5,509 | |
| 2549 | Russian Federation | 1996 | Russia Longitudinal Monitoring Survey- Higher School of Economics Round VII | National | both | 5+ | 5+ | 4,261 | 5,300 | |
| 2550 | Russian Federation | 1997 | Russian Karelia Survey in Pitkaranta | Community | both | 25-64 | 25-64 | 309 | 440 | |
| 2551 | Russian Federation | 1998-1999 | Russia Longitudinal Monitoring Survey- Higher School of Economics Round VIII | National | both | 5+ | 5+ | 4,053 | 5,073 | |
| 2552 | Russian Federation | 2000 | Russia Longitudinal Monitoring Survey- Higher School of Economics Round IX | National | both | 5+ | 5+ | 3,787 | 4,807 | |
| 2553 | Russian Federation | 2001 | Russia Longitudinal Monitoring Survey- Higher School of Economics Round X | National | both | 5+ | 5+ | 3,842 | 5,037 | |
| 2554 | Russian Federation | 2002 | Russian Karelia Survey in Pitkaranta | Community | both | 25-64 | 25-64 | 251 | 334 | |
| 2555 | Russian Federation | 2002 | Russia Longitudinal Monitoring Survey- Higher School of Economics Round XI | National | both | 5+ | 5+ | 3,819 | 4,951 | |
| 2556 | Russian Federation | 2002-2005 | Health, Alcohol and Psychosocial Factors In Eastern Europe | Community | urban | 45-70 | 45-70 | 4,240 | 5,069 | |
| 2557 | Russian Federation | 2003 | Russia Longitudinal Monitoring Survey- Higher School of Economics Round XII | National | both | 5+ | 5+ | 3,745 | 4,875 | |
| 2558 | Russian Federation | 2003 | School Children Moscow | Community | urban | 7-8 | 7-8 | 133 | 112 | |
| 2559 | Russian Federation | 2004 | Russia Longitudinal Monitoring Survey- Higher School of Economics Round XIII | National | both | 5+ | 5+ | 3,688 | 4,839 | |
| 2560 | Russian Federation | 2004 | School Children Moscow | Community | urban | 8-9 | 8-9 | 180 | 185 | |
| 2561 | Russian Federation | 2005 | Russia Longitudinal Monitoring Survey- Higher School of Economics Round XIV | National | both | 5+ | 5+ | 3,427 | 4,534 | |
| 2562 | Russian Federation | 2005 | School Children Moscow | Community | urban | 9-10 | 9-10 | 178 | 191 | |
| 2563 | Russian Federation | 2006 | School Children Moscow | Community | urban | 10-11 | 10-11 | 163 | 195 | |
| 2564 | Russian Federation | 2007 | Russian Karelia Survey in Pitkaranta | Community | both | 25-64 | 25-64 | 176 | 276 | |
| 2565 | Russian Federation | 2007 | School Children Moscow | Community | urban | 11-12 | 11-12 | 143 | 167 | |
| 2566 | Russian Federation | 2007-2010 | WHO Study on global AGEing and adult health (SAGE) | National | both | 50+ | 50+ | 1,195 | 2,148 | |
| 2567 | Russian Federation | 2008 | School Children Moscow | Community | urban | 12-13 | 12-13 | 111 | 141 | |
| 2568 | Russian Federation | 2009 | School Children Moscow | Community | urban | 13-14 | 13-14 | 124 | 140 | |
| 2569 | Russian Federation | 2010 | School Children Moscow | Community | urban | 14-15 | 14-15 | 116 | 137 | |
| 2570 | Russian Federation | 2011 | School Children Moscow | Community | urban | 15-16 | 15-16 | 117 | 125 | |
| 2571 | Russian Federation | 2012 | School Children Moscow | Community | urban | 16-17 | 16-17 | 87 | 108 | |
| 2572 | Russian Federation | 2012-2014 | Epidemiology of Cardiovascular Diseases in Different Regions of Russia (ESSE-RF) | National | both | 25-64 | 25-64 | 8,308 | 13,458 | |
| 2573 | Russian Federation | 2015-2016 | Childhood Obesity Surveillance Initiative 4 - Moscow | Community | urban | 6-8 | 6-8 | 1,499 | 1,529 | |
| 2574 | Russian Federation | 2015-2017 | Ural Eye and Medical Study (UEMS) | Subnational | rural | 40+ | 40+ | 1,530 | 1,870 | |
| 2575 | Russian Federation | 2015-2017 | Ural Eye and Medical Study (UEMS) | Community | urban | 40+ | 40+ | 1,050 | 1,449 | |
| 2576 | Russian Federation | 2017 | Epidemiology of Cardiovascular Diseases in Different Regions of Russia - 2 (ESSE-RF-2) | Subnational | both | 25-64 | 25-64 | 2,979 | 3,670 | |
| 2577 | Russian Federation | 2017-2020 | Ural Very Old Study | Community | both | 85+ | 85+ | 194 | 516 | |
| 2578 | Russian Federation | 2019-2020 | Childhood Obesity Surveillance Initiative 5 - Moscow | Community | urban | 6-7 | 6-7 | 1,201 | 1,202 | |
| 2579 | Russian Federation | 2019-2021 | Ural Children Eye Study | Community | urban | 6-18 | 6-18 | 1,950 | 2,052 | |
| 2580 | Russian Federation | 2020 | Childhood Obesity Surveillance Initiative 5 - Ekaterinburg | Community | urban | 7 | 7 | 1,310 | 1,376 | |
| 2581 | Russian Federation | 2021 | School Children Moscow | Community | urban | 7-18 | 7-18 | 1,591 | 1,435 | |
| 2582 | Rwanda | 2000 | DHS | National | both | | 15-49 | | 9,175 | |
| 2583 | Rwanda | 2005 | DHS | National | both | | 15-49 | | 5,211 | |
| 2584 | Rwanda | 2010 | DHS | National | both | 15-59 | 15-49 | 6,472 | 6,572 | |

| | Country | Study years | Survey/Study name/Citation | Level of representative-ness | Rural, urban, or both | Age range as in NCD-RisC database | | Sample size | | Notes |
|---|---|---|---|---|---|---|---|---|---|---|
| | | | | | | Male | Female | Male | Female | |
| 2585 | Rwanda | 2012 | STEPS | National | both | 15-64 | 15-64 | 2,644 | 4,243 | |
| 2586 | Rwanda | 2014-2015 | DHS | National | both | 15-59 | 15-49 | 6,366 | 6,313 | |
| 2587 | Rwanda | 2019-2020 | DHS | National | both | | 15-49 | | 6,885 | |
| 2588 | Rwanda | 2021-2022 | STEPS | National | both | 18-69 | 18-69 | 2,126 | 3,383 | |
| 2589 | Saint Kitts and Nevis | 2007 | STEPS | Subnational | both | 25-64 | 25-64 | 510 | 852 | |
| 2590 | Saint Kitts and Nevis | 2011 | Global School-based Student Health Survey | National | both | 13-17 | 13-17 | 650 | 814 | |
| 2591 | Saint Lucia | 1981 | Population Study of Blood Pressure and Associated Factors in St Lucia, West Indies | National | both | 15+ | 15+ | 168 | 191 | |
| 2592 | Saint Lucia | 1991-1994 | Cooper et al., Am J Public Health 87(2):160-68, 1997 | Community | urban | 25-100 | 25-100 | 491 | 593 | |
| 2593 | Saint Lucia | 2012 | STEPS | National | both | 25-64 | 25-64 | 665 | 1,097 | |
| 2594 | Saint Lucia | 2019-2020 | STEPS | National | both | 18-69 | 18-69 | 1,242 | 1,567 | |
| 2595 | Saint Vincent and the Grenadines | 2013-2014 | STEPS | National | both | 18-69 | 18-69 | 1,524 | 1,897 | |
| 2596 | Saint Vincent and the Grenadines | 2018 | Global School-based Student Health Survey | National | both | 13-17 | 13-17 | 737 | 839 | |
| 2597 | Samoa | 1979-1982 | McGarvey, Am J Clin Nutr 53(6 Suppl):1586S-1594S, 1991 | National | both | 5+ | 5+ | 469 | 501 | |
| 2598 | Samoa | 1991 | Non-Communicable Disease Risk Factor (NCDRF) | Subnational | rural | 25+ | 25+ | 465 | 496 | |
| 2599 | Samoa | 1991 | Non-Communicable Disease Risk Factor (NCDRF) | Subnational | urban | 25+ | 25+ | 330 | 444 | |
| 2600 | Samoa | 1991 | McGarvey, Pac Health Dialog 8(1):157-62, 2001 | National | both | 25+ | 25+ | 347 | 381 | |
| 2601 | Samoa | 1993 | McGarvey, Pac Health Dialog 8(1):157-62, 2001 | National | both | 27+ | 27+ | 285 | 336 | |
| 2602 | Samoa | 1995 | McGarvey, Pac Health Dialog 8(1):157-62, 2001 | National | both | 29+ | 29+ | 156 | 157 | |
| 2603 | Samoa | 2002 | STEPS | National | both | 25-64 | 25-64 | 1,181 | 1,334 | |
| 2604 | Samoa | 2010 | Samoan Genome-Wide Association Study | National | both | 24-65 | 24-65 | 1,402 | 2,061 | 23 |
| 2605 | Samoa | 2013 | STEPS | National | both | 18-64 | 18-64 | 605 | 918 | |
| 2606 | Sao Tome and Principe | 2008-2009 | DHS | National | both | 15-59 | 15-49 | 2,173 | 2,238 | |
| 2607 | Sao Tome and Principe | 2009 | STEPS | National | both | 25-64 | 25-64 | 998 | 1,286 | |
| 2608 | Sao Tome and Principe | 2019 | STEPS | National | both | 18-69 | 18-69 | 953 | 1,336 | |
| 2609 | Saudi Arabia | 1985-1988 | National Nutrition Survey | National | both | 5-75 | 5-75 | 2,311 | 3,057 | |
| 2610 | Saudi Arabia | 1989-1994 | National Nutrition Survey | National | both | 18-40 | 18-40 | 2,481 | 3,294 | |
| 2611 | Saudi Arabia | 1990-1993 | National Epidemiological Household Survey | National | both | 15-60 | 15-60 | 4,882 | 4,509 | |
| 2612 | Saudi Arabia | 1990-1993 | Saudi National Survey | National | both | 30-70 | 30-70 | 1,612 | 1,648 | |
| 2613 | Saudi Arabia | 1992-1995 | Saudi Health Information Survey | National | both | 14-50 | 14-50 | 4,830 | 7,707 | |
| 2614 | Saudi Arabia | 1995 | National Household Survey | National | both | 20-70 | 20-70 | 7,121 | 7,073 | |
| 2615 | Saudi Arabia | 1995-2000 | National Epidemiological Health Survey | National | both | 30-70 | 30-70 | 8,215 | 9,008 | |
| 2616 | Saudi Arabia | 2004-2005 | Al-Baghli et al., Saudi Med J 29(9):1319-25, 2008 | Subnational | both | 30+ | 30+ | 97,254 | 97,254 | |
| 2617 | Saudi Arabia | 2005 | El Mouzan et al., Ann Saudi Med 30(3):203-208, 2010 | National | both | 5-18 | 5-18 | 9,853 | 9,519 | |
| 2618 | Saudi Arabia | 2005 | STEPS | National | both | 15-64 | 15-64 | 2,245 | 2,345 | |
| 2619 | Saudi Arabia | 2007 | Gulf Cooperation Council World Health Survey | National | both | 18+ | 18+ | 4,854 | 3,610 | |
| 2620 | Saudi Arabia | 2009-2010 | Arab Teens Lifestyle Study (ATLS) | Subnational | urban | 14-19 | 14-19 | 1,384 | 1,479 | |
| 2621 | Saudi Arabia | 2011-2012 | Jeeluna Study- National Assessment of the Health Needs of Adolescents in Saudi Arabia | National | both | 12-19 | 12-19 | 6,234 | 5,790 | |
| 2622 | Saudi Arabia | 2011-2013 | Jeddah City Study | Community | urban | 5+ | 5+ | 957 | 867 | |
| 2623 | Saudi Arabia | 2013 | Saudi Health Information Survey | National | both | 15+ | 15+ | 5,088 | 5,249 | |
| 2624 | Senegal | 1984 | Maire et al., Rev Epidemiol Sante Publique 40:252-58, 1992 | National | rural | | 16-45 | | 1,628 | |
| 2625 | Senegal | 1986 | Astagneau et al., J Hypertens 10(9):1095-101, 1992 | Community | urban | 15+ | 15+ | 651 | 707 | |
| 2626 | Senegal | 1986 | Maire et al., Rev Epidemiol Sante Publique 40:252-58, 1992 | Community | urban | | 16-45 | | 616 | |
| 2627 | Senegal | 1992-1993 | DHS | National | both | | 20-49 | | 2,713 | |
| 2628 | Senegal | 2003 | Perceptions of healthy and desirable body size in urban Senegalese women | Community | urban | | 20-50 | | 287 | |
| 2629 | Senegal | 2005 | DHS | National | both | | 15-49 | | 4,166 | |
| 2630 | Senegal | 2010-2011 | DHS | National | both | 15-59 | 15-49 | 4,715 | 5,497 | |
| 2631 | Senegal | 2010-2012 | Biocultural determinants of overweight and obesity in the context of nutrition transition in Senegal: a holistic anthropological approach | Subnational | both | 18+ | 18+ | 280 | 307 | |
| 2632 | Senegal | 2015 | Les maladies chroniques au Sénégal: Une écologie de la santé comparative entre Dakar et Widou Thiengoly | Community | both | 20+ | 20+ | 734 | 765 | |
| 2633 | Senegal | 2015 | STEPS | National | both | 18-70 | 18-70 | 1,865 | 3,270 | |
| 2634 | Serbia | 1984 | MONICA, Novi Sad | Community | urban | 25-64 | 25-64 | 798 | 777 | |
| 2635 | Serbia | 1988-1989 | MONICA, Novi Sad | Community | urban | 25-64 | 25-64 | 778 | 791 | |
| 2636 | Serbia | 1990 | Longitudinal monitoring of growth and nutritional status of children in North Backa Region of Serbia | Community | both | 6-8 | 6-8 | 7 | 8 | |
| 2637 | Serbia | 1990-1991 | Longitudinal monitoring of growth and nutritional status of children in North Backa Region of Serbia | Community | both | 6-9 | 6-9 | 20 | 12 | |
| 2638 | Serbia | 1991-1992 | Longitudinal monitoring of growth and nutritional status of children in North Backa Region of Serbia | Community | both | 6-9 | 6-9 | 83 | 82 | |

| | Country | Study years | Survey/Study name/Citation | Level of representative-ness | Rural, urban, or both | Age range as in NCD-RisC database | | Sample size | | Notes |
|---|---|---|---|---|---|---|---|---|---|---|
| | | | | | | Male | Female | Male | Female | |
| 2639 | Serbia | 1992-1993 | Longitudinal monitoring of growth and nutritional status of children in North Backa Region of Serbia | Community | both | 6-7 | 6-7 | 99 | 113 | |
| 2640 | Serbia | 1993-1994 | Longitudinal monitoring of growth and nutritional status of children in North Backa Region of Serbia | Community | both | 6-11 | 6-11 | 111 | 96 | |
| 2641 | Serbia | 1994-1995 | MONICA, Novi Sad | Community | urban | 25-64 | 25-64 | 600 | 670 | |
| 2642 | Serbia | 1994-1995 | Longitudinal monitoring of growth and nutritional status of children in North Backa Region of Serbia | Community | both | 6-11 | 6-11 | 1,064 | 1,003 | |
| 2643 | Serbia | 1995-1996 | Longitudinal monitoring of growth and nutritional status of children in North Backa Region of Serbia | Community | both | 6-13 | 6-13 | 1,276 | 1,224 | |
| 2644 | Serbia | 1996-1997 | Longitudinal monitoring of growth and nutritional status of children in North Backa Region of Serbia | Community | both | 5-13 | 5-13 | 2,700 | 2,602 | |
| 2645 | Serbia | 1997-1998 | Longitudinal monitoring of growth and nutritional status of children in North Backa Region of Serbia | Community | both | 6-13 | 6-13 | 1,088 | 1,047 | |
| 2646 | Serbia | 1998 | Yugoslav study of precursors of atherosclerosis in schoolchildren | National | both | 9-10 | 9-10 | 266 | 216 | |
| 2647 | Serbia | 1998-1999 | Longitudinal monitoring of growth and nutritional status of children in North Backa Region of Serbia | Community | both | 6-16 | 6-16 | 944 | 910 | |
| 2648 | Serbia | 1999-2000 | Longitudinal monitoring of growth and nutritional status of children in North Backa Region of Serbia | Community | both | 6-12 | 6-12 | 567 | 521 | |
| 2649 | Serbia | 2000 | Health Status, Health Needs and Utilization of Health Care of the Population of Serbia | National | both | 7+ | 7+ | 5,079 | 6,189 | |
| 2650 | Serbia | 2000-2001 | Longitudinal monitoring of growth and nutritional status of children in North Backa Region of Serbia | Community | both | 9-18 | 9-18 | 2,386 | 2,285 | |
| 2651 | Serbia | 2001-2002 | Longitudinal monitoring of growth and nutritional status of children in North Backa Region of Serbia | Community | both | 9-18 | 9-18 | 2,042 | 2,105 | |
| 2652 | Serbia | 2002-2003 | Longitudinal monitoring of growth and nutritional status of children in North Backa Region of Serbia | Community | both | 5-18 | 5-18 | 3,040 | 2,516 | |
| 2653 | Serbia | 2003 | Yugoslav study of precursors of atherosclerosis in schoolchildren | National | both | 14-15 | 14-15 | 157 | 136 | |
| 2654 | Serbia | 2003-2004 | Longitudinal monitoring of growth and nutritional status of children in North Backa Region of Serbia | Community | both | 5-18 | 5-18 | 3,242 | 2,915 | |
| 2655 | Serbia | 2004-2005 | Longitudinal monitoring of growth and nutritional status of children in North Backa Region of Serbia | Community | both | 5-18 | 5-18 | 3,592 | 3,324 | |
| 2656 | Serbia | 2005 | Longitudinal monitoring of growth and nutritional status of children in North Backa Region of Serbia | Community | both | 8-13 | 8-13 | 666 | 652 | |
| 2657 | Serbia | 2006 | The 2006 National Health Survey for the Population of Serbia | National | both | 7+ | 7+ | 7,888 | 8,558 | |
| 2658 | Serbia | 2013 | The 2013 National Health Survey for the Population of Serbia | National | both | 7+ | 7+ | 7,205 | 8,137 | |
| 2659 | Serbia | 2013-2014 | Stay Fit for Lifelong Health; the Prevalence of Lifestyle Health Conditions in Serbian Population | National | urban | 18-65 | 18-65 | 1,337 | 297 | |
| 2660 | Serbia | 2015-2016 | Childhood Obesity Surveillance Initiative 4 | National | both | 6-8 | 6-8 | 2,475 | 2,386 | |
| 2661 | Serbia | 2019 | Childhood Obesity Surveillance Initiative 5 | National | both | 7-9 | 7-9 | 1,697 | 1,504 | |
| 2662 | Seychelles | 1989 | Seychelles Heart Survey I | National | both | 25-64 | 25-64 | 513 | 568 | |
| 2663 | Seychelles | 1994 | Seychelles Heart Survey II | National | both | 25-64 | 25-64 | 499 | 563 | |
| 2664 | Seychelles | 1998 | School Screening Program | National | both | 5-16 | 5-16 | 1,521 | 1,391 | |
| 2665 | Seychelles | 1999 | School Screening Program | National | both | 5-16 | 5-16 | 2,638 | 2,764 | |
| 2666 | Seychelles | 2000 | School Screening Program | National | both | 5-16 | 5-16 | 1,767 | 1,820 | |
| 2667 | Seychelles | 2001 | School Screening Program | National | both | 5-16 | 5-16 | 2,558 | 2,551 | |
| 2668 | Seychelles | 2002 | School Screening Program | National | both | 5-16 | 5-16 | 2,445 | 2,502 | |
| 2669 | Seychelles | 2003 | School Screening Program | National | both | 5-20 | 5-20 | 3,383 | 3,461 | |
| 2670 | Seychelles | 2004 | Seychelles Heart Survey III | National | both | 25-64 | 25-64 | 568 | 687 | |
| 2671 | Seychelles | 2004 | School Screening Program | National | both | 5-16 | 5-16 | 2,339 | 2,298 | |
| 2672 | Seychelles | 2005 | School Screening Program | National | both | 5-15 | 5-15 | 2,678 | 2,733 | |
| 2673 | Seychelles | 2006 | School Screening Program | National | both | 5-15 | 5-15 | 2,657 | 2,600 | |
| 2674 | Seychelles | 2007 | Global School-based Student Health Survey | National | both | 13-17 | 13-17 | 385 | 467 | |
| 2675 | Seychelles | 2011 | School Screening Program | National | both | 5-15 | 5-15 | 2,233 | 2,205 | |
| 2676 | Seychelles | 2012 | School Screening Program | National | both | 5-15 | 5-15 | 2,261 | 2,290 | |
| 2677 | Seychelles | 2013 | School Screening Program | National | both | 5-15 | 5-15 | 1,921 | 2,069 | |
| 2678 | Seychelles | 2013-2014 | Seychelles Heart Survey IV | National | both | 25-64 | 25-64 | 531 | 699 | |
| 2679 | Seychelles | 2014 | School Screening Program | National | both | 5-15 | 5-15 | 2,083 | 2,170 | |
| 2680 | Seychelles | 2015 | Global School-based Student Health Survey | National | both | 13-17 | 13-17 | 770 | 888 | |
| 2681 | Seychelles | 2015 | School Screening Program | National | both | 5-15 | 5-15 | 1,963 | 1,984 | |
| 2682 | Seychelles | 2016 | School Screening Program | National | both | 5-15 | 5-15 | 1,737 | 1,858 | |
| 2683 | Seychelles | 2017 | School Screening Program | National | both | 5-15 | 5-15 | 1,497 | 1,570 | |
| 2684 | Seychelles | 2018 | School Screening Program | National | both | 8-15 | 8-15 | 1,712 | 1,669 | |
| 2685 | Seychelles | 2019 | School Screening Program | National | both | 8-15 | 8-15 | 1,606 | 1,665 | |
| 2686 | Seychelles | 2020 | School Screening Program | National | both | 8-15 | 8-15 | 1,349 | 1,467 | |
| 2687 | Seychelles | 2022 | School Screening Program | National | both | 8-15 | 8-15 | 1,465 | 1,480 | |
| 2688 | Sierra Leone | 2008 | DHS | National | both | | 15-49 | | 3,274 | |
| 2689 | Sierra Leone | 2009 | STEPS | National | both | 25-64 | 25-64 | 2,200 | 2,319 | |
| 2690 | Sierra Leone | 2013 | DHS | National | both | 15-59 | 15-49 | 7,037 | 7,459 | |
| 2691 | Sierra Leone | 2019 | DHS | National | both | 15-59 | 15-49 | 6,440 | 7,046 | |
| 2692 | Singapore | 1982-1985 | Thyroid Heart Study | National | both | 18+ | 18+ | 1,030 | 990 | |
| 2693 | Singapore | 1992 | National Health Survey 1992 | National | both | 18-64 | 18-64 | 1,743 | 1,704 | |
| 2694 | Singapore | 1993-1995 | NUH Heart Study | National | both | 26-89 | 26-89 | 498 | 484 | |
| 2695 | Singapore | 1998 | National Health Survey 1998 | National | both | 18-69 | 18-69 | 2,283 | 2,264 | |

| | Country | Study years | Survey/Study name/Citation | Level of representative-ness | Rural, urban, or both | Age range as in NCD-RisC database | | Sample size | | Notes |
|---|---|---|---|---|---|---|---|---|---|---|
| | | | | | | Male | Female | Male | Female | |
| 2696 | Singapore | 2003-2005 | Singapore Longitudinal Ageing Study - Cohort 1; SLAS-1 | Community | both | 55+ | 55+ | 1,029 | 1,759 | |
| 2697 | Singapore | 2004 | National Health Survey 2004 | National | both | 18-74 | 18-74 | 2,059 | 2,094 | |
| 2698 | Singapore | 2004-2007 | Singapore Cardiovascular Cohort Study and Singapore Prospective Study Program | National | both | 24+ | 24+ | 2,471 | 2,686 | |
| 2699 | Singapore | 2008-2013 | Singapore Longitudinal Ageing Study - Cohort 2; SLAS-2 | Community | both | 55+ | 55+ | 1,138 | 1,883 | |
| 2700 | Singapore | 2009 | Social Isolation, Health and Lifestyles Survey (SIHLS) 2009 | National | both | 60+ | 60+ | 2,038 | 2,382 | |
| 2701 | Singapore | 2009-2011 | The Singapore Chinese Eye Study | Community | both | 40-80 | 40-80 | 1,652 | 1,679 | |
| 2702 | Singapore | 2012-2013 | Singapore Health Study | National | both | 18-79 | 18-79 | 954 | 1,021 | 24 |
| 2703 | Singapore | 2014-2015 | Singapore Health 2 | National | both | 18-79 | 18-79 | 775 | 973 | 24 |
| 2704 | Singapore | 2015-2017 | The Singapore Chinese Eye Study Follow-Up | Community | both | 50+ | 50+ | 1,278 | 1,348 | |
| 2705 | Singapore | 2016-2017 | Transitions in Health, Employment, Social Engagement and Inter-generational Transfers in Singapore Study | National | both | 60+ | 60+ | 1,723 | 2,131 | |
| 2706 | Slovakia | 1985 | Effects of somatic development and environmental factors on blood pressure in children | Community | urban | 5-7 | 5-7 | 412 | 388 | |
| 2707 | Slovakia | 1993 | Countrywide Integrated Noncommunicable Diseases Intervention Programme | National | both | 15-64 | 15-64 | 876 | 1,293 | |
| 2708 | Slovakia | 1998 | Countrywide Integrated Noncommunicable Diseases Intervention Programme | National | both | 15-64 | 15-64 | 923 | 1,122 | |
| 2709 | Slovakia | 2001 | National Anthropological Survey | National | both | 5-6 | 5-6 | 896 | 815 | |
| 2710 | Slovakia | 2001 | National Anthropological Survey | National | both | 6-18 | 6-18 | 10,881 | 10,667 | |
| 2711 | Slovakia | 2003 | Countrywide Integrated Noncommunicable Diseases Intervention Programme | National | both | 15-64 | 15-64 | 664 | 905 | |
| 2712 | Slovakia | 2008 | Countrywide Integrated Noncommunicable Diseases Intervention Programme | National | both | 15-64 | 15-64 | 412 | 584 | |
| 2713 | Slovakia | 2011 | National Anthropological Survey | National | both | 7-18 | 7-18 | 9,064 | 9,028 | |
| 2714 | Slovakia | 2011-2012 | European Health Examination Survey | National | both | 18-64 | 18-64 | 884 | 1,080 | |
| 2715 | Slovakia | 2015-2016 | Childhood Obesity Surveillance Initiative 4 | National | both | 7 | 7 | 1,390 | 1,379 | |
| 2716 | Slovakia | 2018 | Childhood Obesity Surveillance Initiative 5 | National | both | 7-8 | 7-8 | 2,975 | 3,004 | |
| 2717 | Slovenia | 1982 | The SLOFIT monitoring system | National | both | 6-19 | 6-19 | 16,381 | 17,183 | |
| 2718 | Slovenia | 1983 | Analysis of Children's Development in Slovenia (ACDSi) | National | both | 7-14 | 7-14 | 1,580 | 1,576 | |
| 2719 | Slovenia | 1983 | The SLOFIT monitoring system | National | both | 6-19 | 6-19 | 16,365 | 17,435 | |
| 2720 | Slovenia | 1984 | The SLOFIT monitoring system | National | both | 6-19 | 6-19 | 21,438 | 22,933 | |
| 2721 | Slovenia | 1985 | The SLOFIT monitoring system | National | both | 6-19 | 6-19 | 22,496 | 23,043 | |
| 2722 | Slovenia | 1986 | The SLOFIT monitoring system | National | both | 6-19 | 6-19 | 22,891 | 23,009 | |
| 2723 | Slovenia | 1987 | The SLOFIT monitoring system | National | both | 6-19 | 6-19 | 49,089 | 48,784 | |
| 2724 | Slovenia | 1988 | The SLOFIT monitoring system | National | both | 6-19 | 6-19 | 85,444 | 83,390 | |
| 2725 | Slovenia | 1989 | The SLOFIT monitoring system | National | both | 6-19 | 6-19 | 106,538 | 106,639 | |
| 2726 | Slovenia | 1990 | The SLOFIT monitoring system | National | both | 6-19 | 6-19 | 129,317 | 128,572 | |
| 2727 | Slovenia | 1991 | The SLOFIT monitoring system | National | both | 6-19 | 6-19 | 130,726 | 129,842 | |
| 2728 | Slovenia | 1992 | The SLOFIT monitoring system | National | both | 6-19 | 6-19 | 135,239 | 134,853 | |
| 2729 | Slovenia | 1993 | Analysis of Children's Development in Slovenia (ACDSi) | National | both | 6-14 | 6-14 | 1,674 | 1,678 | |
| 2730 | Slovenia | 1993 | The SLOFIT monitoring system | National | both | 6-19 | 6-19 | 143,182 | 141,966 | |
| 2731 | Slovenia | 1994 | Analysis of Children's Development in Slovenia (ACDSi) | National | both | 14-18 | 14-18 | 683 | 696 | |
| 2732 | Slovenia | 1994 | The SLOFIT monitoring system | National | both | 6-19 | 6-19 | 145,348 | 143,290 | |
| 2733 | Slovenia | 1995 | The SLOFIT monitoring system | National | both | 6-19 | 6-19 | 142,245 | 140,692 | |
| 2734 | Slovenia | 1996 | The SLOFIT monitoring system | National | both | 6-19 | 6-19 | 140,714 | 138,120 | |
| 2735 | Slovenia | 1997 | The SLOFIT monitoring system | National | both | 6-19 | 6-19 | 127,551 | 120,331 | |
| 2736 | Slovenia | 1998 | The SLOFIT monitoring system | National | both | 6-19 | 6-19 | 126,342 | 121,549 | |
| 2737 | Slovenia | 1999 | The SLOFIT monitoring system | National | both | 6-19 | 6-19 | 122,655 | 119,408 | |
| 2738 | Slovenia | 2000 | The SLOFIT monitoring system | National | both | 6-19 | 6-19 | 121,918 | 116,281 | |
| 2739 | Slovenia | 2001 | National Programme of Primary CVD Prevention in Slovenia | National | both | 35-65 | 45-70 | 1,079 | 953 | |
| 2740 | Slovenia | 2001 | The SLOFIT monitoring system | National | both | 6-19 | 6-19 | 118,997 | 114,606 | |
| 2741 | Slovenia | 2002 | National Programme of Primary CVD Prevention in Slovenia | National | both | 35-65 | 45-70 | 34,539 | 34,384 | |
| 2742 | Slovenia | 2002 | The SLOFIT monitoring system | National | both | 6-19 | 6-19 | 114,716 | 110,691 | |
| 2743 | Slovenia | 2003 | Analysis of Children's Development in Slovenia (ACDSi) | National | both | 5-14 | 5-14 | 2,061 | 1,939 | |
| 2744 | Slovenia | 2003 | National Programme of Primary CVD Prevention in Slovenia | National | both | 35-65 | 45-70 | 32,472 | 34,448 | |
| 2745 | Slovenia | 2003 | The SLOFIT monitoring system | National | both | 6-19 | 6-19 | 117,278 | 112,944 | |
| 2746 | Slovenia | 2004 | Analysis of Children's Development in Slovenia (ACDSi) | National | both | 14-19 | 14-19 | 950 | 712 | |
| 2747 | Slovenia | 2004 | National Programme of Primary CVD Prevention in Slovenia | National | both | 35-65 | 45-70 | 33,872 | 35,277 | |
| 2748 | Slovenia | 2004 | The SLOFIT monitoring system | National | both | 6-19 | 6-19 | 117,768 | 112,575 | |
| 2749 | Slovenia | 2005 | National Programme of Primary CVD Prevention in Slovenia | National | both | 35-65 | 45-70 | 27,146 | 26,180 | |
| 2750 | Slovenia | 2005 | The SLOFIT monitoring system | National | both | 6-19 | 6-19 | 114,966 | 109,542 | |
| 2751 | Slovenia | 2006 | National Programme of Primary CVD Prevention in Slovenia | National | both | 35-65 | 45-70 | 23,887 | 21,665 | |
| 2752 | Slovenia | 2006 | The SLOFIT monitoring system | National | both | 6-19 | 6-19 | 109,877 | 102,838 | |

| | Country | Study years | Survey/Study name/Citation | Level of representative-ness | Rural, urban, or both | Age range as in NCD-RisC database | | Sample size | | Notes |
|---|---|---|---|---|---|---|---|---|---|---|
| | | | | | | Male | Female | Male | Female | |
| 2753 | Slovenia | 2007 | National Programme of Primary CVD Prevention in Slovenia | National | both | 35-65 | 45-70 | 17,832 | 15,634 | |
| 2754 | Slovenia | 2007 | The SLOFIT monitoring system | National | both | 6-19 | 6-19 | 103,105 | 100,364 | |
| 2755 | Slovenia | 2008 | National Programme of Primary CVD Prevention in Slovenia | National | both | 35-65 | 45-70 | 14,764 | 13,726 | |
| 2756 | Slovenia | 2008 | The SLOFIT monitoring system | National | both | 6-19 | 6-19 | 103,735 | 98,070 | |
| 2757 | Slovenia | 2009 | National Programme of Primary CVD Prevention in Slovenia | National | both | 35-65 | 45-70 | 13,172 | 11,454 | |
| 2758 | Slovenia | 2009 | The SLOFIT monitoring system | National | both | 6-19 | 6-19 | 103,669 | 98,891 | |
| 2759 | Slovenia | 2010 | EuropeaN Energy balance Research to prevent excessive weight Gain among Youth - The ENERGY-project | National | both | 10-12 | 10-12 | 548 | 579 | |
| 2760 | Slovenia | 2010 | National Programme of Primary CVD Prevention in Slovenia | National | both | 35-65 | 45-70 | 13,752 | 12,605 | |
| 2761 | Slovenia | 2010 | The SLOFIT monitoring system | National | both | 6-19 | 6-19 | 101,163 | 96,686 | |
| 2762 | Slovenia | 2011 | National Programme of Primary CVD Prevention in Slovenia | National | both | 35-65 | 45-70 | 12,835 | 11,953 | |
| 2763 | Slovenia | 2011 | The SLOFIT monitoring system | National | both | 6-19 | 6-19 | 100,802 | 96,309 | |
| 2764 | Slovenia | 2012 | National Programme of Primary CVD Prevention in Slovenia | National | both | 35-65 | 45-70 | 13,881 | 14,178 | |
| 2765 | Slovenia | 2012 | The SLOFIT monitoring system | National | both | 6-19 | 6-19 | 101,172 | 95,917 | |
| 2766 | Slovenia | 2013 | National Programme of Primary CVD Prevention in Slovenia | National | both | 35-65 | 45-70 | 10,590 | 12,118 | |
| 2767 | Slovenia | 2013 | The SLOFIT monitoring system | National | both | 6-19 | 6-19 | 99,191 | 95,666 | |
| 2768 | Slovenia | 2013-2014 | Analysis of Children's Development in Slovenia (ACDSi) | National | both | 6-15 | 6-15 | 1,665 | 1,627 | |
| 2769 | Slovenia | 2014 | Analysis of Children's Development in Slovenia (ACDSi) | National | both | 14-19 | 14-19 | 703 | 724 | |
| 2770 | Slovenia | 2014 | National Programme of Primary CVD Prevention in Slovenia | National | both | 35-65 | 45-70 | 10,448 | 12,467 | |
| 2771 | Slovenia | 2014 | The SLOFIT monitoring system | National | both | 6-21 | 6-21 | 102,790 | 97,859 | |
| 2772 | Slovenia | 2015 | National Programme of Primary CVD Prevention in Slovenia | National | both | 35-65 | 45-70 | 6,877 | 7,387 | |
| 2773 | Slovenia | 2015 | The SLOFIT monitoring system | National | both | 6-19 | 6-19 | 103,568 | 99,293 | |
| 2774 | Slovenia | 2016 | The SLOFIT monitoring system | National | both | 6-19 | 6-19 | 107,421 | 102,174 | |
| 2775 | Slovenia | 2017 | The SLOFIT monitoring system | National | both | 6-19 | 6-19 | 108,780 | 104,582 | |
| 2776 | Slovenia | 2018 | The SLOFIT monitoring system | National | both | 6-19 | 6-19 | 110,213 | 105,861 | |
| 2777 | Slovenia | 2019 | The SLOFIT monitoring system | National | both | 6-19 | 6-19 | 99,445 | 94,791 | |
| 2778 | Slovenia | 2020 | The SLOfit monitoring system | Community | both | 15-19 | 15-19 | 879 | 816 | |
| 2779 | Slovenia | 2020 | The SLOfit monitoring system | National | both | 6-14 | 6-14 | 34,382 | 32,921 | |
| 2780 | Slovenia | 2021 | The SLOfit monitoring system | Community | both | 15-19 | 15-19 | 6,724 | 5,950 | |
| 2781 | Slovenia | 2021 | The SLOfit monitoring system | National | both | 6-14 | 6-14 | 82,486 | 78,808 | |
| 2782 | Slovenia | 2022 | The SLOfit monitoring system | National | both | 6-19 | 6-19 | 100,788 | 98,820 | |
| 2783 | Slovenia | 2023 | The SLOfit monitoring system | National | both | 6-19 | 6-19 | 102,628 | 97,936 | 5 |
| 2784 | Solomon Islands | 2004 | A genetic-ecological study of the risk factors for lifestyle-related diseases in Oceanian populations | Community | rural | 18+ | 18+ | 106 | 109 | |
| 2785 | Solomon Islands | 2004 | A genetic-ecological study of the risk factors for lifestyle-related diseases in Oceanian populations | Community | urban | 18+ | 18+ | 91 | 95 | |
| 2786 | Solomon Islands | 2006 | STEPS | Subnational | both | 15-64 | 15-64 | 1,031 | 1,375 | |
| 2787 | Solomon Islands | 2009-2010 | Furusawa et al., N Z Med J 124(1333):17-28, 2011 | Subnational | rural | 5+ | 5+ | 256 | 317 | |
| 2788 | Solomon Islands | 2009-2010 | Furusawa et al., N Z Med J 124(1333):17-28, 2011 | Subnational | urban | 5-70 | 5-70 | 78 | 118 | |
| 2789 | Solomon Islands | 2015 | STEPS | National | both | 18-69 | 18-69 | 816 | 978 | |
| 2790 | Solomon Islands | 2017-2018 | Impact of sea-level rise and relocation projects on health, ecology, and society in Oceania | Community | both | 5+ | 5+ | 147 | 321 | |
| 2791 | Somalia | 2016 | The prevalence of selected risk factors for non-communicable diseases in Hargeisa, Somaliland: a cross-sectional study | Community | urban | 20-69 | 20-69 | 145 | 955 | |
| 2792 | South Africa | 1989 | Temple et al., Ethn Dis 11(3):431-7, 2001 | Community | both | 15+ | 15+ | 457 | 614 | |
| 2793 | South Africa | 1990 | Steyn et al., East Afr Med J 75(1):35-40, 1998 | Community | urban | 15-64 | 15-64 | 292 | 373 | |
| 2794 | South Africa | 1996 | Temple et al., Ethn Dis 11(3):431-7, 2001 | Community | both | 15+ | 15+ | 302 | 406 | |
| 2795 | South Africa | 1996 | Ellisras Longitudinal Study | Community | rural | 5-10 | 5-10 | 583 | 535 | |
| 2796 | South Africa | 1997 | Ellisras Longitudinal Study | Community | rural | 5-11 | 5-11 | 1,046 | 968 | |
| 2797 | South Africa | 1998 | DHS | National | both | 15+ | 15+ | 5,645 | 7,757 | |
| 2798 | South Africa | 1998 | Ellisras Longitudinal Study | Community | rural | 5-12 | 5-12 | 958 | 856 | |
| 2799 | South Africa | 1999 | Ellisras Longitudinal Study | Community | rural | 5-13 | 5-13 | 991 | 917 | |
| 2800 | South Africa | 2000 | Ellisras Longitudinal Study | Community | rural | 5-14 | 5-14 | 936 | 877 | |
| 2801 | South Africa | 2000-2001 | Transition and Health during Urbanisation of South Africans: Children | Subnational | both | 9-15 | 9-15 | 606 | 639 | |
| 2802 | South Africa | 2001 | Ellisras Longitudinal Study | Community | rural | 6-15 | 6-15 | 962 | 904 | |
| 2803 | South Africa | 2002 | Ellisras Longitudinal Study | Community | rural | 7-16 | 7-16 | 890 | 823 | |
| 2804 | South Africa | 2002 | The 1st South African National Youth Risk Behaviour Survey | National | both | 14-18 | 14-18 | 3,609 | 4,139 | |
| 2805 | South Africa | 2002-2003 | SASPI | Community | rural | 35+ | 35+ | 80 | 275 | |
| 2806 | South Africa | 2003 | DHS | National | both | 15+ | 15+ | 3,200 | 4,497 | |
| 2807 | South Africa | 2003 | Ellisras Longitudinal Study | Community | rural | 8-17 | 8-17 | 911 | 858 | |
| 2808 | South Africa | 2003-2004 | Africa Centre Biomeasure Survey | Community | rural | 25-49 | 25-49 | 778 | 1,693 | |

| | Country | Study years | Survey/Study name/Citation | Level of representative-ness | Rural, urban, or both | Age range as in NCD-RisC database | | Sample size | | Notes |
|---|---|---|---|---|---|---|---|---|---|---|
| | | | | | | Male | Female | Male | Female | |
| 2809 | South Africa | 2004-2006 | Li et al., Curationis 30(4):79-87, 2007 | Community | both | 18-40 | 18-40 | 334 | 270 | |
| 2810 | South Africa | 2007-2008 | WHO Study on global AGEing and adult health (SAGE) | National | both | 50+ | 50+ | 1,541 | 2,059 | |
| 2811 | South Africa | 2007-2008 | Cardiometabolic risk profile of South African Learners | Subnational | both | 10-16 | 10-16 | 496 | 776 | |
| 2812 | South Africa | 2008 | National Income Dynamics Study Wave I | National | both | 5+ | 5+ | 8,131 | 10,624 | |
| 2813 | South Africa | 2008 | The 2nd South African National Youth Risk Behaviour Survey | National | both | 14-18 | 14-18 | 3,910 | 4,201 | |
| 2814 | South Africa | 2008-2009 | Cape Town Bellville South Cohort Study - Baseline evaluation I | Community | urban | 35-65 | 35-65 | 142 | 499 | |
| 2815 | South Africa | 2010 | Africa Centre Biomeasure Survey | Community | rural | 15+ | 15+ | 2,933 | 6,364 | |
| 2816 | South Africa | 2010-2011 | National Income Dynamics Study Wave II | National | both | 5+ | 5+ | 8,383 | 10,996 | |
| 2817 | South Africa | 2011-2012 | South Africa National Health and Nutrition Examination Survey | National | both | 5+ | 5+ | 3,717 | 5,640 | |
| 2818 | South Africa | 2011-2013 | International Study of Childhood Obesity, Lifestyle and the Environment (ISCOLE) | Community | urban | 9-11 | 9-11 | 222 | 327 | |
| 2819 | South Africa | 2012 | National Income Dynamics Study Wave III | National | both | 5+ | 5+ | 10,956 | 14,118 | |
| 2820 | South Africa | 2014-2015 | Health and Aging in Africa: A Longitudinal Study of an INDEPTH Community in South Africa (HAALSI) | Community | rural | 40+ | 40+ | 2,141 | 2,502 | |
| 2821 | South Africa | 2014-2015 | National Income Dynamics Study Wave IV | National | both | 5+ | 5+ | 13,548 | 16,775 | |
| 2822 | South Africa | 2016 | DHS | National | both | 15-59 | 15-49 | 2,807 | 3,263 | |
| 2823 | South Africa | 2017 | National Income Dynamics Study Wave V | National | both | 5+ | 5+ | 13,996 | 17,688 | |
| 2824 | South Africa | 2017-2019 | The Exercise, Arterial Modulation and Nutrition in Youth South Africa (ExAMIN Youth SA) Study | Subnational | urban | 5-9 | 5-9 | 483 | 575 | |
| 2825 | South Africa | 2018-2020 | Vukuzazi Study | Community | both | 15+ | 15+ | 5,735 | 11,836 | |
| 2826 | South Korea | 1986 | INTERSALT | Community | urban | 20-59 | 20-59 | 100 | 98 | |
| 2827 | South Korea | 1990 | Korean National Blood Pressure Survey | National | both | 30+ | 30+ | 9,734 | 12,619 | |
| 2828 | South Korea | 1992-1993 | Park et al., Diabetes Res Clin Pract 34 Suppl:S65-72, 1996 | Subnational | both | 30-89 | 30+ | 1,077 | 1,392 | |
| 2829 | South Korea | 1997-1998 | National Anthropometric Survey in Korean Children and Adolescents | National | both | 5-19 | 5-19 | 29,318 | 26,469 | |
| 2830 | South Korea | 1998 | Korea National Health and Nutrition Examination Survey | National | both | 10+ | 10+ | 4,514 | 5,193 | |
| 2831 | South Korea | 1999 | The South Korean Consciption Database | National | both | 19 | | 401,721 | | |
| 2832 | South Korea | 2000 | The South Korean Consciption Database | National | both | 19 | | 402,758 | | |
| 2833 | South Korea | 2001 | Kim et al., Br J Psychiatry 185:102-7, 2004 | Community | both | 65+ | 65+ | 300 | 432 | |
| 2834 | South Korea | 2001 | The South Korean Consciption Database | National | both | 19 | | 398,653 | | |
| 2835 | South Korea | 2001 | Korea National Health and Nutrition Examination Survey | National | both | 5+ | 5+ | 4,150 | 4,815 | |
| 2836 | South Korea | 2002 | The South Korean Consciption Database | National | both | 19 | | 367,024 | | |
| 2837 | South Korea | 2002-2003 | Korean National Health Insurance | National | both | 40+ | 40+ | 2,993,634 | 2,483,306 | |
| 2838 | South Korea | 2003 | The South Korean Consciption Database | National | both | 19 | | 329,626 | | |
| 2839 | South Korea | 2004 | The South Korean Consciption Database | National | both | 19 | | 323,001 | | |
| 2840 | South Korea | 2004-2005 | Korean National Health Insurance | National | both | 40+ | 40+ | 3,604,097 | 3,261,164 | |
| 2841 | South Korea | 2005 | The South Korean Consciption Database | National | both | 19 | | 313,378 | | |
| 2842 | South Korea | 2005 | Korea National Health and Nutrition Examination Survey | National | both | 5+ | 5+ | 3,183 | 3,896 | |
| 2843 | South Korea | 2005 | National Anthropometric Survey in Korean Children and Adolescents | National | both | 5-19 | 5-19 | 41,727 | 39,200 | |
| 2844 | South Korea | 2006 | The South Korean Consciption Database | National | both | 19 | | 302,587 | | |
| 2845 | South Korea | 2006-2007 | Korean National Health Insurance | National | both | 40+ | 40+ | 4,569,655 | 4,613,826 | |
| 2846 | South Korea | 2007 | The South Korean Consciption Database | National | both | 19 | | 312,795 | | |
| 2847 | South Korea | 2007 | Korea National Health and Nutrition Examination Survey | National | both | 5+ | 5+ | 1,753 | 2,174 | |
| 2848 | South Korea | 2007-2012 | JS High-School Study | Community | rural | 14-17 | 14-17 | 553 | 508 | |
| 2849 | South Korea | 2008 | The South Korean Consciption Database | National | both | 19 | | 312,919 | | |
| 2850 | South Korea | 2008 | Korea National Health and Nutrition Examination Survey | National | both | 5+ | 5+ | 3,849 | 4,824 | |
| 2851 | South Korea | 2008-2009 | Korean National Health Insurance | National | both | 40+ | 40+ | 5,763,909 | 6,089,441 | |
| 2852 | South Korea | 2009 | The South Korean Consciption Database | National | both | 19 | | 324,818 | | |
| 2853 | South Korea | 2009 | Korea National Health and Nutrition Examination Survey | National | both | 5+ | 5+ | 4,288 | 5,182 | |
| 2854 | South Korea | 2009 | Korea National School Health Examination Survey (KNSHES) | National | both | 6-18 | 6-18 | 103,997 | 89,881 | |
| 2855 | South Korea | 2010 | The South Korean Consciption Database | National | both | 19 | | 347,249 | | |
| 2856 | South Korea | 2010 | Korea National Health and Nutrition Examination Survey | National | both | 5+ | 5+ | 3,583 | 4,312 | |
| 2857 | South Korea | 2010 | Korea National School Health Examination Survey (KNSHES) | National | both | 6-18 | 6-18 | 99,865 | 86,167 | |
| 2858 | South Korea | 2010-2011 | Korean National Health Insurance | National | both | 40+ | 40+ | 6,671,572 | 7,127,111 | |
| 2859 | South Korea | 2011 | The South Korean Consciption Database | National | both | 19 | | 364,982 | | |
| 2860 | South Korea | 2011 | Korea National Health and Nutrition Examination Survey | National | both | 5+ | 5+ | 3,363 | 4,193 | |
| 2861 | South Korea | 2011 | Korea National School Health Examination Survey (KNSHES) | National | both | 6-18 | 6-18 | 97,363 | 83,046 | |
| 2862 | South Korea | 2012 | The South Korean Consciption Database | National | both | 19 | | 361,009 | | |
| 2863 | South Korea | 2012 | Korea National Health and Nutrition Examination Survey | National | both | 5+ | 5+ | 3,194 | 4,029 | |
| 2864 | South Korea | 2012 | Korea National School Health Examination Survey (KNSHES) | National | both | 6-18 | 6-18 | 45,066 | 42,005 | |
| 2865 | South Korea | 2012-2013 | Korean National Health Insurance | National | both | 40+ | 40+ | 7,256,898 | 7,782,621 | |

| | Country | Study years | Survey/Study name/Citation | Level of representative-ness | Rural, urban, or both | Age range as in NCD-RisC database | | Sample size | | Notes |
|---|---|---|---|---|---|---|---|---|---|---|
| | | | | | | Male | Female | Male | Female | |
| 2866 | South Korea | 2013 | The South Korean Consciption Database | National | both | 19 | | 363,914 | | |
| 2867 | South Korea | 2013 | Korea National Health and Nutrition Examination Survey | National | both | 5+ | 5+ | 3,211 | 3,934 | |
| 2868 | South Korea | 2013 | Korea National School Health Examination Survey (KNSHES) | National | both | 6-18 | 6-18 | 43,667 | 40,776 | |
| 2869 | South Korea | 2014 | The South Korean Consciption Database | National | both | 19 | | 363,597 | | |
| 2870 | South Korea | 2014 | Korea National Health and Nutrition Examination Survey | National | both | 5+ | 5+ | 2,966 | 3,769 | |
| 2871 | South Korea | 2014 | Korea National School Health Examination Survey (KNSHES) | National | both | 6-18 | 6-18 | 42,570 | 39,987 | |
| 2872 | South Korea | 2014-2015 | Korean National Health Insurance | National | both | 40+ | 40+ | 7,869,485 | 8,354,998 | |
| 2873 | South Korea | 2015 | The South Korean Consciption Database | National | both | 19 | | 350,518 | | |
| 2874 | South Korea | 2015 | Korea National Health and Nutrition Examination Survey | National | both | 5+ | 5+ | 3,022 | 3,640 | |
| 2875 | South Korea | 2015 | Korea National School Health Examination Survey (KNSHES) | National | both | 6-18 | 6-18 | 43,141 | 41,645 | |
| 2876 | South Korea | 2016 | The South Korean Consciption Database | National | both | 19 | | 339,410 | | |
| 2877 | South Korea | 2016 | Korea National Health and Nutrition Examination Survey | National | both | 5+ | 5+ | 3,288 | 4,042 | |
| 2878 | South Korea | 2016 | Korea National School Health Examination Survey (KNSHES) | National | both | 6-18 | 6-18 | 42,242 | 40,631 | |
| 2879 | South Korea | 2016-2017 | Korean National Health Insurance | National | both | 40+ | 40+ | 8,534,031 | 9,071,978 | |
| 2880 | South Korea | 2017 | The South Korean Consciption Database | National | both | 19 | | 323,457 | | |
| 2881 | South Korea | 2017 | Korea National Health and Nutrition Examination Survey | National | both | 5+ | 5+ | 3,341 | 3,986 | |
| 2882 | South Korea | 2018 | Korea National Health and Nutrition Examination Survey | National | both | 5+ | 5+ | 3,277 | 3,985 | |
| 2883 | South Korea | 2018-2019 | Korean National Health Insurance | National | both | 40+ | 40+ | 9,307,694 | 9,938,357 | |
| 2884 | South Korea | 2019 | Korea National Health and Nutrition Examination Survey | National | both | 5+ | 5+ | 3,377 | 3,992 | |
| 2885 | South Korea | 2020 | Korea National Health and Nutrition Examination Survey | National | both | 5+ | 5+ | 3,144 | 3,610 | |
| 2886 | South Korea | 2021 | Korea National Health and Nutrition Examination Survey | National | both | 5+ | 5+ | 2,907 | 3,546 | |
| 2887 | South Sudan | 2017 | Prevalence of hypertension and associated cardiovascular risk factors among adults aged 18-69 years in Juba City, South Sudan | Community | urban | 18-69 | 18-69 | 365 | 464 | |
| 2888 | Spain | 1985 | INTERSALT, Manresa | Community | urban | 20-59 | 20-59 | 100 | 100 | |
| 2889 | Spain | 1986 | INTERSALT, Torrejo | Community | urban | 20-59 | 20-59 | 100 | 100 | |
| 2890 | Spain | 1986-1988 | MONICA, Catalonia | Subnational | both | 25-64 | 25-64 | 1,251 | 1,271 | |
| 2891 | Spain | 1989 | Cardiovascular Risk Factors Study in Catalonia | Subnational | both | 15+ | 15+ | 330 | 371 | |
| 2892 | Spain | 1989-1994 | SEEDO | Subnational | both | 25-60 | 25-60 | 2,533 | 2,855 | |
| 2893 | Spain | 1990 | Banegas et al., Hypertension 32(6):998-1002, 1998 | National | both | 35-65 | 35-65 | 810 | 1,203 | |
| 2894 | Spain | 1990-1992 | MONICA, Catalonia | Subnational | both | 25-64 | 25-64 | 1,719 | 1,191 | |
| 2895 | Spain | 1990-2000 | SEEDO | Subnational | both | 25-60 | 25-60 | 4,707 | 5,178 | |
| 2896 | Spain | 1991-1993 | Encuesta de Factores de Riesgo Cardiovascular en la Región de Murcia (Cardiovascular Risk Factors Survey) | Subnational | both | 18-69 | 18-69 | 1,512 | 1,562 | |
| 2897 | Spain | 1992 | CINDI | Subnational | both | 25-64 | 25-64 | 1,194 | 1,454 | |
| 2898 | Spain | 1992 | ENCAT | Community | both | 15-80 | 15-80 | 786 | 952 | |
| 2899 | Spain | 1994-1995 | Encuesta de Nutrición y Salud Comunidad Valenciana 1994-95 (ENCV) | Subnational | urban | 15+ | 15+ | 830 | 959 | |
| 2900 | Spain | 1994-1996 | MONICA, Catalonia | Subnational | both | 25-64 | 25-64 | 1,800 | 1,628 | |
| 2901 | Spain | 1996 | Guía Study | Community | urban | 30+ | 30+ | 305 | 384 | |
| 2902 | Spain | 1996-2002 | Castells et al., J Epidemiol Community Health 60(4):316-21, 2006 | Community | urban | | 50-69 | | 26,963 | |
| 2903 | Spain | 1997 | Soriguer et al., Eur J Epidemiol 19(1):33-40, 2004 | Community | rural | 18-65 | 18-65 | 613 | 613 | |
| 2904 | Spain | 1998-2000 | EnKID study | National | both | 5-24 | 5-24 | 1,452 | 1,730 | |
| 2905 | Spain | 1999-2000 | ENIB | Community | both | 20-60 | 20-60 | 498 | 702 | |
| 2906 | Spain | 1999-2000 | Factores de riesgo en las islas Baleares: Estudio CORSAIB | Subnational | both | 35-74 | 35-74 | 802 | 857 | |
| 2907 | Spain | 2000-2001 | Regidor et al., J Hum Hypertens 20(1):73-82, 2006 | National | both | 60+ | 60+ | 1,318 | 2,281 | |
| 2908 | Spain | 2000-2001 | EUREYE Study | Subnational | both | 65+ | 65+ | 274 | 324 | |
| 2909 | Spain | 2000-2005 | CDC of the Canary Islands | Subnational | both | 18-75 | 18-75 | 2,878 | 3,719 | |
| 2910 | Spain | 2001-2002 | Catalan Health Interview Survey | Subnational | both | 18-74 | 18-74 | 597 | 745 | |
| 2911 | Spain | 2001-2003 | DIabetes, Nutrición y Obesidad en la población adulta de la Región de Murcia (DINO) | Subnational | both | 20+ | 20+ | 715 | 828 | |
| 2912 | Spain | 2002-2003 | ENCAT | Community | both | 15-80 | 15-80 | 712 | 813 | |
| 2913 | Spain | 2003 | The European Male Ageing Study | Community | both | 40+ | | 405 | | |
| 2914 | Spain | 2003-2005 | Registre Gironi del Cor (REGICOR) | Subnational | both | 35-79 | 35-79 | 2,951 | 3,266 | |
| 2915 | Spain | 2004 | Vioque J et al., Obesity 16(3):664-70, 2008 | Community | urban | 24+ | 24+ | 87 | 115 | |
| 2916 | Spain | 2004 | Cardiovascular Risk Study in Castilla y León (RECCyL) | Subnational | both | 15+ | 15+ | 1,903 | 2,077 | |
| 2917 | Spain | 2004-2006 | PREVICTUS | National | both | 60+ | 60+ | 3,193 | 3,640 | |
| 2918 | Spain | 2006-2007 | HELENA | Community | urban | 12-17 | 12-17 | 188 | 193 | |
| 2919 | Spain | 2006-2008 | Biblioni Mdel et al., Br J Nutr 103(1):99-106, 2010 | Community | both | 12-17 | 12-17 | 571 | 652 | |
| 2920 | Spain | 2007-2009 | Harmonizing Equation of Risk in Mediterraneon countries EXtremadura (HERMEX) | Subnational | both | 25-79 | 25-79 | 1,298 | 1,498 | |

| | Country | Study years | Survey/Study name/Citation | Level of representative-ness | Rural, urban, or both | Age range as in NCD-RisC database | | Sample size | | Notes |
|---|---|---|---|---|---|---|---|---|---|---|
| | | | | | | Male | Female | Male | Female | |
| 2921 | Spain | 2007-2010 | Identification and prevention of Dietary- and lifestyle-induced health EFfects In Children and infantS (IDEFICS) | Community | urban | 5-9 | 5-9 | 474 | 468 | |
| 2922 | Spain | 2008 | The European Male Ageing Study | Community | both | 40+ | | 272 | | |
| 2923 | Spain | 2008-2010 | Study on Nutrition and Cardiovascular Risk in Spain | National | both | 18+ | 18+ | 5,756 | 6,397 | |
| 2924 | Spain | 2009 | Cardiovascular Risk Study in Castilla y León (RECCyL) | Subnational | both | 20+ | 20+ | 1,315 | 1,590 | |
| 2925 | Spain | 2010 | EuropeaN Energy balance Research to prevent excessive weight Gain among Youth - The ENERGY-project | Subnational | urban | 10-12 | 10-12 | 479 | 520 | |
| 2926 | Spain | 2010-2011 | ALimentación, Actividad Física, Desarrollo INfantil y Obesidad (ALADINO); Childhood Obesity Surveillance Initiative 2 | National | both | 6-9 | 6-9 | 3,837 | 3,817 | |
| 2927 | Spain | 2012 | Effects of a lifestyle intervention on the prevention of childhood obesity: a community-based model | Subnational | urban | 8-12 | 8-12 | 1,171 | 1,081 | |
| 2928 | Spain | 2012-2013 | Brain Development and Air Pollution Ultrafine Particles in School Children-BREATHE Project | Subnational | urban | 7-12 | 7-12 | 1,338 | 1,325 | |
| 2929 | Spain | 2012-2013 | INfancia y Medio Ambiente (Childhood and Environment) Project - Menorca | Subnational | both | 14-15 | 14-15 | 162 | 165 | |
| 2930 | Spain | 2012-2013 | Infancia y Medio Ambiente (Childhood and Environment) Birth Cohort study - Sabadell | Subnational | urban | 5-7 | 5-7 | 280 | 260 | 25 |
| 2931 | Spain | 2012-2013 | INfancia y Medio Ambiente (Childhood and Environment) Project - Valencia | Subnational | both | 7-8 | 7-8 | 229 | 232 | |
| 2932 | Spain | 2013 | ANIBES Study | National | both | 9-75 | 9-75 | 1,160 | 1,125 | |
| 2933 | Spain | 2013 | ALimentación, Actividad Física, Desarrollo INfantil y Obesidad (ALADINO); Childhood Obesity Surveillance Initiative 3 | National | both | 7-8 | 7-8 | 1,682 | 1,744 | |
| 2934 | Spain | 2013-2014 | Effects of a lifestyle intervention on the prevention of childhood obesity: a community-based model | Subnational | urban | 9-13 | 9-13 | 1,086 | 1,004 | |
| 2935 | Spain | 2013-2015 | INfancia y Medio Ambiente (Childhood and Environment) Project - Valencia | Subnational | both | 8-9 | 8-9 | 225 | 218 | |
| 2936 | Spain | 2013-2016 | Infancia y Medio Ambiente (Childhood and Environment) Birth Cohort study - Sabadell | Subnational | urban | 7-10 | 7-10 | 253 | 236 | 25 |
| 2937 | Spain | 2014 | Cardiovascular Risk Study in Castilla y León (RECCyL) | Subnational | both | 20+ | 20+ | 1,215 | 1,475 | |
| 2938 | Spain | 2015 | Study on Nutrition and Cardiovascular Risk in Spain (ENRICA) | National | both | 65+ | 65+ | 711 | 770 | |
| 2939 | Spain | 2015-2016 | ALimentación, Actividad Física, Desarrollo INfantil y Obesidad (ALADINO); Childhood Obesity Surveillance Initiative 4 | National | both | 6-9 | 6-9 | 5,532 | 5,367 | |
| 2940 | Spain | 2015-2016 | INfancia y Medio Ambiente (Childhood and Environment) Project - Gipuzkoa | Subnational | both | 7-8 | 7-8 | 195 | 195 | |
| 2941 | Spain | 2015-2017 | INfancia y Medio Ambiente (Childhood and Environment) Project - Valencia | Subnational | both | 9-12 | 9-12 | 197 | 218 | |
| 2942 | Spain | 2016-2017 | Estudio de Nutrición y Riesgo Cardiovascular en España (ENRICA)-Seniors cohort | Subnational | urban | 65-94 | 65-94 | 1,340 | 1,478 | |
| 2943 | Spain | 2016-2018 | Infancia y Medio Ambiente (Childhood and Environment) Birth Cohort study - Sabadell | Subnational | urban | 9-12 | 9-12 | 256 | 239 | 25 |
| 2944 | Spain | 2017-2019 | Urban environment and childhood obesity in Catalonia (ECHOCAT) | Community | urban | 9-12 | 9-12 | 870 | 977 | |
| 2945 | Spain | 2018 | Childhood obesity cohort study of Sant Boi de Llobregat (SANTBOISA study) | Community | urban | 5-10 | 5-10 | 299 | 266 | |
| 2946 | Spain | 2018-2019 | INfancia y Medio Ambiente (Childhood and Environment) Project - Gipuzkoa | Subnational | both | 10-11 | 10-11 | 174 | 204 | |
| 2947 | Spain | 2019 | ALimentación, Actividad Física, Desarrollo INfantil y Obesidad (ALADINO); Childhood Obesity Surveillance Initiative 5 | National | both | 6-9 | 6-9 | 8,512 | 8,151 | |
| 2948 | Spain | 2019 | Estudio de Nutrición y Riesgo Cardiovascular en España (ENRICA)-Seniors cohort | Subnational | urban | 65-95 | 65-95 | 834 | 815 | |
| 2949 | Spain | 2019 | Physical Activity, Sedentarism and Obesity of Spanish youth - PASOS study | National | both | 8-16 | 8-16 | 1,804 | 1,921 | |
| 2950 | Spain | 2021 | INfancia y Medio Ambiente (Childhood and Environment) Project - Gipuzkoa | Subnational | both | 13-15 | 13-15 | 121 | 148 | |
| 2951 | Sri Lanka | 2003 | Wijewardene et al., Ceylon Med J 50:62-70, 2005 | Subnational | both | 30-65 | 30-65 | 275 | 296 | |
| 2952 | Sri Lanka | 2003 | Wijewardene et al., Ceylon Med J 50:62-70, 2005 | Subnational | both | 30-65 | 30-65 | 139 | 192 | |
| 2953 | Sri Lanka | 2003 | Wijewardene et al., Ceylon Med J 50:62-70, 2005 | Subnational | both | 30-65 | 30-65 | 1,891 | 2,410 | |
| 2954 | Sri Lanka | 2003 | Wijewardene et al., Ceylon Med J 50:62-70, 2005 | Subnational | both | 30-65 | 30-65 | 387 | 457 | |
| 2955 | Sri Lanka | 2005-2006 | Sri Lanka Diabetes, Cardiovascular study (SLDCS) | National | both | 18+ | 18+ | 1,773 | 2,714 | |
| 2956 | Sri Lanka | 2006 | STEPS | National | both | 15-64 | 15-64 | 6,140 | 6,213 | |
| 2957 | Sri Lanka | 2006-2007 | DHS | National | both | | 15-49 | | 12,539 | |
| 2958 | Sri Lanka | 2014 | STEPS | National | both | 18-69 | 18-69 | 1,863 | 2,893 | |
| 2959 | Sri Lanka | 2016 | Global School-based Student Health Survey | National | both | 13-17 | 13-17 | 671 | 923 | |
| 2960 | Sri Lanka | 2021 | STEPS | National | both | 18-69 | 18-69 | 2,172 | 3,458 | |
| 2961 | State of Palestine | 1996 | Stene et al., Eur J Clin Nutr 55(9):805-11, 2001 | Community | rural | 30-65 | 30-65 | 208 | 269 | |
| 2962 | State of Palestine | 1996-1998 | Ramallah study | Community | rural | 15-64 | 15-64 | 206 | 482 | |
| 2963 | State of Palestine | 1996-1998 | Ramallah study | Community | urban | 15-64 | 15-64 | 182 | 493 | |
| 2964 | State of Palestine | 1999-2000 | The First National Health and Nutrition Survey | National | both | 18-64 | 18-64 | 1,736 | 1,869 | |
| 2965 | State of Palestine | 2010 | Global School-based Student Health Survey | National | both | 13-17 | 13-17 | 1,822 | 1,865 | |
| 2966 | State of Palestine | 2010 | STEPS | National | both | 15-64 | 15-64 | 2,578 | 4,052 | |
| 2967 | State of Palestine | 2013 | Palestine Micronutrient Survey | National | both | 15-18 | 15-18 | 1,201 | 1,166 | |
| 2968 | State of Palestine | 2022 | STEPS | National | both | 18-69 | 18-69 | 1,677 | 3,637 | |
| 2969 | Sudan | 2005 | STEPS | Subnational | both | 25-64 | 25-64 | 626 | 881 | |
| 2970 | Sudan | 2016 | STEPS | National | both | 18-69 | 18-69 | 2,661 | 4,544 | |
| 2971 | Sudan | 2018 | Prevalence and associated factors of hypertension among adults in Gadarif in eastern Sudan: a community-based study | Community | urban | 18+ | 18+ | 178 | 421 | |

| | Country | Study years | Survey/Study name/Citation | Level of representative-ness | Rural, urban, or both | Age range as in NCD-RisC database | | Sample size | | Notes |
|---|---|---|---|---|---|---|---|---|---|---|
| | | | | | | Male | Female | Male | Female | |
| 2972 | Suriname | 2013-2015 | The Healthy Life in Suriname Study (HELISUR) | Subnational | urban | 18-70 | 18-70 | 424 | 722 | |
| 2973 | Sweden | 1977 | The Swedish Conscription Database | National | both | 17-19 | | 47,898 | | 1 |
| 2974 | Sweden | 1978 | The Swedish Conscription Database | National | both | 17-19 | | 11,486 | | 1 |
| 2975 | Sweden | 1979 | The Swedish Conscription Database | National | both | 17-19 | | 37,487 | | 1 |
| 2976 | Sweden | 1979-1980 | 1973 Birth Cohort | National | both | 6 | 6 | 451 | 439 | |
| 2977 | Sweden | 1980 | BMI Epidemiology Study | Community | urban | 9 | | 324 | | |
| 2978 | Sweden | 1980 | The Swedish Conscription Database | National | both | 17-18 | | 49,630 | | |
| 2979 | Sweden | 1980-1981 | 1973 Birth Cohort | National | both | 7 | 7 | 1,422 | 1,323 | |
| 2980 | Sweden | 1980-1981 | Population Study of Women in Gothenburg | Community | urban | | 50-72 | | 1,153 | |
| 2981 | Sweden | 1980-1985 | Uppsala Longitudinal Study of Adult Men | Community | both | 55-64 | | 1,846 | | 26 |
| 2982 | Sweden | 1981 | The Swedish Conscription Database | National | both | 17-18 | | 52,282 | | |
| 2983 | Sweden | 1981-1982 | 1973 Birth Cohort | National | both | 8 | 8 | 890 | 869 | |
| 2984 | Sweden | 1982 | The Swedish Conscription Database | National | both | 17-18 | | 54,288 | | |
| 2985 | Sweden | 1982-1983 | 1973 Birth Cohort | National | both | 9 | 9 | 670 | 635 | |
| 2986 | Sweden | 1983 | BMI Epidemiology Study | Community | urban | 7 | | 103 | | |
| 2987 | Sweden | 1983 | The Swedish Conscription Database | National | both | 17-18 | | 52,895 | | |
| 2988 | Sweden | 1983-1984 | 1973 Birth Cohort | National | both | 10 | 10 | 1,448 | 1,340 | |
| 2989 | Sweden | 1984 | BMI Epidemiology Study | Community | urban | 8 | | 407 | | |
| 2990 | Sweden | 1984 | The Swedish Conscription Database | National | both | 17-18 | | 36,273 | | |
| 2991 | Sweden | 1984-1985 | 1973 Birth Cohort | National | both | 11 | 11 | 487 | 498 | |
| 2992 | Sweden | 1985 | BMI Epidemiology Study | Community | urban | 9 | | 357 | | |
| 2993 | Sweden | 1985 | MONICA Gothenburg | Community | urban | 25-64 | 25-64 | 666 | 702 | |
| 2994 | Sweden | 1985 | The Swedish Conscription Database | National | both | 17-18 | | 14,918 | | |
| 2995 | Sweden | 1985-1986 | 1973 Birth Cohort | National | both | 12 | 12 | 1,238 | 1,158 | |
| 2996 | Sweden | 1985-1989 | Västerbotten Intervention Project | Subnational | both | 25-64 | 25-64 | 1,676 | 1,554 | |
| 2997 | Sweden | 1985-1996 | EPIC Umea | Subnational | both | 24-72 | 24-72 | 12,359 | 13,217 | |
| 2998 | Sweden | 1986 | MONICA Northern Sweden | Subnational | both | 25-64 | 25-64 | 822 | 798 | |
| 2999 | Sweden | 1986 | The Swedish Conscription Database | National | both | 17-18 | | 48,454 | | |
| 3000 | Sweden | 1986-1987 | 1973 Birth Cohort | National | both | 13 | 13 | 709 | 729 | |
| 3001 | Sweden | 1986-1987 | 1981 Birth Cohort | National | both | 5 | 5 | 935 | 958 | |
| 3002 | Sweden | 1987 | The Swedish Conscription Database | National | both | 17-18 | | 49,306 | | |
| 3003 | Sweden | 1987-1988 | 1973 Birth Cohort | National | both | 14 | 14 | 1,405 | 1,294 | |
| 3004 | Sweden | 1987-1988 | 1981 Birth Cohort | National | both | 6 | 6 | 808 | 780 | |
| 3005 | Sweden | 1988 | BMI Epidemiology Study | Community | urban | 7 | | 65 | | |
| 3006 | Sweden | 1988 | The Swedish Conscription Database | National | both | 17-18 | | 47,923 | | |
| 3007 | Sweden | 1988-1989 | 1973 Birth Cohort | National | both | 15 | 15 | 964 | 929 | |
| 3008 | Sweden | 1988-1989 | 1981 Birth Cohort | National | both | 7 | 7 | 1,508 | 1,489 | |
| 3009 | Sweden | 1989 | BMI Epidemiology Study | Community | urban | 8 | | 407 | | |
| 3010 | Sweden | 1989 | The Swedish Conscription Database | National | both | 17-18 | | 48,117 | | |
| 3011 | Sweden | 1989-1990 | 1973 Birth Cohort | National | both | 16 | 16 | 912 | 785 | |
| 3012 | Sweden | 1989-1990 | 1981 Birth Cohort | National | both | 8 | 8 | 1,497 | 1,511 | |
| 3013 | Sweden | 1990 | BMI Epidemiology Study | Community | urban | 9 | | 379 | | |
| 3014 | Sweden | 1990 | MONICA Northern Sweden | Subnational | both | 25-64 | 25-64 | 773 | 806 | |
| 3015 | Sweden | 1990 | MONICA Gothenburg | Community | urban | 25-64 | 25-64 | 775 | 775 | |
| 3016 | Sweden | 1990 | The Swedish Conscription Database | National | both | 17-18 | | 48,882 | | |
| 3017 | Sweden | 1990-1991 | 1973 Birth Cohort | National | both | 17 | 17 | 881 | 588 | |
| 3018 | Sweden | 1990-1991 | 1981 Birth Cohort | National | both | 9 | 9 | 1,091 | 1,108 | |
| 3019 | Sweden | 1990-1992 | Västerbotten Intervention Project | Subnational | both | 25-64 | 25-64 | 7,263 | 7,804 | |
| 3020 | Sweden | 1991 | The Swedish Conscription Database | National | both | 17-18 | | 49,150 | | |
| 3021 | Sweden | 1991-1992 | 1981 Birth Cohort | National | both | 10 | 10 | 1,405 | 1,415 | |
| 3022 | Sweden | 1991-1995 | Uppsala Longitudinal Study of Adult Men | Community | both | 69-74 | | 1,215 | | 26 |
| 3023 | Sweden | 1991-1996 | Malmö Diet and Cancer | Community | urban | 45-73 | 45-73 | 12,096 | 18,293 | |
| 3024 | Sweden | 1992 | 1973 Birth Cohort | National | both | 18 | 18 | 1,290 | 390 | |
| 3025 | Sweden | 1992 | The Swedish Conscription Database | National | both | 17-18 | | 47,275 | | |
| 3026 | Sweden | 1992-1993 | 1981 Birth Cohort | National | both | 11 | 11 | 785 | 785 | |
| 3027 | Sweden | 1992-1993 | Population Study of Women in Gothenburg | Community | urban | | 62-84 | | 802 | |
| 3028 | Sweden | 1993 | BMI Epidemiology Study | Community | urban | 7 | | 125 | | |

| | Country | Study years | Survey/Study name/Citation | Level of representative-ness | Rural, urban, or both | Age range as in NCD-RisC database | | Sample size | | Notes |
|---|---|---|---|---|---|---|---|---|---|---|
| | | | | | | Male | Female | Male | Female | |
| 3029 | Sweden | 1993 | The Swedish Conscription Database | National | both | 17-18 | | 46,245 | | |
| 3030 | Sweden | 1993-1994 | 1981 Birth Cohort | National | both | 12 | 12 | 1,318 | 1,378 | |
| 3031 | Sweden | 1993-1995 | Västerbotten Intervention Project | Subnational | both | 25-64 | 25-64 | 9,804 | 10,727 | |
| 3032 | Sweden | 1994 | BMI Epidemiology Study | Community | urban | 8 | | 376 | | |
| 3033 | Sweden | 1994 | Nilson et al., Scand J Prim Health Care 18(2):111-112, 2000 | Community | urban | 56-65 | 56-65 | 170 | 217 | |
| 3034 | Sweden | 1994 | MONICA Northern Sweden | Subnational | both | 25-74 | 25-74 | 940 | 961 | |
| 3035 | Sweden | 1994 | The Swedish Conscription Database | National | both | 17-18 | | 43,648 | | |
| 3036 | Sweden | 1994-1995 | 1981 Birth Cohort | National | both | 13 | 13 | 924 | 954 | |
| 3037 | Sweden | 1994-1996 | Kungsholmen Project | Community | urban | 75+ | 75+ | 160 | 160 | |
| 3038 | Sweden | 1995 | BMI Epidemiology Study | Community | urban | 9 | | 293 | | |
| 3039 | Sweden | 1995 | MONICA Gothenburg | Community | urban | 25-64 | 25-64 | 745 | 867 | |
| 3040 | Sweden | 1995 | The Swedish Conscription Database | National | both | 17-18 | | 43,674 | | |
| 3041 | Sweden | 1995-1996 | 1981 Birth Cohort | National | both | 14 | 14 | 1,202 | 1,266 | |
| 3042 | Sweden | 1996 | The Swedish Conscription Database | National | both | 17-18 | | 43,794 | | |
| 3043 | Sweden | 1996-1997 | 1981 Birth Cohort | National | both | 15 | 15 | 1,143 | 1,134 | |
| 3044 | Sweden | 1996-1998 | Västerbotten Intervention Project | Subnational | both | 25-64 | 25-64 | 8,327 | 8,893 | |
| 3045 | Sweden | 1997 | The Swedish Conscription Database | National | both | 17-18 | | 37,078 | | |
| 3046 | Sweden | 1997-1998 | 1981 Birth Cohort | National | both | 16 | 16 | 819 | 862 | |
| 3047 | Sweden | 1997-2001 | Uppsala Longitudinal Study of Adult Men | Community | both | 73-80 | | 783 | | 26 |
| 3048 | Sweden | 1998 | BMI Epidemiology Study | Community | urban | 7 | | 707 | | |
| 3049 | Sweden | 1998 | The Swedish Conscription Database | National | both | 17-18 | | 42,564 | | |
| 3050 | Sweden | 1998-1999 | 1981 Birth Cohort | National | both | 17 | 17 | 406 | 171 | |
| 3051 | Sweden | 1998-1999 | European Youth Heart Study | Subnational | urban | 8-16 | 8-16 | 525 | 602 | |
| 3052 | Sweden | 1998-2001 | The Kalixanda study | Community | both | 20+ | 20+ | 508 | 483 | |
| 3053 | Sweden | 1999 | 1981 Birth Cohort | National | both | 18 | 18 | 831 | 23 | |
| 3054 | Sweden | 1999 | BMI Epidemiology Study | Community | urban | 8 | | 980 | | |
| 3055 | Sweden | 1999 | MONICA Northern Sweden | Subnational | both | 25-74 | 25-74 | 889 | 920 | |
| 3056 | Sweden | 1999 | The Swedish Conscription Database | National | both | 17-18 | | 37,852 | | |
| 3057 | Sweden | 1999-2003 | Västerbotten Intervention Project | Subnational | both | 25-64 | 25-64 | 6,354 | 6,384 | |
| 3058 | Sweden | 2000 | BMI Epidemiology Study | Community | urban | 9 | | 860 | | |
| 3059 | Sweden | 2000 | The Swedish Conscription Database | National | both | 17-18 | | 31,328 | | |
| 3060 | Sweden | 2000-2001 | H70 Study | Community | urban | 70 | 70 | 242 | 270 | |
| 3061 | Sweden | 2000-2002 | The COMPASS study | Community | urban | 14-16 | 14-16 | 1,718 | 1,597 | |
| 3062 | Sweden | 2001 | The Swedish Conscription Database | National | both | 17-18 | | 29,809 | | |
| 3063 | Sweden | 2001-2004 | Swedish INTERGENE Cohort Study | Subnational | both | 24-76 | 24-76 | 1,694 | 1,906 | |
| 3064 | Sweden | 2001-2004 | PIVUS Study | Community | both | 70 | 70 | 1,521 | 1,527 | |
| 3065 | Sweden | 2002 | The Swedish Conscription Database | National | both | 17-18 | | 24,580 | | |
| 3066 | Sweden | 2003 | BMI Epidemiology Study | Community | urban | 7 | | 15 | | |
| 3067 | Sweden | 2003 | The European Male Ageing Study | Community | both | 40+ | | 396 | | |
| 3068 | Sweden | 2003 | The Swedish Conscription Database | National | both | 17-18 | | 29,198 | | |
| 3069 | Sweden | 2003-2004 | Welin et al., BMC Public Health 8:403, 2008 | Community | urban | 50 | 50 | 595 | 655 | |
| 3070 | Sweden | 2003-2004 | Welin et al., BMC Public Health 8:403, 2008 | Community | urban | 60 | | 667 | | |
| 3071 | Sweden | 2003-2005 | Uppsala Longitudinal Study of Adult Men | Community | both | 80-83 | | 512 | | 26 |
| 3072 | Sweden | 2004 | BMI Epidemiology Study | Community | urban | 8 | | 285 | | |
| 3073 | Sweden | 2004 | MONICA Northern Sweden | Subnational | both | 26-75 | 26-75 | 926 | 964 | |
| 3074 | Sweden | 2004 | The Swedish Conscription Database | National | both | 17-18 | | 27,857 | | |
| 3075 | Sweden | 2004-2005 | European Youth Heart Study | Subnational | urban | 15-21 | 15-21 | 196 | 262 | |
| 3076 | Sweden | 2004-2005 | Population Study of Women in Gothenburg | Community | urban | | 38-50 | | 494 | |
| 3077 | Sweden | 2005 | BMI Epidemiology Study | Community | urban | 9 | | 301 | | |
| 3078 | Sweden | 2005 | The Swedish Conscription Database | National | both | 17-18 | | 25,836 | | |
| 3079 | Sweden | 2005-2006 | H70 Study | Community | urban | 75 | 75 | 320 | 422 | |
| 3080 | Sweden | 2006-2007 | HELENA | Community | urban | 12-17 | 12-17 | 132 | 208 | |
| 3081 | Sweden | 2007 | BMI Epidemiology Study | Community | urban | 7 | | 1,327 | | |
| 3082 | Sweden | 2007-2009 | PIVUS Study | Community | both | 75 | 75 | 1,221 | 1,257 | |
| 3083 | Sweden | 2007-2010 | Identification and prevention of Dietary- and lifestyle-induced health EFfects In Children and infantS (IDEFICS) | Community | urban | 5-9 | 5-9 | 557 | 557 | |
| 3084 | Sweden | 2008 | BMI Epidemiology Study | Community | urban | 7-8 | | 2,501 | | |

| | Country | Study years | Survey/Study name/Citation | Level of representative-ness | Rural, urban, or both | Age range as in NCD-RisC database | | Sample size | | Notes |
|---|---|---|---|---|---|---|---|---|---|---|
| | | | | | | Male | Female | Male | Female | |
| 3085 | Sweden | 2008 | Childhood Obesity Surveillance Initiative 1 | National | both | 7-9 | 7-9 | 2,374 | 2,189 | |
| 3086 | Sweden | 2008 | The European Male Ageing Study | Community | both | 40+ | | 353 | | |
| 3087 | Sweden | 2008-2009 | Uppsala Longitudinal Study of Adult Men | Community | both | 84-88 | | 293 | | 26 |
| 3088 | Sweden | 2009 | BMI Epidemiology Study | Community | urban | 7-9 | | 3,706 | | |
| 3089 | Sweden | 2009 | MONICA Northern Sweden | Subnational | both | 25-74 | 25-74 | 849 | 857 | |
| 3090 | Sweden | 2010 | BMI Epidemiology Study | Community | urban | 8-9 | | 2,425 | | |
| 3091 | Sweden | 2011 | BMI Epidemiology Study | Community | urban | 9 | | 1,412 | | |
| 3092 | Sweden | 2011-2014 | EpiHealth | National | both | 45-75 | 45-75 | 4,731 | 6,054 | |
| 3093 | Sweden | 2011-2014 | PIVUS Study | Community | both | 80 | 80 | 909 | 903 | |
| 3094 | Sweden | 2012 | BMI Epidemiology Study | Community | urban | 7 | | 1,551 | | |
| 3095 | Sweden | 2013 | BMI Epidemiology Study | Community | urban | 7-8 | | 2,875 | | |
| 3096 | Sweden | 2013-2015 | Uppsala Longitudinal Study of Adult Men | Community | both | 89-94 | | 119 | | 26 |
| 3097 | Sweden | 2014 | BMI Epidemiology Study | Community | urban | 7-9 | | 4,251 | | |
| 3098 | Sweden | 2014 | MONICA Northern Sweden | Subnational | both | 25-74 | 25-74 | 753 | 795 | |
| 3099 | Sweden | 2014-2016 | Swedish INTERGENE Cohort Study | Subnational | urban | 37-88 | 37-88 | 602 | 653 | |
| 3100 | Sweden | 2015 | BMI Epidemiology Study | Community | urban | 8-9 | | 1,917 | | |
| 3101 | Sweden | 2015-2016 | Childhood Obesity Surveillance Initiative 4 | National | both | 6-9 | 6-9 | 4,070 | 3,798 | |
| 3102 | Sweden | 2016 | BMI Epidemiology Study | Community | urban | 9 | | 35 | | |
| 3103 | Sweden | 2016-2017 | Population Study of Women in Gothenburg | Community | urban | | 38-50 | | 570 | |
| 3104 | Sweden | 2019 | Childhood Obesity Surveillance Initiative 5 | National | both | 6-9 | 6-9 | 31,642 | 30,002 | |
| 3105 | Switzerland | 1984-1986 | The Swiss MONICA Study Wave I | Subnational | both | 25-74 | 25-74 | 1,744 | 1,689 | |
| 3106 | Switzerland | 1988-1989 | The Swiss MONICA Study Wave II | Subnational | both | 25-74 | 25-74 | 1,778 | 1,684 | |
| 3107 | Switzerland | 1992-1993 | The Swiss MONICA Study Wave III | Subnational | both | 25-74 | 25-74 | 1,577 | 1,672 | |
| 3108 | Switzerland | 2002 | Prevalence of overweight and obesity in 6-12-year old children in Switzerland | National | both | 6-12 | 6-12 | 1,196 | 1,235 | |
| 3109 | Switzerland | 2003-2006 | Cohorte Lausannoise | Community | urban | 35-75 | 35-75 | 3,186 | 3,536 | |
| 3110 | Switzerland | 2004 | The Swiss Conscription Database | National | both | 18-20 | | 20,491 | | |
| 3111 | Switzerland | 2005 | The Swiss Conscription Database | National | both | 18-20 | | 32,131 | | |
| 3112 | Switzerland | 2005 | Kinder- und Jugendsportstudie (KISS) | Subnational | both | 6-13 | 6-13 | 239 | 256 | |
| 3113 | Switzerland | 2005-2006 | BMI Monitoring for Switzerland - Study 1 | Community | urban | 5-11 | 5-11 | 4,477 | 4,103 | 27 |
| 3114 | Switzerland | 2005-2006 | Chiolero et al., J Hypertens 25(11):2209-17, 2007 | Subnational | both | 10-14 | 10-14 | 2,621 | 2,586 | |
| 3115 | Switzerland | 2006 | The Swiss Conscription Database | National | both | 18-20 | | 34,530 | | |
| 3116 | Switzerland | 2006 | Kinder- und Jugendsportstudie (KISS) | Subnational | both | 7-14 | 7-14 | 100 | 105 | |
| 3117 | Switzerland | 2006-2007 | BMI Monitoring for Switzerland - Study 1 | Community | urban | 5-11 | 5-11 | 4,061 | 3,896 | 27 |
| 3118 | Switzerland | 2007 | The Swiss Conscription Database | National | both | 18-20 | | 36,194 | | |
| 3119 | Switzerland | 2007 | Prevalence of overweight and obesity in 6-12-year old children in Switzerland | National | both | 6-12 | 6-12 | 1,082 | 1,136 | |
| 3120 | Switzerland | 2007-2008 | BMI Monitoring for Switzerland - Study 1 | Community | urban | 5-11 | 5-11 | 4,067 | 4,009 | 27 |
| 3121 | Switzerland | 2007-2012 | Bus Santé Study | Subnational | urban | 20-80 | 20-80 | 1,884 | 1,911 | |
| 3122 | Switzerland | 2008 | The Swiss Conscription Database | National | both | 18-20 | | 34,497 | | |
| 3123 | Switzerland | 2008-2009 | BMI Monitoring for Switzerland - Study 1 | Community | urban | 5-11 | 5-11 | 3,998 | 3,847 | 27 |
| 3124 | Switzerland | 2008-2010 | BMI Monitoring for Switzerland - Study 2 | Subnational | both | 6 | 6 | 1,048 | 1,110 | |
| 3125 | Switzerland | 2009 | The Swiss Conscription Database | National | both | 18-20 | | 34,896 | | |
| 3126 | Switzerland | 2009 | Kinder- und Jugendsportstudie (KISS) | Subnational | both | 10-17 | 10-17 | 44 | 65 | |
| 3127 | Switzerland | 2009-2010 | BMI Monitoring for Switzerland - Study 1 | Community | urban | 5-11 | 5-11 | 4,051 | 3,913 | 27 |
| 3128 | Switzerland | 2009-2012 | Cohorte Lausannoise | Community | urban | 40-75 | 40-75 | 2,176 | 2,494 | |
| 3129 | Switzerland | 2010 | The Swiss Conscription Database | National | both | 18-20 | | 37,214 | | |
| 3130 | Switzerland | 2010 | EuropeaN Energy balance Research to prevent excessive weight Gain among Youth - The ENERGY-project | Subnational | both | 10-12 | 10-12 | 287 | 270 | |
| 3131 | Switzerland | 2010-2011 | BMI Monitoring for Switzerland - Study 1 | Community | urban | 5-11 | 5-11 | 4,092 | 3,734 | 27 |
| 3132 | Switzerland | 2010-2013 | BMI Monitoring for Switzerland - Study 2 | Subnational | both | 5-11 | 5-11 | 9,735 | 9,346 | |
| 3133 | Switzerland | 2011 | The Swiss Conscription Database | National | both | 18-20 | | 38,108 | | |
| 3134 | Switzerland | 2011-2012 | BMI Monitoring for Switzerland - Study 1 | Community | urban | 5-11 | 5-11 | 4,085 | 3,940 | 27 |
| 3135 | Switzerland | 2012 | The Swiss Conscription Database | National | both | 18-20 | | 36,938 | | |
| 3136 | Switzerland | 2012 | Prevalence of overweight and obesity in 6-12-year old children in Switzerland | National | both | 6-12 | 6-12 | 1,499 | 1,464 | |
| 3137 | Switzerland | 2012-2013 | BMI Monitoring for Switzerland - Study 1 | Community | urban | 5-11 | 5-11 | 4,224 | 4,042 | 27 |
| 3138 | Switzerland | 2013 | The Swiss Conscription Database | National | both | 18-20 | | 32,890 | | |
| 3139 | Switzerland | 2013-2014 | BMI Monitoring for Switzerland - Study 1 | Community | urban | 5-11 | 5-11 | 4,333 | 4,073 | 27 |
| 3140 | Switzerland | 2013-2016 | Bus Santé Study | Subnational | urban | 20-74 | 20-74 | 2,022 | 2,186 | |
| 3141 | Switzerland | 2014 | The Swiss Conscription Database | National | both | 18-20 | | 32,691 | | |

| | Country | Study years | Survey/Study name/Citation | Level of representative-ness | Rural, urban, or both | Age range as in NCD-RisC database | | Sample size | | Notes |
|---|---|---|---|---|---|---|---|---|---|---|
| | | | | | | Male | Female | Male | Female | |
| 3142 | Switzerland | 2014-2015 | National Nutrition Survey menuCH | National | both | 18-75 | 18-75 | 937 | 1,107 | |
| 3143 | Switzerland | 2014-2015 | BMI Monitoring for Switzerland - Study 1 | Community | urban | 5-11 | 5-11 | 4,657 | 4,625 | 27 |
| 3144 | Switzerland | 2014-2016 | BMI Monitoring for Switzerland - Study 2 | Subnational | both | 5-11 | 5-11 | 7,759 | 7,343 | |
| 3145 | Switzerland | 2014-2017 | Cohorte Lausannoise | Community | urban | 45-87 | 45-87 | 2,008 | 2,473 | |
| 3146 | Switzerland | 2015 | The Swiss Conscription Database | National | both | 18-20 | | 32,616 | | |
| 3147 | Switzerland | 2015-2016 | BMI Monitoring for Switzerland - Study 1 | Community | urban | 5-11 | 5-11 | 3,326 | 3,058 | 27 |
| 3148 | Switzerland | 2016 | The Swiss Conscription Database | National | both | 18-20 | | 26,716 | | |
| 3149 | Switzerland | 2016-2017 | BMI Monitoring for Switzerland - Study 1 | Community | urban | 5-11 | 5-11 | 3,457 | 3,121 | 27 |
| 3150 | Switzerland | 2017 | The Swiss Conscription Database | National | both | 18-20 | | 25,509 | | |
| 3151 | Switzerland | 2017-2018 | BMI Monitoring for Switzerland - Study 1 | Community | urban | 5-11 | 5-11 | 6,044 | 5,788 | 27 |
| 3152 | Switzerland | 2017-2018 | National Studie Gesundheit und Ernährung von Primarschülern (CHildHNS) | National | both | 6-12 | 6-12 | 1,135 | 1,144 | |
| 3153 | Switzerland | 2018 | The Swiss Conscription Database | National | both | 18-20 | | 20,906 | | |
| 3154 | Switzerland | 2018-2019 | BMI Monitoring for Switzerland - Study 1 | Community | urban | 5-11 | 5-11 | 6,693 | 6,370 | 27 |
| 3155 | Switzerland | 2018-2020 | BMI Monitoring for Switzerland - Study 2 | Subnational | both | 5-11 | 5-11 | 9,161 | 8,694 | |
| 3156 | Switzerland | 2018-2021 | Cohorte Lausannoise | Community | urban | 49-90 | 49-90 | 1,600 | 1,946 | |
| 3157 | Switzerland | 2019 | The Swiss Conscription Database | National | both | 18-20 | | 18,529 | | |
| 3158 | Switzerland | 2019-2020 | BMI Monitoring for Switzerland - Study 1 | Community | urban | 5-11 | 5-11 | 5,873 | 5,453 | 27 |
| 3159 | Switzerland | 2020 | The Swiss Conscription Database | National | both | 18-20 | | 12,531 | | |
| 3160 | Switzerland | 2020-2021 | BMI Monitoring for Switzerland - Study 1 | Community | urban | 5-11 | 5-11 | 5,697 | 5,586 | 27 |
| 3161 | Switzerland | 2021 | The Swiss Conscription Database | National | both | 18-20 | | 4,384 | | |
| 3162 | Switzerland | 2021-2022 | BMI Monitoring for Switzerland - Study 1 | Community | urban | 5-11 | 5-11 | 5,458 | 5,291 | 27 |
| 3163 | Switzerland | 2022 | The Swiss Conscription Database | National | both | 18-20 | | 4,732 | | |
| 3164 | Syrian Arab Republic | 2002 | National Survey on non-communicable diseases and factors affecting their development | National | both | 15-64 | 15-64 | 3,155 | 4,045 | |
| 3165 | Taiwan | 1985 | INTERSALT | Community | rural | 20-59 | 20-59 | 89 | 92 | |
| 3166 | Taiwan | 1989-1991 | Chiu et al., J Gerontol A Biol Sci Med Sci 55(11):M684-90, 2000 | Subnational | both | 65+ | 65+ | 1,322 | 1,308 | |
| 3167 | Taiwan | 1993-1994 | The Kinmen Neurological Disorders Survey | Community | urban | 50+ | 50+ | 672 | 593 | |
| 3168 | Taiwan | 1993-1996 | Nutrition and Health Survey in Taiwan | National | both | 5+ | 5+ | 2,959 | 3,216 | |
| 3169 | Taiwan | 1999-2000 | Nutrition and Health Survey in Taiwan | National | both | 65+ | 65+ | 1,271 | 1,202 | |
| 3170 | Taiwan | 2000 | Social Environment and Biomarkers of Aging Study | National | both | 50+ | 50+ | 590 | 433 | |
| 3171 | Taiwan | 2001-2002 | Nutrition and Health Survey in Taiwan | National | both | 6-12 | 6-12 | 1,334 | 1,139 | |
| 3172 | Taiwan | 2004-2005 | Taichung Community Health Study (TCHS) | Community | urban | 40+ | 40+ | 1,147 | 1,212 | |
| 3173 | Taiwan | 2005-2008 | Nutrition and Health Survey in Taiwan | National | both | 19+ | 19+ | 1,311 | 1,355 | |
| 3174 | Taiwan | 2006 | Social Environment and Biomarkers of Aging Study | National | both | 53+ | 53+ | 548 | 476 | |
| 3175 | Taiwan | 2007 | Taiwanese Survey on Hypertension, Hyperglycemia and Hyperlipidemia | National | both | 20+ | 20+ | 2,155 | 2,469 | |
| 3176 | Taiwan | 2010 | Nutrition and Health Survey in Taiwan | National | both | 13-15 | 13-15 | 852 | 927 | |
| 3177 | Taiwan | 2011 | Nutrition and Health Survey in Taiwan | National | both | 16-18 | 16-18 | 580 | 591 | |
| 3178 | Taiwan | 2012 | Global School-based Student Health Survey | National | both | 13-17 | 13-17 | 2,998 | 2,927 | |
| 3179 | Taiwan | 2012 | Nutrition and Health Survey in Taiwan | National | both | 7-12 | 7-12 | 510 | 499 | |
| 3180 | Taiwan | 2013-2016 | Nutrition and Health Survey in Taiwan | National | both | 5+ | 5+ | 2,556 | 2,672 | |
| 3181 | Taiwan | 2019 | Global School-based Student Health Survey | National | both | 15-17 | 15-17 | 1,701 | 1,854 | |
| 3182 | Tajikistan | 2003 | Micronutrient Status Survey | National | both | | 15-49 | | 2,044 | |
| 3183 | Tajikistan | 2012 | DHS | National | both | | 15-49 | | 8,930 | |
| 3184 | Tajikistan | 2015-2016 | Childhood Obesity Surveillance Initiative 4 | National | both | 7 | 7 | 1,438 | 1,457 | |
| 3185 | Tajikistan | 2016 | STEPS | National | both | 18-69 | 18-69 | 1,091 | 1,553 | |
| 3186 | Tajikistan | 2017 | DHS | National | both | | 15-49 | | 9,922 | |
| 3187 | Tajikistan | 2019 | Childhood Obesity Surveillance Initiative 5 | National | both | 7-8 | 7-8 | 1,809 | 1,622 | |
| 3188 | Tanzania | 1991-1992 | DHS | National | both | | 20-49 | | 4,039 | |
| 3189 | Tanzania | 1996 | DHS | National | both | | 20-49 | | 3,512 | |
| 3190 | Tanzania | 1996-1997 | Aspray et al., Trans R Soc Trop Med Hyg 94:637-44, 2000 | Community | rural | 15+ | 15+ | 251 | 324 | |
| 3191 | Tanzania | 1996-1997 | Aspray et al., Trans R Soc Trop Med Hyg 94:637-44, 2000 | Community | urban | 15+ | 15+ | 117 | 118 | |
| 3192 | Tanzania | 1998-1999 | Bovet et al., Int J Epidemiol 31(1):240-7, 2002 | Community | urban | 25-64 | 25-64 | 3,593 | 5,646 | |
| 3193 | Tanzania | 2004-2005 | DHS | National | both | | 15-49 | | 9,160 | |
| 3194 | Tanzania | 2009 | Ilembula School Study | Community | rural | 8-9 | 8-9 | 151 | 150 | |
| 3195 | Tanzania | 2010 | DHS | National | both | | 15-49 | | 9,099 | |
| 3196 | Tanzania | 2011 | STEPS | Subnational | both | 25-64 | 25-64 | 1,008 | 1,517 | |
| 3197 | Tanzania | 2012 | STEPS | National | both | 25-64 | 25-64 | 2,581 | 2,827 | |
| 3198 | Tanzania | 2014 | Dar es Salaam Urban Cohort Hypertension Study | Community | urban | 40+ | 40+ | 965 | 1,266 | |

| | Country | Study years | Survey/Study name/Citation | Level of representative-ness | Rural, urban, or both | Age range as in NCD-RisC database | | Sample size | | Notes |
|---|---|---|---|---|---|---|---|---|---|---|
| | | | | | | Male | Female | Male | Female | |
| 3199 | Tanzania | 2015-2016 | DHS | National | both | | 15-49 | | 12,036 | |
| 3200 | Tanzania | 2017 | ARISE Network Adolescent Health Study (Dodoma) | Community | rural | 10-19 | 10-19 | 471 | 585 | |
| 3201 | Thailand | 1987 | INCLEN | Community | rural | 35-65 | | 244 | | |
| 3202 | Thailand | 1989 | INCLEN | Community | rural | 35-65 | | 209 | | |
| 3203 | Thailand | 1989 | INCLEN | Community | urban | 35-65 | | 207 | | |
| 3204 | Thailand | 1991 | Thailand National Health Examination Survey I | National | both | 5+ | 5+ | 8,698 | 11,027 | |
| 3205 | Thailand | 1995 | The Fourth National Nutrition Survey of Thailand- 1995 | National | both | 20-60 | 20-60 | 1,405 | 3,631 | |
| 3206 | Thailand | 1997 | Thailand National Health Examination Survey II | National | both | 5-59 | 5-59 | 4,117 | 4,876 | |
| 3207 | Thailand | 2000 | InterASIA | National | both | 35+ | 35+ | 2,092 | 3,211 | |
| 3208 | Thailand | 2003 | Socio Fitness of Thai Children | National | rural | 6-12 | 6-12 | 1,180 | 1,023 | |
| 3209 | Thailand | 2003 | Socio Fitness of Thai Children | National | urban | 6-12 | 6-12 | 2,010 | 1,722 | |
| 3210 | Thailand | 2003 | The Fifth National Food and Nutrition Survey of Thailand | National | both | 19-74 | 19-74 | 1,960 | 3,366 | |
| 3211 | Thailand | 2004 | Thailand National Health Examination Survey III | National | both | 15+ | 15+ | 18,819 | 20,143 | |
| 3212 | Thailand | 2009 | Thailand National Health Examination Survey IV | National | both | 18+ | 18+ | 12,700 | 13,502 | |
| 3213 | Thailand | 2011 | SEANUTS | National | both | 5-12 | 5-12 | 922 | 939 | |
| 3214 | Thailand | 2013-2014 | The Metabolic Syndrome and Health Behaviours in School Children Aged 13-16 Years in Ubon Ratchathani | Community | both | 13-17 | 13-17 | 98 | 278 | |
| 3215 | Thailand | 2014 | Thailand National Health Examination Survey V (18+) | National | both | 18+ | 18+ | 6,172 | 8,571 | |
| 3216 | Thailand | 2014 | Thailand National Health Examination Survey V (10-18) | National | rural | 10+ | 10+ | 875 | 872 | |
| 3217 | Thailand | 2014 | Thailand National Health Examination Survey V (10-18) | National | urban | 10+ | 10+ | 657 | 705 | |
| 3218 | Thailand | 2015 | Global School-based Student Health Survey | National | both | 12-17 | 12-17 | 2,240 | 3,106 | |
| 3219 | Thailand | 2019-2020 | Thailand National Health Examination Survey VI | National | both | 10+ | 10+ | 10,709 | 14,154 | |
| 3220 | Thailand | 2021 | Global School-based Student Health Survey | National | both | 12-16 | 12-17 | 1,911 | 2,703 | |
| 3221 | Timor-Leste | 2009-2010 | DHS | National | both | | 15-49 | | 11,983 | |
| 3222 | Timor-Leste | 2009-2010 | Timor-Leste Eye Health Survey | Subnational | both | 40+ | 40+ | 245 | 247 | |
| 3223 | Timor-Leste | 2013 | Child measurements in Ossu and in Natarbora, Timor Leste | Subnational | rural | 5-19 | 5-19 | 245 | 239 | |
| 3224 | Timor-Leste | 2014 | STEPS | National | both | 18-69 | 18-69 | 1,048 | 1,437 | |
| 3225 | Timor-Leste | 2016 | DHS | National | both | 15-59 | 15-49 | 4,556 | 11,823 | |
| 3226 | Timor-Leste | 2018 | Child measurements in Ossu and in Natarbora, Timor Leste - Harvest season | Subnational | rural | 5-19 | 5-19 | 234 | 246 | |
| 3227 | Timor-Leste | 2018 | Child measurements in Ossu and in Natarbora, Timor Leste - Post rainy season | Subnational | rural | 5-19 | 5-19 | 97 | 132 | |
| 3228 | Togo | 1998 | DHS | National | both | | 20-49 | | 3,114 | |
| 3229 | Togo | 2010 | STEPS | National | both | 15-64 | 15-64 | 2,063 | 2,095 | |
| 3230 | Togo | 2013-2014 | DHS | National | both | | 15-49 | | 4,398 | |
| 3231 | Togo | 2014 | Impact evaluation of a cash transfer program in North Togo | Subnational | rural | | 20-65 | | 3,588 | |
| 3232 | Togo | 2021-2022 | STEPS | National | both | 18-69 | 18-69 | 1,585 | 2,165 | |
| 3233 | Tokelau | 2005 | STEPS | National | both | 15-64 | 15-64 | 270 | 296 | |
| 3234 | Tokelau | 2014 | STEPS | National | both | 18-64 | 18-64 | 261 | 276 | |
| 3235 | Tonga | 2004 | STEPS | National | both | 15-64 | 15-64 | 403 | 552 | |
| 3236 | Tonga | 2005-2007 | Pacific Obesity Prevention in Communities - Maālahi Youth Project | Subnational | rural | 11-19 | 11-19 | 1,206 | 1,445 | |
| 3237 | Tonga | 2007-2008 | Pacific Obesity Prevention in Communities - Maālahi Youth Project | Subnational | rural | 13-22 | 13-22 | 434 | 579 | |
| 3238 | Tonga | 2010 | Global School-based Student Health Survey | National | both | 13-17 | 13-17 | 926 | 1,069 | |
| 3239 | Tonga | 2011 | STEPS | National | both | 15-64 | 15-64 | 878 | 1,401 | |
| 3240 | Tonga | 2017 | Global School-based Student Health Survey | National | both | 12-17 | 12-17 | 1,254 | 1,452 | |
| 3241 | Tonga | 2017 | STEPS | National | both | 18-69 | 18-69 | 1,260 | 2,327 | |
| 3242 | Trinidad and Tobago | 1985 | INTERSALT | Community | urban | 20-59 | 20-59 | 84 | 92 | |
| 3243 | Trinidad and Tobago | 1999 | Child Health Survey | National | both | 5-9 | 5-9 | 3,060 | 3,272 | |
| 3244 | Trinidad and Tobago | 2001 | Adult Survey | National | rural | 25+ | 25+ | 198 | 267 | |
| 3245 | Trinidad and Tobago | 2003 | Child Health Survey | National | both | 5-9 | 5-9 | 1,832 | 1,974 | |
| 3246 | Trinidad and Tobago | 2003 | National Survey of Senior School Health | National | both | 15-16 | 15-16 | 828 | 1,112 | |
| 3247 | Trinidad and Tobago | 2011 | STEPS | National | both | 15-64 | 15-64 | 1,112 | 1,608 | |
| 3248 | Trinidad and Tobago | 2013-2014 | National Eye Survey | National | both | 40+ | 40+ | 1,077 | 1,396 | |
| 3249 | Tunisia | 1996-1997 | Ariana Healthy Project 1997 | Community | both | 35-65 | 35-65 | 2,645 | 2,701 | |
| 3250 | Tunisia | 1996-1997 | Tunisian National Nutrition Survey 1996-1997 | National | both | 5+ | 5+ | 2,724 | 4,125 | |
| 3251 | Tunisia | 2005 | Aounallah et al., Public Health 12(1):98, 2012 | National | both | 15-19 | 15-19 | 1,290 | 1,566 | |
| 3252 | Tunisia | 2005 | Tunisian National Survey 2005 (TAHINA) | National | both | 35-71 | 35-71 | 3,265 | 4,313 | |
| 3253 | Tunisia | 2009-2010 | ObeMaghreb | Subnational | urban | 5-49 | 5-49 | 1,841 | 1,601 | |
| 3254 | Türkiye | 1990 | Turkish Adult Risk Factor Study | National | both | 20+ | 20+ | 1,338 | 1,369 | |
| 3255 | Türkiye | 1993 | DHS | National | both | | 20-49 | | 2,294 | |

| | Country | Study years | Survey/Study name/Citation | Level of representative-ness | Rural, urban, or both | Age range as in NCD-RisC database | | Sample size | | Notes |
|---|---|---|---|---|---|---|---|---|---|---|
| | | | | | | Male | Female | Male | Female | |
| 3256 | Türkiye | 1995 | Turkish Adult Risk Factor Study | National | both | 25+ | 25+ | 855 | 878 | |
| 3257 | Türkiye | 1998 | DHS | National | both | | 20-49 | | 2,210 | |
| 3258 | Türkiye | 1998 | Turkish Adult Risk Factor Study | National | both | 28+ | 28+ | 877 | 909 | |
| 3259 | Türkiye | 1998-1999 | Erem et al., Diabetes Res Clin Pract 54(3):203-08, 2001 | Community | urban | 20+ | 20+ | 1,324 | 1,322 | |
| 3260 | Türkiye | 2000 | Manisa Demographic and Health Survey | Subnational | urban | | 15-49 | | 1,420 | |
| 3261 | Türkiye | 2000 | Turkish Adult Risk Factor Study | National | both | 30+ | 30+ | 890 | 938 | |
| 3262 | Türkiye | 2000-2002 | The Healthy Nutrition for Healthy Heart Study | National | both | 25-84 | 25-84 | 4,718 | 10,631 | |
| 3263 | Türkiye | 2001 | Yumuk et al., Diabetes Res Clin Pract 70(2):151-58, 2005 | Community | urban | 20+ | 20+ | 1,042 | 1,789 | |
| 3264 | Türkiye | 2001-2002 | Turkish Adult Risk Factor Study | National | both | 32+ | 32+ | 1,098 | 1,209 | |
| 3265 | Türkiye | 2002 | Onal et al., Blood Press 13(1):31-6, 2004 | Subnational | urban | 25+ | 25+ | 67 | 355 | |
| 3266 | Türkiye | 2003 | DHS | National | both | | 20-49 | | 2,934 | |
| 3267 | Türkiye | 2003 | Prevalence, awareness, treatment and control of hypertension in Turkey in 2003 | National | both | 18+ | 18+ | 1,988 | 2,847 | |
| 3268 | Türkiye | 2003-2004 | Turkish Adult Risk Factor Study | National | both | 34+ | 34+ | 1,097 | 1,130 | |
| 3269 | Türkiye | 2003-2005 | Prevalence of prehypertension and associated risk factors among Turkish adults: Trabzon Hypertension Study | Subnational | both | 20+ | 20+ | 2,208 | 2,601 | |
| 3270 | Türkiye | 2004 | Nationally Representative Cross-sectional Survey | National | both | 20+ | 20+ | 2,110 | 2,154 | |
| 3271 | Türkiye | 2005-2006 | Turkish Adult Risk Factor Study | National | both | 35+ | 35+ | 965 | 1,029 | |
| 3272 | Türkiye | 2007 | National Household survey | National | both | 20-85 | 20-85 | 2,263 | 1,842 | |
| 3273 | Türkiye | 2007-2008 | Turkish Adult Risk Factor Study | National | both | 37+ | 37+ | 1,048 | 1,070 | |
| 3274 | Türkiye | 2007-2009 | Balcova Heart Study | Community | urban | 30+ | 30+ | 4,274 | 8,609 | |
| 3275 | Türkiye | 2008 | DHS | National | both | | 15-49 | | 6,167 | |
| 3276 | Türkiye | 2009-2010 | Turkish Adult Risk Factor Study | National | both | 39+ | 39+ | 462 | 501 | |
| 3277 | Türkiye | 2009-2012 | Prevalence of diabetes and associated risk factors among adult population in Trabzon city | Subnational | both | 20+ | 20+ | 1,562 | 2,115 | |
| 3278 | Türkiye | 2011 | Chronic Diseases and Risk Factors Survey in Turkey | National | both | 15+ | 15+ | 8,060 | 8,923 | |
| 3279 | Türkiye | 2012-2013 | Turkish Adult Risk Factor Study | National | both | 37+ | 37+ | 1,012 | 1,087 | |
| 3280 | Türkiye | 2013 | Childhood Obesity Surveillance Initiative 3 | National | both | 7-8 | 7-8 | 2,483 | 2,475 | |
| 3281 | Türkiye | 2013 | DHS | National | both | | 15-49 | | 8,270 | |
| 3282 | Türkiye | 2014-2015 | Turkish Adult Risk Factor Study | National | both | 44+ | 44+ | 437 | 484 | |
| 3283 | Türkiye | 2015-2016 | Childhood Obesity Surveillance Initiative 4 | National | both | 6-7 | 6-7 | 5,479 | 5,336 | |
| 3284 | Türkiye | 2016-2017 | Erasmus plus KA2, Healthyland | Community | urban | 6 | 6 | 29 | 22 | |
| 3285 | Türkiye | 2017 | STEPS | National | both | 15+ | 15+ | 2,306 | 3,426 | |
| 3286 | Türkiye | 2017-2018 | Erasmus plus KA2, Healthyland | Community | urban | 6 | 6 | 50 | 50 | |
| 3287 | Türkiye | 2018 | DHS | National | both | | 15-49 | | 6,464 | |
| 3288 | Turkmenistan | 2000 | DHS | National | both | | 15-49 | | 2,084 | |
| 3289 | Turkmenistan | 2013 | STEPS | National | both | 18-64 | 18-64 | 1,879 | 2,741 | |
| 3290 | Turkmenistan | 2015-2016 | Childhood Obesity Surveillance Initiative 4 | National | both | 7-8 | 7-8 | 1,952 | 1,956 | |
| 3291 | Turkmenistan | 2018 | STEPS | National | both | 18-69 | 18-69 | 1,713 | 2,236 | |
| 3292 | Tuvalu | 2013 | Global School-based Student Health Survey | National | both | 13-17 | 13-17 | 210 | 215 | |
| 3293 | Tuvalu | 2015 | STEPS | National | both | 18-69 | 18-69 | 478 | 550 | |
| 3294 | Uganda | 1995 | DHS | National | both | | 20-49 | | 2,831 | |
| 3295 | Uganda | 2000-2001 | DHS | National | both | | 15-49 | | 5,829 | |
| 3296 | Uganda | 2006 | DHS | National | both | 15-54 | 15-49 | 2,475 | 2,538 | |
| 3297 | Uganda | 2011 | DHS | National | both | 15-54 | 15-49 | 2,361 | 2,501 | |
| 3298 | Uganda | 2011-2012 | The Prevalence and Distribution of Non-communicable Diseases and their Risk Factors in Kasese District, Uganda | Subnational | both | 25-79 | 25-79 | 277 | 221 | |
| 3299 | Uganda | 2011-2013 | Gulu Health and Demographic Surveillance Site (HDSS) | Community | rural | 5+ | 5+ | 3,938 | 4,820 | |
| 3300 | Uganda | 2012 | Prevalence, awareness and control of hypertension in Uganda | Subnational | both | 15+ | 15+ | 1,619 | 2,740 | |
| 3301 | Uganda | 2014 | STEPS | National | both | 18-69 | 18-69 | 1,560 | 2,120 | |
| 3302 | Uganda | 2014-2015 | Gulu Health and Demographic Surveillance Site (HDSS) | Community | rural | 15-24 | 15-24 | 671 | 517 | |
| 3303 | Uganda | 2016 | DHS | National | both | 15-54 | 15-54 | 5,191 | 5,415 | |
| 3304 | Uganda | 2018-2019 | Scaling up Packages of Intervention for Cardiovascular disease prevention in selected sites in Europe and Sub-Saharan Africa (SPICES) | Subnational | both | 25-70 | 25-70 | 1,740 | 2,479 | |
| 3305 | Uganda | 2023 | STEPS | National | both | 18-69 | 18-69 | 1,425 | 2,061 | 5 |
| 3306 | Ukraine | 2002 | National Micronutrient Survey | National | both | | 15-50 | | 816 | |
| 3307 | Ukraine | 2006-2007 | Physical Development of School Children Ukraine | Community | rural | 6-17 | 6-17 | 1,226 | 1,336 | |
| 3308 | Ukraine | 2007-2008 | Physical Development of School Children Ukraine | Community | urban | 6-17 | 6-17 | 1,224 | 1,126 | |
| 3309 | Ukraine | 2012 | Epidemiological aspects of obesity and systemic hypertension among school children of Western Ukraine | Community | urban | 10-17 | 10-17 | 271 | 257 | |

| | Country | Study years | Survey/Study name/Citation | Level of representative-ness | Rural, urban, or both | Age range as in NCD-RisC database | | Sample size | | Notes |
|---|---|---|---|---|---|---|---|---|---|---|
| | | | | | | Male | Female | Male | Female | |
| 3310 | Ukraine | 2013-2014 | The prevalence of underweight, overweight and obesity in children and adolescents from Ukraine | National | both | 6-18 | 6-18 | 6,596 | 7,143 | |
| 3311 | Ukraine | 2018 | Prevalence of obesity in Ukrainian children and adolescents | National | both | 7-17 | 7-17 | 4,471 | 4,667 | |
| 3312 | Ukraine | 2018-2020 | Physical development of children 3-6 years old - Kyiv | Community | urban | 5-6 | 5-6 | 407 | 334 | |
| 3313 | Ukraine | 2019 | Prevalence of obesity in Ukrainian children and adolescents | National | both | 7-17 | 7-17 | 4,245 | 4,760 | |
| 3314 | Ukraine | 2019 | STEPS | National | both | 18-69 | 18-69 | 1,569 | 2,600 | |
| 3315 | United Arab Emirates | 1989-1990 | El Mugamer et al., J Trop Med Hyg 98(6):407-15, 1995 | Community | both | 20+ | 20+ | 122 | 199 | |
| 3316 | United Arab Emirates | 1999-2000 | Emirates National Diabetes and Coronary Artery Disease Risk Factor Study | National | both | 20-80 | 20-80 | 2,822 | 3,743 | |
| 3317 | United Arab Emirates | 2000-2001 | Carter et al., J Health Popul Nutr 22(1):75-83, 2004 | Community | both | | 20-79 | | 521 | |
| 3318 | United Arab Emirates | 2005 | Global School-based Student Health Survey | National | both | 12-15 | 12-15 | 5,595 | 6,268 | |
| 3319 | United Arab Emirates | 2009 | Gulf Cooperation Council World Health Survey | National | both | 18+ | 18+ | 605 | 645 | |
| 3320 | United Arab Emirates | 2010 | Global School-based Student Health Survey | National | both | 13-17 | 13-17 | 948 | 1,257 | |
| 3321 | United Arab Emirates | 2016 | Global School-based Student Health Survey | National | both | 12-17 | 12-17 | 2,323 | 2,674 | |
| 3322 | United Arab Emirates | 2017-2018 | STEPS | National | both | 18+ | 18+ | 2,148 | 2,323 | |
| 3323 | United Kingdom | 1980 | British Cohort Study 1970 | National | both | 10 | 10 | 6,252 | 5,907 | |
| 3324 | United Kingdom | 1982 | MRC National Survey of Health and Development | National | both | 36-37 | 36-37 | 1,632 | 1,648 | |
| 3325 | United Kingdom | 1983-1984 | MONICA, Belfast | Subnational | both | 25-64 | 25-64 | 1,158 | 1,183 | |
| 3326 | United Kingdom | 1984-1986 | Scottish Heart Health Survey | Subnational | both | 40-59 | 40-59 | 4,364 | 4,465 | |
| 3327 | United Kingdom | 1985 | INTERSALT, Birmingham | Community | urban | 20-59 | 20-59 | 100 | 100 | |
| 3328 | United Kingdom | 1985 | INTERSALT, South Wales | Community | urban | 20-59 | 20-59 | 100 | 99 | |
| 3329 | United Kingdom | 1985-1986 | INTERSALT, Belfast | Community | urban | 20-59 | 20-59 | 99 | 100 | |
| 3330 | United Kingdom | 1986 | British Cohort Study 1970 | National | both | 16 | 16 | 2,773 | 2,950 | |
| 3331 | United Kingdom | 1986-1987 | Dietary and Nutritional Survey of British Adults 1986-1987 | National | both | 16-64 | 16-64 | 1,158 | 1,161 | |
| 3332 | United Kingdom | 1986-1987 | MONICA, Belfast | Subnational | both | 25-64 | 25-64 | 1,155 | 1,185 | |
| 3333 | United Kingdom | 1987-1988 | Edinburgh Artery Study | Community | urban | 54-75 | 54-75 | 808 | 783 | |
| 3334 | United Kingdom | 1989 | MRC National Survey of Health and Development | National | both | 42-44 | 42-44 | 1,617 | 1,608 | |
| 3335 | United Kingdom | 1991 | National Child Development Study (1958 British Cohort Study) | National | both | 33 | 33 | 5,426 | 5,605 | |
| 3336 | United Kingdom | 1991-1992 | Health Survey for England | National | both | 16+ | 16+ | 3,114 | 3,430 | |
| 3337 | United Kingdom | 1991-1992 | MONICA, Belfast | Subnational | both | 25-64 | 25-64 | 998 | 996 | |
| 3338 | United Kingdom | 1992 | MONICA, Glasgow | Community | urban | 25-64 | 25-64 | 696 | 775 | |
| 3339 | United Kingdom | 1992-1993 | Whickham Survey | Community | urban | 35+ | 35+ | 676 | 784 | |
| 3340 | United Kingdom | 1992-1994 | Edinburgh Artery Study | Community | urban | 60-81 | 60-81 | 580 | 582 | |
| 3341 | United Kingdom | 1993 | Health Survey for England | National | both | 16+ | 16+ | 7,461 | 8,297 | |
| 3342 | United Kingdom | 1993-1997 | EPIC Norfolk | Subnational | both | 40-79 | 40-79 | 11,574 | 13,995 | |
| 3343 | United Kingdom | 1994 | Health Survey for England | National | both | 16+ | 16+ | 6,825 | 7,939 | |
| 3344 | United Kingdom | 1994-1995 | Hertfordshire Ageing Study | Subnational | both | 63-73 | 63-73 | 411 | 304 | |
| 3345 | United Kingdom | 1994-1995 | National Diet and Nutrition Survey (NDNS) | National | both | 65+ | 65+ | 701 | 687 | |
| 3346 | United Kingdom | 1995 | Health Survey for England | National | both | 5+ | 5+ | 8,038 | 9,027 | |
| 3347 | United Kingdom | 1995 | MONICA, Glasgow | Community | urban | 25-64 | 25-64 | 855 | 958 | |
| 3348 | United Kingdom | 1995 | Scottish Health Survey (SHeS) | Subnational | both | 16-64 | 16-64 | 3,303 | 4,005 | |
| 3349 | United Kingdom | 1996 | British Cohort Study 1970 | National | both | 26 | 26 | 81 | 78 | |
| 3350 | United Kingdom | 1996 | Health Survey for England | National | both | 5+ | 5+ | 8,469 | 9,461 | |
| 3351 | United Kingdom | 1997 | Health Survey for England | National | both | 5+ | 5+ | 6,285 | 6,841 | |
| 3352 | United Kingdom | 1997 | National Diet and Nutrition Survey (NDNS) | National | both | 5-18 | 5-18 | 933 | 896 | |
| 3353 | United Kingdom | 1997-1999 | INTERMAP, West Bromwich | Community | urban | 40-59 | 40-59 | 141 | 138 | |
| 3354 | United Kingdom | 1998 | Health Survey for England | National | both | 5+ | 5+ | 7,980 | 9,047 | |
| 3355 | United Kingdom | 1998 | Scottish Health Survey (SHeS) | Subnational | both | 5-74 | 5-74 | 5,047 | 5,908 | |
| 3356 | United Kingdom | 1998-1999 | INTERMAP, Belfast | Community | urban | 40-59 | 40-59 | 125 | 97 | |
| 3357 | United Kingdom | 1998-1999 | Avon Longitudinal Study of Parents and Children | Community | both | 7 | 7 | 3,693 | 3,567 | |
| 3358 | United Kingdom | 1998-1999 | SportsLinx | Community | urban | 9-10 | 9-10 | 1,429 | 1,364 | |
| 3359 | United Kingdom | 1998-2000 | The British Regional Heart Study | National | urban | 60-79 | | 4,138 | | |
| 3360 | United Kingdom | 1999 | Health Survey for England | National | both | 5+ | 5+ | 3,880 | 4,304 | |
| 3361 | United Kingdom | 1999 | MRC National Survey of Health and Development | National | both | 53-54 | 53-54 | 1,452 | 1,496 | |
| 3362 | United Kingdom | 1999-2000 | Avon Longitudinal Study of Parents and Children | Community | both | 8 | 8 | 3,048 | 3,017 | |
| 3363 | United Kingdom | 1999-2000 | SportsLinx | Community | urban | 9-10 | 9-10 | 1,469 | 1,439 | |
| 3364 | United Kingdom | 1999-2001 | British Women's Heart and Health Study | National | both | | 60-79 | | 3,678 | 28 |
| 3365 | United Kingdom | 1999-2001 | Edinburgh Artery Study | Community | urban | 66-87 | 66-87 | 373 | 404 | |
| 3366 | United Kingdom | 1999-2004 | Hertfordshire Cohort Study | Subnational | both | 59-73 | 59-73 | 1,571 | 1,416 | |

| | Country | Study years | Survey/Study name/Citation | Level of representative-ness | Rural, urban, or both | Age range as in NCD-RisC database | | Sample size | | Notes |
|---|---|---|---|---|---|---|---|---|---|---|
| | | | | | | Male | Female | Male | Female | |
| 3367 | United Kingdom | 2000 | Health Survey for England | National | both | 5+ | 5+ | 4,073 | 4,607 | |
| 3368 | United Kingdom | 2000-2001 | Avon Longitudinal Study of Parents and Children | Community | both | 9 | 9 | 3,360 | 3,411 | |
| 3369 | United Kingdom | 2000-2001 | National Diet and Nutrition Survey (NDNS) | National | both | 19-64 | 19-64 | 807 | 973 | |
| 3370 | United Kingdom | 2000-2001 | SportsLinx | Community | urban | 9-10 | 9-10 | 1,166 | 1,154 | |
| 3371 | United Kingdom | 2001 | Health Survey for England | National | both | 5+ | 5+ | 7,463 | 8,657 | |
| 3372 | United Kingdom | 2001-2002 | Avon Longitudinal Study of Parents and Children | Community | both | 10 | 10 | 3,298 | 3,338 | |
| 3373 | United Kingdom | 2001-2002 | SportsLinx | Community | urban | 9-10 | 9-10 | 866 | 743 | |
| 3374 | United Kingdom | 2002 | Health Survey for England | National | both | 5+ | 5+ | 6,797 | 7,578 | |
| 3375 | United Kingdom | 2002-2003 | Avon Longitudinal Study of Parents and Children | Community | both | 11 | 11 | 3,132 | 3,207 | |
| 3376 | United Kingdom | 2002-2003 | SportsLinx | Community | urban | 9-10 | 9-10 | 725 | 749 | |
| 3377 | United Kingdom | 2003 | The European Male Ageing Study | Community | both | 40+ | | 394 | | |
| 3378 | United Kingdom | 2003 | Health Survey for England | National | both | 5+ | 5+ | 7,136 | 8,268 | |
| 3379 | United Kingdom | 2003 | Scottish Health Survey (SHeS) | Subnational | both | 5+ | 5+ | 3,988 | 4,687 | |
| 3380 | United Kingdom | 2003-2004 | SportsLinx | Community | urban | 9-10 | 9-10 | 1,906 | 1,931 | |
| 3381 | United Kingdom | 2003-2005 | Hertfordshire Ageing Study | Subnational | both | 72-82 | 72-82 | 171 | 119 | |
| 3382 | United Kingdom | 2004 | Health Survey for England | National | both | 5+ | 5+ | 2,975 | 3,608 | |
| 3383 | United Kingdom | 2004-2005 | Avon Longitudinal Study of Parents and Children | Community | both | 13 | 13 | 2,939 | 3,016 | |
| 3384 | United Kingdom | 2004-2005 | English Longitudinal Study of Ageing Wave 2 2004-2005 | National | both | 52+ | 52+ | 3,259 | 3,966 | |
| 3385 | United Kingdom | 2004-2005 | SportsLinx | Community | urban | 9-10 | 9-10 | 1,724 | 1,712 | |
| 3386 | United Kingdom | 2005 | Health Survey for England | National | both | 5+ | 5+ | 4,839 | 5,503 | |
| 3387 | United Kingdom | 2005-2006 | Avon Longitudinal Study of Parents and Children | Community | both | 14 | 14 | 2,699 | 2,761 | |
| 3388 | United Kingdom | 2005-2006 | SportsLinx | Community | urban | 9-10 | 9-10 | 1,455 | 1,409 | |
| 3389 | United Kingdom | 2006 | Health Survey for England | National | both | 5+ | 5+ | 8,005 | 8,912 | |
| 3390 | United Kingdom | 2006 | Millennium Cohort Study | National | both | 5-6 | 5-6 | 6,258 | 5,999 | |
| 3391 | United Kingdom | 2006-2007 | Avon Longitudinal Study of Parents and Children | Community | both | 15 | 15 | 2,289 | 2,537 | |
| 3392 | United Kingdom | 2006-2007 | National Child Measurement Programme | National | both | 5-11 | 5-11 | 380,586 | 358,276 | 29 |
| 3393 | United Kingdom | 2006-2007 | SportsLinx | Community | urban | 9-10 | 9-10 | 1,759 | 1,683 | |
| 3394 | United Kingdom | 2006-2010 | MRC National Survey of Health and Development | National | both | 60-65 | 60-65 | 1,061 | 1,156 | |
| 3395 | United Kingdom | 2007 | Health Survey for England | National | both | 5+ | 5+ | 5,354 | 5,708 | |
| 3396 | United Kingdom | 2007 | Welsh Health Survey (WHS) | Subnational | both | 5-15 | 5-15 | 782 | 751 | |
| 3397 | United Kingdom | 2007-2008 | National Child Measurement Programme | National | both | 5-11 | 5-11 | 396,795 | 374,775 | 29 |
| 3398 | United Kingdom | 2007-2008 | SportsLinx | Community | urban | 9-10 | 9-10 | 1,852 | 1,815 | |
| 3399 | United Kingdom | 2008 | The European Male Ageing Study | Community | both | 40+ | | 301 | | |
| 3400 | United Kingdom | 2008 | Health Survey for England | National | both | 5+ | 5+ | 8,317 | 9,472 | |
| 3401 | United Kingdom | 2008 | Millennium Cohort Study | National | both | 6-8 | 6-8 | 6,961 | 6,836 | |
| 3402 | United Kingdom | 2008 | Scottish Health Survey (SHeS) | Subnational | both | 5+ | 5+ | 2,970 | 3,533 | |
| 3403 | United Kingdom | 2008 | Welsh Health Survey (WHS) | Subnational | both | 5-15 | 5-15 | 737 | 624 | |
| 3404 | United Kingdom | 2008-2009 | English Longitudinal Study of Ageing Wave 4 2008-2009 | National | both | 50+ | 50+ | 3,540 | 4,296 | |
| 3405 | United Kingdom | 2008-2009 | National Child Measurement Programme | National | both | 5-11 | 5-11 | 398,496 | 377,146 | 29 |
| 3406 | United Kingdom | 2008-2009 | SportsLinx | Community | urban | 9-10 | 9-10 | 1,824 | 1,854 | |
| 3407 | United Kingdom | 2008-2012 | National Diet and Nutrition Survey (NDNS) | National | both | 5+ | 5+ | 2,580 | 3,094 | |
| 3408 | United Kingdom | 2009 | Health Survey for England | National | both | 5+ | 5+ | 3,242 | 3,384 | |
| 3409 | United Kingdom | 2009 | Scottish Health Survey (SHeS) | Subnational | both | 5+ | 5+ | 3,621 | 4,168 | |
| 3410 | United Kingdom | 2009 | Welsh Health Survey (WHS) | Subnational | both | 5-15 | 5-15 | 907 | 857 | |
| 3411 | United Kingdom | 2009-2010 | Avon Longitudinal Study of Parents and Children | Community | both | 18 | 18 | 1,950 | 2,486 | |
| 3412 | United Kingdom | 2009-2010 | National Child Measurement Programme | National | both | 5-11 | 5-11 | 398,272 | 377,960 | 29 |
| 3413 | United Kingdom | 2009-2010 | SportsLinx | Community | urban | 9-10 | 9-10 | 1,493 | 1,429 | |
| 3414 | United Kingdom | 2010 | Health Survey for England | National | both | 5+ | 5+ | 4,959 | 5,547 | |
| 3415 | United Kingdom | 2010 | Scottish Health Survey (SHeS) | Subnational | both | 5+ | 5+ | 3,206 | 3,767 | |
| 3416 | United Kingdom | 2010 | Understanding Society: the UK Household Longitudinal Study | National | both | 16+ | 16+ | 6,507 | 8,422 | |
| 3417 | United Kingdom | 2010 | Welsh Health Survey (WHS) | Subnational | both | 5-15 | 5-15 | 892 | 859 | |
| 3418 | United Kingdom | 2010-2011 | National Child Measurement Programme | National | both | 5-11 | 5-11 | 393,146 | 373,472 | 29 |
| 3419 | United Kingdom | 2010-2011 | SportsLinx | Community | urban | 9-10 | 9-10 | 1,332 | 1,252 | |
| 3420 | United Kingdom | 2010-2012 | The British Regional Heart Study | National | urban | 72-91 | | 1,676 | | |
| 3421 | United Kingdom | 2011 | British Household Panel Survey | National | both | 16+ | 16+ | 1,917 | 2,391 | |
| 3422 | United Kingdom | 2011 | Health Survey for England | National | both | 5+ | 5+ | 3,680 | 4,404 | |
| 3423 | United Kingdom | 2011 | Scottish Health Survey (SHeS) | Subnational | both | 5+ | 5+ | 3,253 | 3,862 | |

| | Country | Study years | Survey/Study name/Citation | Level of representative-ness | Rural, urban, or both | Age range as in NCD-RisC database | | Sample size | | Notes |
|---|---|---|---|---|---|---|---|---|---|---|
| | | | | | | Male | Female | Male | Female | |
| 3424 | United Kingdom | 2011 | Welsh Health Survey (WHS) | Subnational | both | 5-15 | 5-15 | 949 | 836 | |
| 3425 | United Kingdom | 2011-2012 | National Child Measurement Programme | National | both | 5-11 | 5-11 | 390,383 | 373,067 | 29 |
| 3426 | United Kingdom | 2011-2012 | SportsLinx | Community | urban | 9-10 | 9-10 | 1,286 | 1,330 | |
| 3427 | United Kingdom | 2011-2013 | International Study of Childhood Obesity, Lifestyle and the Environment (ISCOLE) | Community | urban | 9-11 | 9-11 | 237 | 287 | |
| 3428 | United Kingdom | 2012 | Health Survey for England | National | both | 5+ | 5+ | 3,648 | 4,278 | |
| 3429 | United Kingdom | 2012 | Millennium Cohort Study | National | both | 10-12 | 10-12 | 6,574 | 6,451 | |
| 3430 | United Kingdom | 2012 | Scottish Health Survey (SHeS) | Subnational | both | 5+ | 5+ | 2,378 | 2,734 | |
| 3431 | United Kingdom | 2012 | Welsh Health Survey (WHS) | Subnational | both | 5-15 | 5-15 | 804 | 712 | |
| 3432 | United Kingdom | 2012-2013 | English Longitudinal Study of Ageing Wave 6 2012-2013 | National | both | 50+ | 50+ | 3,257 | 4,015 | |
| 3433 | United Kingdom | 2012-2013 | National Child Measurement Programme | National | both | 5-11 | 5-11 | 396,700 | 377,109 | 29 |
| 3434 | United Kingdom | 2013 | Health Survey for England | National | both | 5+ | 5+ | 3,910 | 4,577 | |
| 3435 | United Kingdom | 2013 | Scottish Health Survey (SHeS) | Subnational | both | 5+ | 5+ | 2,340 | 2,747 | |
| 3436 | United Kingdom | 2013-2014 | National Child Measurement Programme | National | both | 5-11 | 5-11 | 407,631 | 389,421 | 29 |
| 3437 | United Kingdom | 2013-2014 | National Diet and Nutrition Survey (NDNS) | National | both | 5+ | 5+ | 940 | 1,194 | |
| 3438 | United Kingdom | 2013-2014 | Swan-Linx Project | Community | both | 9-11 | 9-11 | 329 | 333 | |
| 3439 | United Kingdom | 2014 | Health Survey for England | National | both | 5+ | 5+ | 3,712 | 4,332 | |
| 3440 | United Kingdom | 2014 | Scottish Health Survey (SHeS) | Subnational | both | 5+ | 5+ | 2,248 | 2,675 | |
| 3441 | United Kingdom | 2014-2015 | National Child Measurement Programme | National | both | 5-11 | 5-11 | 412,685 | 395,006 | 29 |
| 3442 | United Kingdom | 2015 | Health Survey for England | National | both | 5+ | 5+ | 4,837 | 5,491 | |
| 3443 | United Kingdom | 2015 | Millennium Cohort Study | National | both | 13-15 | 13-15 | 5,635 | 5,474 | |
| 3444 | United Kingdom | 2015 | MRC National Survey of Health and Development | National | both | 69-70 | 69-70 | 1,040 | 1,082 | |
| 3445 | United Kingdom | 2015 | Scottish Health Survey (SHeS) | Subnational | both | 5+ | 5+ | 2,264 | 2,530 | |
| 3446 | United Kingdom | 2015 | Swan-Linx Project | Community | both | 9-11 | 9-11 | 420 | 395 | |
| 3447 | United Kingdom | 2015-2016 | National Child Measurement Programme | National | both | 5-11 | 5-11 | 427,039 | 410,085 | 29 |
| 3448 | United Kingdom | 2015-2016 | National Diet and Nutrition Survey (NDNS) | National | both | 5+ | 5+ | 1,021 | 1,188 | |
| 3449 | United Kingdom | 2015-2018 | British Cohort Study 1970 | National | both | 45-48 | 45-48 | 3,587 | 3,826 | |
| 3450 | United Kingdom | 2016 | Health Survey for England | National | both | 18+ | 18+ | 2,851 | 3,489 | |
| 3451 | United Kingdom | 2016 | Scottish Health Survey (SHeS) | Subnational | both | 5+ | 5+ | 2,041 | 2,395 | |
| 3452 | United Kingdom | 2016 | Swan-Linx Project | Community | both | 9-11 | 9-11 | 604 | 653 | |
| 3453 | United Kingdom | 2016-2017 | National Child Measurement Programme | National | both | 5-11 | 5-11 | 430,101 | 412,604 | 29 |
| 3454 | United Kingdom | 2016-2019 | National Diet and Nutrition Survey (NDNS) | National | both | 5+ | 5+ | 1,322 | 1,588 | |
| 3455 | United Kingdom | 2017 | Health Survey for England | National | both | 18+ | 18+ | 2,841 | 3,564 | |
| 3456 | United Kingdom | 2017 | Scottish Health Survey | Subnational | both | 5+ | 5+ | 1,734 | 2,056 | |
| 3457 | United Kingdom | 2017-2018 | National Child Measurement Programme | National | both | 5-11 | 5-11 | 444,135 | 426,632 | 29 |
| 3458 | United Kingdom | 2018 | Health Survey for England | National | both | 5+ | 5+ | 3,581 | 4,268 | |
| 3459 | United Kingdom | 2018 | Scottish Health Survey | Subnational | both | 5+ | 5+ | 2,192 | 2,546 | |
| 3460 | United Kingdom | 2018-2019 | National Child Measurement Programme | National | both | 5-11 | 5-11 | 449,768 | 433,526 | 29 |
| 3461 | United Kingdom | 2019 | Health Survey for England | National | both | 5+ | 5+ | 3,622 | 4,212 | |
| 3462 | United Kingdom | 2019 | Scottish Health Survey | Subnational | both | 5+ | 5+ | 2,309 | 2,661 | |
| 3463 | United States of America | 1976-1980 | US NHANES II | National | both | 5-74 | 5-74 | 6,952 | 7,508 | 1 |
| 3464 | United States of America | 1979-1980 | MONICA, Stanford | Subnational | urban | 25-64 | 25-64 | 703 | 806 | 30 |
| 3465 | United States of America | 1980-1982 | The Minnesota Heart Survey | Community | both | 25-75 | 25-75 | 1,611 | 1,837 | |
| 3466 | United States of America | 1981-1982 | The Bogalusa Heart Study | Community | rural | 5-17 | 5-17 | 1,630 | 1,641 | |
| 3467 | United States of America | 1983-1985 | The Bogalusa Heart Study | Community | rural | 5-17 | 5-17 | 1,227 | 1,301 | |
| 3468 | United States of America | 1985-1986 | Coronary Artery Risk Development in Young Adults (CARDIA) | Subnational | urban | 18-30 | 18-30 | 2,321 | 2,775 | |
| 3469 | United States of America | 1985-1986 | INTERSALT, Chicago | Community | urban | 20-59 | 20-59 | 97 | 99 | |
| 3470 | United States of America | 1985-1986 | MONICA, Stanford | Subnational | urban | 25-64 | 25-64 | 713 | 848 | 30 |
| 3471 | United States of America | 1985-1987 | The Minnesota Heart Survey | Community | both | 25-75 | 25-75 | 5,220 | 2,421 | |
| 3472 | United States of America | 1986 | INTERSALT, Goodman | Community | urban | 20-59 | 20-59 | 192 | 192 | |
| 3473 | United States of America | 1987-1988 | The Bogalusa Heart Study | Community | rural | 5-17 | 5-17 | 1,646 | 1,588 | |
| 3474 | United States of America | 1987-1988 | Coronary Artery Risk Development in Young Adults (CARDIA) | Subnational | urban | 20-32 | 20-32 | 2,082 | 2,506 | |
| 3475 | United States of America | 1987-1989 | Atherosclerosis Risk in Communities Study | Subnational | both | 44-66 | 44-66 | 5,041 | 6,213 | |
| 3476 | United States of America | 1988-1994 | US NHANES III | National | both | 5+ | 5+ | 11,389 | 12,256 | |
| 3477 | United States of America | 1989-1990 | Cardiovascular Health Study | Subnational | both | 65+ | 65+ | 2,458 | 3,318 | |
| 3478 | United States of America | 1989-1990 | MONICA, Stanford | Subnational | urban | 25-64 | 25-64 | 720 | 842 | 30 |
| 3479 | United States of America | 1990-1991 | Coronary Artery Risk Development in Young Adults (CARDIA) | Subnational | urban | 23-35 | 23-35 | 1,945 | 2,382 | |
| 3480 | United States of America | 1990-1991 | Cardiovascular Health Study | Subnational | both | 65+ | 65+ | 2,070 | 2,707 | |

| | Country | Study years | Survey/Study name/Citation | Level of representative-ness | Rural, urban, or both | Age range as in NCD-RisC database | | Sample size | | Notes |
|---|---|---|---|---|---|---|---|---|---|---|
| | | | | | | Male | Female | Male | Female | |
| 3481 | United States of America | 1990-1992 | Atherosclerosis Risk in Communities Study | Subnational | both | 46-70 | 46-70 | 4,537 | 5,624 | |
| 3482 | United States of America | 1991-1992 | Cardiovascular Health Study | Subnational | both | 65+ | 65+ | 1,919 | 2,563 | |
| 3483 | United States of America | 1992-1993 | Coronary Artery Risk Development in Young Adults (CARDIA) | Subnational | urban | 25-37 | 25-37 | 1,823 | 2,163 | |
| 3484 | United States of America | 1992-1993 | Cardiovascular Health Study | Subnational | both | 65+ | 65+ | 1,985 | 2,764 | |
| 3485 | United States of America | 1992-1994 | The Bogalusa Heart Study | Community | rural | 5-17 | 5-17 | 1,525 | 1,593 | |
| 3486 | United States of America | 1993-1994 | Cardiovascular Health Study | Subnational | both | 65+ | 65+ | 1,751 | 2,471 | |
| 3487 | United States of America | 1993-1995 | Atherosclerosis Risk in Communities Study | Subnational | both | 48-73 | 48-73 | 4,000 | 5,015 | |
| 3488 | United States of America | 1993-1998 | Women's Health Initiative - Observational Study | National | both | | 50-79 | | 92,691 | |
| 3489 | United States of America | 1994-1995 | Cardiovascular Health Study | Subnational | both | 66+ | 66+ | 1,617 | 2,354 | |
| 3490 | United States of America | 1995-1996 | Coronary Artery Risk Development in Young Adults (CARDIA) | Subnational | urban | 28-40 | 28-40 | 1,739 | 2,145 | |
| 3491 | United States of America | 1995-1996 | Cardiovascular Health Study | Subnational | both | 66+ | 66+ | 1,478 | 2,194 | |
| 3492 | United States of America | 1996 | National Longitudinal Study of Adolescent Health Wave II | National | both | 11-21 | 11-21 | 2,287 | 2,459 | 31 |
| 3493 | United States of America | 1996-1997 | Cardiovascular Health Study | Subnational | both | 67+ | 67+ | 1,356 | 2,043 | |
| 3494 | United States of America | 1996-1997 | INTERMAP, Baltimore | Community | urban | 40-59 | 40-59 | 146 | 134 | |
| 3495 | United States of America | 1996-1997 | INTERMAP, Jackson | Community | urban | 40-59 | 40-59 | 132 | 134 | |
| 3496 | United States of America | 1996-1997 | INTERMAP, Pittsburgh | Community | urban | 40-59 | 40-59 | 132 | 128 | |
| 3497 | United States of America | 1996-1997 | Study of Women's Health Across the Nation | Subnational | both | | 40-55 | | 3,200 | 32 |
| 3498 | United States of America | 1996-1998 | Atherosclerosis Risk in Communities Study | Subnational | both | 50-75 | 50-75 | 3,550 | 4,485 | |
| 3499 | United States of America | 1996-1998 | INTERMAP, Minneapolis | Community | urban | 40-59 | 40-59 | 130 | 130 | |
| 3500 | United States of America | 1997-1998 | INTERMAP, Chicago | Community | urban | 40-59 | 40-59 | 156 | 159 | |
| 3501 | United States of America | 1997-1998 | INTERMAP, Corpus Christi | Community | urban | 40-59 | 40-59 | 271 | 276 | |
| 3502 | United States of America | 1997-1998 | Cardiovascular Health Study | Subnational | both | 68+ | 68+ | 1,172 | 1,801 | |
| 3503 | United States of America | 1997-1999 | Study of Women's Health Across the Nation | Subnational | both | | 40-55 | | 2,761 | 32 |
| 3504 | United States of America | 1998-1999 | Coronary Artery Risk Detection in Appalachian Communities (CARDIAC), 5th Grade | Subnational | both | 10-12 | 10-12 | 541 | 461 | |
| 3505 | United States of America | 1998-1999 | Cardiovascular Health Study | Subnational | both | 69+ | 69+ | 1,092 | 1,684 | |
| 3506 | United States of America | 1998-2000 | Study of Women's Health Across the Nation | Subnational | both | | 40-55 | | 2,596 | 32 |
| 3507 | United States of America | 1999 | Early Childhood Longitudinal Study | National | both | 5-7 | 5-7 | 2,540 | 2,495 | |
| 3508 | United States of America | 1999-2000 | US NHANES 1999-2000 | National | both | 5+ | 5+ | 3,809 | 3,791 | |
| 3509 | United States of America | 1999-2001 | Study of Women's Health Across the Nation | Subnational | both | | 40-56 | | 2,507 | 32 |
| 3510 | United States of America | 2000 | Early Childhood Longitudinal Study | National | both | 6-8 | 6-8 | 7,950 | 7,598 | |
| 3511 | United States of America | 2000-2001 | Coronary Artery Risk Detection in Appalachian Communities (CARDIAC), 5th Grade | Subnational | both | 10-12 | 10-12 | 639 | 569 | |
| 3512 | United States of America | 2000-2001 | Coronary Artery Risk Development in Young Adults (CARDIA) | Subnational | urban | 33-45 | 33-45 | 1,570 | 1,949 | |
| 3513 | United States of America | 2000-2002 | Study of Women's Health Across the Nation | Subnational | both | | 40-57 | | 2,441 | 32 |
| 3514 | United States of America | 2001 | Coronary Artery Risk Detection in Appalachian Communities (CARDIAC), 5th Grade | Subnational | both | 10-12 | 10-12 | 1,732 | 1,631 | |
| 3515 | United States of America | 2001-2002 | National Longitudinal Study of Adolescent Health Wave III | National | both | 18-28 | 18-28 | 2,139 | 2,443 | 31 |
| 3516 | United States of America | 2001-2002 | US NHANES 2001-2002 | National | both | 5+ | 5+ | 4,045 | 4,006 | |
| 3517 | United States of America | 2002 | Early Childhood Longitudinal Study | National | both | 8-9 | 8-9 | 7,043 | 6,821 | |
| 3518 | United States of America | 2002-2003 | Coronary Artery Risk Detection in Appalachian Communities (CARDIAC), 5th Grade | Subnational | both | 10-12 | 10-12 | 2,635 | 2,594 | |
| 3519 | United States of America | 2003-2004 | Coronary Artery Risk Detection in Appalachian Communities (CARDIAC), 5th Grade | Subnational | both | 10-12 | 10-12 | 4,581 | 4,061 | |
| 3520 | United States of America | 2003-2004 | US NHANES 2003-2004 | National | both | 5+ | 5+ | 3,938 | 3,838 | |
| 3521 | United States of America | 2004 | Early Childhood Longitudinal Study | National | both | 10-11 | 10-11 | 5,342 | 5,335 | |
| 3522 | United States of America | 2004 | Health and Retirement Study | National | both | 24+ | 24+ | 240 | 260 | |
| 3523 | United States of America | 2004-2005 | Coronary Artery Risk Detection in Appalachian Communities (CARDIAC), 5th Grade | Subnational | both | 10-12 | 10-12 | 4,340 | 4,277 | |
| 3524 | United States of America | 2005-2006 | Coronary Artery Risk Detection in Appalachian Communities (CARDIAC), 2nd Grade | Subnational | both | 7-9 | 7-9 | 327 | 270 | |
| 3525 | United States of America | 2005-2006 | Coronary Artery Risk Detection in Appalachian Communities (CARDIAC), 5th Grade | Subnational | both | 10-12 | 10-12 | 4,859 | 4,205 | |
| 3526 | United States of America | 2005-2006 | Coronary Artery Risk Development in Young Adults (CARDIA) | Subnational | urban | 38-50 | 38-50 | 1,528 | 2,000 | |
| 3527 | United States of America | 2005-2006 | Cardiovascular Health Study | Subnational | both | 70+ | 70+ | 375 | 684 | |
| 3528 | United States of America | 2005-2006 | US NHANES 2005-2006 | National | both | 5+ | 5+ | 3,984 | 3,835 | |
| 3529 | United States of America | 2005-2006 | National Social Life Health and Aging Project | National | both | 57-85 | 57-85 | 1,355 | 1,435 | 33 |
| 3530 | United States of America | 2006 | Health and Retirement Study | National | both | 53+ | 53+ | 2,809 | 3,788 | |
| 3531 | United States of America | 2006-2007 | Coronary Artery Risk Detection in Appalachian Communities (CARDIAC), 2nd Grade | Subnational | both | 7-9 | 7-9 | 843 | 798 | |
| 3532 | United States of America | 2006-2007 | Coronary Artery Risk Detection in Appalachian Communities (CARDIAC), 5th Grade | Subnational | both | 10-12 | 10-12 | 4,039 | 3,418 | |
| 3533 | United States of America | 2007 | Early Childhood Longitudinal Study | National | both | 13-14 | 13-14 | 4,256 | 4,251 | |
| 3534 | United States of America | 2007-2008 | Coronary Artery Risk Detection in Appalachian Communities (CARDIAC), 2nd Grade | Subnational | both | 7-9 | 7-9 | 3,873 | 3,975 | |
| 3535 | United States of America | 2007-2008 | Coronary Artery Risk Detection in Appalachian Communities (CARDIAC), 5th Grade | Subnational | both | 10-12 | 10-12 | 4,046 | 3,634 | |
| 3536 | United States of America | 2007-2008 | US NHANES 2007-2008 | National | both | 5+ | 5+ | 4,086 | 4,038 | |
| 3537 | United States of America | 2008 | Health and Retirement Study | National | both | 55+ | 55+ | 2,469 | 3,448 | |

| | Country | Study years | Survey/Study name/Citation | Level of representative-ness | Rural, urban, or both | Age range as in NCD-RisC database | | Sample size | | Notes |
|---|---|---|---|---|---|---|---|---|---|---|
| | | | | | | Male | Female | Male | Female | |
| 3538 | United States of America | 2008-2009 | Coronary Artery Risk Detection in Appalachian Communities (CARDIAC), 2nd Grade | Subnational | both | 7-9 | 7-9 | 4,907 | 5,178 | |
| 3539 | United States of America | 2008-2009 | Coronary Artery Risk Detection in Appalachian Communities (CARDIAC), 5th Grade | Subnational | both | 10-12 | 10-12 | 4,152 | 3,593 | |
| 3540 | United States of America | 2008-2009 | National Longitudinal Study of Adolescent Health Wave IV | National | both | 24-34 | 24-34 | 2,317 | 2,725 | 31 |
| 3541 | United States of America | 2009-2010 | Coronary Artery Risk Detection in Appalachian Communities (CARDIAC), 2nd Grade | Subnational | both | 7-9 | 7-9 | 5,217 | 5,562 | |
| 3542 | United States of America | 2009-2010 | Coronary Artery Risk Detection in Appalachian Communities (CARDIAC), 5th Grade | Subnational | both | 10-12 | 10-12 | 3,990 | 3,513 | |
| 3543 | United States of America | 2009-2010 | US NHANES 2009-2010 | National | both | 5+ | 5+ | 4,291 | 4,332 | |
| 3544 | United States of America | 2010 | Early Childhood Longitudinal Study | National | both | 5-6 | 5-6 | 7,811 | 7,391 | |
| 3545 | United States of America | 2010-2011 | Coronary Artery Risk Detection in Appalachian Communities (CARDIAC), 2nd Grade | Subnational | both | 7-9 | 7-9 | 4,994 | 5,286 | |
| 3546 | United States of America | 2010-2011 | Coronary Artery Risk Detection in Appalachian Communities (CARDIAC), 5th Grade | Subnational | both | 10-12 | 10-12 | 3,313 | 2,819 | |
| 3547 | United States of America | 2010-2011 | Coronary Artery Risk Development in Young Adults (CARDIA) | Subnational | urban | 43-55 | 43-55 | 1,513 | 1,976 | |
| 3548 | United States of America | 2010-2011 | Health and Retirement Study | National | both | 57+ | 57+ | 2,533 | 3,465 | |
| 3549 | United States of America | 2010-2011 | National Social Life Health and Aging Project | National | both | 61-91 | 61-91 | 1,440 | 1,640 | 33 |
| 3550 | United States of America | 2011 | Early Childhood Longitudinal Study - Fall | National | both | 5-7 | 5-7 | 2,722 | 2,493 | |
| 3551 | United States of America | 2011 | Early Childhood Longitudinal Study - Spring | National | both | 5-7 | 5-7 | 8,729 | 8,373 | |
| 3552 | United States of America | 2011-2012 | Coronary Artery Risk Detection in Appalachian Communities (CARDIAC), 2nd Grade | Subnational | both | 7-9 | 7-9 | 4,122 | 4,412 | |
| 3553 | United States of America | 2011-2012 | Coronary Artery Risk Detection in Appalachian Communities (CARDIAC), 5th Grade | Subnational | both | 10-12 | 10-12 | 2,644 | 2,112 | |
| 3554 | United States of America | 2011-2012 | US NHANES 2011-2012 | National | both | 5+ | 5+ | 3,951 | 3,887 | |
| 3555 | United States of America | 2011-2013 | Atherosclerosis Risk in Communities Study | Subnational | both | 67-90 | 67-90 | 1,787 | 2,431 | |
| 3556 | United States of America | 2011-2013 | International Study of Childhood Obesity, Lifestyle and the Environment (ISCOLE) | Community | urban | 9-11 | 9-11 | 281 | 368 | |
| 3557 | United States of America | 2012 | Early Childhood Longitudinal Study - Fall | National | both | 6-8 | 6-8 | 2,446 | 2,262 | |
| 3558 | United States of America | 2012 | Early Childhood Longitudinal Study - Spring | National | both | 6-8 | 6-8 | 7,667 | 7,358 | |
| 3559 | United States of America | 2012 | Health and Retirement Study | National | both | 59+ | 59+ | 2,294 | 3,095 | |
| 3560 | United States of America | 2012-2013 | Coronary Artery Risk Detection in Appalachian Communities (CARDIAC), 2nd Grade | Subnational | both | 7-9 | 7-9 | 4,565 | 5,184 | |
| 3561 | United States of America | 2012-2013 | Coronary Artery Risk Detection in Appalachian Communities (CARDIAC), 5th Grade | Subnational | both | 10-12 | 10-12 | 2,796 | 2,332 | |
| 3562 | United States of America | 2013 | Early Childhood Longitudinal Study | National | both | 7-9 | 7-9 | 6,975 | 6,705 | |
| 3563 | United States of America | 2013-2014 | Coronary Artery Risk Detection in Appalachian Communities (CARDIAC), 2nd Grade | Subnational | both | 7-9 | 7-9 | 4,994 | 5,415 | |
| 3564 | United States of America | 2013-2014 | Coronary Artery Risk Detection in Appalachian Communities (CARDIAC), 5th Grade | Subnational | both | 10-12 | 10-12 | 2,408 | 1,987 | |
| 3565 | United States of America | 2013-2014 | US NHANES 2013-2014 | National | both | 5+ | 5+ | 4,105 | 4,225 | |
| 3566 | United States of America | 2014 | Early Childhood Longitudinal Study | National | both | 8-10 | 8-10 | 6,458 | 6,158 | |
| 3567 | United States of America | 2014 | Health and Retirement Study | National | both | 61+ | 61+ | 2,152 | 2,972 | |
| 3568 | United States of America | 2014-2015 | Coronary Artery Risk Detection in Appalachian Communities (CARDIAC), 2nd Grade | Subnational | both | 7-9 | 7-9 | 5,182 | 5,495 | |
| 3569 | United States of America | 2014-2015 | Coronary Artery Risk Detection in Appalachian Communities (CARDIAC), 5th Grade | Subnational | both | 10-12 | 10-12 | 2,286 | 1,889 | |
| 3570 | United States of America | 2015 | Early Childhood Longitudinal Study | National | both | 9-11 | 9-11 | 5,954 | 5,679 | |
| 3571 | United States of America | 2015-2016 | US NHANES 2015-2016 | National | both | 5+ | 5+ | 3,959 | 4,091 | |
| 3572 | United States of America | 2015-2016 | National Social Life Health and Aging Project | Community | both | 57-96 | 57-96 | 1,577 | 1,895 | 34 |
| 3573 | United States of America | 2016 | Early Childhood Longitudinal Study | National | both | 10-12 | 10-12 | 5,625 | 5,337 | |
| 3574 | United States of America | 2016-2018 | Health and Retirement Study | National | both | 63+ | 63+ | 1,638 | 2,321 | |
| 3575 | United States of America | 2017-2018 | US NHANES 2017-2018 | National | both | 5+ | 5+ | 3,642 | 3,784 | |
| 3576 | United States of America | 2018-2019 | Health and Retirement Study | National | both | 65+ | 65+ | 1,424 | 2,034 | |
| 3577 | United States of America | 2019-2020 | US NHANES 2019-2020 | Subnational | both | 5+ | 5+ | 2,396 | 2,354 | 35 |
| 3578 | Uruguay | 1999-2000 | The Survey on Health, Well-Being, and Aging in Latin America and the Caribbean (SABE) | Community | urban | 60+ | 60+ | 492 | 828 | 3 |
| 3579 | Uruguay | 2004 | CUiiDARTE Project | National | urban | 5-8 | 5-8 | 127 | 149 | |
| 3580 | Uruguay | 2005 | CUiiDARTE Project | National | urban | 6-10 | 6-10 | 105 | 105 | |
| 3581 | Uruguay | 2006 | STEPS | National | urban | 25-64 | 25-64 | 261 | 641 | |
| 3582 | Uruguay | 2009-2010 | CUiiDARTE Project | National | urban | 10-14 | 10-14 | 117 | 129 | |
| 3583 | Uruguay | 2011-2012 | CESCAS Study | Community | urban | 35-74 | 35-74 | 640 | 900 | |
| 3584 | Uruguay | 2012 | Global School-based Student Health Survey | National | both | | 13-15 | | 1,377 | |
| 3585 | Uruguay | 2012-2016 | Genotype, Phenotype and Environment of Hypertension in Uruguay (GEFA-HT-UY) | Community | urban | 19+ | 19+ | 124 | 189 | |
| 3586 | Uruguay | 2013 | STEPS | National | urban | 15-64 | 15-64 | 821 | 1,400 | |
| 3587 | Uruguay | 2015 | CUiiDARTE Project | National | urban | 17-20 | 17-20 | 135 | 142 | |
| 3588 | Uruguay | 2016-2017 | CUiiDARTE Project | Community | urban | 5-6 | 5-6 | 395 | 375 | |
| 3589 | Uruguay | 2018 | Evaluation of the School Feeding Program and monitoring of the nutritional status of children in public and private schools in Uruguay | National | both | 5-11 | 5-11 | 1,447 | 1,538 | |
| 3590 | Uruguay | 2019 | Global School-based Student Health Survey | National | both | 13-16 | 13-16 | 901 | 1,022 | |
| 3591 | Uzbekistan | 1996 | DHS | National | both | | 15-49 | | 4,082 | |
| 3592 | Uzbekistan | 2002 | DHS | National | both | 15-59 | 15-49 | 2,331 | 5,275 | |
| 3593 | Uzbekistan | 2014 | STEPS | National | both | 18-64 | 18-64 | 1,533 | 2,164 | |

| | Country | Study years | Survey/Study name/Citation | Level of representative-ness | Rural, urban, or both | Age range as in NCD-RisC database | | Sample size | | Notes |
|---|---|---|---|---|---|---|---|---|---|---|
| | | | | | | Male | Female | Male | Female | |
| 3594 | Uzbekistan | 2015-2016 | Epidemiology of Diabetes and Prediabetes in Uzbekistan Screening Results | Subnational | both | 35+ | 35+ | 714 | 1,511 | |
| 3595 | Uzbekistan | 2019 | STEPS | National | both | 18-69 | 18-69 | 1,462 | 2,226 | |
| 3596 | Vanuatu | 1996 | Second National Nutrition Survey | National | both | | 15-50 | | 1,353 | |
| 3597 | Vanuatu | 1998 | Vanuatu Non-comunicable Disease Survey | National | both | 20-60 | 20-60 | 533 | 730 | |
| 3598 | Vanuatu | 2005 | STEPS | Subnational | both | 15-60 | 15-60 | 626 | 759 | |
| 3599 | Vanuatu | 2011 | STEPS | National | both | 25-64 | 25-64 | 2,251 | 2,183 | |
| 3600 | Venezuela | 1998-2001 | Maracaibo aging study Santa lucia cohort | Community | urban | 55-100 | 55-100 | 760 | 1,526 | |
| 3601 | Venezuela | 1999-2001 | Florez et al., Diabetes Res Clin Pract 69(1):63-77, 2005 | Subnational | both | 15+ | 15+ | 1,134 | 2,599 | |
| 3602 | Venezuela | 2000 | Diaz et al., Invest Clin 46(2):111-19, 2005 | Community | urban | 60+ | 60+ | 42 | 59 | |
| 3603 | Venezuela | 2004-2005 | CArdiovascular Risk factors Multiple Evaluation in Latin America (CARMELA) | Community | urban | 25-64 | 25-64 | 713 | 1,123 | |
| 3604 | Venezuela | 2005-2006 | Brajkovich et al., Rev Ven Endoc Metab 4(3):31-32, 2006 | Community | urban | 20-65 | 20-65 | 205 | 439 | |
| 3605 | Venezuela | 2007-2008 | Venezuelan Study of Metabolic Syndrome, Obesity and Lifestyle (VEMSOLS) | Community | urban | 20+ | 20+ | 107 | 230 | |
| 3606 | Venezuela | 2008-2009 | Venezuelan Study of Metabolic Syndrome, Obesity and Lifestyle (VEMSOLS) | Community | rural | 20+ | 20+ | 51 | 89 | |
| 3607 | Venezuela | 2010-2011 | Cardiometabolic risk factors in schoolchildren and adolescents of Mérida, Venezuela (CREDEFAR) | Community | urban | 9-18 | 9-18 | 443 | 475 | |
| 3608 | Venezuela | 2010-2011 | Venezuelan Study of Metabolic Syndrome, Obesity and Lifestyle (VEMSOLS) | Community | urban | 20+ | 20+ | 66 | 193 | |
| 3609 | Venezuela | 2014-2015 | Latin American Study of Nutrition and Health (ELANS) | National | urban | 15-65 | 15-65 | 552 | 580 | |
| 3610 | Venezuela | 2014-2017 | Maracaibo aging study Santa Rosa cohort | Community | urban | 37-100 | 37-100 | 115 | 292 | |
| 3611 | Venezuela | 2015-2017 | Cardio-Metabolic Health Venezuelan Study (EVESCAM) | National | both | 20+ | 20+ | 1,056 | 2,346 | |
| 3612 | Venezuela | 2018-2020 | Cardio-metabolic Health Venezuelan Study (EVESCAM) follow-up | National | both | 22+ | 22+ | 355 | 892 | |
| 3613 | Viet Nam | 1981-1985 | National Nutrition Survey | Subnational | rural | 18+ | 18+ | 4,815 | 7,985 | |
| 3614 | Viet Nam | 1987-1989 | General Nutrition Survey | National | both | 15-70 | 15-70 | 13,776 | 17,271 | |
| 3615 | Viet Nam | 1992-1993 | Living Standard Survey | National | both | 5+ | 5+ | 9,418 | 10,209 | |
| 3616 | Viet Nam | 1997-1998 | Living Standard Survey | National | both | 5+ | 5+ | 12,052 | 13,118 | |
| 3617 | Viet Nam | 2000 | National Nutrition Survey | National | both | 20+ | 20+ | 8,985 | 9,464 | |
| 3618 | Viet Nam | 2001-2002 | Viet Nam National Health Survey 2001-2002 | National | both | 5+ | 5+ | 66,723 | 71,616 | |
| 3619 | Viet Nam | 2001-2003 | The National Epidemiological Survey on Hypertension and Its Risk Factors (North) | Subnational | both | 25-74 | 25-74 | 2,386 | 3,604 | |
| 3620 | Viet Nam | 2003-2004 | The Survey on Heart Failure and Its Risk Factors | Subnational | both | 25-74 | 25-74 | 1,853 | 2,636 | |
| 3621 | Viet Nam | 2004 | Cuong et al., Eur J Clin Nutr 61(5):673-81, 2007 | Community | urban | 20-60 | 20-60 | 717 | 771 | |
| 3622 | Viet Nam | 2004 | The Hypertension Management Programme in Rural Communes (Hanoi) | Community | rural | 25-74 | 25-74 | 855 | 1,288 | |
| 3623 | Viet Nam | 2005 | STEPS Bavi district | Subnational | rural | 25-64 | 25-64 | 987 | 997 | |
| 3624 | Viet Nam | 2005 | National Adult Overweight Survey | National | both | 25-64 | 25-64 | 8,474 | 8,725 | |
| 3625 | Viet Nam | 2005 | The Survey on Non-Communicable Disease Risk Factors | Subnational | both | 25-74 | 25-74 | 1,136 | 1,220 | |
| 3626 | Viet Nam | 2005 | Non-communicable disease risk factors in Ho Chi Minh City | Community | urban | 25-64 | 25-64 | 908 | 1,063 | |
| 3627 | Viet Nam | 2006 | The Hypertension Management Programme in Rural Communes (Bavi) | Community | rural | 25-74 | 25-74 | 395 | 643 | |
| 3628 | Viet Nam | 2006 | Qualitative and quantitative assessment of nutritional status and lifestyles of Vietnamese adolescents | Subnational | rural | 15-17 | 15-17 | 252 | 363 | |
| 3629 | Viet Nam | 2006 | Qualitative and quantitative assessment of nutritional status and lifestyles of Vietnamese adolescents | Subnational | urban | 15-17 | 15-17 | 254 | 334 | |
| 3630 | Viet Nam | 2006-2008 | The National Epidemiological Survey on Hypertension and Its Risk Factors (South) | Subnational | both | 25-74 | 25-74 | 1,310 | 2,078 | |
| 3631 | Viet Nam | 2007 | The Hypertension Management Programme in Rural Communes (Phu Phuong) | Community | rural | 25-74 | 25-74 | 364 | 616 | |
| 3632 | Viet Nam | 2008-2009 | The Survey on Diabetes and Its Risk Factors | Subnational | both | 25+ | 25+ | 830 | 1,446 | |
| 3633 | Viet Nam | 2009 | The Hypertension Management Programme in Rural Communes (Phu Cuong) | Community | rural | 25-74 | 25-74 | 362 | 677 | |
| 3634 | Viet Nam | 2009 | STEPS | National | both | 25-64 | 25-64 | 6,703 | 7,776 | |
| 3635 | Viet Nam | 2009-2010 | Vietnam National Nutrition Survey 2009-2010 | National | both | 5+ | 5+ | 16,036 | 16,619 | |
| 3636 | Viet Nam | 2011 | SEANUTS | National | both | 5-11 | 5-11 | 975 | 980 | |
| 3637 | Viet Nam | 2012 | National Survey of Diabetes in Vietnam | National | both | 30-69 | 30-69 | 5,319 | 5,855 | |
| 3638 | Viet Nam | 2013 | Global School-based Student Health Survey | National | both | 13-17 | 13-17 | 1,368 | 1,578 | |
| 3639 | Viet Nam | 2015 | STEPS | National | both | 18-69 | 18-69 | 1,316 | 1,722 | |
| 3640 | Viet Nam | 2019 | Global School-based Student Health Survey | National | both | 13-18 | 13-18 | 3,572 | 4,117 | |
| 3641 | Viet Nam | 2021 | STEPS | National | both | 18+ | 18+ | 1,837 | 1,886 | |
| 3642 | Yemen | 1997 | DHS | National | both | | 15-49 | | 5,123 | |
| 3643 | Yemen | 2005-2006 | Yemen Household Budget Survey 2005-2006 | National | both | 5+ | 5+ | 3,290 | 3,307 | |
| 3644 | Yemen | 2007-2009 | Hypertension and Diabetes in Yemen (HYDY) | National | rural | 6-70 | 6-70 | 3,023 | 3,065 | |
| 3645 | Yemen | 2007-2009 | Hypertension and Diabetes in Yemen (HYDY) | National | urban | 6-70 | 6-70 | 2,996 | 3,077 | |
| 3646 | Yemen | 2013 | DHS | National | both | | 15-49 | | 22,527 | |
| 3647 | Zambia | 1992 | DHS | National | both | | 20-49 | | 2,829 | |
| 3648 | Zambia | 1996 | DHS | National | both | | 20-49 | | 3,485 | |
| 3649 | Zambia | 2001-2002 | DHS | National | both | | 15-49 | | 6,732 | |
| 3650 | Zambia | 2003 | Kelly et al., Am J Clin Nut 88(4):1010-17, 2008 | Community | urban | 15-74 | 15-84 | 132 | 217 | |

| | Country | Study years | Survey/Study name/Citation | Level of representative-ness | Rural, urban, or both | Age range as in NCD-RisC database | | Sample size | | Notes |
|---|---|---|---|---|---|---|---|---|---|---|
| | | | | | | Male | Female | Male | Female | |
| 3651 | Zambia | 2007 | DHS | National | both | | 15-49 | | 6,378 | |
| 3652 | Zambia | 2008 | STEPS | Subnational | urban | 25+ | 25+ | 626 | 1,214 | |
| 3653 | Zambia | 2013-2014 | DHS | National | both | | 15-49 | | 14,837 | |
| 3654 | Zambia | 2017 | STEPS | National | both | 18-69 | 18-69 | 1,565 | 2,439 | |
| 3655 | Zimbabwe | 1985-1986 | INTERSALT | Community | urban | 20-59 | 20-59 | 100 | 95 | |
| 3656 | Zimbabwe | 1991 | Zinyowera et al., Cent Afr J Med 40(2):33-8, 1994 | Community | both | 18+ | 18+ | 775 | 734 | |
| 3657 | Zimbabwe | 1994 | DHS | National | both | | 20-49 | | 1,776 | |
| 3658 | Zimbabwe | 1995 | Mufunda et al., J Hum Hypertens 14(1):65-73, 2000 | Community | urban | 25+ | 25+ | 384 | 391 | |
| 3659 | Zimbabwe | 1999 | DHS | National | both | | 15-49 | | 5,169 | |
| 3660 | Zimbabwe | 2005 | STEPS | National | both | 25+ | 25+ | 569 | 1,808 | |
| 3661 | Zimbabwe | 2005-2006 | DHS | National | both | | 15-49 | | 8,186 | |
| 3662 | Zimbabwe | 2010-2011 | DHS | National | both | 15-54 | 15-49 | 7,383 | 8,329 | |
| 3663 | Zimbabwe | 2015 | DHS | National | both | 15-54 | 15-49 | 8,386 | 9,396 | |

1. National studies for the 3 years prior to 1980 were assigned to 1980 so that they inform the estimates in countries with slightly earlier national data.
2. This research uses data from Australia Health Survey (AHS). We thank the Health Section, Australian Bureau of Statistics, Belconnen, ACT, Australia for support for AHS 2011-2012.
3. The bibliographic citation for this data source is: Pelaez, Martha, Alberto Palloni, Cecilia Albala, Juan C. Alfonso, Roberto Ham-Chande, Anselm Hennis, Maria Lucia Lebrao, Esther Lesn-Diaz, Edith Pantelides, and Omar Prats. SABE - SURVEY ON HEALTH, WELL-BEING, AND AGING IN LATIN AMERICA AND THE CARIBBEAN, 2000 [Computer file]. ICPSR version. Washington, D.C.: Pan American Health Organization/World Health Organization (PAHO/WHO) [producers], 2004. Ann Arbor, MI: Inter-university Consortium for Political and Social Research [distributor], 2005.
4. Sciensano, OD Public health and surveillance (2020). Health Interview Survey 2018 [Data file and code book]. Conditionally obtainable from the Sciensano website: https://www.sciensano.be/en/node/55737/health-interview-survey-microdata-request-procedure.
5. National studies from 2023 were assigned to 2022 so that they inform the estimates in countries with slightly later national data.
6. This research uses data from China Health and Nutrition Survey (CHNS). We thank the National Institute for Health (NIH), the Eunice Kennedy Shriver National Institute of Child Health and Human Development (NICHD) for R01 HD30880, National Institute on Aging (NIA) for R01 AG065357, National Institute of Diabetes and Digestive and Kidney Diseases (NIDDK) for R01 DK104371 and P30 DK056350, National Heart, Lung, and Blood Institute (NHLBI) for R01 HL108427, the NIH Fogarty grant D43 TW009077, the Carolina Population Center for P2C HD050924 and P30 AG066615. We also thank the National Institute for Nutrition and Health, China Center for Disease Control and Prevention; Beijing Municipal Center for Disease Control and Prevention; the Chinese National Human Genome Center at Shanghai; and the China-Japan Friendship Hospital, National Health Commission of China.
7. The bibliographic citations for this data source are: Zeng, Yi, Vaupel, James, Xiao, Zhenyu, Liu, Yuzhi, and Zhang, Chunyuan. Chinese Longitudinal Healthy Longevity Survey (CLHLS), 1998-2014. Inter-university Consortium for Political and Social Research [distributor], 2017-04-11. https://doi.org/10.3886/ICPSR36692.v1; Center for Healthy Aging and Development Studies, 2020, "The Chinese Longitudinal Healthy Longevity Survey (CLHLS)-Longitudinal Data (1998-2018)", https://doi.org/10.18170/DVN/WBO7LK, Peking University Open Research Data Platform, V2.
8. Santé publique France, en tant qu'investigateur principal, promoteur et financeur de l'étude ENNS.; Aux Centres d'examen de santé de la Caisse nationale d'assurance maladie des travailleurs salariés (CnamTS) et leurs laboratoires.
9. Santé publique France, en tant qu'investigateur principal, promoteur et financeur de l'étude Esteban.; Aux Centres d'examen de santé de la Caisse nationale d'assurance maladie des travailleurs salariés (CnamTS) et leurs laboratoires.
10. Data have been provided by the Study of Health in Pomerania (SHIP) from the University Medicine Greifswald.
11. The authors thank the Heinz Nixdorf Foundation [Chairman: Martin Nixdorf; Past Chairman: Dr jur. Gerhard Schmidt], for their generous support of this study. Parts of the study were also supported by the German Research Council (DFG) [DFG project: EI 969/2-3, ER 155/6-1;6-2, HO 3314/2-1;2-2;2-3;4-3, INST 58219/32-1, JO 170/8-1, KN 885/3-1, PE 2309/2-1, SI 236/8-1;9-1;10-1,], the German Ministry of Education and Science [BMBF project: 01EG0401, 01GI0856, 01GI0860, 01GS0820_WB2-C, 01ER1001D, 01GI0205], the Ministry of Innovation, Science, Research and Technology, North Rhine-Westphalia (MIWFT-NRW), the Else Kröner-Fresenius-Stiftung [project: 2015_A119] and the German Social Accident Insurance [DGUV project: FF-FP295]. Furthermore the study was supported by the Competence Network for HIV/AIDS, the deanship of the University Hospital and IFORES of the University Duisburg-Essen, the European Union, the German Competence Network Heart Failure, Kulturstiftung Essen, the Protein Research Unit within Europe (PURE), the Dr. Werner-Jackstädt Stiftung and the following companies: Celgene GmbH München, Imatron/GE-Imatron, Janssen, Merck KG, Philips, ResMed Foundation, Roche Diagnostics, Sarstedt AG&Co, Siemens HealthCare Diagnostics, Volkswagen Foundation. The authors express their gratitude to all study participants of the Heinz Nixdorf Recall (HNR) Study, the personnel of the HNR study center and the EBT-scanner facilities, the investigative group and all former employees of the HNR study. The authors also thank the Advisory Board of the HNR Study: T. Meinertz, Hamburg, Germany (Chair); C. Bode, Freiburg, Germany; P.J. de Feyter, Rotterdam, Netherlands; B. Güntert, Hall i.T., Austria; F. Gutzwiller, Bern, Switzerland; H. Heinen, Bonn, Germany; O. Hess (†), Bern, Switzerland; B. Klein (†), Essen, Germany; H. Löwel, Neuherberg, Germany; M. Reiser, Munich, Germany; G. Schmidt (†), Essen, Germany; M. Schwaiger, Munich, Germany; C. Steinmüller, Bonn, Germany; T. Theorell, Stockholm, Sweden; and S.N Willich, Berlin, Germany.
12. The bibliographic citation for this data source is: Desai, Sonalde, Vanneman, Reeve, and National Council of Applied Economic Research, New Delhi. India Human Development Survey (IHDS), 2005. Inter-university Consortium for Political and Social Research [distributor], 2018-08-08. https://doi.org/10.3886/ICPSR22626.v12.
13. The CARRS Study was funded in part by the National Heart, Lung, and Blood Institute (NHLBI), National Institutes of Health (NIH), Department of Health and Human Services, under Contract No. HHSN268200900026C, the United Health Group, Minneapolis, MN, USA, and by the by the National Heart, Lung, And Blood Institute of the National Institutes of Health under Award Number P01HL154996.
14. The bibliographic citation for this data source is: Desai, Sonalde, Reeve Vanneman and National Council of Applied Economic Research. India Human Development Survey-II (IHDS-II), 2011-12. Inter-university Consortium for Political and Social Research [distributor], 2018-08-08. https://doi.org/10.3886/ICPSR36151.v6.
15. Accessed via the Irish Social Science Data Archive - www.ucd.ie/issda.
16. The Older Persons in Jamaica Study was funded by the National Health Fund, Jamaica.
17. The study was supported by the grant of the Ministry of Healthcare of the Republic of Kazakhstan National Programme for the Introduction of Personalized and Preventive Medicine in The Republic of Kazakhstan (2021-2023) (Grant number OR12165486).
18. The MHAS Cognitive Aging Ancillary Study (Mex-Cog) is sponsored by the National Institutes of Health/National Institute on Aging (NIH R01AG051158). Data files and documentation are public use and available at www.MHASweb.org.
19. The Longitudinal Aging Study Amsterdam is supported by a grant from the Netherlands Ministry of Health Welfare and Sports, Directorate of Long-Term Care.

20. The data collection [in 2012-2013 and 2013-2014] was financially supported by the Netherlands Organization for Scientific Research (NWO) in the framework of the project New Cohorts of young old in the 21st century (file number 480-10-014).
21. The bibliographic citation for this data source is: Am J Hypertens 2009 Jan;22(1):100-5 and Atherosclerosis. 2009 Mar;203(1):257-62.
22. The bibliographic citation for this data source is: Palloni, Alberto, Ana Luisa Davila, and Melba Sanchez-Ayendez. Puerto Rican Elderly: Health Conditions (PREHCO) Project, 2002-2003, 2006-2007. ICPSR34596-v1. Ann Arbor, MI: Inter-university Consortium for Political and Social Research[distributor], 2013-09-13. doi:10.3886/ICPSR34596.v1.
23. Dr Take Naseri (Ministry of Health, Samoa), and Muagututia Sefuiva Reupena (Lutia I Puava Ae Mapu I Fagalele) contributed to the GWAS studies in Samoa.
24. The SH2012 and SH2 studies are supported by infrastructure funding from the Singapore Ministry of Health (Population Health Metrics Population Health Metrics and Analytics PHMA), National University of Singapore and National University Health System, Singapore.
25. Data used for this research was provided by the INMA - INfancia y Medio Ambiente [Environment and Childhood] Project (www.proyectoinma.org), which is supported in part by funds. This study was funded by grants from Instituto de Salud Carlos III (Red INMA G03/176 and CB06/02/0041), Spanish Ministry of Health (FIS-97/1102, FIS-07/0252, FIS-PS09/00362, 97/0588, 00/0021-2, PI061756, PS0901958, PI14/00677 incl.FEDER funds, FIS-PS09/00090, PI041436, FIS-PI042018, FIS-PI06/0867, PI081151 incl. FEDER funds, FIS-PI09/02311 and FIS-PI13/02187, and FIS-FEDER 03/1615, 04/1509, 04/1112, 04/1931, 05/1079, 05/1052, 06/1213, 07/0314, 09/02647, 11/01007, 11/0178, 11/02591, 11/02038, PI12/01890 incl. FEDER funds, 13/1944, 13/2032, FIS-PI13/02429, 14/0891, 14/1687 and CP13/00054 incl. FEDER funds), Spanish Ministry of Economy and Competitiveness (SAF2012-32991 incl. FEDER funds), CIBERESP, Generalitat de Catalunya-CIRIT 1999SGR 00241, Generalitat de Catalunya-AGAUR (2009 SGR 501, 2014 SGR 822), the Conselleria de Sanitat Generalitat Valenciana, Department of Health of the Basque Government (2005111093, 2009111069 and 2013111089), the Provincial Government of Gipuzkoa (DFG06/004 and DFG08/001), Fundació La Caixa (97/009-00 and 00/077-00), Beca de la IV convocatoria de Ayudas a la Investigación en Enfermedades Neurodegenerativas de La Caixa, Fundació La marató de TV3 (090430), Obra Social Cajastur/Fundación Liberbank, Universidad de Oviedo, the EU Commission (QLK4-1999-01422, QLK4-CT-2000-00263, QLK4-2002-00603, CONTAMED FP7-ENV-212502, FP7-ENV-2011 cod 282957, HEALTH.2010.2.4.5-1, 261357, 308333, 603794), Agence Nationale de Securite Sanitaire de l'Alimentation de l'Environnement et du Travail (1262C0010), Consejería de Salud de la Junta de Andalucía (grant number 183/07) and Annual agreements with municipalities in the study area (Zumarraga, Urretxu, Legazpi, Azkoitia y Azpeitia y Beasain).
26. The ULSAM study was supported by Uppsala University and Uppsala University Hospital.
27. The Swiss BMI Monitoring Study was supported by the Health Service of the town of Berne, Basel Health Service for Children and Adolescents, and the School-Medical Service of the town of Zurich.
28. The British Women's Heart and Health Study is supported by the British Heart Foundation (PG/13/66/30442). British Women's Heart and Health Study data are available to bona fide researchers for research purposes. Please refer to the BWHHS data sharing policy at http://www.ucl.ac.uk/british-womens-heart-health-study.
29. NHS Digital was the data source for National Child Measurement Programme data.
30. Prof Stephen Fortmann contributed data from the Stanford Five-City Project.
31. This research uses data from Add Health, a program project designed by J. Richard Udry, Peter S. Bearman, and Kathleen Mullan Harris, and funded by a grant P01-HD31921 from the Eunice Kennedy Shriver National Institute of Child Health and Human Development, with cooperative funding from 17 other agencies. Special acknowledgment is due Ronald R. Rindfuss and Barbara Entwisle for assistance in the original design. Persons interested in obtaining data files from Add Health should contact Add Health, Carolina Population Center, 123 W. Franklin Street, Chapel Hill, NC 27516-2524 (addhealth@unc.edu). No direct support was received from grant P01-HD31921 for this analysis.
32. The bibliographic citation for this data source is: Sutton-Tyrrell, Kim, Faith Selzer, MaryFran Sowers, Robert Neer, Lynda Powell, Ellen Gold, Gail Greendale, Gerson Weiss, Karen Matthews, and Sonja McKinlay. Study of Women's Health Across the Nation (SWAN), 1996-1997: Baseline Dataset. ICPSR28762-v2. Ann Arbor, MI: Inter-university Consortium for Political and Social Research[distributor], 2014-02-04. http://doi.org/10.3886/ICPSR28762.v2.
33. The bibliographic citation for this data source is: Waite, Linda J., Kathleen Cagney, William Dale, Elbert Huang, Edward O. Laumann, Martha McClintock, Colm A. O'Muircheartaigh, L. Phillip Schumm, and Benjamin Cornwell. National Social Life, Health, and Aging Project (NSHAP): Wave 2 and Partner Data Collection. ICPSR34921-v1. Ann Arbor, MI: Inter-university Consortium for Political and Social Research [distributor], 2014-04-29. https://doi.org/10.3886/ICPSR34921.v1.
34. The bibliographic citation for this data source is: Waite, Linda J., Cagney, Kathleen A., Dale, William, Hawkley, Louise C., Huang, Elbert S., Lauderdale, Diane S., . Schumm, L. Philip. National Social Life, Health, and Aging Project (NSHAP): Round 3 and COVID-19 Study, [United States], 2015-2016, 2020-2021. Inter-university Consortium for Political and Social Research [distributor], 2021-12-13. https://doi.org/10.3886/ICPSR36873.v5.
35. Due the COVID-19 pandemic the NHANES 2019-2020 cycle was not completed. As a result the data are not nationally representative and considered subnational.

**Appendix Table 2.** List of analysis regions and “super-regions”, and countries in each region.

| Super-region | Region |
|---|---|
| **Central and eastern Europe** | **Central Europe**: Albania, Bosnia and Herzegovina, Bulgaria, Croatia, Czechia, Hungary, Montenegro, North Macedonia, Poland, Romania, Serbia, Slovakia, Slovenia |
| | **Eastern Europe**: Belarus, Estonia, Latvia, Lithuania, Moldova, Russian Federation, Ukraine |
| **Central Asia, Middle East and north Africa** | **Central Asia**: Armenia, Azerbaijan, Georgia, Kazakhstan, Kyrgyzstan, Mongolia, Tajikistan, Turkmenistan, Uzbekistan |
| | **Middle East and north Africa**: Algeria, Bahrain, Egypt, Iran, Iraq, Jordan, Kuwait, Lebanon, Libya, Morocco, Oman, Qatar, Saudi Arabia, State of Palestine, Syrian Arab Republic, Tunisia, Türkiye, United Arab Emirates, Yemen |
| **High-income western** | **High-income English-speaking countries***: Australia, Canada, Ireland, New Zealand, United Kingdom, United States of America |
| | **Northwestern Europe**: Austria, Belgium, Denmark, Finland, Germany, Greenland, Iceland, Luxembourg, Netherlands, Norway, Sweden, Switzerland |
| | **Southwestern Europe**: Andorra, Cyprus, France, Greece, Israel, Italy, Malta, Portugal, Spain |
| **Latin America and the Caribbean** | **Andean Latin America**: Bolivia, Ecuador, Peru |
| | **The Caribbean**: Antigua and Barbuda, Bahamas, Barbados, Belize, Bermuda, Cuba, Dominica, Dominican Republic, Grenada, Guyana, Haiti, Jamaica, Puerto Rico, Saint Kitts and Nevis, Saint Lucia, Saint Vincent and the Grenadines, Suriname, Trinidad and Tobago |
| | **Central Latin America**: Colombia, Costa Rica, El Salvador, Guatemala, Honduras, Mexico, Nicaragua, Panama, Venezuela |
| | **Southern Latin America**: Argentina, Brazil, Chile, Paraguay, Uruguay |
| **Oceania** | **Melanesia:** Fiji, Papua New Guinea, Solomon Islands, Vanuatu |
| | **Polynesia and Micronesia**: American Samoa, Cook Islands, French Polynesia, Kiribati, Marshall Islands, Micronesia, Nauru, Niue, Palau, Samoa, Tokelau, Tonga, Tuvalu |
| **South Asia** | **South Asia**: Afghanistan, Bangladesh, Bhutan, India, Nepal, Pakistan, Sri Lanka |
| **East and southeast Asia and the Pacific** | **East Asia and the Pacific**: China, Japan, Singapore, South Korea, Taiwan |
| | **Southeast Asia**: Brunei Darussalam, Cambodia, Indonesia, Lao PDR, Malaysia, Maldives, Myanmar, North Korea, Philippines, Thailand, Timor-Leste, Viet Nam |
| **Sub-Saharan Africa** | **Central and southern Africa**: Angola, Botswana, Central African Republic, Congo, DR Congo, Equatorial Guinea, Gabon, Namibia |
| | **East Africa**: Burundi, Comoros, Djibouti, Eritrea, Eswatini, Ethiopia, Kenya, Lesotho, Madagascar, Malawi, Mozambique, Rwanda, Somalia, South Sudan, Sudan, Tanzania, Uganda, Zambia, Zimbabwe |
| | **West Africa**: Benin, Burkina Faso, Cabo Verde, Cameroon, Chad, Côte d'Ivoire, Gambia, Ghana, Guinea, Guinea Bissau, Liberia, Mali, Mauritania, Niger, Nigeria, Sao Tome and Principe, Senegal, Sierra Leone, Togo |
| | **Other sub-Saharan Africa:** Mauritius, Seychelles, South Africa |

* Although high-income English-speaking countries are geographically separated, they experienced similar trends in cardiometabolic risk factors and outcomes.[1-5,20,52-54] They were therefore grouped together so that the statistical model shares information amongst them more than it does with other countries that are geographically closer but epidemiologically more distinct.

**Appendix Figure 1.** Number of data sources used in this analysis, by country.

# School-aged children and adolescents

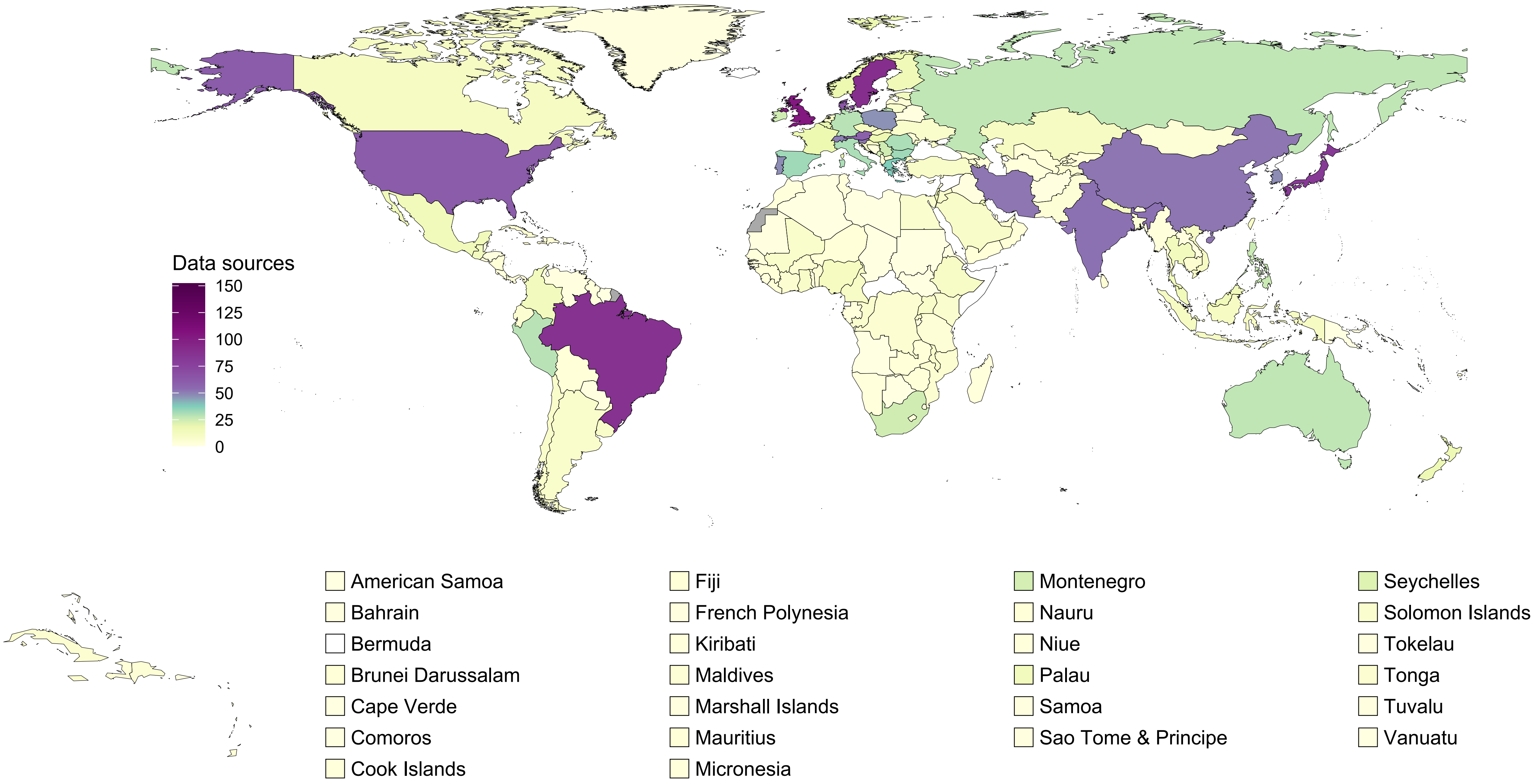


# Adults

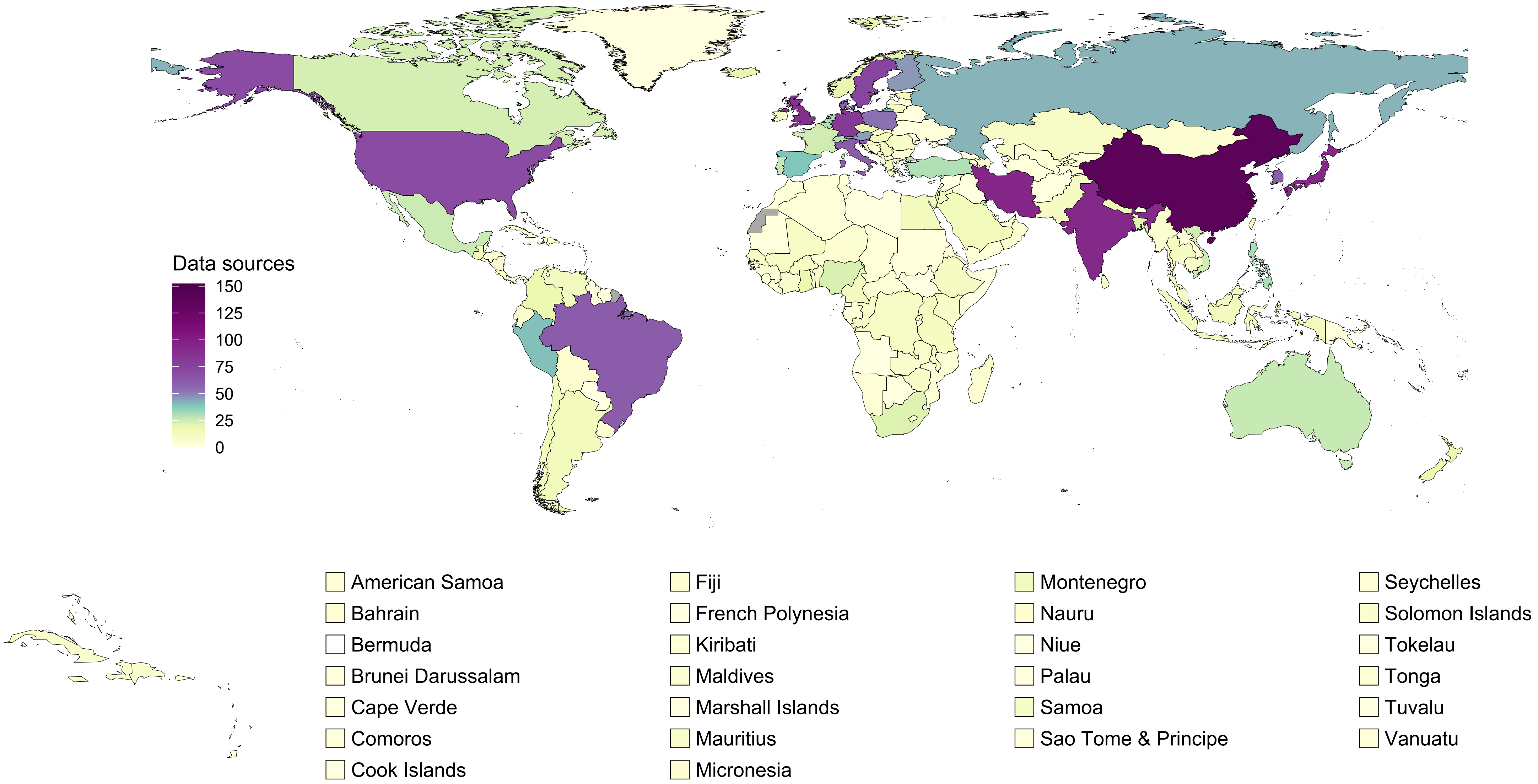

**Appendix Figure 2.** Number of data sources used in this analysis, by region and year. The size of each circle shows the number of data sources for each region and year, and the colours indicate the relative count of national, subnational and community data sources.

School-aged children and adolescents

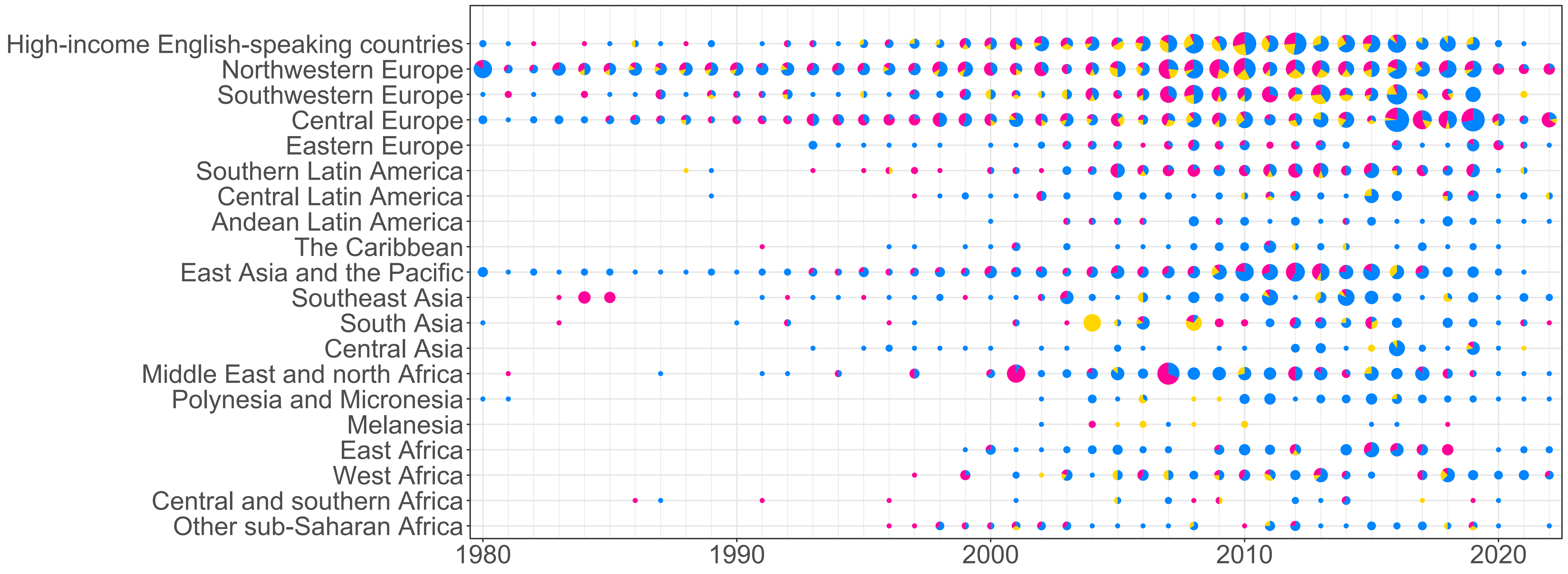


Adults

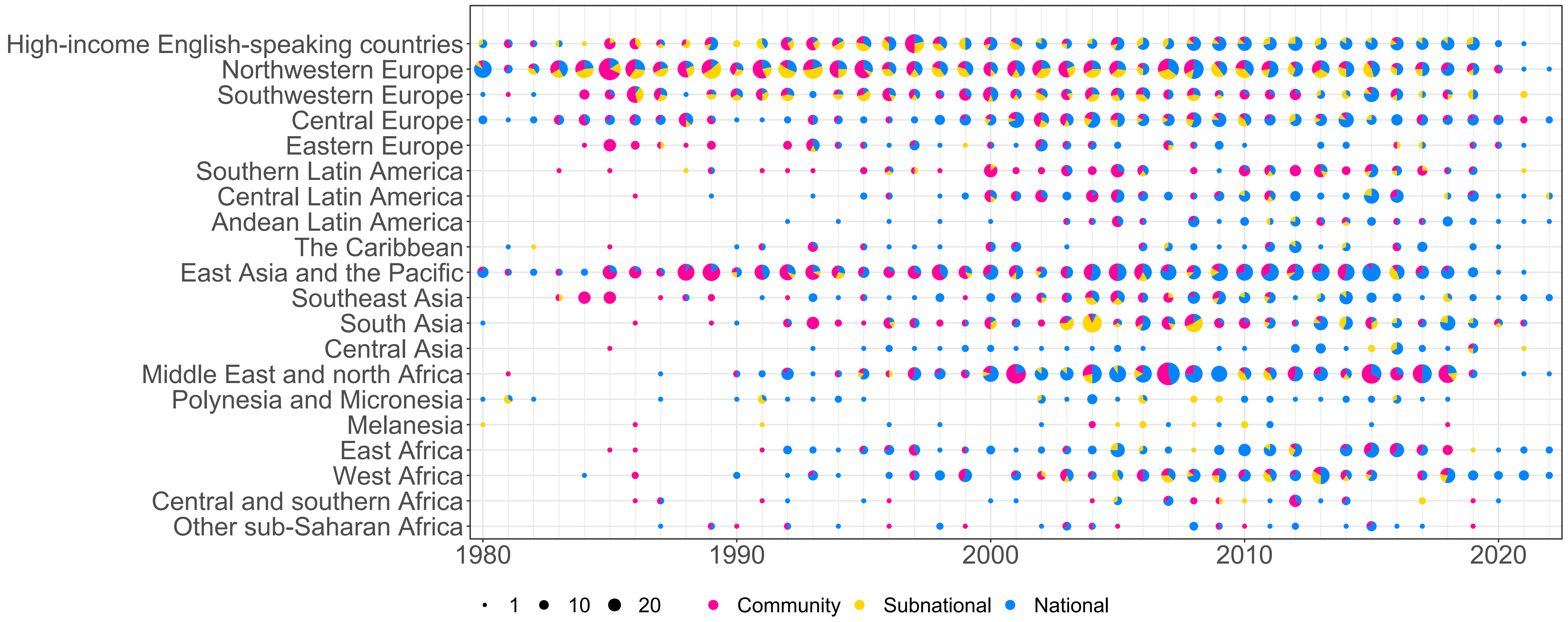

**Appendix Figure 3**. Change in age-standardised prevalence of underweight and obesity, for adults.

(A) Change in underweight from 1990 to 2022 in relation to underweight in 1990. (B) Change in obesity from 1990 to 2022 in relation to obesity in 1990. (C) Change in obesity in relation to change in underweight from 1990 to 2022. The sloped solid line intersecting the origin delineates regions where an increase (above the line) or a decrease (below) in the combined prevalence of underweight and obesity took place, and the dashed parallel lines indicate ten percentage point intervals in change of combined prevalence.

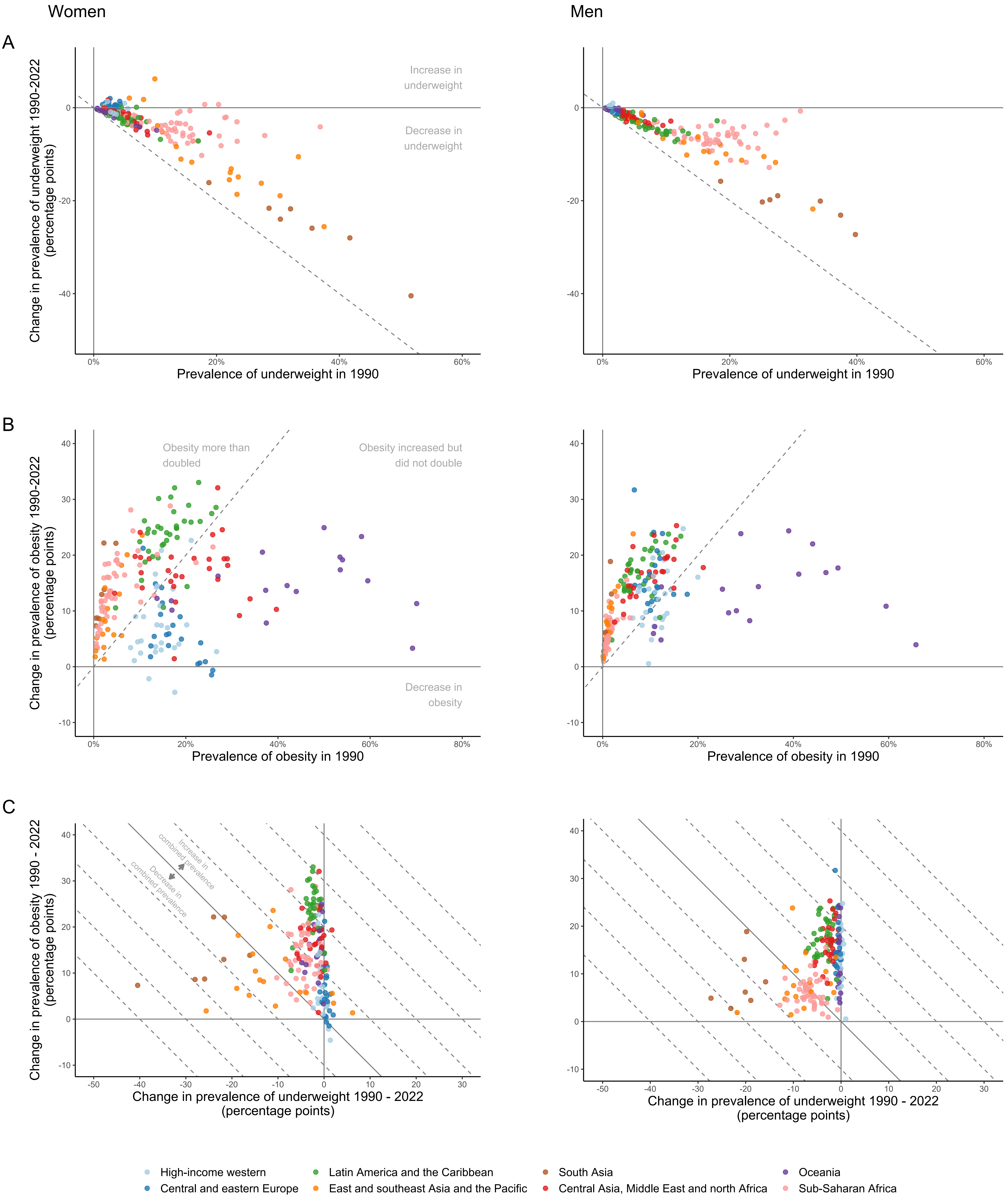

Women
Men
A
Increase in underweight
Decrease in underweight
Change in prevalence of underweight 1990-2022 (percentage points)
Prevalence of underweight in 1990
B
Obesity more than doubled
Obesity increased but did not double
Decrease in obesity
Change in prevalence of obesity 1990-2022 (percentage points)
Prevalence of obesity in 1990
C
Increase in combined prevalence
Decrease in combined prevalence
Change in prevalence of obesity 1990 - 2022 (percentage points)
Change in prevalence of underweight 1990 - 2022 (percentage points)
High-income western
Central and eastern Europe
Latin America and the Caribbean
East and southeast Asia and the Pacific
South Asia
Central Asia, Middle East and north Africa
Oceania
Sub-Saharan Africa

**Appendix Figure 4.** Age-standardised prevalence of all BMI categories in 1990 and 2022, for adults.

Country names are coloured by region. The numbers in brackets after each country's name show the total number of data sources and the number of nationally representative data sources, respectively. Countries are ordered alphabetically within each region.

Women

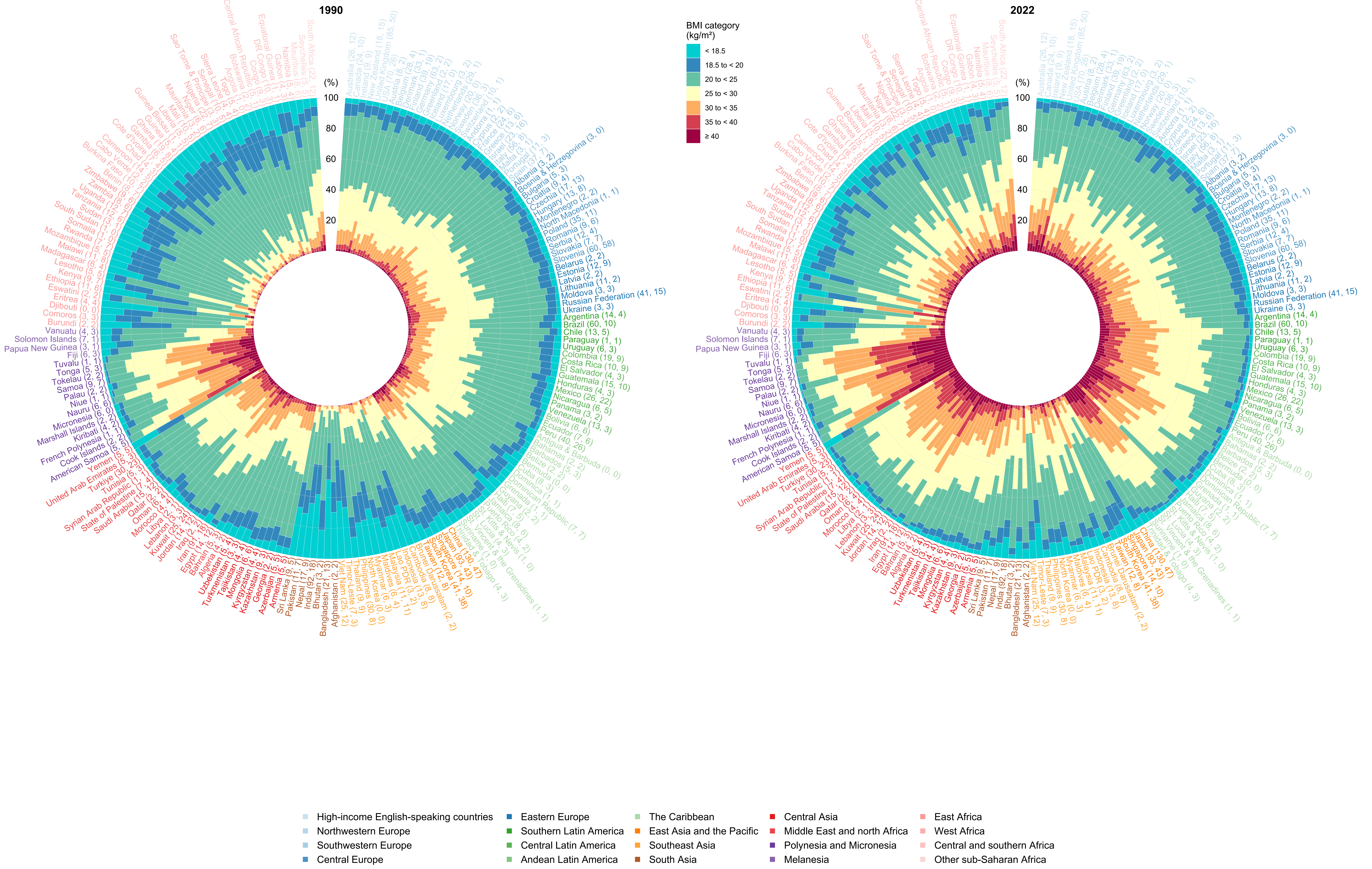
1990
2022
(%)
100
80
60
40
20
BMI category (kg/m²)
< 18.5
18.5 to < 20
20 to < 25
25 to < 30
30 to < 35
35 to < 40
≥ 40
High-income English-speaking countries
Northwestern Europe
Southwestern Europe
Central Europe
Eastern Europe
Southern Latin America
Central Latin America
Andean Latin America
The Caribbean
East Asia and the Pacific
Southeast Asia
South Asia
Central Asia
Middle East and north Africa
Polynesia and Micronesia
Melanesia
East Africa
West Africa
Central and southern Africa
Other sub-Saharan Africa

Men

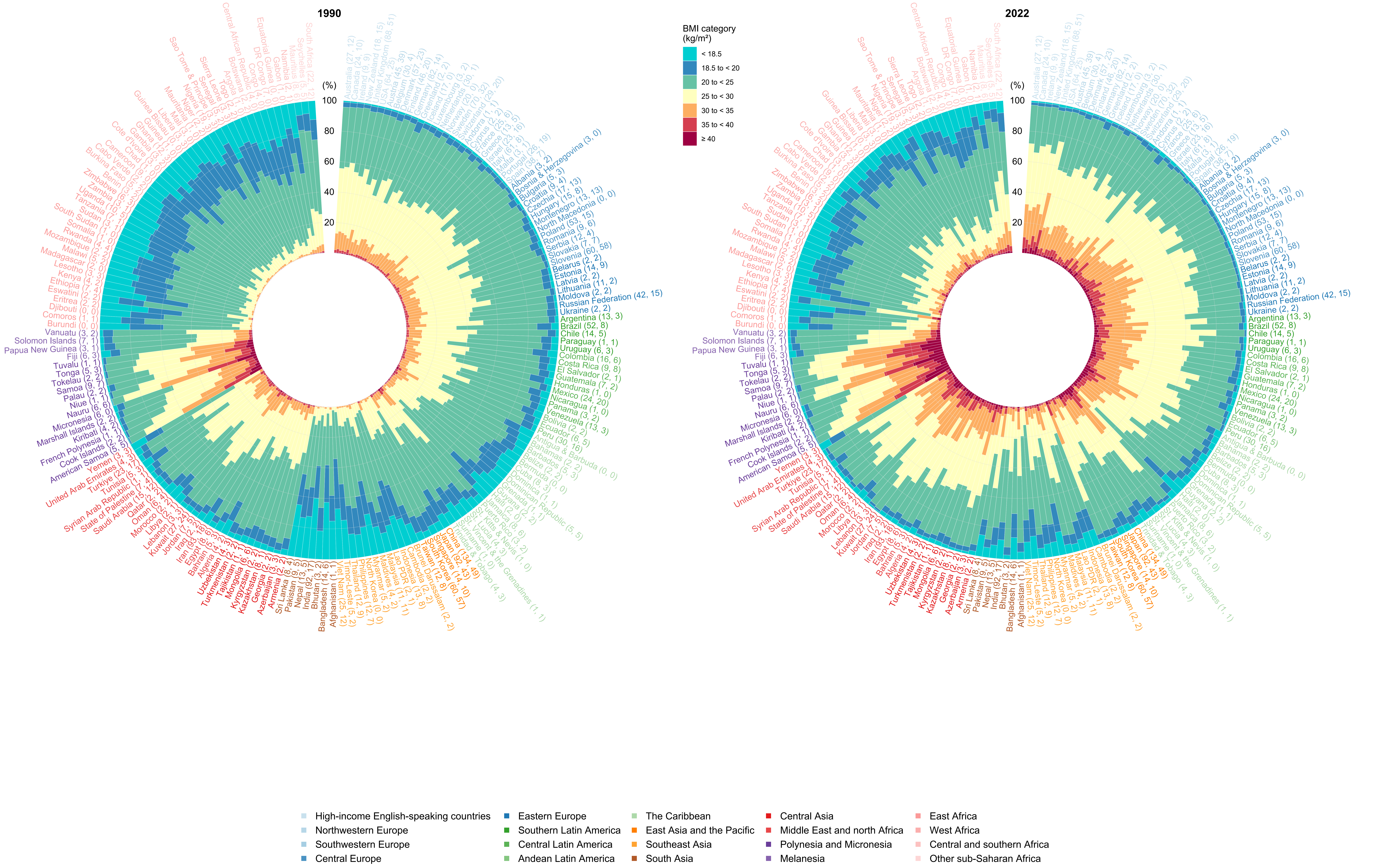
1990
2022
(%)
100
80
60
40
20
BMI category (kg/m²)
< 18.5
18.5 to < 20
20 to < 25
25 to < 30
30 to < 35
35 to < 40
≥ 40
High-income English-speaking countries
Northwestern Europe
Southwestern Europe
Central Europe
Eastern Europe
Southern Latin America
Central Latin America
Andean Latin America
The Caribbean
East Asia and the Pacific
Southeast Asia
South Asia
Central Asia
Middle East and north Africa
Polynesia and Micronesia
Melanesia
East Africa
West Africa
Central and southern Africa
Other sub-Saharan Africa

**Appendix Figure 5.** Age-standardised prevalence of underweight in 1990 and 2022, its change from 1990 to 2022, and posterior probability that the prevalence increased from 1990 to 2022, for adults.

# Women

1990

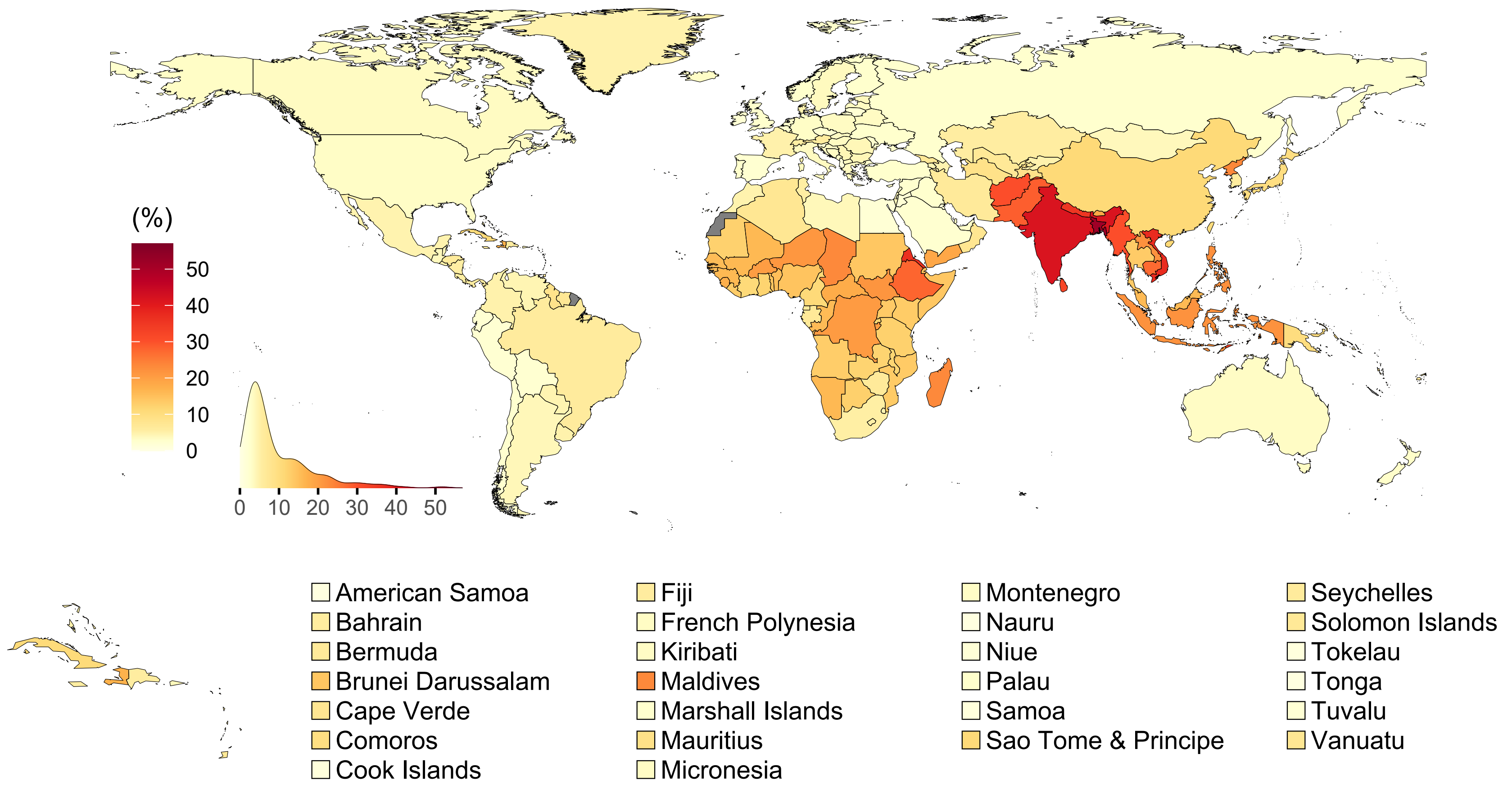


Change from 1990 to 2022

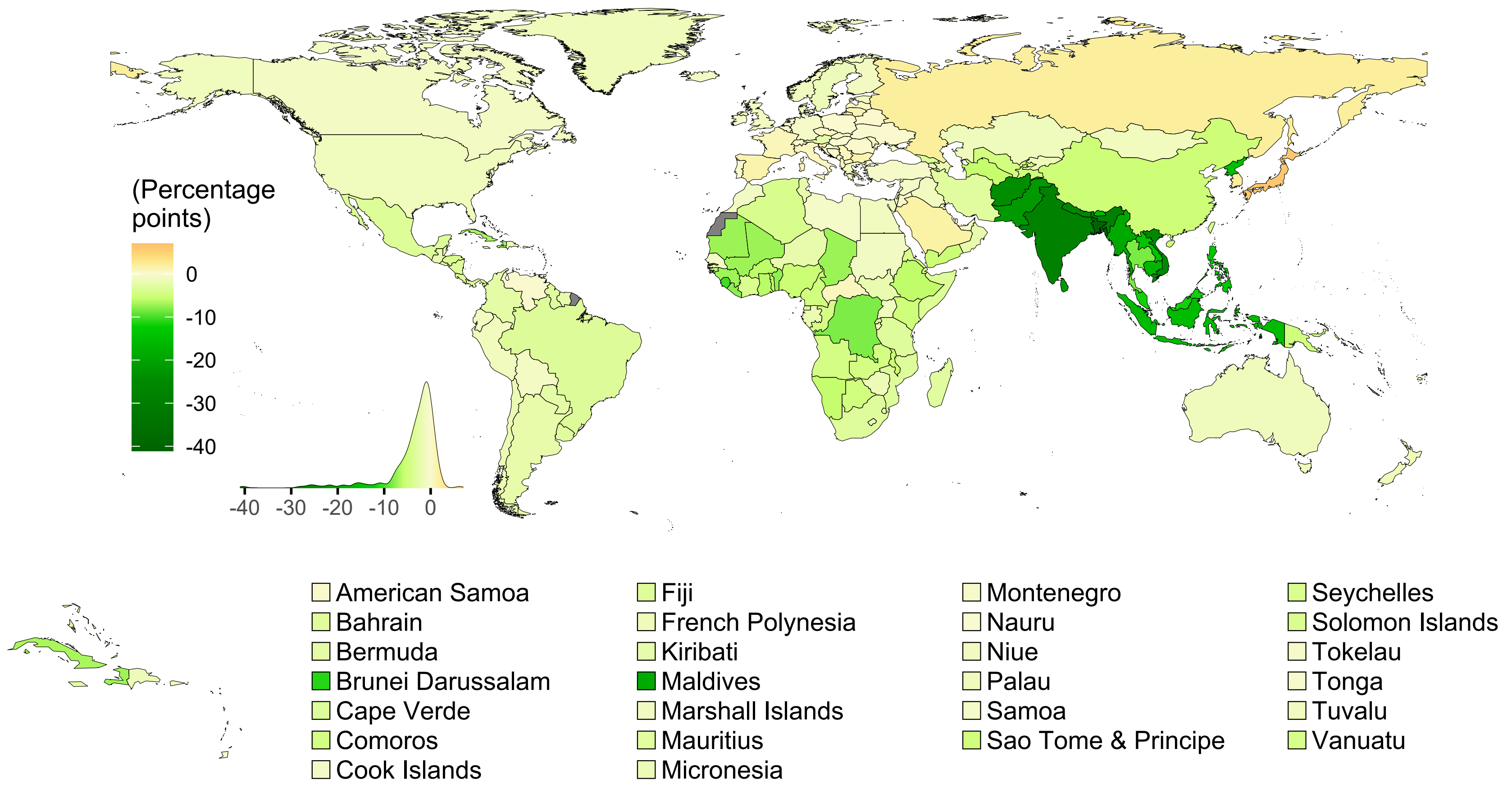


2022

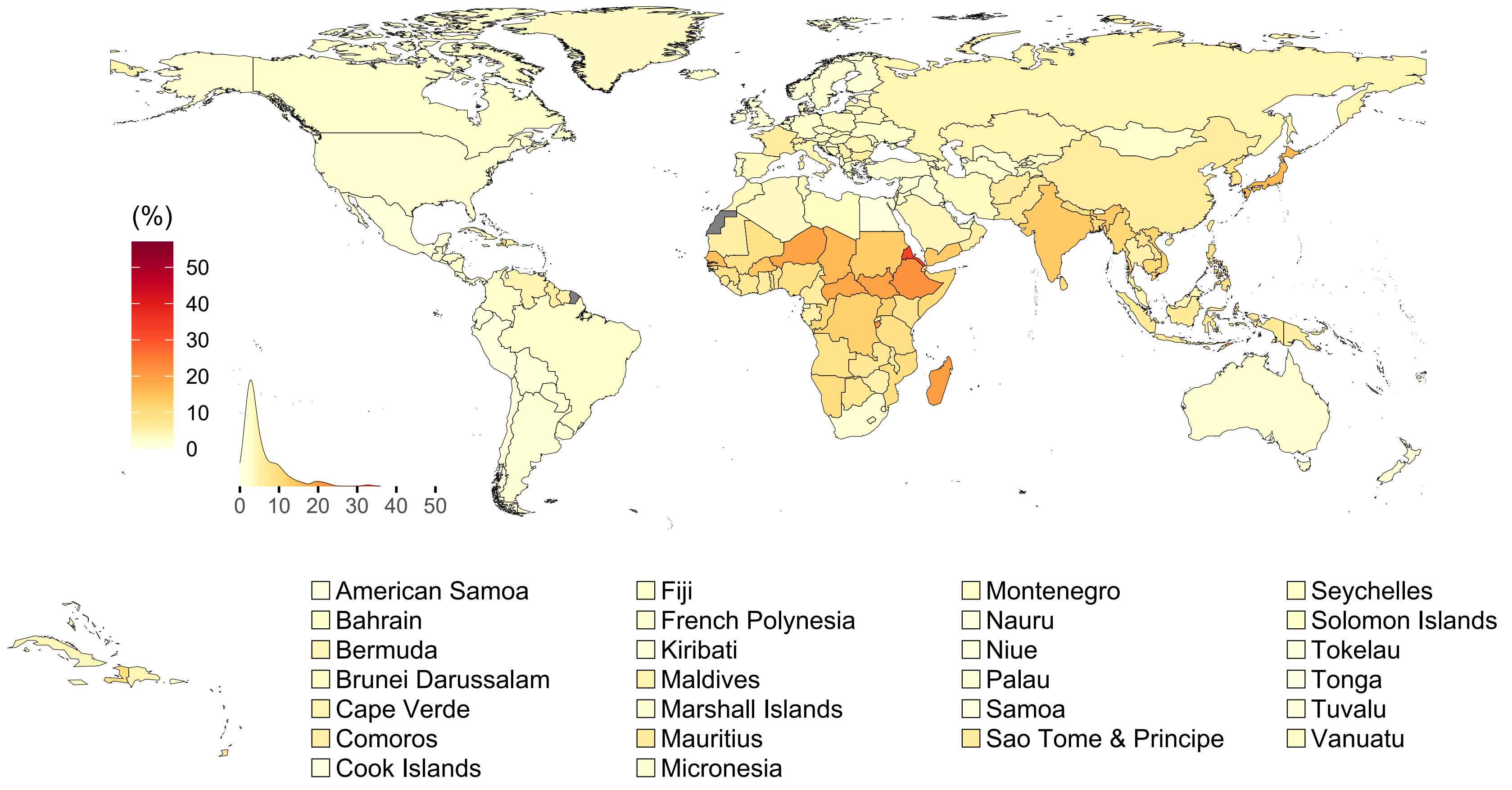


Posterior probability of an increase from 1990 to 2022

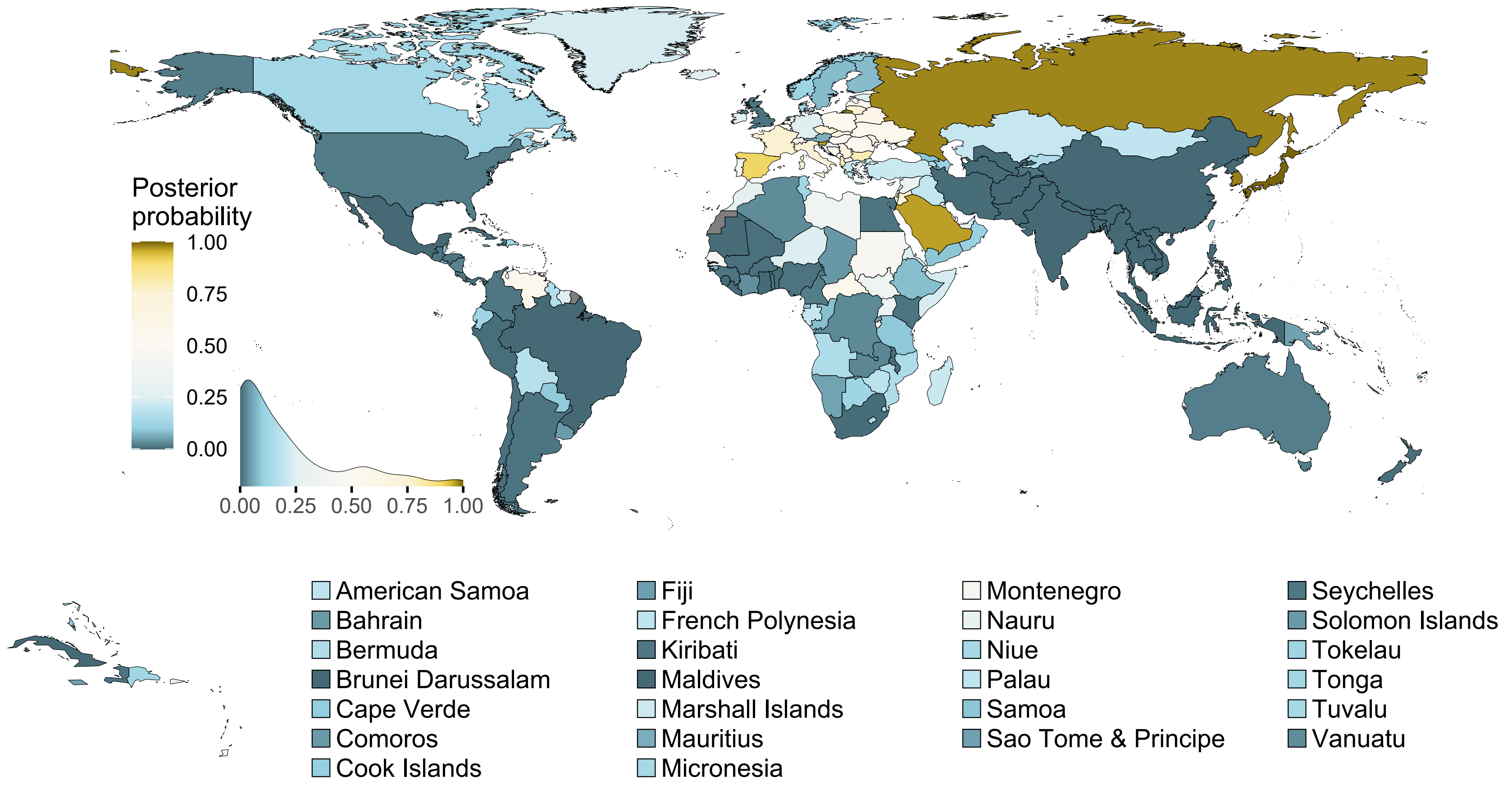

# Men

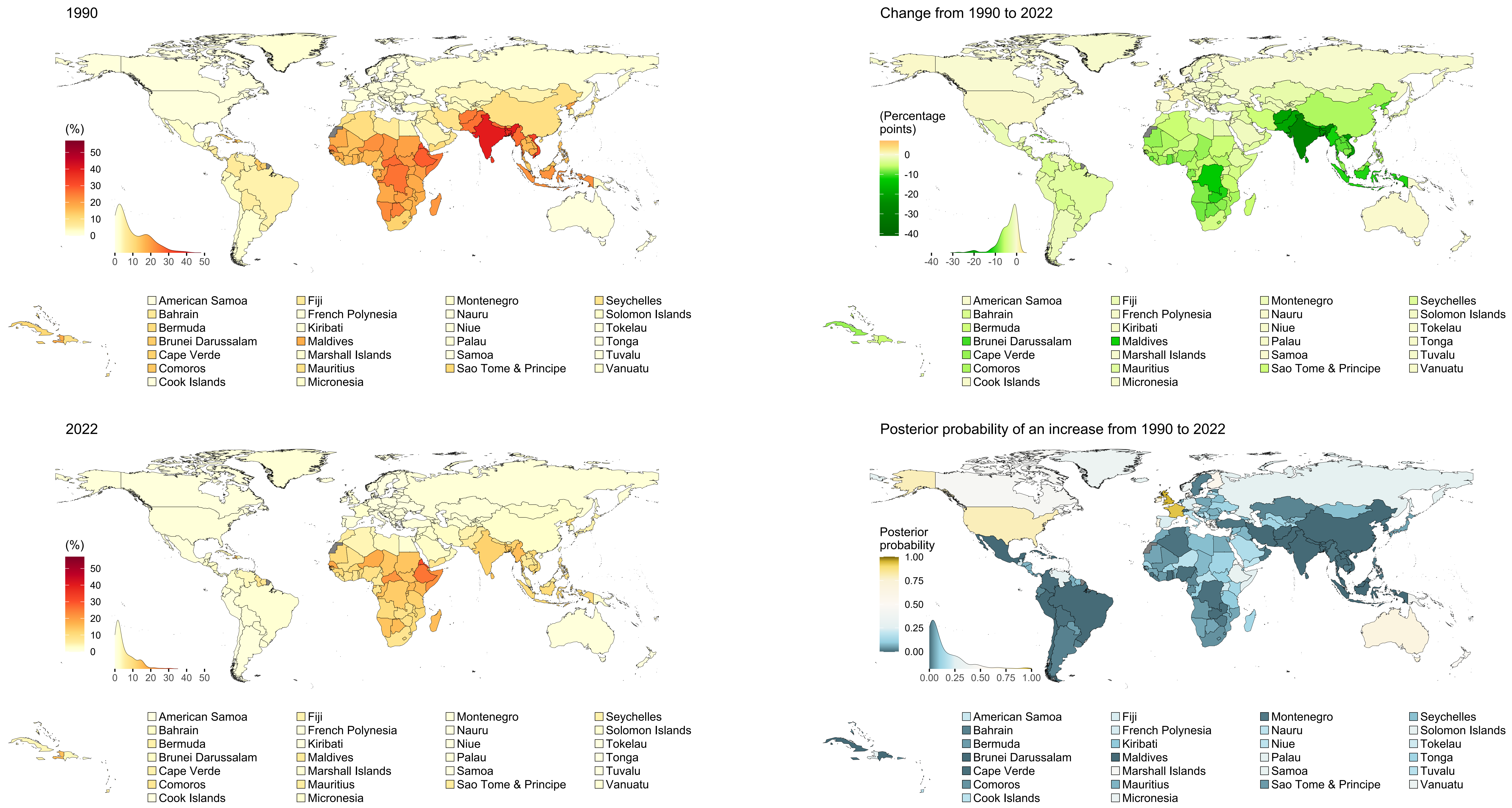
1990
(%)
50
40
30
20
10
0
0 10 20 30 40 50
American Samoa
Bahrain
Bermuda
Brunei Darussalam
Cape Verde
Comoros
Cook Islands
Fiji
French Polynesia
Kiribati
Maldives
Marshall Islands
Mauritius
Micronesia
Montenegro
Nauru
Niue
Palau
Samoa
Sao Tome & Principe
Seychelles
Solomon Islands
Tokelau
Tonga
Tuvalu
Vanuatu
Change from 1990 to 2022
(Percentage points)
0
-10
-20
-30
-40
-40 -30 -20 -10 0
American Samoa
Bahrain
Bermuda
Brunei Darussalam
Cape Verde
Comoros
Cook Islands
Fiji
French Polynesia
Kiribati
Maldives
Marshall Islands
Mauritius
Micronesia
Montenegro
Nauru
Niue
Palau
Samoa
Sao Tome & Principe
Seychelles
Solomon Islands
Tokelau
Tonga
Tuvalu
Vanuatu
2022
(%)
50
40
30
20
10
0
0 10 20 30 40 50
American Samoa
Bahrain
Bermuda
Brunei Darussalam
Cape Verde
Comoros
Cook Islands
Fiji
French Polynesia
Kiribati
Maldives
Marshall Islands
Mauritius
Micronesia
Montenegro
Nauru
Niue
Palau
Samoa
Sao Tome & Principe
Seychelles
Solomon Islands
Tokelau
Tonga
Tuvalu
Vanuatu
Posterior probability of an increase from 1990 to 2022
Posterior probability
1.00
0.75
0.50
0.25
0.00
0.00 0.25 0.50 0.75 1.00
American Samoa
Bahrain
Bermuda
Brunei Darussalam
Cape Verde
Comoros
Cook Islands
Fiji
French Polynesia
Kiribati
Maldives
Marshall Islands
Mauritius
Micronesia
Montenegro
Nauru
Niue
Palau
Samoa
Sao Tome & Principe
Seychelles
Solomon Islands
Tokelau
Tonga
Tuvalu
Vanuatu

**Appendix Figure 6.** Age-standardised prevalence of obesity in 1990 and 2022, its change from 1990 to 2022, and posterior probability that the prevalence increased from 1990 to 2022, for adults.

# Women

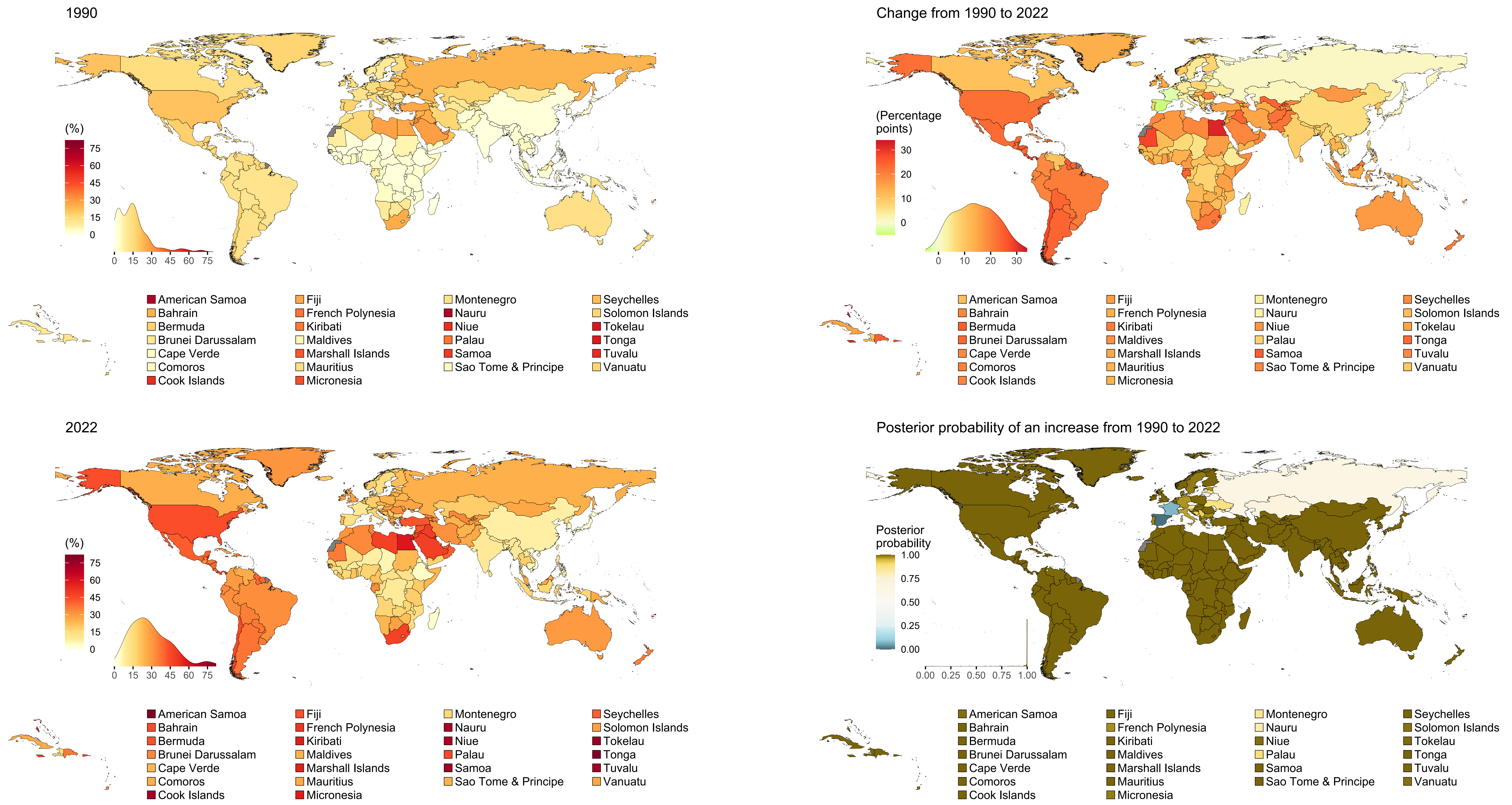
1990
(%)
75
60
45
30
15
0
0 15 30 45 60 75
American Samoa
Bahrain
Bermuda
Brunei Darussalam
Cape Verde
Comoros
Cook Islands
Fiji
French Polynesia
Kiribati
Maldives
Marshall Islands
Mauritius
Micronesia
Montenegro
Nauru
Niue
Palau
Samoa
Sao Tome & Principe
Seychelles
Solomon Islands
Tokelau
Tonga
Tuvalu
Vanuatu
Change from 1990 to 2022
(Percentage points)
30
20
10
0
0 10 20 30
American Samoa
Bahrain
Bermuda
Brunei Darussalam
Cape Verde
Comoros
Cook Islands
Fiji
French Polynesia
Kiribati
Maldives
Marshall Islands
Mauritius
Micronesia
Montenegro
Nauru
Niue
Palau
Samoa
Sao Tome & Principe
Seychelles
Solomon Islands
Tokelau
Tonga
Tuvalu
Vanuatu
2022
(%)
75
60
45
30
15
0
0 15 30 45 60 75
American Samoa
Bahrain
Bermuda
Brunei Darussalam
Cape Verde
Comoros
Cook Islands
Fiji
French Polynesia
Kiribati
Maldives
Marshall Islands
Mauritius
Micronesia
Montenegro
Nauru
Niue
Palau
Samoa
Sao Tome & Principe
Seychelles
Solomon Islands
Tokelau
Tonga
Tuvalu
Vanuatu
Posterior probability of an increase from 1990 to 2022
Posterior probability
1.00
0.75
0.50
0.25
0.00
0.00 0.25 0.50 0.75 1.00
American Samoa
Bahrain
Bermuda
Brunei Darussalam
Cape Verde
Comoros
Cook Islands
Fiji
French Polynesia
Kiribati
Maldives
Marshall Islands
Mauritius
Micronesia
Montenegro
Nauru
Niue
Palau
Samoa
Sao Tome & Principe
Seychelles
Solomon Islands
Tokelau
Tonga
Tuvalu
Vanuatu

# Men

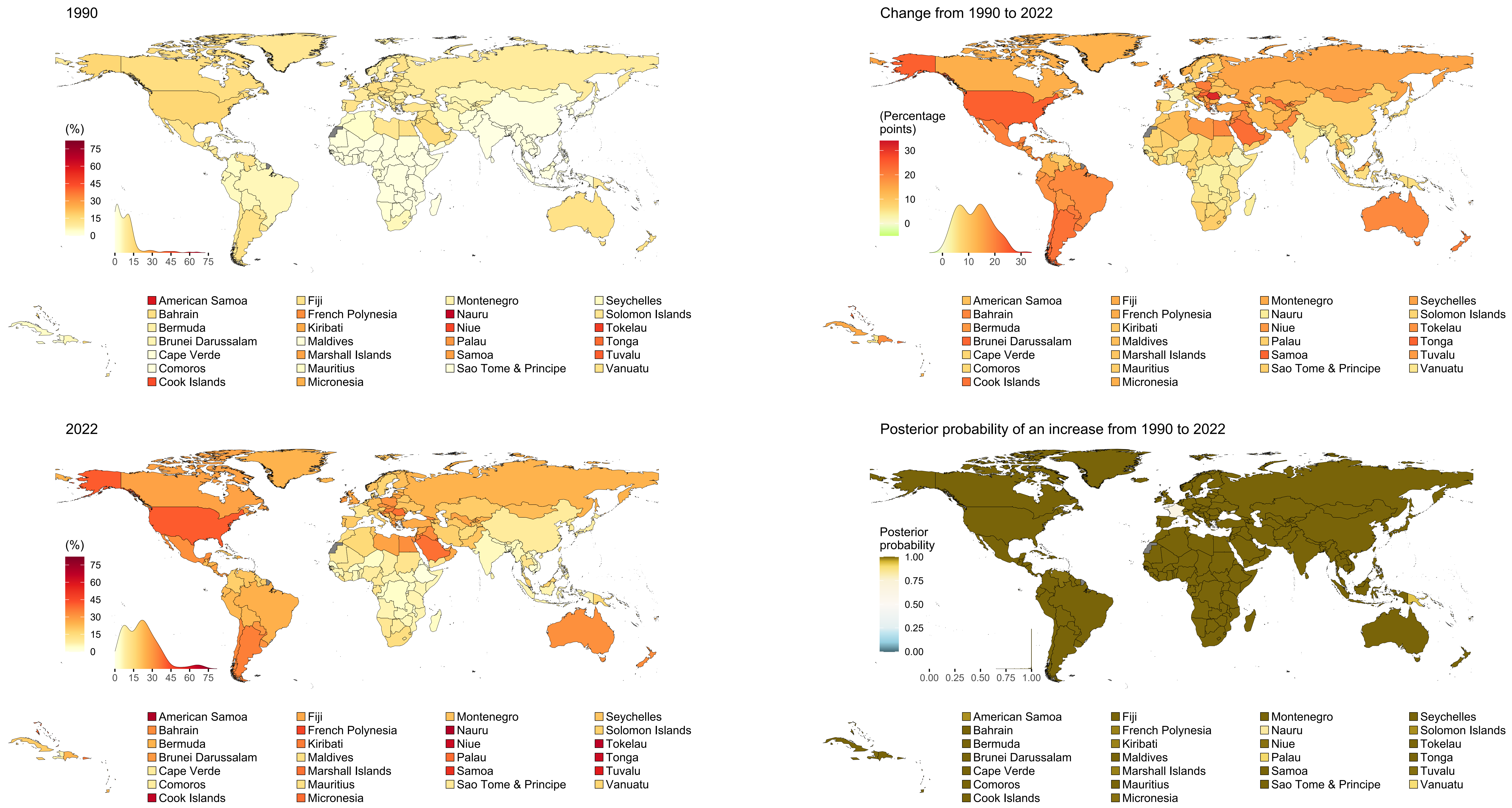
1990
(%)
75
60
45
30
15
0
0 15 30 45 60 75
American Samoa
Bahrain
Bermuda
Brunei Darussalam
Cape Verde
Comoros
Cook Islands
Fiji
French Polynesia
Kiribati
Maldives
Marshall Islands
Mauritius
Micronesia
Montenegro
Nauru
Niue
Palau
Samoa
Sao Tome & Principe
Seychelles
Solomon Islands
Tokelau
Tonga
Tuvalu
Vanuatu
Change from 1990 to 2022
(Percentage points)
30
20
10
0
0 10 20 30
American Samoa
Bahrain
Bermuda
Brunei Darussalam
Cape Verde
Comoros
Cook Islands
Fiji
French Polynesia
Kiribati
Maldives
Marshall Islands
Mauritius
Micronesia
Montenegro
Nauru
Niue
Palau
Samoa
Sao Tome & Principe
Seychelles
Solomon Islands
Tokelau
Tonga
Tuvalu
Vanuatu
2022
(%)
75
60
45
30
15
0
0 15 30 45 60 75
American Samoa
Bahrain
Bermuda
Brunei Darussalam
Cape Verde
Comoros
Cook Islands
Fiji
French Polynesia
Kiribati
Maldives
Marshall Islands
Mauritius
Micronesia
Montenegro
Nauru
Niue
Palau
Samoa
Sao Tome & Principe
Seychelles
Solomon Islands
Tokelau
Tonga
Tuvalu
Vanuatu
Posterior probability of an increase from 1990 to 2022
Posterior probability
1.00
0.75
0.50
0.25
0.00
0.00 0.25 0.50 0.75 1.00
American Samoa
Bahrain
Bermuda
Brunei Darussalam
Cape Verde
Comoros
Cook Islands
Fiji
French Polynesia
Kiribati
Maldives
Marshall Islands
Mauritius
Micronesia
Montenegro
Nauru
Niue
Palau
Samoa
Sao Tome & Principe
Seychelles
Solomon Islands
Tokelau
Tonga
Tuvalu
Vanuatu

**Appendix Figure 7.** Posterior probability that age-standardised combined prevalence of underweight and obesity increased from 1990 to 2022, for adults.

If an increase in the prevalence of the combined prevalence is statistically indistinguishable from a decrease, the PP is 0.5. PPs closer to 1 indicate more certainty of an increase in the prevalence of the combined prevalence, those towards 0 indicate more certainty of a decrease, and those closer to 0.5 indicate less certainty of an increase or decrease.

# Women

Posterior probability of an increase from 1990 to 2022

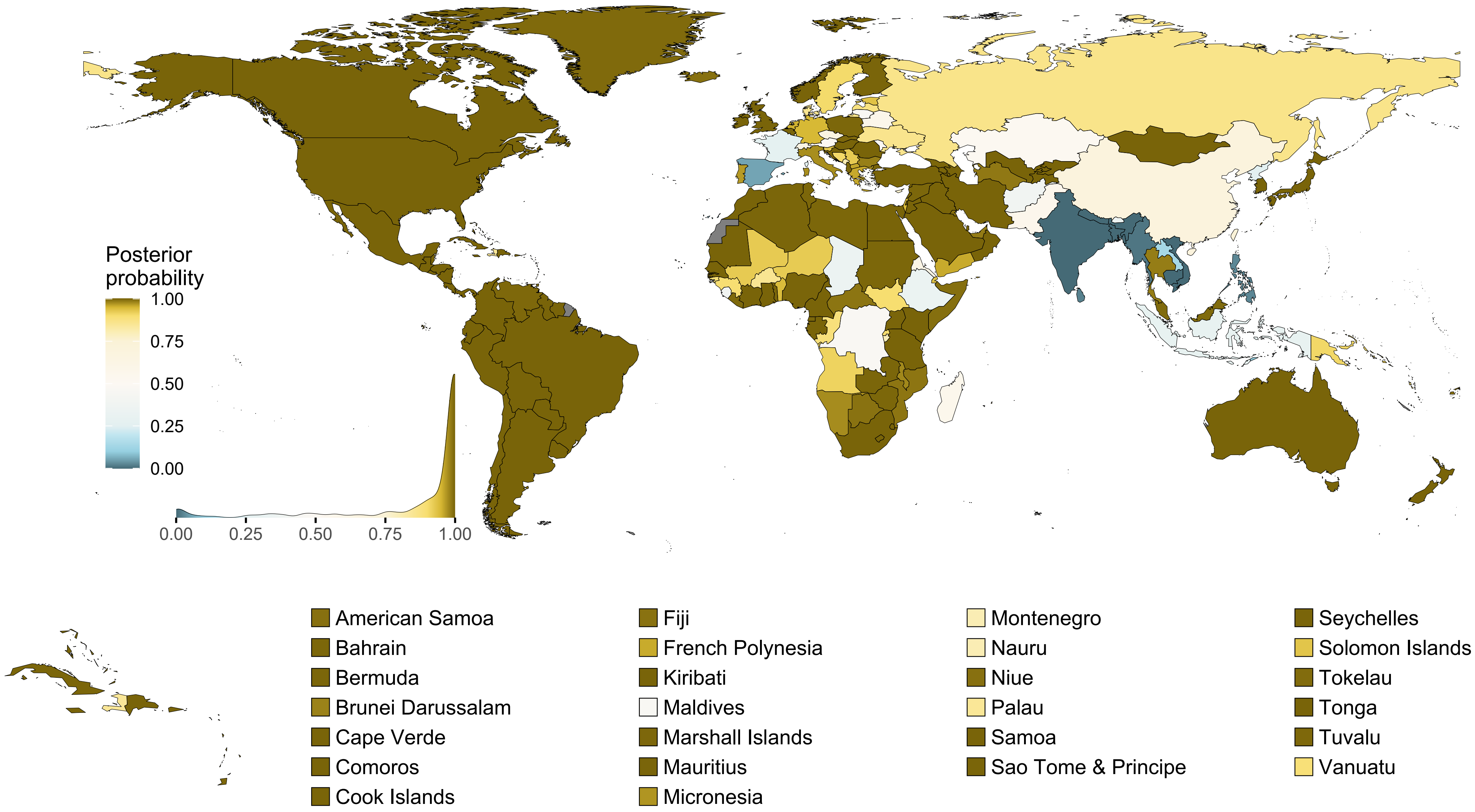


# Men

Posterior probability of an increase from 1990 to 2022

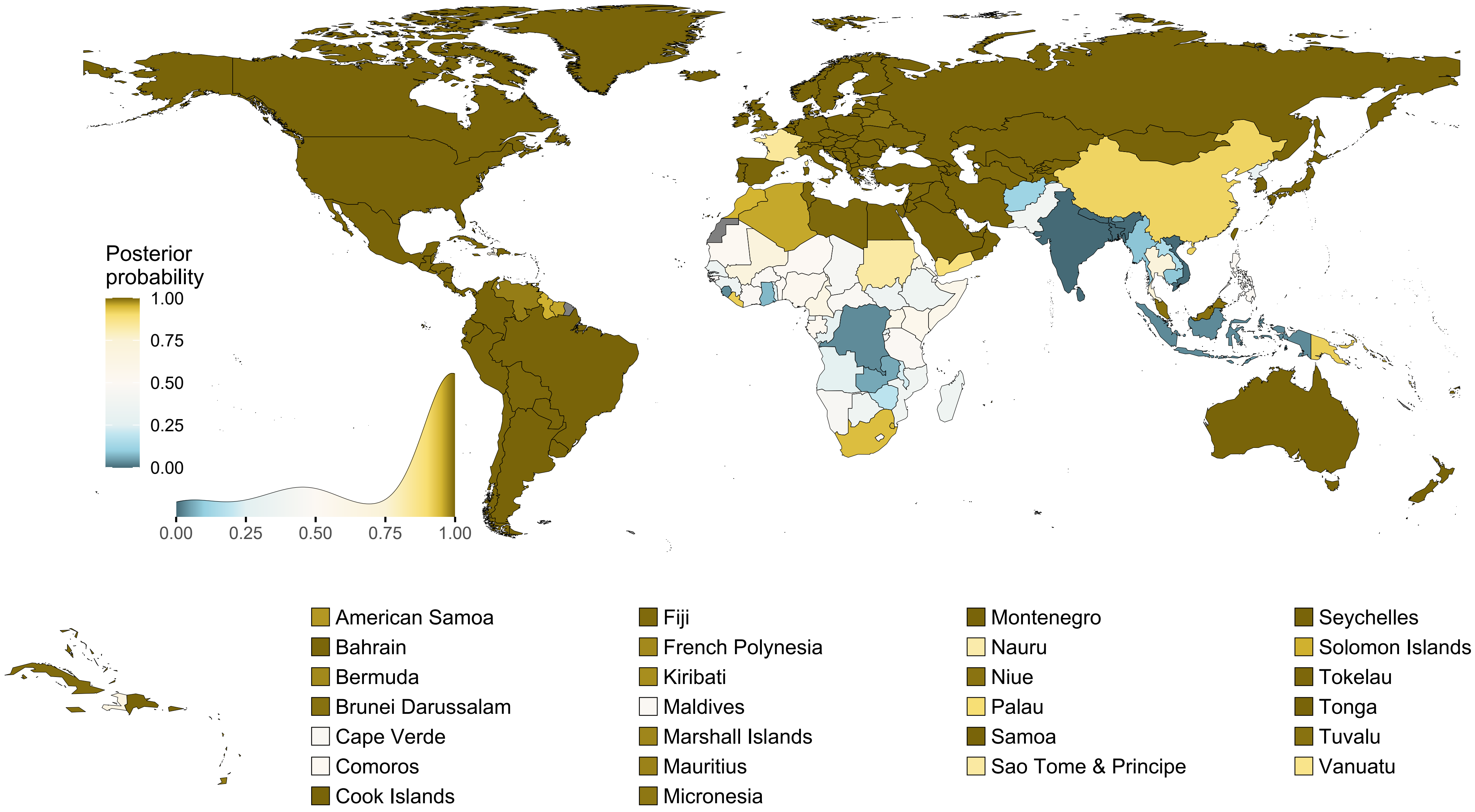

**Appendix Figure 8.** Change in age-standardised proportion of double burden composed of obesity and posterior probability that the proportion increased from 1990 to 2022, for adults.

# Women

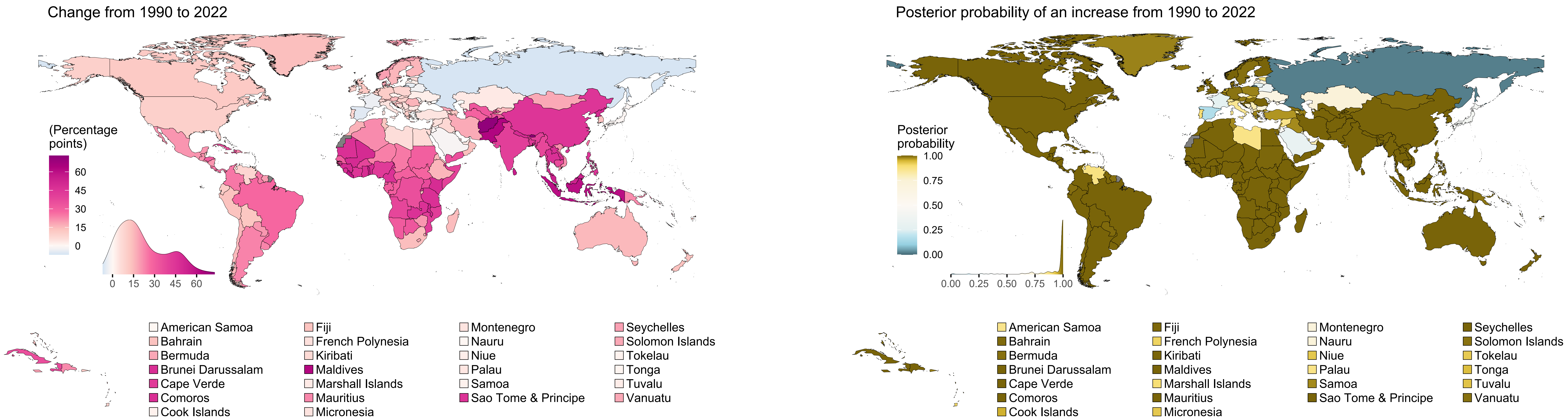
Change from 1990 to 2022
(Percentage points)
60
45
30
15
0
0 15 30 45 60
American Samoa
Bahrain
Bermuda
Brunei Darussalam
Cape Verde
Comoros
Cook Islands
Fiji
French Polynesia
Kiribati
Maldives
Marshall Islands
Mauritius
Micronesia
Montenegro
Nauru
Niue
Palau
Samoa
Sao Tome & Principe
Seychelles
Solomon Islands
Tokelau
Tonga
Tuvalu
Vanuatu
Posterior probability of an increase from 1990 to 2022
Posterior probability
1.00
0.75
0.50
0.25
0.00
0.00 0.25 0.50 0.75 1.00
American Samoa
Bahrain
Bermuda
Brunei Darussalam
Cape Verde
Comoros
Cook Islands
Fiji
French Polynesia
Kiribati
Maldives
Marshall Islands
Mauritius
Micronesia
Montenegro
Nauru
Niue
Palau
Samoa
Sao Tome & Principe
Seychelles
Solomon Islands
Tokelau
Tonga
Tuvalu
Vanuatu

# Men

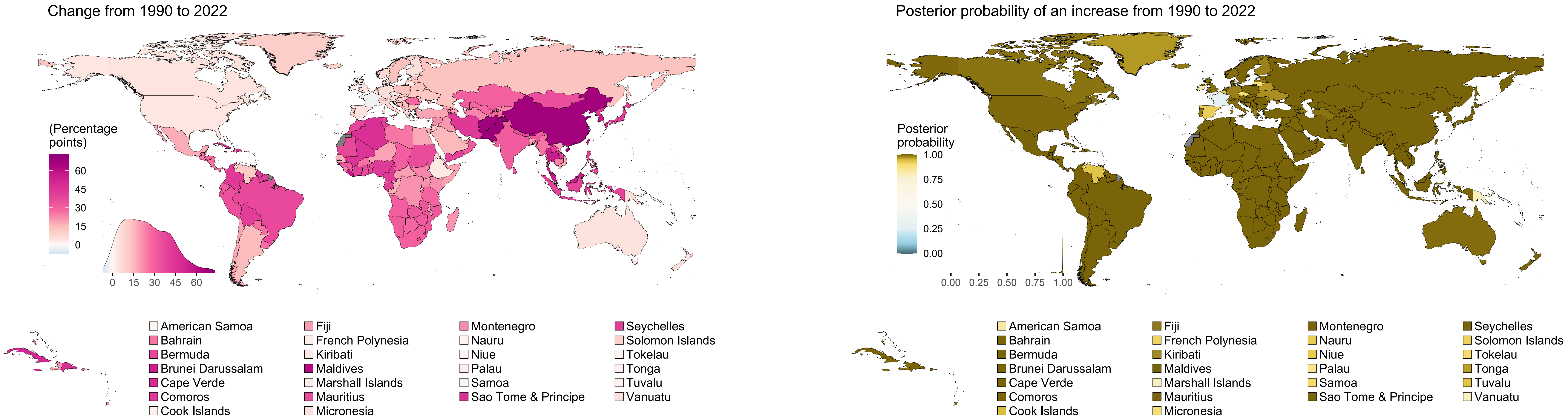
Change from 1990 to 2022
(Percentage points)
60
45
30
15
0
0 15 30 45 60
American Samoa
Bahrain
Bermuda
Brunei Darussalam
Cape Verde
Comoros
Cook Islands
Fiji
French Polynesia
Kiribati
Maldives
Marshall Islands
Mauritius
Micronesia
Montenegro
Nauru
Niue
Palau
Samoa
Sao Tome & Principe
Seychelles
Solomon Islands
Tokelau
Tonga
Tuvalu
Vanuatu
Posterior probability of an increase from 1990 to 2022
Posterior probability
1.00
0.75
0.50
0.25
0.00
0.00 0.25 0.50 0.75 1.00
American Samoa
Bahrain
Bermuda
Brunei Darussalam
Cape Verde
Comoros
Cook Islands
Fiji
French Polynesia
Kiribati
Maldives
Marshall Islands
Mauritius
Micronesia
Montenegro
Nauru
Niue
Palau
Samoa
Sao Tome & Principe
Seychelles
Solomon Islands
Tokelau
Tonga
Tuvalu
Vanuatu

**Appendix Figure 9.** Combined prevalence of underweight and obesity for adults, by age group.

The circular bar plots show the burden of underweight and obesity in 1990 and 2022. The lengths of the blue and red bars show the age-standardised prevalence of underweight and obesity respectively, and their sum shows the age-standardised combined prevalence. Country names are coloured by region. The numbers in brackets after each country's name show the total number of data sources and the number of nationally representative data sources, respectively. Countries are ordered by decreasing combined prevalence within each region. The maps show the change in combined prevalence of underweight and obesity from 1990 to 2022, and its level in 2022. The density plot alongside each map shows the smoothed distribution of estimates across countries.

Prevalence was age-standardised within each age group presented.

# Women, 20-39 years

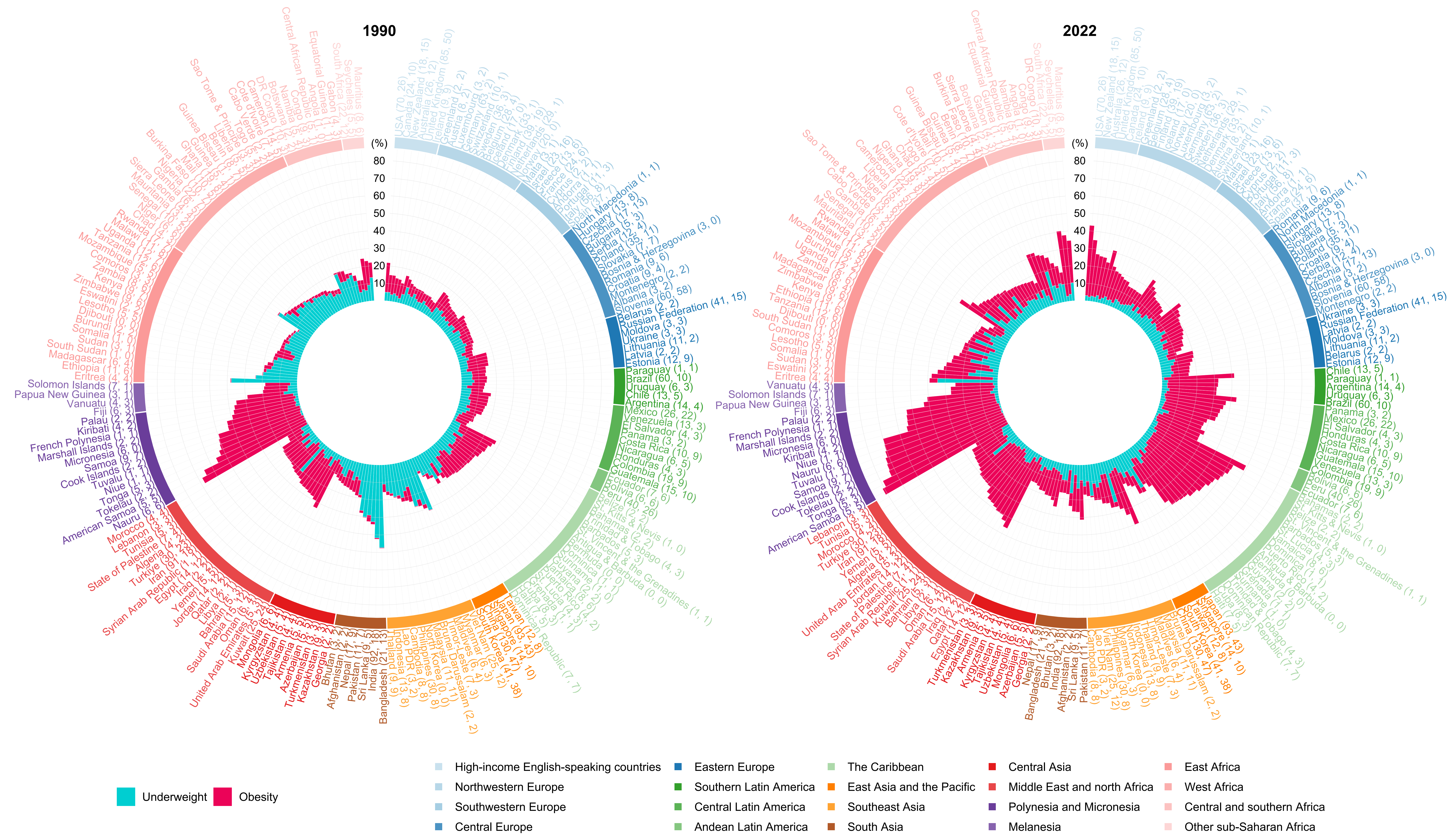


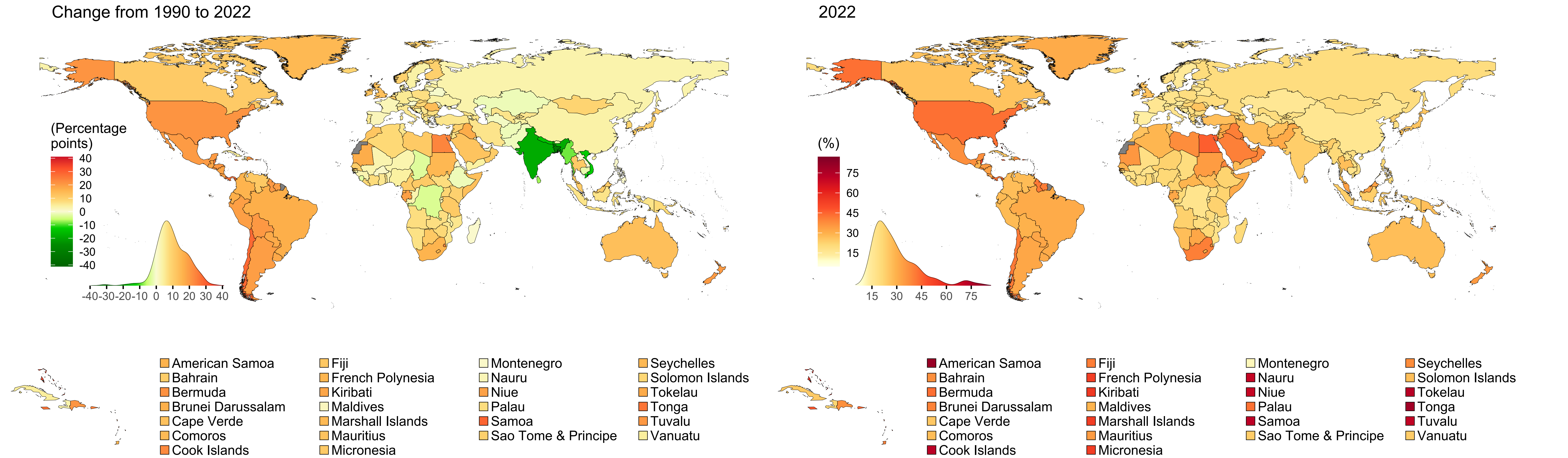

# Women, 40-64 years

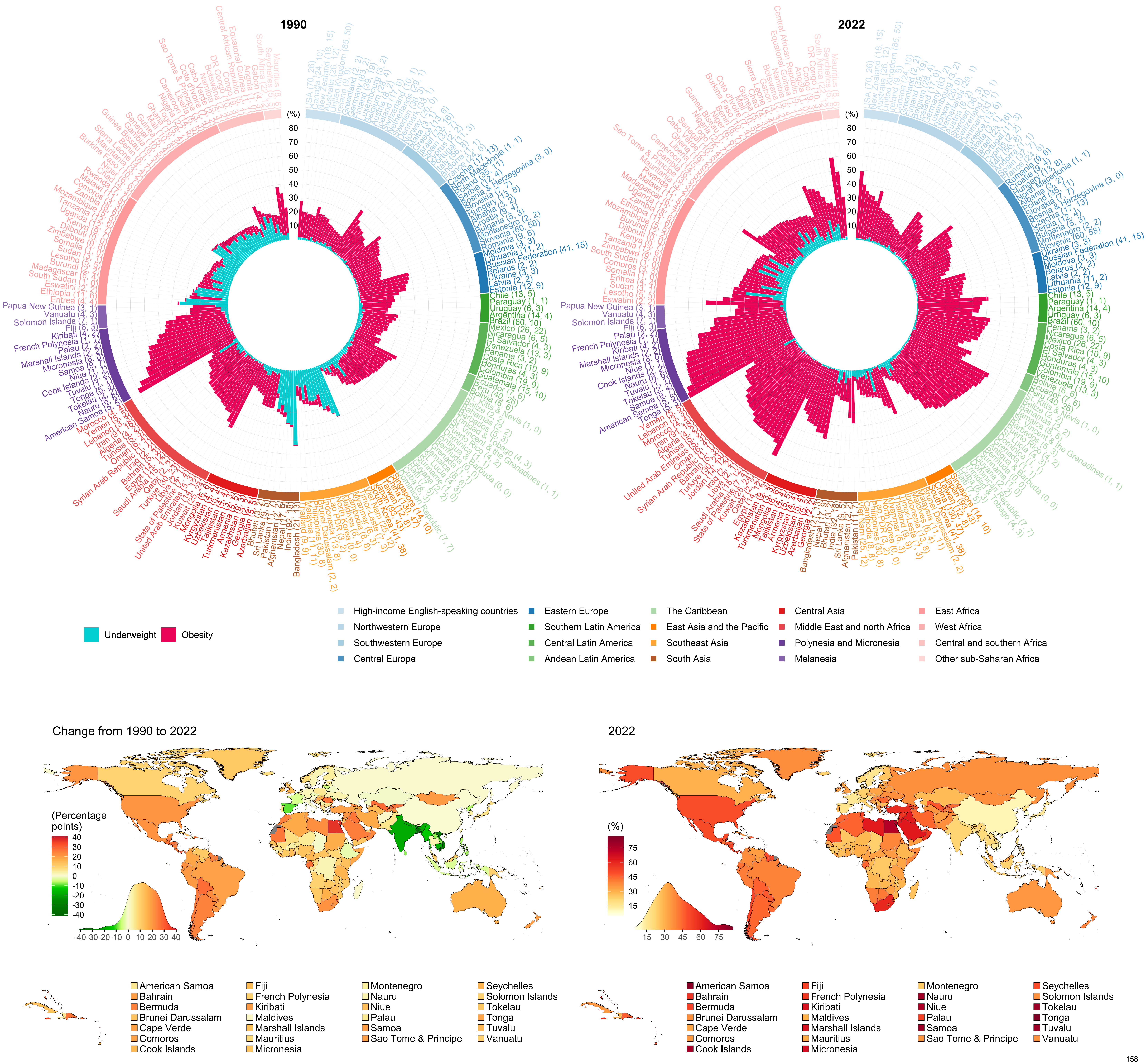

# Women, 65+ years

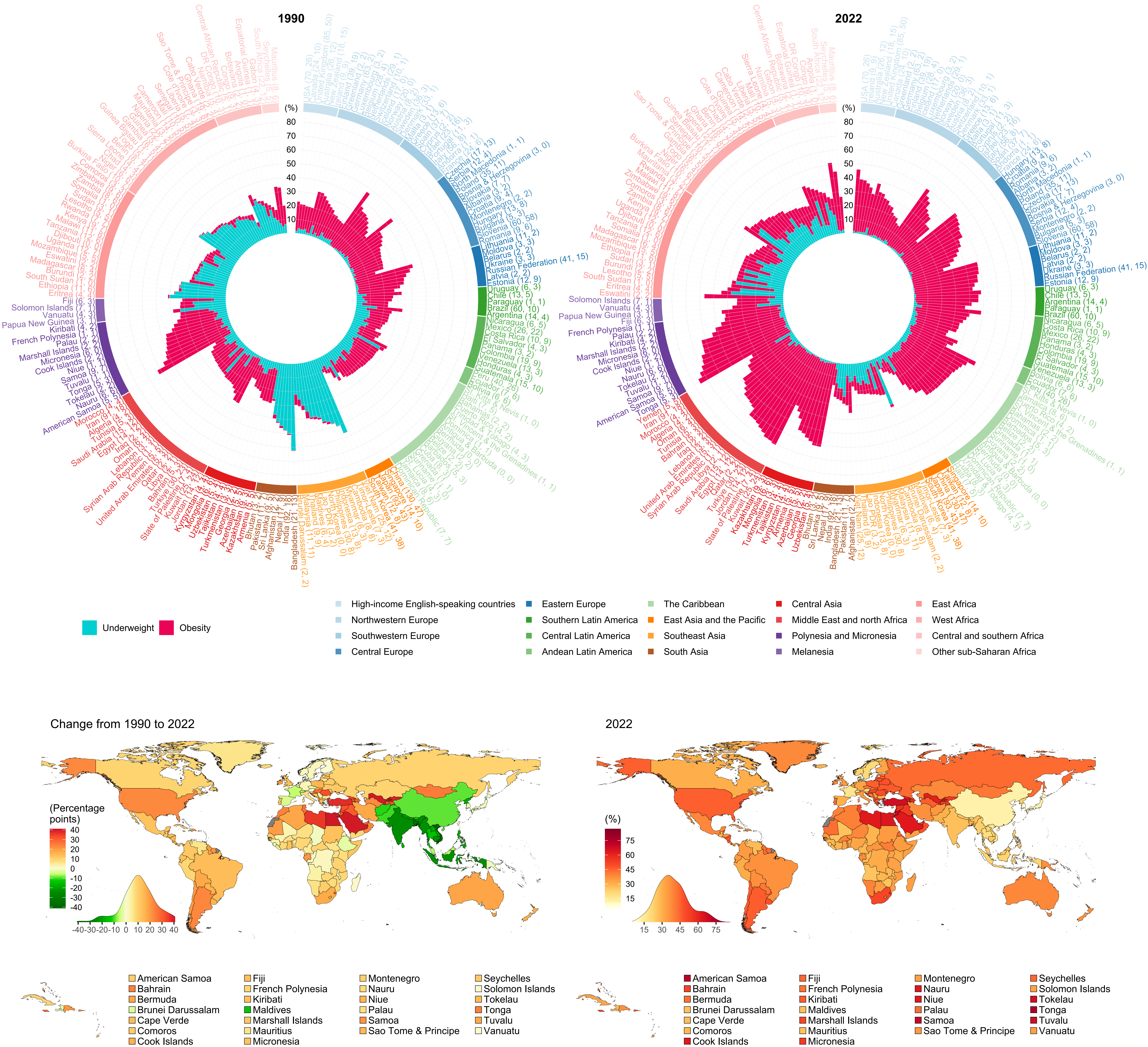

# Men, 20-39 years

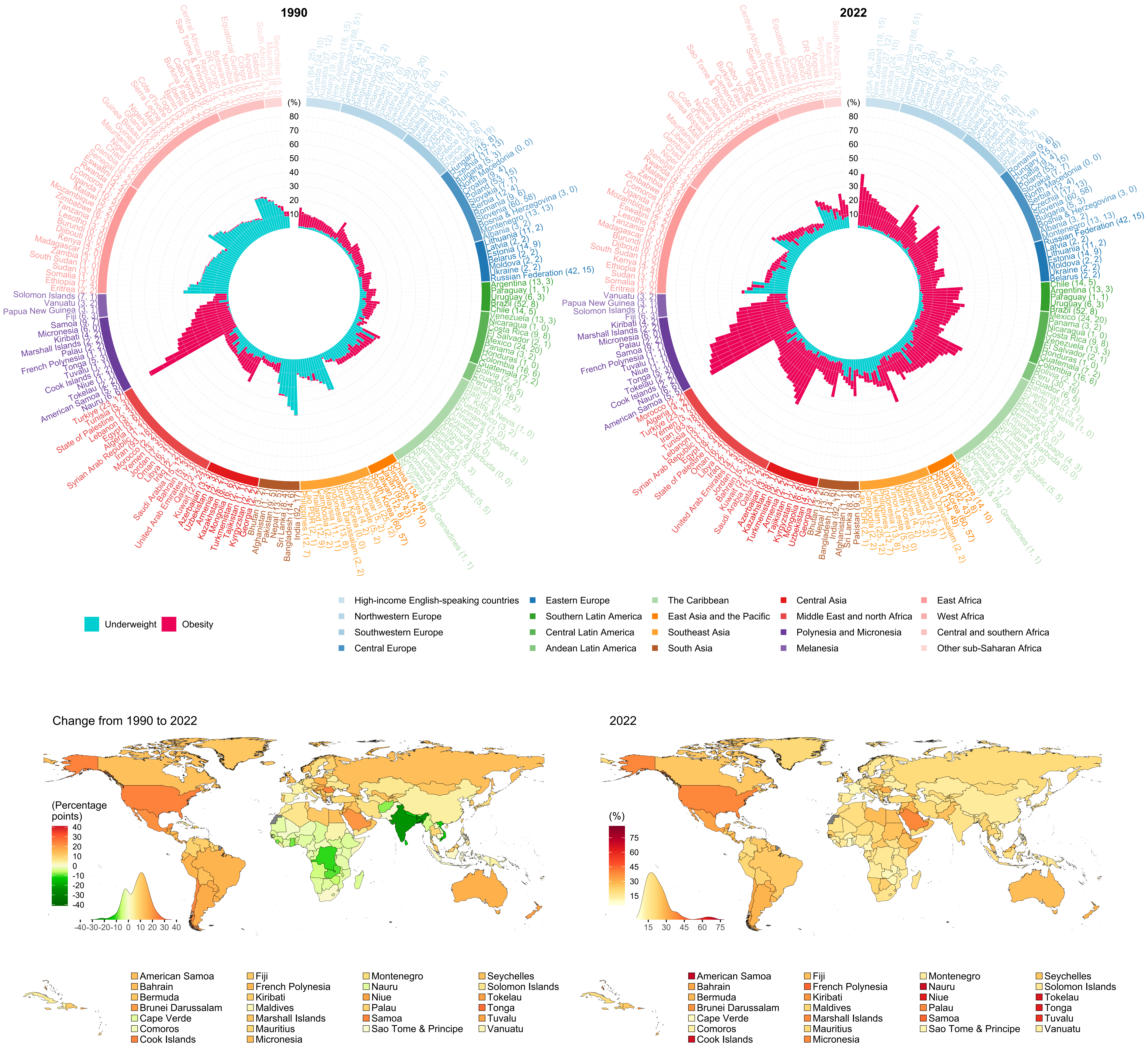

# Men, 40-64 years

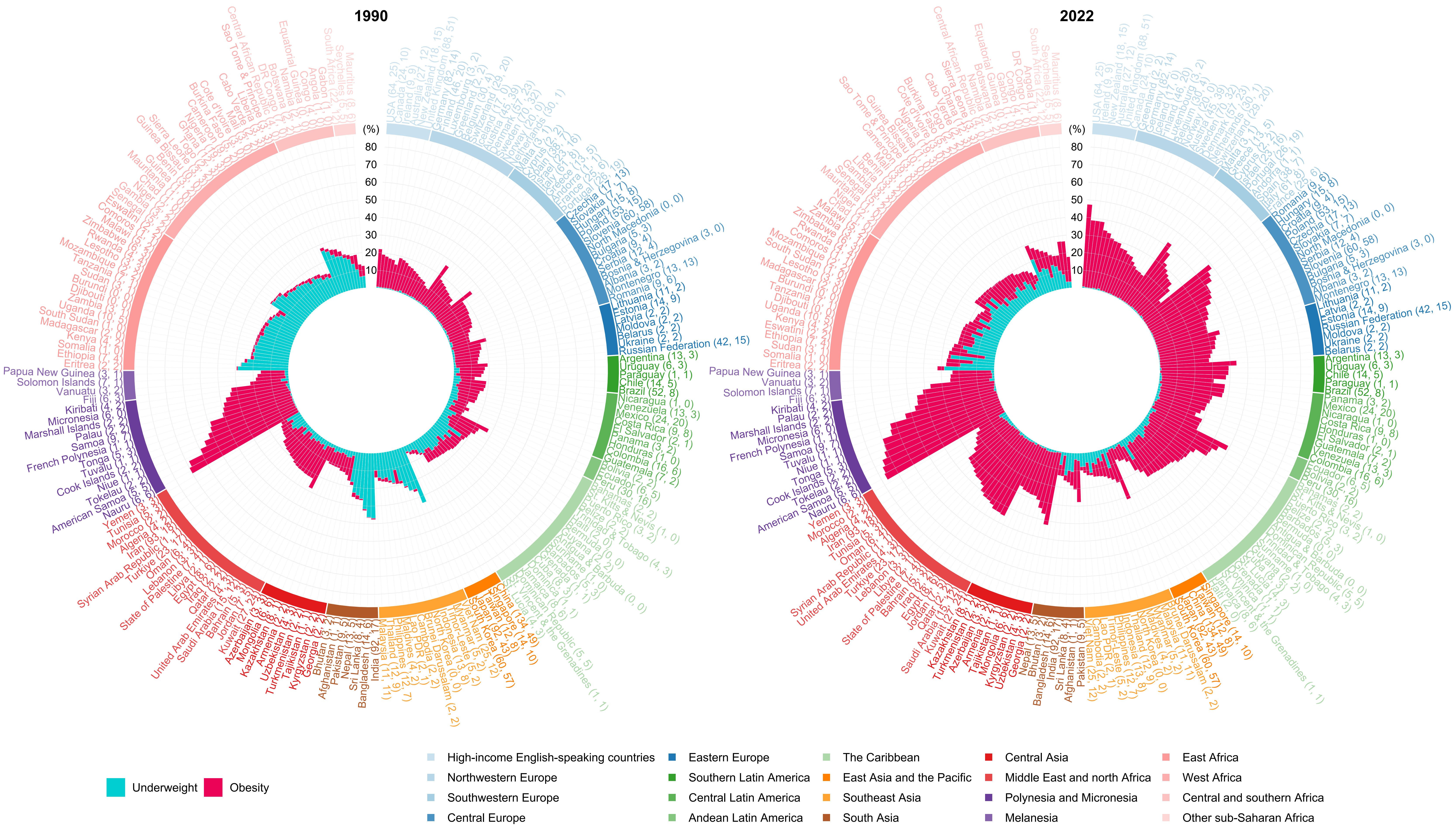


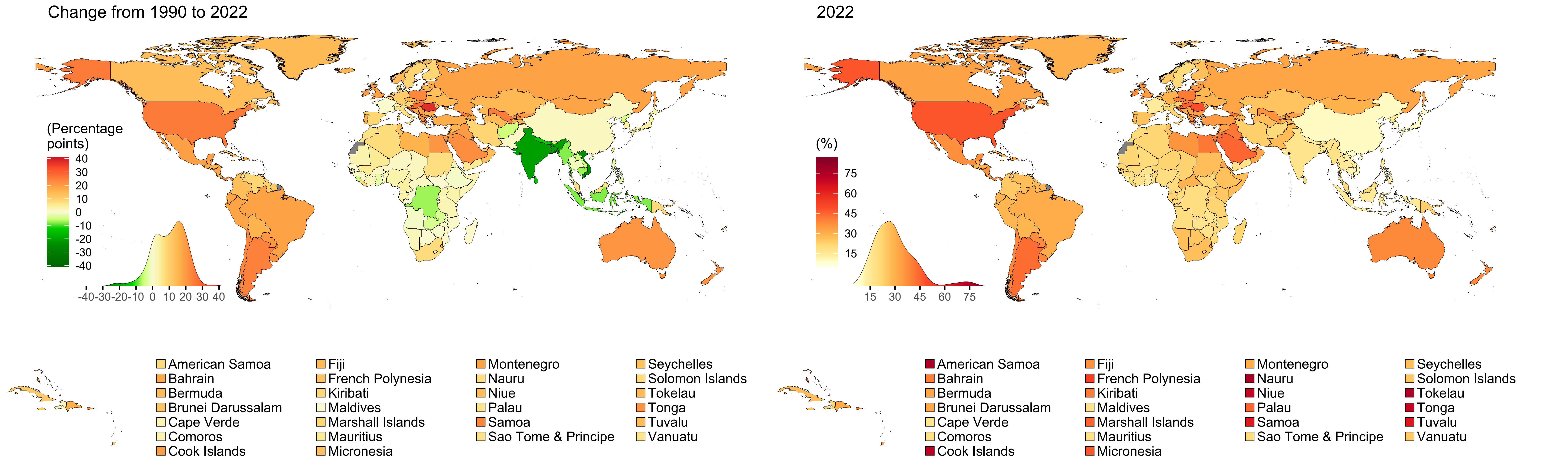

# Men, 65+ years

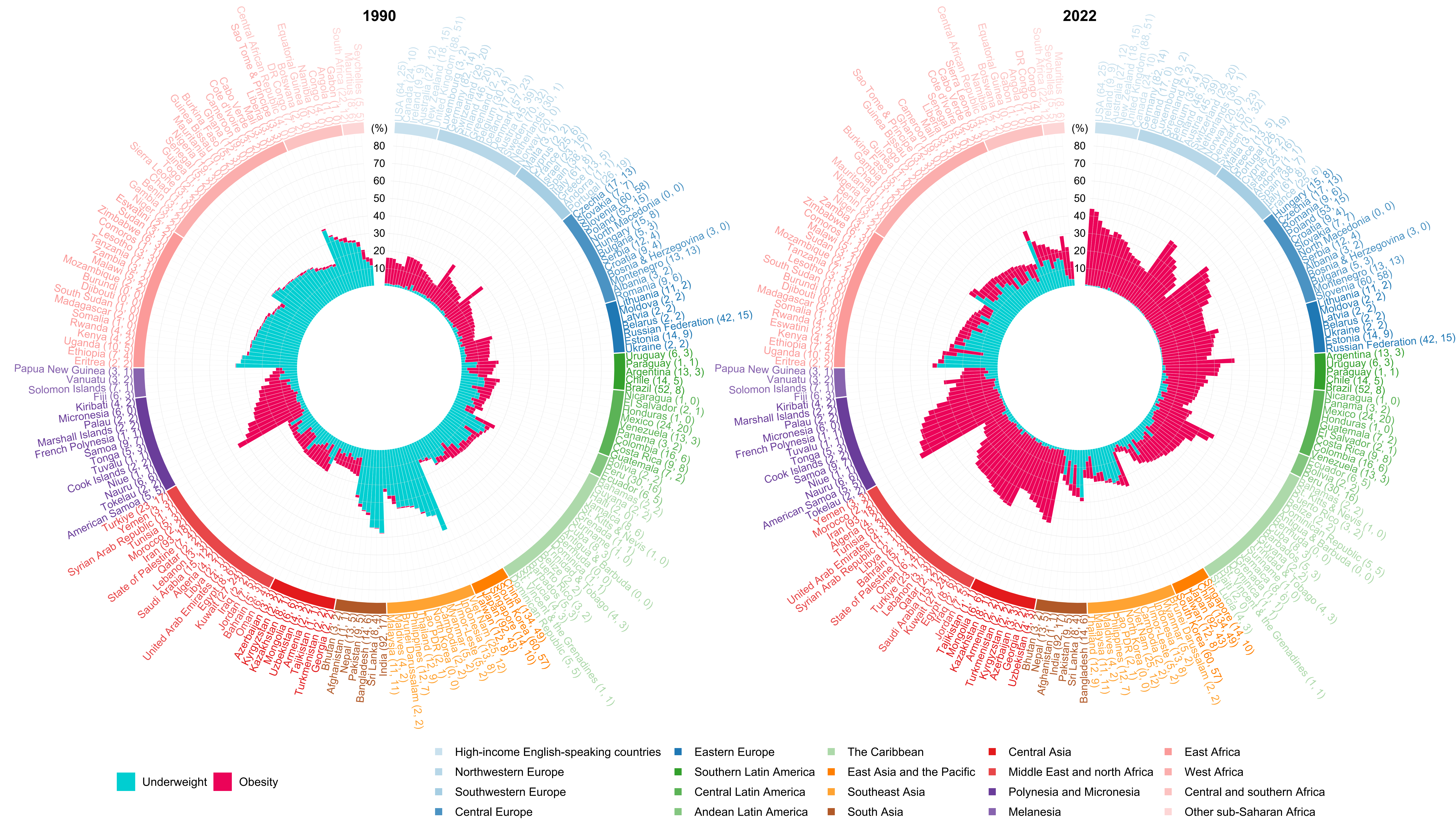

1990
2022
(%)
Underweight
Obesity
High-income English-speaking countries
Northwestern Europe
Southwestern Europe
Central Europe
Eastern Europe
Southern Latin America
Central Latin America
Andean Latin America
The Caribbean
East Asia and the Pacific
Southeast Asia
South Asia
Central Asia
Middle East and north Africa
Polynesia and Micronesia
Melanesia
East Africa
West Africa
Central and southern Africa
Other sub-Saharan Africa


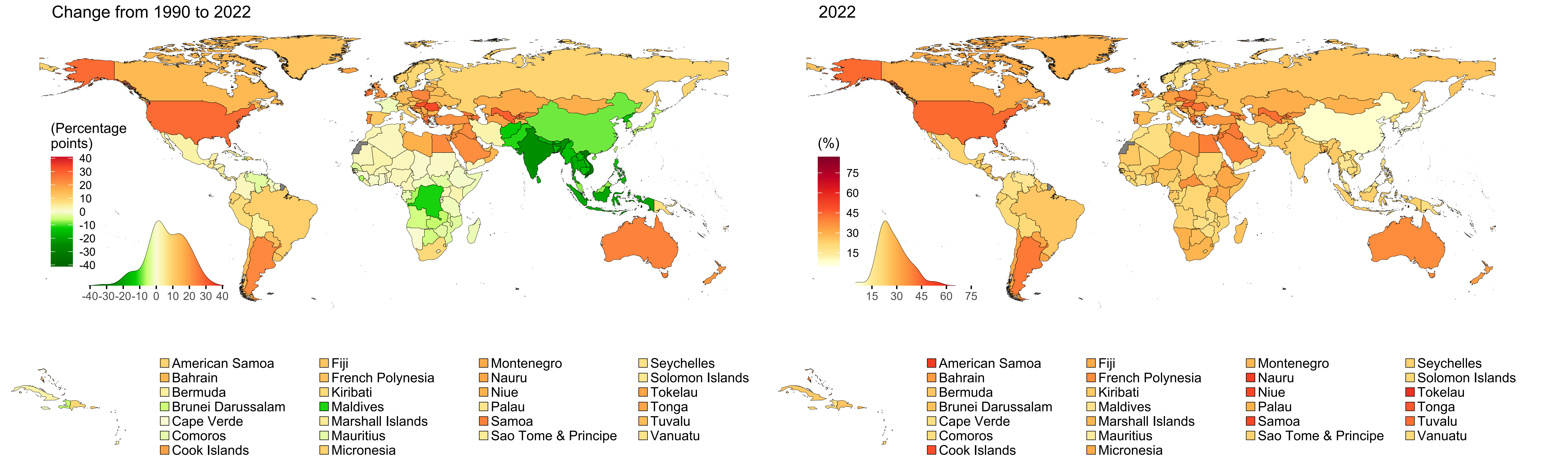

Change from 1990 to 2022
(Percentage points)
2022
(%)
American Samoa
Bahrain
Bermuda
Brunei Darussalam
Cape Verde
Comoros
Cook Islands
Fiji
French Polynesia
Kiribati
Maldives
Marshall Islands
Mauritius
Micronesia
Montenegro
Nauru
Niue
Palau
Samoa
Sao Tome & Principe
Seychelles
Solomon Islands
Tokelau
Tonga
Tuvalu
Vanuatu

**Appendix Figure 10.** Age-standardised proportion of the combined burden of underweight and obesity composed of obesity in 1990 and 2022, in relation to the uncertainty of the proportion measured by posterior standard deviation, for adults.

Each point shows one country. Points that are filled have posterior probability (PP) ≥0.80 of obesity being more prevalent than underweight, or of underweight being more prevalent than obesity. If neither is more prevalent than the other at PP = 0.80, the point is hollow.

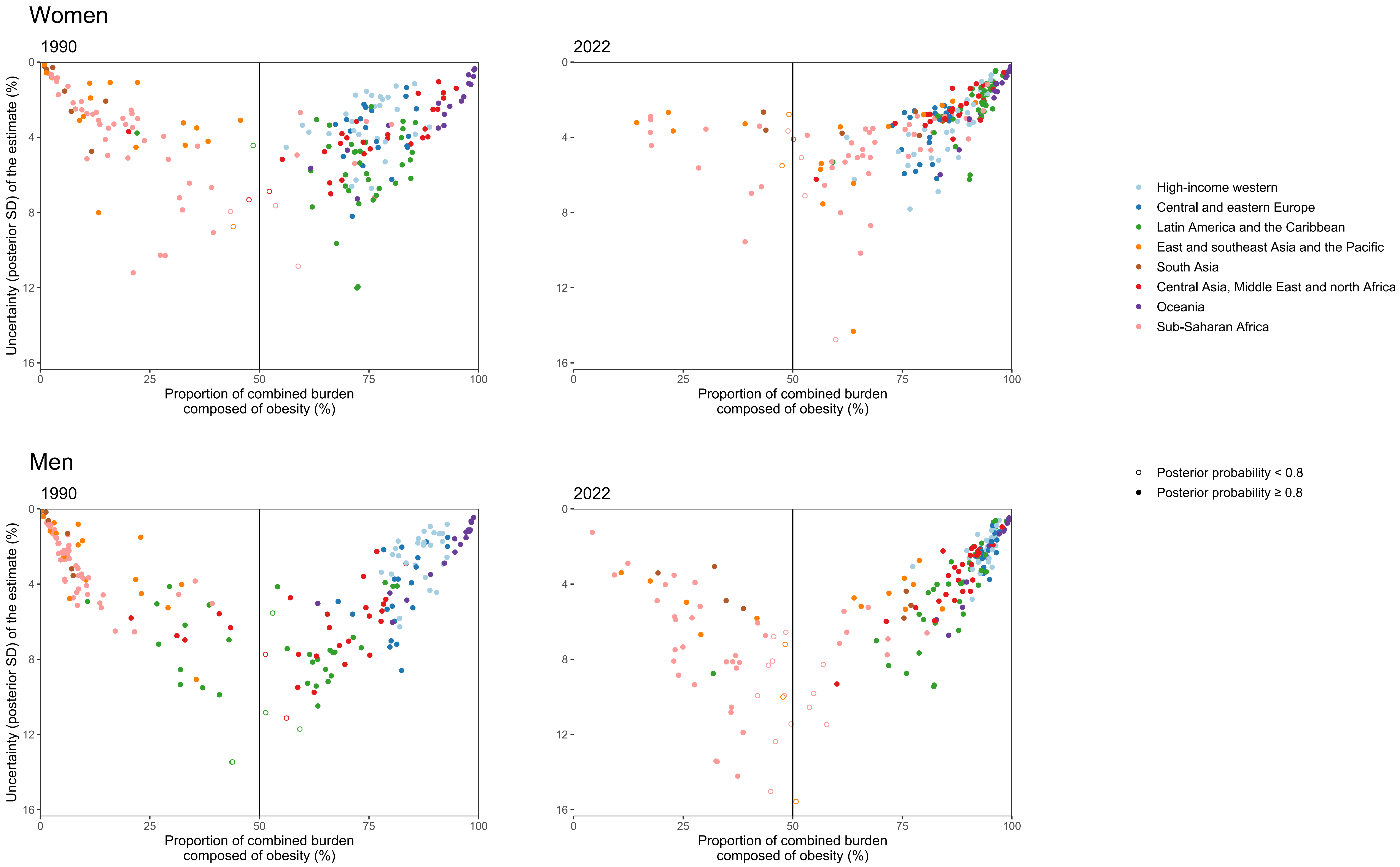
Women
1990
2022
Men
1990
2022
Uncertainty (posterior SD) of the estimate (%)
Proportion of combined burden composed of obesity (%)
0
4
8
12
16
0
25
50
75
100
High-income western
Central and eastern Europe
Latin America and the Caribbean
East and southeast Asia and the Pacific
South Asia
Central Asia, Middle East and north Africa
Oceania
Sub-Saharan Africa
Posterior probability < 0.8
Posterior probability ≥ 0.8

**Appendix Figure 11.** Proportion of double burden composed of obesity for adults, by age group.

Age-standardised proportion of double burden from obesity in 1990 and 2022. The density plot alongside each map shows the smoothed distribution of estimates across countries. Prevalence was age-standardised within each age group presented.

# Women, 20-39 years

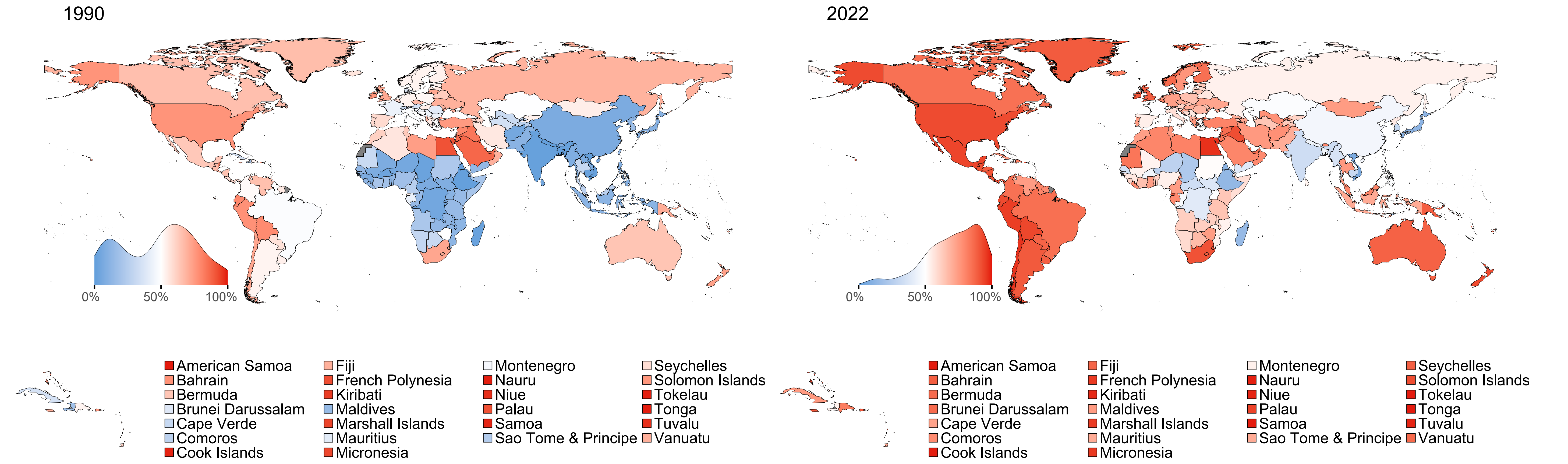


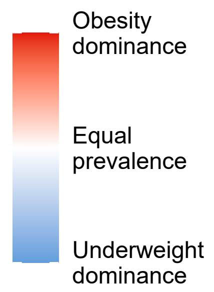


# Men, 20-39 years

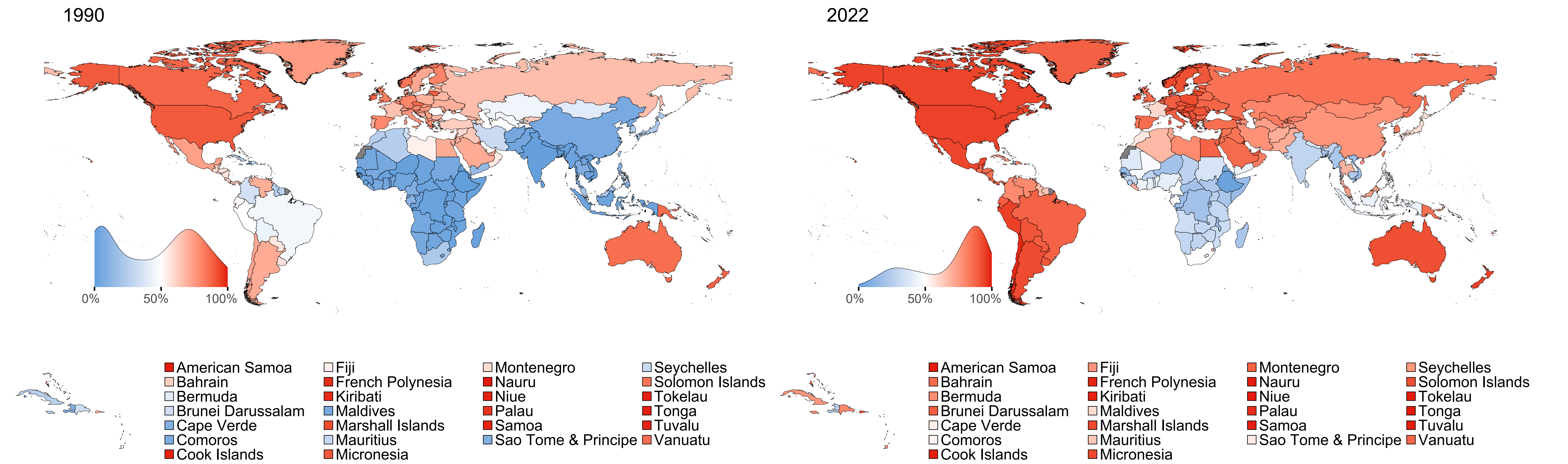

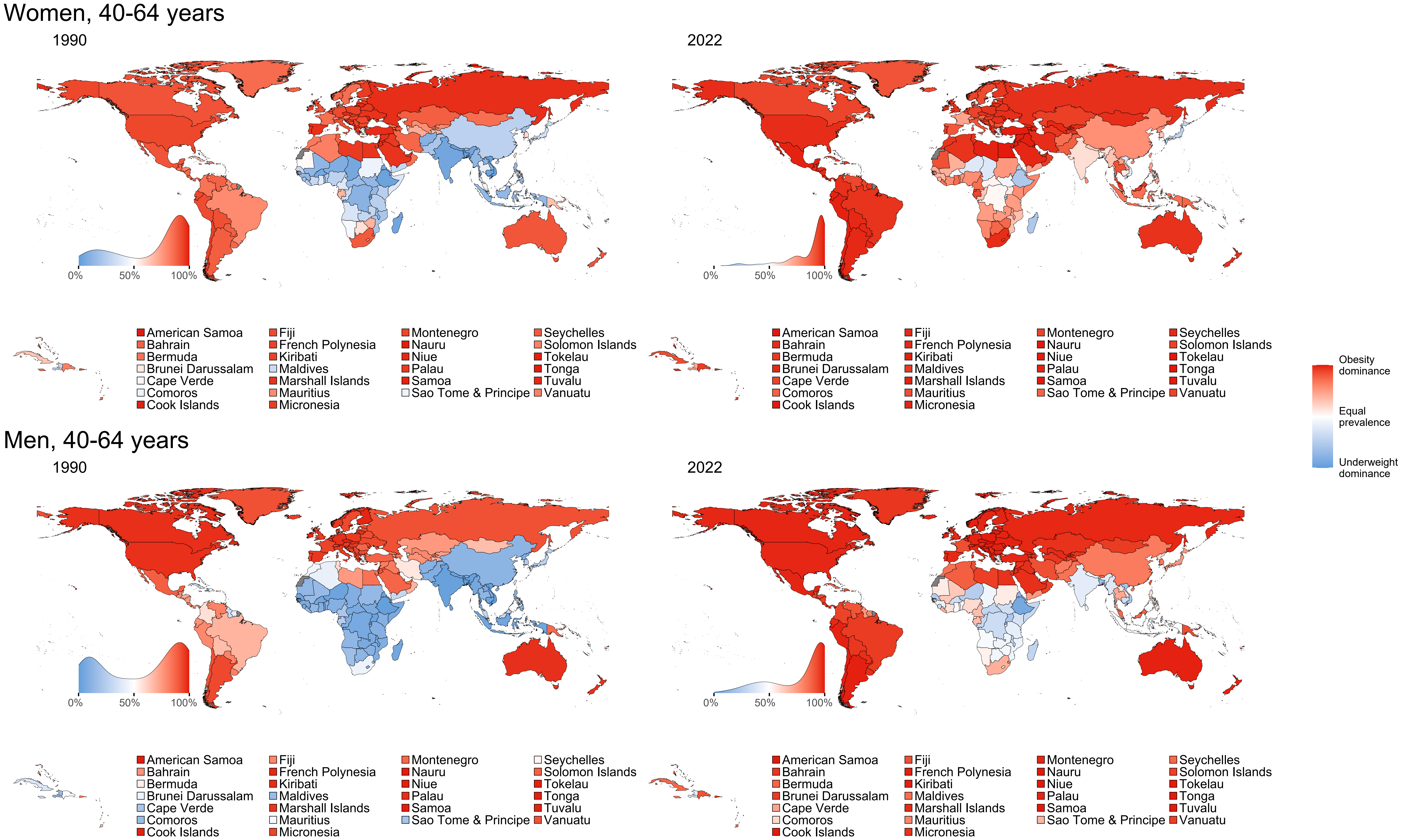

Women, 40-64 years
1990
2022
0%
50%
100%
American Samoa
Bahrain
Bermuda
Brunei Darussalam
Cape Verde
Comoros
Cook Islands
Fiji
French Polynesia
Kiribati
Maldives
Marshall Islands
Mauritius
Micronesia
Montenegro
Nauru
Niue
Palau
Samoa
Sao Tome & Principe
Seychelles
Solomon Islands
Tokelau
Tonga
Tuvalu
Vanuatu
Men, 40-64 years
Obesity dominance
Equal prevalence
Underweight dominance

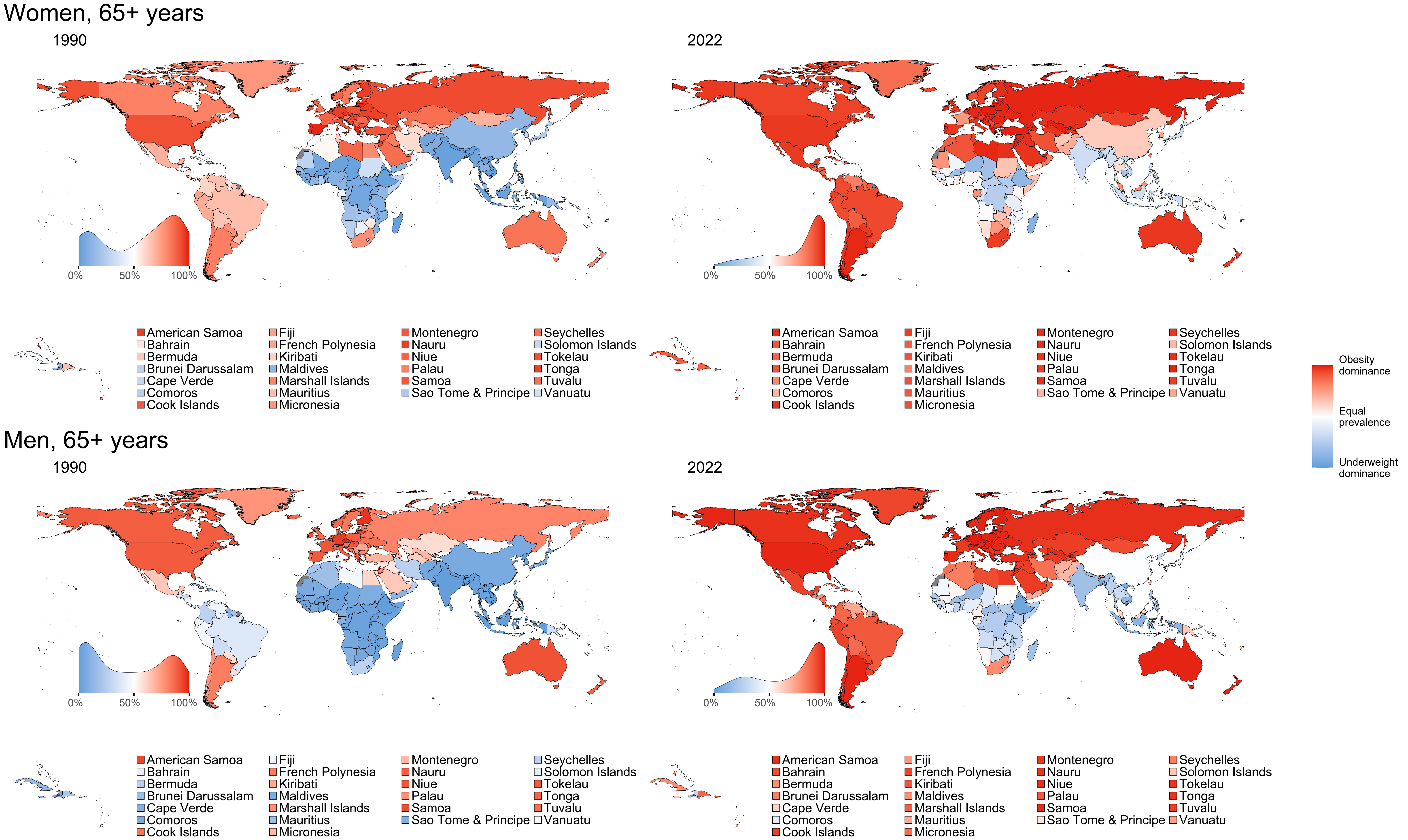

Women, 65+ years
1990
0%
50%
100%
American Samoa
Bahrain
Bermuda
Brunei Darussalam
Cape Verde
Comoros
Cook Islands
Fiji
French Polynesia
Kiribati
Maldives
Marshall Islands
Mauritius
Micronesia
Montenegro
Nauru
Niue
Palau
Samoa
Sao Tome & Principe
Seychelles
Solomon Islands
Tokelau
Tonga
Tuvalu
Vanuatu
2022
0%
50%
100%
American Samoa
Bahrain
Bermuda
Brunei Darussalam
Cape Verde
Comoros
Cook Islands
Fiji
French Polynesia
Kiribati
Maldives
Marshall Islands
Mauritius
Micronesia
Montenegro
Nauru
Niue
Palau
Samoa
Sao Tome & Principe
Seychelles
Solomon Islands
Tokelau
Tonga
Tuvalu
Vanuatu
Obesity dominance
Equal prevalence
Underweight dominance
Men, 65+ years
1990
0%
50%
100%
American Samoa
Bahrain
Bermuda
Brunei Darussalam
Cape Verde
Comoros
Cook Islands
Fiji
French Polynesia
Kiribati
Maldives
Marshall Islands
Mauritius
Micronesia
Montenegro
Nauru
Niue
Palau
Samoa
Sao Tome & Principe
Seychelles
Solomon Islands
Tokelau
Tonga
Tuvalu
Vanuatu
2022
0%
50%
100%
American Samoa
Bahrain
Bermuda
Brunei Darussalam
Cape Verde
Comoros
Cook Islands
Fiji
French Polynesia
Kiribati
Maldives
Marshall Islands
Mauritius
Micronesia
Montenegro
Nauru
Niue
Palau
Samoa
Sao Tome & Principe
Seychelles
Solomon Islands
Tokelau
Tonga
Tuvalu
Vanuatu

**Appendix Figure 12.** Change in age-standardised underweight, obesity, combined burden, and the proportion of combined burden composed of obesity from 1990 to 2022, in relation to the uncertainty of the change measured by posterior standard deviation, for adults.

Each point shows one country. Points that are filled have posterior probability (PP) ≥0.80 of the observed change being a true decrease or increase. If neither an increase nor a decrease was detected at PP = 0.80 the point is hollow.

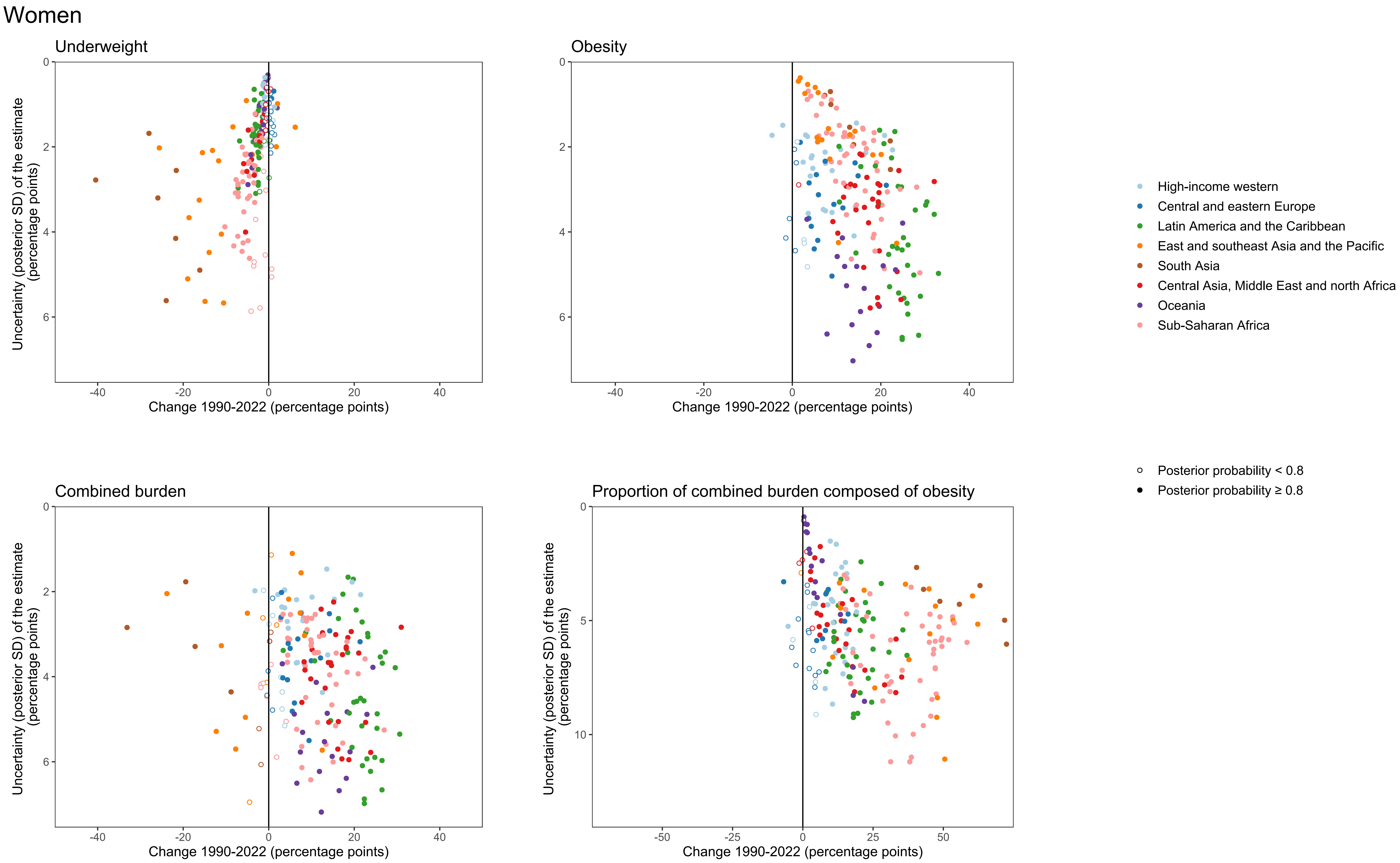

Women
Underweight
Obesity
Combined burden
Proportion of combined burden composed of obesity
Uncertainty (posterior SD) of the estimate (percentage points)
Change 1990-2022 (percentage points)
High-income western
Central and eastern Europe
Latin America and the Caribbean
East and southeast Asia and the Pacific
South Asia
Central Asia, Middle East and north Africa
Oceania
Sub-Saharan Africa
Posterior probability < 0.8
Posterior probability ≥ 0.8

Men

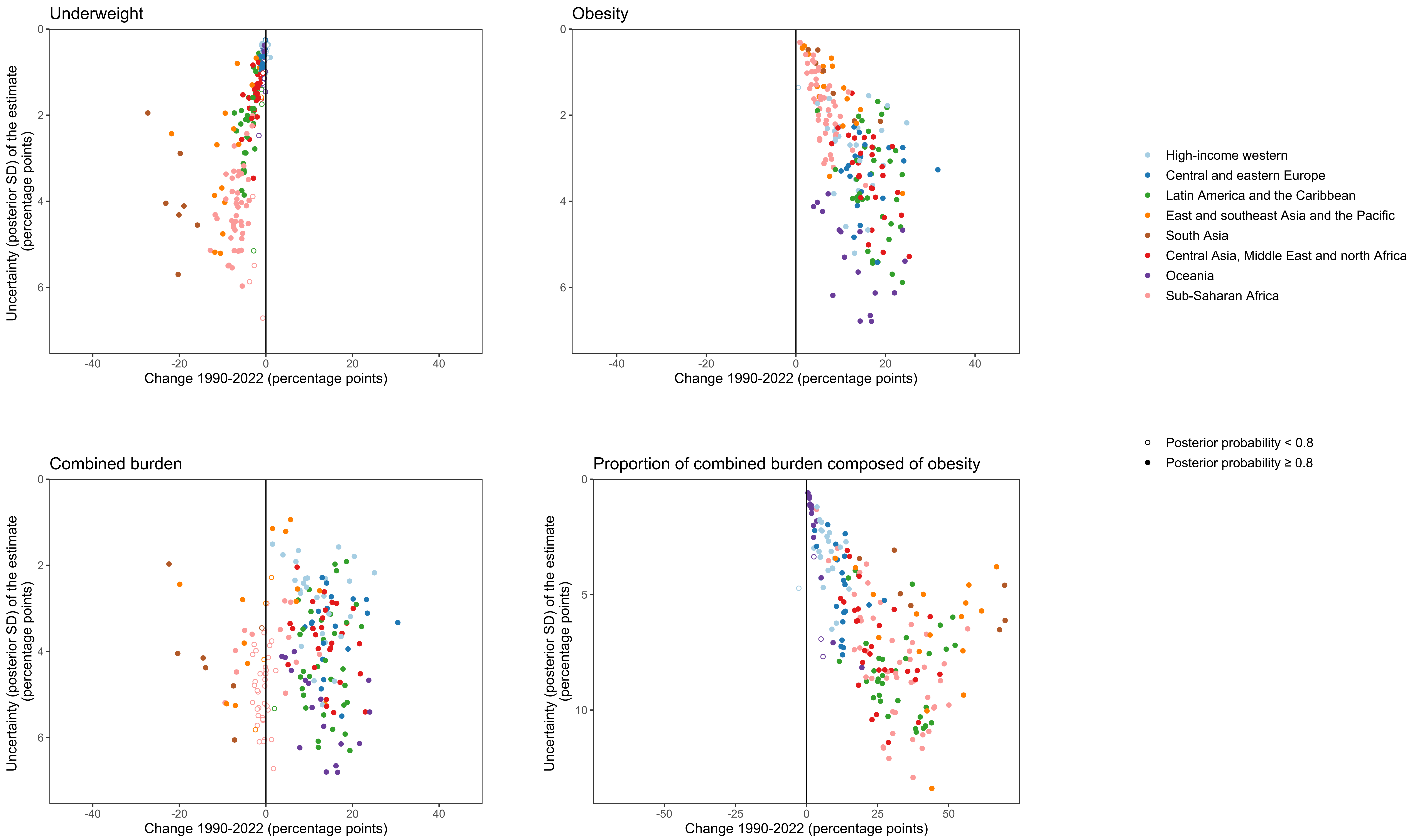
Underweight
Obesity
Combined burden
Proportion of combined burden composed of obesity
Uncertainty (posterior SD) of the estimate (percentage points)
Change 1990-2022 (percentage points)
0
2
4
6
10
5
-40
-20
0
20
40
-50
-25
25
50
High-income western
Central and eastern Europe
Latin America and the Caribbean
East and southeast Asia and the Pacific
South Asia
Central Asia, Middle East and north Africa
Oceania
Sub-Saharan Africa
Posterior probability < 0.8
Posterior probability ≥ 0.8

**Appendix Figure 13.** Contributions of the change in age-standardised underweight and obesity prevalence to their combined prevalence for adults, by age group.

The blue and red bars show the change in age-standardised prevalence of underweight and obesity respectively, and the grey points show the change in the age-standardised combined prevalence. The numbers in brackets after each country's name show the total number of data sources and the number of nationally representative data sources, respectively. Prevalence was age-standardised within each age group presented.

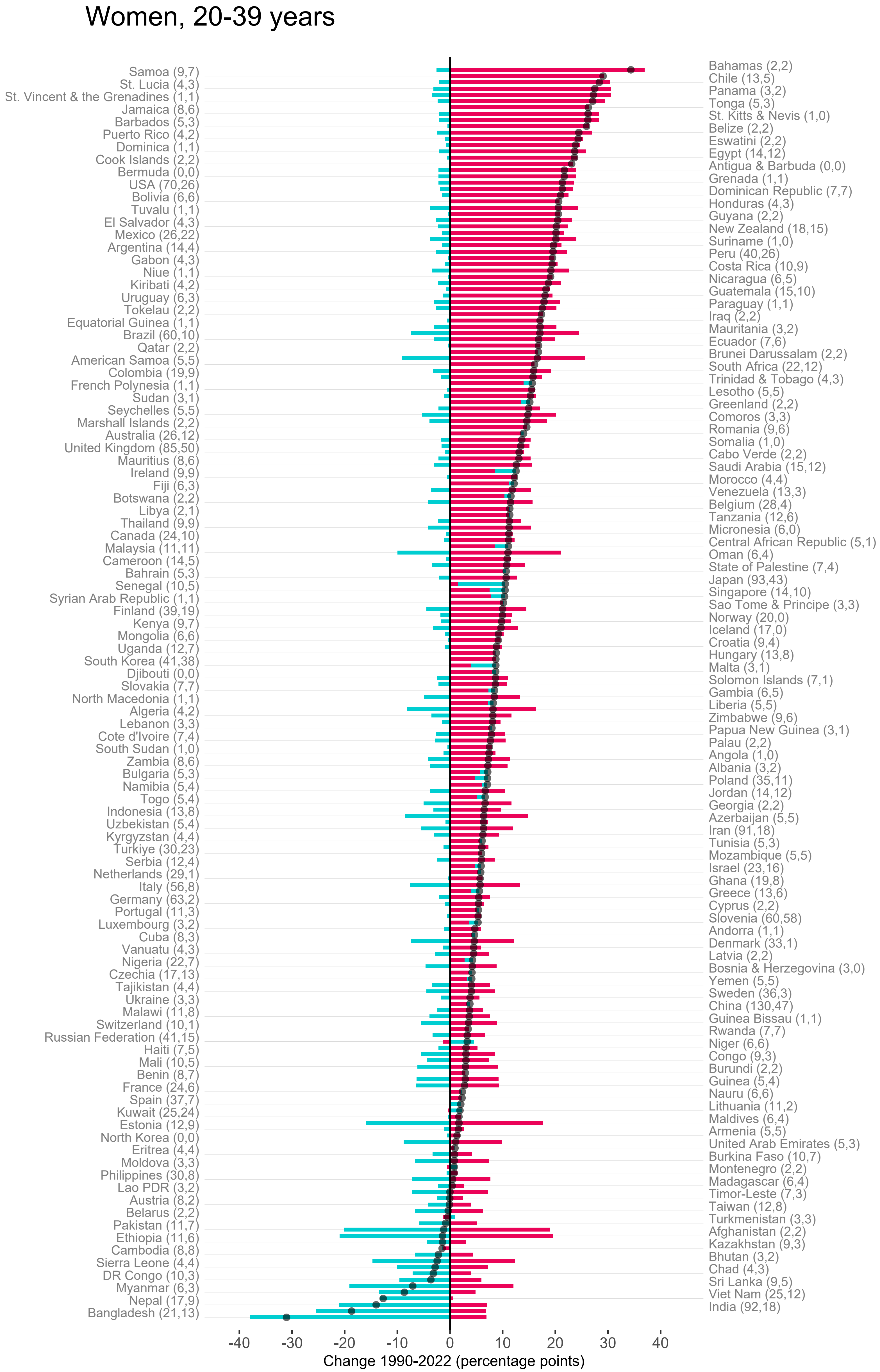
Women, 20-39 years
Samoa (9,7)
St. Lucia (4,3)
St. Vincent & the Grenadines (1,1)
Jamaica (8,6)
Barbados (5,3)
Puerto Rico (4,2)
Dominica (1,1)
Cook Islands (2,2)
Bermuda (0,0)
USA (70,26)
Bolivia (6,6)
Tuvalu (1,1)
El Salvador (4,3)
Mexico (26,22)
Argentina (14,4)
Gabon (4,3)
Niue (1,1)
Kiribati (4,2)
Uruguay (6,3)
Tokelau (2,2)
Equatorial Guinea (1,1)
Brazil (60,10)
Qatar (2,2)
American Samoa (5,5)
Colombia (19,9)
French Polynesia (1,1)
Sudan (3,1)
Seychelles (5,5)
Marshall Islands (2,2)
Australia (26,12)
United Kingdom (85,50)
Mauritius (8,6)
Ireland (9,9)
Fiji (6,3)
Botswana (2,2)
Libya (2,1)
Thailand (9,9)
Canada (24,10)
Malaysia (11,11)
Cameroon (14,5)
Bahrain (5,3)
Senegal (10,5)
Syrian Arab Republic (1,1)
Finland (39,19)
Kenya (9,7)
Mongolia (6,6)
Uganda (12,7)
South Korea (41,38)
Djibouti (0,0)
Slovakia (7,7)
North Macedonia (1,1)
Algeria (4,2)
Lebanon (3,3)
Cote d'Ivoire (7,4)
South Sudan (1,0)
Zambia (8,6)
Bulgaria (5,3)
Namibia (5,4)
Togo (5,4)
Indonesia (13,8)
Uzbekistan (5,4)
Kyrgyzstan (4,4)
Turkiye (30,23)
Serbia (12,4)
Netherlands (29,1)
Italy (56,8)
Germany (63,2)
Portugal (11,3)
Luxembourg (3,2)
Cuba (8,3)
Vanuatu (4,3)
Nigeria (22,7)
Czechia (17,13)
Tajikistan (4,4)
Ukraine (3,3)
Malawi (11,8)
Switzerland (10,1)
Russian Federation (41,15)
Haiti (7,5)
Mali (10,5)
Benin (8,7)
France (24,6)
Spain (37,7)
Kuwait (25,24)
Estonia (12,9)
North Korea (0,0)
Eritrea (4,4)
Moldova (3,3)
Philippines (30,8)
Lao PDR (3,2)
Austria (8,2)
Belarus (2,2)
Pakistan (11,7)
Ethiopia (11,6)
Cambodia (8,8)
Sierra Leone (4,4)
DR Congo (10,3)
Myanmar (6,3)
Nepal (17,9)
Bangladesh (21,13)
Bahamas (2,2)
Chile (13,5)
Panama (3,2)
Tonga (5,3)
St. Kitts & Nevis (1,0)
Belize (2,2)
Eswatini (2,2)
Egypt (14,12)
Antigua & Barbuda (0,0)
Grenada (1,1)
Dominican Republic (7,7)
Honduras (4,3)
Guyana (2,2)
New Zealand (18,15)
Suriname (1,0)
Peru (40,26)
Costa Rica (10,9)
Nicaragua (6,5)
Guatemala (15,10)
Paraguay (1,1)
Iraq (2,2)
Mauritania (3,2)
Ecuador (7,6)
Brunei Darussalam (2,2)
South Africa (22,12)
Trinidad & Tobago (4,3)
Lesotho (5,5)
Greenland (2,2)
Comoros (3,3)
Romania (9,6)
Somalia (1,0)
Cabo Verde (2,2)
Saudi Arabia (15,12)
Morocco (4,4)
Venezuela (13,3)
Belgium (28,4)
Tanzania (12,6)
Micronesia (6,0)
Central African Republic (5,1)
Oman (6,4)
State of Palestine (7,4)
Japan (93,43)
Singapore (14,10)
Sao Tome & Principe (3,3)
Norway (20,0)
Iceland (17,0)
Croatia (9,4)
Hungary (13,8)
Malta (3,1)
Solomon Islands (7,1)
Gambia (6,5)
Liberia (5,5)
Zimbabwe (9,6)
Papua New Guinea (3,1)
Palau (2,2)
Angola (1,0)
Albania (3,2)
Poland (35,11)
Jordan (14,12)
Georgia (2,2)
Azerbaijan (5,5)
Iran (91,18)
Tunisia (5,3)
Mozambique (5,5)
Israel (23,16)
Ghana (19,8)
Greece (13,6)
Cyprus (2,2)
Slovenia (60,58)
Andorra (1,1)
Denmark (33,1)
Latvia (2,2)
Bosnia & Herzegovina (3,0)
Yemen (5,5)
Sweden (36,3)
China (130,47)
Guinea Bissau (1,1)
Rwanda (7,7)
Niger (6,6)
Congo (9,3)
Burundi (2,2)
Guinea (5,4)
Nauru (6,6)
Lithuania (11,2)
Maldives (6,4)
Armenia (5,5)
United Arab Emirates (5,3)
Burkina Faso (10,7)
Montenegro (2,2)
Madagascar (6,4)
Timor-Leste (7,3)
Taiwan (12,8)
Turkmenistan (3,3)
Afghanistan (2,2)
Kazakhstan (9,3)
Bhutan (3,2)
Chad (4,3)
Sri Lanka (9,5)
Viet Nam (25,12)
India (92,18)
-40 -30 -20 -10 0 10 20 30 40
Change 1990-2022 (percentage points)

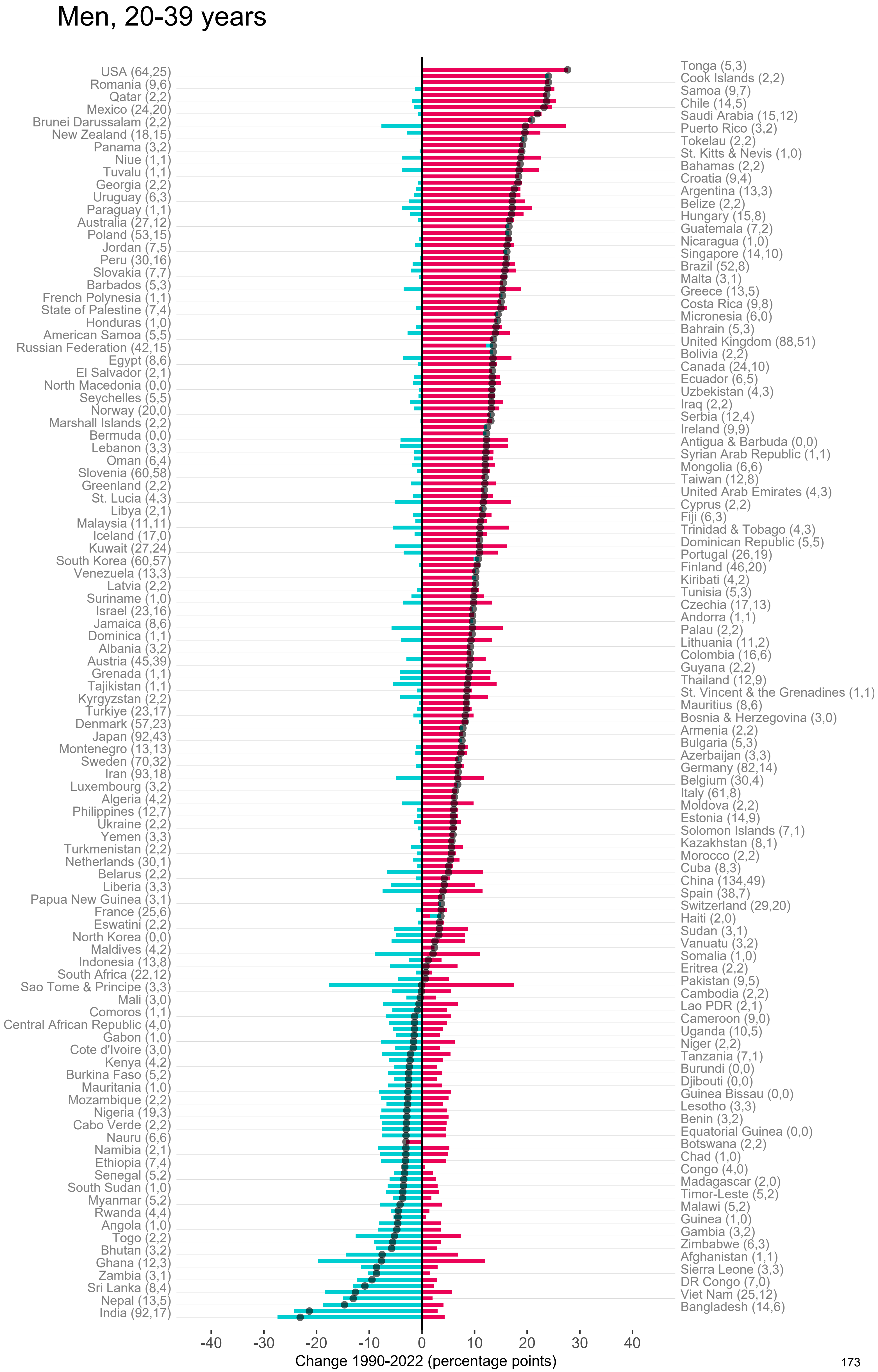
Men, 20-39 years
USA (64,25)
Romania (9,6)
Qatar (2,2)
Mexico (24,20)
Brunei Darussalam (2,2)
New Zealand (18,15)
Panama (3,2)
Niue (1,1)
Tuvalu (1,1)
Georgia (2,2)
Uruguay (6,3)
Paraguay (1,1)
Australia (27,12)
Poland (53,15)
Jordan (7,5)
Peru (30,16)
Slovakia (7,7)
Barbados (5,3)
French Polynesia (1,1)
State of Palestine (7,4)
Honduras (1,0)
American Samoa (5,5)
Russian Federation (42,15)
Egypt (8,6)
El Salvador (2,1)
North Macedonia (0,0)
Seychelles (5,5)
Norway (20,0)
Marshall Islands (2,2)
Bermuda (0,0)
Lebanon (3,3)
Oman (6,4)
Slovenia (60,58)
Greenland (2,2)
St. Lucia (4,3)
Libya (2,1)
Malaysia (11,11)
Iceland (17,0)
Kuwait (27,24)
South Korea (60,57)
Venezuela (13,3)
Latvia (2,2)
Suriname (1,0)
Israel (23,16)
Jamaica (8,6)
Dominica (1,1)
Albania (3,2)
Austria (45,39)
Grenada (1,1)
Tajikistan (1,1)
Kyrgyzstan (2,2)
Turkiye (23,17)
Denmark (57,23)
Japan (92,43)
Montenegro (13,13)
Sweden (70,32)
Iran (93,18)
Luxembourg (3,2)
Algeria (4,2)
Philippines (12,7)
Ukraine (2,2)
Yemen (3,3)
Turkmenistan (2,2)
Netherlands (30,1)
Belarus (2,2)
Liberia (3,3)
Papua New Guinea (3,1)
France (25,6)
Eswatini (2,2)
North Korea (0,0)
Maldives (4,2)
Indonesia (13,8)
South Africa (22,12)
Sao Tome & Principe (3,3)
Mali (3,0)
Comoros (1,1)
Central African Republic (4,0)
Gabon (1,0)
Cote d'Ivoire (3,0)
Kenya (4,2)
Burkina Faso (5,2)
Mauritania (1,0)
Mozambique (2,2)
Nigeria (19,3)
Cabo Verde (2,2)
Nauru (6,6)
Namibia (2,1)
Ethiopia (7,4)
Senegal (5,2)
South Sudan (1,0)
Myanmar (5,2)
Rwanda (4,4)
Angola (1,0)
Togo (2,2)
Bhutan (3,2)
Ghana (12,3)
Zambia (3,1)
Sri Lanka (8,4)
Nepal (13,5)
India (92,17)
Tonga (5,3)
Cook Islands (2,2)
Samoa (9,7)
Chile (14,5)
Saudi Arabia (15,12)
Puerto Rico (3,2)
Tokelau (2,2)
St. Kitts & Nevis (1,0)
Bahamas (2,2)
Croatia (9,4)
Argentina (13,3)
Belize (2,2)
Hungary (15,8)
Guatemala (7,2)
Nicaragua (1,0)
Singapore (14,10)
Brazil (52,8)
Malta (3,1)
Greece (13,5)
Costa Rica (9,8)
Micronesia (6,0)
Bahrain (5,3)
United Kingdom (88,51)
Bolivia (2,2)
Canada (24,10)
Ecuador (6,5)
Uzbekistan (4,3)
Iraq (2,2)
Serbia (12,4)
Ireland (9,9)
Antigua & Barbuda (0,0)
Syrian Arab Republic (1,1)
Mongolia (6,6)
Taiwan (12,8)
United Arab Emirates (4,3)
Cyprus (2,2)
Fiji (6,3)
Trinidad & Tobago (4,3)
Dominican Republic (5,5)
Portugal (26,19)
Finland (46,20)
Kiribati (4,2)
Tunisia (5,3)
Czechia (17,13)
Andorra (1,1)
Palau (2,2)
Lithuania (11,2)
Colombia (16,6)
Guyana (2,2)
Thailand (12,9)
St. Vincent & the Grenadines (1,1)
Mauritius (8,6)
Bosnia & Herzegovina (3,0)
Armenia (2,2)
Bulgaria (5,3)
Azerbaijan (3,3)
Germany (82,14)
Belgium (30,4)
Italy (61,8)
Moldova (2,2)
Estonia (14,9)
Solomon Islands (7,1)
Kazakhstan (8,1)
Morocco (2,2)
Cuba (8,3)
China (134,49)
Spain (38,7)
Switzerland (29,20)
Haiti (2,0)
Sudan (3,1)
Vanuatu (3,2)
Somalia (1,0)
Eritrea (2,2)
Pakistan (9,5)
Cambodia (2,2)
Lao PDR (2,1)
Cameroon (9,0)
Uganda (10,5)
Niger (2,2)
Tanzania (7,1)
Burundi (0,0)
Djibouti (0,0)
Guinea Bissau (0,0)
Lesotho (3,3)
Benin (3,2)
Equatorial Guinea (0,0)
Botswana (2,2)
Chad (1,0)
Congo (4,0)
Madagascar (2,0)
Timor-Leste (5,2)
Malawi (5,2)
Guinea (1,0)
Gambia (3,2)
Zimbabwe (6,3)
Afghanistan (1,1)
Sierra Leone (3,3)
DR Congo (7,0)
Viet Nam (25,12)
Bangladesh (14,6)
-40 -30 -20 -10 0 10 20 30 40
Change 1990-2022 (percentage points)

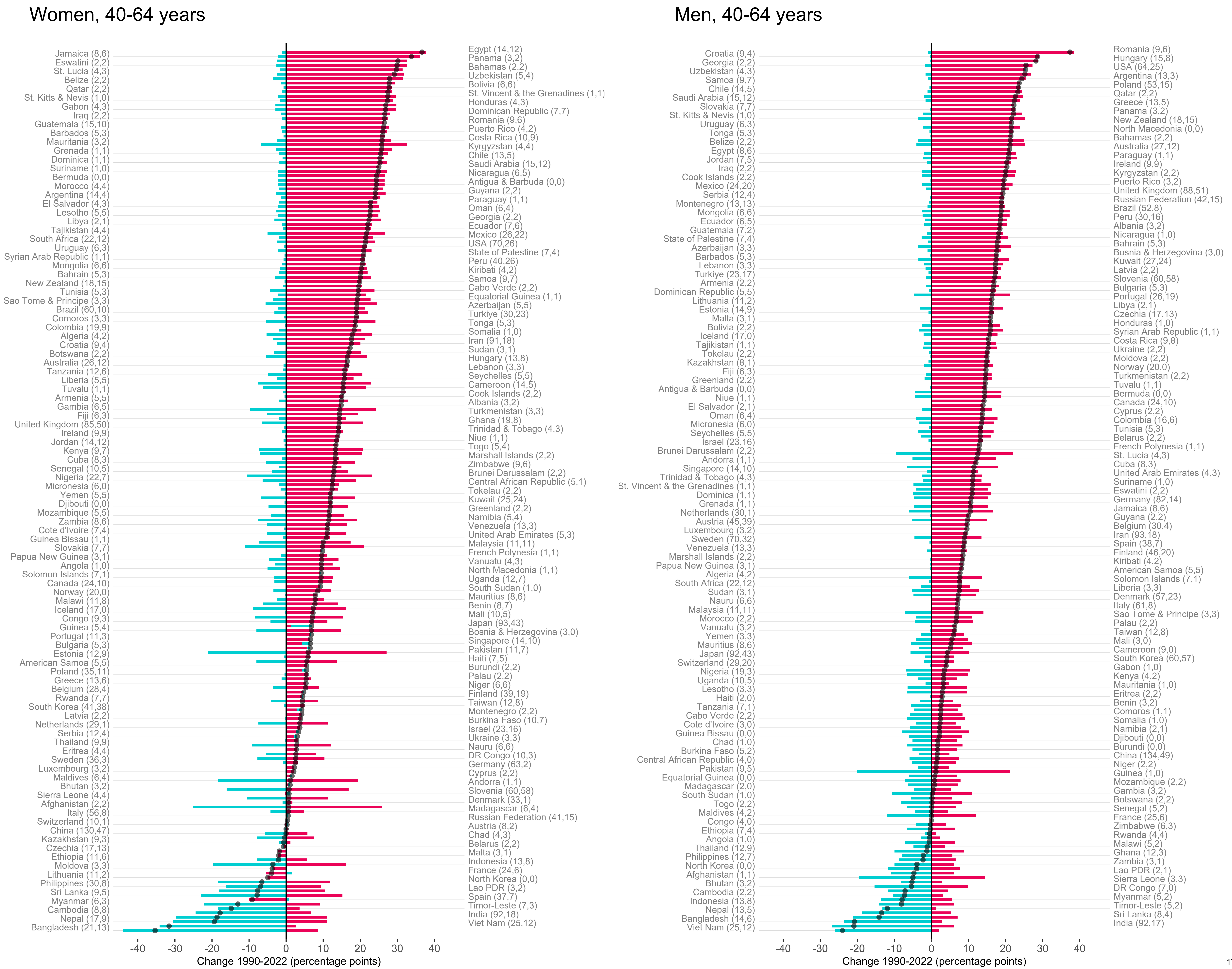
Women, 40-64 years
Jamaica (8,6)
Eswatini (2,2)
St. Lucia (4,3)
Belize (2,2)
Qatar (2,2)
St. Kitts & Nevis (1,0)
Gabon (4,3)
Iraq (2,2)
Guatemala (15,10)
Barbados (5,3)
Mauritania (3,2)
Grenada (1,1)
Dominica (1,1)
Suriname (1,0)
Bermuda (0,0)
Morocco (4,4)
Argentina (14,4)
El Salvador (4,3)
Lesotho (5,5)
Libya (2,1)
Tajikistan (4,4)
South Africa (22,12)
Uruguay (6,3)
Syrian Arab Republic (1,1)
Mongolia (6,6)
Bahrain (5,3)
New Zealand (18,15)
Tunisia (5,3)
Sao Tome & Principe (3,3)
Brazil (60,10)
Comoros (3,3)
Colombia (19,9)
Algeria (4,2)
Croatia (9,4)
Botswana (2,2)
Australia (26,12)
Tanzania (12,6)
Liberia (5,5)
Tuvalu (1,1)
Armenia (5,5)
Gambia (6,5)
Fiji (6,3)
United Kingdom (85,50)
Ireland (9,9)
Jordan (14,12)
Kenya (9,7)
Cuba (8,3)
Senegal (10,5)
Nigeria (22,7)
Micronesia (6,0)
Yemen (5,5)
Djibouti (0,0)
Mozambique (5,5)
Zambia (8,6)
Cote d'Ivoire (7,4)
Guinea Bissau (1,1)
Slovakia (7,7)
Papua New Guinea (3,1)
Angola (1,0)
Solomon Islands (7,1)
Canada (24,10)
Norway (20,0)
Malawi (11,8)
Iceland (17,0)
Congo (9,3)
Guinea (5,4)
Portugal (11,3)
Bulgaria (5,3)
Estonia (12,9)
American Samoa (5,5)
Poland (35,11)
Greece (13,6)
Belgium (28,4)
Rwanda (7,7)
South Korea (41,38)
Latvia (2,2)
Netherlands (29,1)
Serbia (12,4)
Thailand (9,9)
Eritrea (4,4)
Sweden (36,3)
Luxembourg (3,2)
Maldives (6,4)
Bhutan (3,2)
Sierra Leone (4,4)
Afghanistan (2,2)
Italy (56,8)
Switzerland (10,1)
China (130,47)
Kazakhstan (9,3)
Czechia (17,13)
Ethiopia (11,6)
Moldova (3,3)
Lithuania (11,2)
Philippines (30,8)
Sri Lanka (9,5)
Myanmar (6,3)
Cambodia (8,8)
Nepal (17,9)
Bangladesh (21,13)
Egypt (14,12)
Panama (3,2)
Bahamas (2,2)
Uzbekistan (5,4)
Bolivia (6,6)
St. Vincent & the Grenadines (1,1)
Honduras (4,3)
Dominican Republic (7,7)
Romania (9,6)
Puerto Rico (4,2)
Costa Rica (10,9)
Kyrgyzstan (4,4)
Chile (13,5)
Saudi Arabia (15,12)
Nicaragua (6,5)
Antigua & Barbuda (0,0)
Guyana (2,2)
Paraguay (1,1)
Oman (6,4)
Georgia (2,2)
Ecuador (7,6)
Mexico (26,22)
USA (70,26)
State of Palestine (7,4)
Peru (40,26)
Kiribati (4,2)
Samoa (9,7)
Cabo Verde (2,2)
Equatorial Guinea (1,1)
Azerbaijan (5,5)
Turkiye (30,23)
Tonga (5,3)
Somalia (1,0)
Iran (91,18)
Sudan (3,1)
Hungary (13,8)
Lebanon (3,3)
Seychelles (5,5)
Cameroon (14,5)
Cook Islands (2,2)
Albania (3,2)
Turkmenistan (3,3)
Ghana (19,8)
Trinidad & Tobago (4,3)
Niue (1,1)
Togo (5,4)
Marshall Islands (2,2)
Zimbabwe (9,6)
Brunei Darussalam (2,2)
Central African Republic (5,1)
Tokelau (2,2)
Kuwait (25,24)
Greenland (2,2)
Namibia (5,4)
Venezuela (13,3)
United Arab Emirates (5,3)
Malaysia (11,11)
French Polynesia (1,1)
Vanuatu (4,3)
North Macedonia (1,1)
Uganda (12,7)
South Sudan (1,0)
Mauritius (8,6)
Benin (8,7)
Mali (10,5)
Japan (93,43)
Bosnia & Herzegovina (3,0)
Singapore (14,10)
Pakistan (11,7)
Haiti (7,5)
Burundi (2,2)
Palau (2,2)
Niger (6,6)
Finland (39,19)
Taiwan (12,8)
Montenegro (2,2)
Burkina Faso (10,7)
Israel (23,16)
Ukraine (3,3)
Nauru (6,6)
DR Congo (10,3)
Germany (63,2)
Cyprus (2,2)
Andorra (1,1)
Slovenia (60,58)
Denmark (33,1)
Madagascar (6,4)
Russian Federation (41,15)
Austria (8,2)
Chad (4,3)
Belarus (2,2)
Malta (3,1)
Indonesia (13,8)
France (24,6)
North Korea (0,0)
Lao PDR (3,2)
Spain (37,7)
Timor-Leste (7,3)
India (92,18)
Viet Nam (25,12)
-40
-30
-20
-10
0
10
20
30
40
Change 1990-2022 (percentage points)
Men, 40-64 years
Croatia (9,4)
Georgia (2,2)
Uzbekistan (4,3)
Samoa (9,7)
Chile (14,5)
Saudi Arabia (15,12)
Slovakia (7,7)
St. Kitts & Nevis (1,0)
Uruguay (6,3)
Tonga (5,3)
Belize (2,2)
Egypt (8,6)
Jordan (7,5)
Iraq (2,2)
Cook Islands (2,2)
Mexico (24,20)
Serbia (12,4)
Montenegro (13,13)
Mongolia (6,6)
Ecuador (6,5)
Guatemala (7,2)
State of Palestine (7,4)
Azerbaijan (3,3)
Barbados (5,3)
Lebanon (3,3)
Turkiye (23,17)
Armenia (2,2)
Dominican Republic (5,5)
Lithuania (11,2)
Estonia (14,9)
Malta (3,1)
Bolivia (2,2)
Iceland (17,0)
Tajikistan (1,1)
Tokelau (2,2)
Kazakhstan (8,1)
Fiji (6,3)
Greenland (2,2)
Antigua & Barbuda (0,0)
Niue (1,1)
El Salvador (2,1)
Oman (6,4)
Micronesia (6,0)
Seychelles (5,5)
Israel (23,16)
Brunei Darussalam (2,2)
Andorra (1,1)
Singapore (14,10)
Trinidad & Tobago (4,3)
St. Vincent & the Grenadines (1,1)
Dominica (1,1)
Grenada (1,1)
Netherlands (30,1)
Austria (45,39)
Luxembourg (3,2)
Sweden (70,32)
Venezuela (13,3)
Marshall Islands (2,2)
Papua New Guinea (3,1)
Algeria (4,2)
South Africa (22,12)
Sudan (3,1)
Nauru (6,6)
Malaysia (11,11)
Morocco (2,2)
Vanuatu (3,2)
Yemen (3,3)
Mauritius (8,6)
Japan (92,43)
Switzerland (29,20)
Nigeria (19,3)
Uganda (10,5)
Lesotho (3,3)
Haiti (2,0)
Tanzania (7,1)
Cabo Verde (2,2)
Cote d'Ivoire (3,0)
Guinea Bissau (0,0)
Chad (1,0)
Burkina Faso (5,2)
Central African Republic (4,0)
Pakistan (9,5)
Equatorial Guinea (0,0)
Madagascar (2,0)
South Sudan (1,0)
Togo (2,2)
Maldives (4,2)
Congo (4,0)
Ethiopia (7,4)
Angola (1,0)
Thailand (12,9)
Philippines (12,7)
North Korea (0,0)
Afghanistan (1,1)
Bhutan (3,2)
Cambodia (2,2)
Indonesia (13,8)
Nepal (13,5)
Bangladesh (14,6)
Viet Nam (25,12)
Romania (9,6)
Hungary (15,8)
USA (64,25)
Argentina (13,3)
Poland (53,15)
Qatar (2,2)
Greece (13,5)
Panama (3,2)
New Zealand (18,15)
North Macedonia (0,0)
Bahamas (2,2)
Australia (27,12)
Paraguay (1,1)
Ireland (9,9)
Kyrgyzstan (2,2)
Puerto Rico (3,2)
United Kingdom (88,51)
Russian Federation (42,15)
Brazil (52,8)
Peru (30,16)
Albania (3,2)
Nicaragua (1,0)
Bahrain (5,3)
Bosnia & Herzegovina (3,0)
Kuwait (27,24)
Latvia (2,2)
Slovenia (60,58)
Bulgaria (5,3)
Portugal (26,19)
Libya (2,1)
Czechia (17,13)
Honduras (1,0)
Syrian Arab Republic (1,1)
Costa Rica (9,8)
Ukraine (2,2)
Moldova (2,2)
Norway (20,0)
Turkmenistan (2,2)
Tuvalu (1,1)
Bermuda (0,0)
Canada (24,10)
Cyprus (2,2)
Colombia (16,6)
Tunisia (5,3)
Belarus (2,2)
French Polynesia (1,1)
St. Lucia (4,3)
Cuba (8,3)
United Arab Emirates (4,3)
Suriname (1,0)
Eswatini (2,2)
Germany (82,14)
Jamaica (8,6)
Guyana (2,2)
Belgium (30,4)
Iran (93,18)
Spain (38,7)
Finland (46,20)
Kiribati (4,2)
American Samoa (5,5)
Solomon Islands (7,1)
Liberia (3,3)
Denmark (57,23)
Italy (61,8)
Sao Tome & Principe (3,3)
Palau (2,2)
Taiwan (12,8)
Mali (3,0)
Cameroon (9,0)
South Korea (60,57)
Gabon (1,0)
Kenya (4,2)
Mauritania (1,0)
Eritrea (2,2)
Benin (3,2)
Comoros (1,1)
Somalia (1,0)
Namibia (2,1)
Djibouti (0,0)
Burundi (0,0)
China (134,49)
Niger (2,2)
Guinea (1,0)
Mozambique (2,2)
Gambia (3,2)
Botswana (2,2)
Senegal (5,2)
France (25,6)
Zimbabwe (6,3)
Rwanda (4,4)
Malawi (5,2)
Ghana (12,3)
Zambia (3,1)
Lao PDR (2,1)
Sierra Leone (3,3)
DR Congo (7,0)
Myanmar (5,2)
Timor-Leste (5,2)
Sri Lanka (8,4)
India (92,17)
-40
-30
-20
-10
0
10
20
30
40
Change 1990-2022 (percentage points)

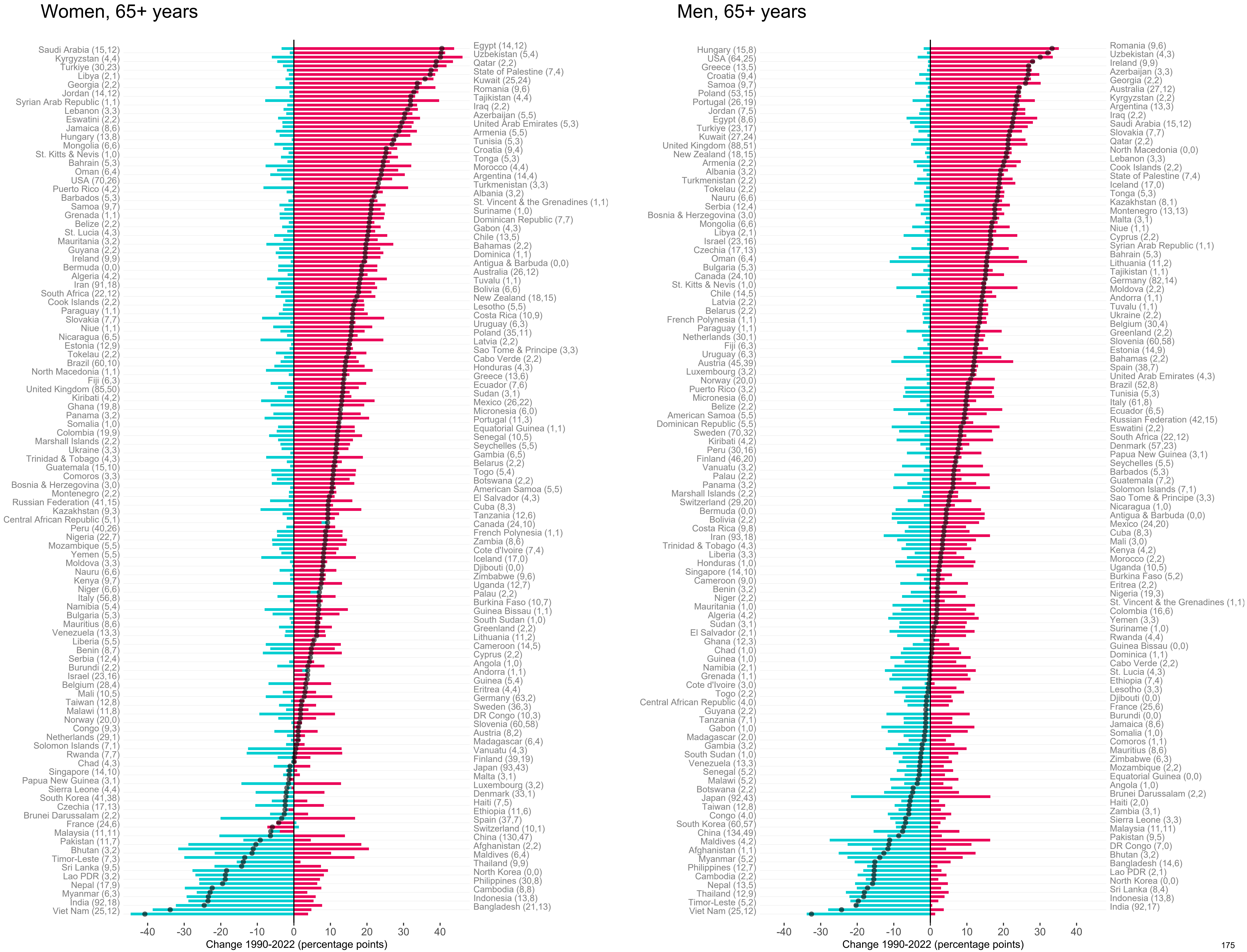
Women, 65+ years
Saudi Arabia (15,12)
Kyrgyzstan (4,4)
Turkiye (30,23)
Libya (2,1)
Georgia (2,2)
Jordan (14,12)
Syrian Arab Republic (1,1)
Lebanon (3,3)
Eswatini (2,2)
Jamaica (8,6)
Hungary (13,8)
Mongolia (6,6)
St. Kitts & Nevis (1,0)
Bahrain (5,3)
Oman (6,4)
USA (70,26)
Puerto Rico (4,2)
Barbados (5,3)
Samoa (9,7)
Grenada (1,1)
Belize (2,2)
St. Lucia (4,3)
Mauritania (3,2)
Guyana (2,2)
Ireland (9,9)
Bermuda (0,0)
Algeria (4,2)
Iran (91,18)
South Africa (22,12)
Cook Islands (2,2)
Paraguay (1,1)
Slovakia (7,7)
Niue (1,1)
Nicaragua (6,5)
Estonia (12,9)
Tokelau (2,2)
Brazil (60,10)
North Macedonia (1,1)
Fiji (6,3)
United Kingdom (85,50)
Kiribati (4,2)
Ghana (19,8)
Panama (3,2)
Somalia (1,0)
Colombia (19,9)
Marshall Islands (2,2)
Ukraine (3,3)
Trinidad & Tobago (4,3)
Guatemala (15,10)
Comoros (3,3)
Bosnia & Herzegovina (3,0)
Montenegro (2,2)
Russian Federation (41,15)
Kazakhstan (9,3)
Central African Republic (5,1)
Peru (40,26)
Nigeria (22,7)
Mozambique (5,5)
Yemen (5,5)
Moldova (3,3)
Nauru (6,6)
Kenya (9,7)
Niger (6,6)
Italy (56,8)
Namibia (5,4)
Bulgaria (5,3)
Mauritius (8,6)
Venezuela (13,3)
Liberia (5,5)
Benin (8,7)
Serbia (12,4)
Burundi (2,2)
Israel (23,16)
Belgium (28,4)
Mali (10,5)
Taiwan (12,8)
Malawi (11,8)
Norway (20,0)
Congo (9,3)
Netherlands (29,1)
Solomon Islands (7,1)
Rwanda (7,7)
Chad (4,3)
Singapore (14,10)
Papua New Guinea (3,1)
Sierra Leone (4,4)
South Korea (41,38)
Czechia (17,13)
Brunei Darussalam (2,2)
France (24,6)
Malaysia (11,11)
Pakistan (11,7)
Bhutan (3,2)
Timor-Leste (7,3)
Sri Lanka (9,5)
Lao PDR (3,2)
Nepal (17,9)
Myanmar (6,3)
India (92,18)
Viet Nam (25,12)
Egypt (14,12)
Uzbekistan (5,4)
Qatar (2,2)
State of Palestine (7,4)
Kuwait (25,24)
Romania (9,6)
Tajikistan (4,4)
Iraq (2,2)
Azerbaijan (5,5)
United Arab Emirates (5,3)
Armenia (5,5)
Tunisia (5,3)
Croatia (9,4)
Tonga (5,3)
Morocco (4,4)
Argentina (14,4)
Turkmenistan (3,3)
Albania (3,2)
St. Vincent & the Grenadines (1,1)
Suriname (1,0)
Dominican Republic (7,7)
Gabon (4,3)
Chile (13,5)
Bahamas (2,2)
Dominica (1,1)
Antigua & Barbuda (0,0)
Australia (26,12)
Tuvalu (1,1)
Bolivia (6,6)
New Zealand (18,15)
Lesotho (5,5)
Costa Rica (10,9)
Uruguay (6,3)
Poland (35,11)
Latvia (2,2)
Sao Tome & Principe (3,3)
Cabo Verde (2,2)
Honduras (4,3)
Greece (13,6)
Ecuador (7,6)
Sudan (3,1)
Mexico (26,22)
Micronesia (6,0)
Portugal (11,3)
Equatorial Guinea (1,1)
Senegal (10,5)
Seychelles (5,5)
Gambia (6,5)
Belarus (2,2)
Togo (5,4)
Botswana (2,2)
American Samoa (5,5)
El Salvador (4,3)
Cuba (8,3)
Tanzania (12,6)
Canada (24,10)
French Polynesia (1,1)
Zambia (8,6)
Cote d'Ivoire (7,4)
Iceland (17,0)
Djibouti (0,0)
Zimbabwe (9,6)
Uganda (12,7)
Palau (2,2)
Burkina Faso (10,7)
Guinea Bissau (1,1)
South Sudan (1,0)
Greenland (2,2)
Lithuania (11,2)
Cameroon (14,5)
Cyprus (2,2)
Angola (1,0)
Andorra (1,1)
Guinea (5,4)
Eritrea (4,4)
Germany (63,2)
Sweden (36,3)
DR Congo (10,3)
Slovenia (60,58)
Austria (8,2)
Madagascar (6,4)
Vanuatu (4,3)
Finland (39,19)
Japan (93,43)
Malta (3,1)
Luxembourg (3,2)
Denmark (33,1)
Haiti (7,5)
Ethiopia (11,6)
Spain (37,7)
Switzerland (10,1)
China (130,47)
Afghanistan (2,2)
Maldives (6,4)
Thailand (9,9)
North Korea (0,0)
Philippines (30,8)
Cambodia (8,8)
Indonesia (13,8)
Bangladesh (21,13)
-40
-30
-20
-10
0
10
20
30
40
Change 1990-2022 (percentage points)
Men, 65+ years
Hungary (15,8)
USA (64,25)
Greece (13,5)
Croatia (9,4)
Samoa (9,7)
Poland (53,15)
Portugal (26,19)
Jordan (7,5)
Egypt (8,6)
Turkiye (23,17)
Kuwait (27,24)
United Kingdom (88,51)
New Zealand (18,15)
Armenia (2,2)
Albania (3,2)
Turkmenistan (2,2)
Tokelau (2,2)
Nauru (6,6)
Serbia (12,4)
Bosnia & Herzegovina (3,0)
Mongolia (6,6)
Libya (2,1)
Israel (23,16)
Czechia (17,13)
Oman (6,4)
Bulgaria (5,3)
Canada (24,10)
St. Kitts & Nevis (1,0)
Chile (14,5)
Latvia (2,2)
Belarus (2,2)
French Polynesia (1,1)
Paraguay (1,1)
Netherlands (30,1)
Fiji (6,3)
Uruguay (6,3)
Austria (45,39)
Luxembourg (3,2)
Norway (20,0)
Puerto Rico (3,2)
Micronesia (6,0)
Belize (2,2)
American Samoa (5,5)
Dominican Republic (5,5)
Sweden (70,32)
Kiribati (4,2)
Peru (30,16)
Finland (46,20)
Vanuatu (3,2)
Palau (2,2)
Panama (3,2)
Marshall Islands (2,2)
Switzerland (29,20)
Bermuda (0,0)
Bolivia (2,2)
Costa Rica (9,8)
Iran (93,18)
Trinidad & Tobago (4,3)
Liberia (3,3)
Honduras (1,0)
Singapore (14,10)
Cameroon (9,0)
Benin (3,2)
Niger (2,2)
Mauritania (1,0)
Algeria (4,2)
Sudan (3,1)
El Salvador (2,1)
Ghana (12,3)
Chad (1,0)
Guinea (1,0)
Namibia (2,1)
Grenada (1,1)
Cote d'Ivoire (3,0)
Togo (2,2)
Central African Republic (4,0)
Guyana (2,2)
Tanzania (7,1)
Gabon (1,0)
Madagascar (2,0)
Gambia (3,2)
South Sudan (1,0)
Venezuela (13,3)
Senegal (5,2)
Malawi (5,2)
Botswana (2,2)
Japan (92,43)
Taiwan (12,8)
Congo (4,0)
South Korea (60,57)
China (134,49)
Maldives (4,2)
Afghanistan (1,1)
Myanmar (5,2)
Philippines (12,7)
Cambodia (2,2)
Nepal (13,5)
Thailand (12,9)
Timor-Leste (5,2)
Viet Nam (25,12)
Romania (9,6)
Uzbekistan (4,3)
Ireland (9,9)
Azerbaijan (3,3)
Georgia (2,2)
Australia (27,12)
Kyrgyzstan (2,2)
Argentina (13,3)
Iraq (2,2)
Saudi Arabia (15,12)
Slovakia (7,7)
Qatar (2,2)
North Macedonia (0,0)
Lebanon (3,3)
Cook Islands (2,2)
State of Palestine (7,4)
Iceland (17,0)
Tonga (5,3)
Kazakhstan (8,1)
Montenegro (13,13)
Malta (3,1)
Niue (1,1)
Cyprus (2,2)
Syrian Arab Republic (1,1)
Bahrain (5,3)
Lithuania (11,2)
Tajikistan (1,1)
Germany (82,14)
Moldova (2,2)
Andorra (1,1)
Tuvalu (1,1)
Ukraine (2,2)
Belgium (30,4)
Greenland (2,2)
Slovenia (60,58)
Estonia (14,9)
Bahamas (2,2)
Spain (38,7)
United Arab Emirates (4,3)
Brazil (52,8)
Tunisia (5,3)
Italy (61,8)
Ecuador (6,5)
Russian Federation (42,15)
Eswatini (2,2)
South Africa (22,12)
Denmark (57,23)
Papua New Guinea (3,1)
Seychelles (5,5)
Barbados (5,3)
Guatemala (7,2)
Solomon Islands (7,1)
Sao Tome & Principe (3,3)
Nicaragua (1,0)
Antigua & Barbuda (0,0)
Mexico (24,20)
Cuba (8,3)
Mali (3,0)
Kenya (4,2)
Morocco (2,2)
Uganda (10,5)
Burkina Faso (5,2)
Eritrea (2,2)
Nigeria (19,3)
St. Vincent & the Grenadines (1,1)
Colombia (16,6)
Yemen (3,3)
Suriname (1,0)
Rwanda (4,4)
Guinea Bissau (0,0)
Dominica (1,1)
Cabo Verde (2,2)
St. Lucia (4,3)
Ethiopia (7,4)
Lesotho (3,3)
Djibouti (0,0)
France (25,6)
Burundi (0,0)
Jamaica (8,6)
Somalia (1,0)
Comoros (1,1)
Mauritius (8,6)
Zimbabwe (6,3)
Mozambique (2,2)
Equatorial Guinea (0,0)
Angola (1,0)
Brunei Darussalam (2,2)
Haiti (2,0)
Zambia (3,1)
Sierra Leone (3,3)
Malaysia (11,11)
Pakistan (9,5)
DR Congo (7,0)
Bhutan (3,2)
Bangladesh (14,6)
Lao PDR (2,1)
North Korea (0,0)
Sri Lanka (8,4)
Indonesia (13,8)
India (92,17)
-40
-30
-20
-10
0
10
20
30
40
Change 1990-2022 (percentage points)

**Appendix Figure 14.** Change in composition of double burden from 1990 to 2022 for adults, by age group.

The arrows start from the proportion of double burden that was from obesity in 1990 and end at the proportion in 2022; they are ordered by the proportion in 2022. The arrows in darker shade show countries where double burden shifted from underweight dominance to obesity dominance.  Prevalence was age-standardised within each age group presented.

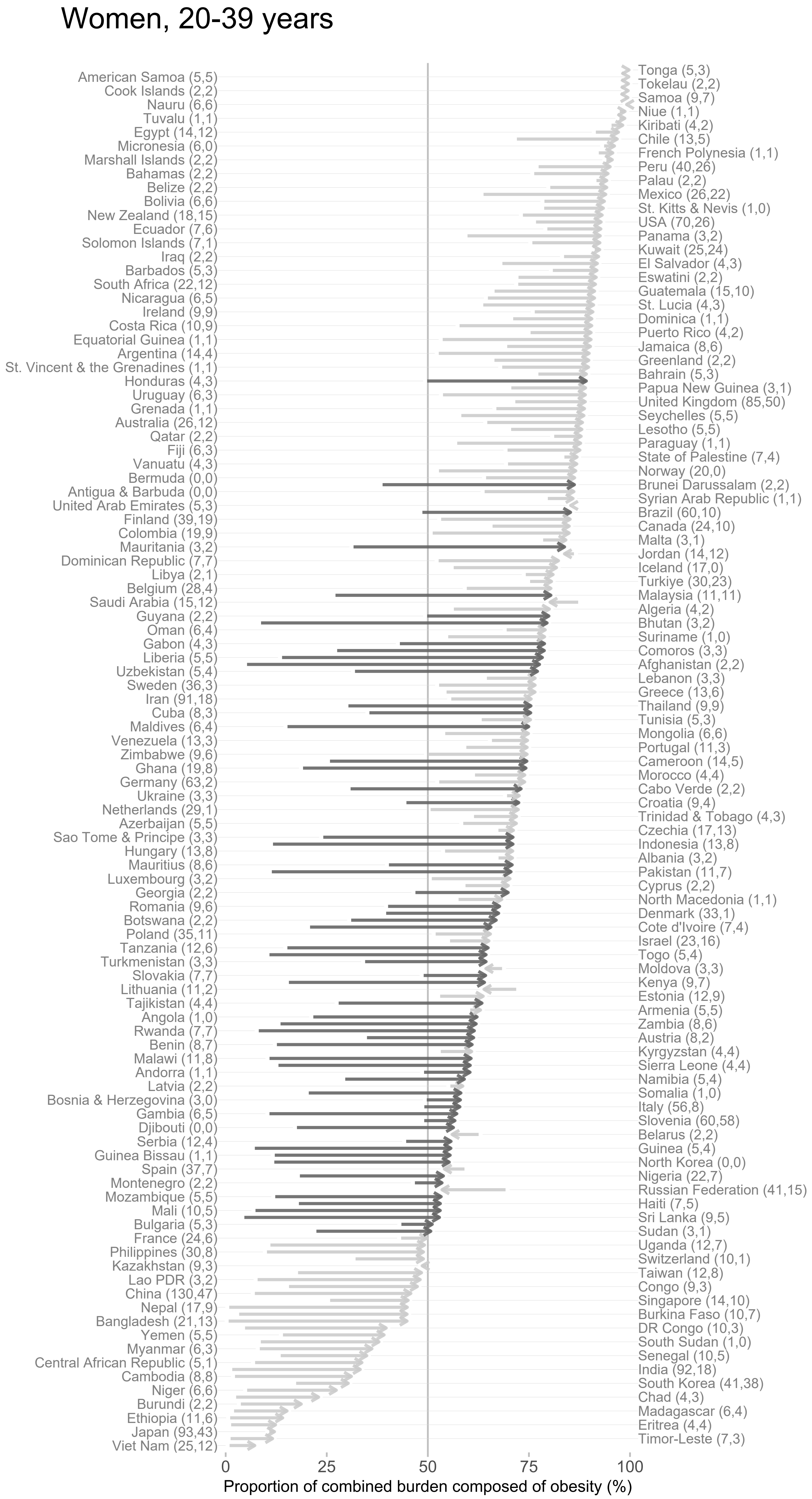
Women, 20-39 years
American Samoa (5,5)
Cook Islands (2,2)
Nauru (6,6)
Tuvalu (1,1)
Egypt (14,12)
Micronesia (6,0)
Marshall Islands (2,2)
Bahamas (2,2)
Belize (2,2)
Bolivia (6,6)
New Zealand (18,15)
Ecuador (7,6)
Solomon Islands (7,1)
Iraq (2,2)
Barbados (5,3)
South Africa (22,12)
Nicaragua (6,5)
Ireland (9,9)
Costa Rica (10,9)
Equatorial Guinea (1,1)
Argentina (14,4)
St. Vincent & the Grenadines (1,1)
Honduras (4,3)
Uruguay (6,3)
Grenada (1,1)
Australia (26,12)
Qatar (2,2)
Fiji (6,3)
Vanuatu (4,3)
Bermuda (0,0)
Antigua & Barbuda (0,0)
United Arab Emirates (5,3)
Finland (39,19)
Colombia (19,9)
Mauritania (3,2)
Dominican Republic (7,7)
Libya (2,1)
Belgium (28,4)
Saudi Arabia (15,12)
Guyana (2,2)
Oman (6,4)
Gabon (4,3)
Liberia (5,5)
Uzbekistan (5,4)
Sweden (36,3)
Iran (91,18)
Cuba (8,3)
Maldives (6,4)
Venezuela (13,3)
Zimbabwe (9,6)
Ghana (19,8)
Germany (63,2)
Ukraine (3,3)
Netherlands (29,1)
Azerbaijan (5,5)
Sao Tome & Principe (3,3)
Hungary (13,8)
Mauritius (8,6)
Luxembourg (3,2)
Georgia (2,2)
Romania (9,6)
Botswana (2,2)
Poland (35,11)
Tanzania (12,6)
Turkmenistan (3,3)
Slovakia (7,7)
Lithuania (11,2)
Tajikistan (4,4)
Angola (1,0)
Rwanda (7,7)
Benin (8,7)
Malawi (11,8)
Andorra (1,1)
Latvia (2,2)
Bosnia & Herzegovina (3,0)
Gambia (6,5)
Djibouti (0,0)
Serbia (12,4)
Guinea Bissau (1,1)
Spain (37,7)
Montenegro (2,2)
Mozambique (5,5)
Mali (10,5)
Bulgaria (5,3)
France (24,6)
Philippines (30,8)
Kazakhstan (9,3)
Lao PDR (3,2)
China (130,47)
Nepal (17,9)
Bangladesh (21,13)
Yemen (5,5)
Myanmar (6,3)
Central African Republic (5,1)
Cambodia (8,8)
Niger (6,6)
Burundi (2,2)
Ethiopia (11,6)
Japan (93,43)
Viet Nam (25,12)
Tonga (5,3)
Tokelau (2,2)
Samoa (9,7)
Niue (1,1)
Kiribati (4,2)
Chile (13,5)
French Polynesia (1,1)
Peru (40,26)
Palau (2,2)
Mexico (26,22)
St. Kitts & Nevis (1,0)
USA (70,26)
Panama (3,2)
Kuwait (25,24)
El Salvador (4,3)
Eswatini (2,2)
Guatemala (15,10)
St. Lucia (4,3)
Dominica (1,1)
Puerto Rico (4,2)
Jamaica (8,6)
Greenland (2,2)
Bahrain (5,3)
Papua New Guinea (3,1)
United Kingdom (85,50)
Seychelles (5,5)
Lesotho (5,5)
Paraguay (1,1)
State of Palestine (7,4)
Norway (20,0)
Brunei Darussalam (2,2)
Syrian Arab Republic (1,1)
Brazil (60,10)
Canada (24,10)
Malta (3,1)
Jordan (14,12)
Iceland (17,0)
Turkiye (30,23)
Malaysia (11,11)
Algeria (4,2)
Bhutan (3,2)
Suriname (1,0)
Comoros (3,3)
Afghanistan (2,2)
Lebanon (3,3)
Greece (13,6)
Thailand (9,9)
Tunisia (5,3)
Mongolia (6,6)
Portugal (11,3)
Cameroon (14,5)
Morocco (4,4)
Cabo Verde (2,2)
Croatia (9,4)
Trinidad & Tobago (4,3)
Czechia (17,13)
Indonesia (13,8)
Albania (3,2)
Pakistan (11,7)
Cyprus (2,2)
North Macedonia (1,1)
Denmark (33,1)
Cote d'Ivoire (7,4)
Israel (23,16)
Togo (5,4)
Moldova (3,3)
Kenya (9,7)
Estonia (12,9)
Armenia (5,5)
Zambia (8,6)
Austria (8,2)
Kyrgyzstan (4,4)
Sierra Leone (4,4)
Namibia (5,4)
Somalia (1,0)
Italy (56,8)
Slovenia (60,58)
Belarus (2,2)
Guinea (5,4)
North Korea (0,0)
Nigeria (22,7)
Russian Federation (41,15)
Haiti (7,5)
Sri Lanka (9,5)
Sudan (3,1)
Uganda (12,7)
Switzerland (10,1)
Taiwan (12,8)
Congo (9,3)
Singapore (14,10)
Burkina Faso (10,7)
DR Congo (10,3)
South Sudan (1,0)
Senegal (10,5)
India (92,18)
South Korea (41,38)
Chad (4,3)
Madagascar (6,4)
Eritrea (4,4)
Timor-Leste (7,3)
0
25
50
75
100
Proportion of combined burden composed of obesity (%)

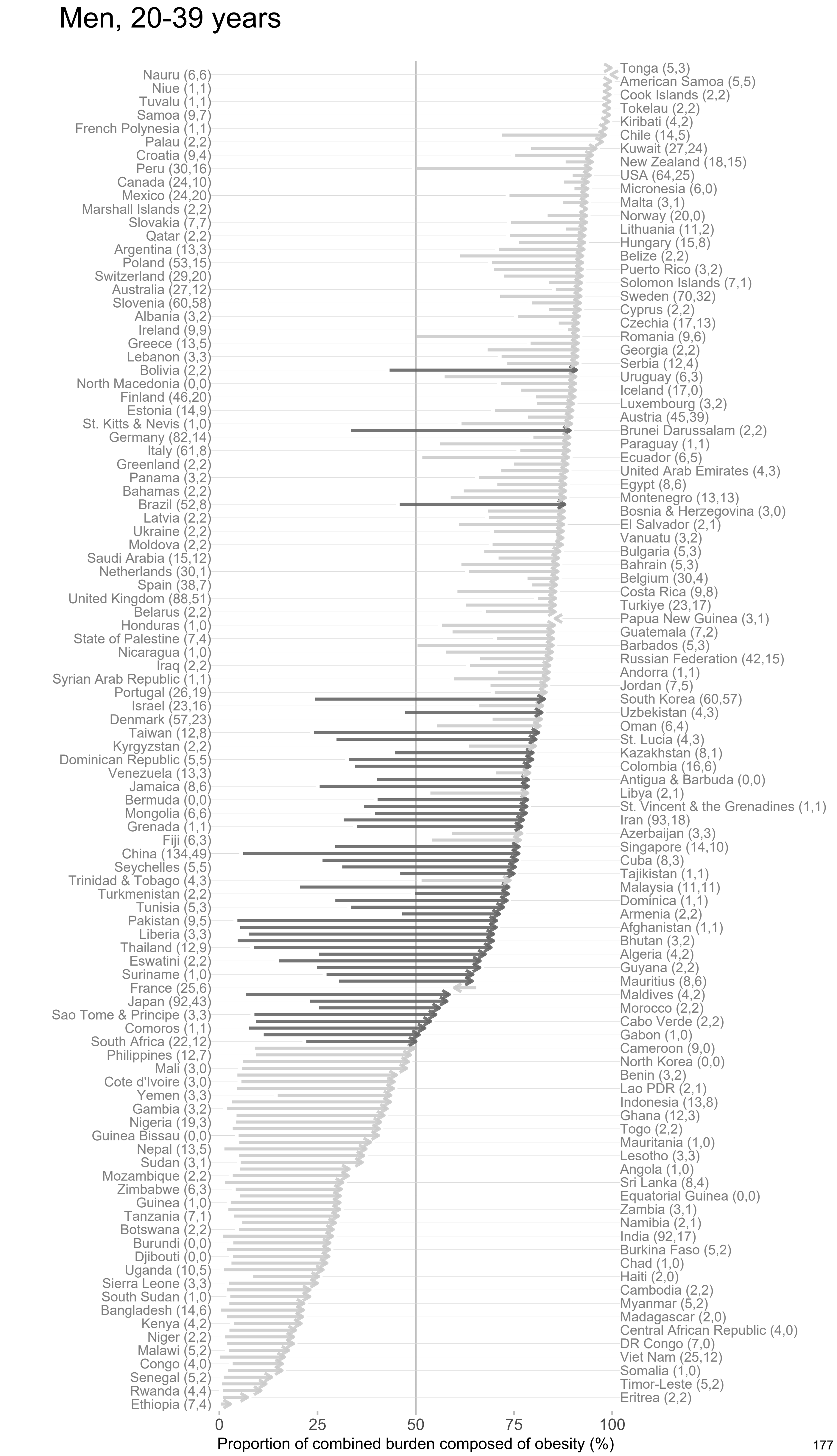
Men, 20-39 years
Nauru (6,6)
Niue (1,1)
Tuvalu (1,1)
Samoa (9,7)
French Polynesia (1,1)
Palau (2,2)
Croatia (9,4)
Peru (30,16)
Canada (24,10)
Mexico (24,20)
Marshall Islands (2,2)
Slovakia (7,7)
Qatar (2,2)
Argentina (13,3)
Poland (53,15)
Switzerland (29,20)
Australia (27,12)
Slovenia (60,58)
Albania (3,2)
Ireland (9,9)
Greece (13,5)
Lebanon (3,3)
Bolivia (2,2)
North Macedonia (0,0)
Finland (46,20)
Estonia (14,9)
St. Kitts & Nevis (1,0)
Germany (82,14)
Italy (61,8)
Greenland (2,2)
Panama (3,2)
Bahamas (2,2)
Brazil (52,8)
Latvia (2,2)
Ukraine (2,2)
Moldova (2,2)
Saudi Arabia (15,12)
Netherlands (30,1)
Spain (38,7)
United Kingdom (88,51)
Belarus (2,2)
Honduras (1,0)
State of Palestine (7,4)
Nicaragua (1,0)
Iraq (2,2)
Syrian Arab Republic (1,1)
Portugal (26,19)
Israel (23,16)
Denmark (57,23)
Taiwan (12,8)
Kyrgyzstan (2,2)
Dominican Republic (5,5)
Venezuela (13,3)
Jamaica (8,6)
Bermuda (0,0)
Mongolia (6,6)
Grenada (1,1)
Fiji (6,3)
China (134,49)
Seychelles (5,5)
Trinidad & Tobago (4,3)
Turkmenistan (2,2)
Tunisia (5,3)
Pakistan (9,5)
Liberia (3,3)
Thailand (12,9)
Eswatini (2,2)
Suriname (1,0)
France (25,6)
Japan (92,43)
Sao Tome & Principe (3,3)
Comoros (1,1)
South Africa (22,12)
Philippines (12,7)
Mali (3,0)
Cote d'Ivoire (3,0)
Yemen (3,3)
Gambia (3,2)
Nigeria (19,3)
Guinea Bissau (0,0)
Nepal (13,5)
Sudan (3,1)
Mozambique (2,2)
Zimbabwe (6,3)
Guinea (1,0)
Tanzania (7,1)
Botswana (2,2)
Burundi (0,0)
Djibouti (0,0)
Uganda (10,5)
Sierra Leone (3,3)
South Sudan (1,0)
Bangladesh (14,6)
Kenya (4,2)
Niger (2,2)
Malawi (5,2)
Congo (4,0)
Senegal (5,2)
Rwanda (4,4)
Ethiopia (7,4)
Tonga (5,3)
American Samoa (5,5)
Cook Islands (2,2)
Tokelau (2,2)
Kiribati (4,2)
Chile (14,5)
Kuwait (27,24)
New Zealand (18,15)
USA (64,25)
Micronesia (6,0)
Malta (3,1)
Norway (20,0)
Lithuania (11,2)
Hungary (15,8)
Belize (2,2)
Puerto Rico (3,2)
Solomon Islands (7,1)
Sweden (70,32)
Cyprus (2,2)
Czechia (17,13)
Romania (9,6)
Georgia (2,2)
Serbia (12,4)
Uruguay (6,3)
Iceland (17,0)
Luxembourg (3,2)
Austria (45,39)
Brunei Darussalam (2,2)
Paraguay (1,1)
Ecuador (6,5)
United Arab Emirates (4,3)
Egypt (8,6)
Montenegro (13,13)
Bosnia & Herzegovina (3,0)
El Salvador (2,1)
Vanuatu (3,2)
Bulgaria (5,3)
Bahrain (5,3)
Belgium (30,4)
Costa Rica (9,8)
Turkiye (23,17)
Papua New Guinea (3,1)
Guatemala (7,2)
Barbados (5,3)
Russian Federation (42,15)
Andorra (1,1)
Jordan (7,5)
South Korea (60,57)
Uzbekistan (4,3)
Oman (6,4)
St. Lucia (4,3)
Kazakhstan (8,1)
Colombia (16,6)
Antigua & Barbuda (0,0)
Libya (2,1)
St. Vincent & the Grenadines (1,1)
Iran (93,18)
Azerbaijan (3,3)
Singapore (14,10)
Cuba (8,3)
Tajikistan (1,1)
Malaysia (11,11)
Dominica (1,1)
Armenia (2,2)
Afghanistan (1,1)
Bhutan (3,2)
Algeria (4,2)
Guyana (2,2)
Mauritius (8,6)
Maldives (4,2)
Morocco (2,2)
Cabo Verde (2,2)
Gabon (1,0)
Cameroon (9,0)
North Korea (0,0)
Benin (3,2)
Lao PDR (2,1)
Indonesia (13,8)
Ghana (12,3)
Togo (2,2)
Mauritania (1,0)
Lesotho (3,3)
Angola (1,0)
Sri Lanka (8,4)
Equatorial Guinea (0,0)
Zambia (3,1)
Namibia (2,1)
India (92,17)
Burkina Faso (5,2)
Chad (1,0)
Haiti (2,0)
Cambodia (2,2)
Myanmar (5,2)
Madagascar (2,0)
Central African Republic (4,0)
DR Congo (7,0)
Viet Nam (25,12)
Somalia (1,0)
Timor-Leste (5,2)
Eritrea (2,2)
0
25
50
75
100
Proportion of combined burden composed of obesity (%)

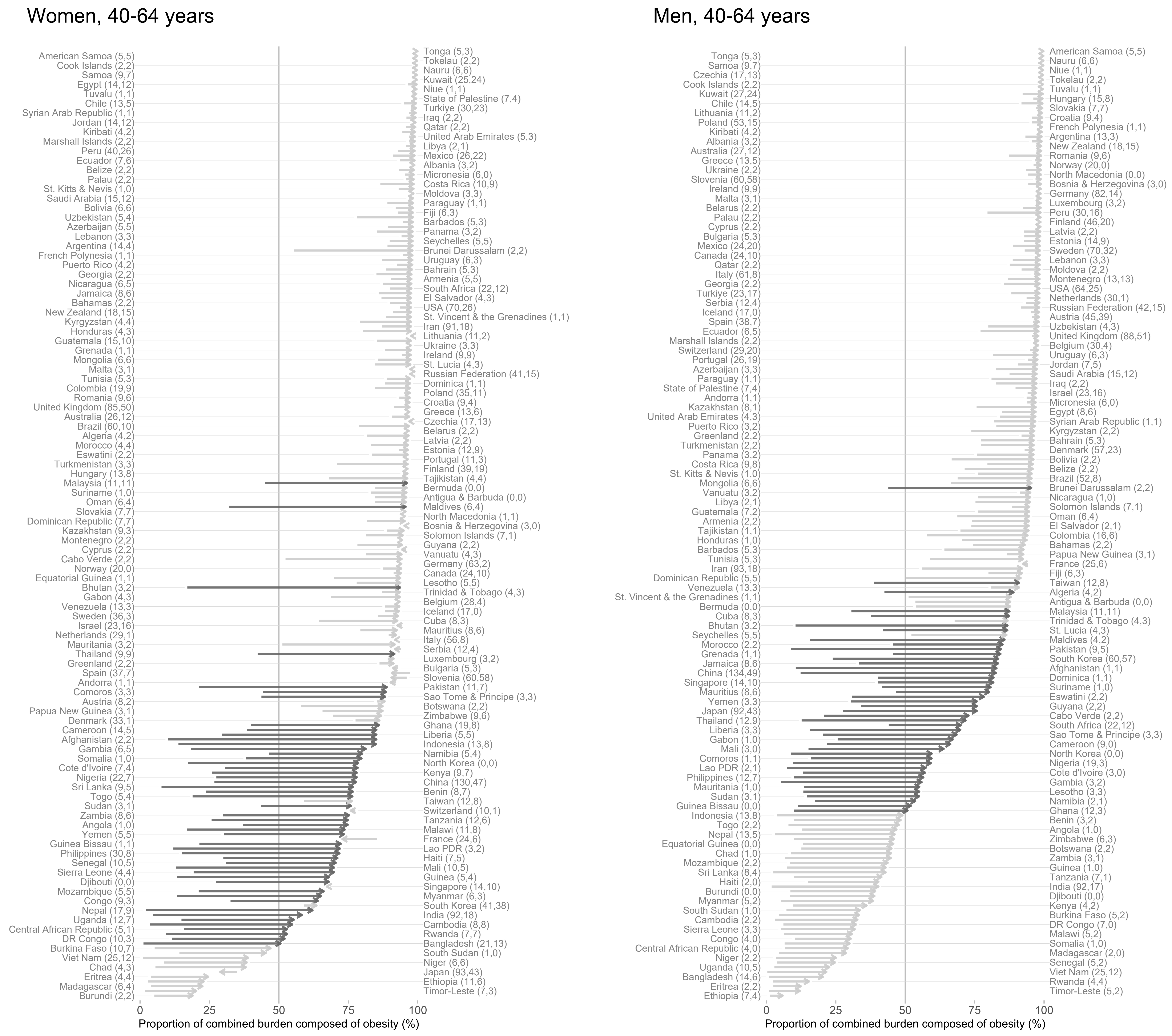
Women, 40-64 years
American Samoa (5,5)
Cook Islands (2,2)
Samoa (9,7)
Egypt (14,12)
Tuvalu (1,1)
Chile (13,5)
Syrian Arab Republic (1,1)
Jordan (14,12)
Kiribati (4,2)
Marshall Islands (2,2)
Peru (40,26)
Ecuador (7,6)
Belize (2,2)
Palau (2,2)
St. Kitts & Nevis (1,0)
Saudi Arabia (15,12)
Bolivia (6,6)
Uzbekistan (5,4)
Azerbaijan (5,5)
Lebanon (3,3)
Argentina (14,4)
French Polynesia (1,1)
Puerto Rico (4,2)
Georgia (2,2)
Nicaragua (6,5)
Jamaica (8,6)
Bahamas (2,2)
New Zealand (18,15)
Kyrgyzstan (4,4)
Honduras (4,3)
Guatemala (15,10)
Grenada (1,1)
Mongolia (6,6)
Malta (3,1)
Tunisia (5,3)
Colombia (19,9)
Romania (9,6)
United Kingdom (85,50)
Australia (26,12)
Brazil (60,10)
Algeria (4,2)
Morocco (4,4)
Eswatini (2,2)
Turkmenistan (3,3)
Hungary (13,8)
Malaysia (11,11)
Suriname (1,0)
Oman (6,4)
Slovakia (7,7)
Dominican Republic (7,7)
Kazakhstan (9,3)
Montenegro (2,2)
Cyprus (2,2)
Cabo Verde (2,2)
Norway (20,0)
Equatorial Guinea (1,1)
Bhutan (3,2)
Gabon (4,3)
Venezuela (13,3)
Sweden (36,3)
Israel (23,16)
Netherlands (29,1)
Mauritania (3,2)
Thailand (9,9)
Greenland (2,2)
Spain (37,7)
Andorra (1,1)
Comoros (3,3)
Austria (8,2)
Papua New Guinea (3,1)
Denmark (33,1)
Cameroon (14,5)
Afghanistan (2,2)
Gambia (6,5)
Somalia (1,0)
Cote d'Ivoire (7,4)
Nigeria (22,7)
Sri Lanka (9,5)
Togo (5,4)
Sudan (3,1)
Zambia (8,6)
Angola (1,0)
Yemen (5,5)
Guinea Bissau (1,1)
Philippines (30,8)
Senegal (10,5)
Sierra Leone (4,4)
Djibouti (0,0)
Mozambique (5,5)
Congo (9,3)
Nepal (17,9)
Uganda (12,7)
Central African Republic (5,1)
DR Congo (10,3)
Burkina Faso (10,7)
Viet Nam (25,12)
Chad (4,3)
Eritrea (4,4)
Madagascar (6,4)
Burundi (2,2)
Tonga (5,3)
Tokelau (2,2)
Nauru (6,6)
Kuwait (25,24)
Niue (1,1)
State of Palestine (7,4)
Turkiye (30,23)
Iraq (2,2)
Qatar (2,2)
United Arab Emirates (5,3)
Libya (2,1)
Mexico (26,22)
Albania (3,2)
Micronesia (6,0)
Costa Rica (10,9)
Moldova (3,3)
Paraguay (1,1)
Fiji (6,3)
Barbados (5,3)
Panama (3,2)
Seychelles (5,5)
Brunei Darussalam (2,2)
Uruguay (6,3)
Bahrain (5,3)
Armenia (5,5)
South Africa (22,12)
El Salvador (4,3)
USA (70,26)
St. Vincent & the Grenadines (1,1)
Iran (91,18)
Lithuania (11,2)
Ukraine (3,3)
Ireland (9,9)
St. Lucia (4,3)
Russian Federation (41,15)
Dominica (1,1)
Poland (35,11)
Croatia (9,4)
Greece (13,6)
Czechia (17,13)
Belarus (2,2)
Latvia (2,2)
Estonia (12,9)
Portugal (11,3)
Finland (39,19)
Tajikistan (4,4)
Bermuda (0,0)
Antigua & Barbuda (0,0)
Maldives (6,4)
North Macedonia (1,1)
Bosnia & Herzegovina (3,0)
Solomon Islands (7,1)
Guyana (2,2)
Vanuatu (4,3)
Germany (63,2)
Canada (24,10)
Lesotho (5,5)
Trinidad & Tobago (4,3)
Belgium (28,4)
Iceland (17,0)
Cuba (8,3)
Mauritius (8,6)
Italy (56,8)
Serbia (12,4)
Luxembourg (3,2)
Bulgaria (5,3)
Slovenia (60,58)
Pakistan (11,7)
Sao Tome & Principe (3,3)
Botswana (2,2)
Zimbabwe (9,6)
Ghana (19,8)
Liberia (5,5)
Indonesia (13,8)
Namibia (5,4)
North Korea (0,0)
Kenya (9,7)
China (130,47)
Benin (8,7)
Taiwan (12,8)
Switzerland (10,1)
Tanzania (12,6)
Malawi (11,8)
France (24,6)
Lao PDR (3,2)
Haiti (7,5)
Mali (10,5)
Guinea (5,4)
Singapore (14,10)
Myanmar (6,3)
South Korea (41,38)
India (92,18)
Cambodia (8,8)
Rwanda (7,7)
Bangladesh (21,13)
South Sudan (1,0)
Niger (6,6)
Japan (93,43)
Ethiopia (11,6)
Timor-Leste (7,3)
0
25
50
75
100
Proportion of combined burden composed of obesity (%)
Men, 40-64 years
Tonga (5,3)
Samoa (9,7)
Czechia (17,13)
Cook Islands (2,2)
Kuwait (27,24)
Chile (14,5)
Lithuania (11,2)
Poland (53,15)
Kiribati (4,2)
Albania (3,2)
Australia (27,12)
Greece (13,5)
Ukraine (2,2)
Slovenia (60,58)
Ireland (9,9)
Malta (3,1)
Belarus (2,2)
Palau (2,2)
Cyprus (2,2)
Bulgaria (5,3)
Mexico (24,20)
Canada (24,10)
Qatar (2,2)
Italy (61,8)
Georgia (2,2)
Turkiye (23,17)
Serbia (12,4)
Iceland (17,0)
Spain (38,7)
Ecuador (6,5)
Marshall Islands (2,2)
Switzerland (29,20)
Portugal (26,19)
Azerbaijan (3,3)
Paraguay (1,1)
State of Palestine (7,4)
Andorra (1,1)
Kazakhstan (8,1)
United Arab Emirates (4,3)
Puerto Rico (3,2)
Greenland (2,2)
Turkmenistan (2,2)
Panama (3,2)
Costa Rica (9,8)
St. Kitts & Nevis (1,0)
Mongolia (6,6)
Vanuatu (3,2)
Libya (2,1)
Guatemala (7,2)
Armenia (2,2)
Tajikistan (1,1)
Honduras (1,0)
Barbados (5,3)
Tunisia (5,3)
Iran (93,18)
Dominican Republic (5,5)
Venezuela (13,3)
St. Vincent & the Grenadines (1,1)
Bermuda (0,0)
Cuba (8,3)
Bhutan (3,2)
Seychelles (5,5)
Morocco (2,2)
Grenada (1,1)
Jamaica (8,6)
China (134,49)
Singapore (14,10)
Mauritius (8,6)
Yemen (3,3)
Japan (92,43)
Thailand (12,9)
Liberia (3,3)
Gabon (1,0)
Mali (3,0)
Comoros (1,1)
Lao PDR (2,1)
Philippines (12,7)
Mauritania (1,0)
Sudan (3,1)
Guinea Bissau (0,0)
Indonesia (13,8)
Togo (2,2)
Nepal (13,5)
Equatorial Guinea (0,0)
Chad (1,0)
Mozambique (2,2)
Sri Lanka (8,4)
Haiti (2,0)
Burundi (0,0)
Myanmar (5,2)
South Sudan (1,0)
Cambodia (2,2)
Sierra Leone (3,3)
Congo (4,0)
Central African Republic (4,0)
Niger (2,2)
Uganda (10,5)
Bangladesh (14,6)
Eritrea (2,2)
Ethiopia (7,4)
American Samoa (5,5)
Nauru (6,6)
Niue (1,1)
Tokelau (2,2)
Tuvalu (1,1)
Hungary (15,8)
Slovakia (7,7)
Croatia (9,4)
French Polynesia (1,1)
Argentina (13,3)
New Zealand (18,15)
Romania (9,6)
Norway (20,0)
North Macedonia (0,0)
Bosnia & Herzegovina (3,0)
Germany (82,14)
Luxembourg (3,2)
Peru (30,16)
Finland (46,20)
Latvia (2,2)
Estonia (14,9)
Sweden (70,32)
Lebanon (3,3)
Moldova (2,2)
Montenegro (13,13)
USA (64,25)
Netherlands (30,1)
Russian Federation (42,15)
Austria (45,39)
Uzbekistan (4,3)
United Kingdom (88,51)
Belgium (30,4)
Uruguay (6,3)
Jordan (7,5)
Saudi Arabia (15,12)
Iraq (2,2)
Israel (23,16)
Micronesia (6,0)
Egypt (8,6)
Syrian Arab Republic (1,1)
Kyrgyzstan (2,2)
Bahrain (5,3)
Denmark (57,23)
Bolivia (2,2)
Belize (2,2)
Brazil (52,8)
Brunei Darussalam (2,2)
Nicaragua (1,0)
Solomon Islands (7,1)
Oman (6,4)
El Salvador (2,1)
Colombia (16,6)
Bahamas (2,2)
Papua New Guinea (3,1)
France (25,6)
Fiji (6,3)
Taiwan (12,8)
Algeria (4,2)
Antigua & Barbuda (0,0)
Malaysia (11,11)
Trinidad & Tobago (4,3)
St. Lucia (4,3)
Maldives (4,2)
Pakistan (9,5)
South Korea (60,57)
Afghanistan (1,1)
Dominica (1,1)
Suriname (1,0)
Eswatini (2,2)
Guyana (2,2)
Cabo Verde (2,2)
South Africa (22,12)
Sao Tome & Principe (3,3)
Cameroon (9,0)
North Korea (0,0)
Nigeria (19,3)
Cote d'Ivoire (3,0)
Gambia (3,2)
Lesotho (3,3)
Namibia (2,1)
Ghana (12,3)
Benin (3,2)
Angola (1,0)
Zimbabwe (6,3)
Botswana (2,2)
Zambia (3,1)
Guinea (1,0)
Tanzania (7,1)
India (92,17)
Djibouti (0,0)
Kenya (4,2)
Burkina Faso (5,2)
DR Congo (7,0)
Malawi (5,2)
Somalia (1,0)
Madagascar (2,0)
Senegal (5,2)
Viet Nam (25,12)
Rwanda (4,4)
Timor-Leste (5,2)
0
25
50
75
100
Proportion of combined burden composed of obesity (%)

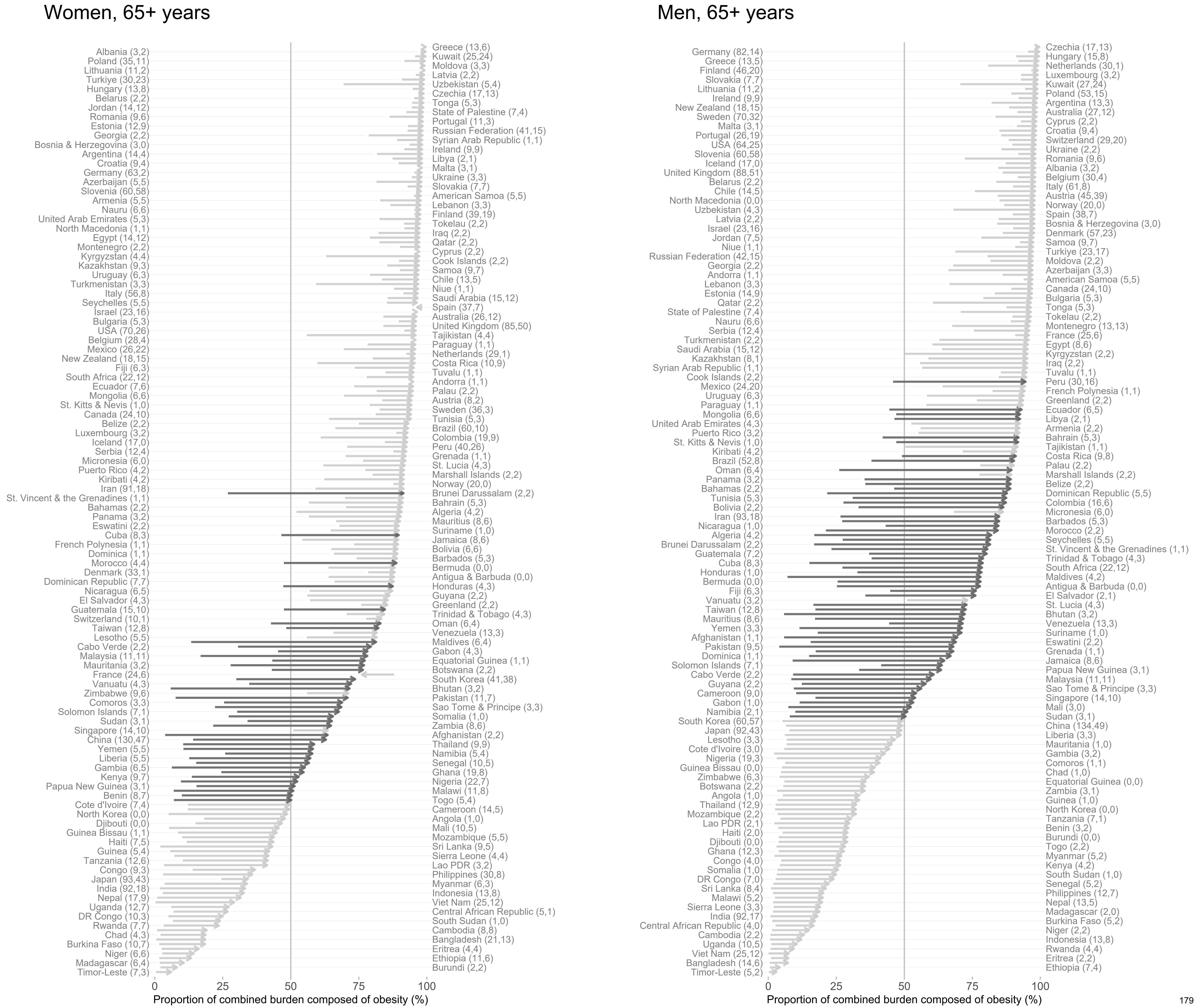
Women, 65+ years
Albania (3,2)
Poland (35,11)
Lithuania (11,2)
Turkiye (30,23)
Hungary (13,8)
Belarus (2,2)
Jordan (14,12)
Romania (9,6)
Estonia (12,9)
Georgia (2,2)
Bosnia & Herzegovina (3,0)
Argentina (14,4)
Croatia (9,4)
Germany (63,2)
Azerbaijan (5,5)
Slovenia (60,58)
Armenia (5,5)
Nauru (6,6)
United Arab Emirates (5,3)
North Macedonia (1,1)
Egypt (14,12)
Montenegro (2,2)
Kyrgyzstan (4,4)
Kazakhstan (9,3)
Uruguay (6,3)
Turkmenistan (3,3)
Italy (56,8)
Seychelles (5,5)
Israel (23,16)
Bulgaria (5,3)
USA (70,26)
Belgium (28,4)
Mexico (26,22)
New Zealand (18,15)
Fiji (6,3)
South Africa (22,12)
Ecuador (7,6)
Mongolia (6,6)
St. Kitts & Nevis (1,0)
Canada (24,10)
Belize (2,2)
Luxembourg (3,2)
Iceland (17,0)
Serbia (12,4)
Micronesia (6,0)
Puerto Rico (4,2)
Kiribati (4,2)
Iran (91,18)
St. Vincent & the Grenadines (1,1)
Bahamas (2,2)
Panama (3,2)
Eswatini (2,2)
Cuba (8,3)
French Polynesia (1,1)
Dominica (1,1)
Morocco (4,4)
Denmark (33,1)
Dominican Republic (7,7)
Nicaragua (6,5)
El Salvador (4,3)
Guatemala (15,10)
Switzerland (10,1)
Taiwan (12,8)
Lesotho (5,5)
Cabo Verde (2,2)
Malaysia (11,11)
Mauritania (3,2)
France (24,6)
Vanuatu (4,3)
Zimbabwe (9,6)
Comoros (3,3)
Solomon Islands (7,1)
Sudan (3,1)
Singapore (14,10)
China (130,47)
Yemen (5,5)
Liberia (5,5)
Gambia (6,5)
Kenya (9,7)
Papua New Guinea (3,1)
Benin (8,7)
Cote d'Ivoire (7,4)
North Korea (0,0)
Djibouti (0,0)
Guinea Bissau (1,1)
Haiti (7,5)
Guinea (5,4)
Tanzania (12,6)
Congo (9,3)
Japan (93,43)
India (92,18)
Nepal (17,9)
Uganda (12,7)
DR Congo (10,3)
Rwanda (7,7)
Chad (4,3)
Burkina Faso (10,7)
Niger (6,6)
Madagascar (6,4)
Timor-Leste (7,3)
Greece (13,6)
Kuwait (25,24)
Moldova (3,3)
Latvia (2,2)
Uzbekistan (5,4)
Czechia (17,13)
Tonga (5,3)
State of Palestine (7,4)
Portugal (11,3)
Russian Federation (41,15)
Syrian Arab Republic (1,1)
Ireland (9,9)
Libya (2,1)
Malta (3,1)
Ukraine (3,3)
Slovakia (7,7)
American Samoa (5,5)
Lebanon (3,3)
Finland (39,19)
Tokelau (2,2)
Iraq (2,2)
Qatar (2,2)
Cyprus (2,2)
Cook Islands (2,2)
Samoa (9,7)
Chile (13,5)
Niue (1,1)
Saudi Arabia (15,12)
Spain (37,7)
Australia (26,12)
United Kingdom (85,50)
Tajikistan (4,4)
Paraguay (1,1)
Netherlands (29,1)
Costa Rica (10,9)
Tuvalu (1,1)
Andorra (1,1)
Palau (2,2)
Austria (8,2)
Sweden (36,3)
Tunisia (5,3)
Brazil (60,10)
Colombia (19,9)
Peru (40,26)
Grenada (1,1)
St. Lucia (4,3)
Marshall Islands (2,2)
Norway (20,0)
Brunei Darussalam (2,2)
Bahrain (5,3)
Algeria (4,2)
Mauritius (8,6)
Suriname (1,0)
Jamaica (8,6)
Bolivia (6,6)
Barbados (5,3)
Bermuda (0,0)
Antigua & Barbuda (0,0)
Honduras (4,3)
Guyana (2,2)
Greenland (2,2)
Trinidad & Tobago (4,3)
Oman (6,4)
Venezuela (13,3)
Maldives (6,4)
Gabon (4,3)
Equatorial Guinea (1,1)
Botswana (2,2)
South Korea (41,38)
Bhutan (3,2)
Pakistan (11,7)
Sao Tome & Principe (3,3)
Somalia (1,0)
Zambia (8,6)
Afghanistan (2,2)
Thailand (9,9)
Namibia (5,4)
Senegal (10,5)
Ghana (19,8)
Nigeria (22,7)
Malawi (11,8)
Togo (5,4)
Cameroon (14,5)
Angola (1,0)
Mali (10,5)
Mozambique (5,5)
Sri Lanka (9,5)
Sierra Leone (4,4)
Lao PDR (3,2)
Philippines (30,8)
Myanmar (6,3)
Indonesia (13,8)
Viet Nam (25,12)
Central African Republic (5,1)
South Sudan (1,0)
Cambodia (8,8)
Bangladesh (21,13)
Eritrea (4,4)
Ethiopia (11,6)
Burundi (2,2)
0
25
50
75
100
Proportion of combined burden composed of obesity (%)
Men, 65+ years
Germany (82,14)
Greece (13,5)
Finland (46,20)
Slovakia (7,7)
Lithuania (11,2)
Ireland (9,9)
New Zealand (18,15)
Sweden (70,32)
Malta (3,1)
Portugal (26,19)
USA (64,25)
Slovenia (60,58)
Iceland (17,0)
United Kingdom (88,51)
Belarus (2,2)
Chile (14,5)
North Macedonia (0,0)
Uzbekistan (4,3)
Latvia (2,2)
Israel (23,16)
Jordan (7,5)
Niue (1,1)
Russian Federation (42,15)
Georgia (2,2)
Andorra (1,1)
Lebanon (3,3)
Estonia (14,9)
Qatar (2,2)
State of Palestine (7,4)
Nauru (6,6)
Serbia (12,4)
Turkmenistan (2,2)
Saudi Arabia (15,12)
Kazakhstan (8,1)
Syrian Arab Republic (1,1)
Cook Islands (2,2)
Mexico (24,20)
Uruguay (6,3)
Paraguay (1,1)
Mongolia (6,6)
United Arab Emirates (4,3)
Puerto Rico (3,2)
St. Kitts & Nevis (1,0)
Kiribati (4,2)
Brazil (52,8)
Oman (6,4)
Panama (3,2)
Bahamas (2,2)
Tunisia (5,3)
Bolivia (2,2)
Iran (93,18)
Nicaragua (1,0)
Algeria (4,2)
Brunei Darussalam (2,2)
Guatemala (7,2)
Cuba (8,3)
Honduras (1,0)
Bermuda (0,0)
Fiji (6,3)
Vanuatu (3,2)
Taiwan (12,8)
Mauritius (8,6)
Yemen (3,3)
Afghanistan (1,1)
Pakistan (9,5)
Dominica (1,1)
Solomon Islands (7,1)
Cabo Verde (2,2)
Guyana (2,2)
Cameroon (9,0)
Gabon (1,0)
Namibia (2,1)
South Korea (60,57)
Japan (92,43)
Lesotho (3,3)
Cote d'Ivoire (3,0)
Nigeria (19,3)
Guinea Bissau (0,0)
Zimbabwe (6,3)
Botswana (2,2)
Angola (1,0)
Thailand (12,9)
Mozambique (2,2)
Lao PDR (2,1)
Haiti (2,0)
Djibouti (0,0)
Ghana (12,3)
Congo (4,0)
Somalia (1,0)
DR Congo (7,0)
Sri Lanka (8,4)
Malawi (5,2)
Sierra Leone (3,3)
India (92,17)
Central African Republic (4,0)
Cambodia (2,2)
Uganda (10,5)
Viet Nam (25,12)
Bangladesh (14,6)
Timor-Leste (5,2)
Czechia (17,13)
Hungary (15,8)
Netherlands (30,1)
Luxembourg (3,2)
Kuwait (27,24)
Poland (53,15)
Argentina (13,3)
Australia (27,12)
Cyprus (2,2)
Croatia (9,4)
Switzerland (29,20)
Ukraine (2,2)
Romania (9,6)
Albania (3,2)
Belgium (30,4)
Italy (61,8)
Austria (45,39)
Norway (20,0)
Spain (38,7)
Bosnia & Herzegovina (3,0)
Denmark (57,23)
Samoa (9,7)
Turkiye (23,17)
Moldova (2,2)
Azerbaijan (3,3)
American Samoa (5,5)
Canada (24,10)
Bulgaria (5,3)
Tonga (5,3)
Tokelau (2,2)
Montenegro (13,13)
France (25,6)
Egypt (8,6)
Kyrgyzstan (2,2)
Iraq (2,2)
Tuvalu (1,1)
Peru (30,16)
French Polynesia (1,1)
Greenland (2,2)
Ecuador (6,5)
Libya (2,1)
Armenia (2,2)
Bahrain (5,3)
Tajikistan (1,1)
Costa Rica (9,8)
Palau (2,2)
Marshall Islands (2,2)
Belize (2,2)
Dominican Republic (5,5)
Colombia (16,6)
Micronesia (6,0)
Barbados (5,3)
Morocco (2,2)
Seychelles (5,5)
St. Vincent & the Grenadines (1,1)
Trinidad & Tobago (4,3)
South Africa (22,12)
Maldives (4,2)
Antigua & Barbuda (0,0)
El Salvador (2,1)
St. Lucia (4,3)
Bhutan (3,2)
Venezuela (13,3)
Suriname (1,0)
Eswatini (2,2)
Grenada (1,1)
Jamaica (8,6)
Papua New Guinea (3,1)
Malaysia (11,11)
Sao Tome & Principe (3,3)
Singapore (14,10)
Mali (3,0)
Sudan (3,1)
China (134,49)
Liberia (3,3)
Mauritania (1,0)
Gambia (3,2)
Comoros (1,1)
Chad (1,0)
Equatorial Guinea (0,0)
Zambia (3,1)
Guinea (1,0)
North Korea (0,0)
Tanzania (7,1)
Benin (3,2)
Burundi (0,0)
Togo (2,2)
Myanmar (5,2)
Kenya (4,2)
South Sudan (1,0)
Senegal (5,2)
Philippines (12,7)
Nepal (13,5)
Madagascar (2,0)
Burkina Faso (5,2)
Niger (2,2)
Indonesia (13,8)
Rwanda (4,4)
Eritrea (2,2)
Ethiopia (7,4)
0
25
50
75
100
Proportion of combined burden composed of obesity (%)

**Appendix Figure 15.** Change in age-standardised prevalence of thinness and obesity, for school-aged children and adolescents.

(A) Change in thinness from 1990 to 2022 in relation to thinness in 1990. (B) Change in obesity from 1990 to 2022 in relation to obesity in 1990. (C) Change in obesity in relation to change in thinness. The sloped solid line intersecting the origin delineates regions where an increase (above the line) or decrease (below) in the combined prevalence of thinness and obesity took place, and the dashed parallel lines indicate 10 percentage point intervals in change in combined prevalence.

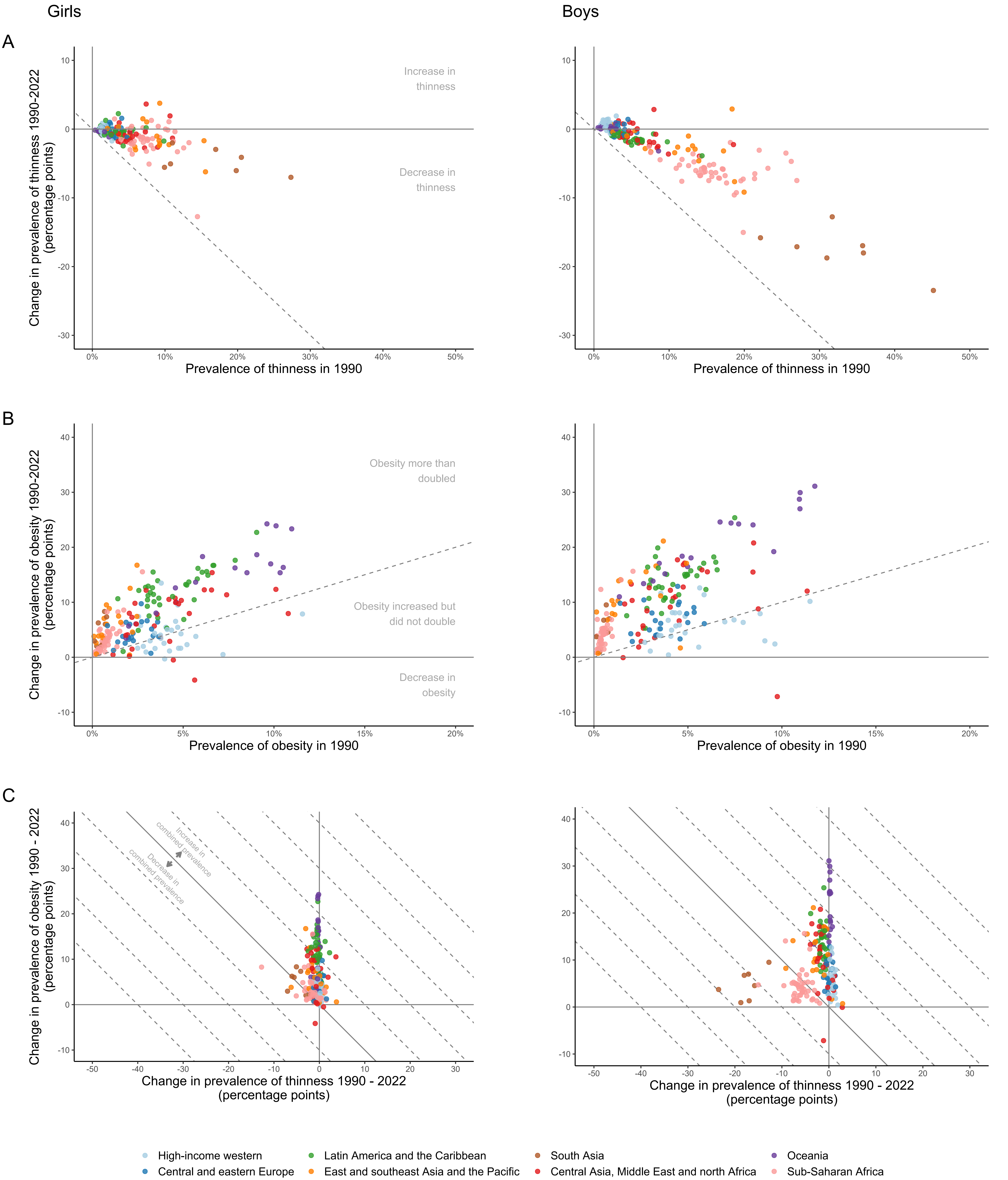
Girls
Boys
A
Increase in thinness
Decrease in thinness
Change in prevalence of thinness 1990-2022 (percentage points)
Prevalence of thinness in 1990
B
Obesity more than doubled
Obesity increased but did not double
Decrease in obesity
Change in prevalence of obesity 1990-2022 (percentage points)
Prevalence of obesity in 1990
C
Increase in combined prevalence
Decrease in combined prevalence
Change in prevalence of obesity 1990 - 2022 (percentage points)
Change in prevalence of thinness 1990 - 2022 (percentage points)
High-income western
Central and eastern Europe
Latin America and the Caribbean
East and southeast Asia and the Pacific
South Asia
Central Asia, Middle East and north Africa
Oceania
Sub-Saharan Africa

**Appendix Figure 16.** Age-standardised prevalence of all BMI categories in 1990 and 2022, for school-aged children and adolescents.

Country names are coloured by region. The numbers in brackets after each country's name show the total number of data sources and the number of nationally representative data sources, respectively. Countries are ordered alphabetically within each region.

# Girls

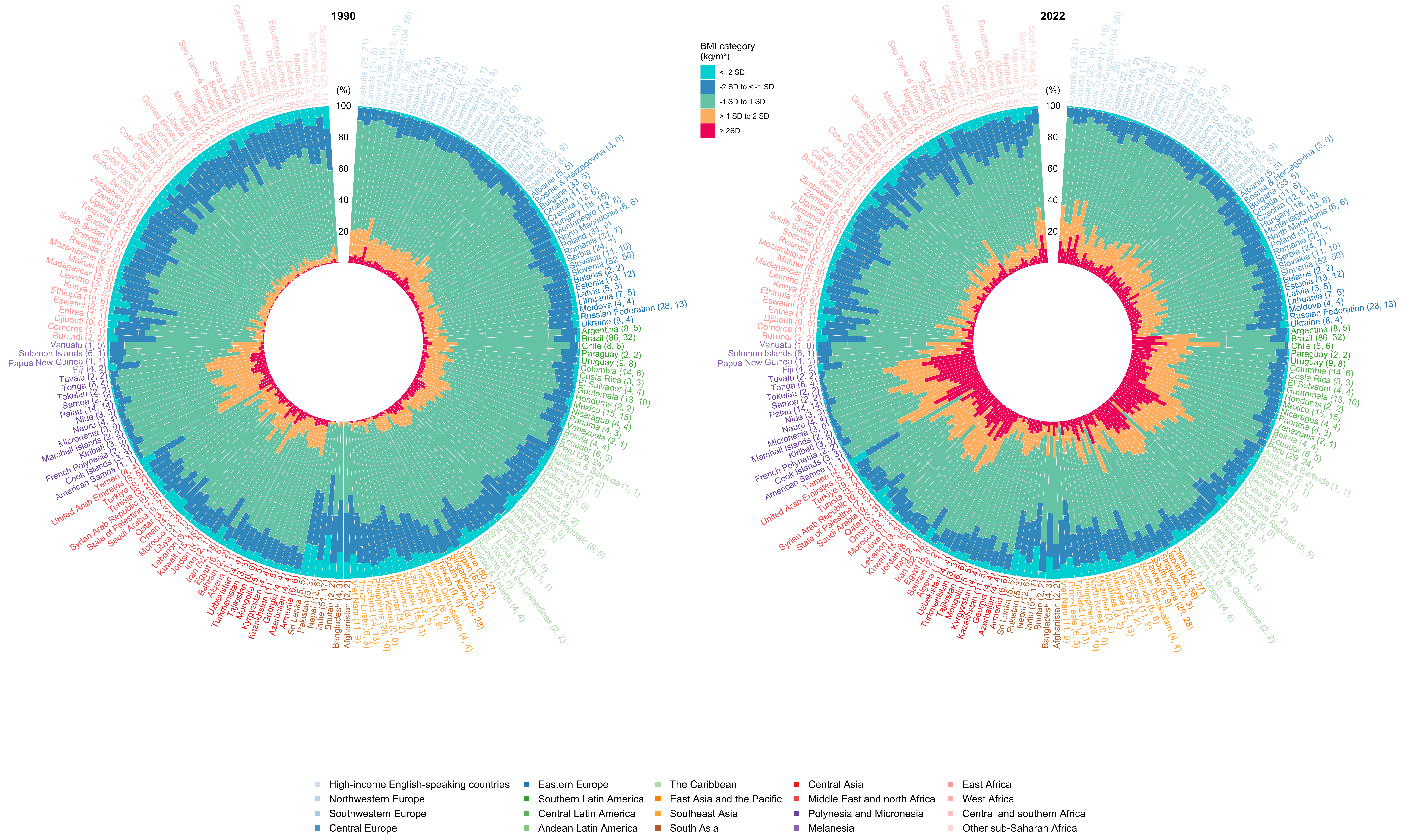

1990
2022
(%)
100
80
60
40
20
BMI category (kg/m²)
< -2 SD
-2 SD to < -1 SD
-1 SD to 1 SD
> 1 SD to 2 SD
> 2SD
High-income English-speaking countries
Northwestern Europe
Southwestern Europe
Central Europe
Eastern Europe
Southern Latin America
Central Latin America
Andean Latin America
The Caribbean
East Asia and the Pacific
Southeast Asia
South Asia
Central Asia
Middle East and north Africa
Polynesia and Micronesia
Melanesia
East Africa
West Africa
Central and southern Africa
Other sub-Saharan Africa

# Boys

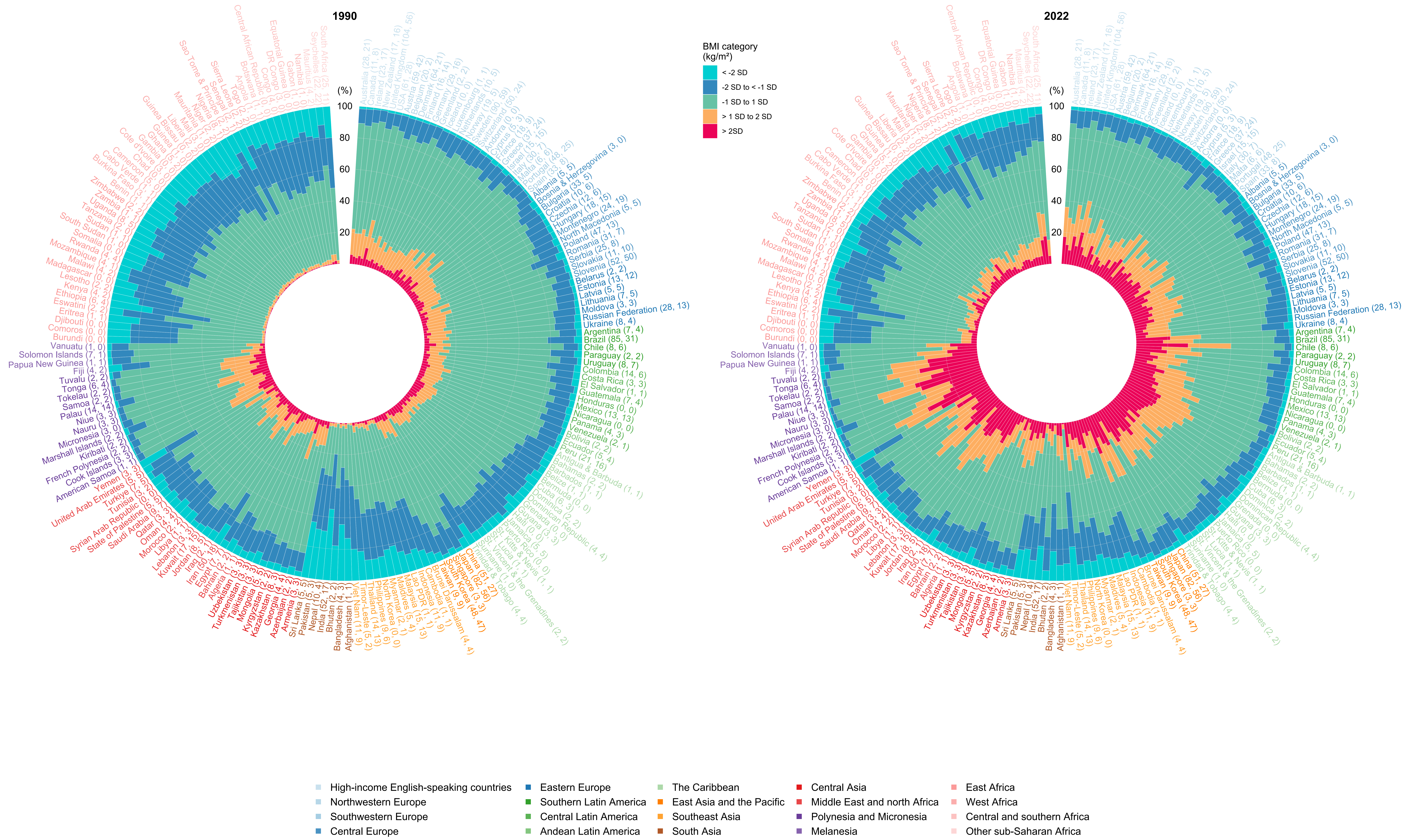

1990
2022
(%)
100
80
60
40
20
BMI category (kg/m²)
< -2 SD
-2 SD to < -1 SD
-1 SD to 1 SD
> 1 SD to 2 SD
> 2SD
High-income English-speaking countries
Northwestern Europe
Southwestern Europe
Central Europe
Eastern Europe
Southern Latin America
Central Latin America
Andean Latin America
The Caribbean
East Asia and the Pacific
Southeast Asia
South Asia
Central Asia
Middle East and north Africa
Polynesia and Micronesia
Melanesia
East Africa
West Africa
Central and southern Africa
Other sub-Saharan Africa

**Appendix Figure 17.** Age-standardised prevalence of thinness in 1990 and 2022, its change from 1990 to 2022, and posterior probability that the prevalence increased from 1990 to 2022, for school-aged children and adolescents.

Girls

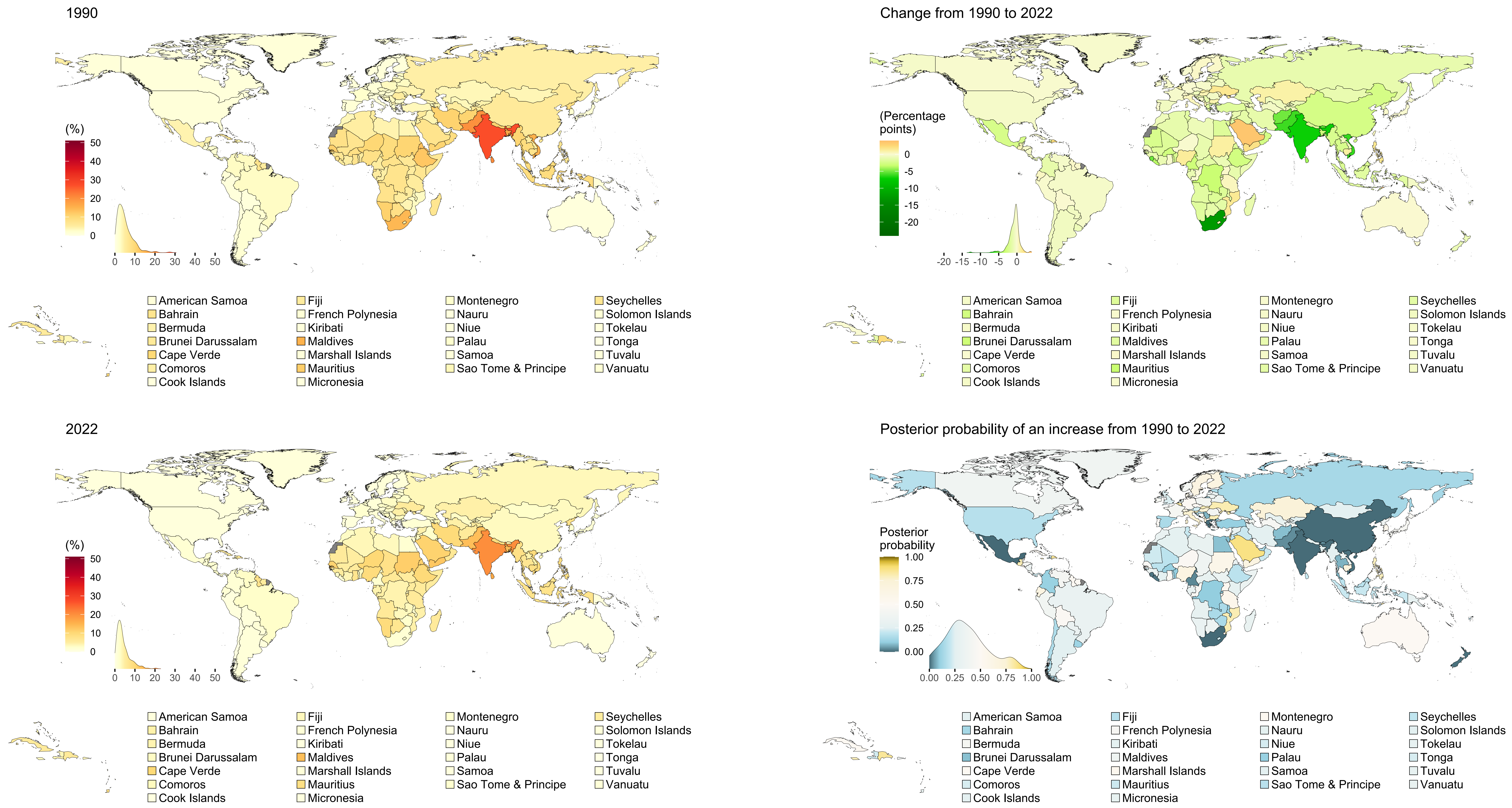
1990
(%)
50
40
30
20
10
0
0 10 20 30 40 50
American Samoa
Bahrain
Bermuda
Brunei Darussalam
Cape Verde
Comoros
Cook Islands
Fiji
French Polynesia
Kiribati
Maldives
Marshall Islands
Mauritius
Micronesia
Montenegro
Nauru
Niue
Palau
Samoa
Sao Tome & Principe
Seychelles
Solomon Islands
Tokelau
Tonga
Tuvalu
Vanuatu
Change from 1990 to 2022
(Percentage points)
0
-5
-10
-15
-20
-20 -15 -10 -5 0
American Samoa
Bahrain
Bermuda
Brunei Darussalam
Cape Verde
Comoros
Cook Islands
Fiji
French Polynesia
Kiribati
Maldives
Marshall Islands
Mauritius
Micronesia
Montenegro
Nauru
Niue
Palau
Samoa
Sao Tome & Principe
Seychelles
Solomon Islands
Tokelau
Tonga
Tuvalu
Vanuatu
2022
(%)
50
40
30
20
10
0
0 10 20 30 40 50
American Samoa
Bahrain
Bermuda
Brunei Darussalam
Cape Verde
Comoros
Cook Islands
Fiji
French Polynesia
Kiribati
Maldives
Marshall Islands
Mauritius
Micronesia
Montenegro
Nauru
Niue
Palau
Samoa
Sao Tome & Principe
Seychelles
Solomon Islands
Tokelau
Tonga
Tuvalu
Vanuatu
Posterior probability of an increase from 1990 to 2022
Posterior probability
1.00
0.75
0.50
0.25
0.00
0.00 0.25 0.50 0.75 1.00
American Samoa
Bahrain
Bermuda
Brunei Darussalam
Cape Verde
Comoros
Cook Islands
Fiji
French Polynesia
Kiribati
Maldives
Marshall Islands
Mauritius
Micronesia
Montenegro
Nauru
Niue
Palau
Samoa
Sao Tome & Principe
Seychelles
Solomon Islands
Tokelau
Tonga
Tuvalu
Vanuatu

# Boys

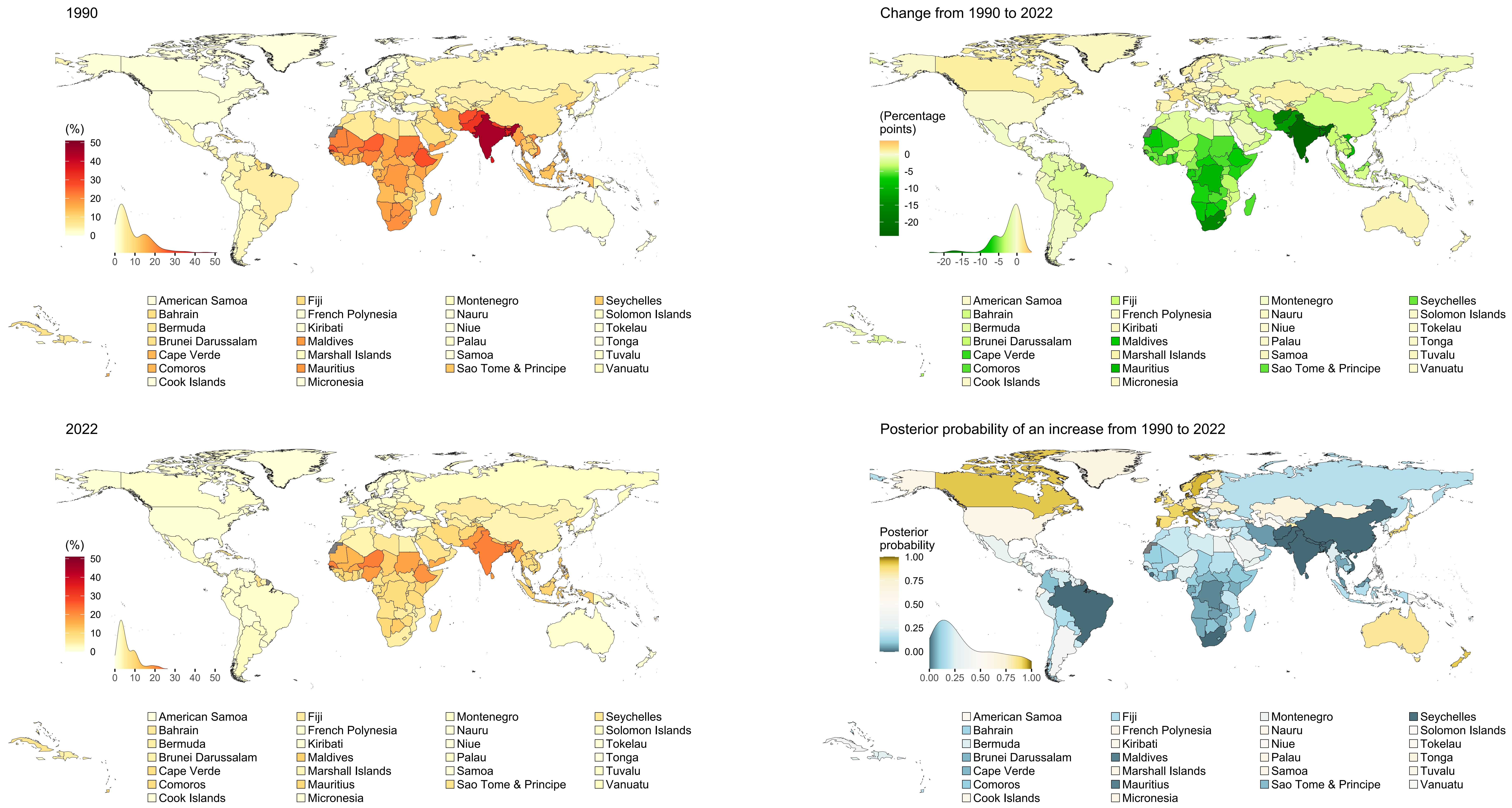

1990
(%)
50
40
30
20
10
0
0 10 20 30 40 50
American Samoa
Bahrain
Bermuda
Brunei Darussalam
Cape Verde
Comoros
Cook Islands
Fiji
French Polynesia
Kiribati
Maldives
Marshall Islands
Mauritius
Micronesia
Montenegro
Nauru
Niue
Palau
Samoa
Sao Tome & Principe
Seychelles
Solomon Islands
Tokelau
Tonga
Tuvalu
Vanuatu
Change from 1990 to 2022
(Percentage points)
0
-5
-10
-15
-20
-20 -15 -10 -5 0
American Samoa
Bahrain
Bermuda
Brunei Darussalam
Cape Verde
Comoros
Cook Islands
Fiji
French Polynesia
Kiribati
Maldives
Marshall Islands
Mauritius
Micronesia
Montenegro
Nauru
Niue
Palau
Samoa
Sao Tome & Principe
Seychelles
Solomon Islands
Tokelau
Tonga
Tuvalu
Vanuatu
2022
(%)
50
40
30
20
10
0
0 10 20 30 40 50
American Samoa
Bahrain
Bermuda
Brunei Darussalam
Cape Verde
Comoros
Cook Islands
Fiji
French Polynesia
Kiribati
Maldives
Marshall Islands
Mauritius
Micronesia
Montenegro
Nauru
Niue
Palau
Samoa
Sao Tome & Principe
Seychelles
Solomon Islands
Tokelau
Tonga
Tuvalu
Vanuatu
Posterior probability of an increase from 1990 to 2022
Posterior probability
1.00
0.75
0.50
0.25
0.00
0.00 0.25 0.50 0.75 1.00
American Samoa
Bahrain
Bermuda
Brunei Darussalam
Cape Verde
Comoros
Cook Islands
Fiji
French Polynesia
Kiribati
Maldives
Marshall Islands
Mauritius
Micronesia
Montenegro
Nauru
Niue
Palau
Samoa
Sao Tome & Principe
Seychelles
Solomon Islands
Tokelau
Tonga
Tuvalu
Vanuatu

**Appendix Figure 18.** Age-standardised prevalence of obesity in 1990 and 2022, its change from 1990 to 2022, and posterior probability that the prevalence increased from 1990 to 2022, for school-aged children and adolescents.

# Girls

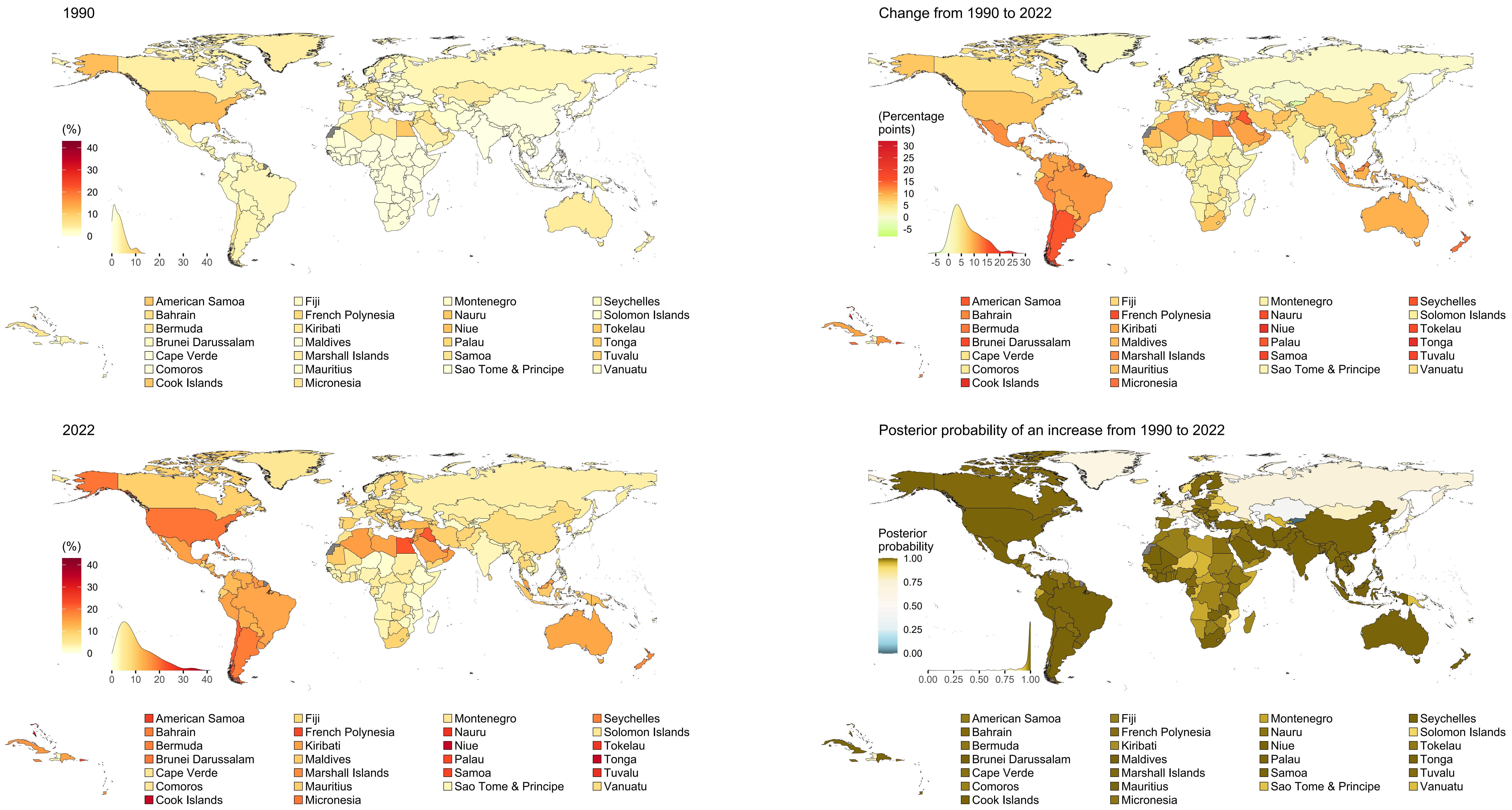
1990
(%)
40
30
20
10
0
0 10 20 30 40
American Samoa
Bahrain
Bermuda
Brunei Darussalam
Cape Verde
Comoros
Cook Islands
Fiji
French Polynesia
Kiribati
Maldives
Marshall Islands
Mauritius
Micronesia
Montenegro
Nauru
Niue
Palau
Samoa
Sao Tome & Principe
Seychelles
Solomon Islands
Tokelau
Tonga
Tuvalu
Vanuatu
Change from 1990 to 2022
(Percentage points)
30
25
20
15
10
5
0
-5
-5 0 5 10 15 20 25 30
American Samoa
Bahrain
Bermuda
Brunei Darussalam
Cape Verde
Comoros
Cook Islands
Fiji
French Polynesia
Kiribati
Maldives
Marshall Islands
Mauritius
Micronesia
Montenegro
Nauru
Niue
Palau
Samoa
Sao Tome & Principe
Seychelles
Solomon Islands
Tokelau
Tonga
Tuvalu
Vanuatu
2022
(%)
40
30
20
10
0
0 10 20 30 40
American Samoa
Bahrain
Bermuda
Brunei Darussalam
Cape Verde
Comoros
Cook Islands
Fiji
French Polynesia
Kiribati
Maldives
Marshall Islands
Mauritius
Micronesia
Montenegro
Nauru
Niue
Palau
Samoa
Sao Tome & Principe
Seychelles
Solomon Islands
Tokelau
Tonga
Tuvalu
Vanuatu
Posterior probability of an increase from 1990 to 2022
Posterior probability
1.00
0.75
0.50
0.25
0.00
0.00 0.25 0.50 0.75 1.00
American Samoa
Bahrain
Bermuda
Brunei Darussalam
Cape Verde
Comoros
Cook Islands
Fiji
French Polynesia
Kiribati
Maldives
Marshall Islands
Mauritius
Micronesia
Montenegro
Nauru
Niue
Palau
Samoa
Sao Tome & Principe
Seychelles
Solomon Islands
Tokelau
Tonga
Tuvalu
Vanuatu

Boys

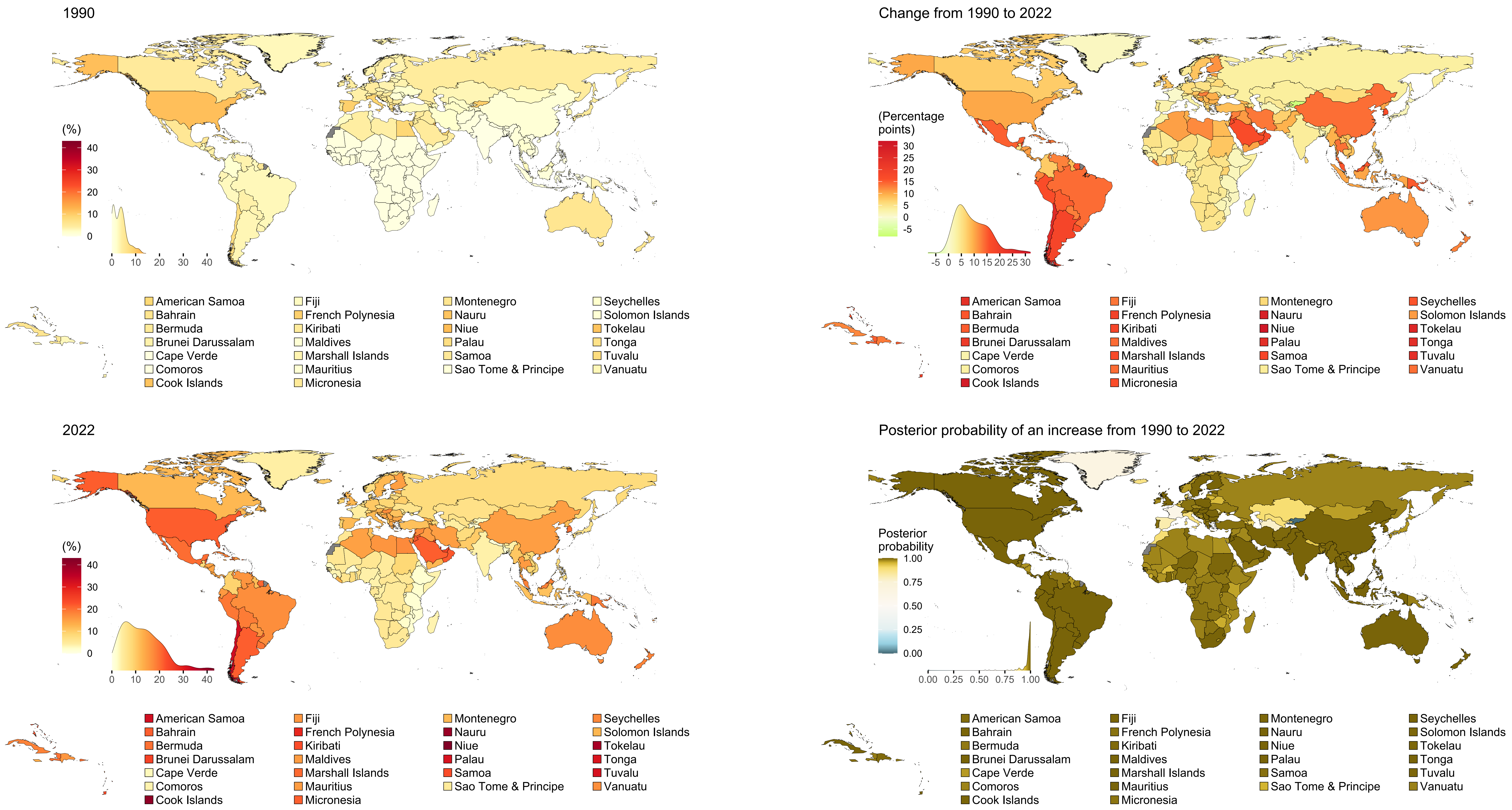
1990
(%)
40
30
20
10
0
0 10 20 30 40
American Samoa
Bahrain
Bermuda
Brunei Darussalam
Cape Verde
Comoros
Cook Islands
Fiji
French Polynesia
Kiribati
Maldives
Marshall Islands
Mauritius
Micronesia
Montenegro
Nauru
Niue
Palau
Samoa
Sao Tome & Principe
Seychelles
Solomon Islands
Tokelau
Tonga
Tuvalu
Vanuatu
Change from 1990 to 2022
(Percentage points)
30
25
20
15
10
5
0
-5
-5 0 5 10 15 20 25 30
2022
(%)
40
30
20
10
0
0 10 20 30 40
Posterior probability of an increase from 1990 to 2022
Posterior probability
1.00
0.75
0.50
0.25
0.00
0.00 0.25 0.50 0.75 1.00

**Appendix Figure 19.** Posterior probability that the age-standardised combined prevalence of thinness and obesity increased from 1990 to 2022, for school-aged children and adolescents.

# Girls

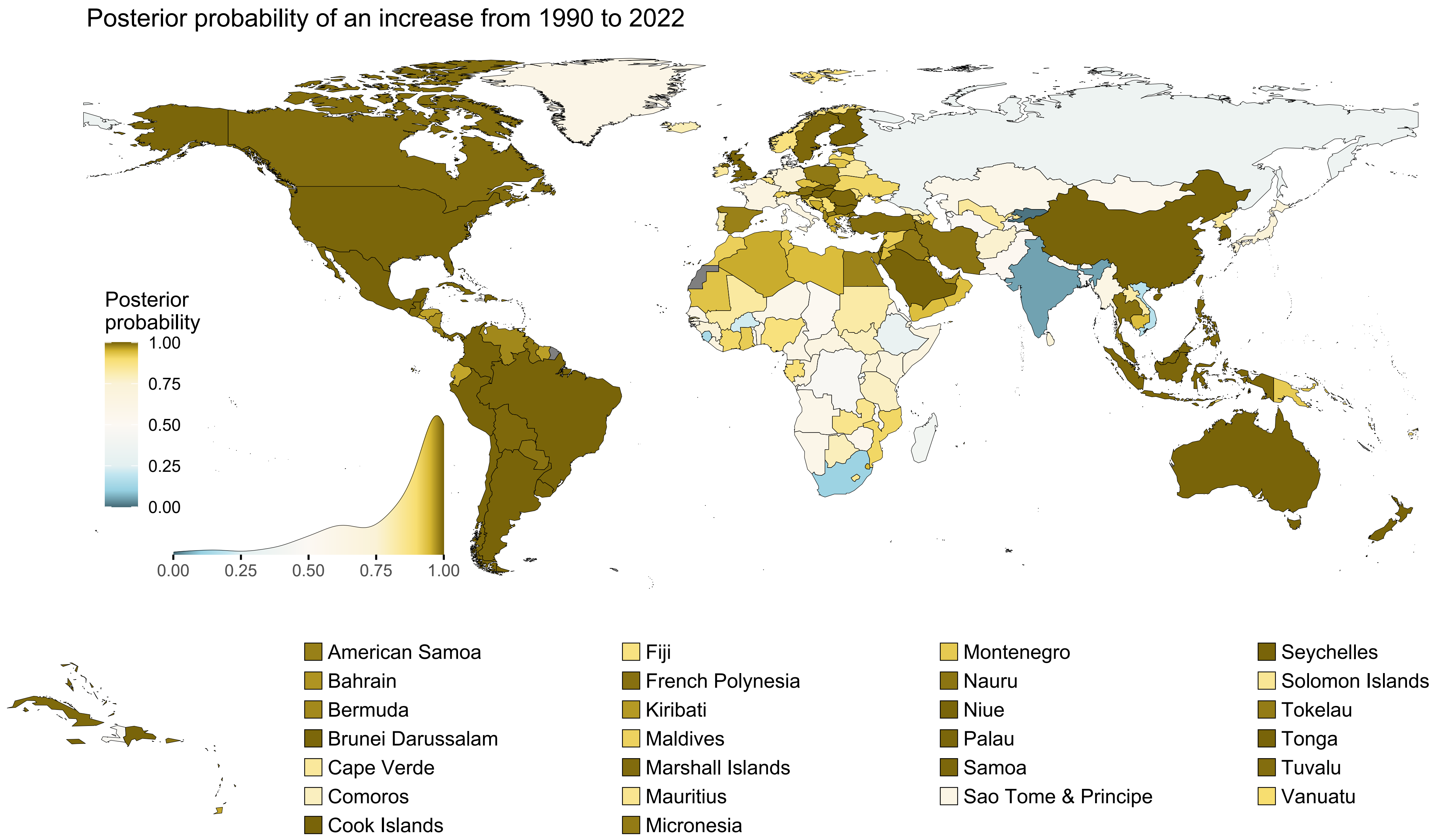

Posterior probability of an increase from 1990 to 2022
Posterior probability
1.00
0.75
0.50
0.25
0.00
0.00 0.25 0.50 0.75 1.00
American Samoa
Bahrain
Bermuda
Brunei Darussalam
Cape Verde
Comoros
Cook Islands
Fiji
French Polynesia
Kiribati
Maldives
Marshall Islands
Mauritius
Micronesia
Montenegro
Nauru
Niue
Palau
Samoa
Sao Tome & Principe
Seychelles
Solomon Islands
Tokelau
Tonga
Tuvalu
Vanuatu


# Boys

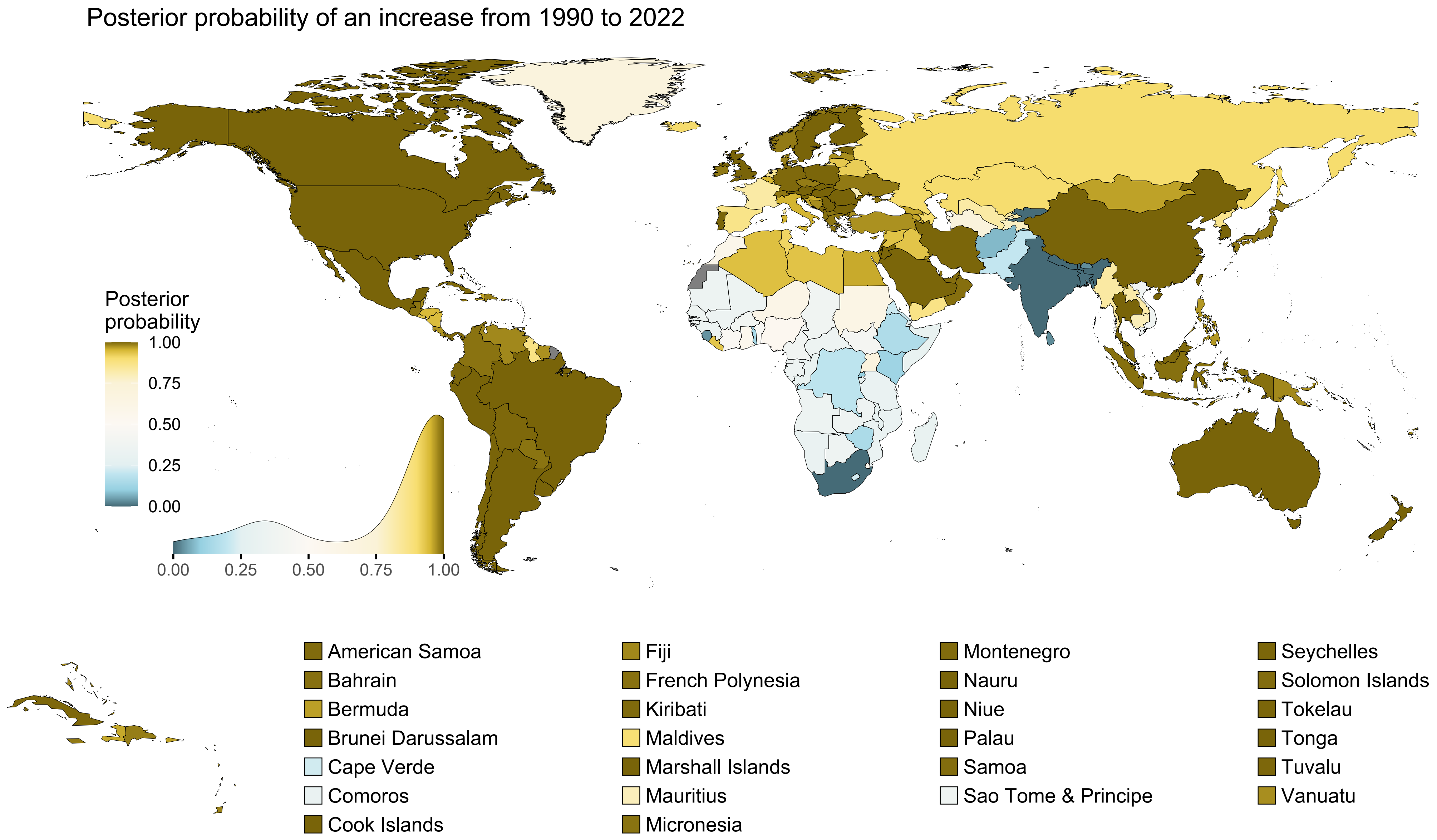

Posterior probability of an increase from 1990 to 2022
Posterior probability
1.00
0.75
0.50
0.25
0.00
0.00 0.25 0.50 0.75 1.00
American Samoa
Bahrain
Bermuda
Brunei Darussalam
Cape Verde
Comoros
Cook Islands
Fiji
French Polynesia
Kiribati
Maldives
Marshall Islands
Mauritius
Micronesia
Montenegro
Nauru
Niue
Palau
Samoa
Sao Tome & Principe
Seychelles
Solomon Islands
Tokelau
Tonga
Tuvalu
Vanuatu

**Appendix Figure 20.** Change in age-standardised proportion of combined burden composed of obesity and posterior probability that the proportion increased from 1990 to 2022, for school-aged children and adolescents.

# Girls

Change from 1990 to 2022

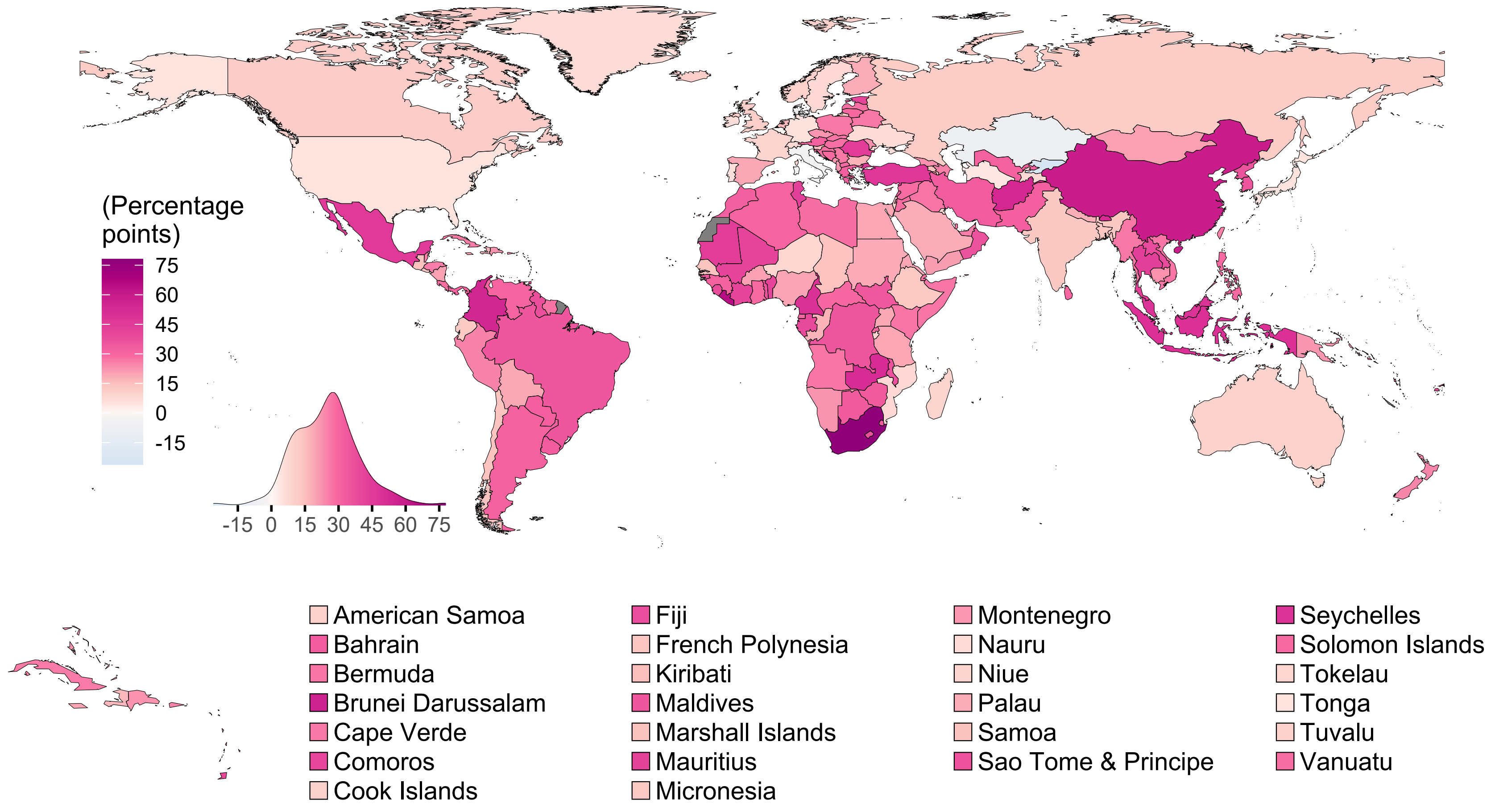


Posterior probability of an increase from 1990 to 2022

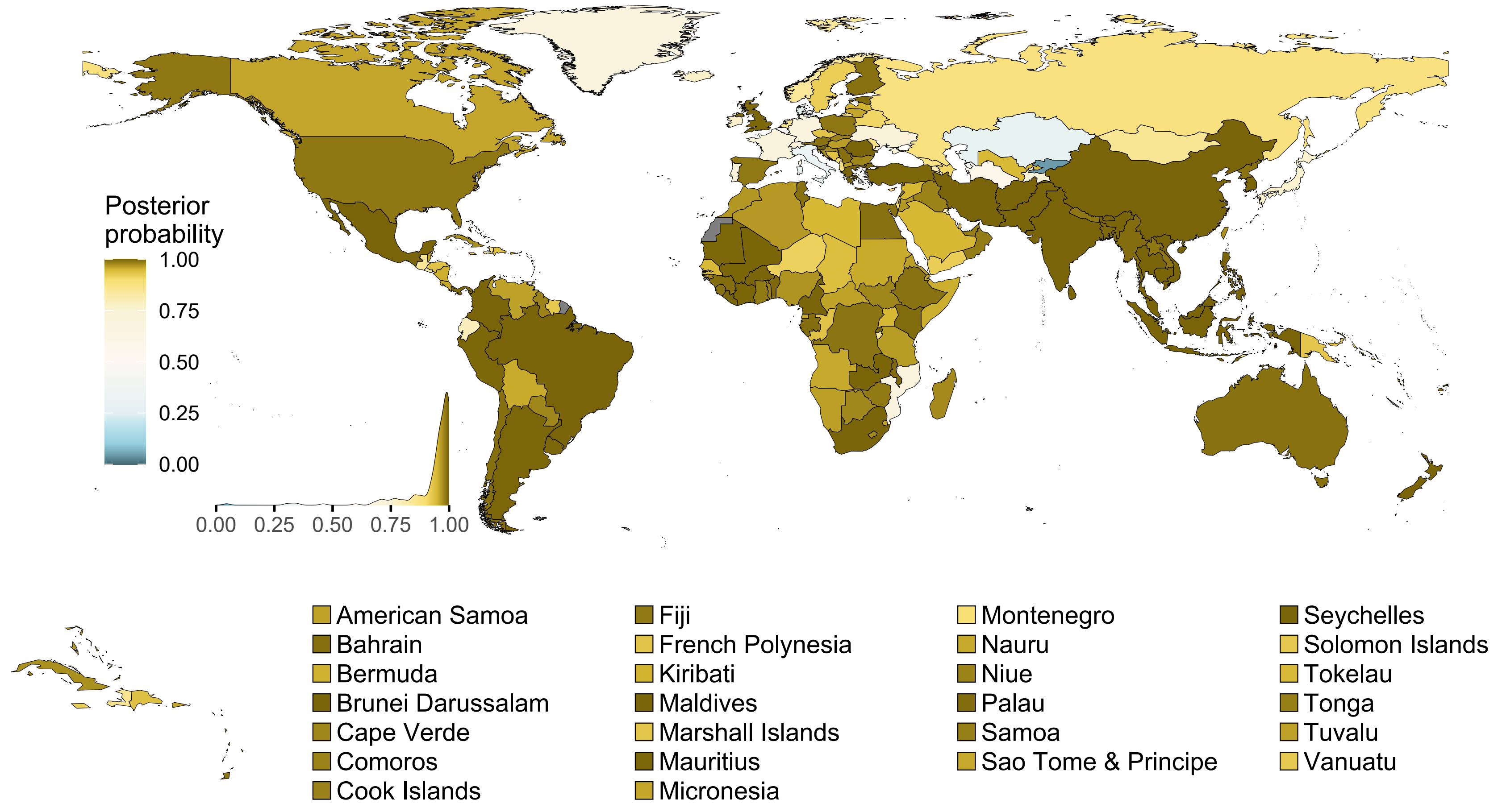


# Boys

Change from 1990 to 2022

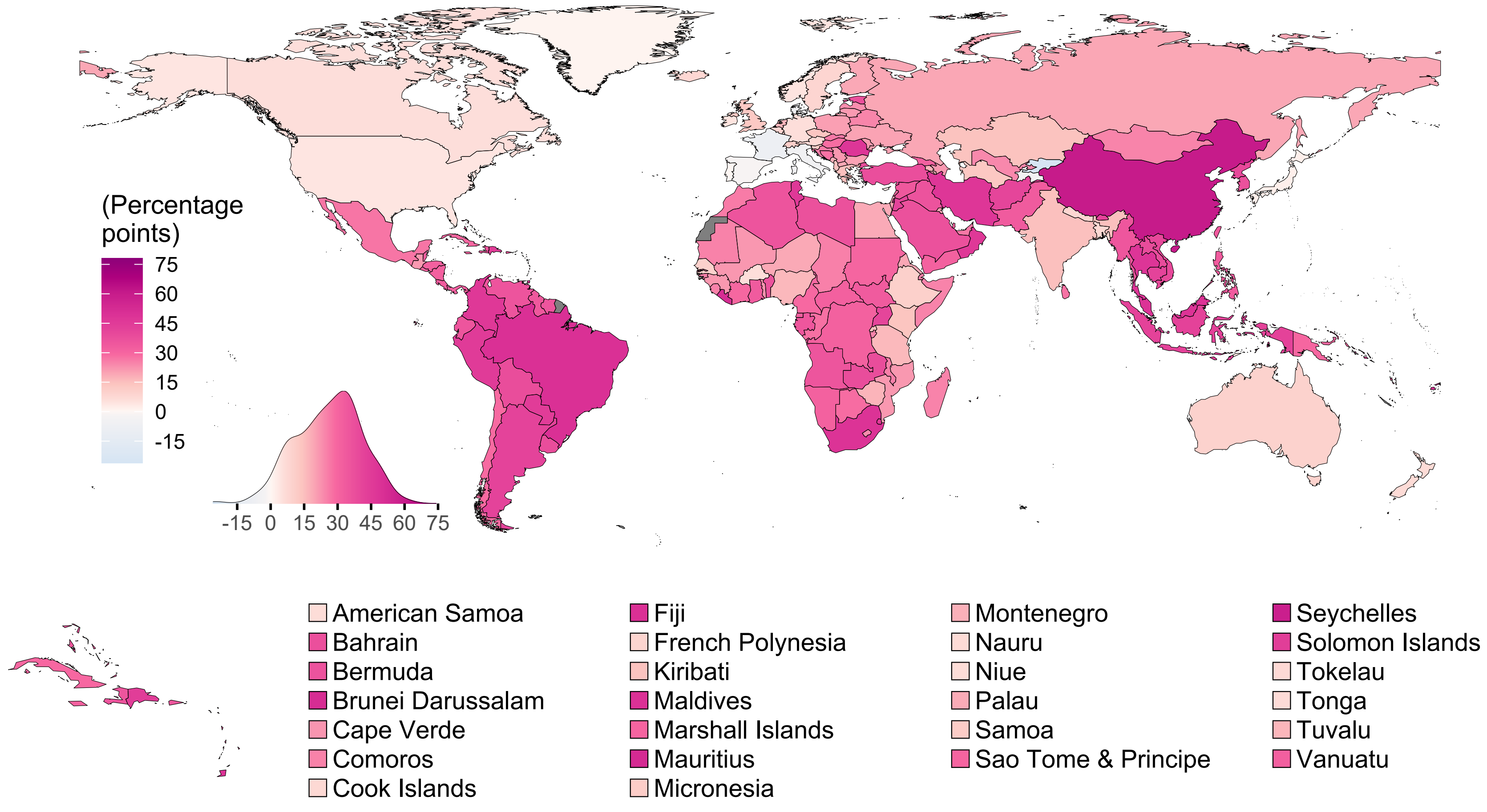


Posterior probability of an increase from 1990 to 2022

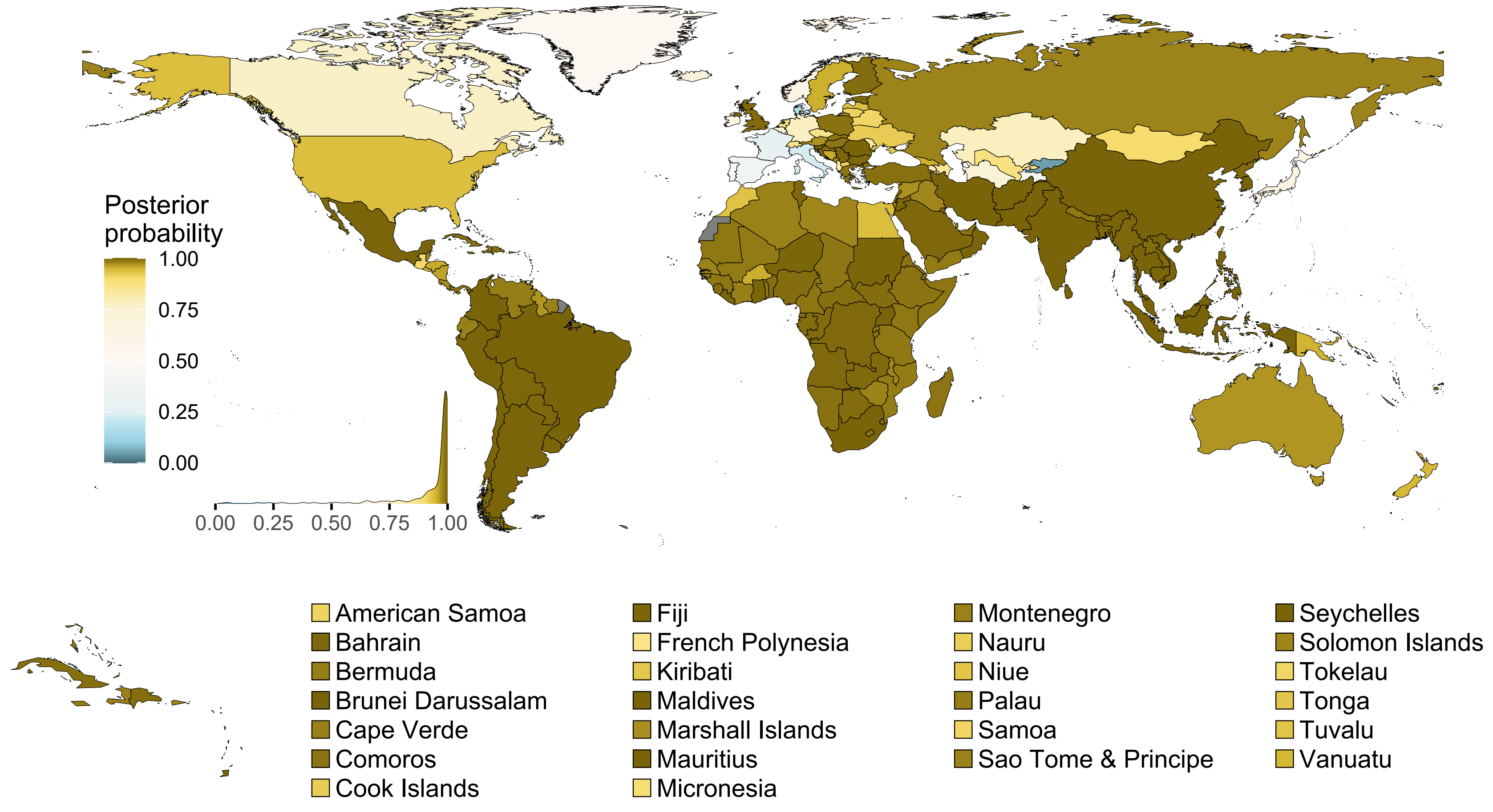

**Appendix Figure 21.** Age-standardised proportion of the combined burden of thinness and obesity composed of obesity in 1990 and 2022, in relation to the uncertainty of the proportion measured by posterior standard deviation, for school-aged children and adolescents.

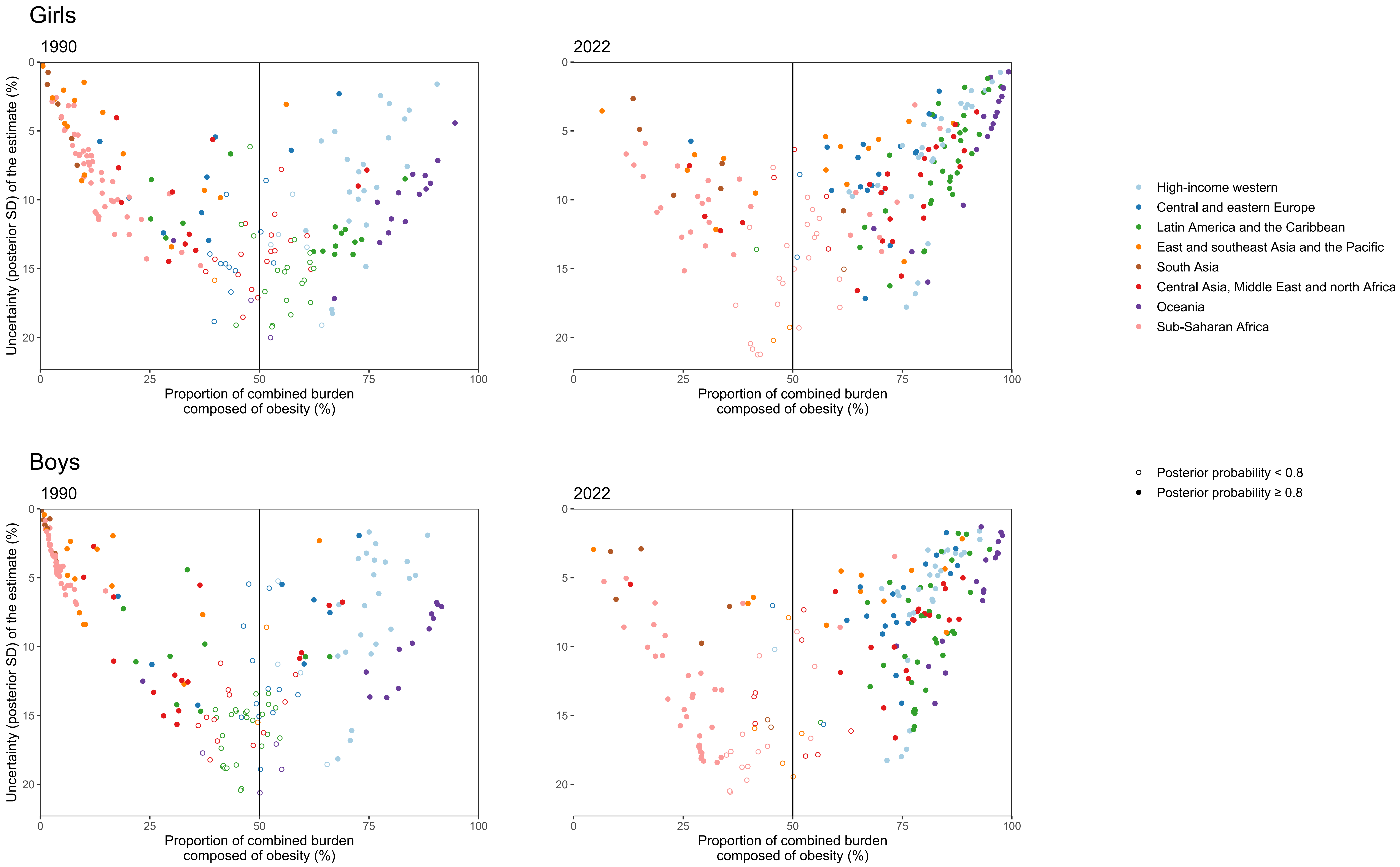

Girls
1990
2022
Boys
1990
2022
Uncertainty (posterior SD) of the estimate (%)
Proportion of combined burden composed of obesity (%)
0
5
10
15
20
25
50
75
100
High-income western
Central and eastern Europe
Latin America and the Caribbean
East and southeast Asia and the Pacific
South Asia
Central Asia, Middle East and north Africa
Oceania
Sub-Saharan Africa
Posterior probability < 0.8
Posterior probability ≥ 0.8

**Appendix Figure 22.** Change in age-standardised thinness, obesity, combined burden, and the proportion of combined burden composed of obesity from 1990 to 2022, in relation to the uncertainty of the change measured by posterior standard deviation, for school-aged children and adolescents.

# Girls

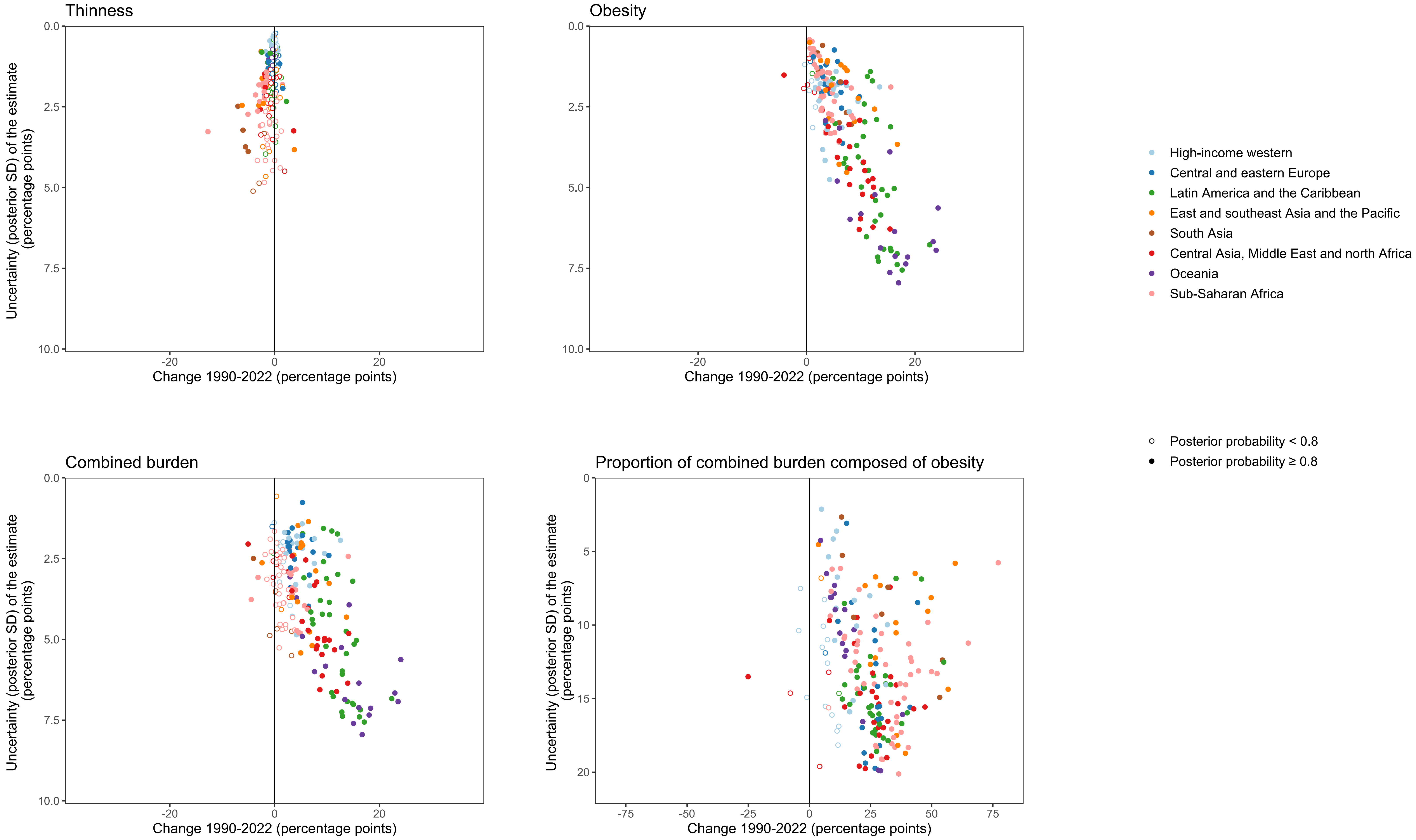
Thinness
Obesity
Combined burden
Proportion of combined burden composed of obesity
Uncertainty (posterior SD) of the estimate (percentage points)
Change 1990-2022 (percentage points)
0.0
2.5
5.0
7.5
10.0
0
5
10
15
20
-20
0
20
-75
-50
-25
0
25
50
75
High-income western
Central and eastern Europe
Latin America and the Caribbean
East and southeast Asia and the Pacific
South Asia
Central Asia, Middle East and north Africa
Oceania
Sub-Saharan Africa
Posterior probability < 0.8
Posterior probability ≥ 0.8

# Boys

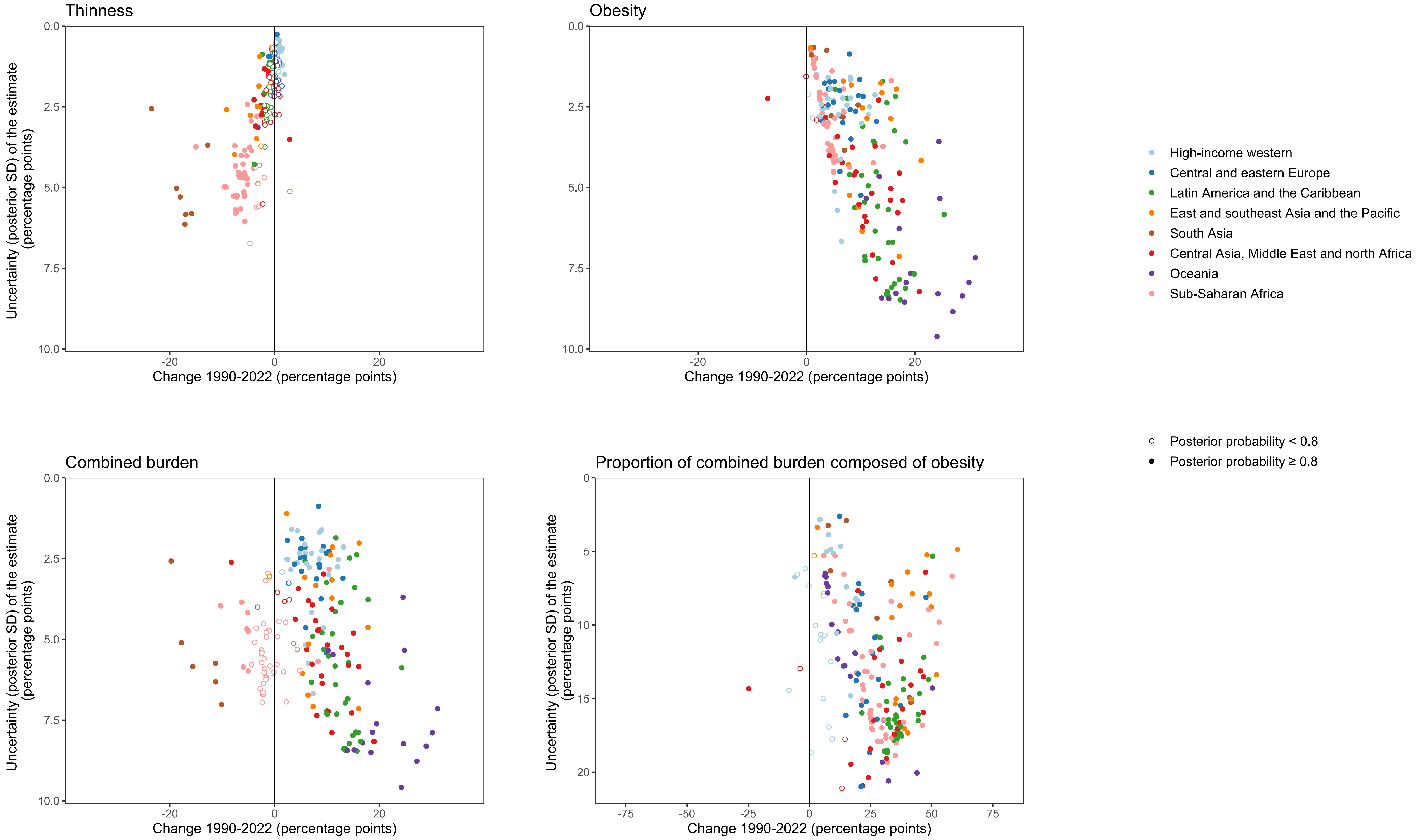
Thinness
Obesity
Combined burden
Proportion of combined burden composed of obesity
Uncertainty (posterior SD) of the estimate (percentage points)
Change 1990-2022 (percentage points)
0.0
2.5
5.0
7.5
10.0
-20
0
20
5
10
15
20
-75
-50
-25
25
50
75
High-income western
Central and eastern Europe
Latin America and the Caribbean
East and southeast Asia and the Pacific
South Asia
Central Asia, Middle East and north Africa
Oceania
Sub-Saharan Africa
Posterior probability < 0.8
Posterior probability ≥ 0.8

**Appendix Figure 23.** Comparison of age-standardised prevalence of thinness in school-aged children and adolescents and of underweight in adults.

Each point shows one country. The dotted line shows the ordinary least squares linear regression fit.

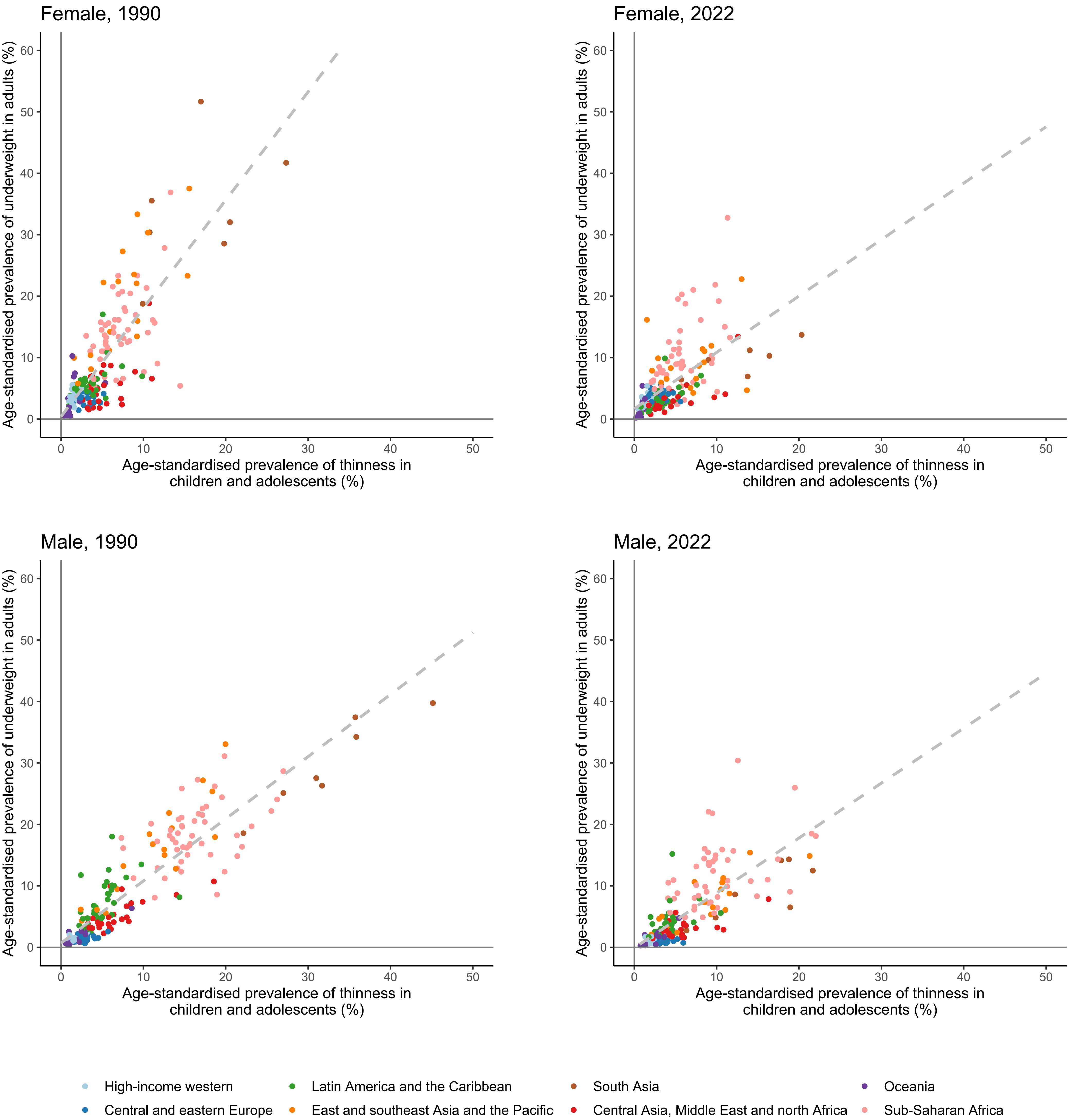
Female, 1990
Female, 2022
Male, 1990
Male, 2022
Age-standardised prevalence of underweight in adults (%)
Age-standardised prevalence of thinness in children and adolescents (%)
0
10
20
30
40
50
60
High-income western
Central and eastern Europe
Latin America and the Caribbean
East and southeast Asia and the Pacific
South Asia
Central Asia, Middle East and north Africa
Oceania
Sub-Saharan Africa

**Appendix Figure 24.** Comparison of age-standardised prevalence of obesity in school-aged children and adolescents and in adults.

Each point shows one country. The dotted line shows the ordinary least squares linear regression fit.

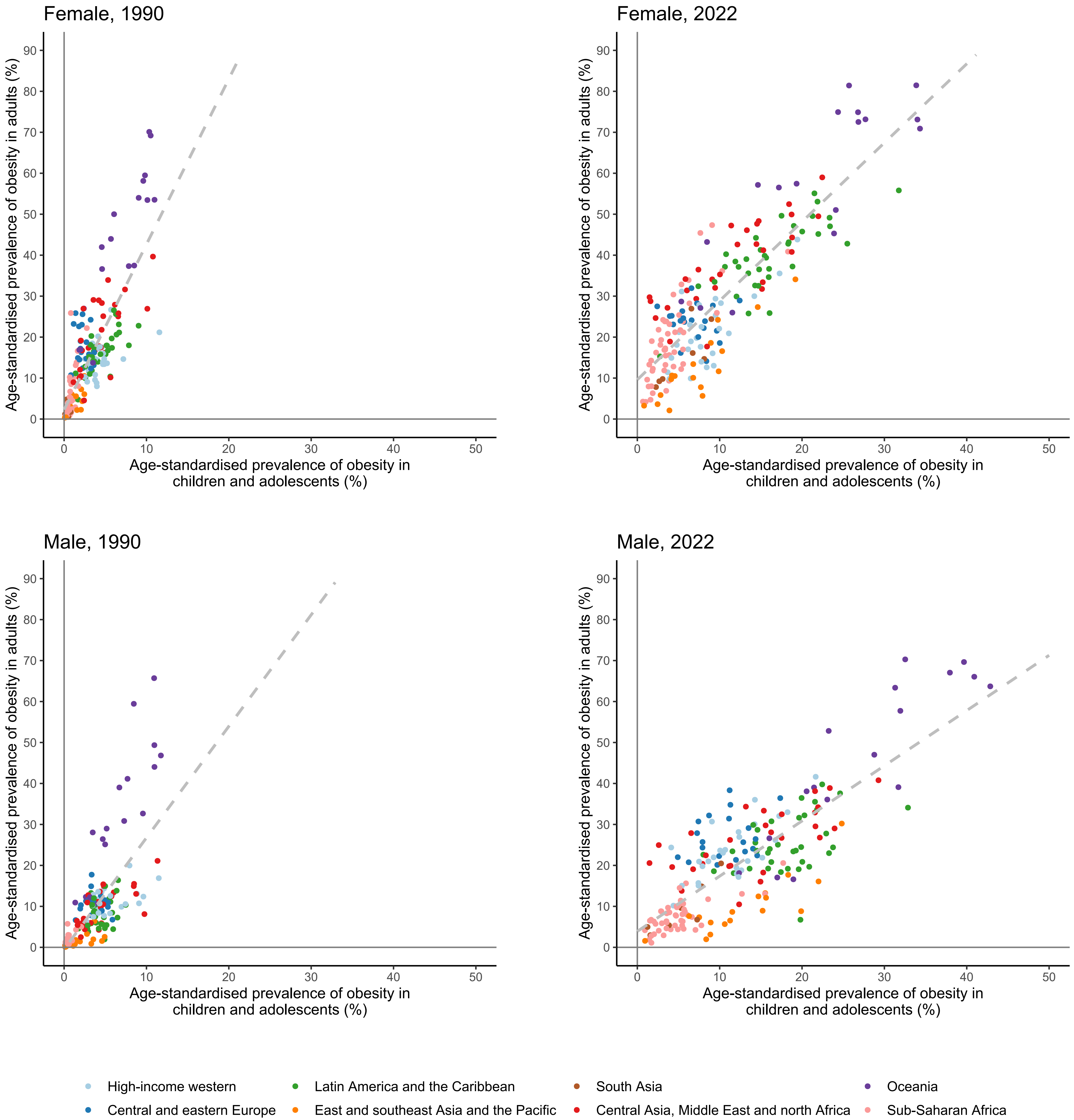
Female, 1990
Female, 2022
Male, 1990
Male, 2022
Age-standardised prevalence of obesity in adults (%)
Age-standardised prevalence of obesity in children and adolescents (%)
0
10
20
30
40
50
60
70
80
90
High-income western
Central and eastern Europe
Latin America and the Caribbean
East and southeast Asia and the Pacific
South Asia
Central Asia, Middle East and north Africa
Oceania
Sub-Saharan Africa

**Appendix Figure 25.** Age standardised prevalence of thinness, obesity and their combined burden for school-aged children and adolescents, and underweight, obesity and their combined burden for adults, from 1990 to 2022, by country.

Trends were obtained by connecting the annual estimates. The solid lines show the posterior mean and the shaded areas the 95% uncertainty interval.

# Afghanistan

## School-aged children and adolescents

### Girls
2 studies (2 national)

### Boys
1 study (1 national)

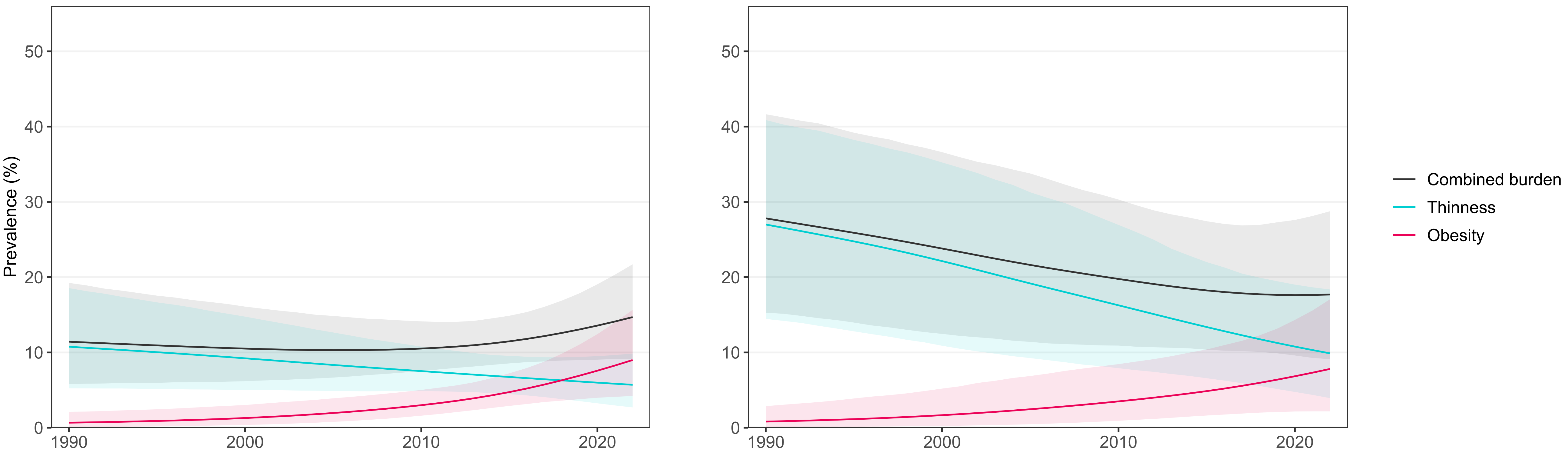


## Adults

### Women
2 studies (2 national)

### Men
1 study (1 national)

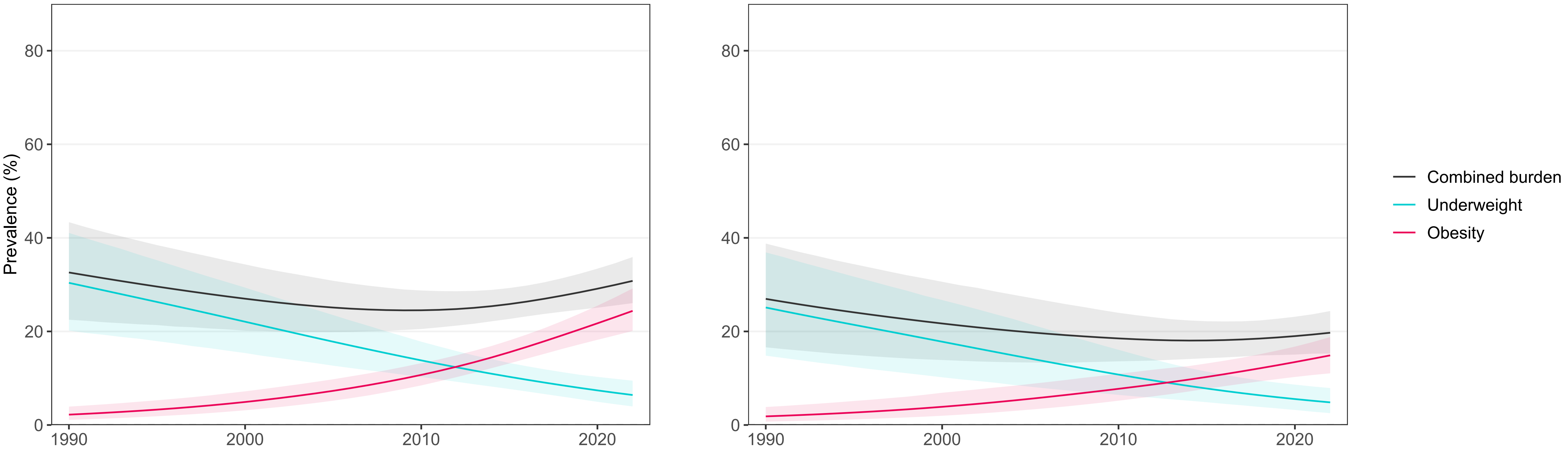

# Albania

## School-aged children and adolescents

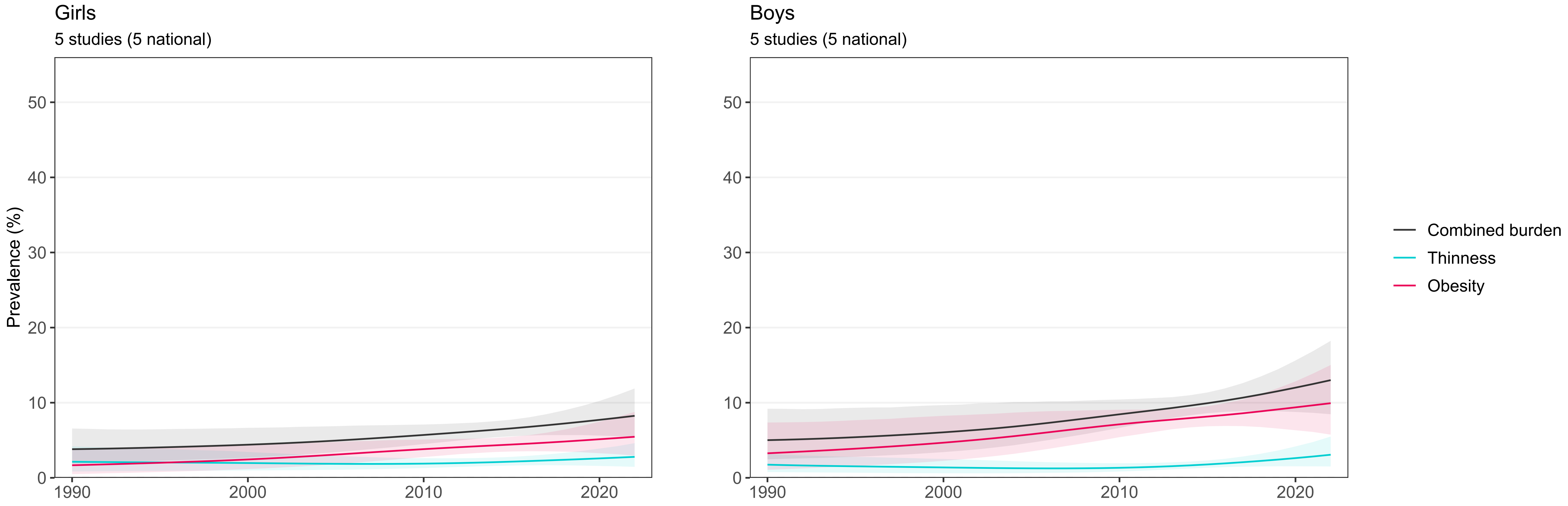


## Adults

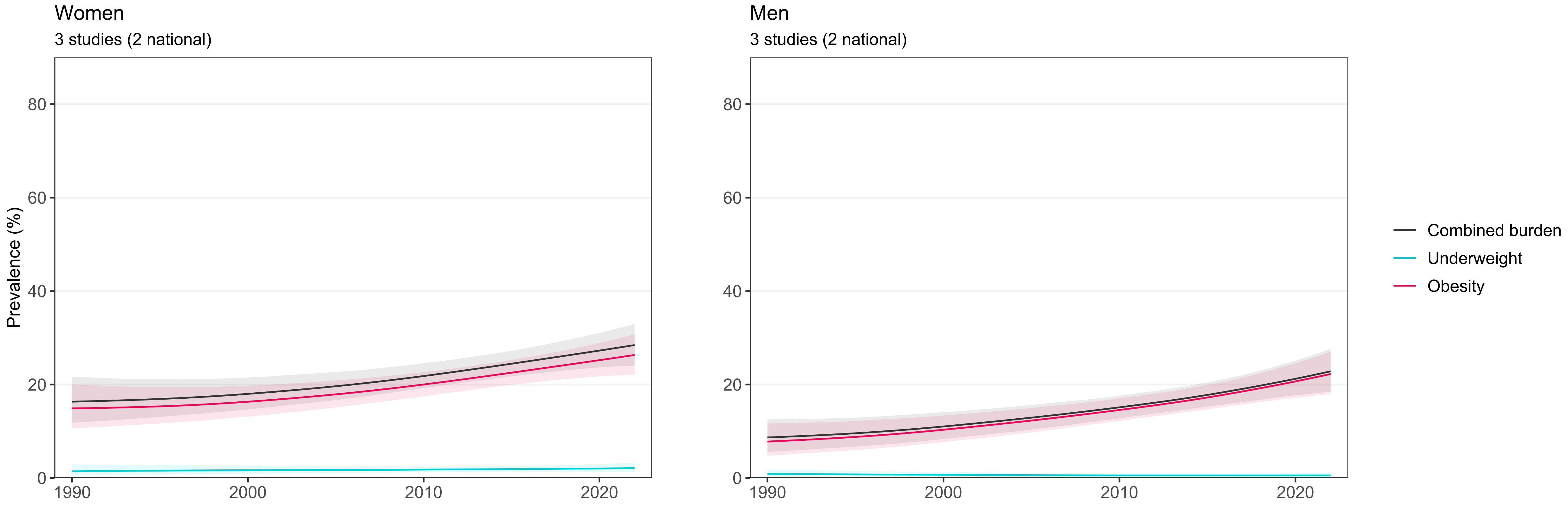

# Algeria

## School-aged children and adolescents

### Girls

1 study (1 national)

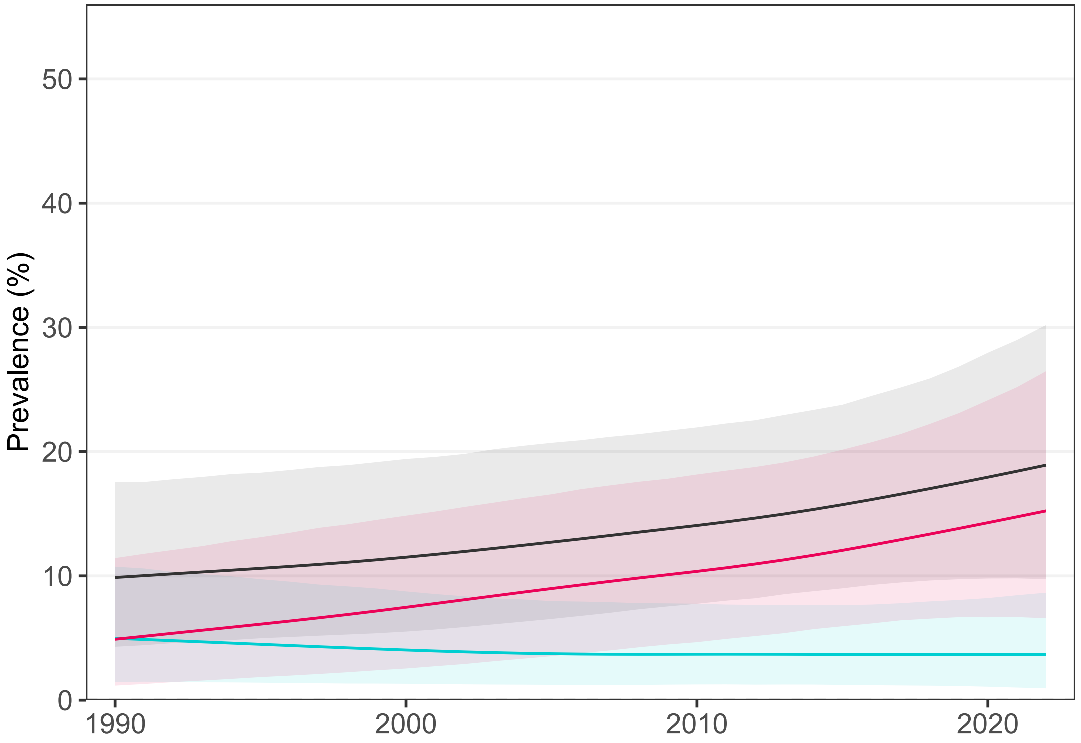


### Boys

1 study (1 national)

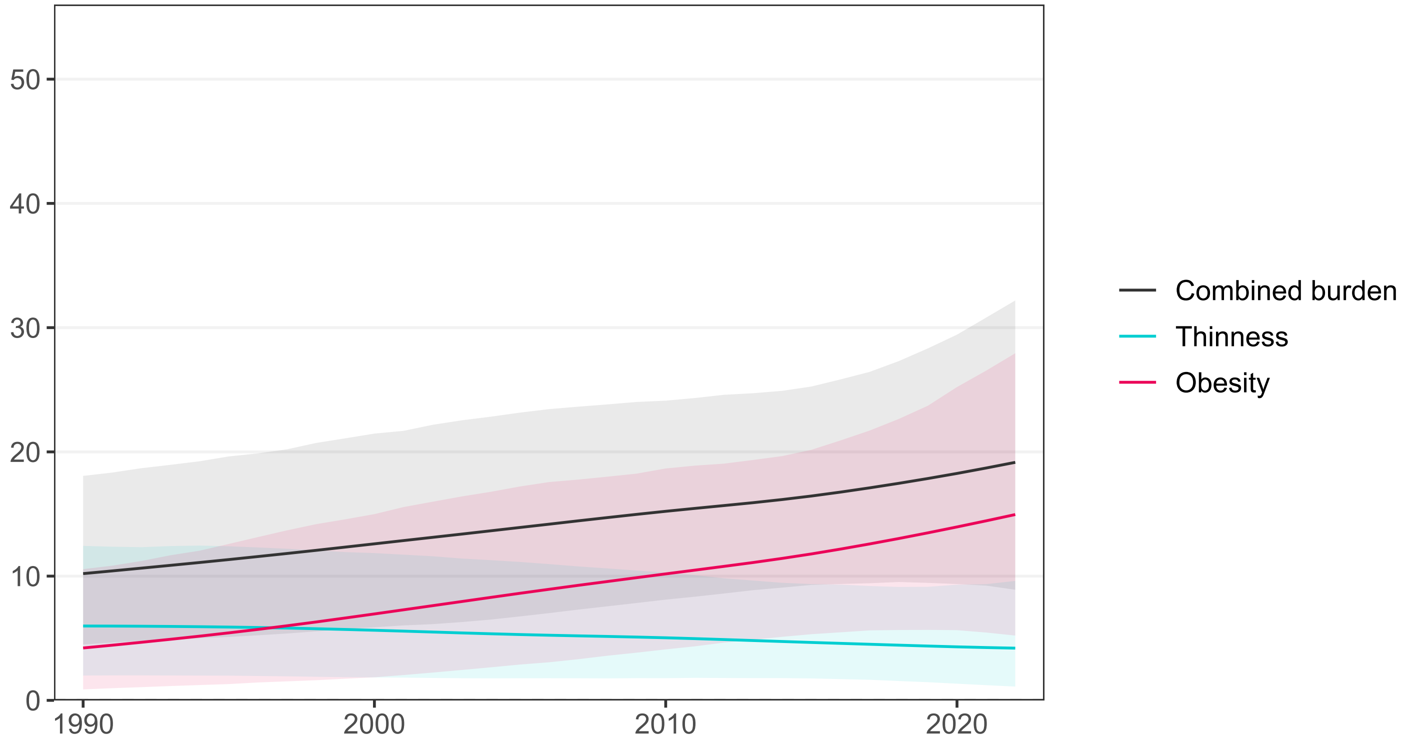


## Adults

### Women

4 studies (2 national)

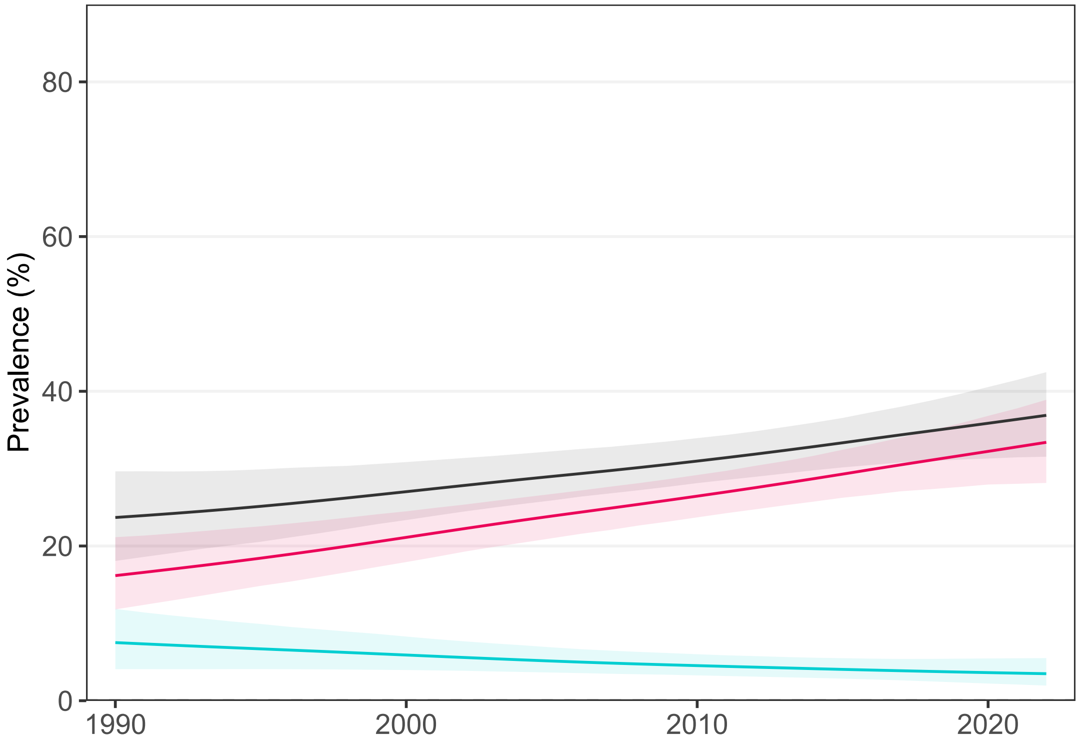


### Men

4 studies (2 national)

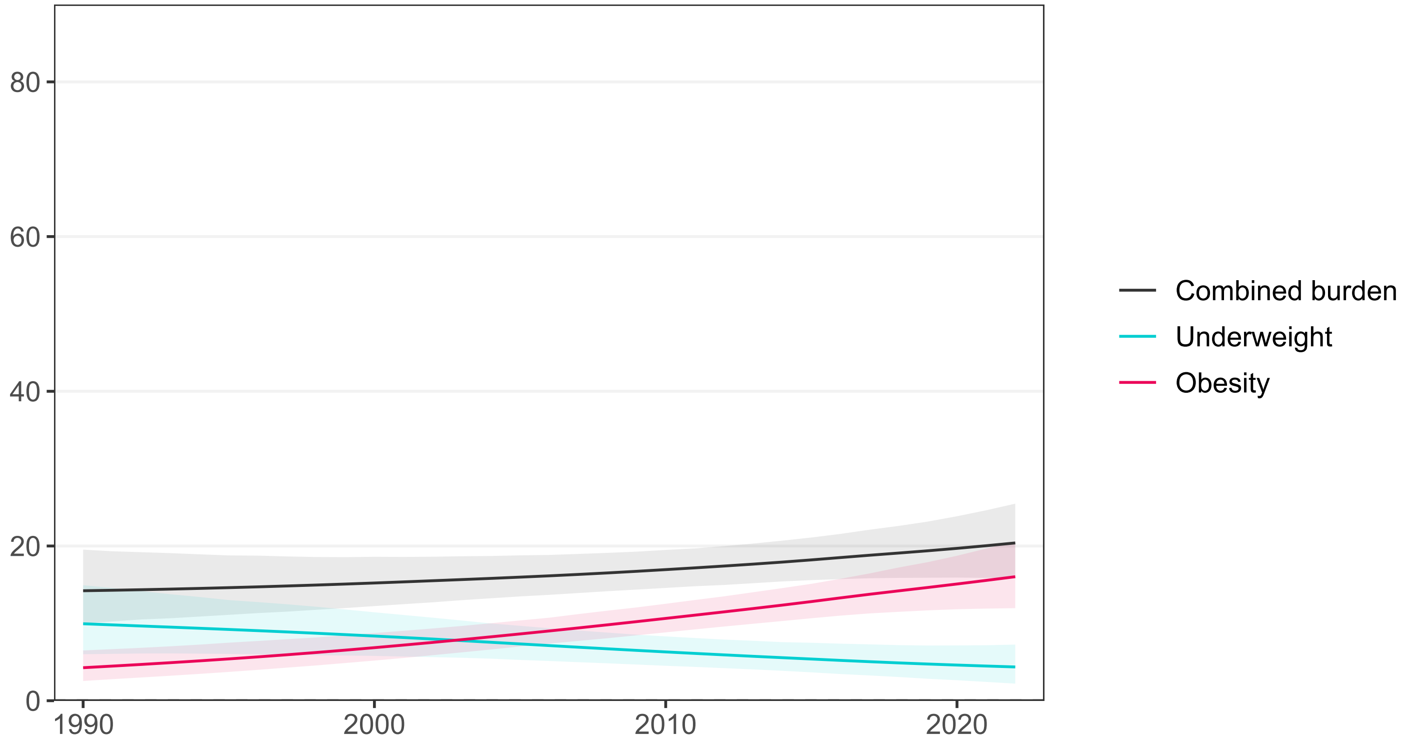

# American Samoa

## School-aged children and adolescents

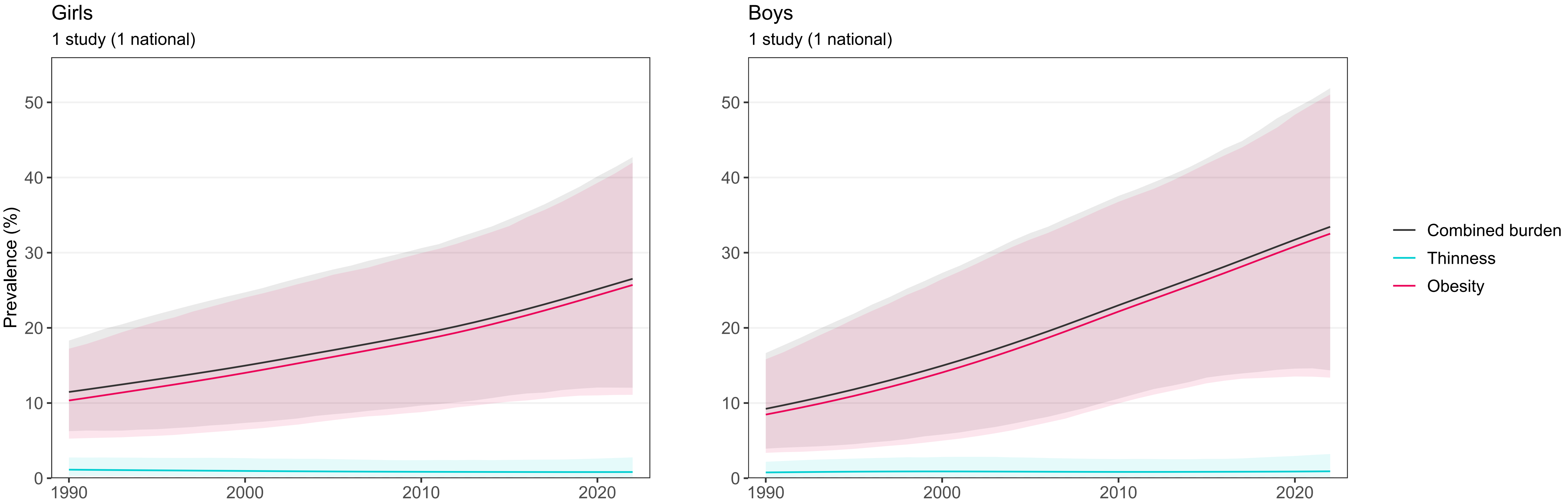


## Adults

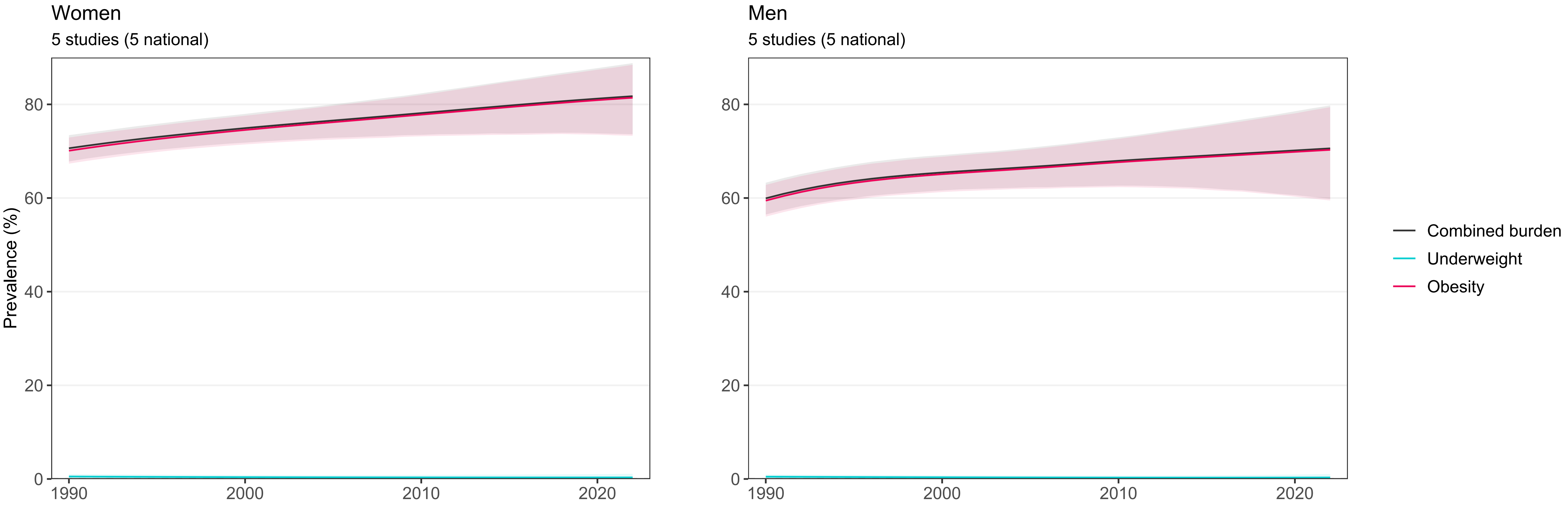

# Andorra

## School-aged children and adolescents

### Girls

No studies

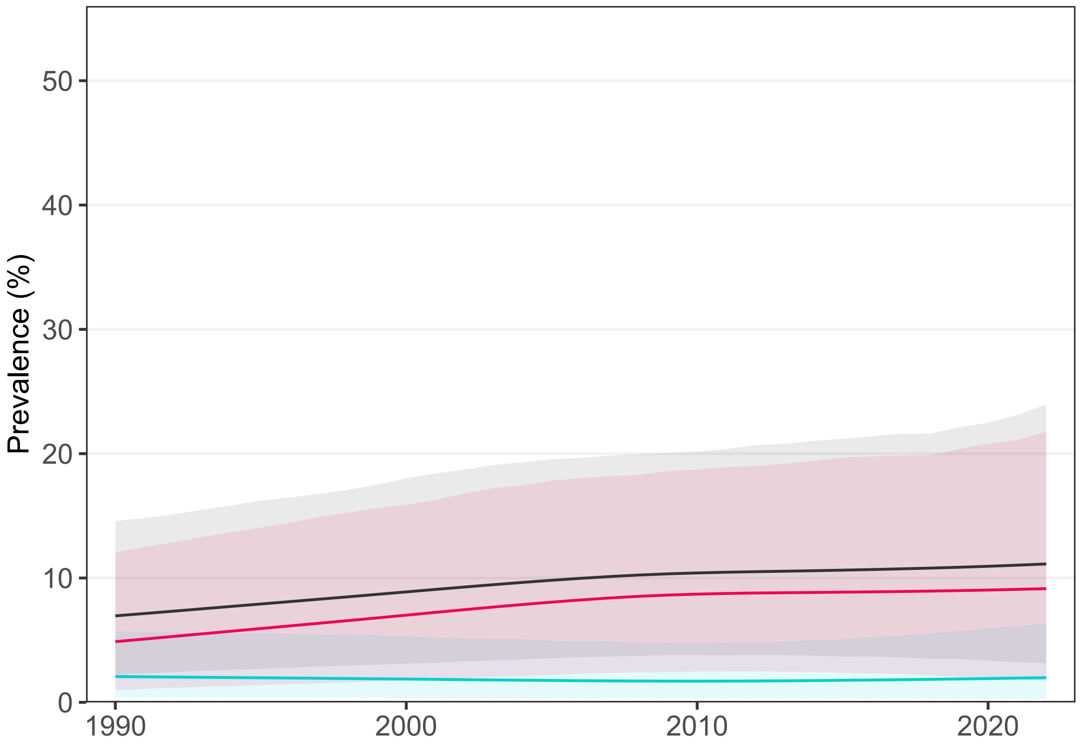


### Boys

No studies

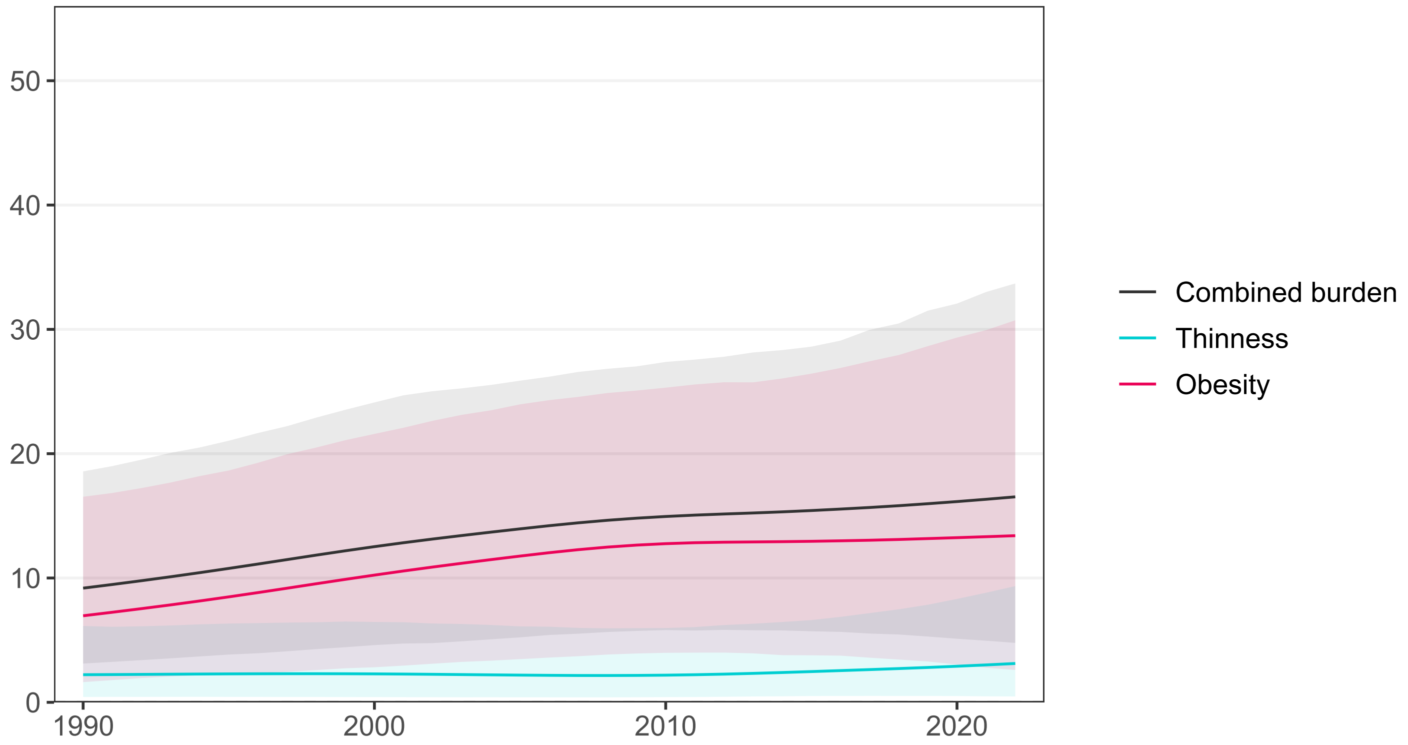


## Adults

### Women

1 study (1 national)

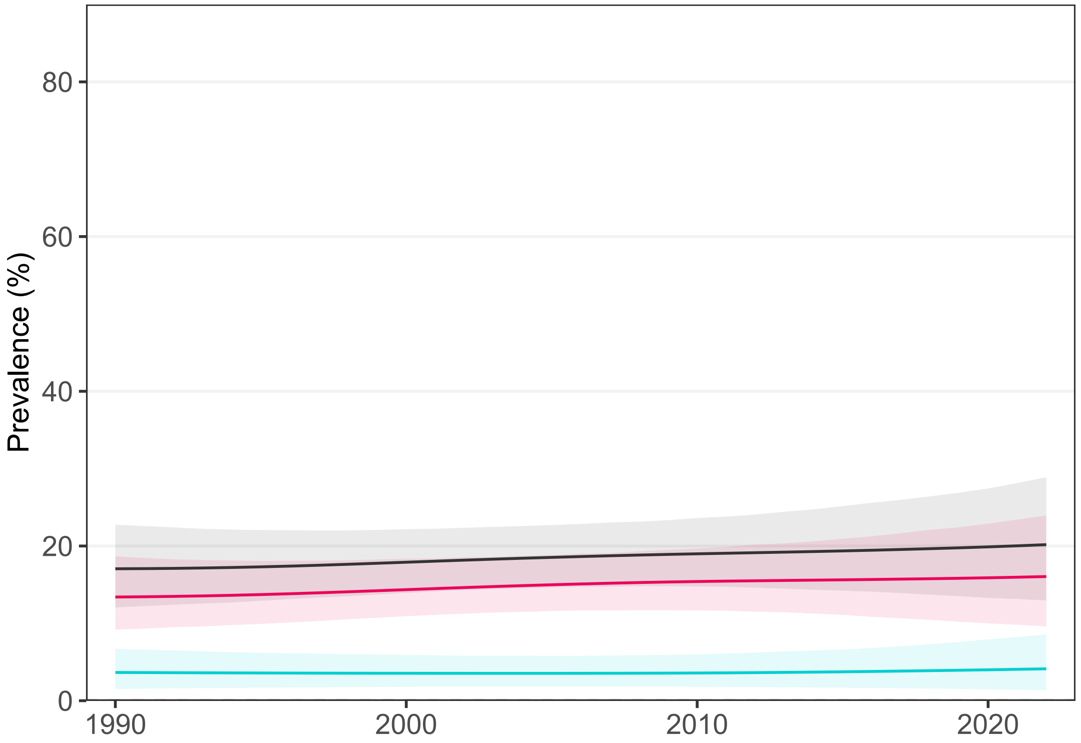


### Men

1 study (1 national)

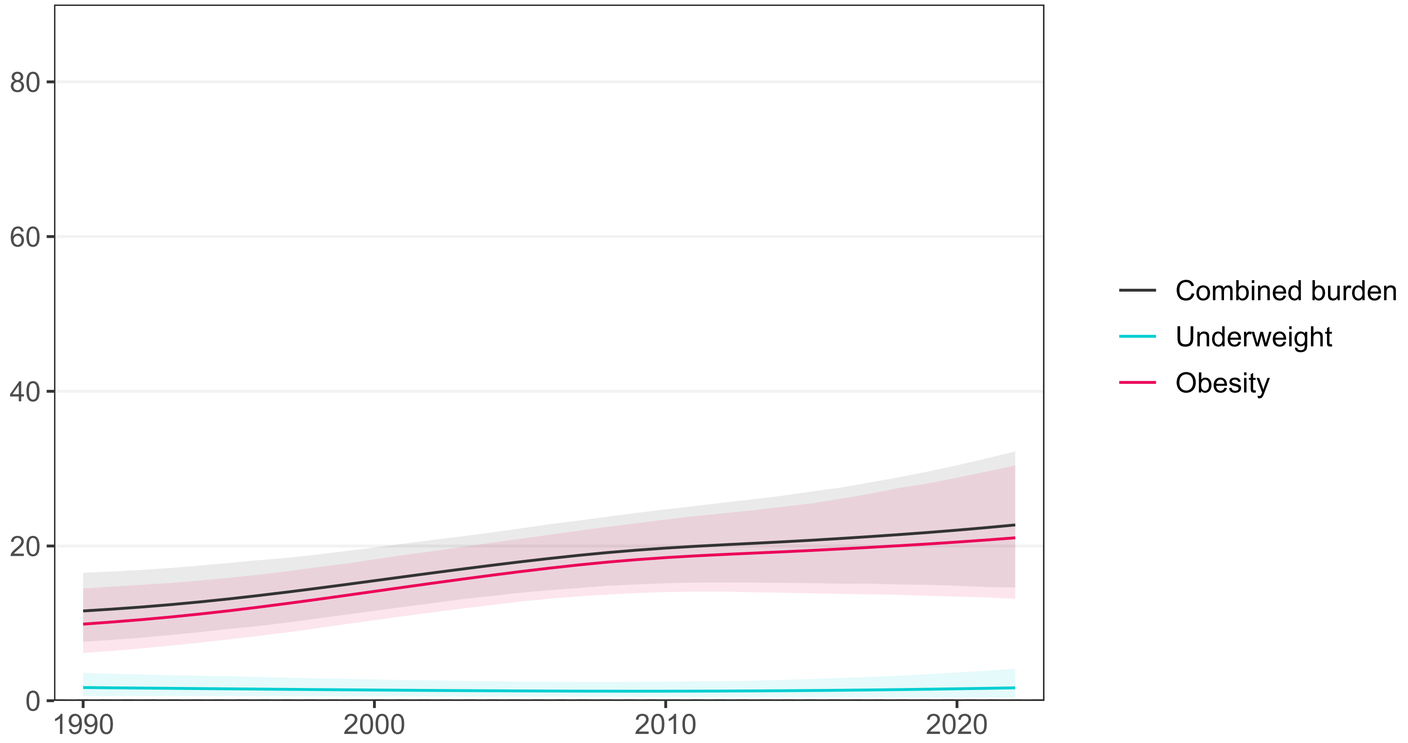

# Angola

## School-aged children and adolescents

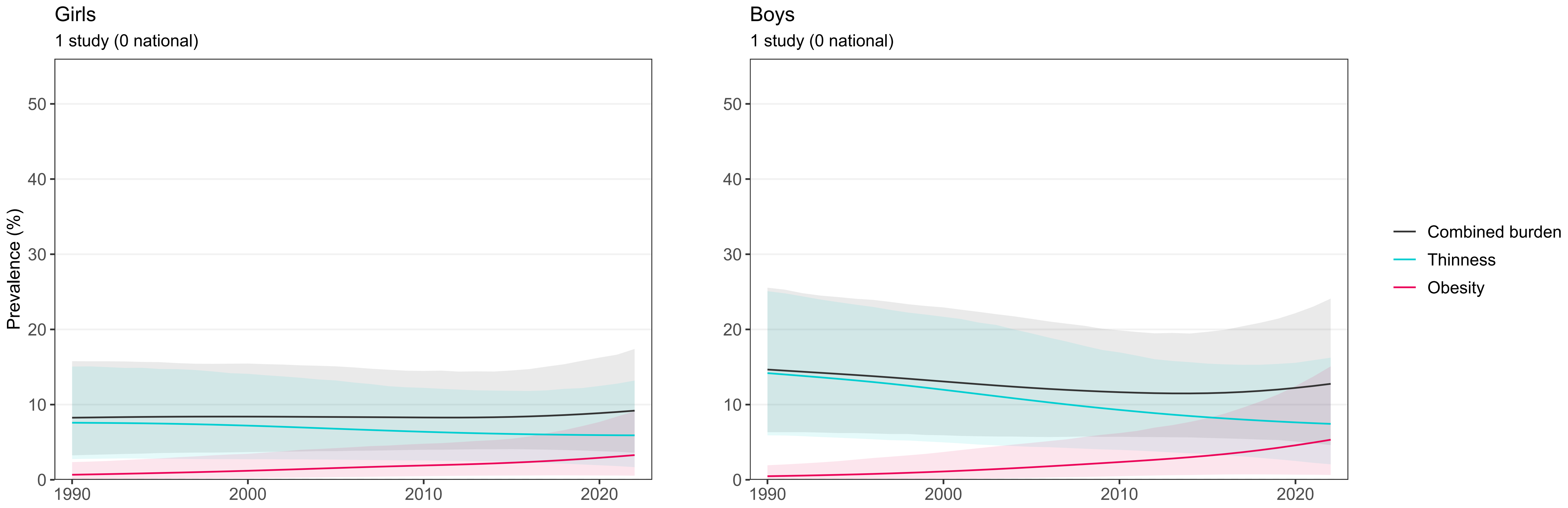


## Adults

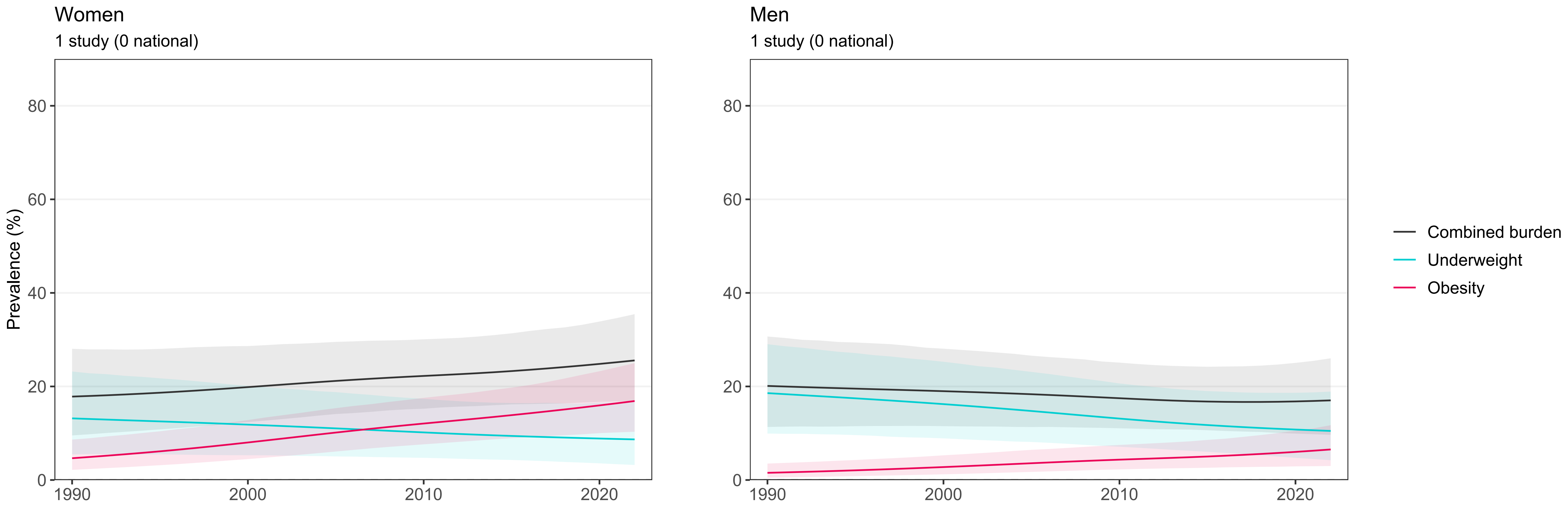

# Antigua & Barbuda

## School-aged children and adolescents

### Girls

1 study (1 national)

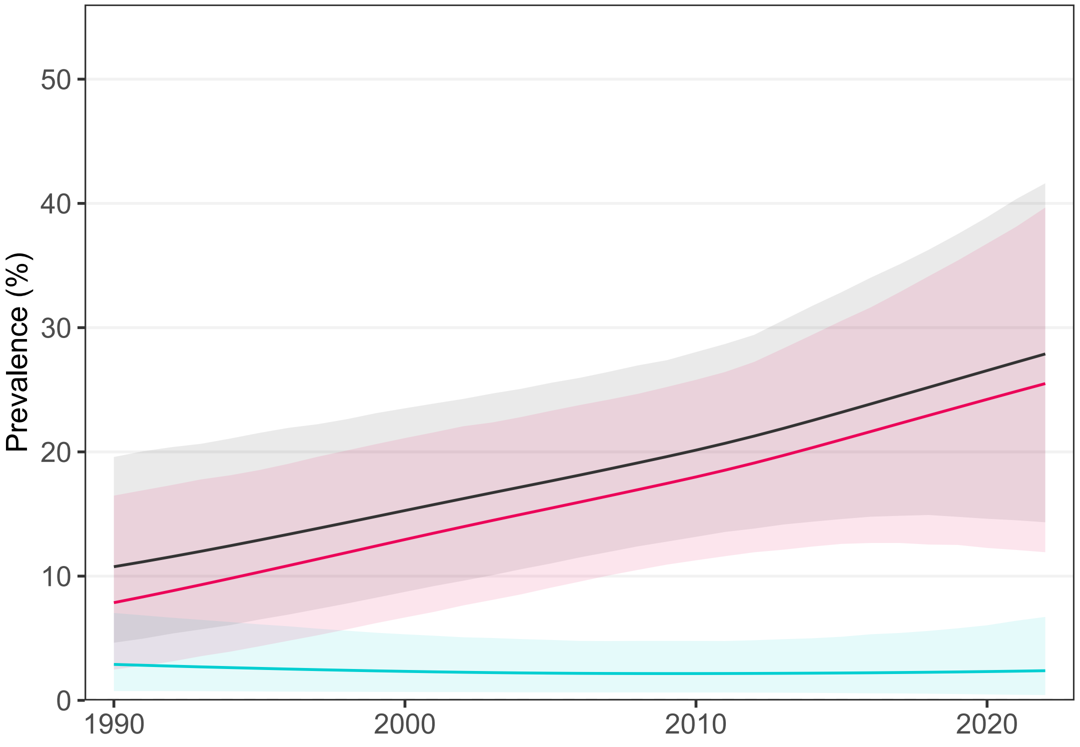


### Boys

1 study (1 national)

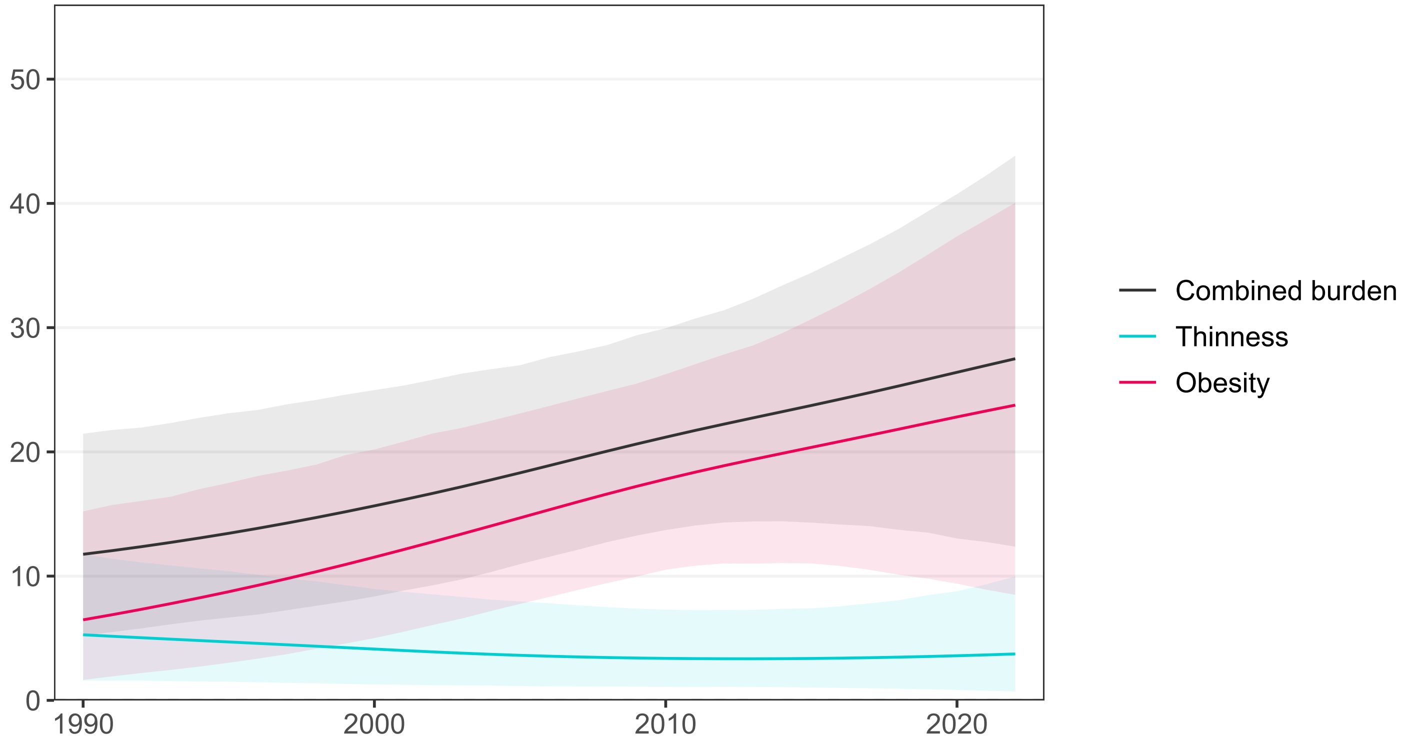


## Adults

### Women

No studies

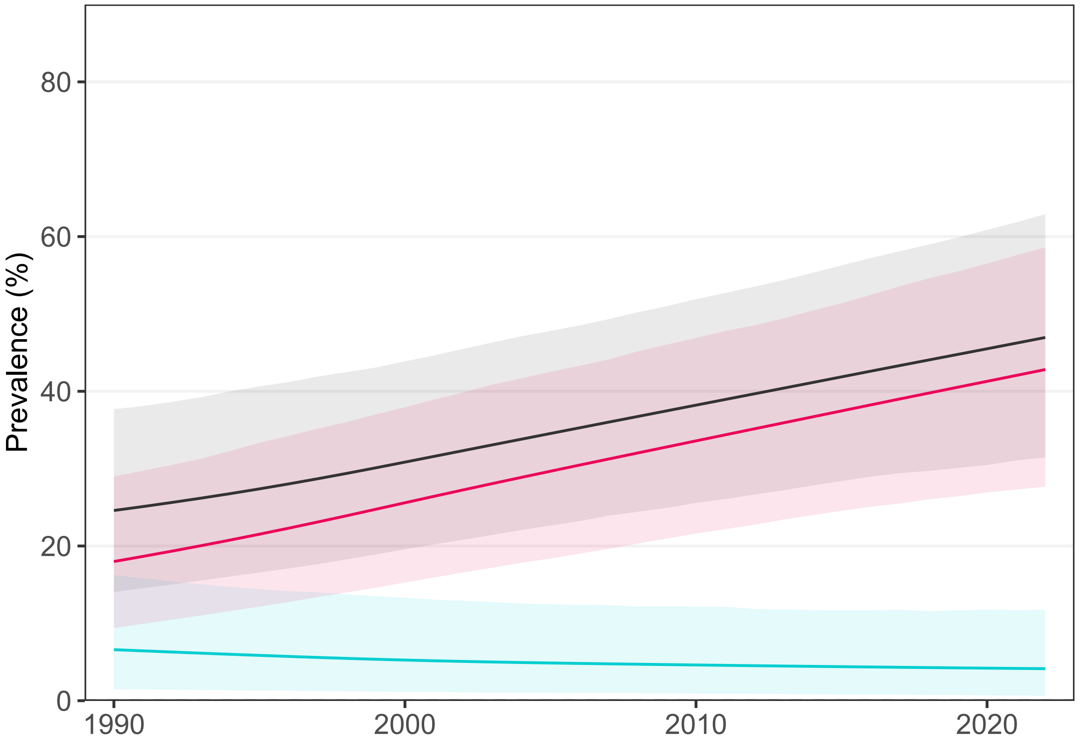


### Men

No studies

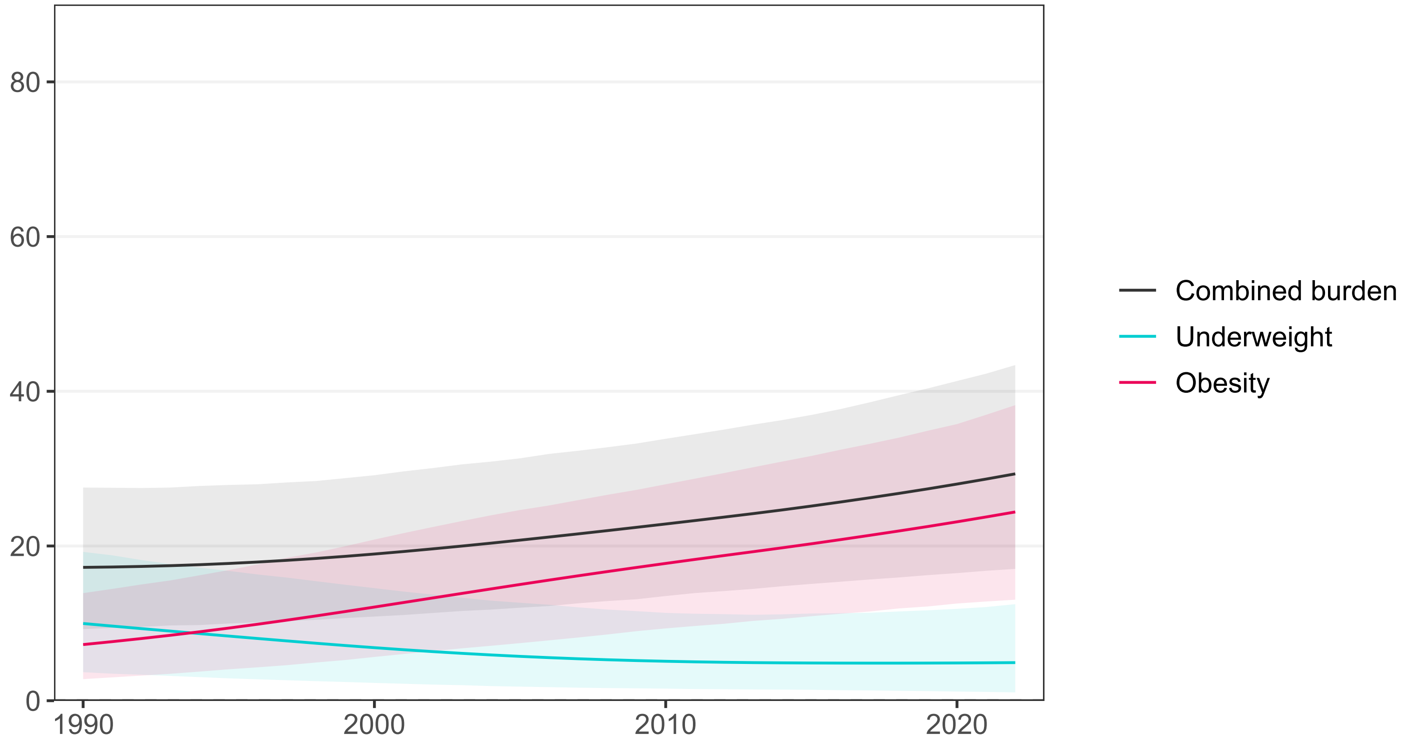

# Argentina

## School-aged children and adolescents

### Girls

8 studies (5 national)

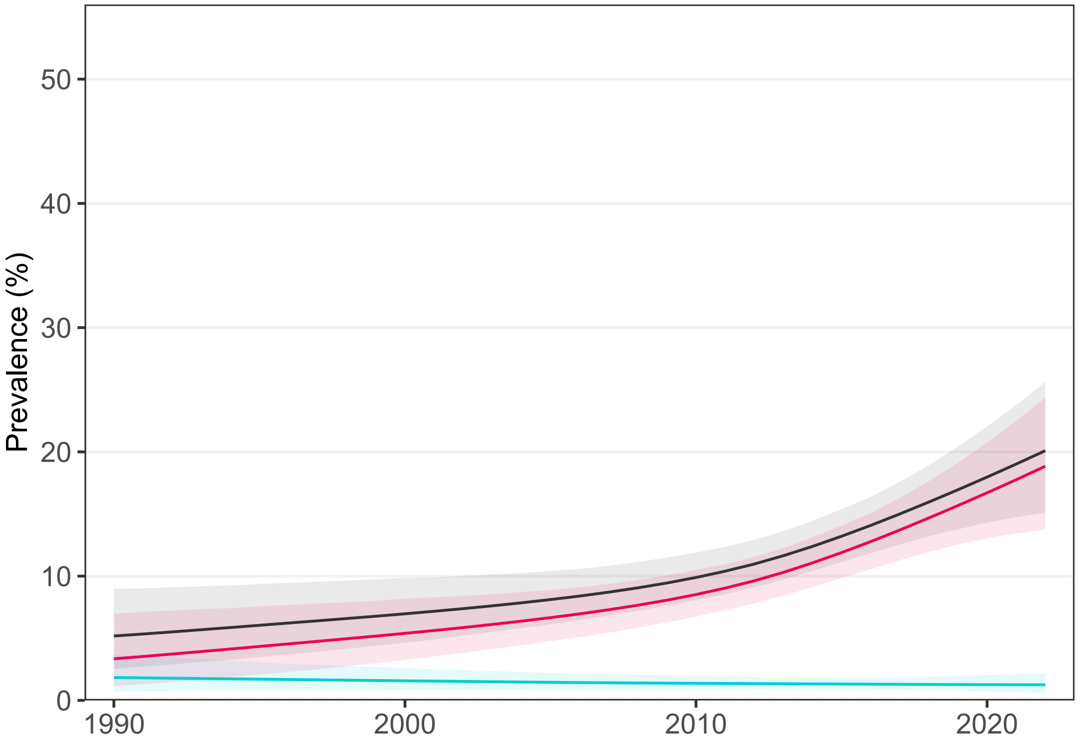


### Boys

7 studies (4 national)

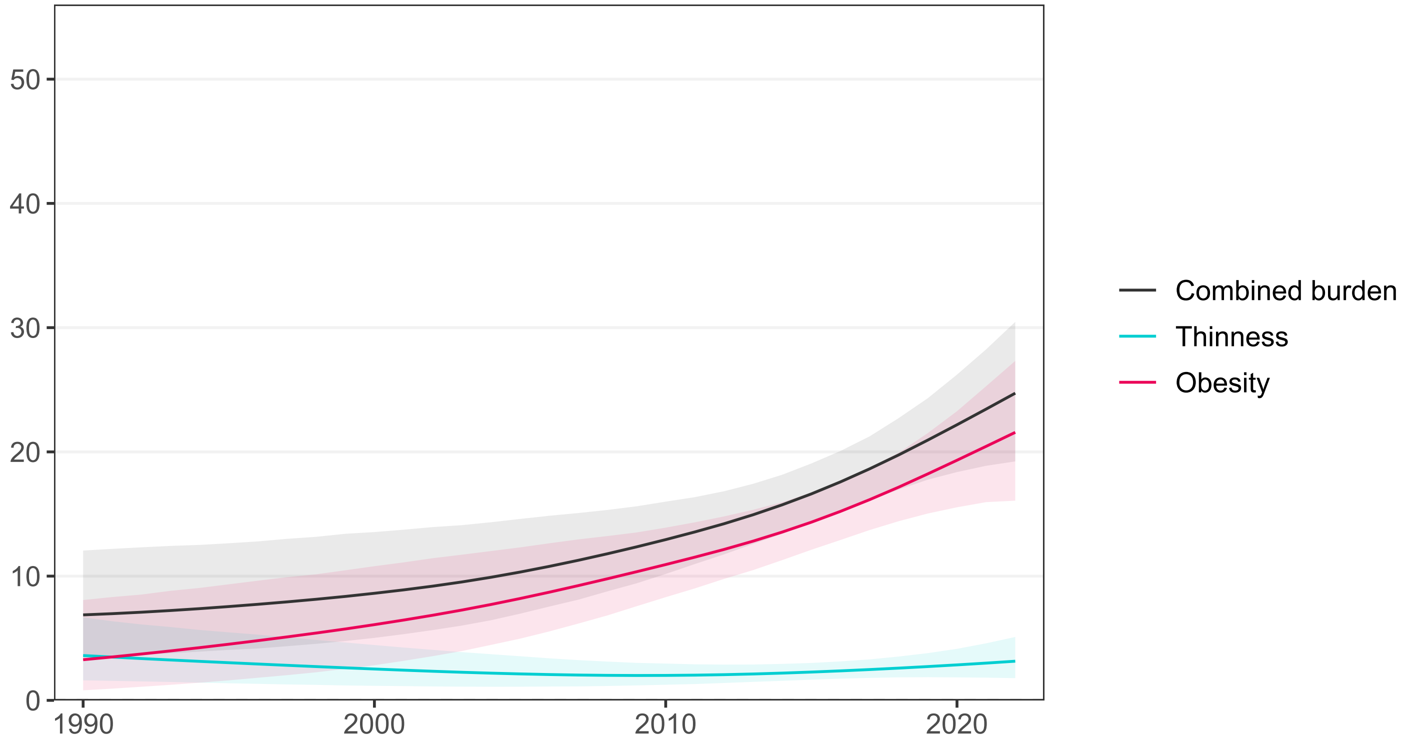


## Adults

### Women

14 studies (4 national)

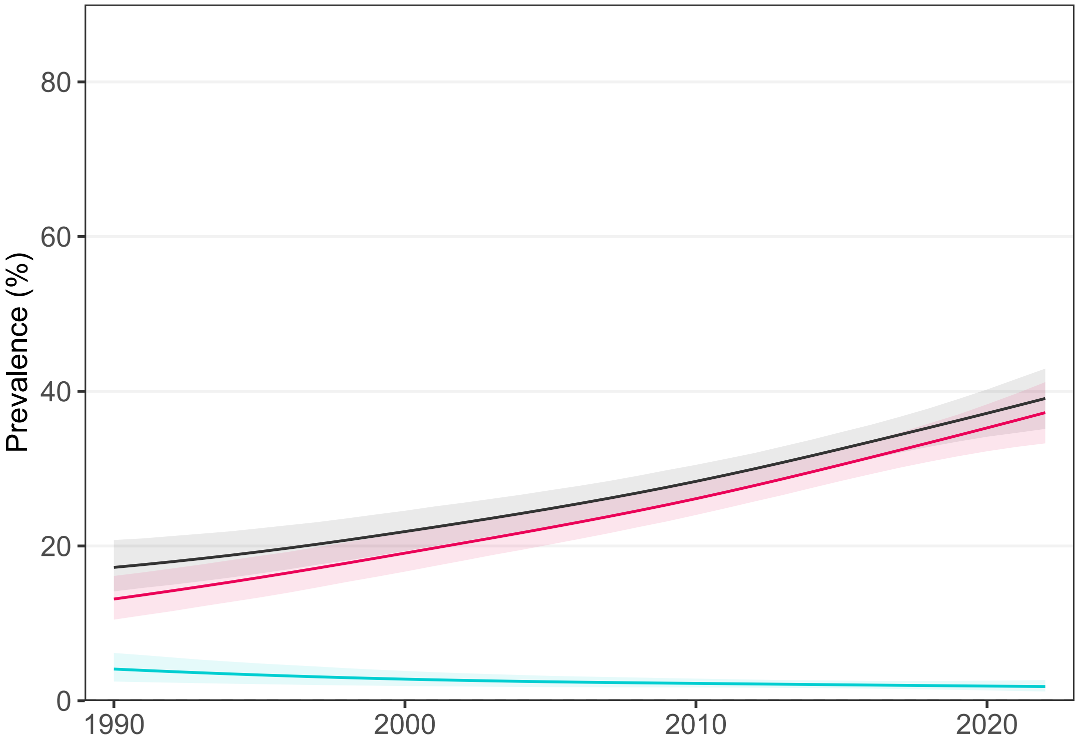


### Men

13 studies (3 national)

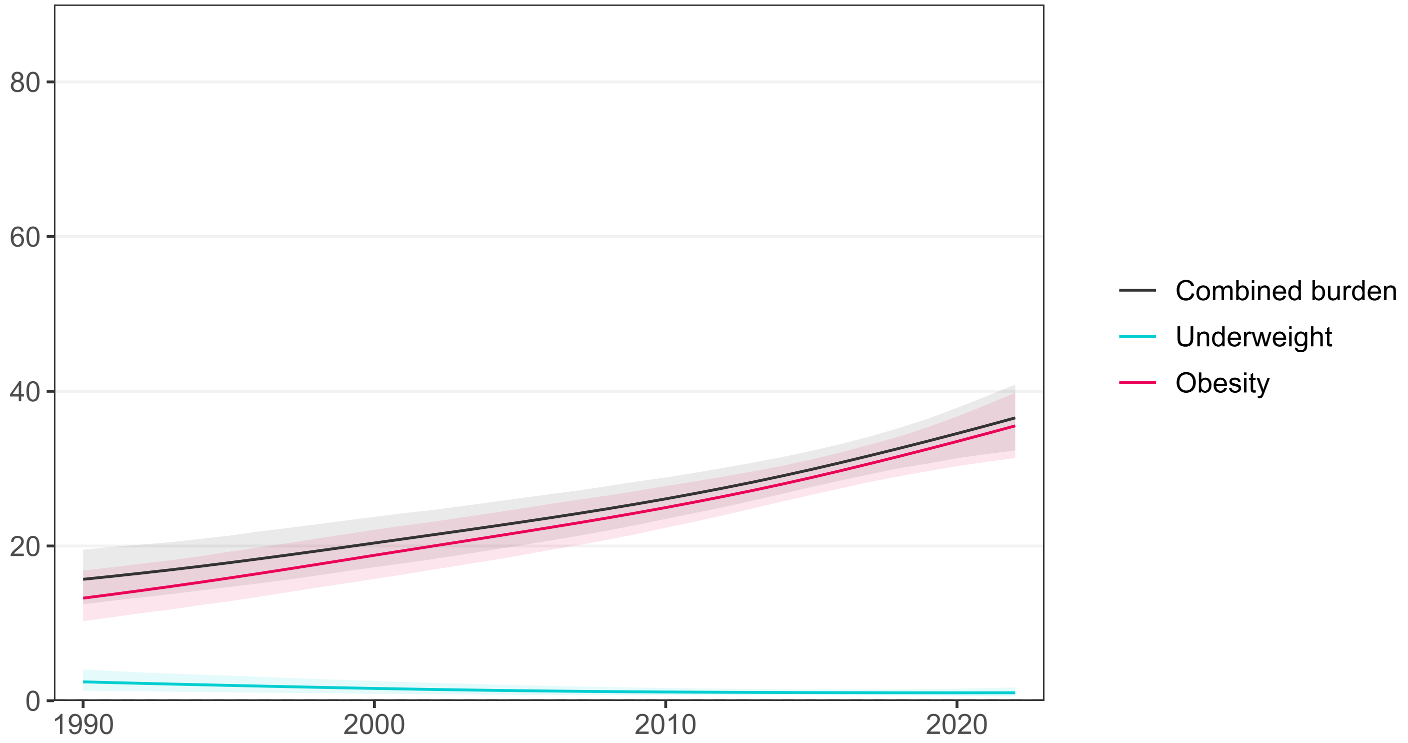

# Armenia

## School-aged children and adolescents

### Girls

6 studies (6 national)

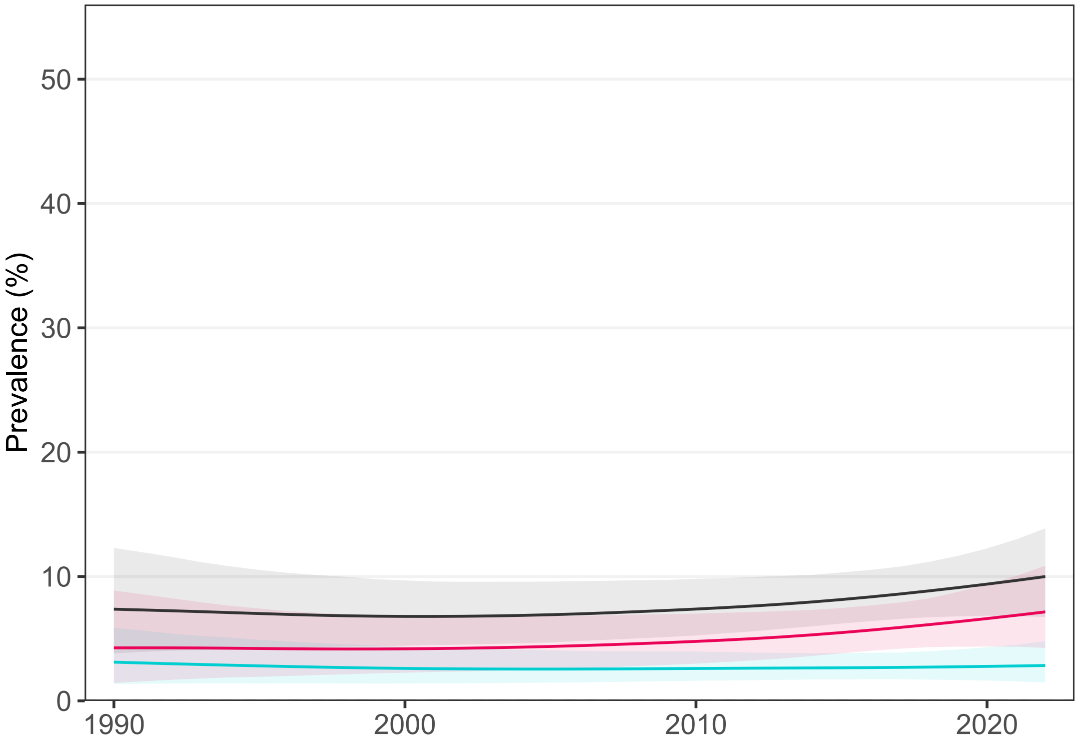


### Boys

3 studies (3 national)

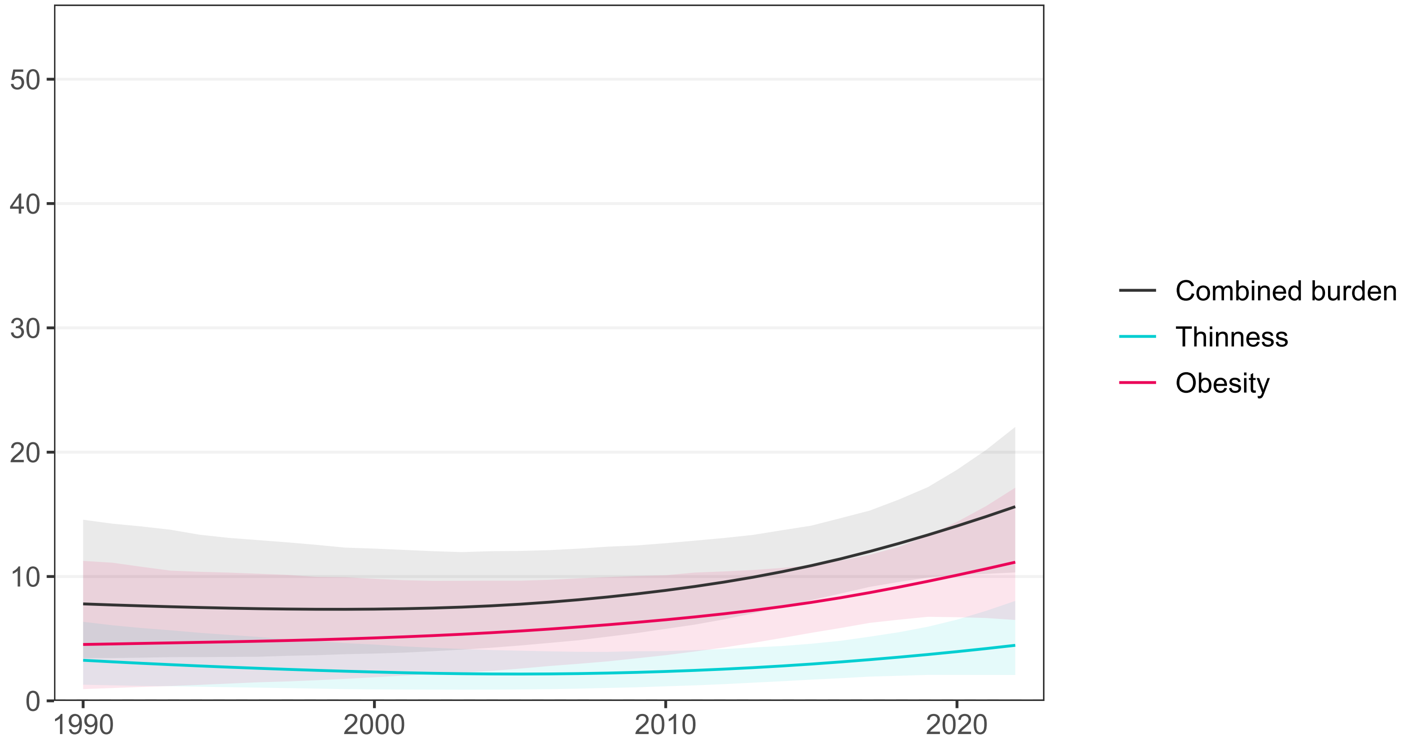


## Adults

### Women

5 studies (5 national)

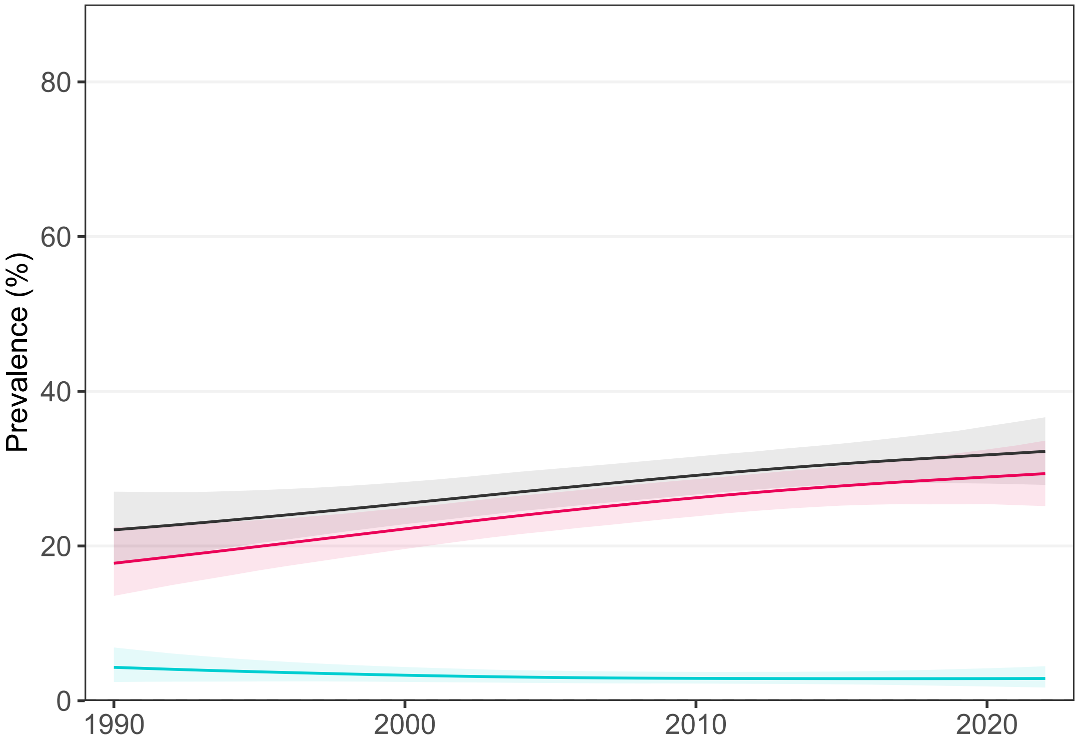


### Men

2 studies (2 national)

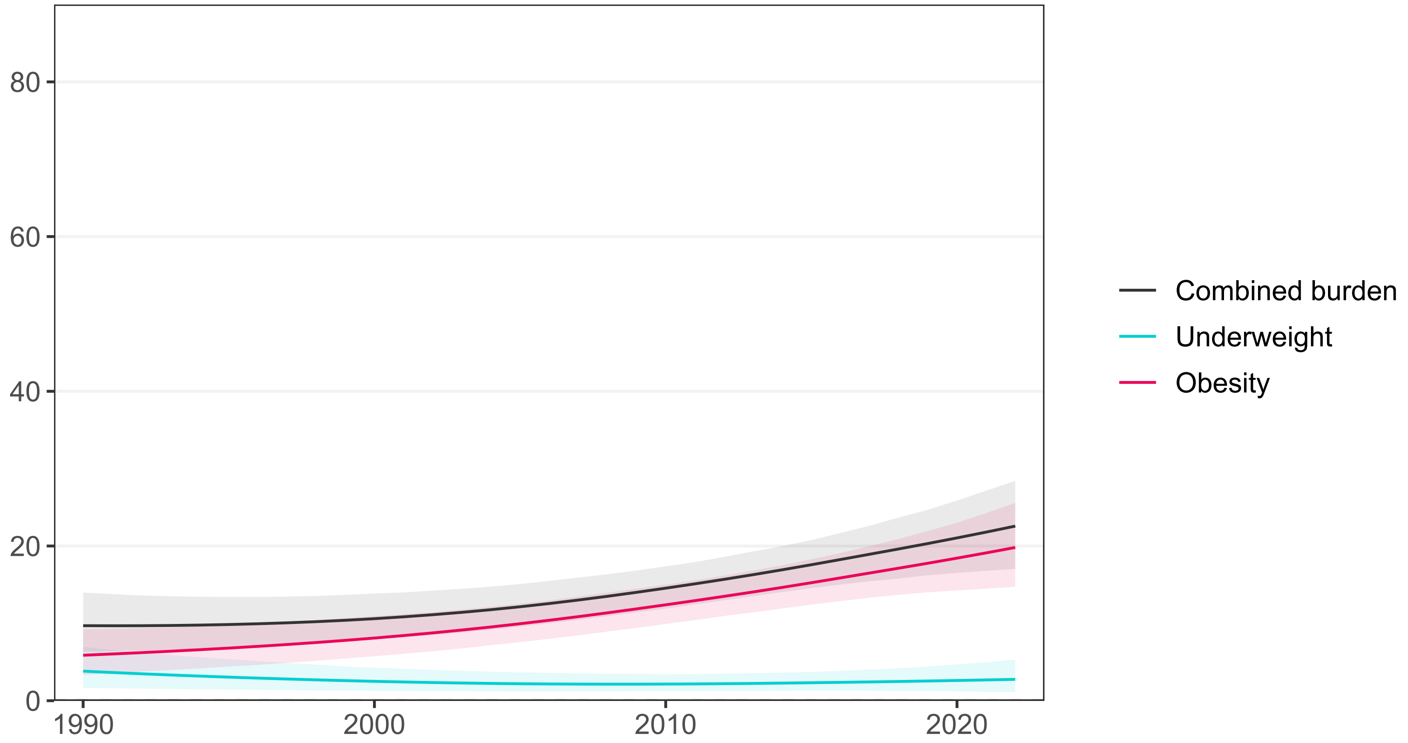

# Australia

## School-aged children and adolescents

Girls
28 studies (21 national)

Boys
28 studies (21 national)

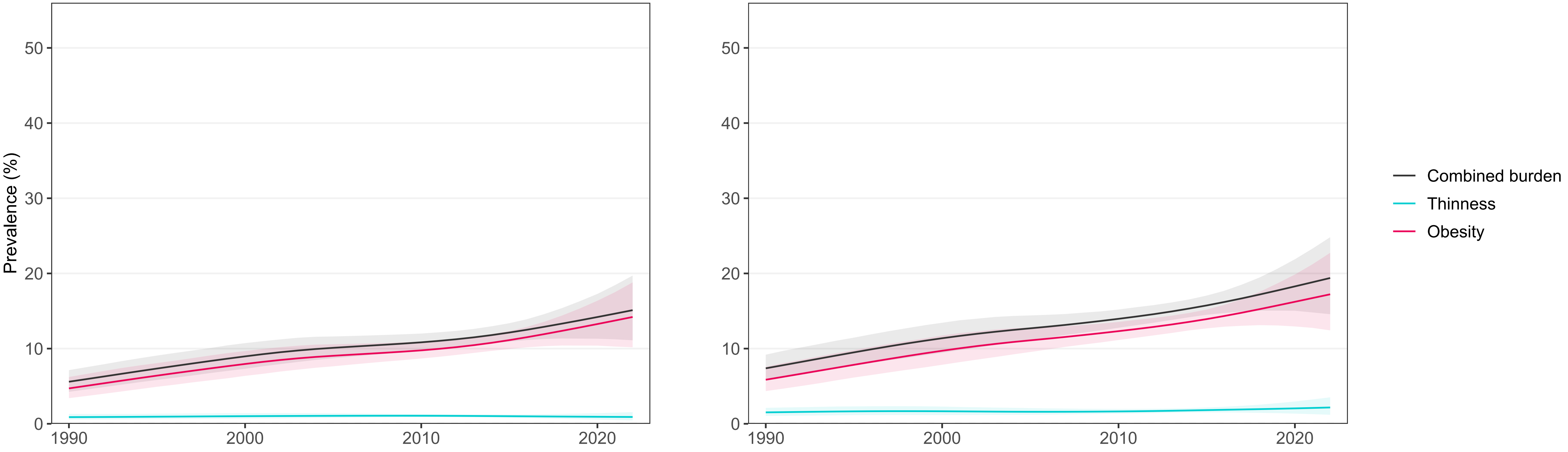


## Adults

Women
26 studies (12 national)

Men
27 studies (12 national)

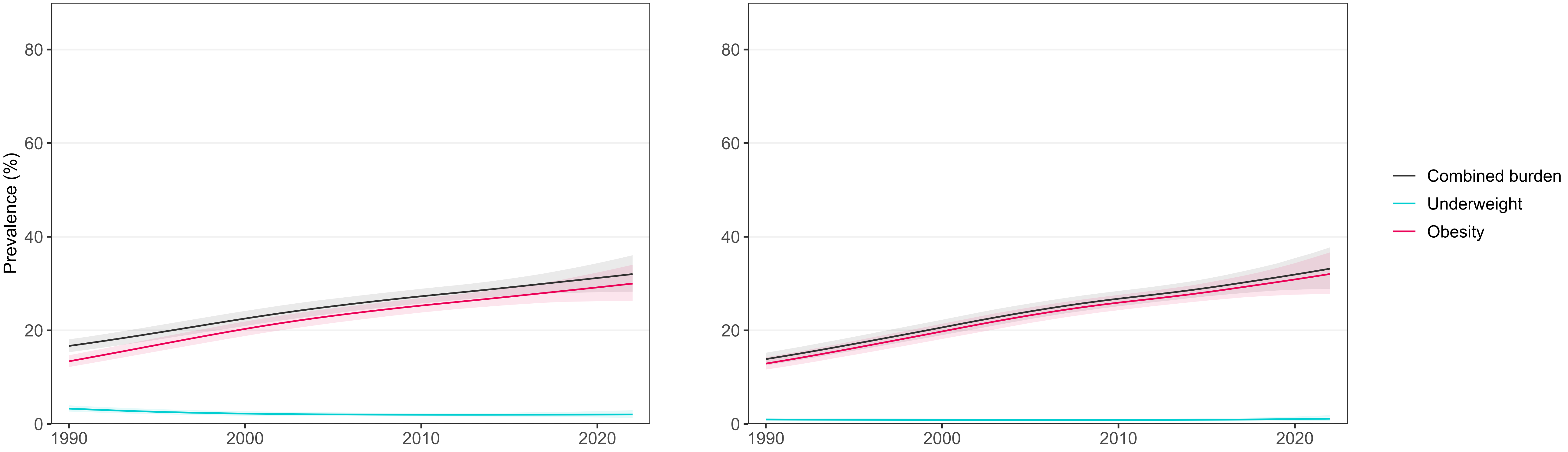

# Austria

## School-aged children and adolescents

Girls
22 studies (5 national)

Boys
59 studies (42 national)

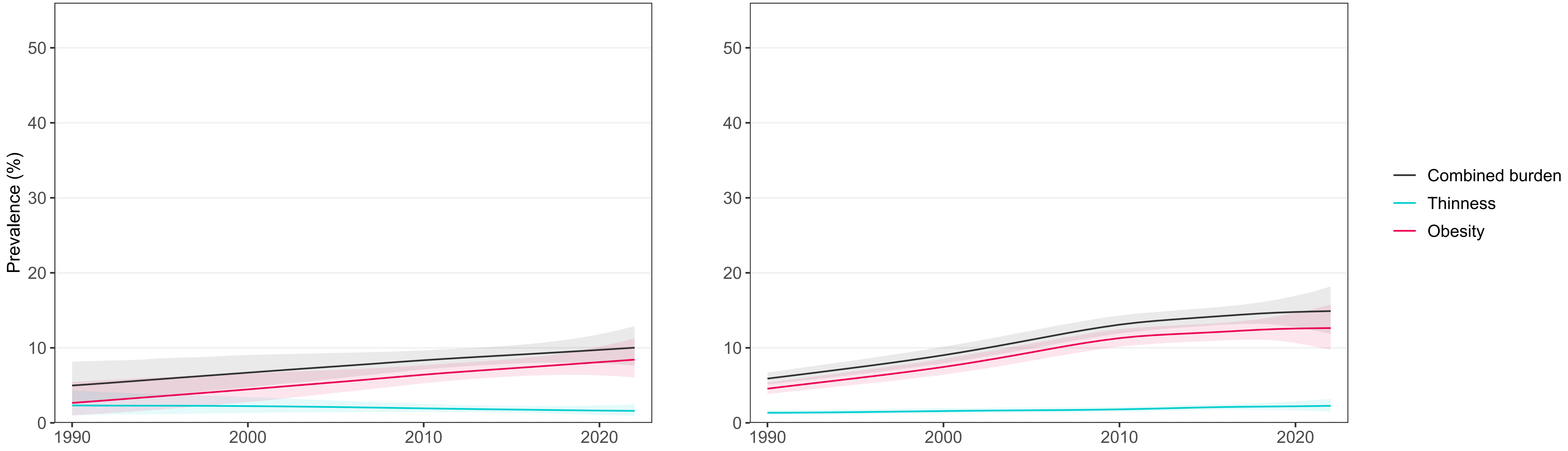


## Adults

Women
8 studies (2 national)

Men
45 studies (39 national)

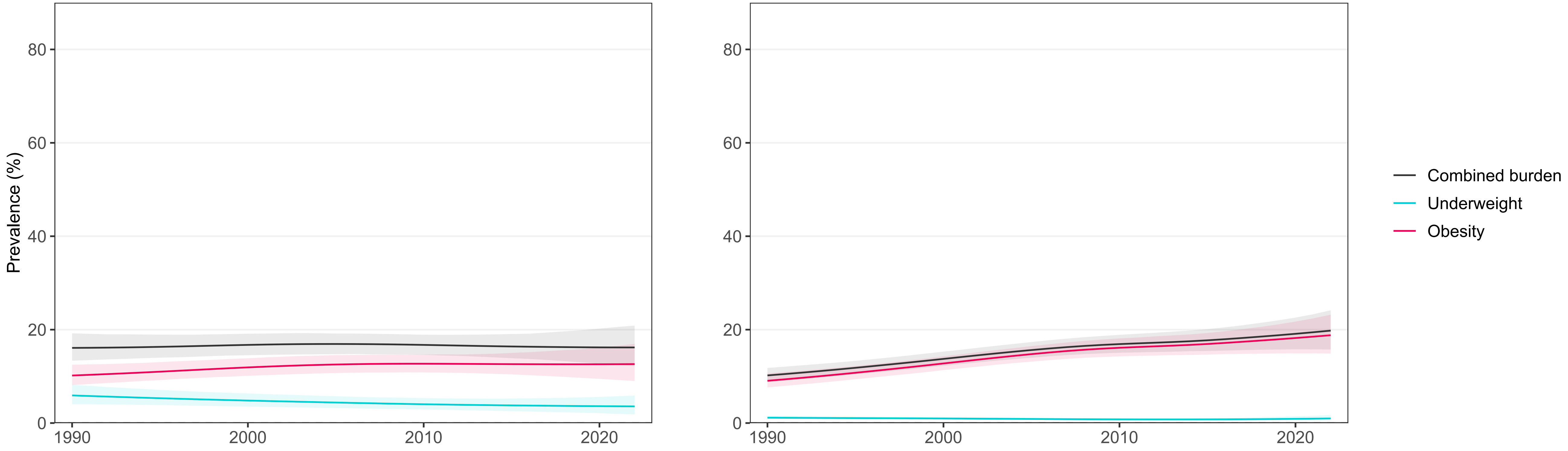

# Azerbaijan

## School-aged children and adolescents

### Girls
4 studies (4 national)

### Boys
2 studies (2 national)

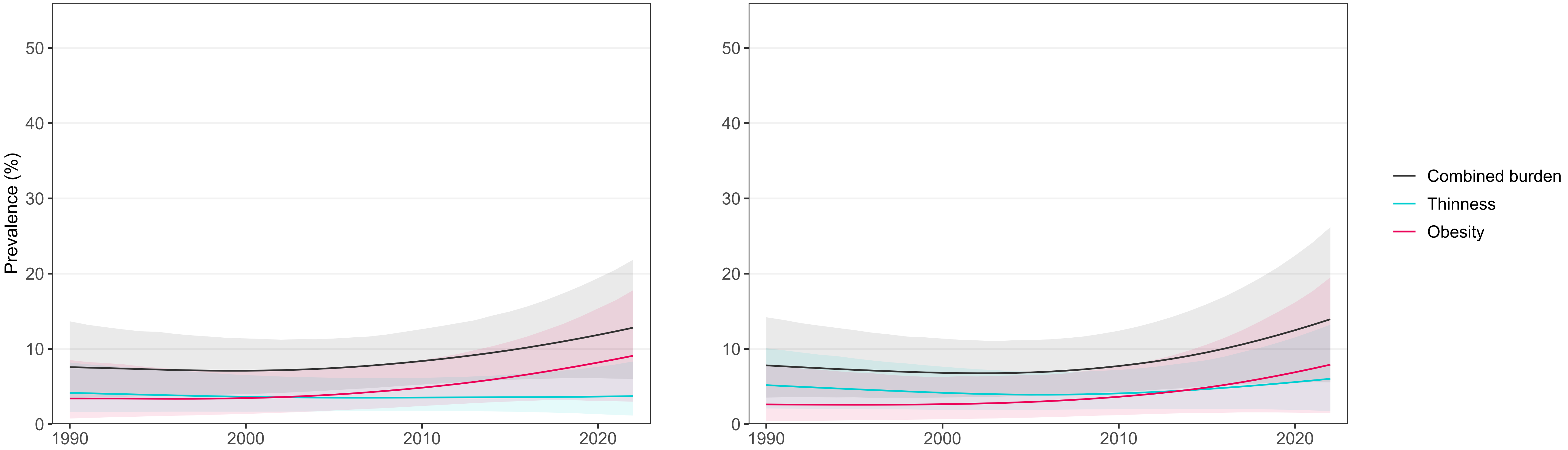


## Adults

### Women
5 studies (5 national)

### Men
3 studies (3 national)

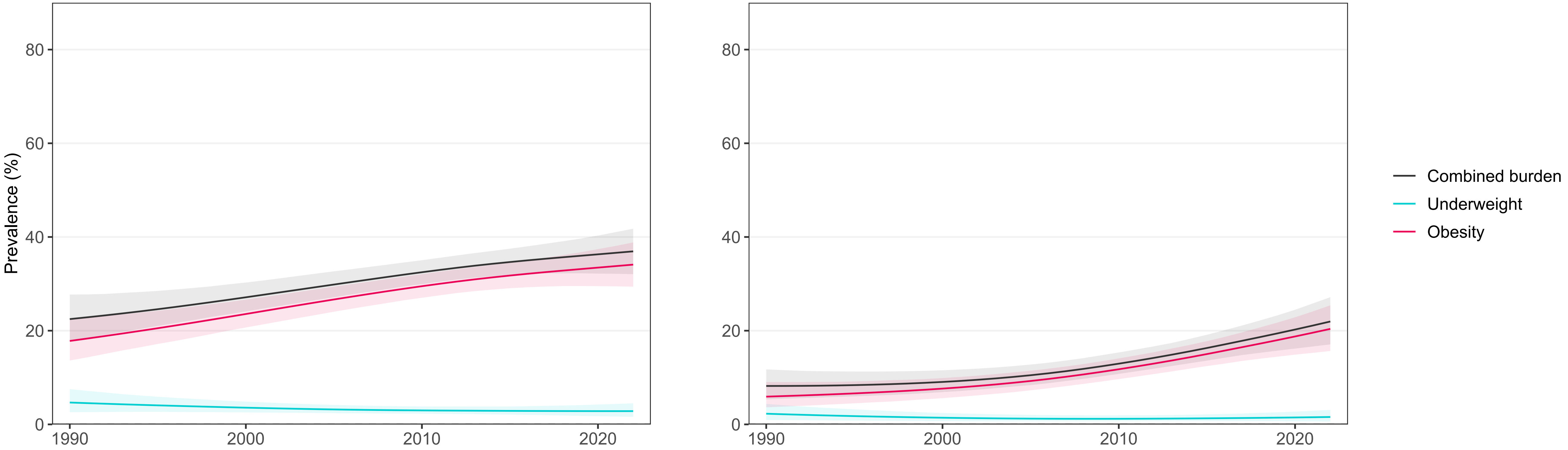

# Bahamas

## School-aged children and adolescents

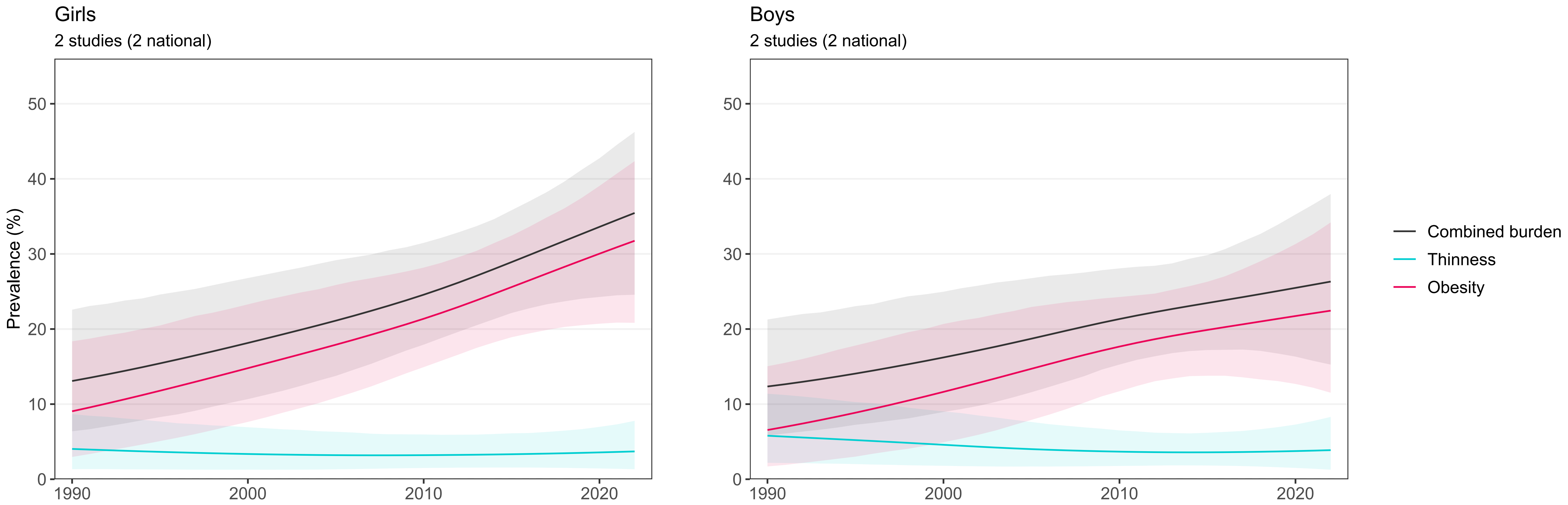


## Adults

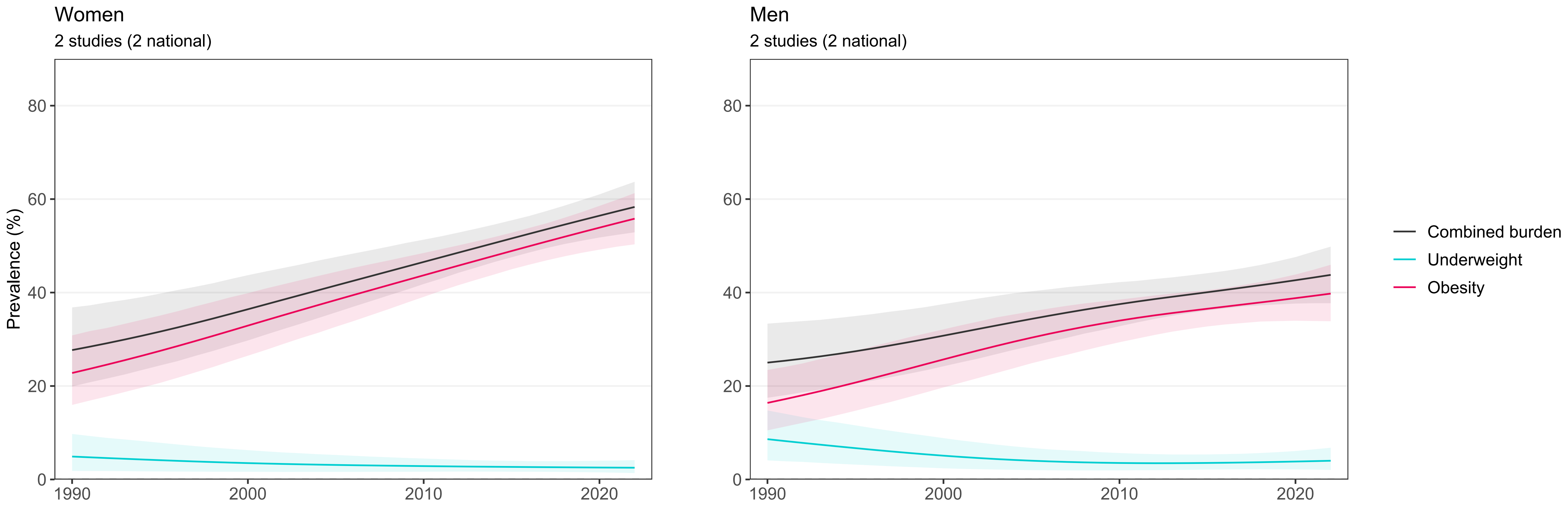

# Bahrain

## School-aged children and adolescents

### Girls

2 studies (2 national)

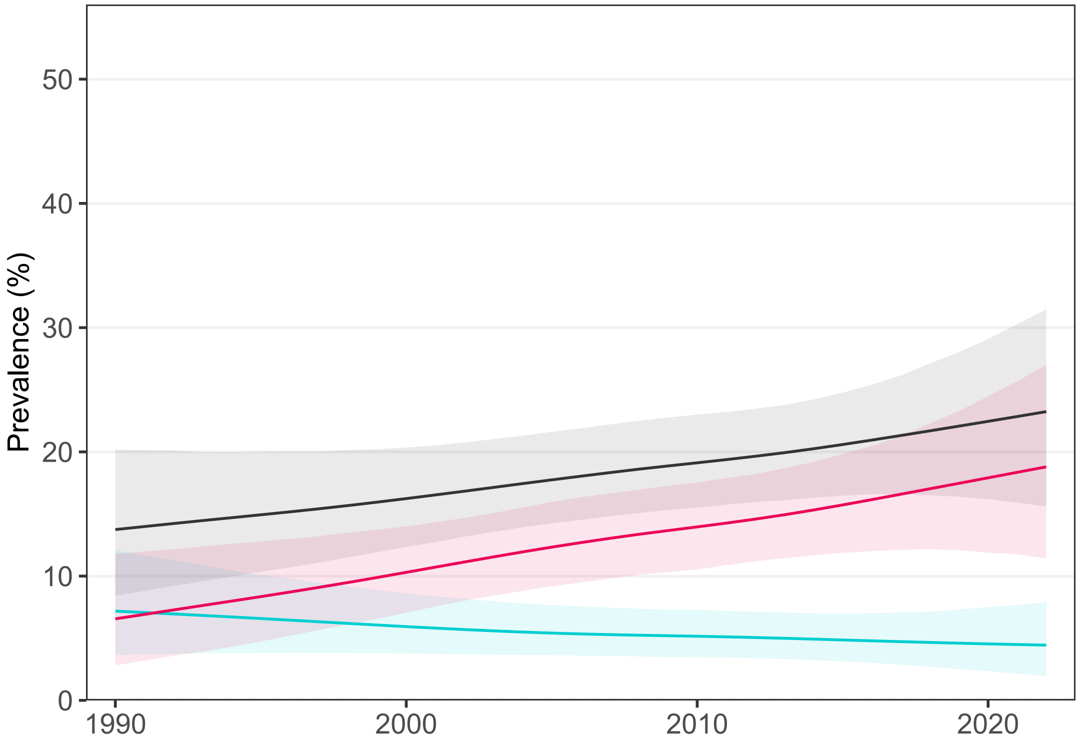


### Boys

2 studies (2 national)

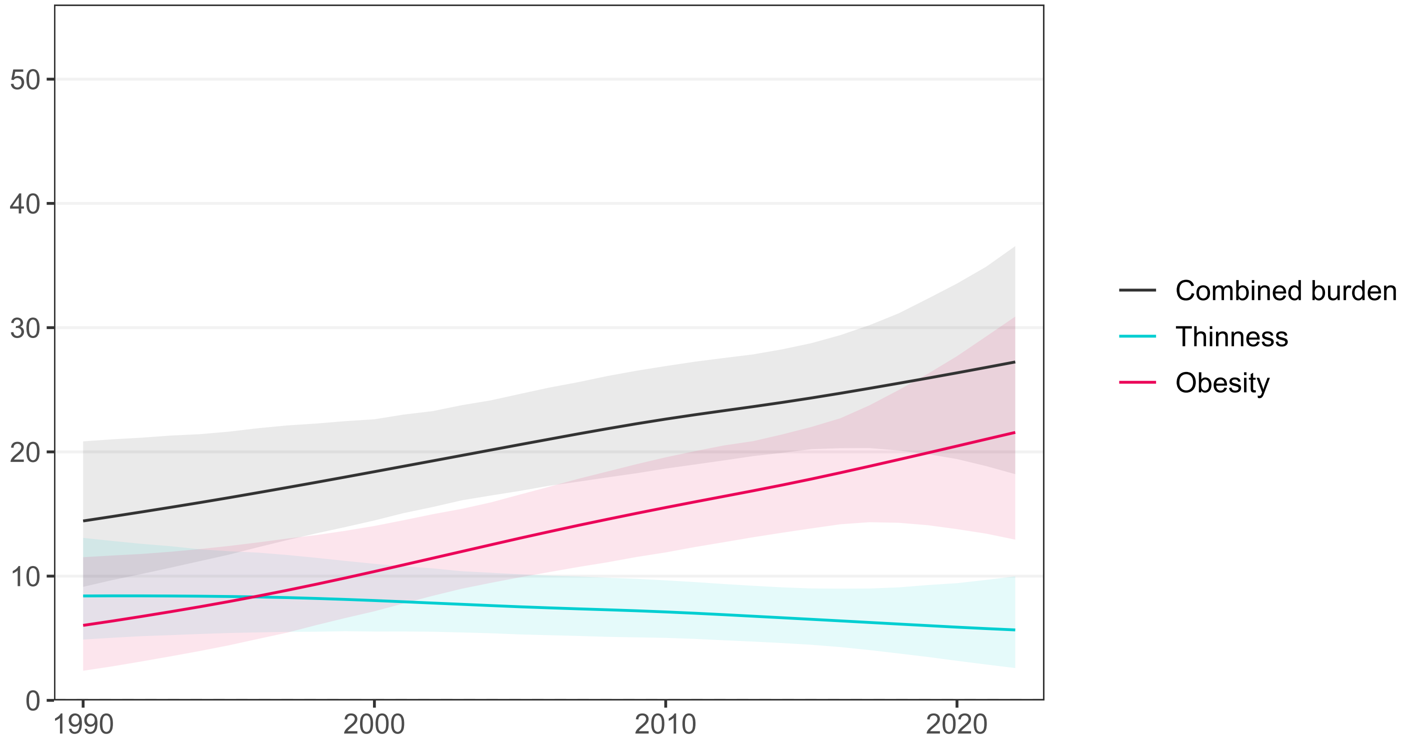


## Adults

### Women

5 studies (3 national)

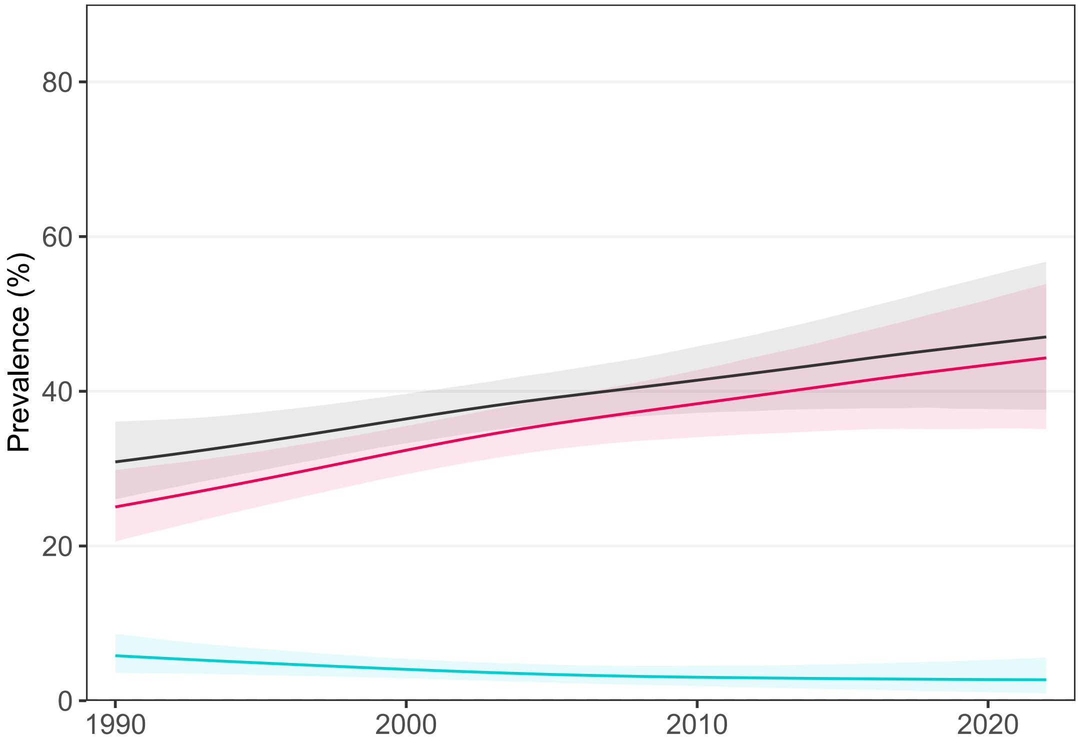


### Men

5 studies (3 national)

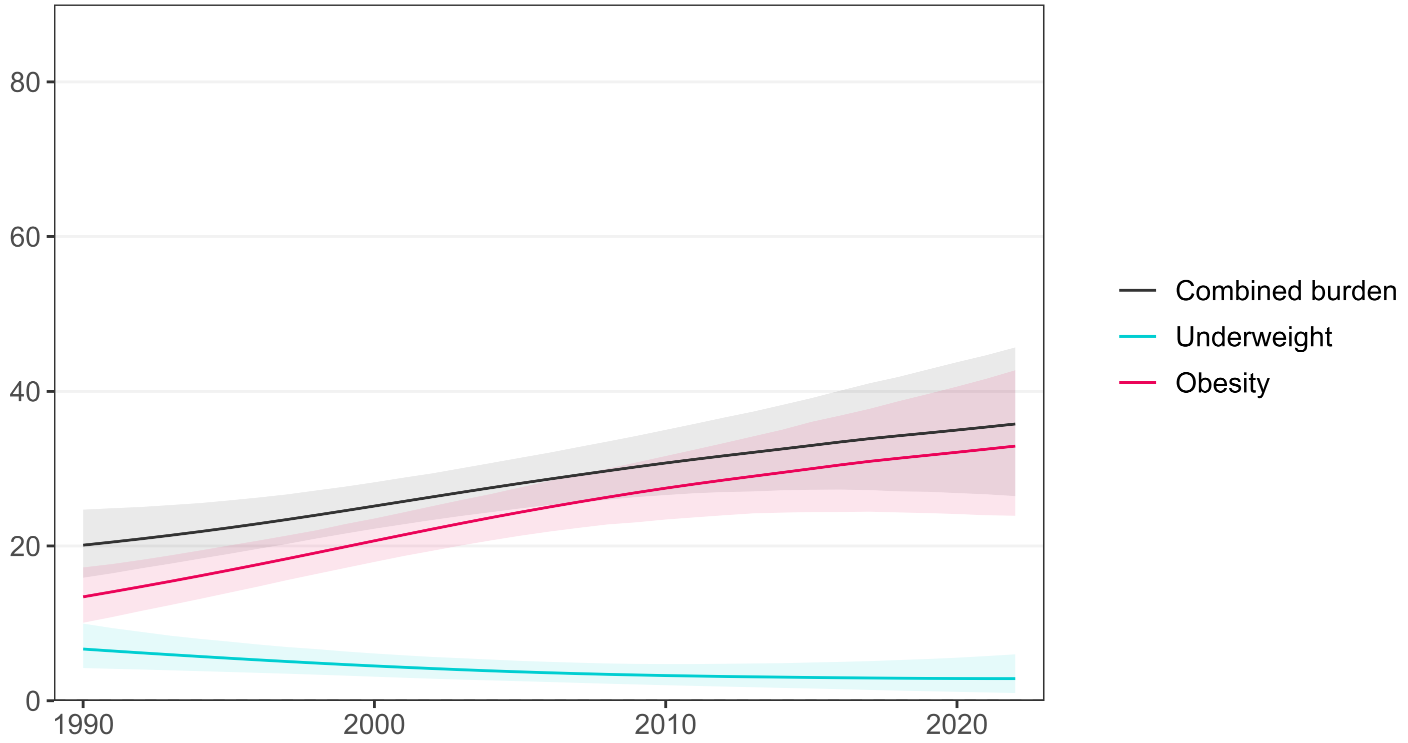

# Bangladesh

## School-aged children and adolescents

### Girls

4 studies (3 national)

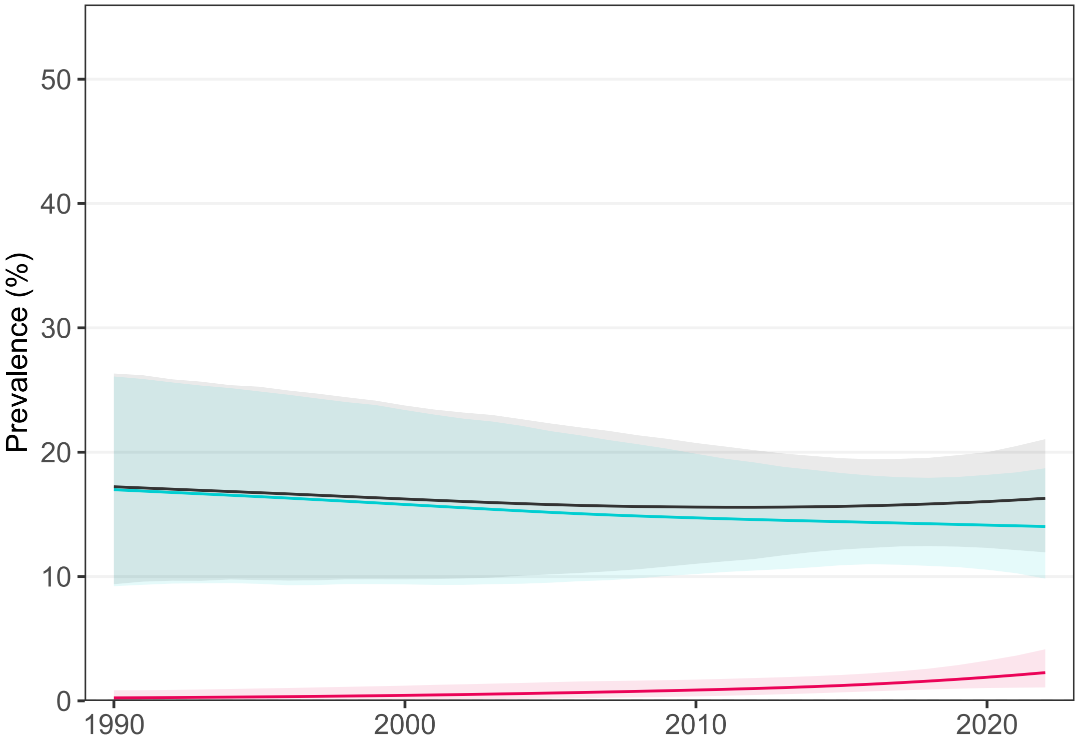


### Boys

4 studies (3 national)

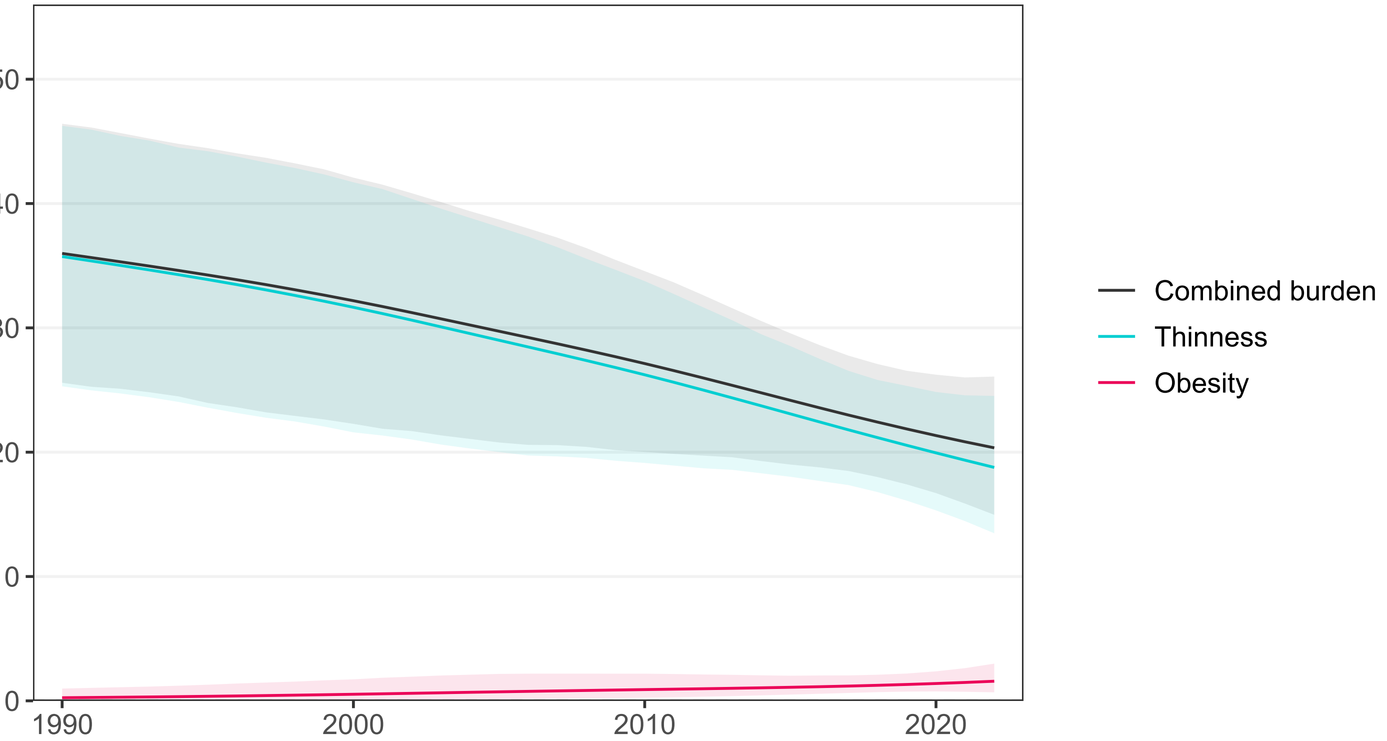


## Adults

### Women

21 studies (13 national)

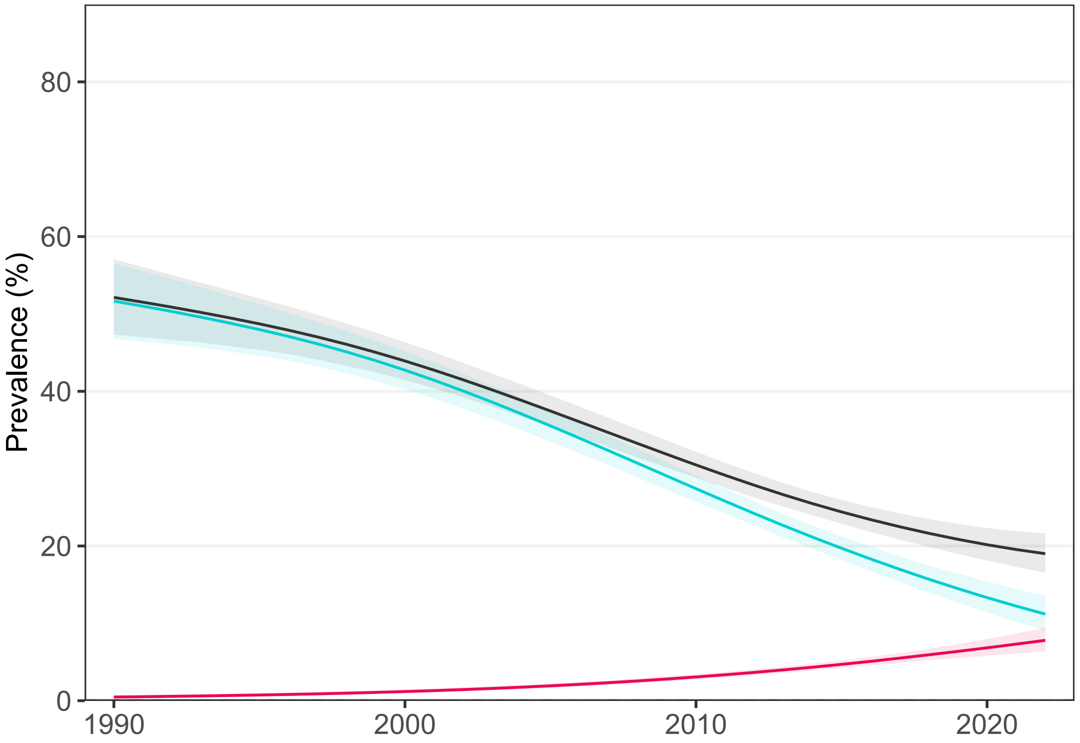


### Men

14 studies (6 national)

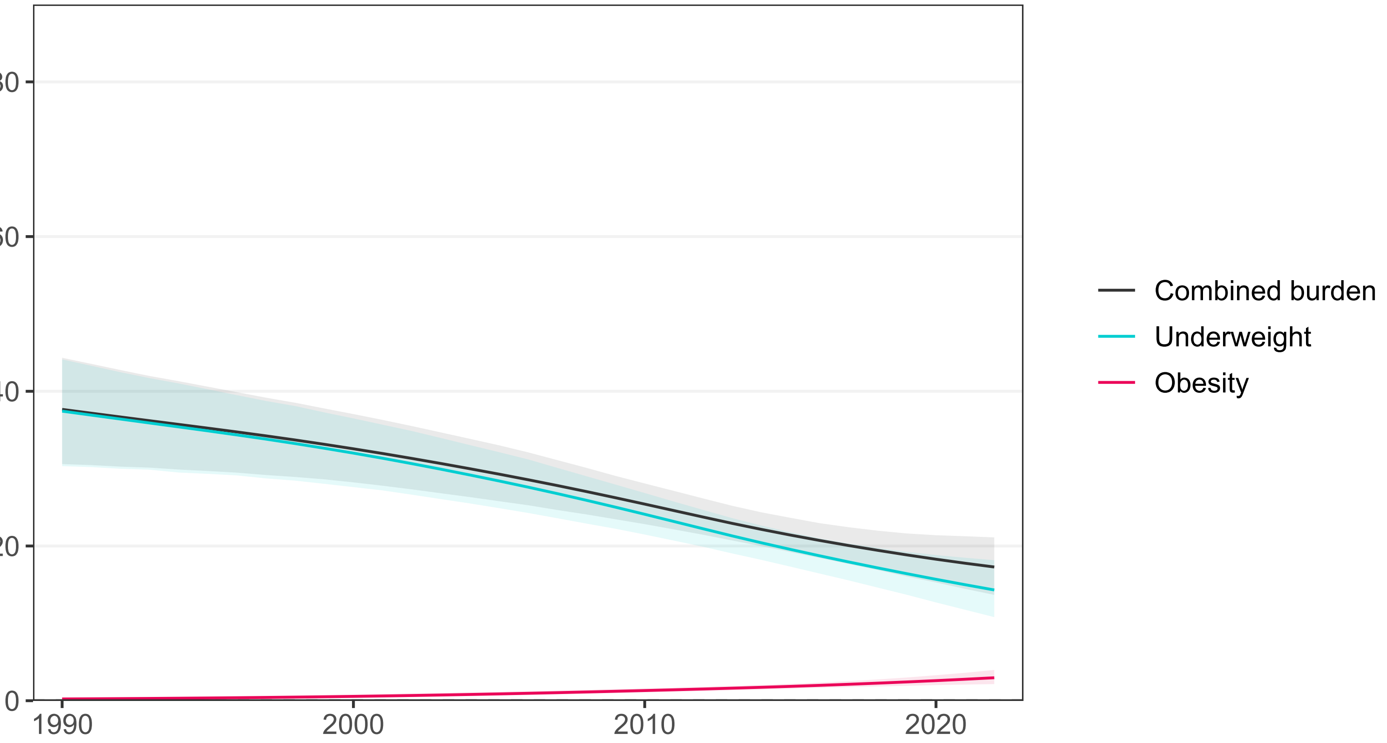

# Barbados

## School-aged children and adolescents

### Girls

1 study (1 national)

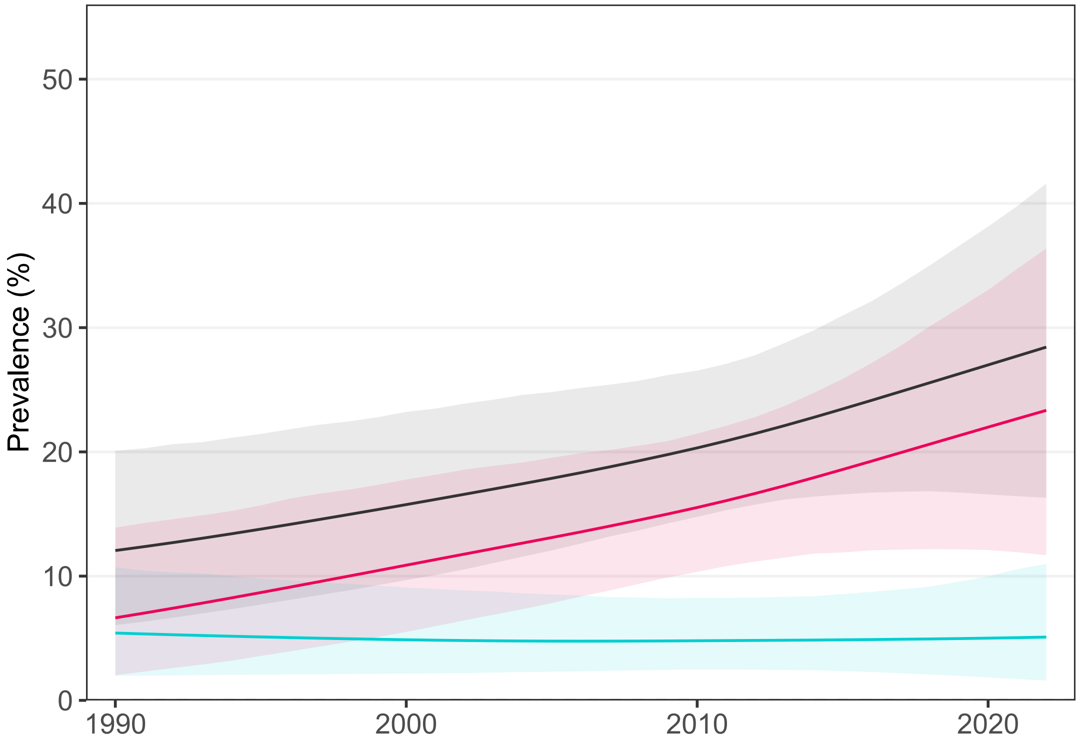


### Boys

1 study (1 national)

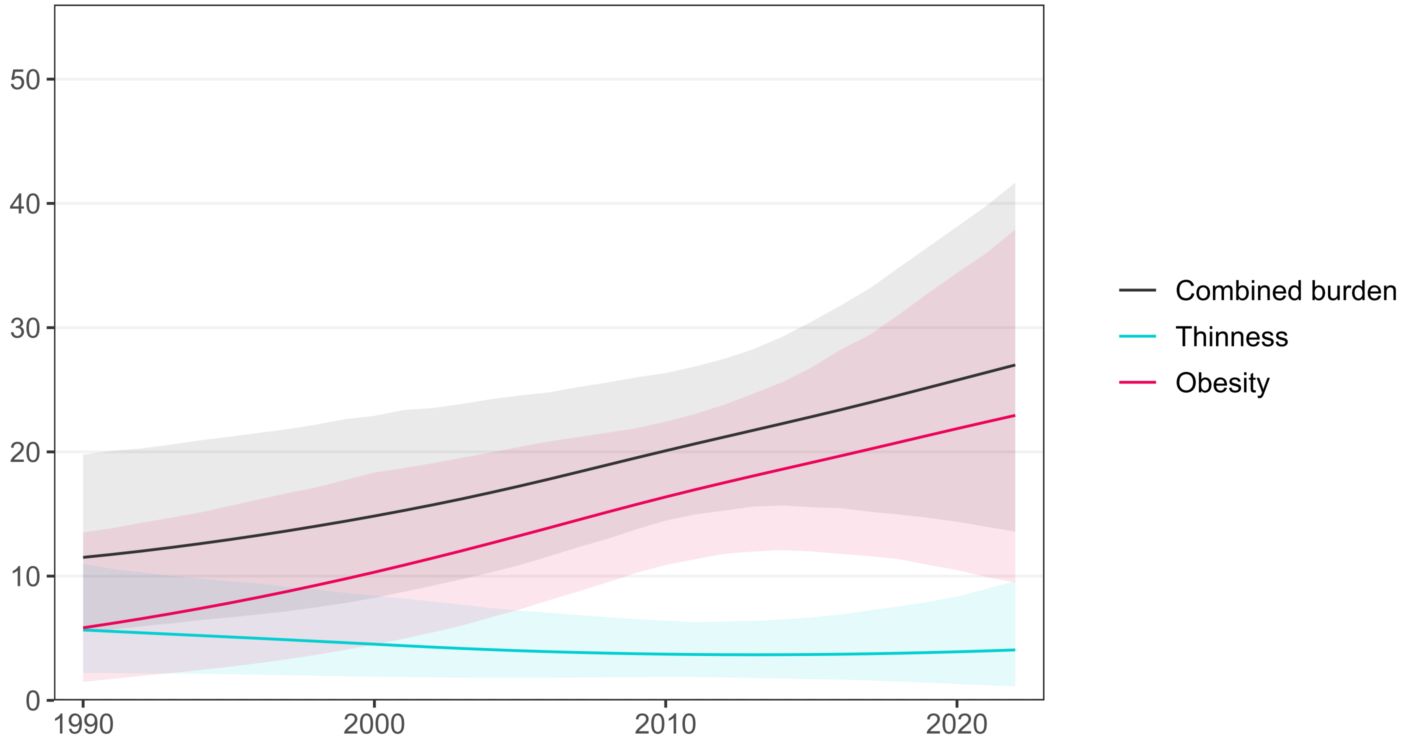


## Adults

### Women

5 studies (3 national)

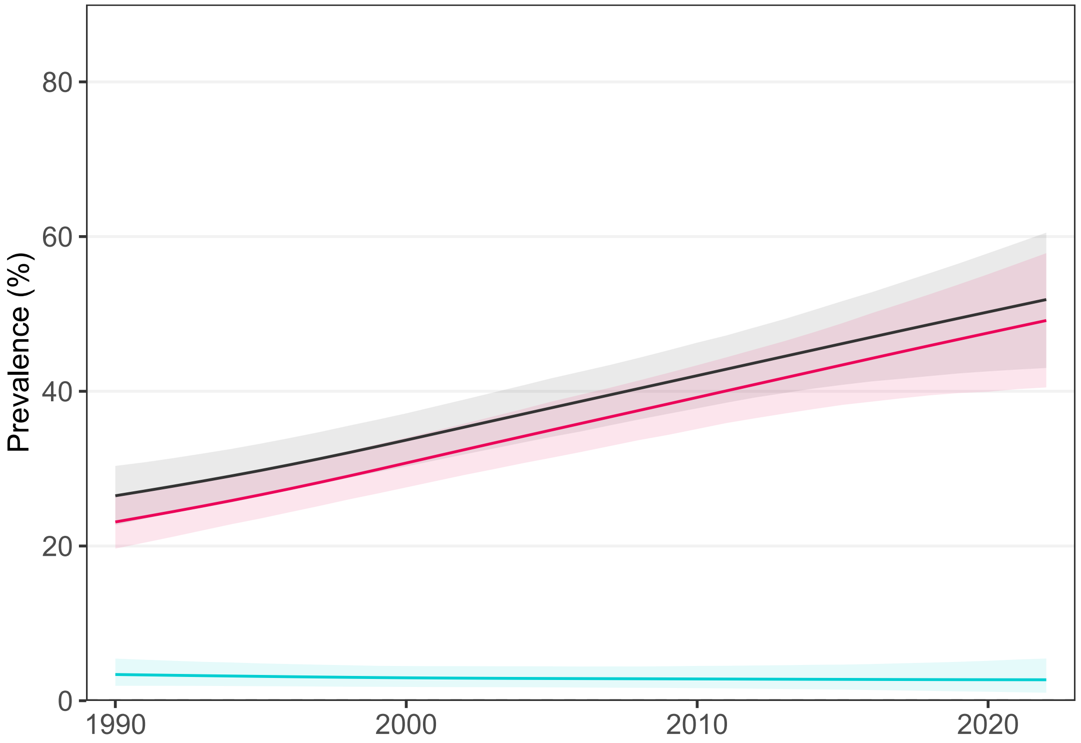


### Men

5 studies (3 national)

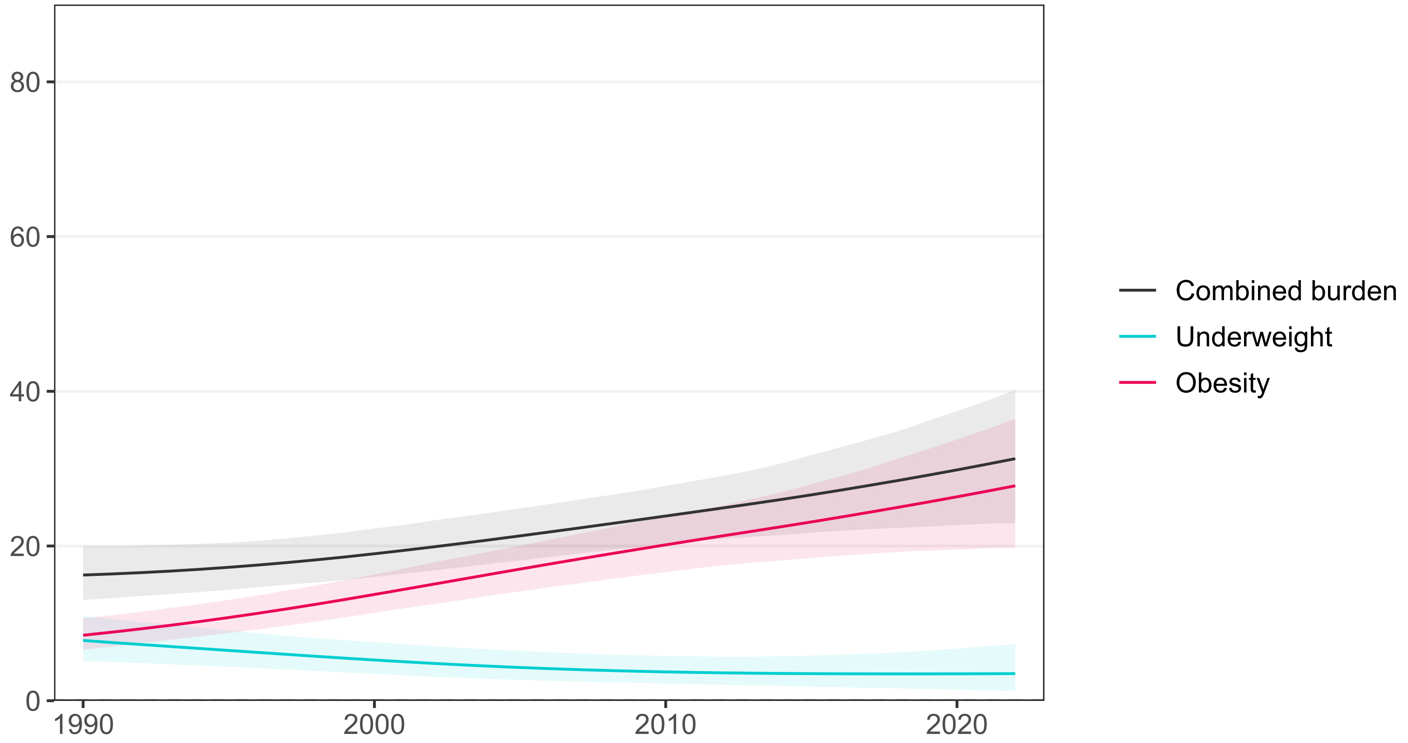

# Belarus

## School-aged children and adolescents

### Girls
2 studies (2 national)

### Boys
2 studies (2 national)

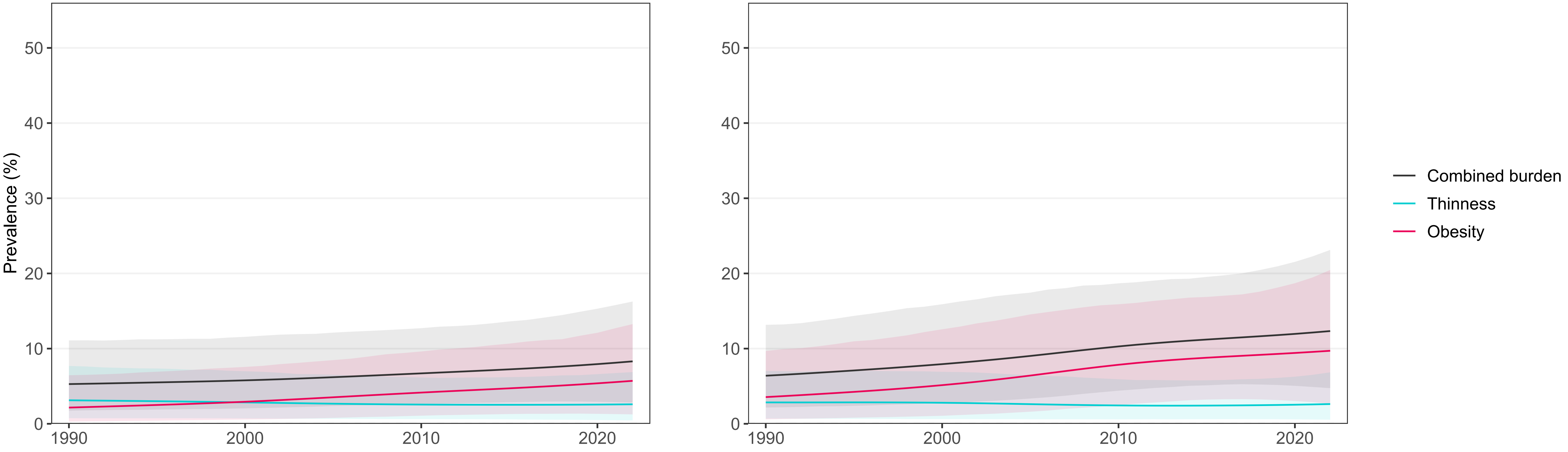


## Adults

### Women
2 studies (2 national)

### Men
2 studies (2 national)

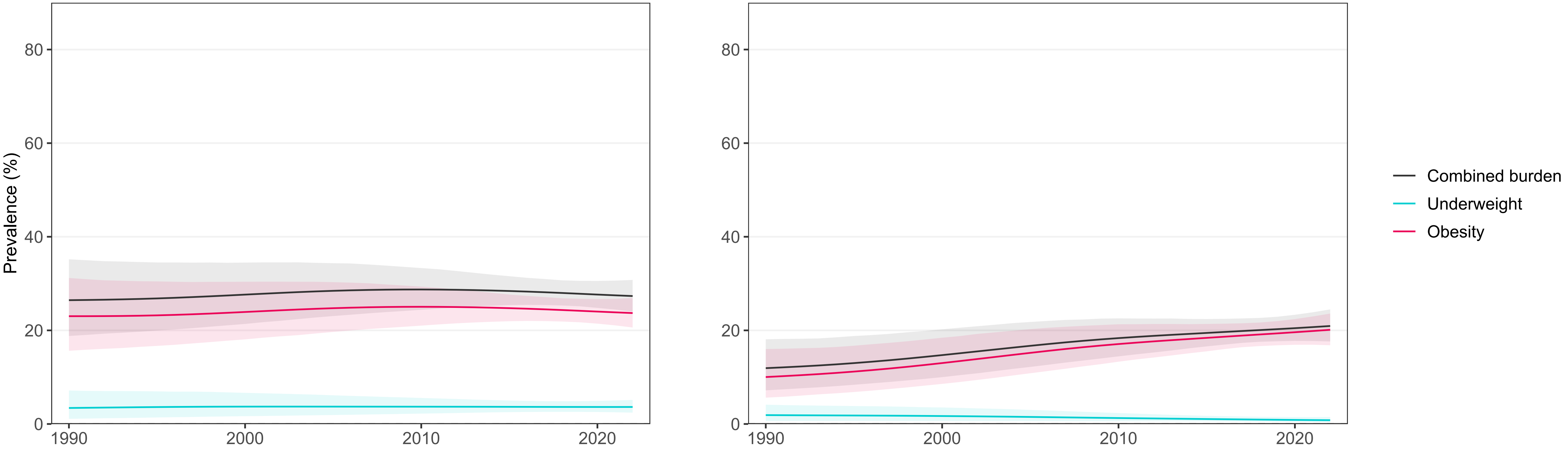

# Belgium

## School-aged children and adolescents

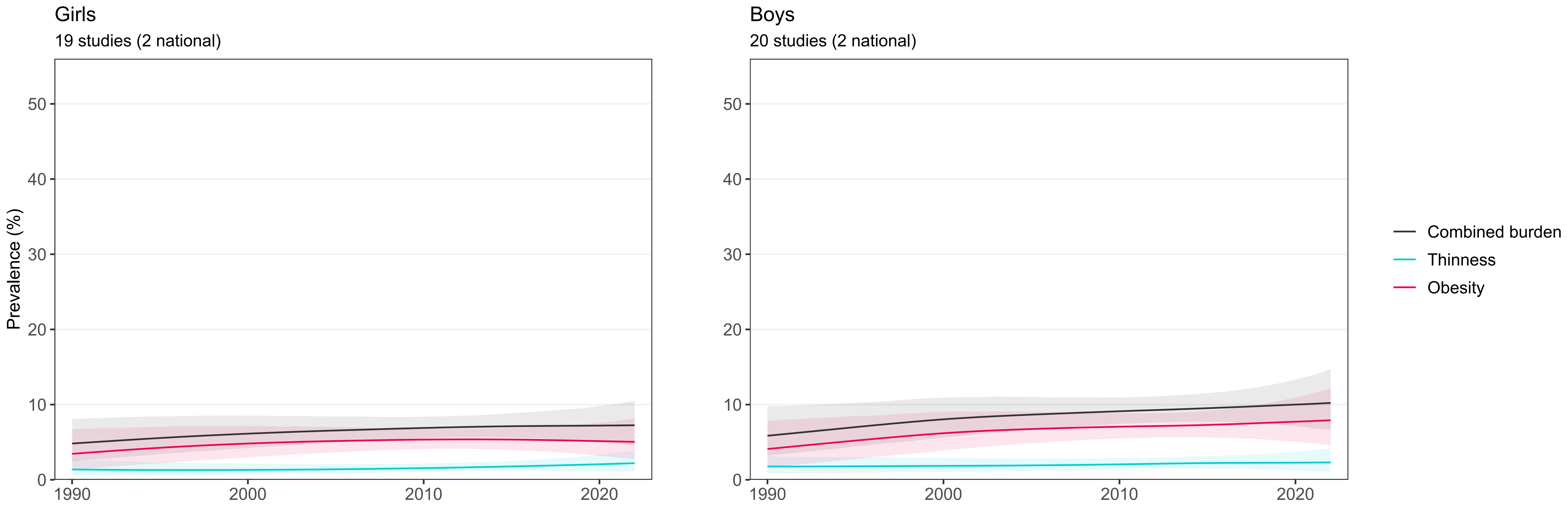


## Adults

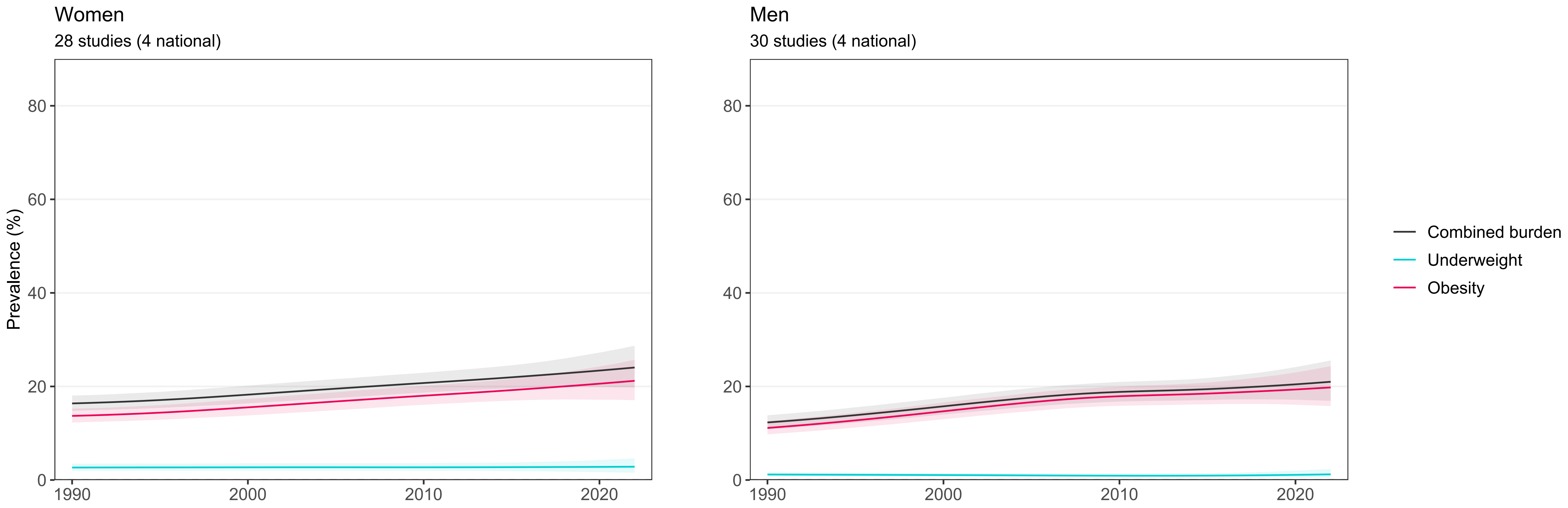

# Belize

## School-aged children and adolescents

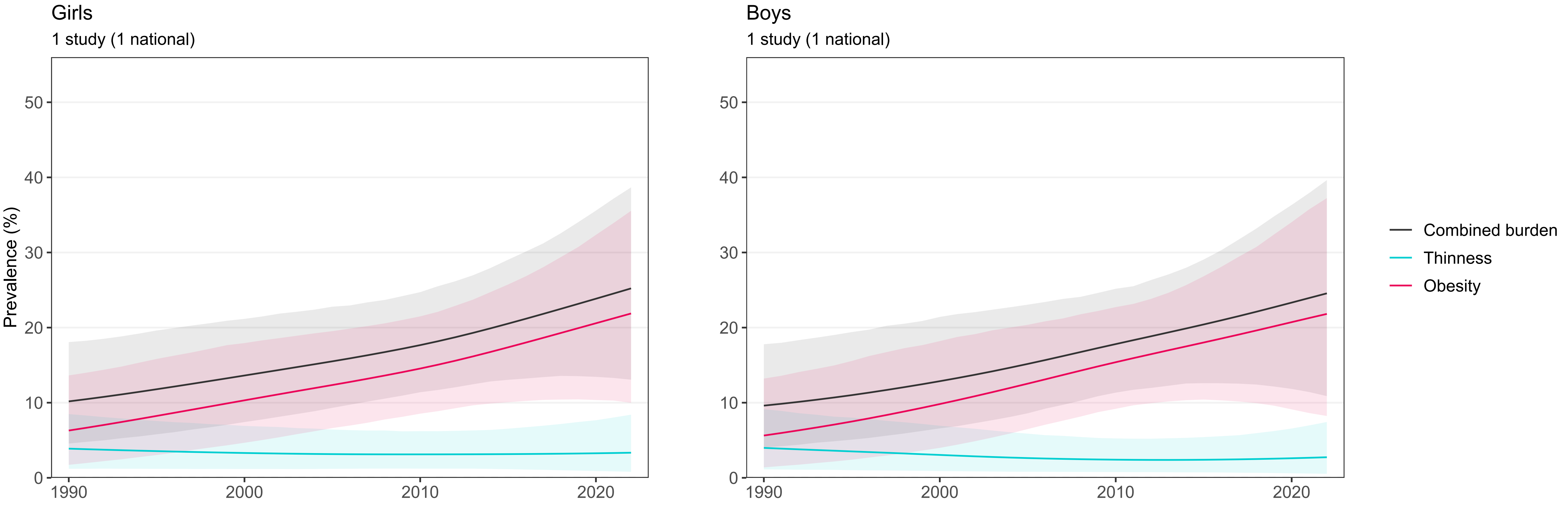


## Adults

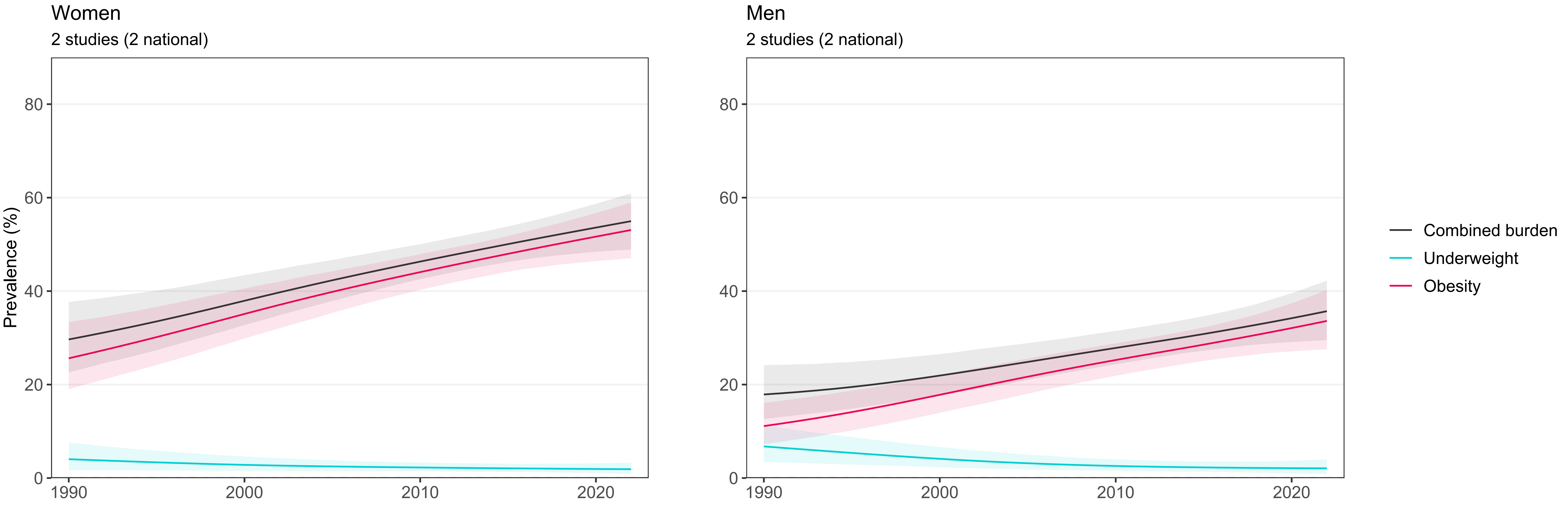

# Benin

## School-aged children and adolescents

Girls
5 studies (5 national)

Boys
1 study (1 national)

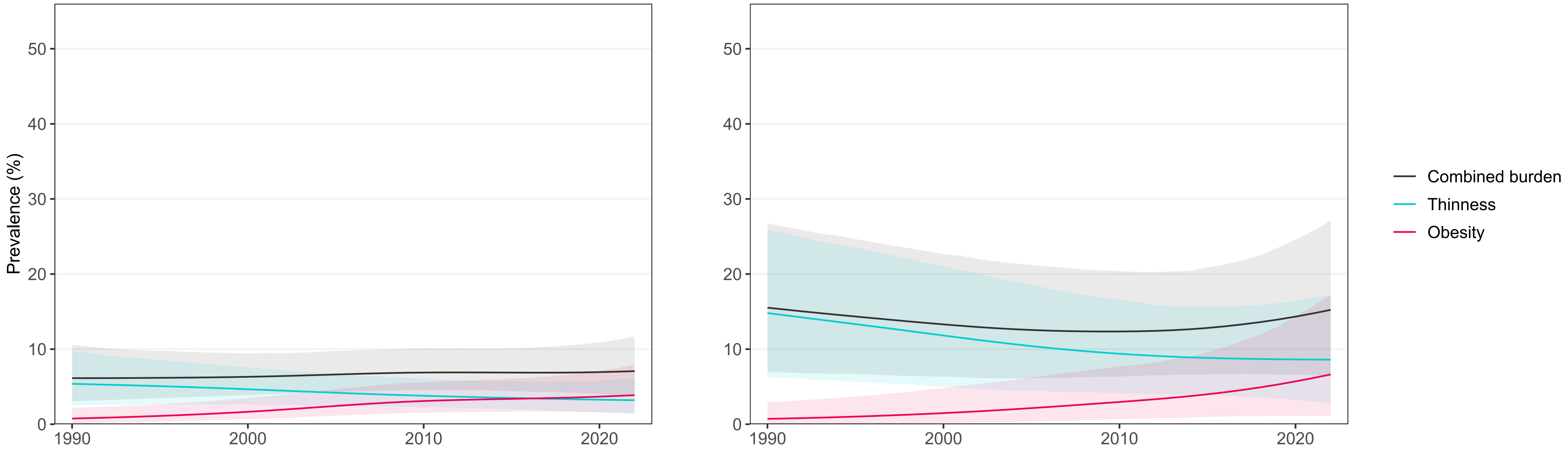


## Adults

Women
8 studies (7 national)

Men
3 studies (2 national)

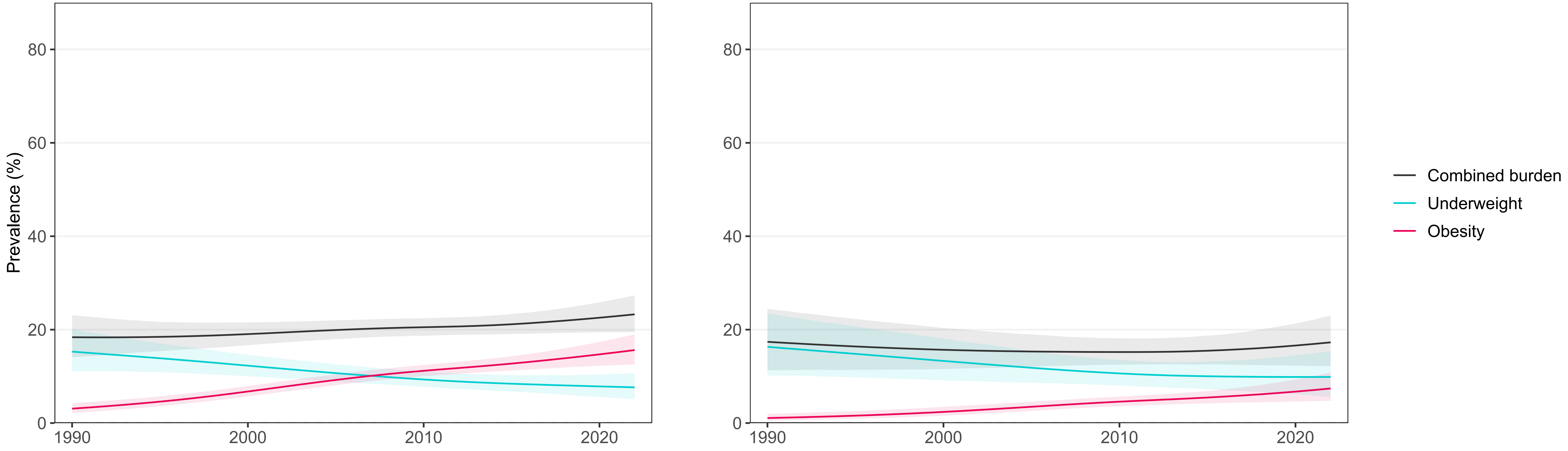

# Bermuda

## School-aged children and adolescents

### Girls

No studies

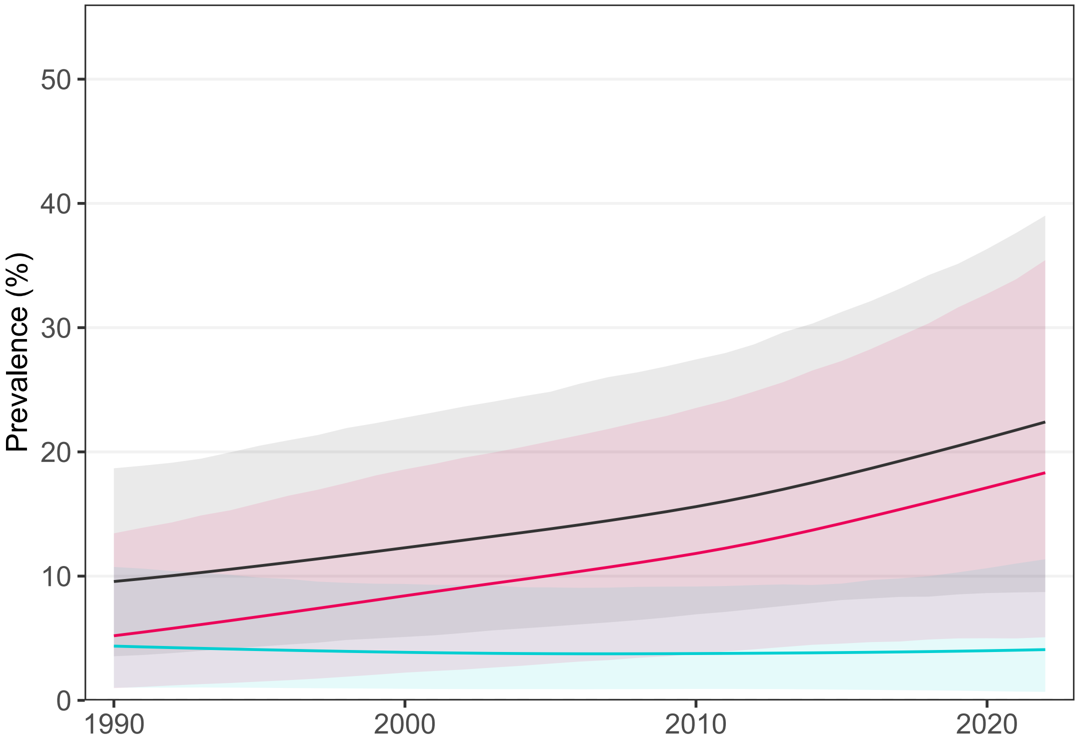


### Boys

No studies

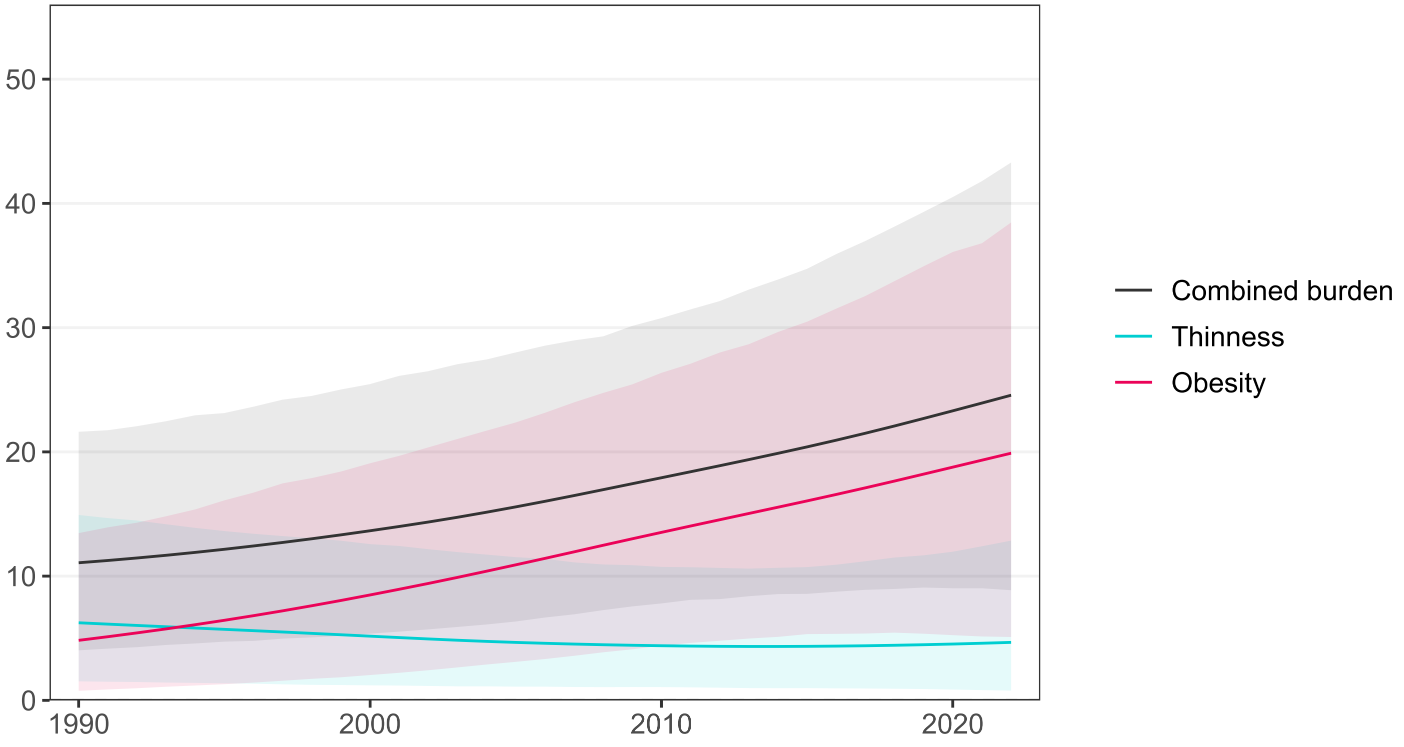


## Adults

### Women

No studies

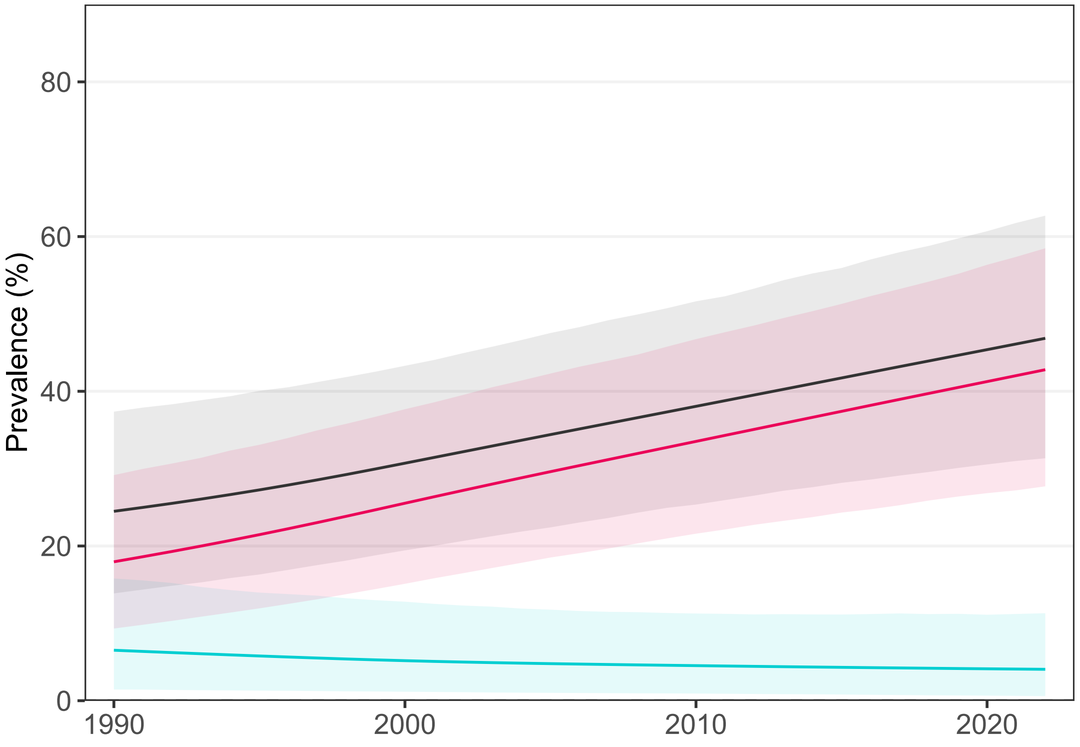


### Men

No studies

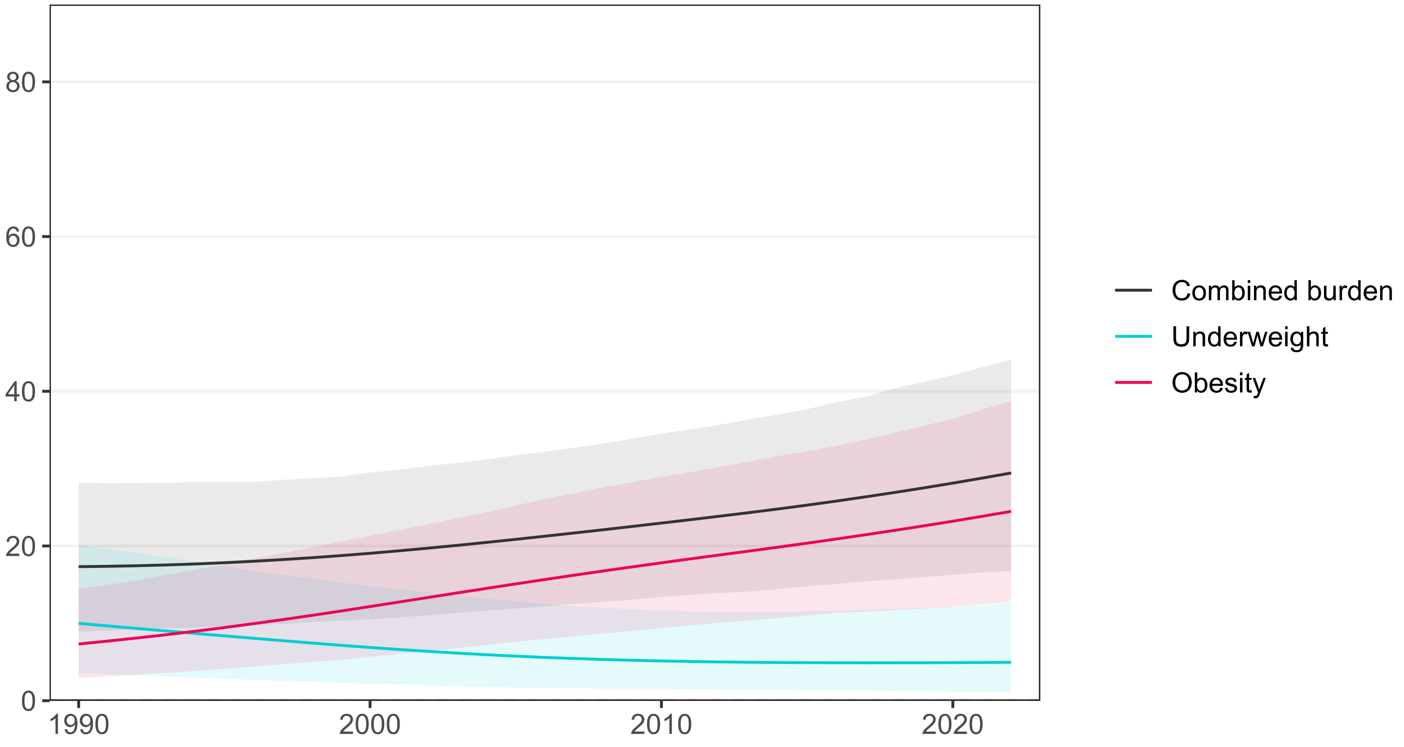

# Bhutan

## School-aged children and adolescents

### Girls

2 studies (2 national)

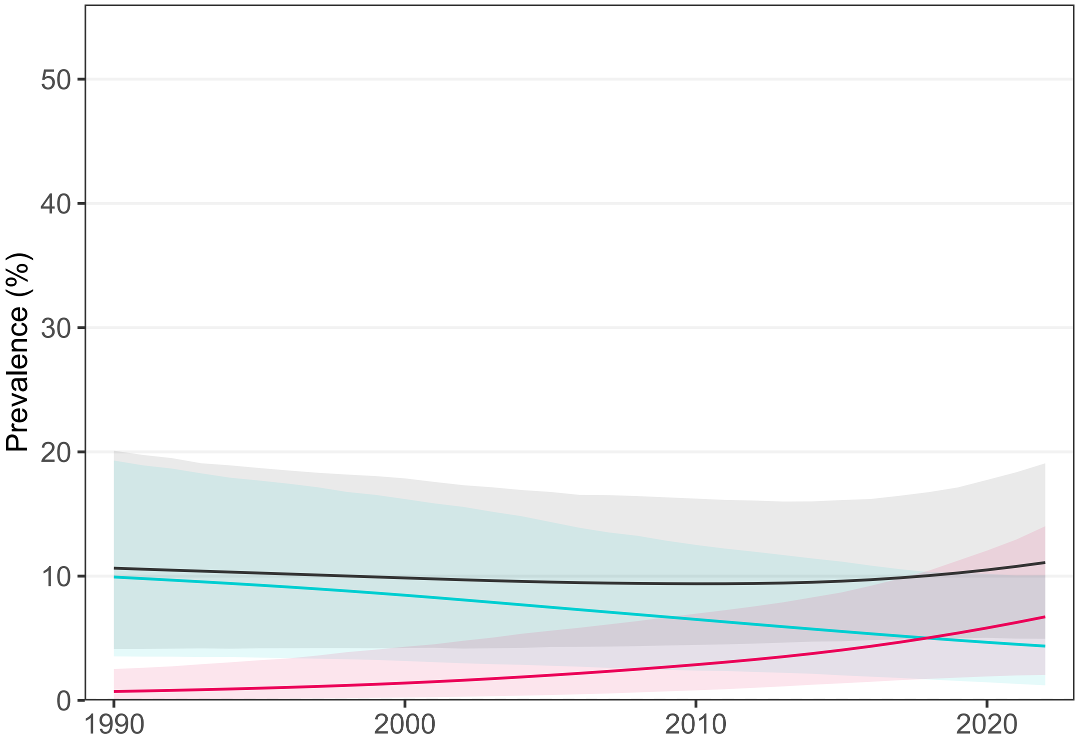


### Boys

2 studies (2 national)

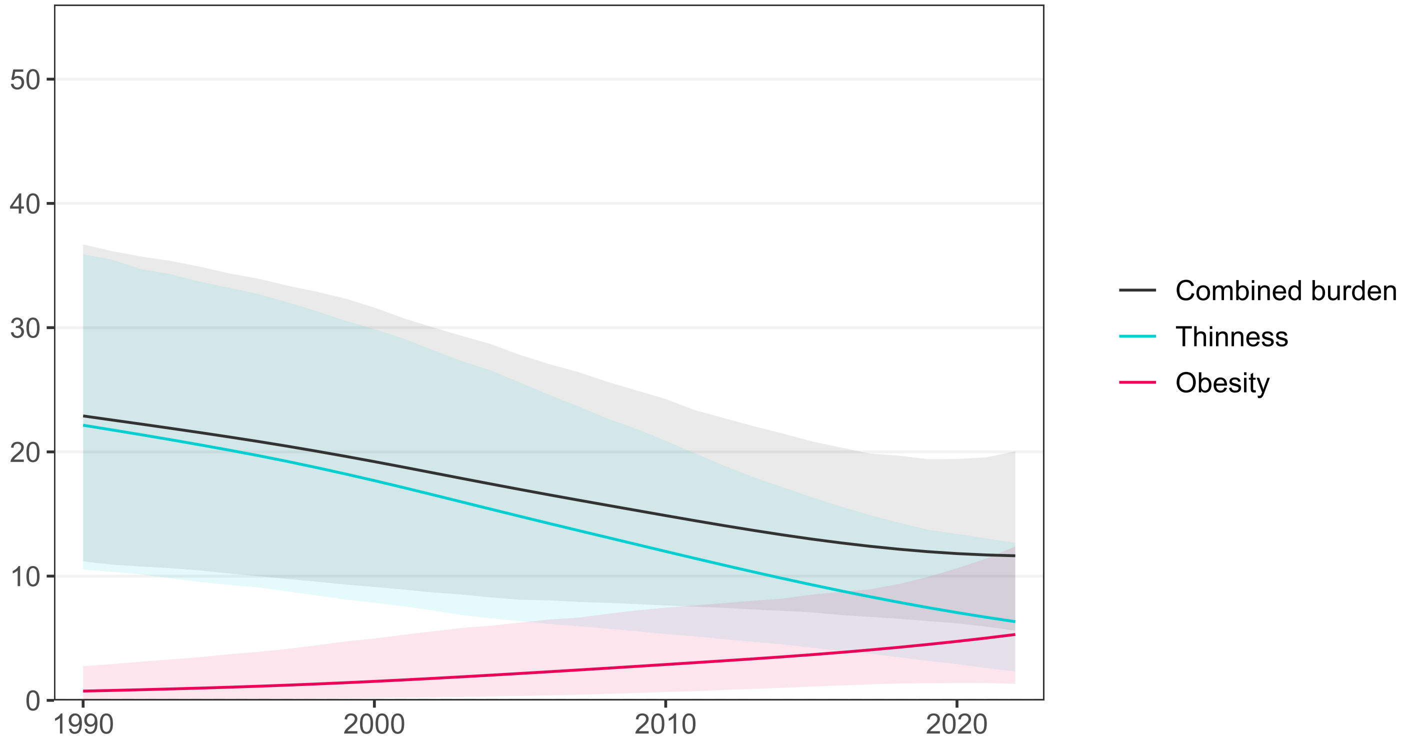


## Adults

### Women

3 studies (2 national)

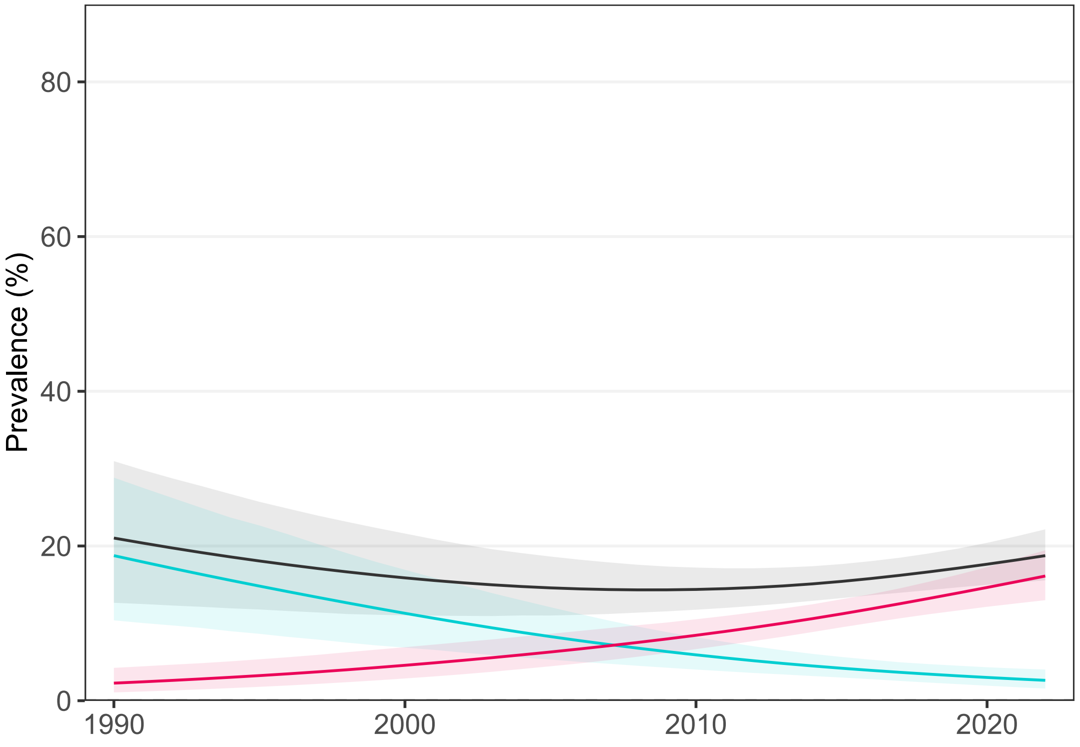


### Men

3 studies (2 national)

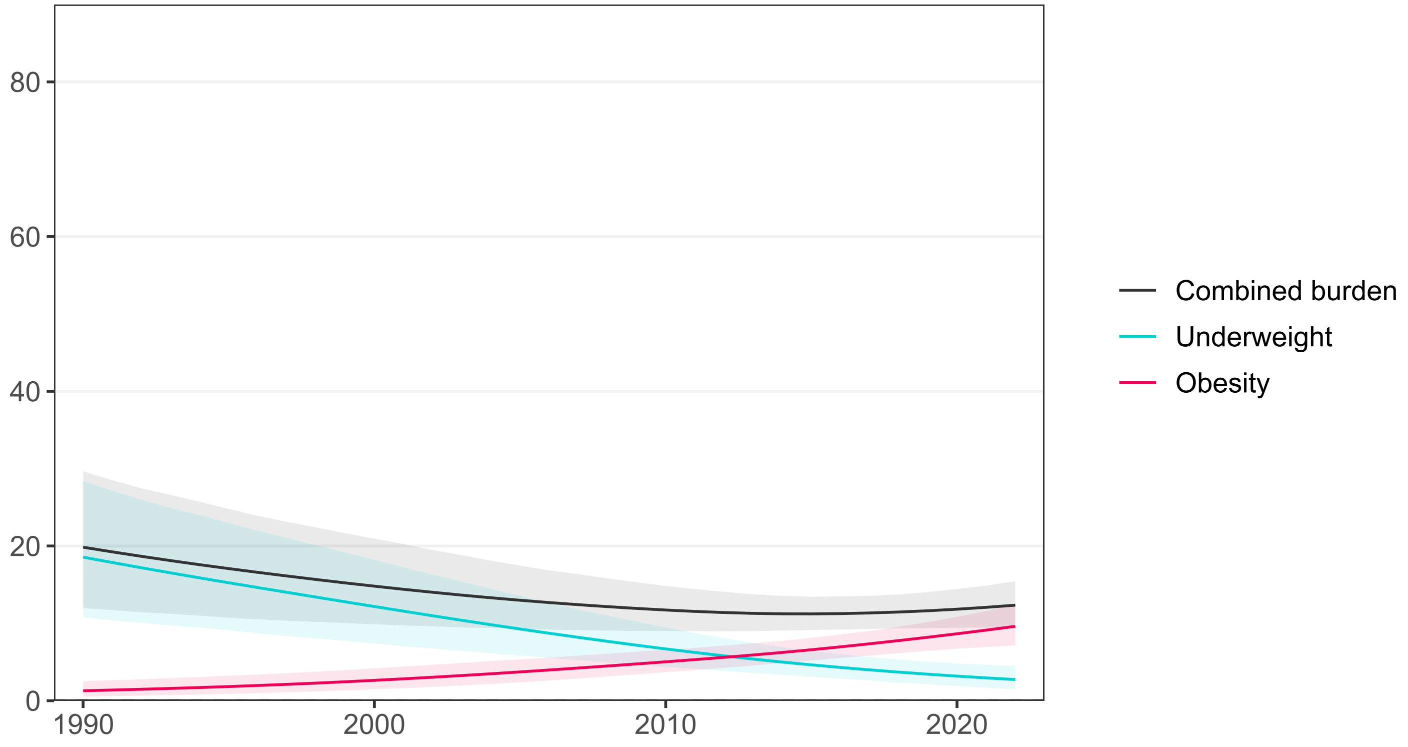

# Bolivia

## School-aged children and adolescents

Girls
4 studies (4 national)

Boys
2 studies (2 national)

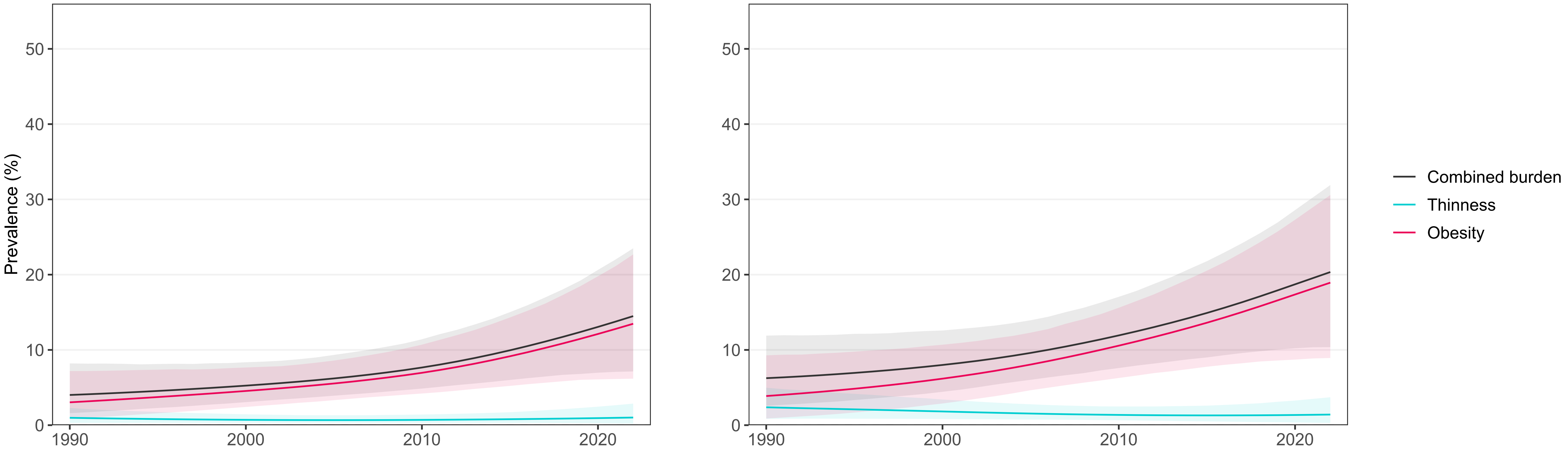


## Adults

Women
6 studies (6 national)

Men
2 studies (2 national)

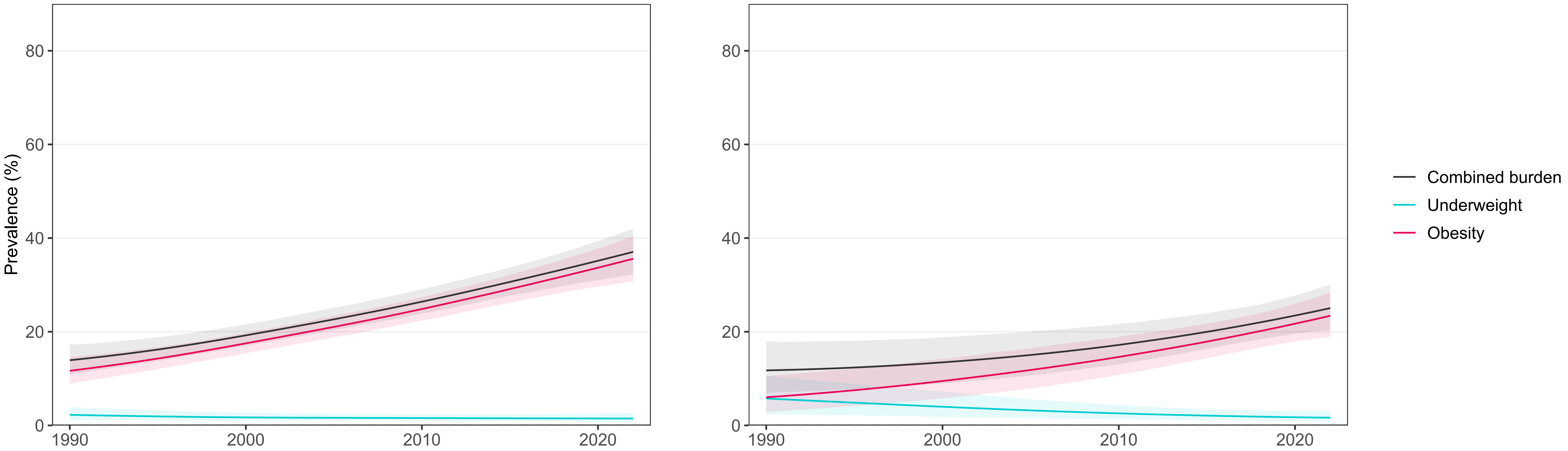

# Bosnia & Herzegovina

## School-aged children and adolescents

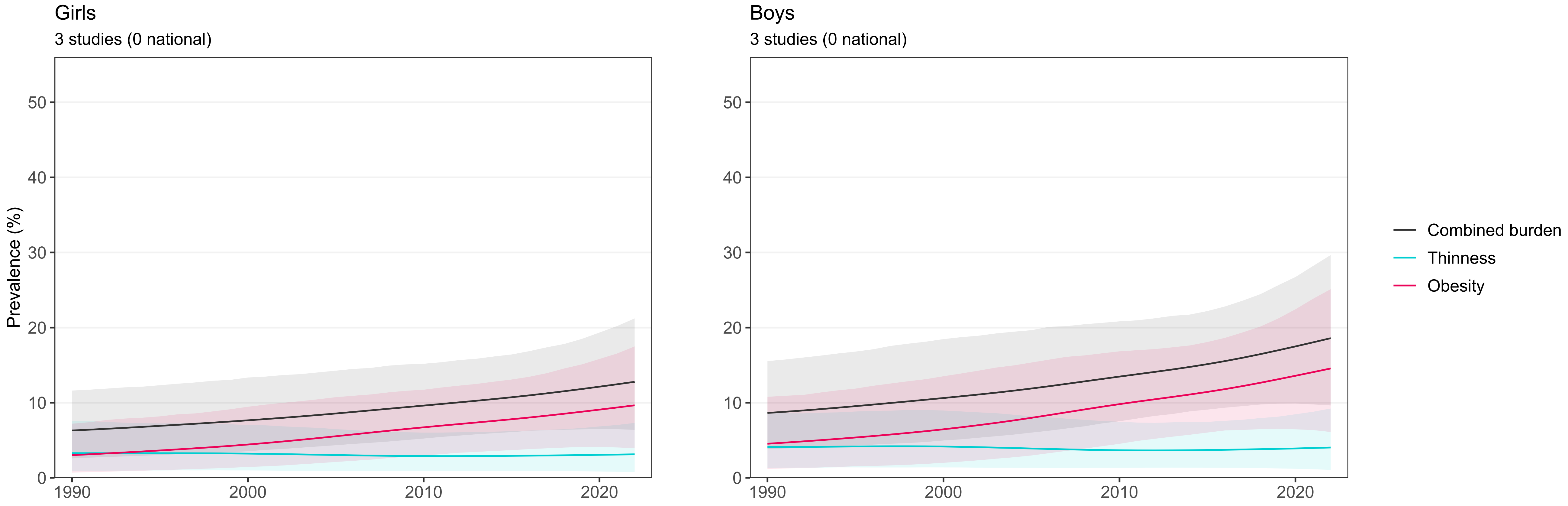


## Adults

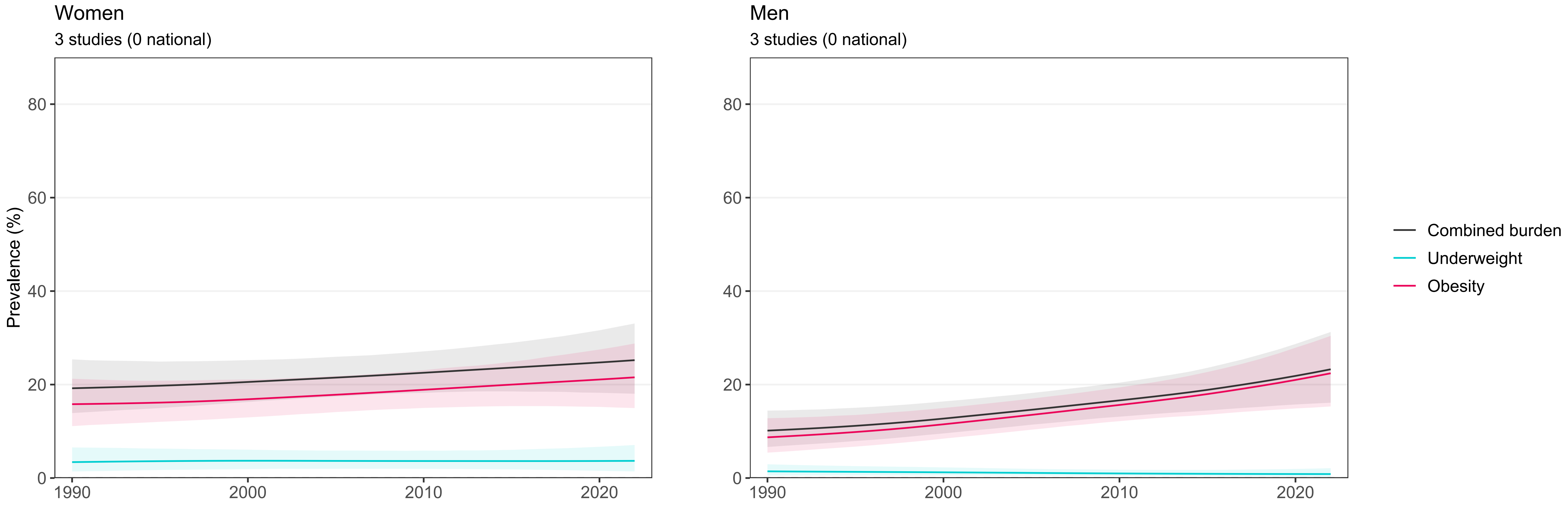

# Botswana

## School-aged children and adolescents

### Girls
1 study (1 national)

### Boys
1 study (1 national)

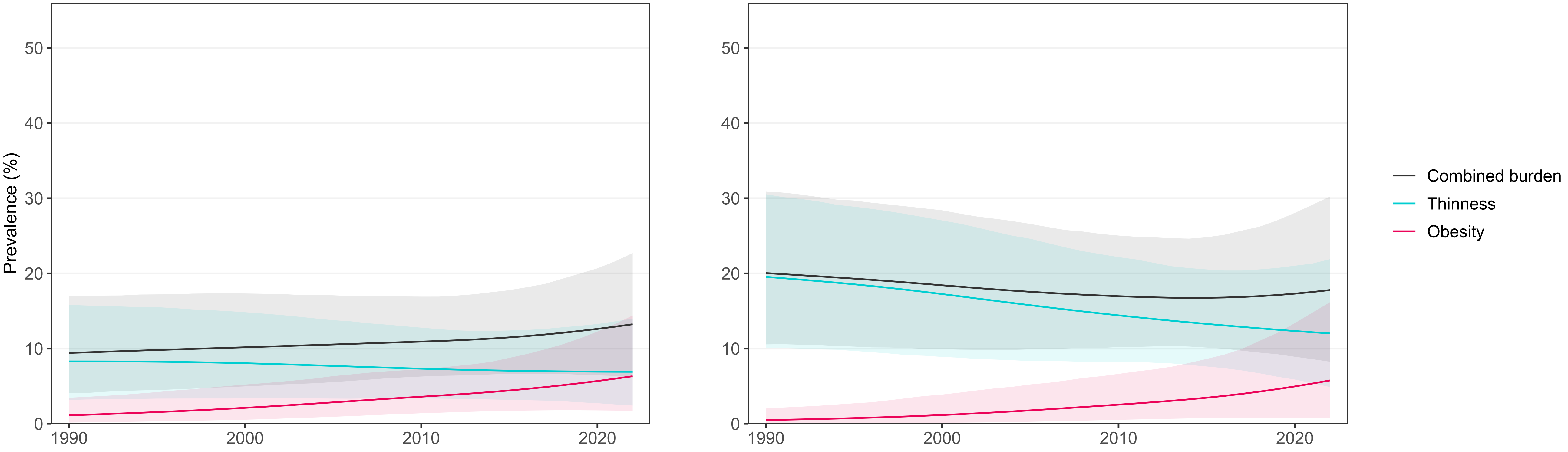


## Adults

### Women
2 studies (2 national)

### Men
2 studies (2 national)

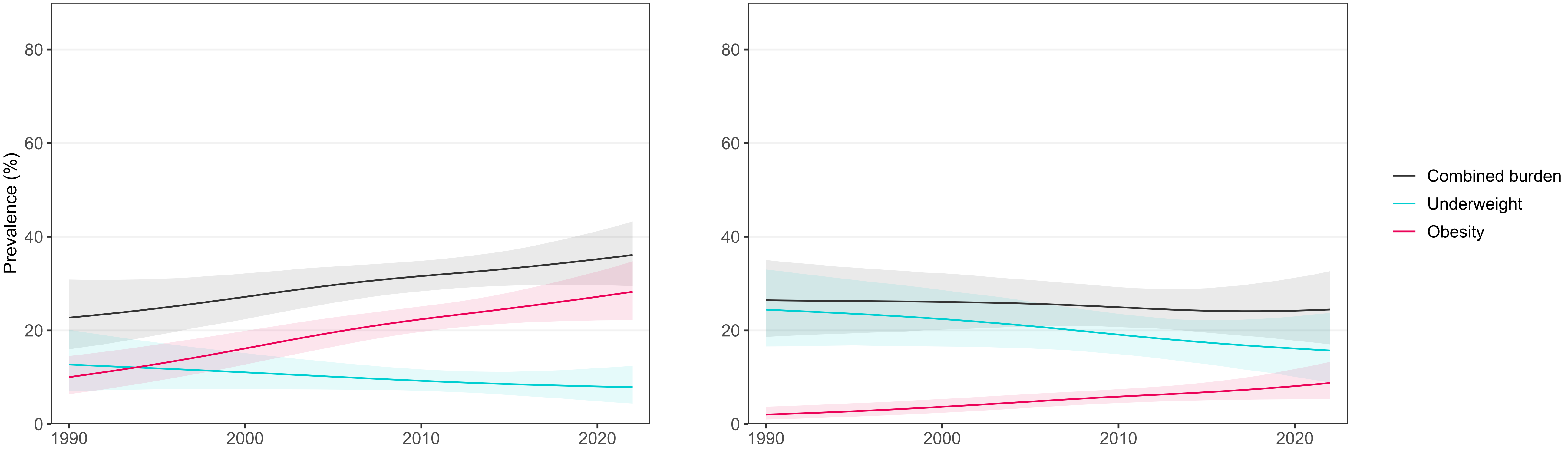

# Brazil

## School-aged children and adolescents

Girls
86 studies (32 national)

Boys
85 studies (31 national)

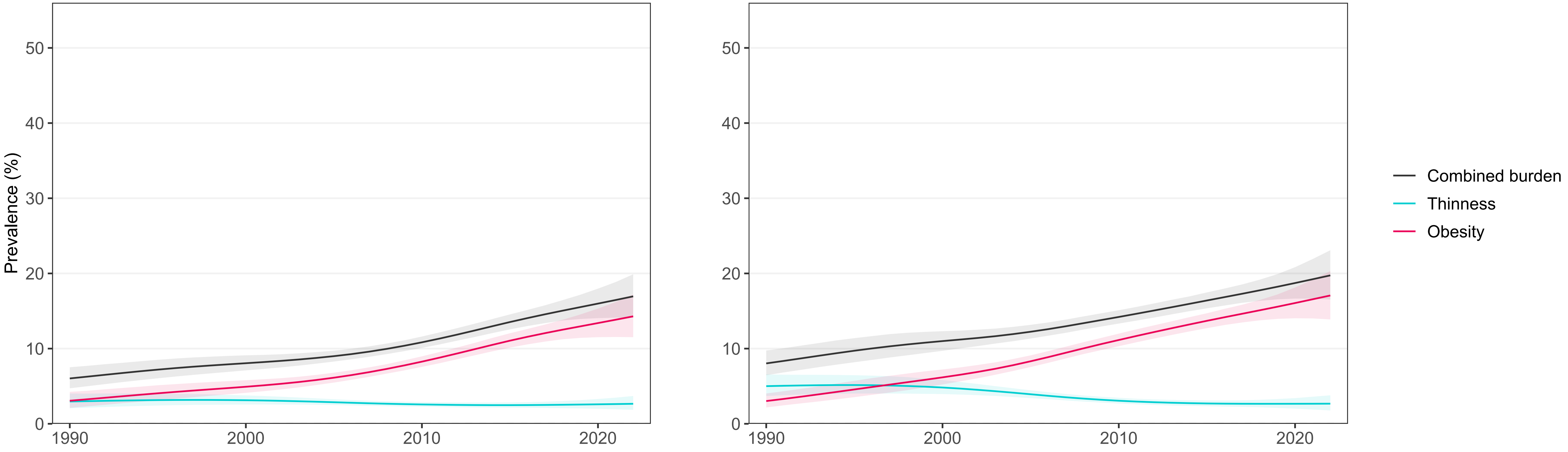


## Adults

Women
60 studies (10 national)

Men
52 studies (8 national)

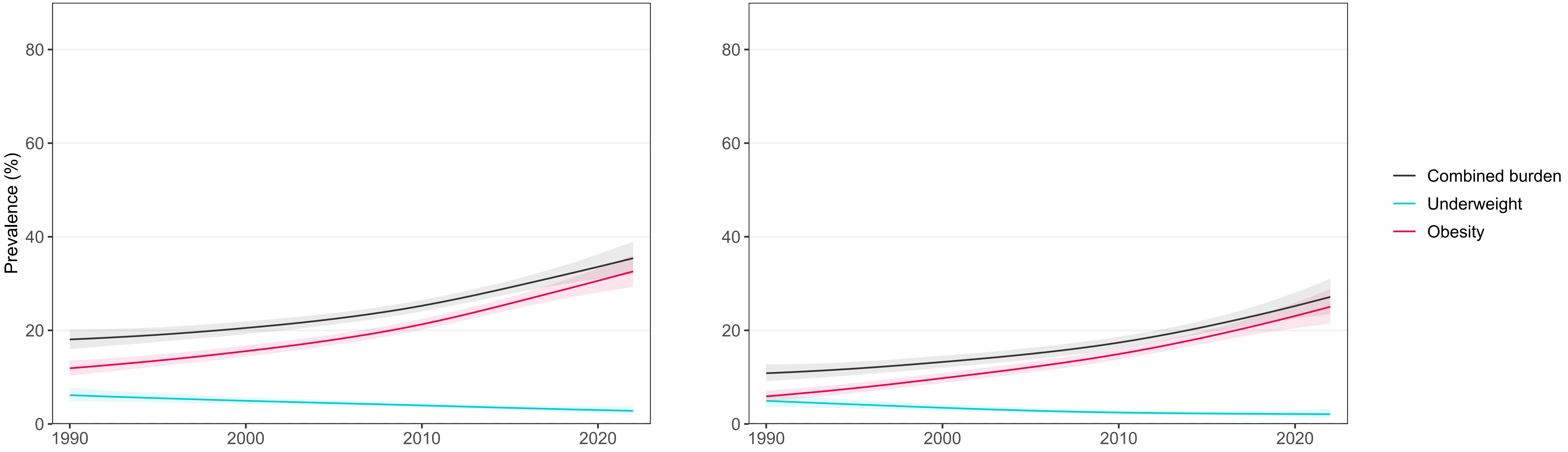

# Brunei Darussalam

## School-aged children and adolescents

### Girls

4 studies (4 national)

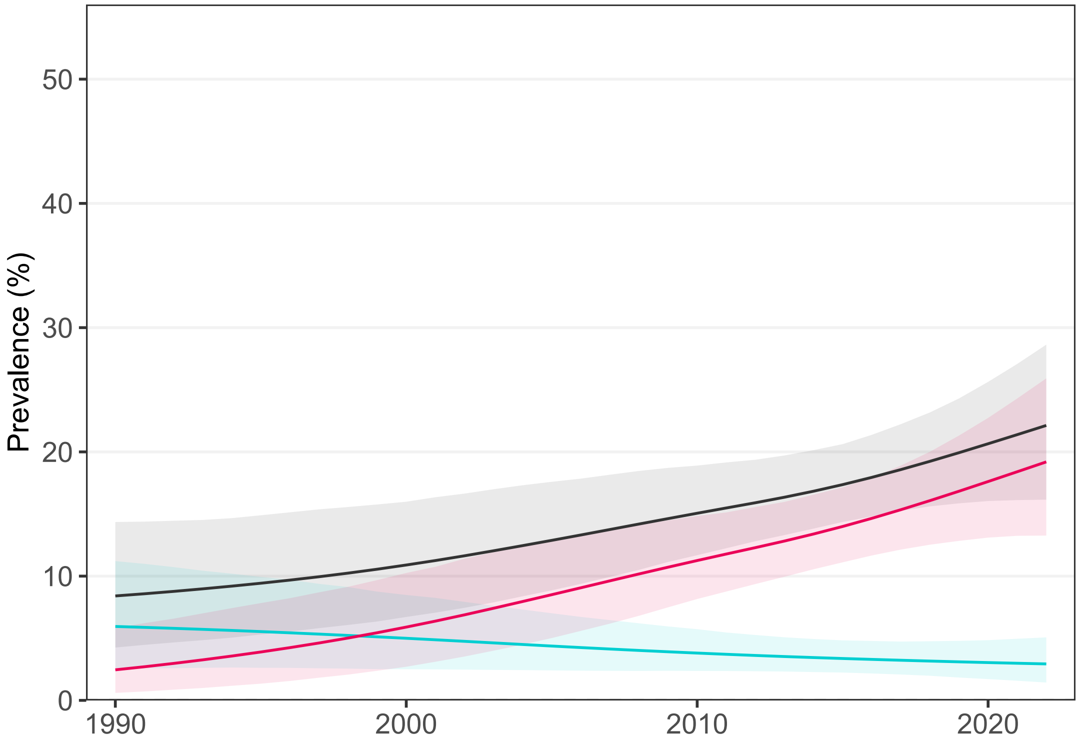


### Boys

4 studies (4 national)

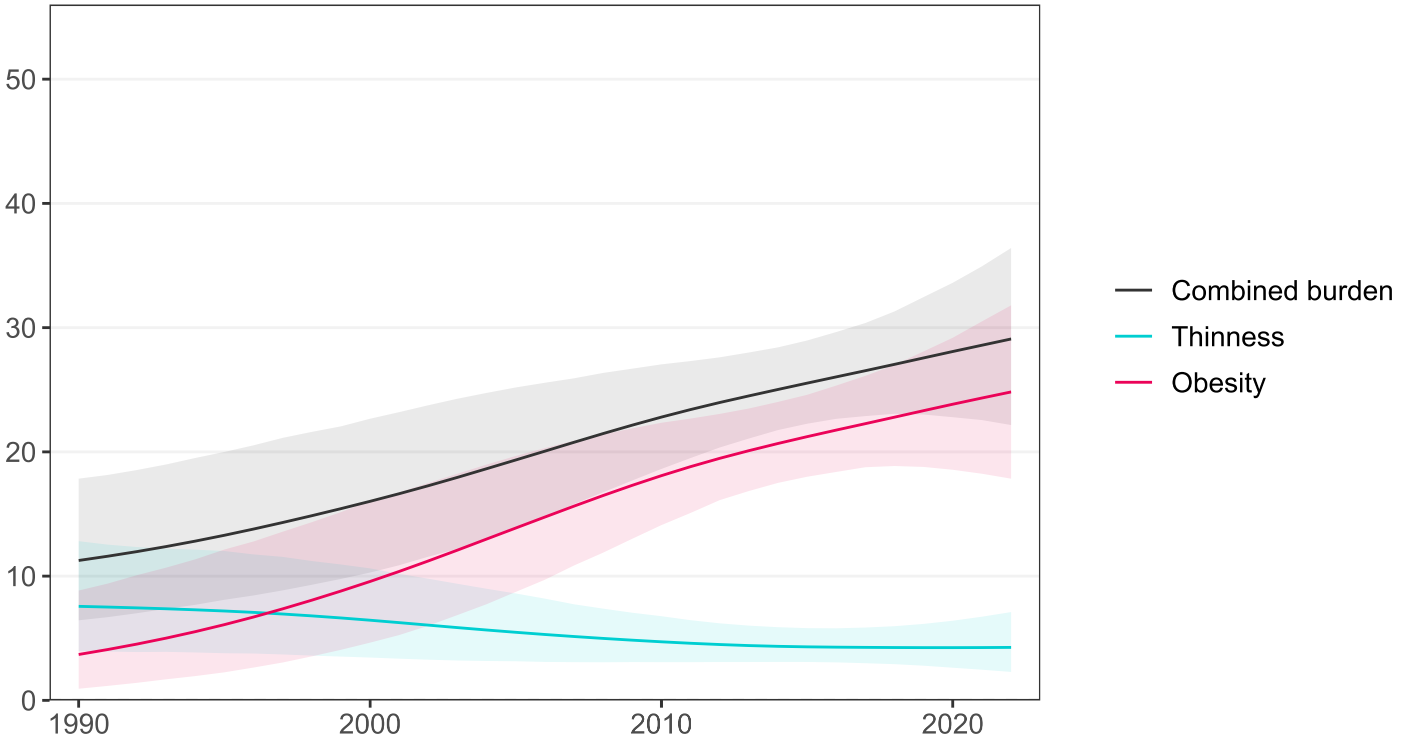


## Adults

### Women

2 studies (2 national)

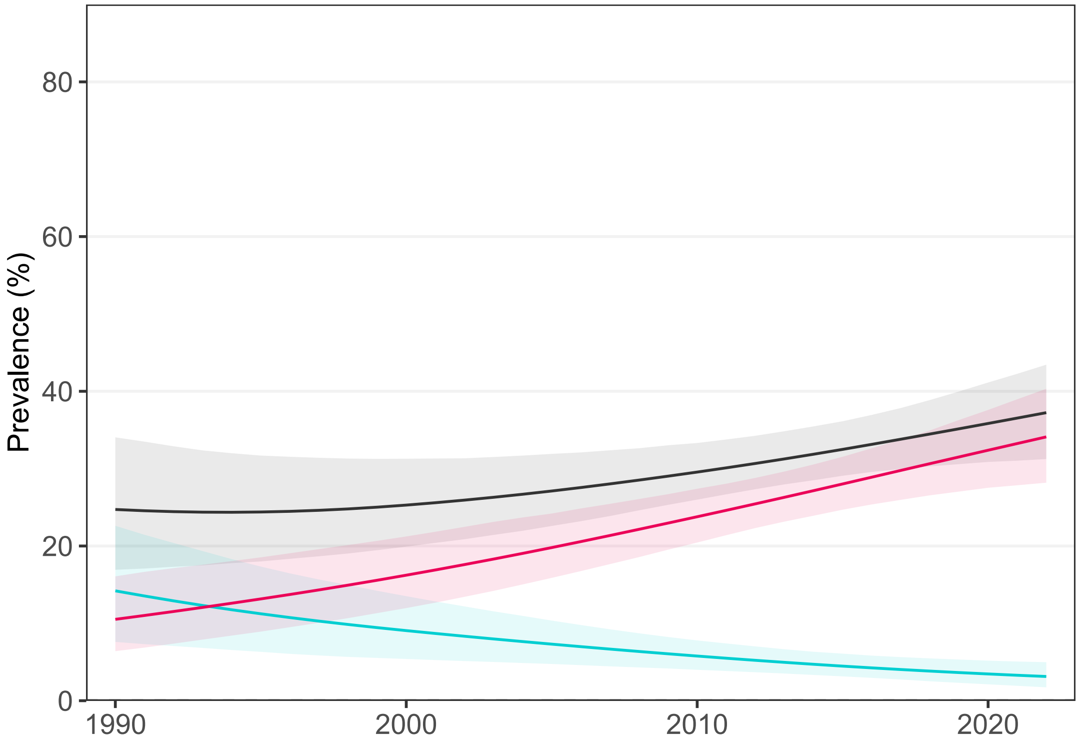


### Men

2 studies (2 national)

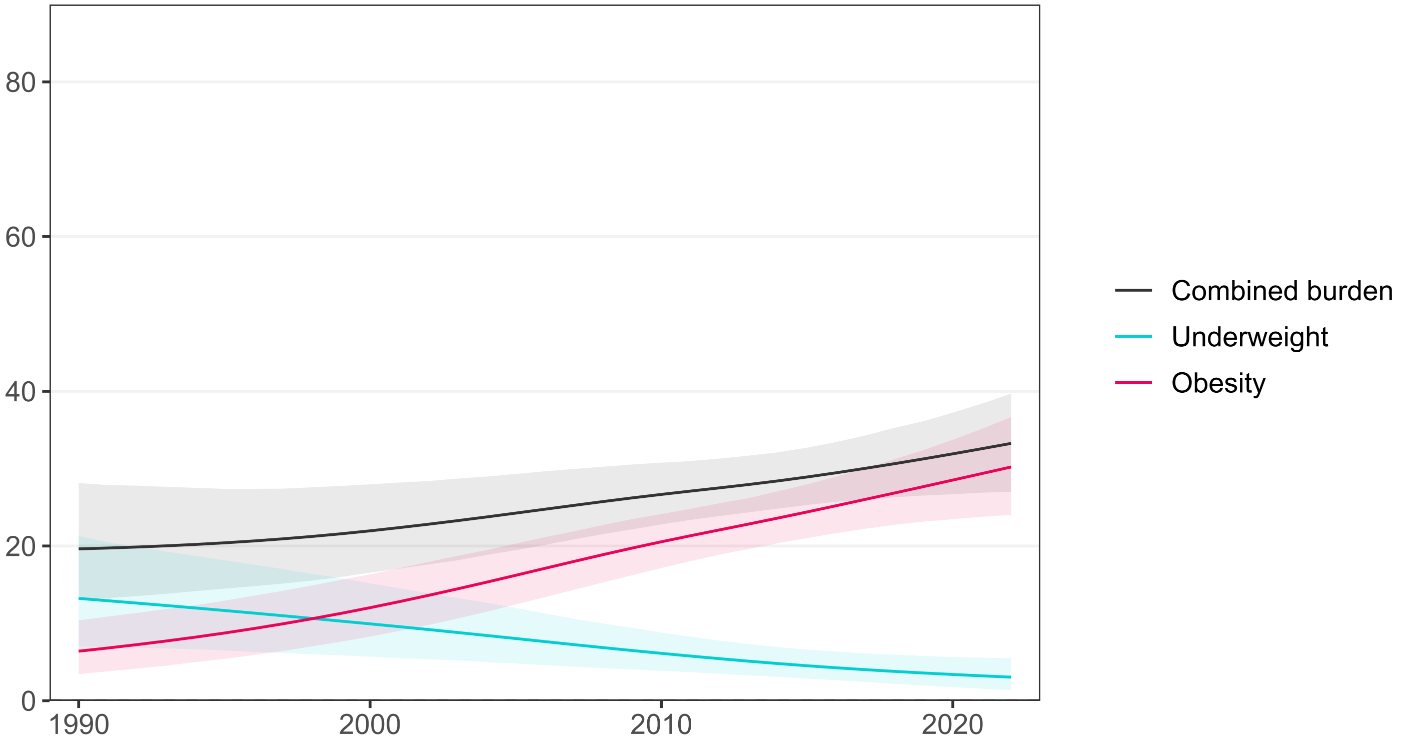

# Bulgaria

## School-aged children and adolescents

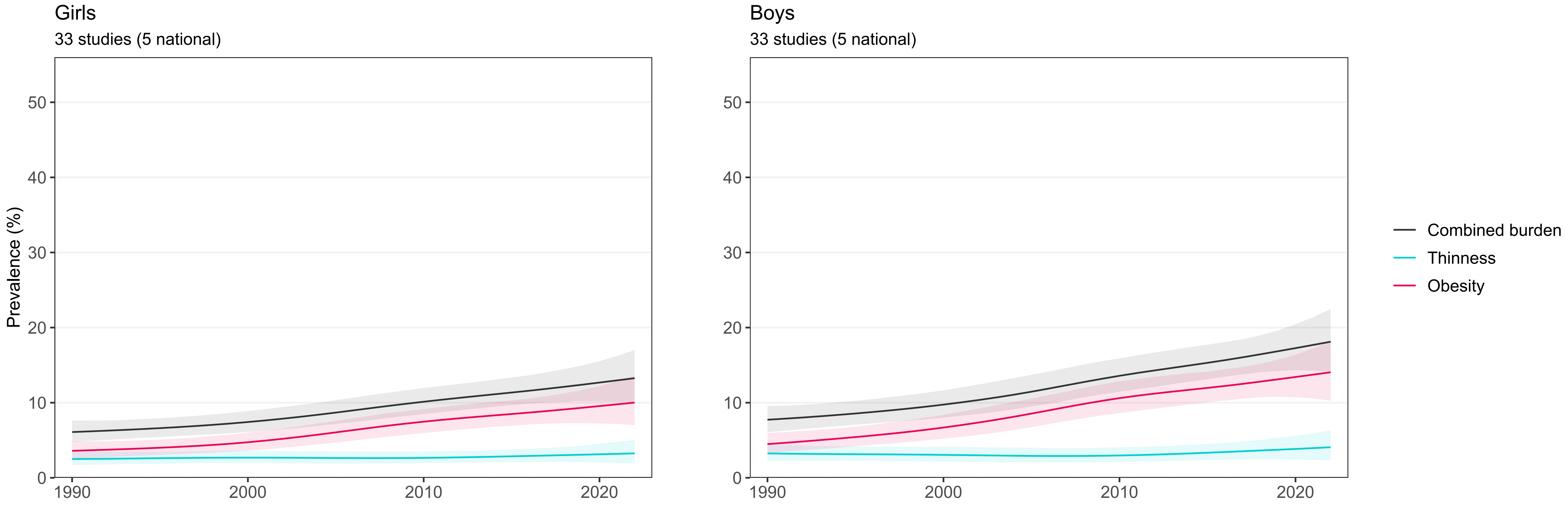


## Adults

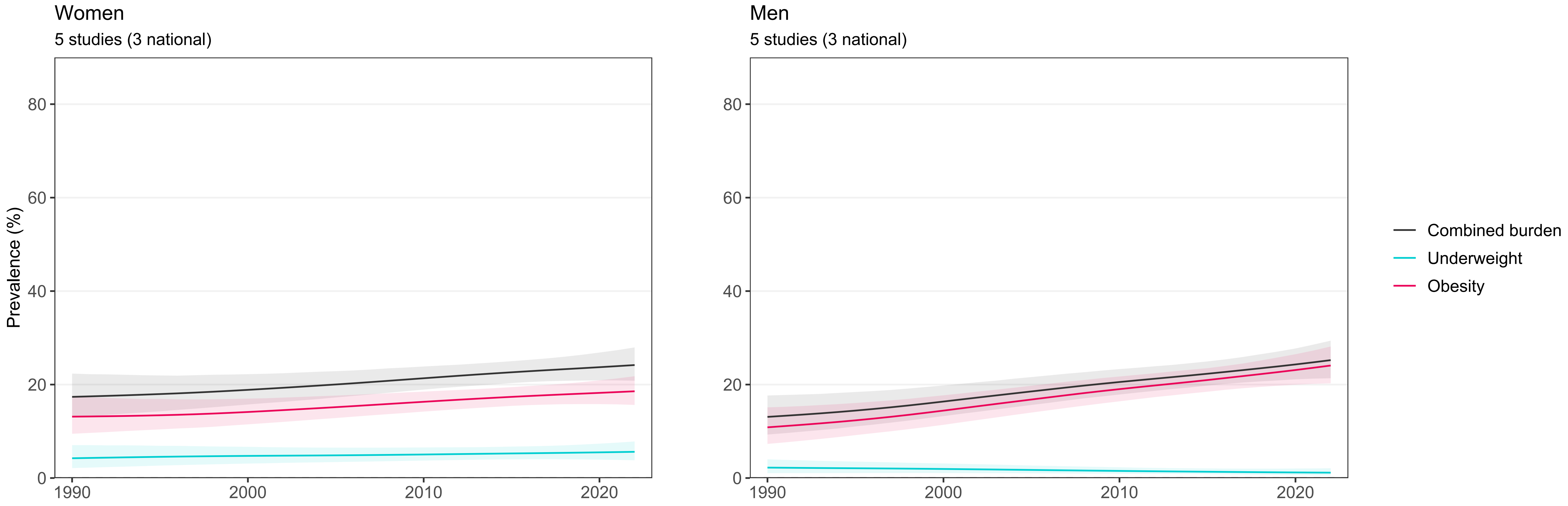

# Burkina Faso

## School-aged children and adolescents

### Girls

6 studies (4 national)

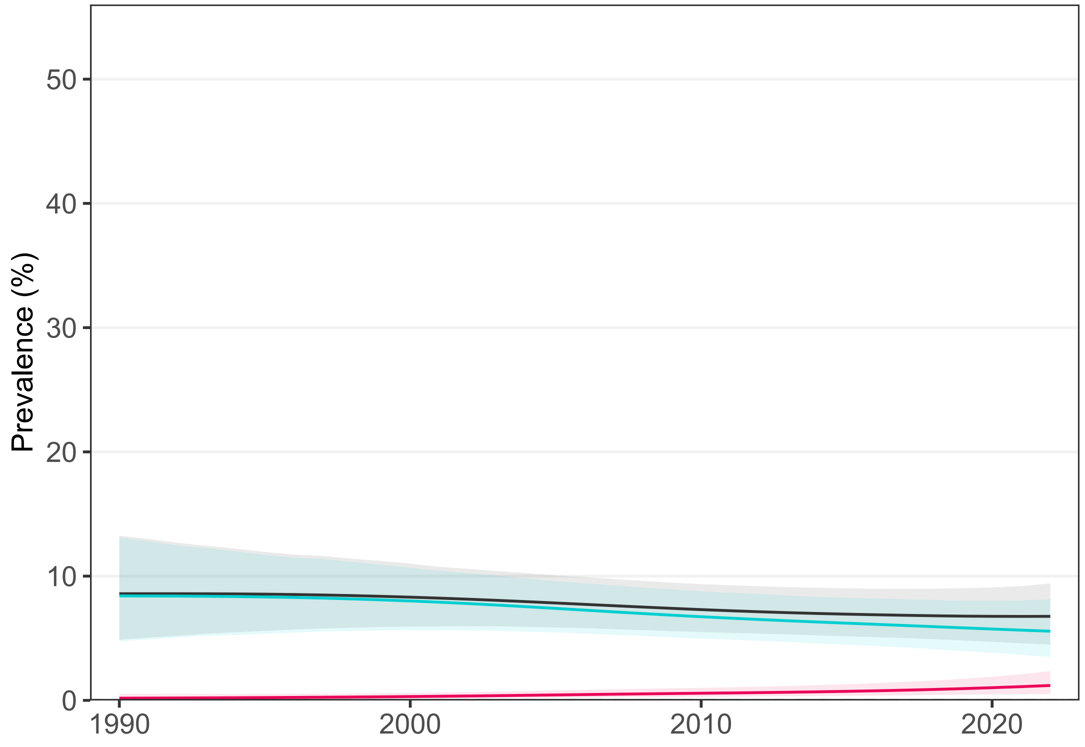


### Boys

3 studies (1 national)

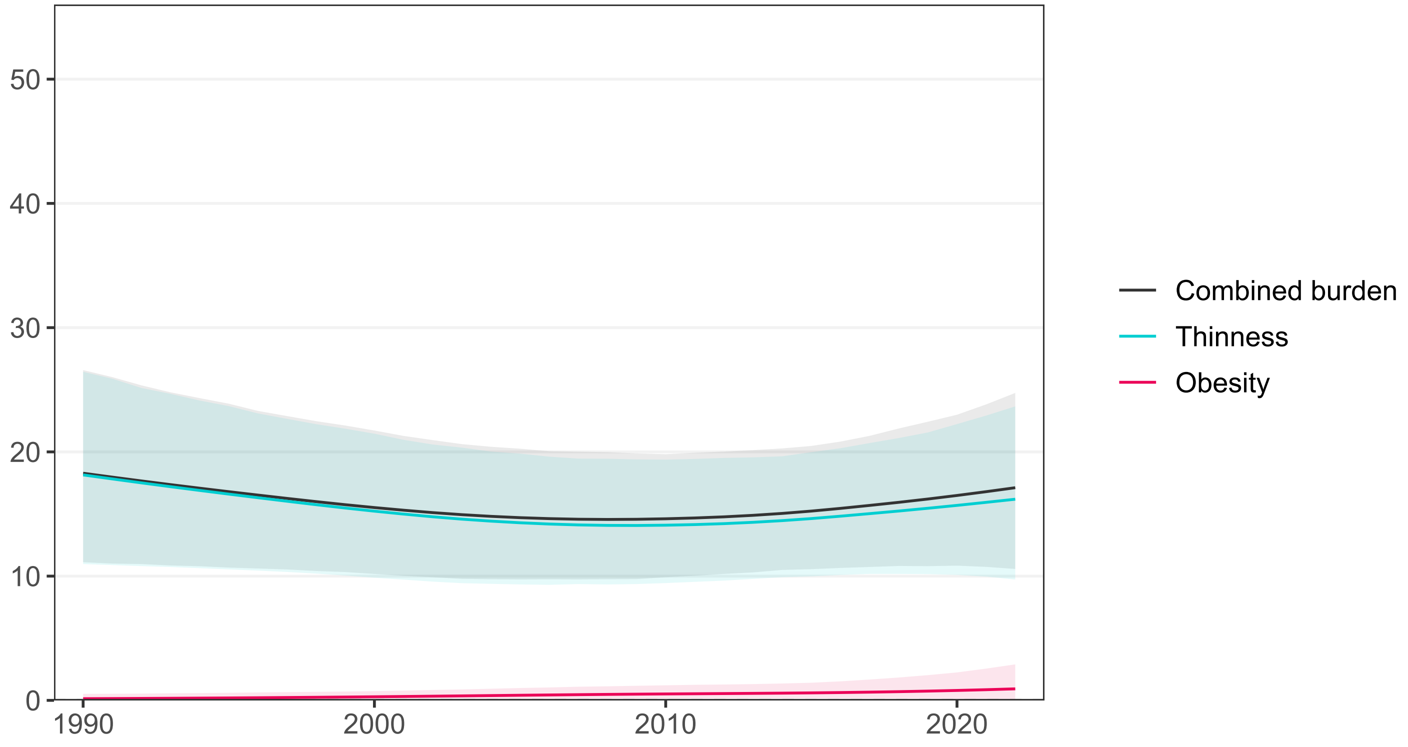


## Adults

### Women

10 studies (7 national)

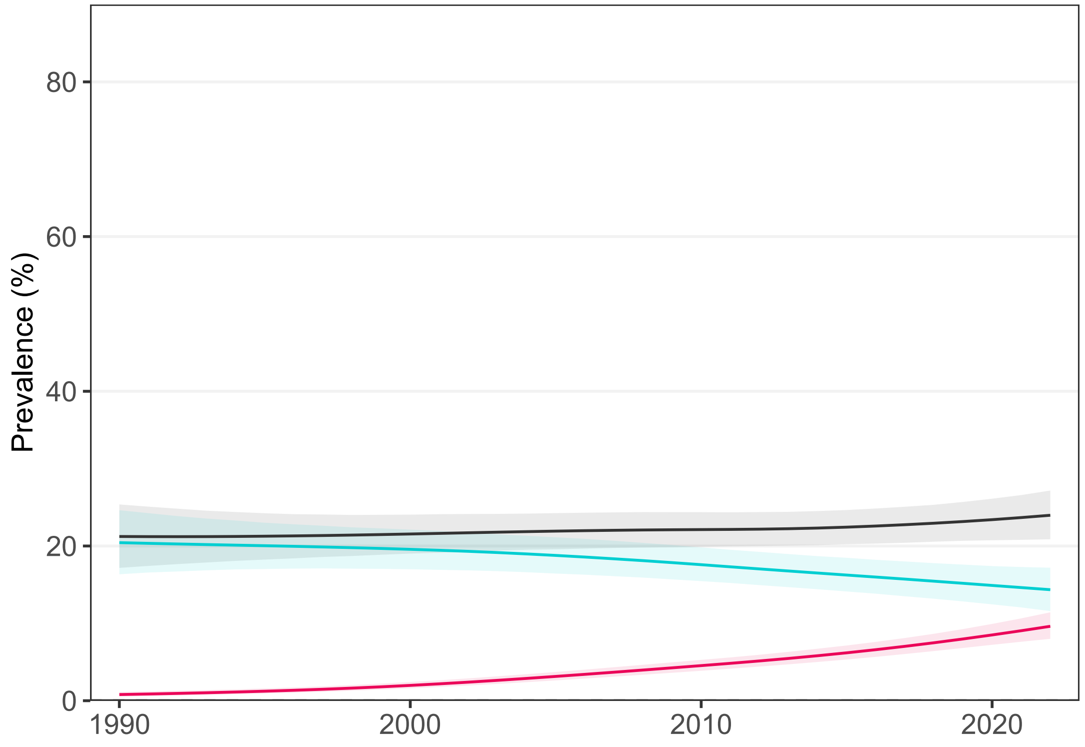


### Men

5 studies (2 national)

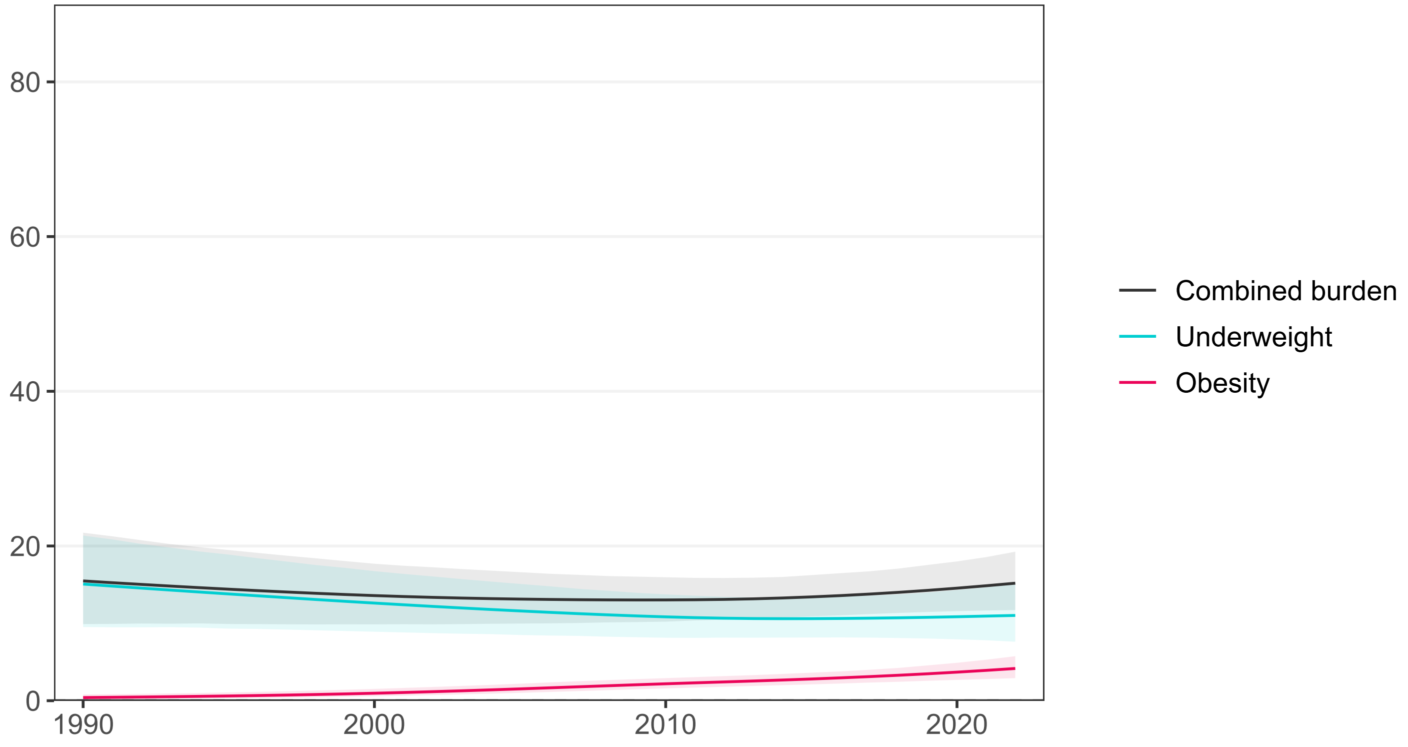

# Burundi

## School-aged children and adolescents

### Girls
2 studies (2 national)

### Boys
No studies

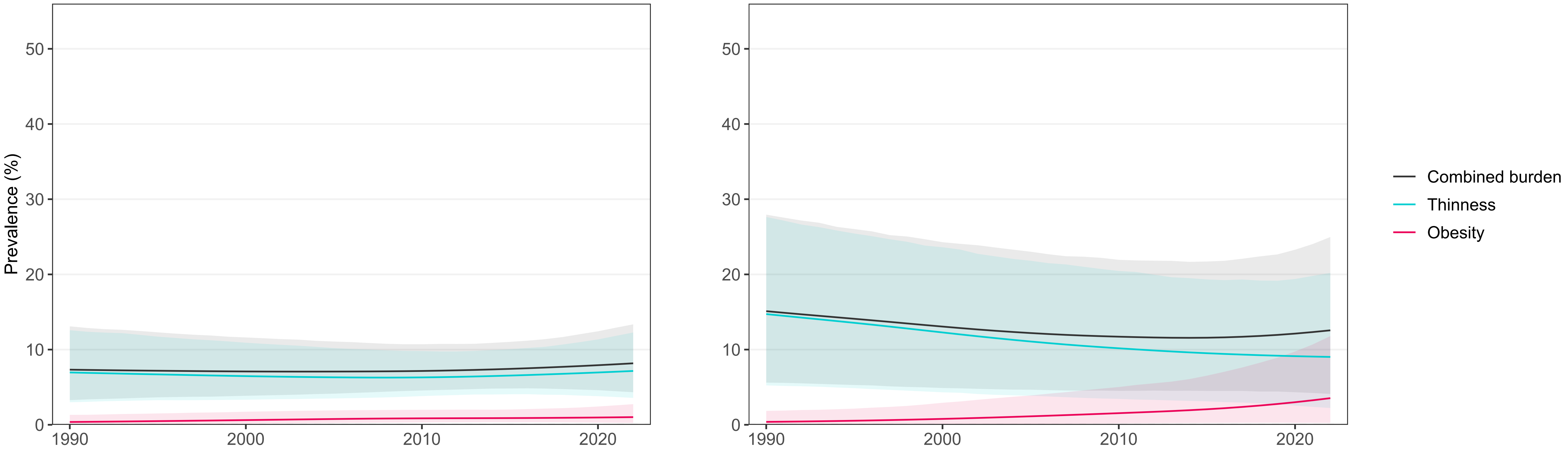


## Adults

### Women
2 studies (2 national)

### Men
No studies

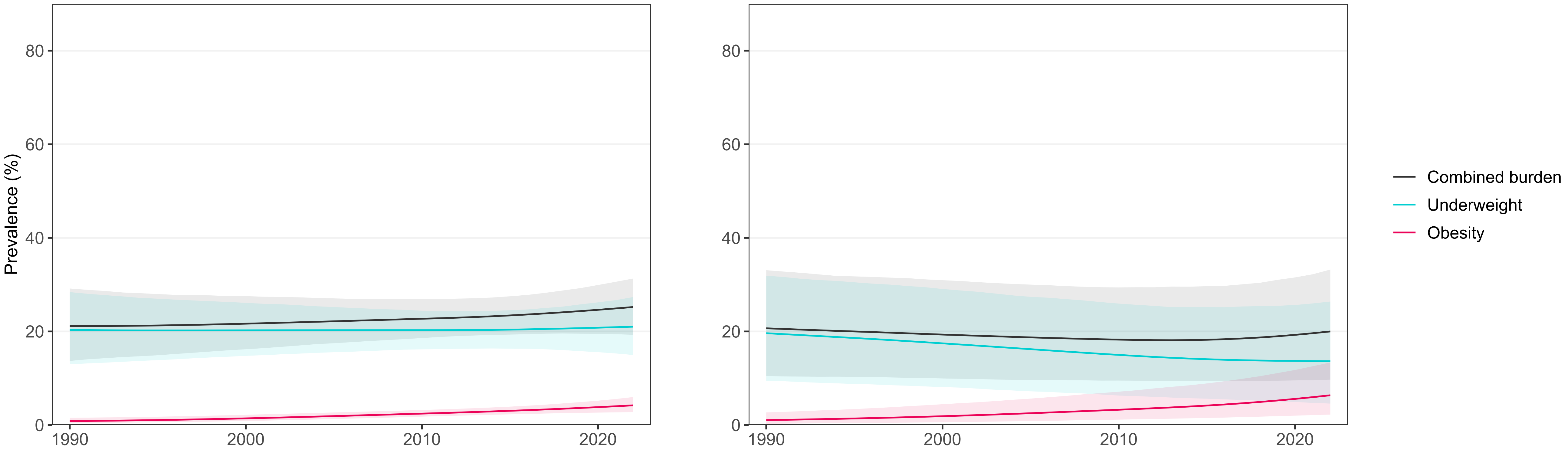

# Cabo Verde

## School-aged children and adolescents

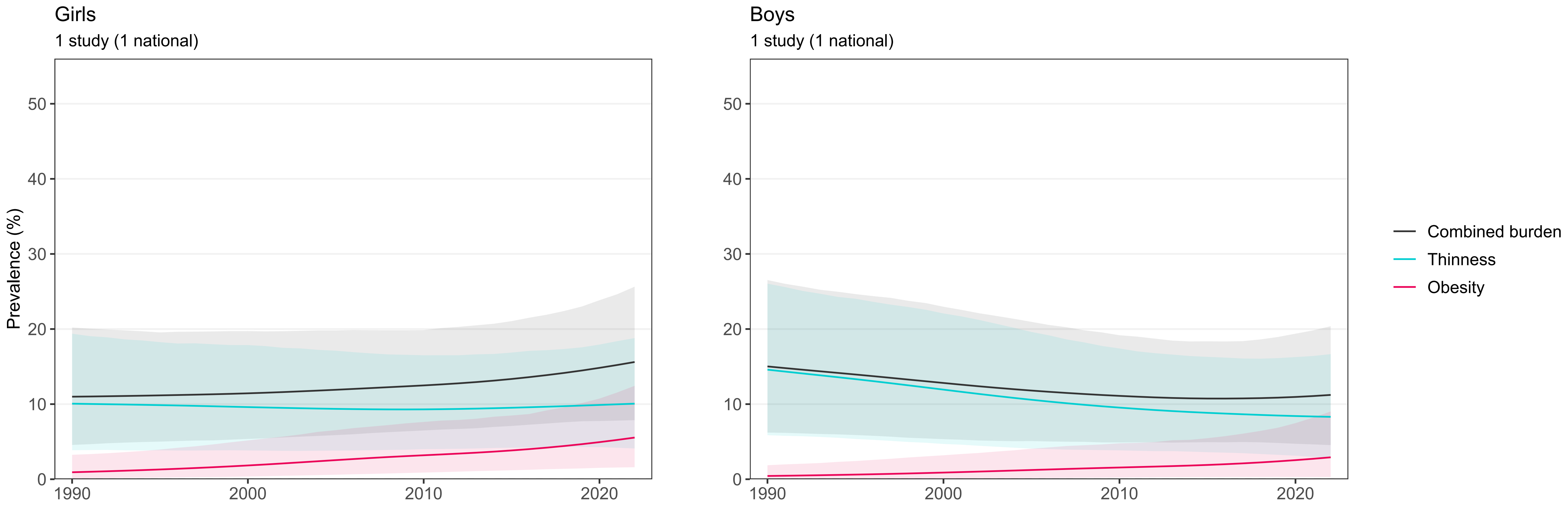


## Adults

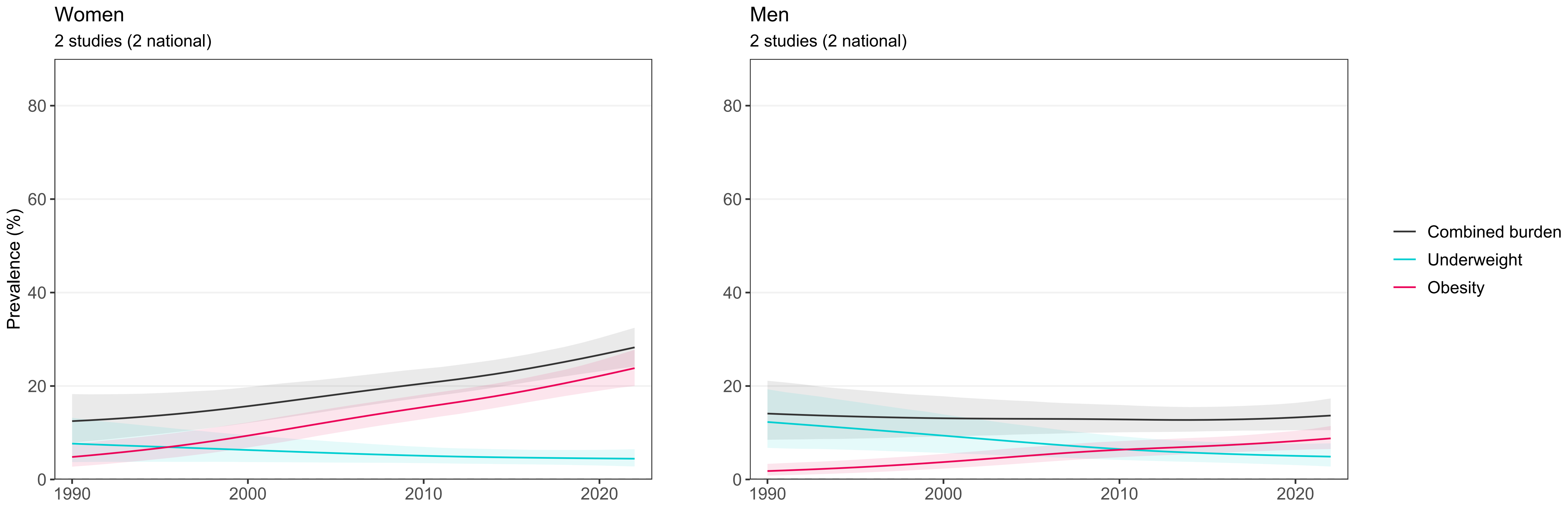

# Cambodia

## School-aged children and adolescents

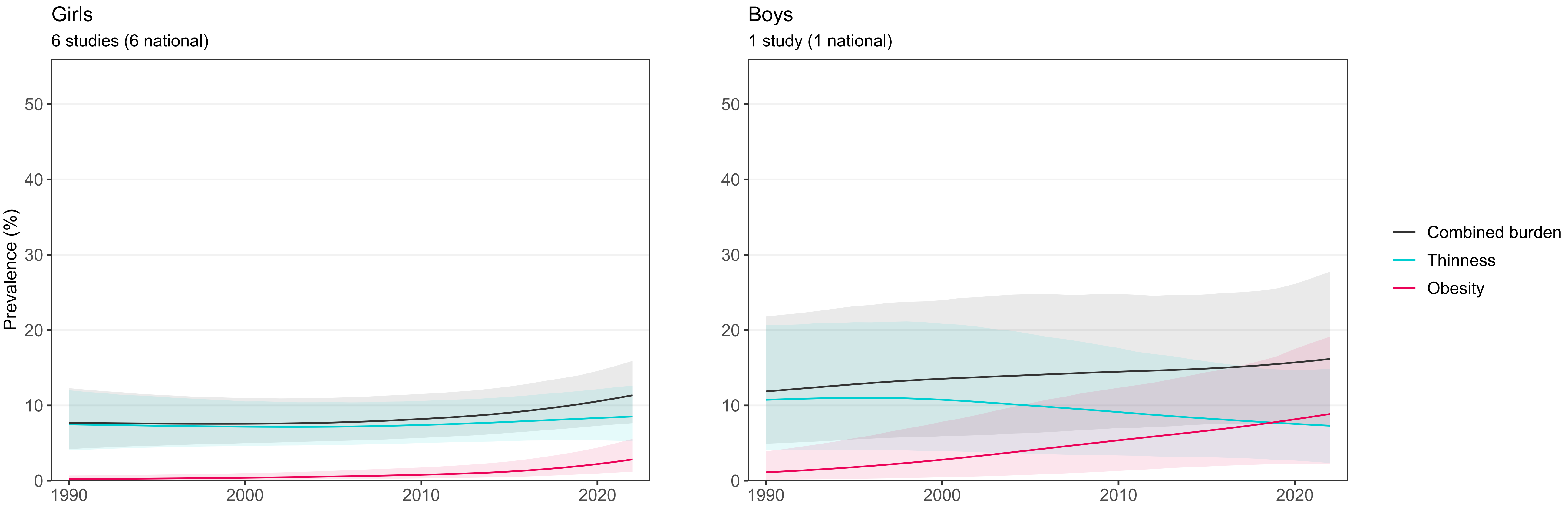


## Adults

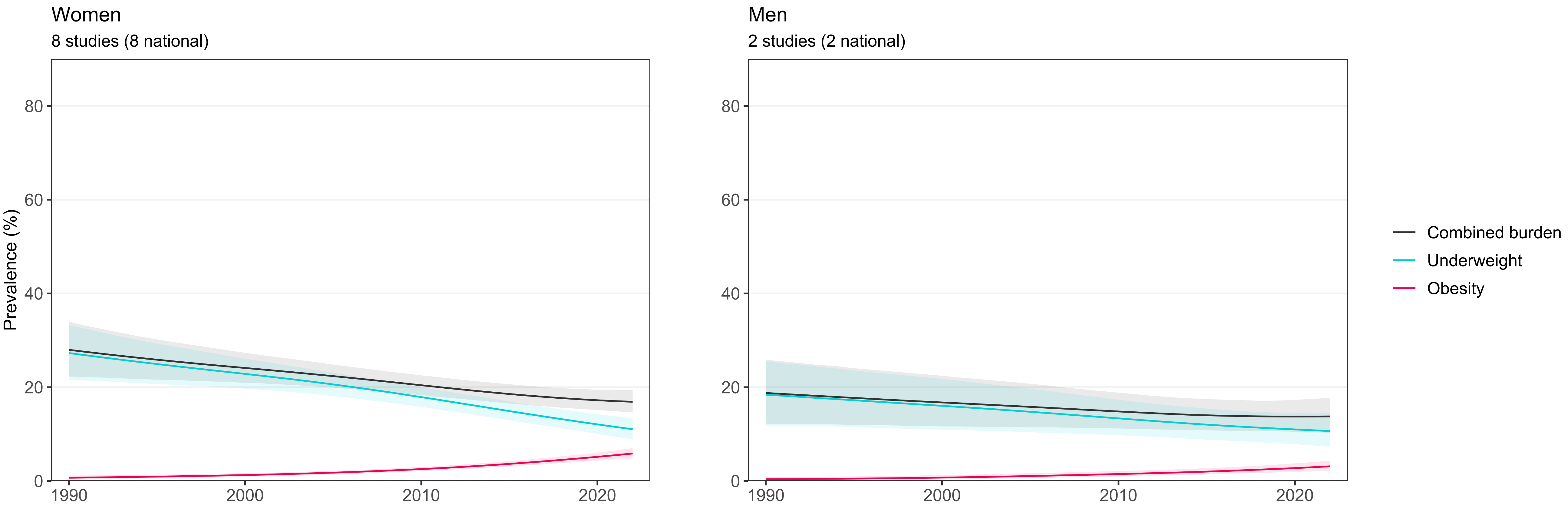

# Cameroon

## School-aged children and adolescents

### Girls

9 studies (4 national)

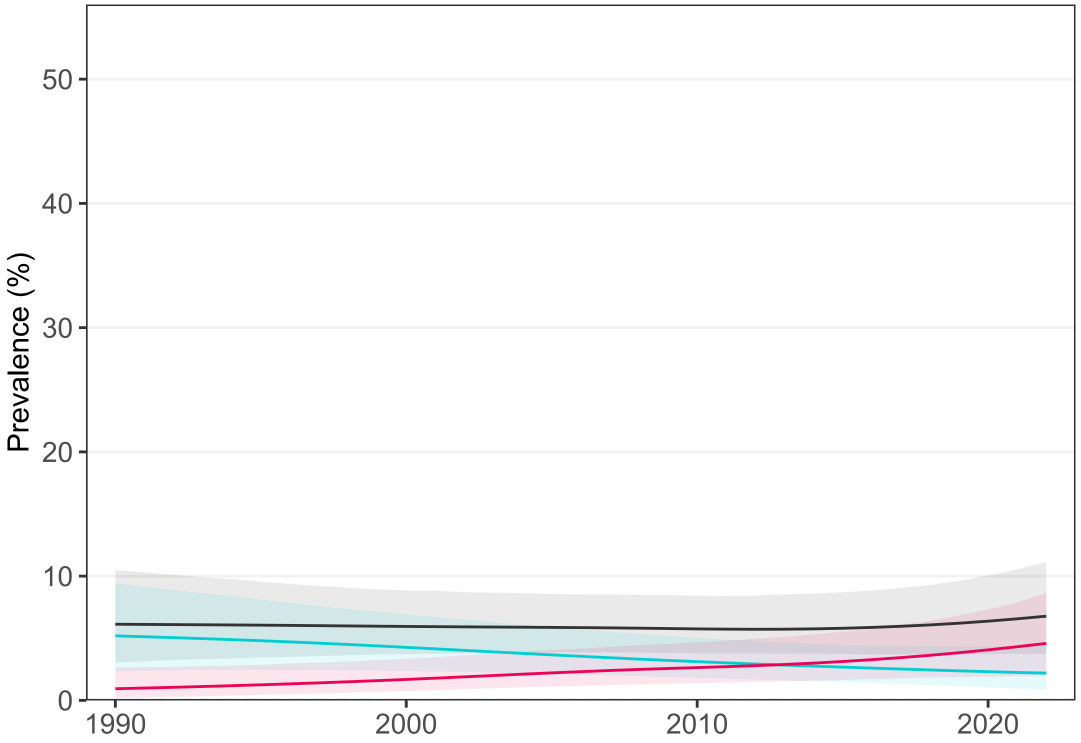


### Boys

5 studies (0 national)

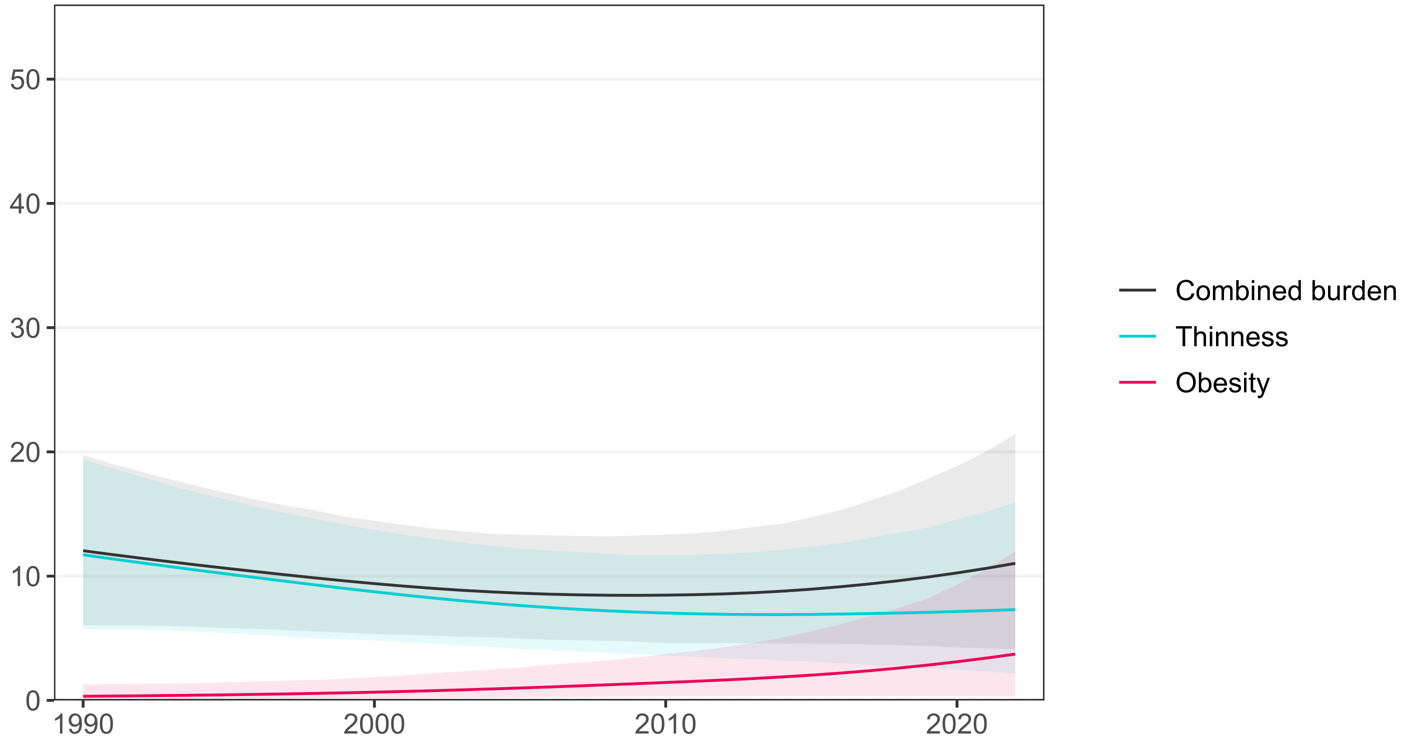


## Adults

### Women

14 studies (5 national)

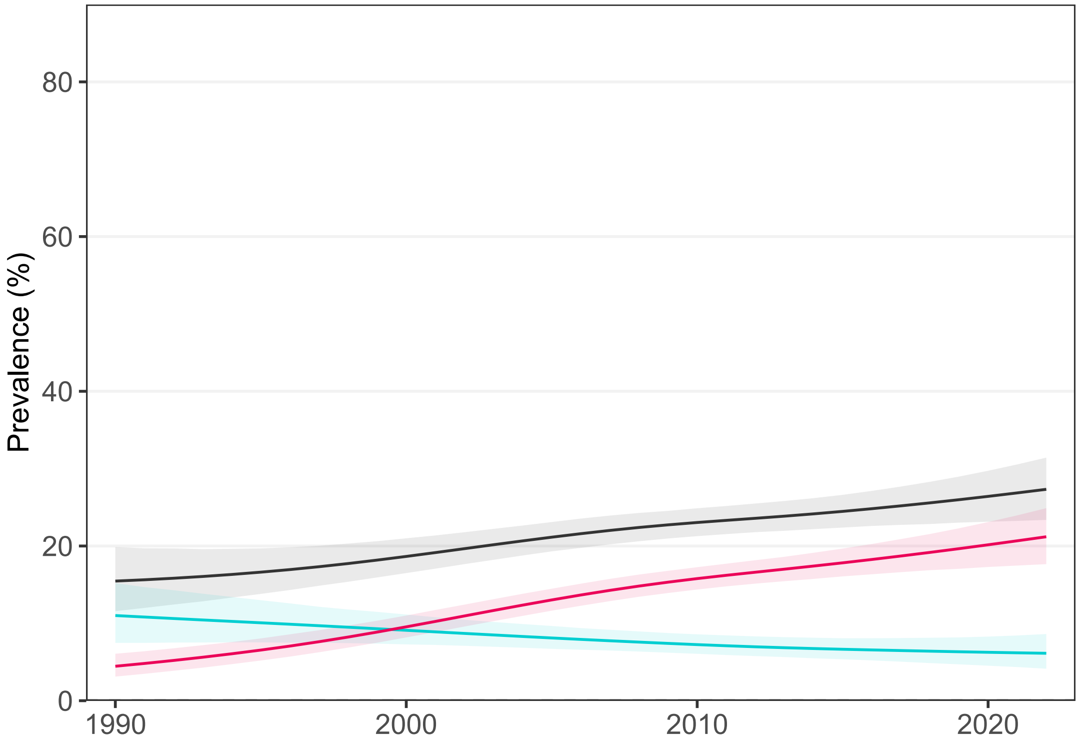


### Men

9 studies (0 national)

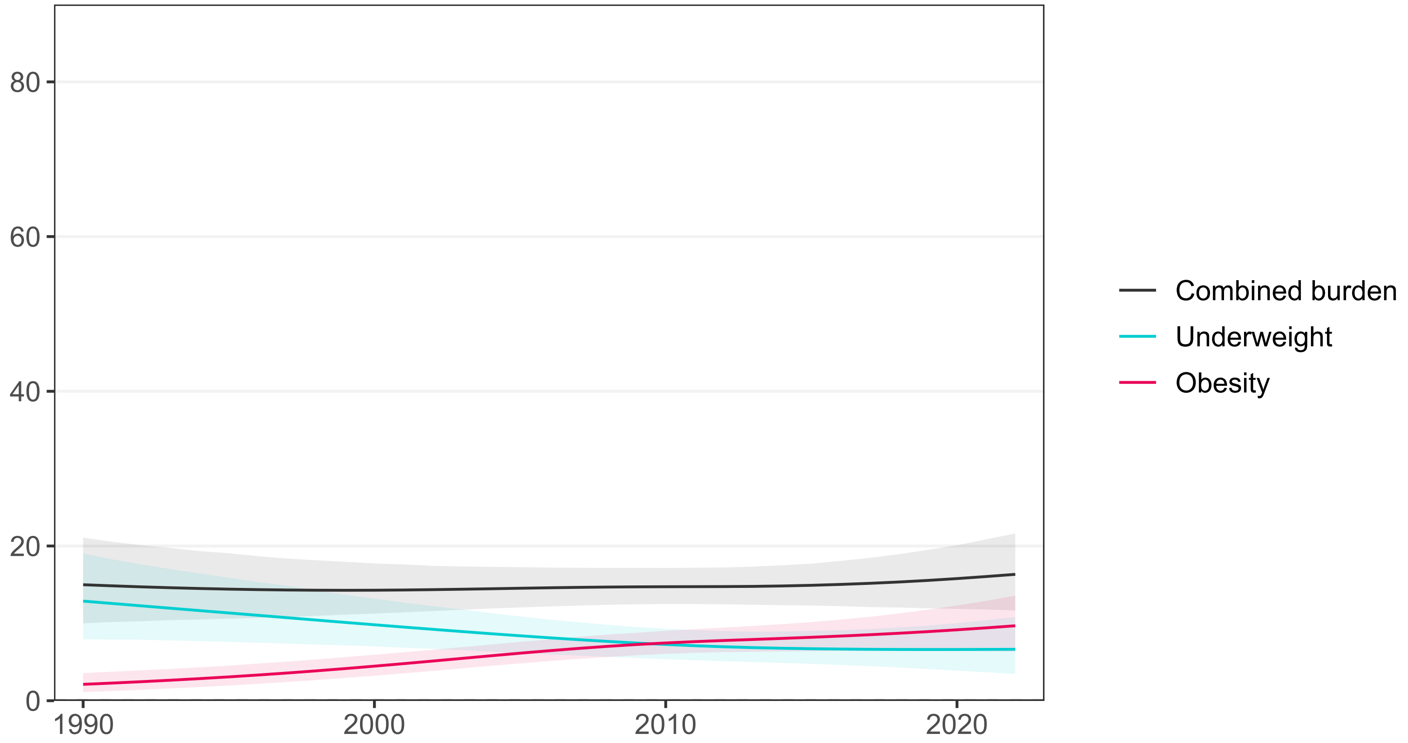

# Canada

## School-aged children and adolescents

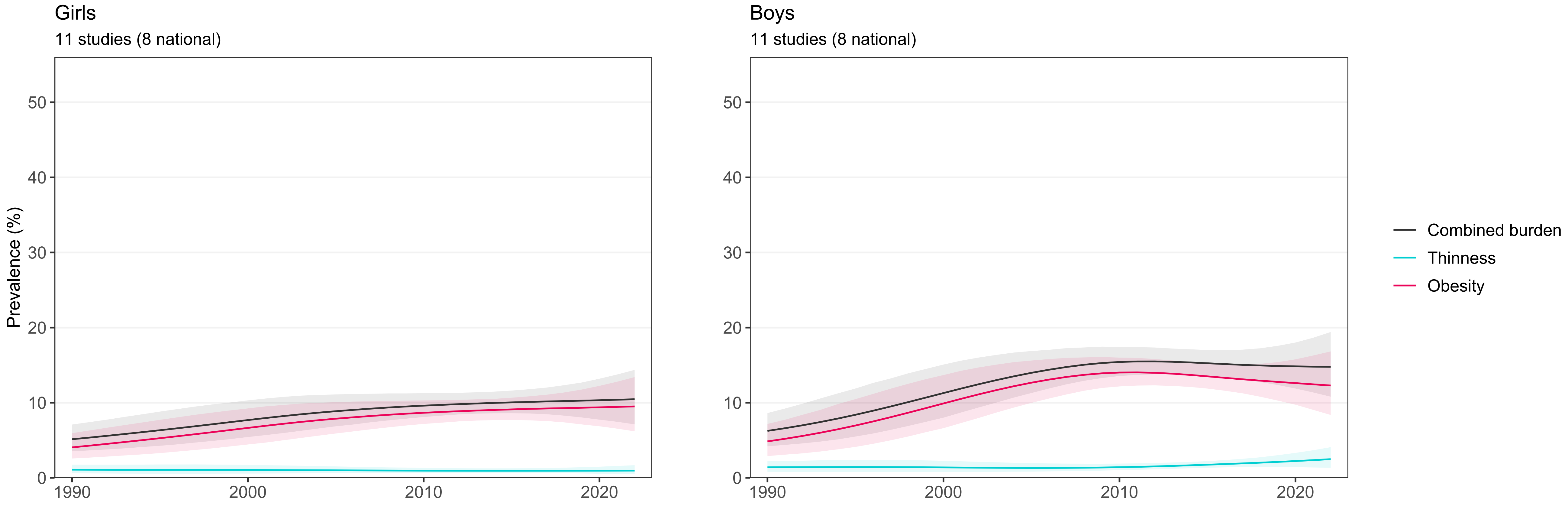


## Adults

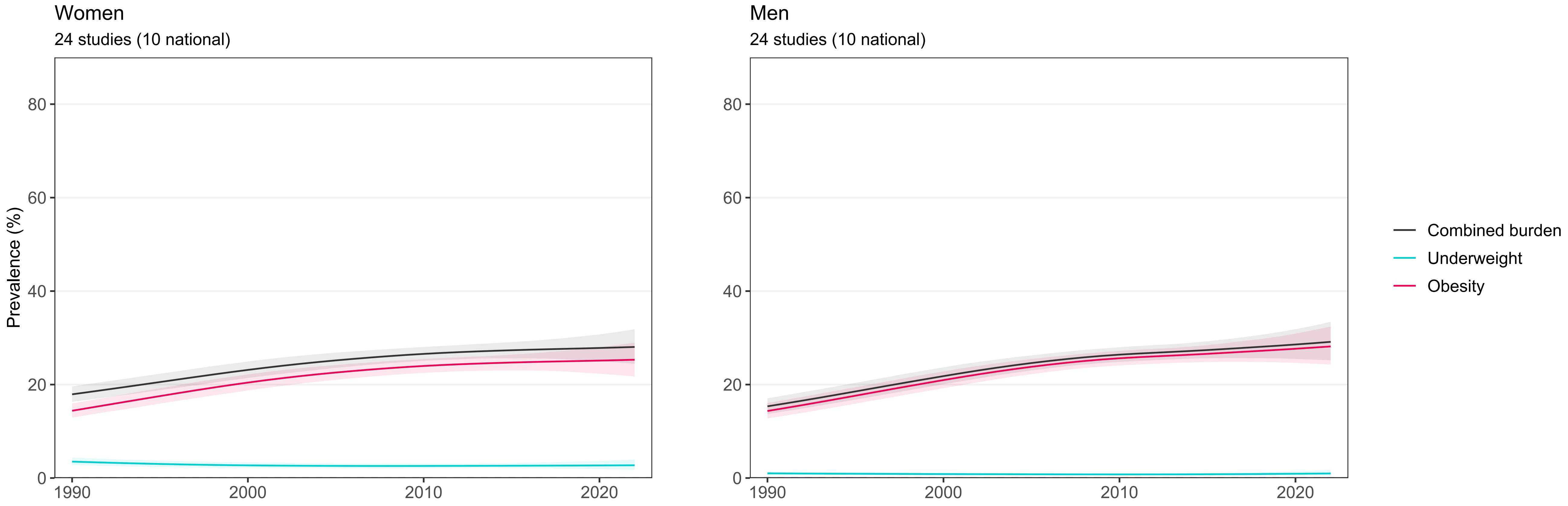

# Central African Republic

## School-aged children and adolescents

### Girls

No studies

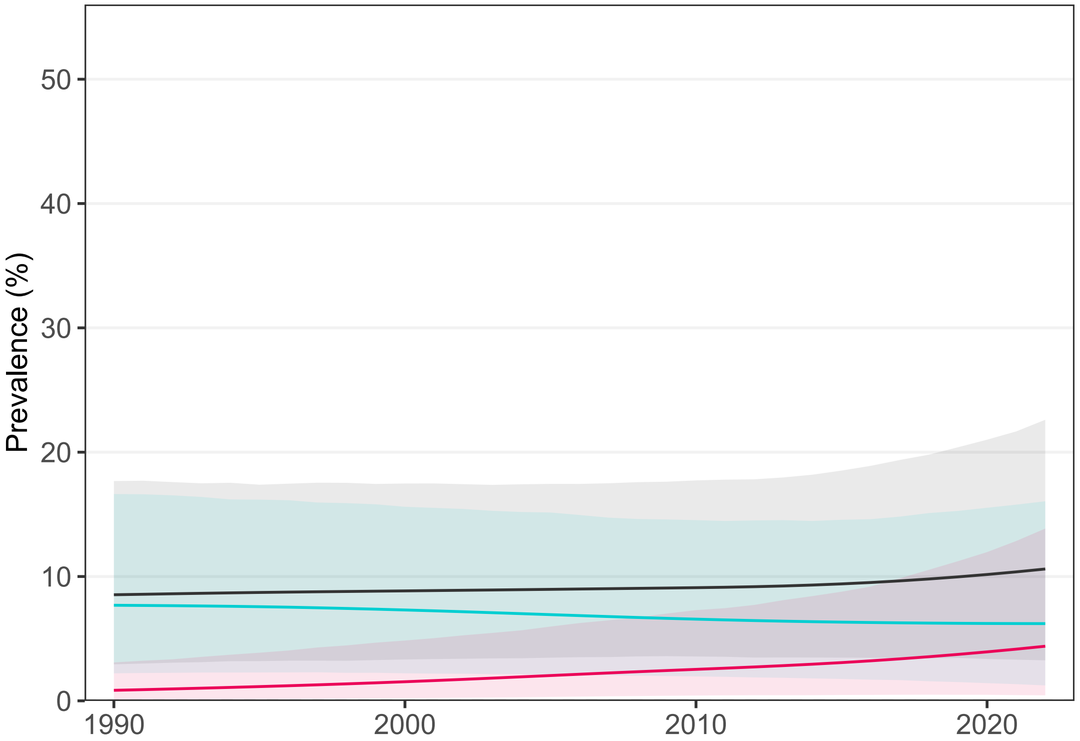


### Boys

No studies

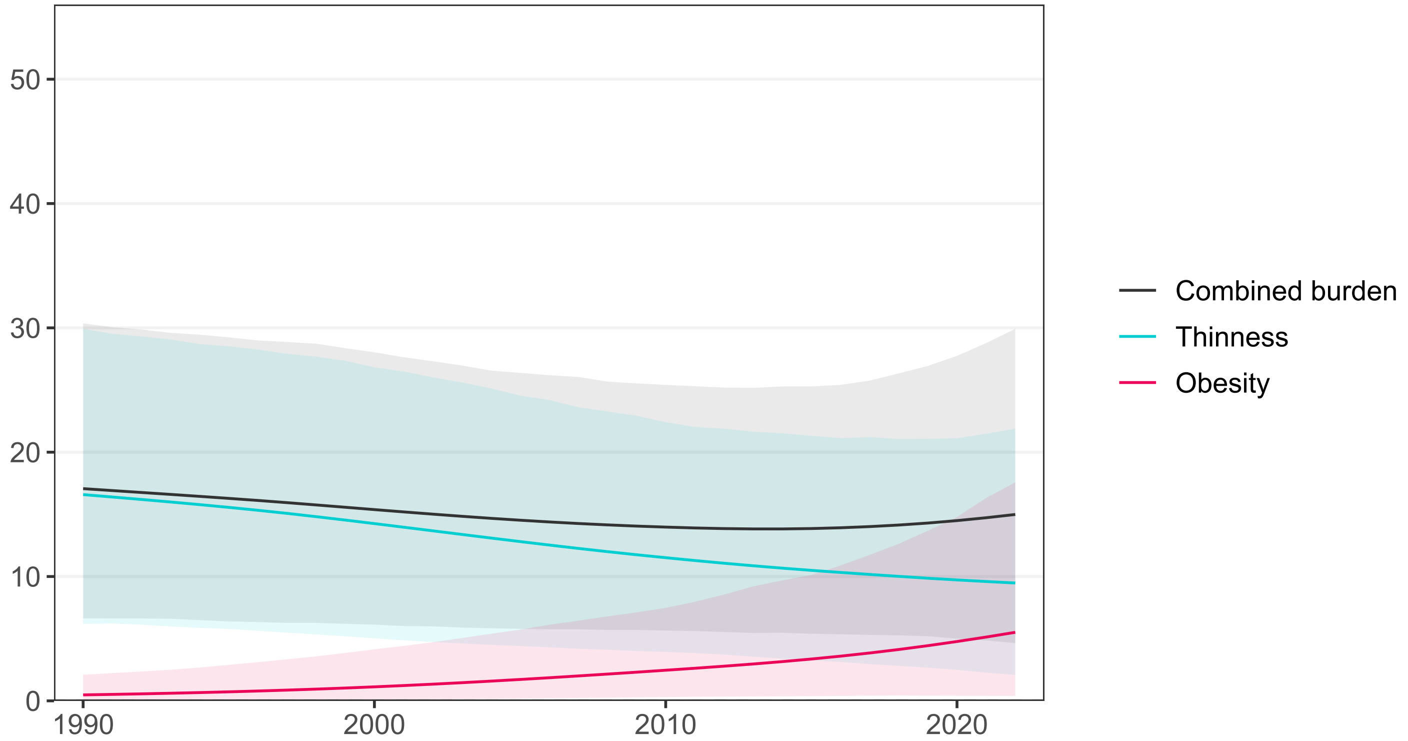


## Adults

### Women

5 studies (1 national)

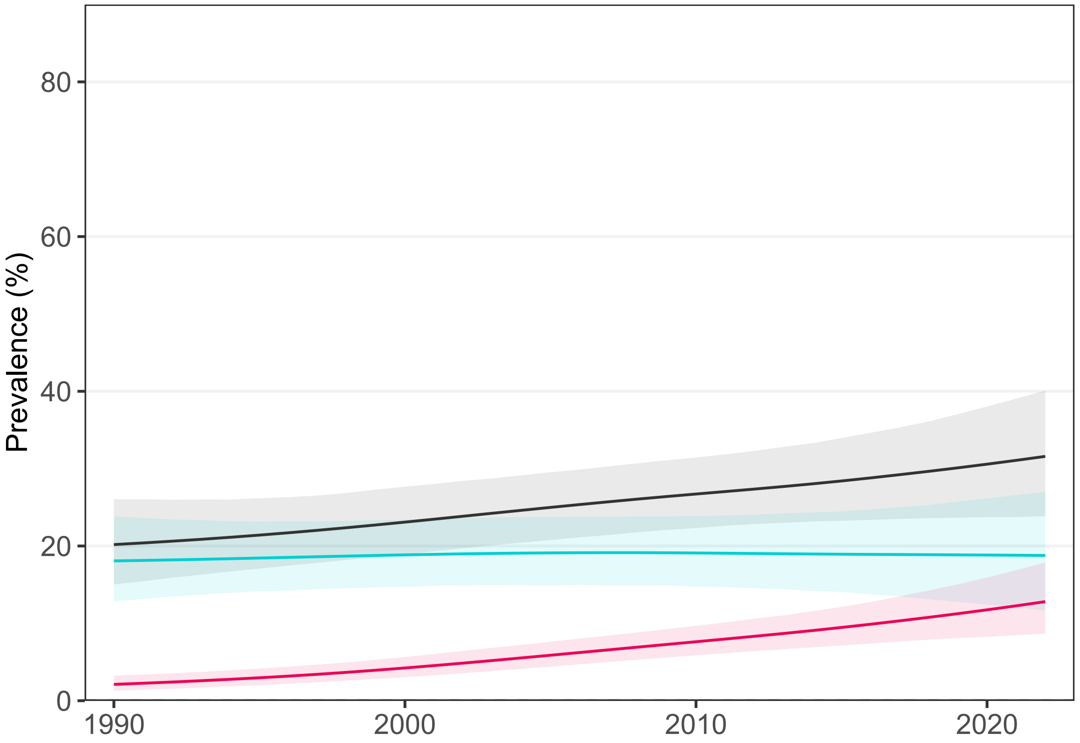


### Men

4 studies (0 national)

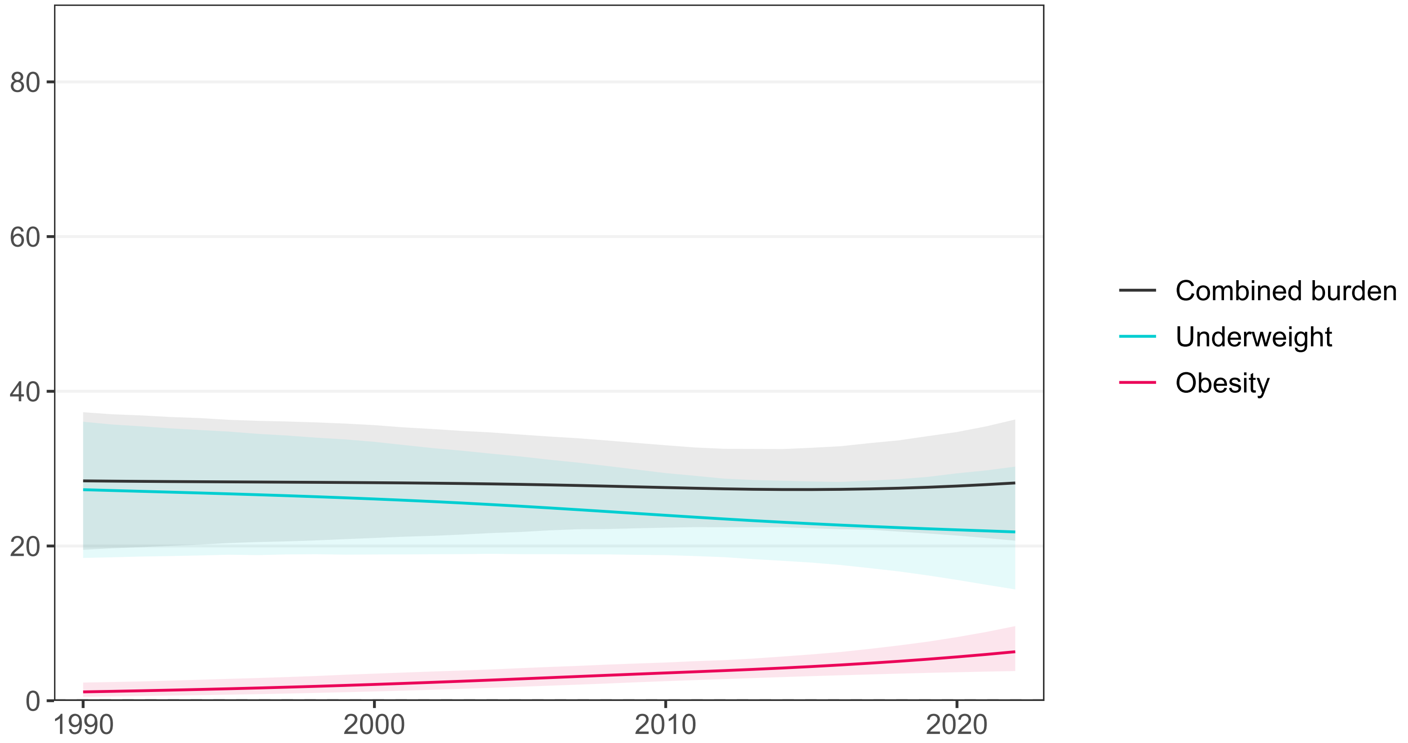

# Chad

## School-aged children and adolescents

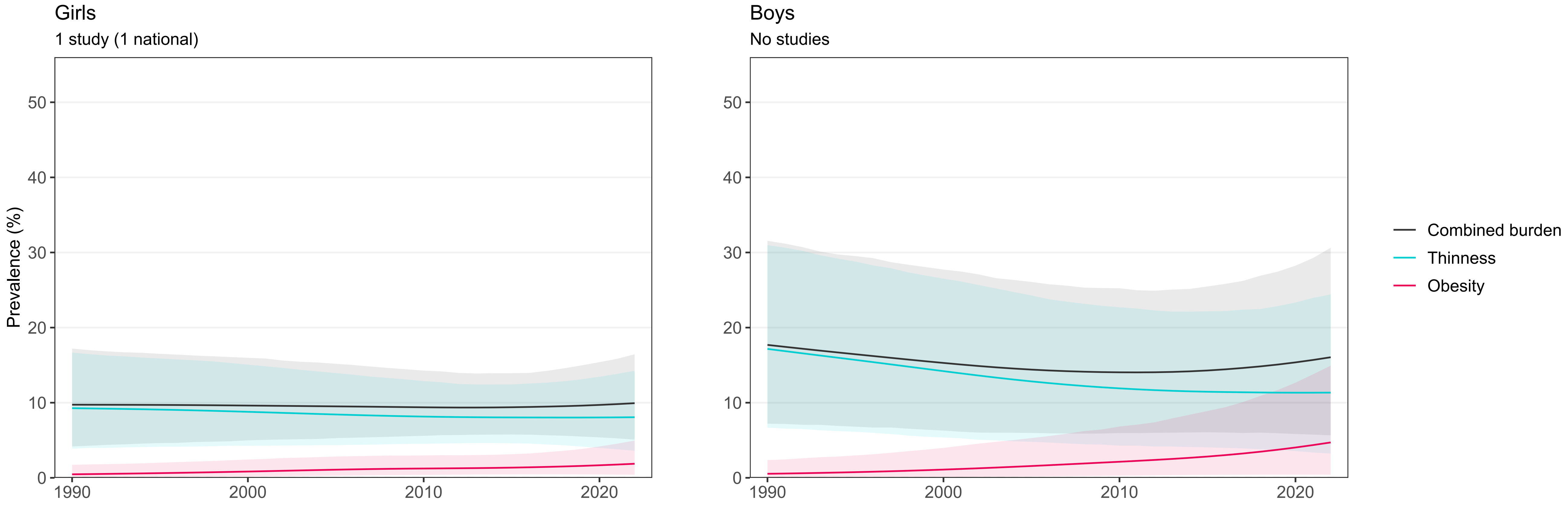


## Adults

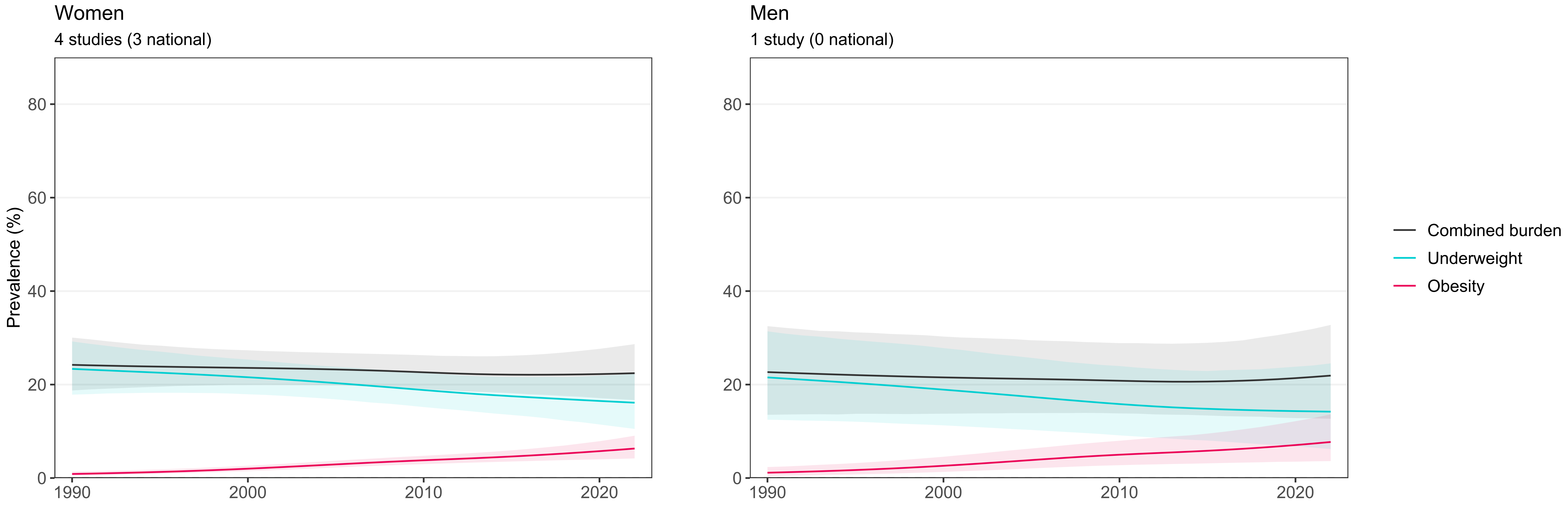

# Chile

## School-aged children and adolescents

### Girls
8 studies (6 national)

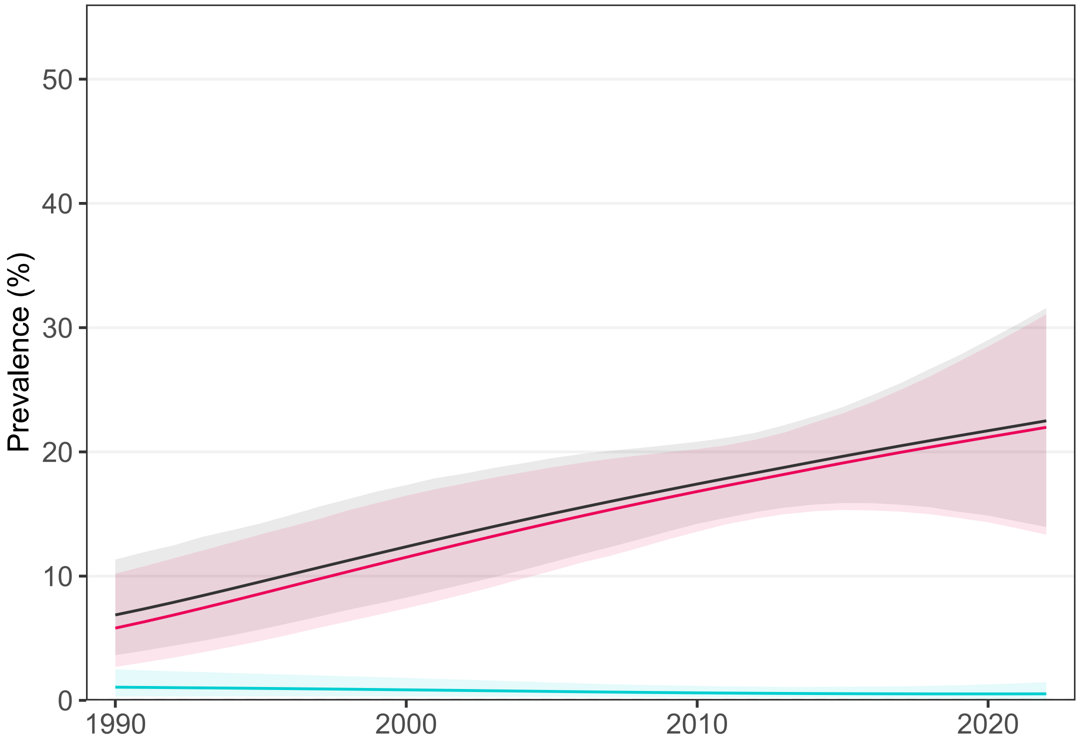


### Boys
8 studies (6 national)

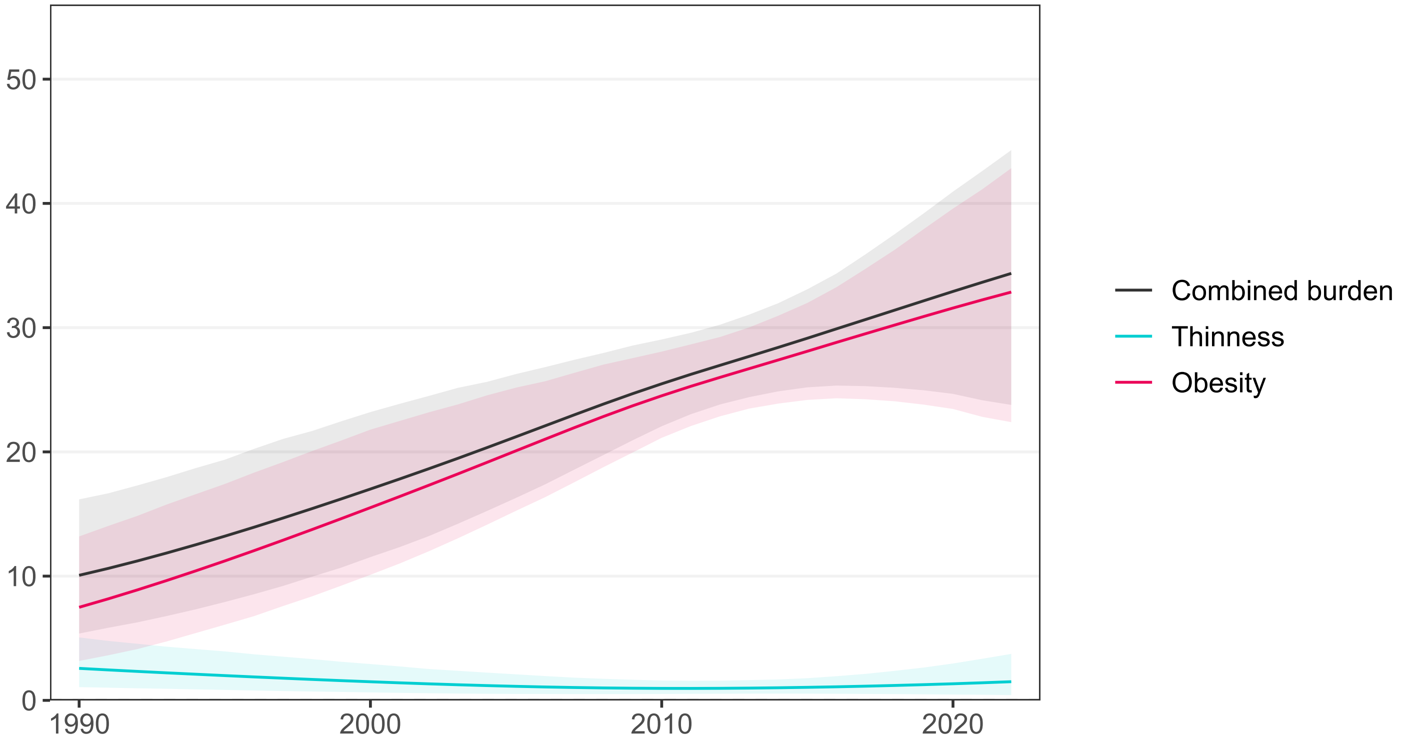


## Adults

### Women
13 studies (5 national)

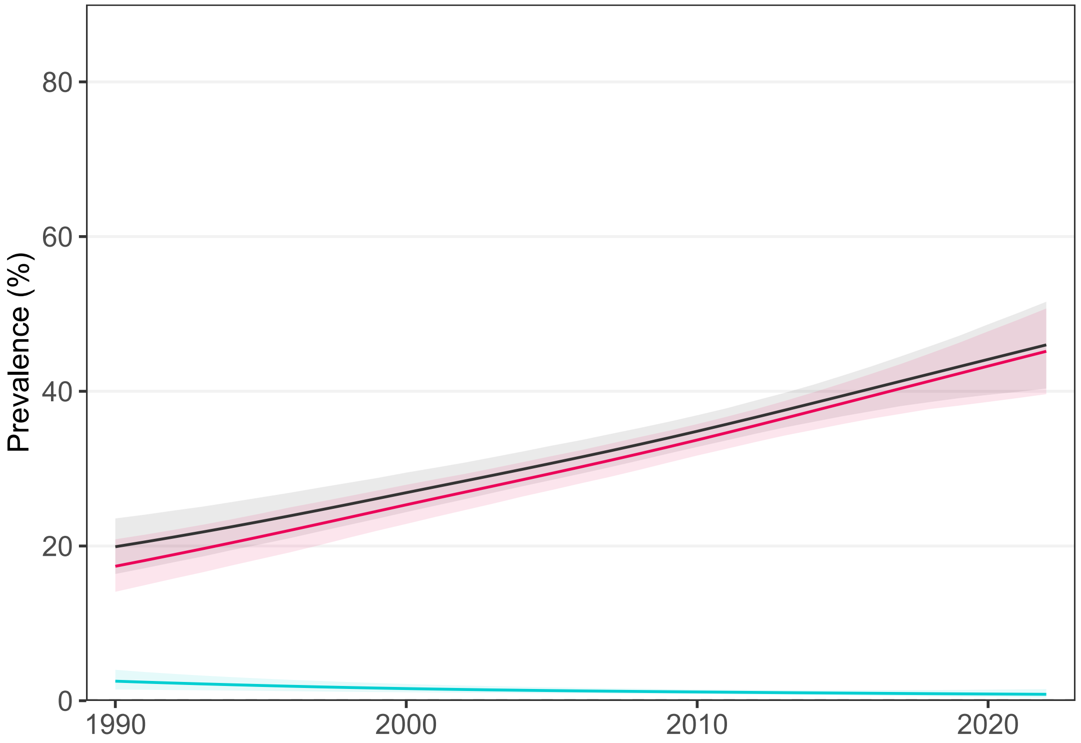


### Men
14 studies (5 national)

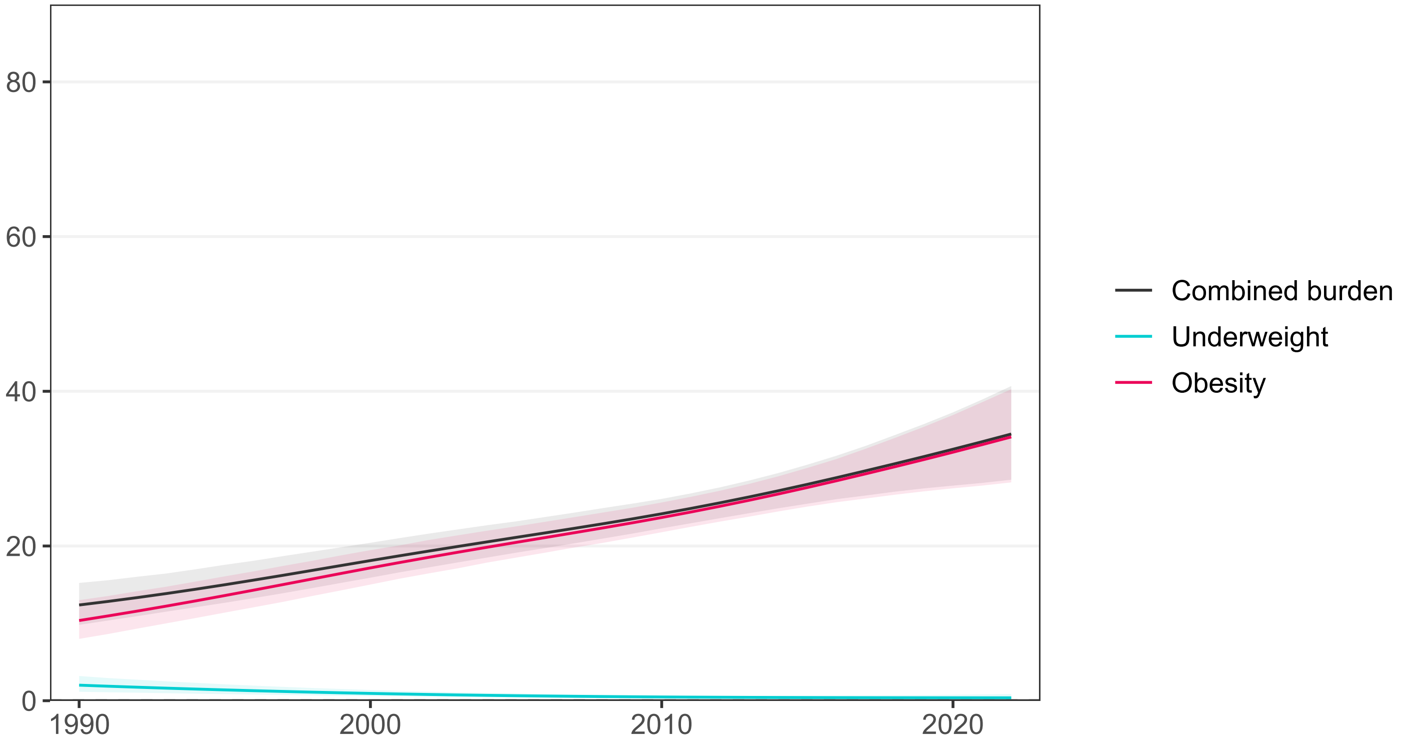

# China

## School-aged children and adolescents

### Girls

50 studies (27 national)

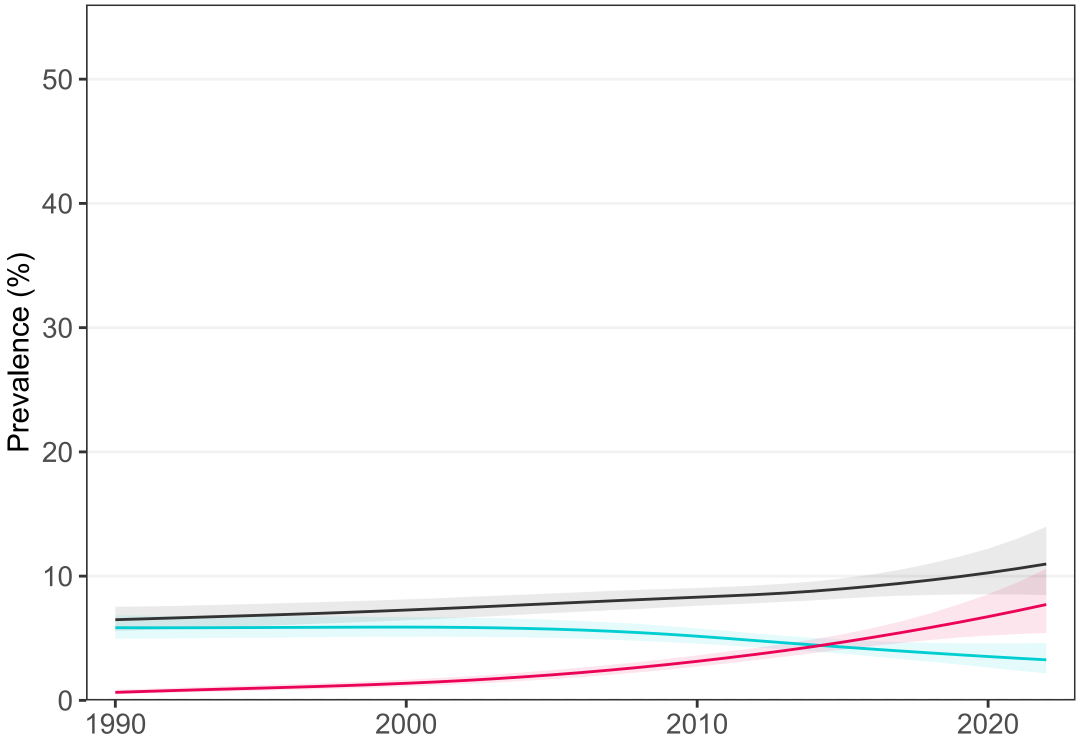


### Boys

51 studies (27 national)

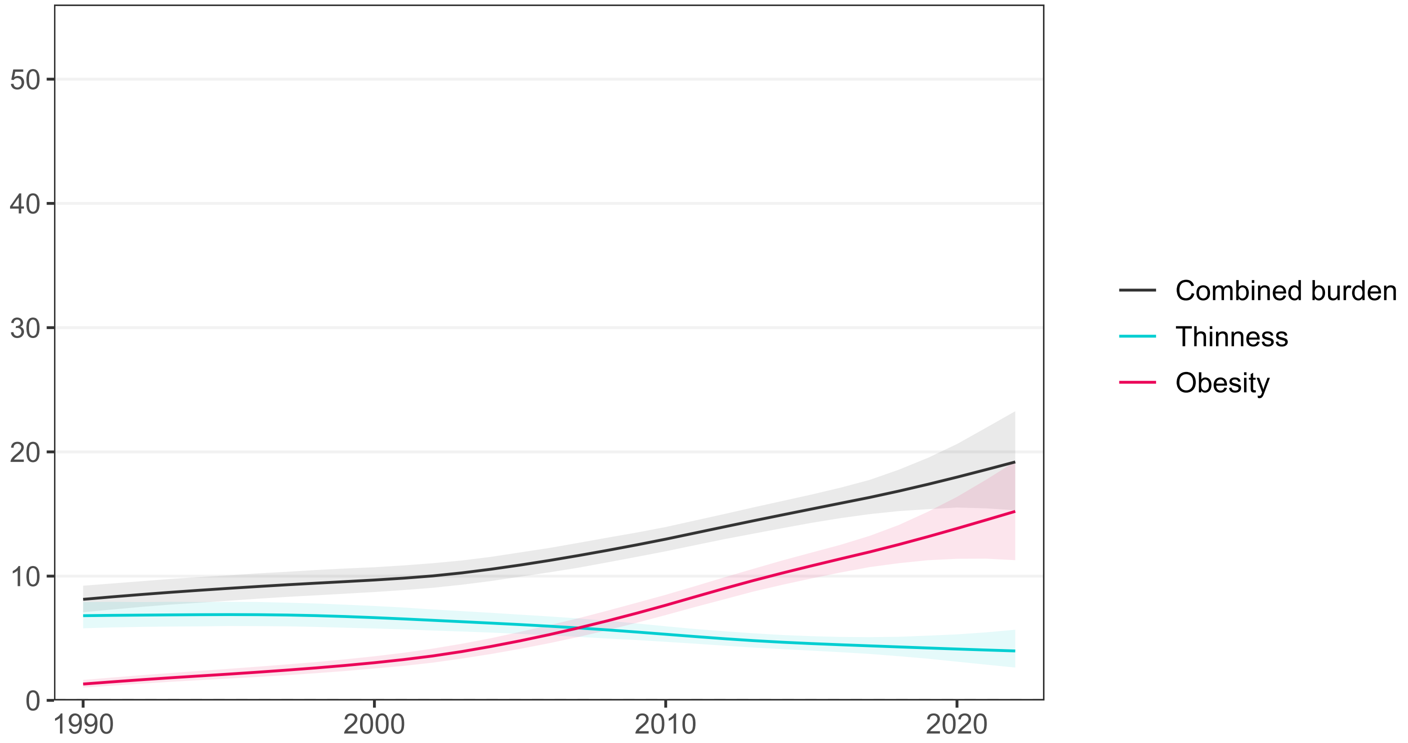


## Adults

### Women

130 studies (47 national)

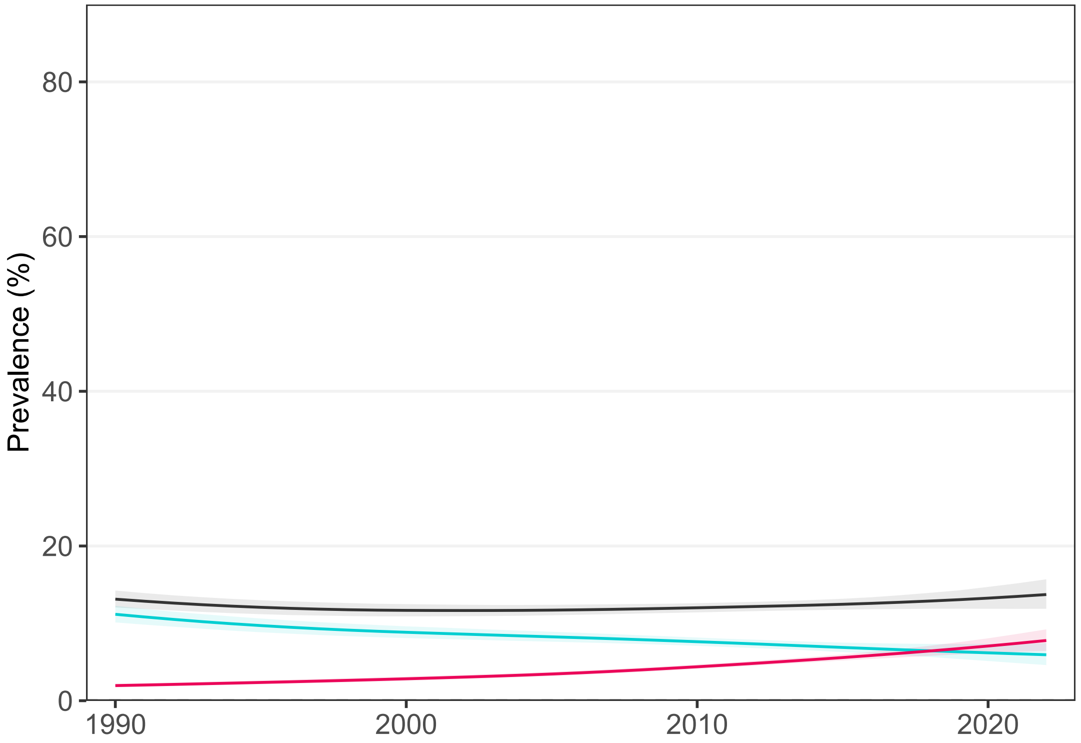


### Men

134 studies (49 national)

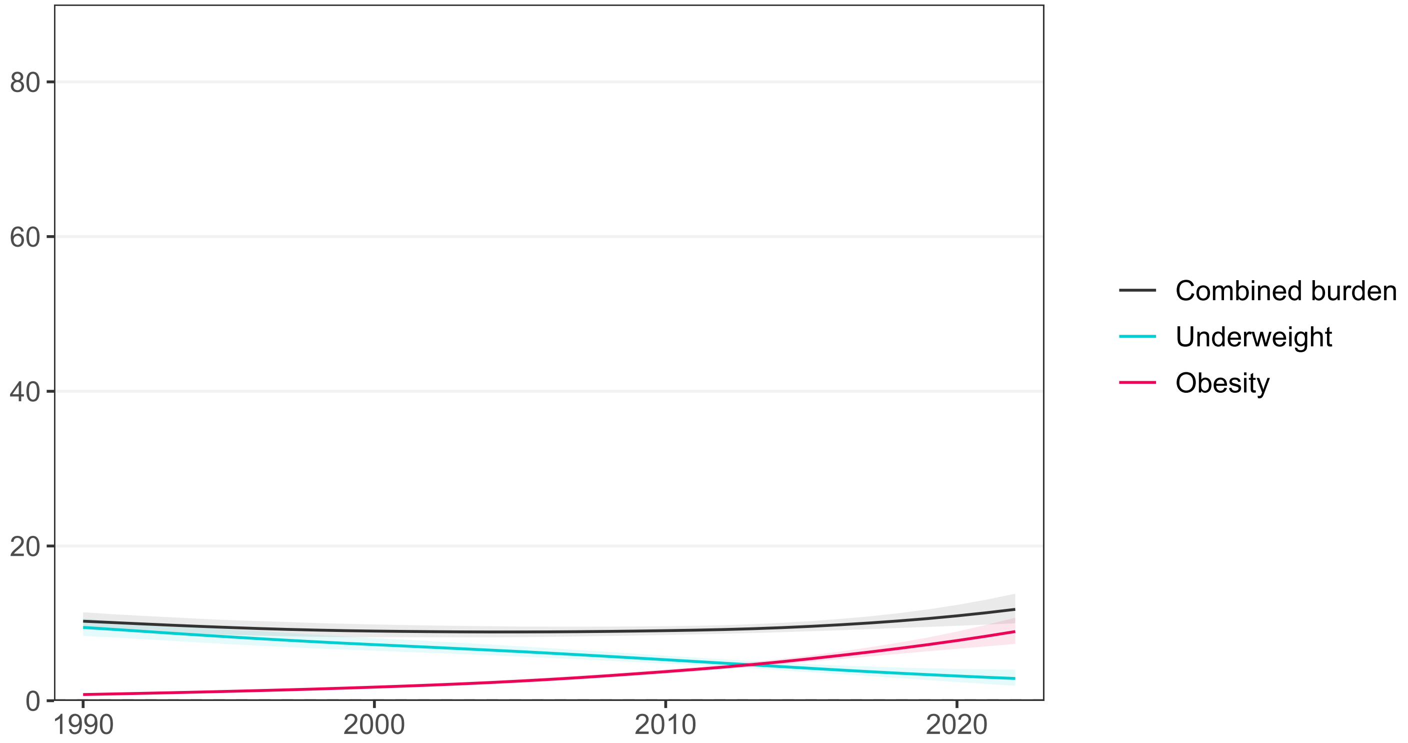

# Colombia

## School-aged children and adolescents

### Girls

14 studies (6 national)

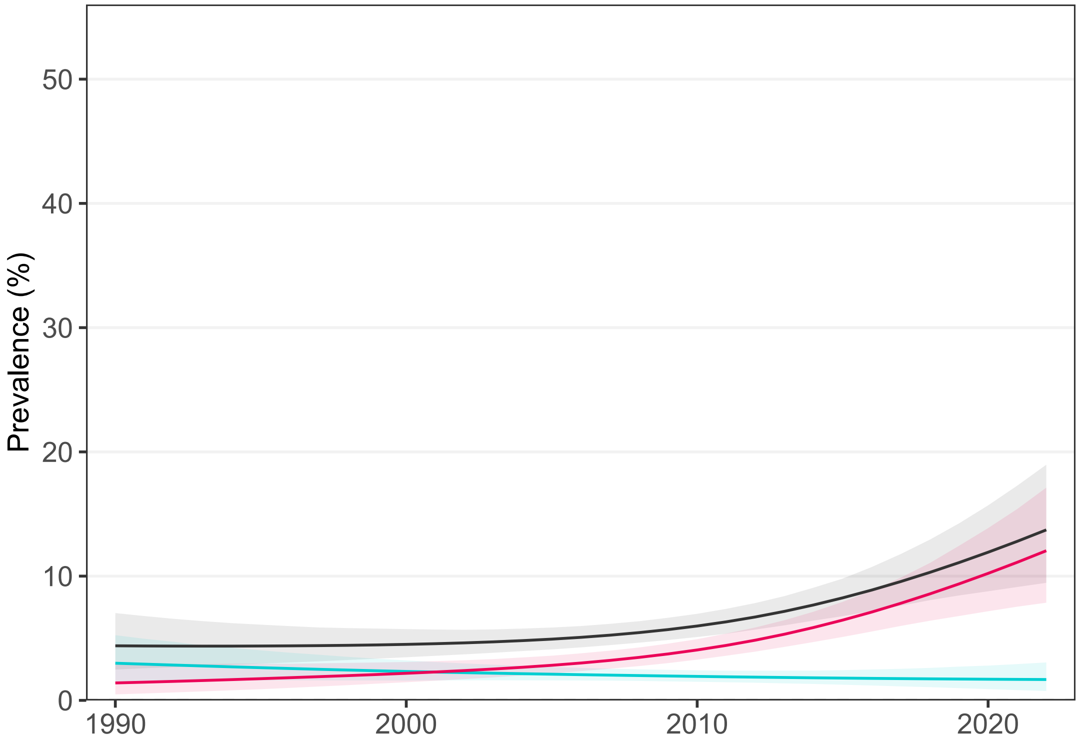


### Boys

14 studies (6 national)

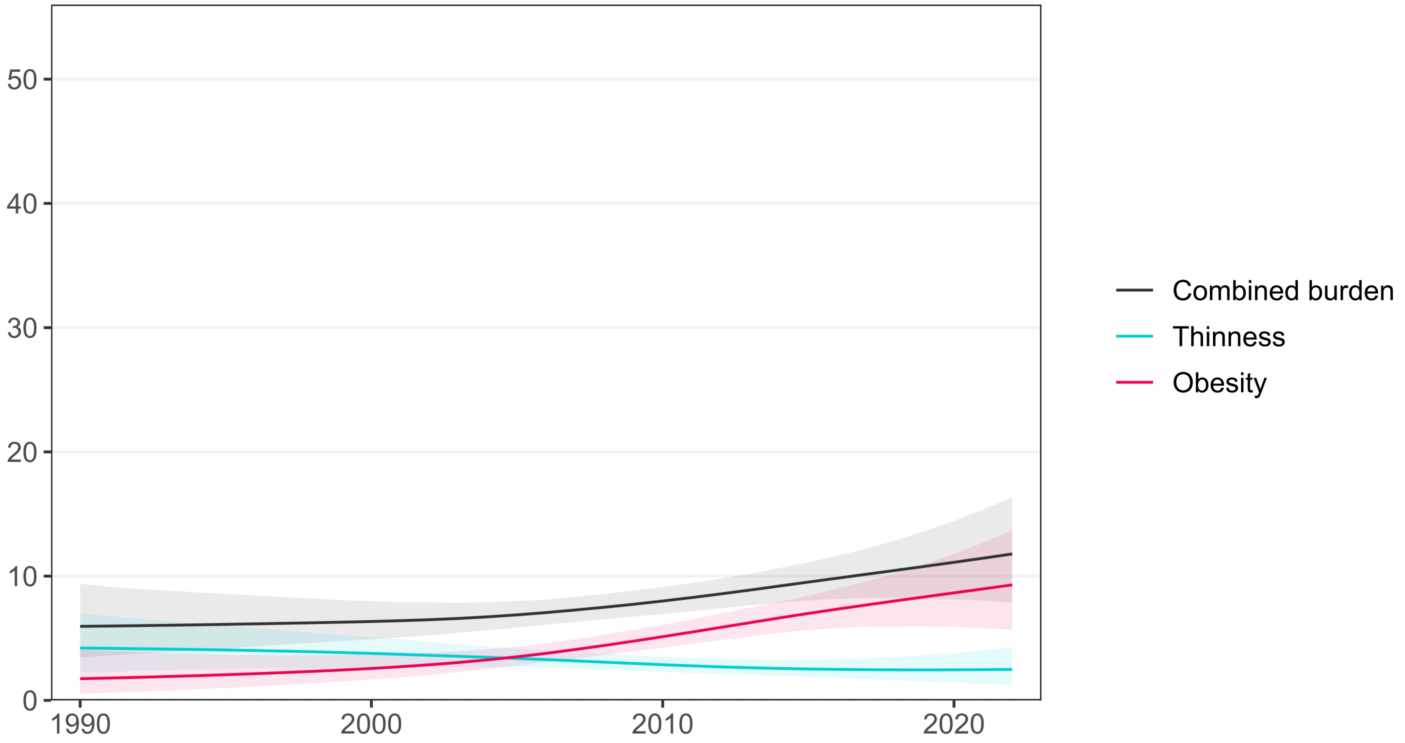


## Adults

### Women

19 studies (9 national)

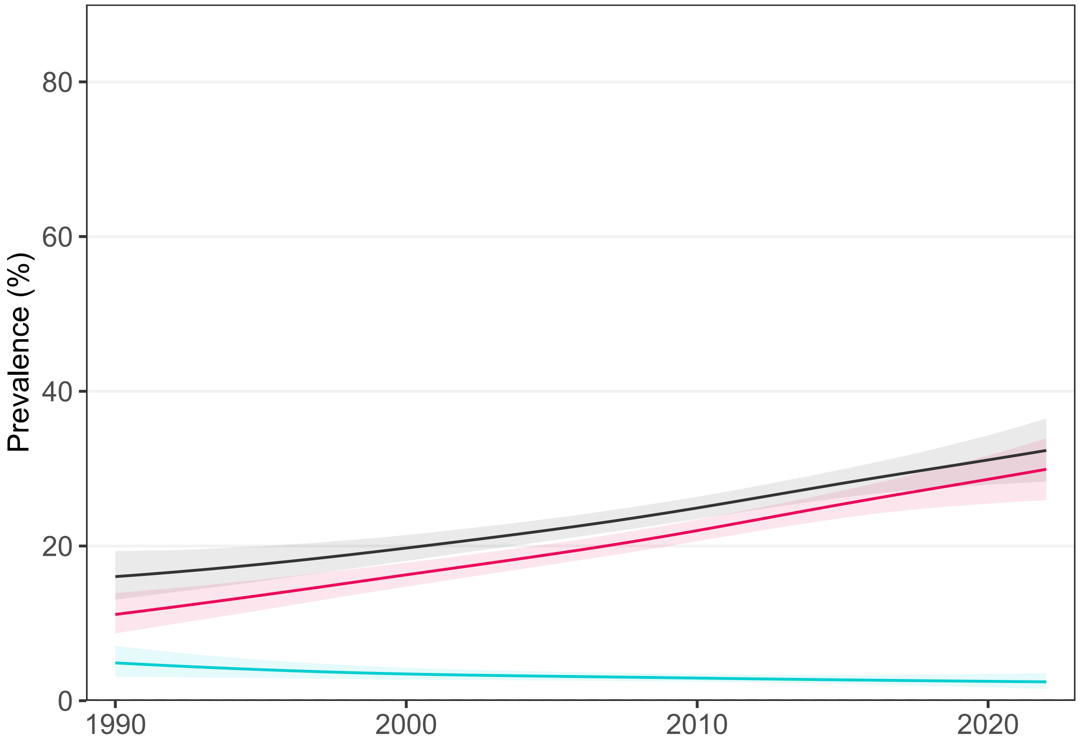


### Men

16 studies (6 national)

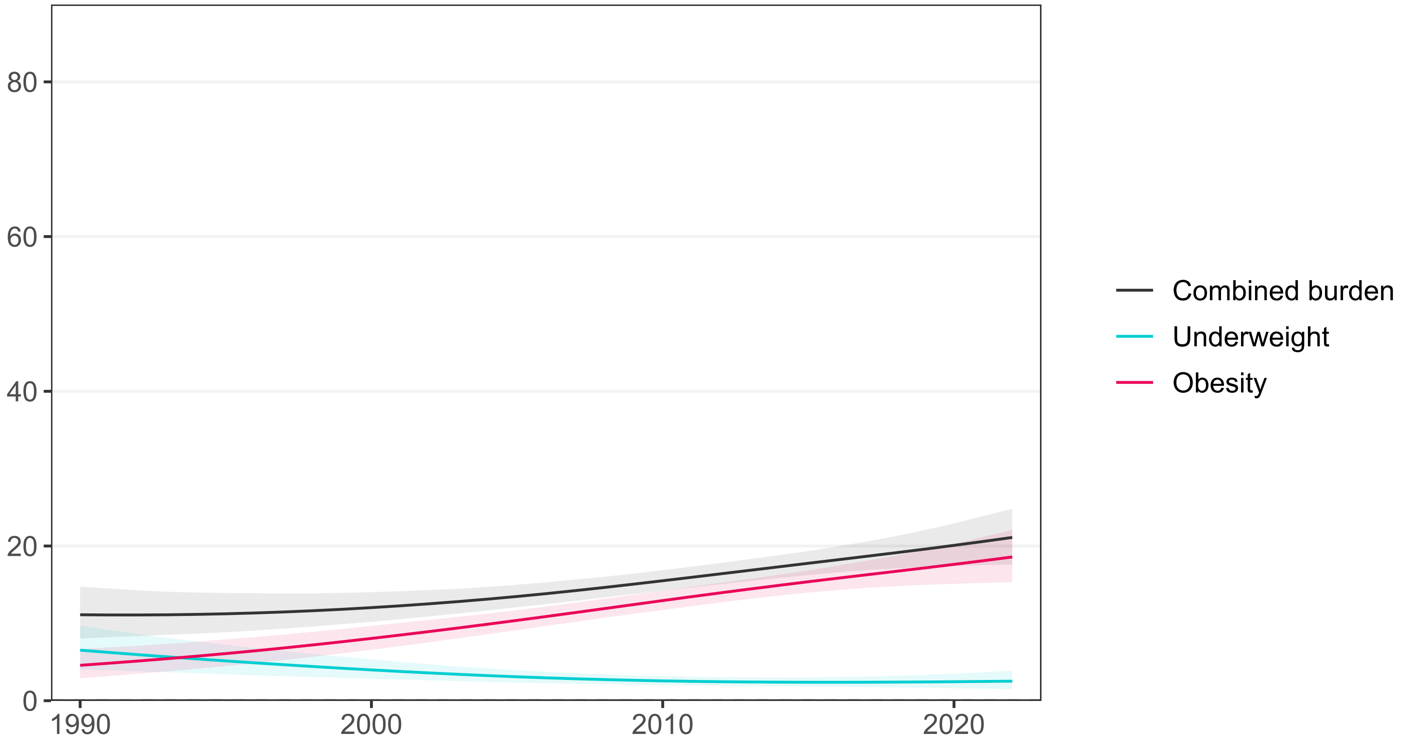

# Comoros

## School-aged children and adolescents

### Girls
1 study (1 national)

### Boys
No studies

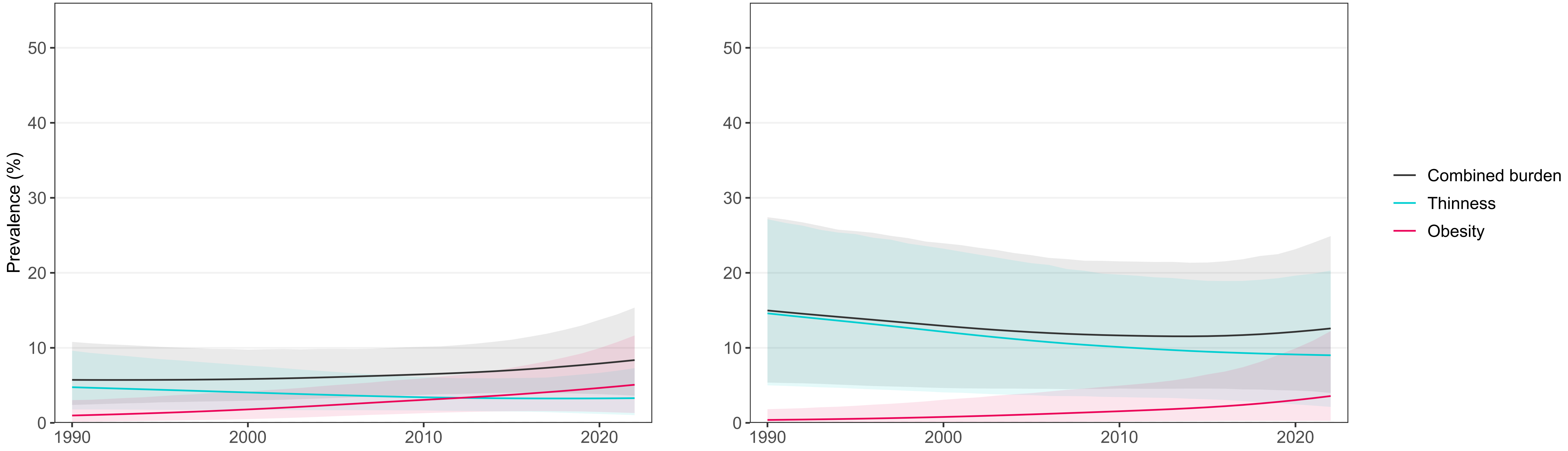


## Adults

### Women
3 studies (3 national)

### Men
1 study (1 national)

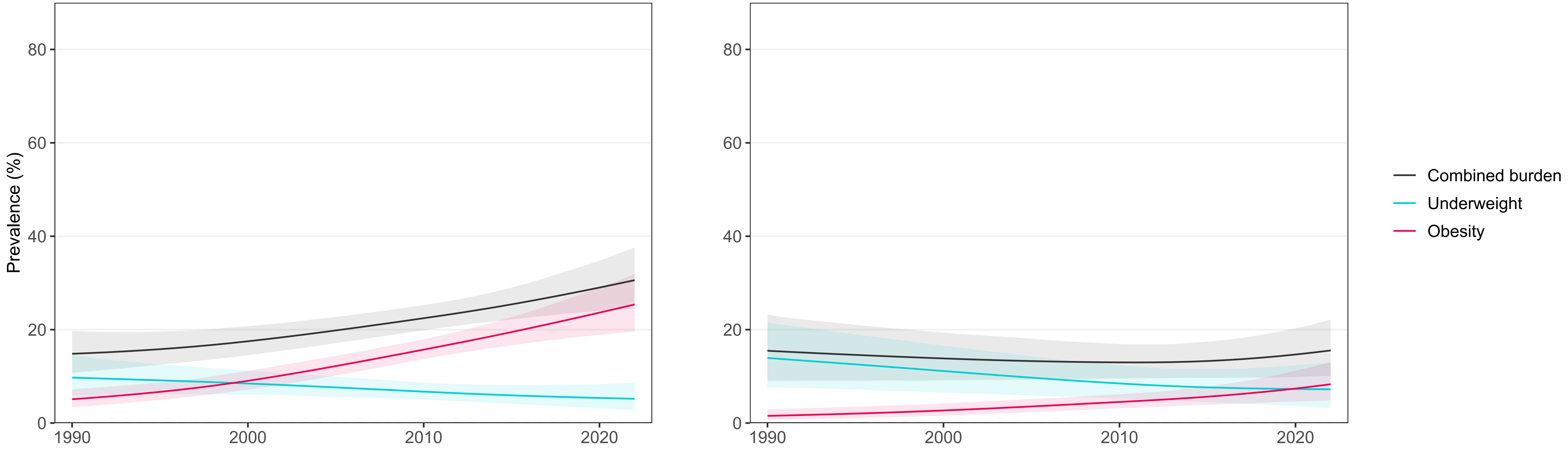

# Congo

## School-aged children and adolescents

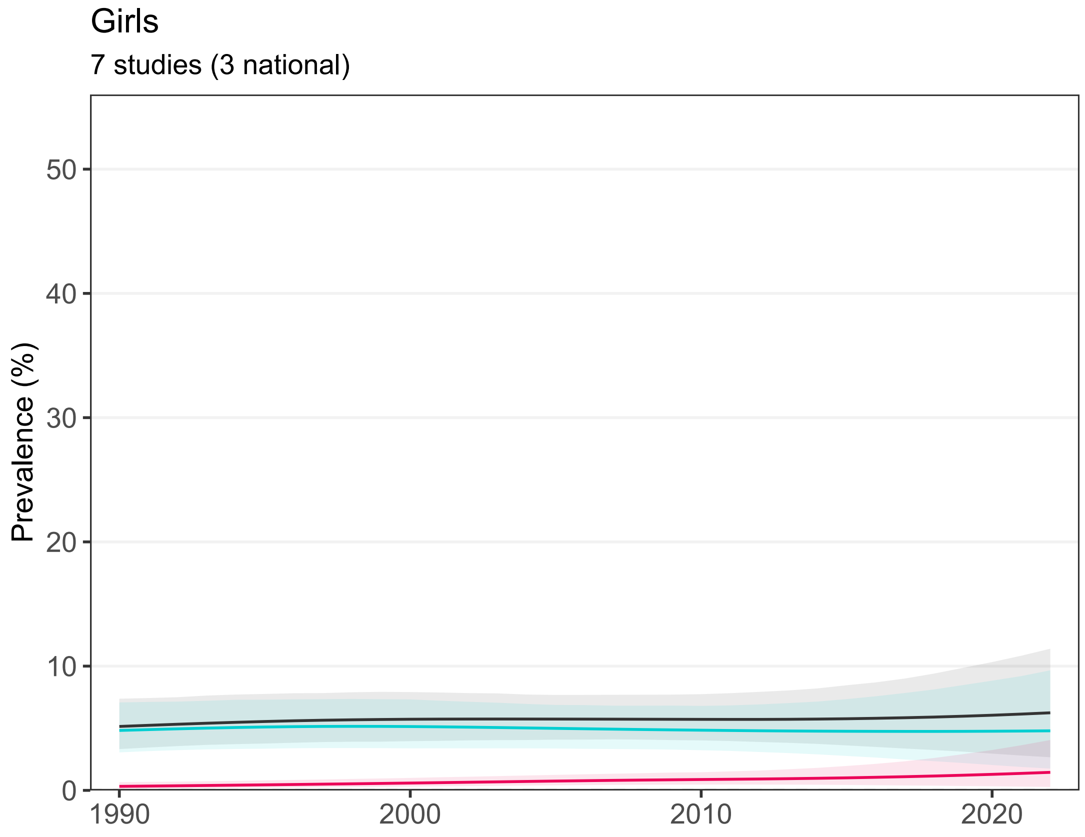


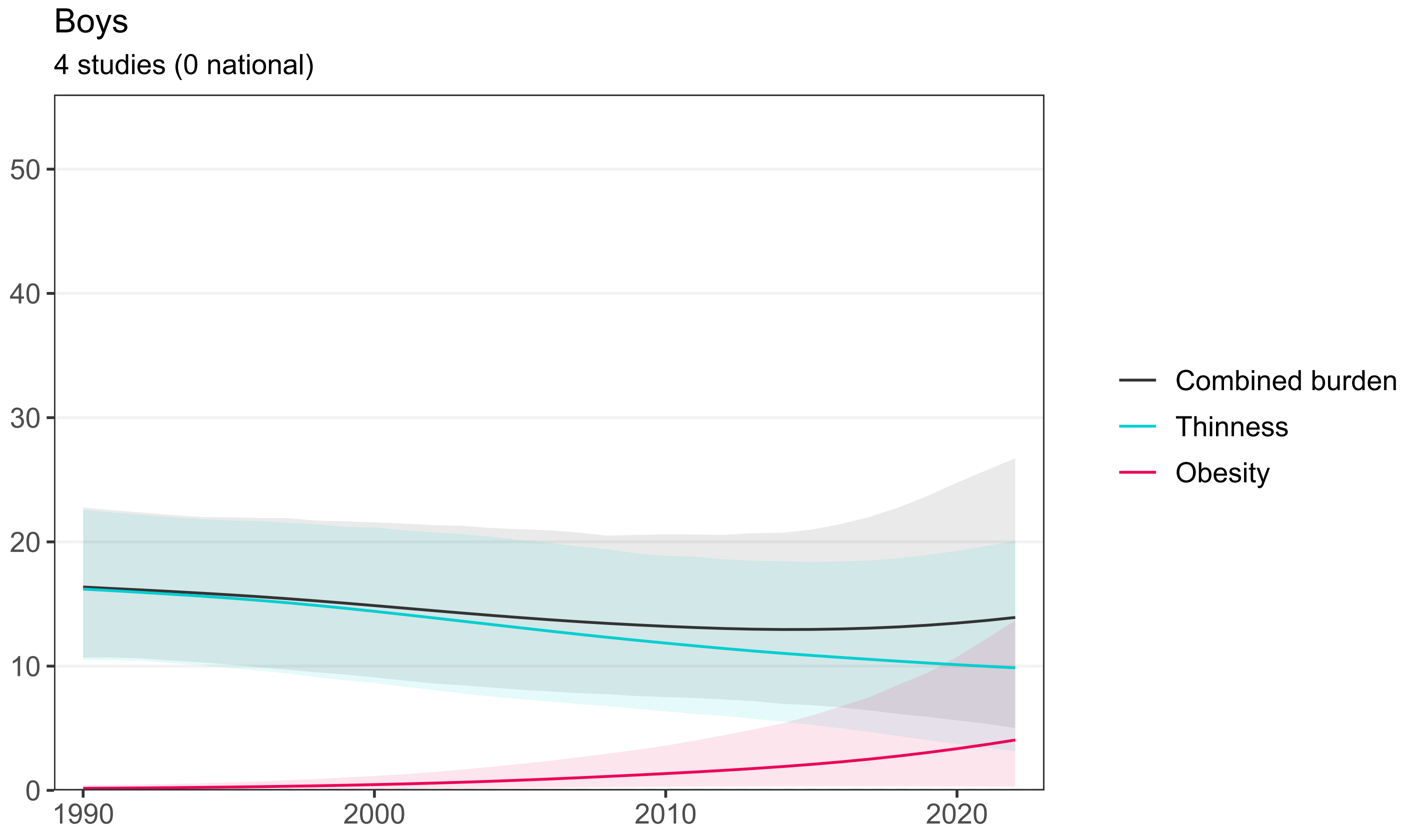


## Adults

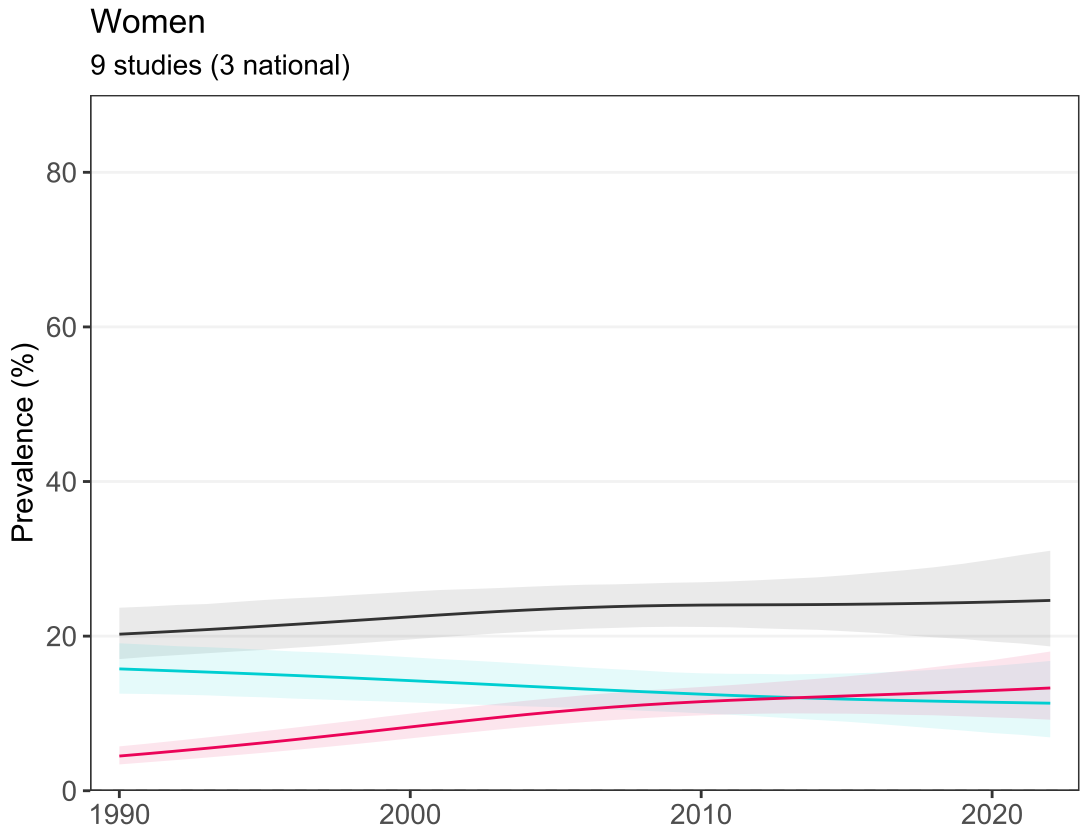


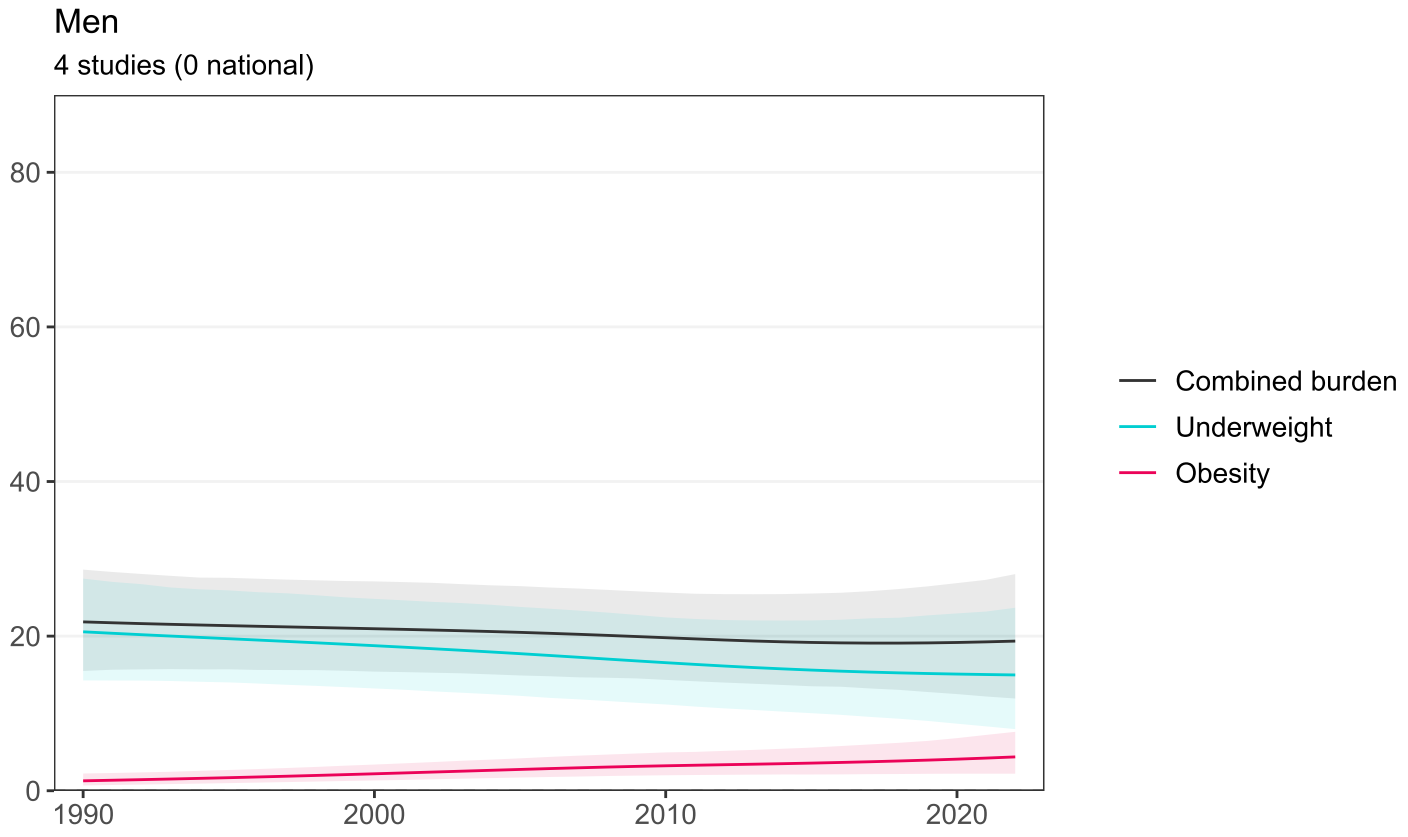

# Cook Islands

## School-aged children and adolescents

Girls
3 studies (3 national)

Boys
3 studies (3 national)

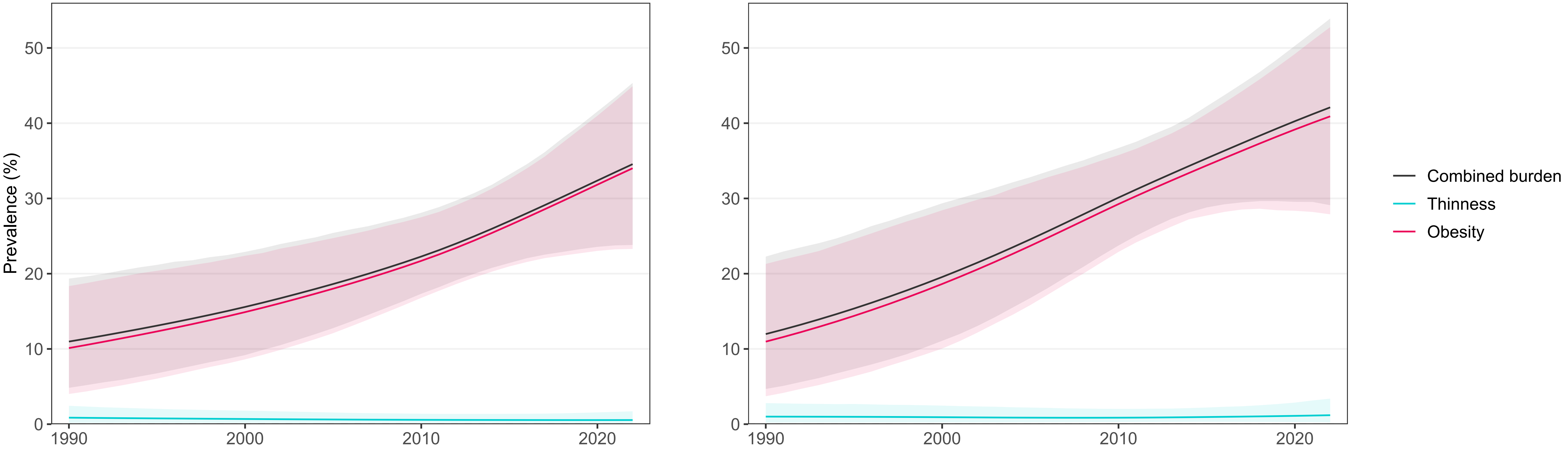


## Adults

Women
2 studies (2 national)

Men
2 studies (2 national)

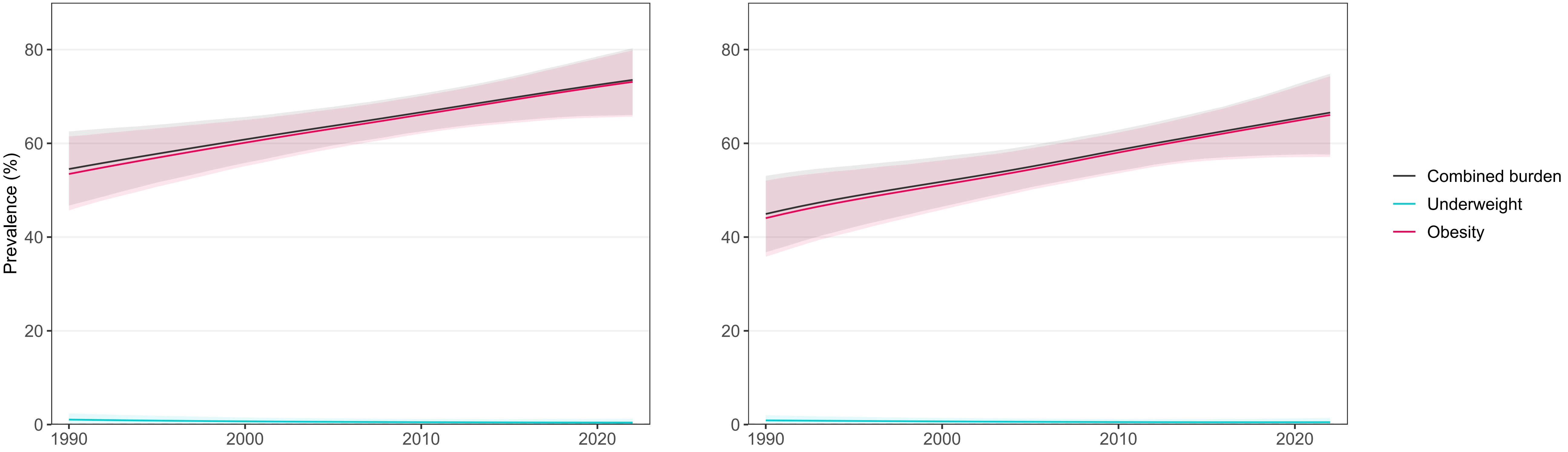

# Costa Rica

## School-aged children and adolescents

### Girls

3 studies (3 national)

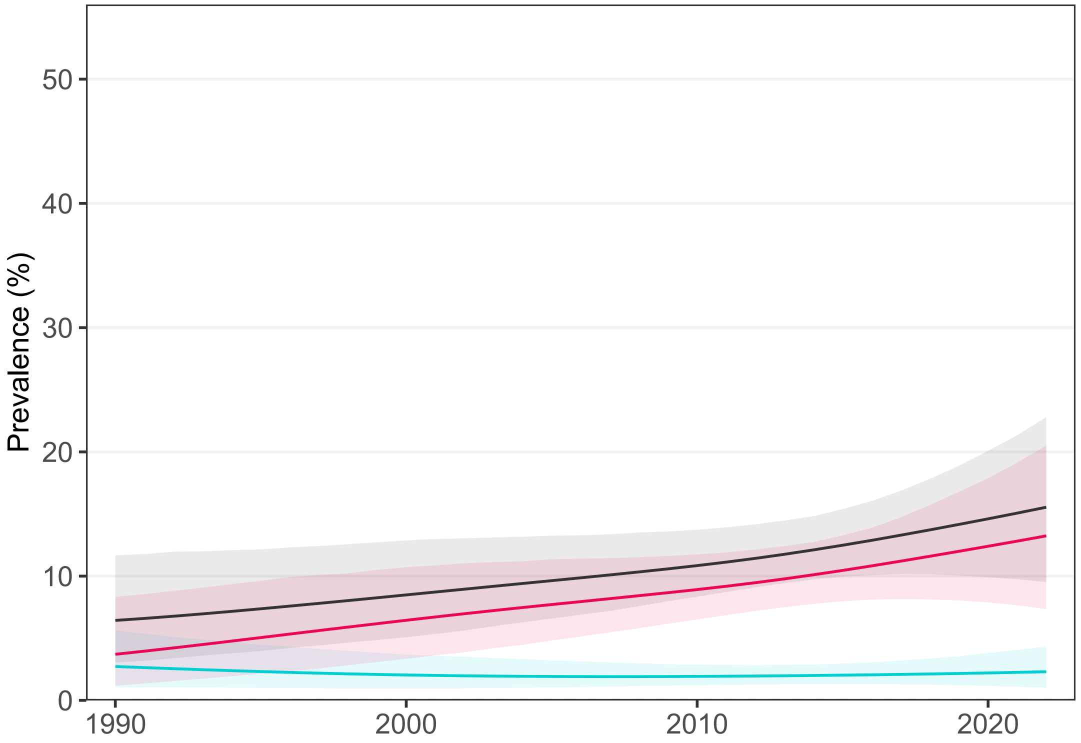


### Boys

3 studies (3 national)

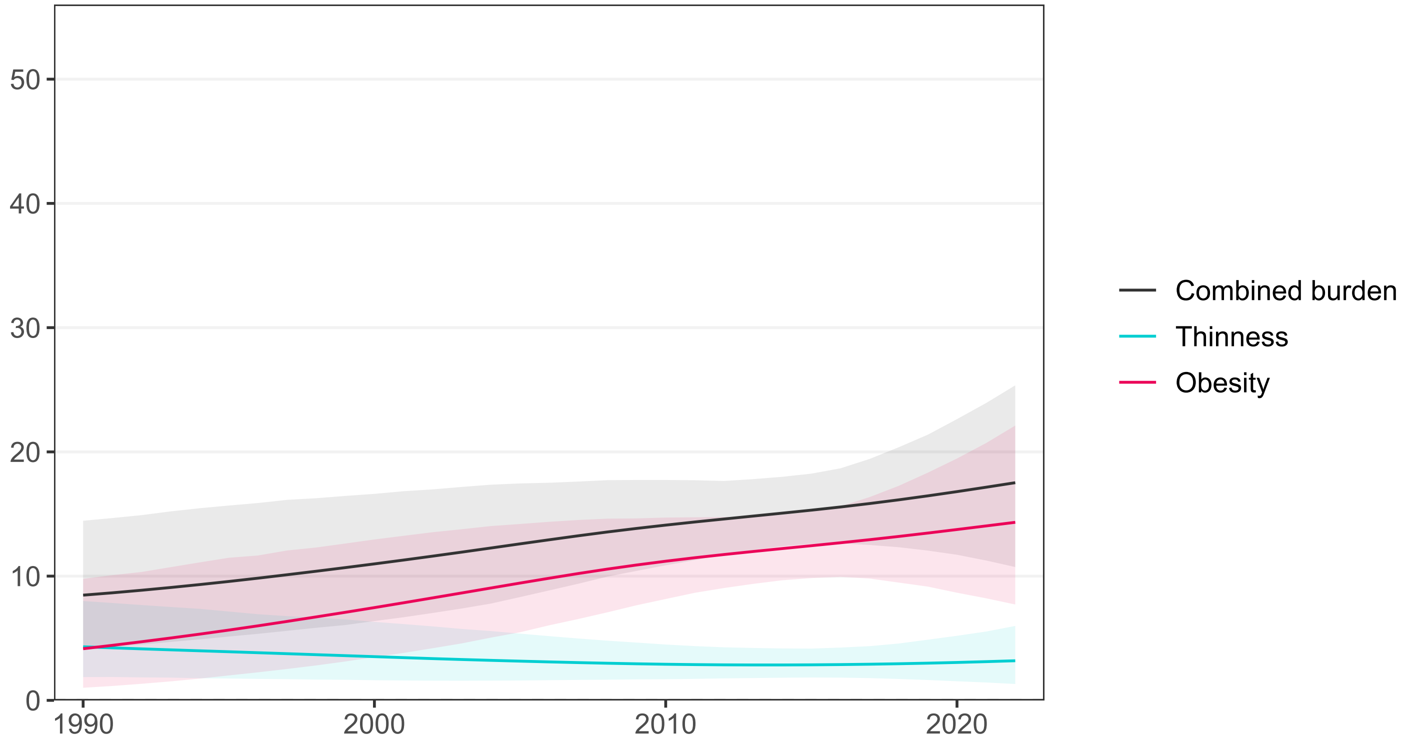


## Adults

### Women

10 studies (9 national)

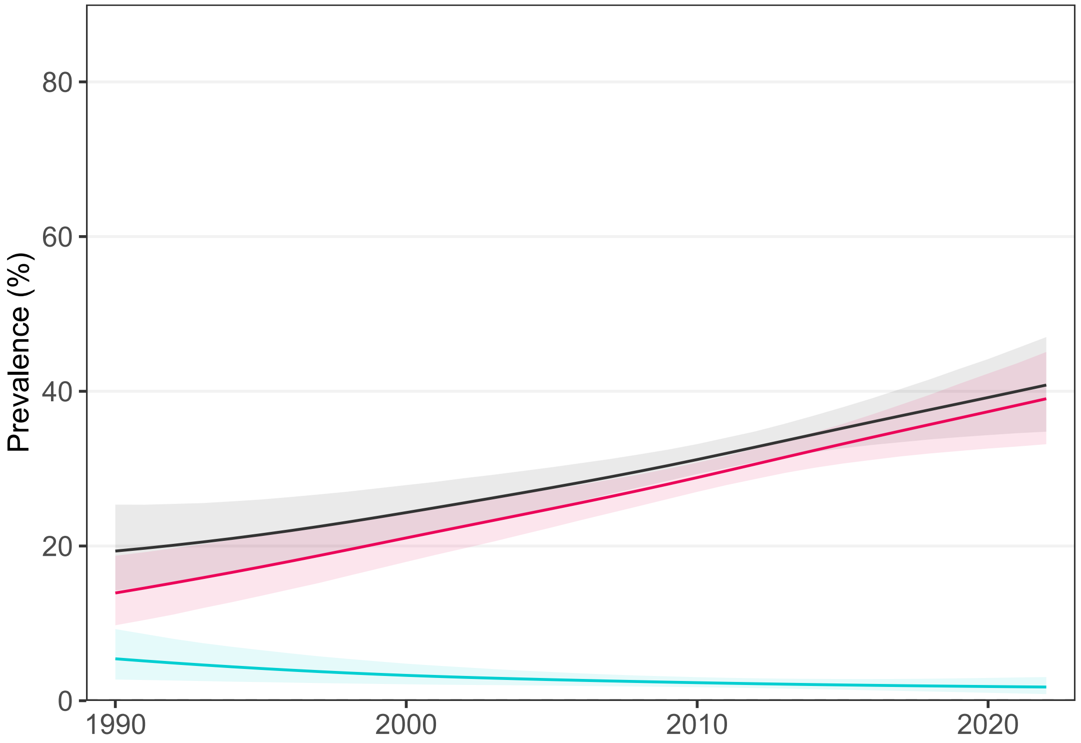


### Men

9 studies (8 national)

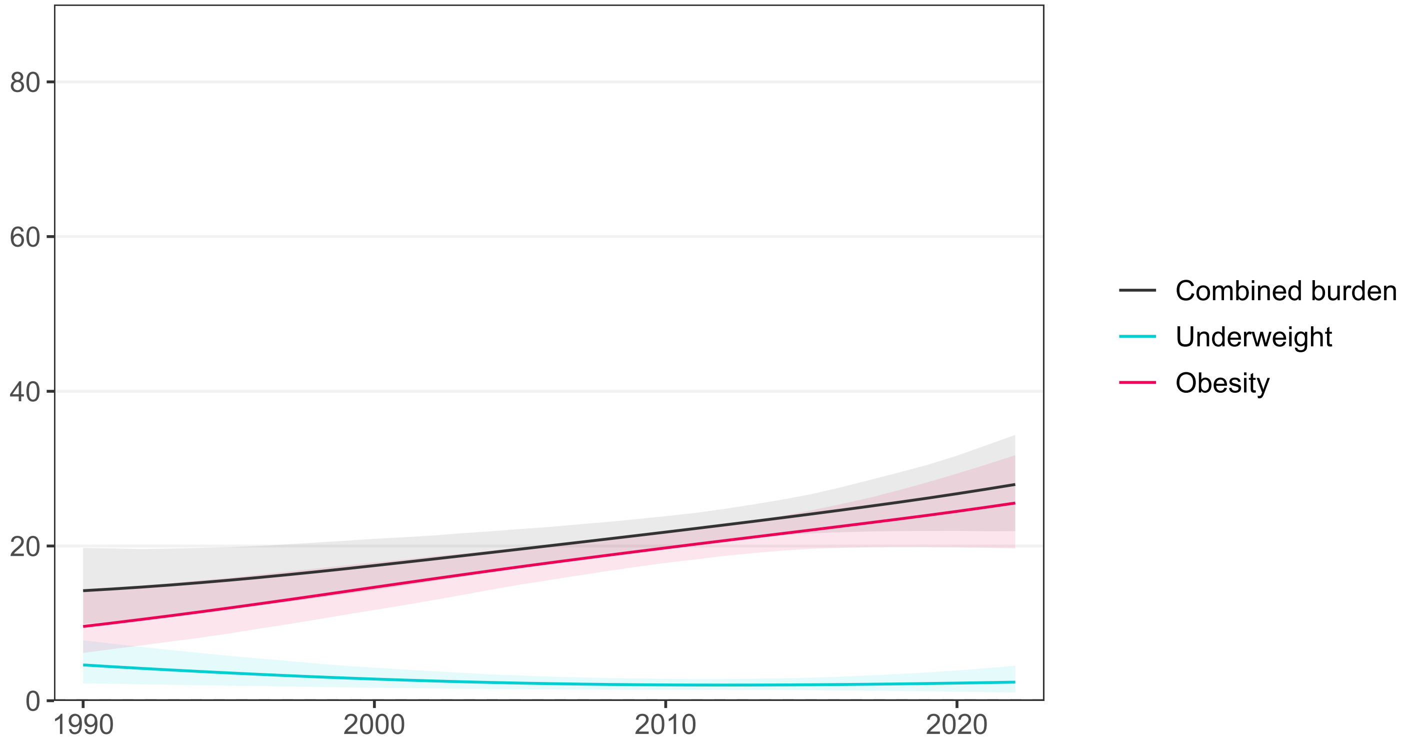

# Cote d'Ivoire

## School-aged children and adolescents

### Girls
6 studies (3 national)

### Boys
3 studies (0 national)

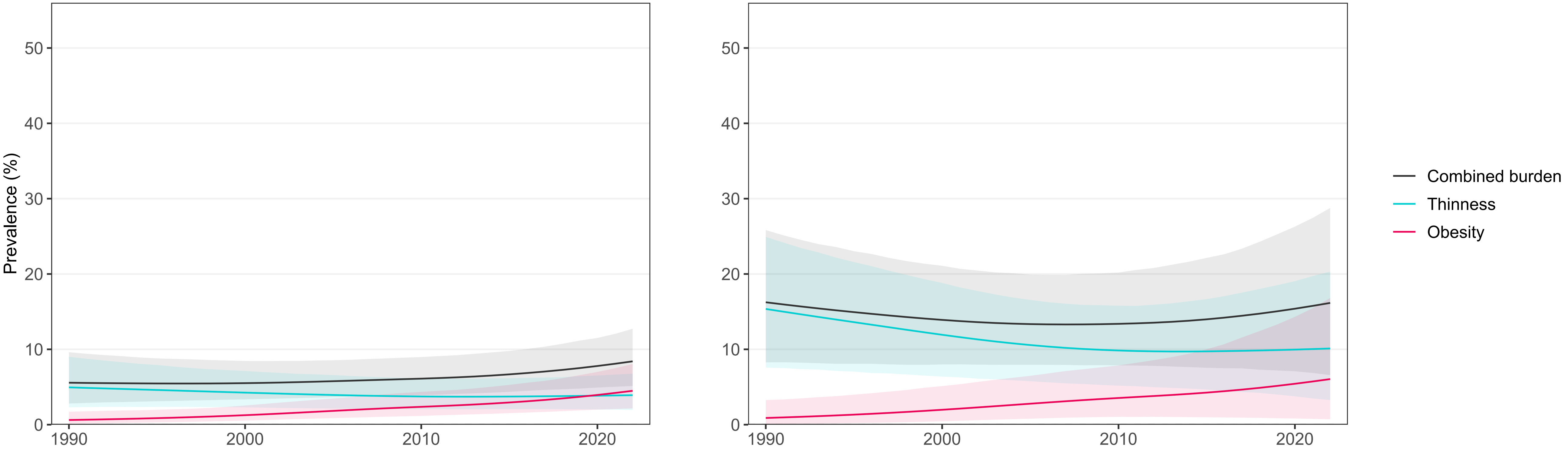


## Adults

### Women
7 studies (4 national)

### Men
3 studies (0 national)

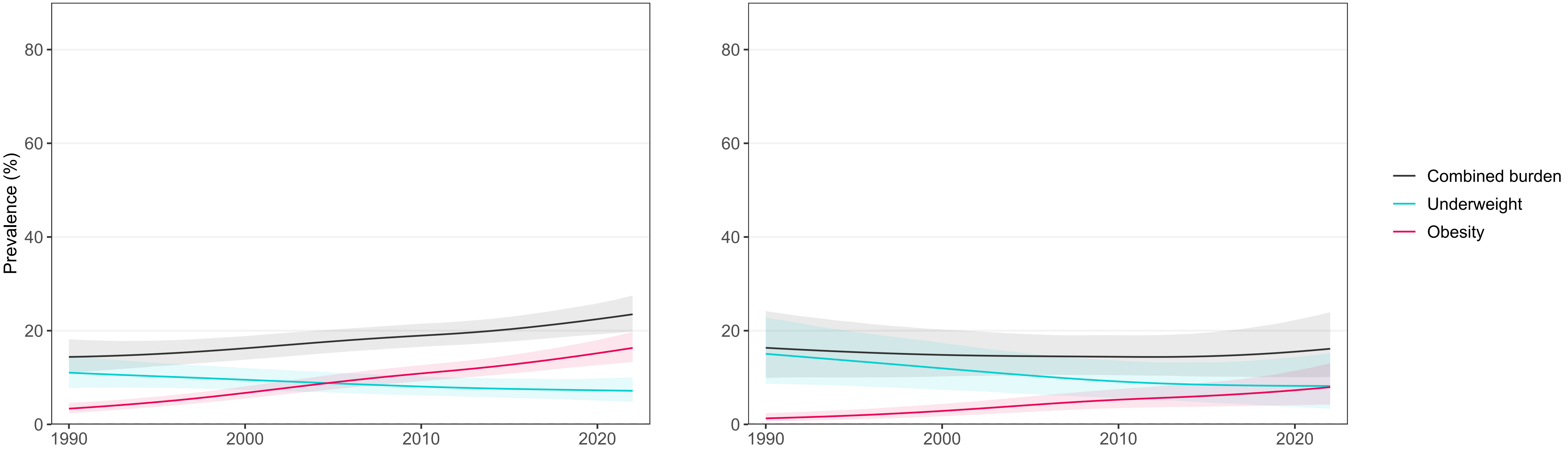

# Croatia

## School-aged children and adolescents

### Girls
11 studies (6 national)

### Boys
10 studies (6 national)

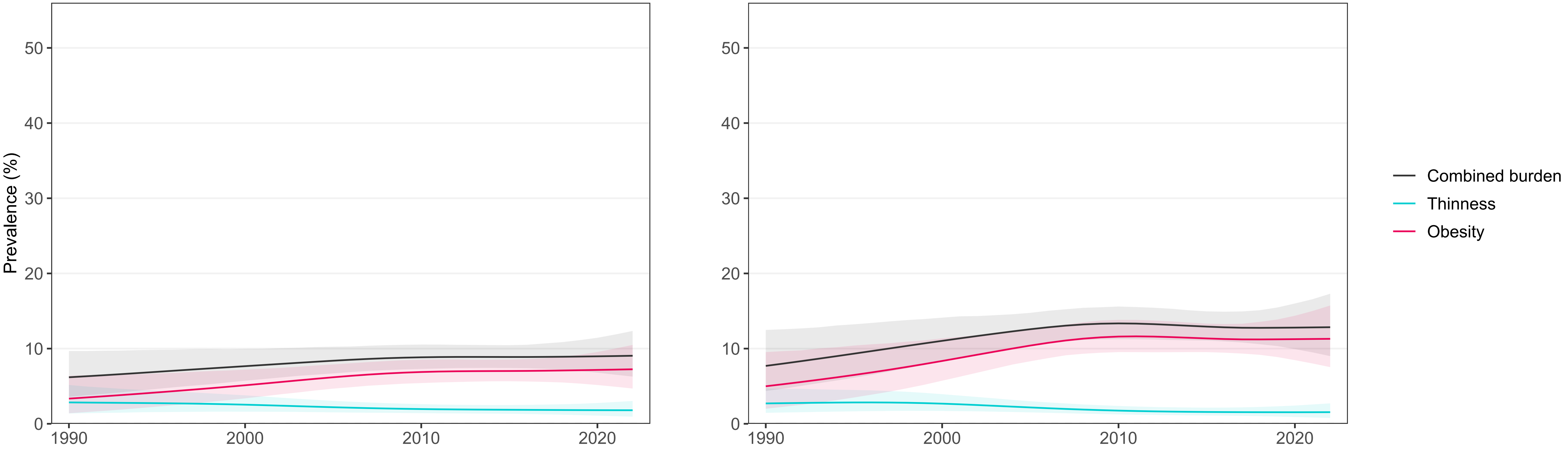


## Adults

### Women
9 studies (4 national)

### Men
9 studies (4 national)

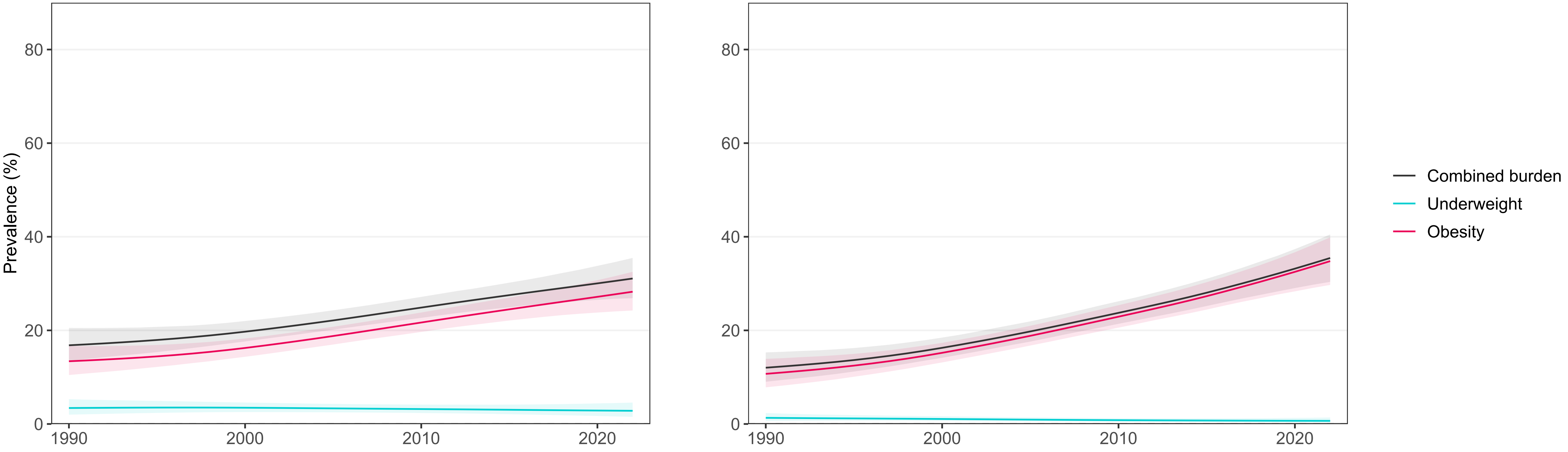

# Cuba

## School-aged children and adolescents

Girls
6 studies (3 national)

Boys
6 studies (3 national)

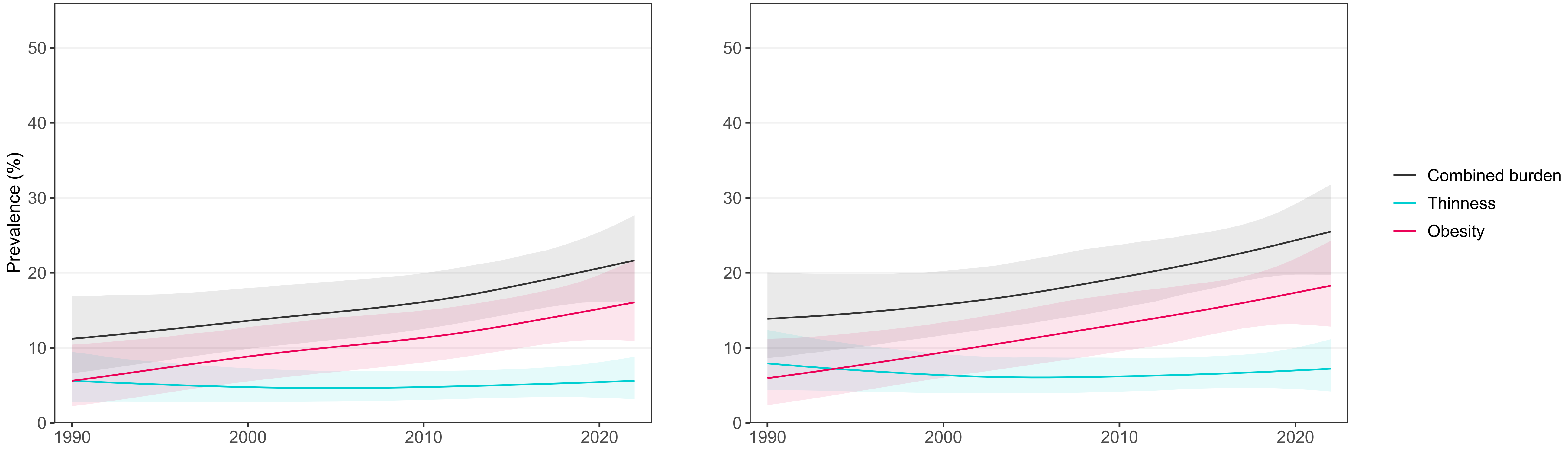


## Adults

Women
8 studies (3 national)

Men
8 studies (3 national)

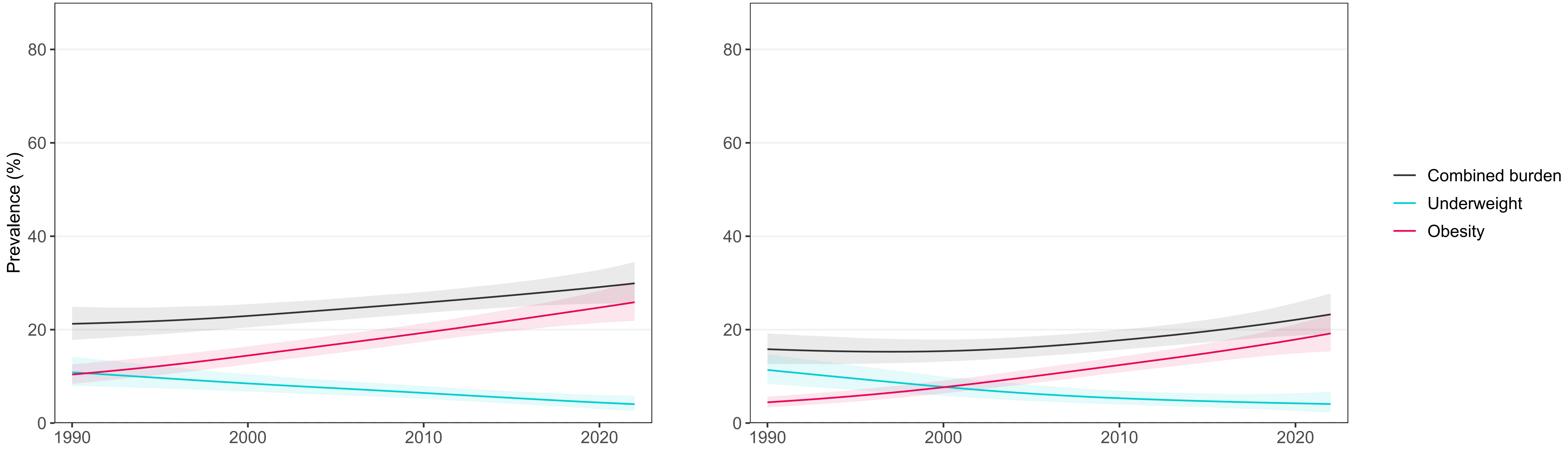

# Cyprus

## School-aged children and adolescents

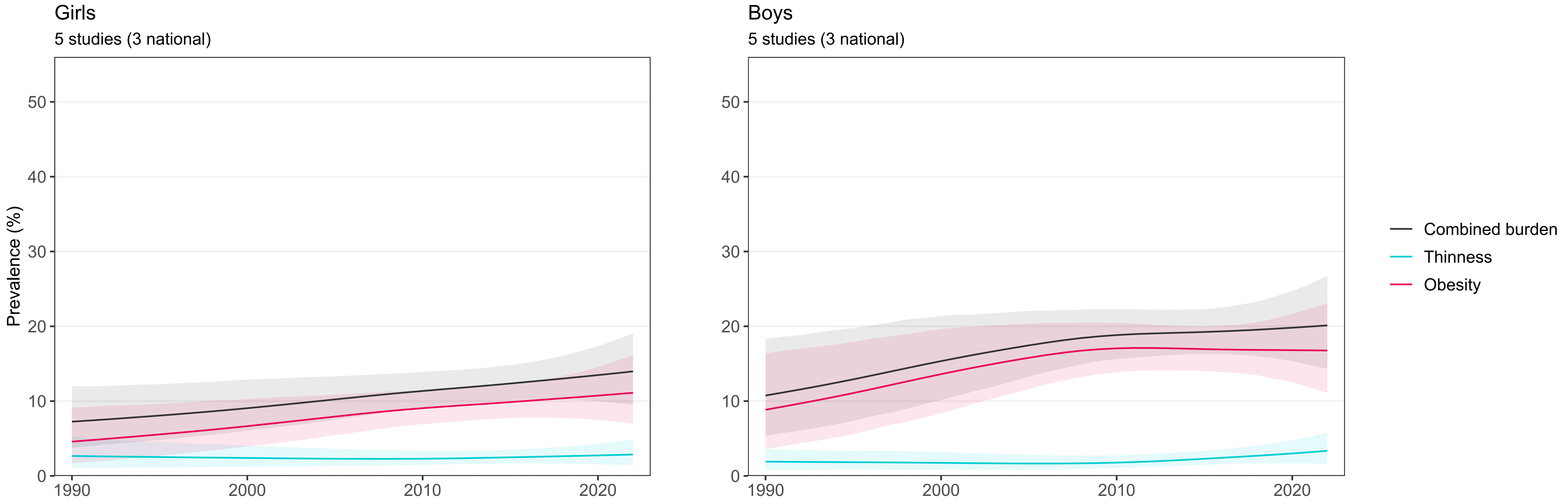


## Adults

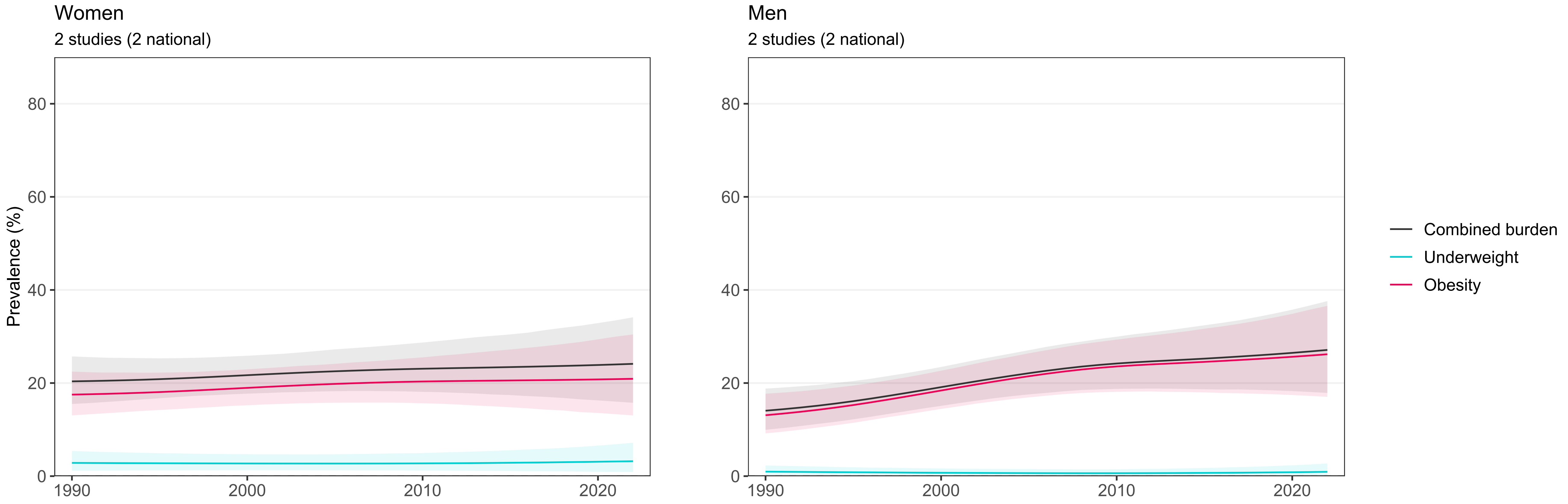

# Czechia

## School-aged children and adolescents

### Girls
12 studies (6 national)

### Boys
12 studies (6 national)

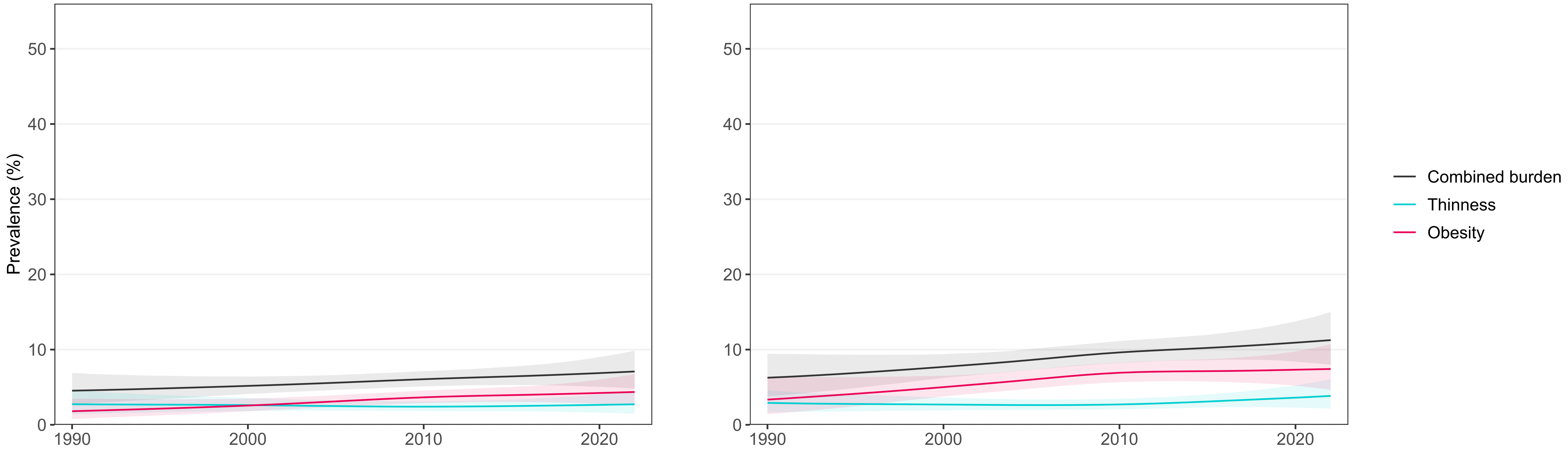


## Adults

### Women
17 studies (13 national)

### Men
17 studies (13 national)

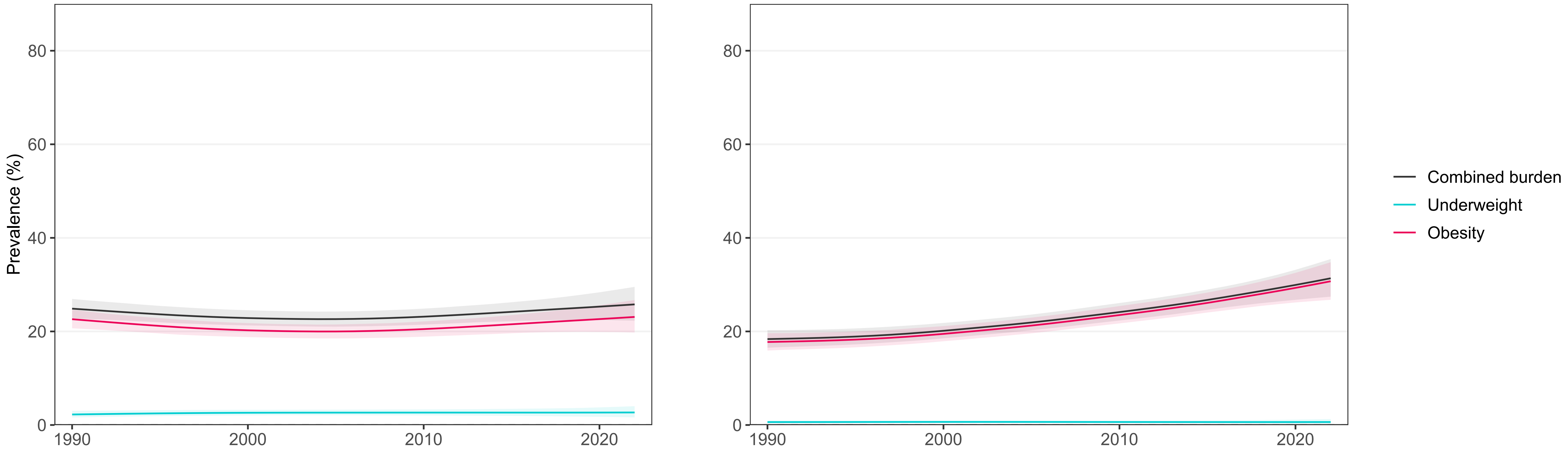

# Denmark

## School-aged children and adolescents

### Girls
46 studies (3 national)

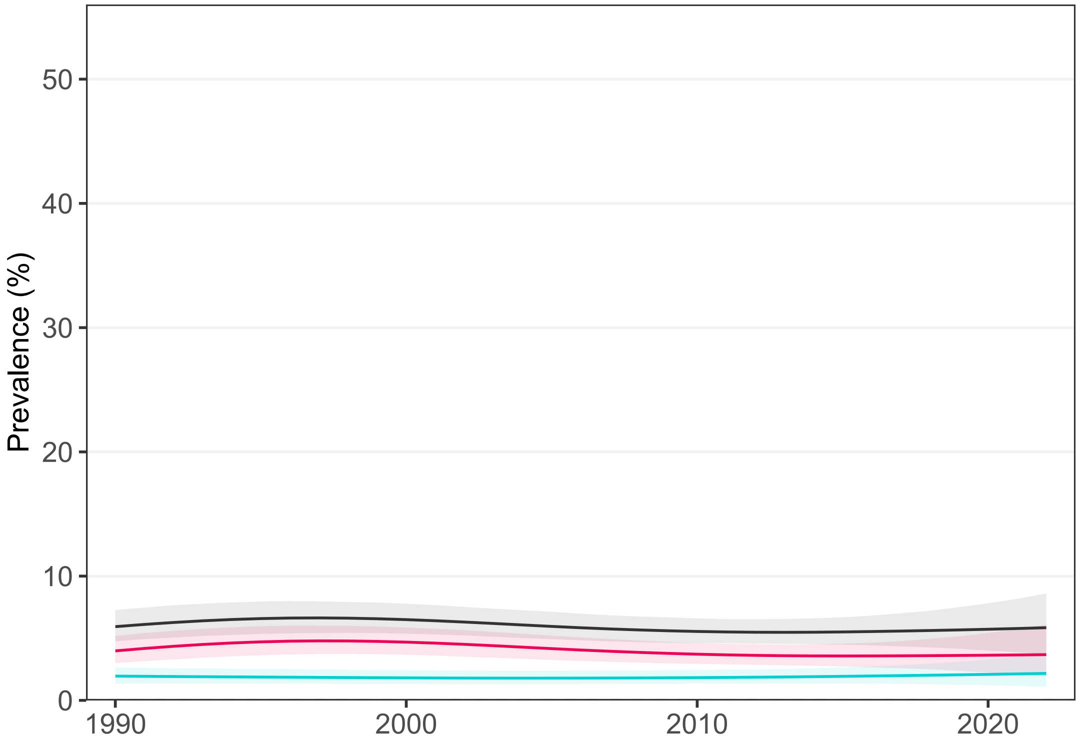


### Boys
64 studies (21 national)

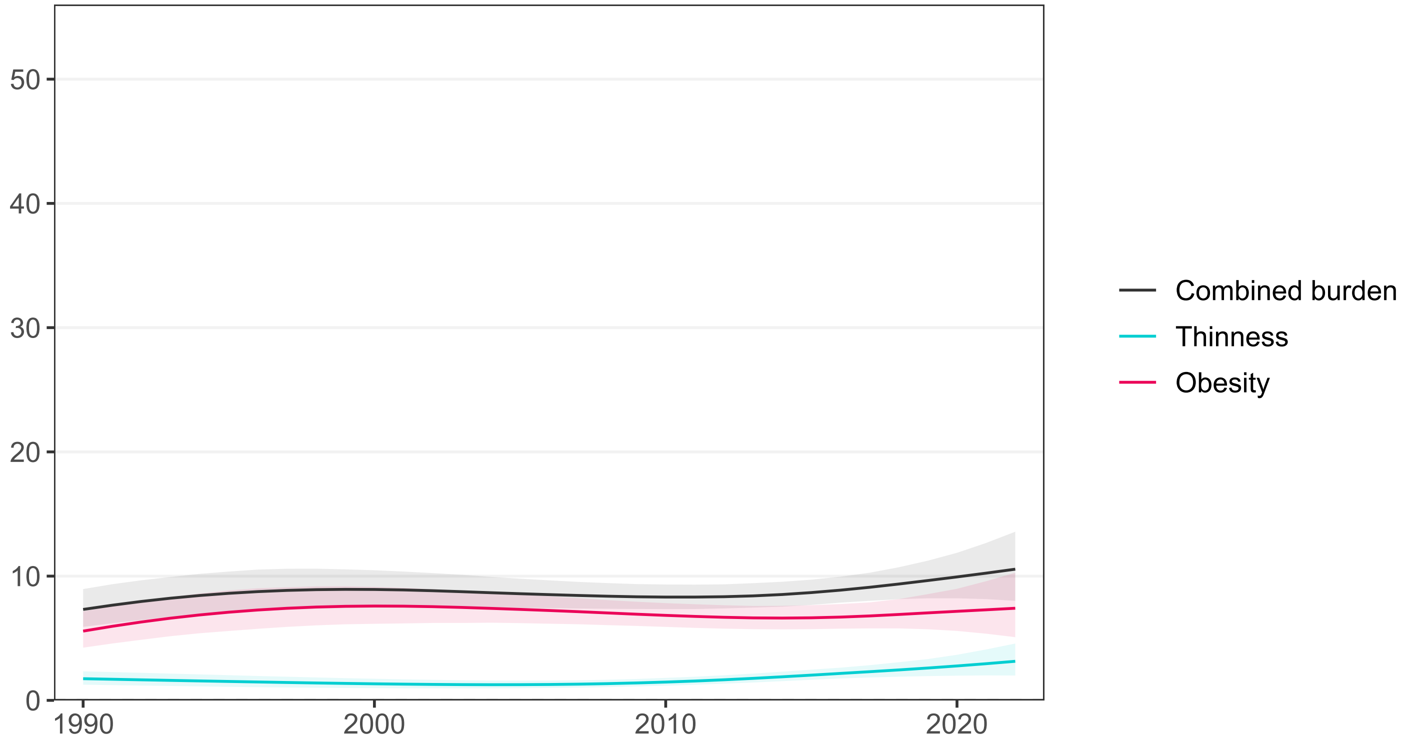


## Adults

### Women
33 studies (1 national)

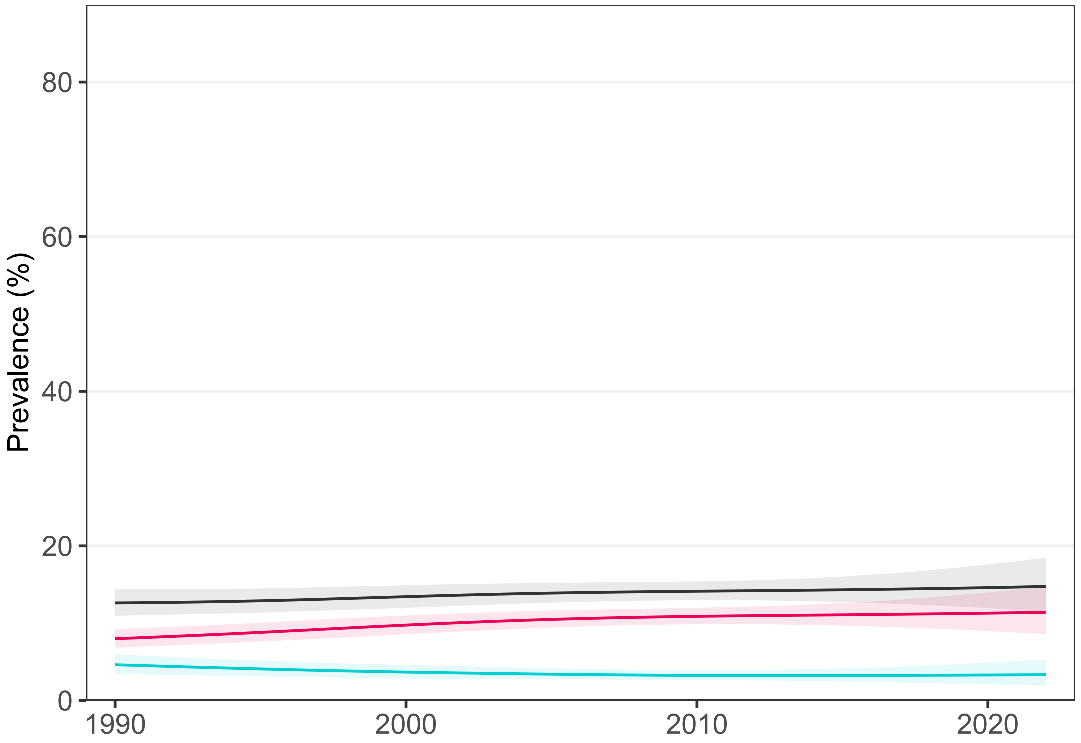


### Men
57 studies (23 national)

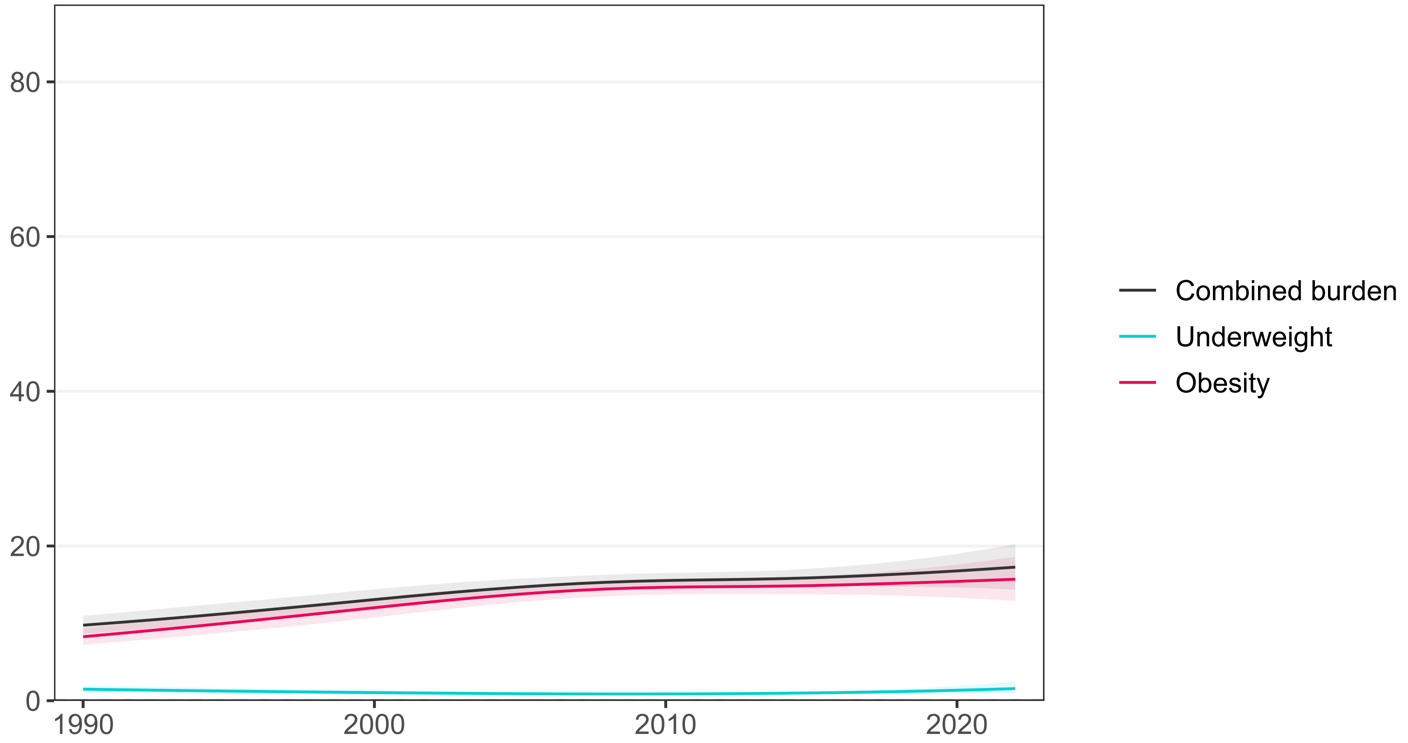

# Djibouti

## School-aged children and adolescents

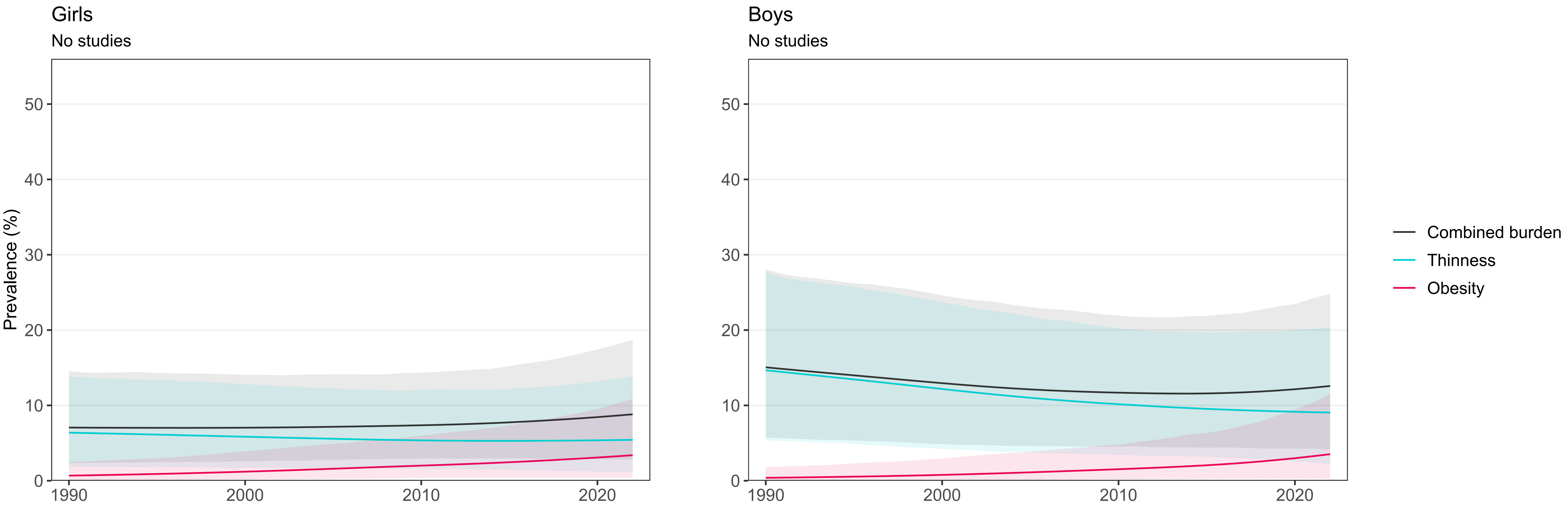


## Adults

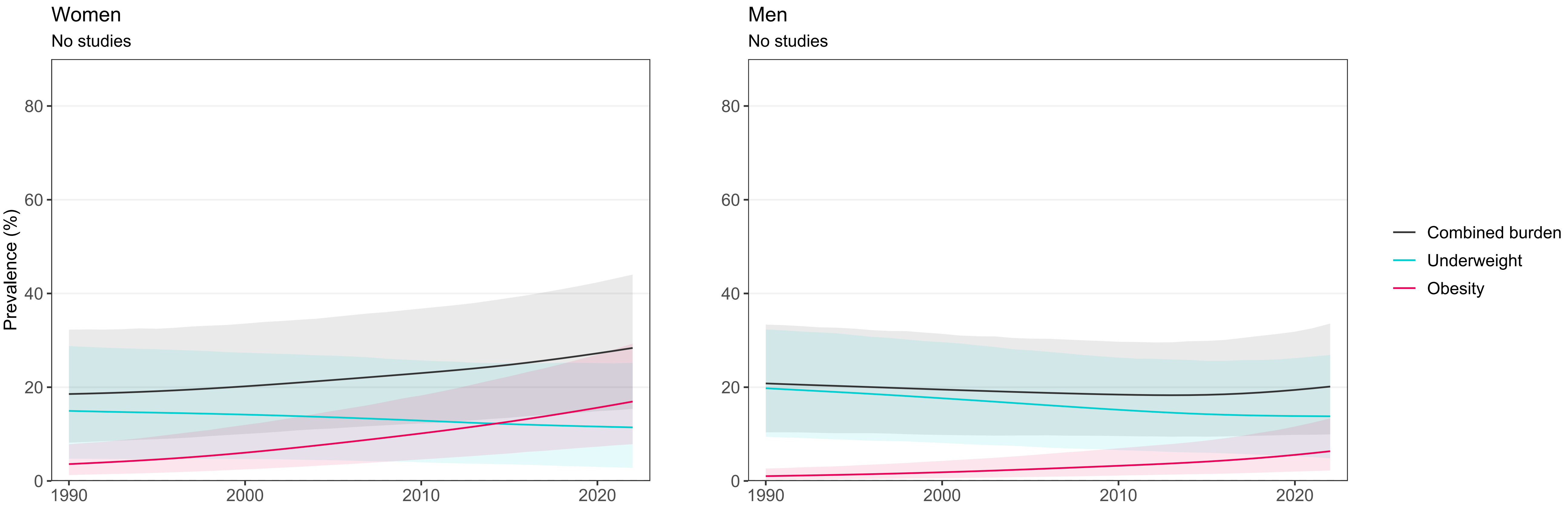

# Dominica

## School-aged children and adolescents

### Girls

2 studies (2 national)

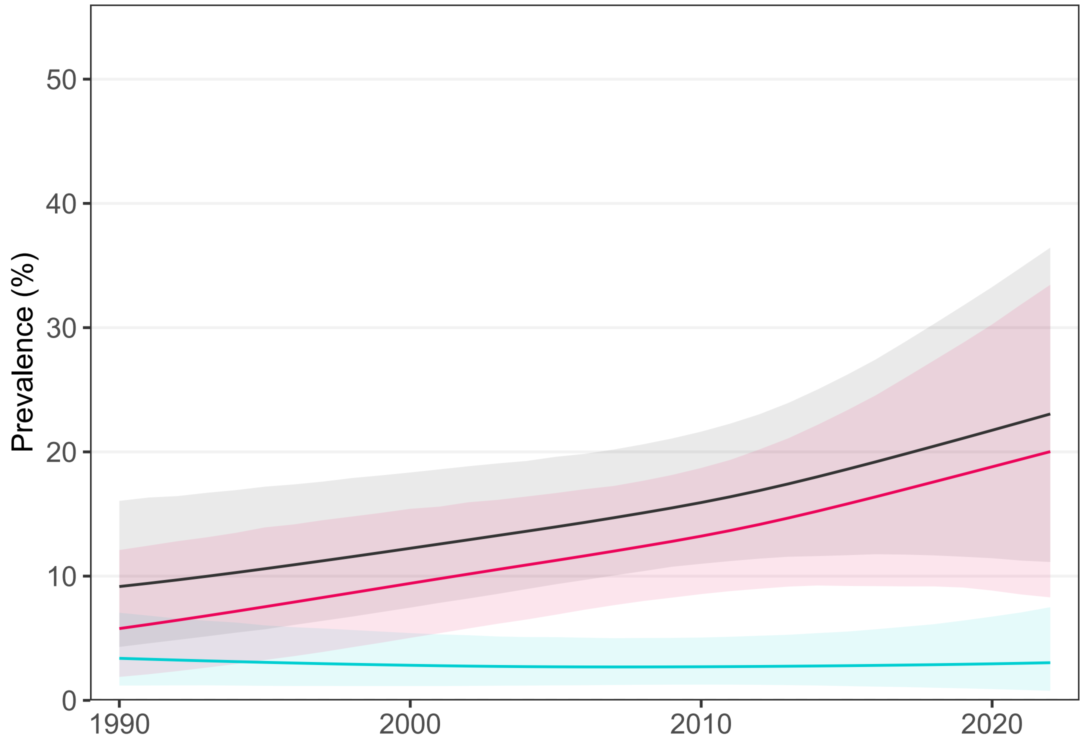


### Boys

2 studies (2 national)

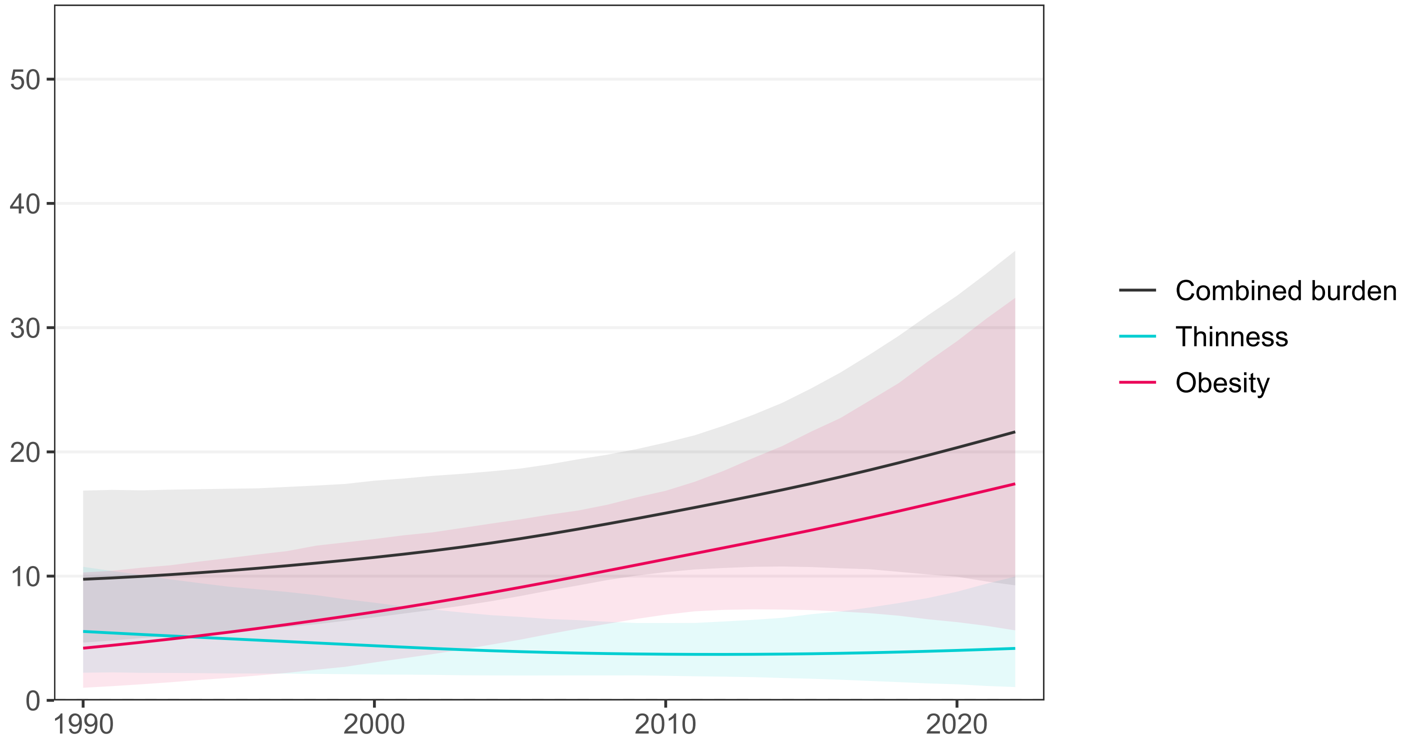


## Adults

### Women

1 study (1 national)

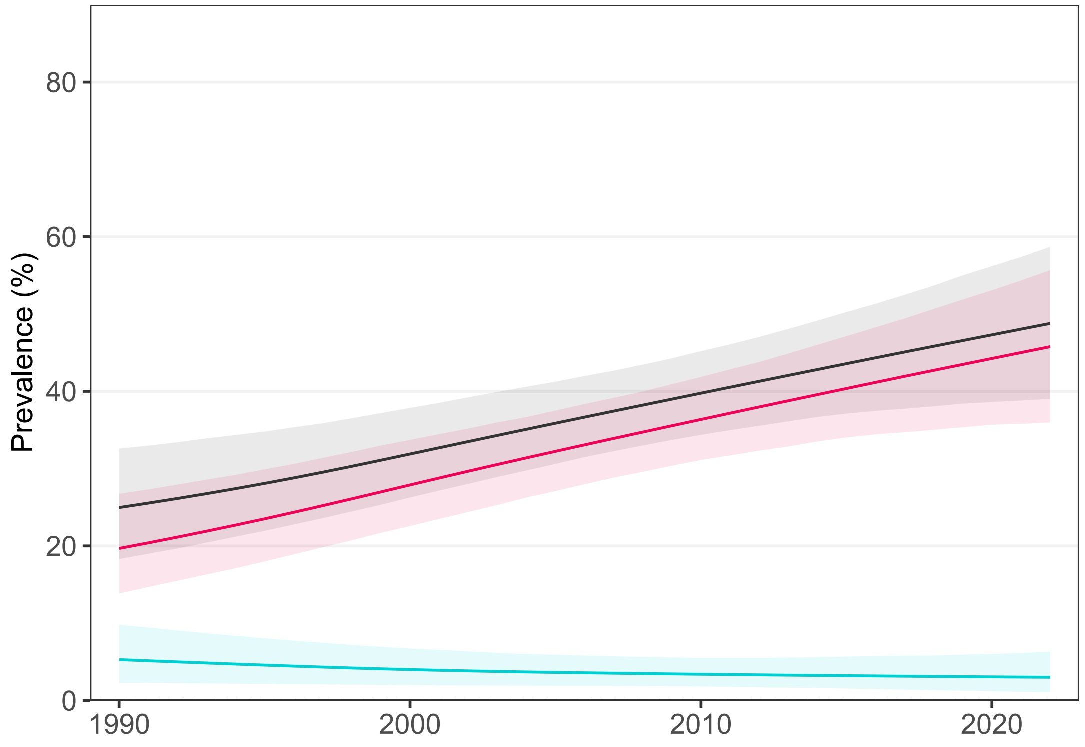


### Men

1 study (1 national)

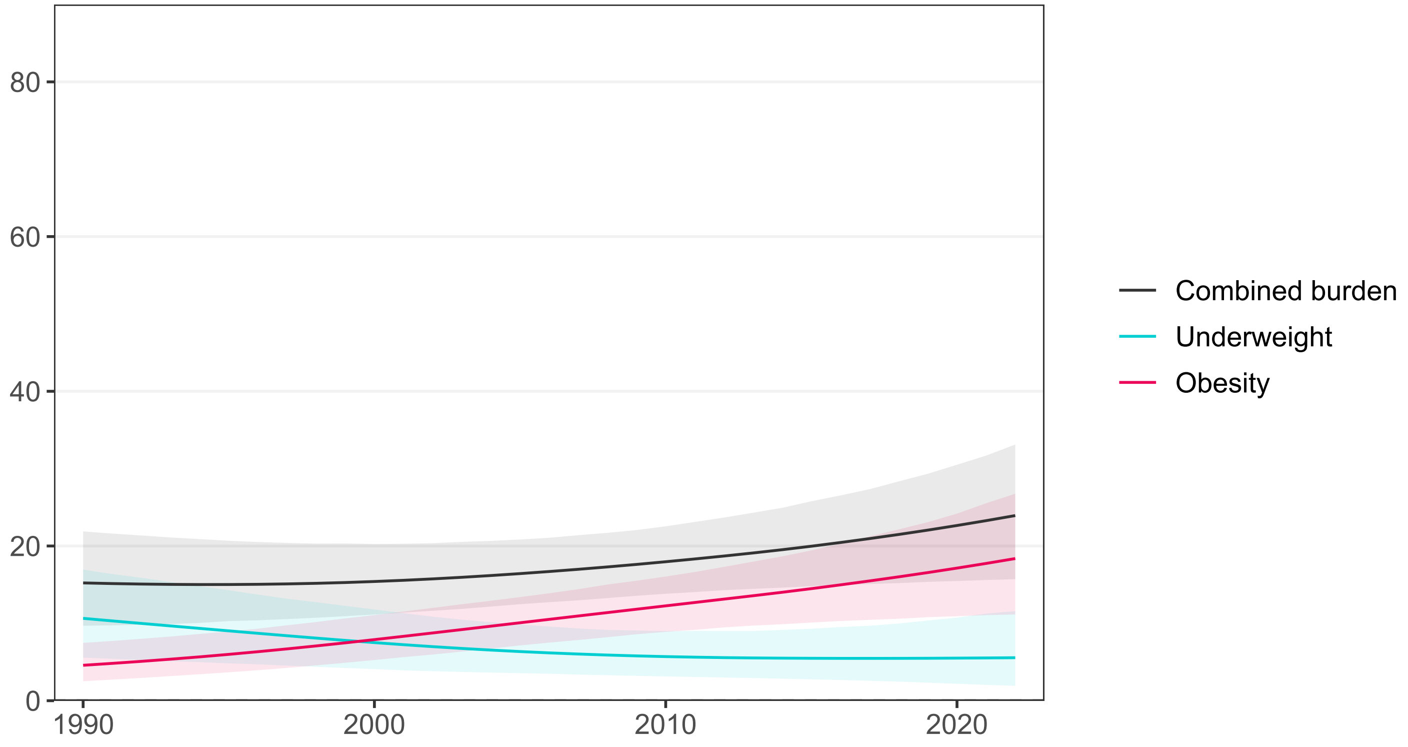

# Dominican Republic

## School-aged children and adolescents

### Girls

5 studies (5 national)

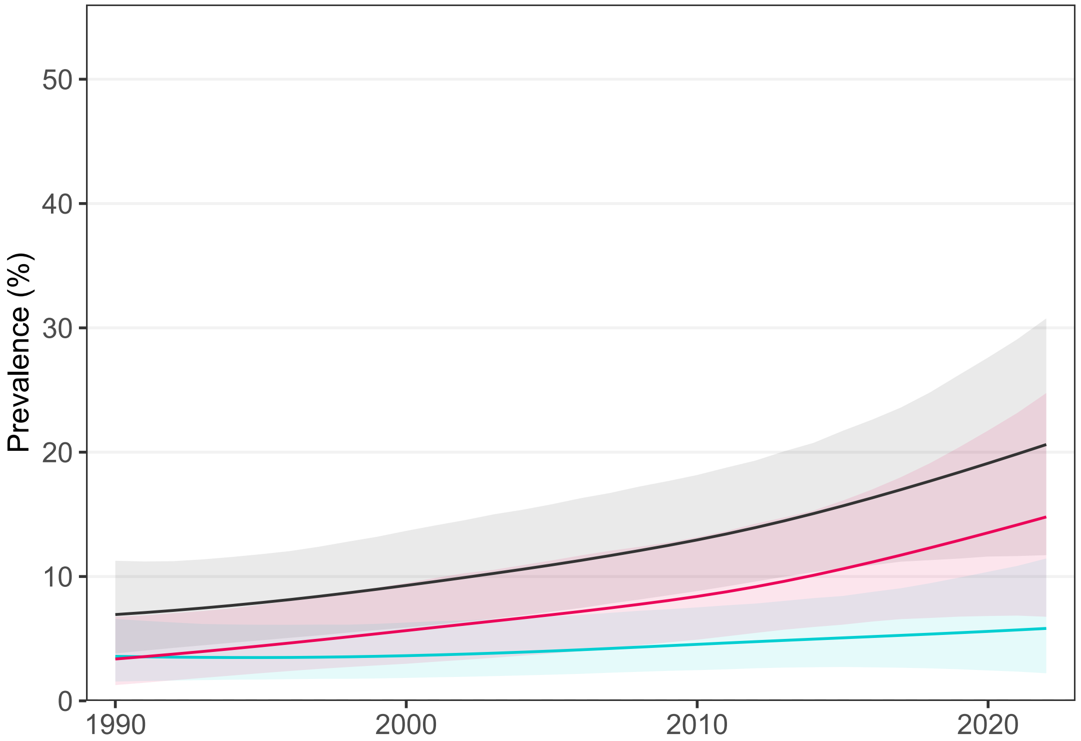


### Boys

4 studies (4 national)

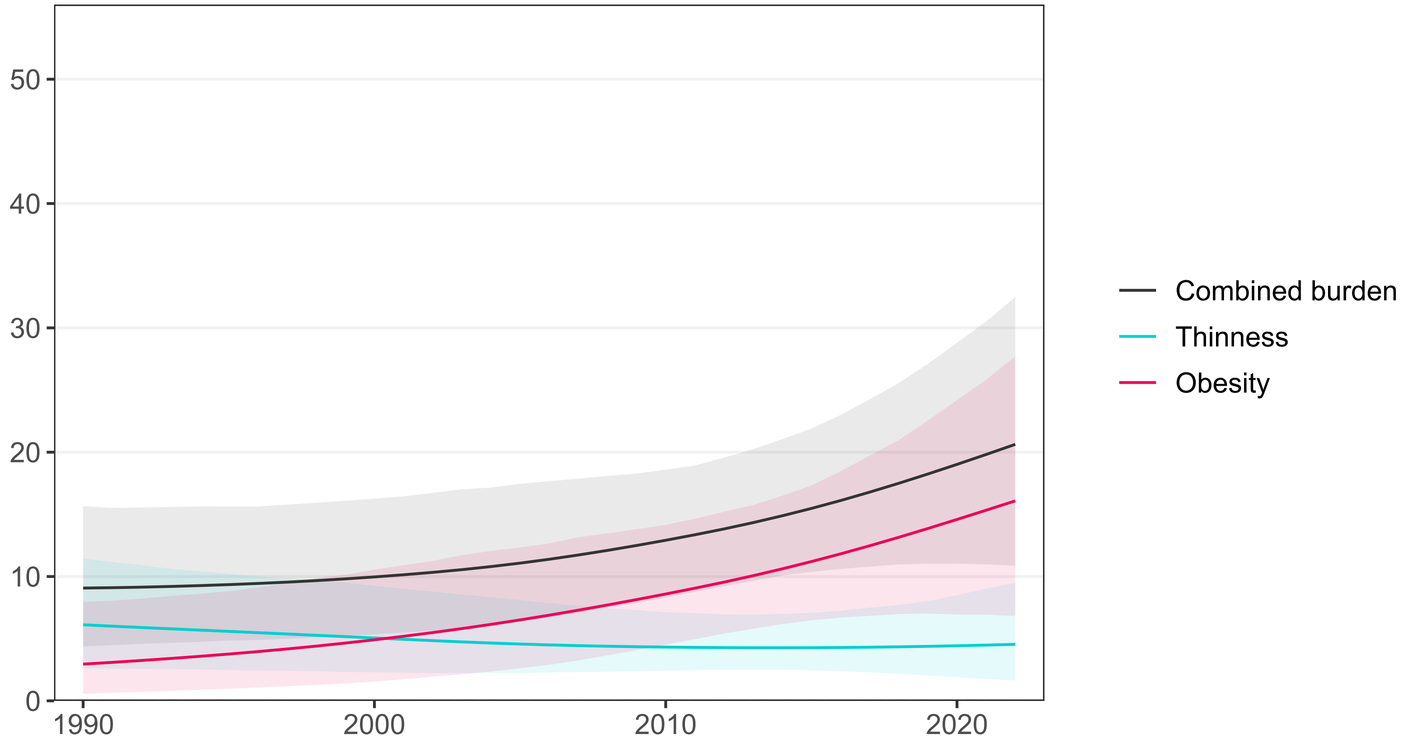


## Adults

### Women

7 studies (7 national)

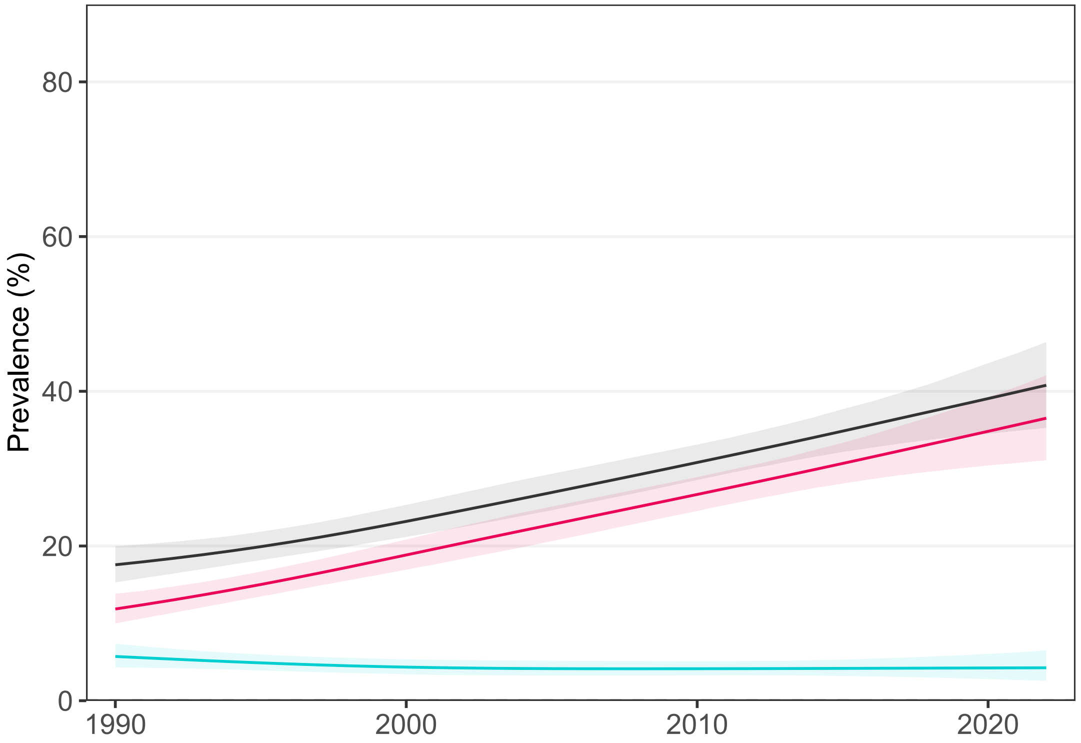


### Men

5 studies (5 national)

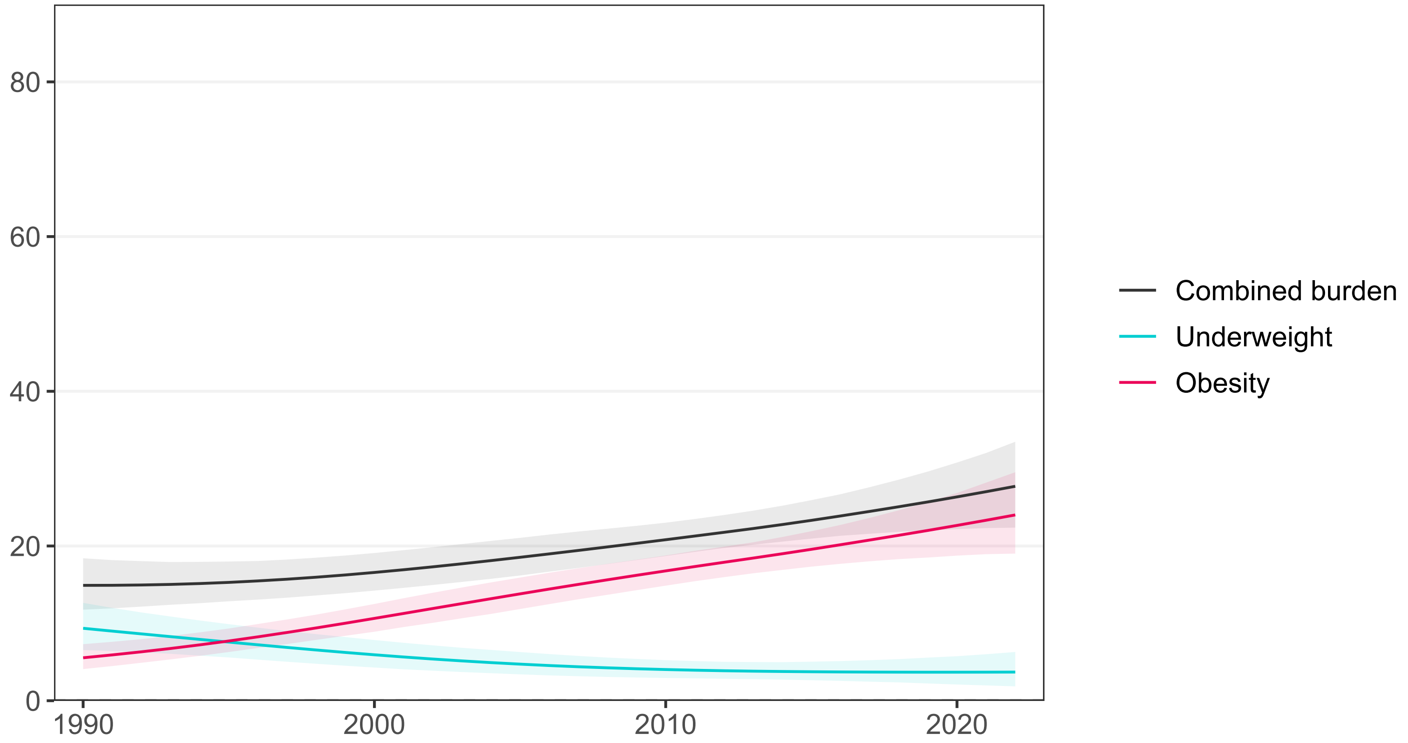

# DR Congo

## School-aged children and adolescents

### Girls

6 studies (3 national)

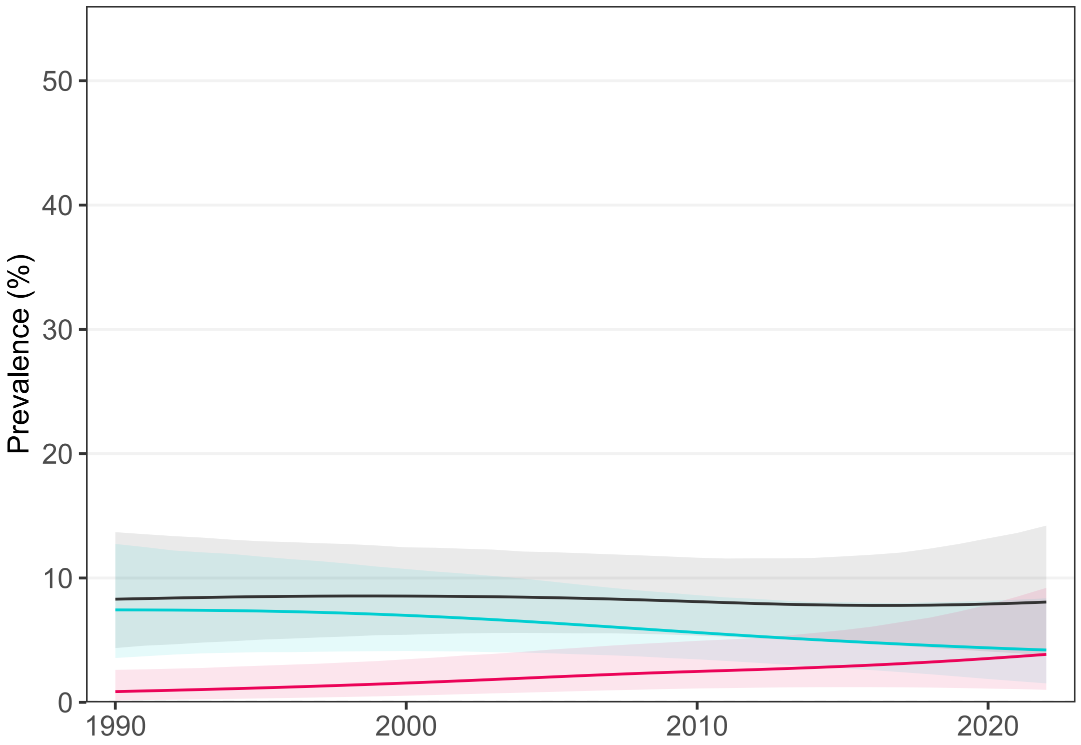


### Boys

3 studies (0 national)

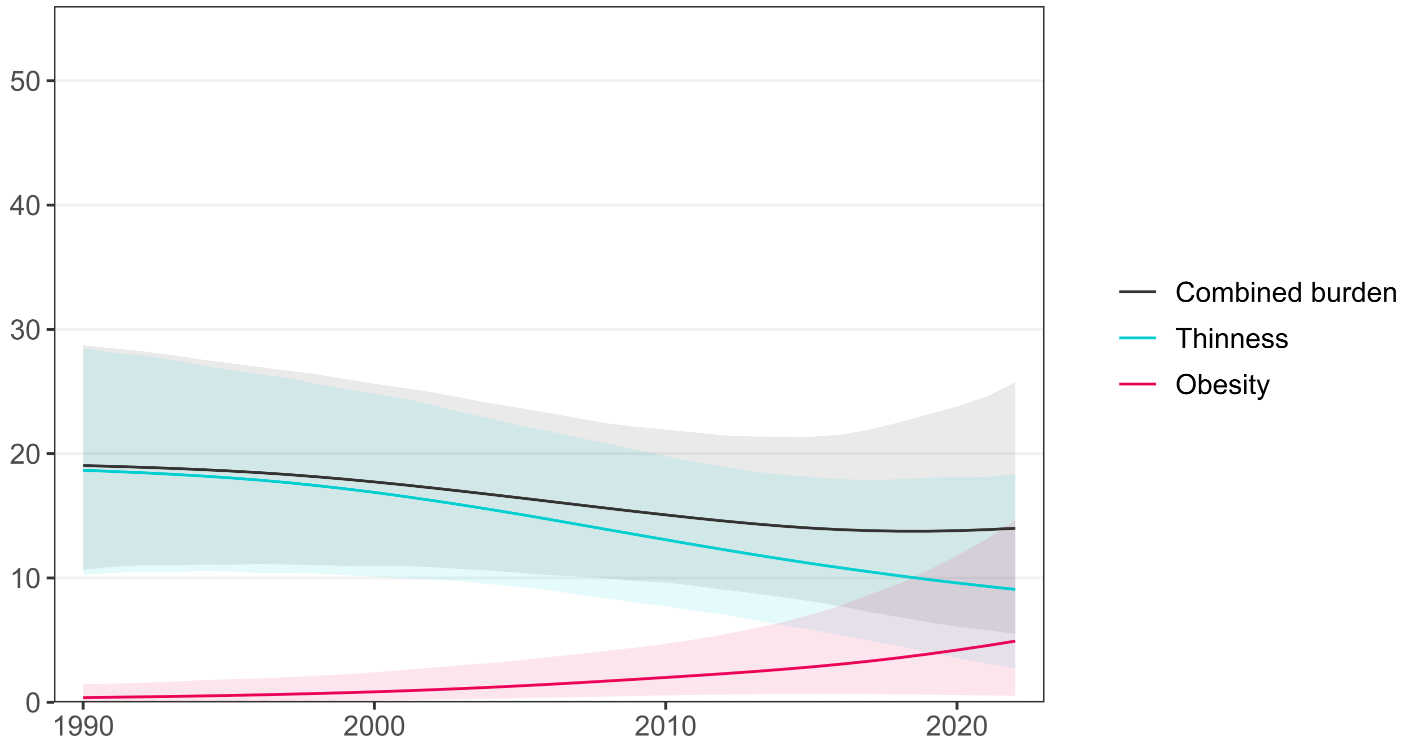


## Adults

### Women

10 studies (3 national)

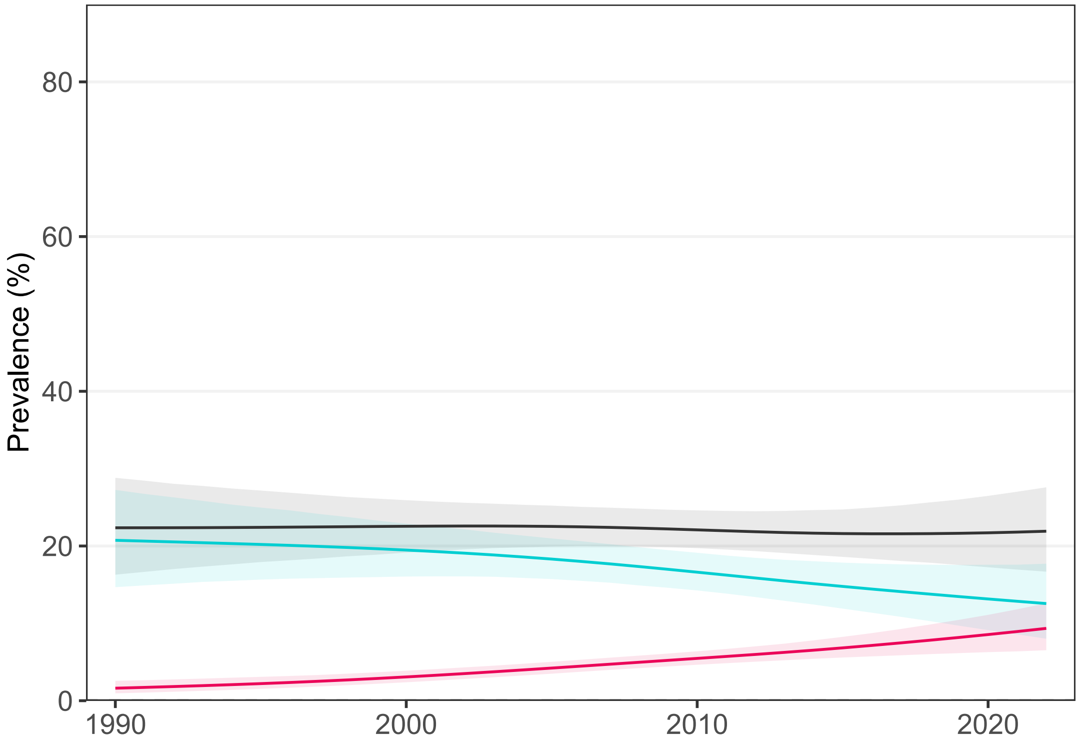


### Men

7 studies (0 national)

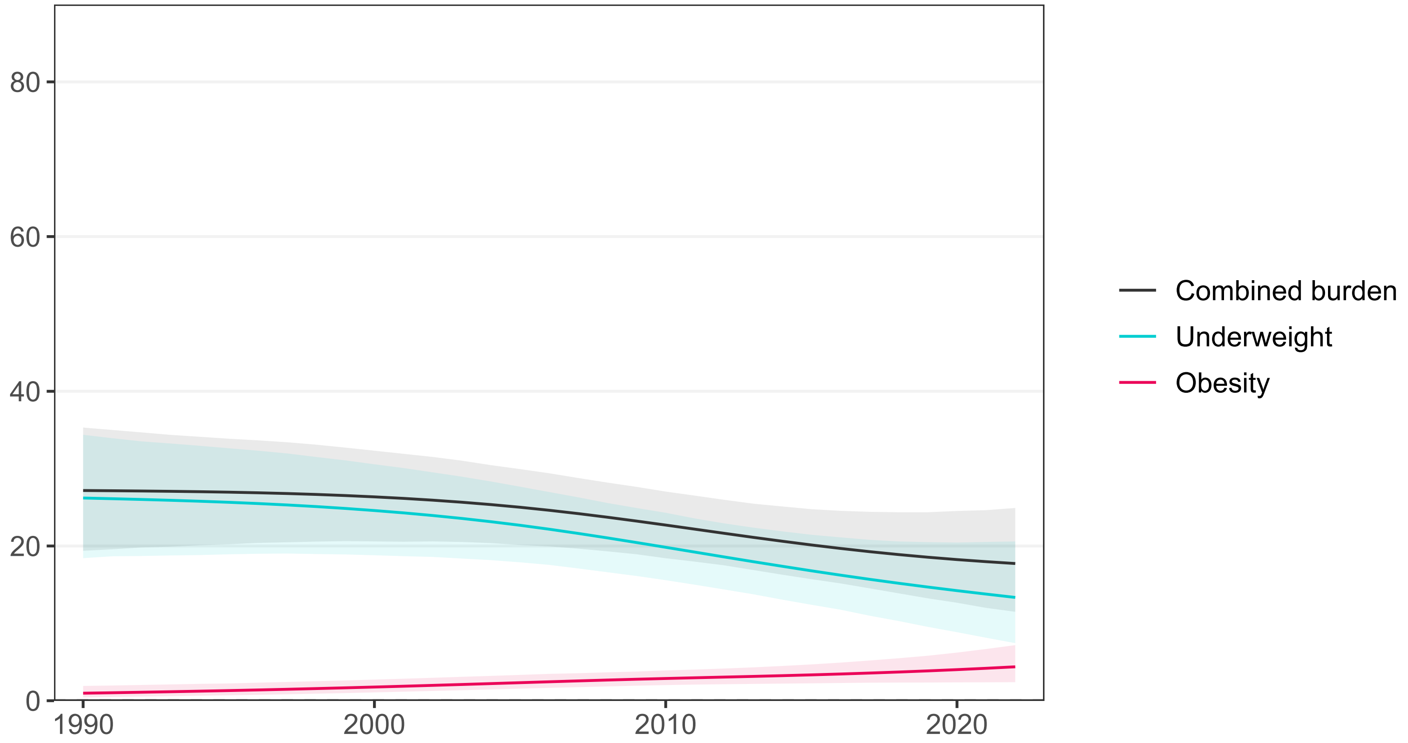

# Ecuador

## School-aged children and adolescents

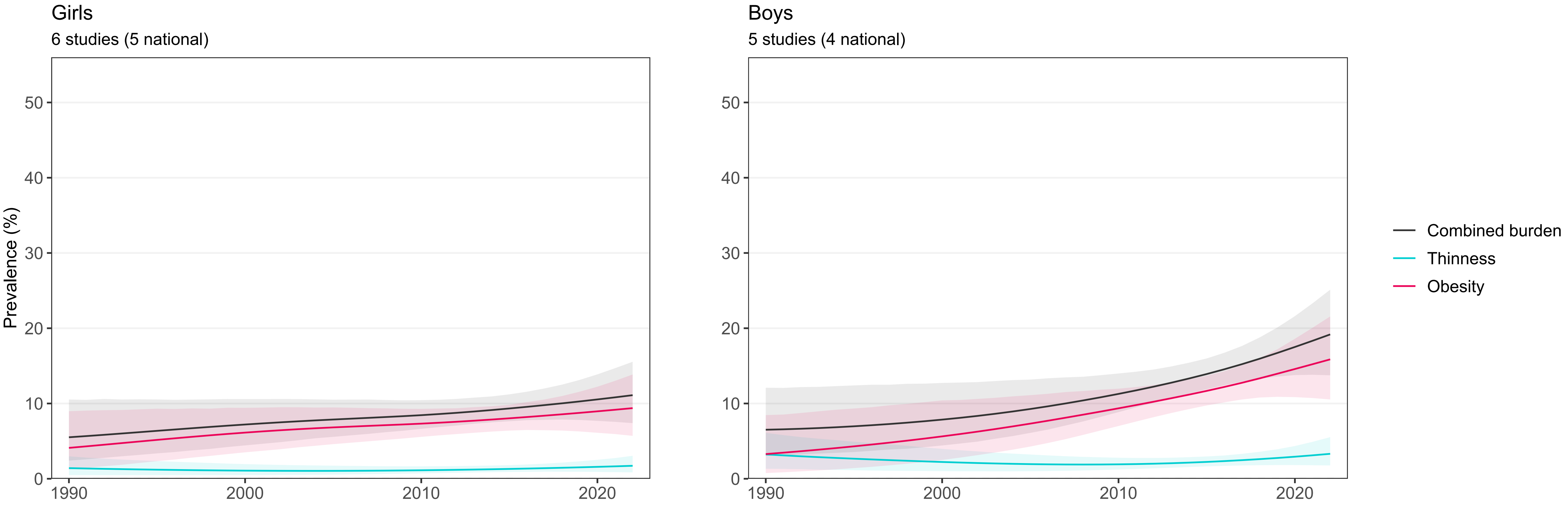


## Adults

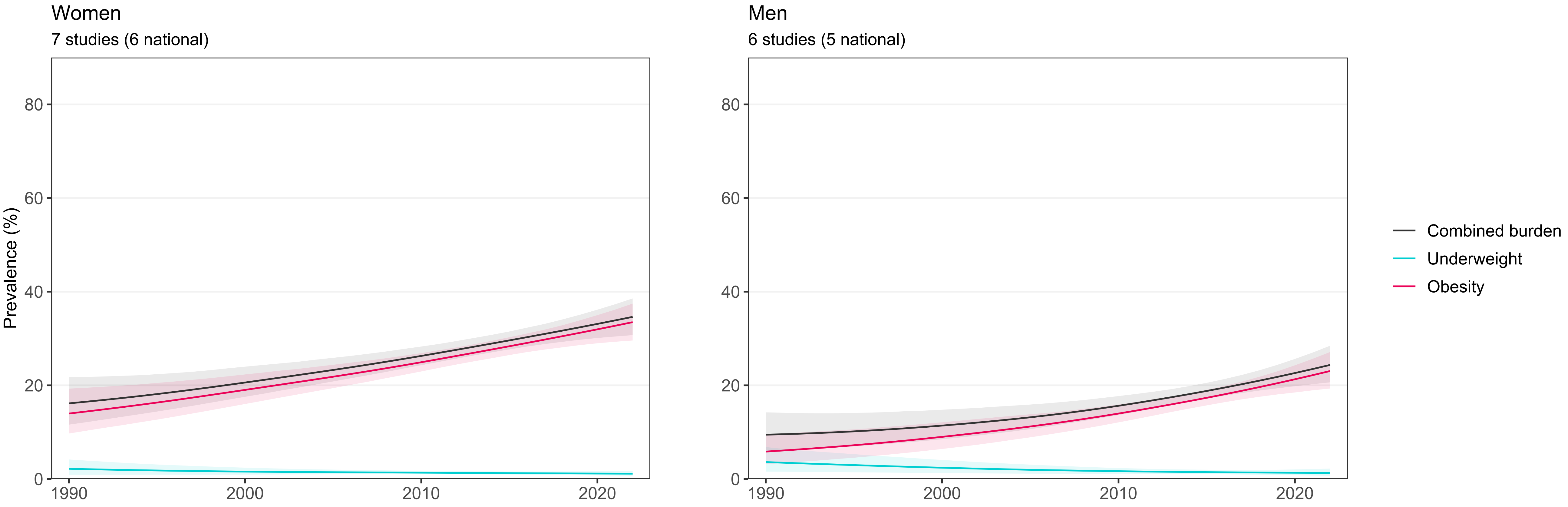

# Egypt

## School-aged children and adolescents

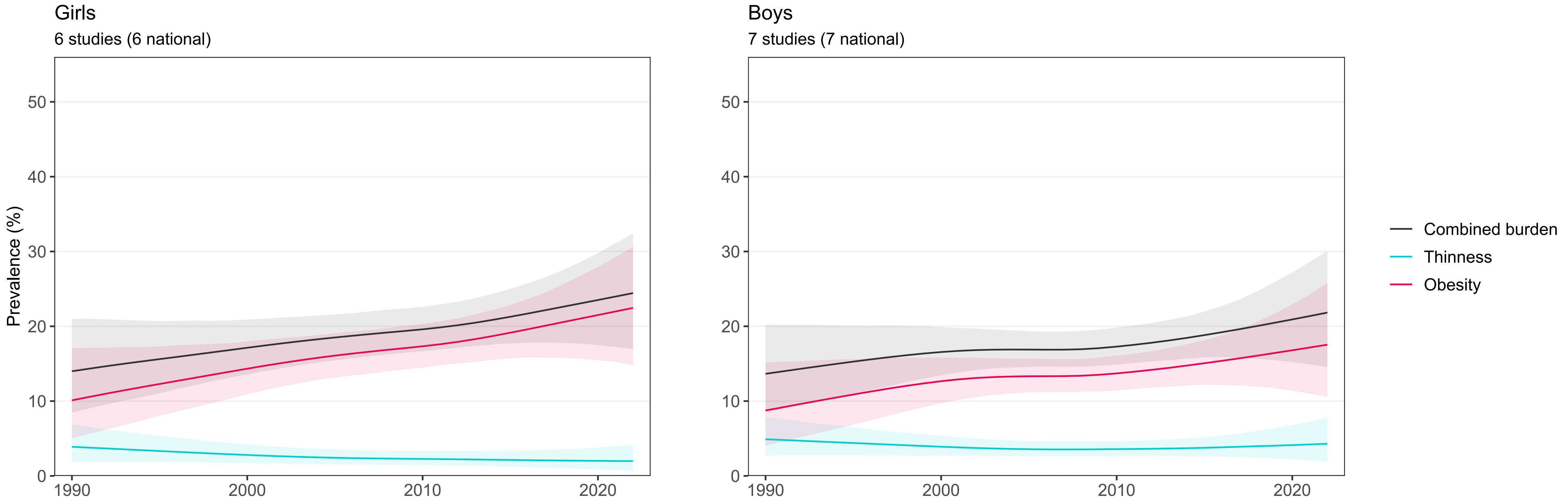


## Adults

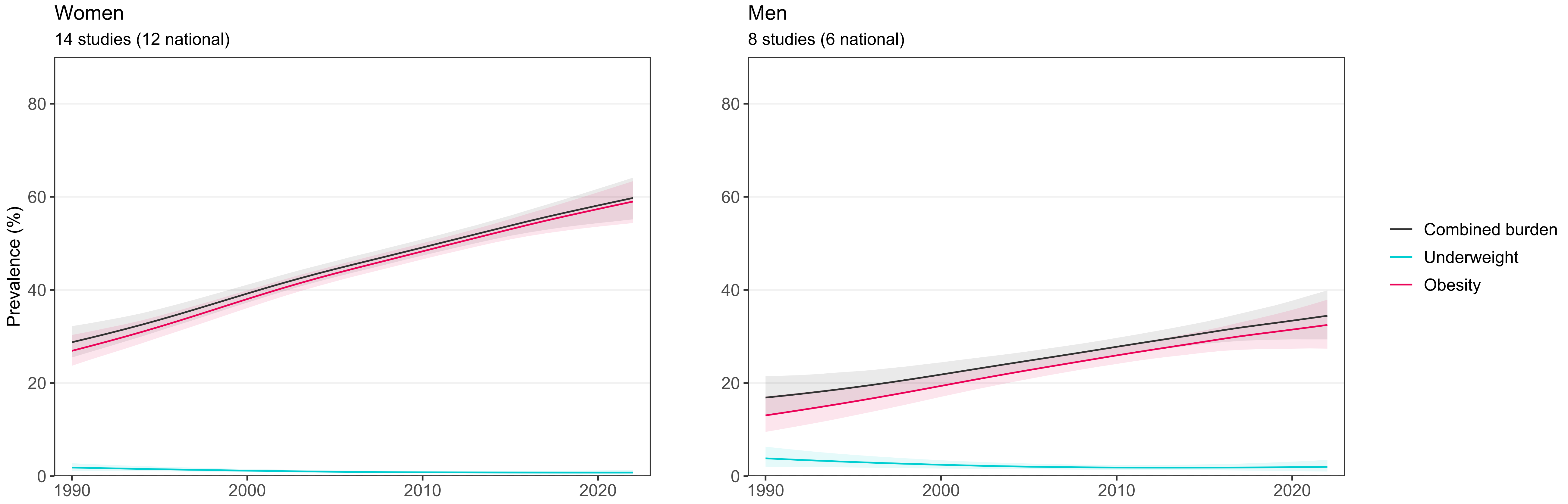

# El Salvador

## School-aged children and adolescents

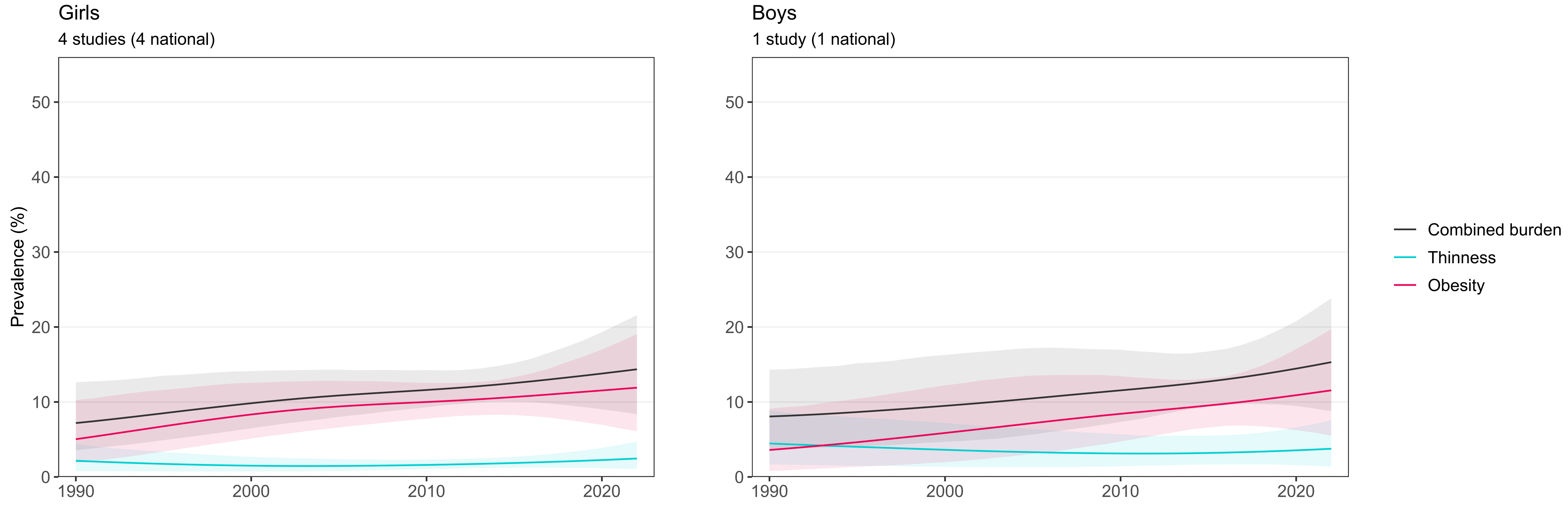


## Adults

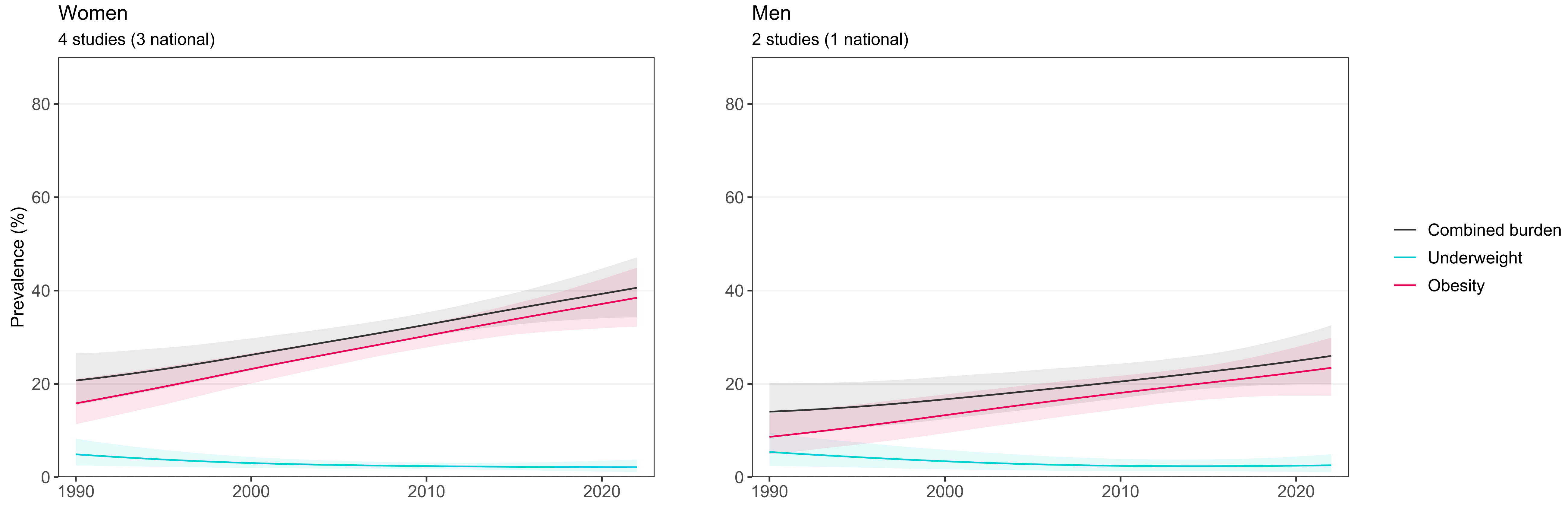

# Equatorial Guinea

## School-aged children and adolescents

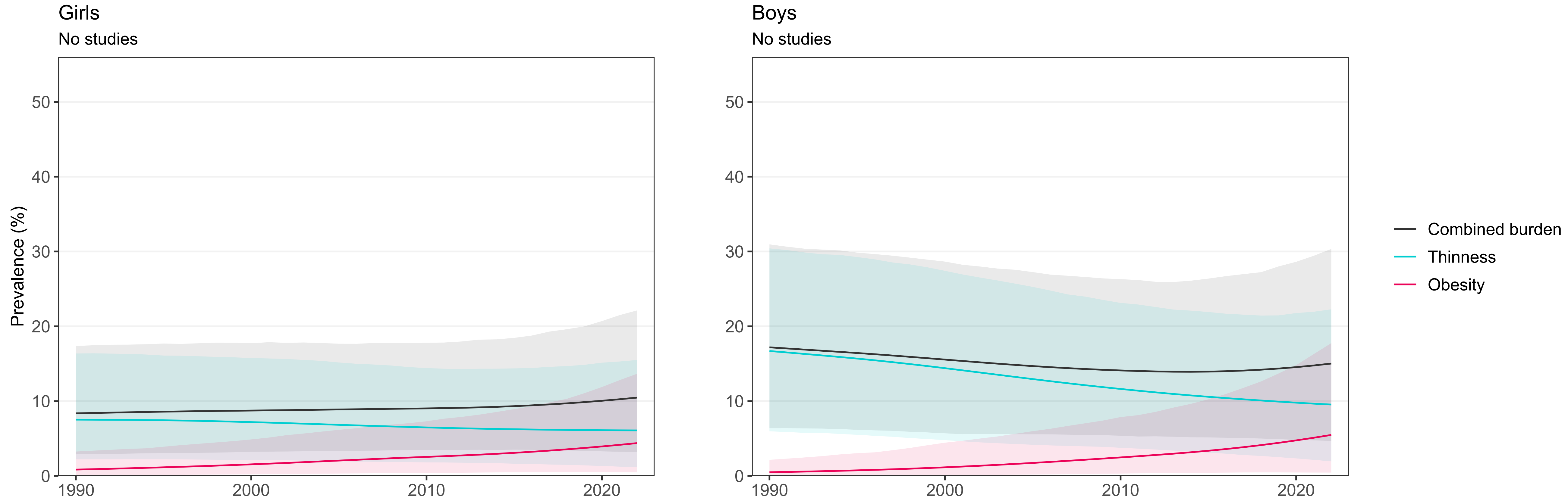


## Adults

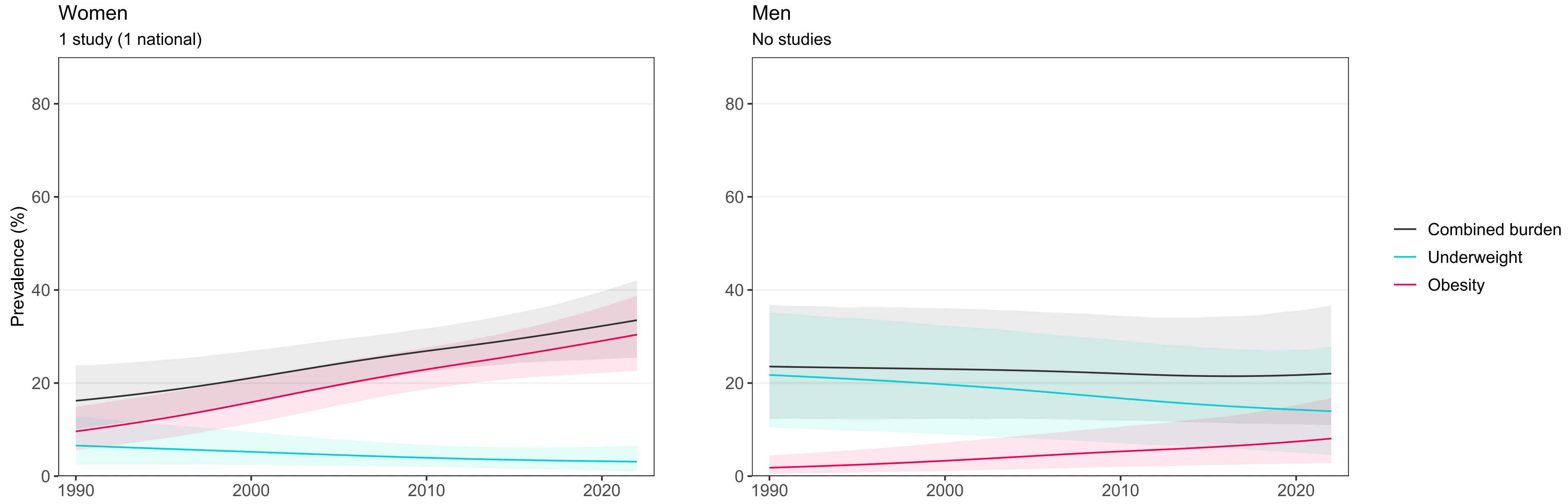

# Eritrea

## School-aged children and adolescents

### Girls

1 study (1 national)

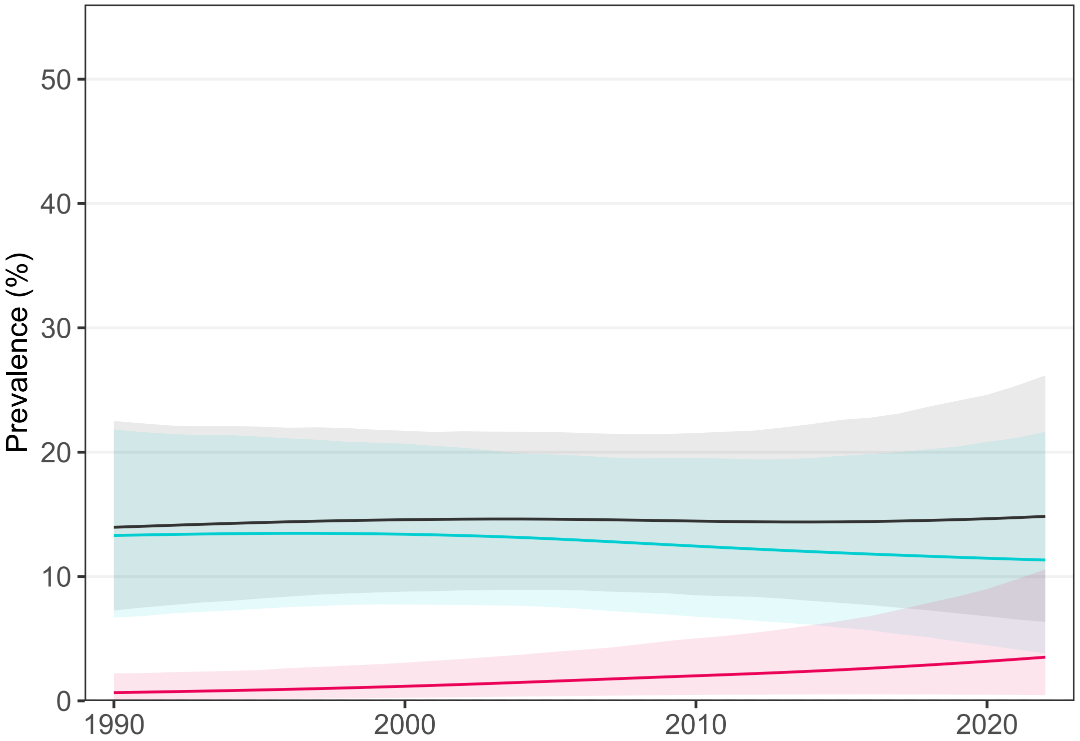


### Boys

1 study (1 national)

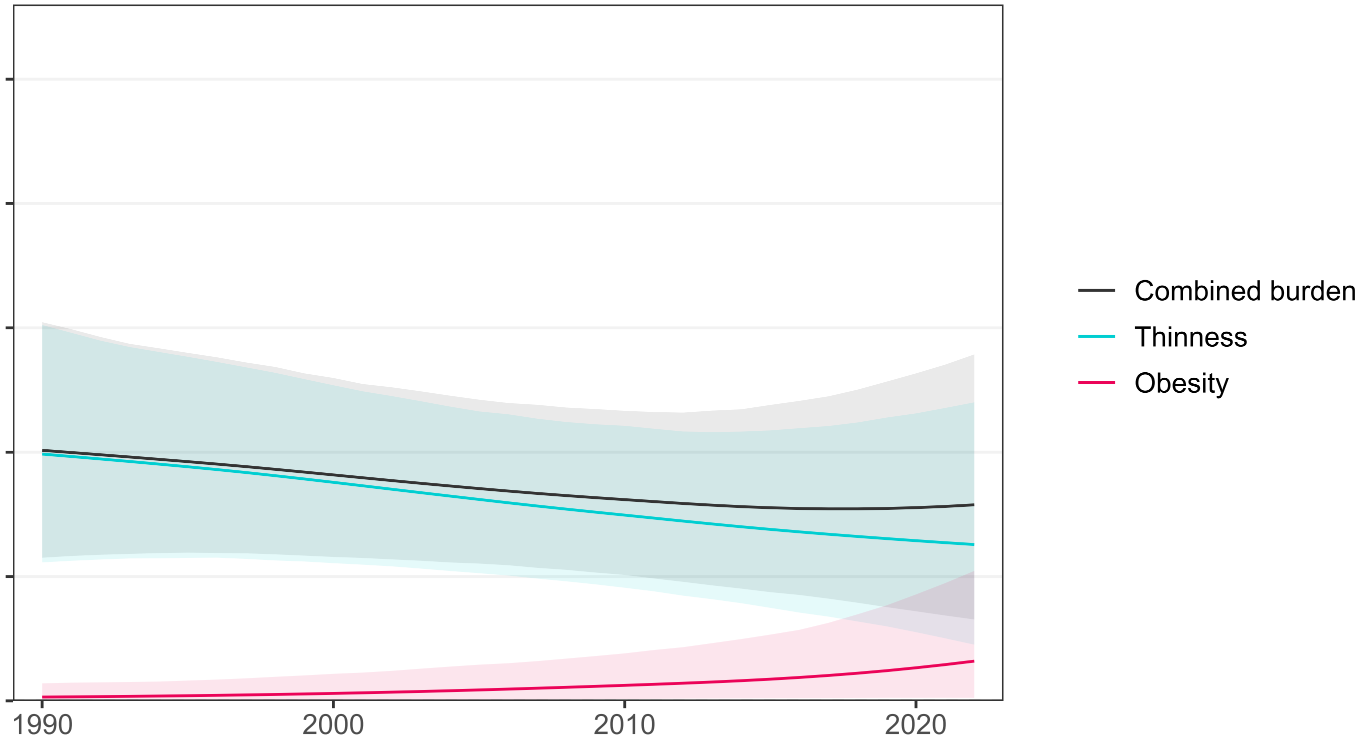


## Adults

### Women

4 studies (4 national)

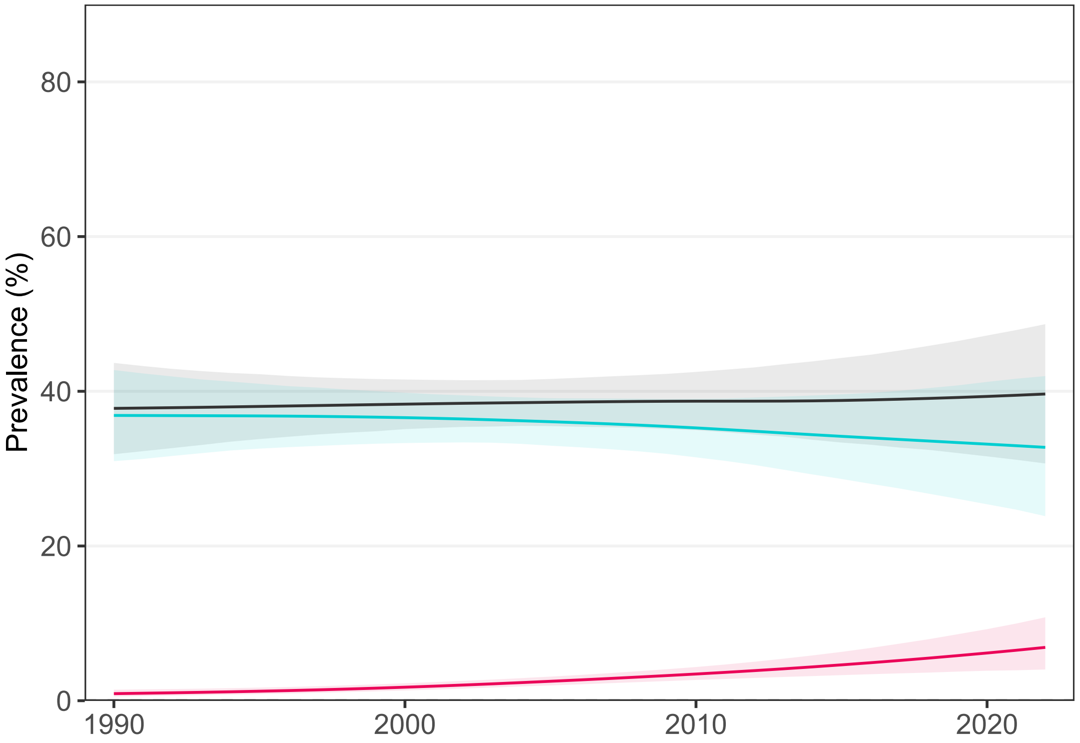


### Men

2 studies (2 national)

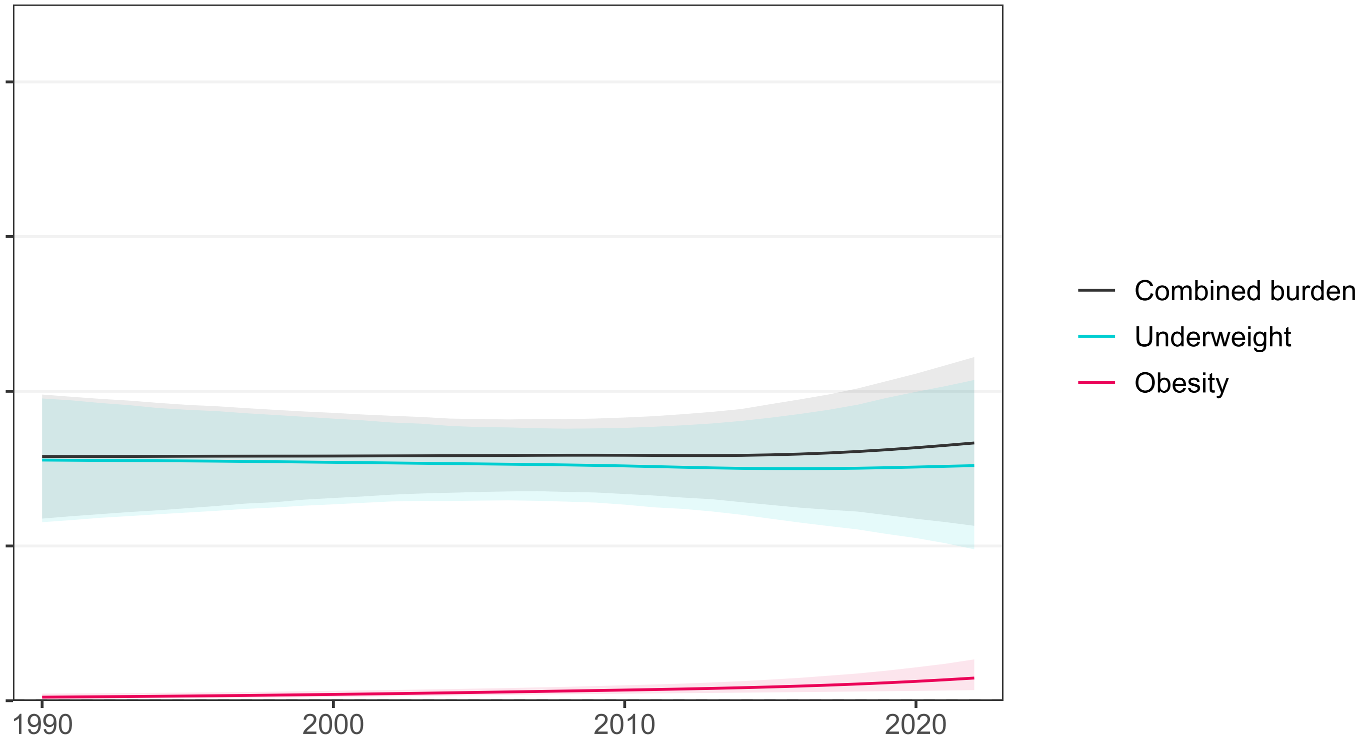

# Estonia

## School-aged children and adolescents

Girls
13 studies (12 national)

Boys
13 studies (12 national)

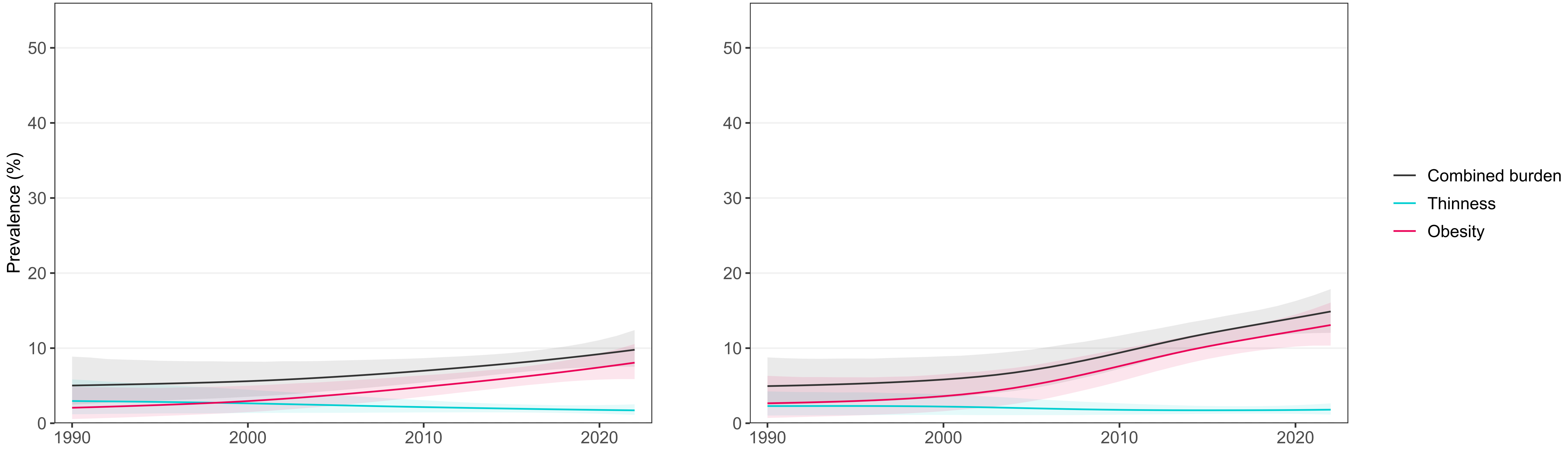


## Adults

Women
12 studies (9 national)

Men
14 studies (9 national)

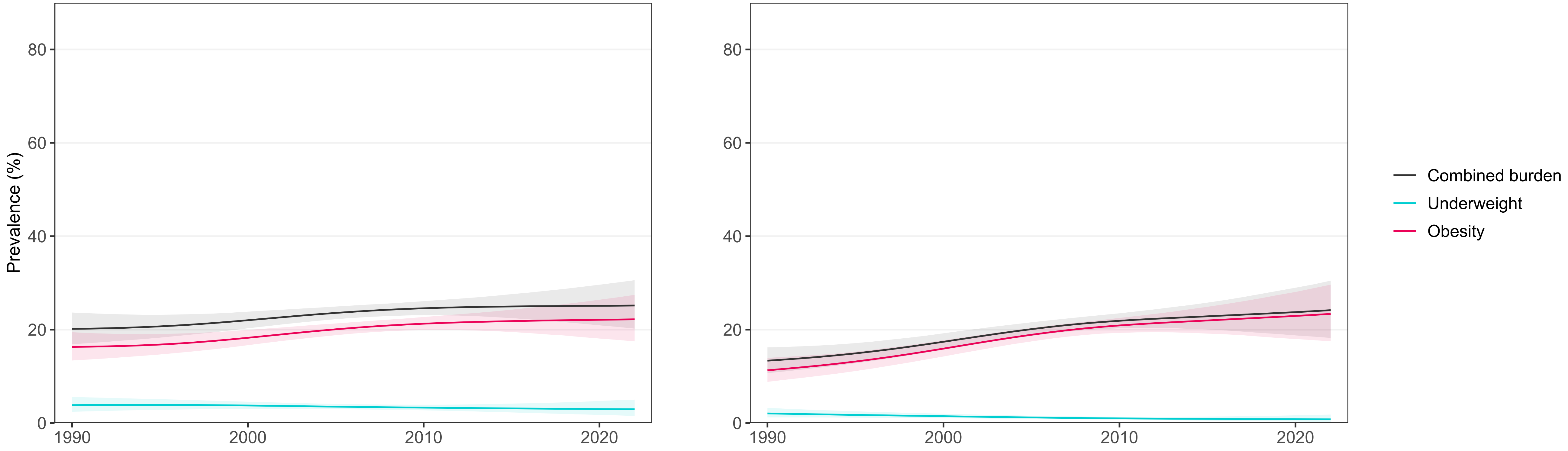

# Eswatini

## School-aged children and adolescents

### Girls
2 studies (2 national)

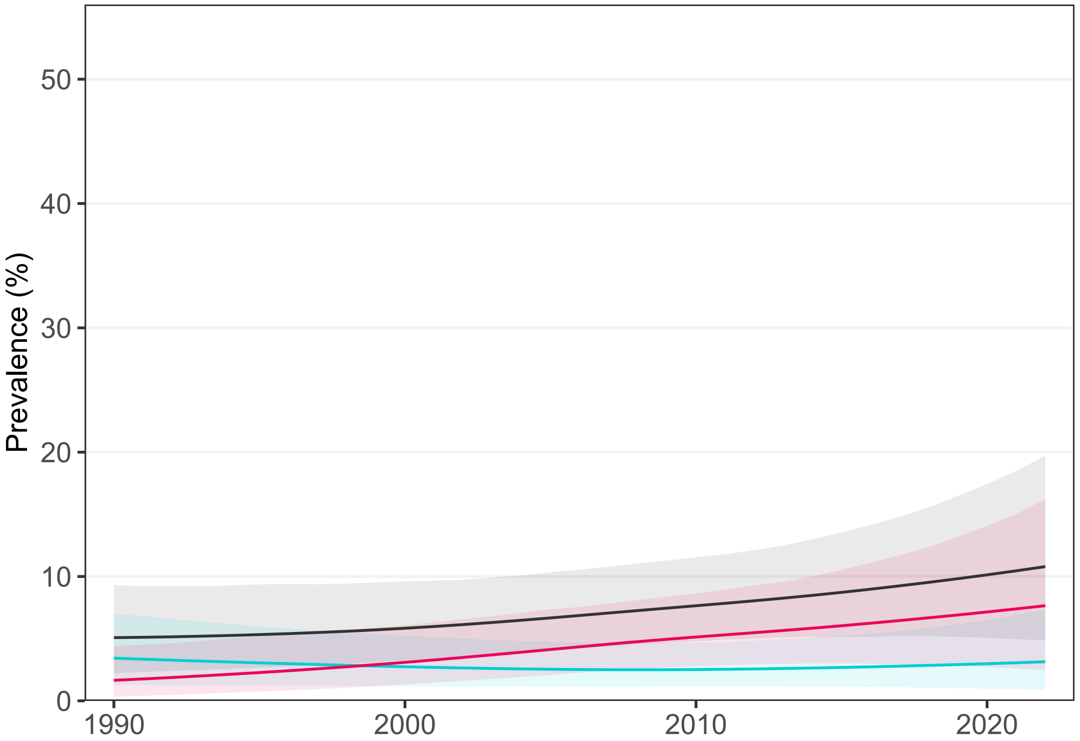


### Boys
2 studies (2 national)

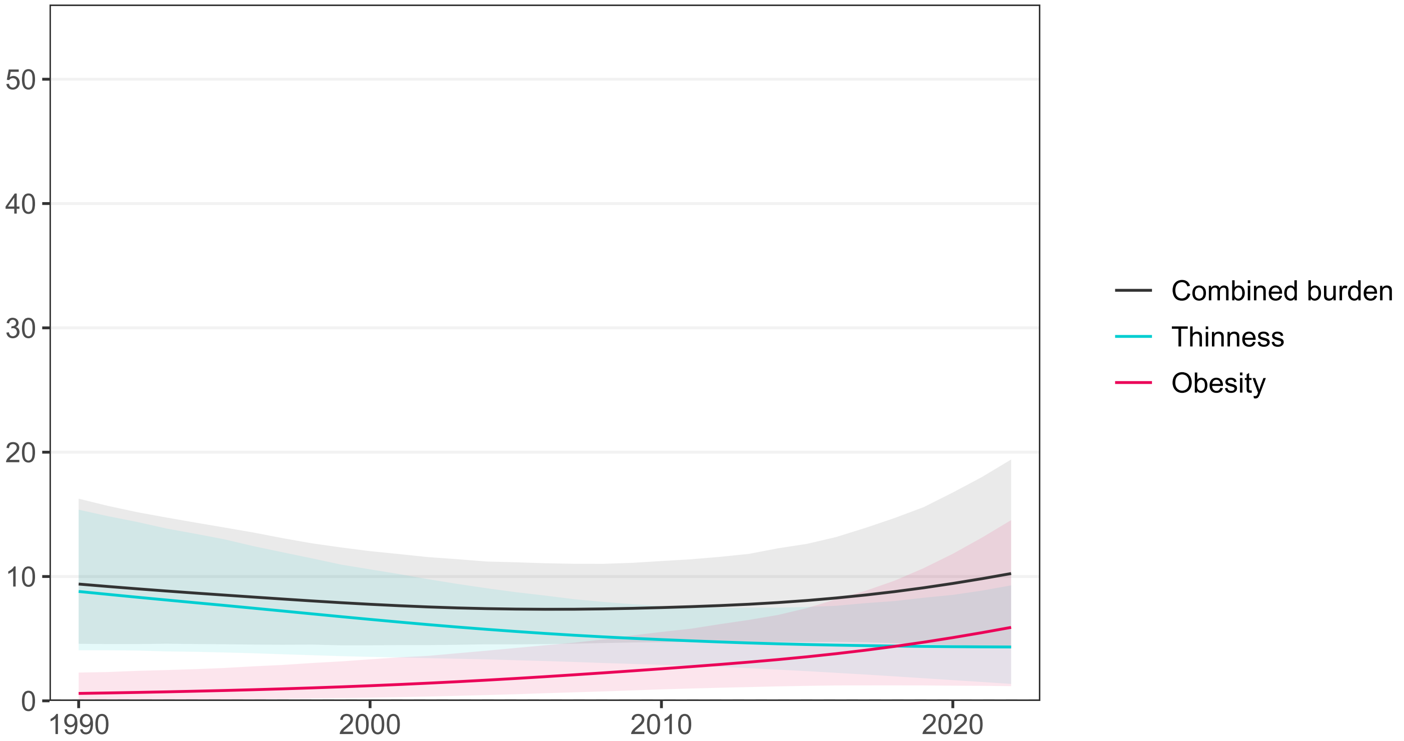


## Adults

### Women
2 studies (2 national)

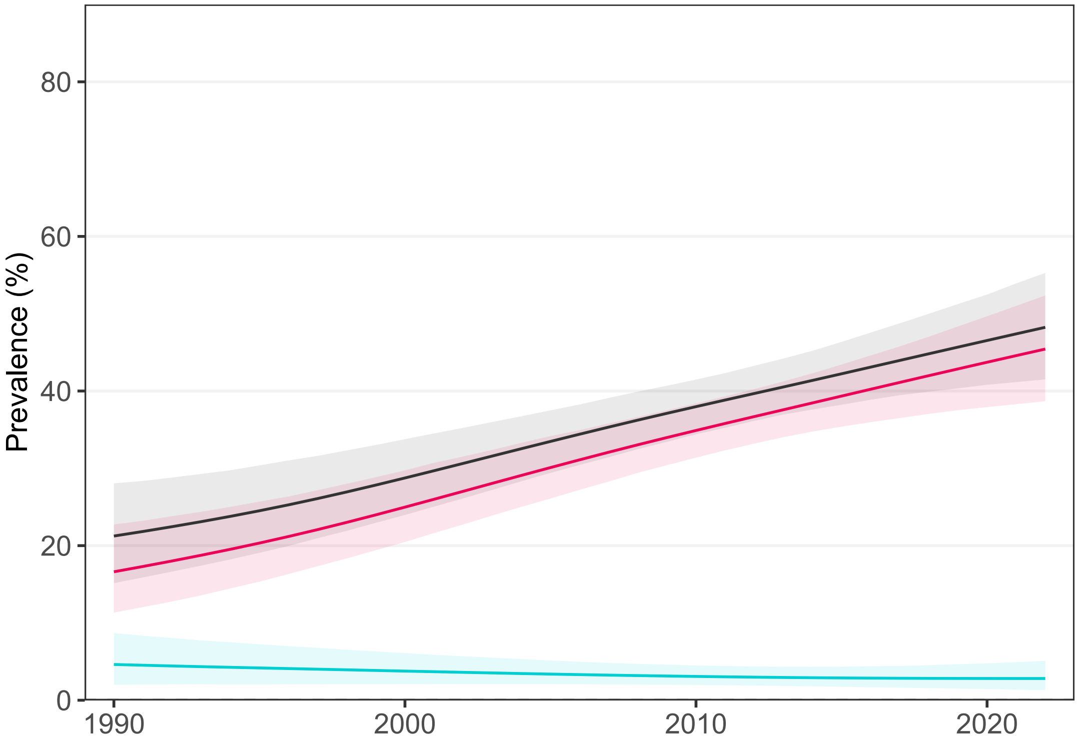


### Men
2 studies (2 national)

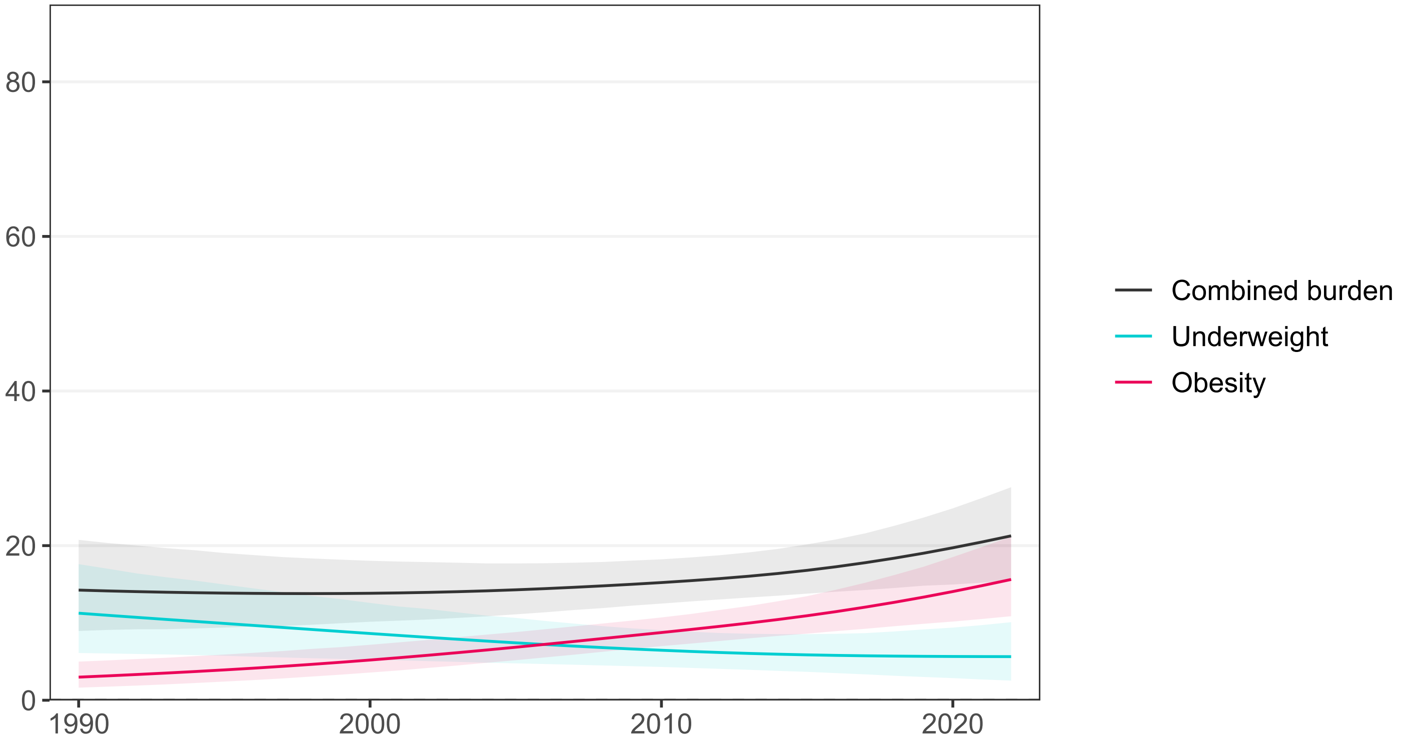

# Ethiopia

## School-aged children and adolescents

### Girls

10 studies (6 national)

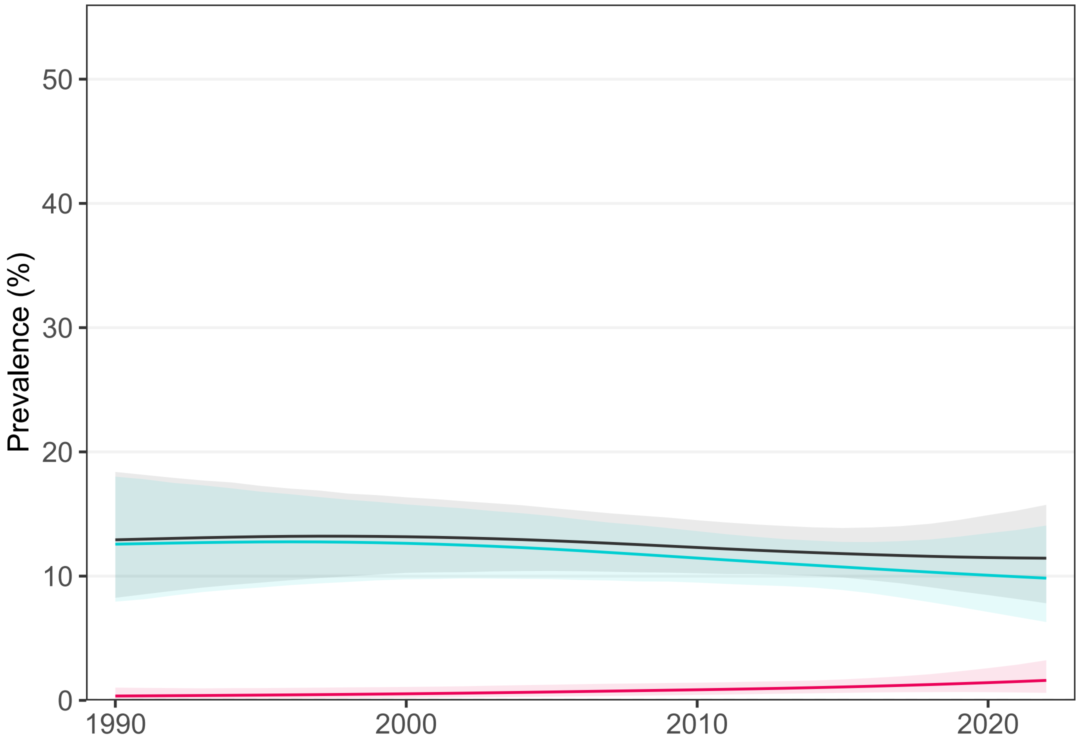


### Boys

6 studies (4 national)

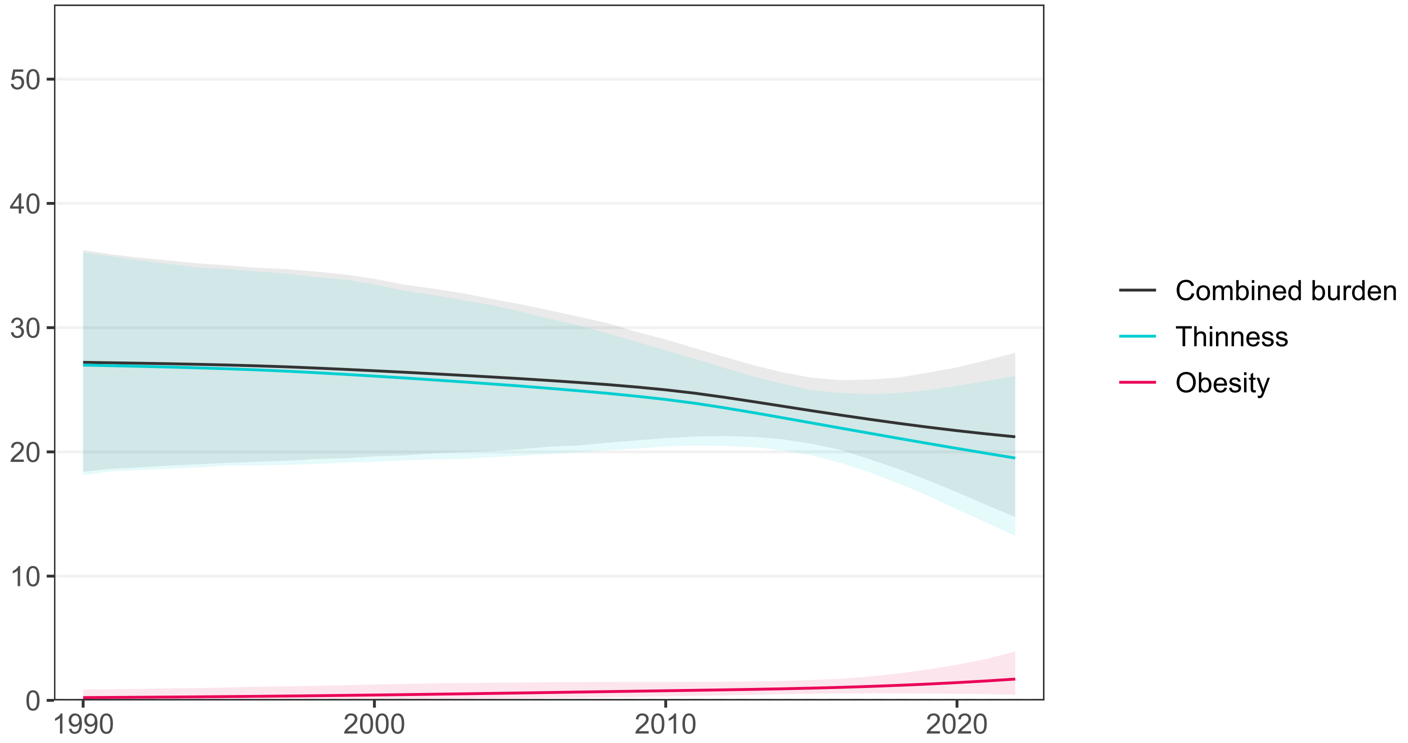


## Adults

### Women

11 studies (6 national)

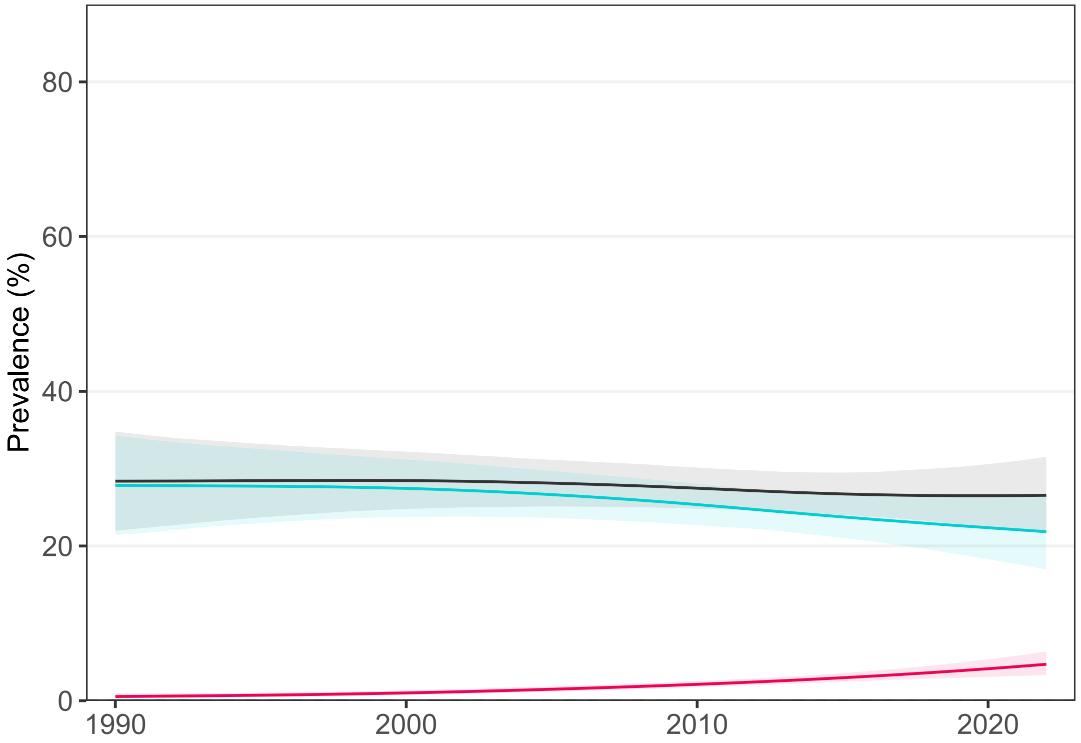


### Men

7 studies (4 national)

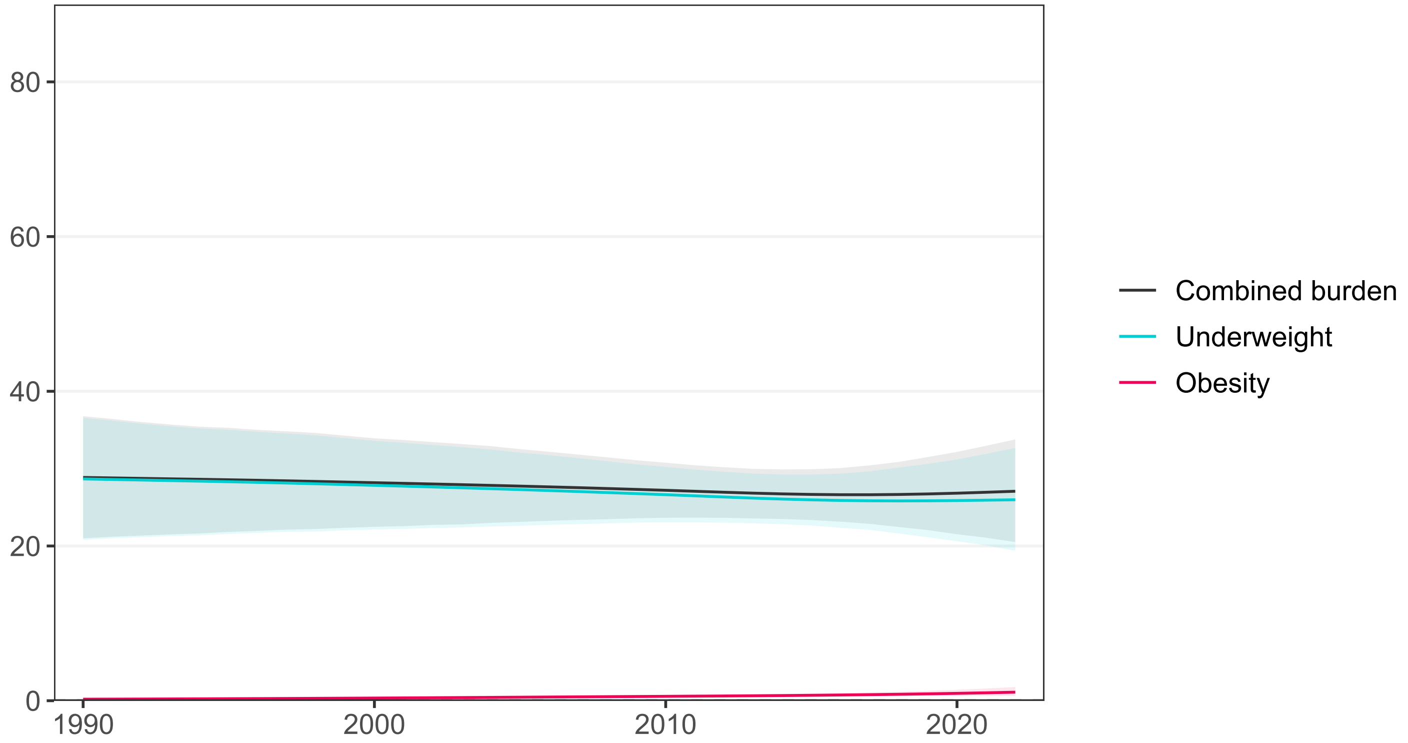

# Fiji

## School-aged children and adolescents

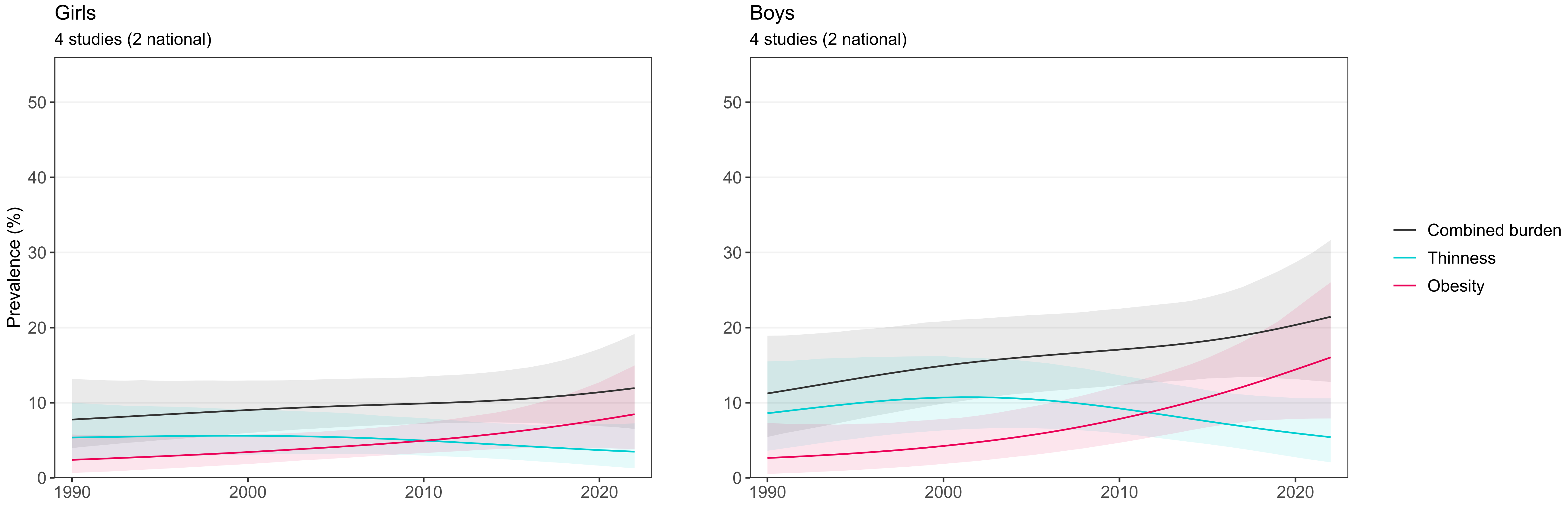


## Adults

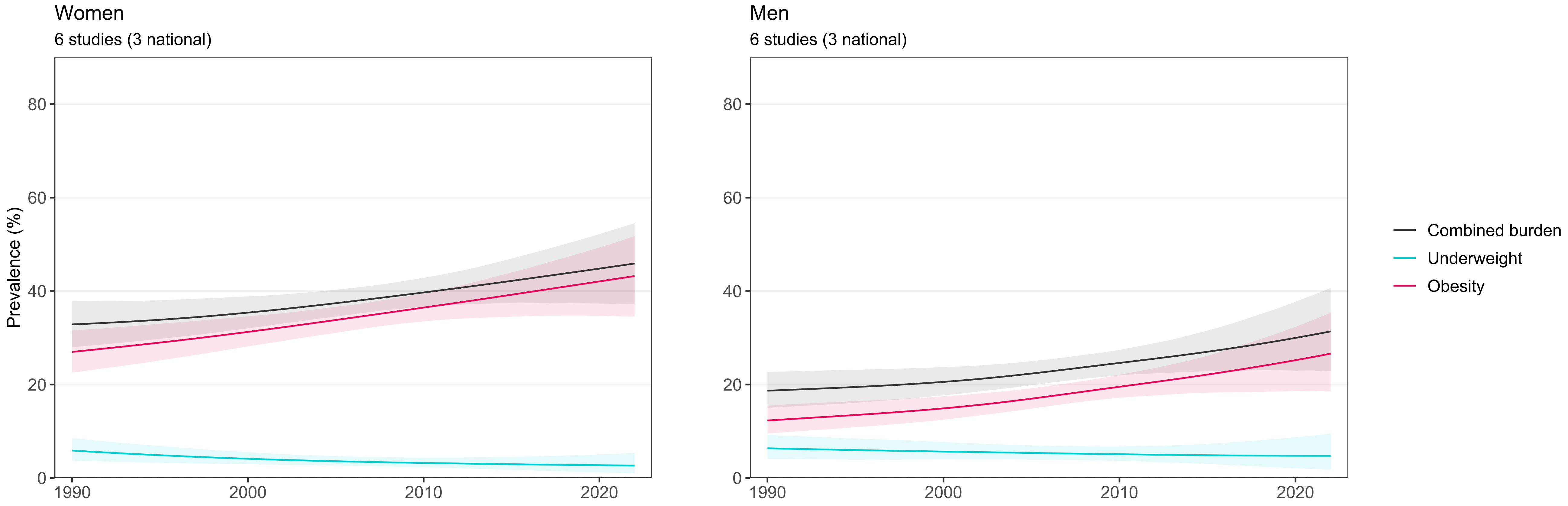

# Finland

## School-aged children and adolescents

Girls

16 studies (14 national)

Boys

16 studies (14 national)

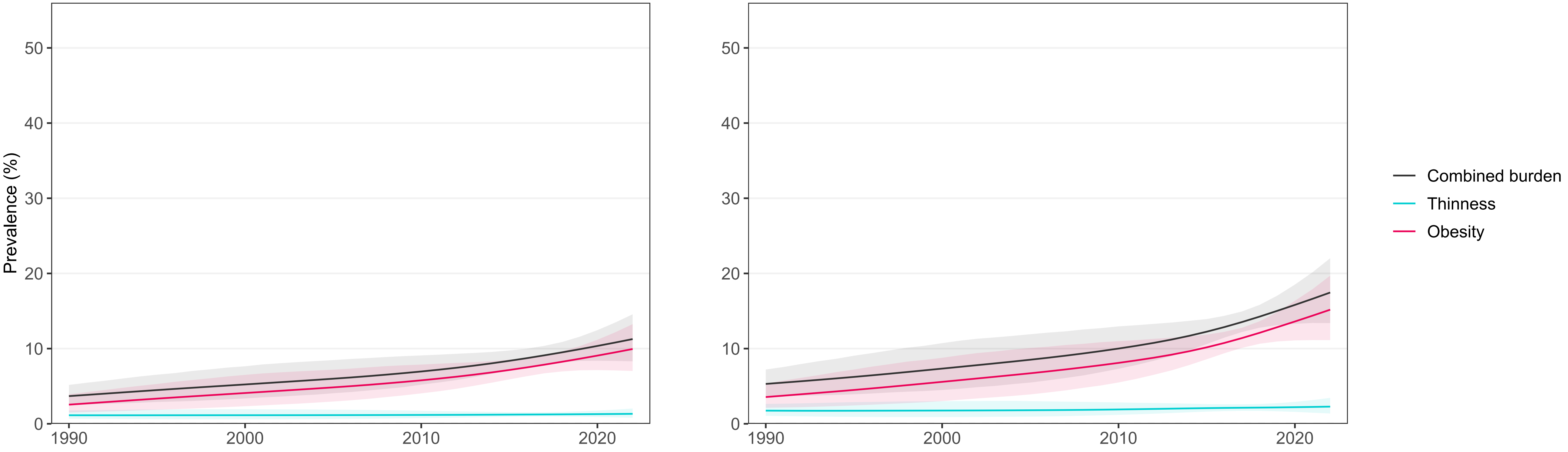


## Adults

Women

39 studies (19 national)

Men

46 studies (20 national)

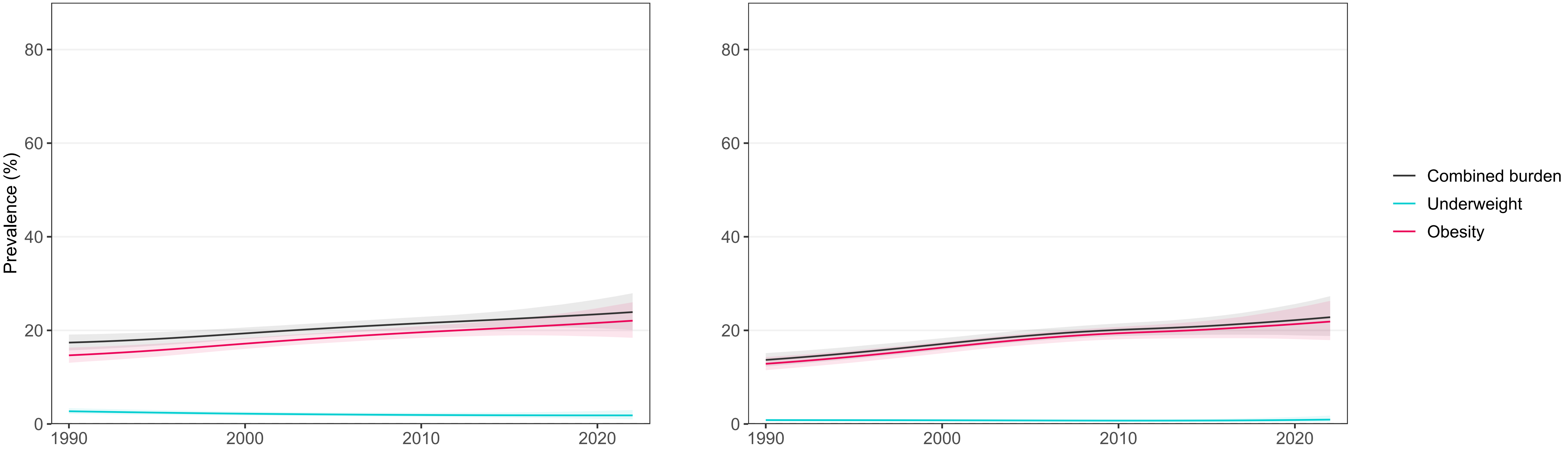

# France

## School-aged children and adolescents

### Girls
19 studies (9 national)

### Boys
18 studies (9 national)

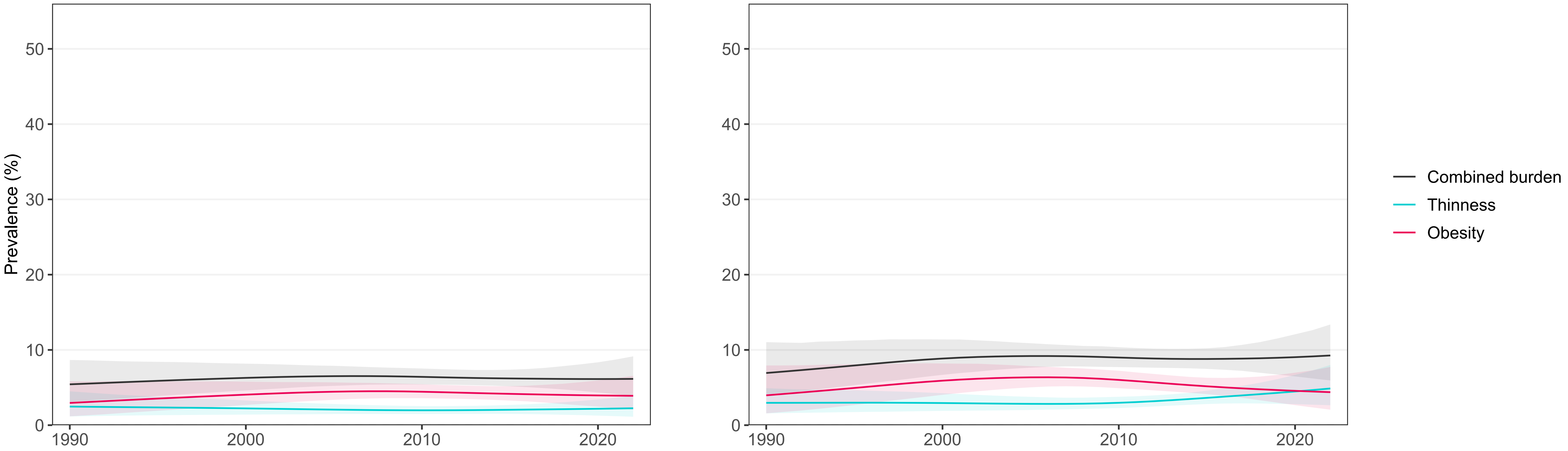


## Adults

### Women
24 studies (6 national)

### Men
25 studies (6 national)

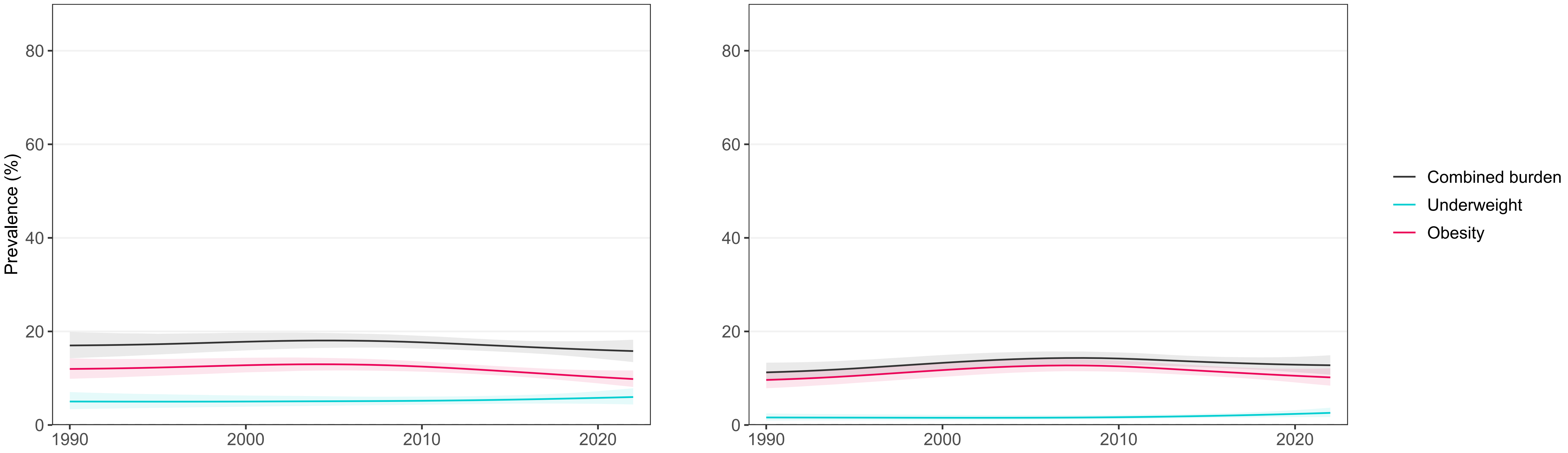

# French Polynesia

## School-aged children and adolescents

### Girls

2 studies (2 national)

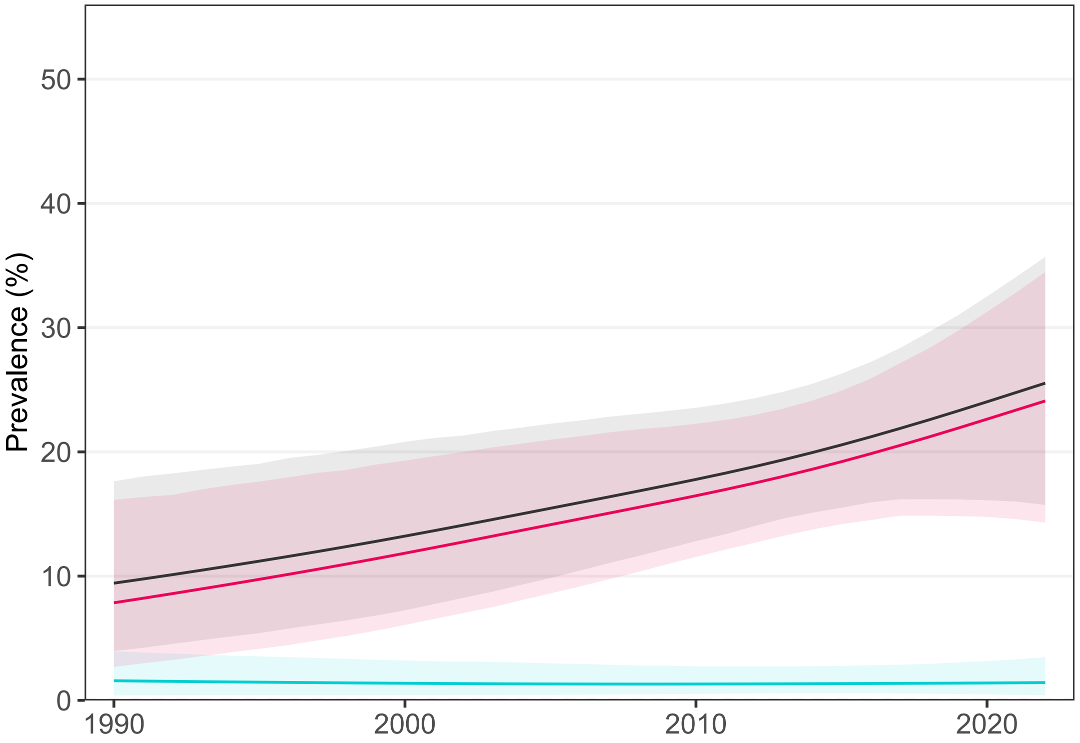


### Boys

2 studies (2 national)

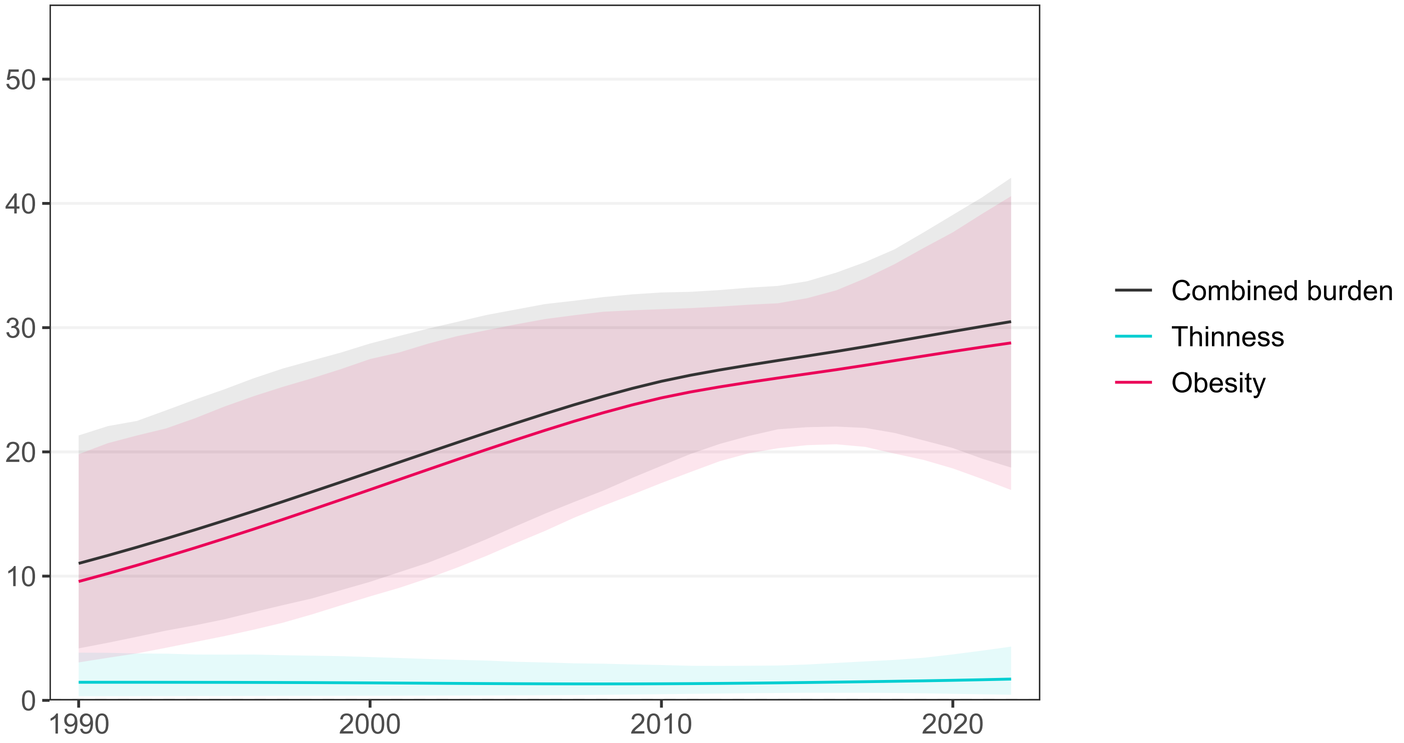


## Adults

### Women

1 study (1 national)

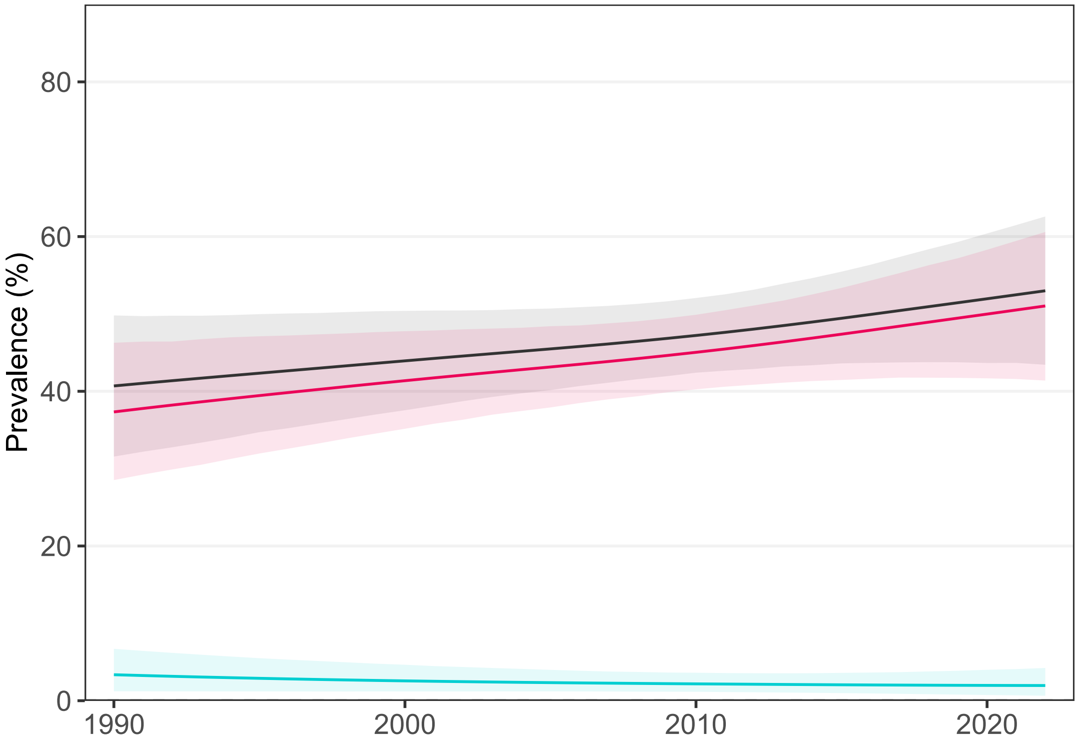


### Men

1 study (1 national)

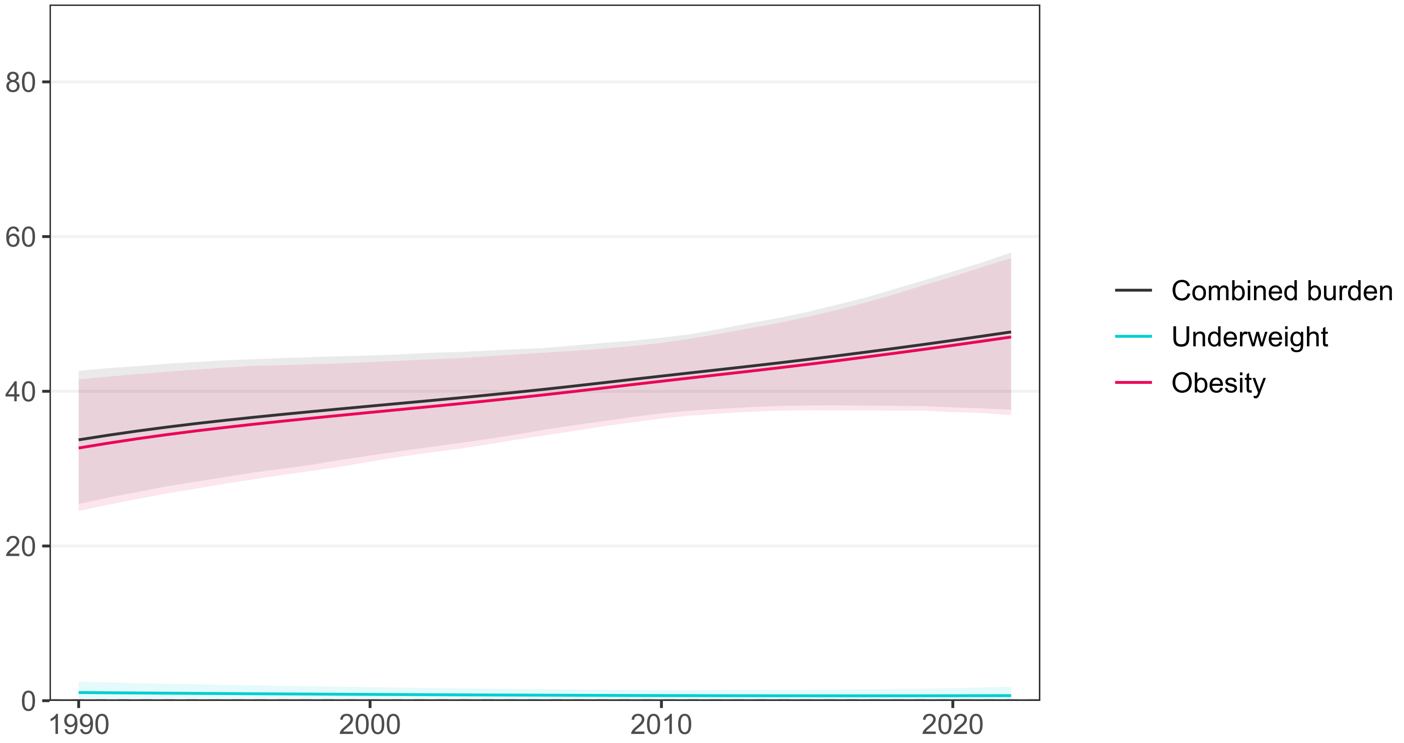

# Gabon

## School-aged children and adolescents

### Girls

3 studies (2 national)

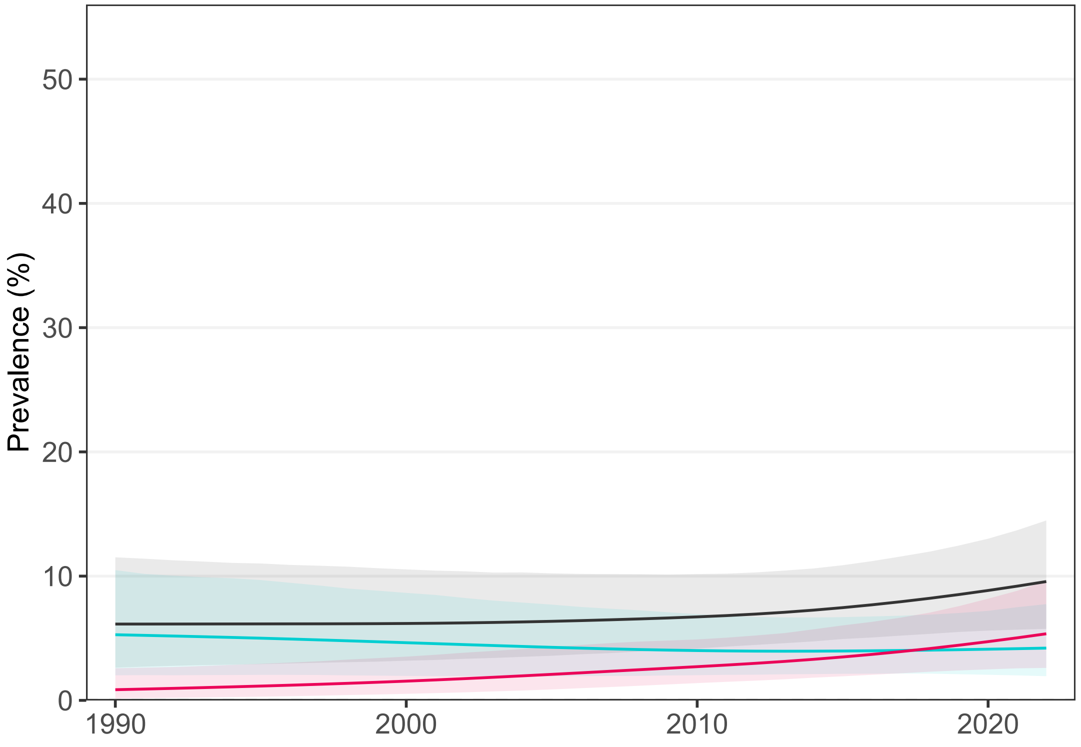


### Boys

1 study (0 national)

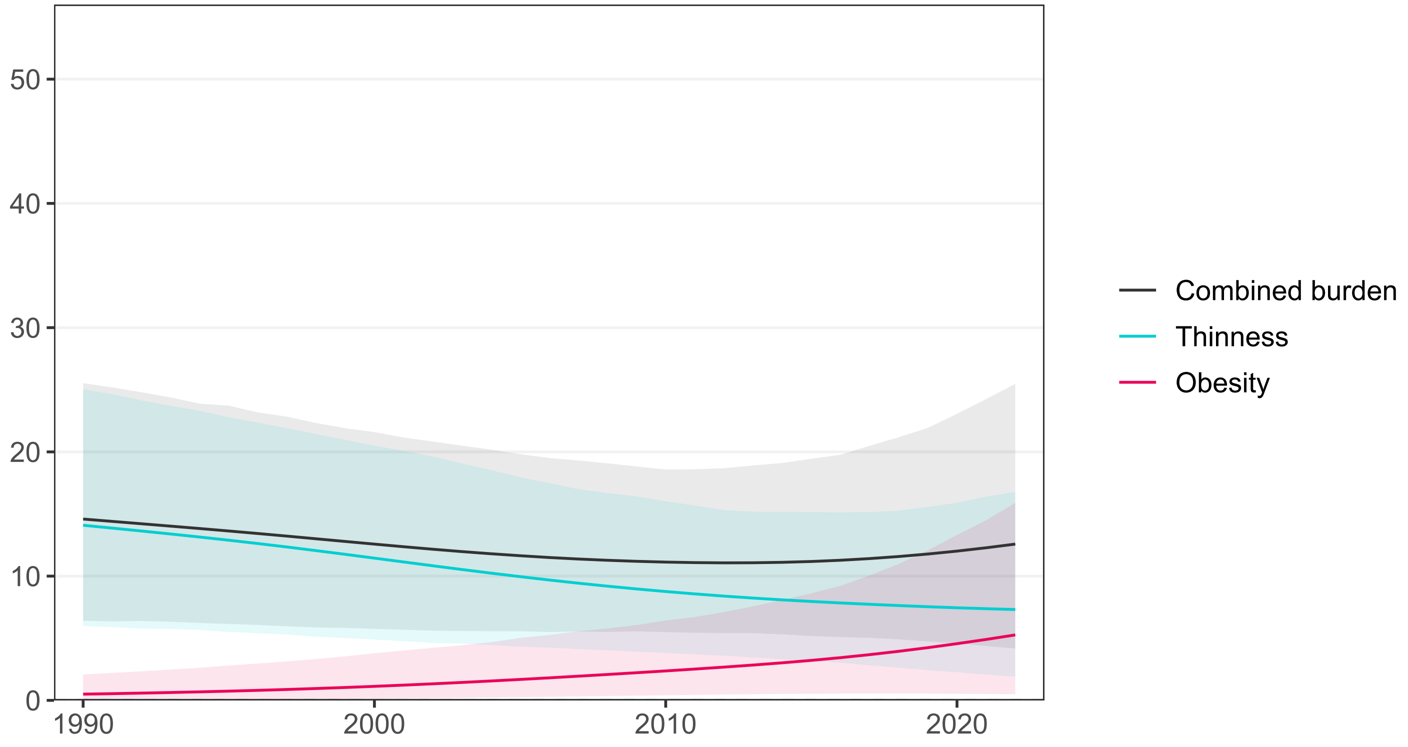


## Adults

### Women

4 studies (3 national)

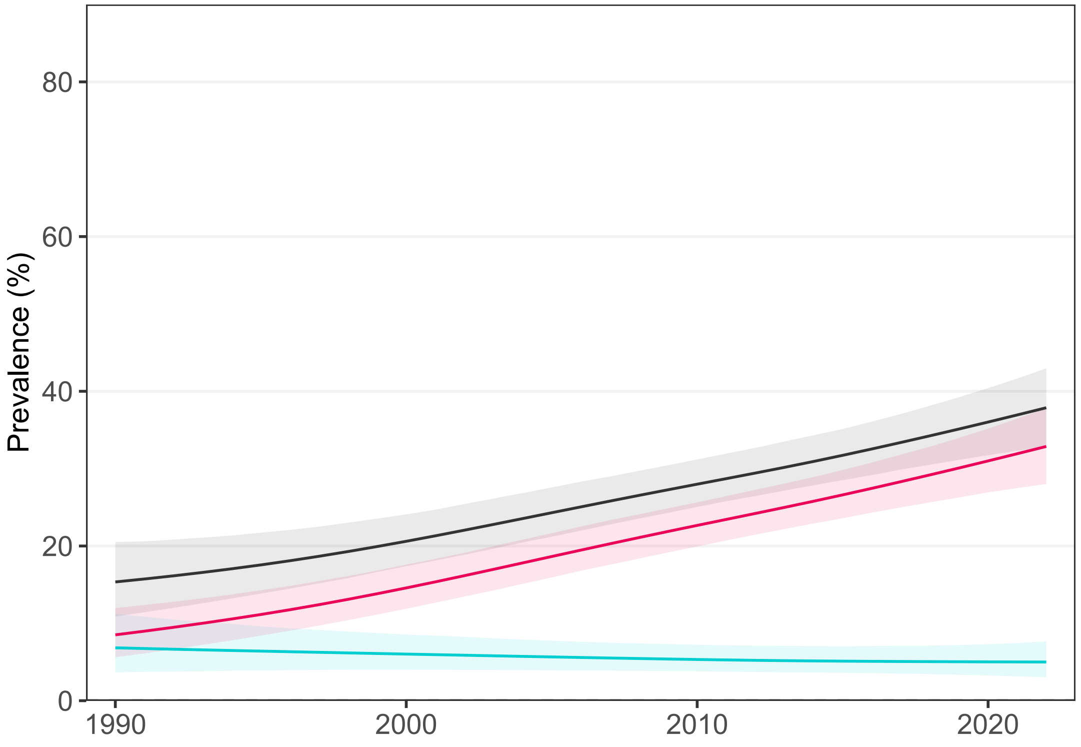


### Men

1 study (0 national)

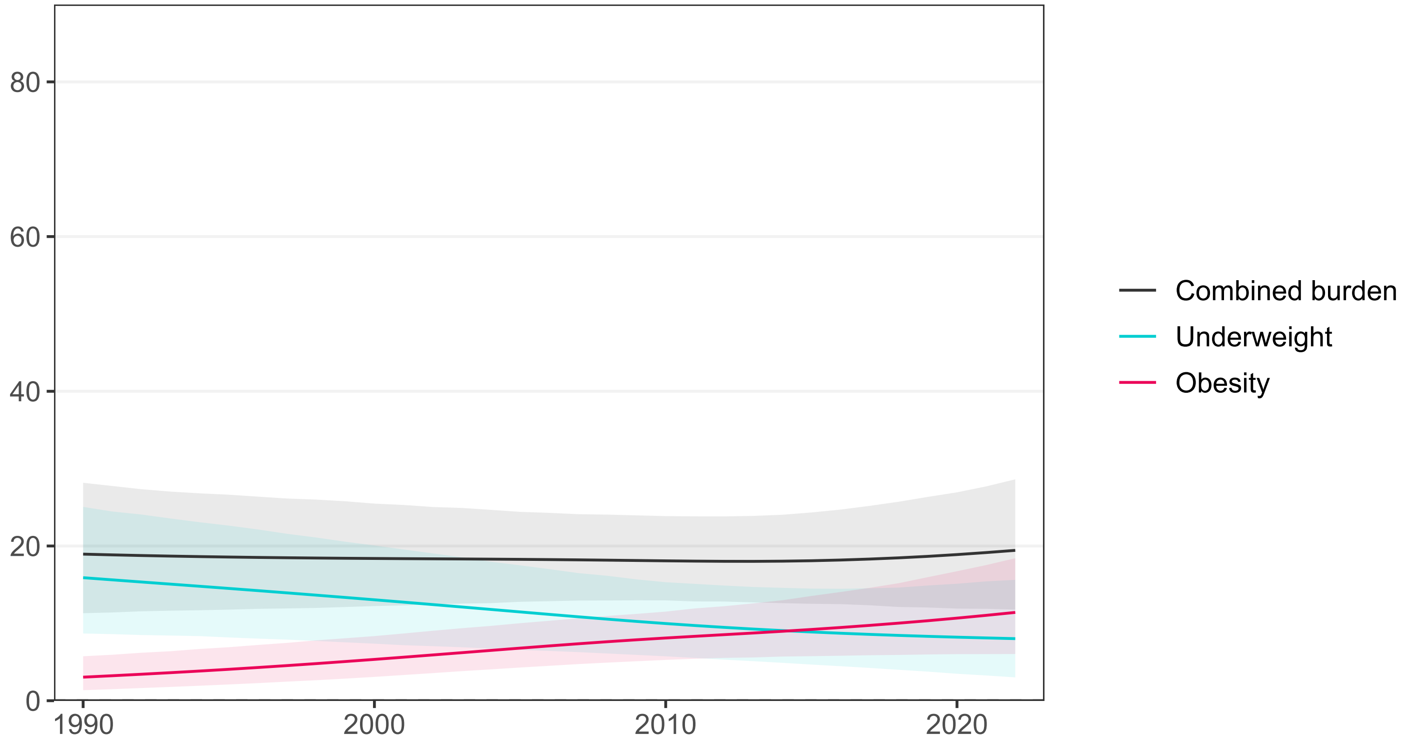

# Gambia

## School-aged children and adolescents

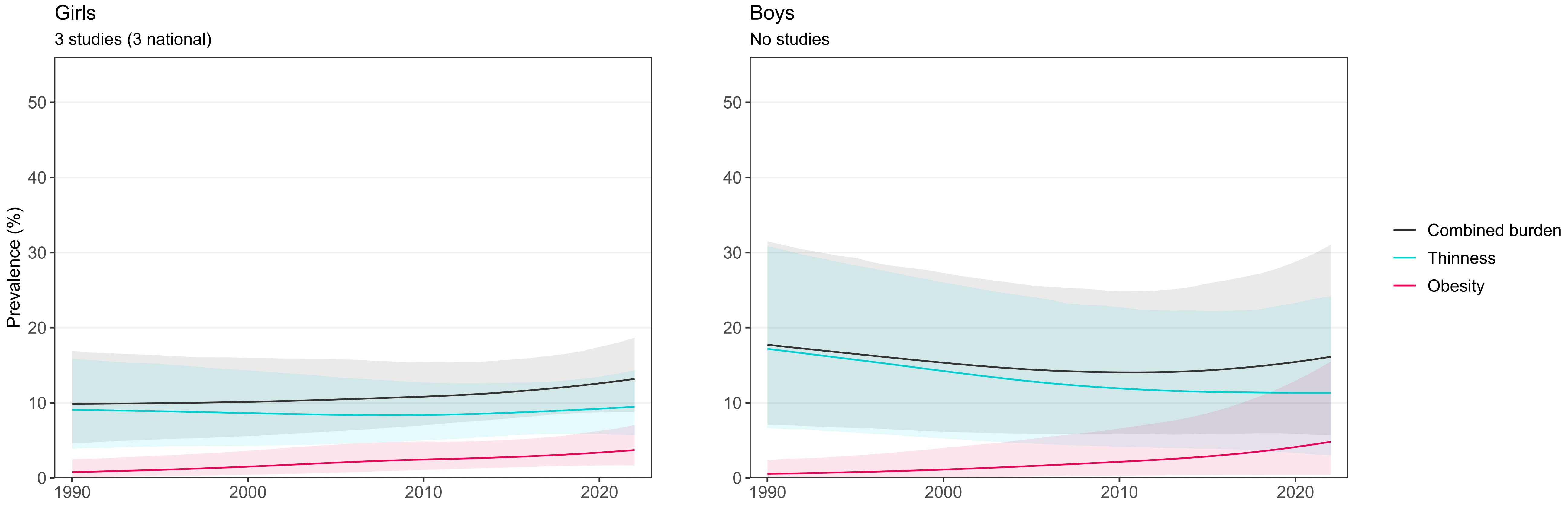


## Adults

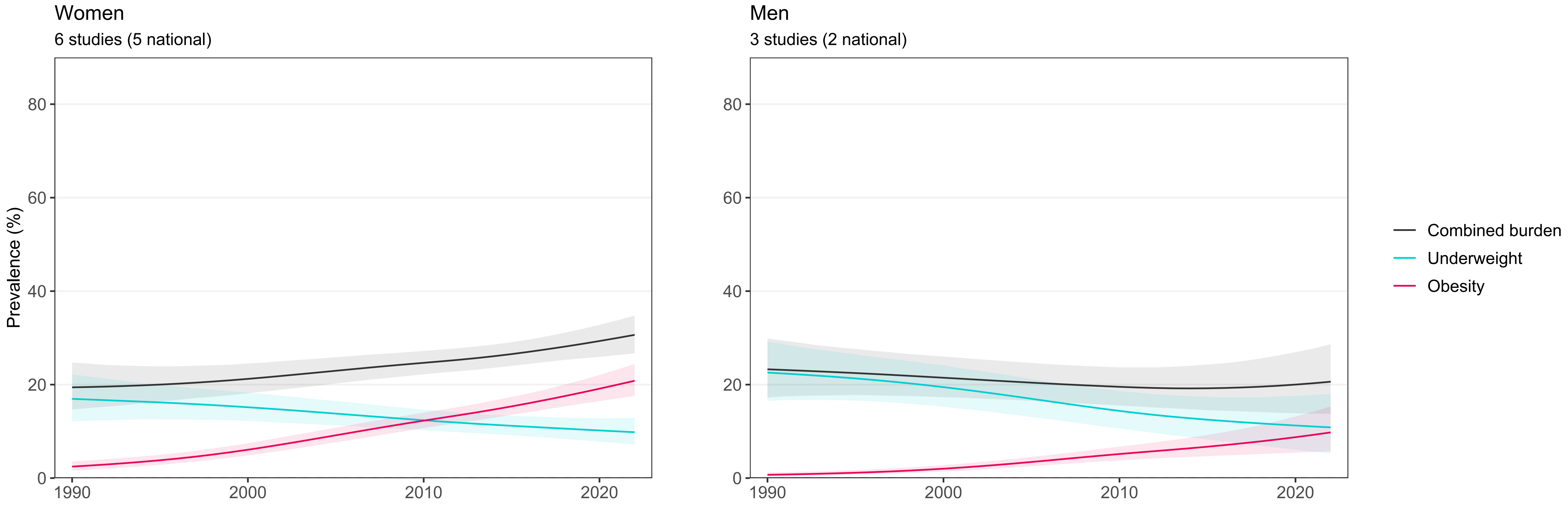

# Georgia

## School-aged children and adolescents

### Girls

4 studies (4 national)

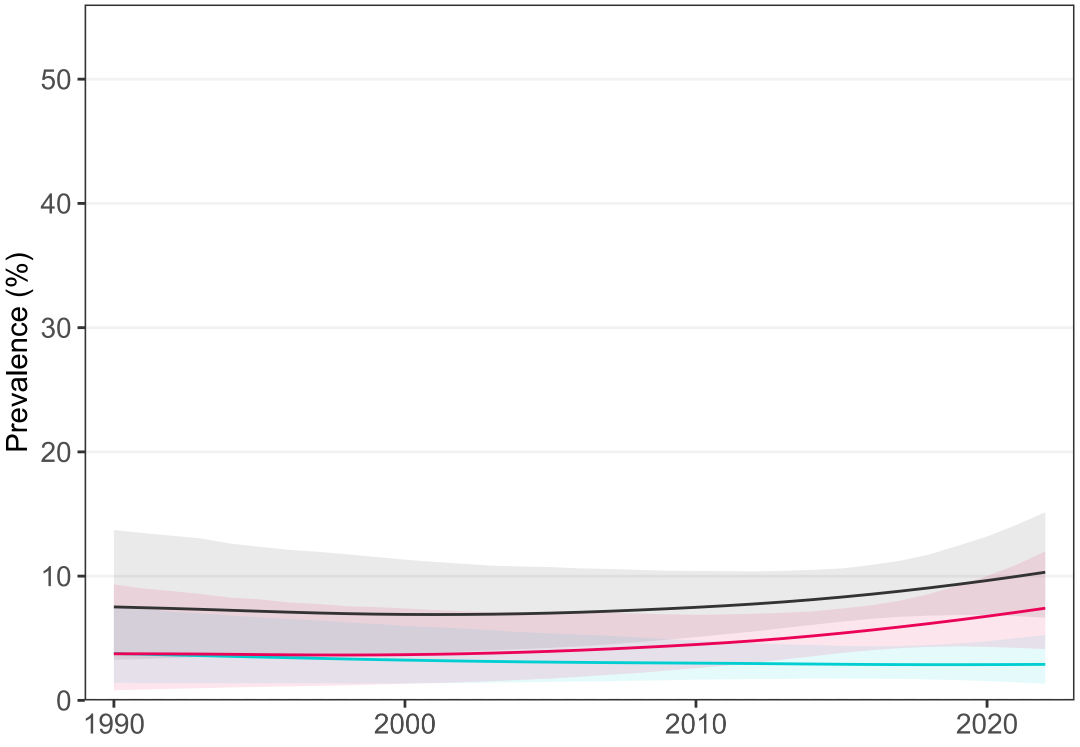


### Boys

4 studies (4 national)

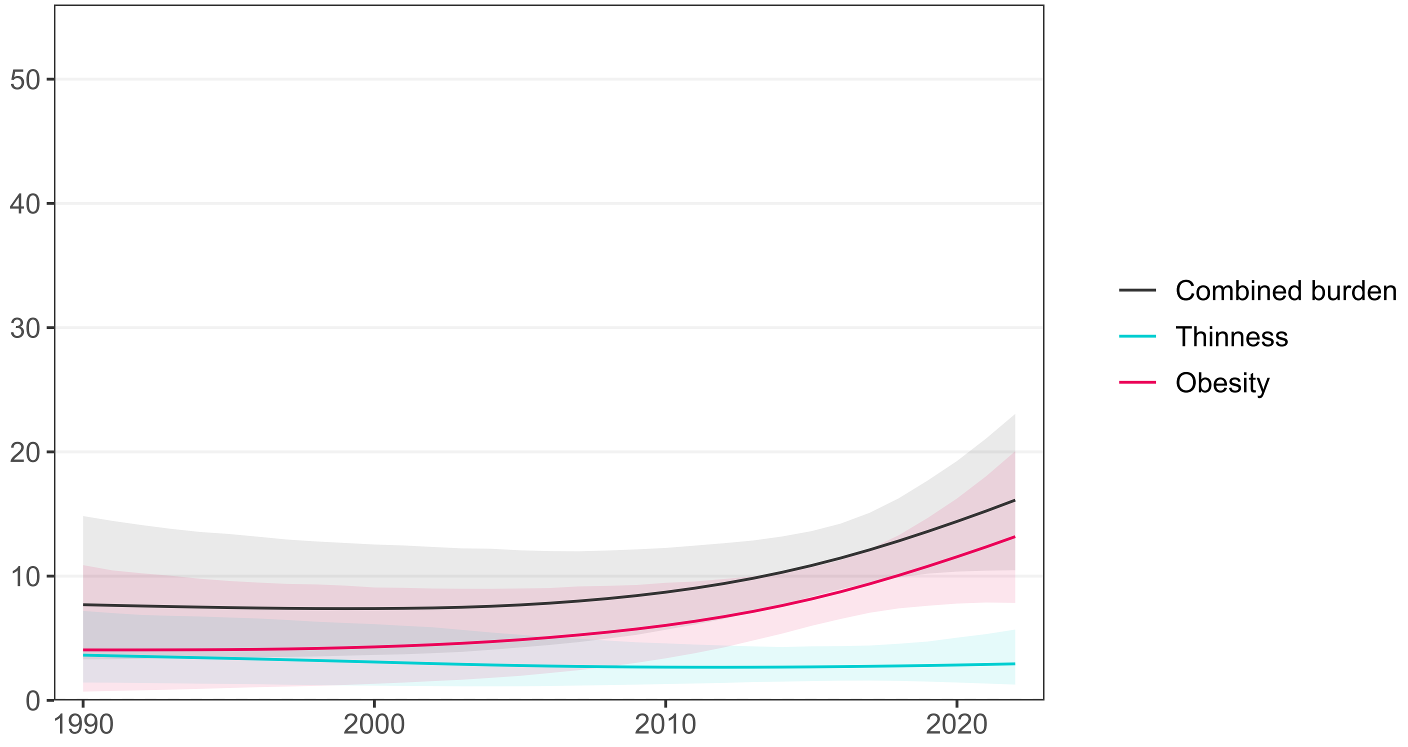


## Adults

### Women

2 studies (2 national)

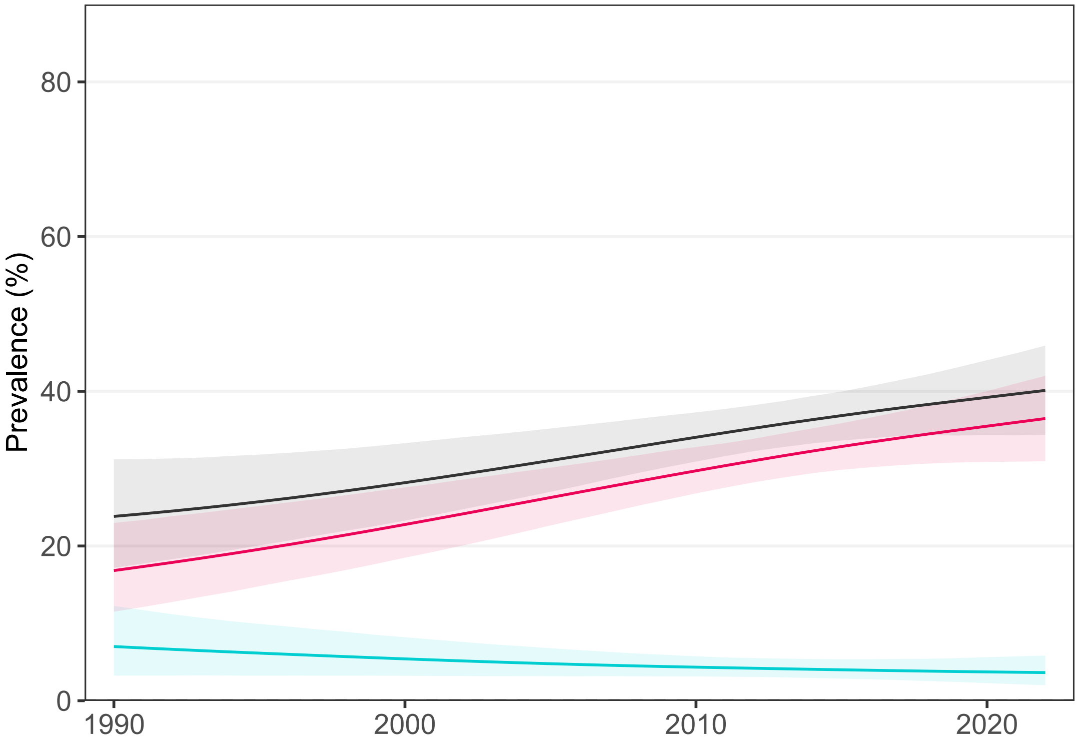


### Men

2 studies (2 national)

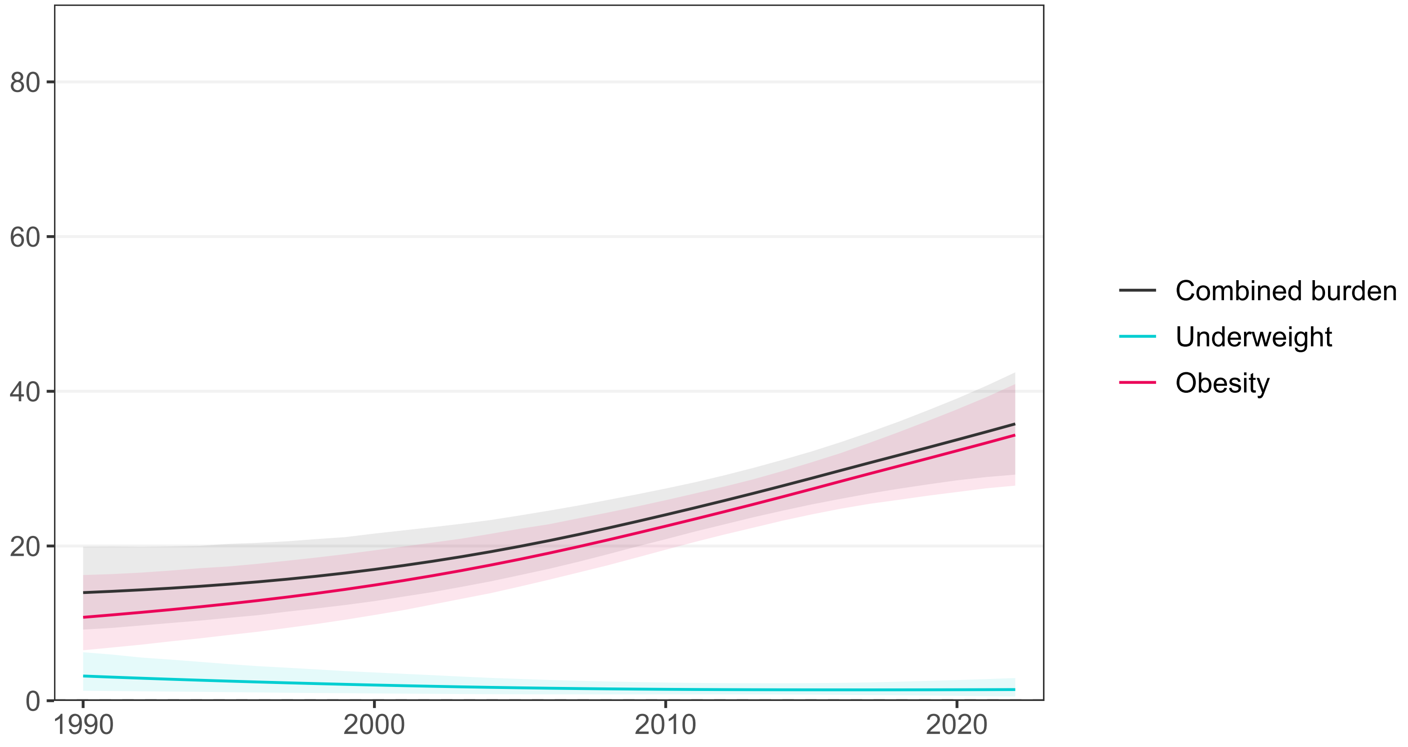

# Germany

## School-aged children and adolescents

### Girls

10 studies (4 national)

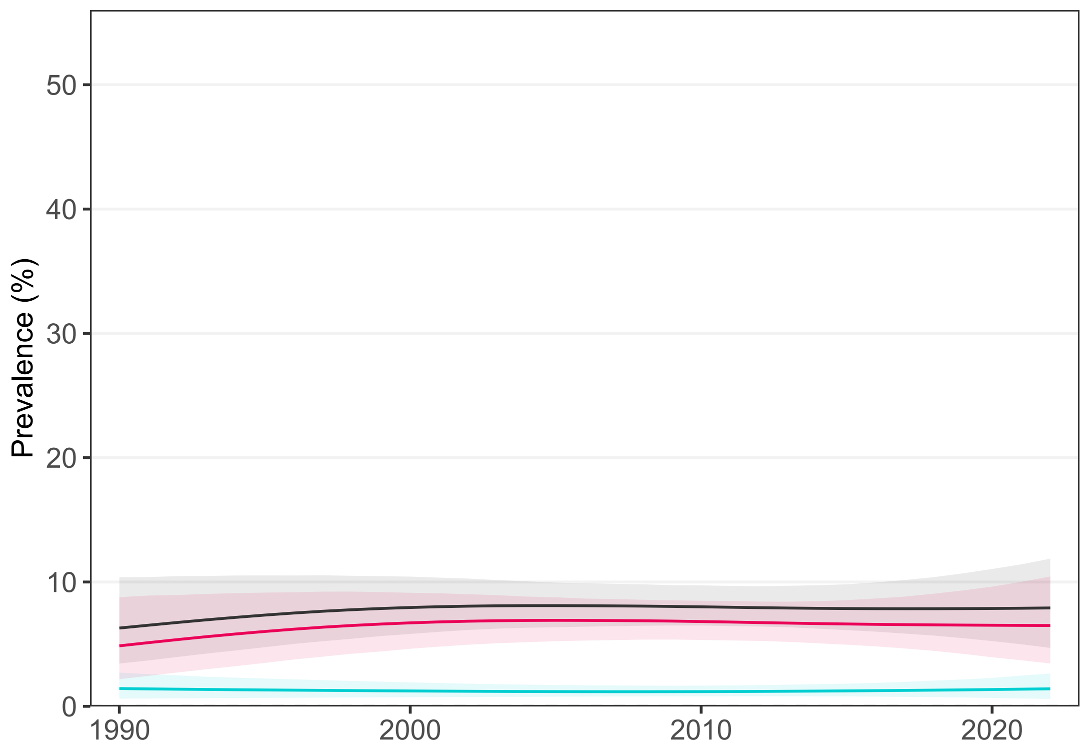


### Boys

29 studies (16 national)

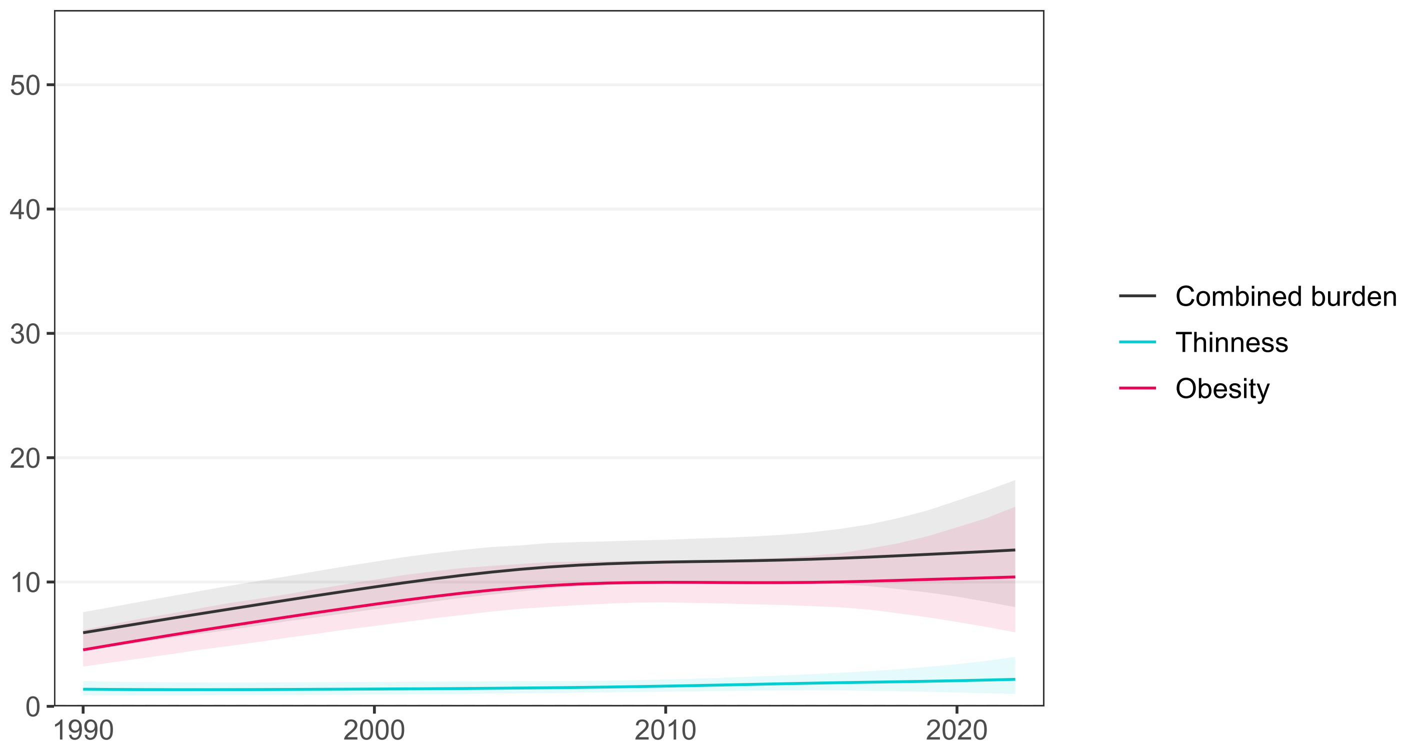


## Adults

### Women

63 studies (2 national)

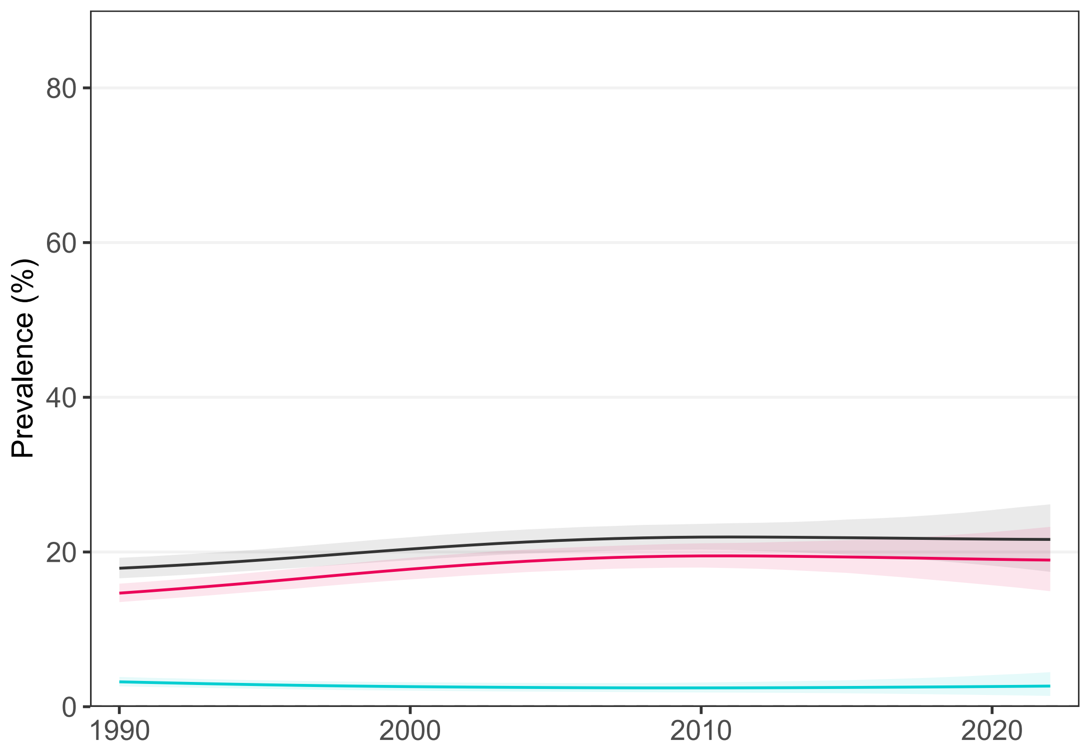


### Men

82 studies (14 national)

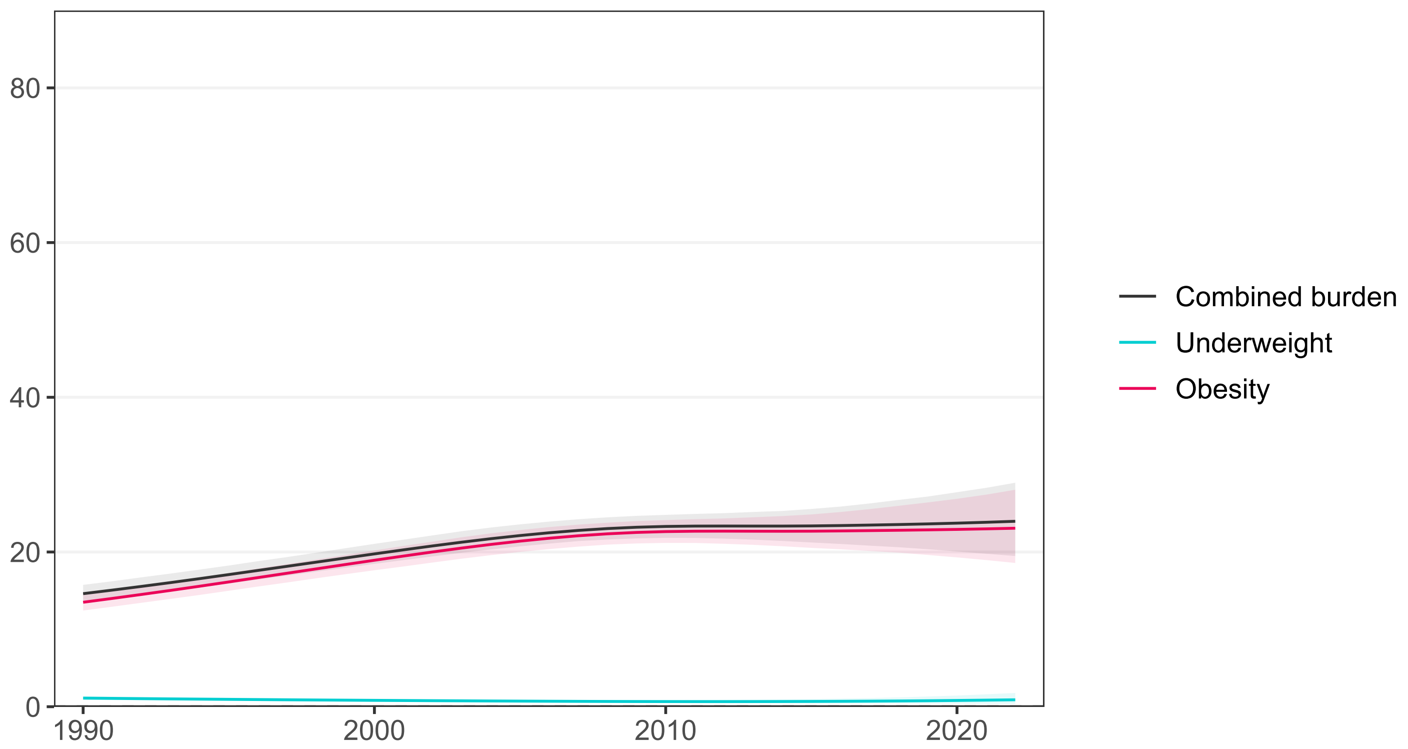

# Ghana

## School-aged children and adolescents

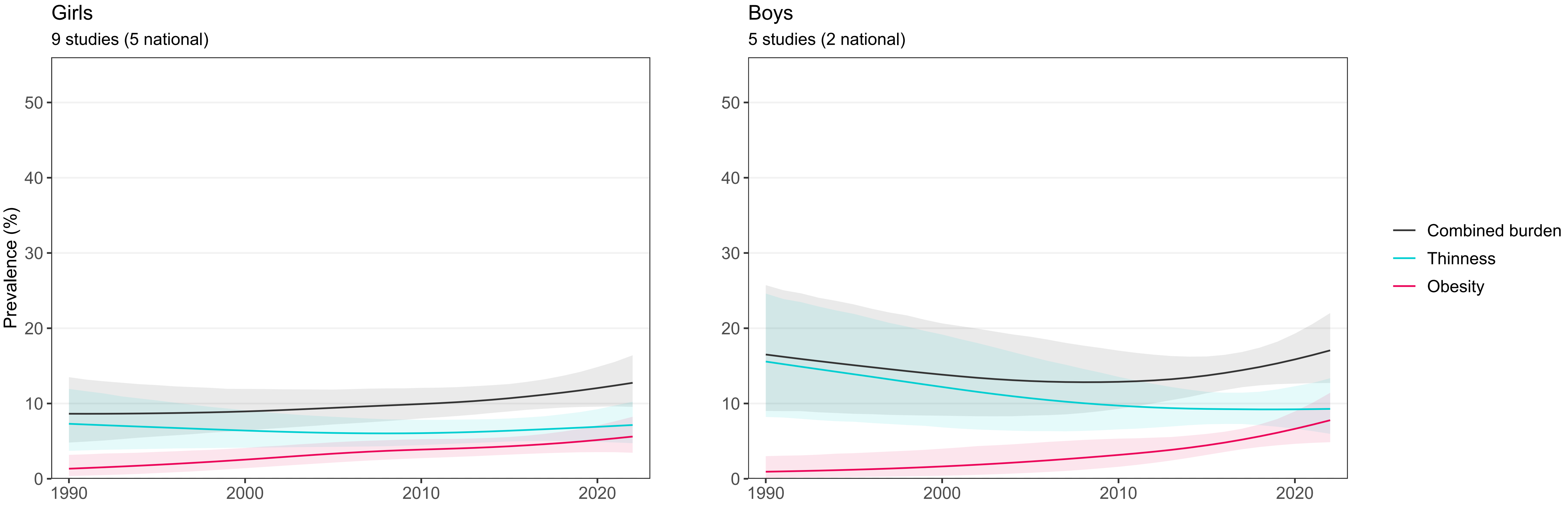


## Adults

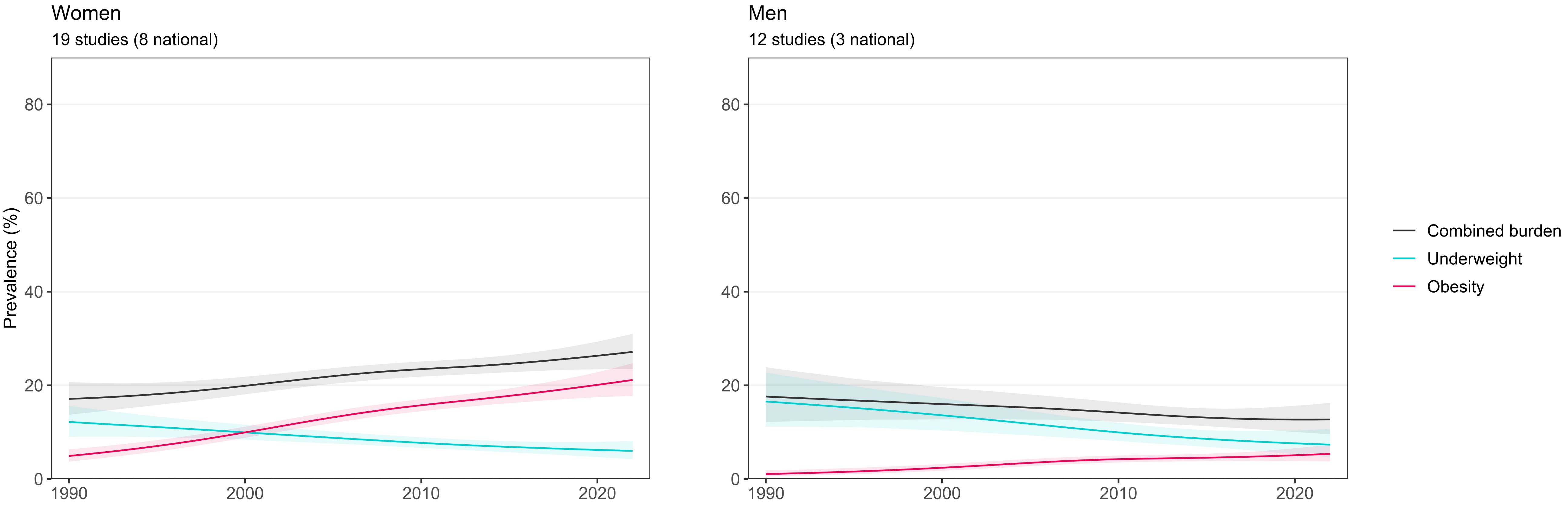

# Greece

## School-aged children and adolescents

### Girls

38 studies (24 national)

### Boys

37 studies (24 national)

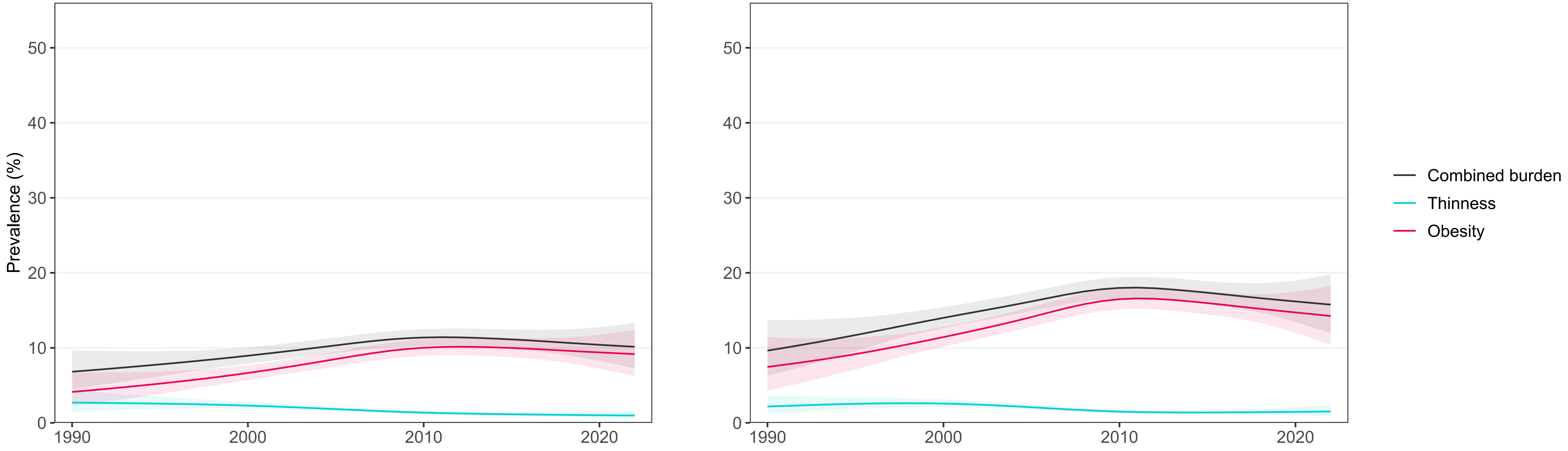


## Adults

### Women

13 studies (6 national)

### Men

13 studies (5 national)

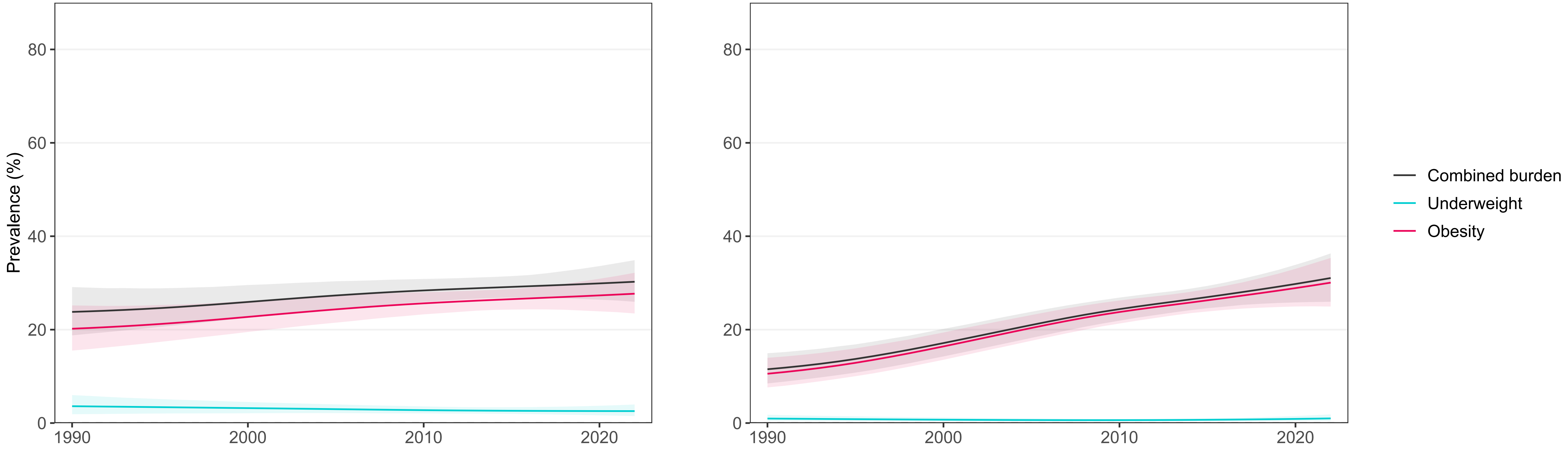

# Greenland

## School-aged children and adolescents

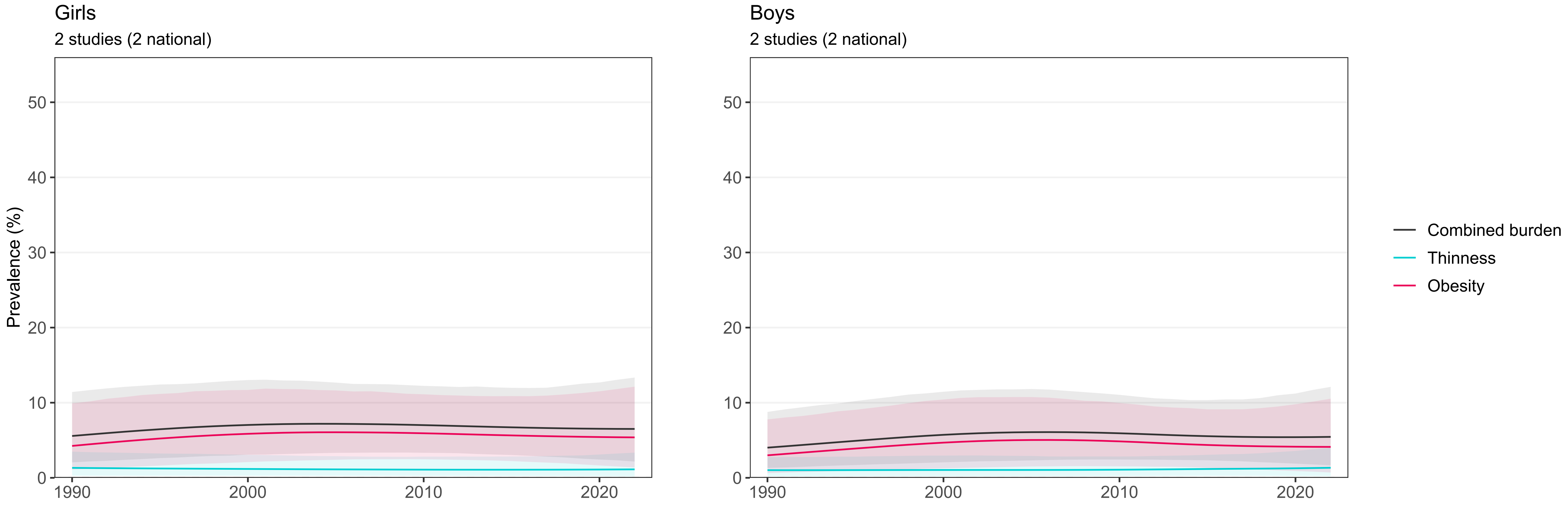


## Adults

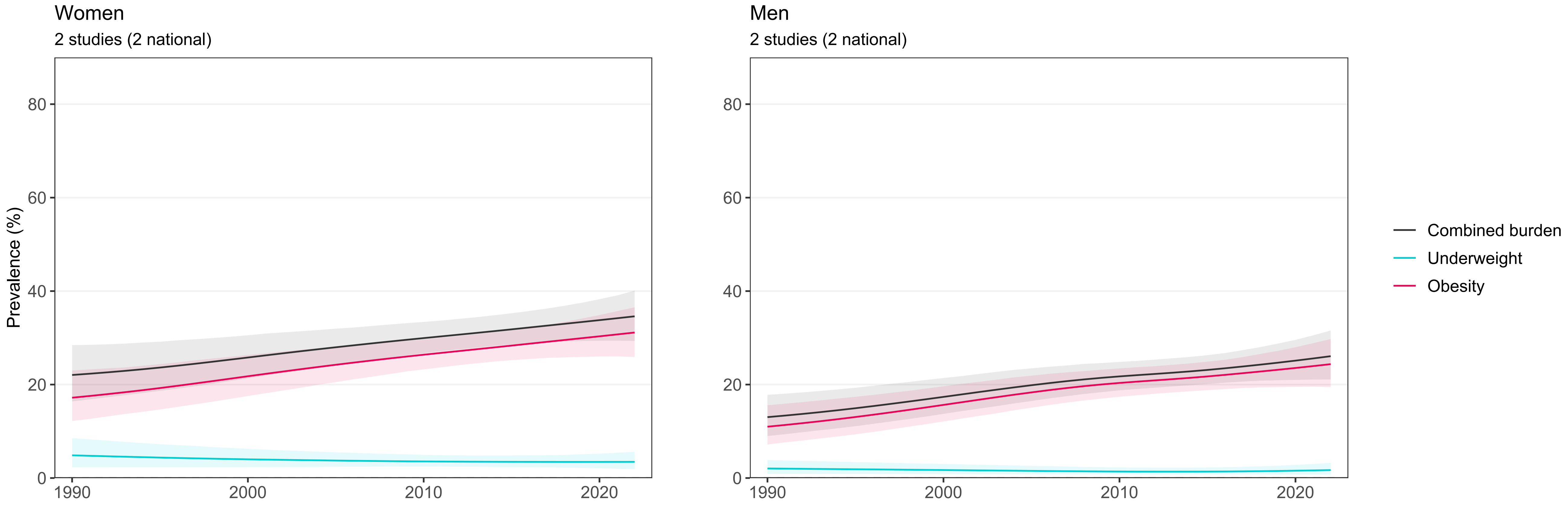

# Grenada

## School-aged children and adolescents

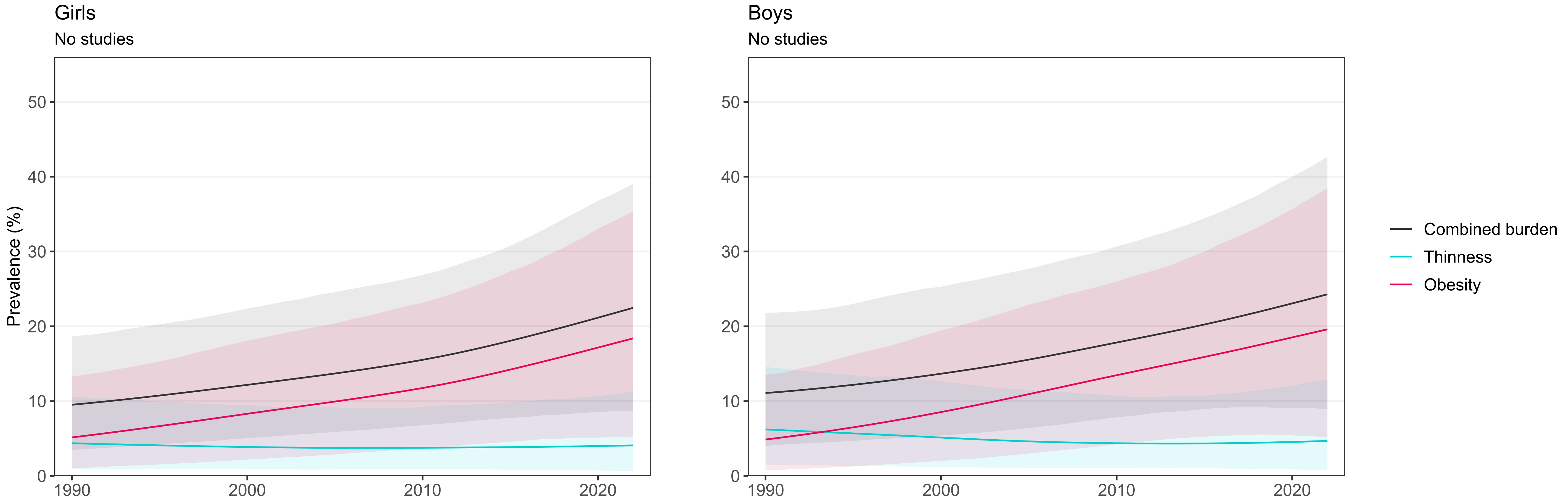


## Adults

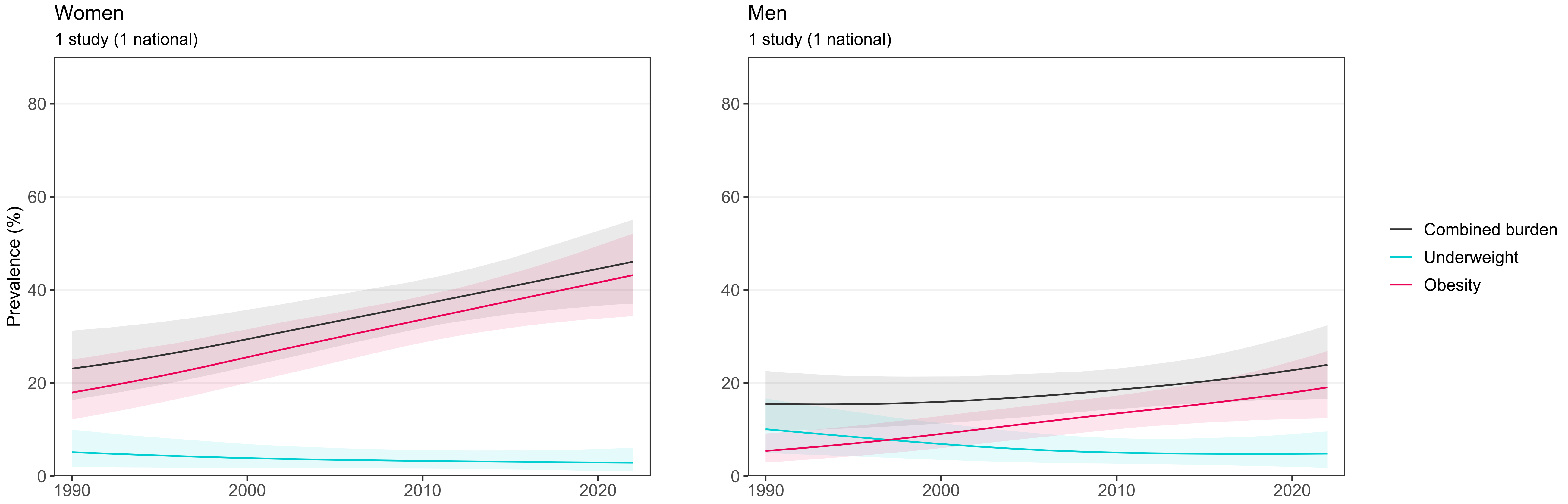

# Guatemala

## School-aged children and adolescents

### Girls
13 studies (10 national)

### Boys
7 studies (4 national)

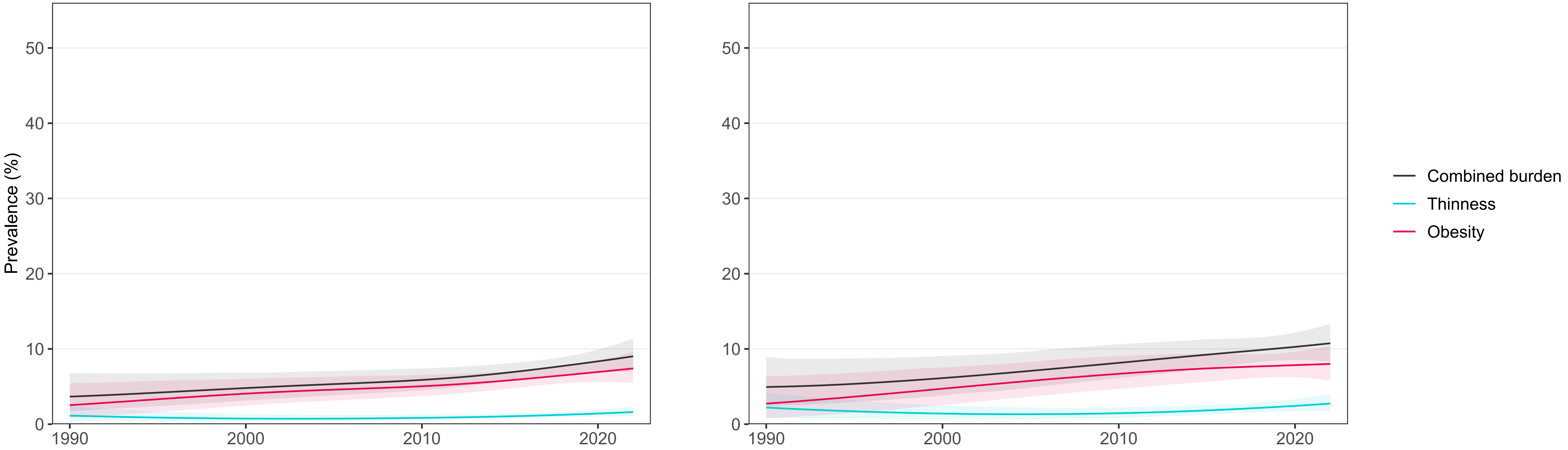


## Adults

### Women
15 studies (10 national)

### Men
7 studies (2 national)

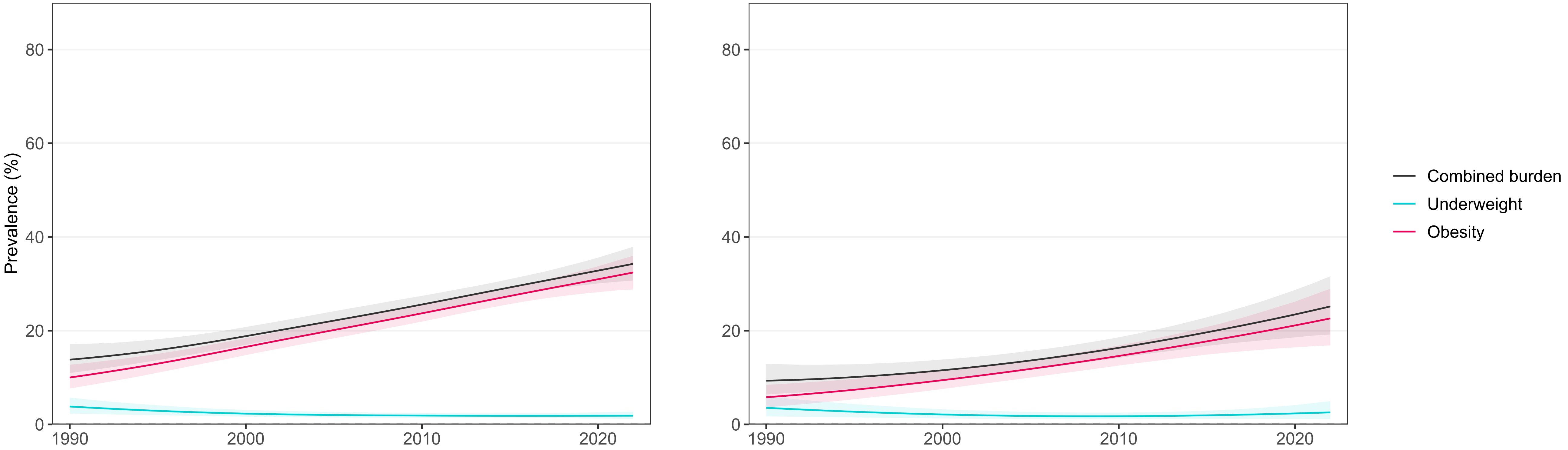

# Guinea

## School-aged children and adolescents

### Girls

4 studies (3 national)

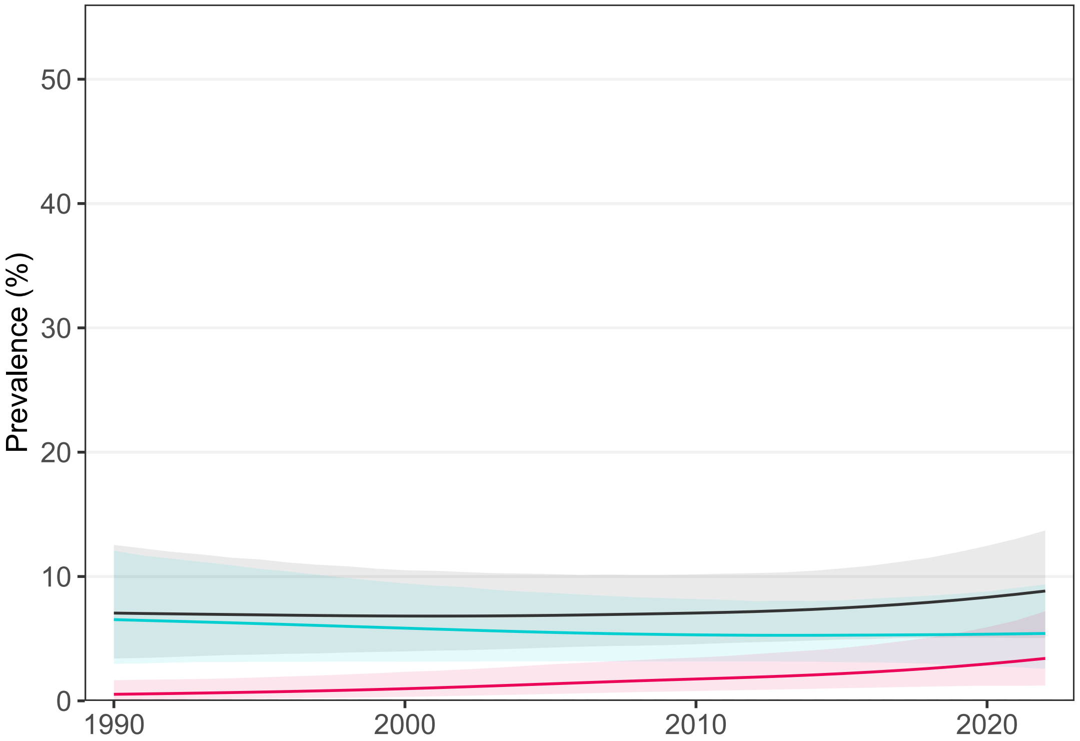


### Boys

1 study (0 national)

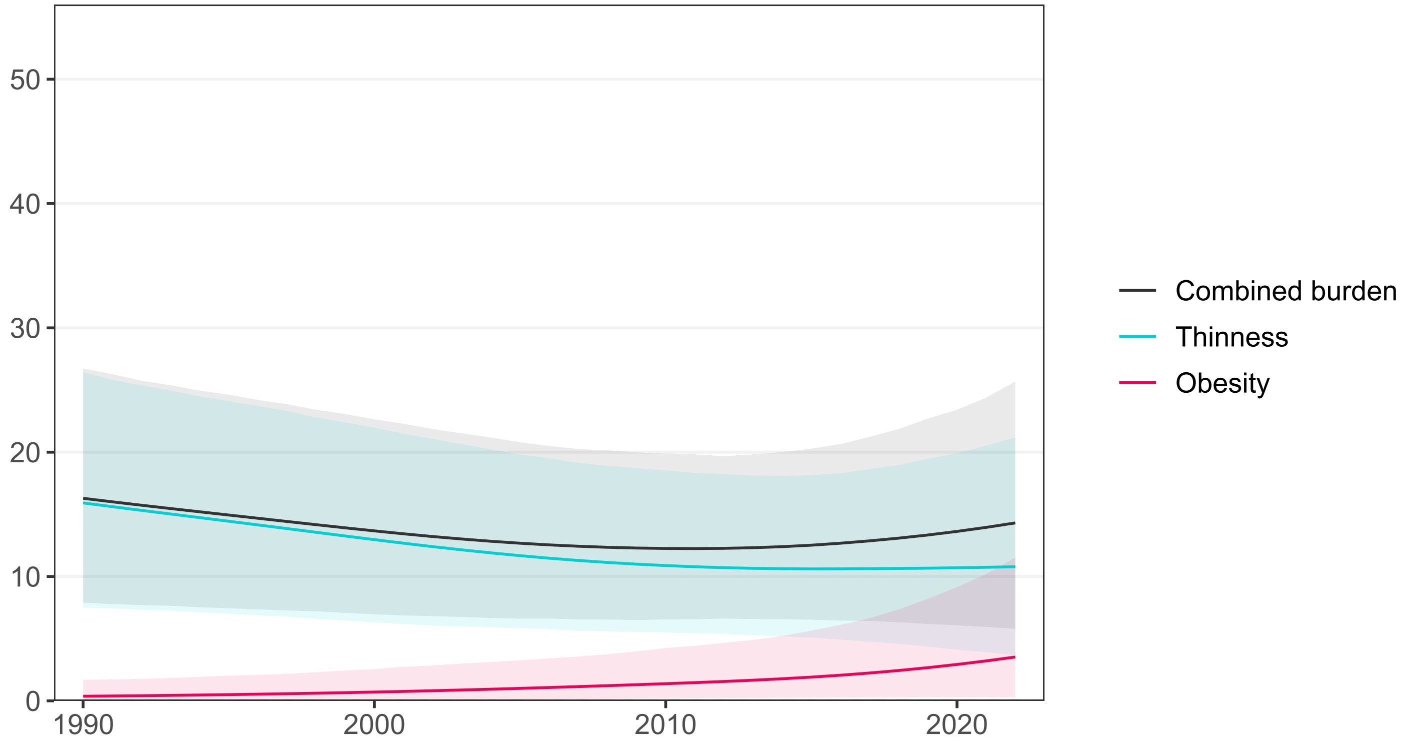


## Adults

### Women

5 studies (4 national)

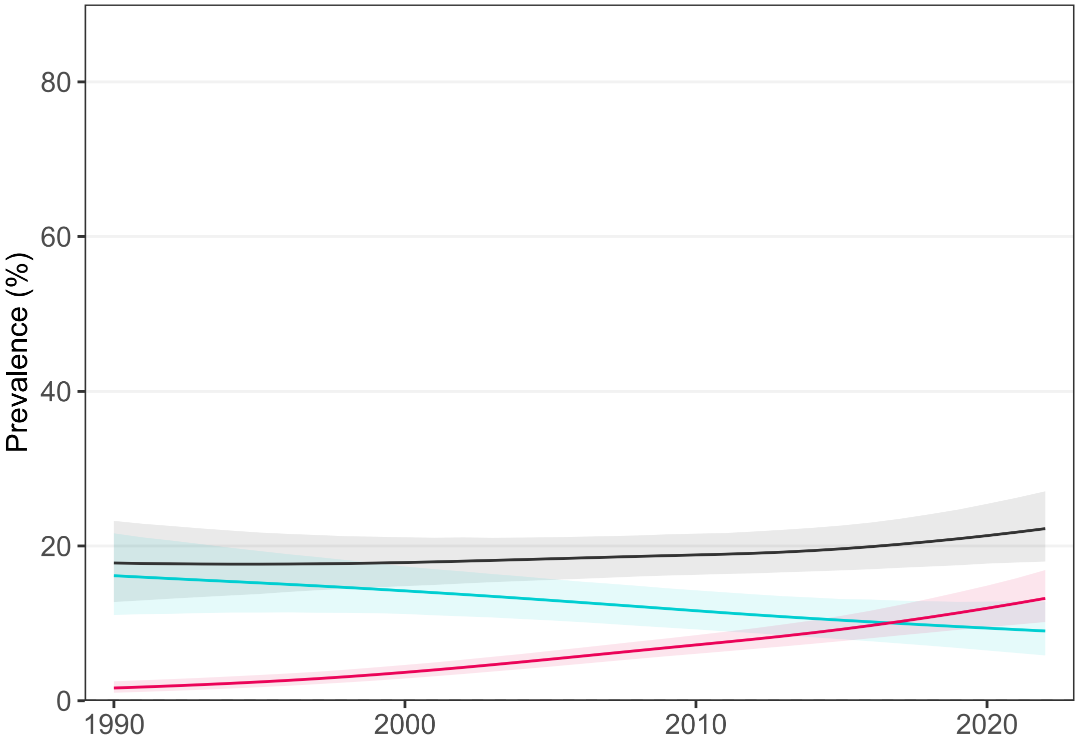


### Men

1 study (0 national)

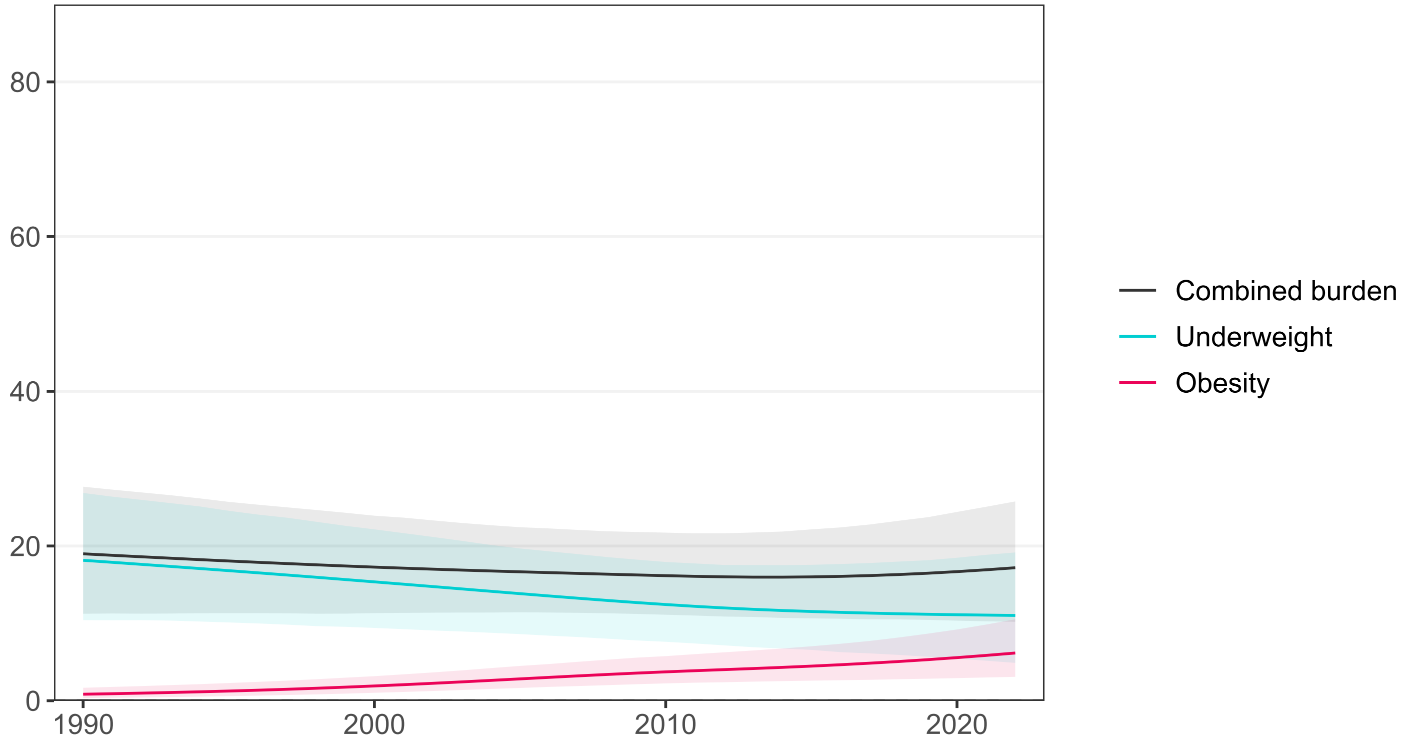

# Guinea Bissau

## School-aged children and adolescents

### Girls

1 study (1 national)

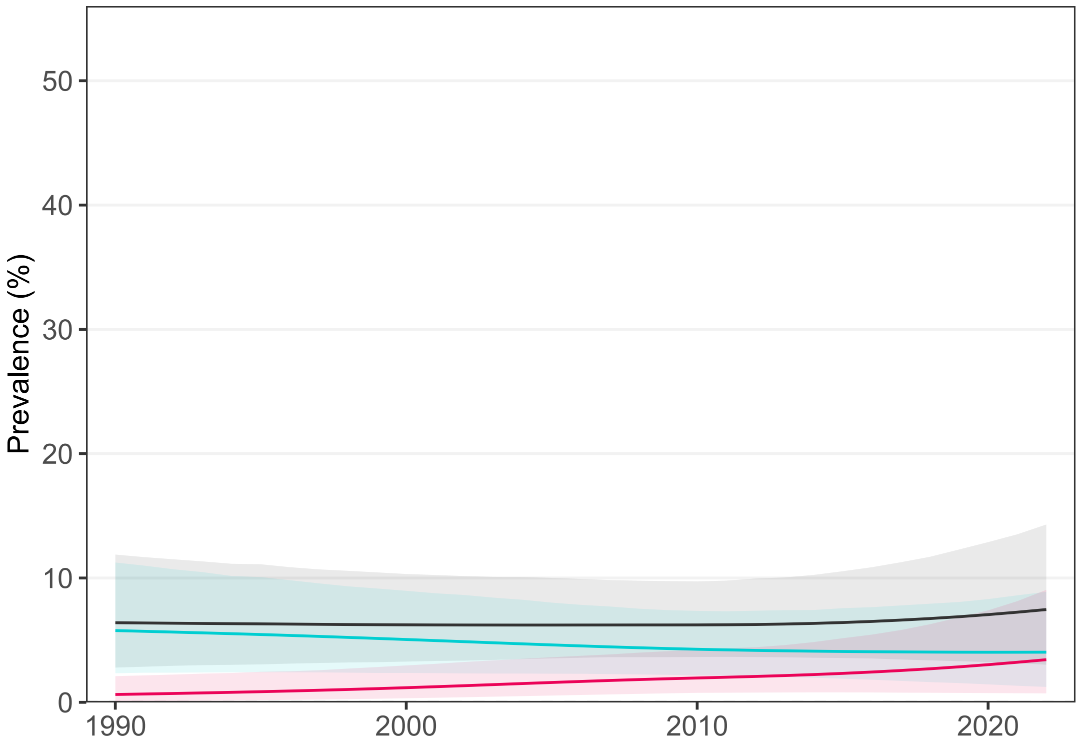


### Boys

No studies

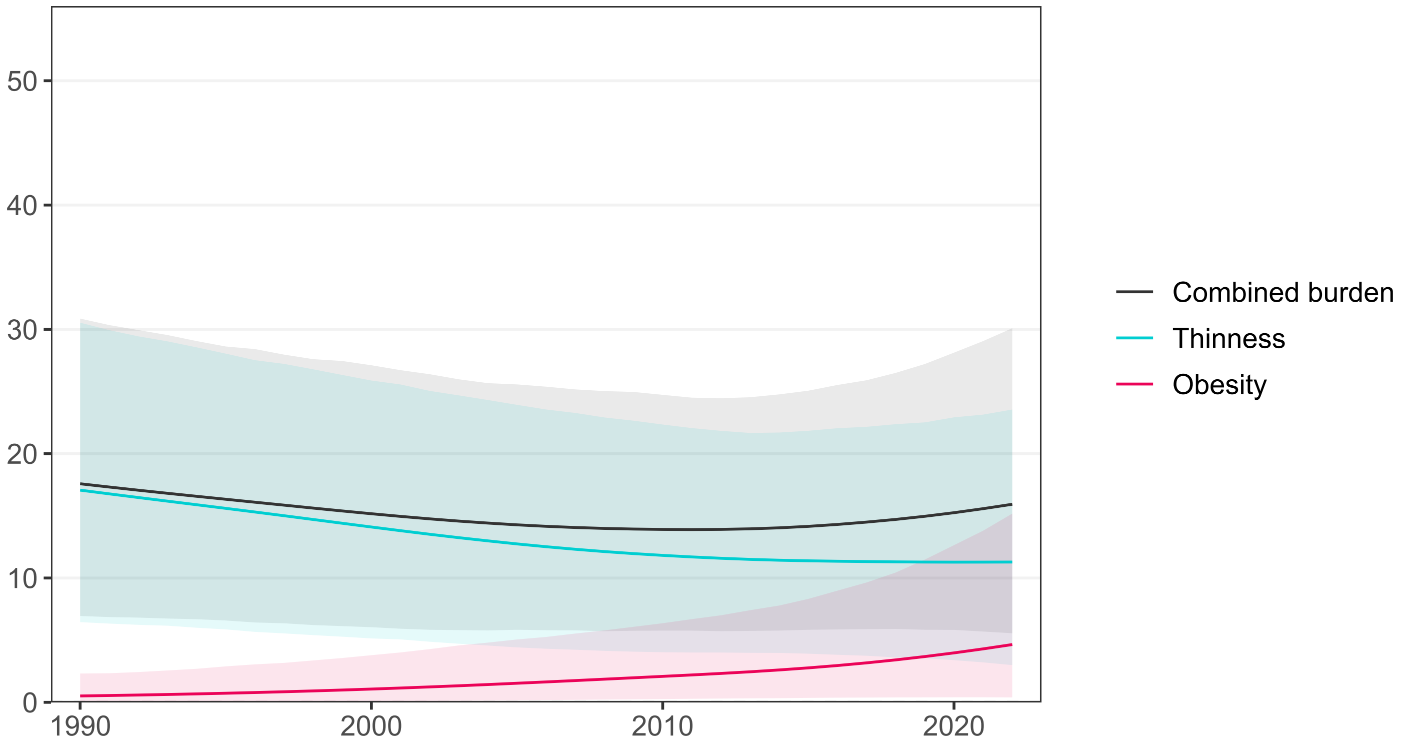


## Adults

### Women

1 study (1 national)

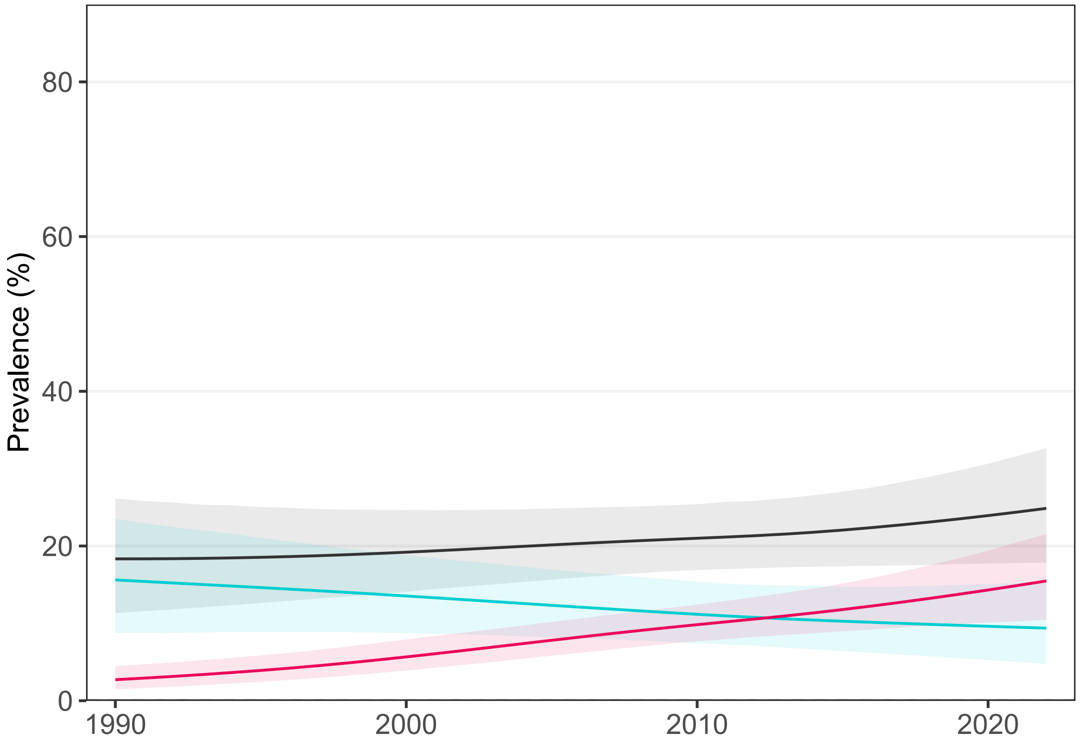


### Men

No studies

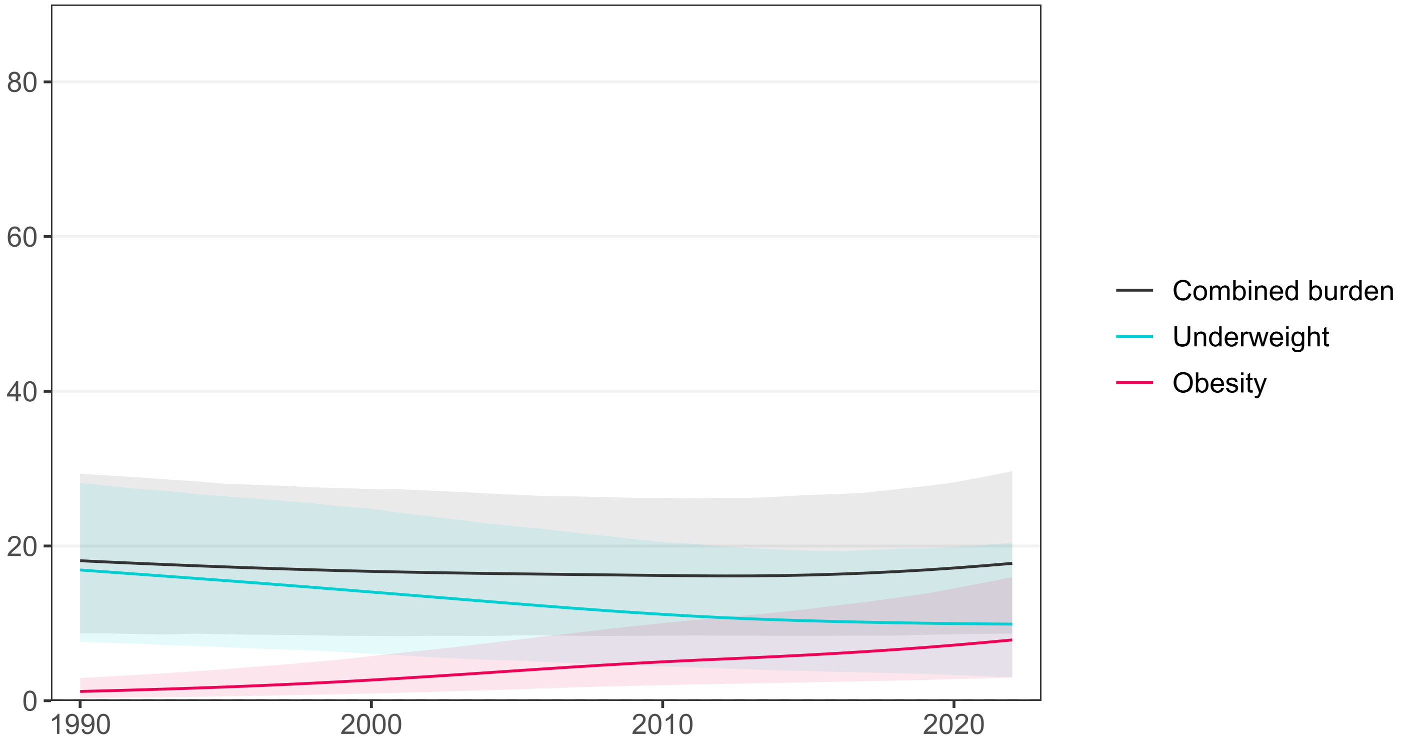

# Guyana

## School-aged children and adolescents

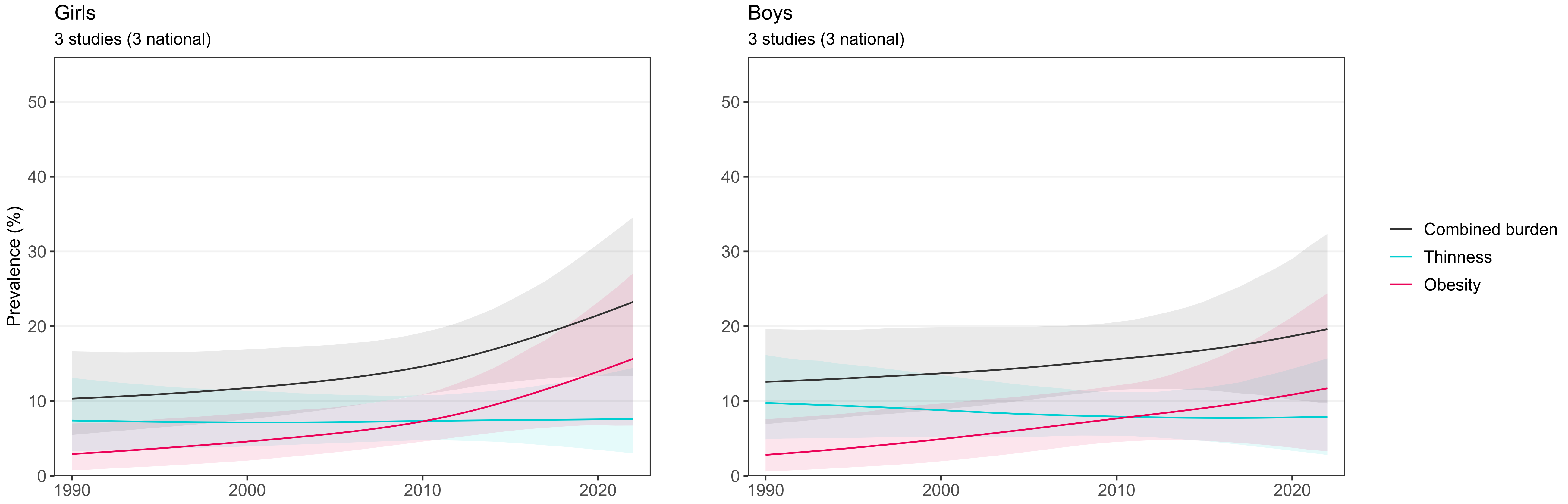


## Adults

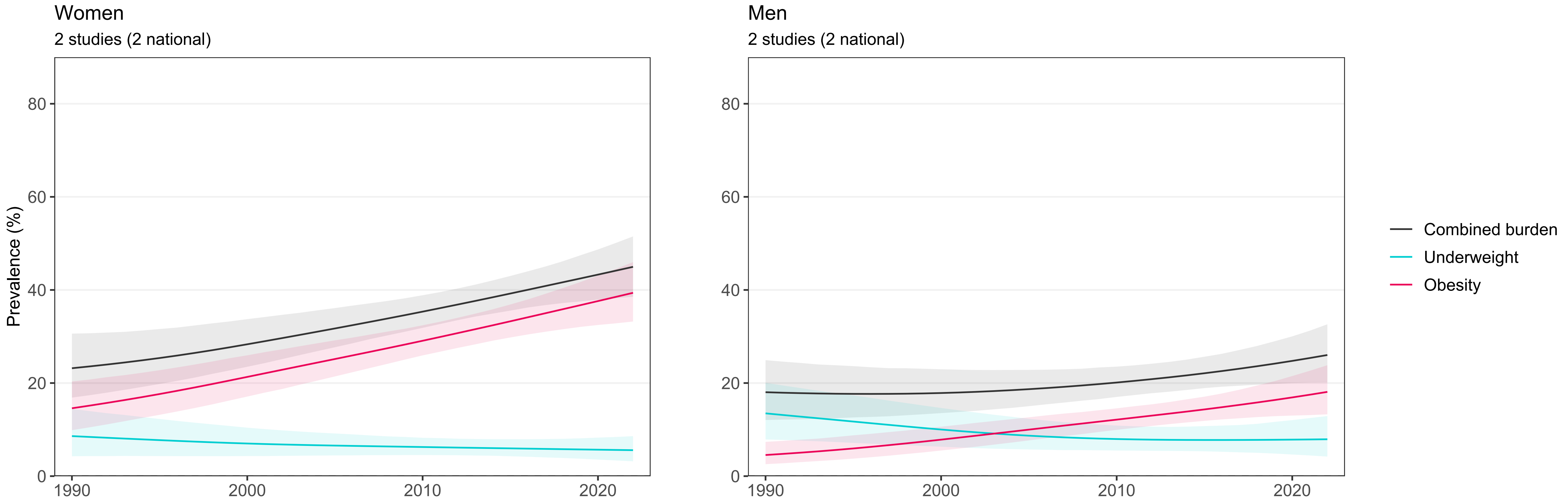

# Haiti

## School-aged children and adolescents

### Girls

4 studies (4 national)

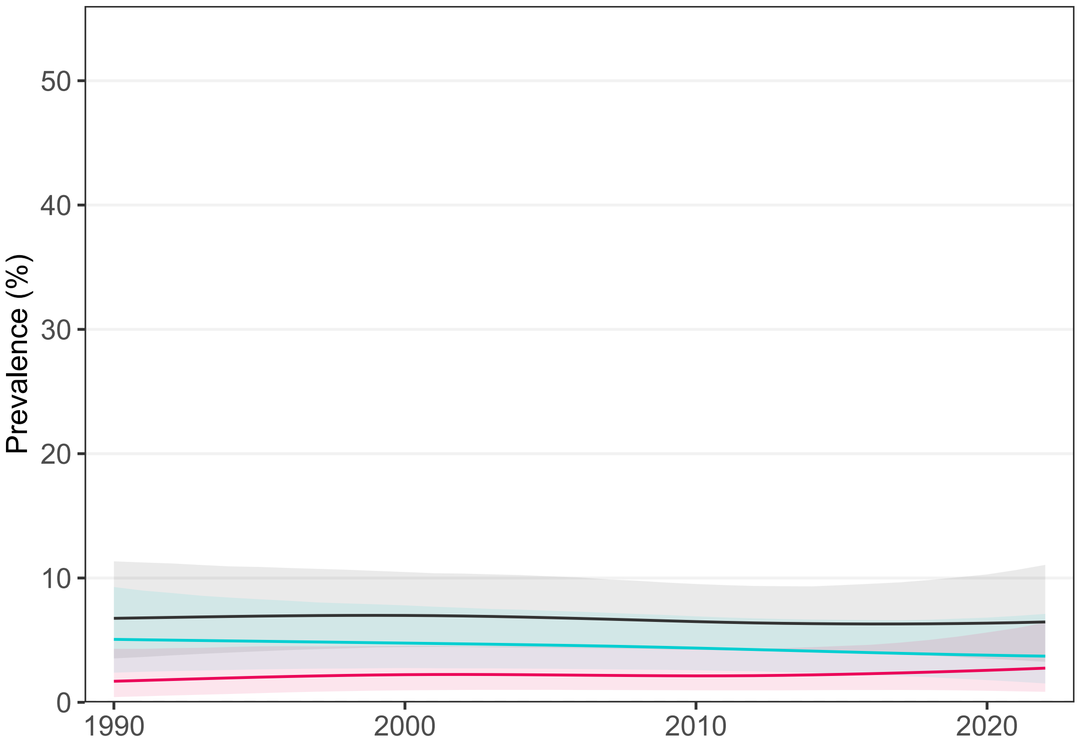


### Boys

No studies

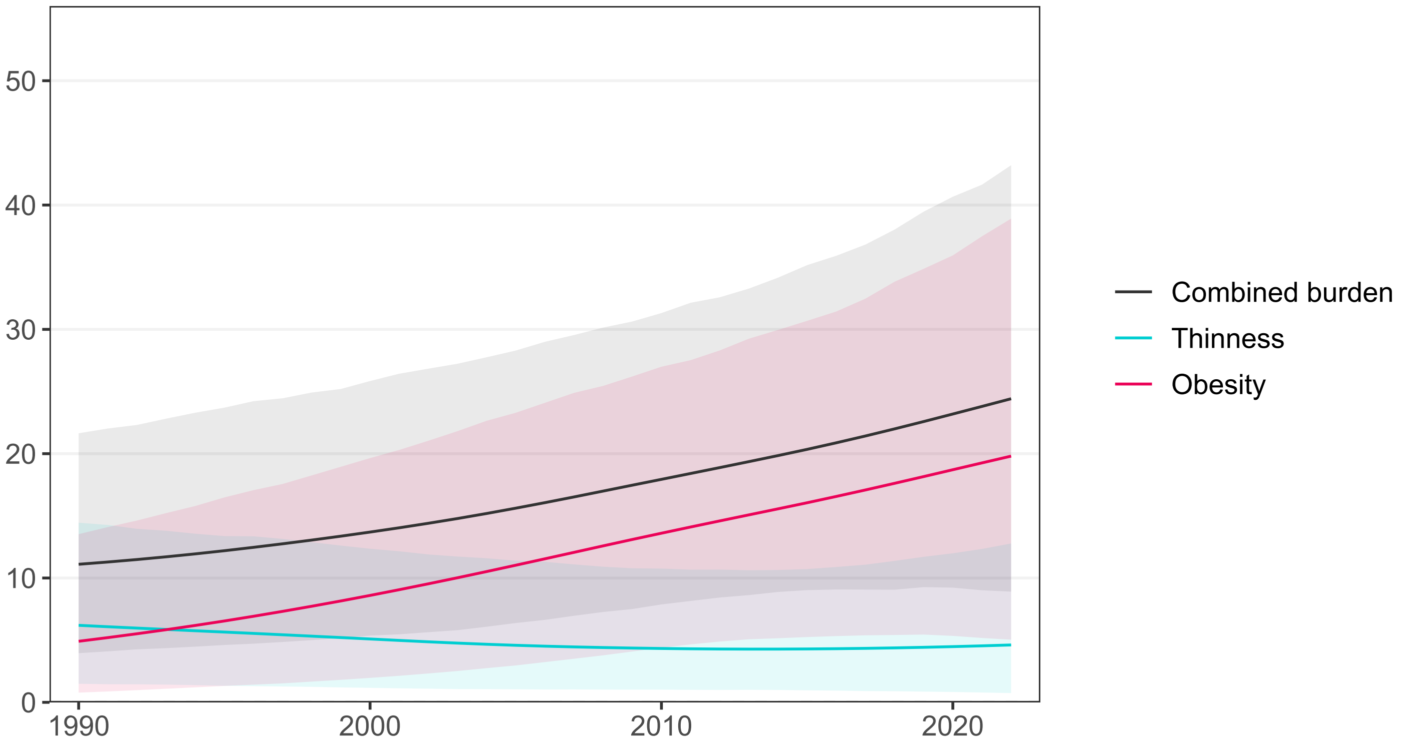


## Adults

### Women

7 studies (5 national)

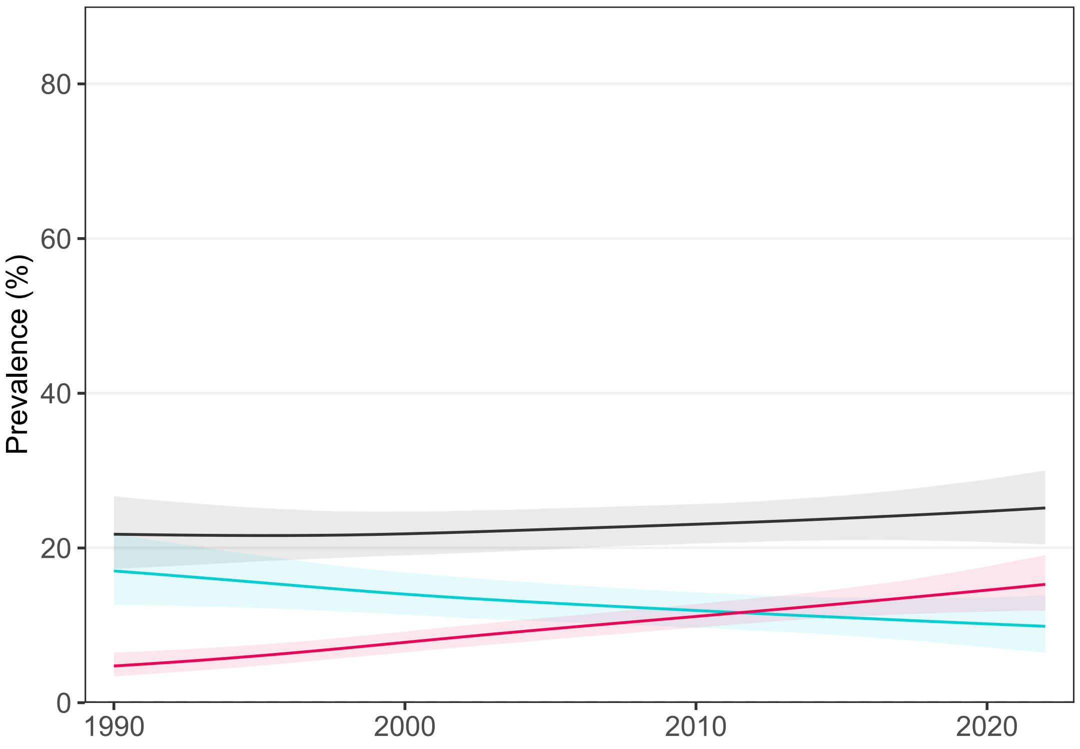


### Men

2 studies (0 national)

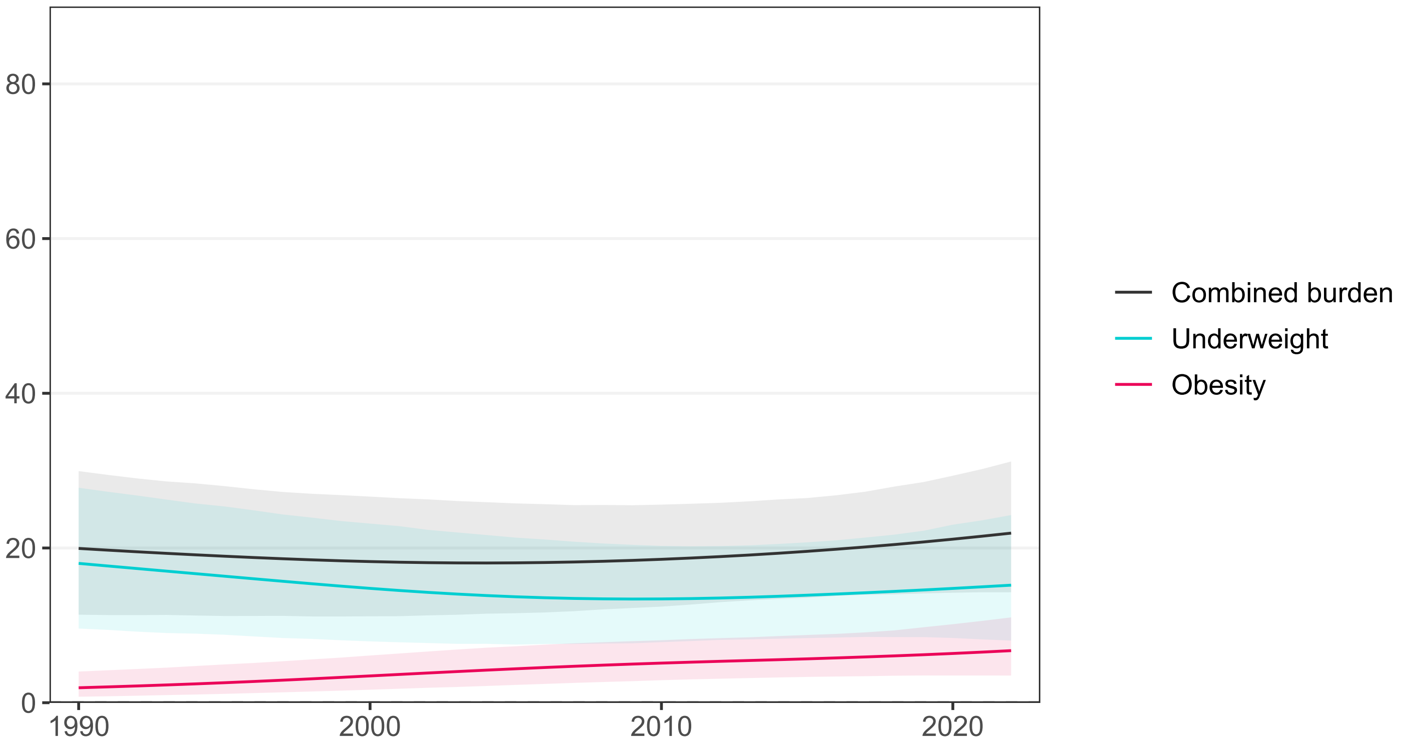

# Honduras

## School-aged children and adolescents

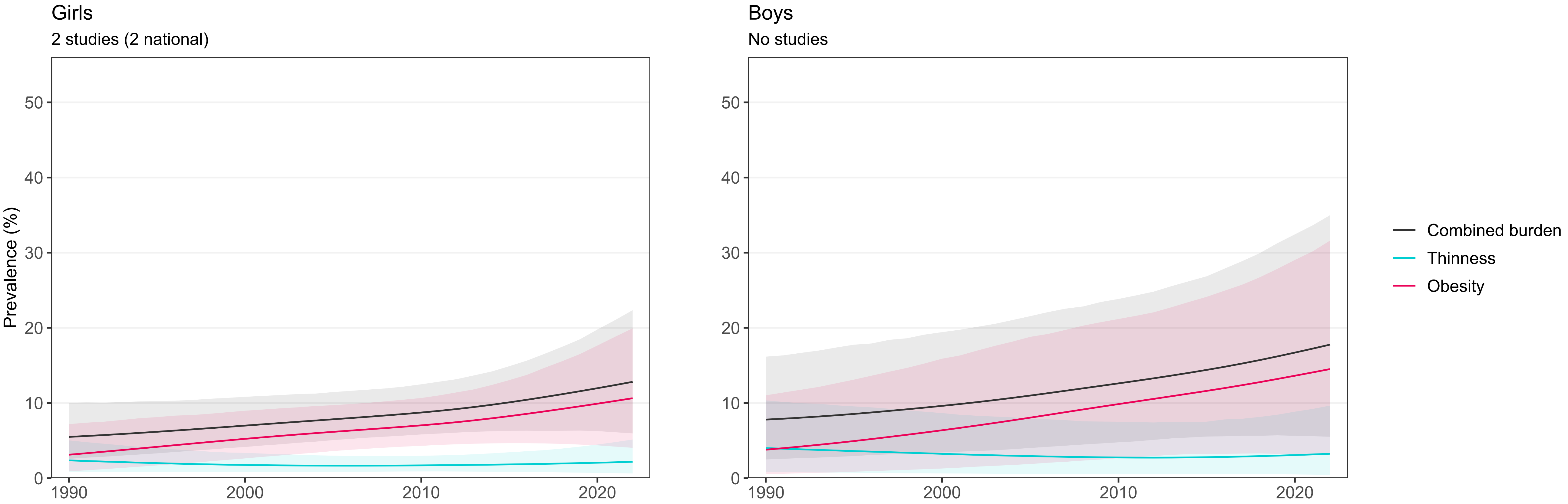


## Adults

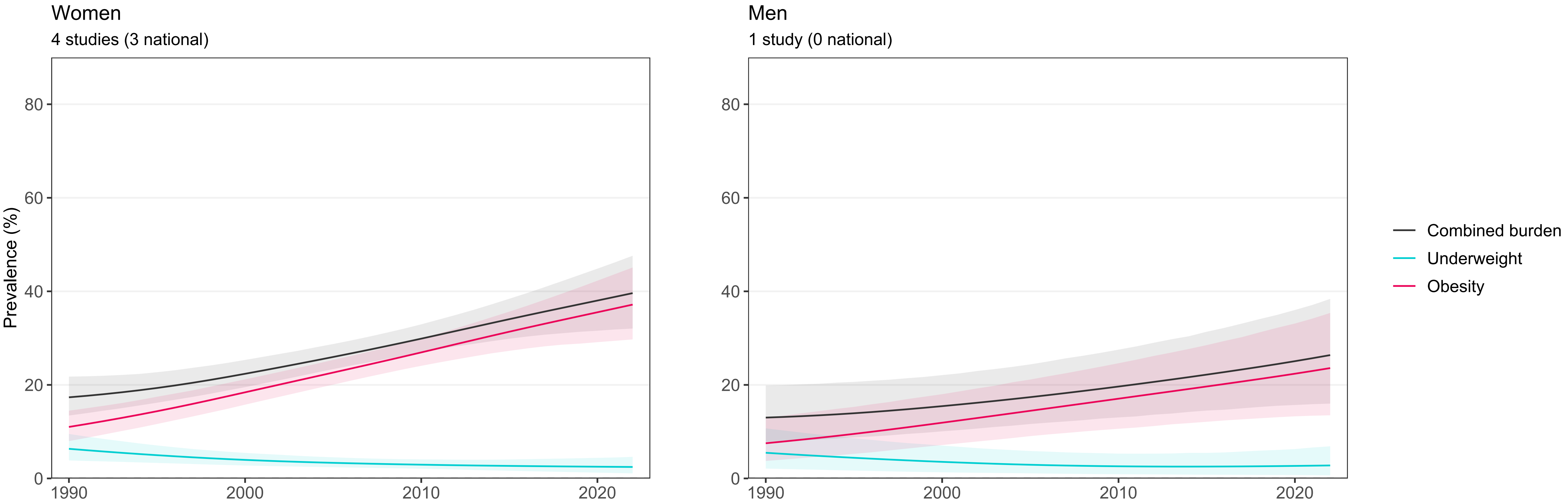

# Hungary

## School-aged children and adolescents

Girls
18 studies (15 national)

Boys
18 studies (15 national)

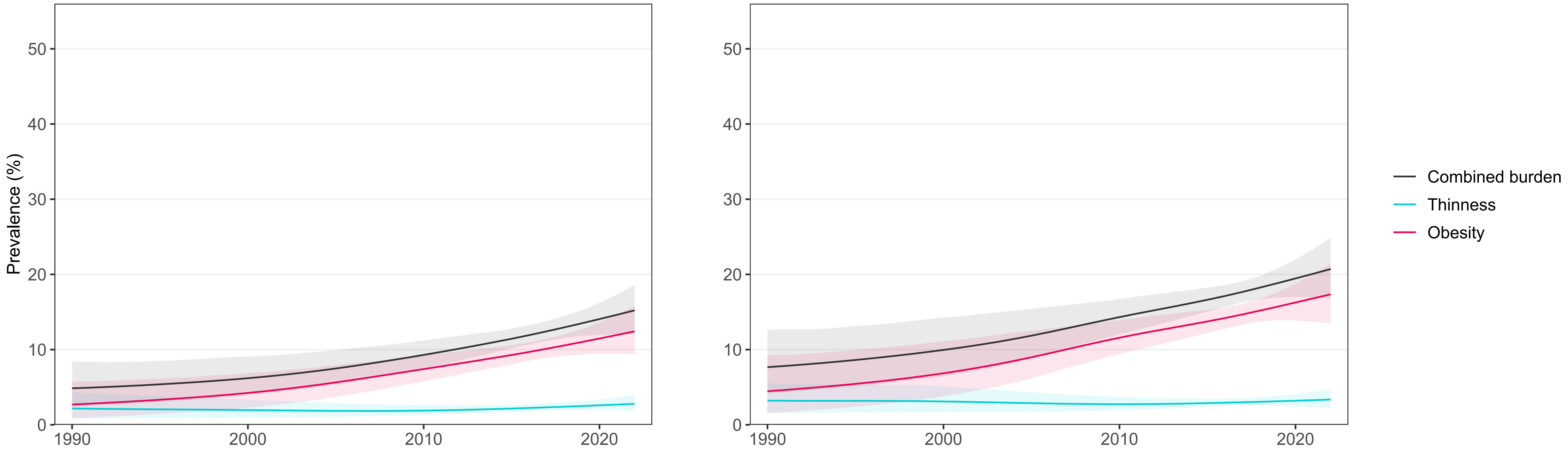


## Adults

Women
13 studies (8 national)

Men
15 studies (8 national)

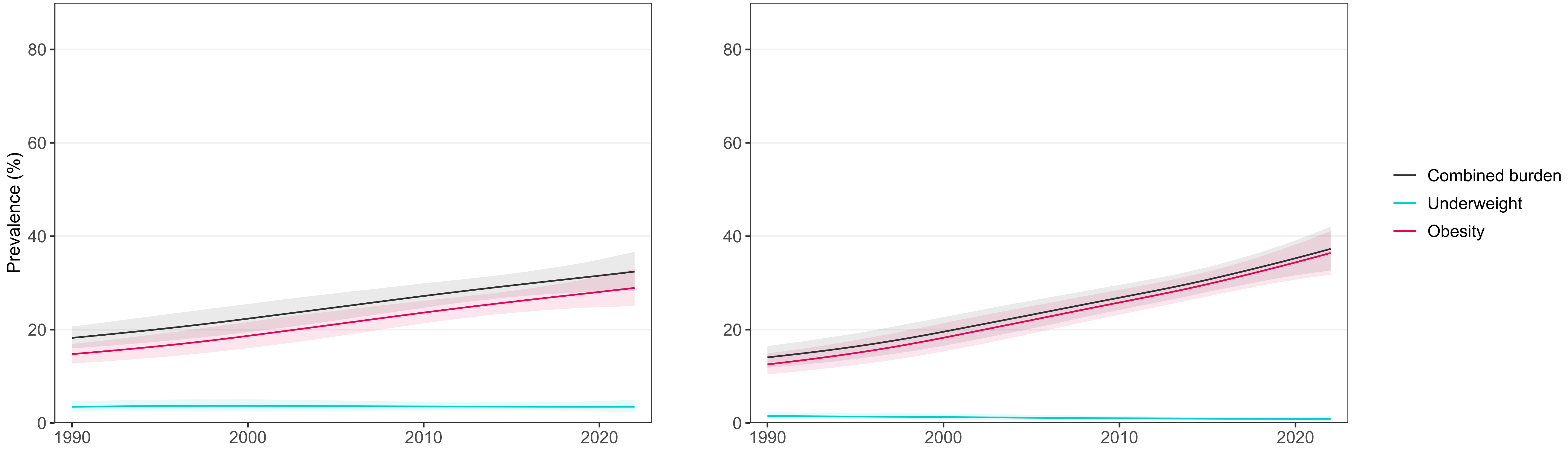

# Iceland

## School-aged children and adolescents

### Girls

No studies

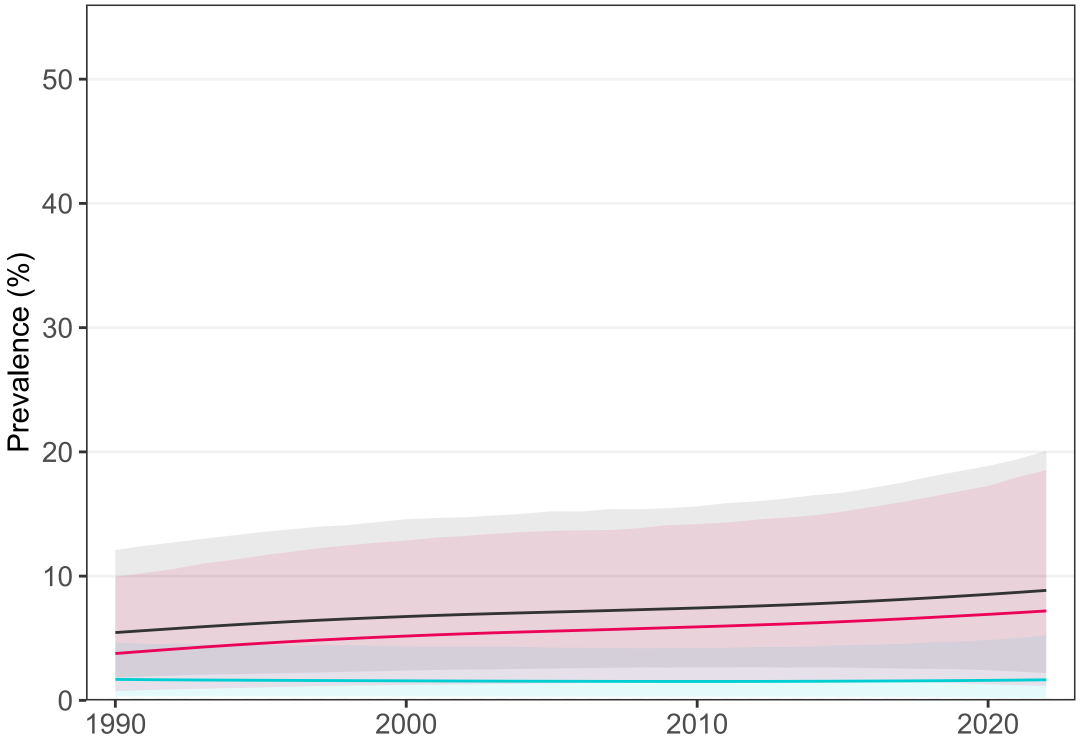


### Boys

No studies

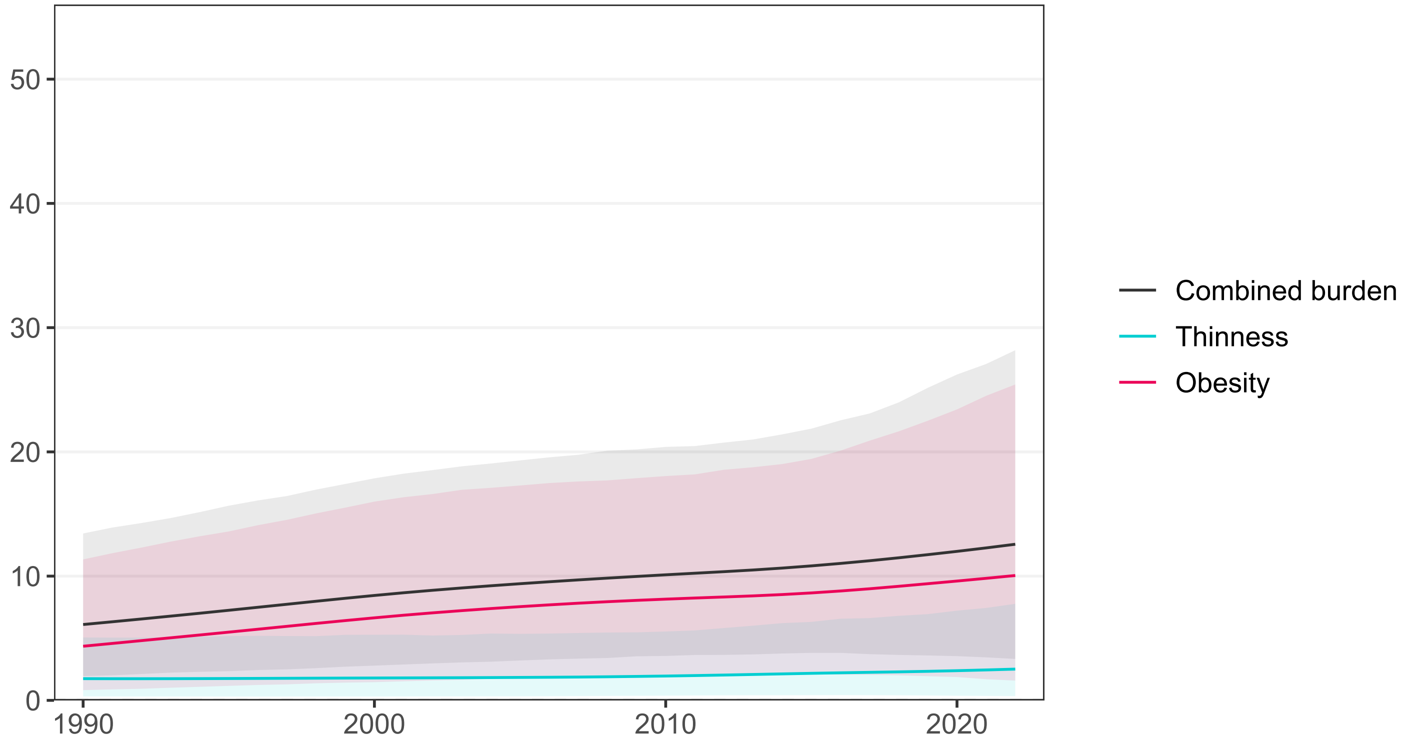


## Adults

### Women

17 studies (0 national)

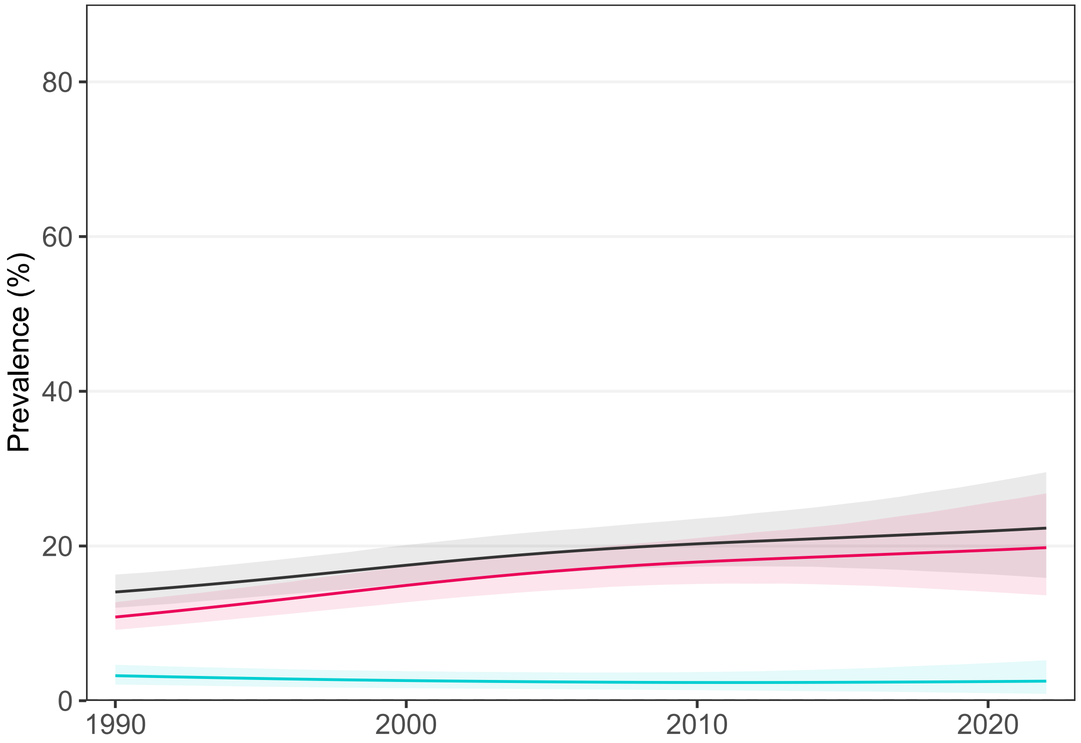


### Men

17 studies (0 national)

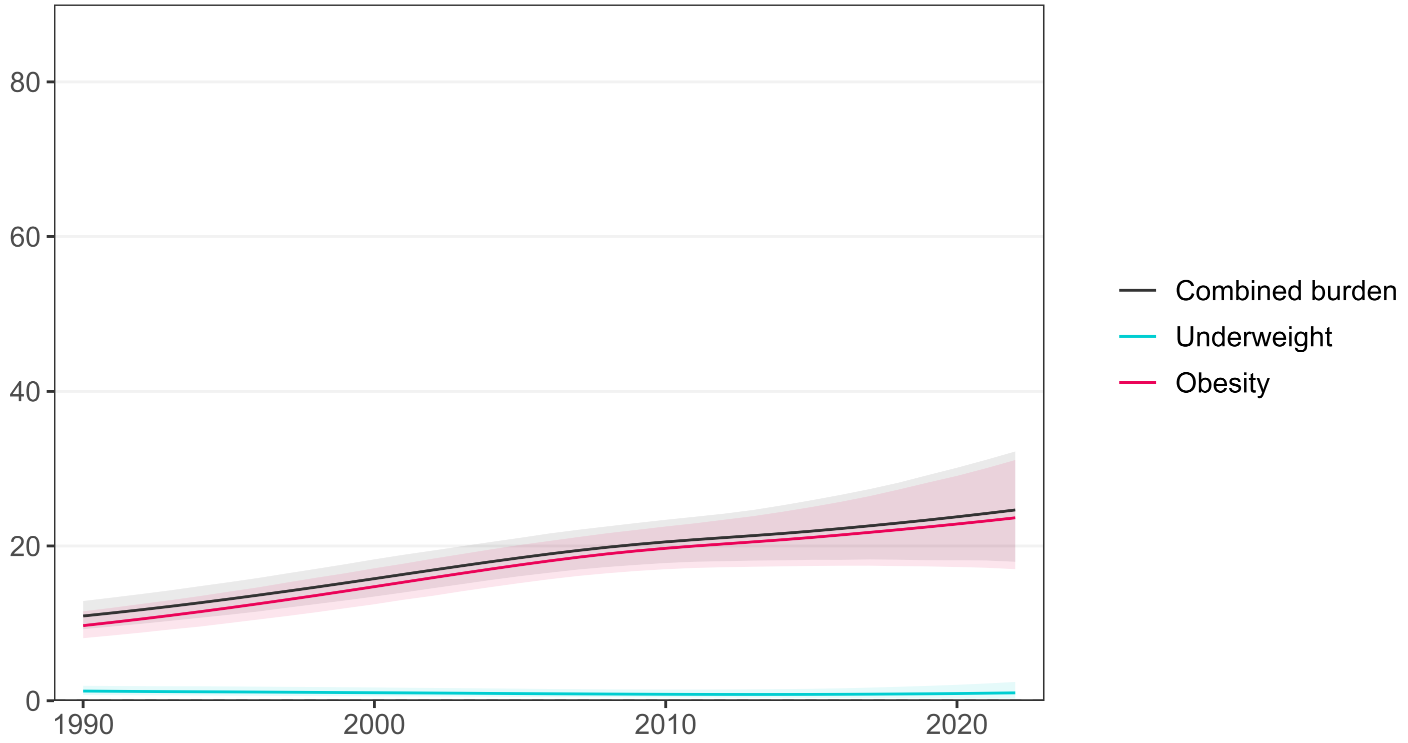

# India

## School-aged children and adolescents

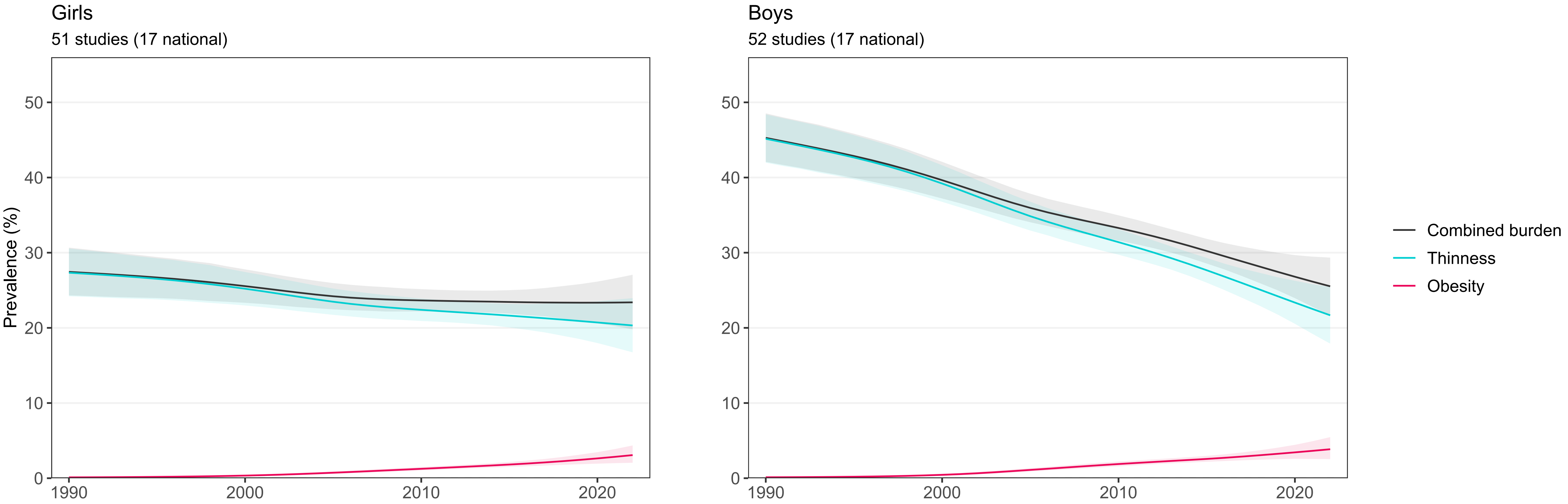


## Adults

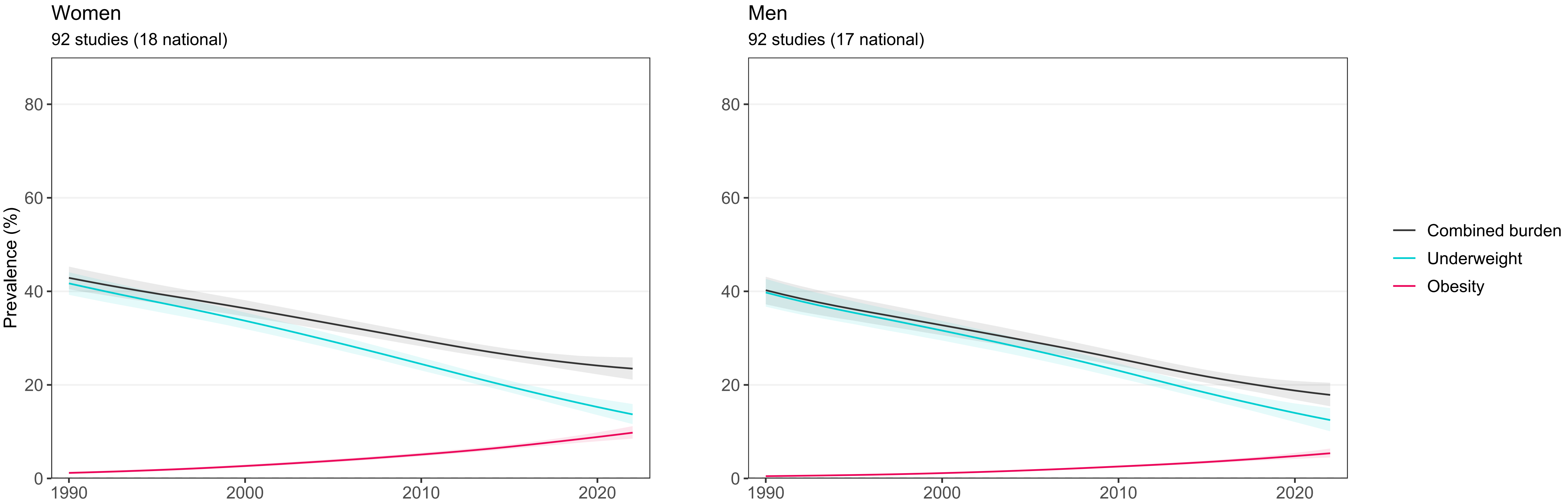

# Indonesia

## School-aged children and adolescents

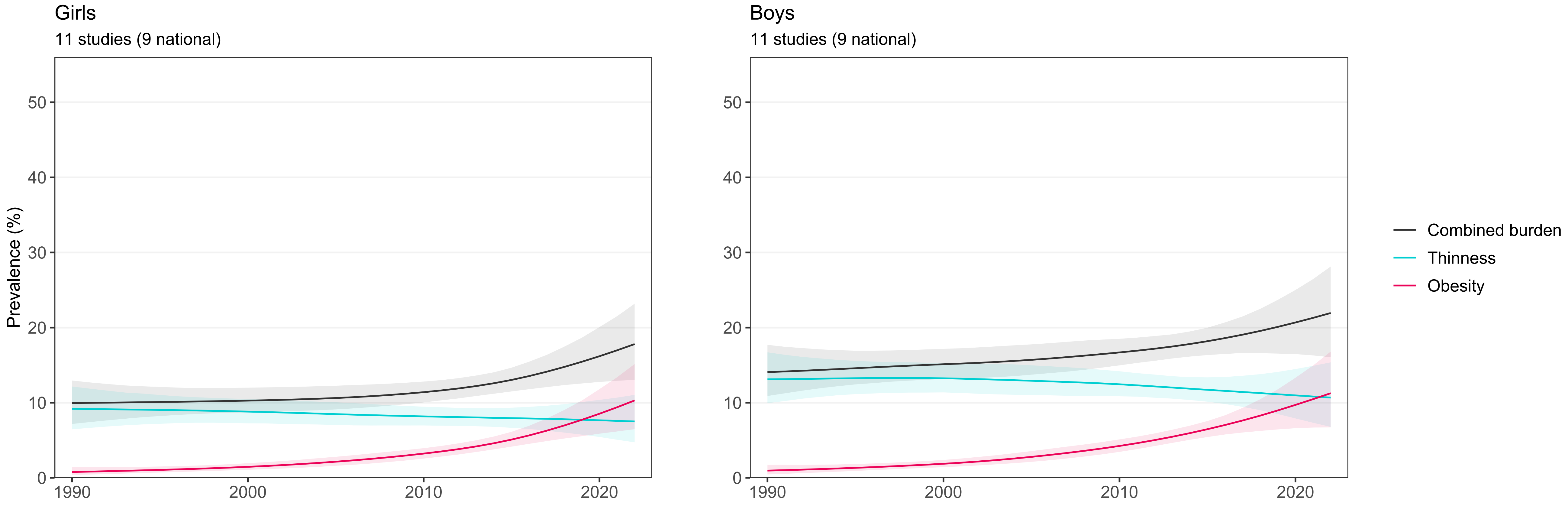


## Adults

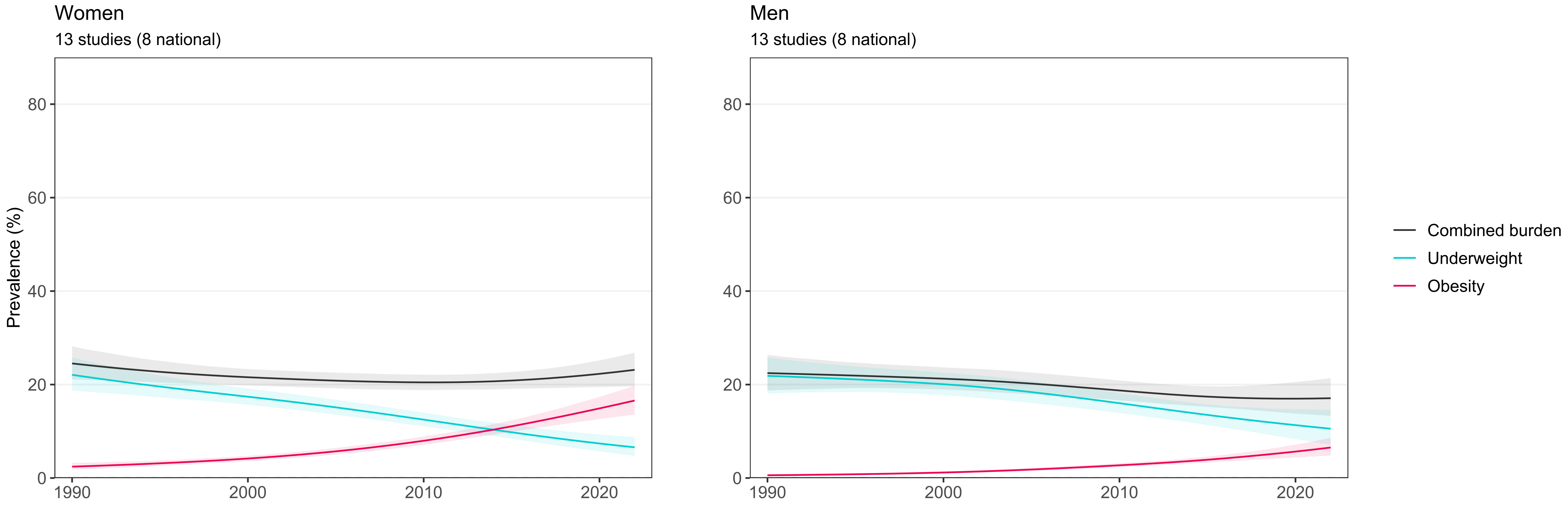

# Iran

## School-aged children and adolescents

### Girls
52 studies (18 national)

### Boys
50 studies (18 national)

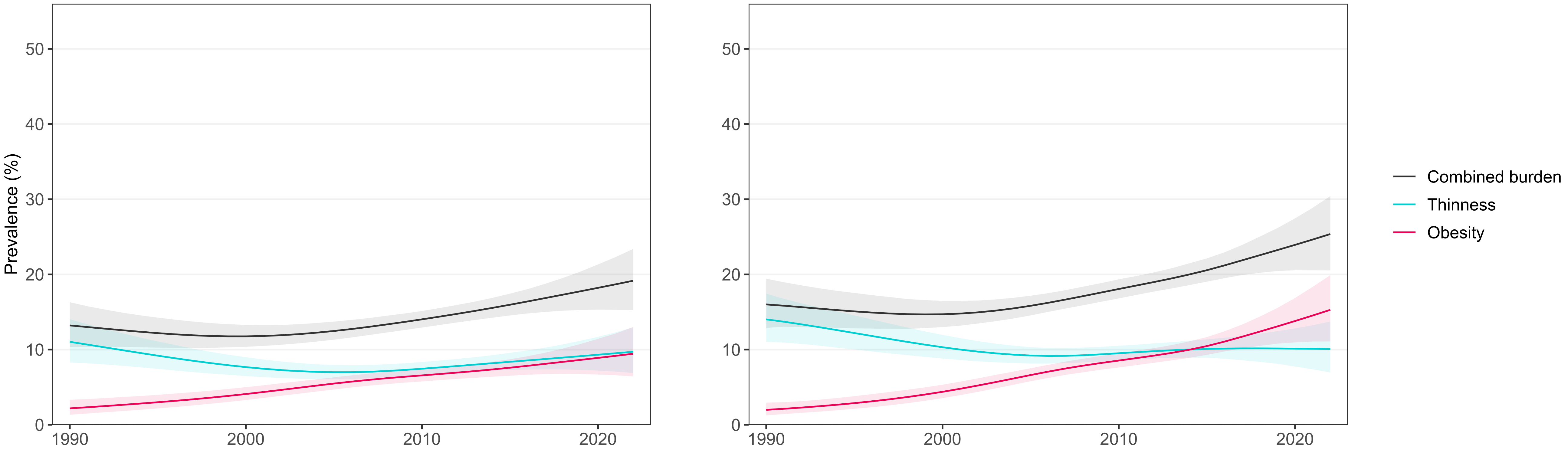


## Adults

### Women
91 studies (18 national)

### Men
93 studies (18 national)

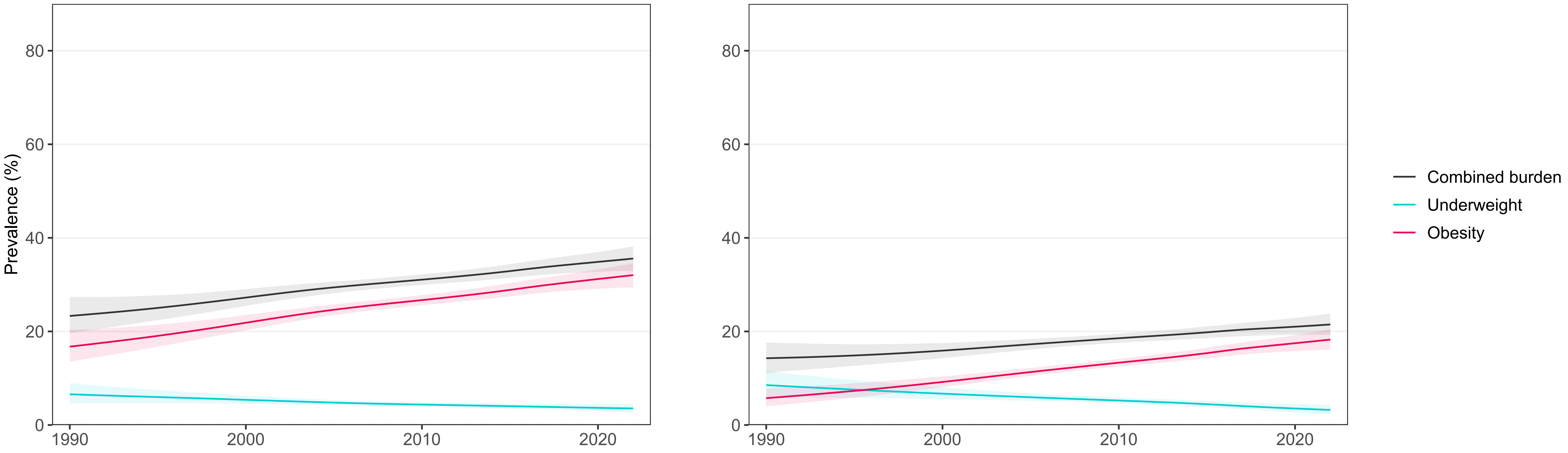

# Iraq

## School-aged children and adolescents

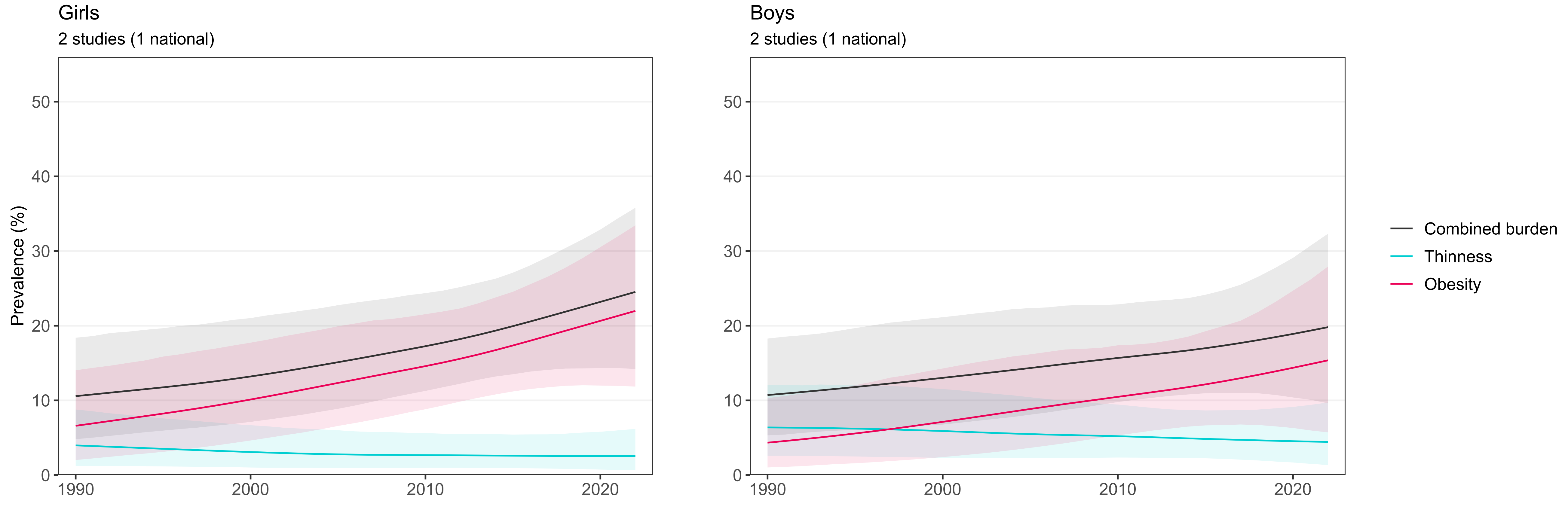


## Adults

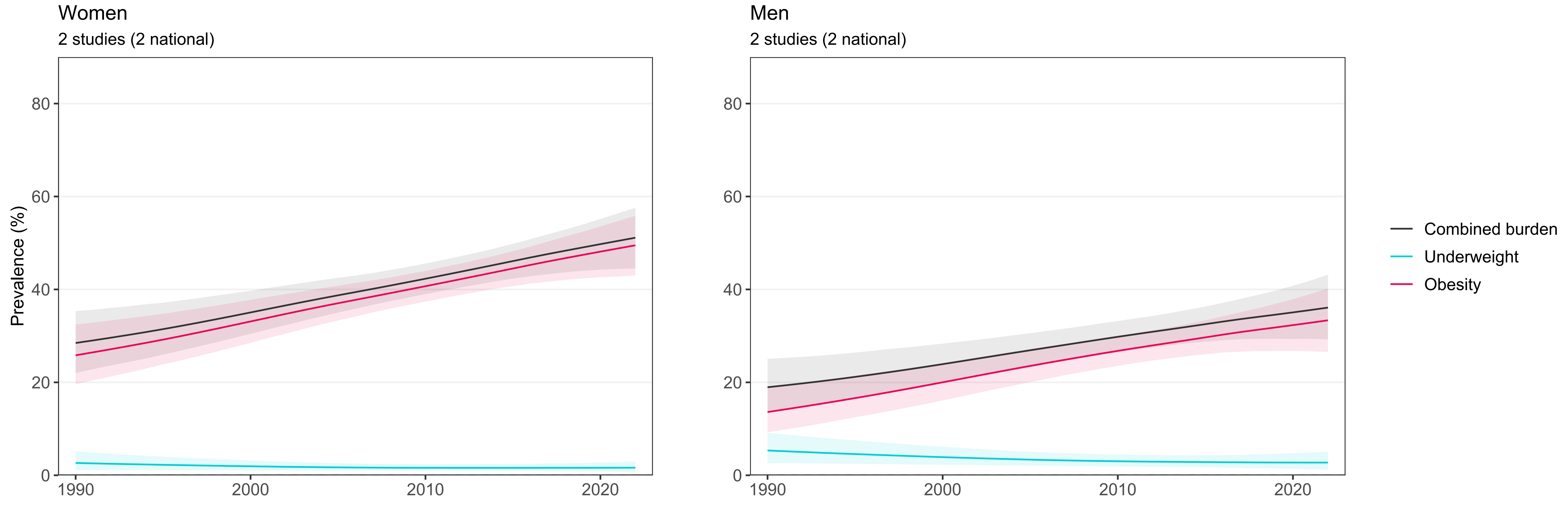

# Ireland

## School-aged children and adolescents

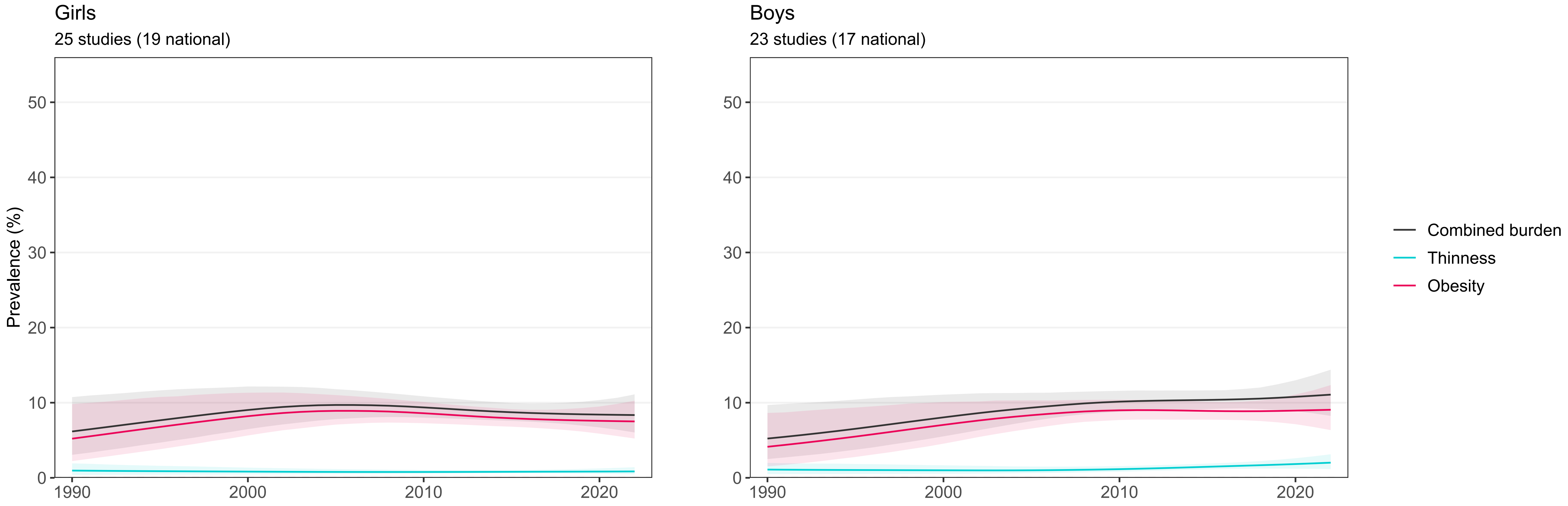


## Adults

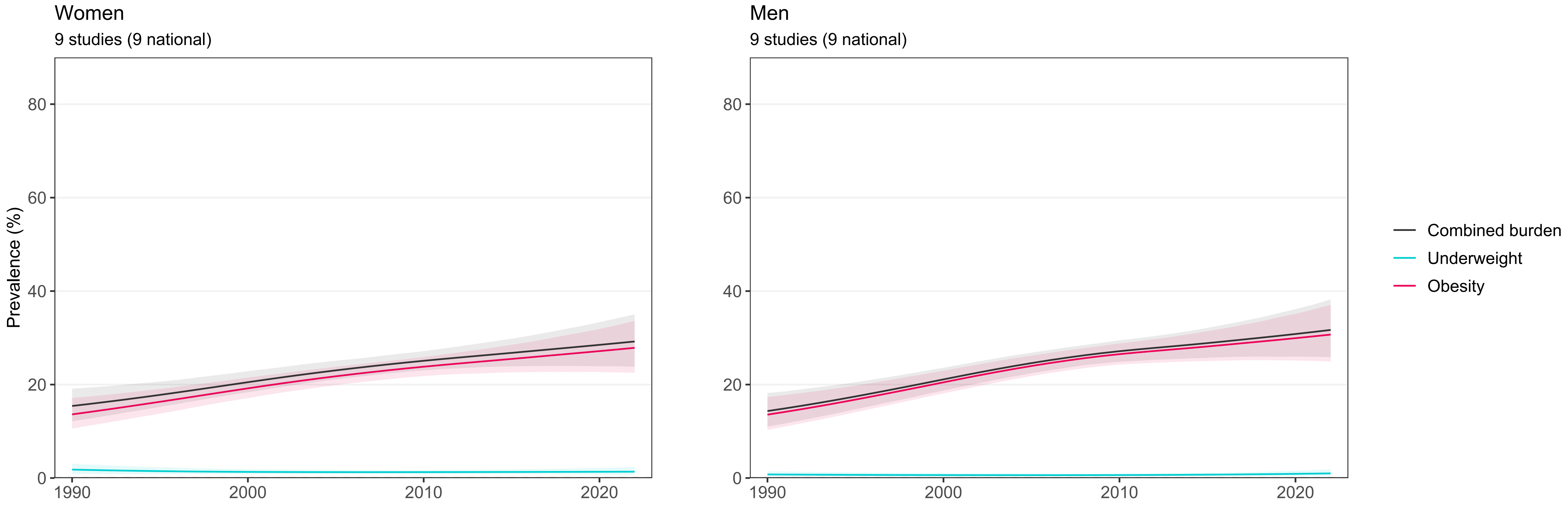

# Israel

## School-aged children and adolescents

### Girls
15 studies (15 national)

### Boys
15 studies (15 national)

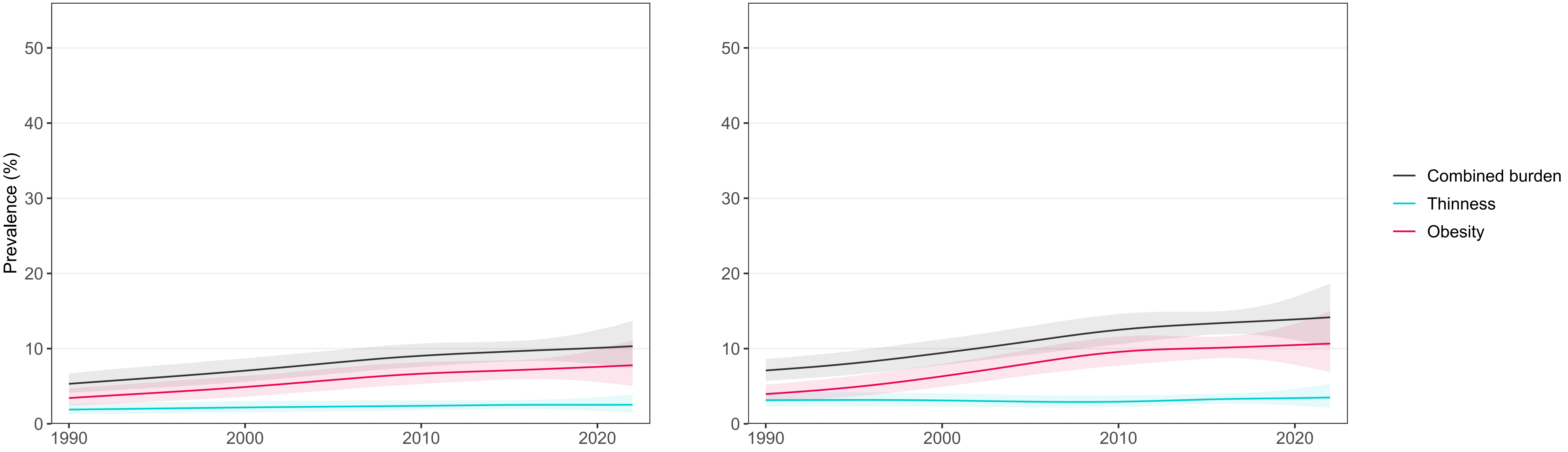


## Adults

### Women
23 studies (16 national)

### Men
23 studies (16 national)

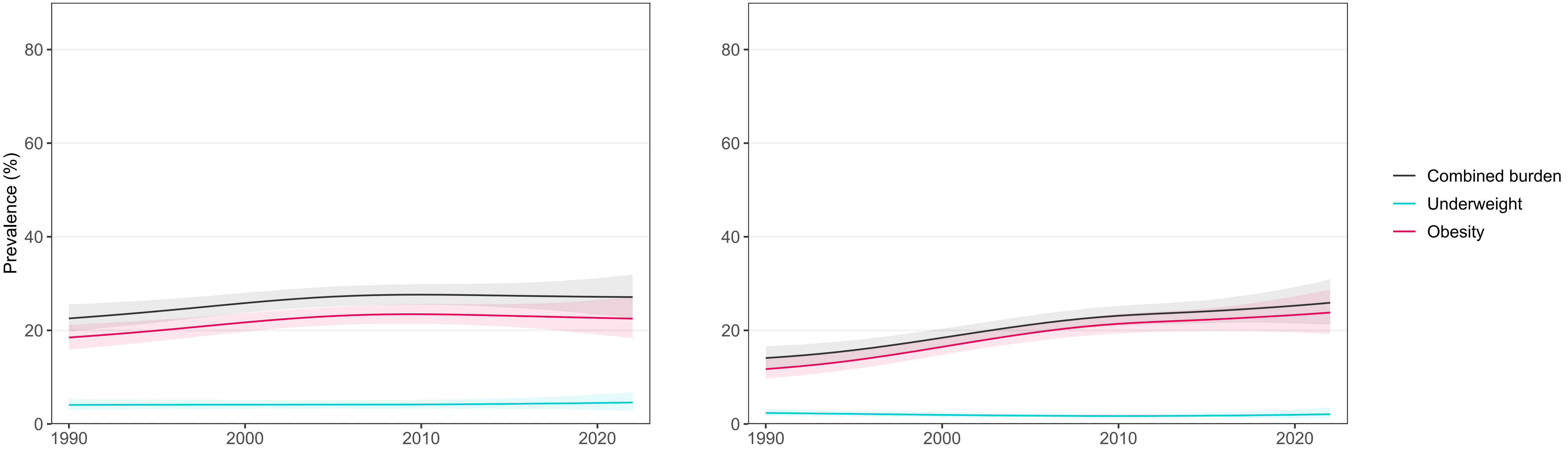

# Italy

## School-aged children and adolescents

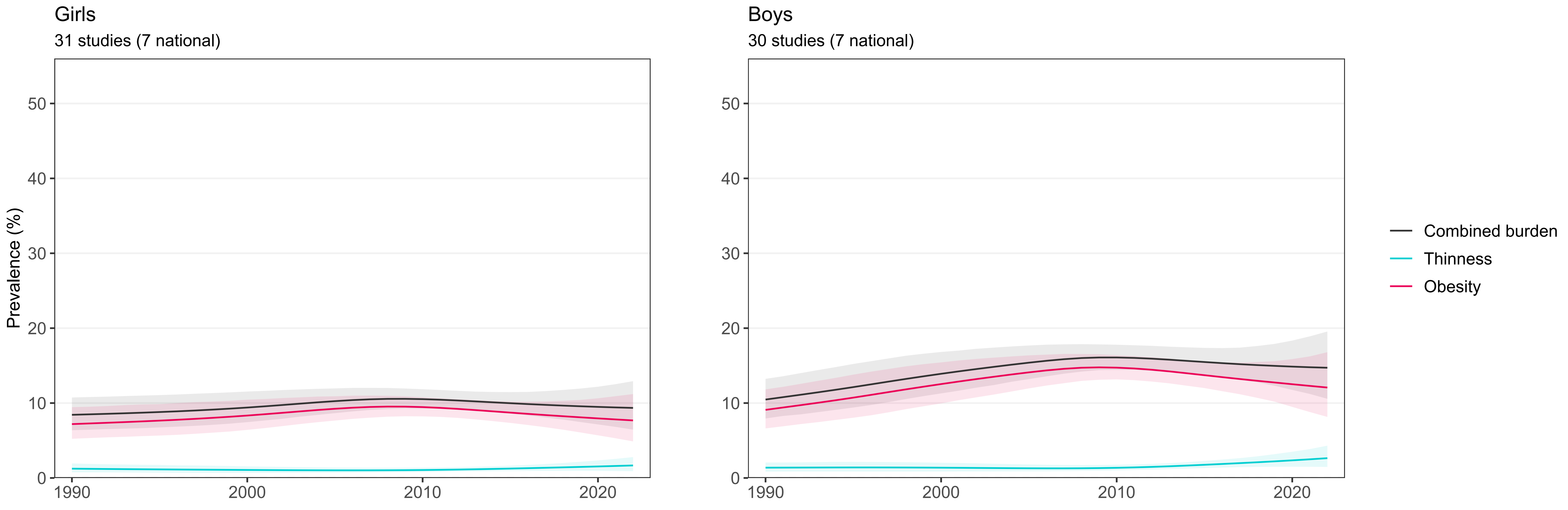


## Adults

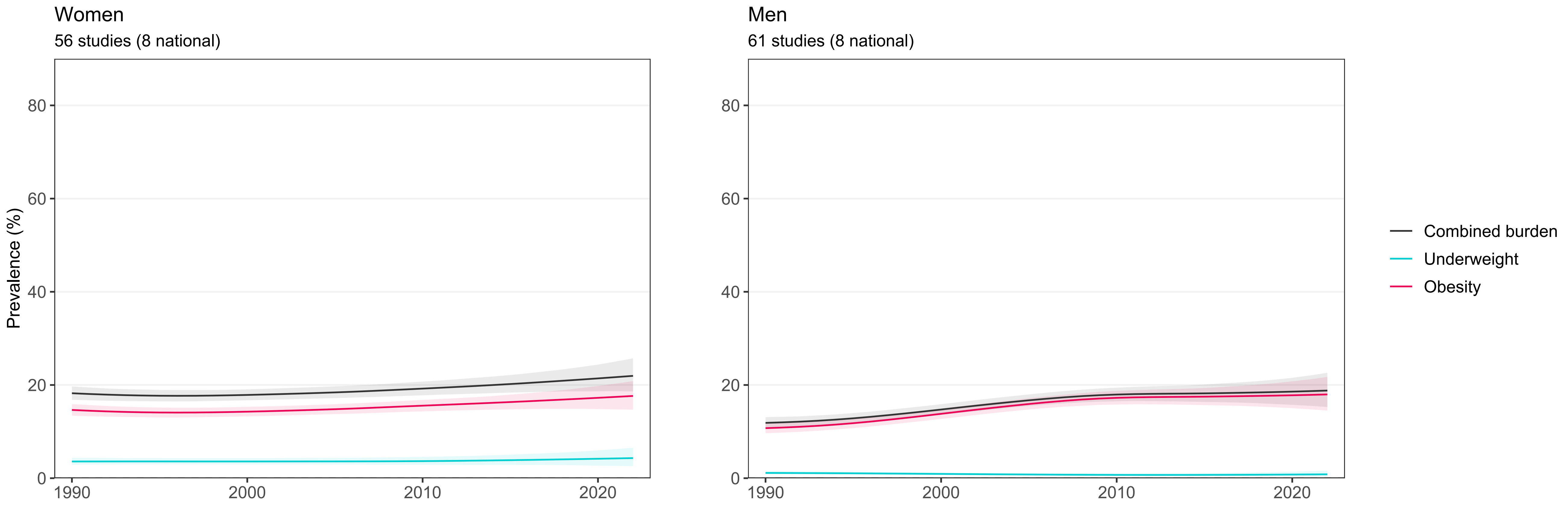

# Jamaica

## School-aged children and adolescents

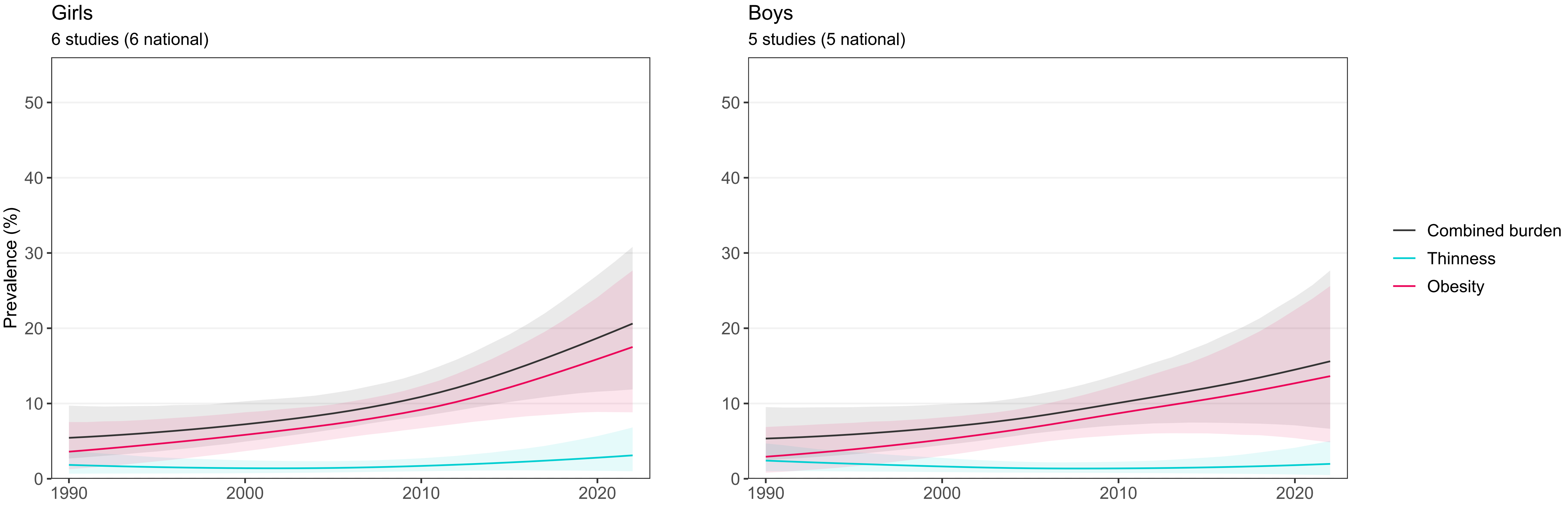


## Adults

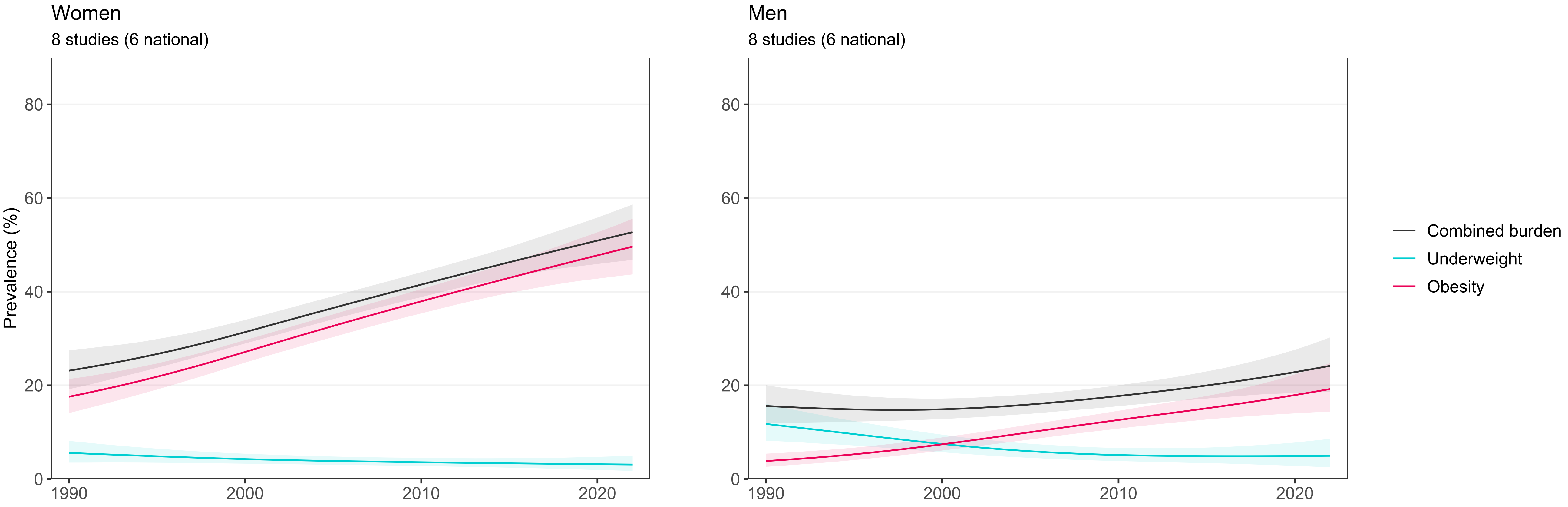

# Japan

## School-aged children and adolescents

### Girls

82 studies (56 national)

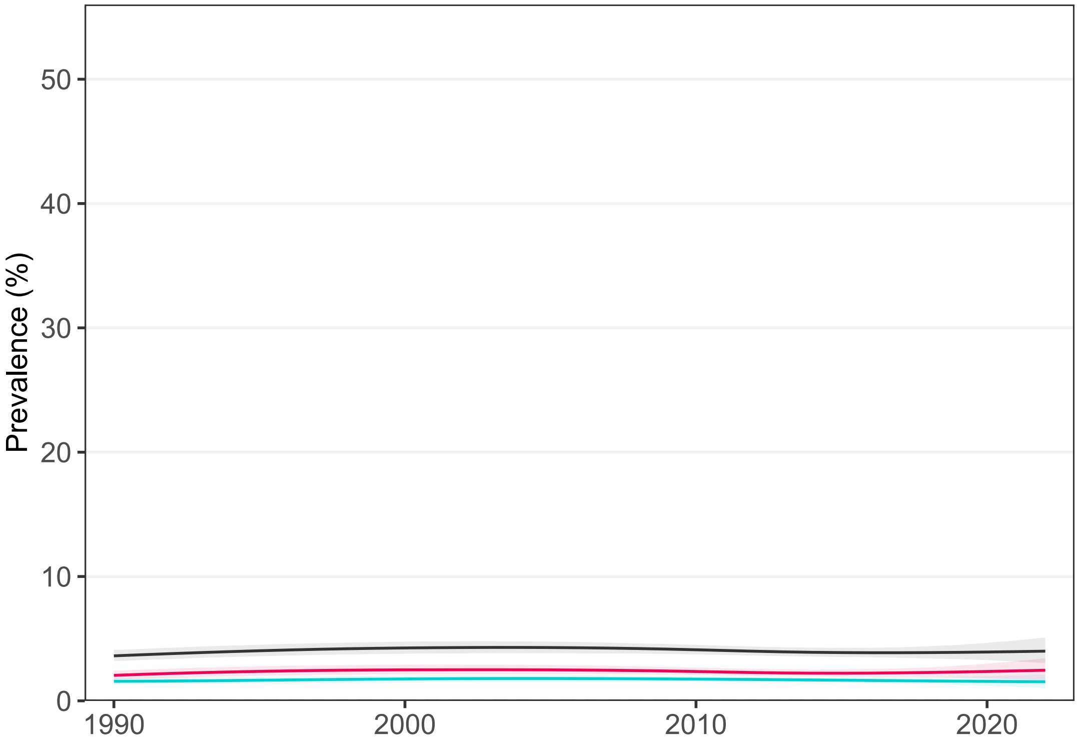


### Boys

82 studies (56 national)

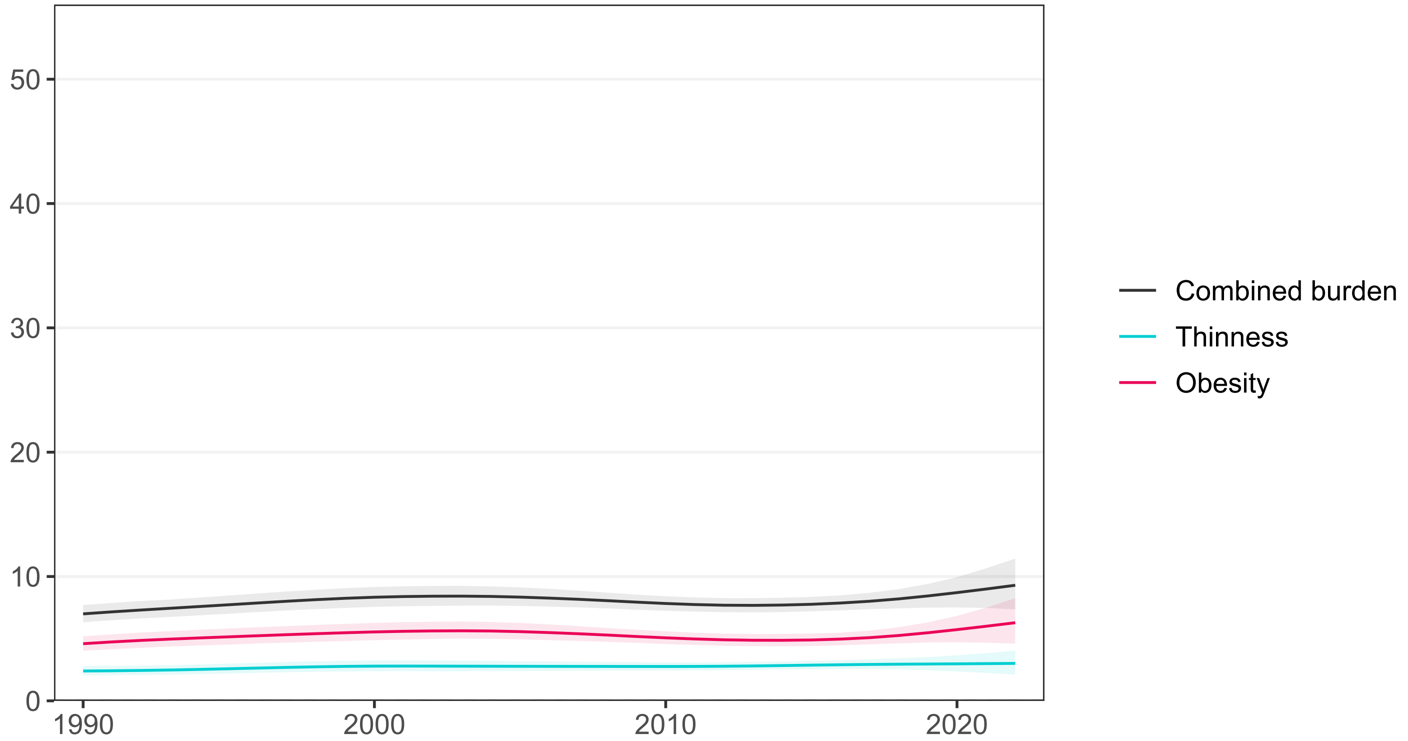


## Adults

### Women

93 studies (43 national)

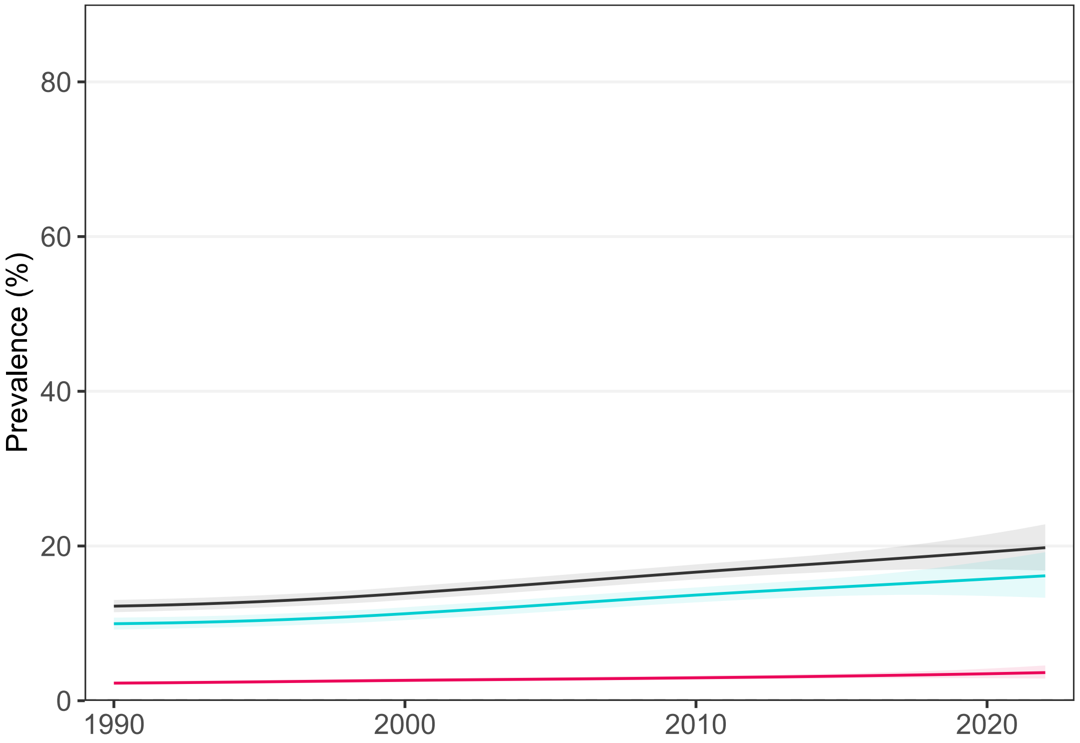


### Men

92 studies (43 national)

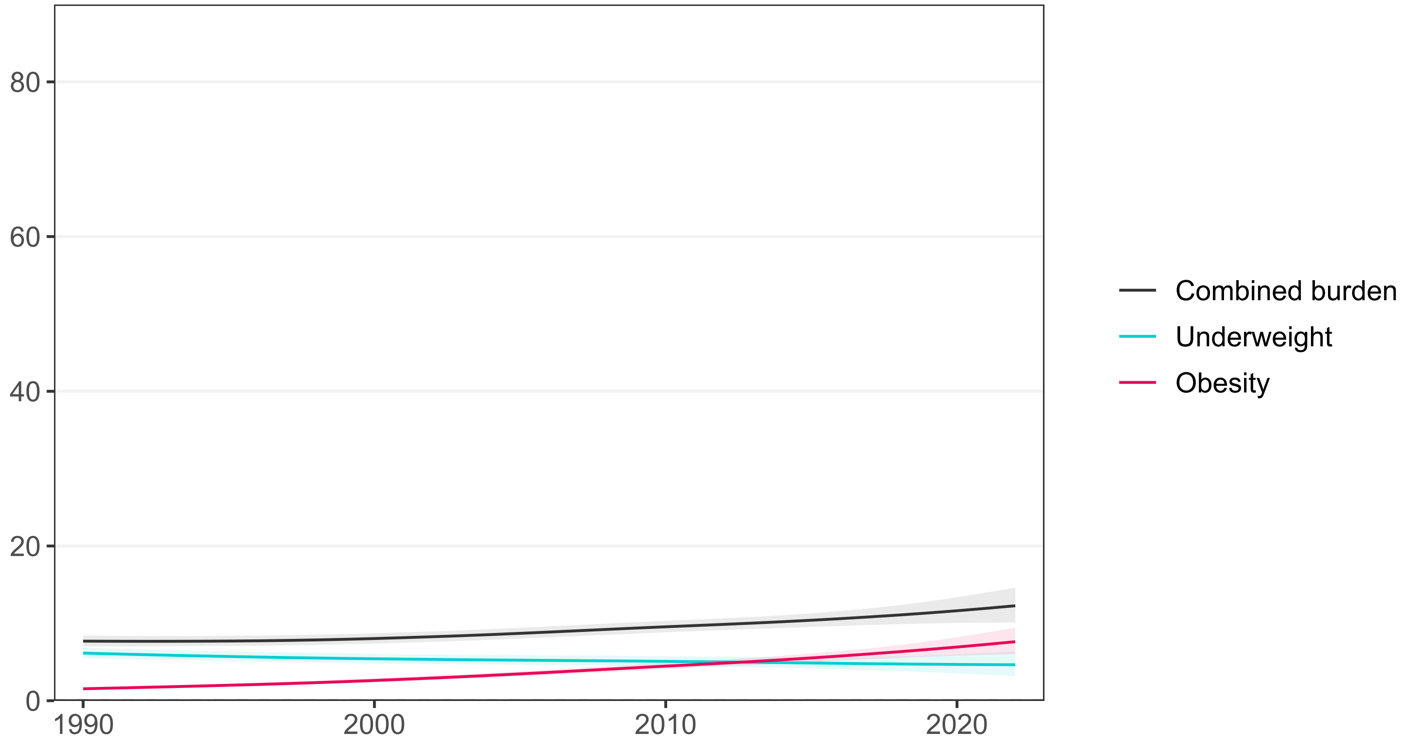

# Jordan

## School-aged children and adolescents

Girls

8 studies (5 national)

Boys

8 studies (5 national)

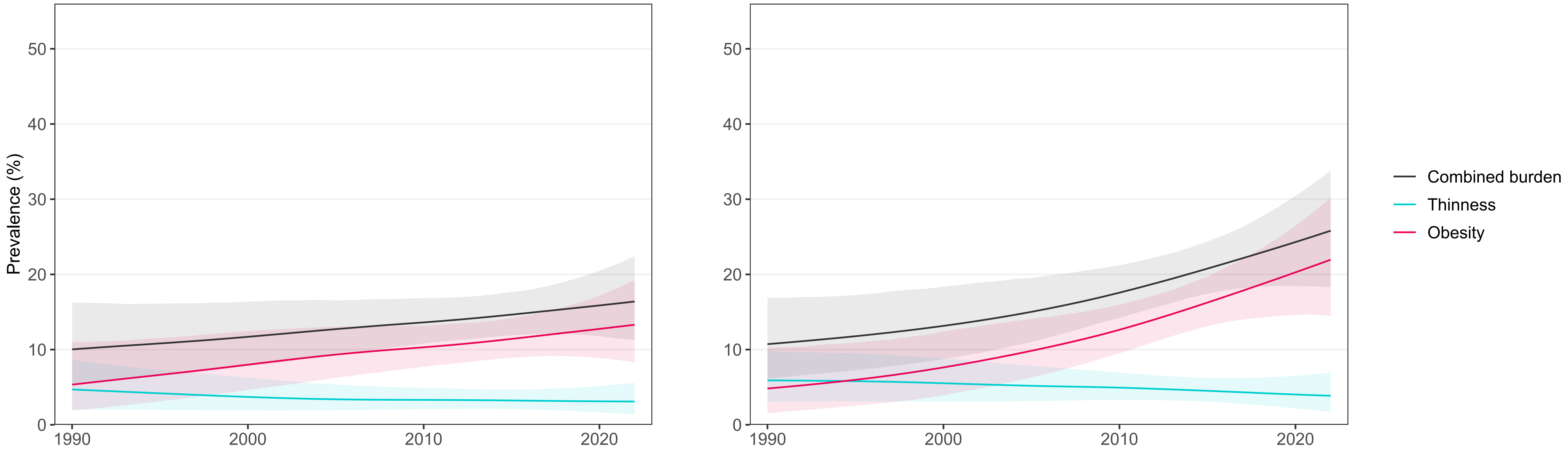


## Adults

Women

14 studies (12 national)

Men

7 studies (5 national)

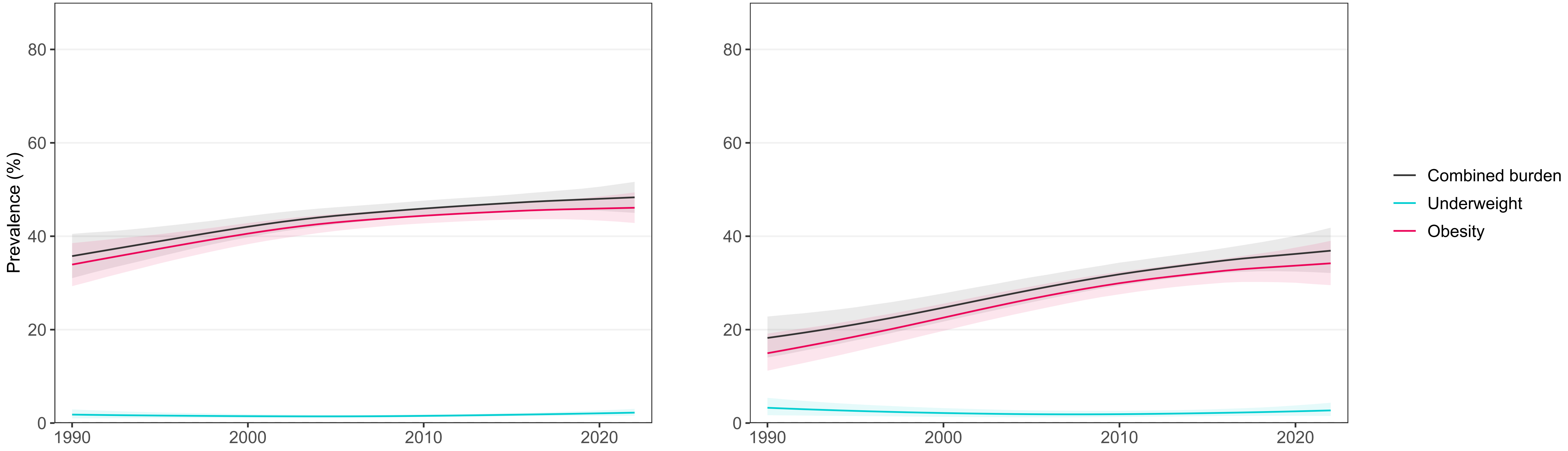

# Kazakhstan

## School-aged children and adolescents

### Girls

11 studies (5 national)

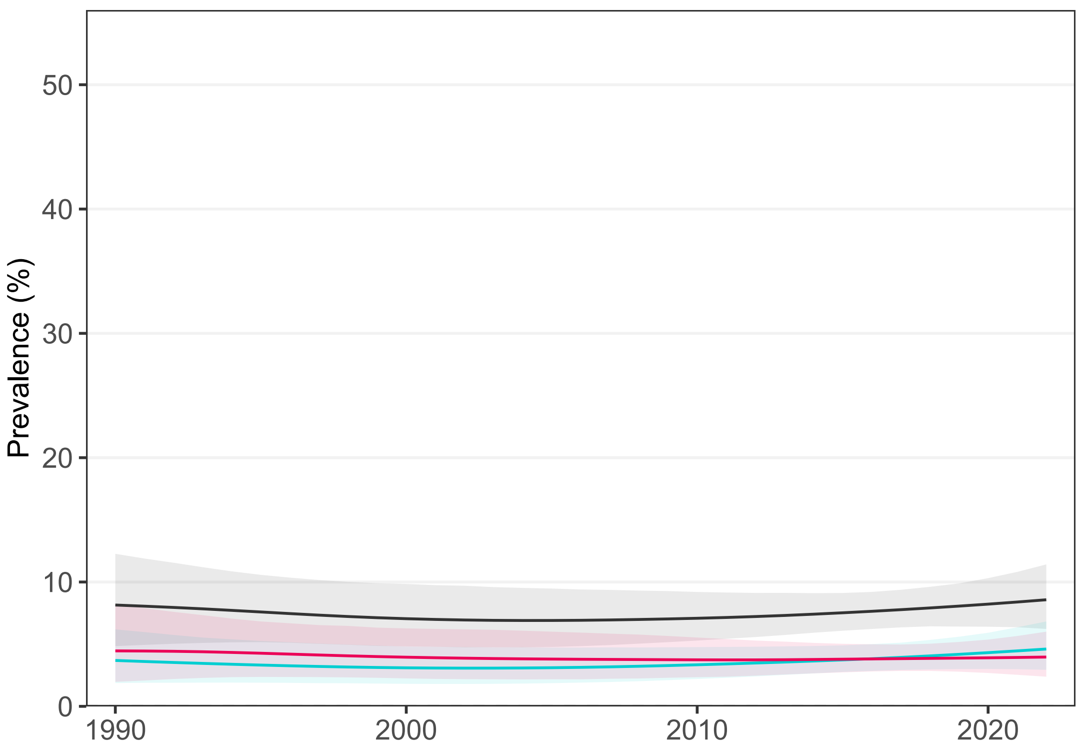


### Boys

8 studies (3 national)

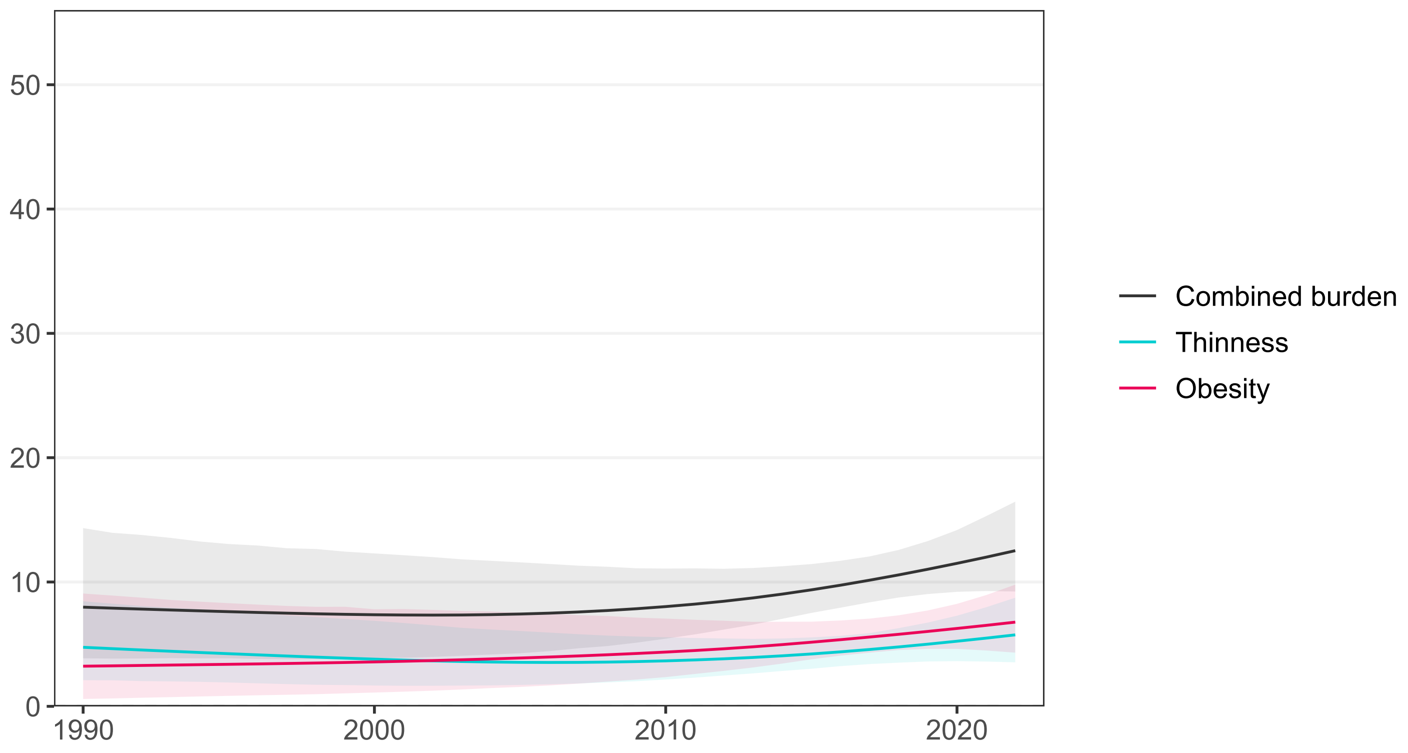


## Adults

### Women

9 studies (3 national)

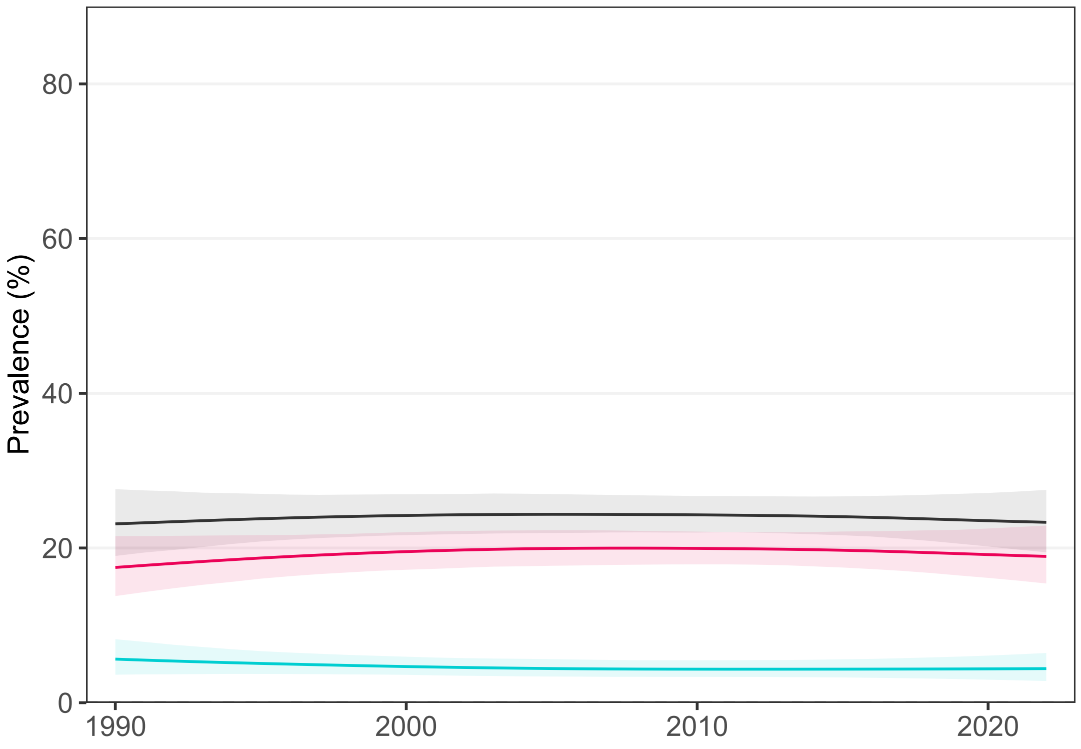


### Men

8 studies (1 national)

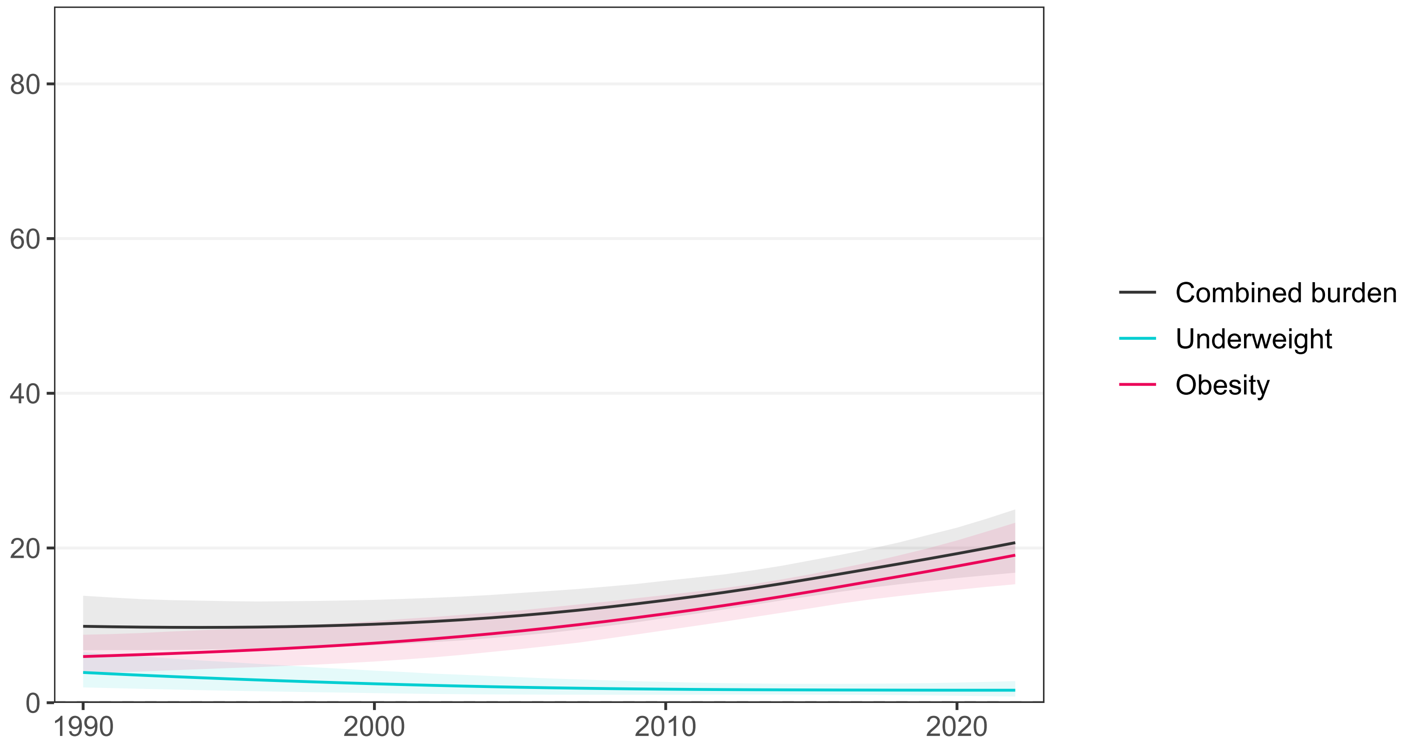

# Kenya

## School-aged children and adolescents

### Girls

7 studies (5 national)

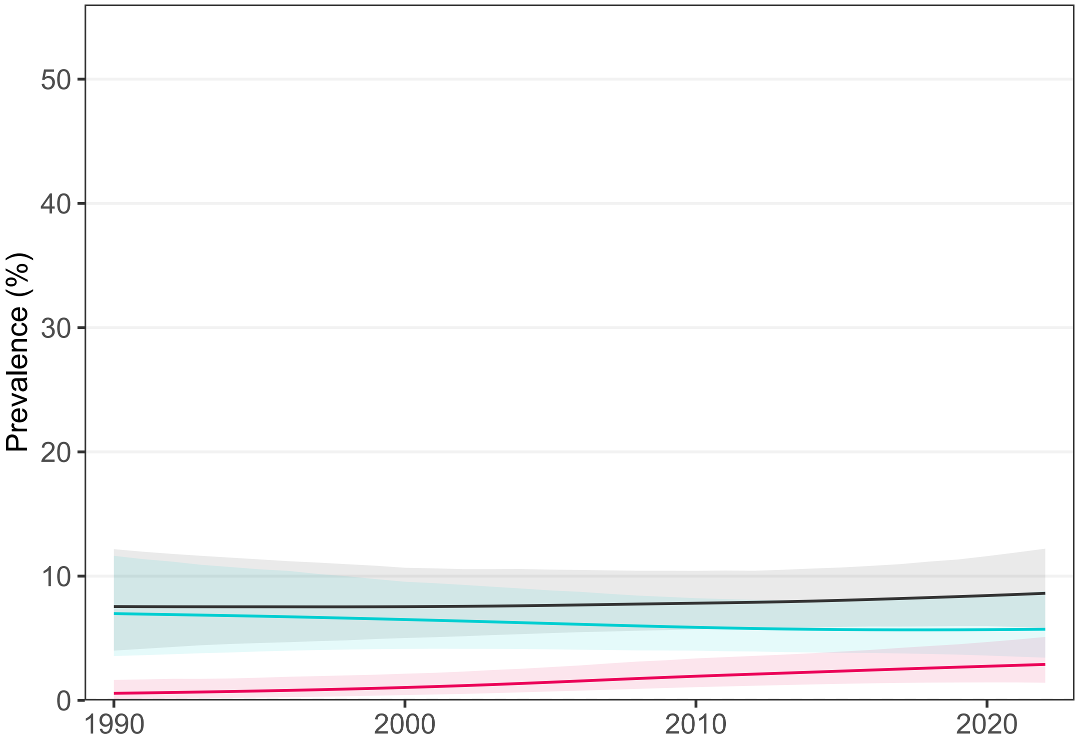


### Boys

4 studies (2 national)

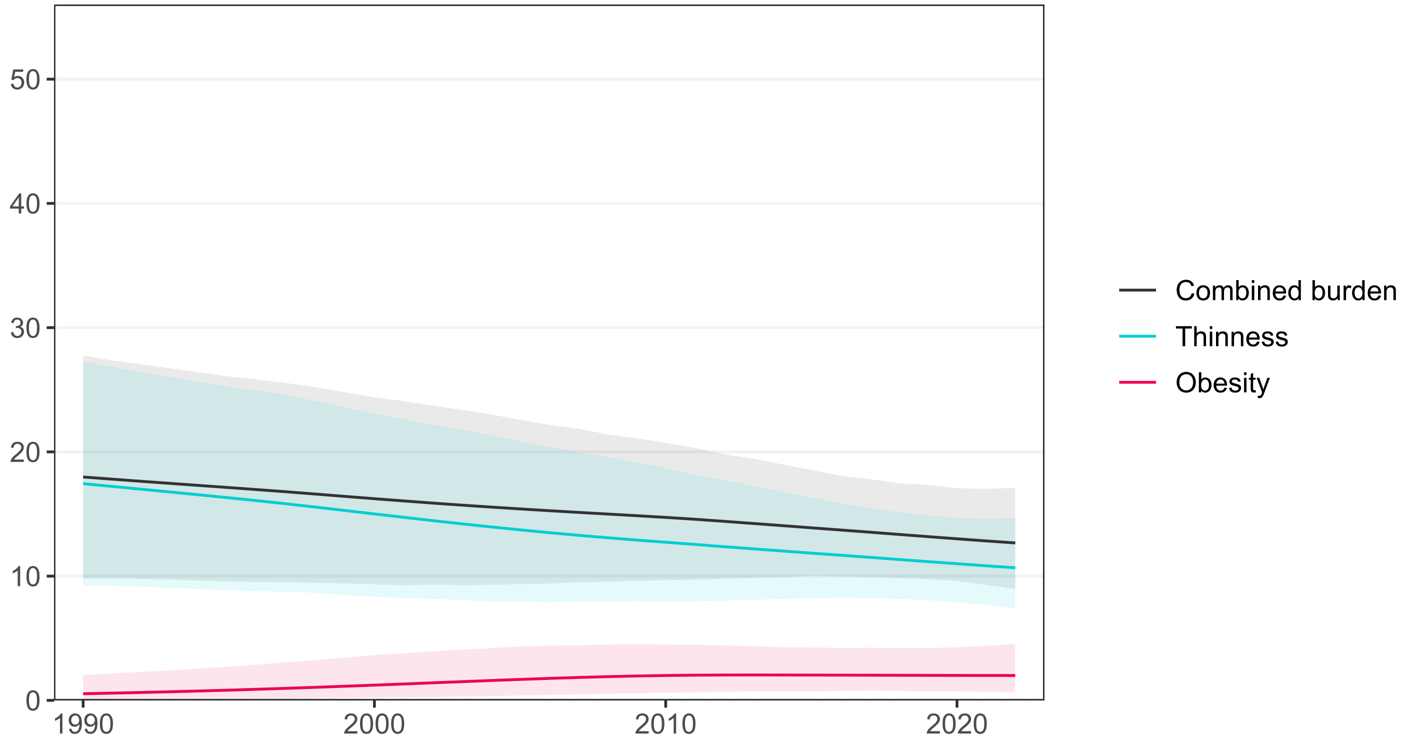


## Adults

### Women

9 studies (7 national)

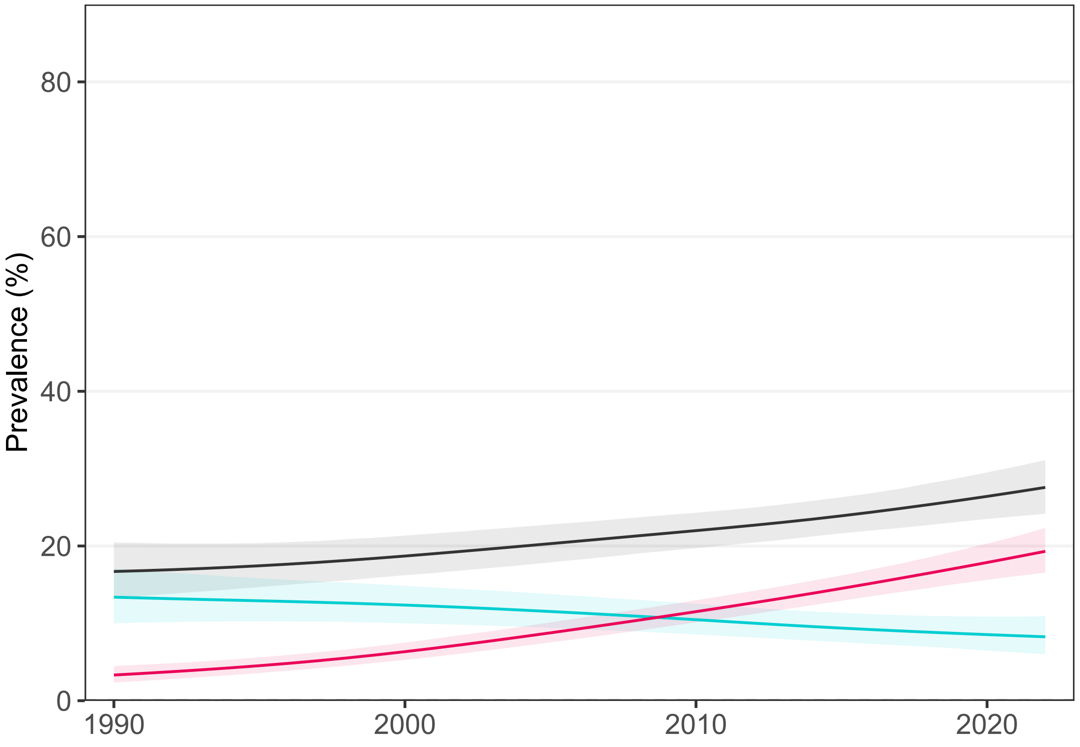


### Men

4 studies (2 national)

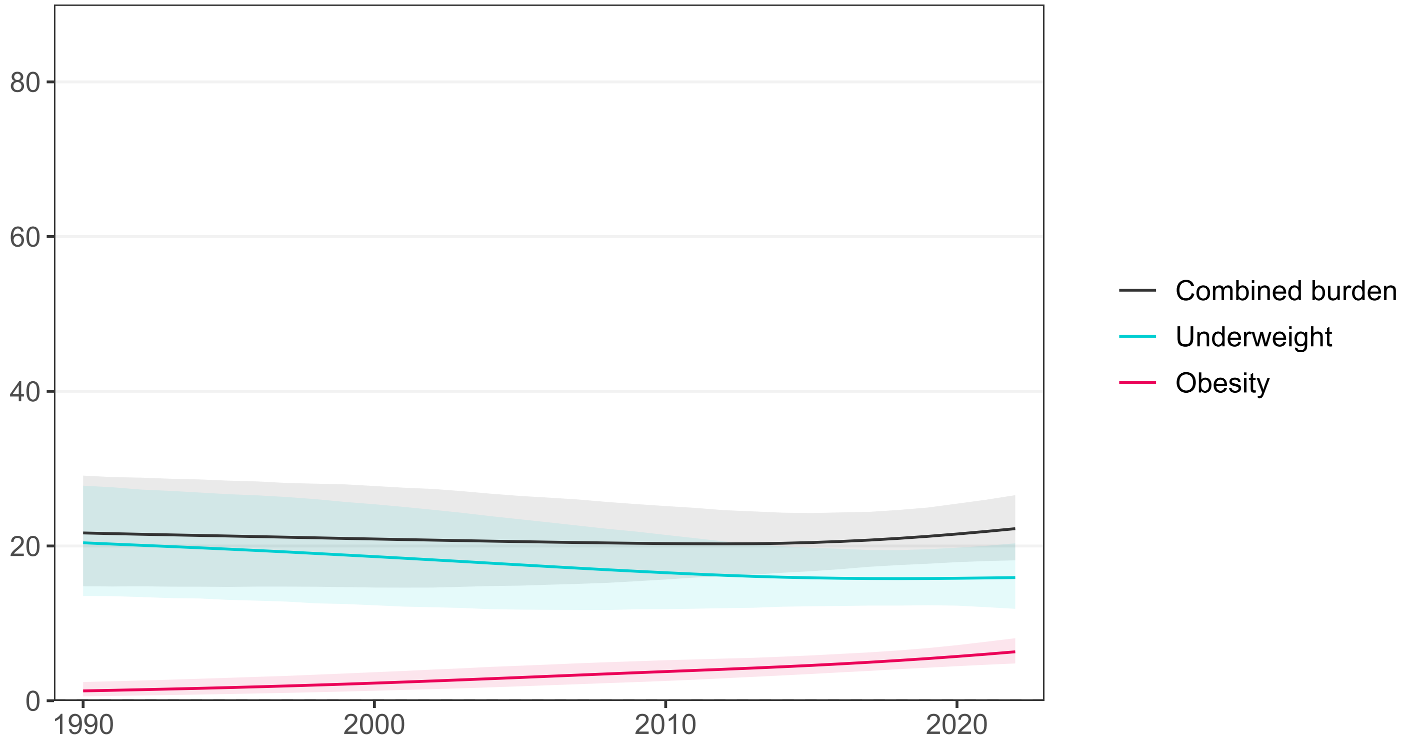

# Kiribati

## School-aged children and adolescents

### Girls
3 studies (3 national)

### Boys
2 studies (2 national)

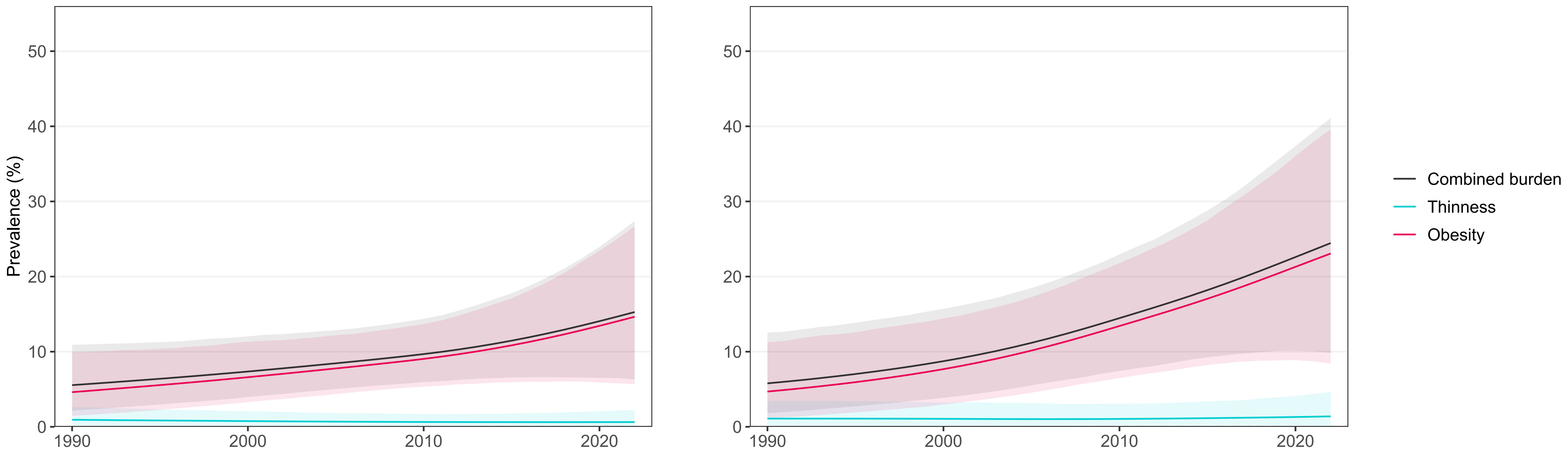


## Adults

### Women
4 studies (2 national)

### Men
4 studies (2 national)

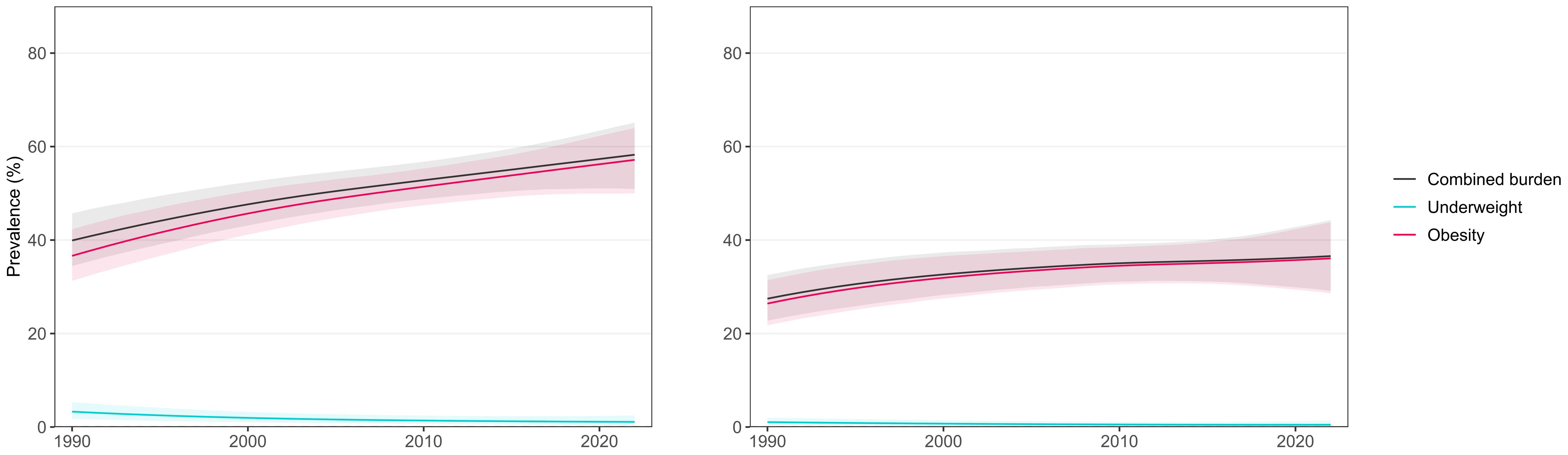

# Kuwait

## School-aged children and adolescents

Girls
15 studies (15 national)

Boys
17 studies (15 national)

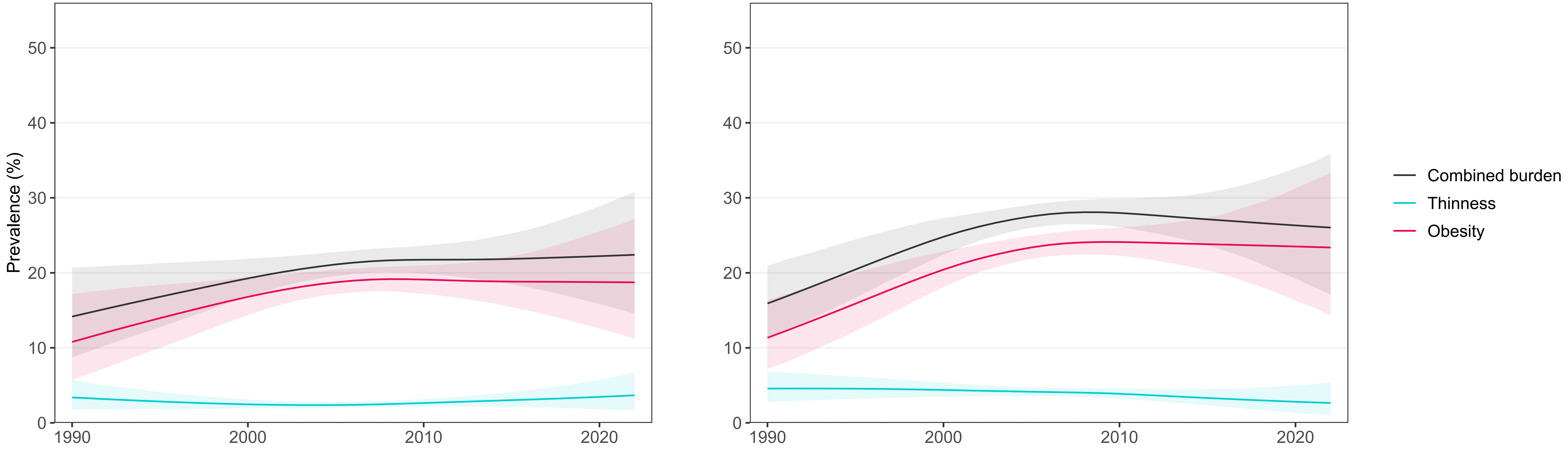


## Adults

Women
25 studies (24 national)

Men
27 studies (24 national)

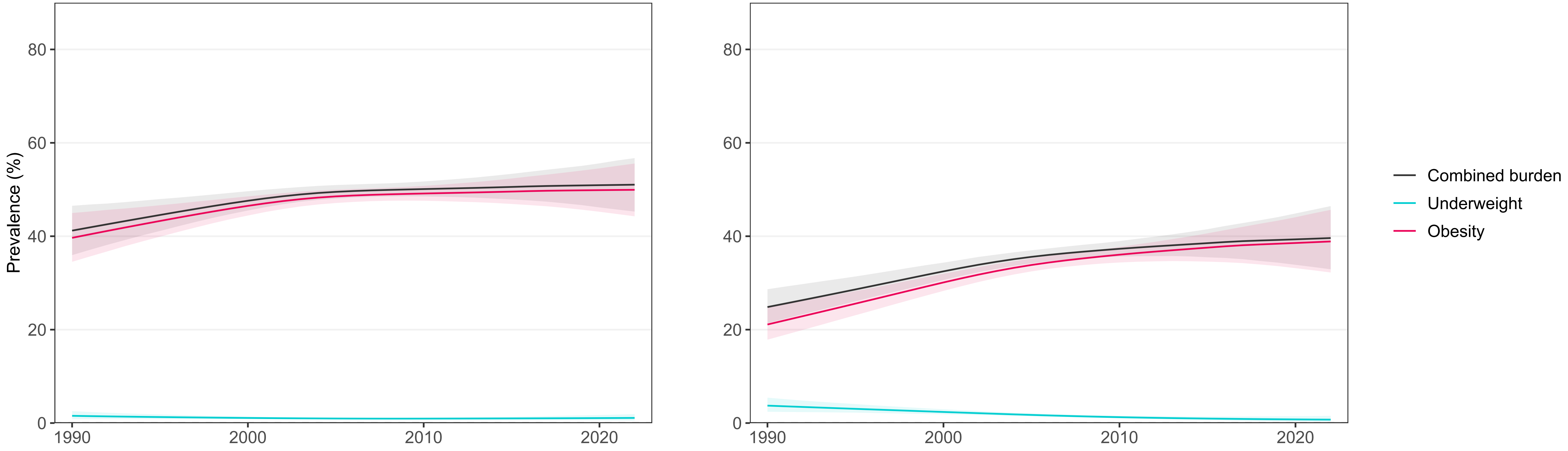

# Kyrgyzstan

## School-aged children and adolescents

### Girls

4 studies (4 national)

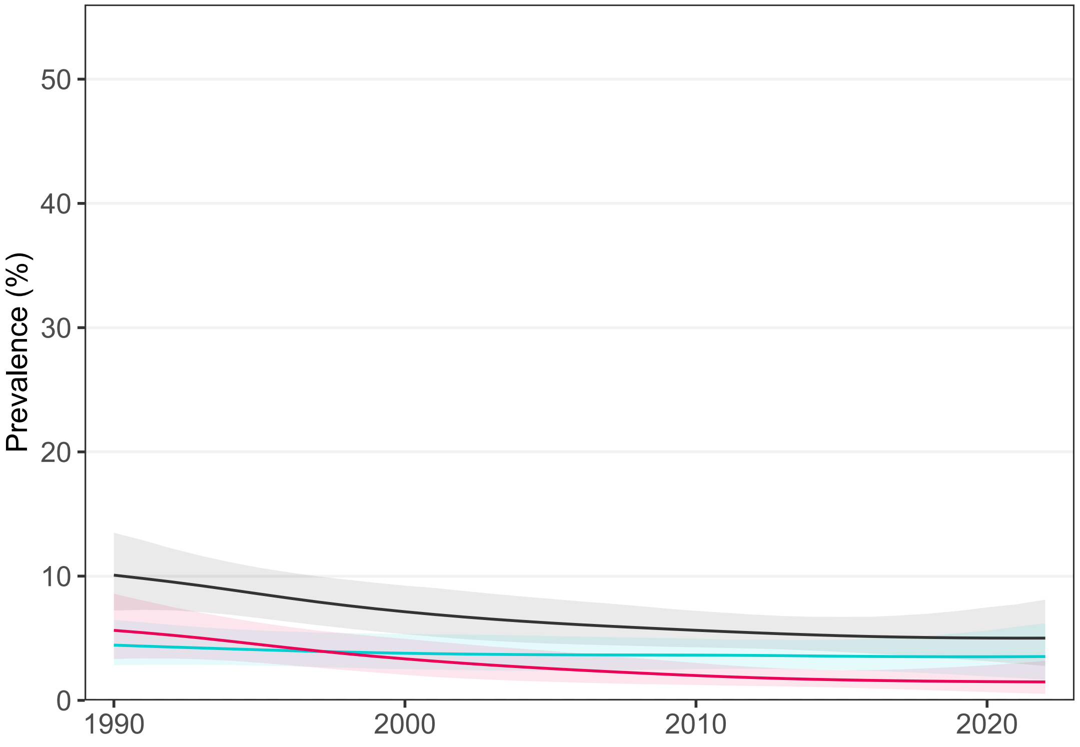


### Boys

2 studies (2 national)

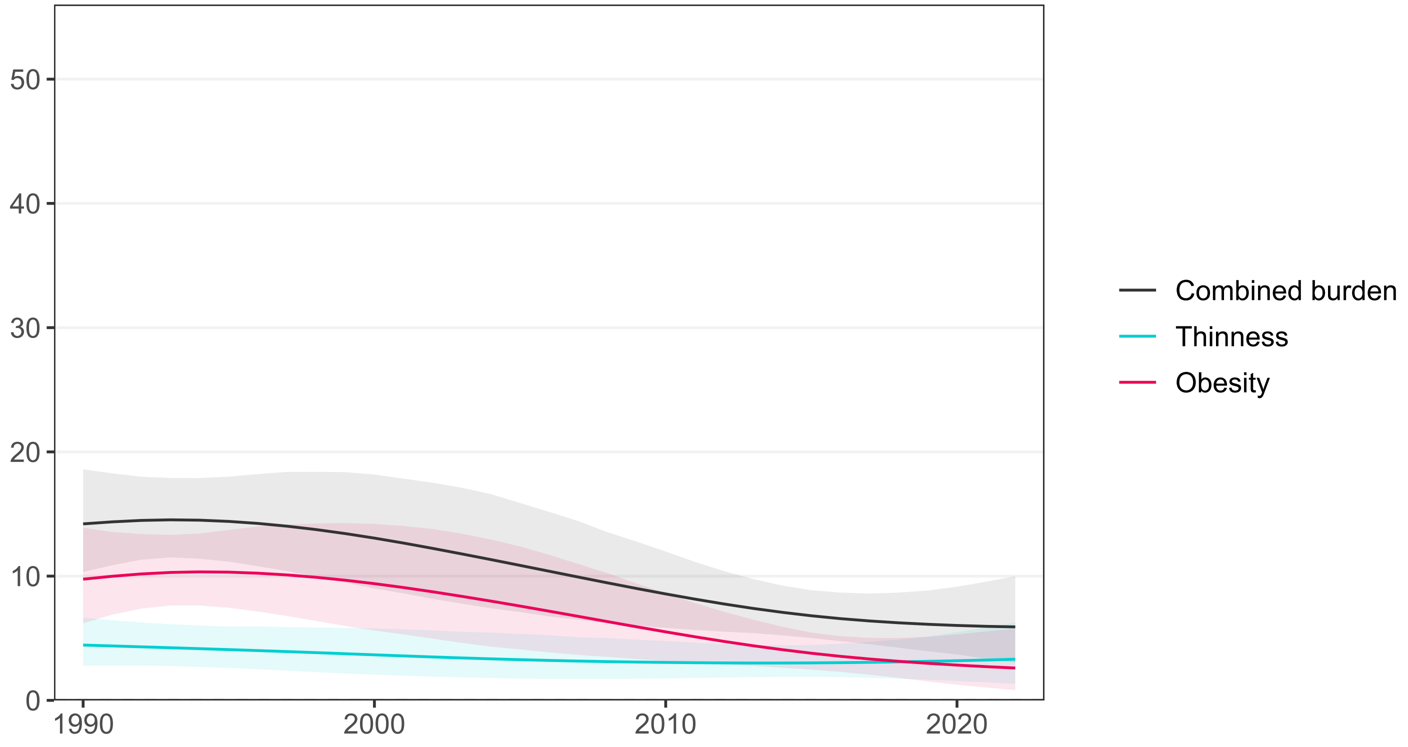


## Adults

### Women

4 studies (4 national)

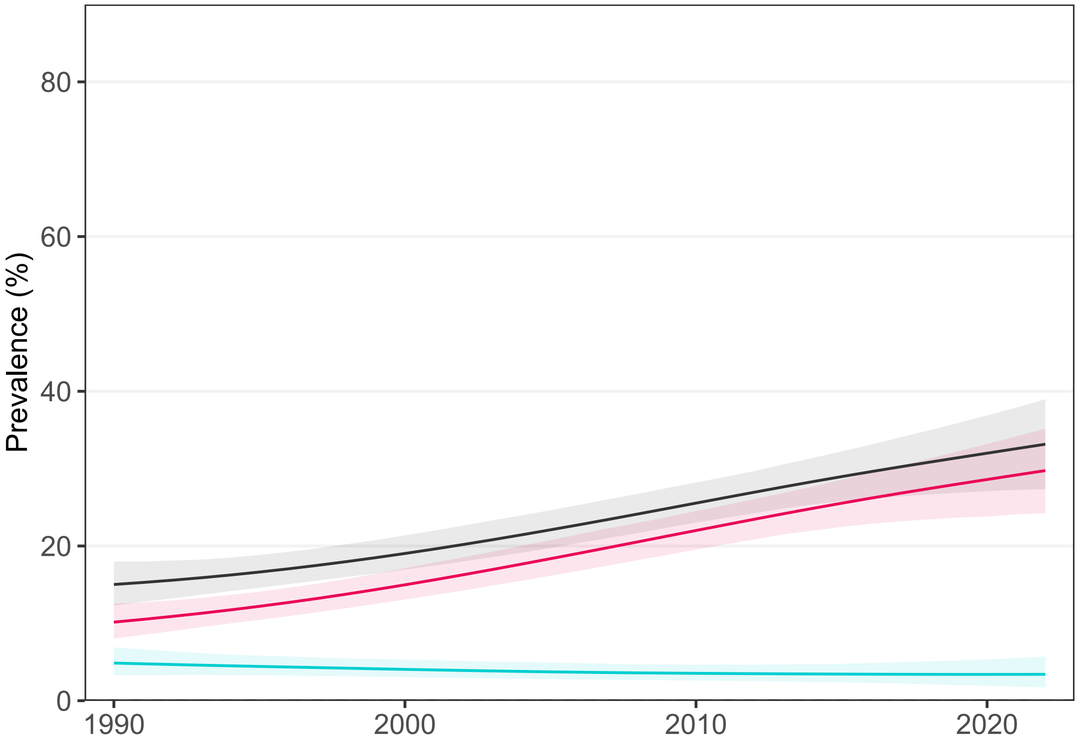


### Men

2 studies (2 national)

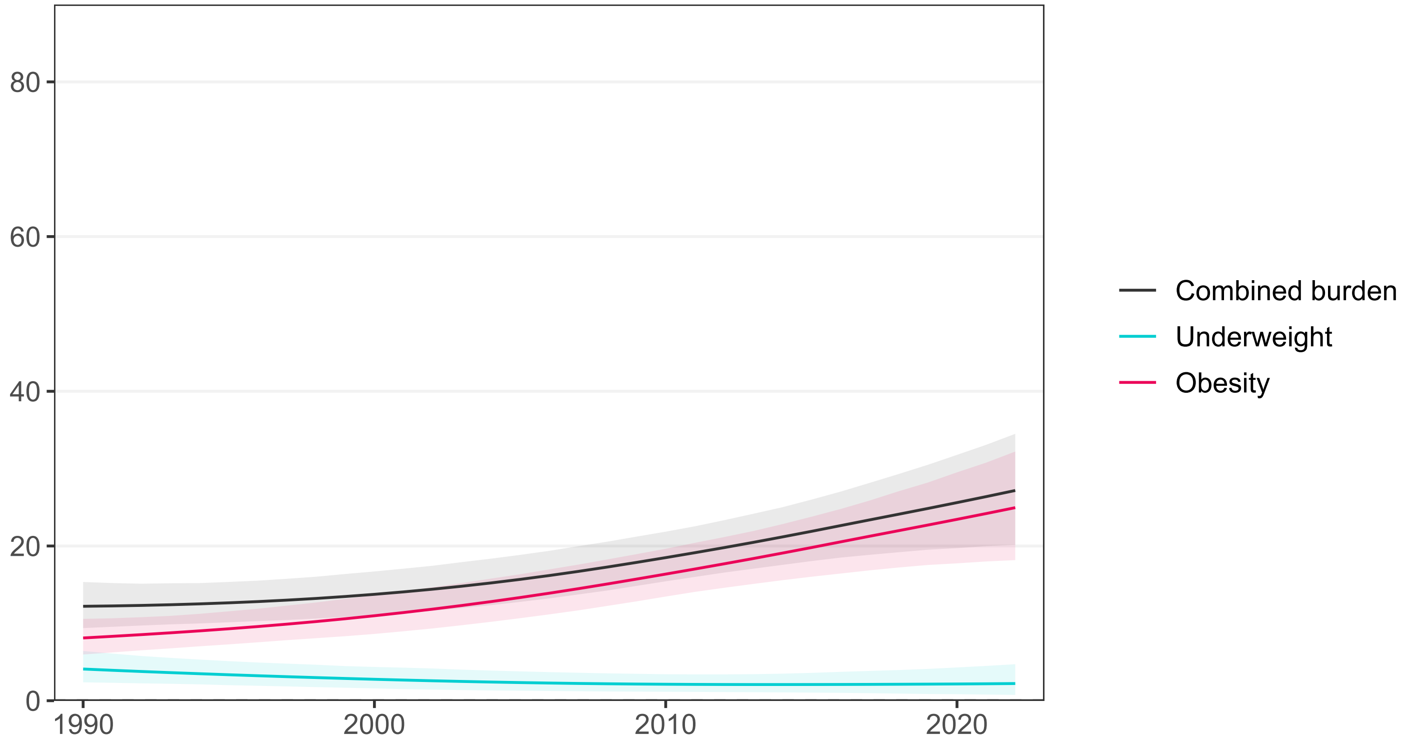

# Lao PDR

## School-aged children and adolescents

### Girls

2 studies (2 national)

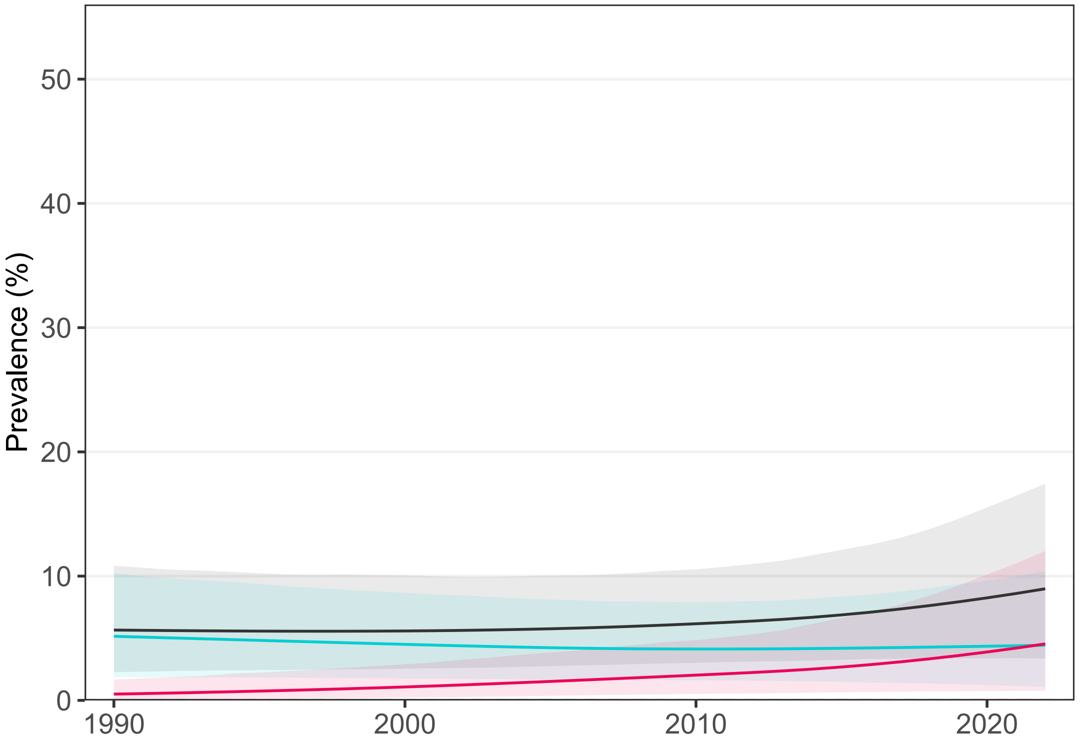


### Boys

1 study (1 national)

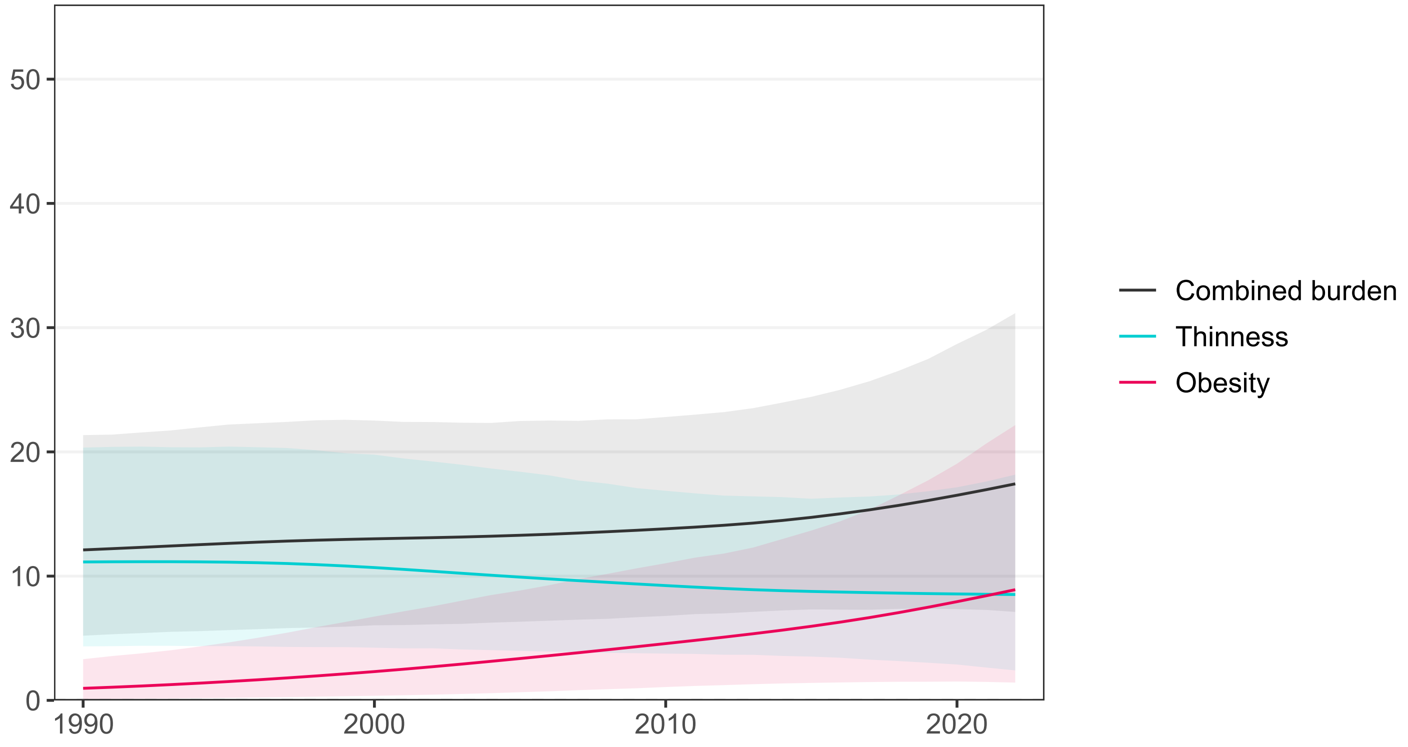


## Adults

### Women

3 studies (2 national)

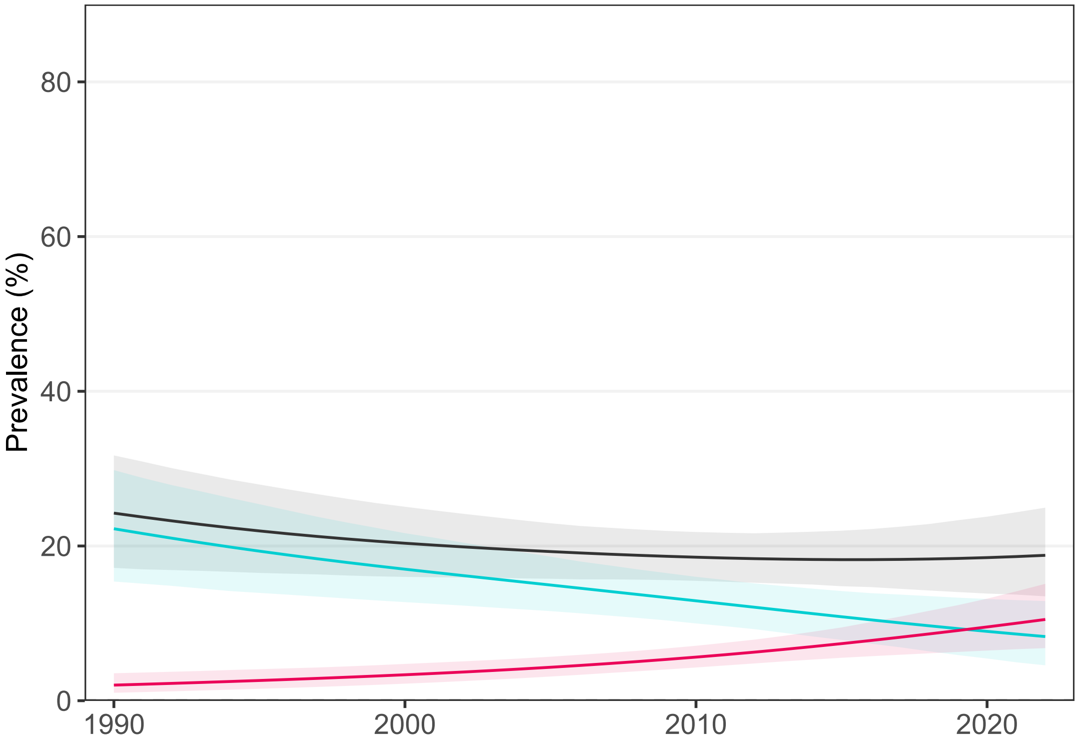


### Men

2 studies (1 national)

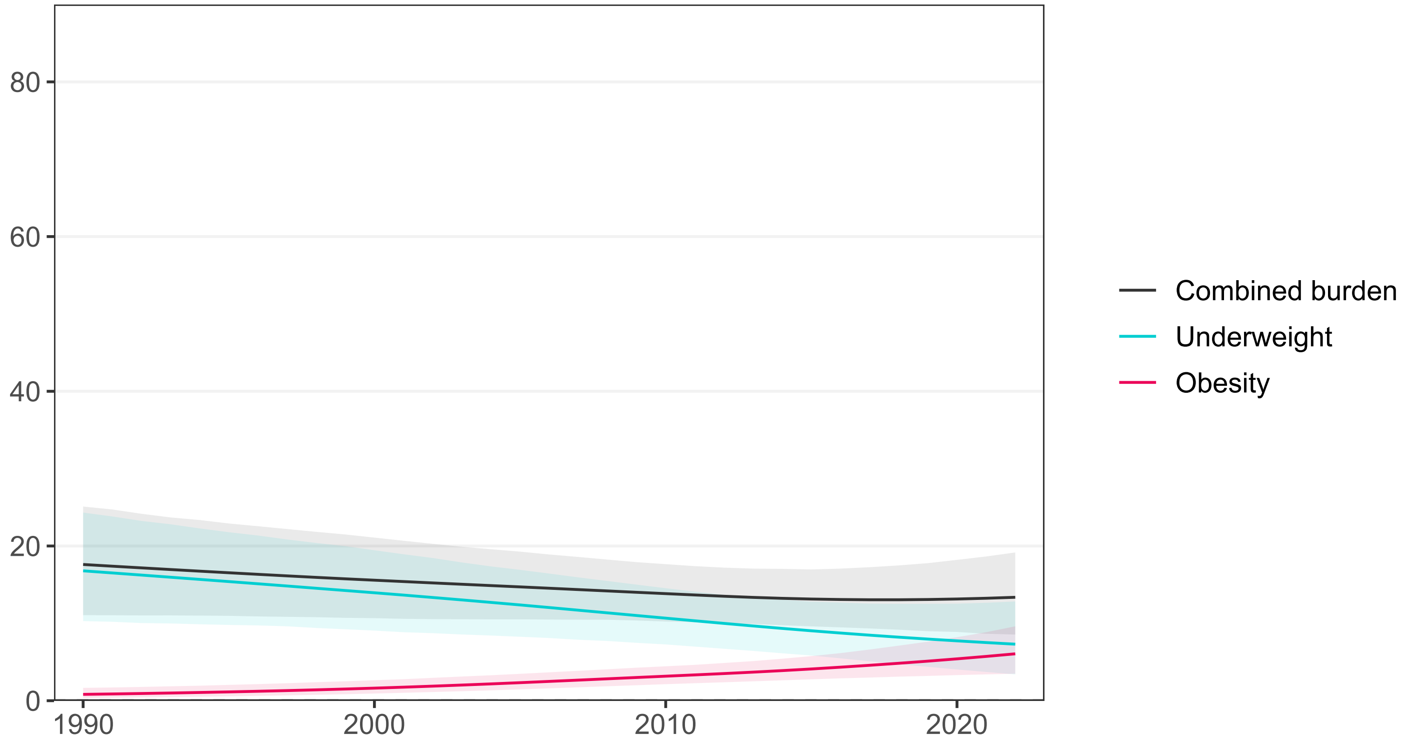

# Latvia

## School-aged children and adolescents

### Girls

5 studies (5 national)

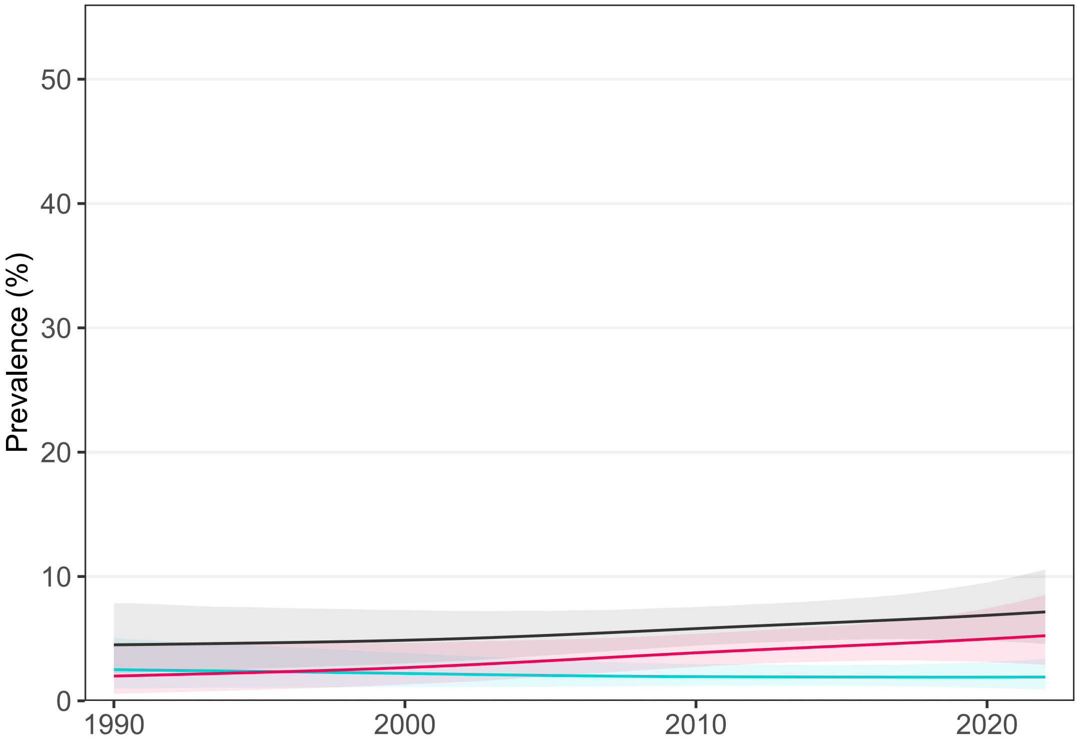


### Boys

5 studies (5 national)

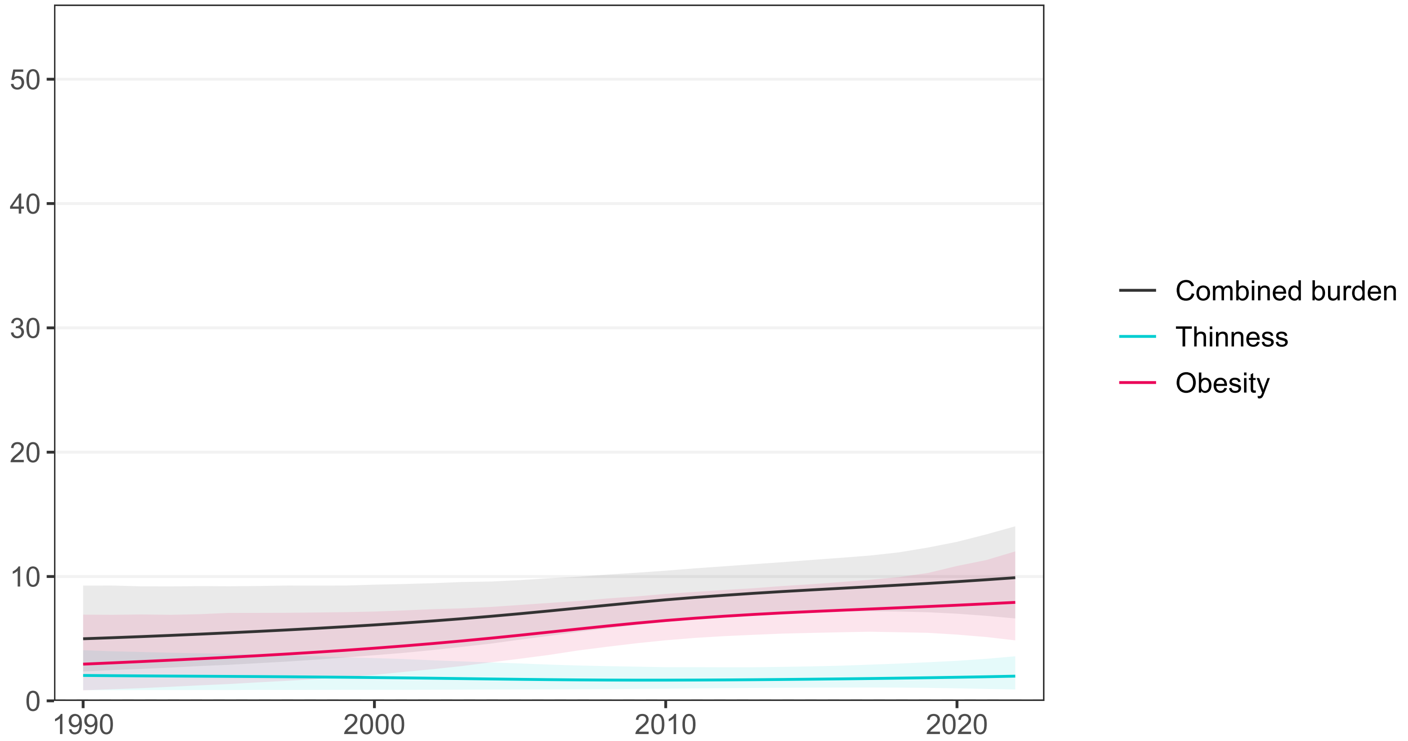


## Adults

### Women

2 studies (2 national)

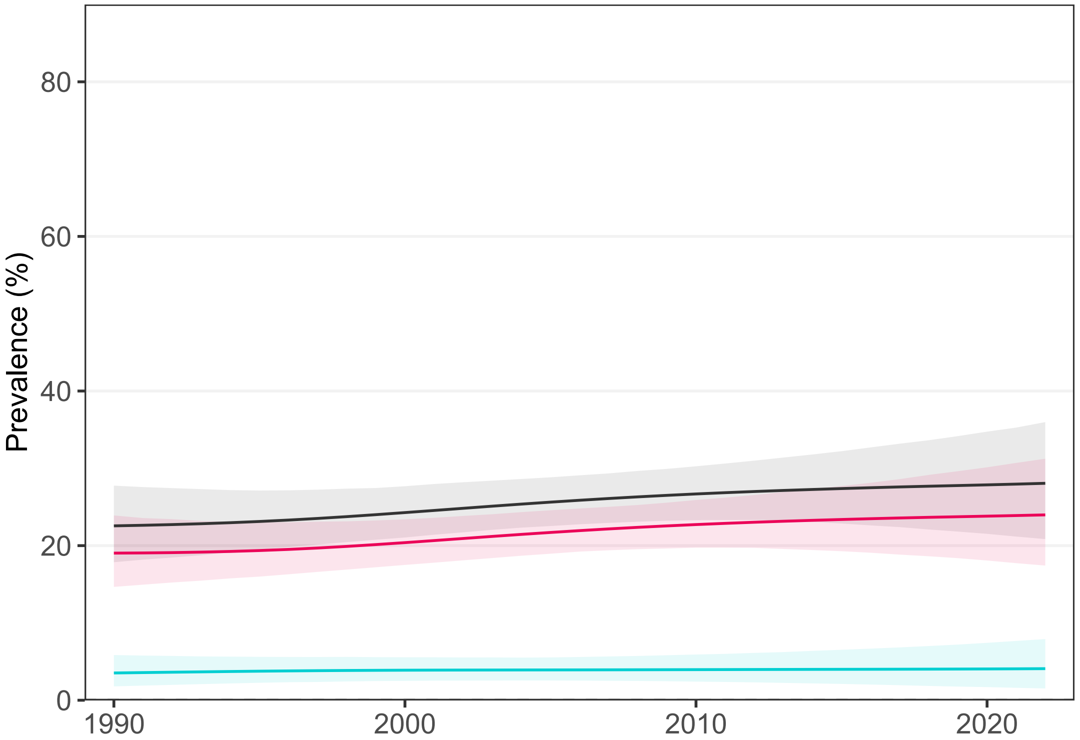


### Men

2 studies (2 national)

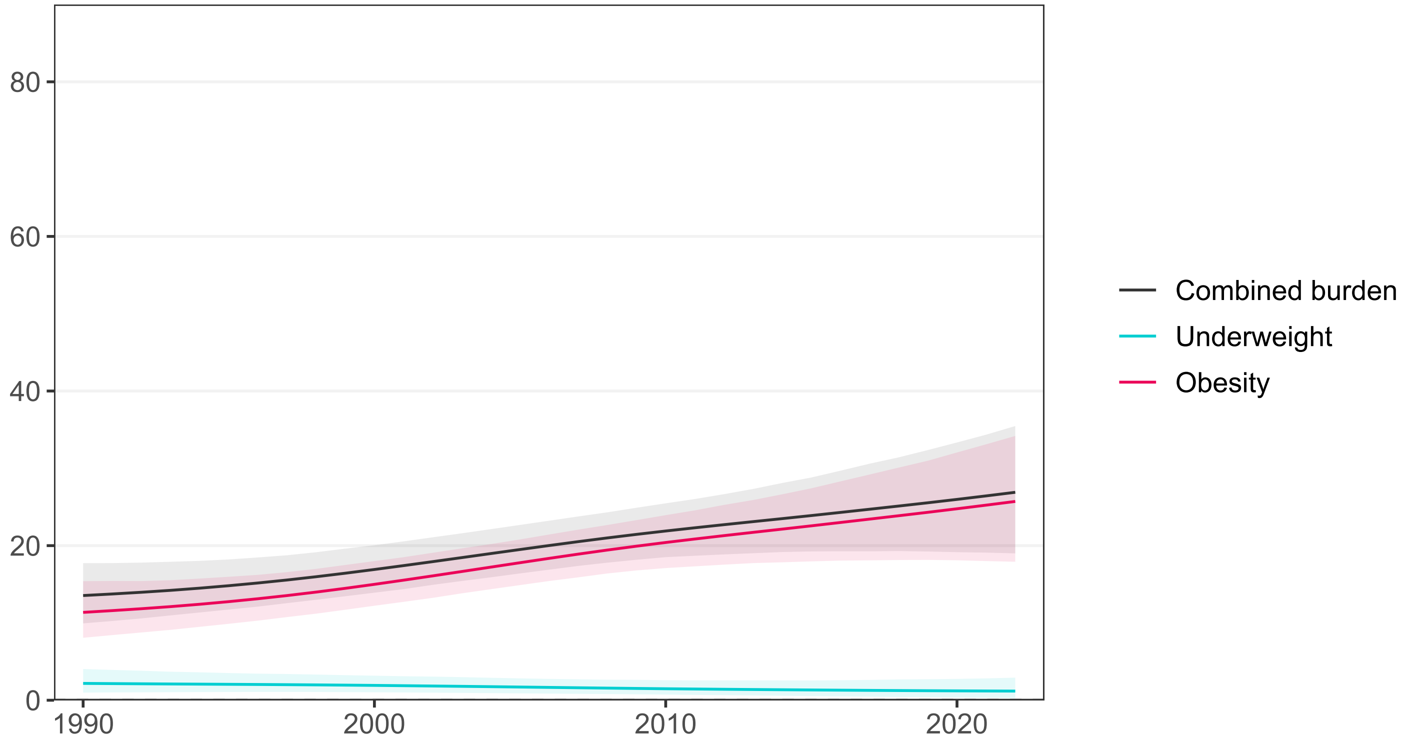

# Lebanon

## School-aged children and adolescents

### Girls

3 studies (3 national)

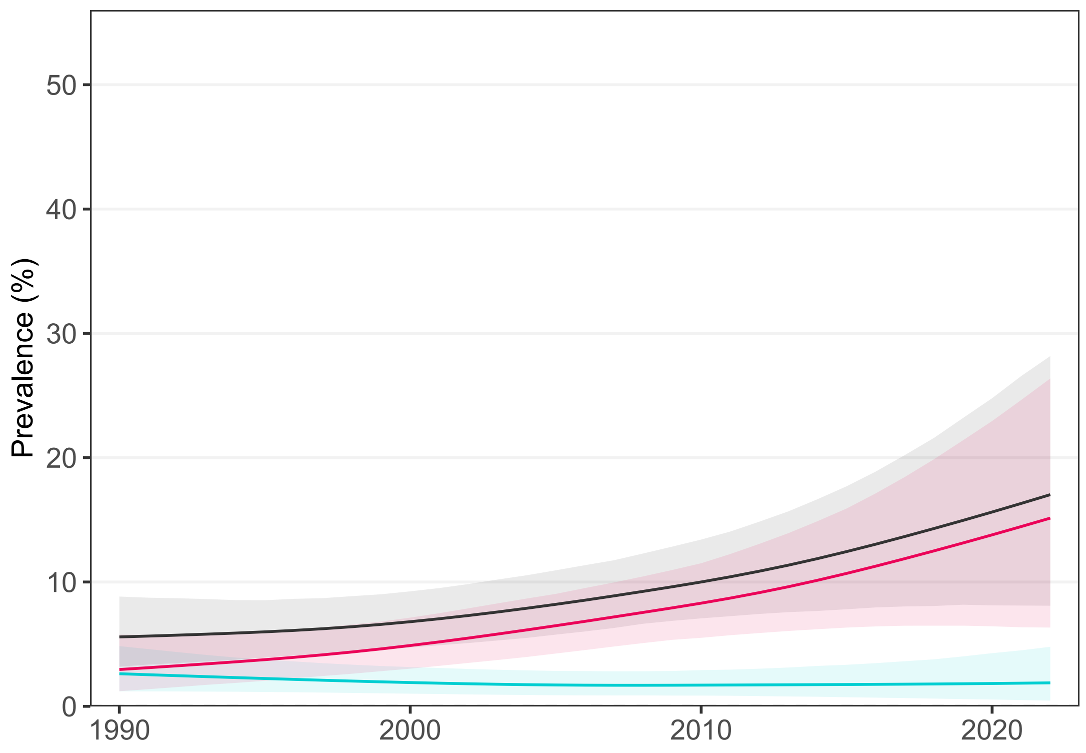


### Boys

3 studies (3 national)

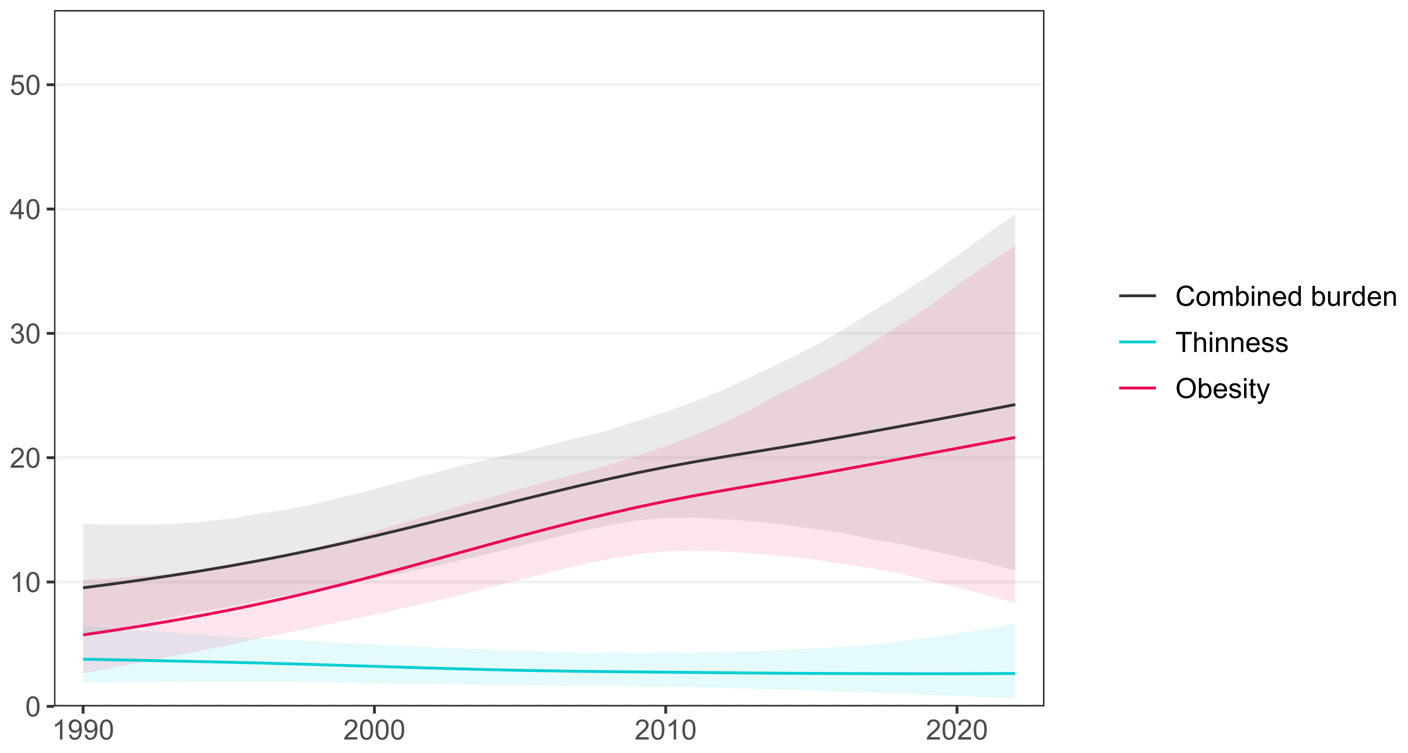


## Adults

### Women

3 studies (3 national)

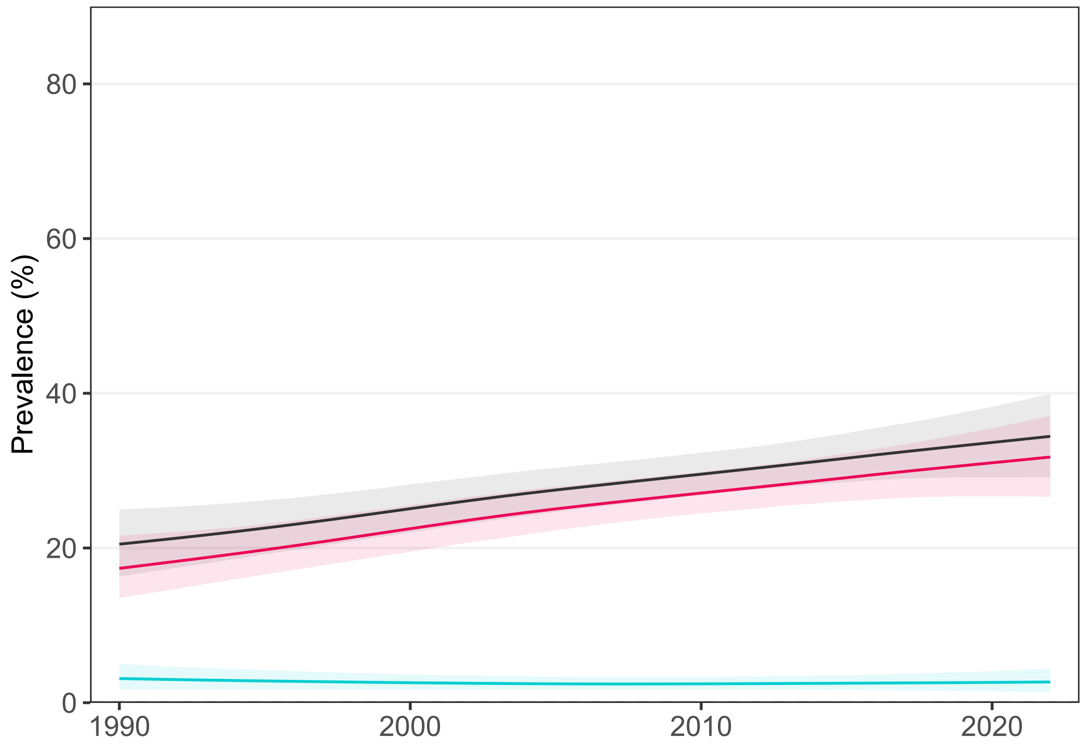


### Men

3 studies (3 national)

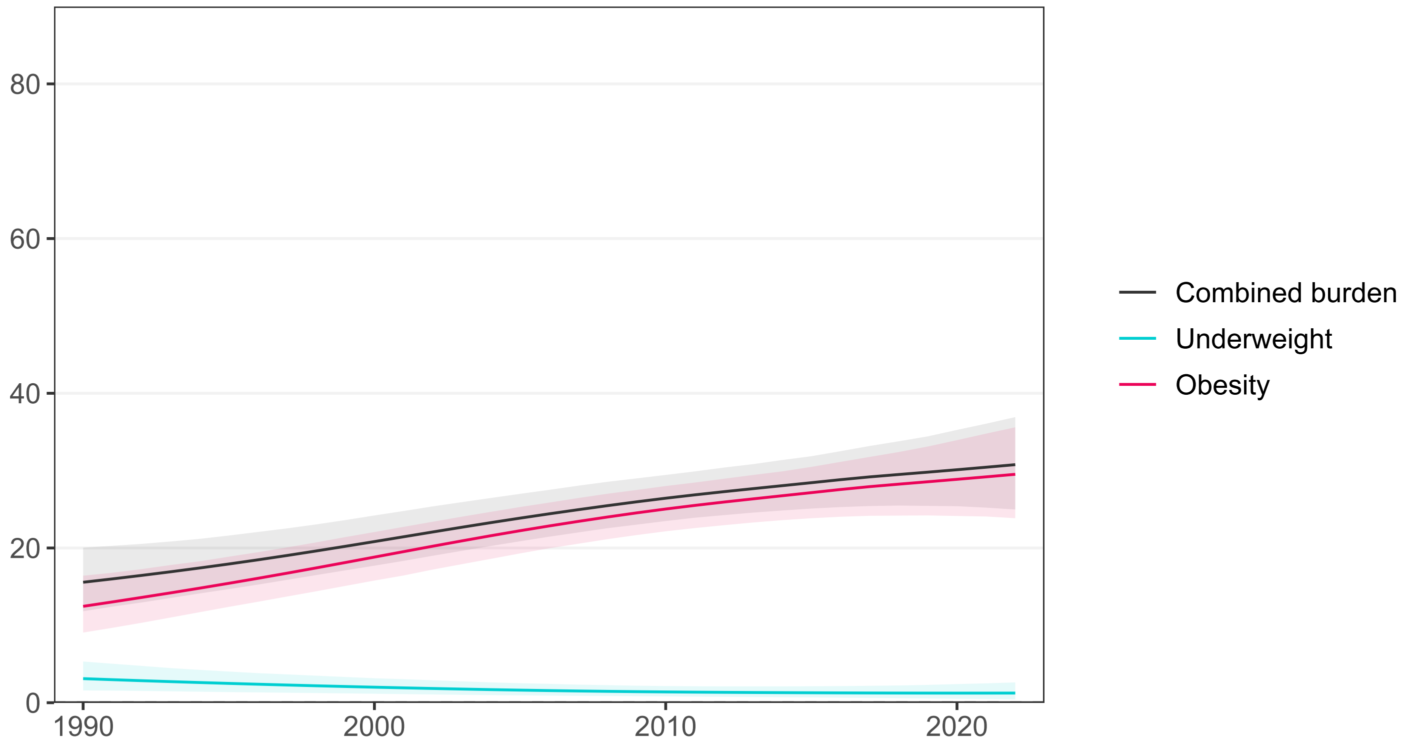

# Lesotho

## School-aged children and adolescents

### Girls

3 studies (3 national)

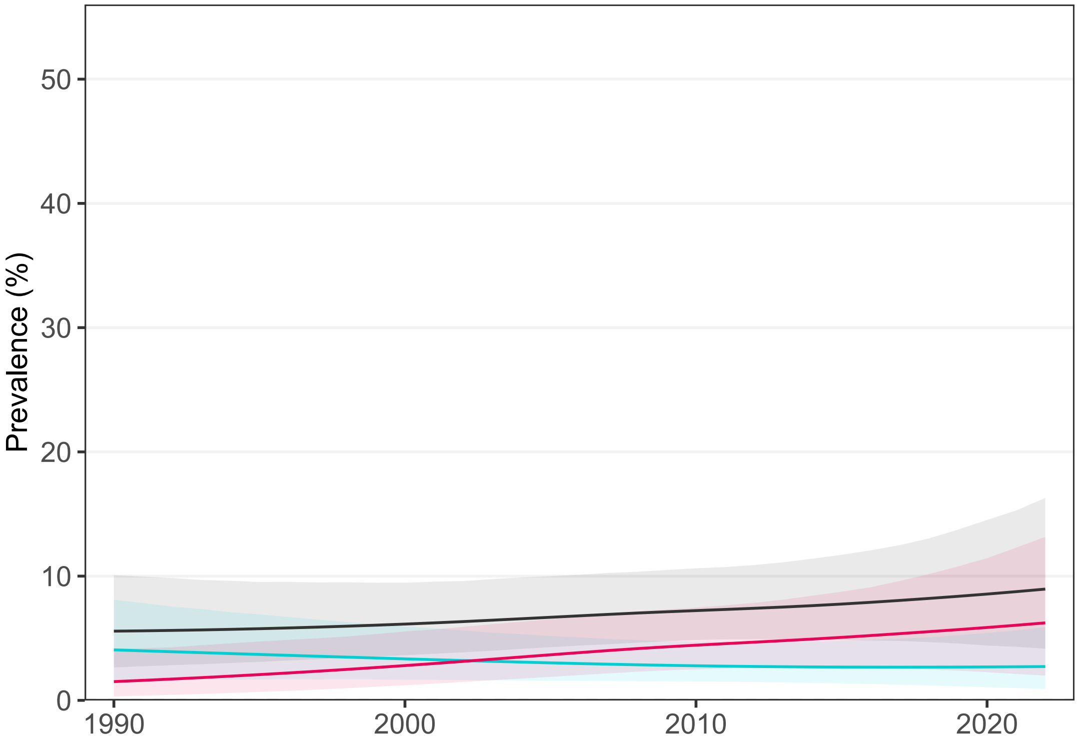


### Boys

2 studies (2 national)

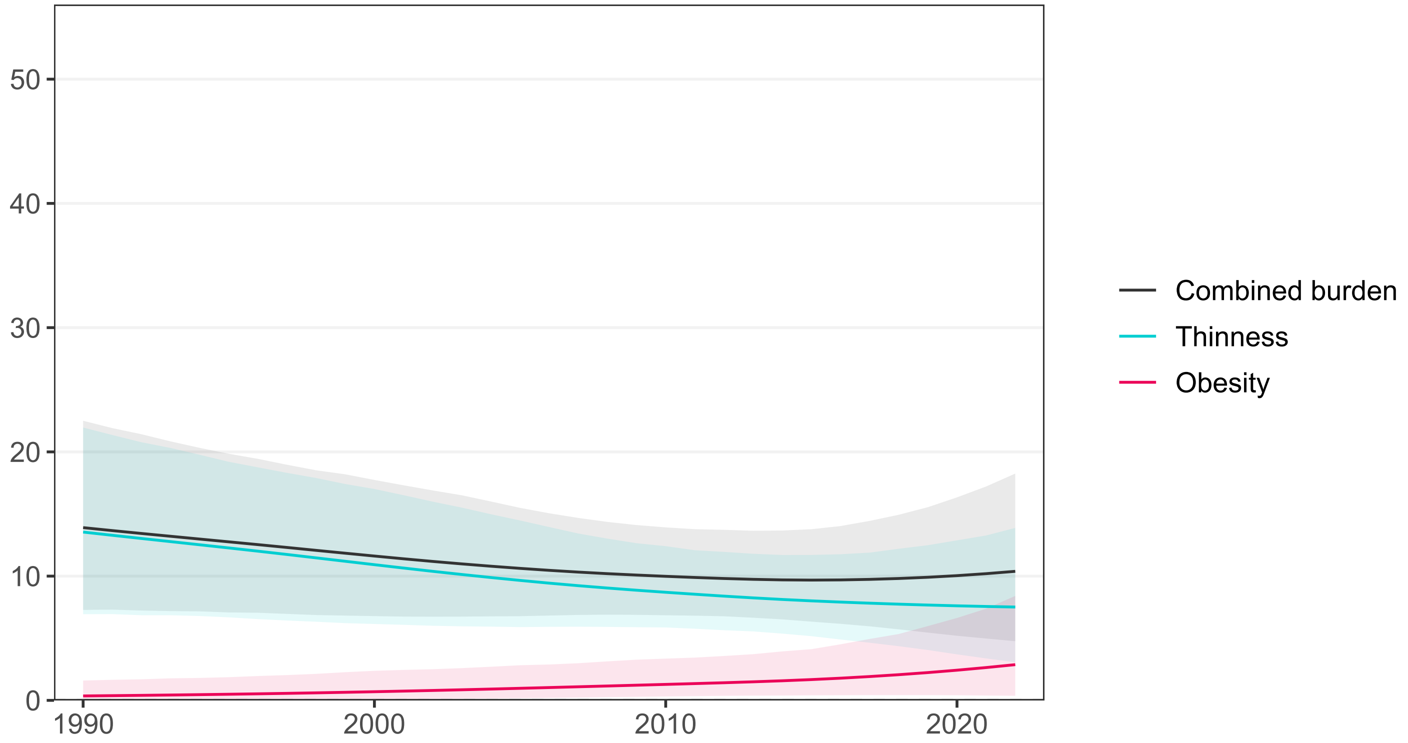


## Adults

### Women

5 studies (5 national)

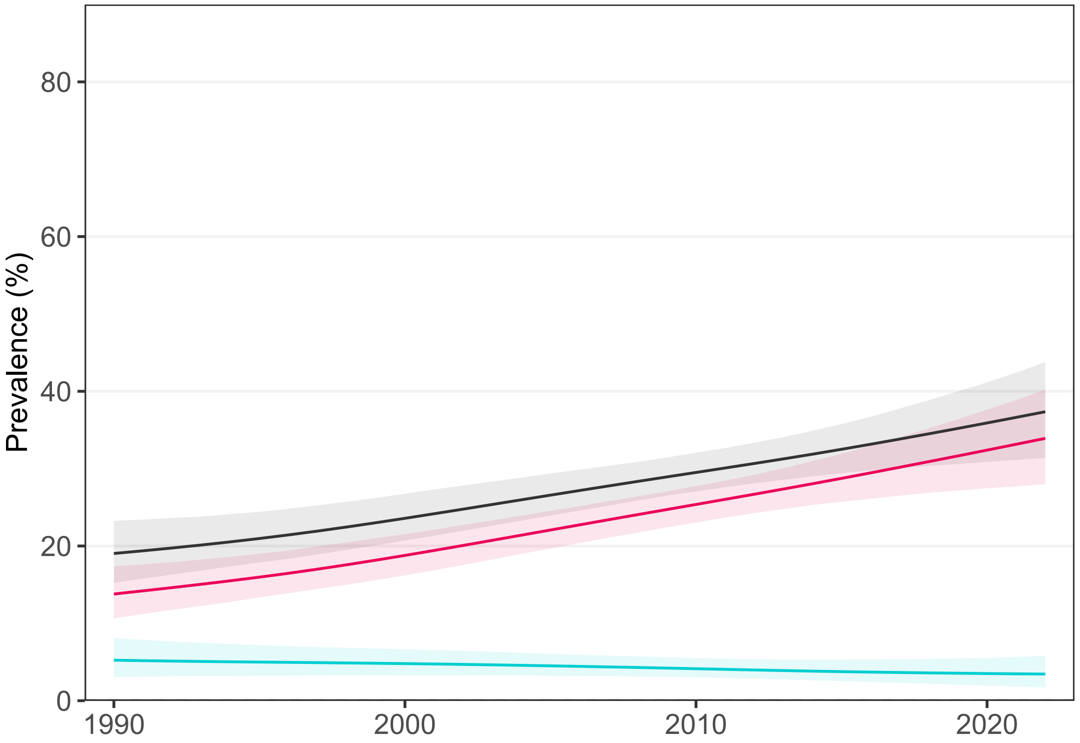


### Men

3 studies (3 national)

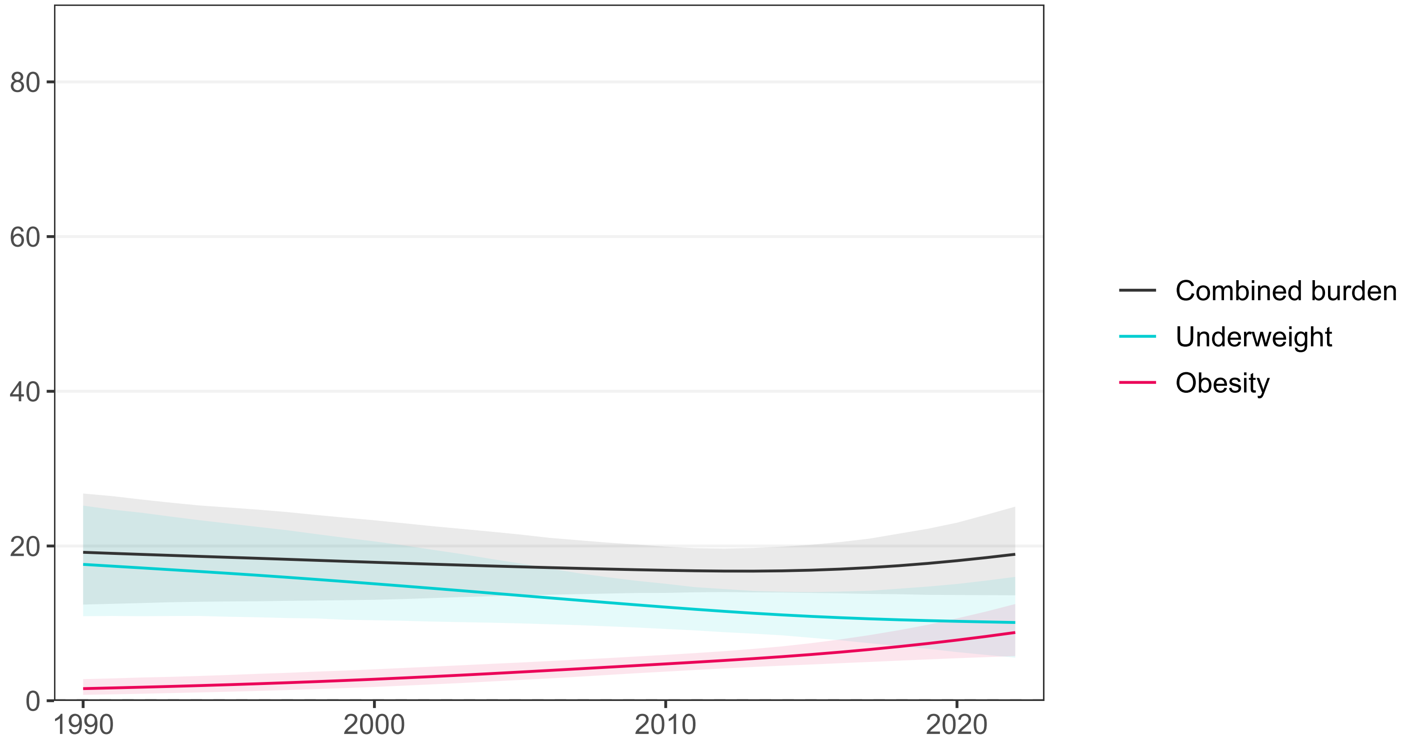

# Liberia

## School-aged children and adolescents

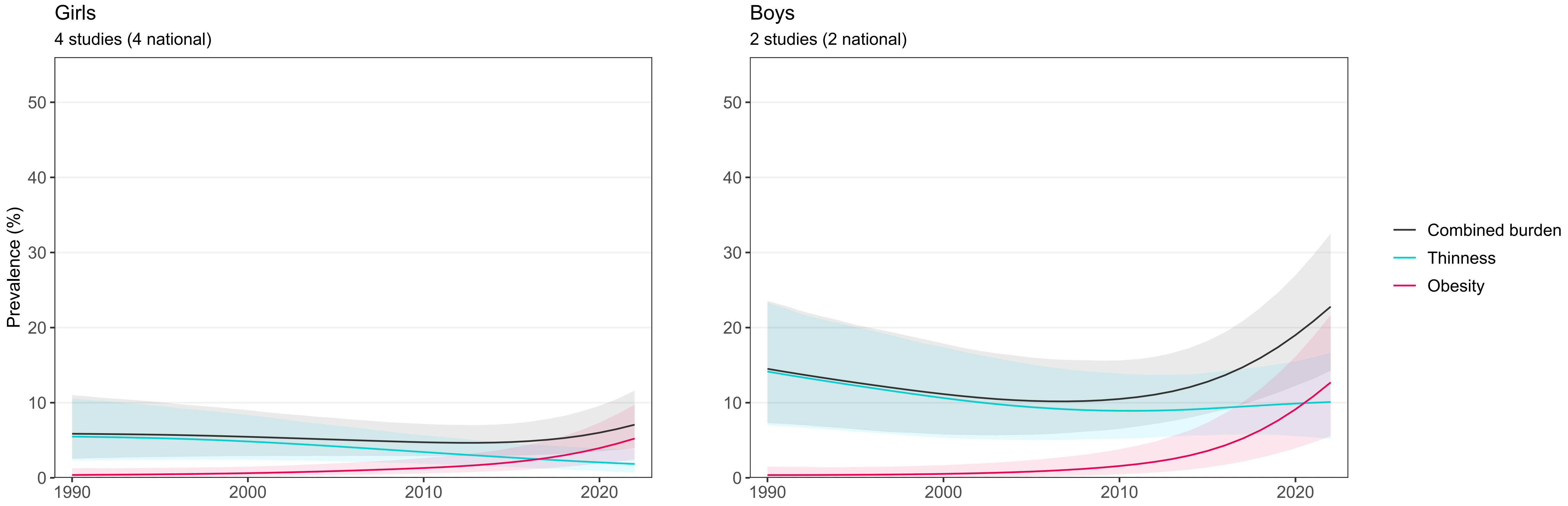


## Adults

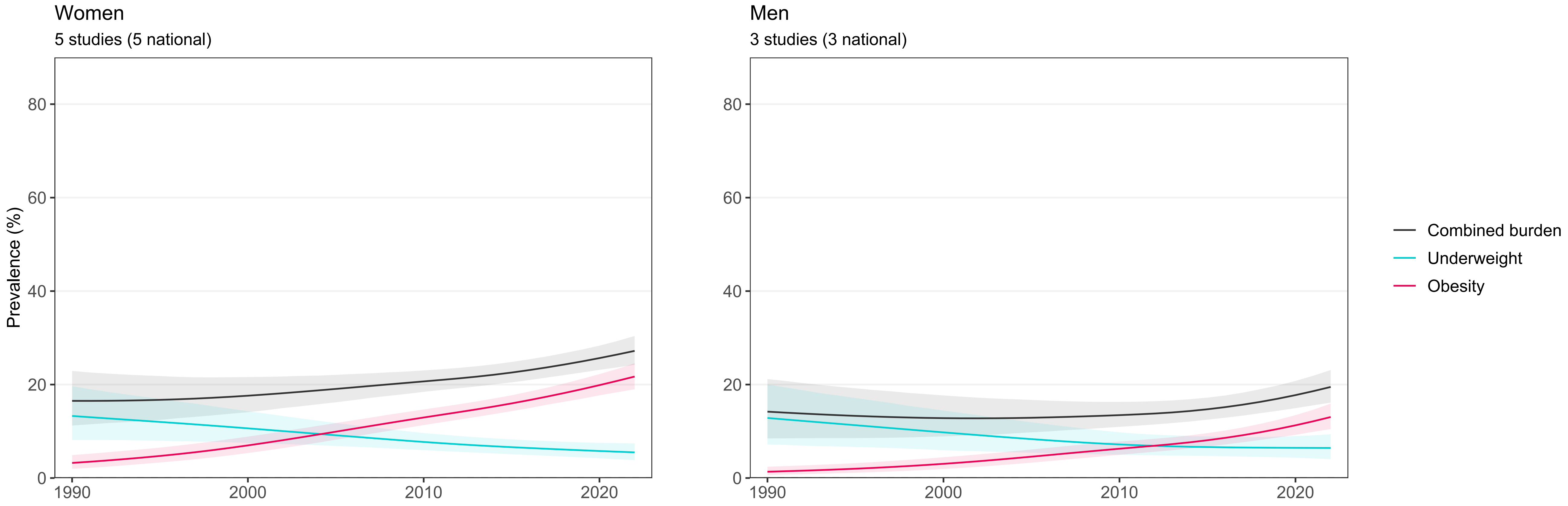

# Libya

## School-aged children and adolescents

### Girls

1 study (1 national)

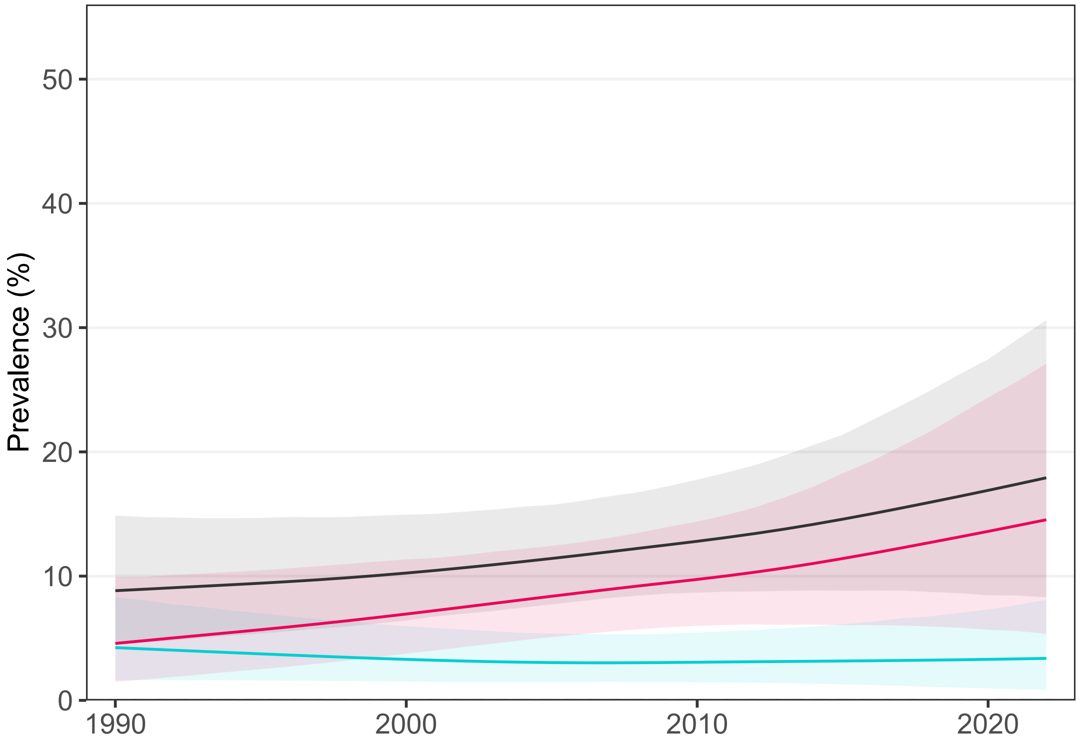


### Boys

1 study (1 national)

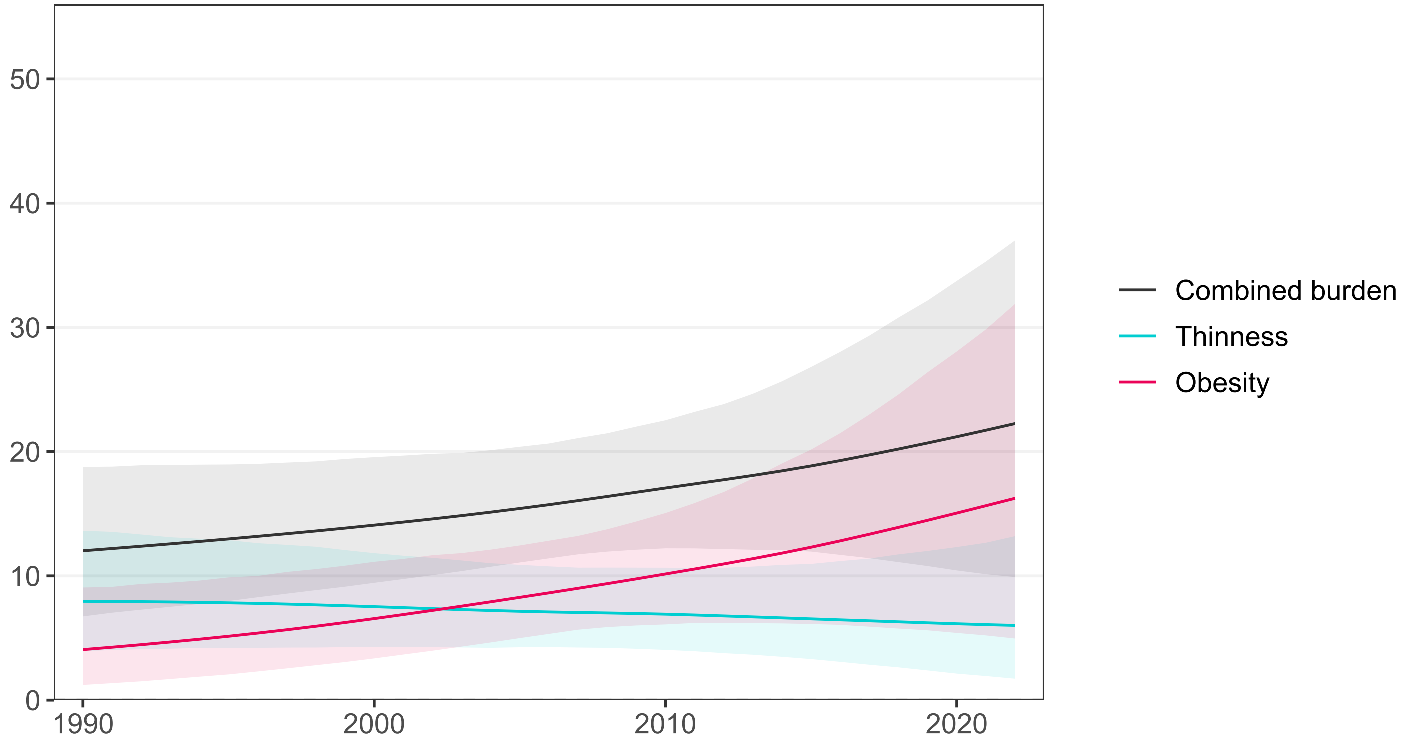


## Adults

### Women

2 studies (1 national)

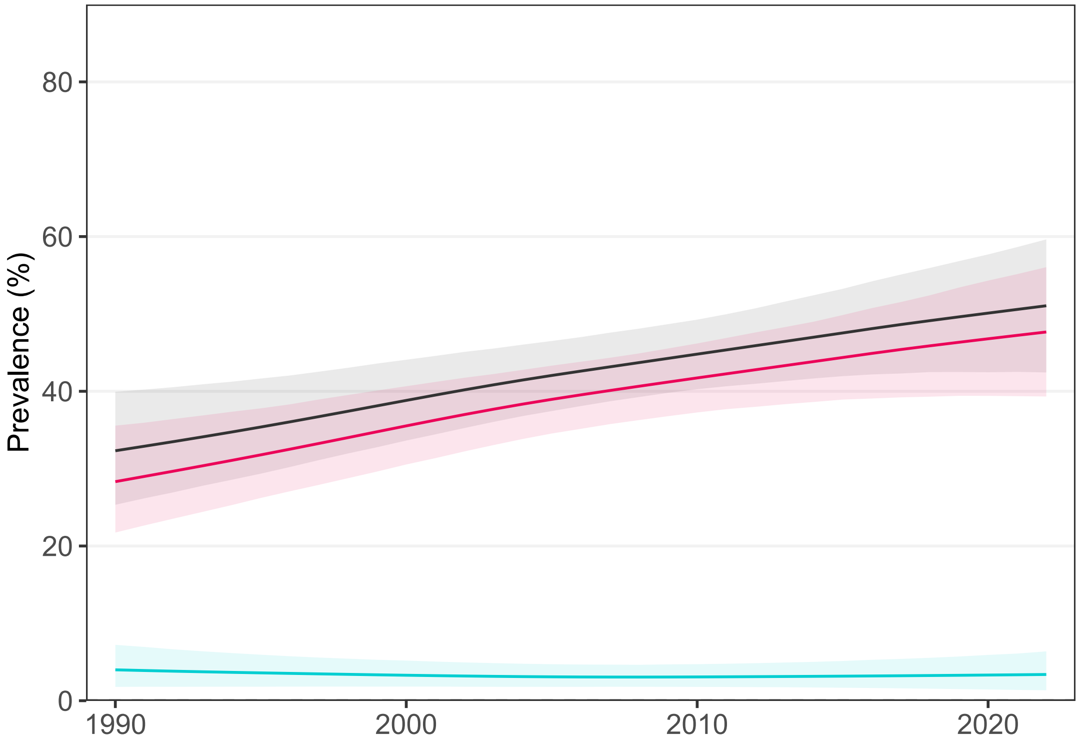


### Men

2 studies (1 national)

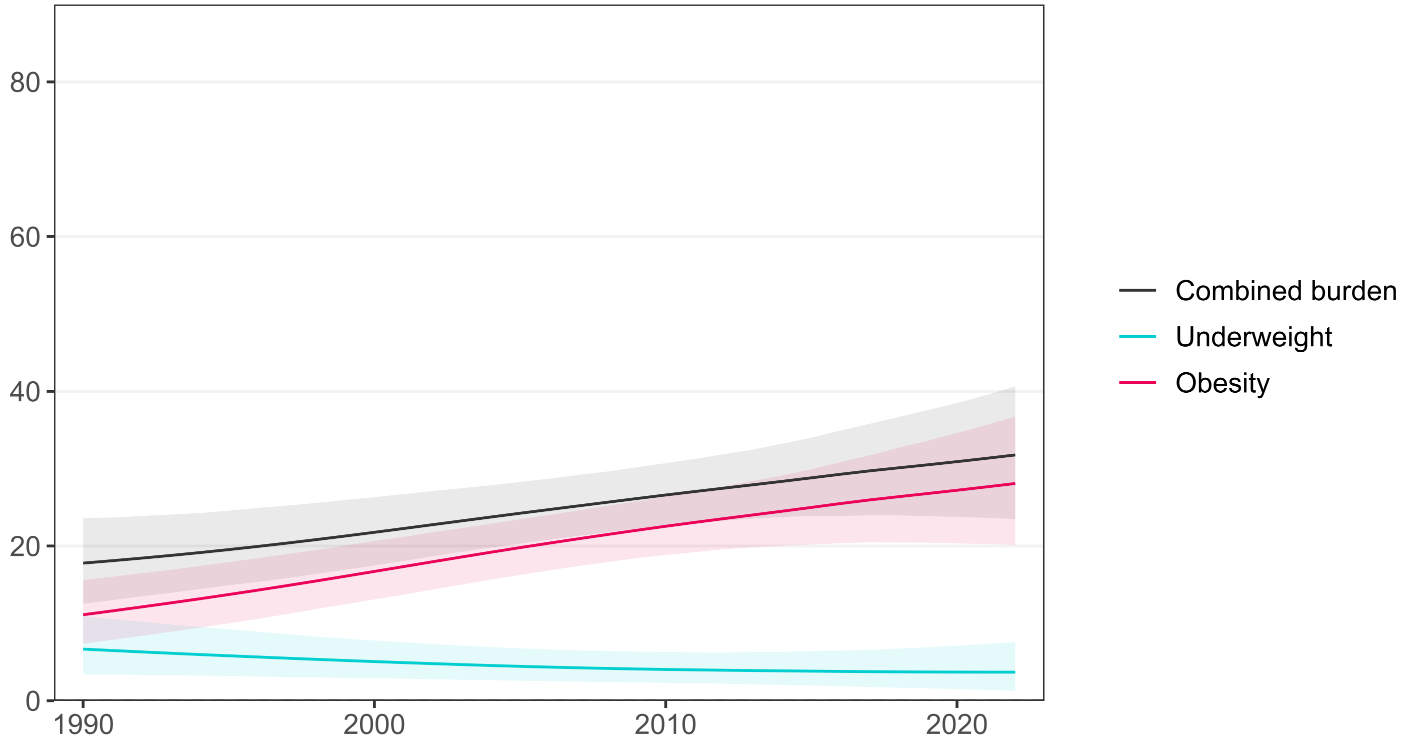

# Lithuania

## School-aged children and adolescents

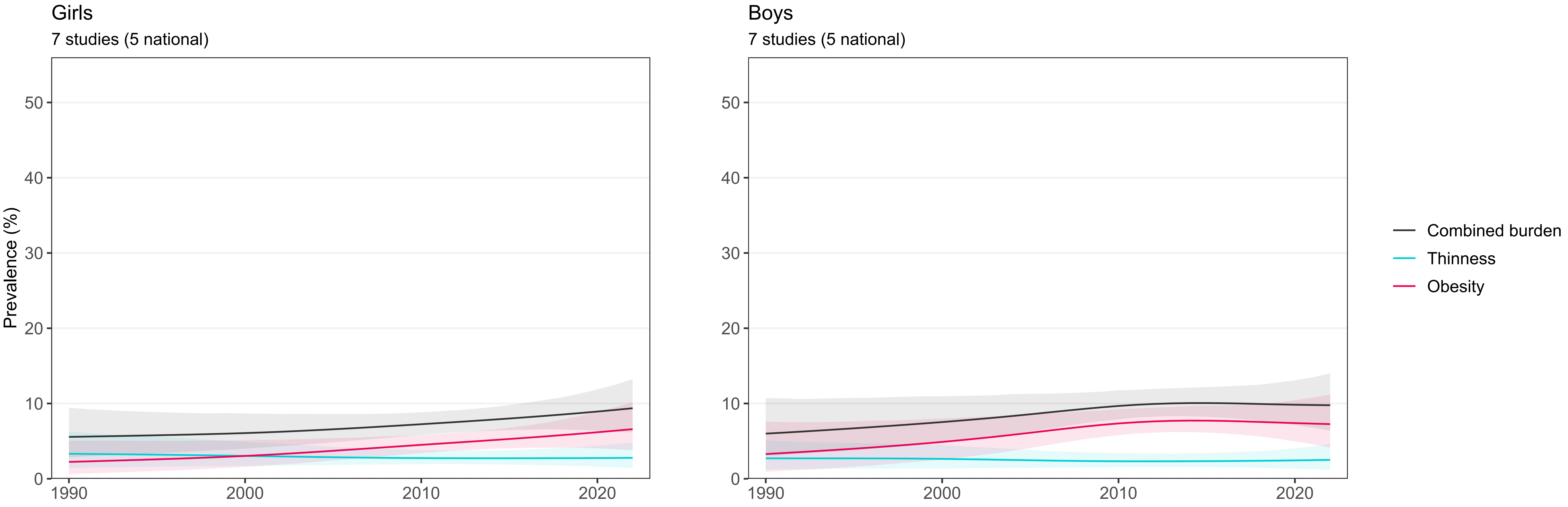


## Adults

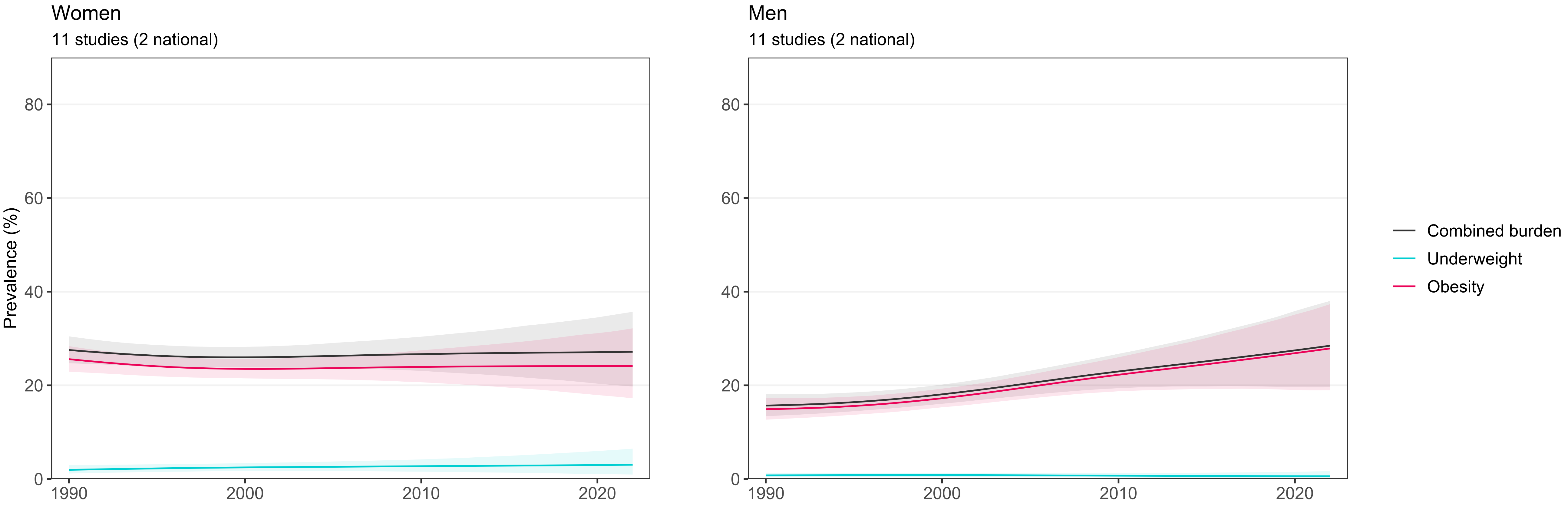

# Luxembourg

## School-aged children and adolescents

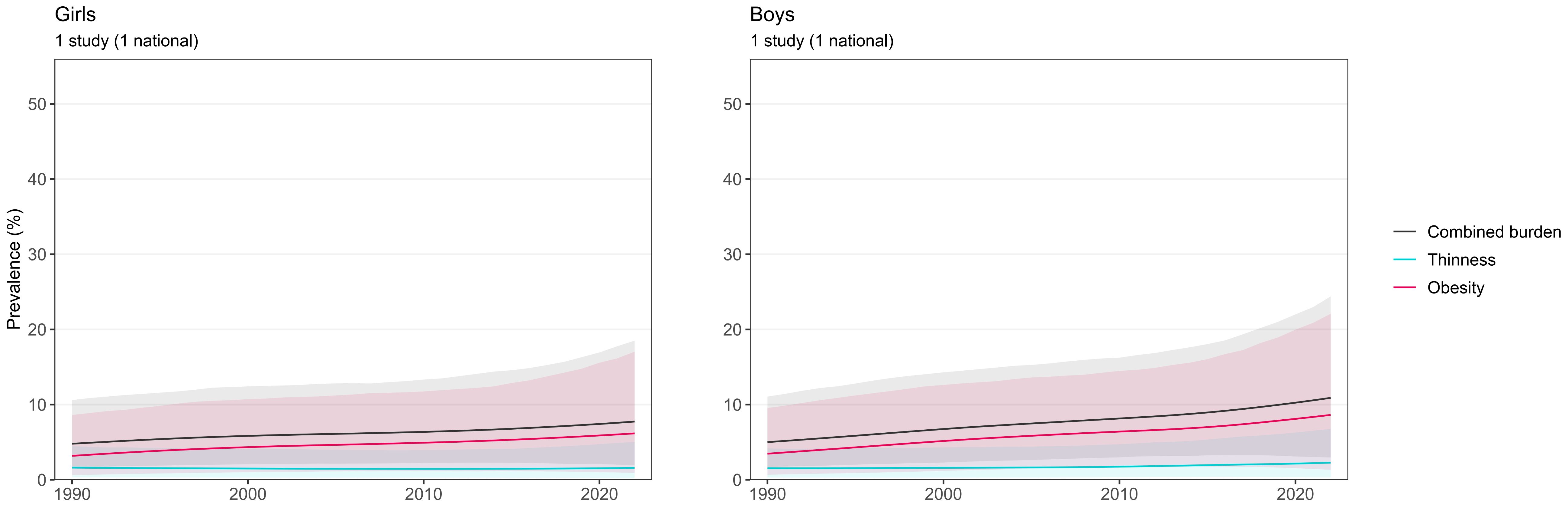


## Adults

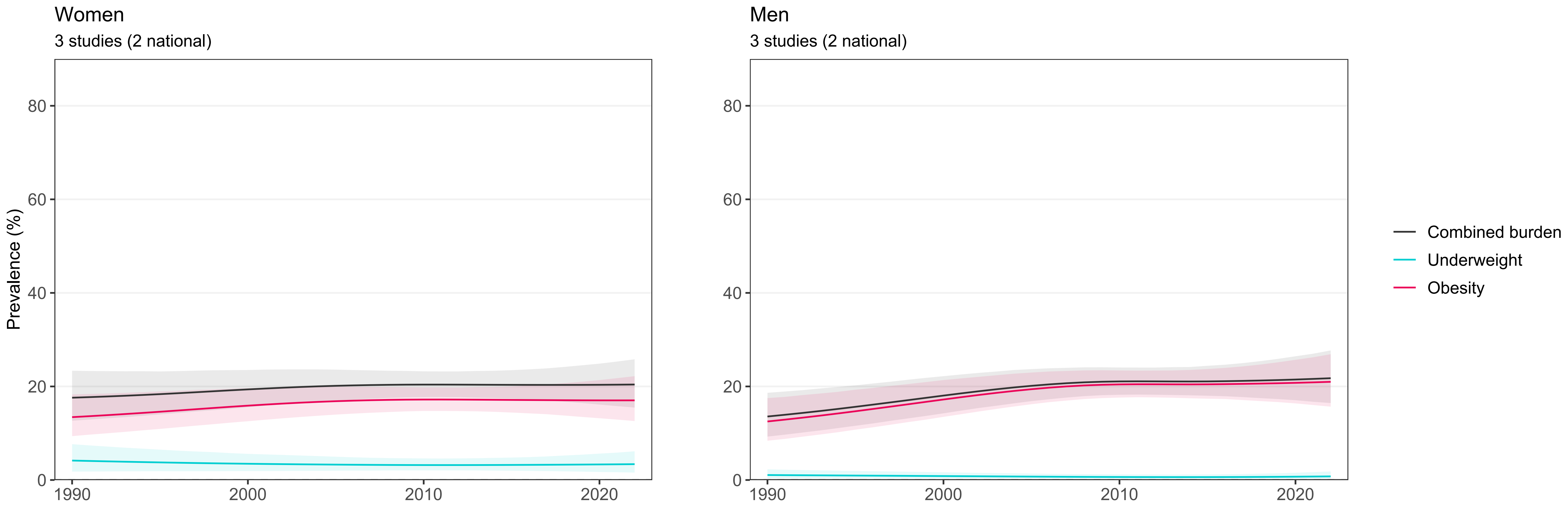

# Madagascar

## School-aged children and adolescents

### Girls

3 studies (3 national)

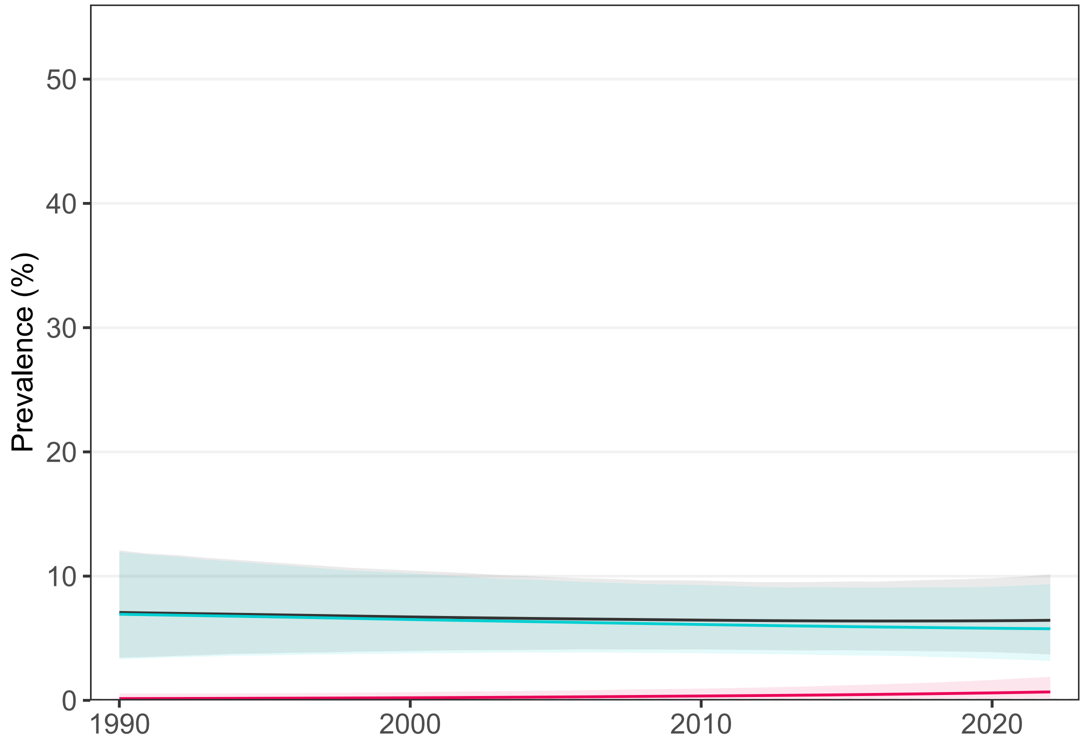


### Boys

No studies

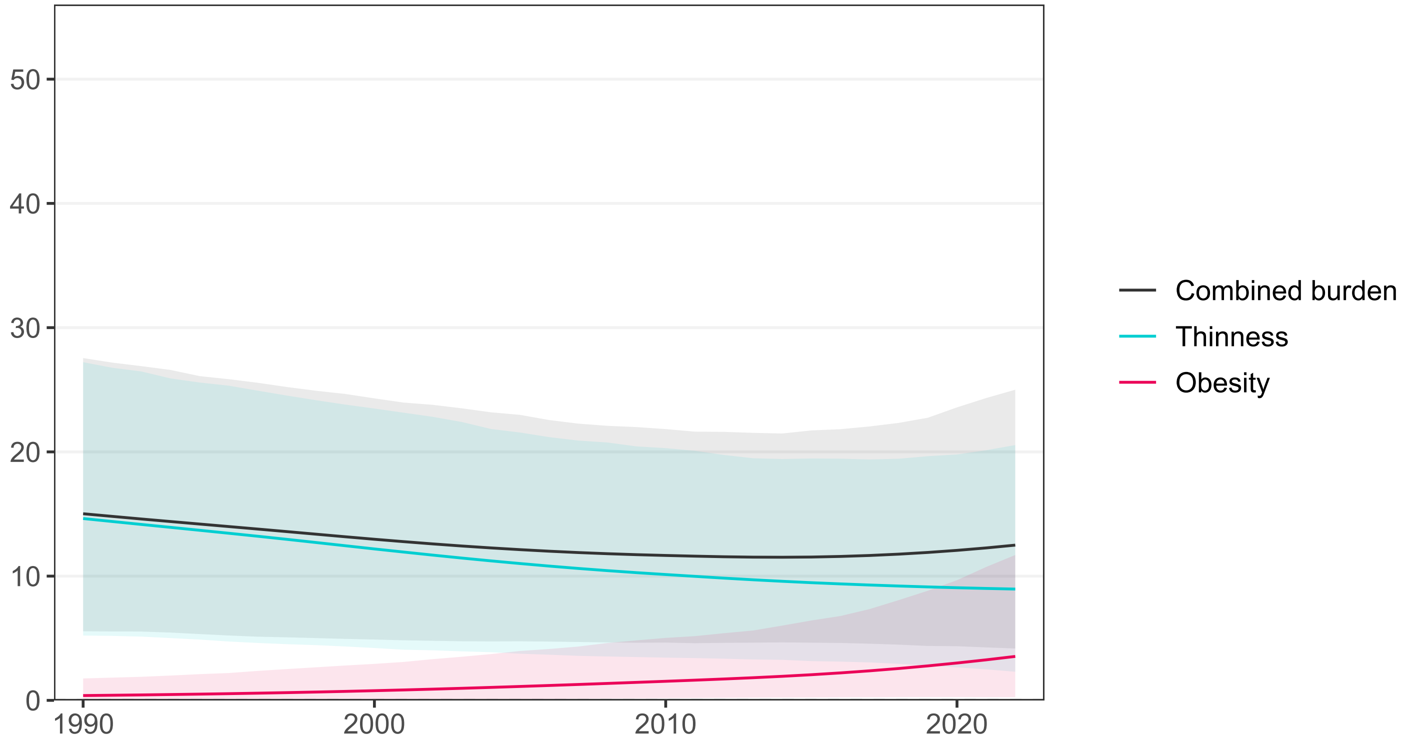


## Adults

### Women

6 studies (4 national)

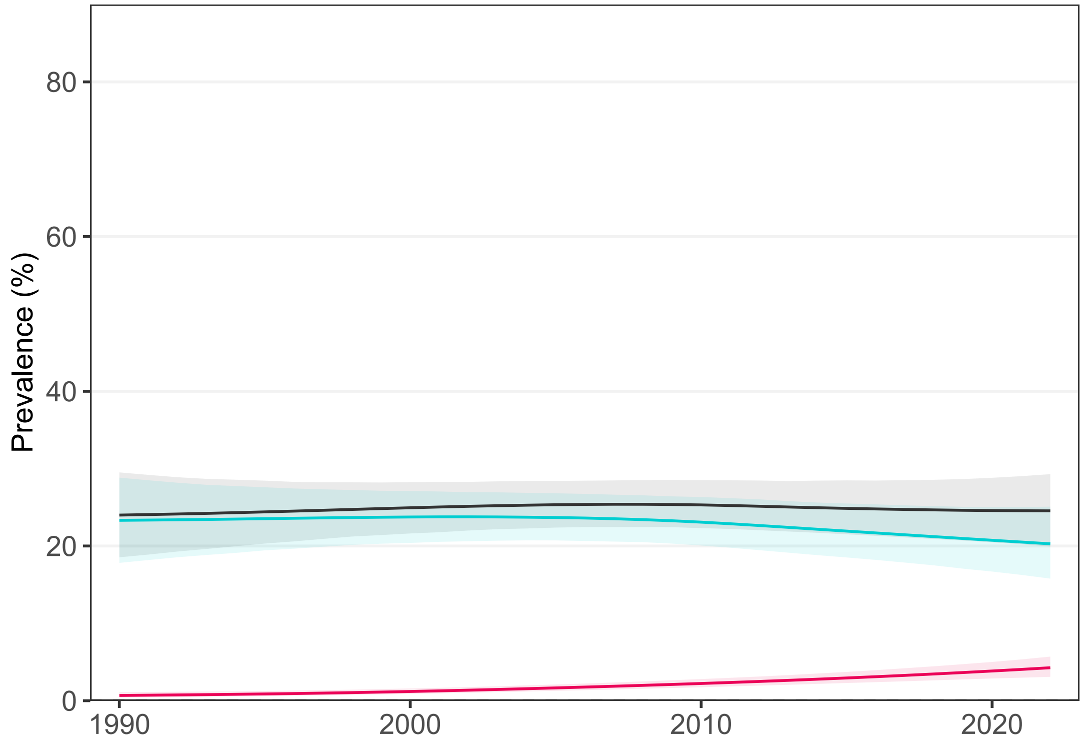


### Men

2 studies (0 national)

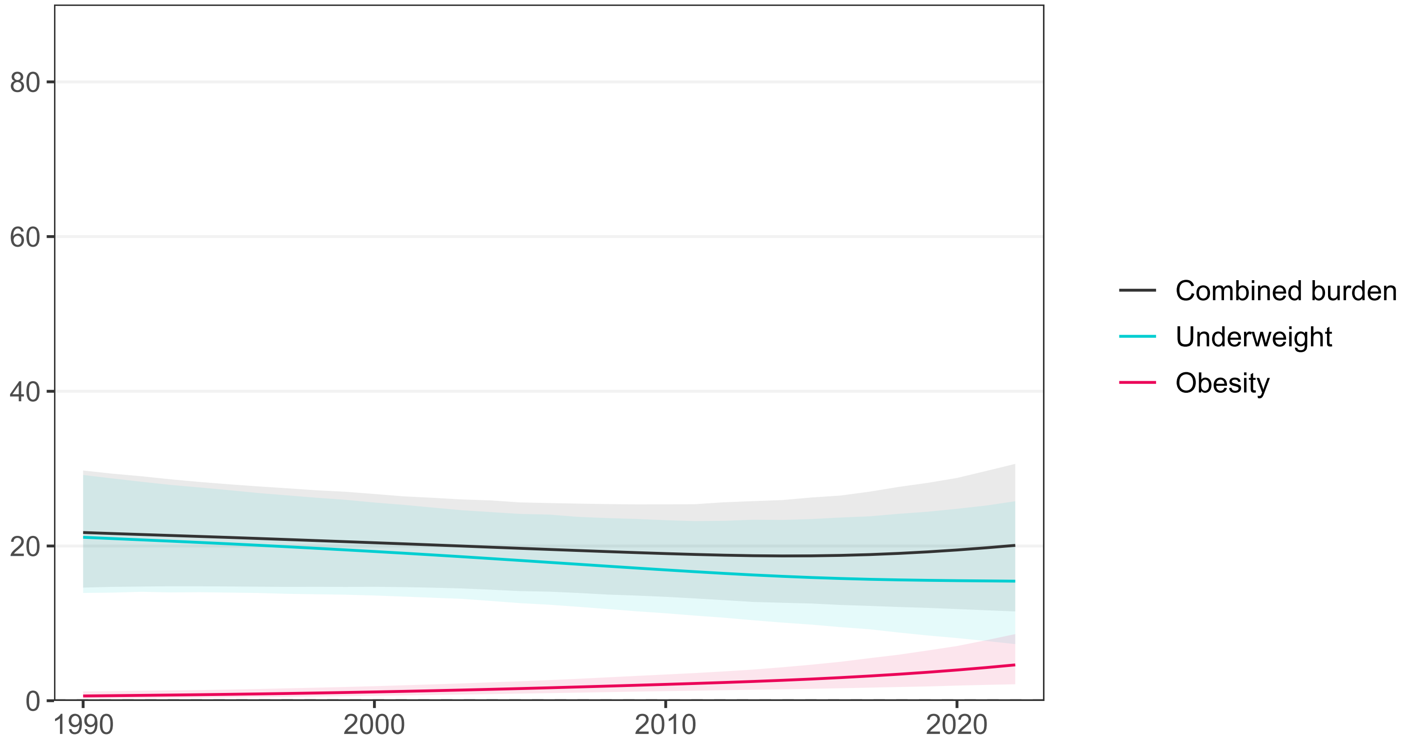

# Malawi

## School-aged children and adolescents

Girls
8 studies (6 national)

Boys
4 studies (2 national)

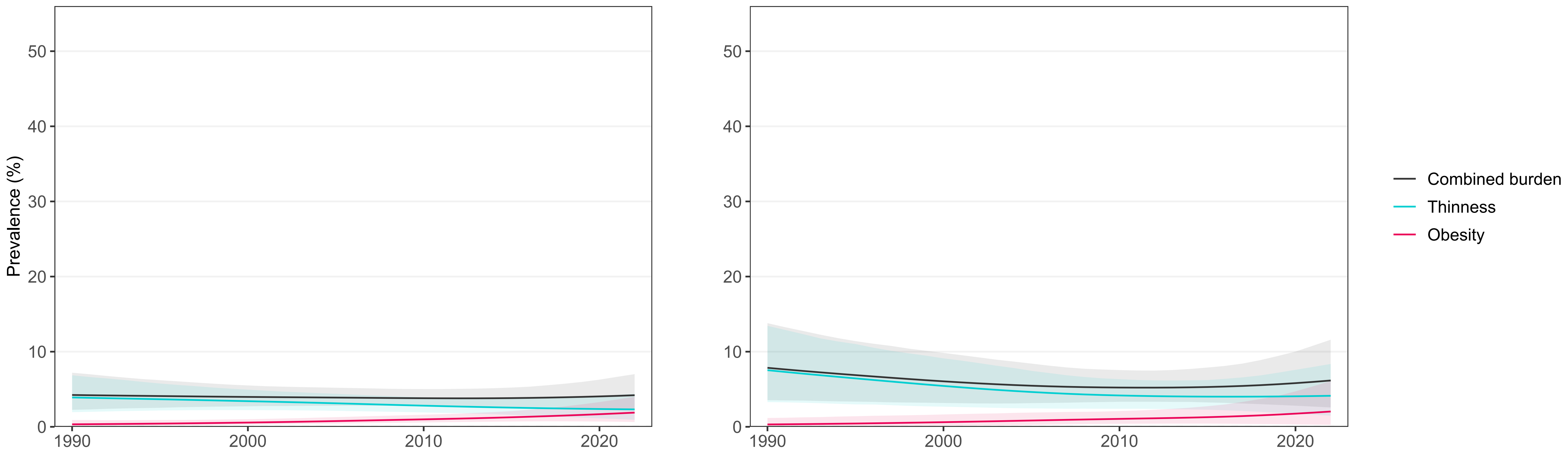


## Adults

Women
11 studies (8 national)

Men
5 studies (2 national)

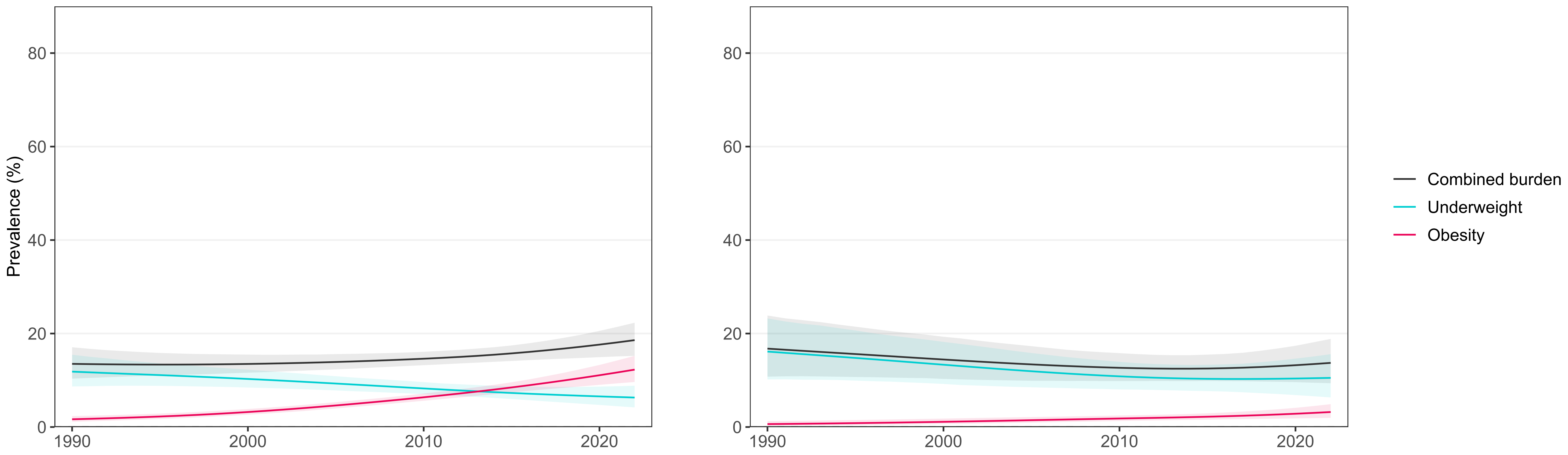

# Malaysia

## School-aged children and adolescents

### Girls

15 studies (13 national)

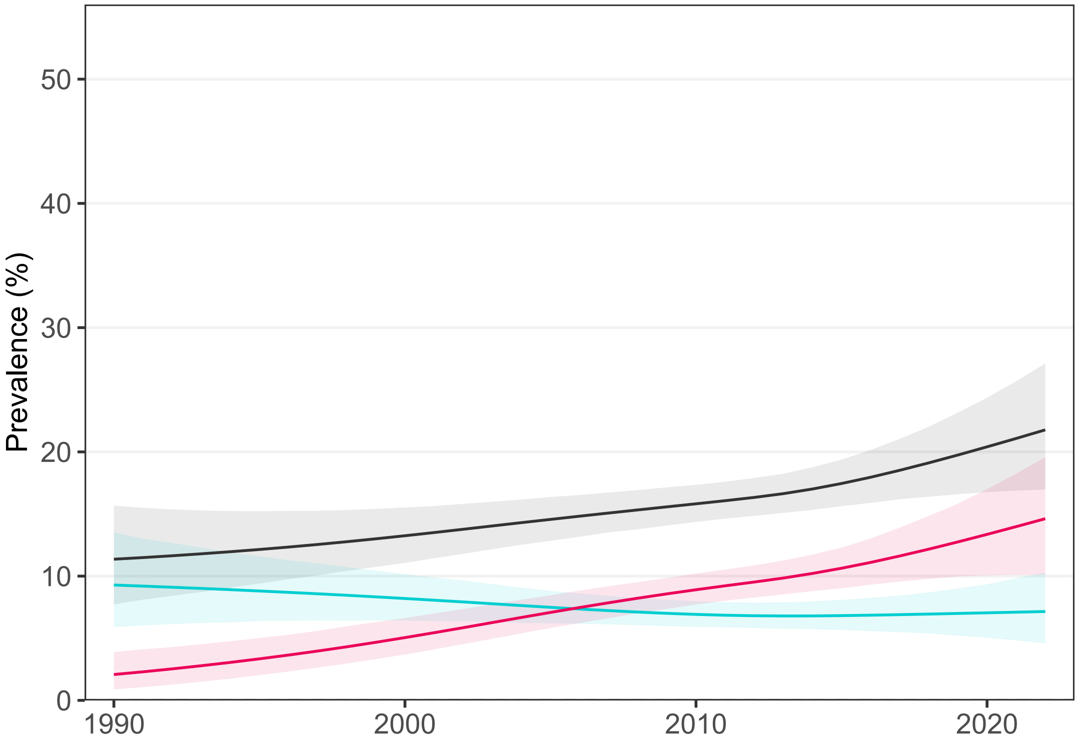


### Boys

15 studies (13 national)

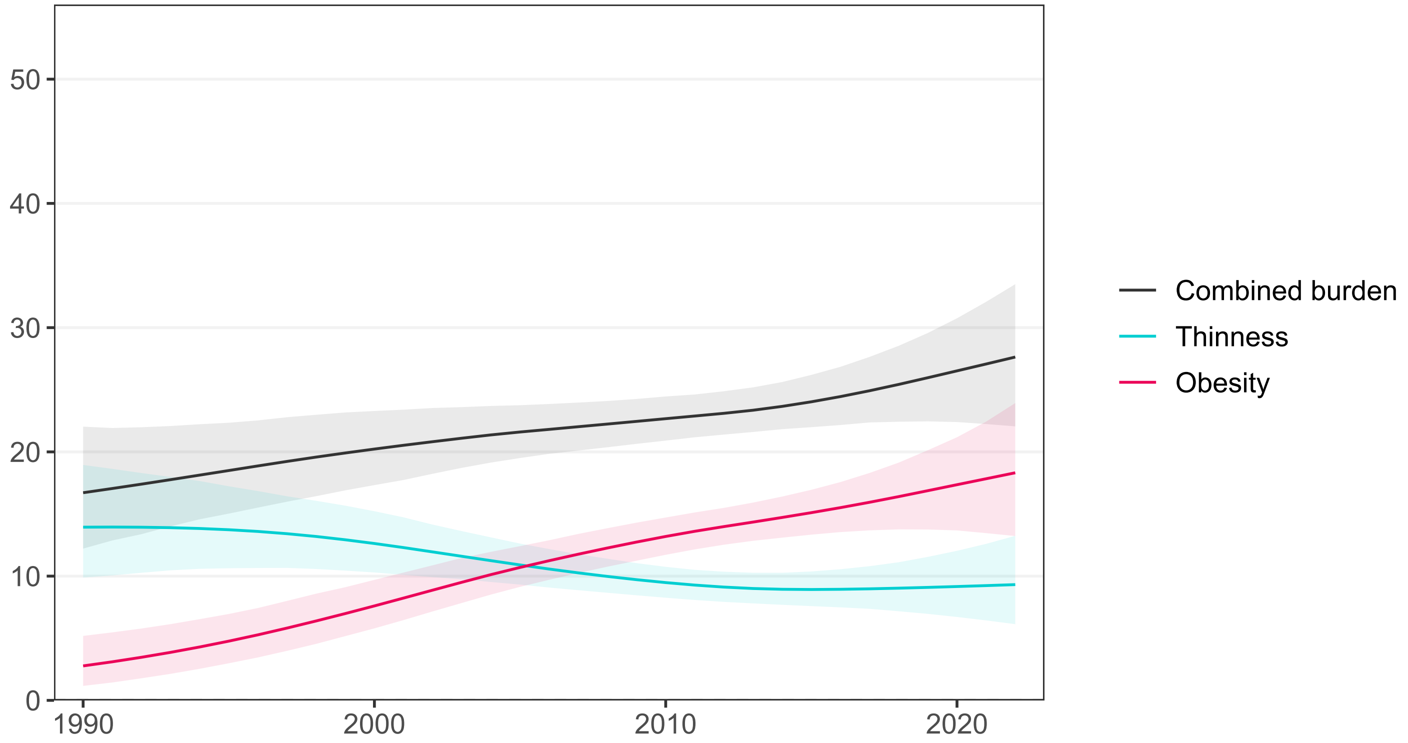


## Adults

### Women

11 studies (11 national)

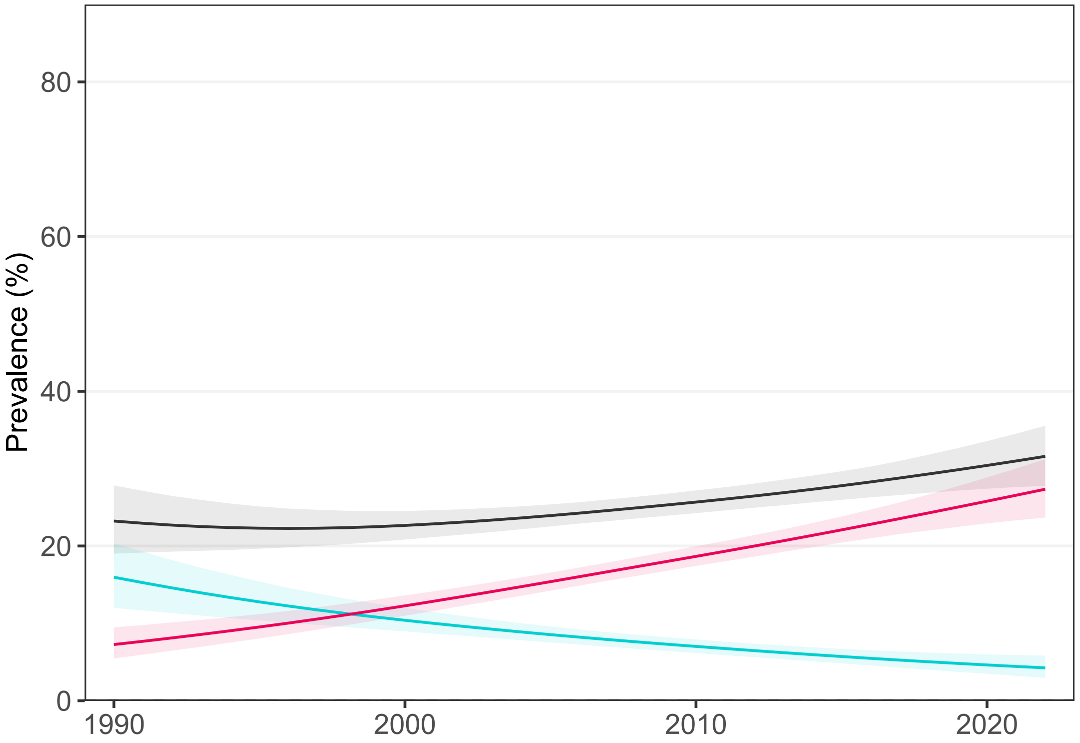


### Men

11 studies (11 national)

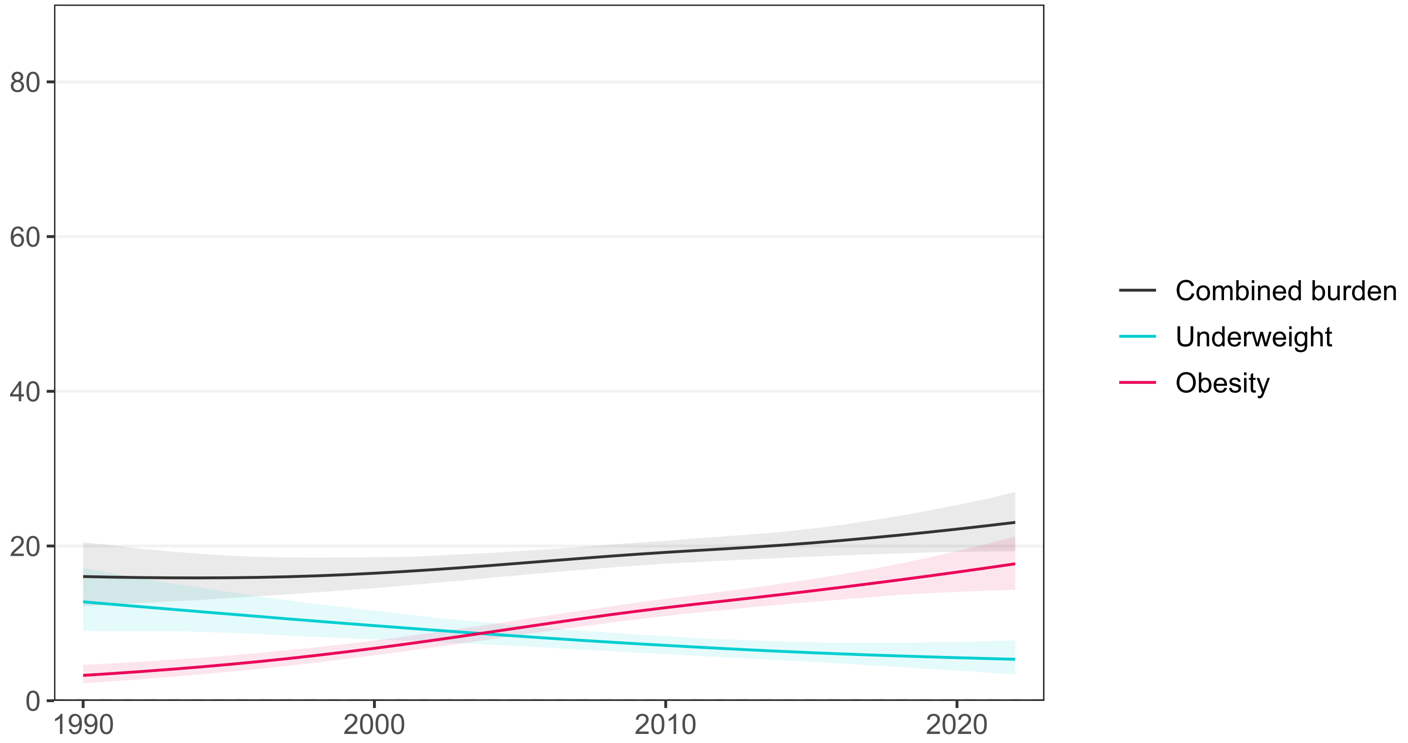

# Maldives

## School-aged children and adolescents

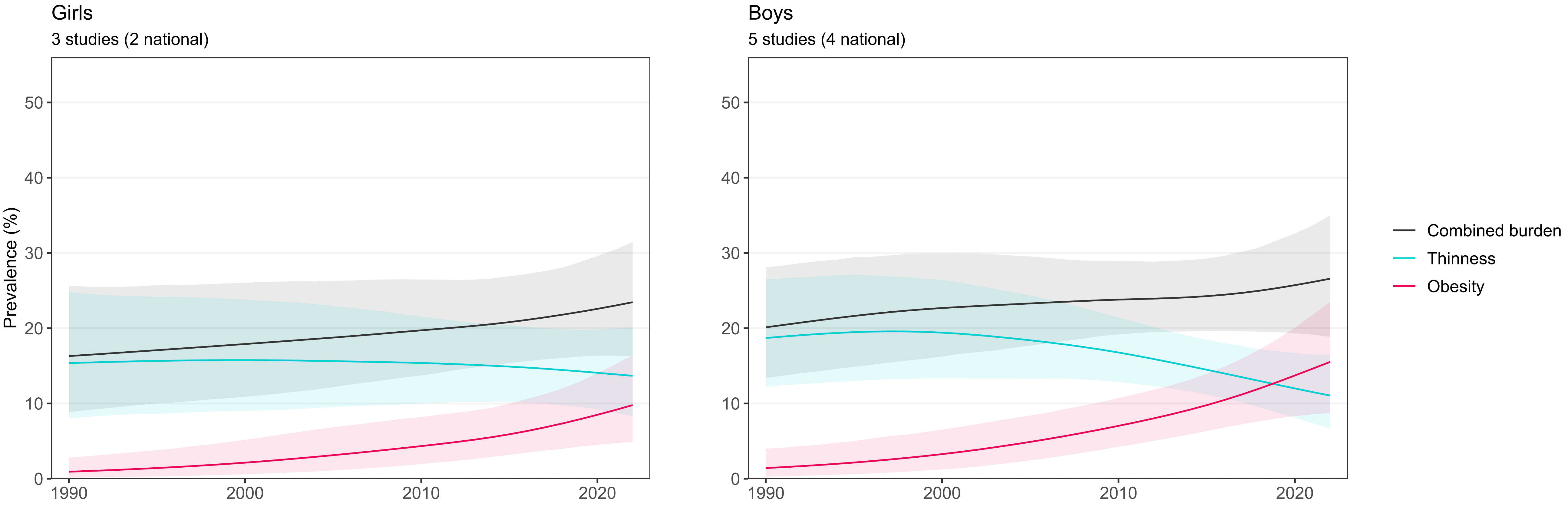


## Adults

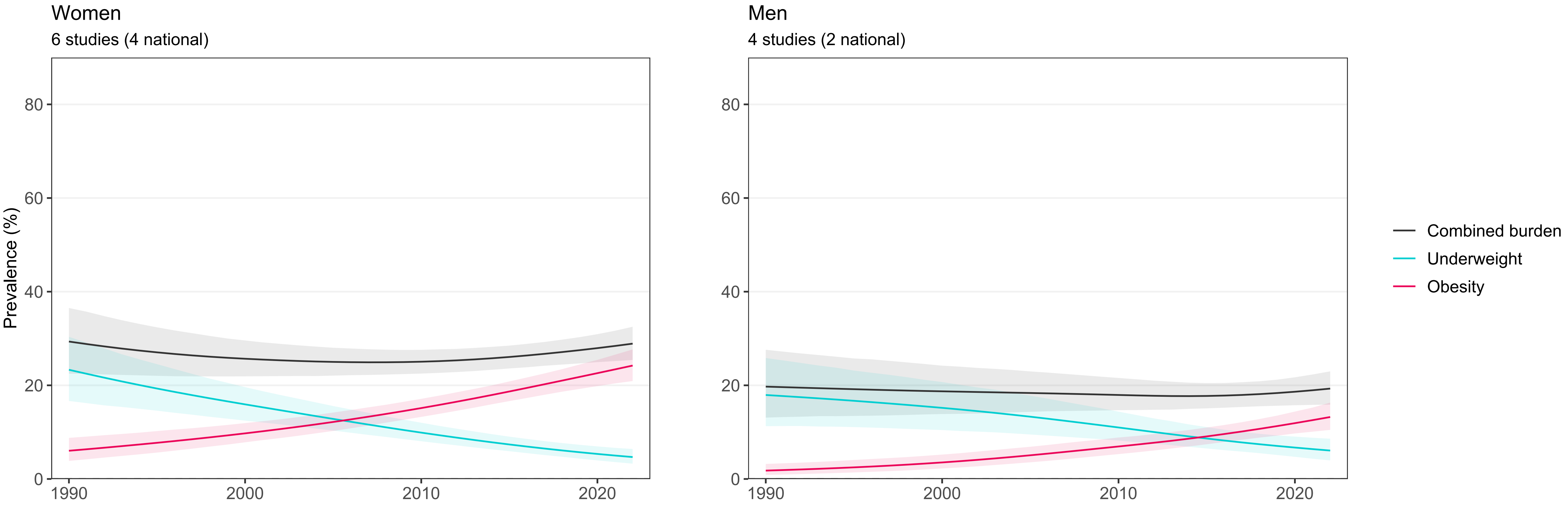

# Mali

## School-aged children and adolescents

Girls
8 studies (4 national)

Boys
3 studies (0 national)

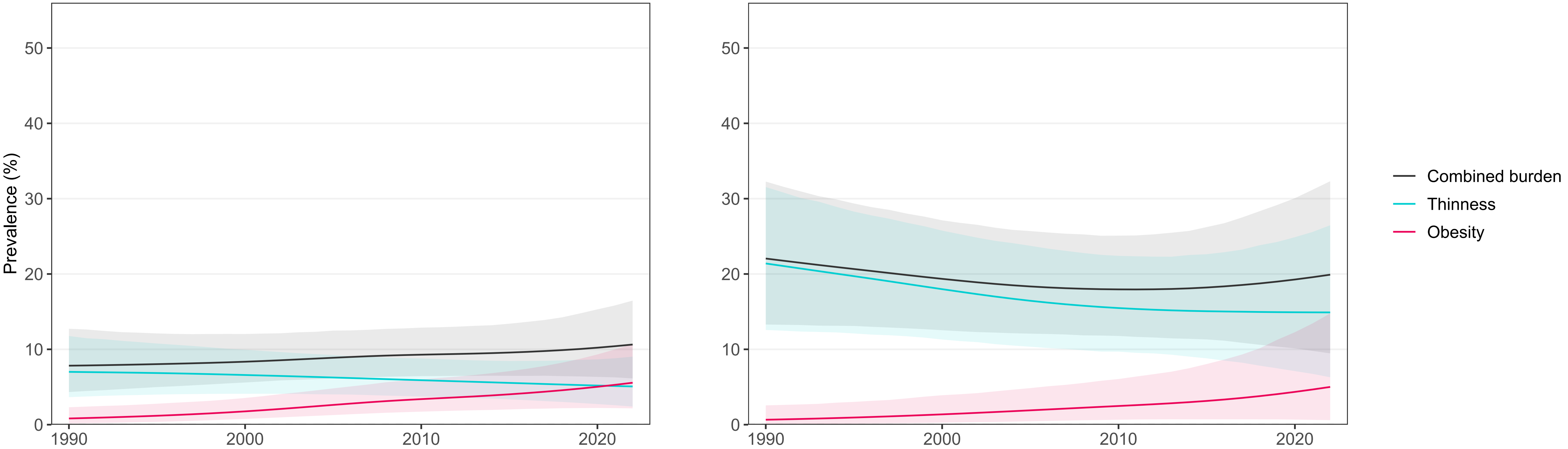


## Adults

Women
10 studies (5 national)

Men
3 studies (0 national)

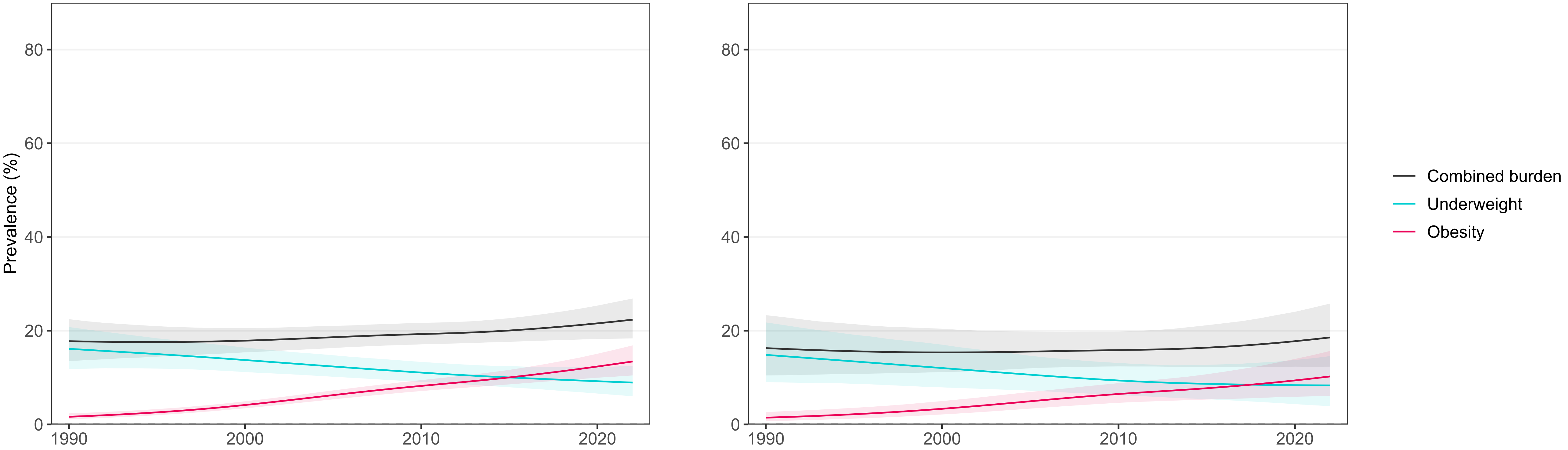

# Malta

## School-aged children and adolescents

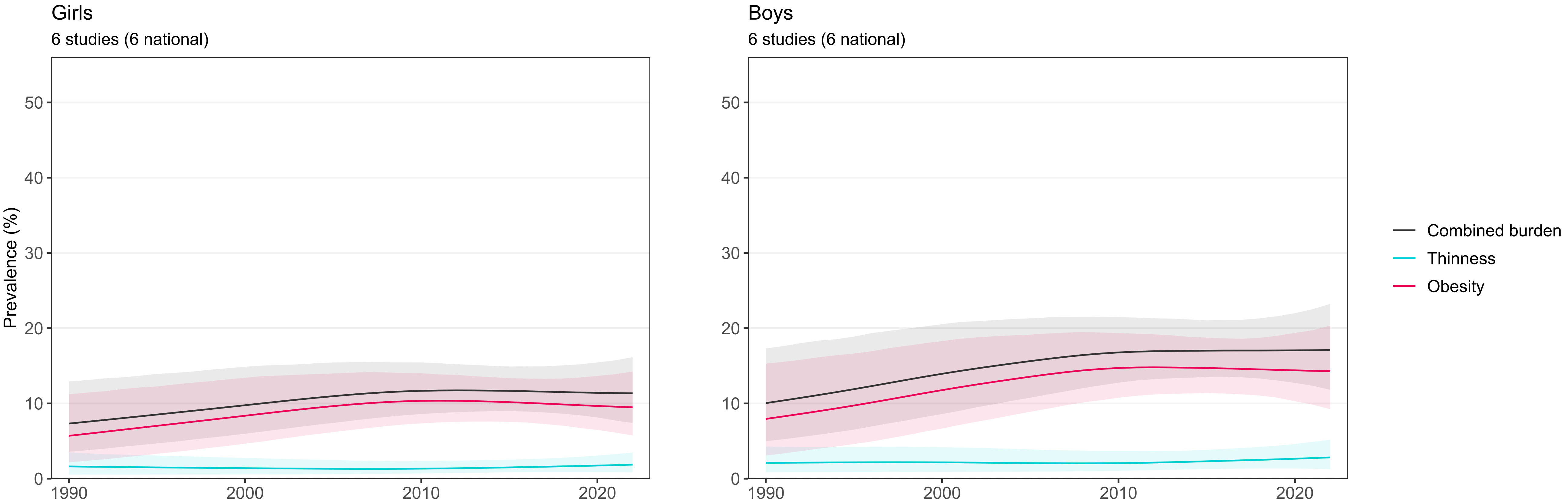


## Adults

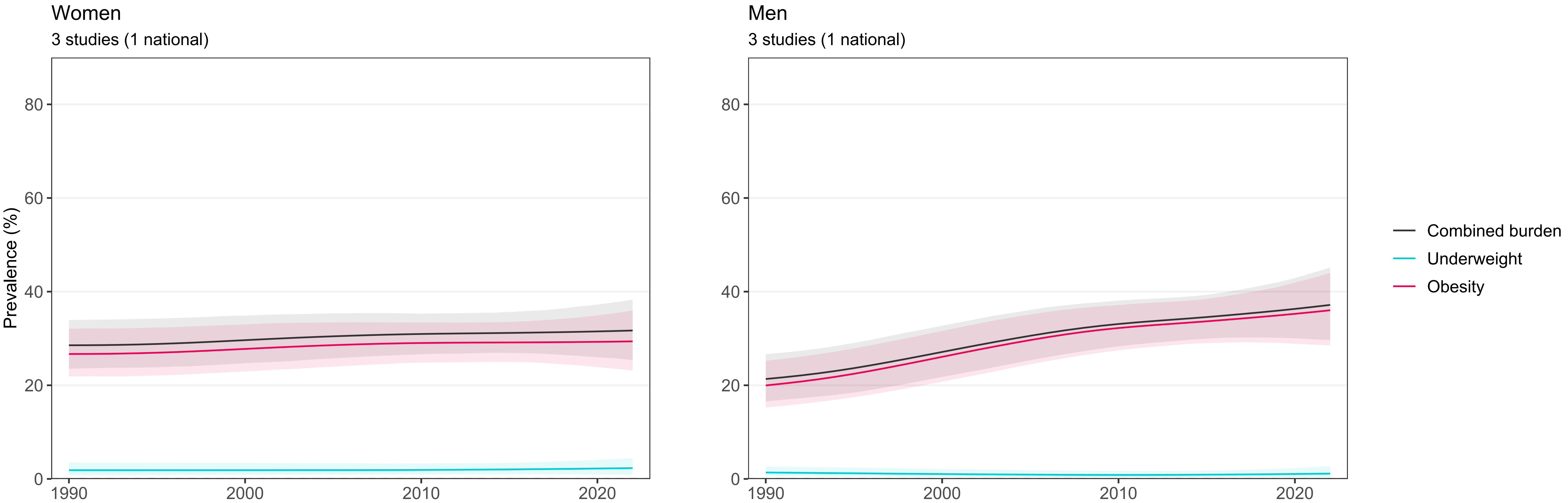

# Marshall Islands

## School-aged children and adolescents

### Girls

2 studies (2 national)

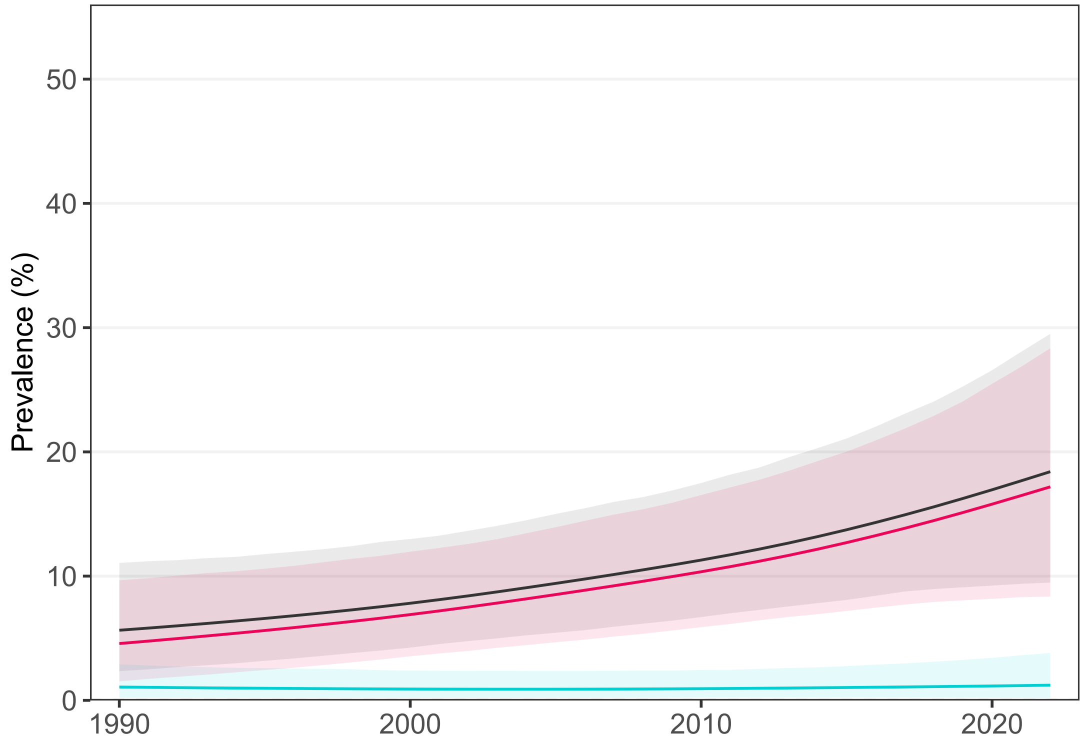


### Boys

2 studies (2 national)

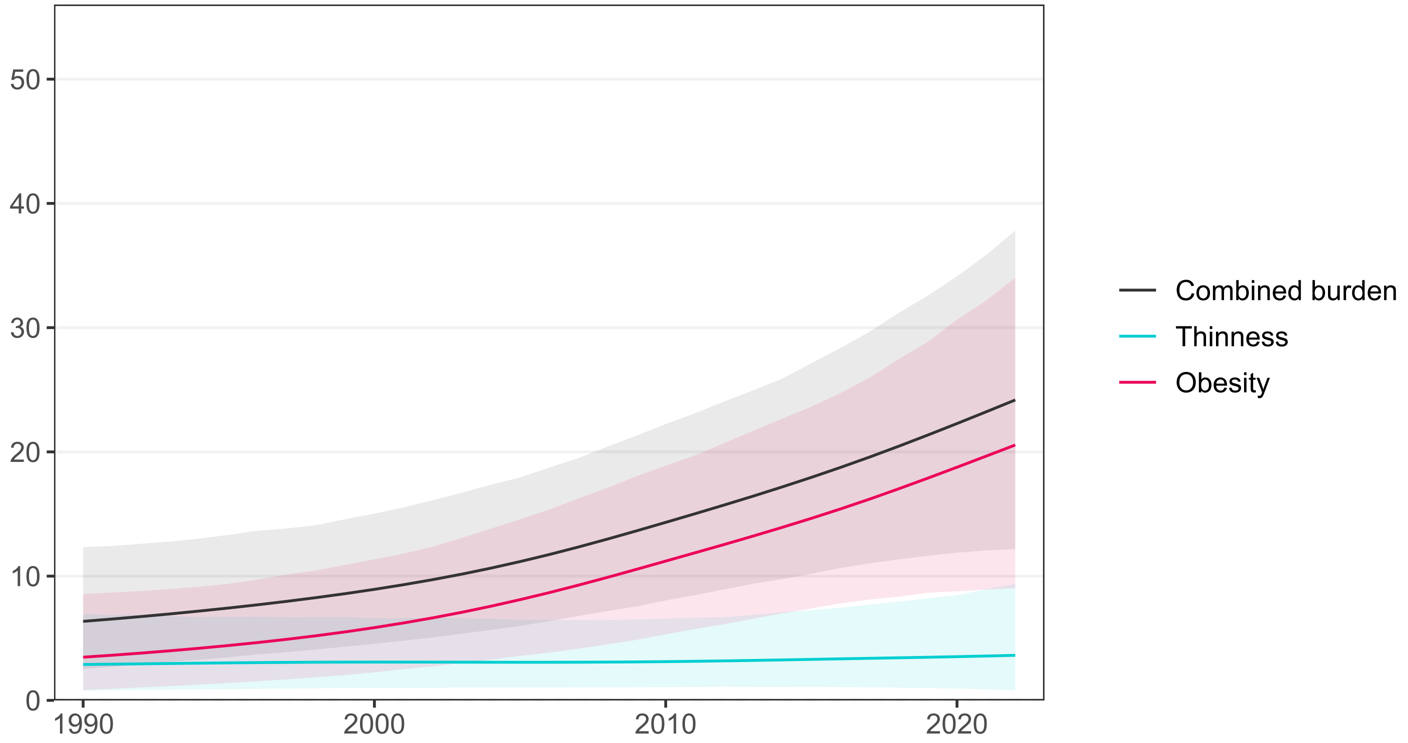


## Adults

### Women

2 studies (2 national)

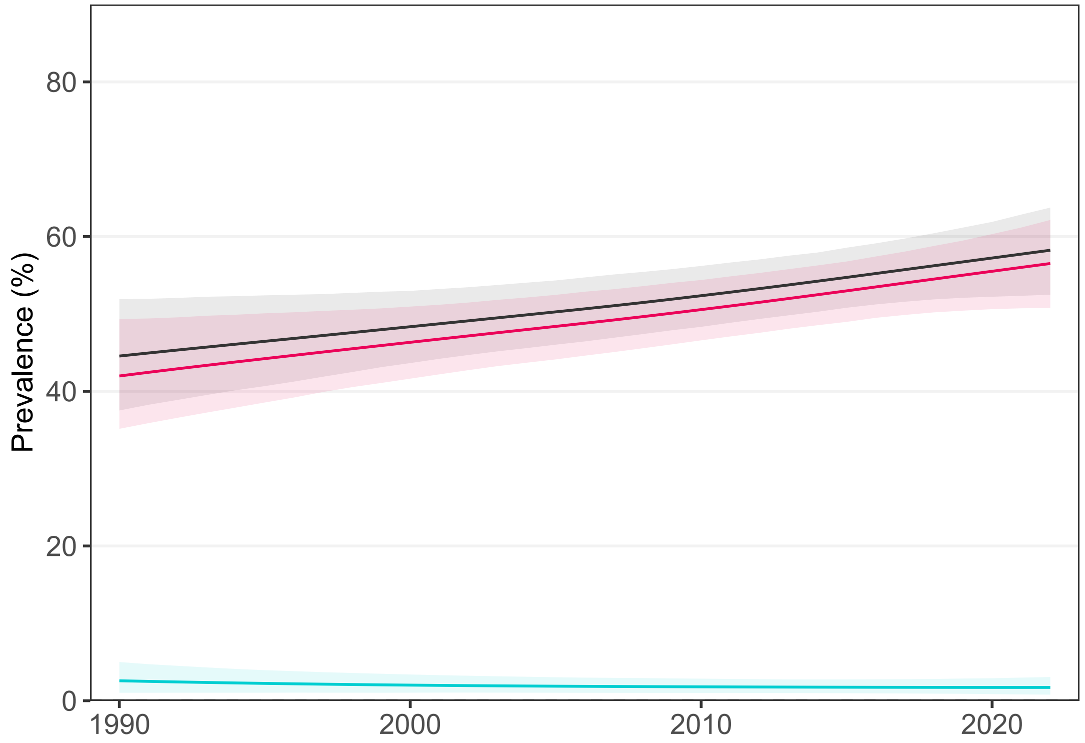


### Men

2 studies (2 national)

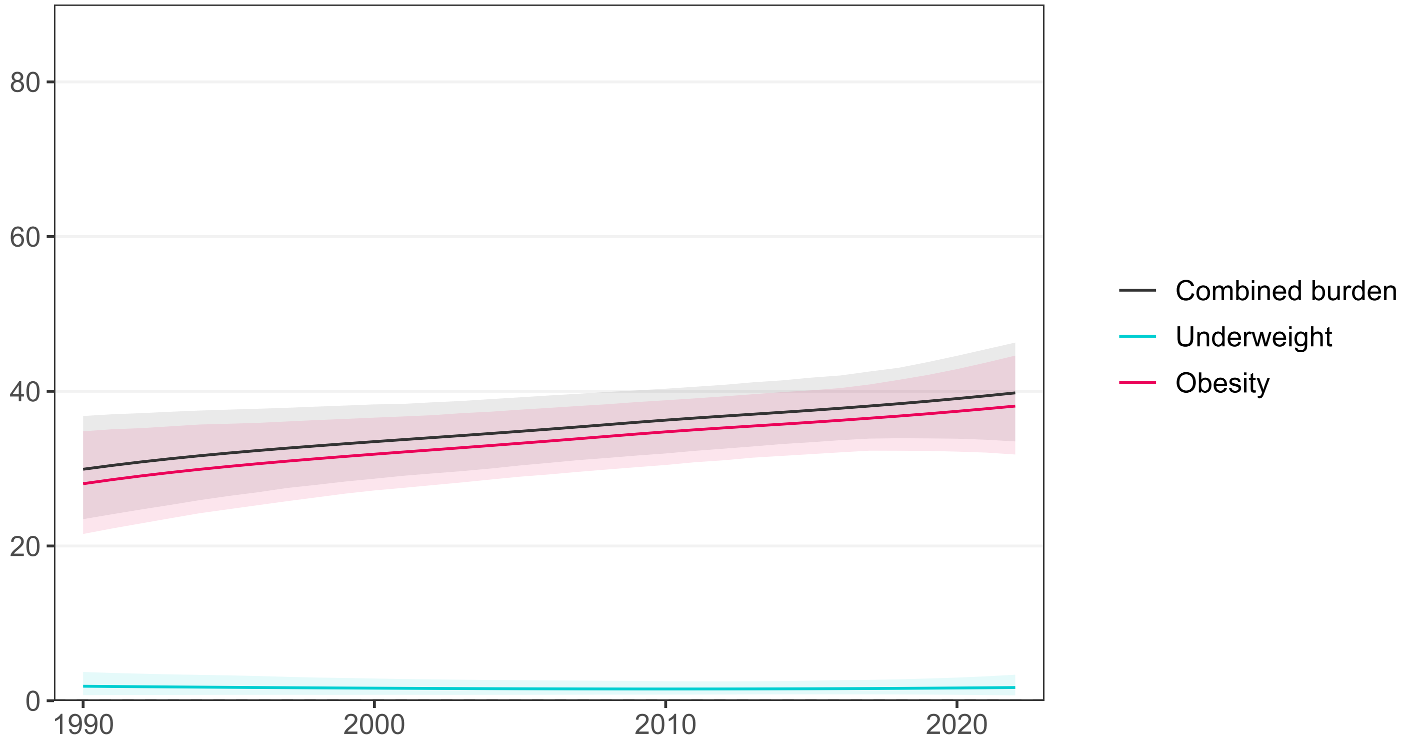

# Mauritania

## School-aged children and adolescents

### Girls

2 studies (1 national)

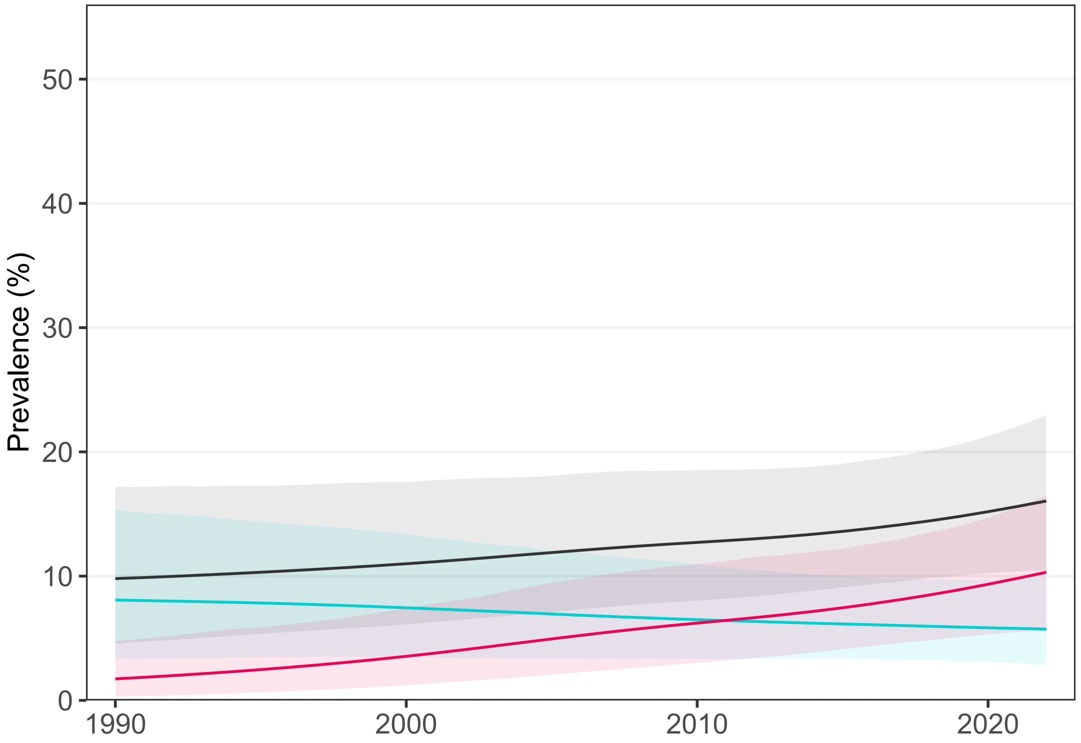


### Boys

1 study (0 national)

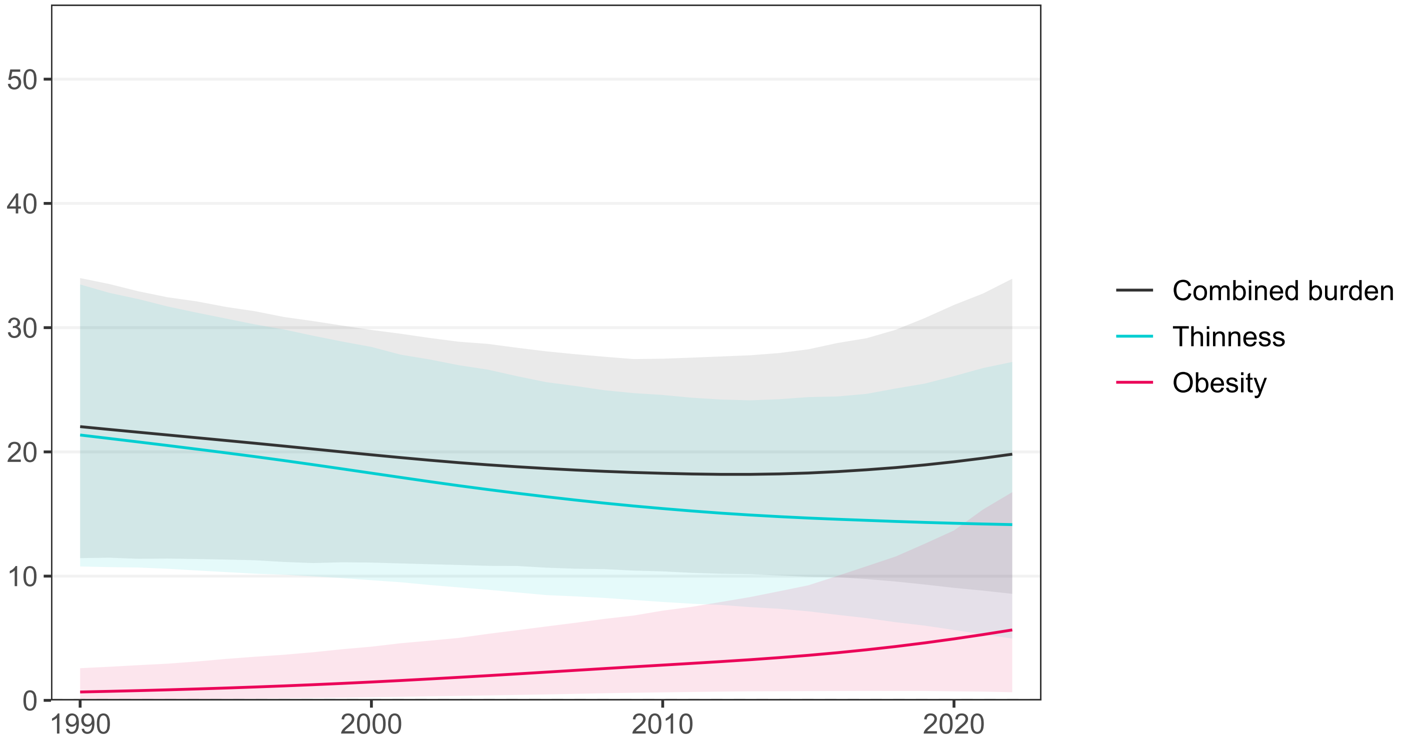


## Adults

### Women

3 studies (2 national)

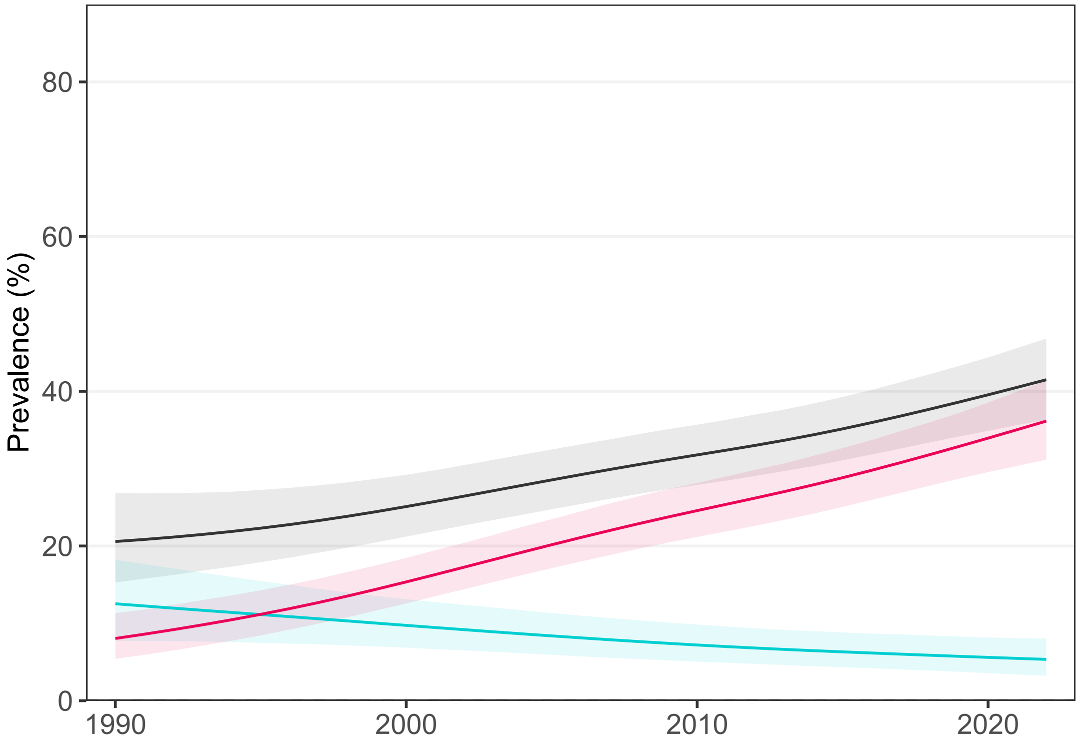


### Men

1 study (0 national)

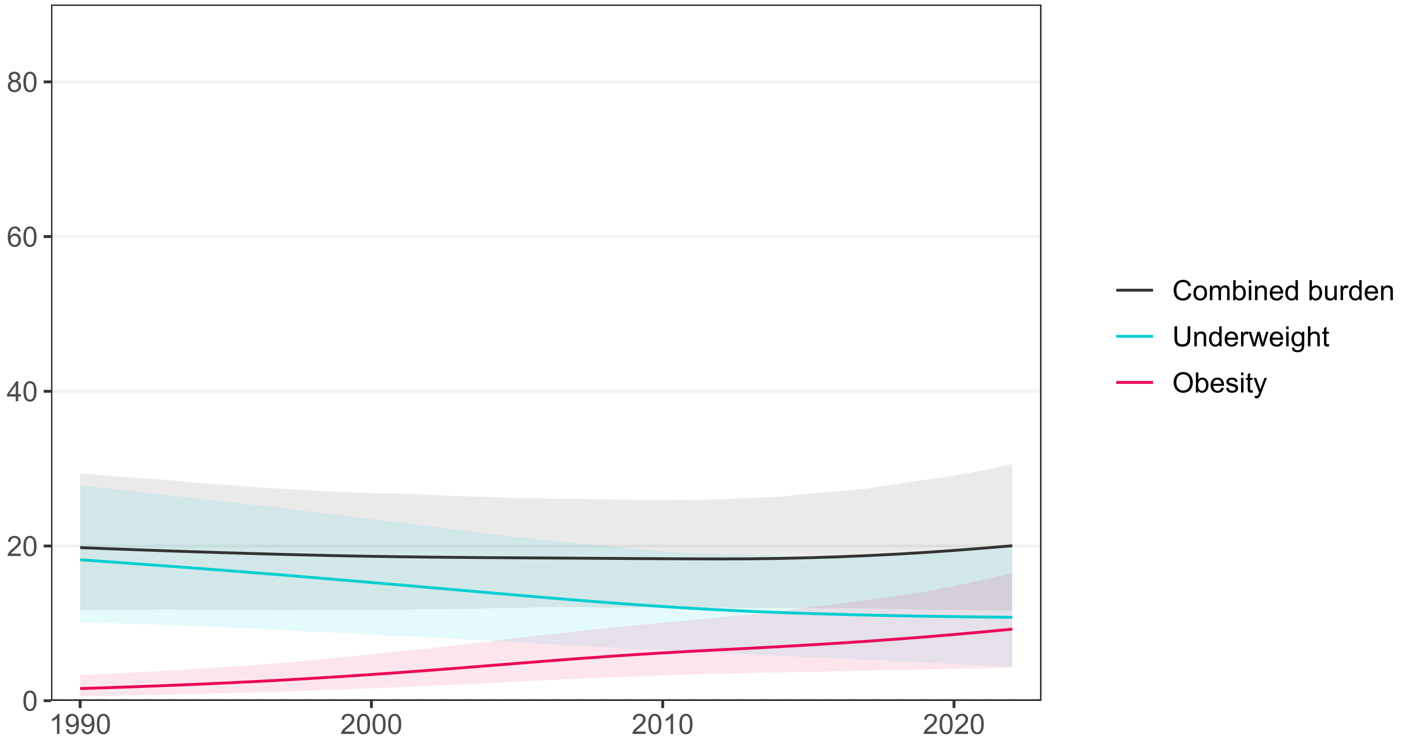

# Mauritius

## School-aged children and adolescents

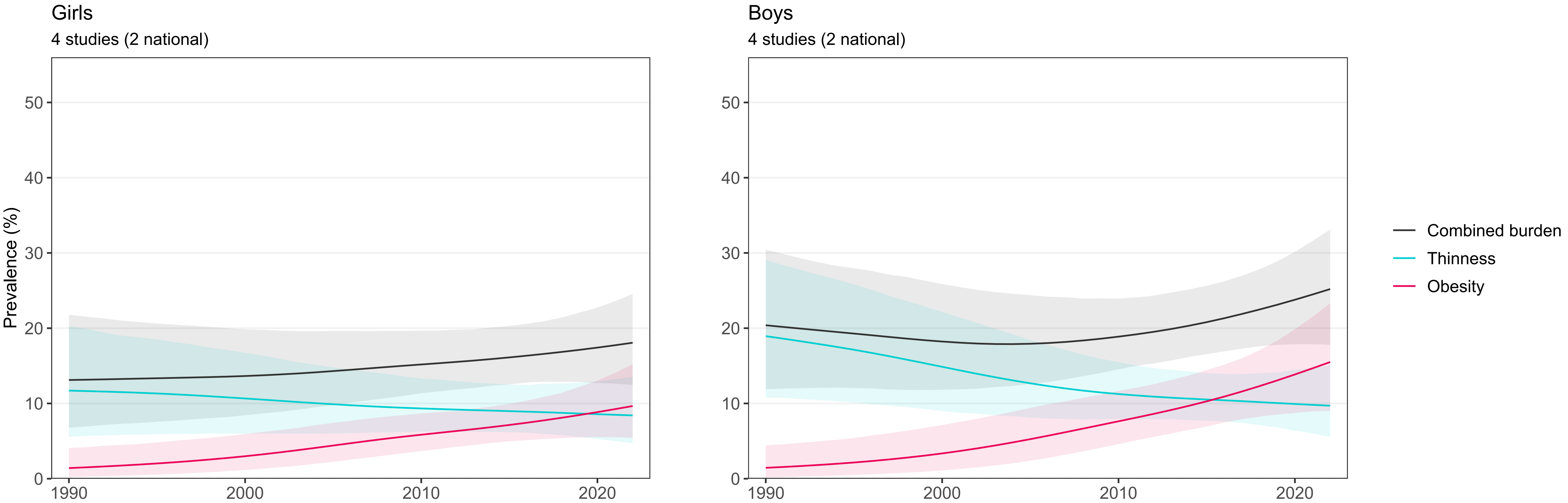


## Adults

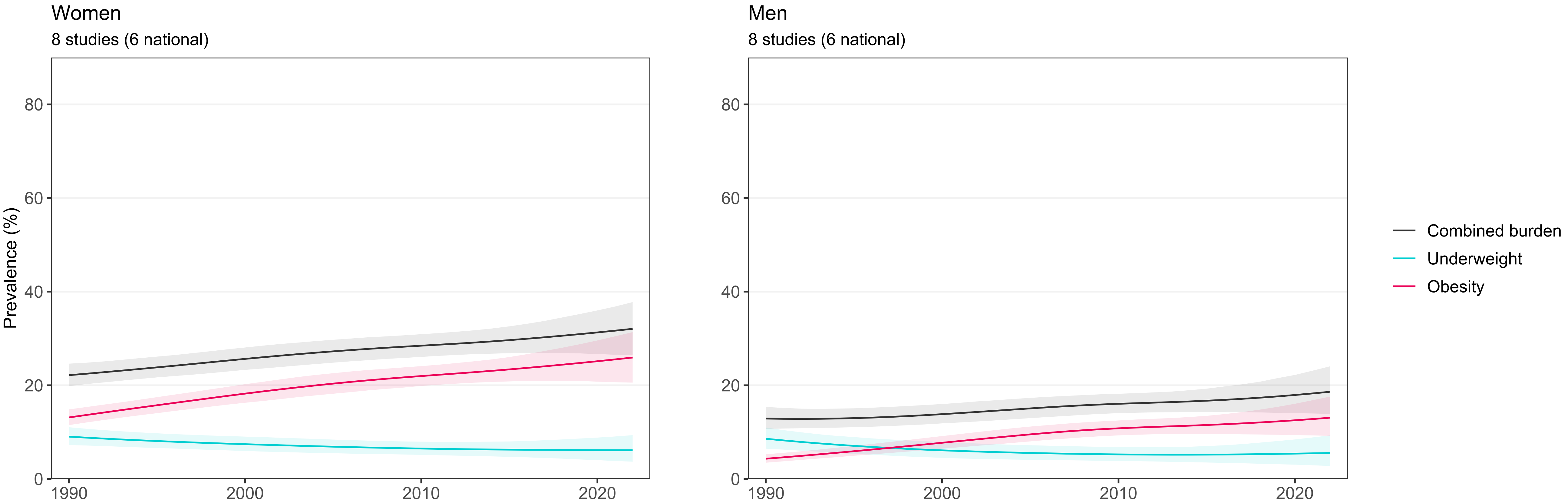

# Mexico

## School-aged children and adolescents

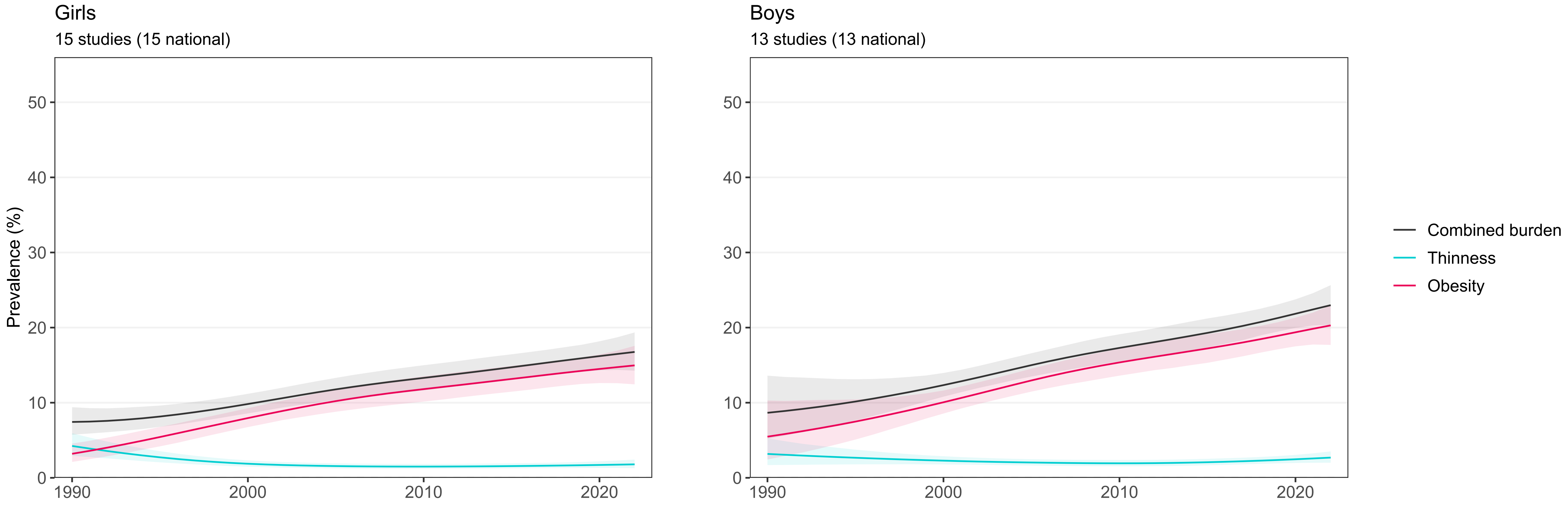


## Adults

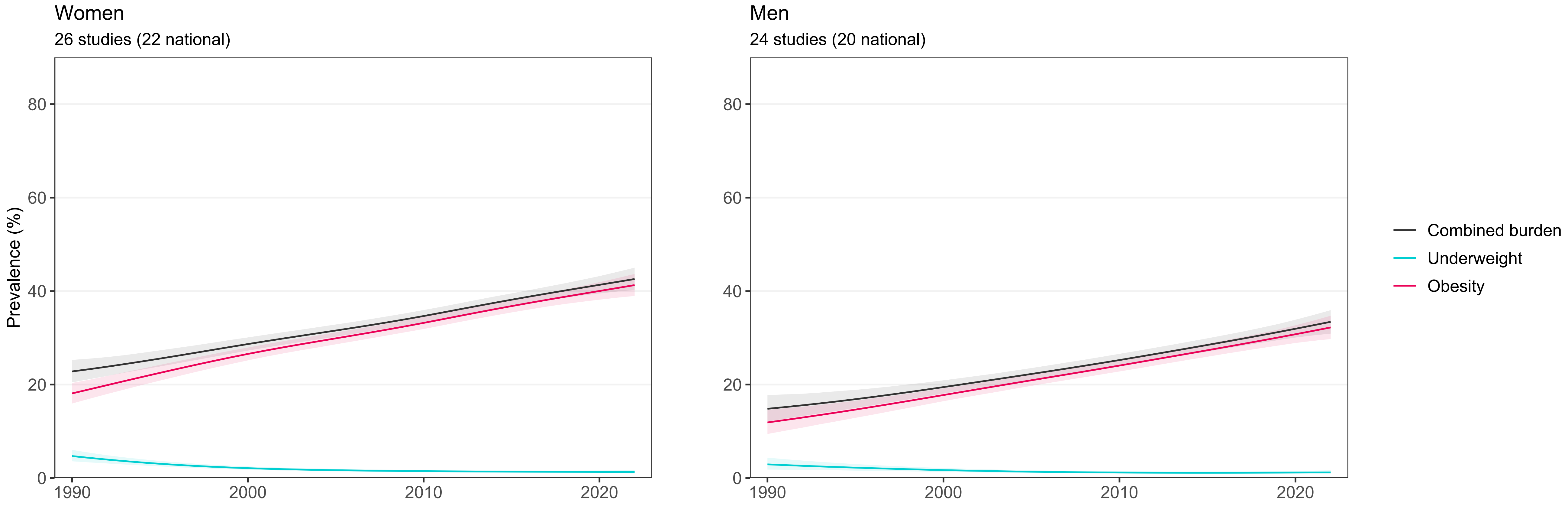

# Micronesia

## School-aged children and adolescents

### Girls
3 studies (0 national)

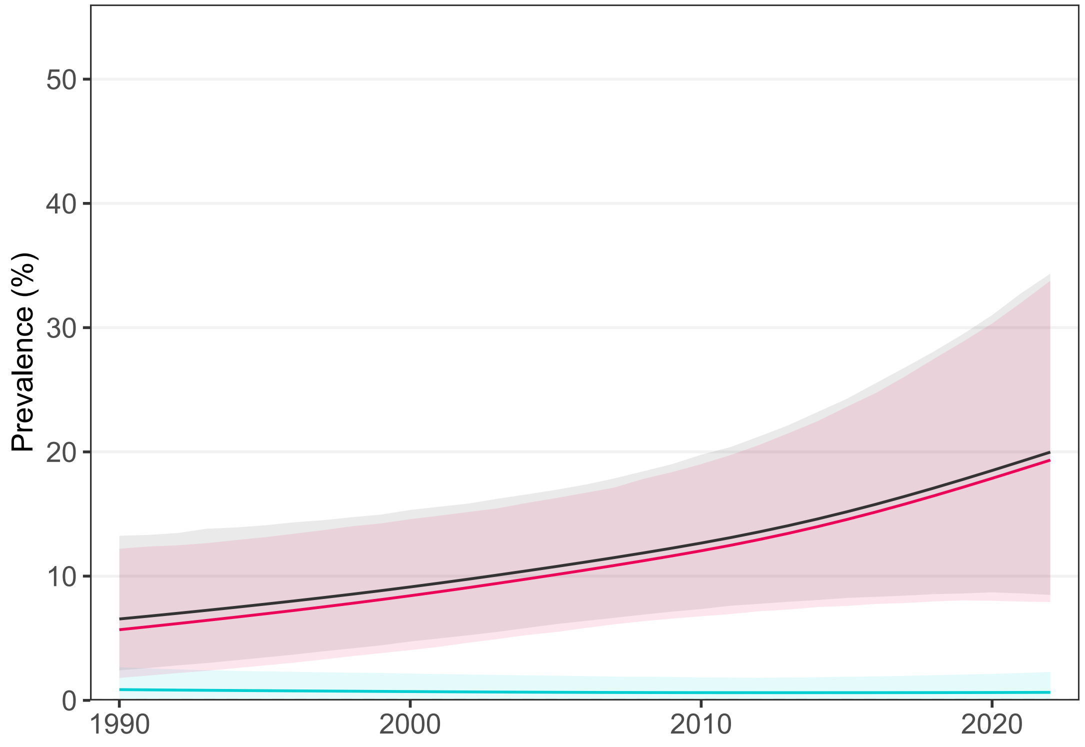


### Boys
3 studies (0 national)

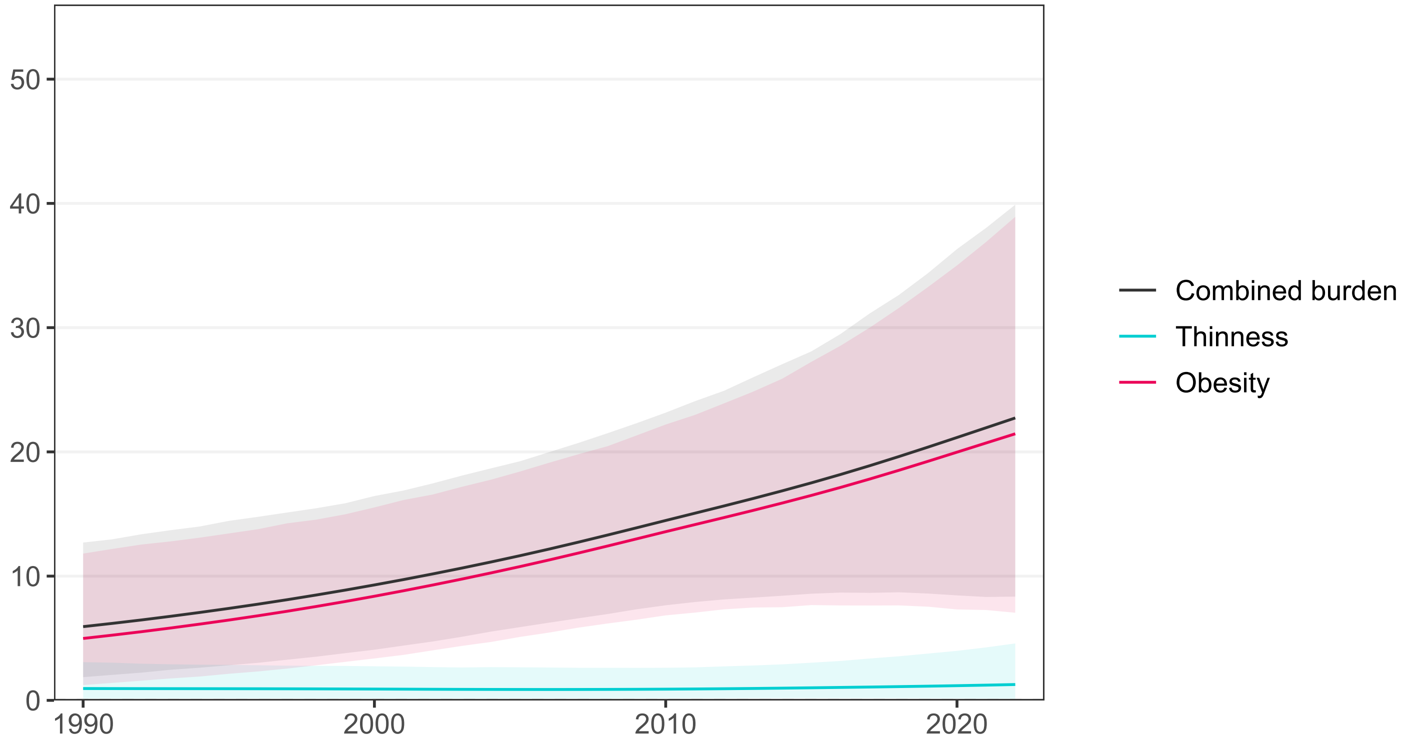


## Adults

### Women
6 studies (0 national)

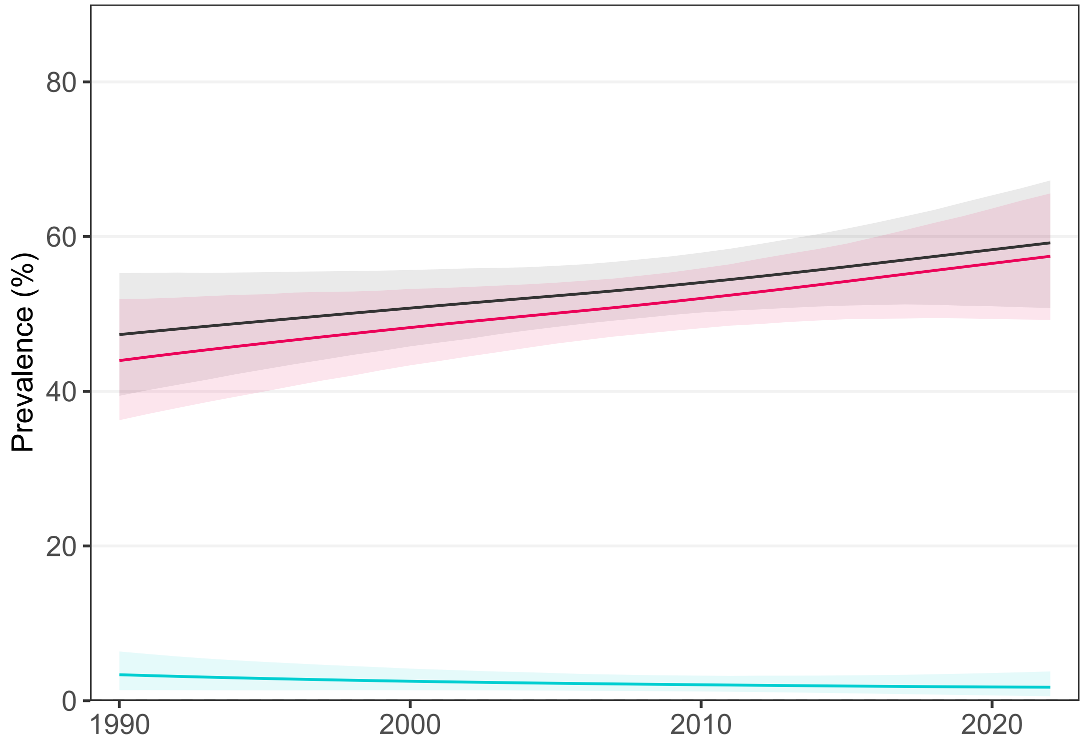


### Men
6 studies (0 national)

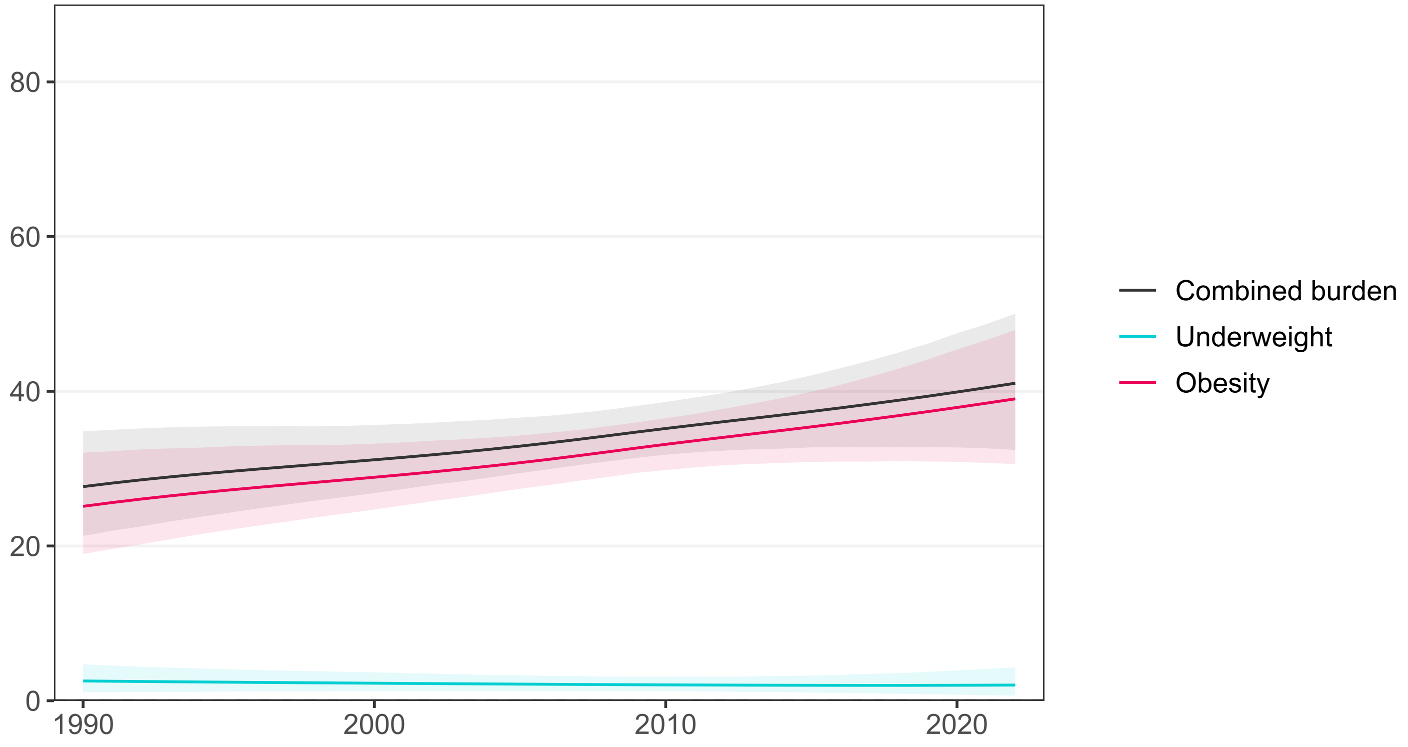

# Moldova

## School-aged children and adolescents

### Girls

4 studies (4 national)

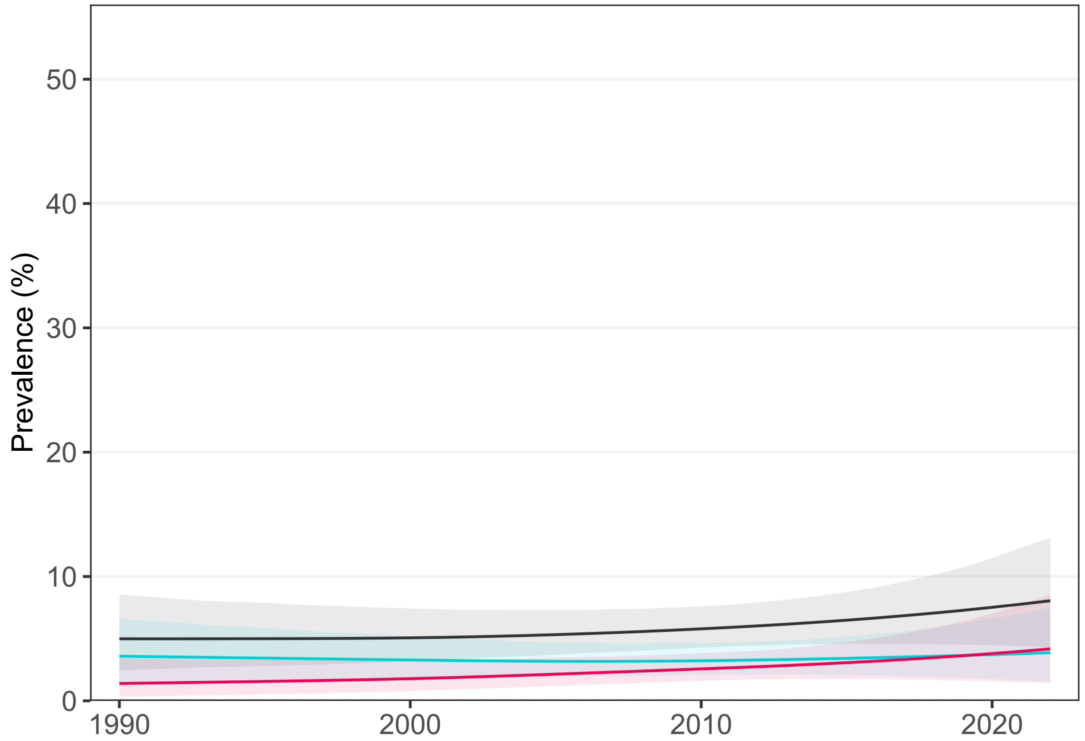


### Boys

3 studies (3 national)

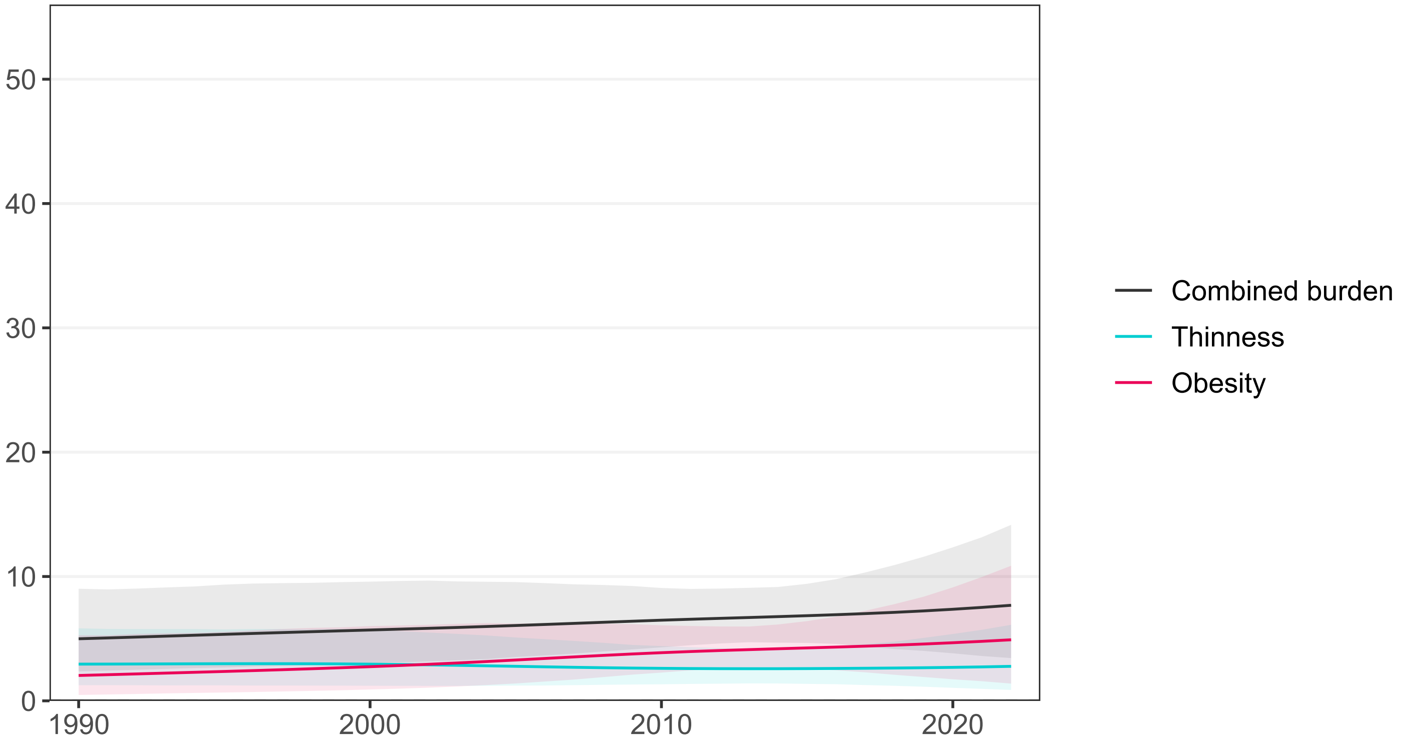


## Adults

### Women

3 studies (3 national)

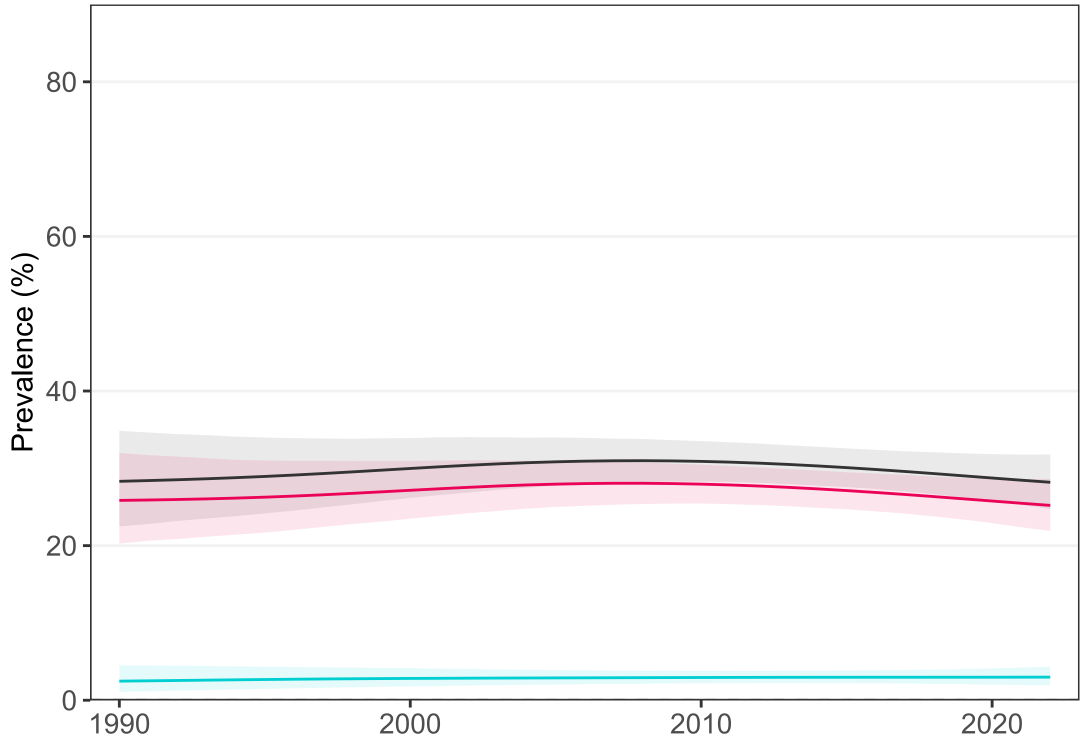


### Men

2 studies (2 national)

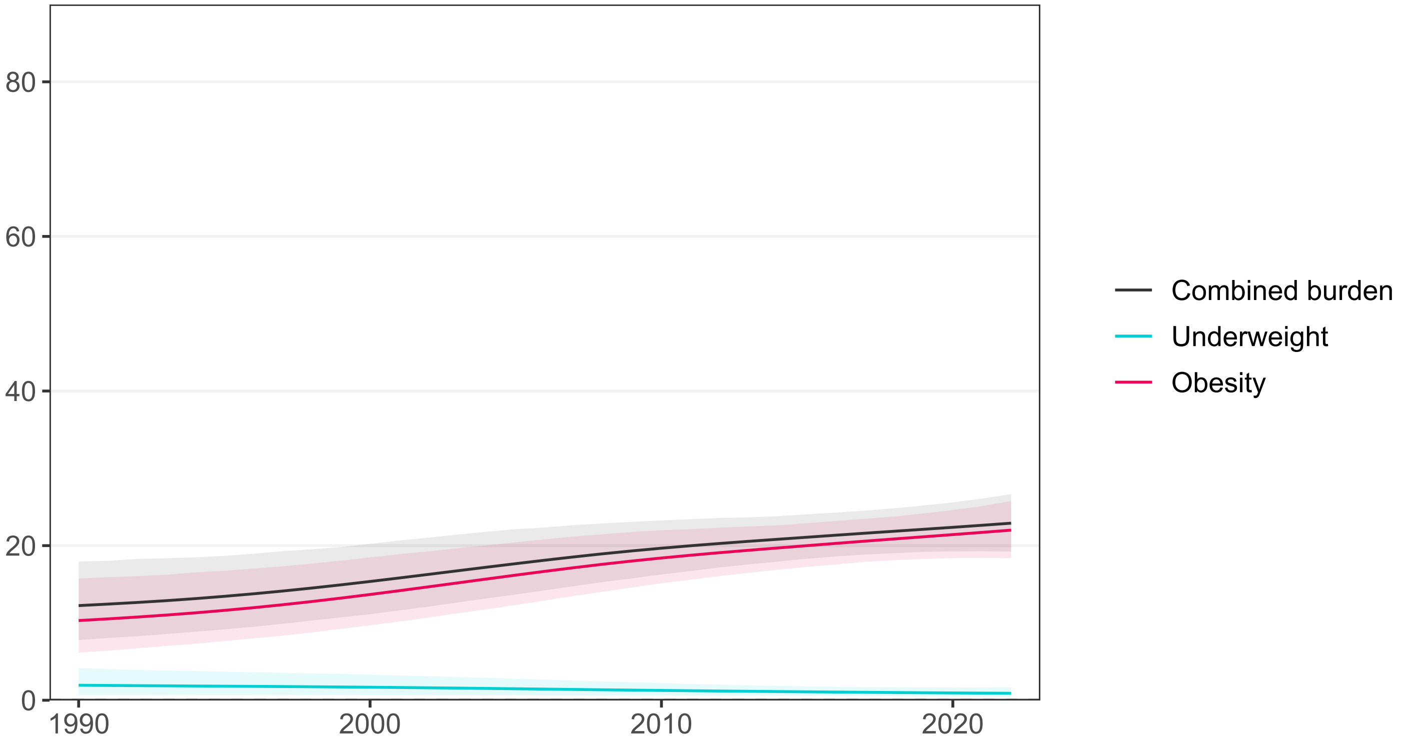

# Mongolia

## School-aged children and adolescents

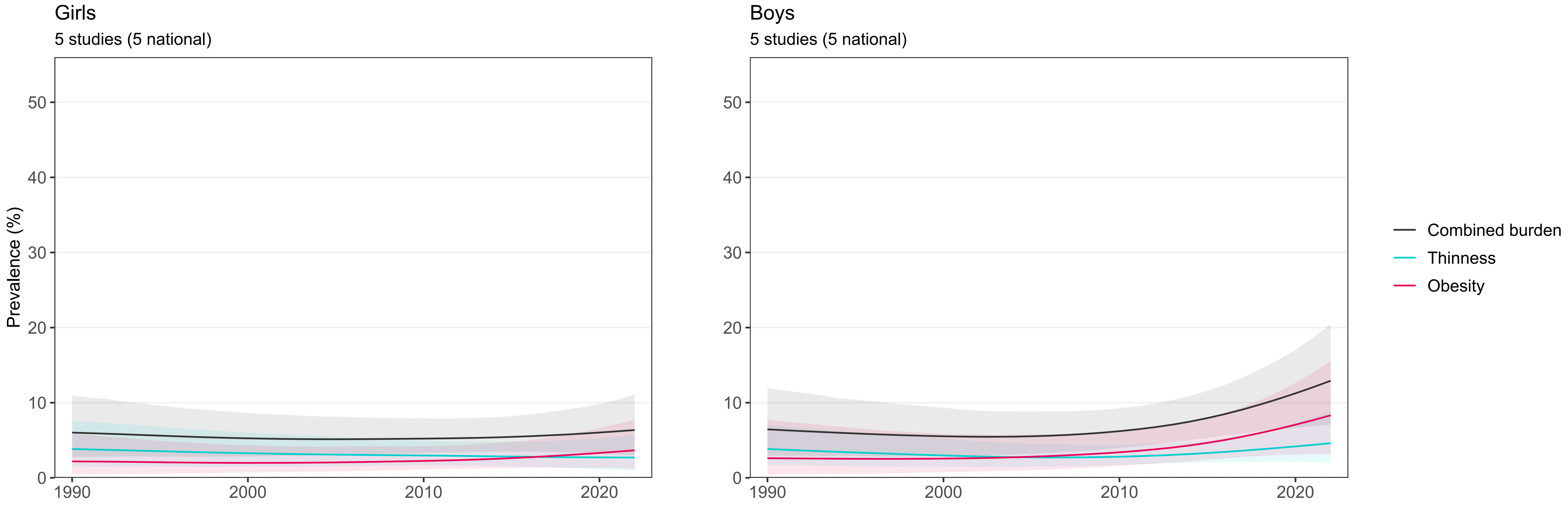


## Adults

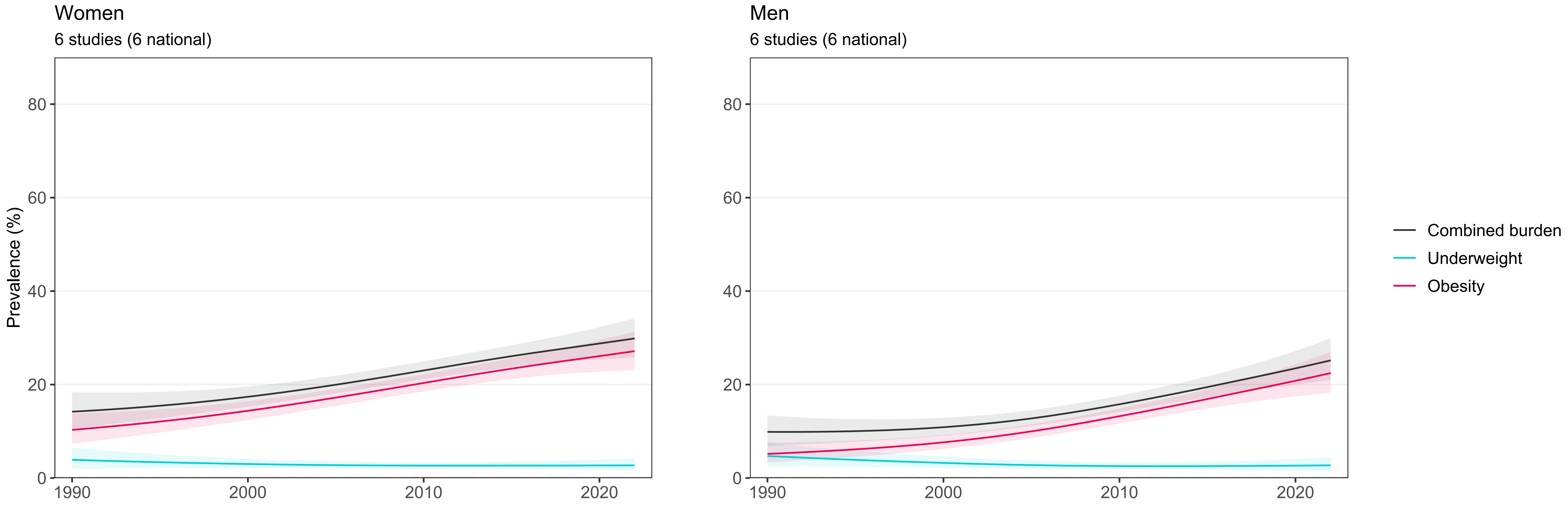

# Montenegro

## School-aged children and adolescents

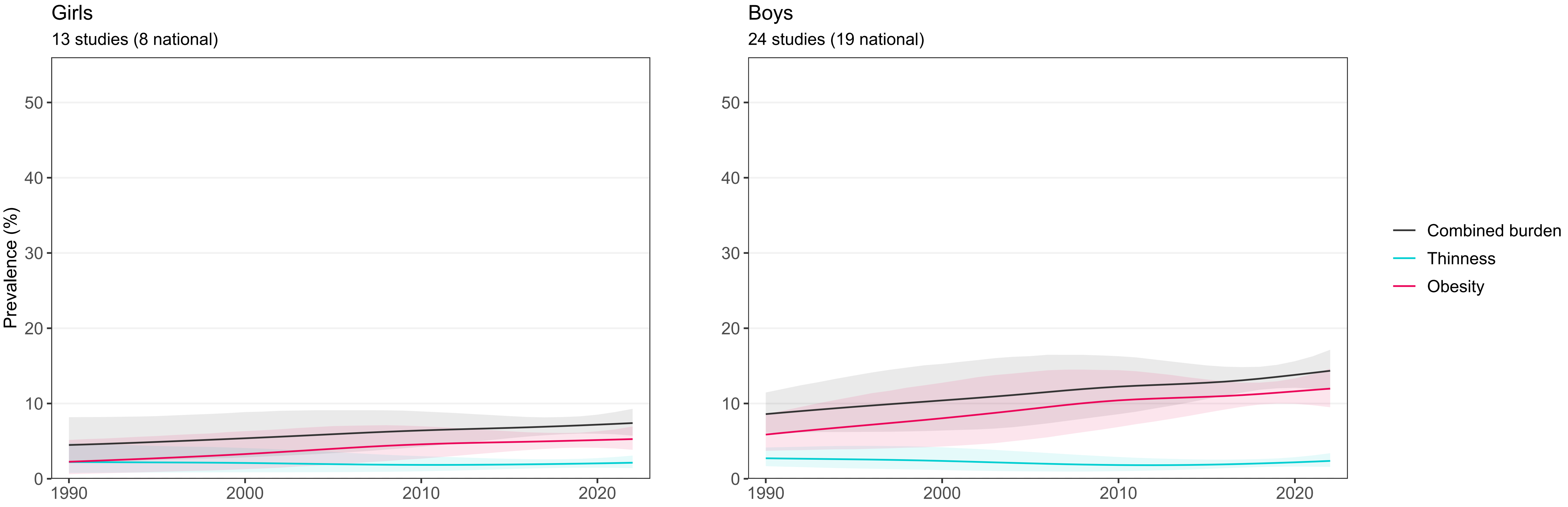


## Adults

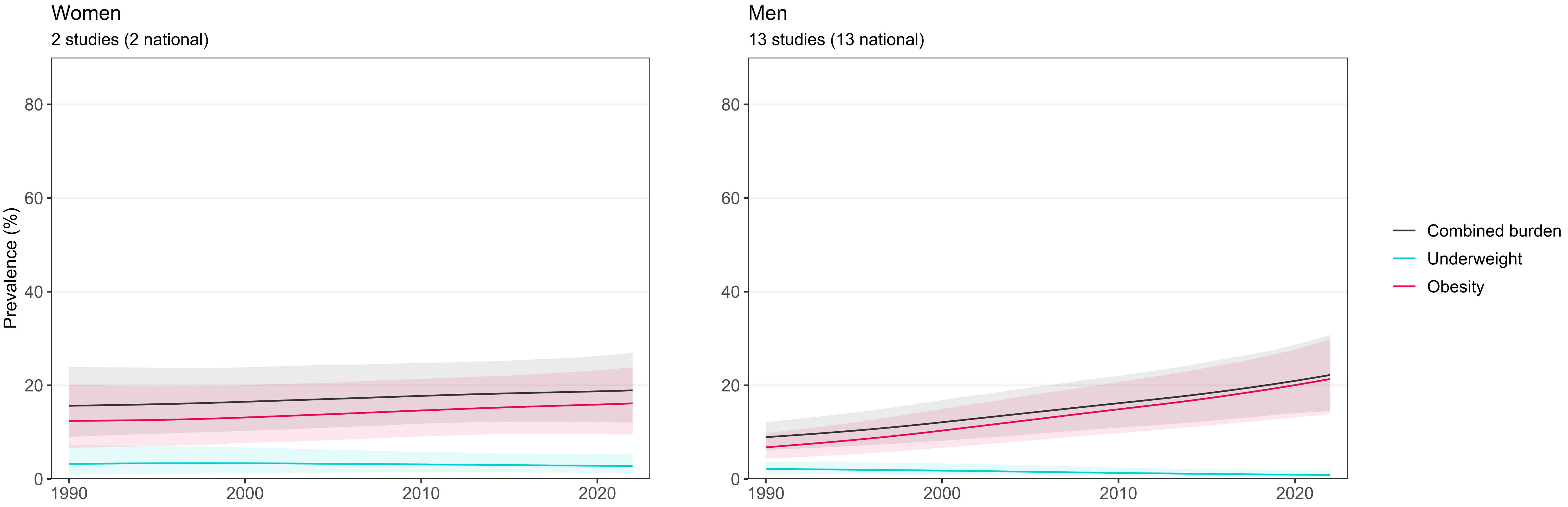

# Morocco

## School-aged children and adolescents

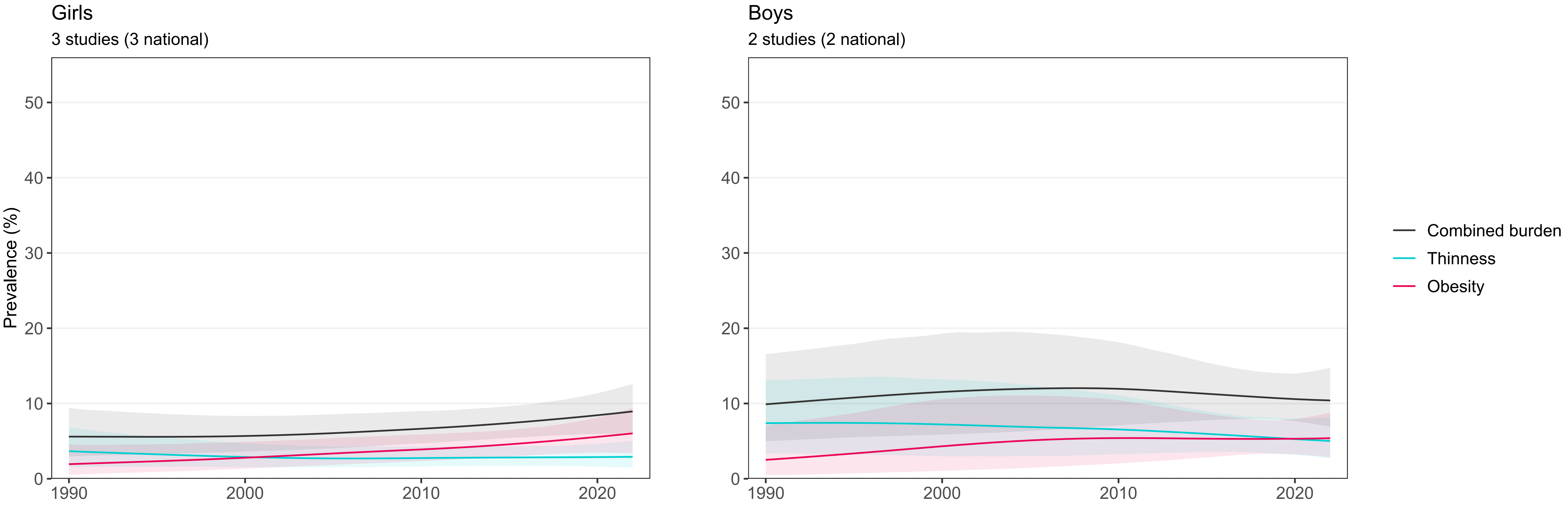


## Adults

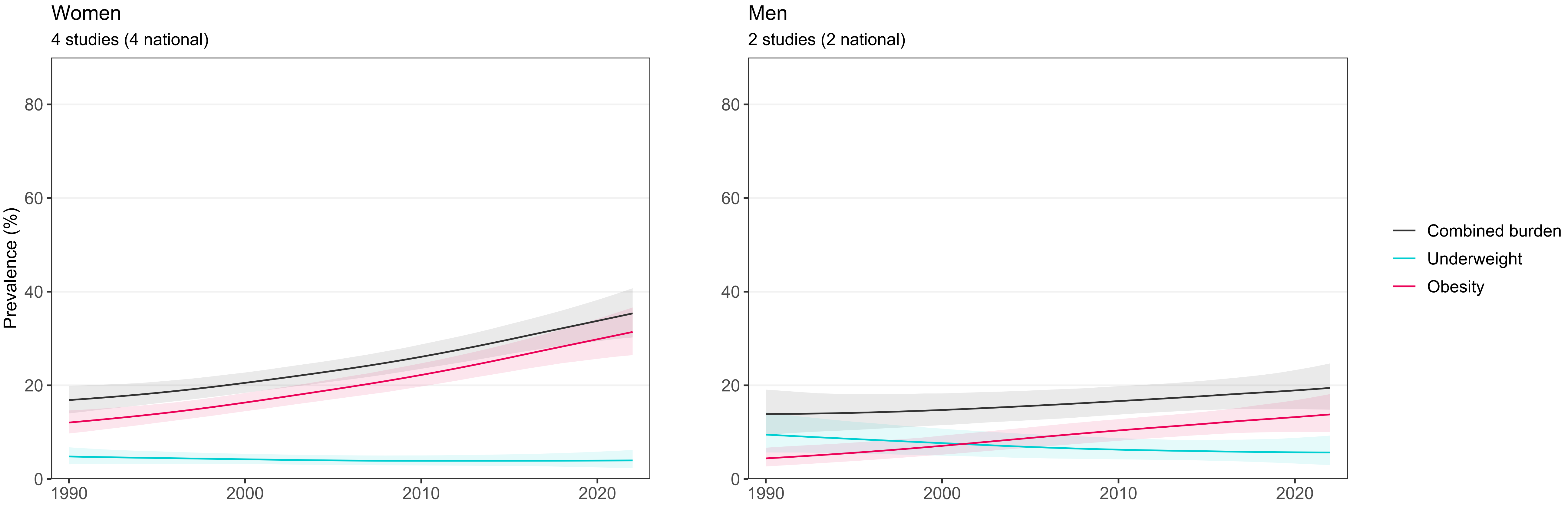

# Mozambique

## School-aged children and adolescents

### Girls
6 studies (3 national)

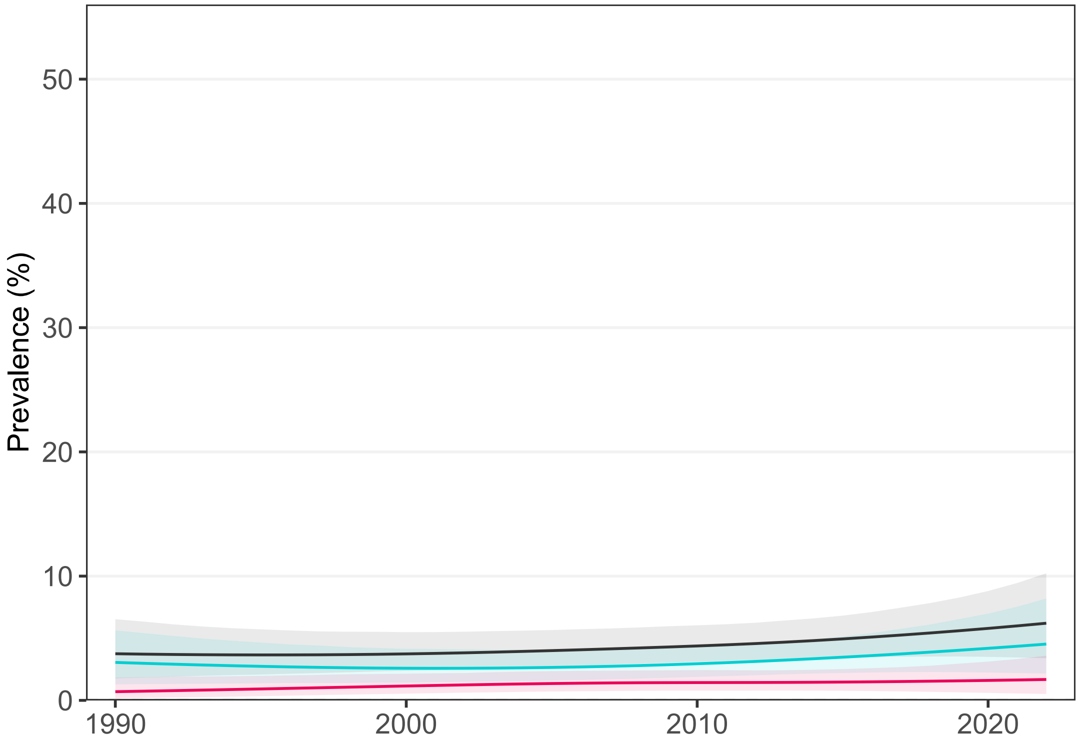


### Boys
4 studies (1 national)

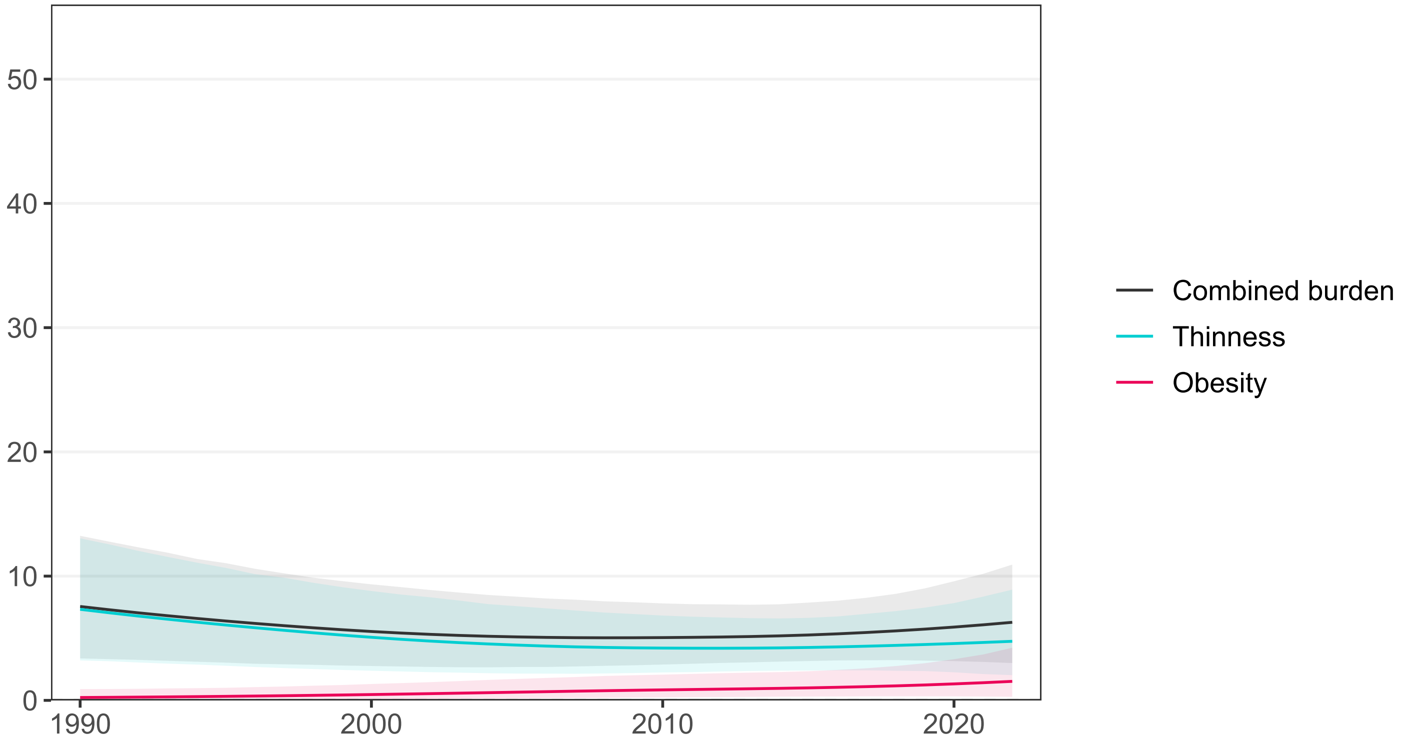


## Adults

### Women
5 studies (5 national)

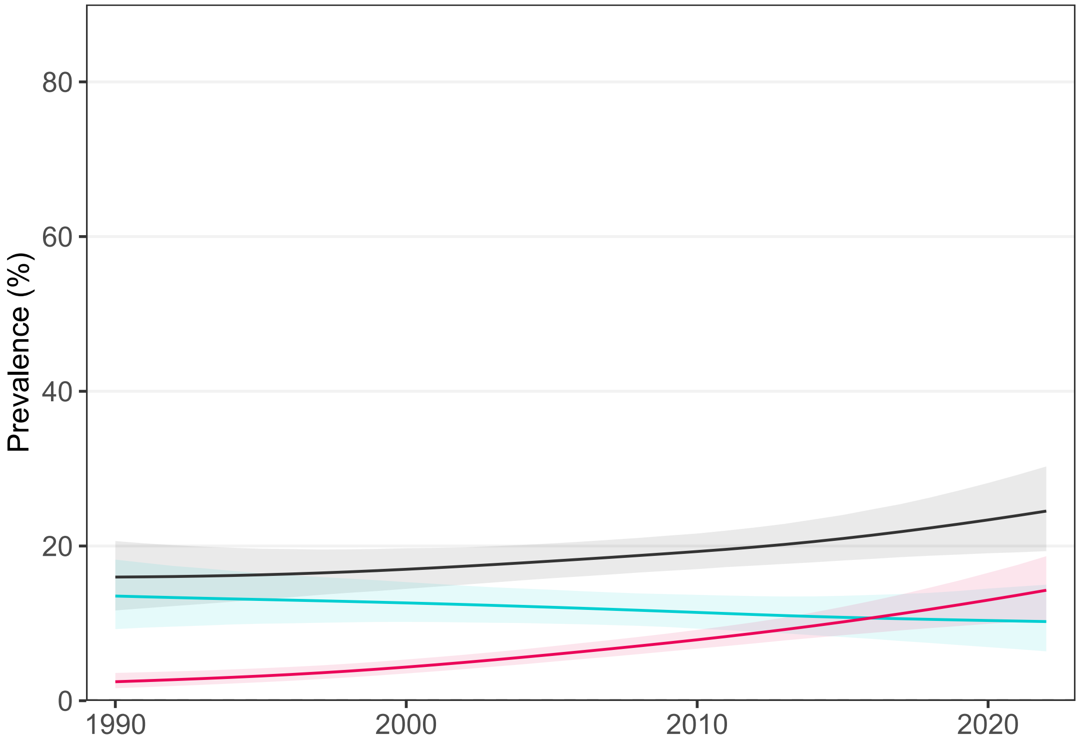


### Men
2 studies (2 national)

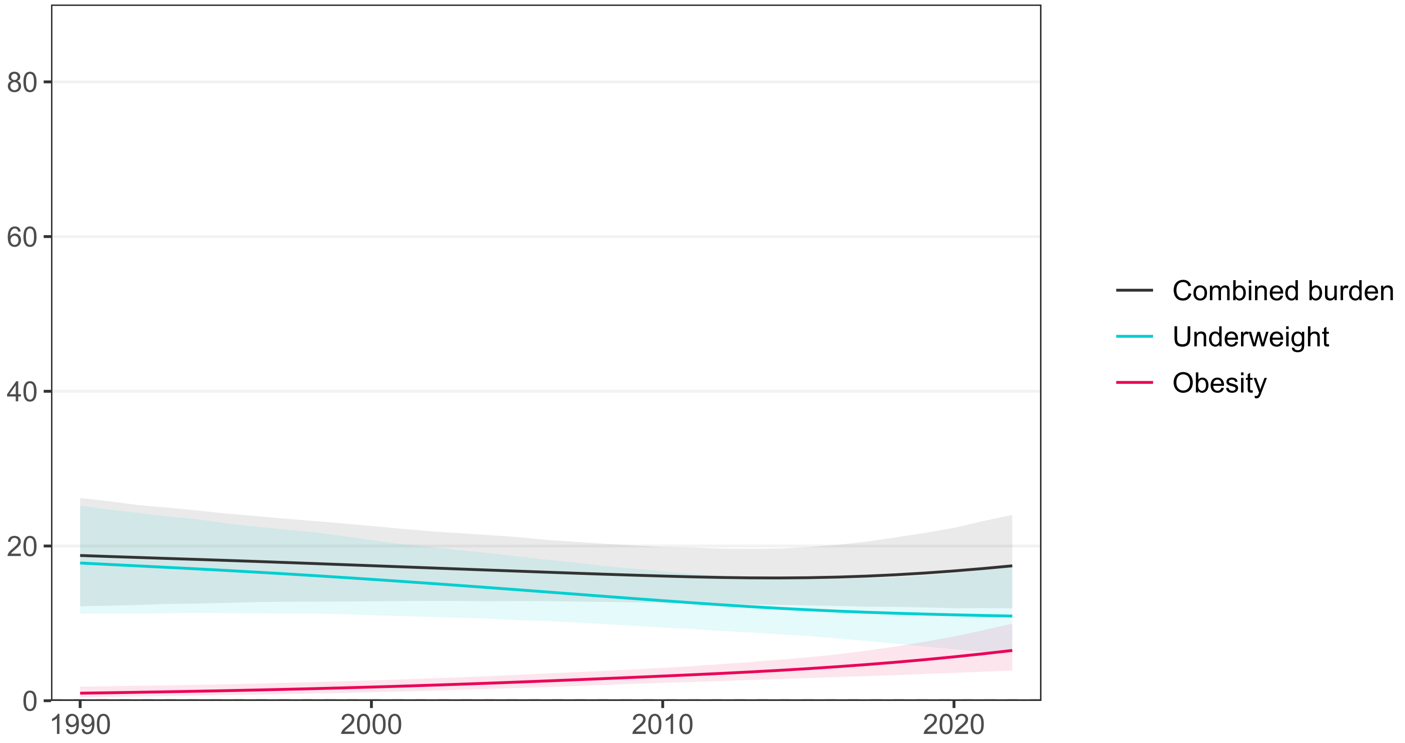

# Myanmar

## School-aged children and adolescents

### Girls

3 studies (2 national)

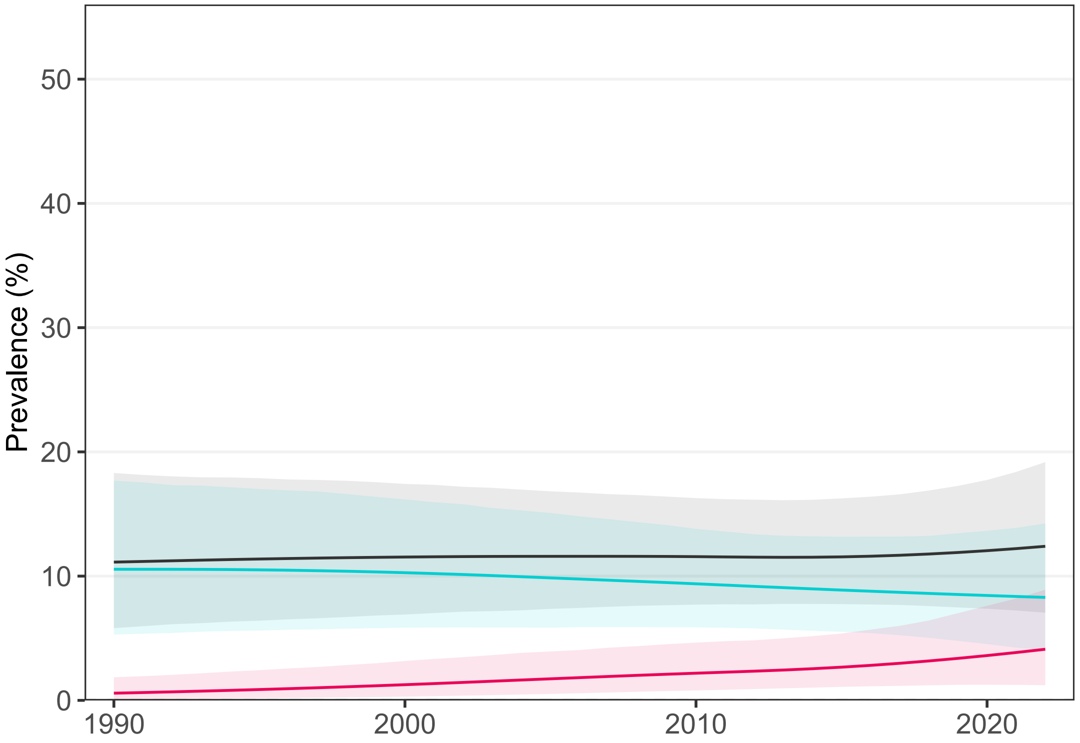


### Boys

2 studies (1 national)

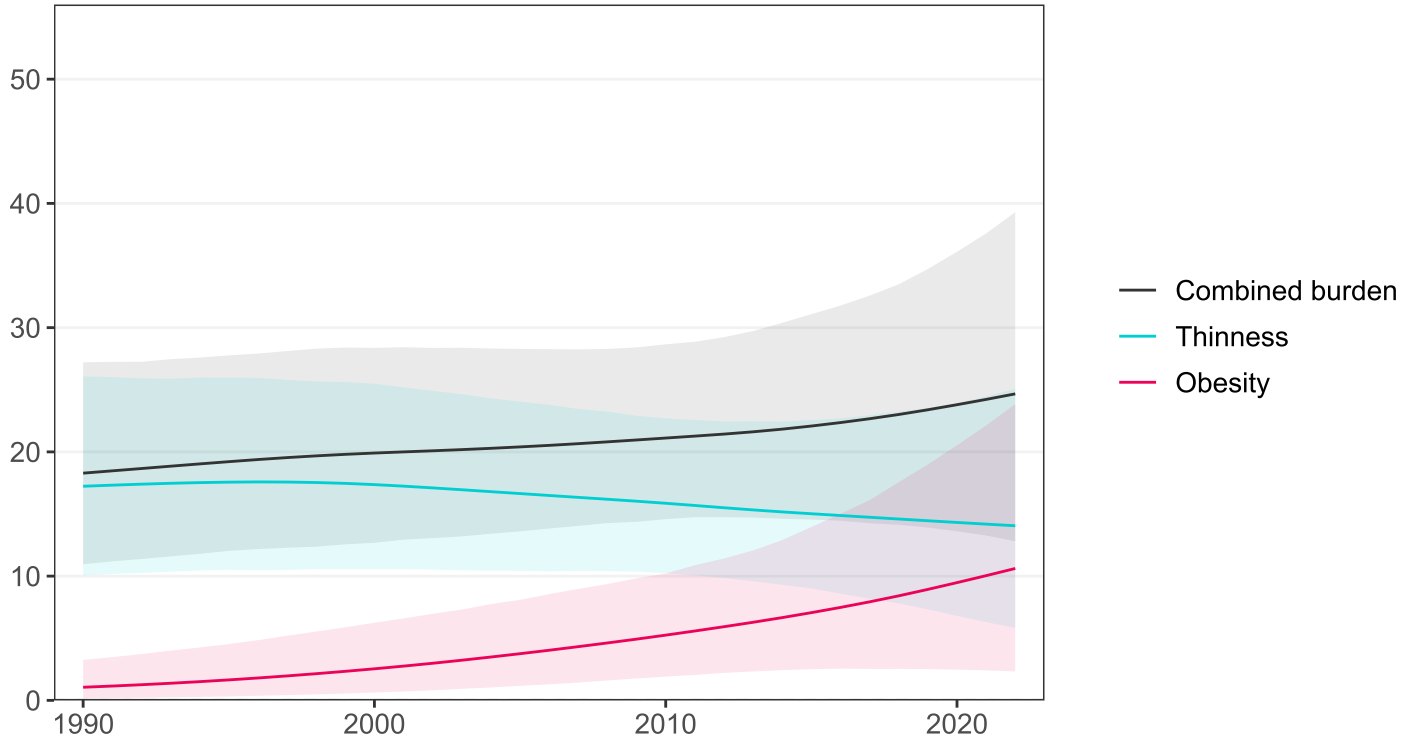


## Adults

### Women

6 studies (3 national)

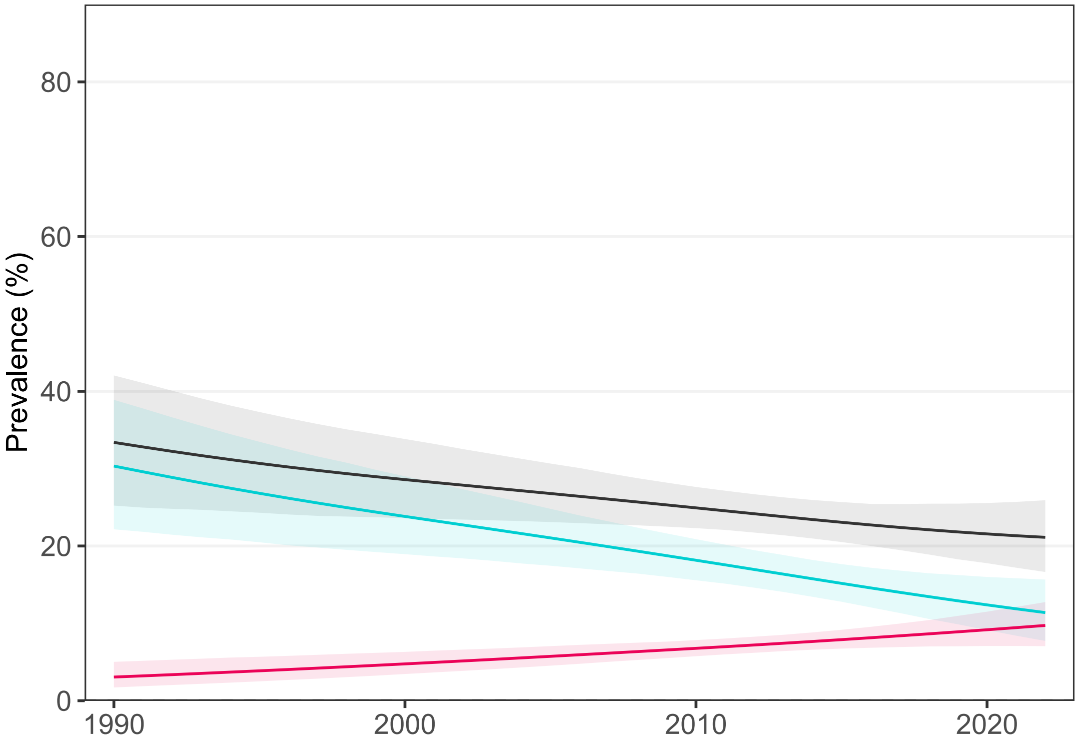


### Men

5 studies (2 national)

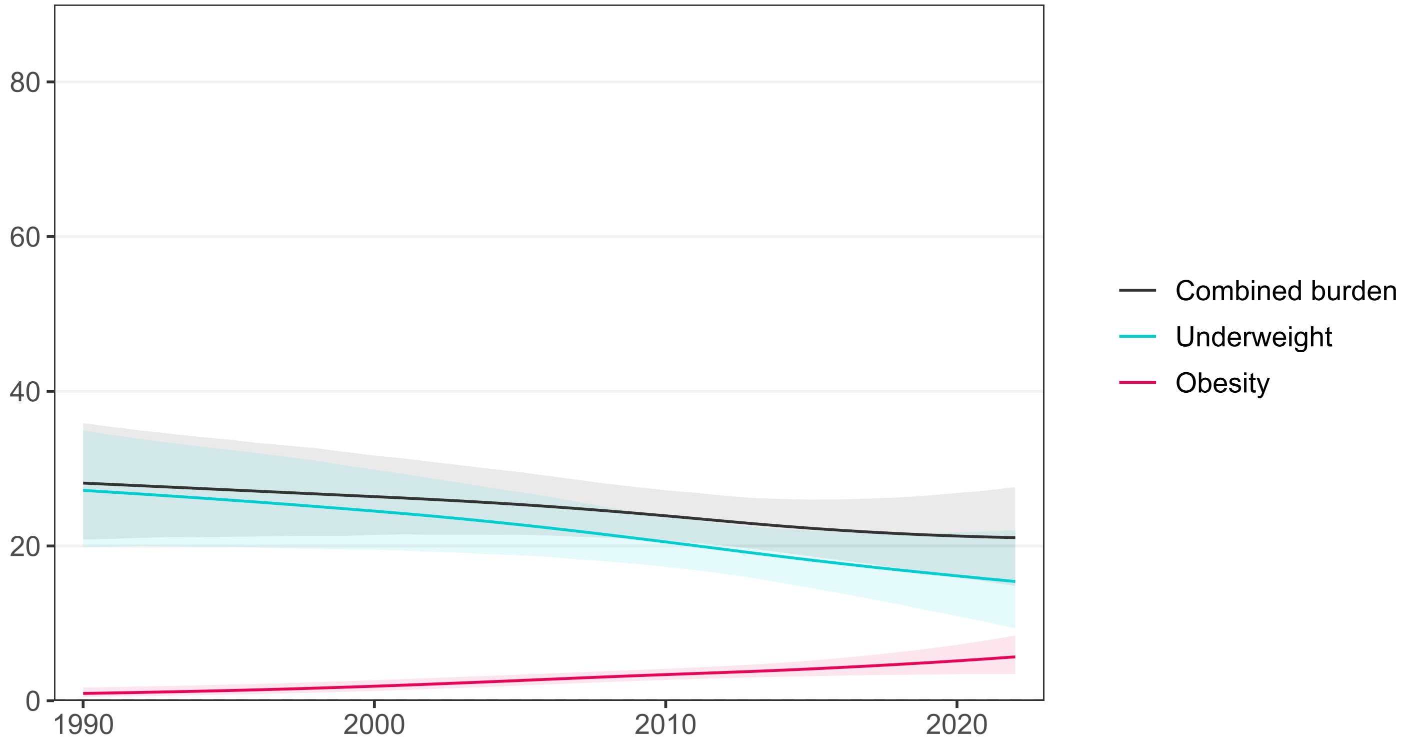

# Namibia

## School-aged children and adolescents

### Girls

3 studies (2 national)

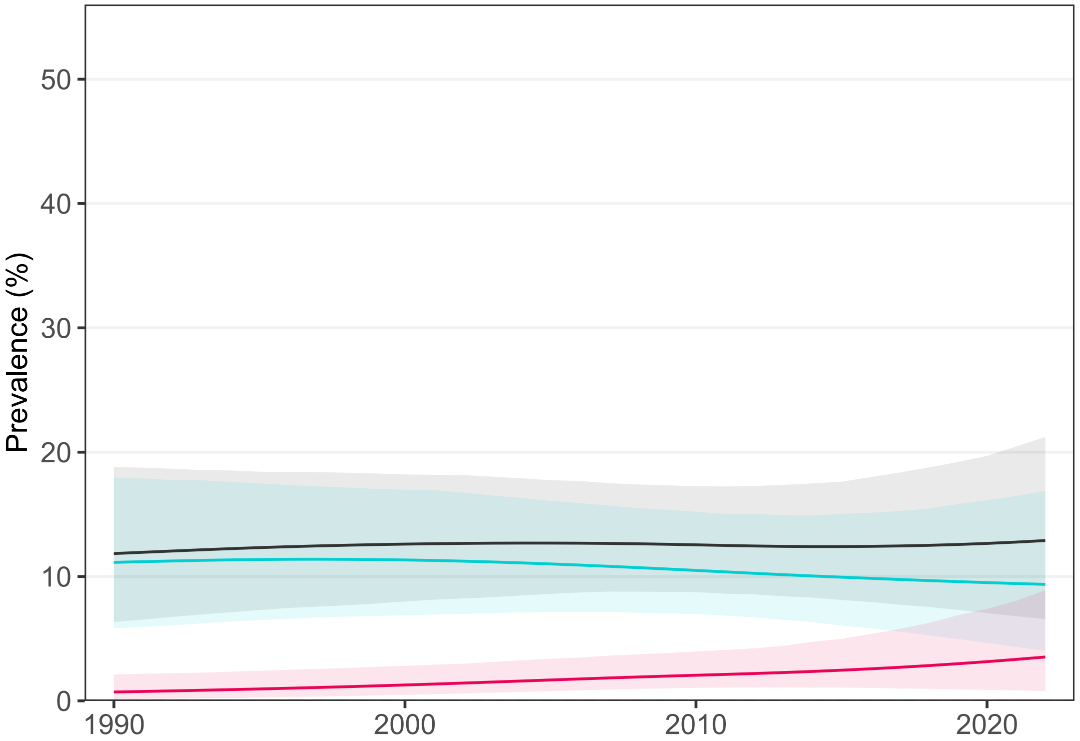


### Boys

1 study (0 national)

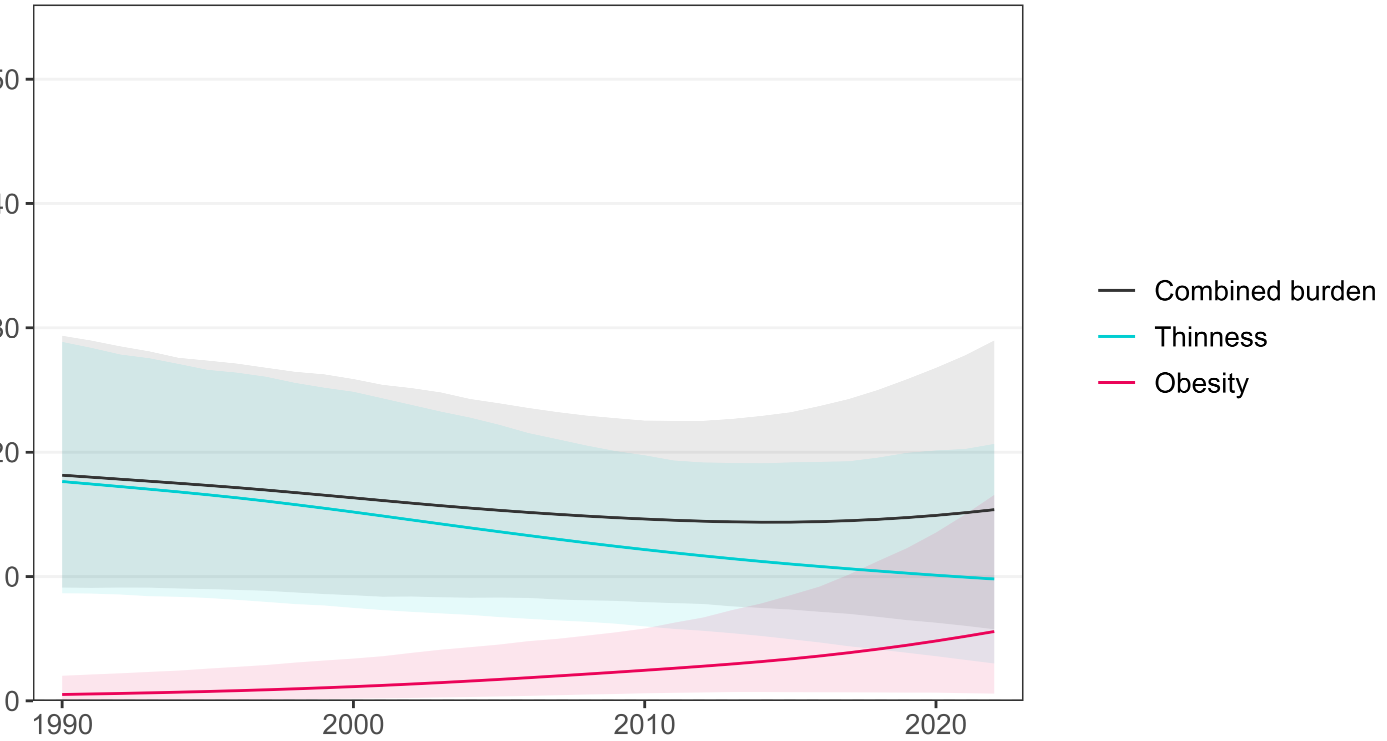


## Adults

### Women

5 studies (4 national)

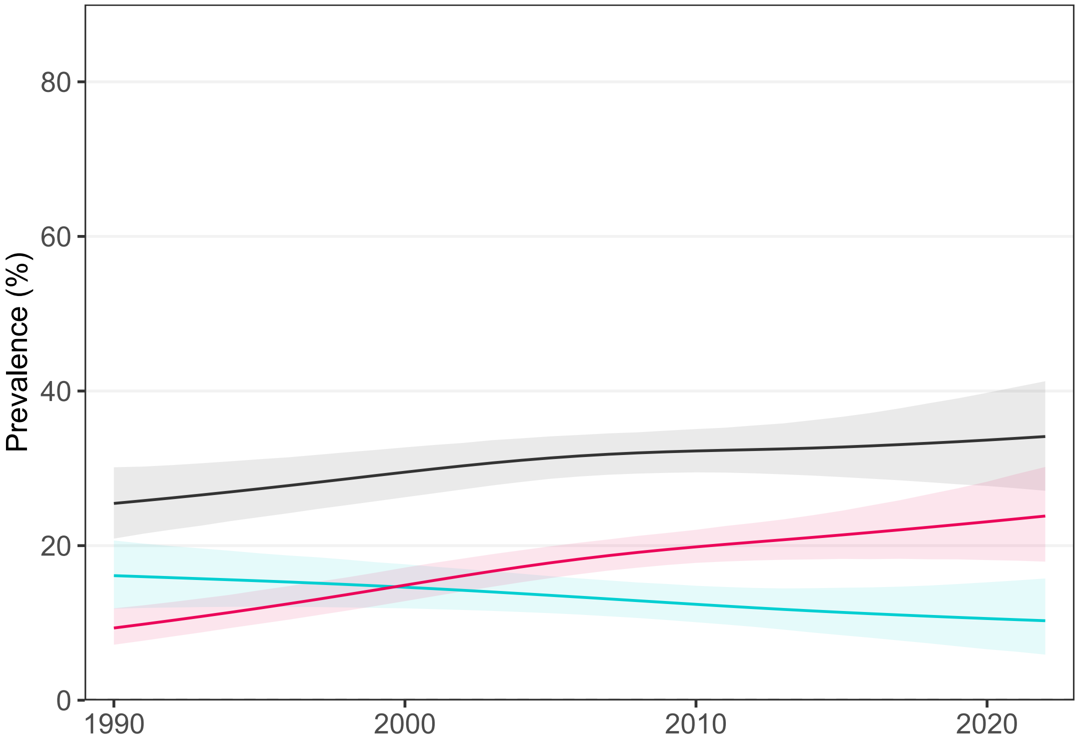


### Men

2 studies (1 national)

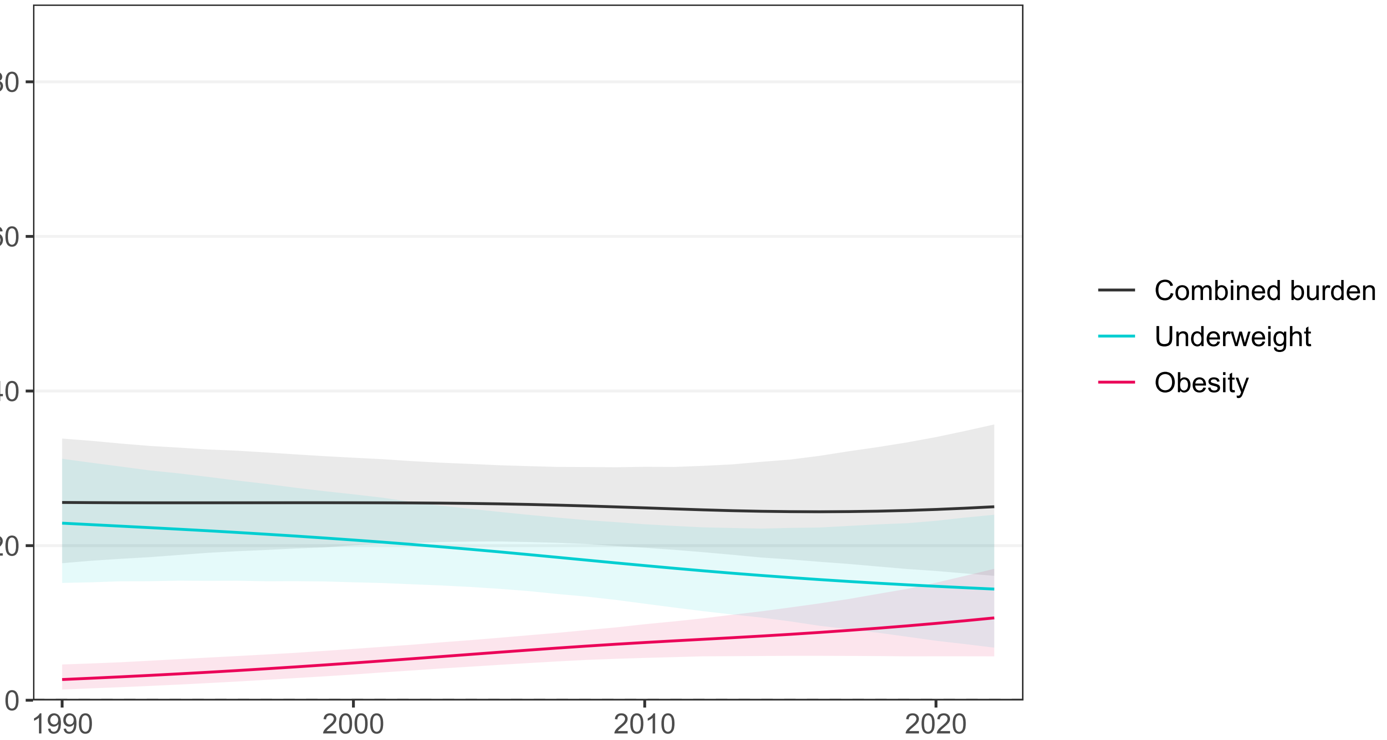

# Nauru

## School-aged children and adolescents

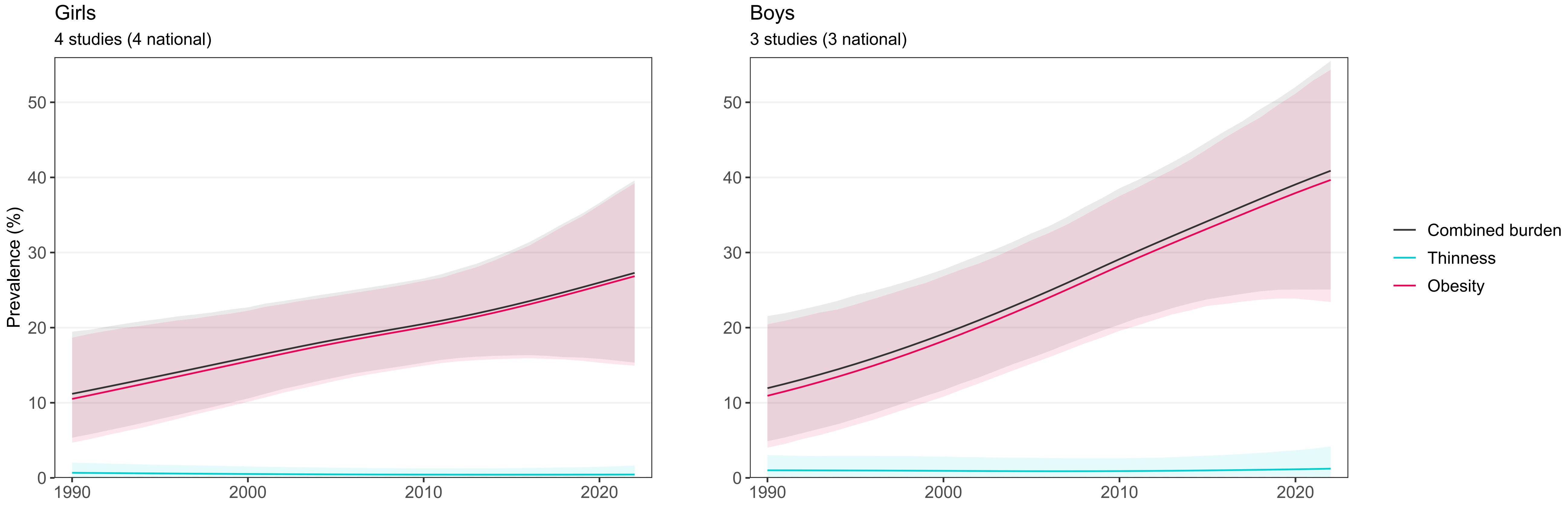


## Adults

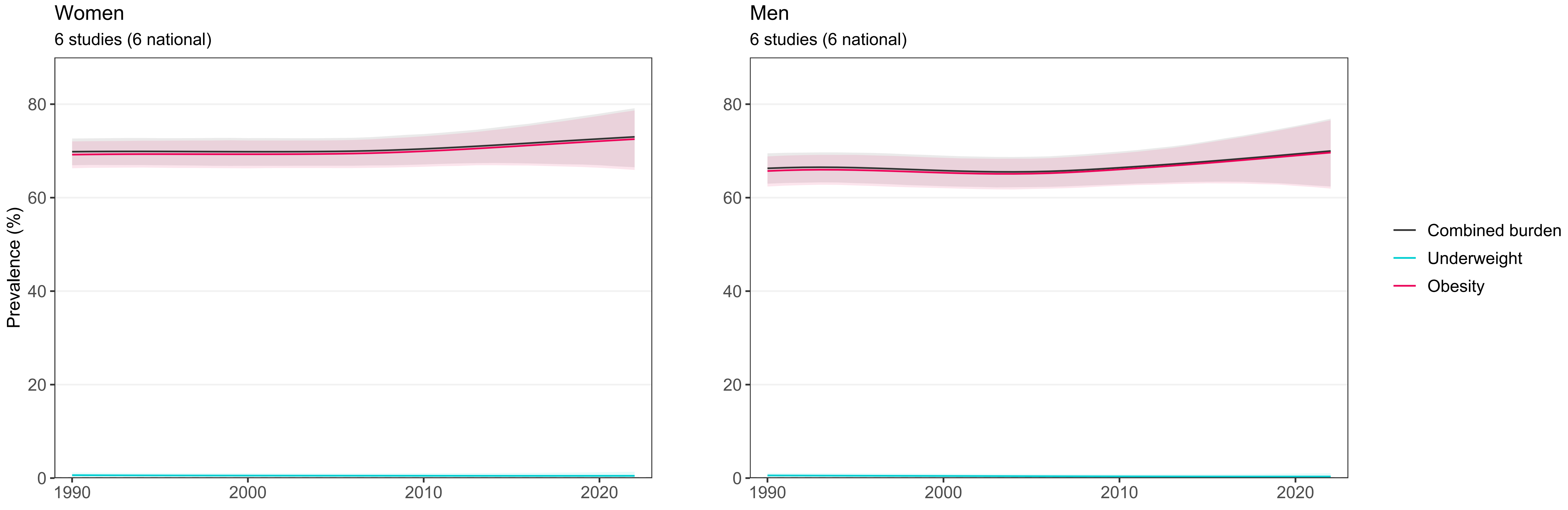

# Nepal

## School-aged children and adolescents

### Girls

12 studies (6 national)

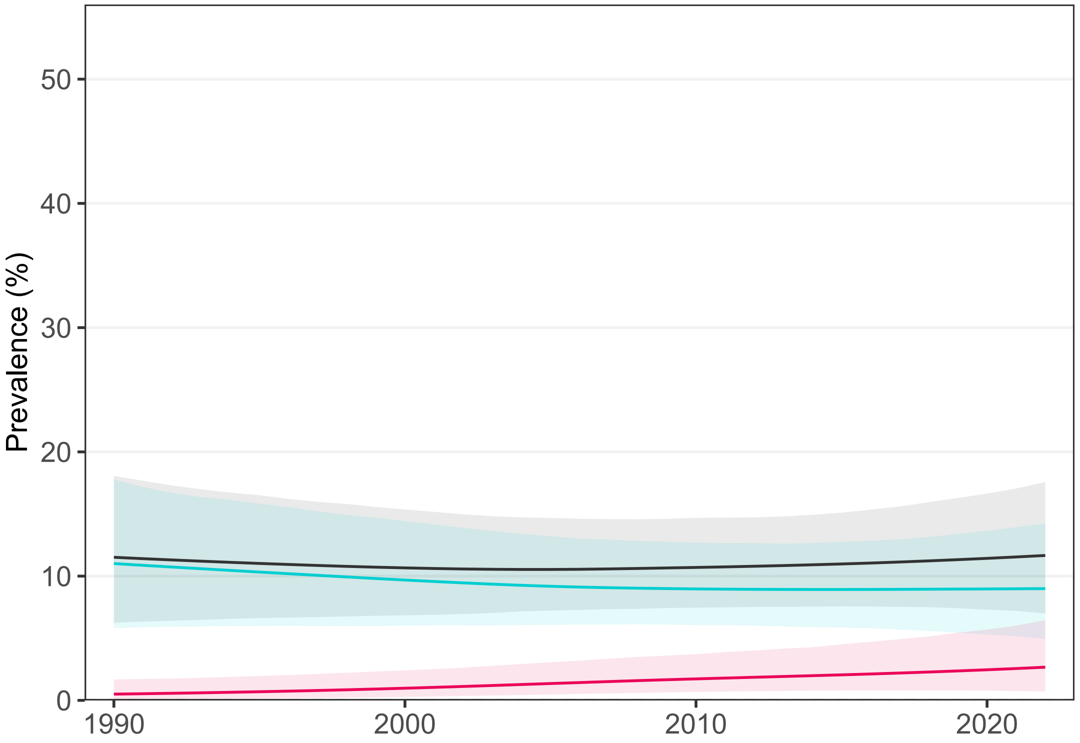


### Boys

10 studies (4 national)

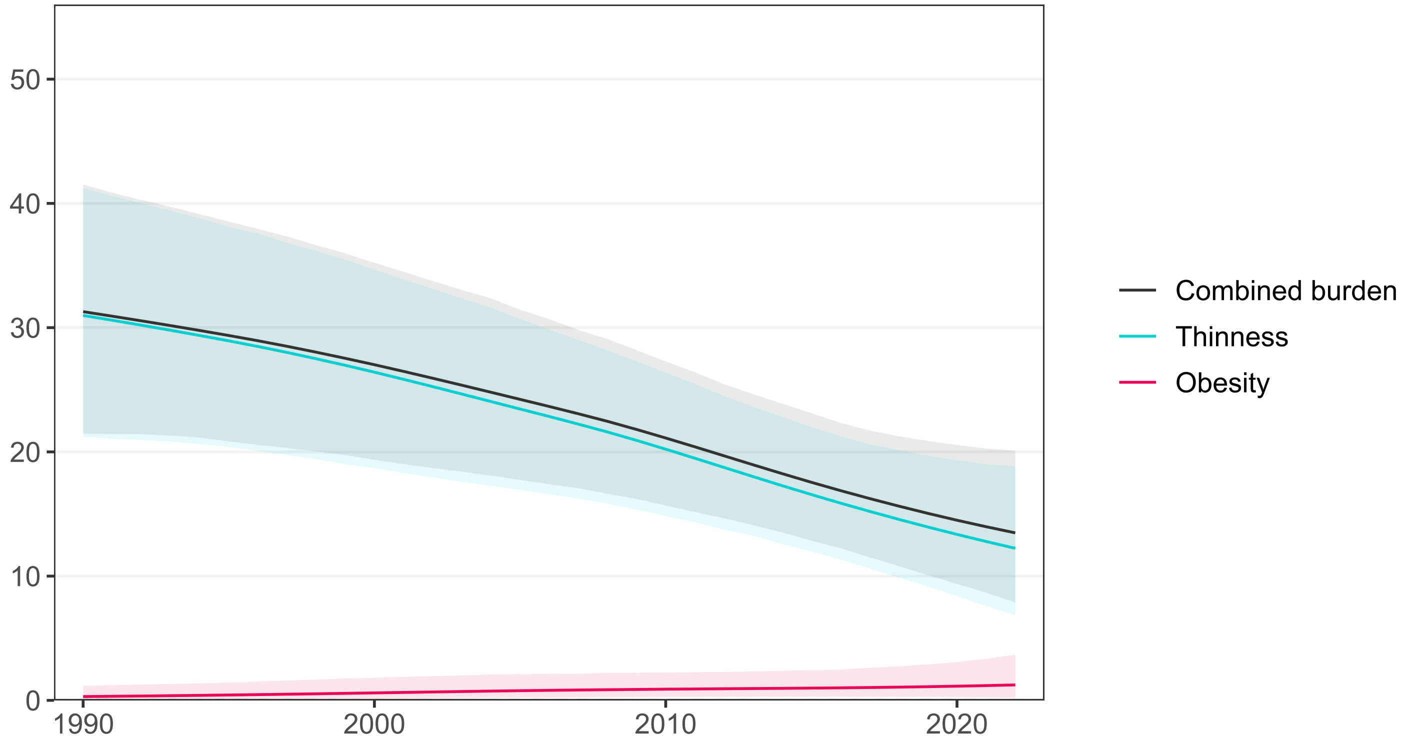


## Adults

### Women

17 studies (9 national)

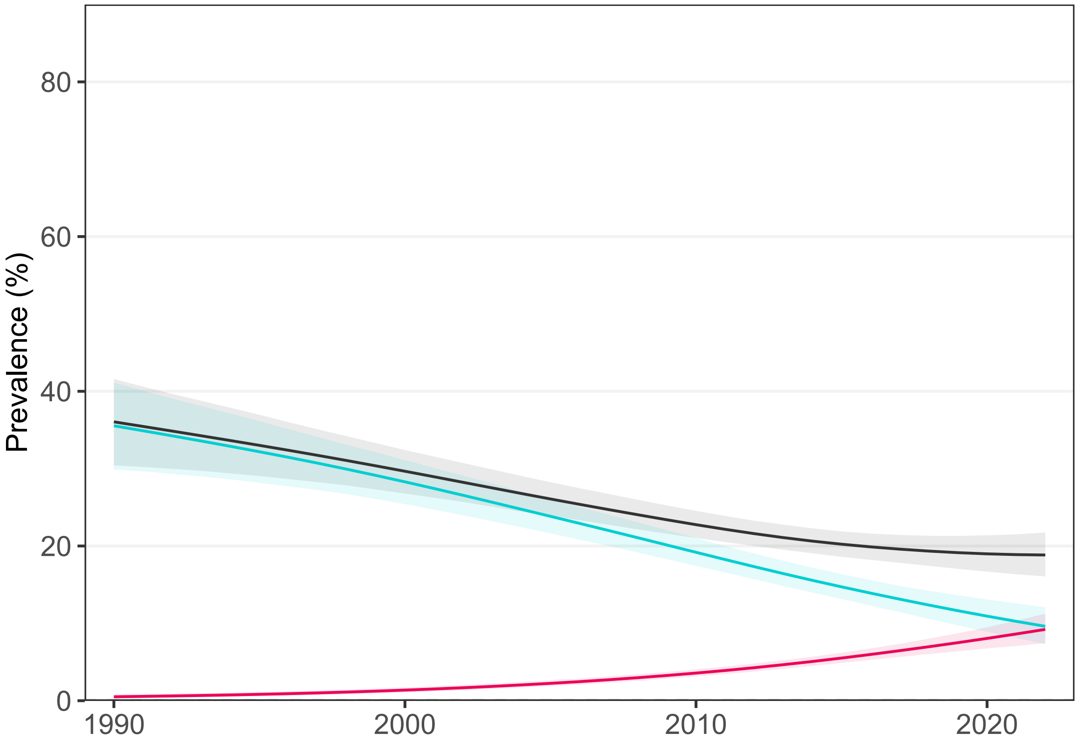


### Men

13 studies (5 national)

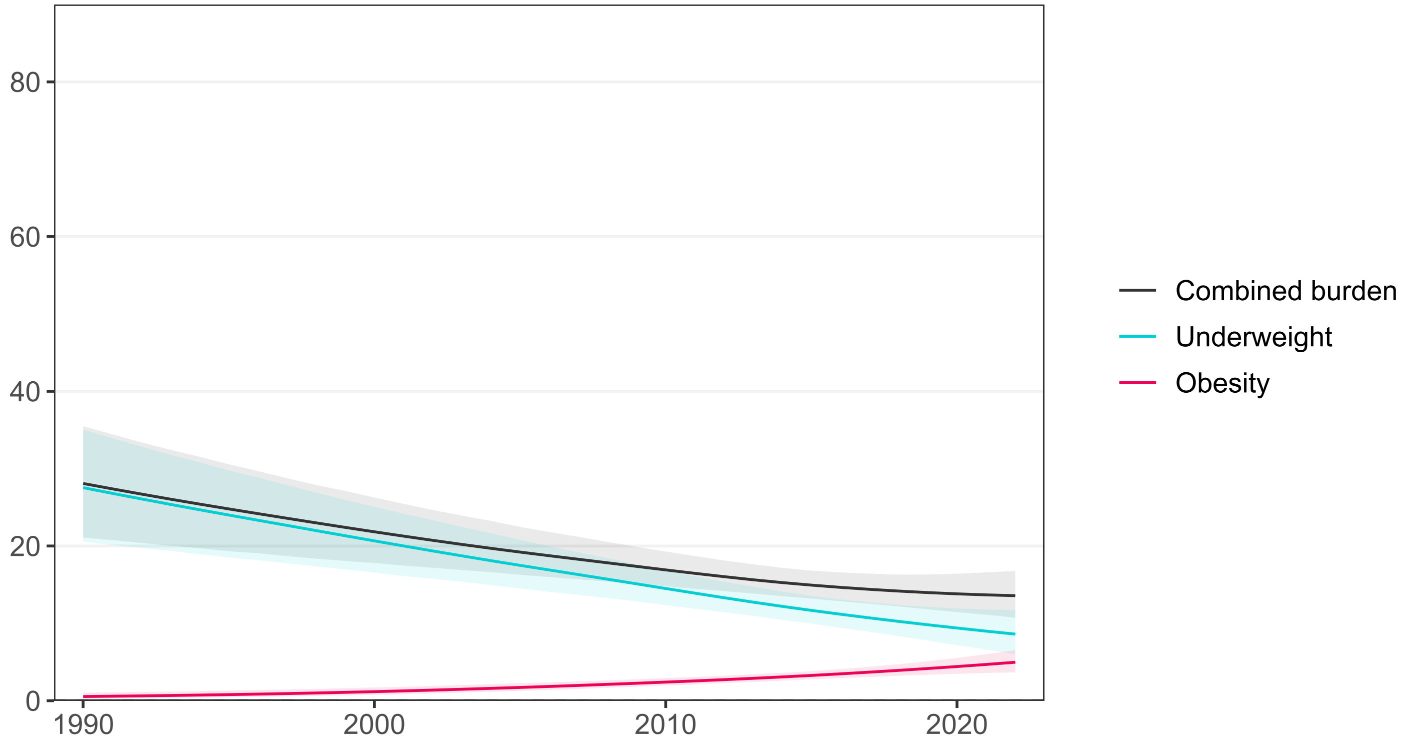

# Netherlands

## School-aged children and adolescents

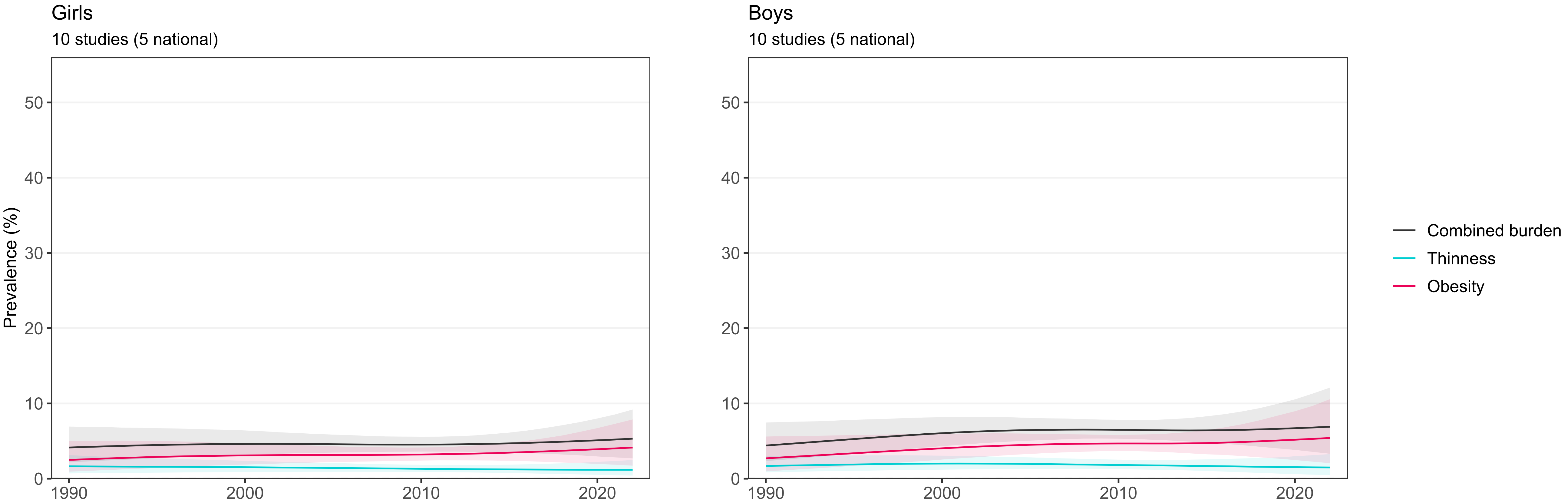


## Adults

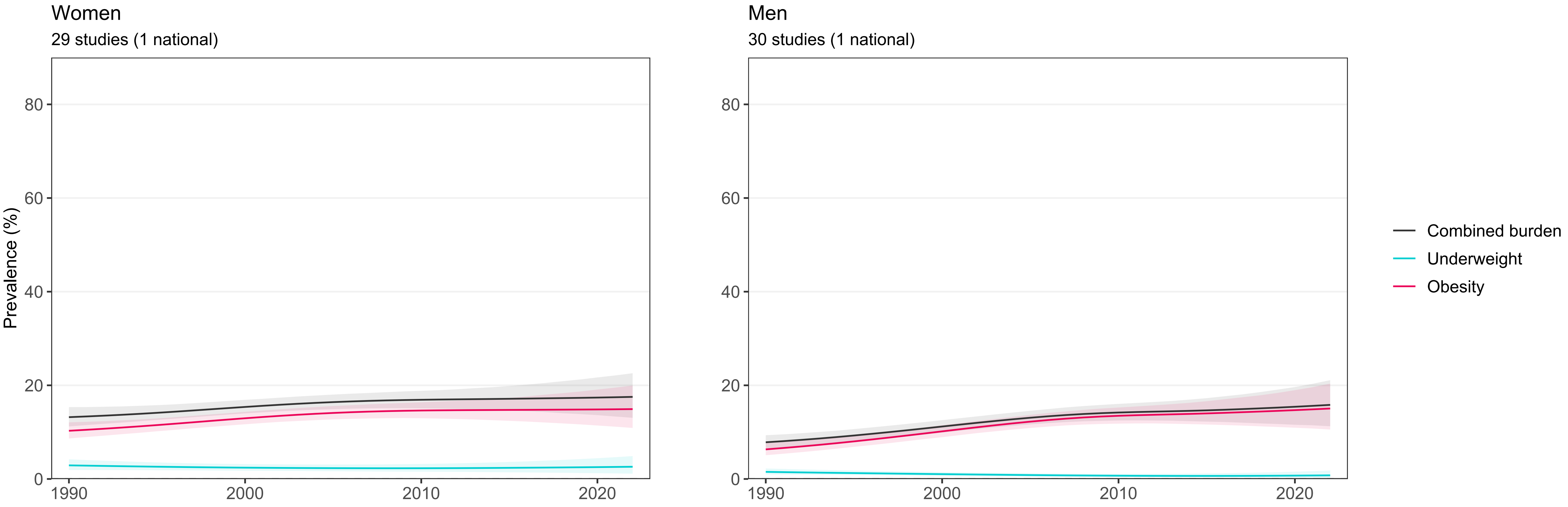

# New Zealand

## School-aged children and adolescents

### Girls

17 studies (16 national)

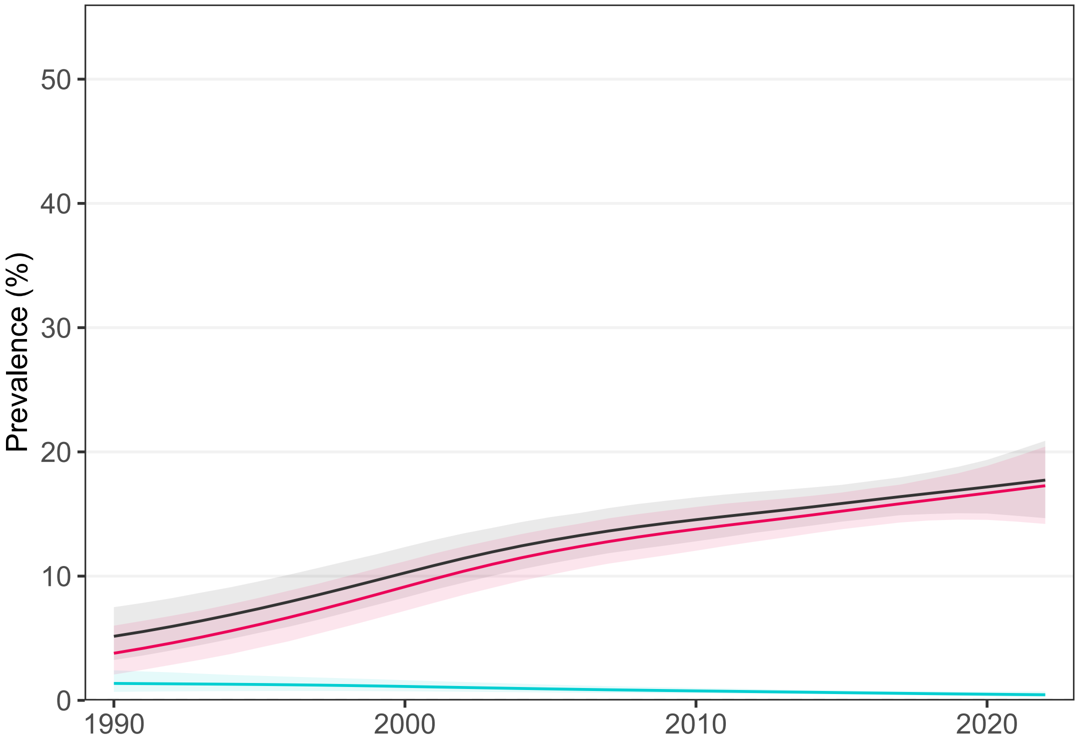


### Boys

17 studies (16 national)

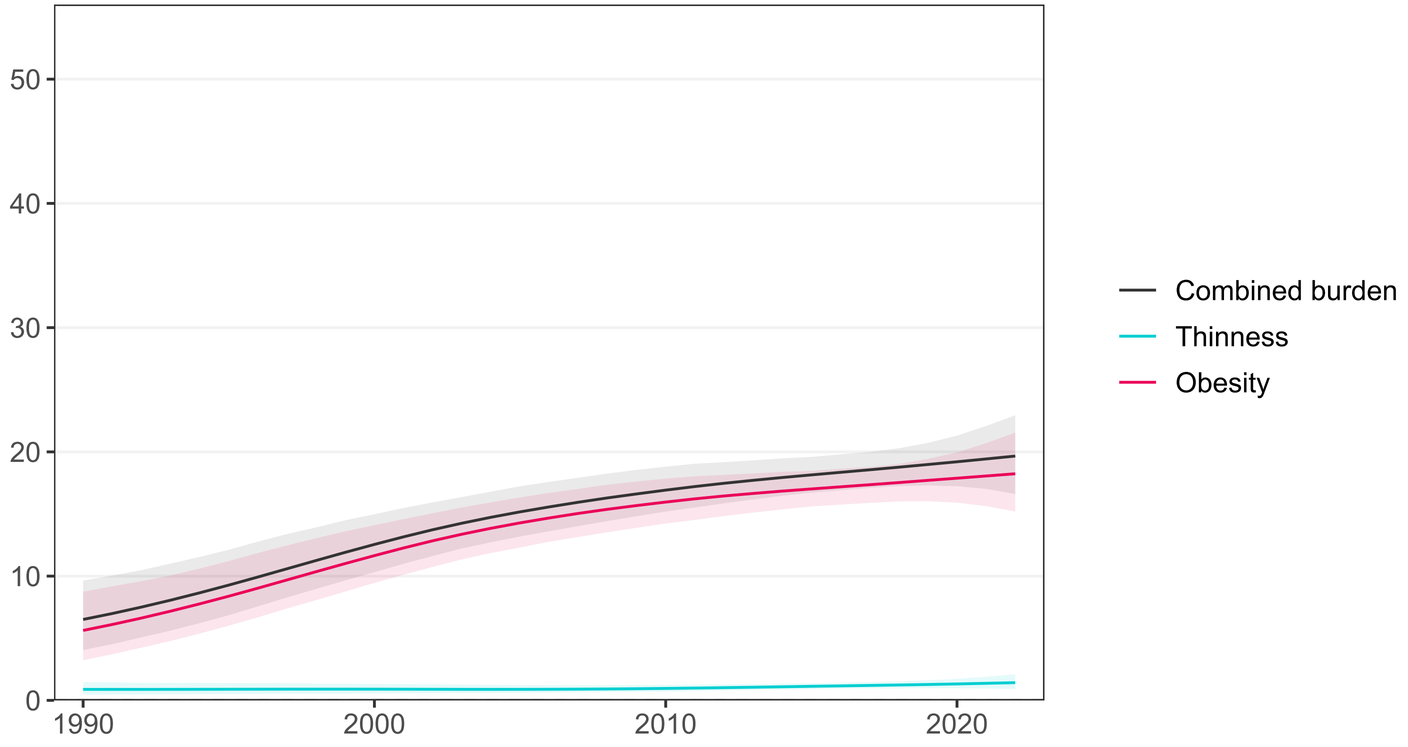


## Adults

### Women

18 studies (15 national)

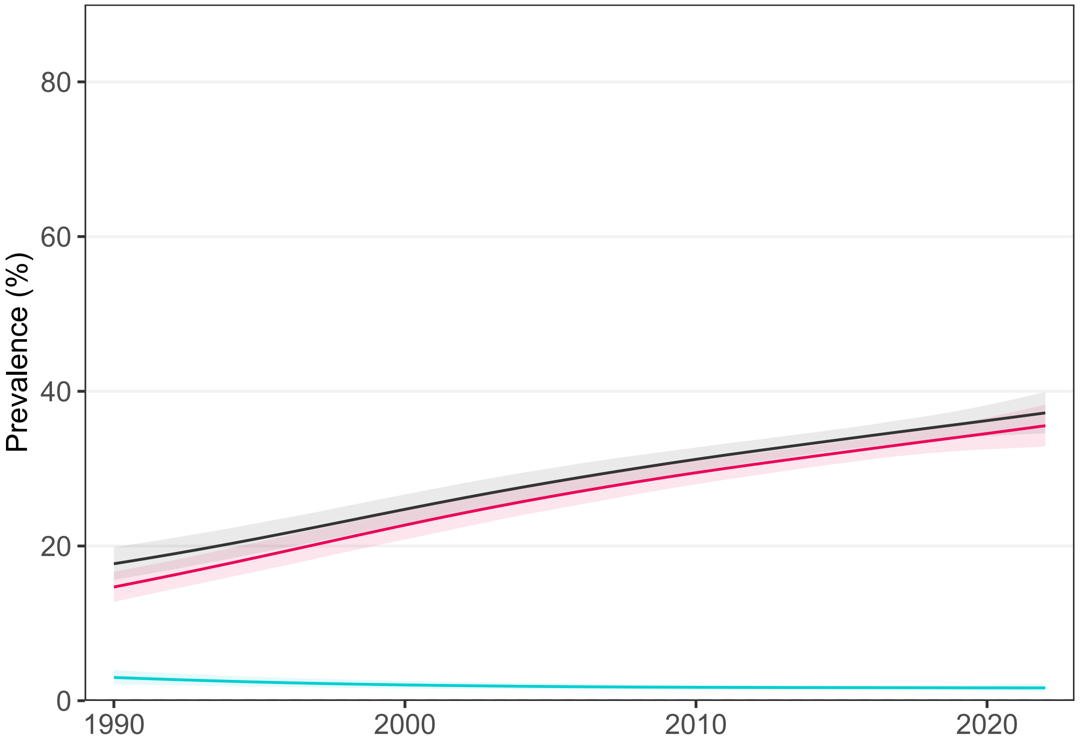


### Men

18 studies (15 national)

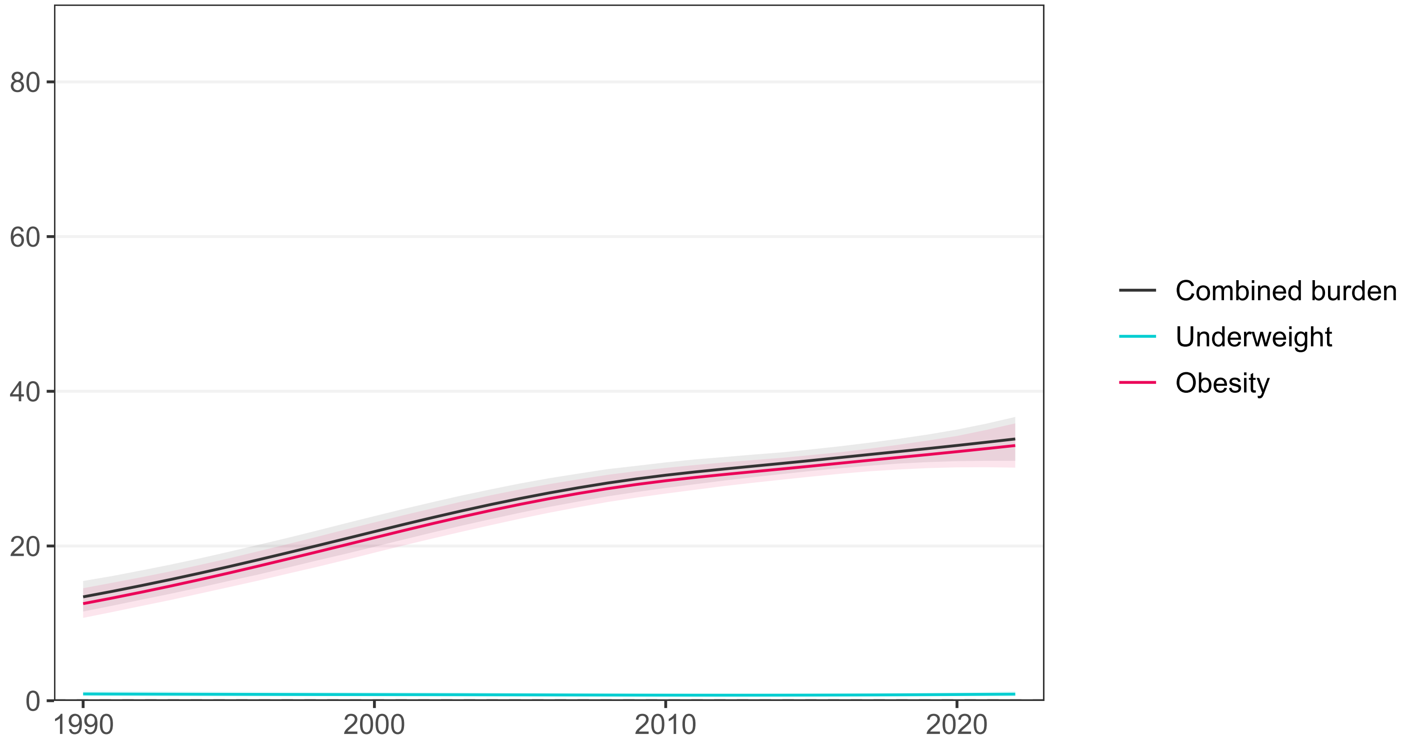

# Nicaragua

## School-aged children and adolescents

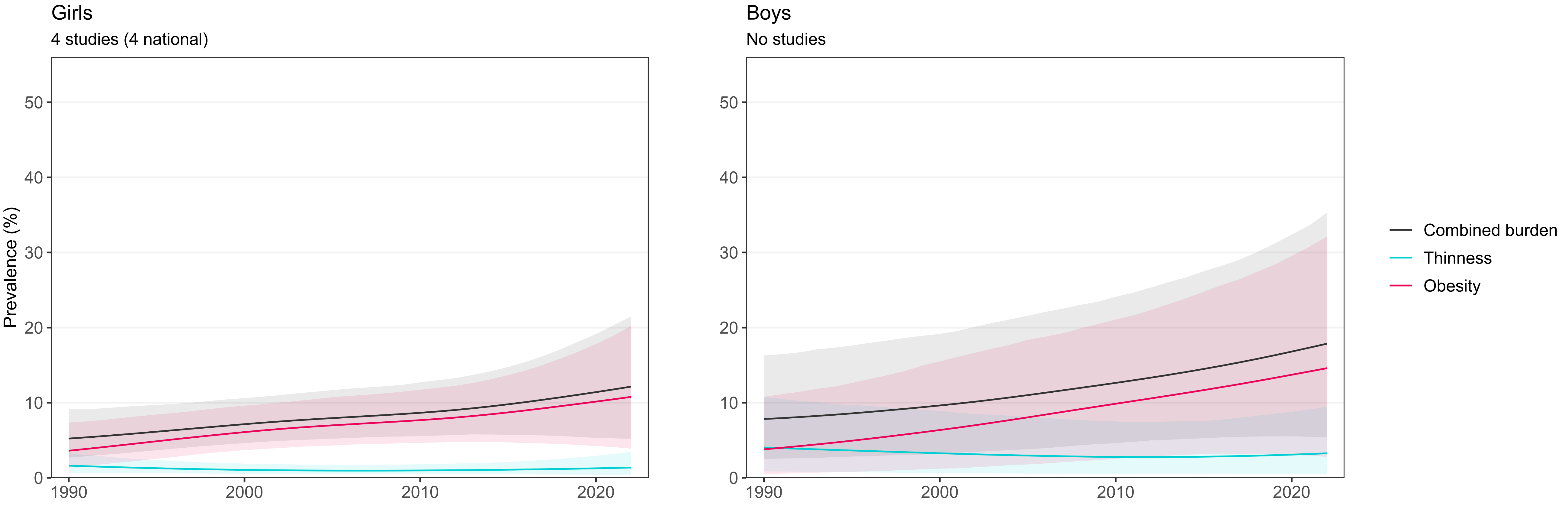


## Adults

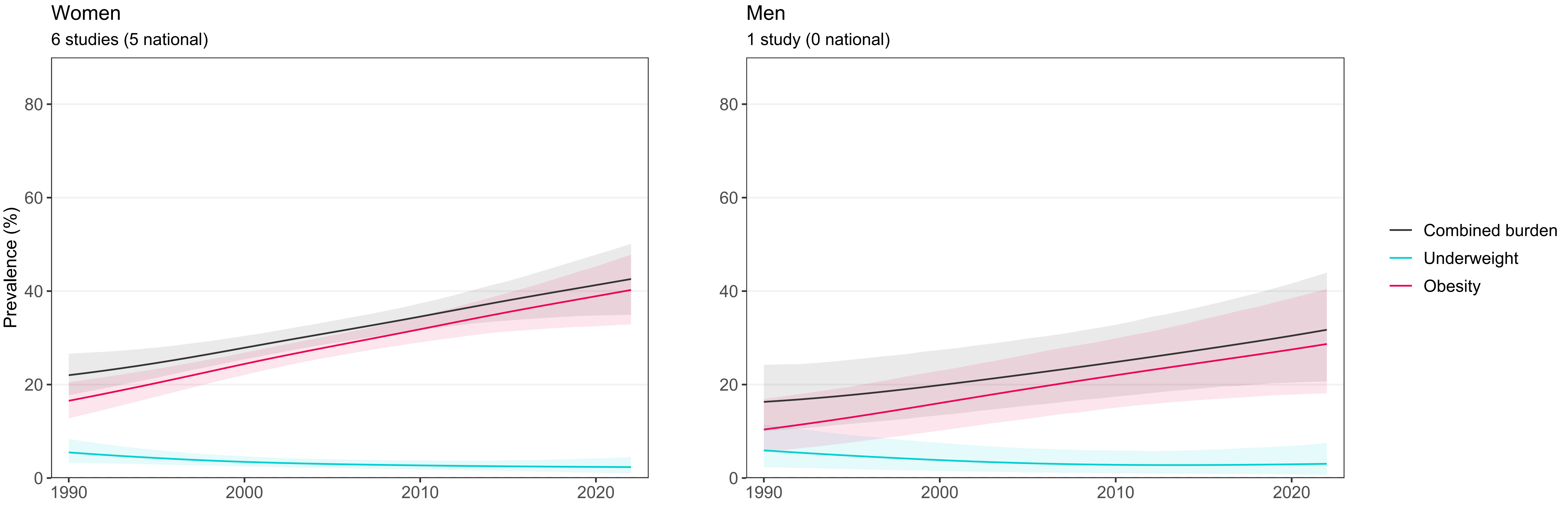

# Niger

## School-aged children and adolescents

### Girls

4 studies (4 national)

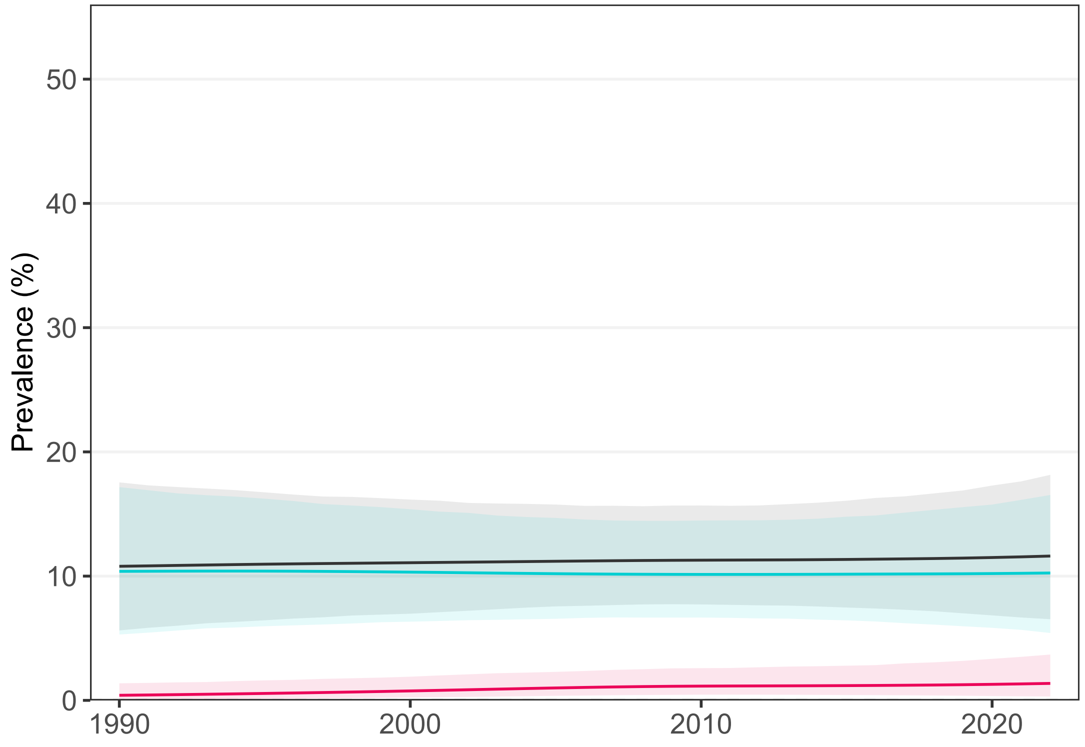


### Boys

2 studies (2 national)

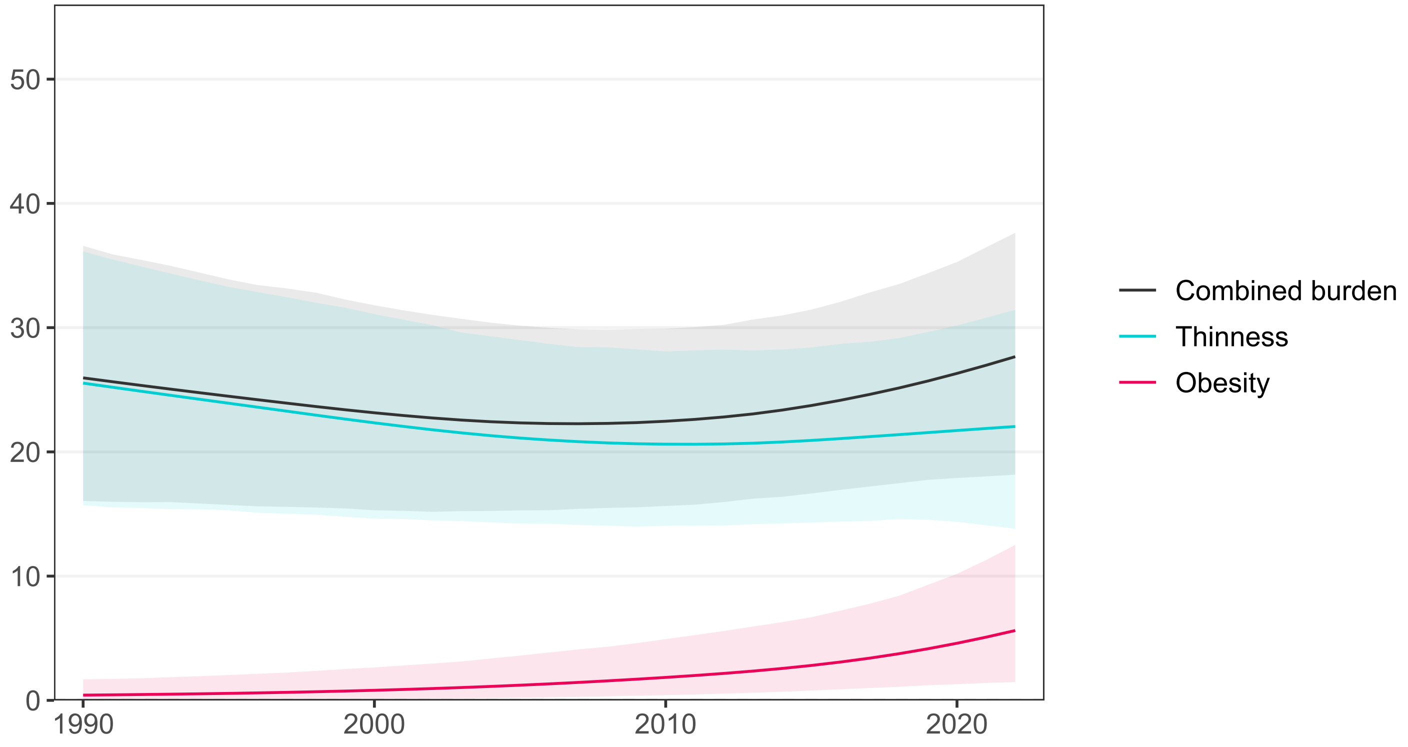


## Adults

### Women

6 studies (6 national)

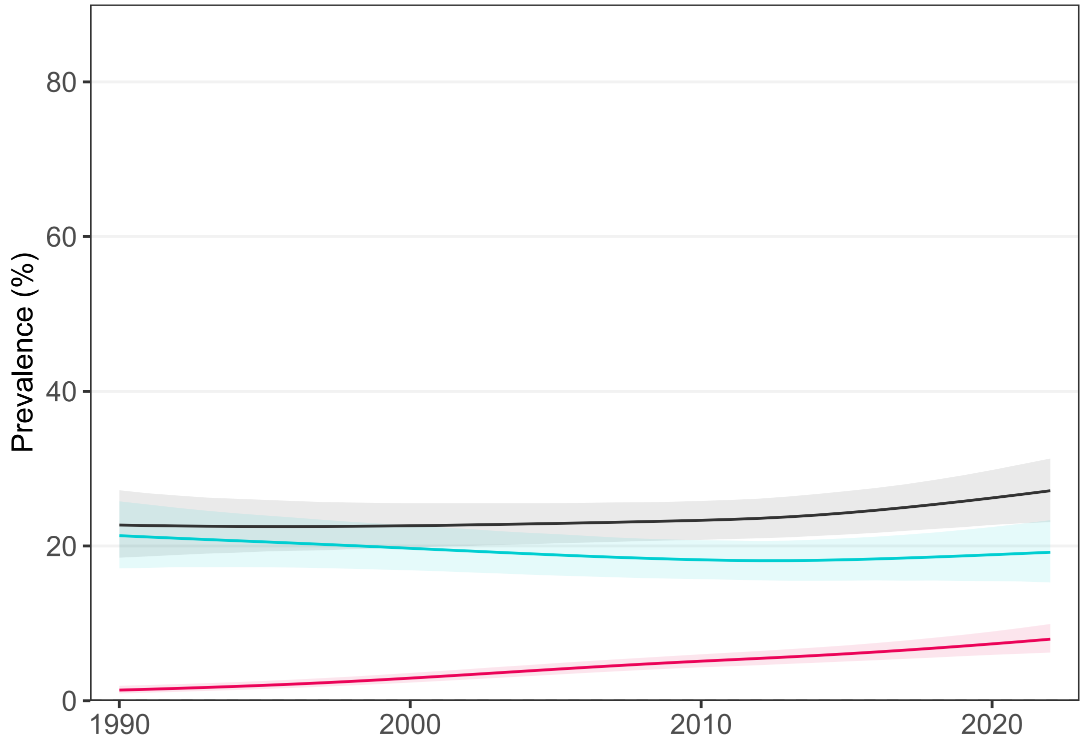


### Men

2 studies (2 national)

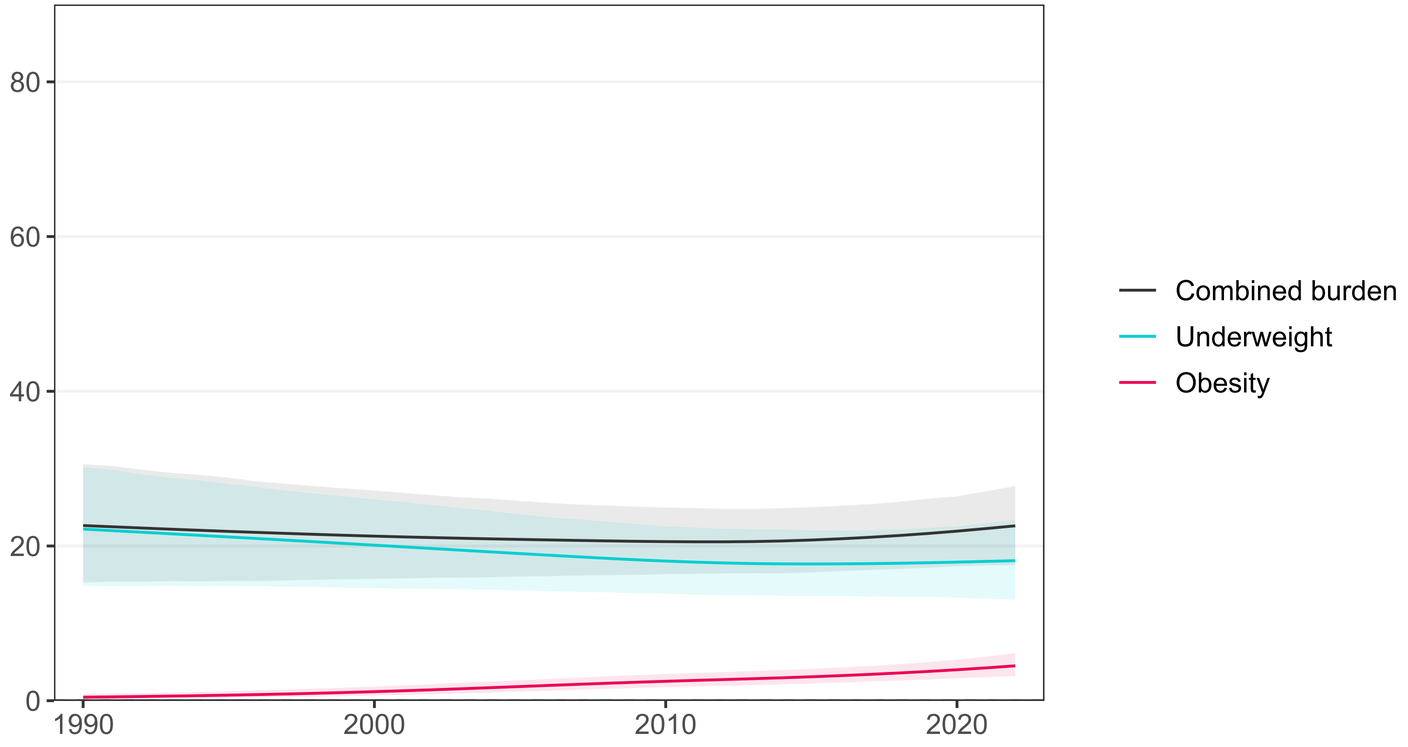

# Nigeria

## School-aged children and adolescents

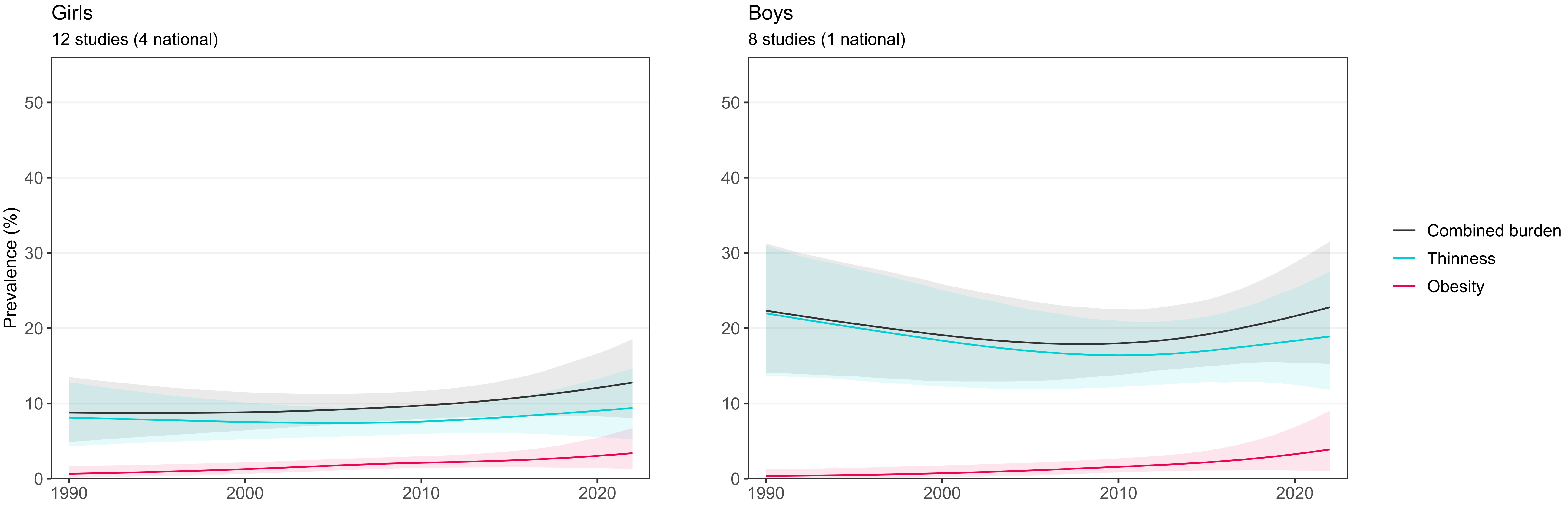


## Adults

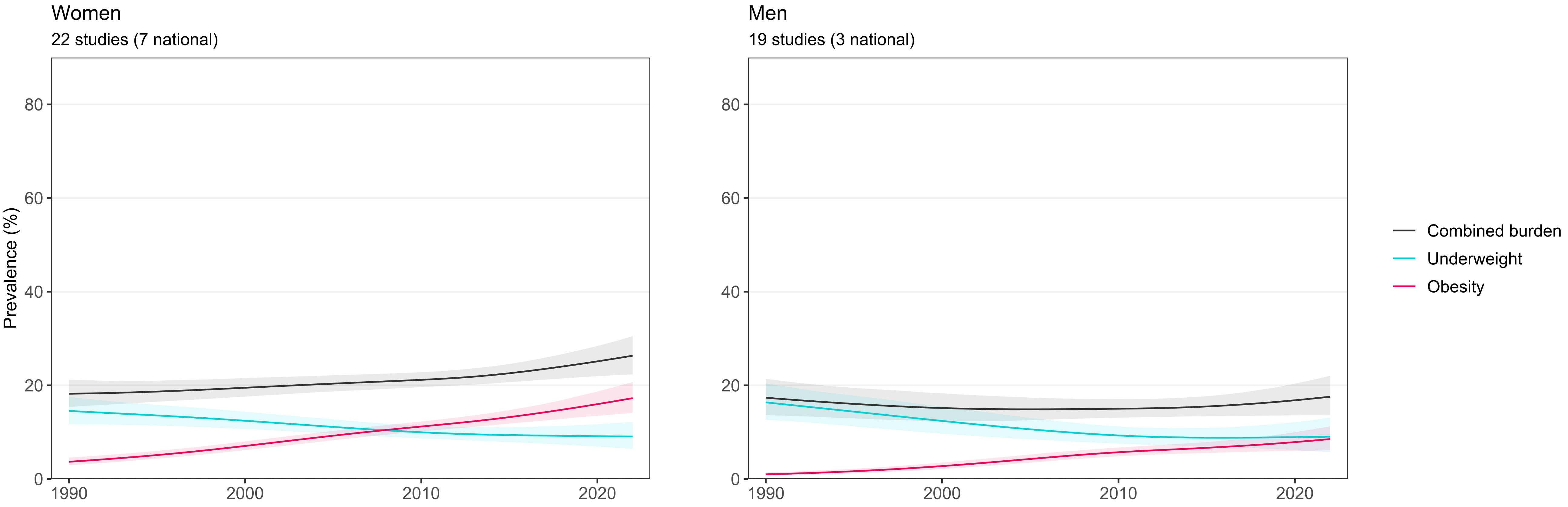

# Niue

## School-aged children and adolescents

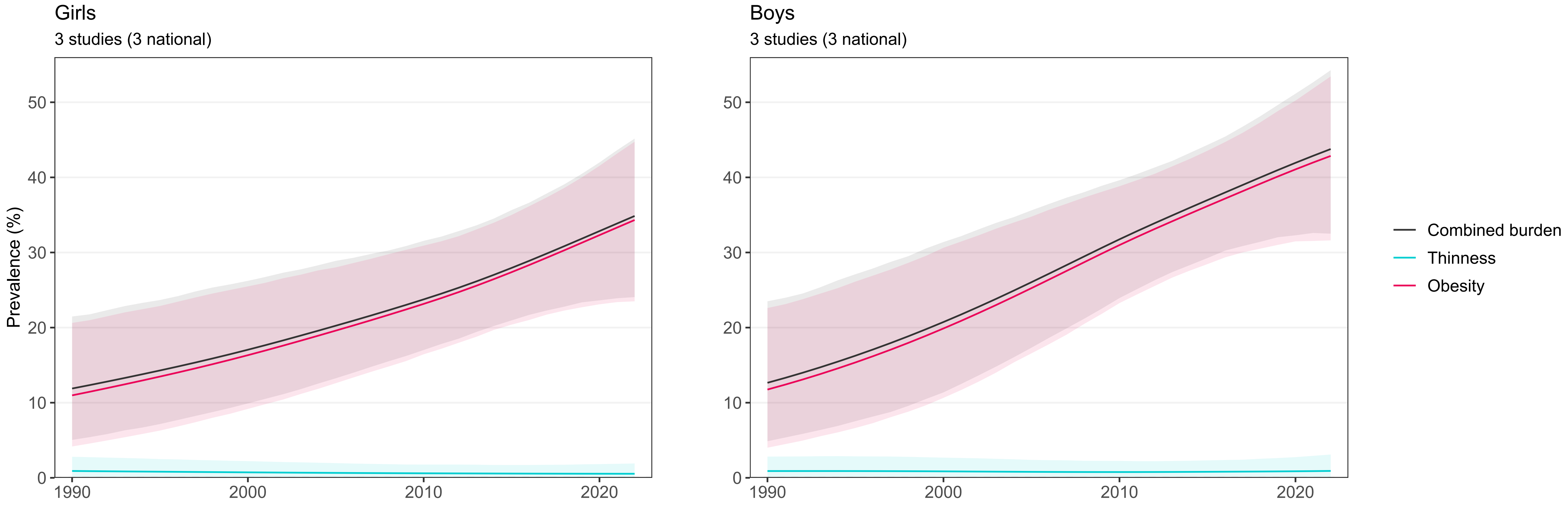


## Adults

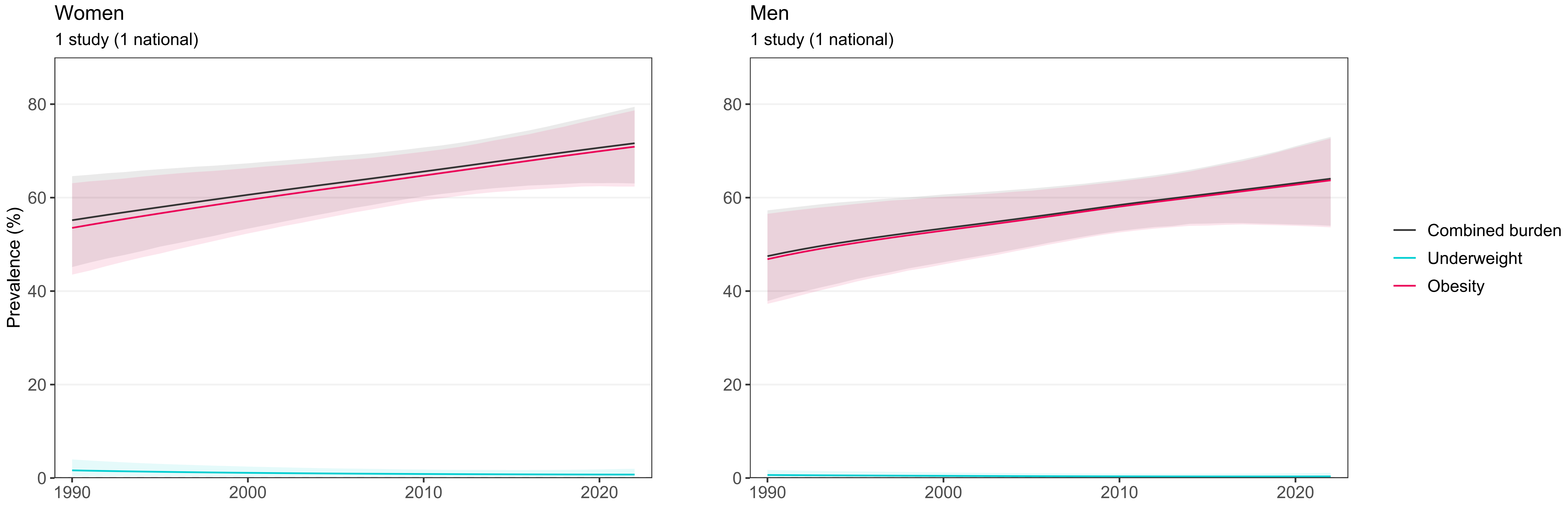

# North Korea

## School-aged children and adolescents

### Girls

No studies

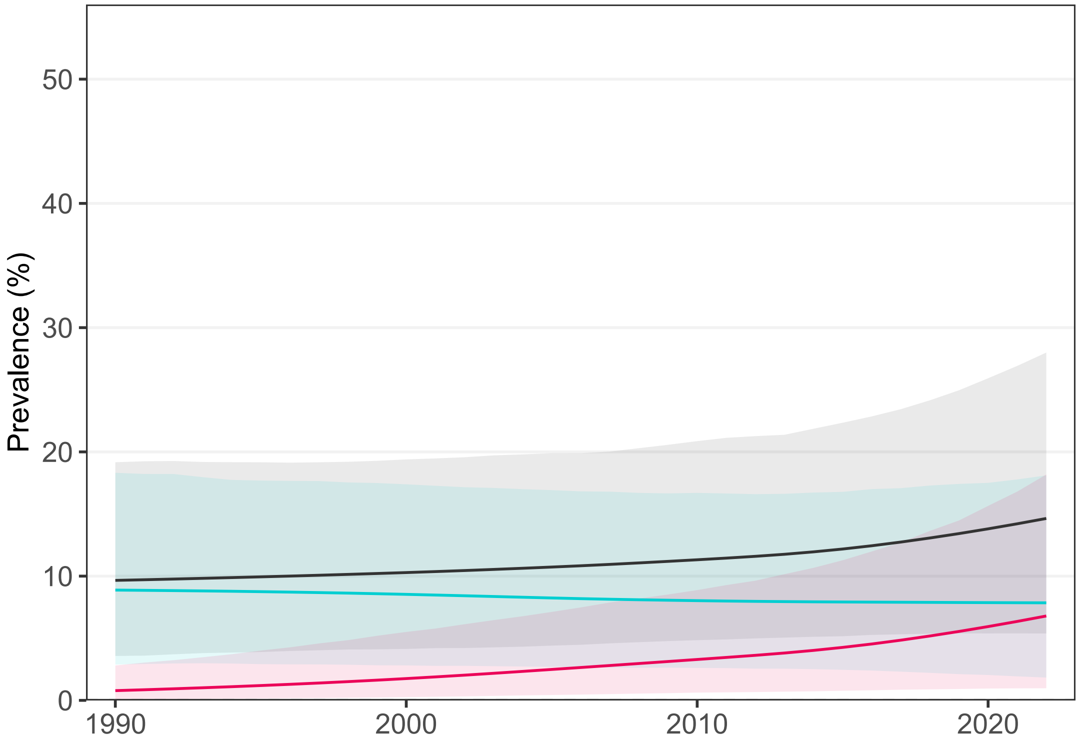


### Boys

No studies

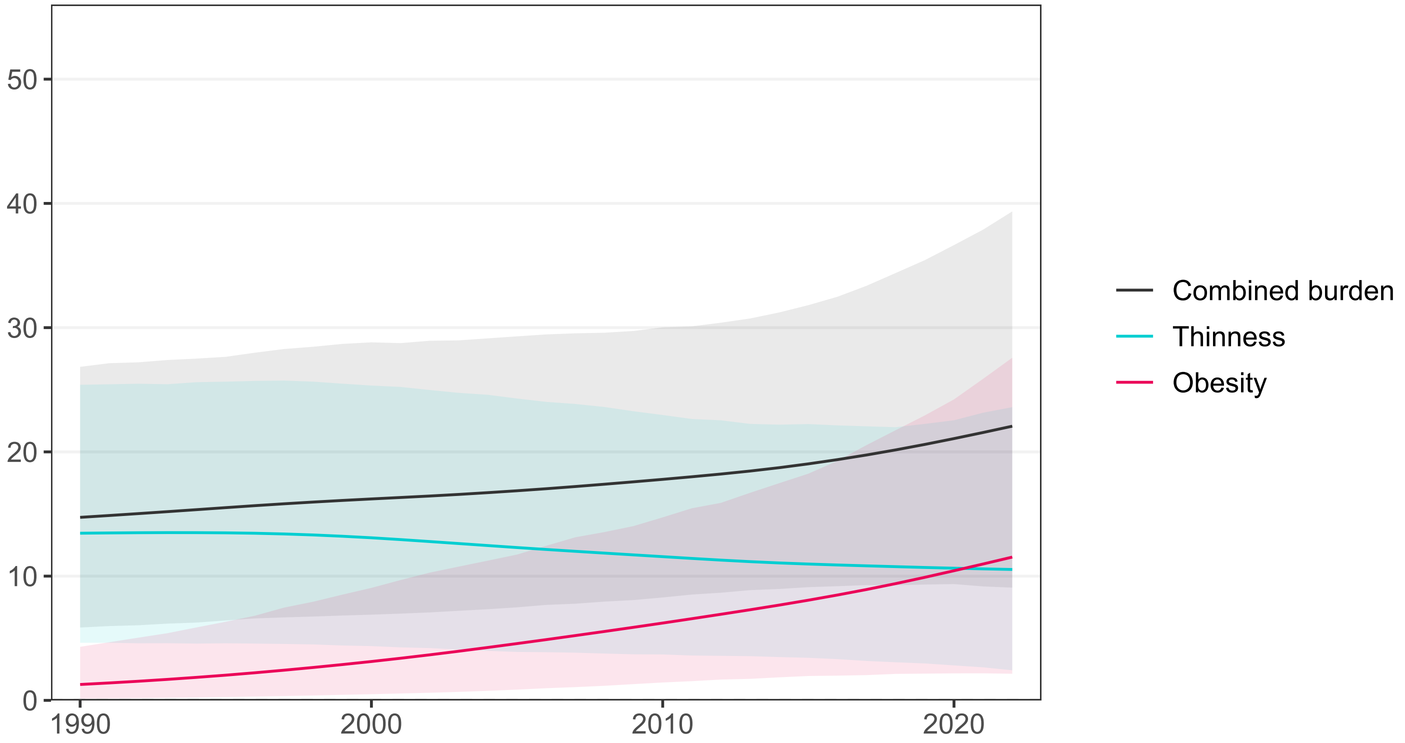


## Adults

### Women

No studies

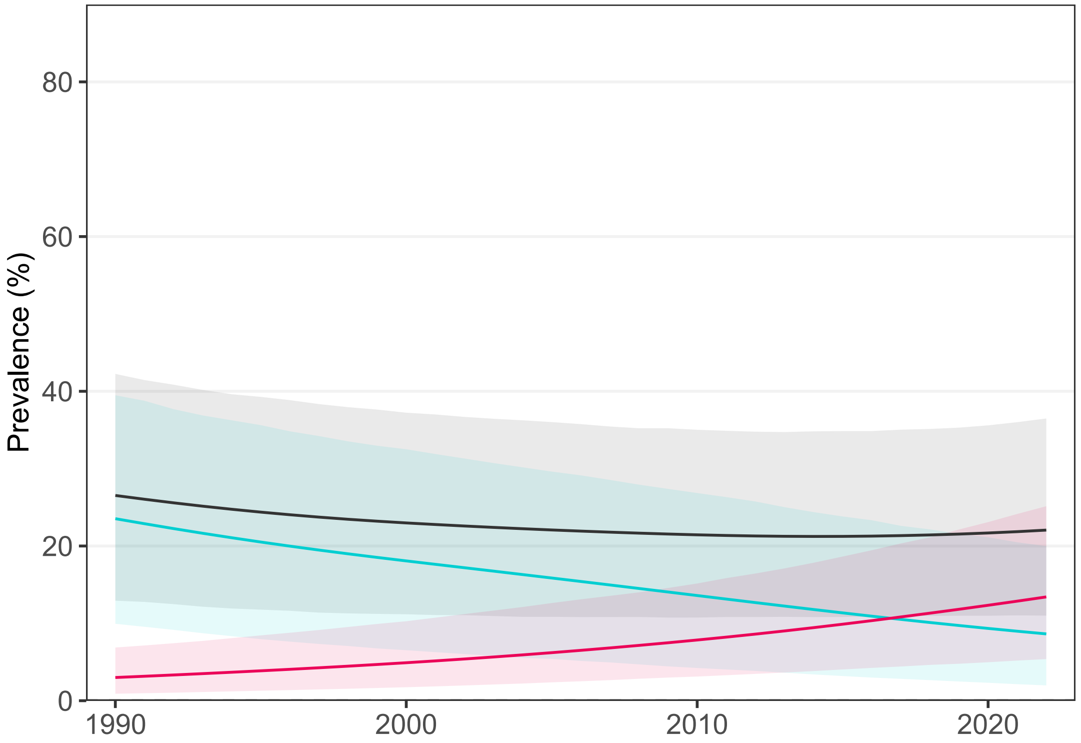


### Men

No studies

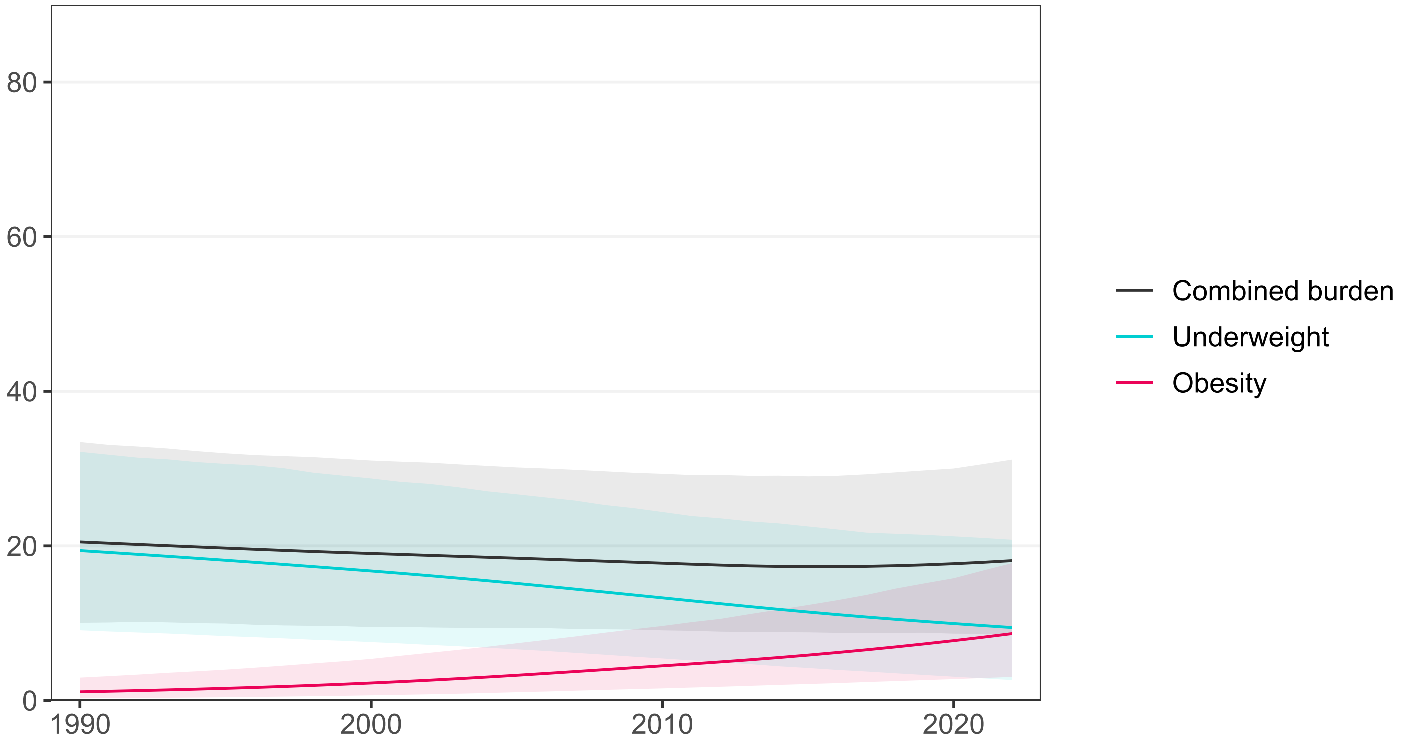

# North Macedonia

## School-aged children and adolescents

Girls
6 studies (6 national)

Boys
5 studies (5 national)

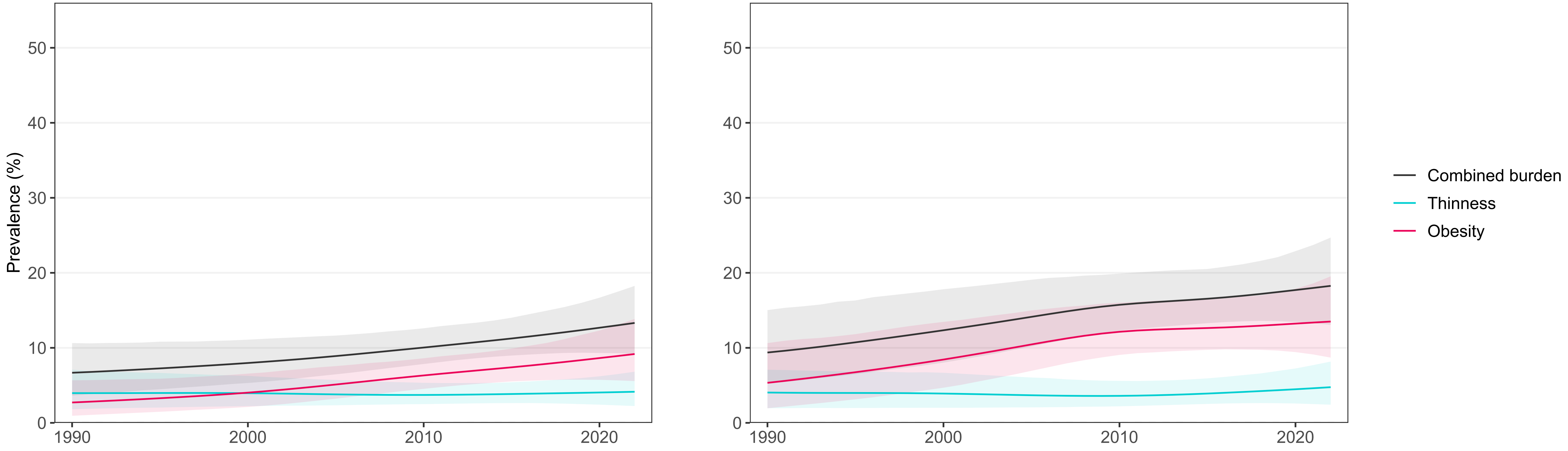


## Adults

Women
1 study (1 national)

Men
No studies

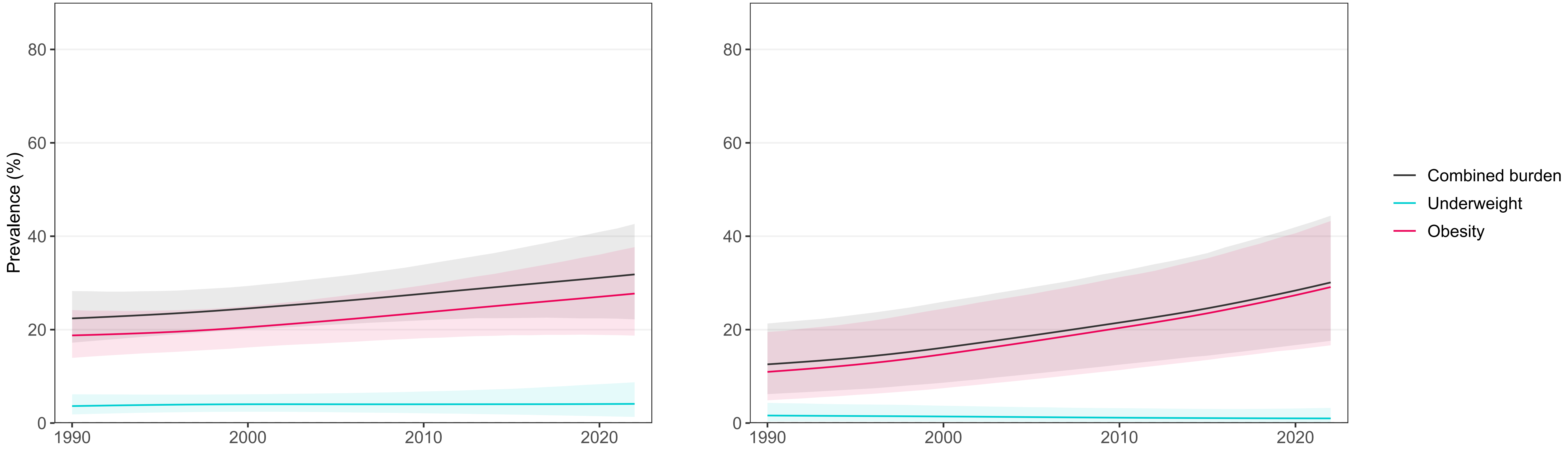

# Norway

## School-aged children and adolescents

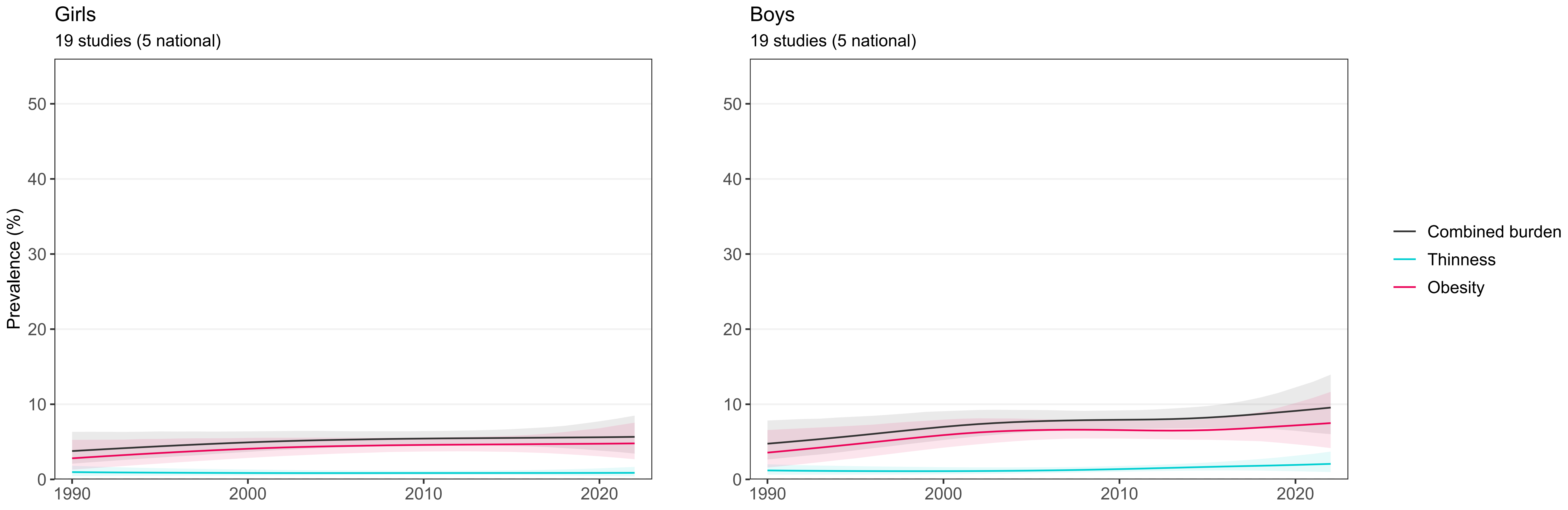


## Adults

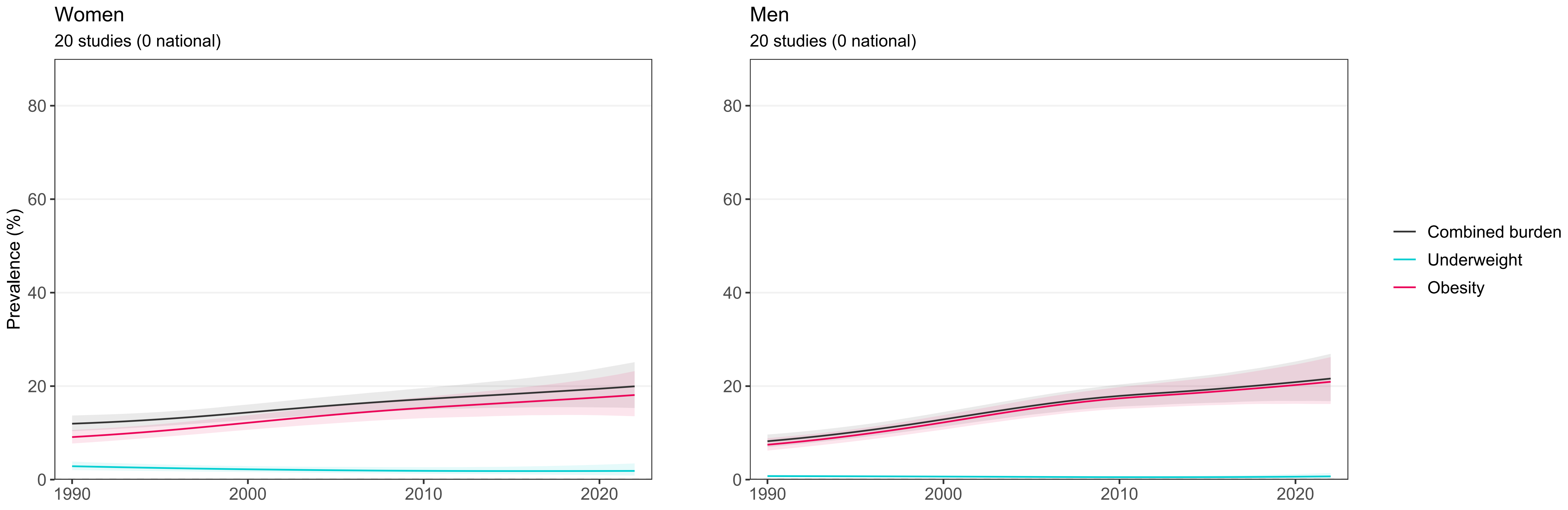

# Oman

## School-aged children and adolescents

### Girls

4 studies (4 national)

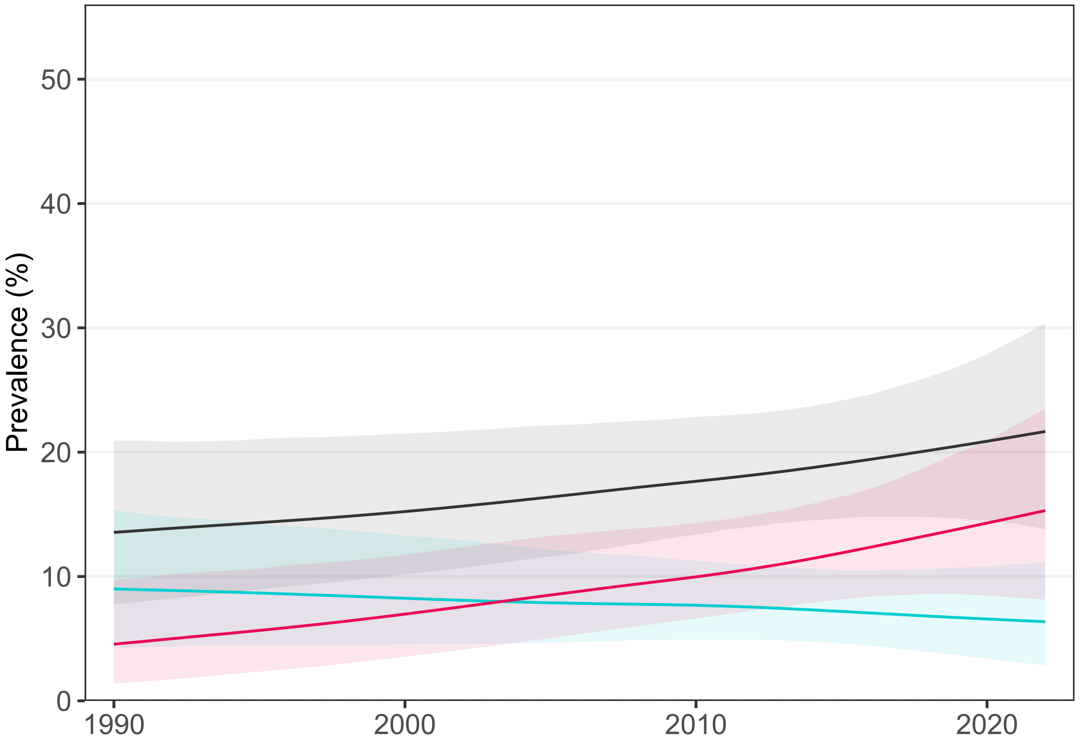


### Boys

4 studies (4 national)

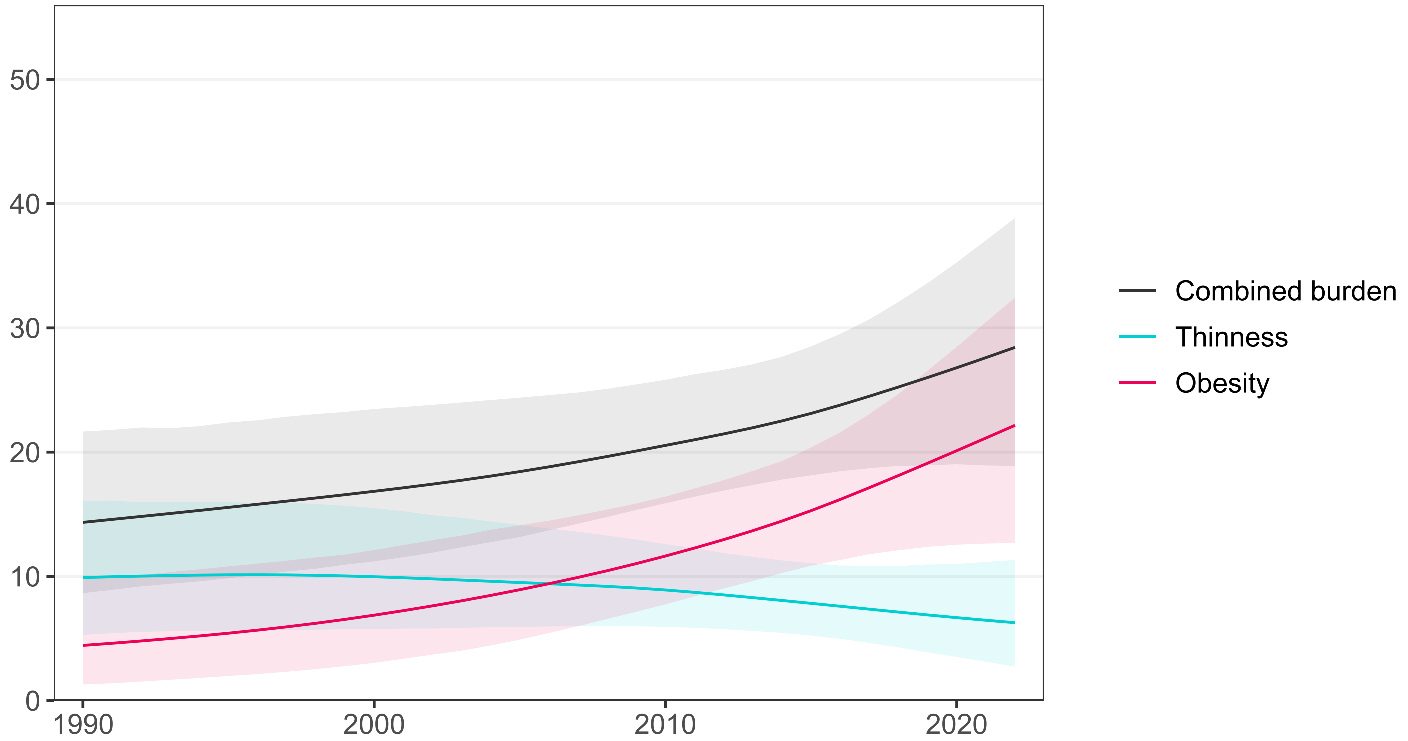


## Adults

### Women

6 studies (4 national)

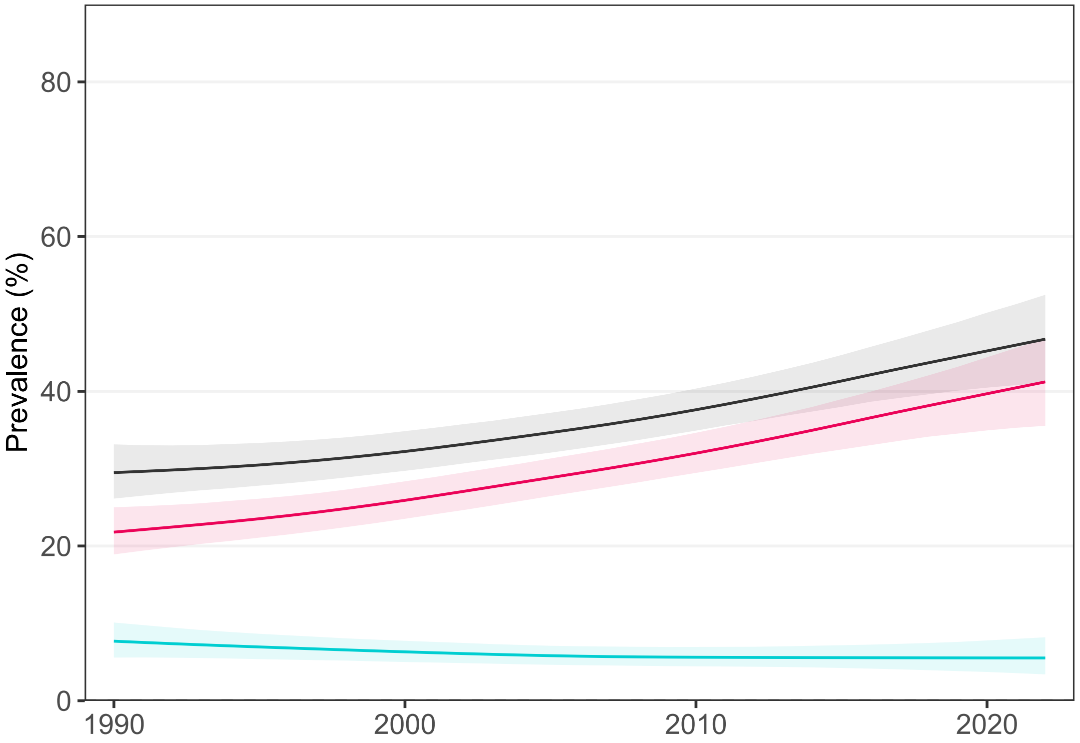


### Men

6 studies (4 national)

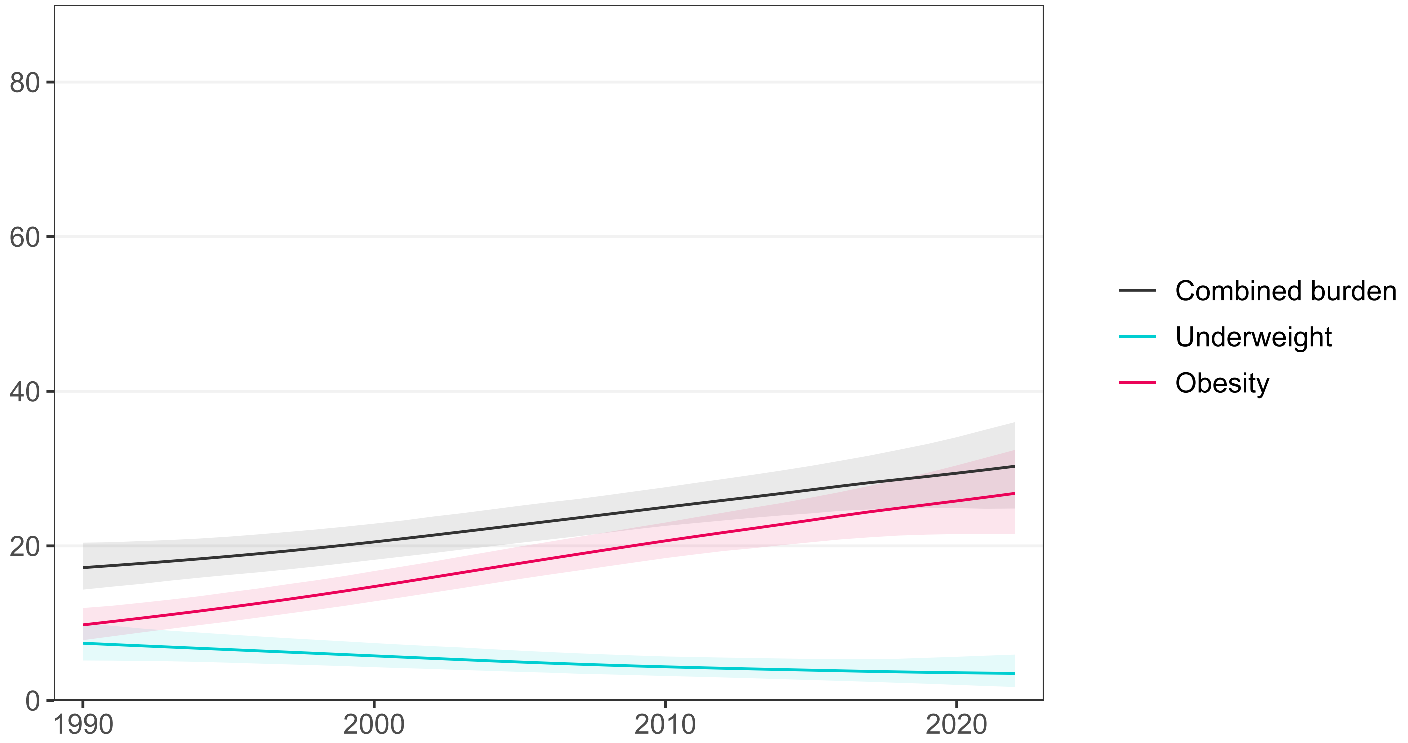

# Pakistan

## School-aged children and adolescents

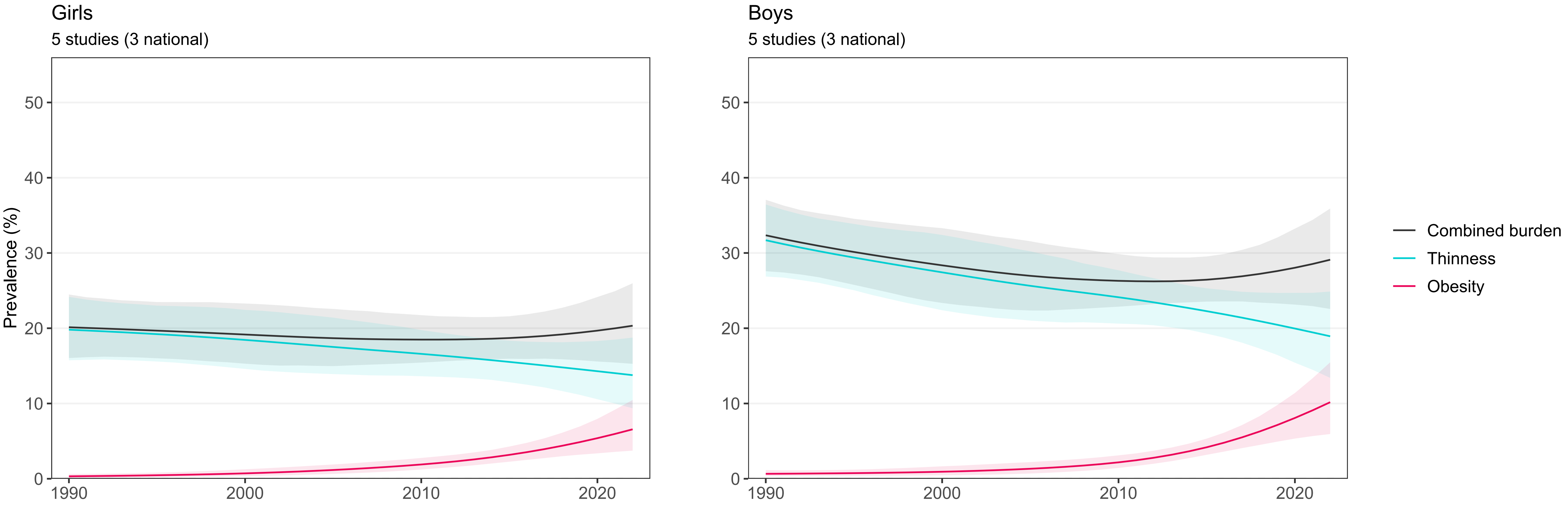


## Adults

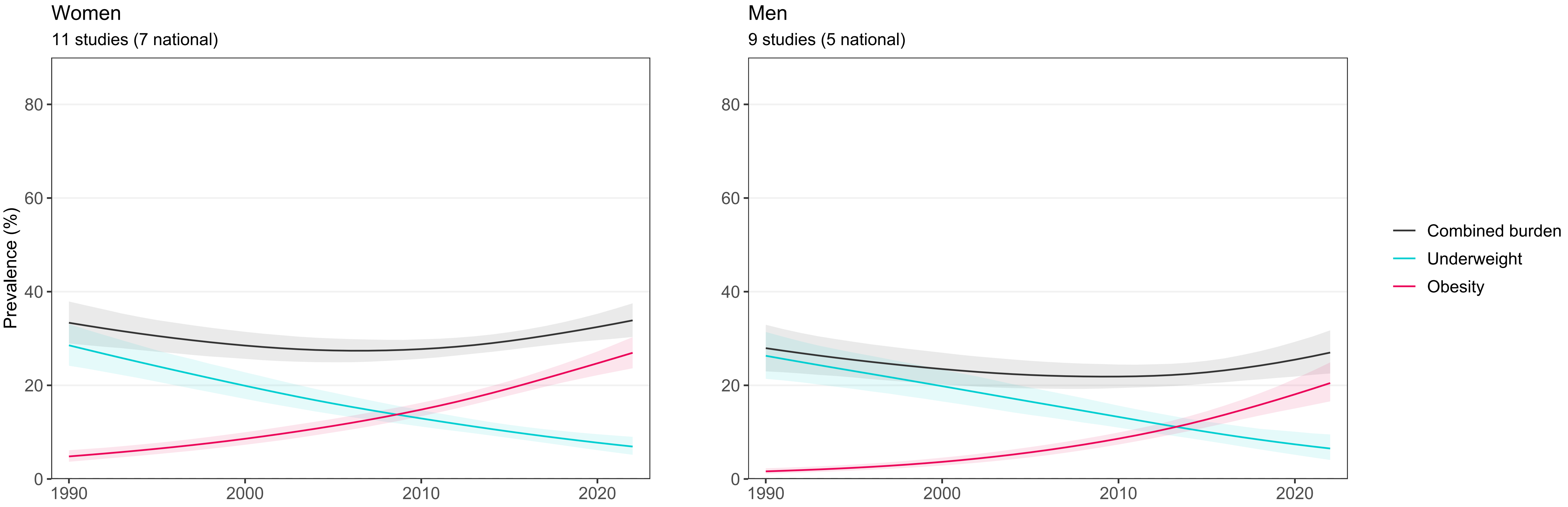

# Palau

## School-aged children and adolescents

Girls
14 studies (14 national)

Boys
14 studies (14 national)

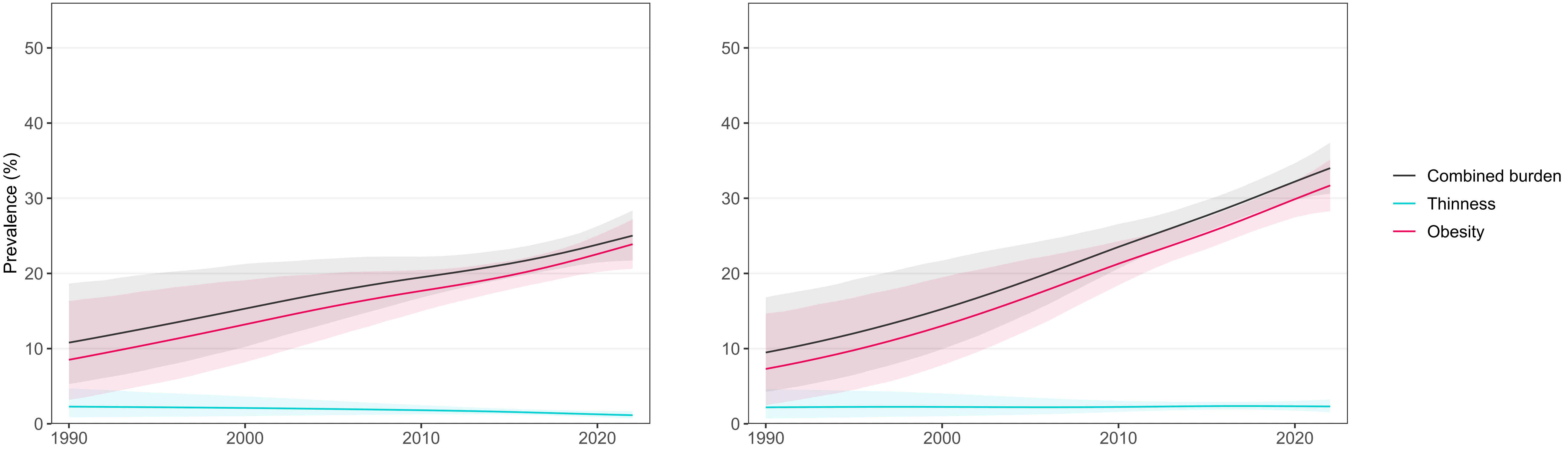


## Adults

Women
2 studies (2 national)

Men
2 studies (2 national)

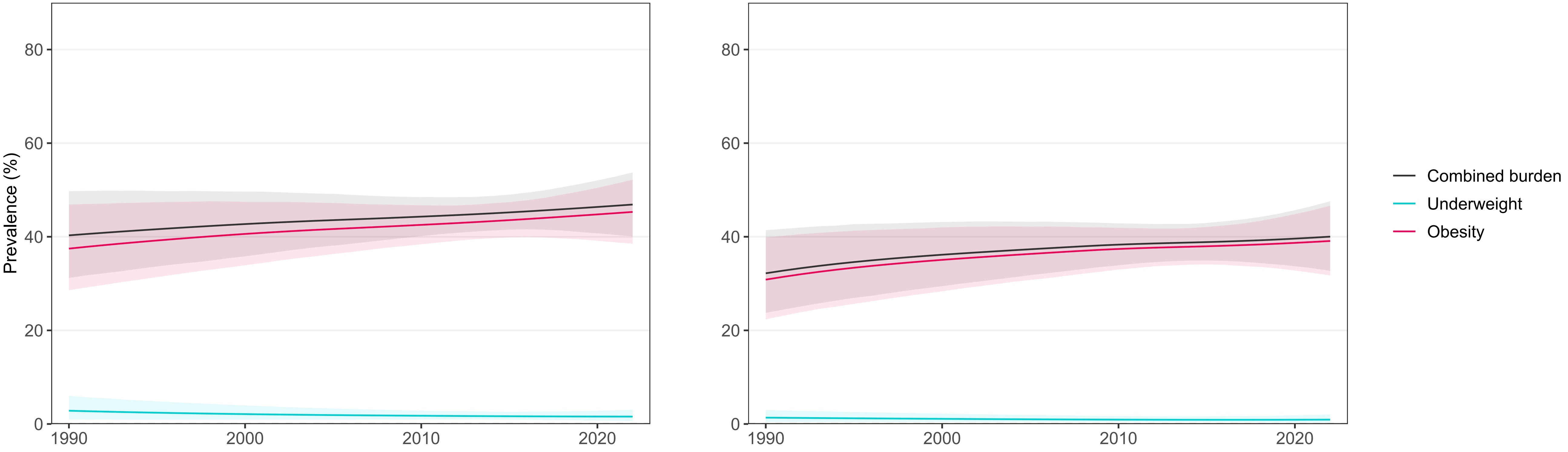

# Panama

## School-aged children and adolescents

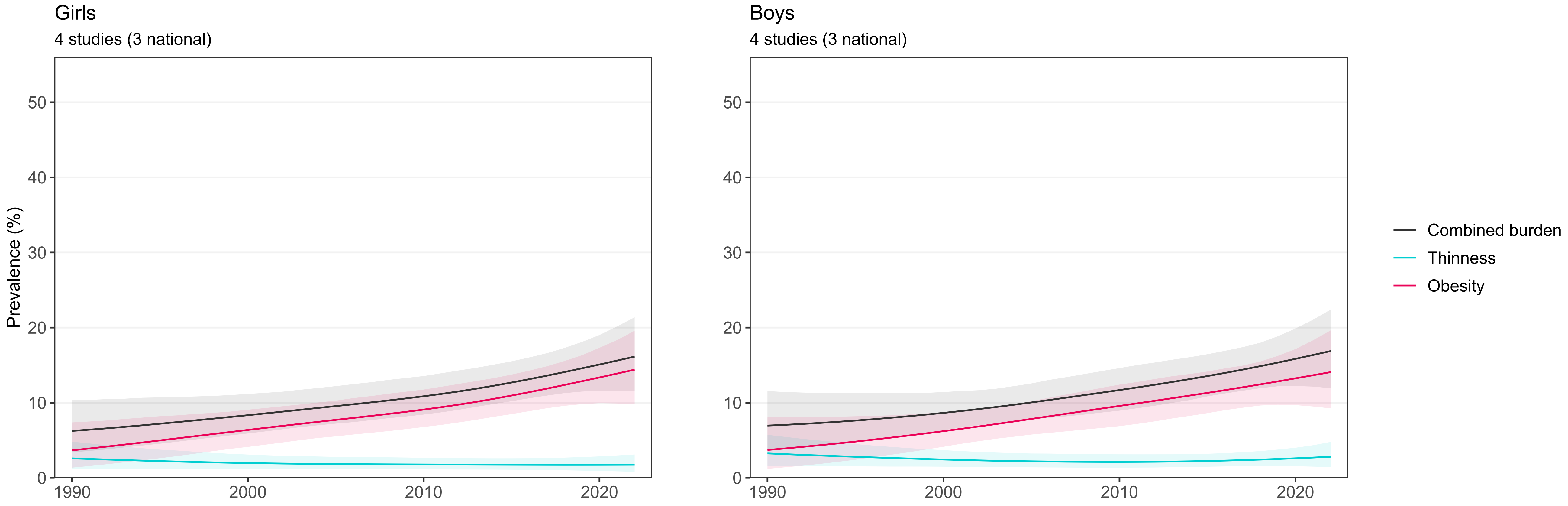


## Adults

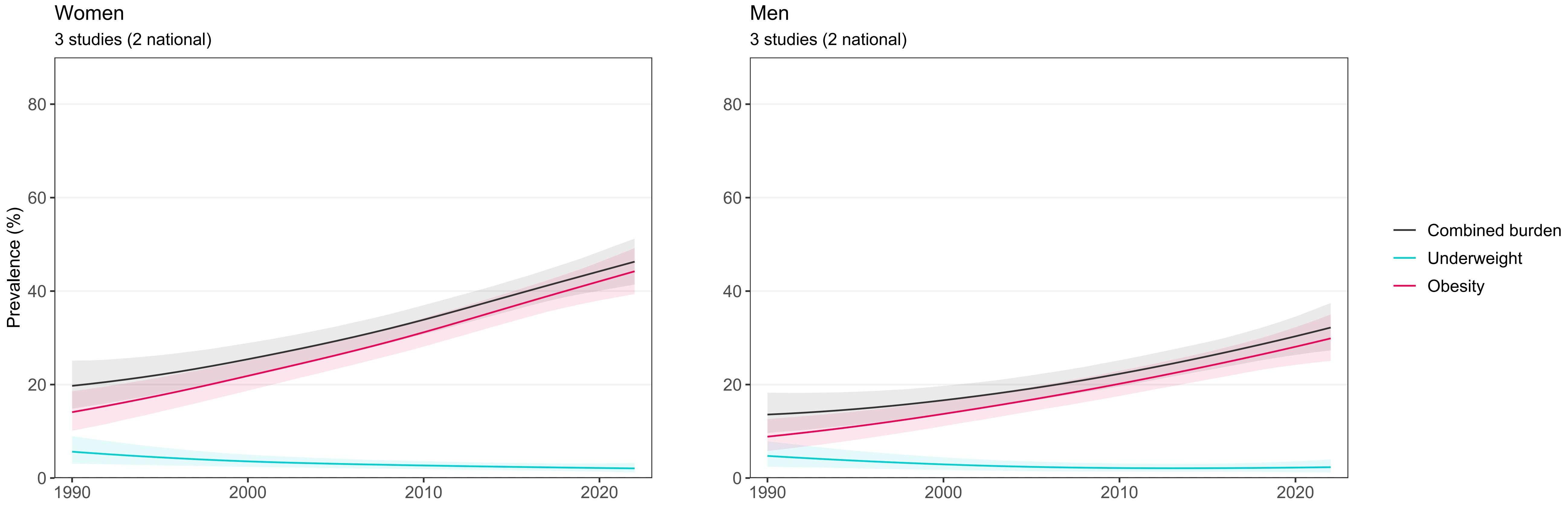

# Papua New Guinea

## School-aged children and adolescents

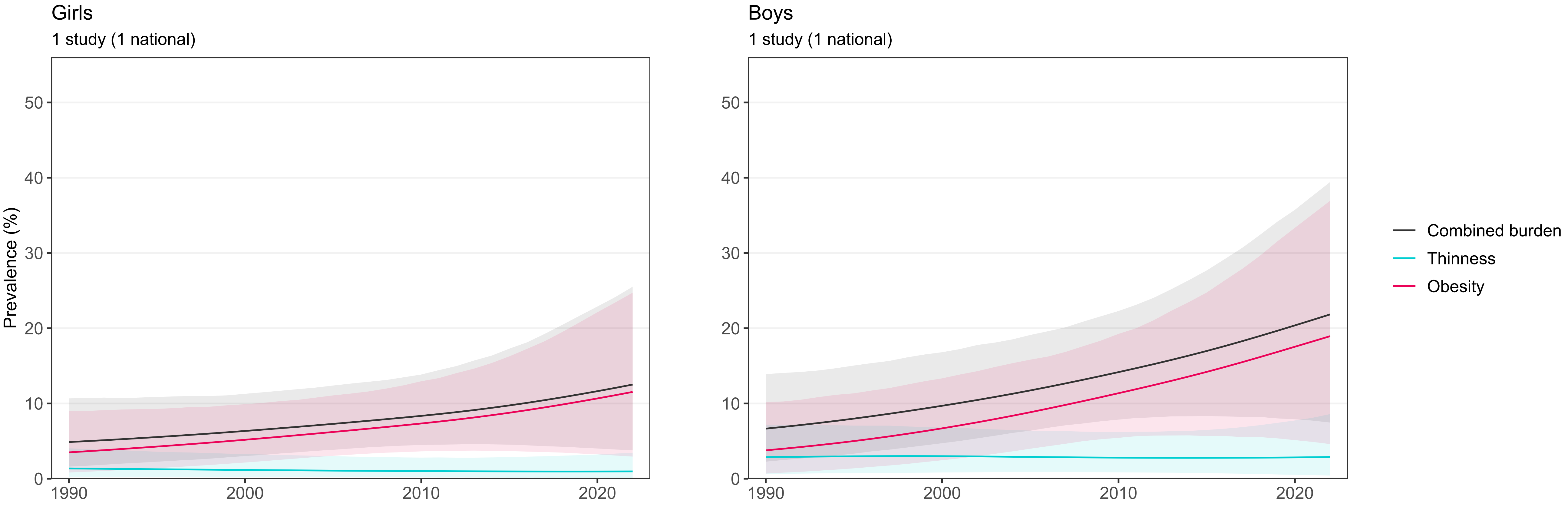


## Adults

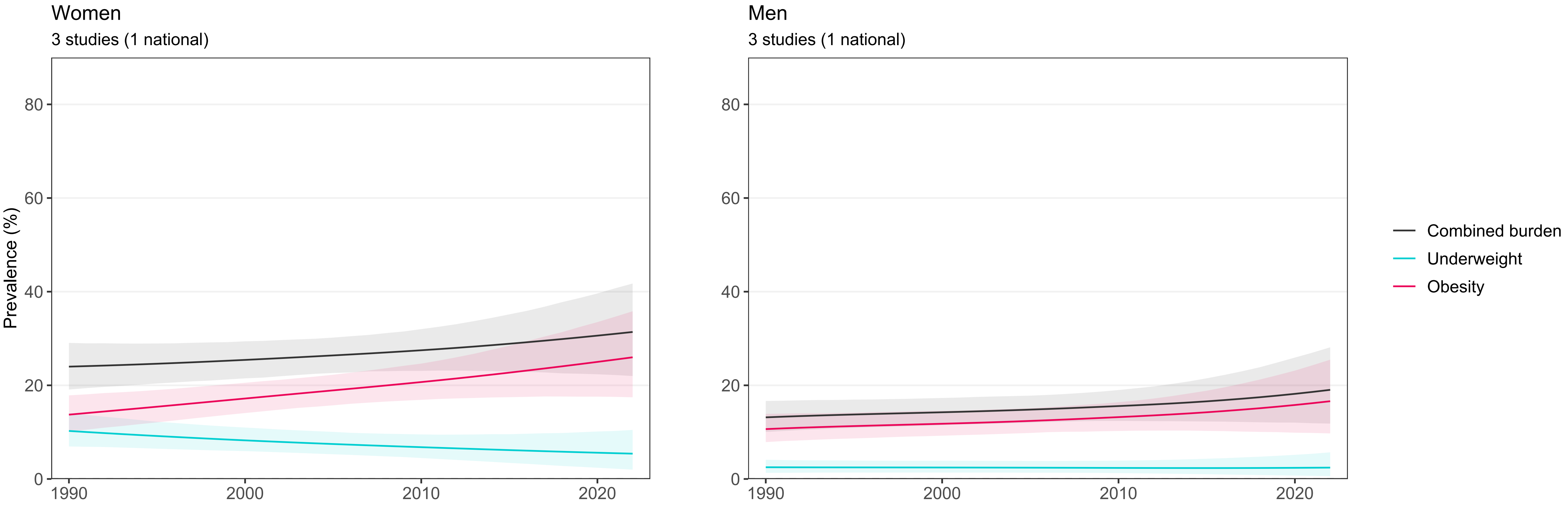

# Paraguay

## School-aged children and adolescents

Girls
2 studies (2 national)

Boys
2 studies (2 national)

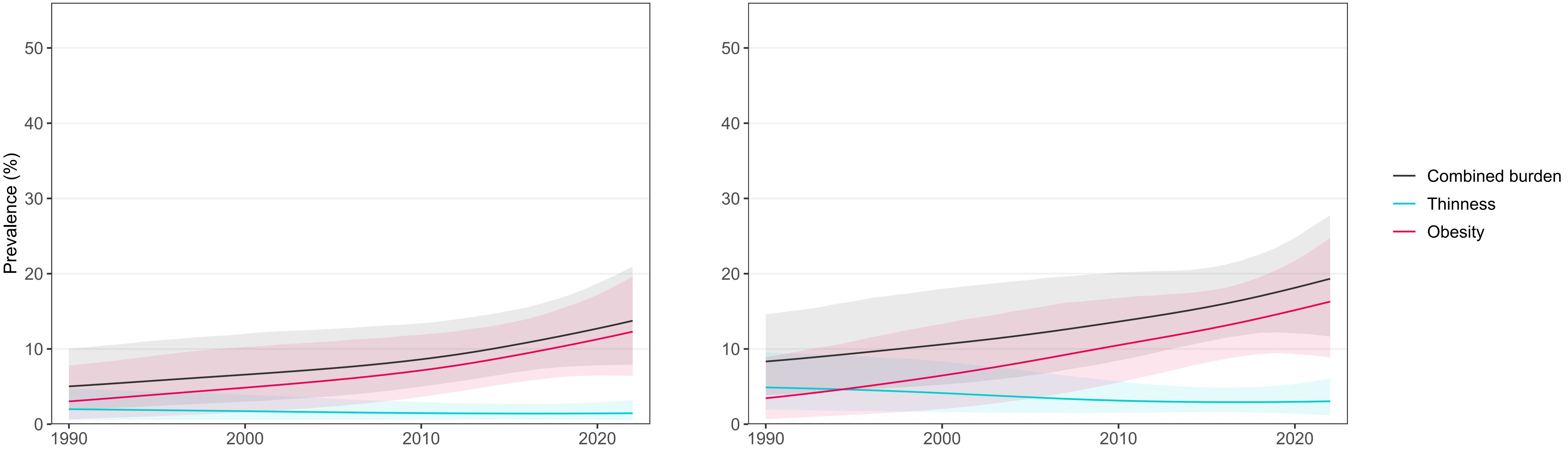


## Adults

Women
1 study (1 national)

Men
1 study (1 national)

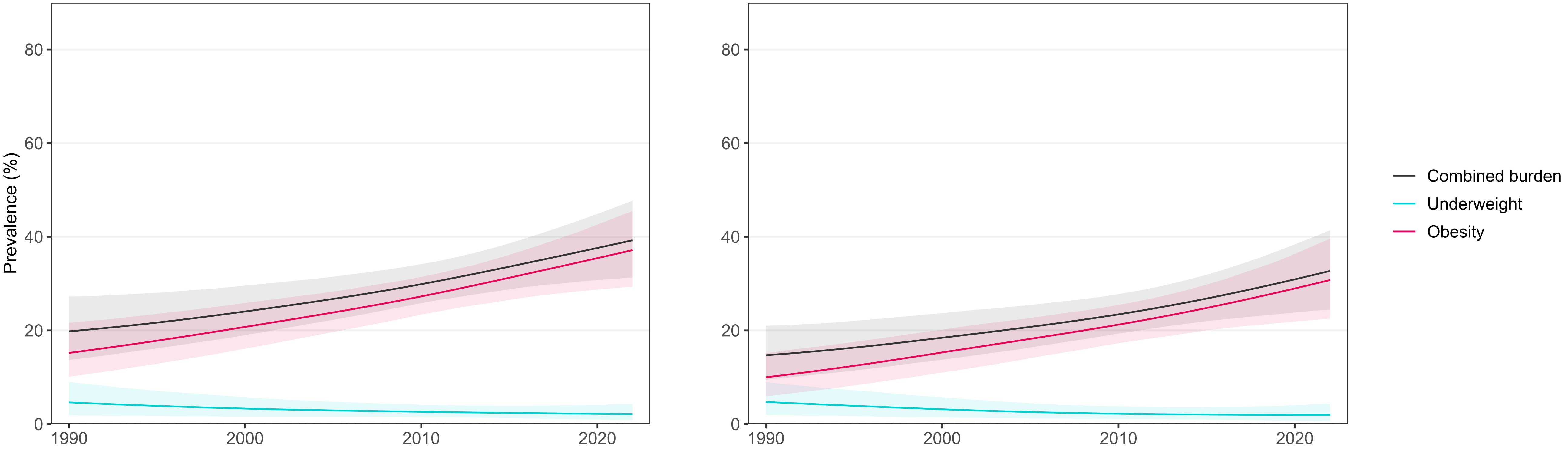

# Peru

## School-aged children and adolescents

### Girls
29 studies (24 national)

### Boys
21 studies (16 national)

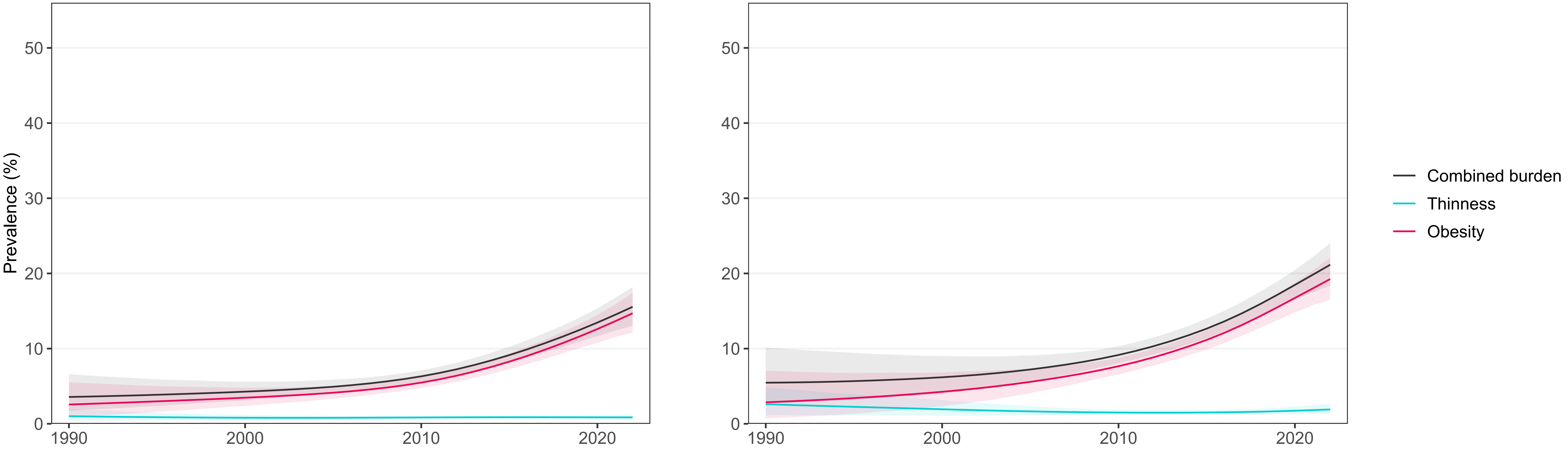


## Adults

### Women
40 studies (26 national)

### Men
30 studies (16 national)

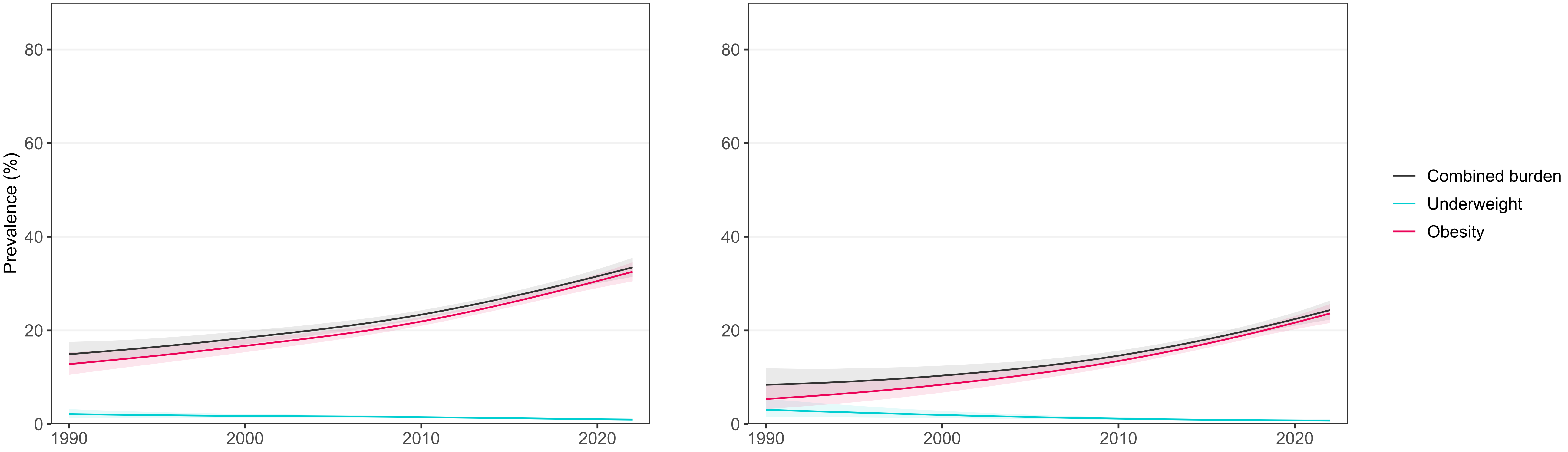

# Philippines

## School-aged children and adolescents

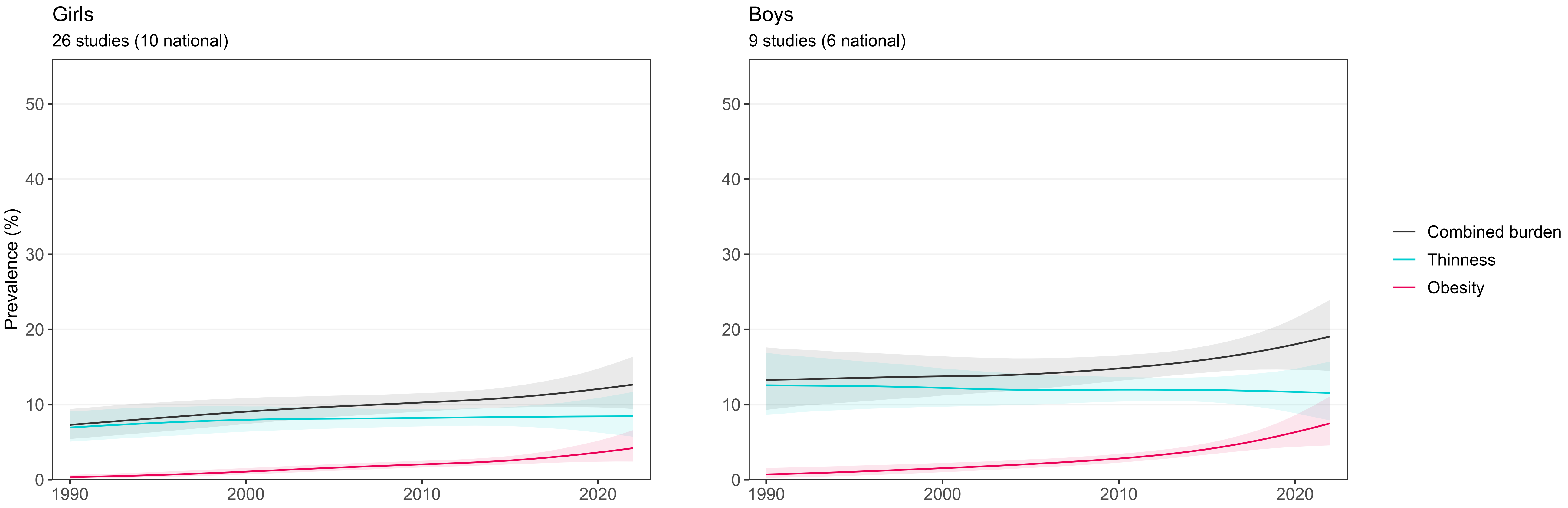


## Adults

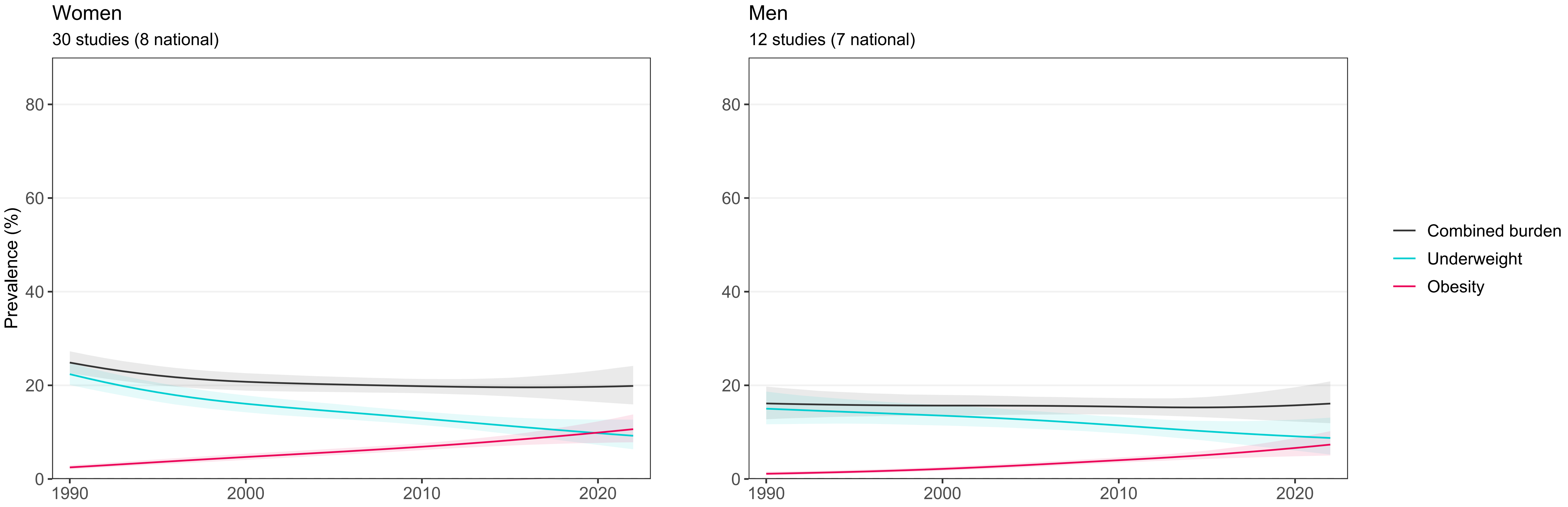

# Poland

## School-aged children and adolescents

### Girls
31 studies (9 national)

### Boys
47 studies (13 national)

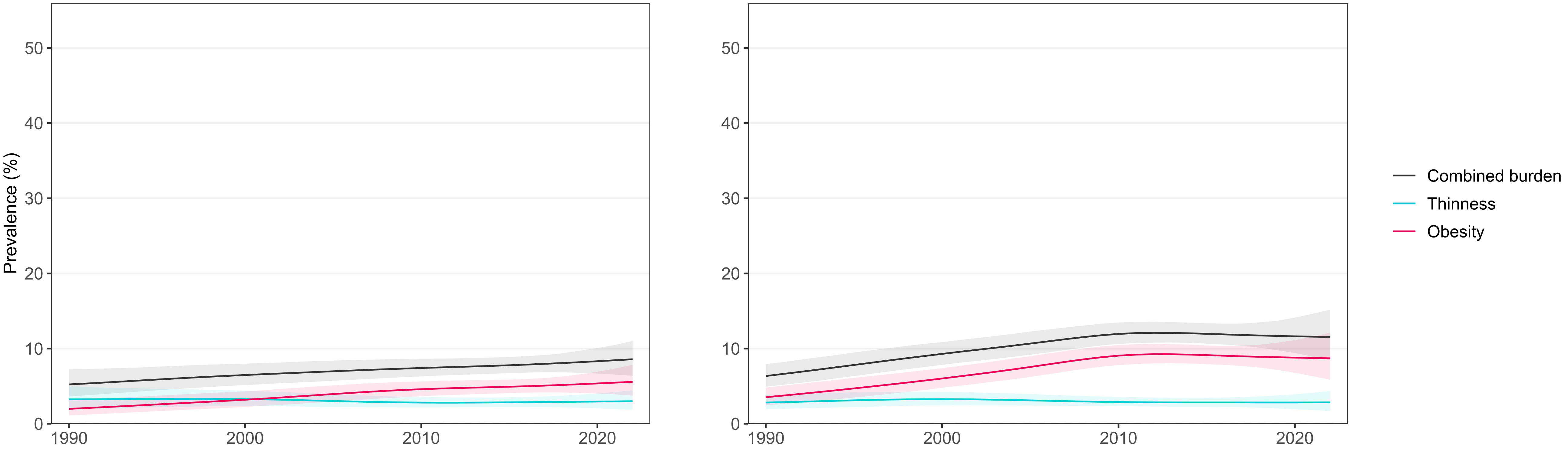


## Adults

### Women
35 studies (11 national)

### Men
53 studies (15 national)

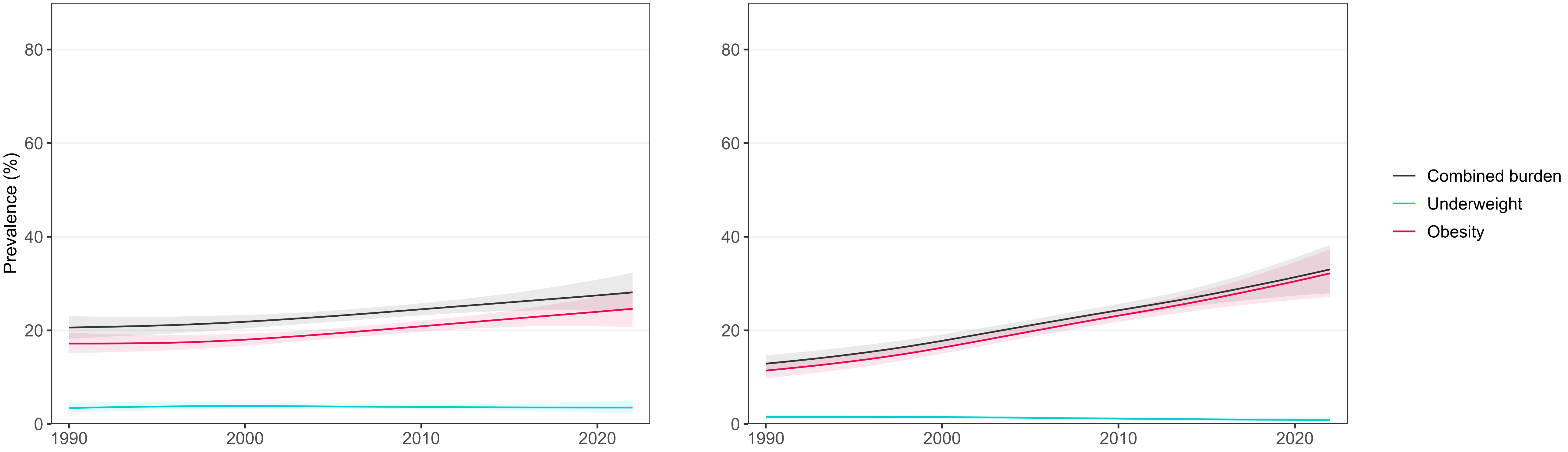

# Portugal

## School-aged children and adolescents

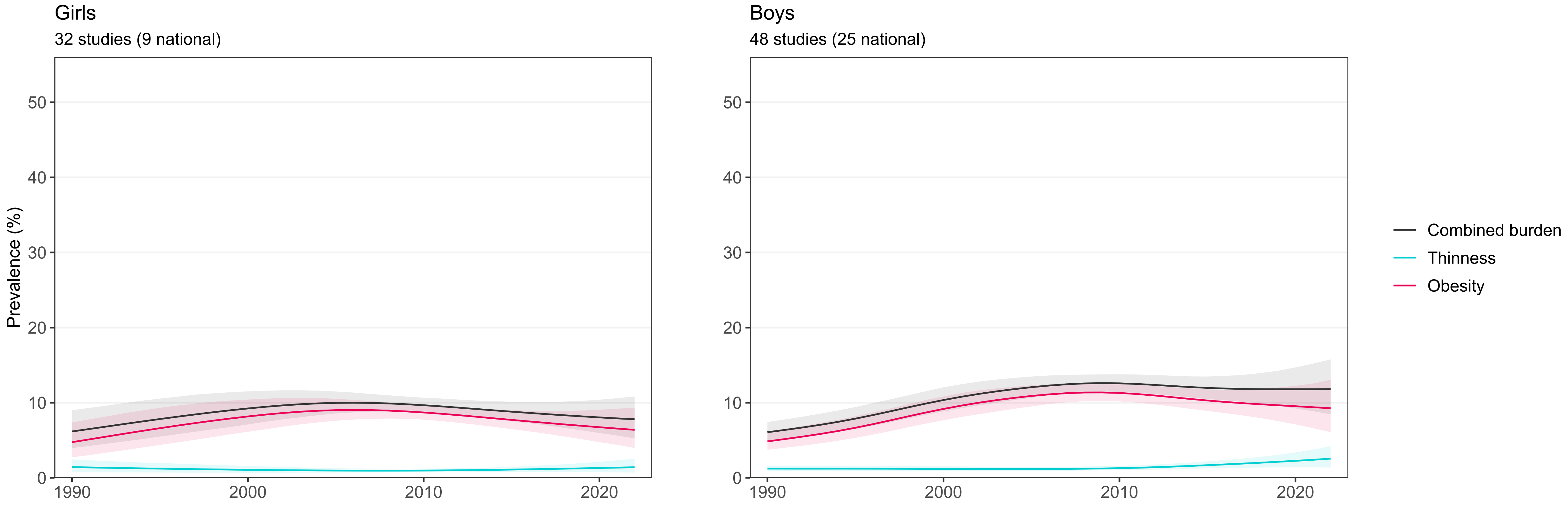


## Adults

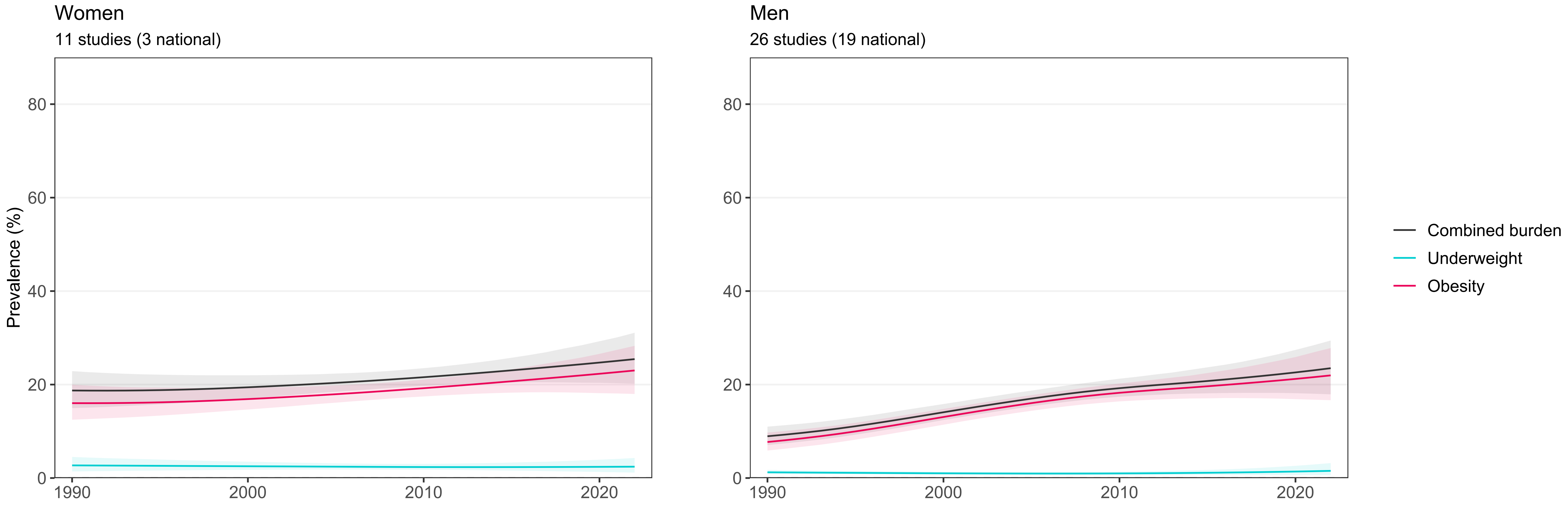

# Puerto Rico

## School-aged children and adolescents

### Girls

1 study (0 national)

### Boys

No studies

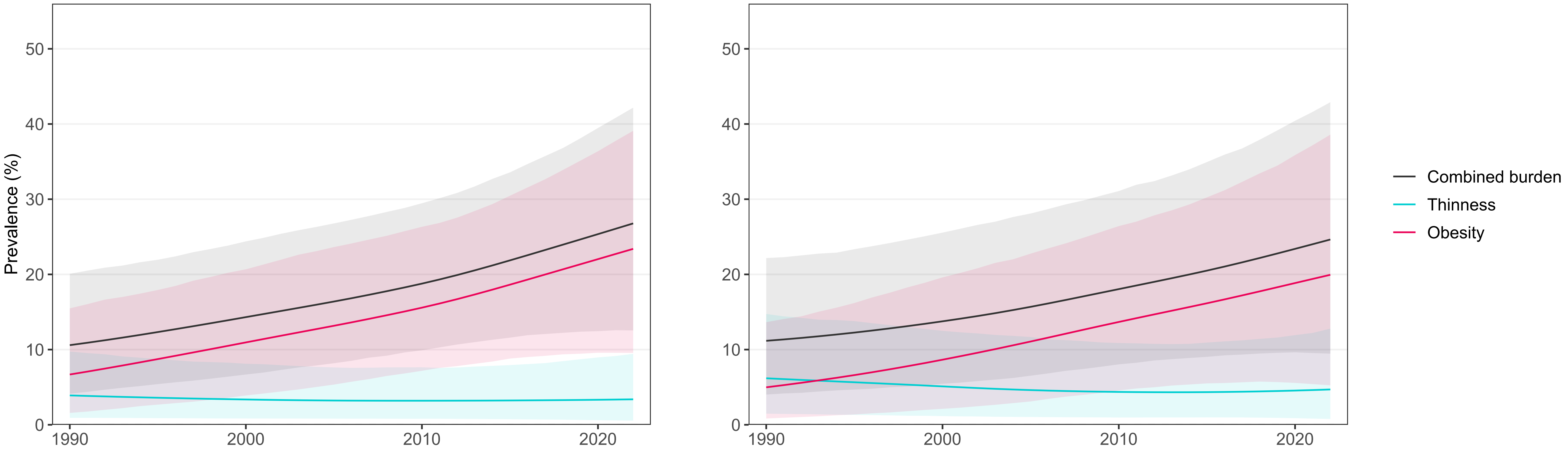


## Adults

### Women

4 studies (2 national)

### Men

3 studies (2 national)

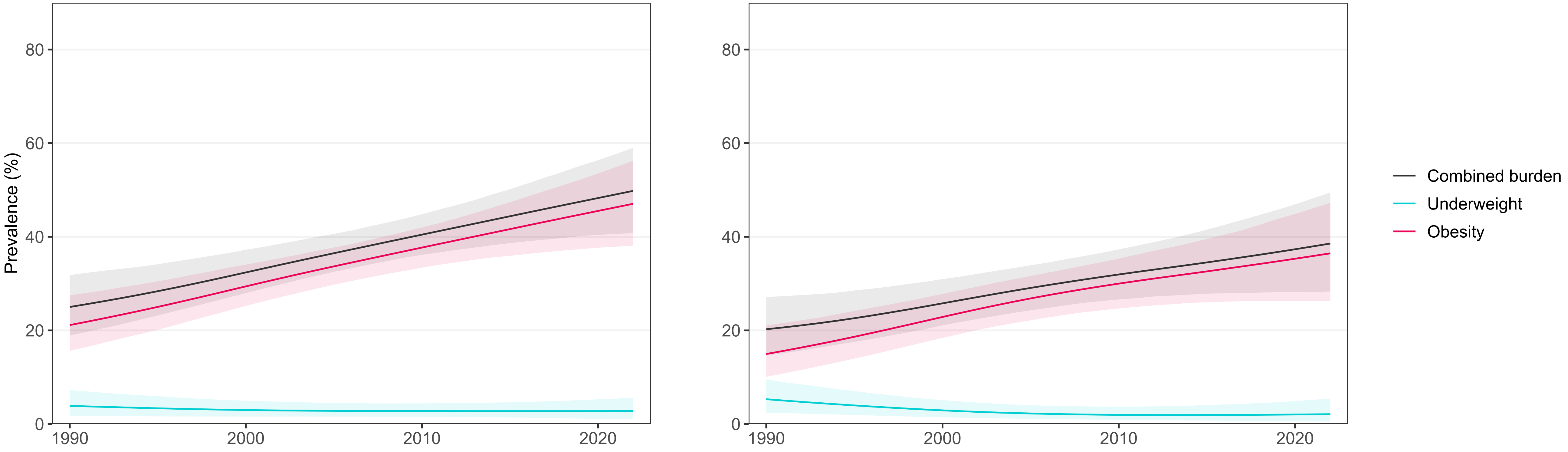

# Qatar

## School-aged children and adolescents

### Girls
3 studies (3 national)

### Boys
3 studies (3 national)

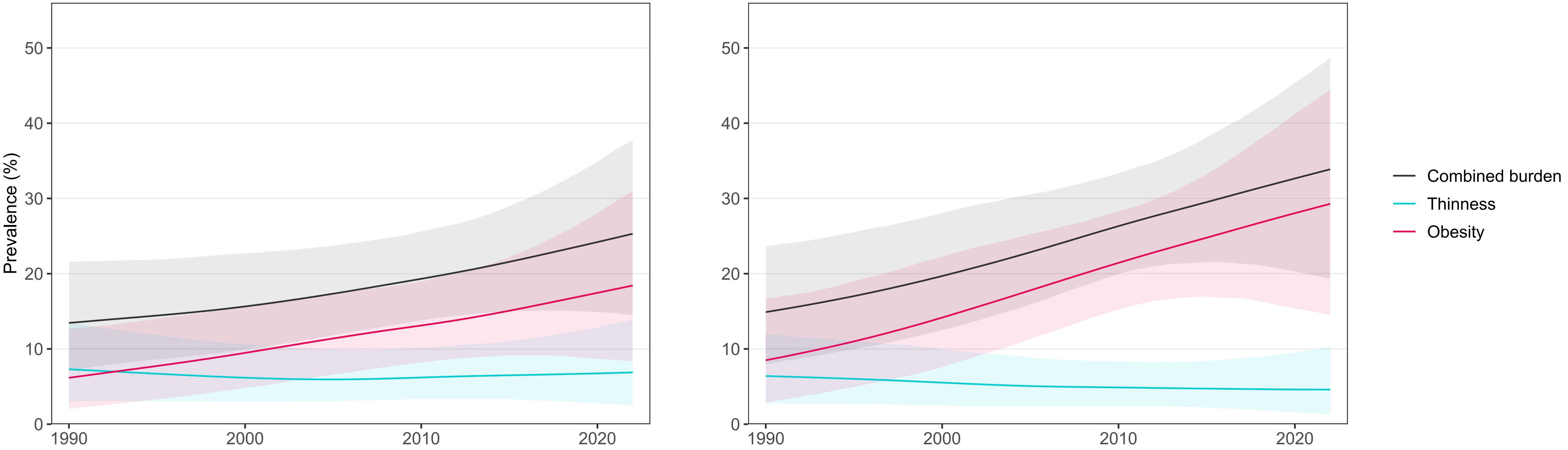


## Adults

### Women
2 studies (2 national)

### Men
2 studies (2 national)

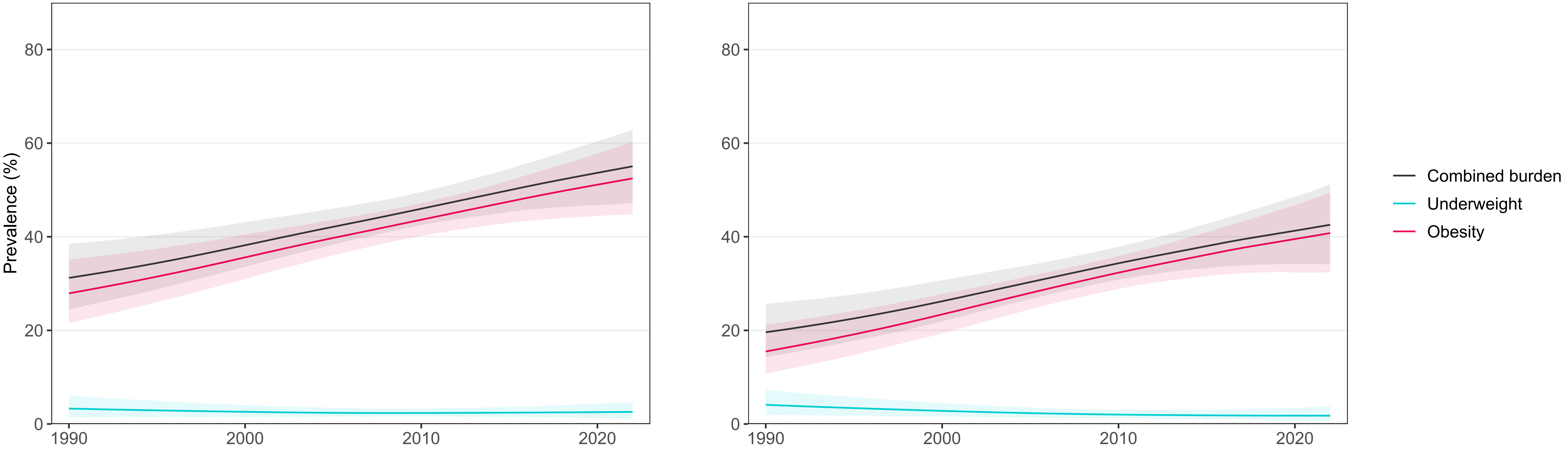

# Romania

## School-aged children and adolescents

Girls
31 studies (7 national)

Boys
31 studies (7 national)

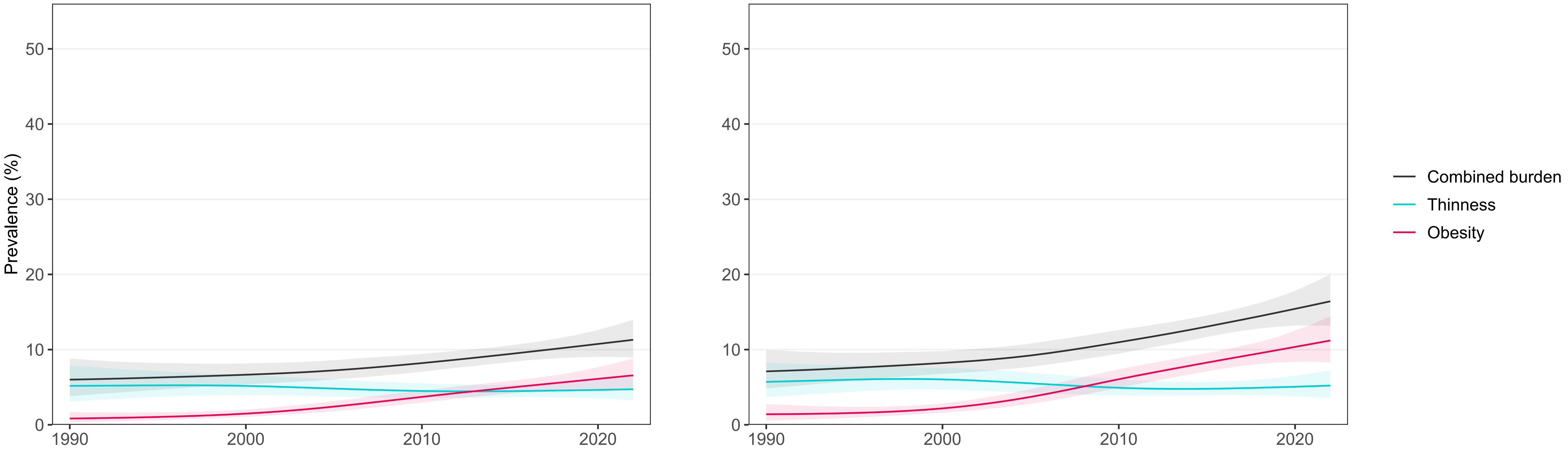


## Adults

Women
9 studies (6 national)

Men
9 studies (6 national)

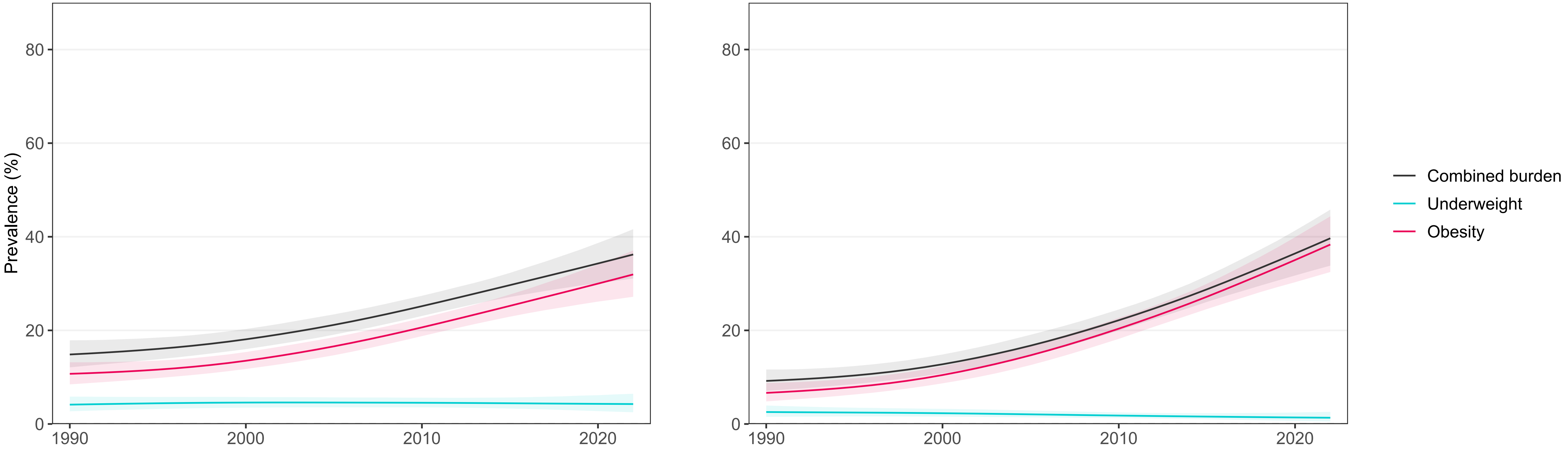

# Russian Federation

## School-aged children and adolescents

### Girls

28 studies (13 national)

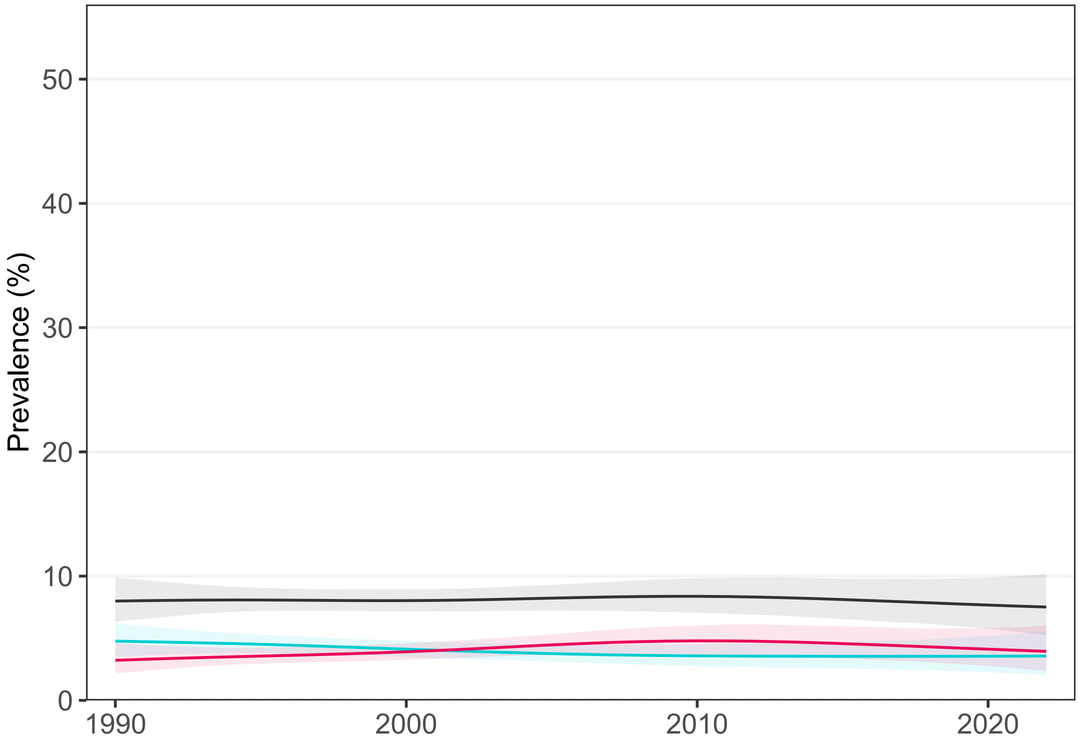


### Boys

28 studies (13 national)

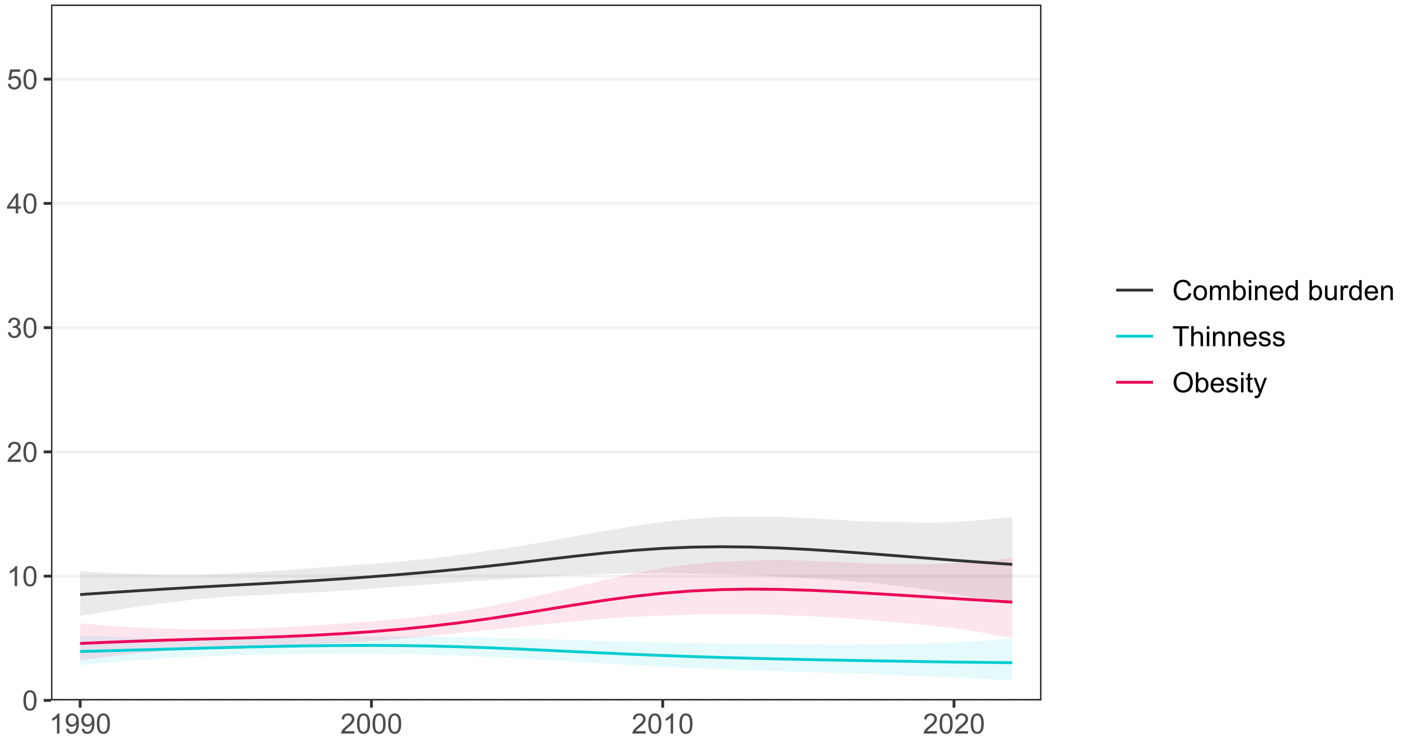


## Adults

### Women

41 studies (15 national)

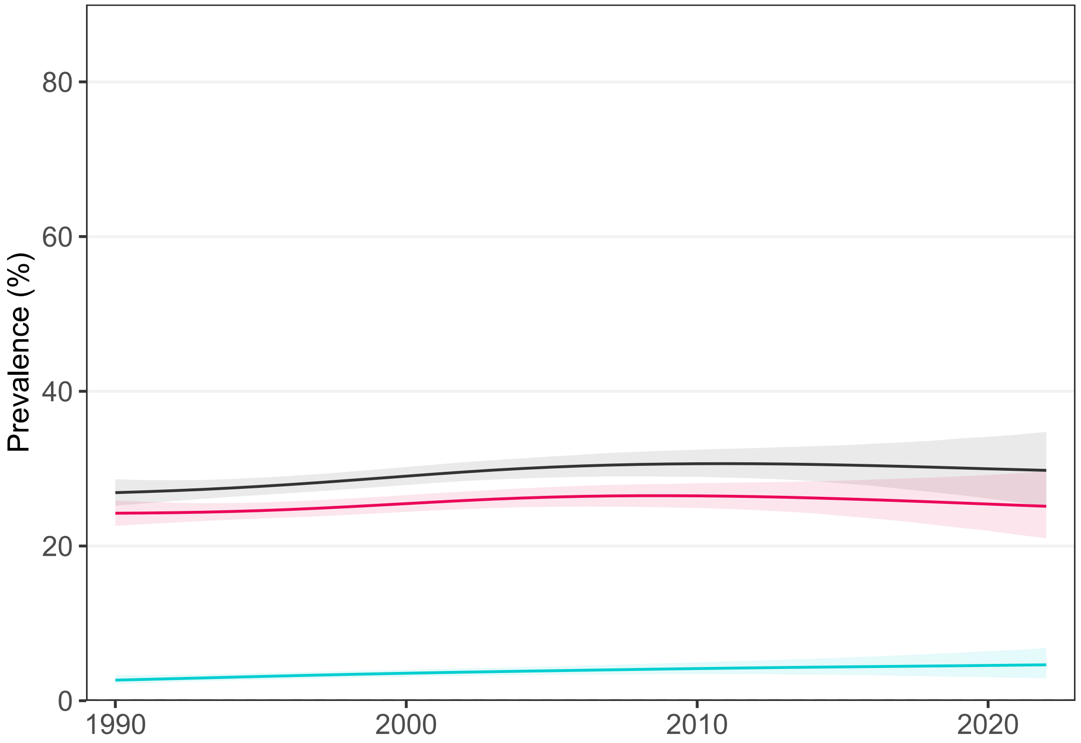


### Men

42 studies (15 national)

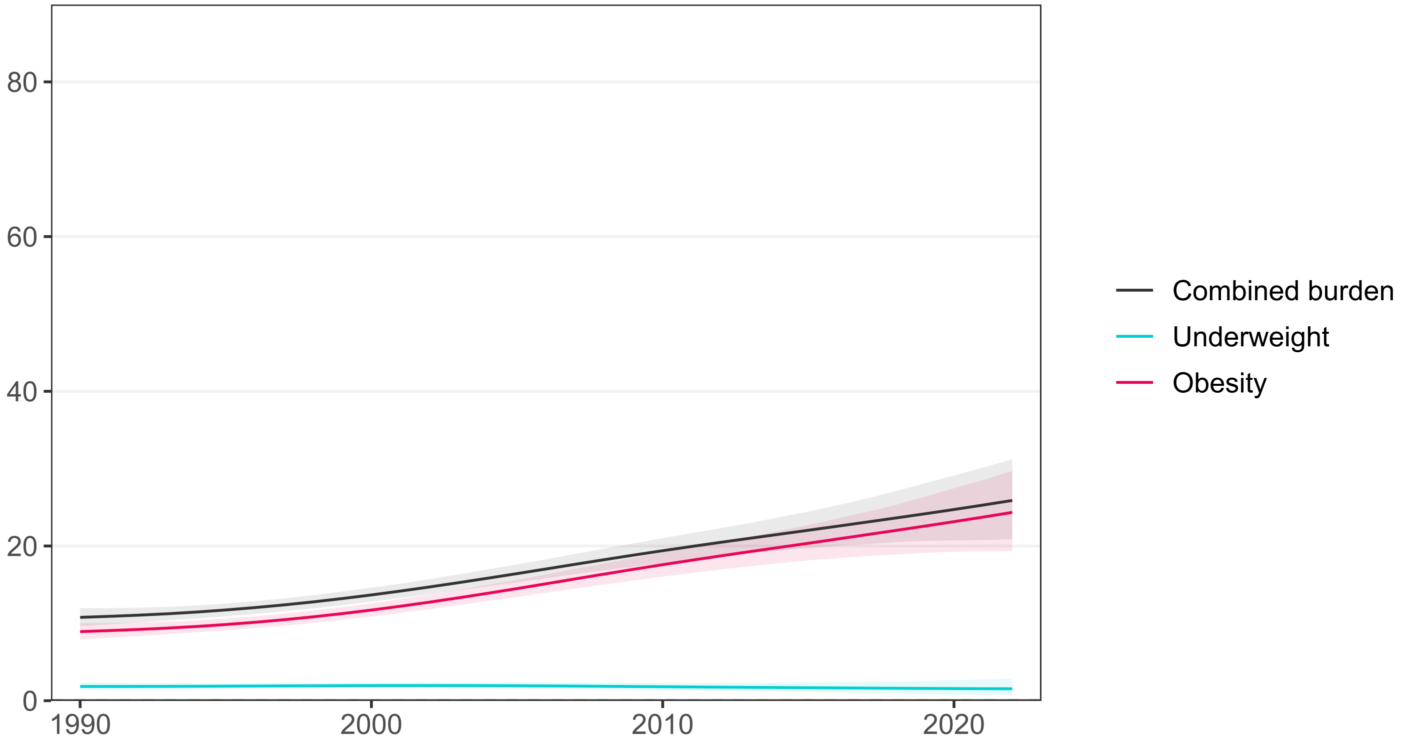

# Rwanda

## School-aged children and adolescents

### Girls

7 studies (7 national)

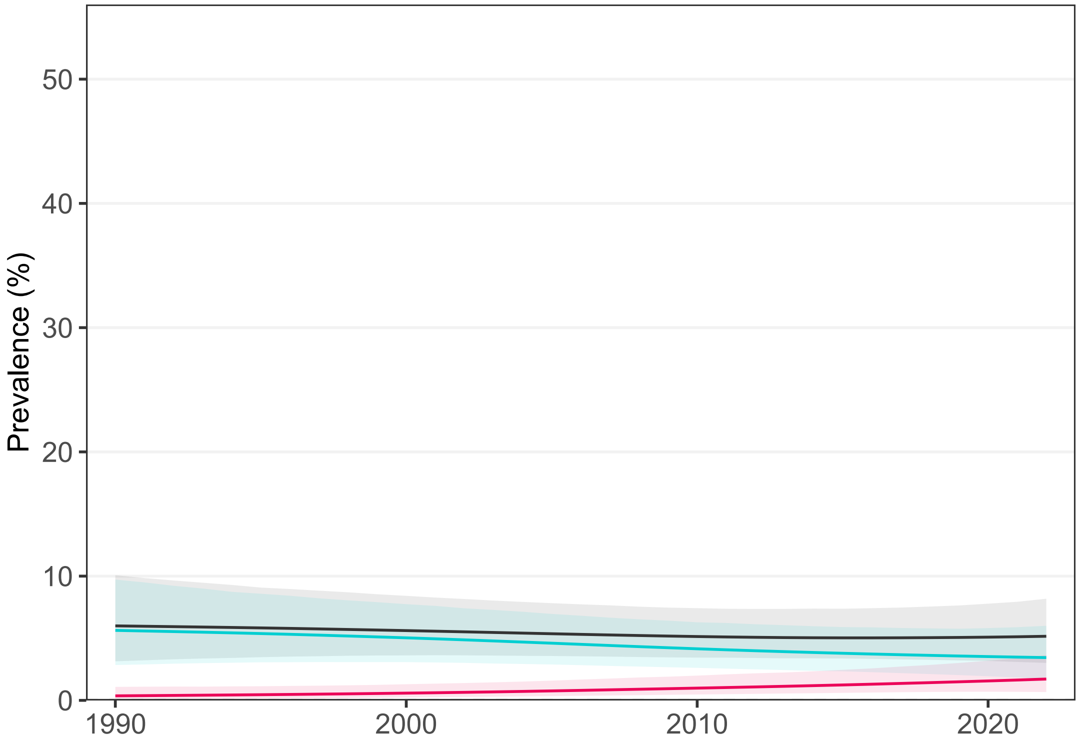


### Boys

4 studies (4 national)

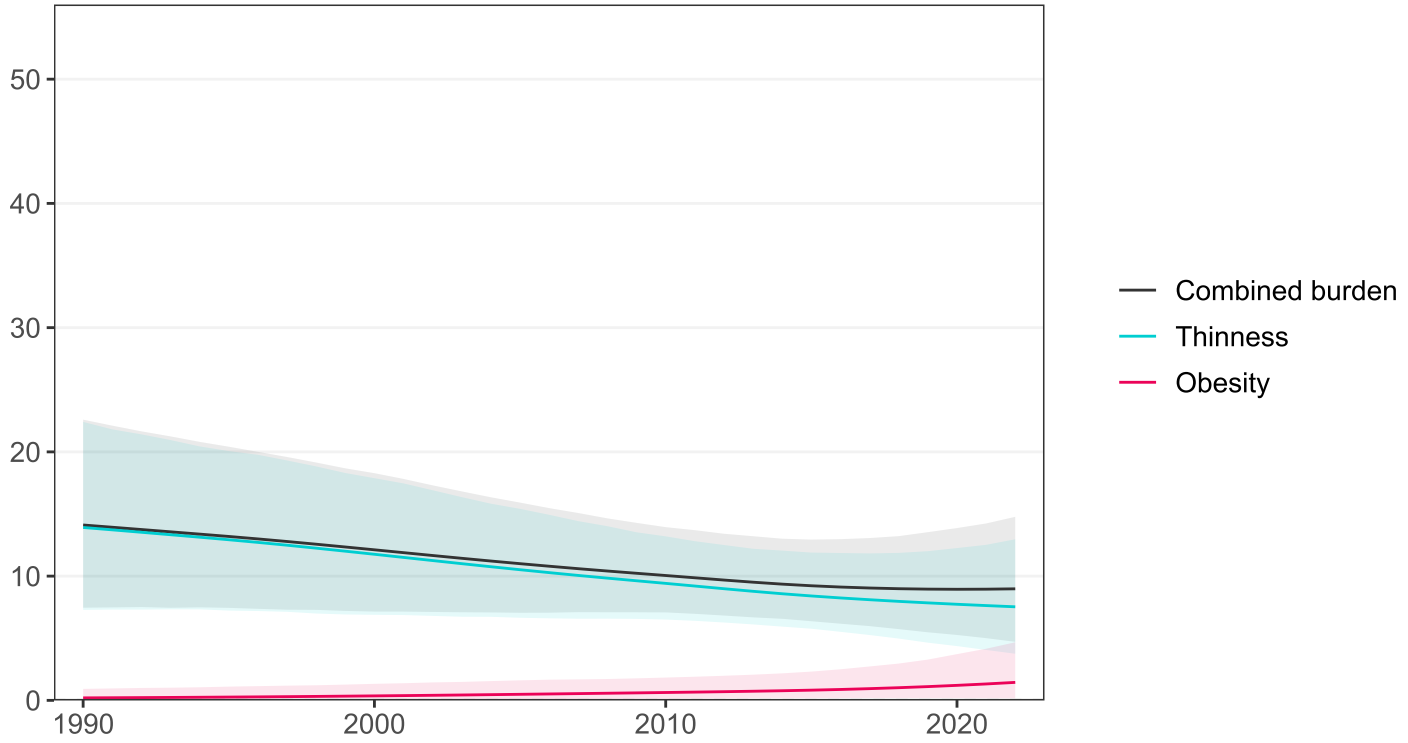


## Adults

### Women

7 studies (7 national)

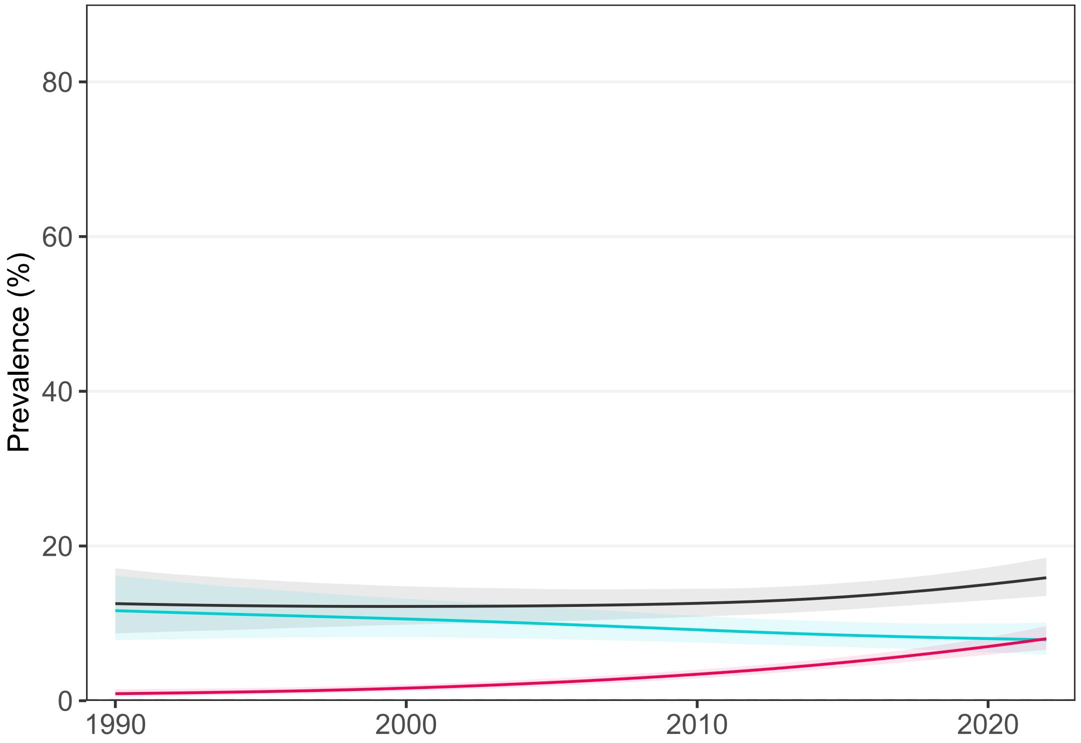


### Men

4 studies (4 national)

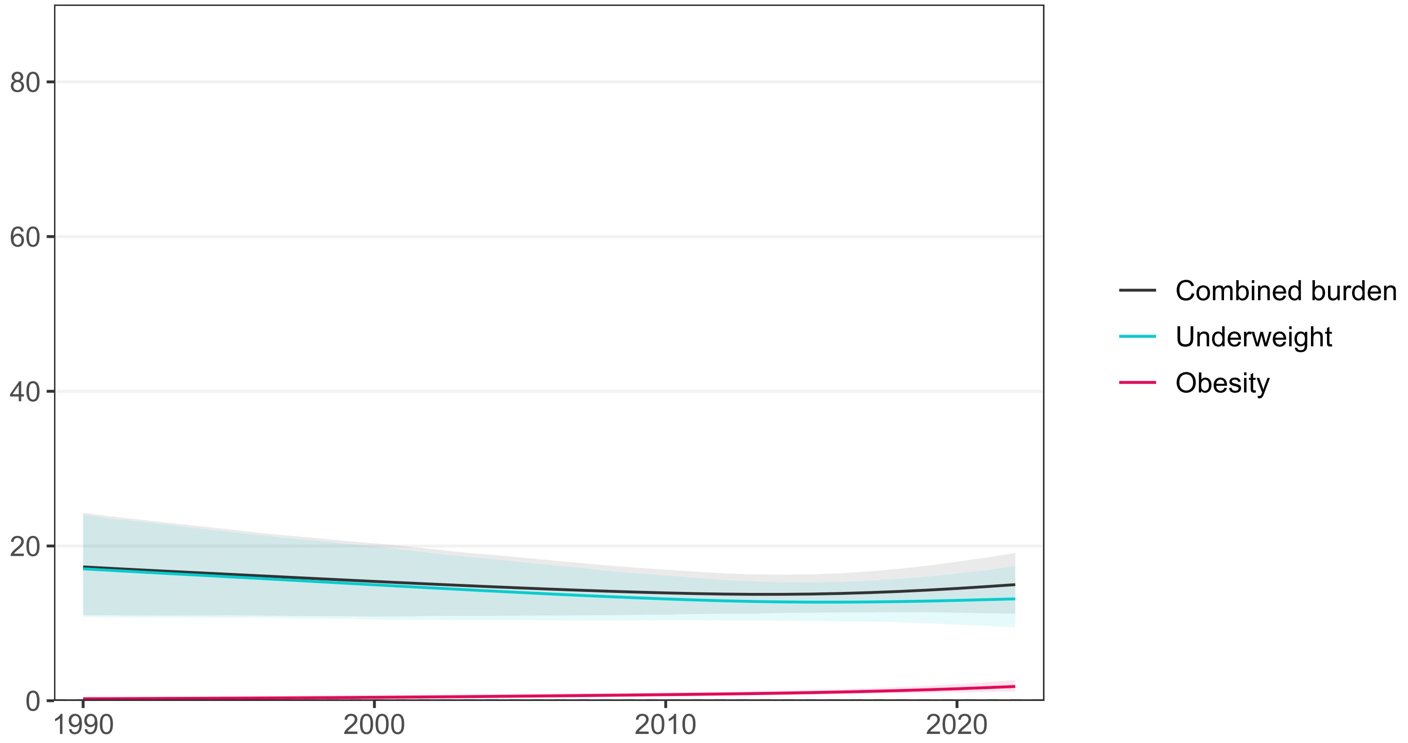

# Samoa

## School-aged children and adolescents

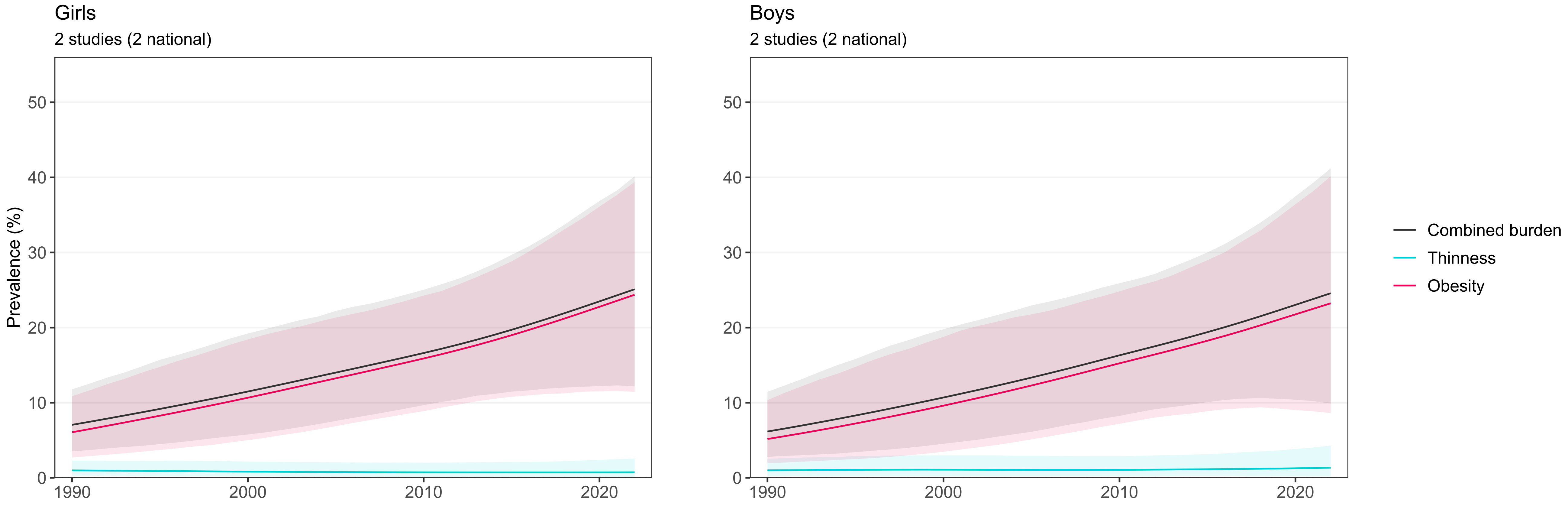


## Adults

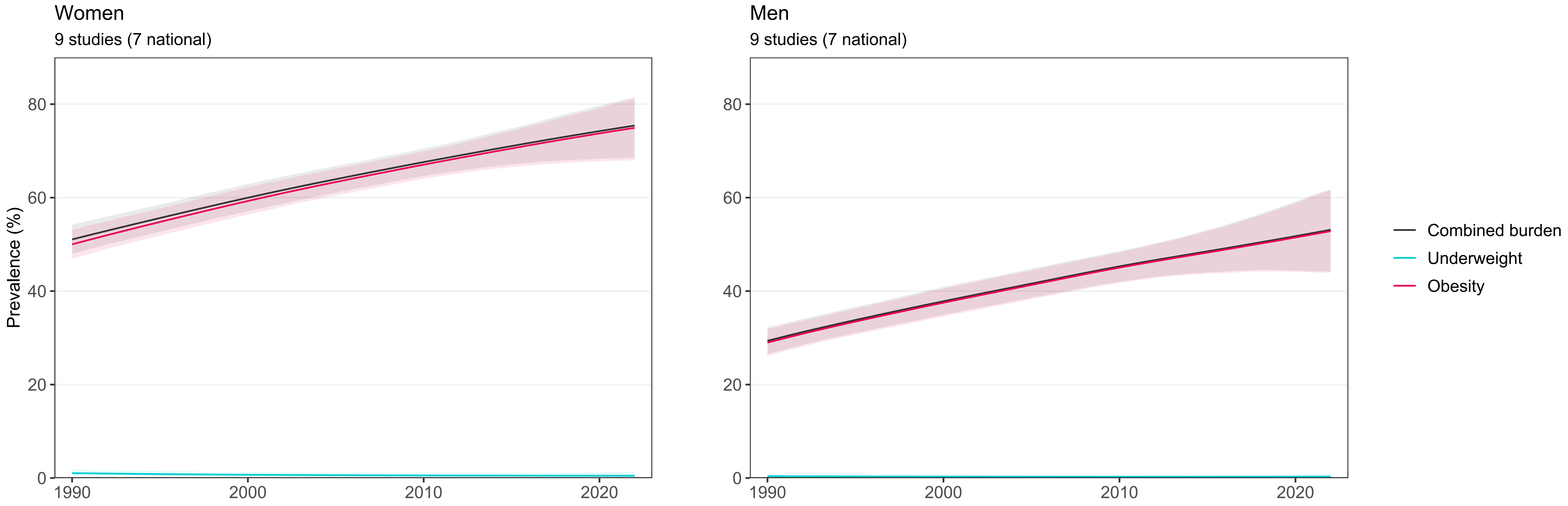

# Sao Tome & Principe

## School-aged children and adolescents

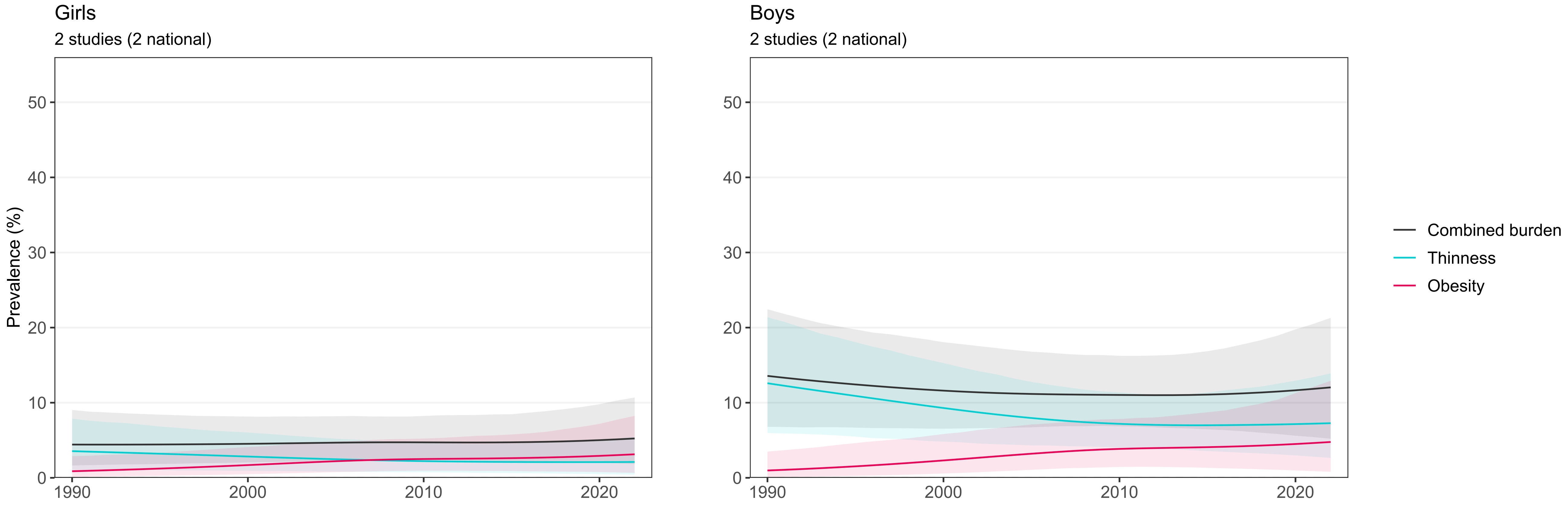


## Adults

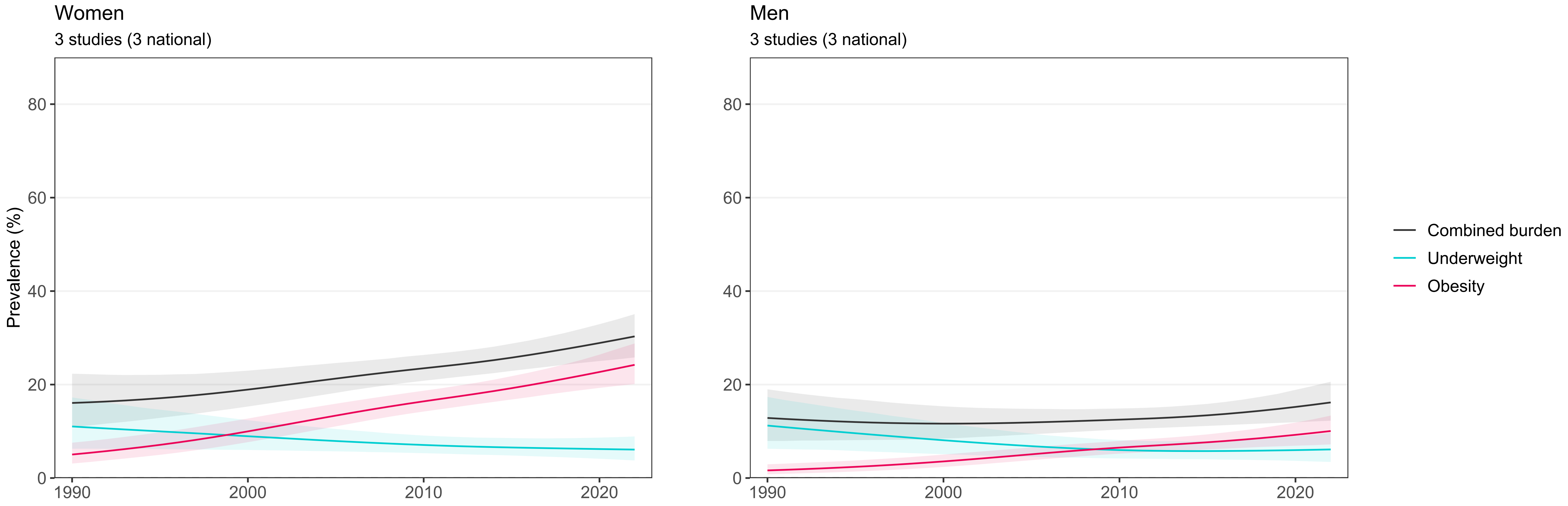

# Saudi Arabia

## School-aged children and adolescents

### Girls

9 studies (7 national)

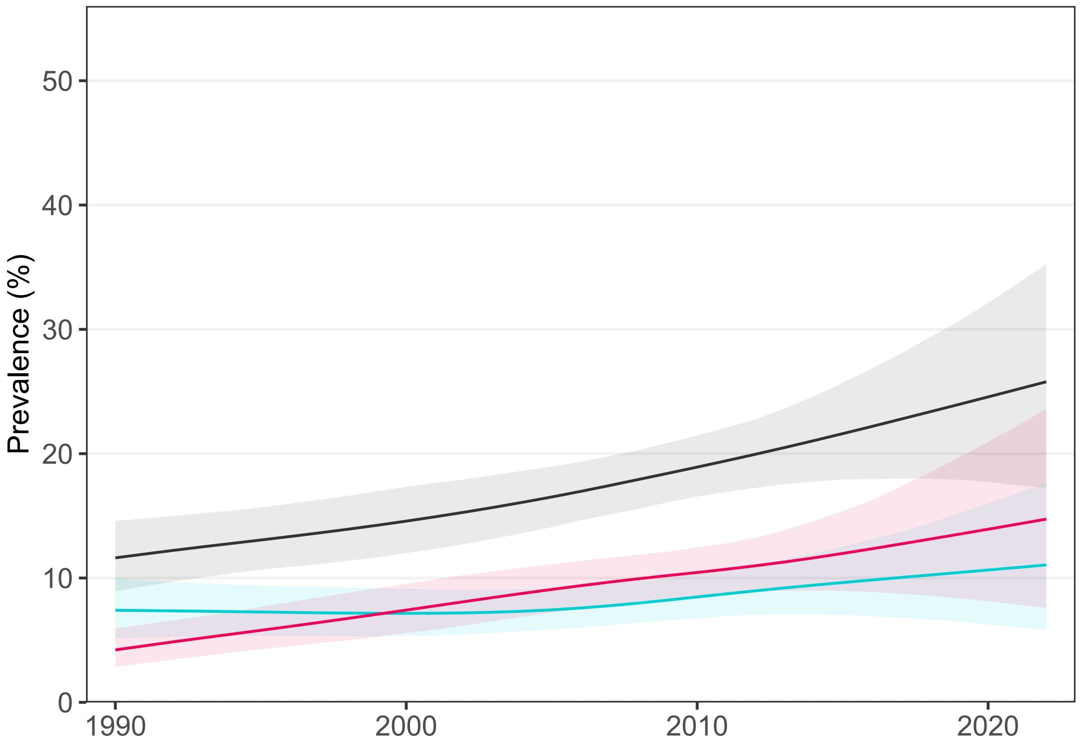


### Boys

9 studies (7 national)

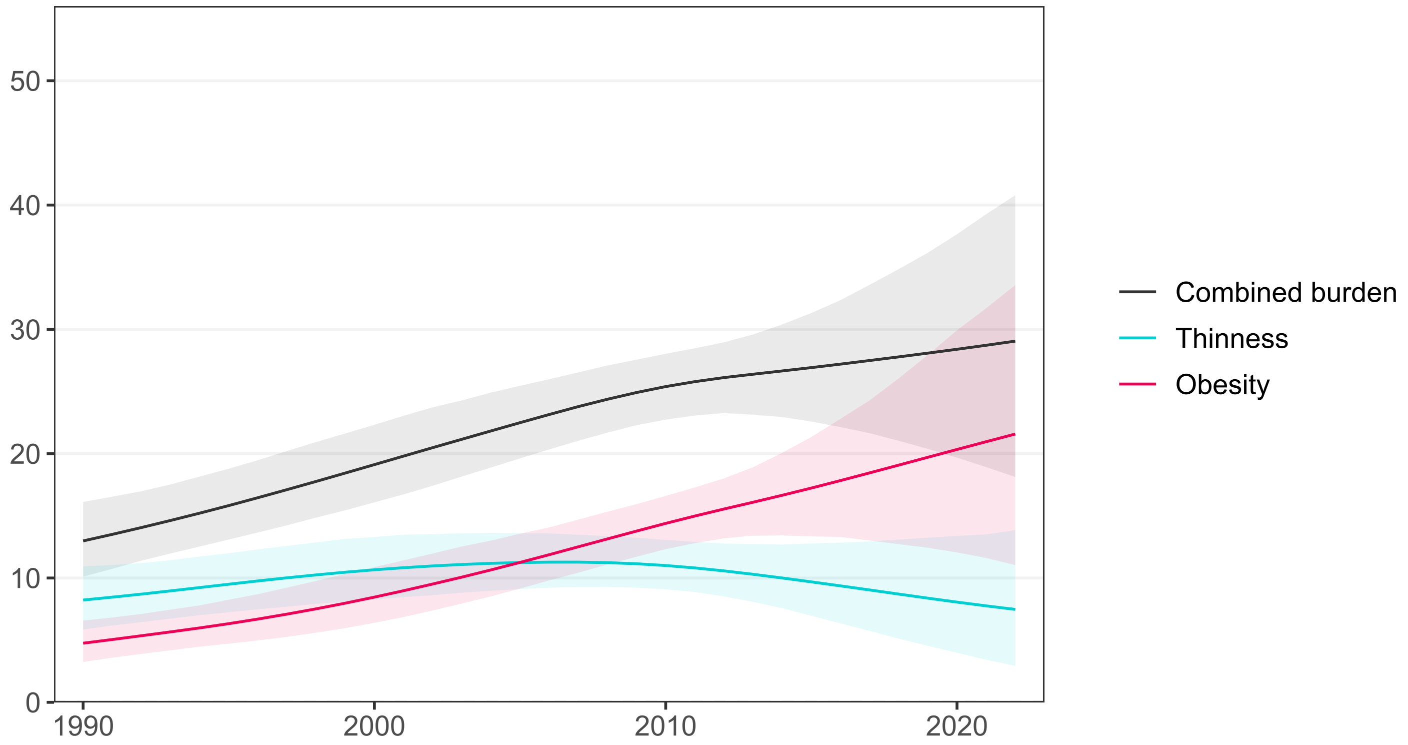


## Adults

### Women

15 studies (12 national)

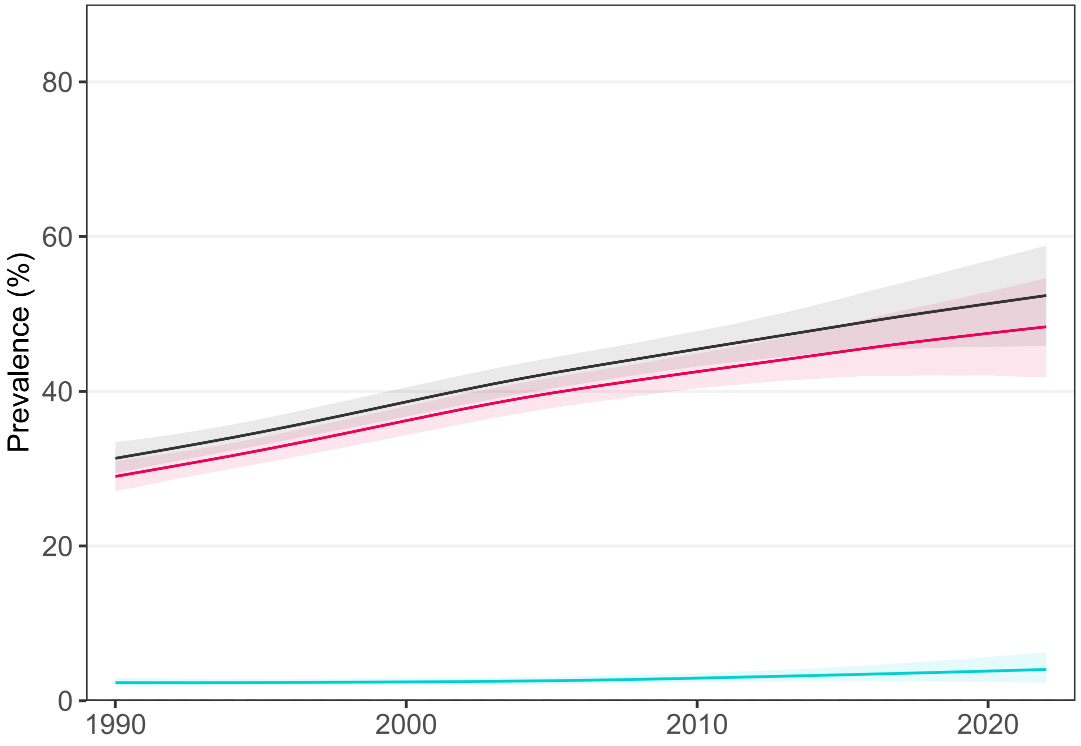


### Men

15 studies (12 national)

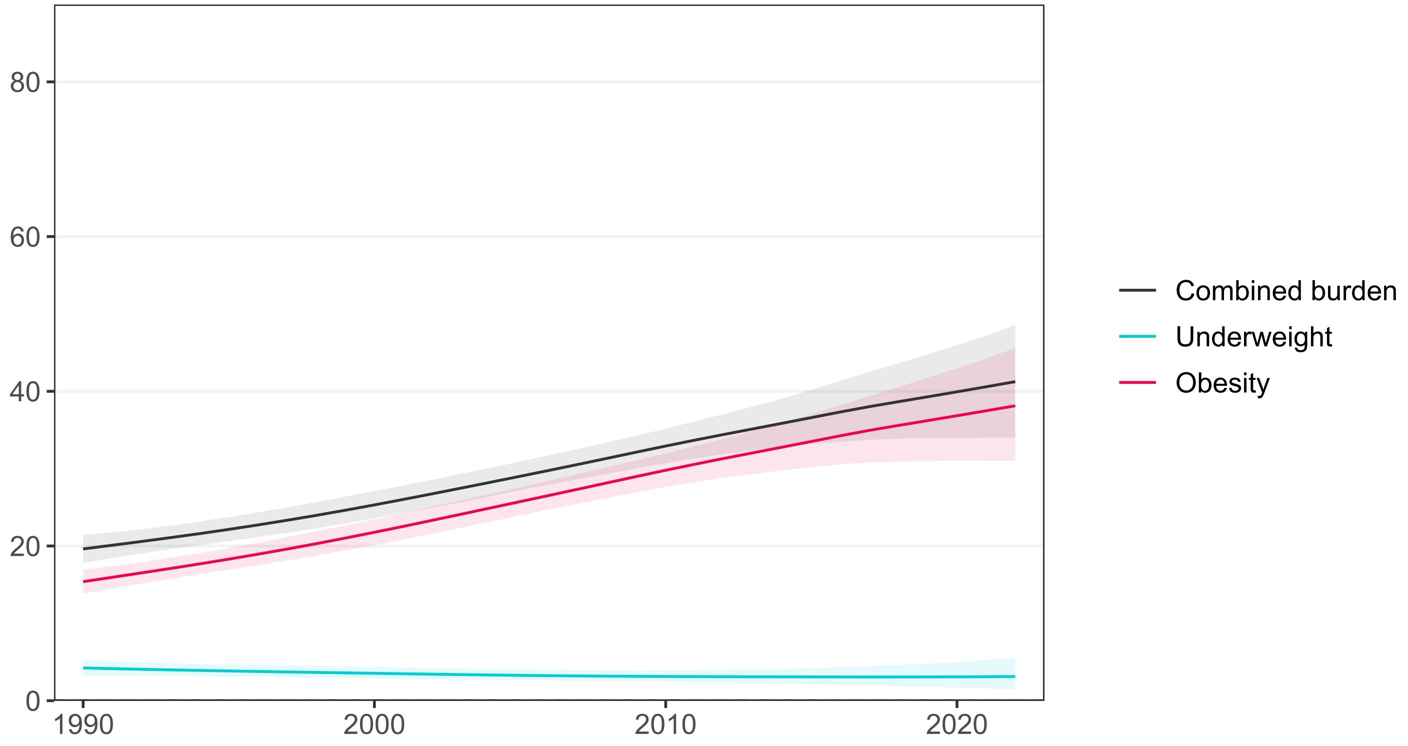

# Senegal

## School-aged children and adolescents

### Girls

3 studies (2 national)

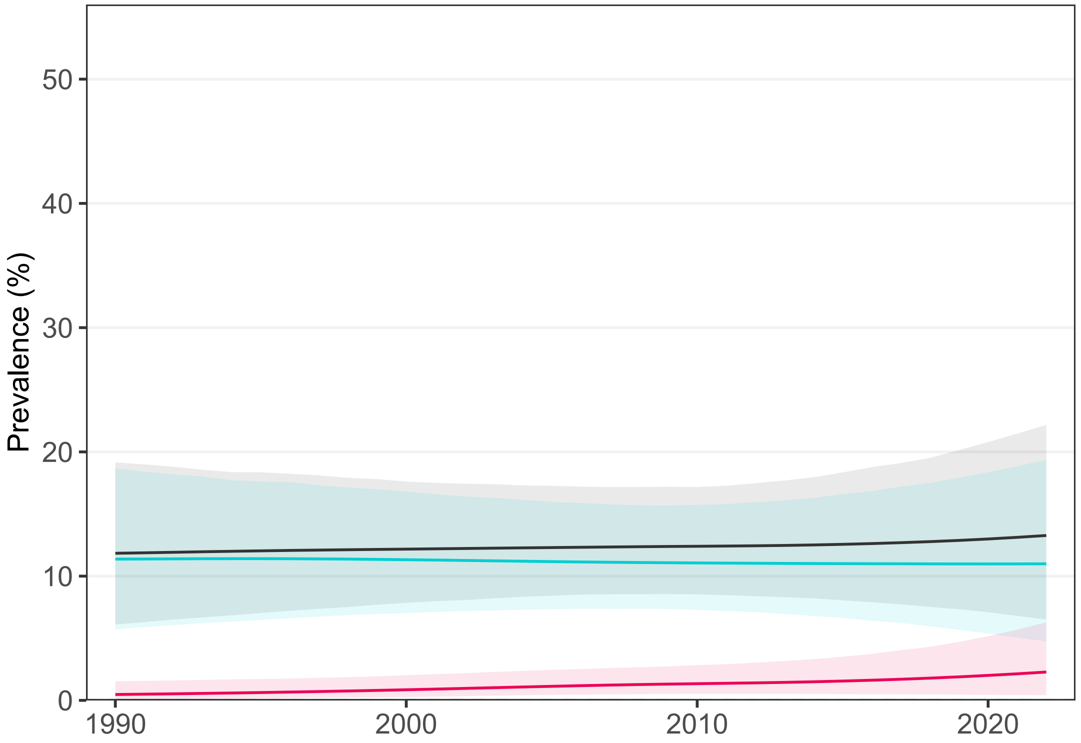


### Boys

2 studies (1 national)

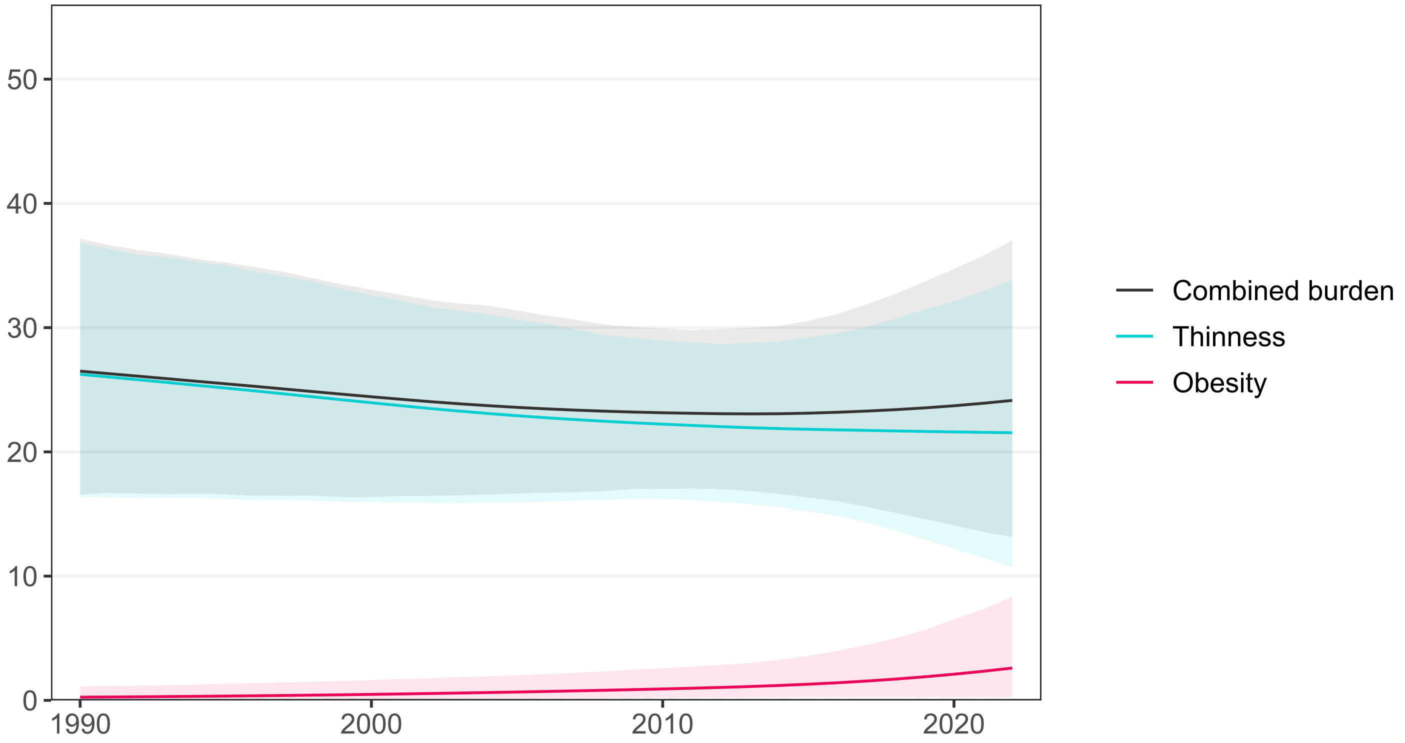


## Adults

### Women

10 studies (5 national)

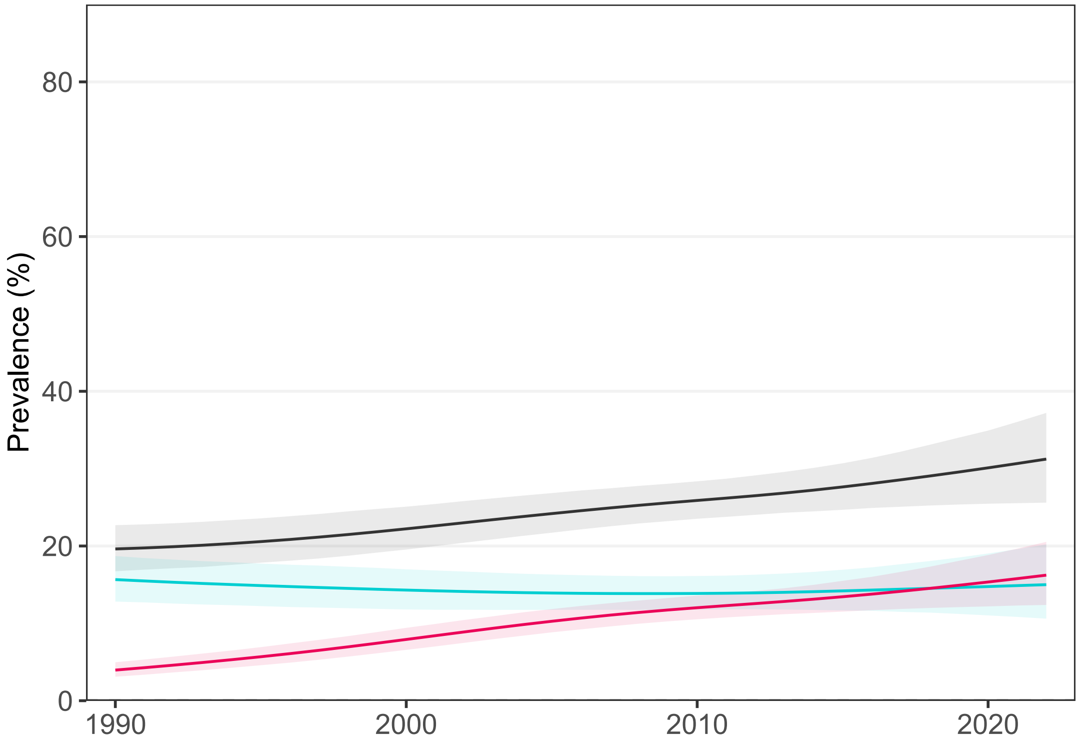


### Men

5 studies (2 national)

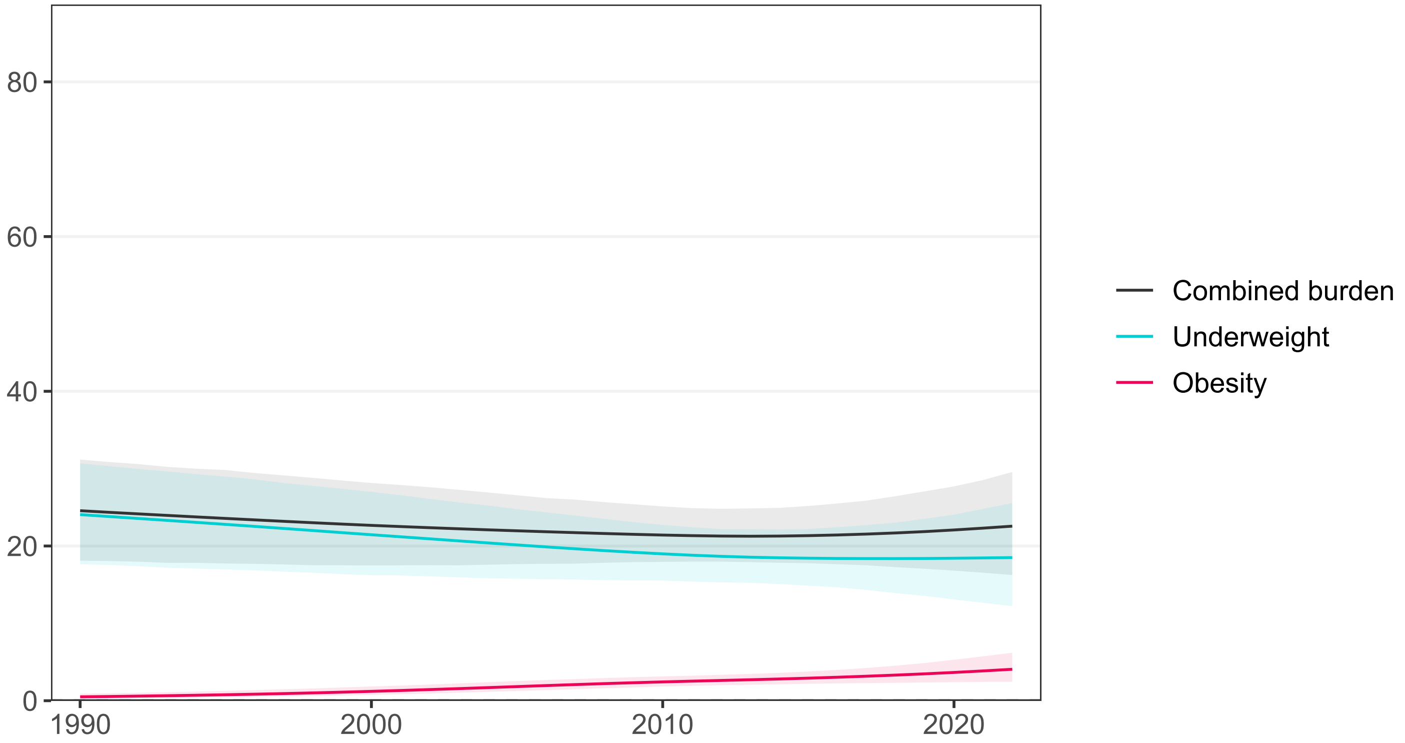

# Serbia

## School-aged children and adolescents

### Girls

24 studies (7 national)

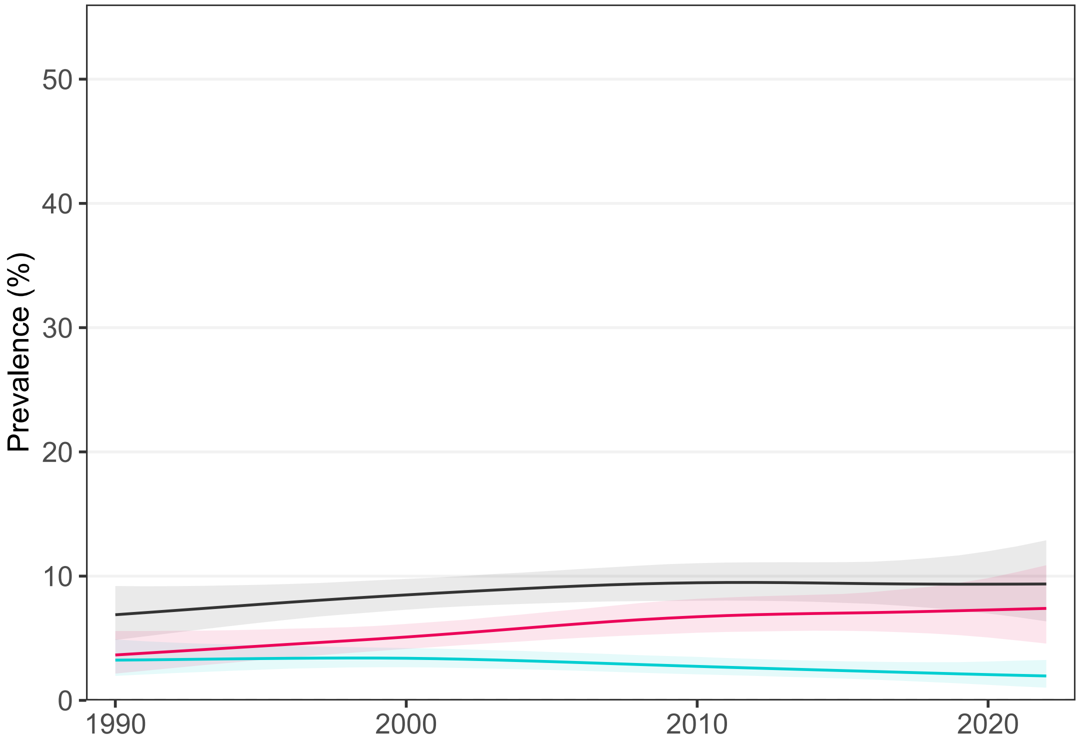


### Boys

25 studies (8 national)

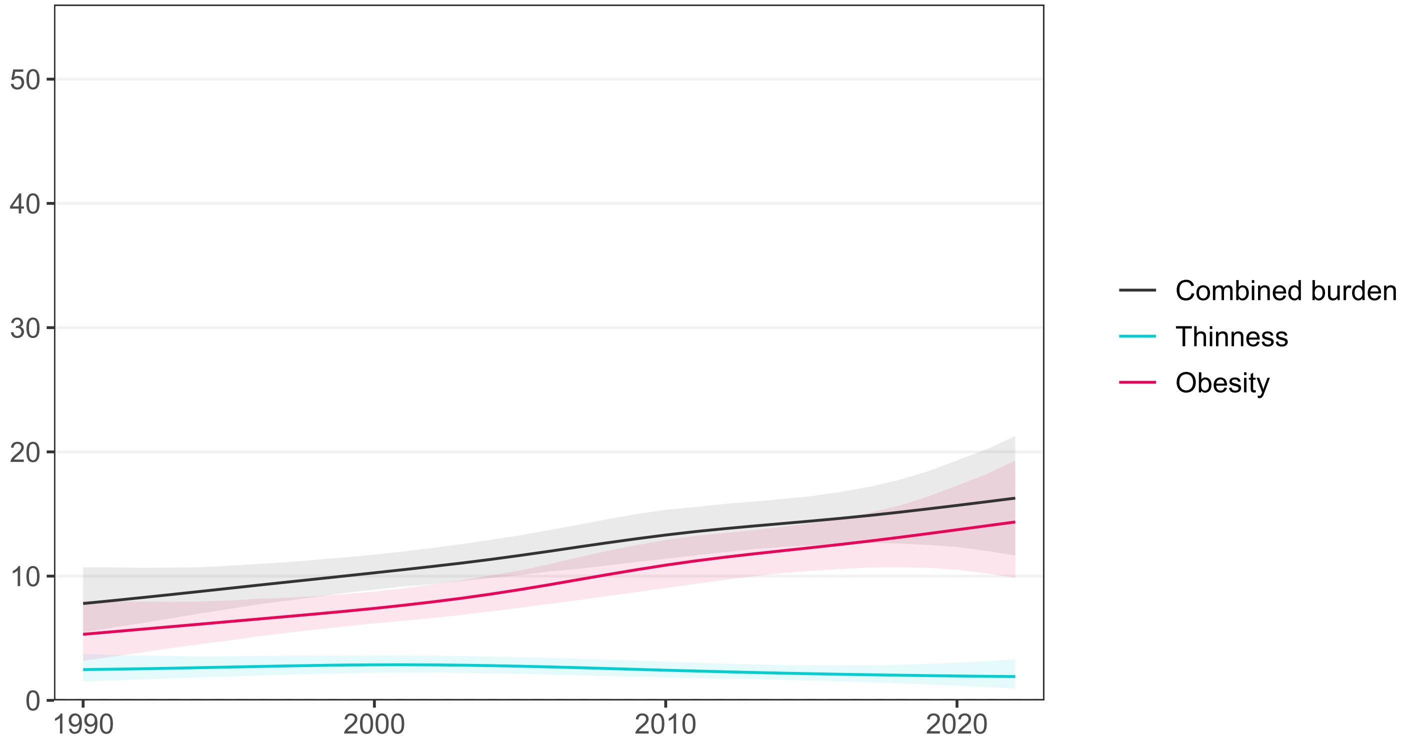


## Adults

### Women

12 studies (4 national)

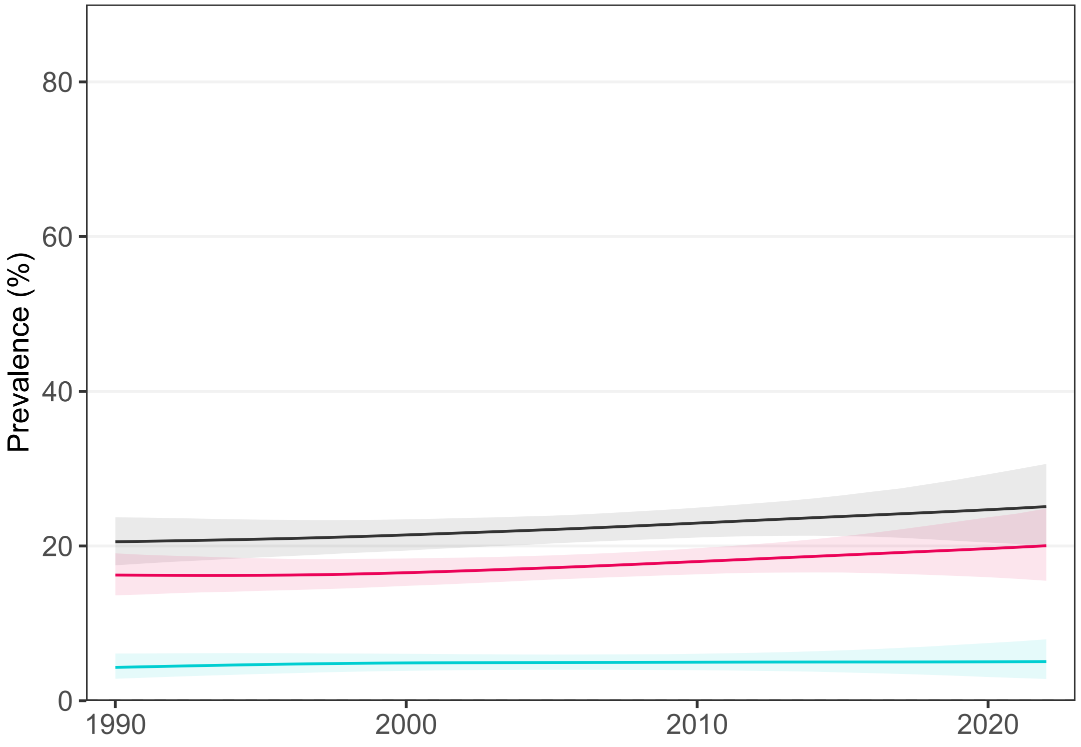


### Men

12 studies (4 national)

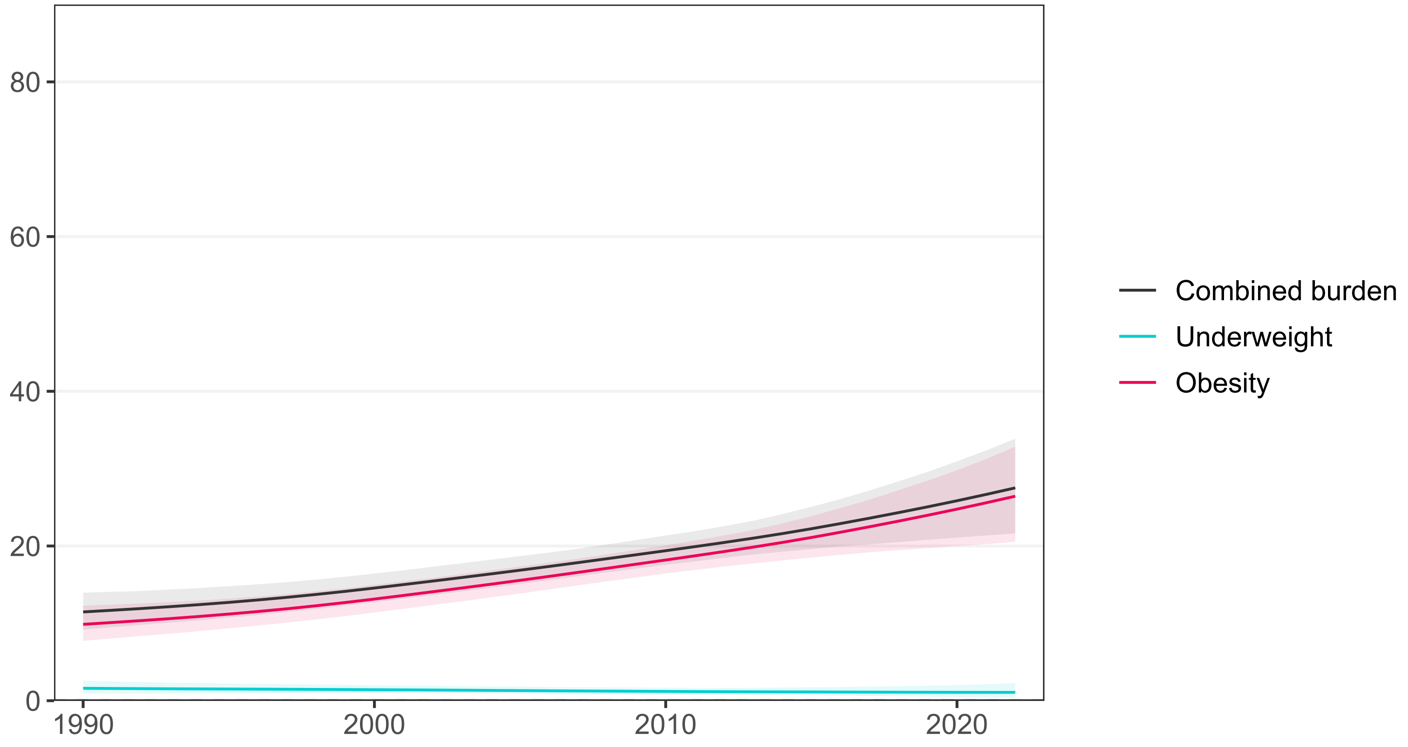

# Seychelles

## School-aged children and adolescents

### Girls

22 studies (22 national)

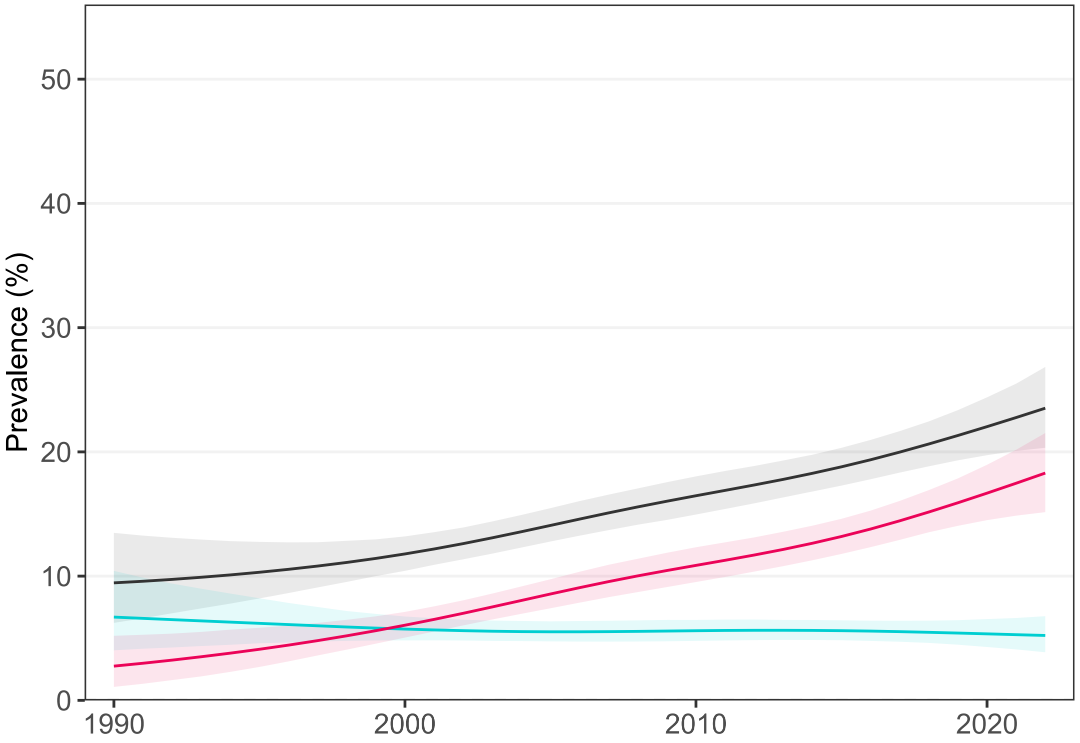


### Boys

22 studies (22 national)

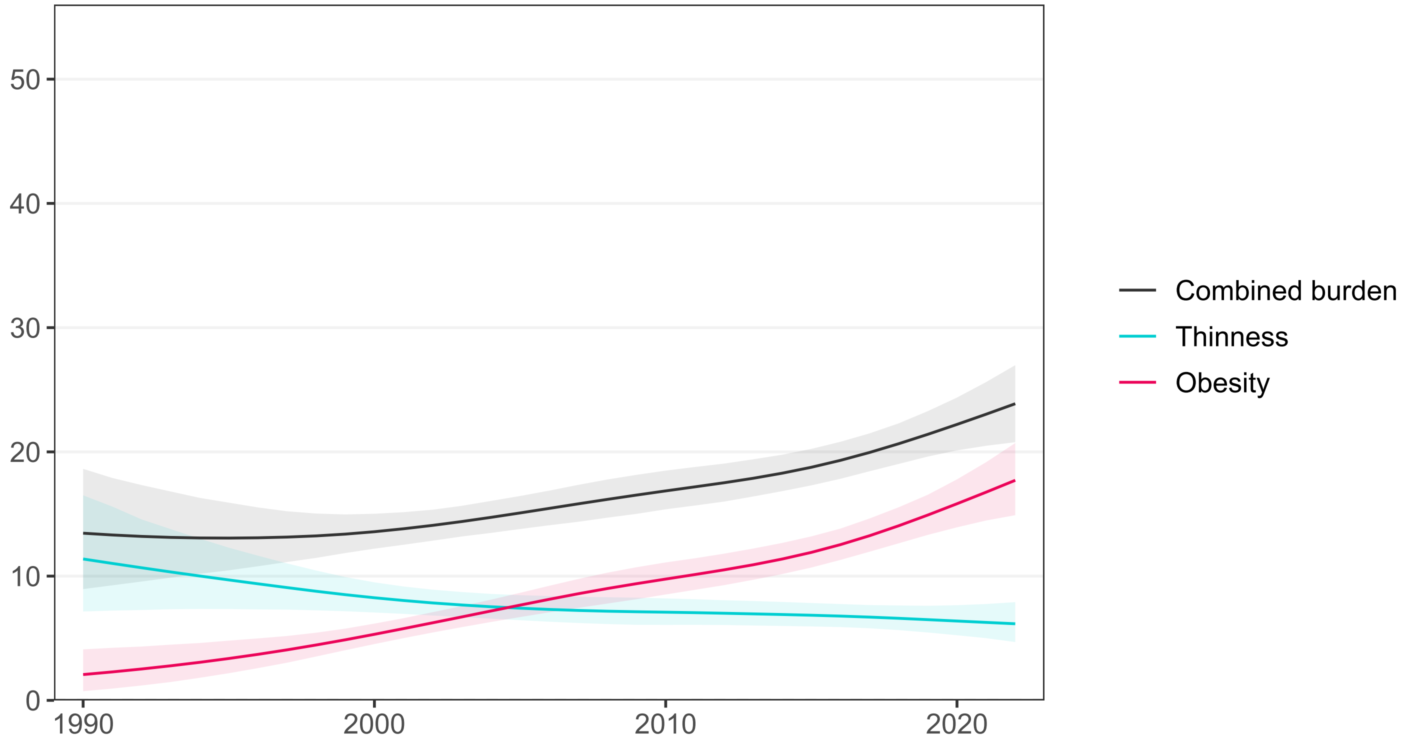


## Adults

### Women

5 studies (5 national)

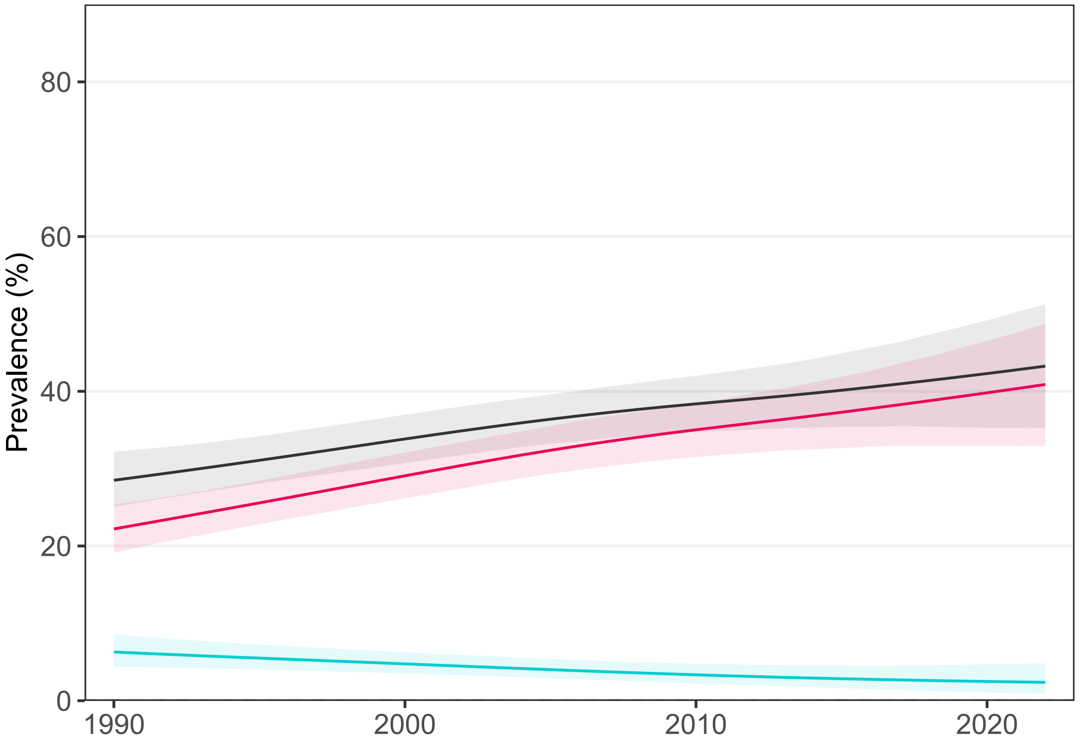


### Men

5 studies (5 national)

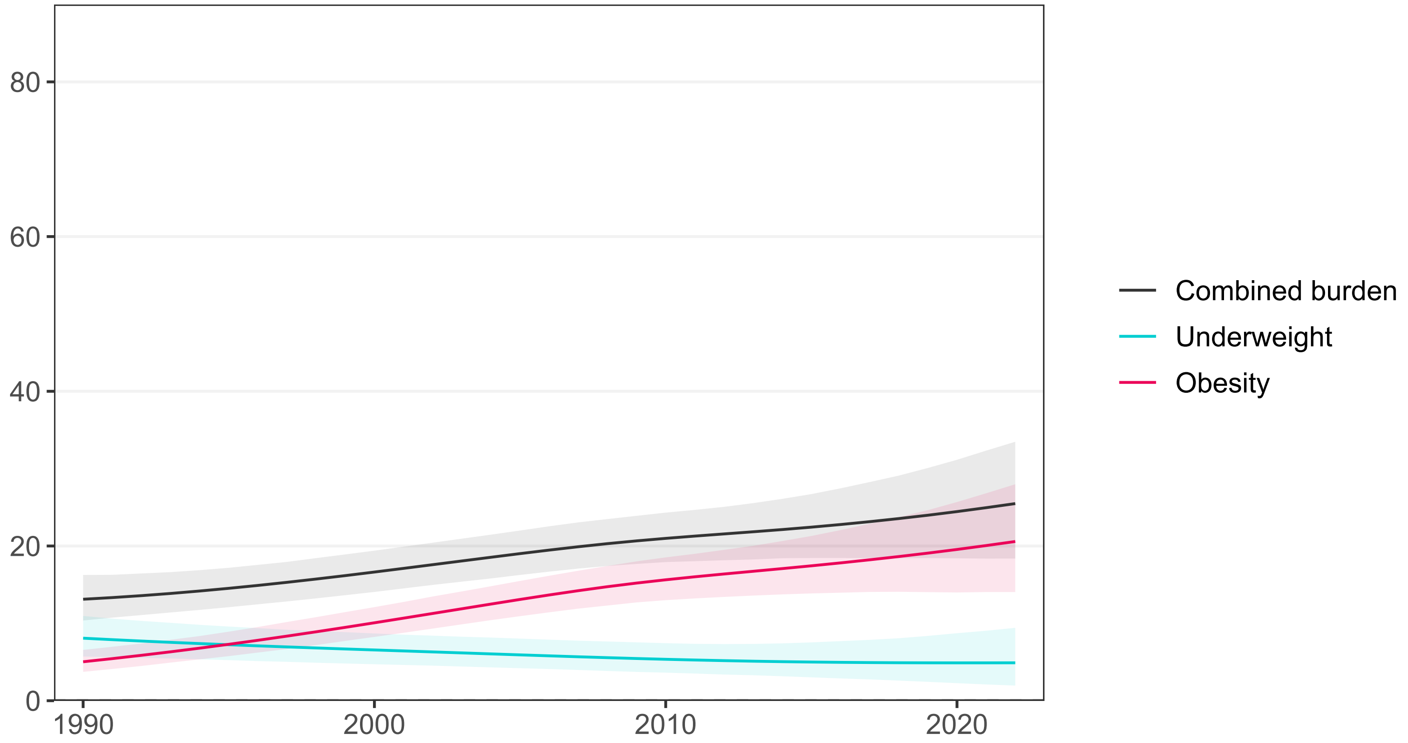

# Sierra Leone

## School-aged children and adolescents

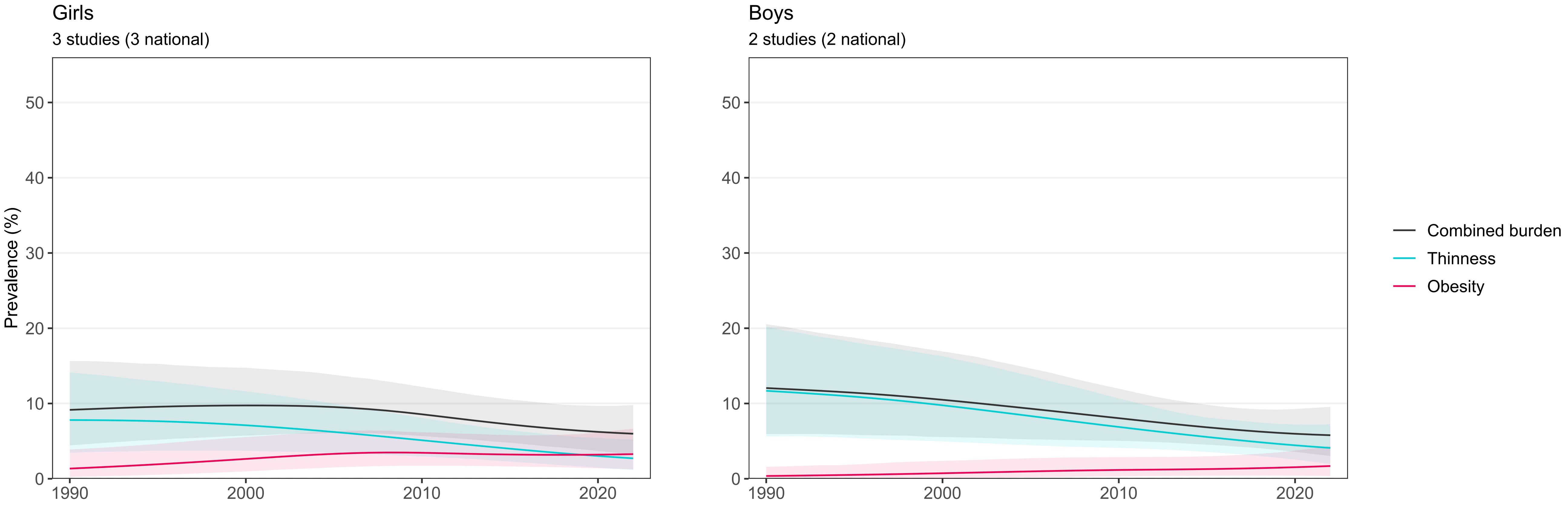


## Adults

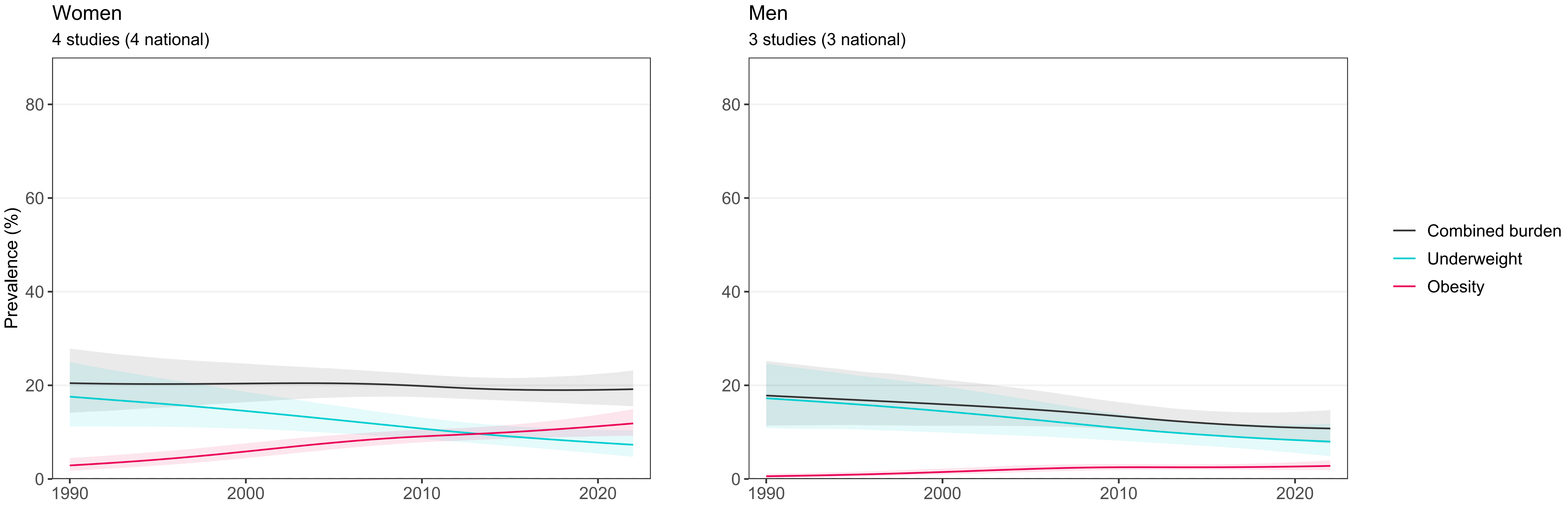

# Singapore

## School-aged children and adolescents

Girls
3 studies (3 national)

Boys
3 studies (3 national)

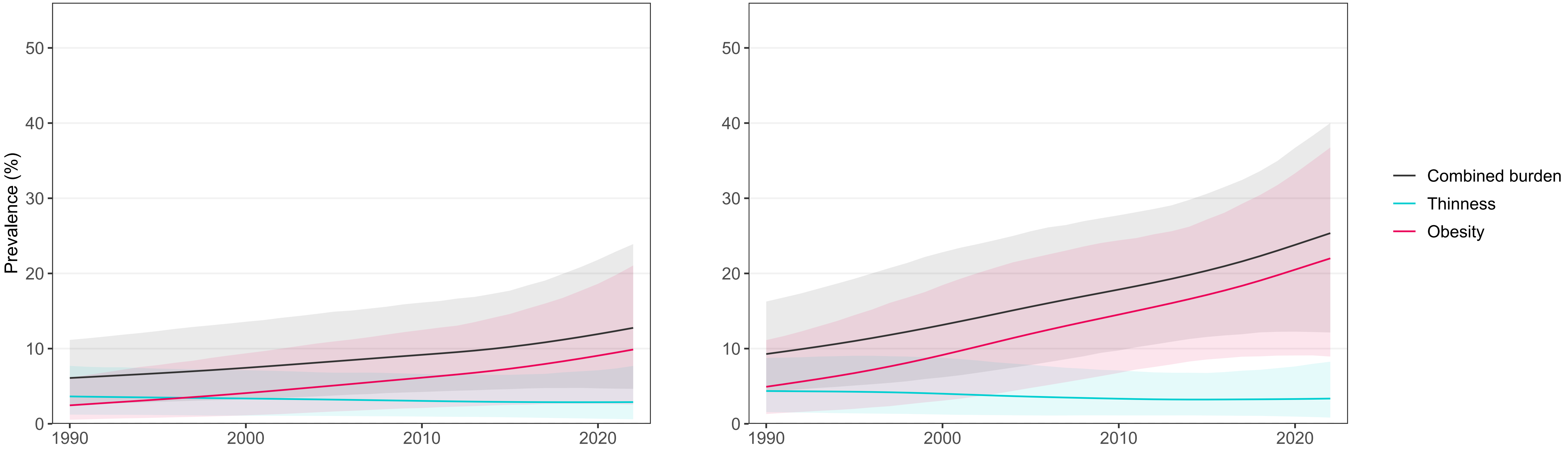


## Adults

Women
14 studies (10 national)

Men
14 studies (10 national)

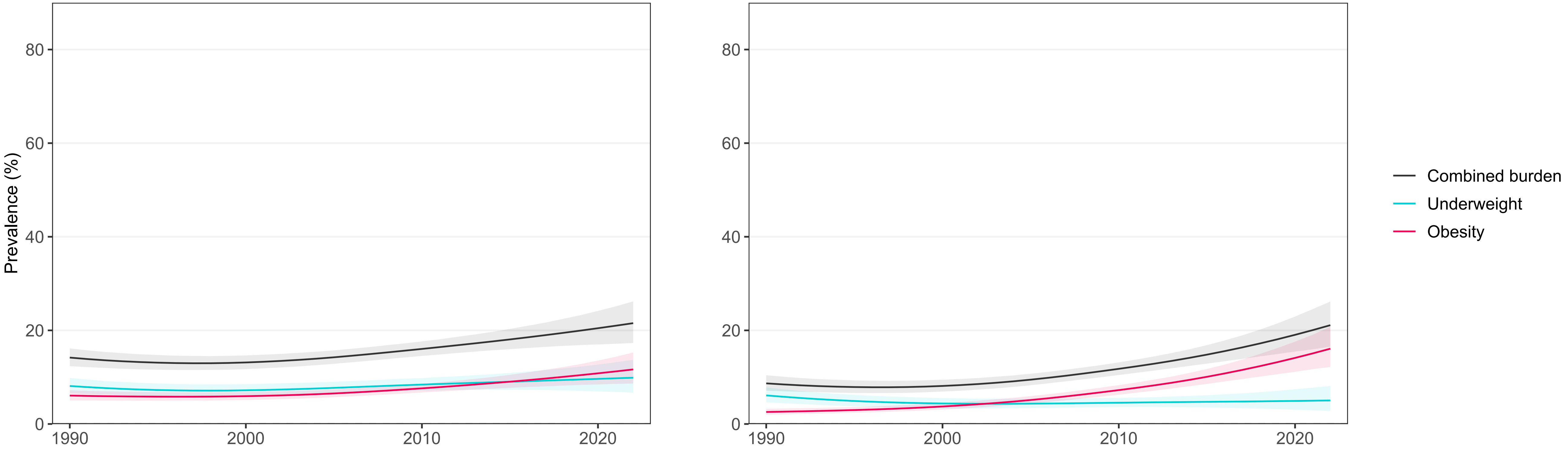

# Slovakia

## School-aged children and adolescents

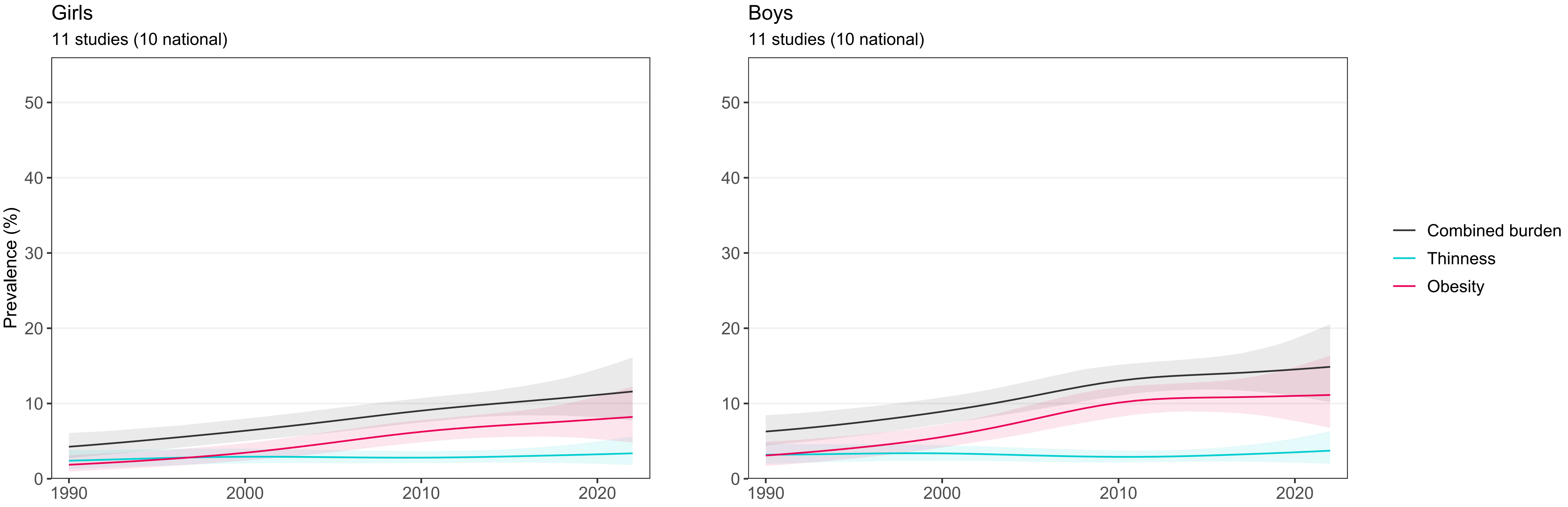


## Adults

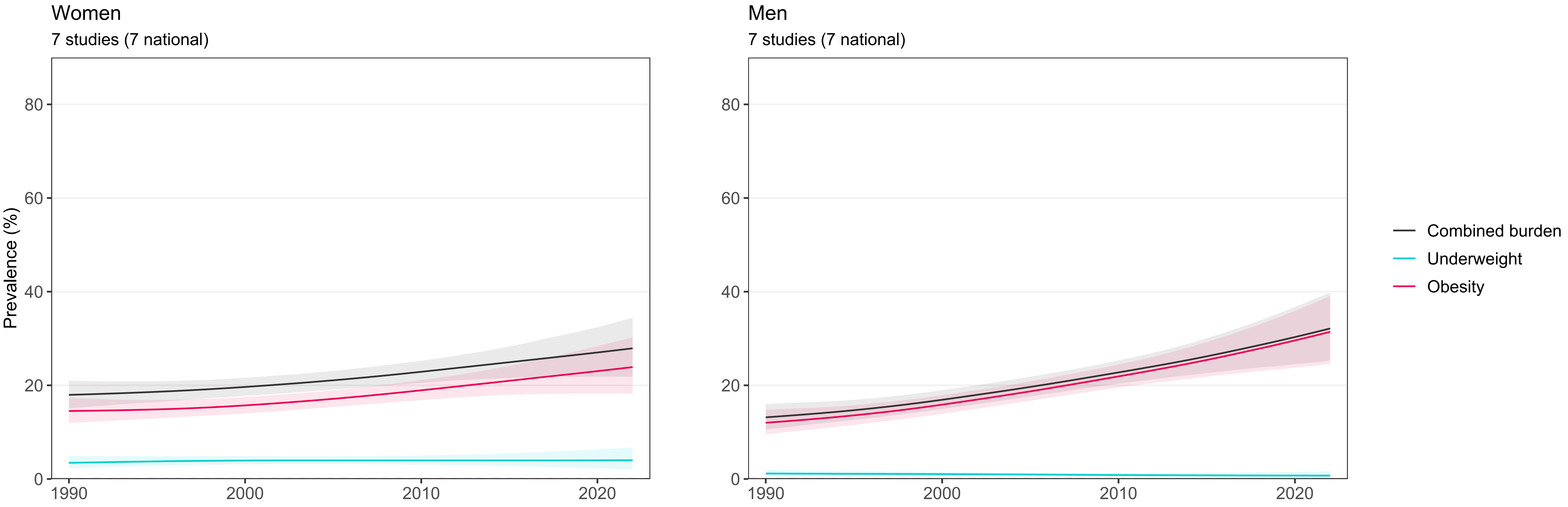

# Slovenia

## School-aged children and adolescents

Girls
52 studies (50 national)

Boys
52 studies (50 national)

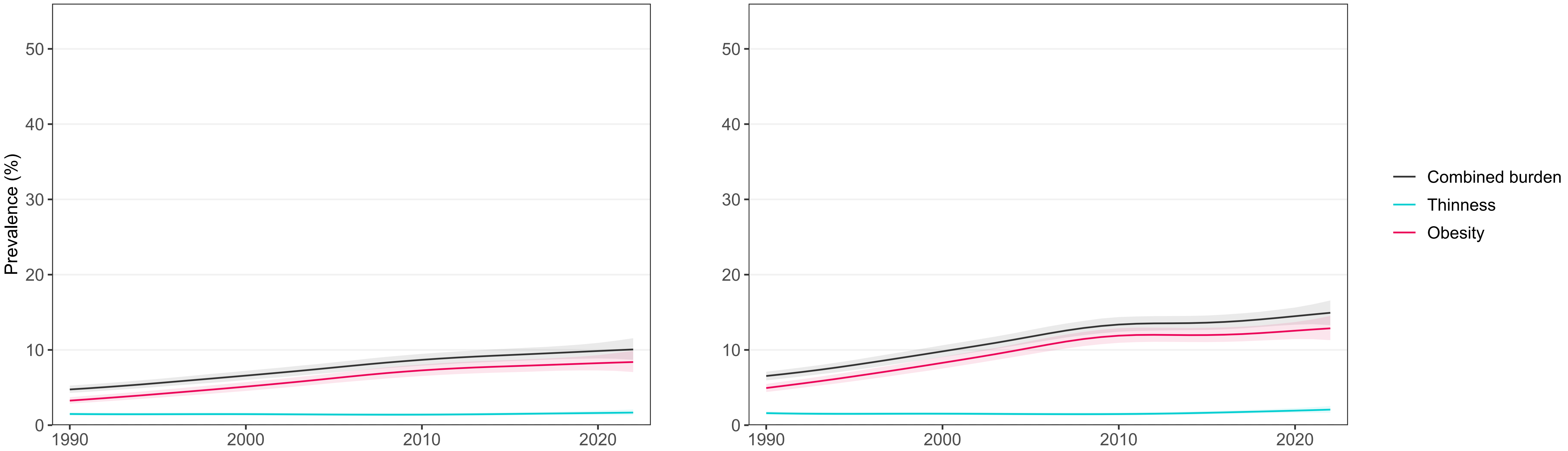


## Adults

Women
60 studies (58 national)

Men
60 studies (58 national)

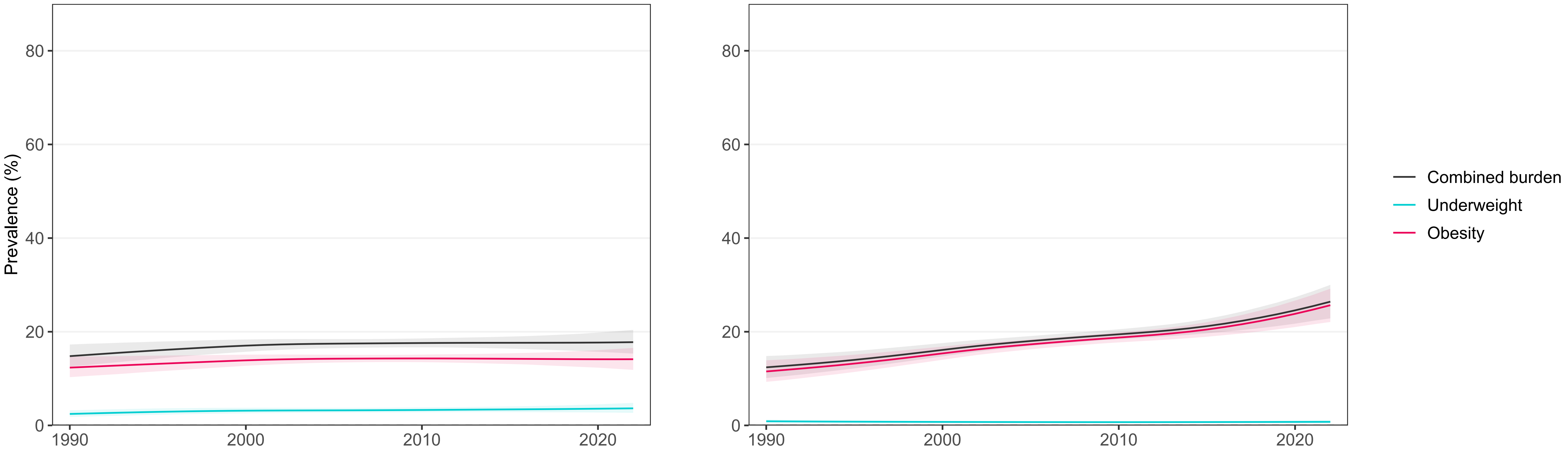

# Solomon Islands

## School-aged children and adolescents

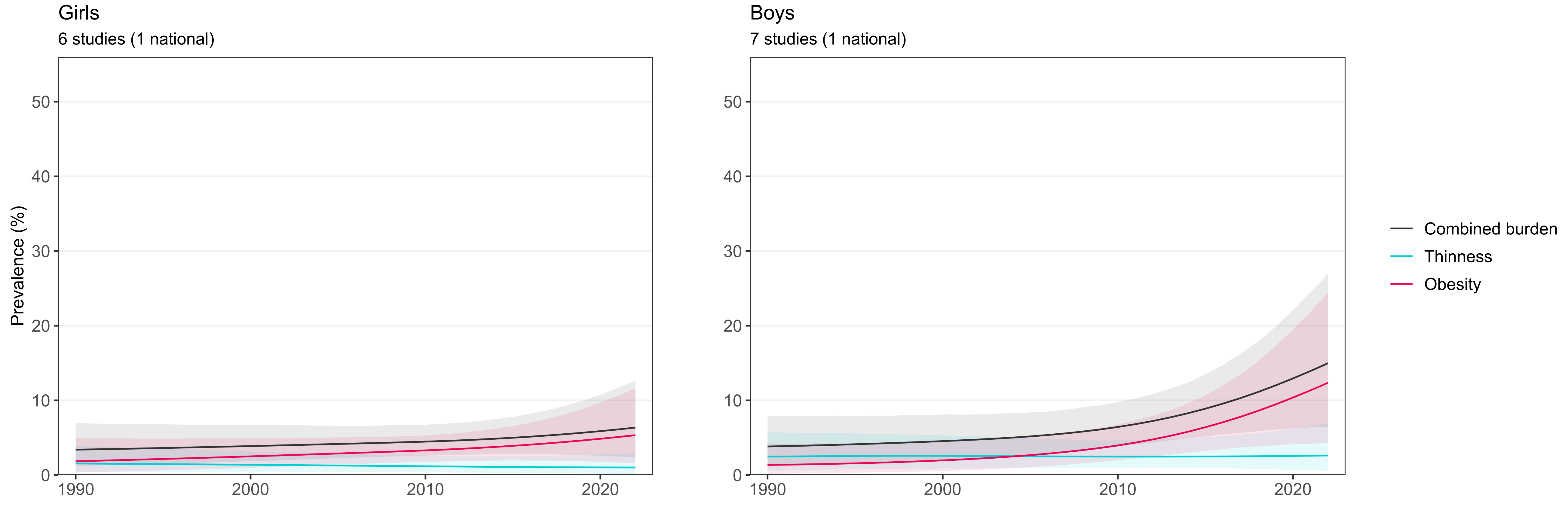


## Adults

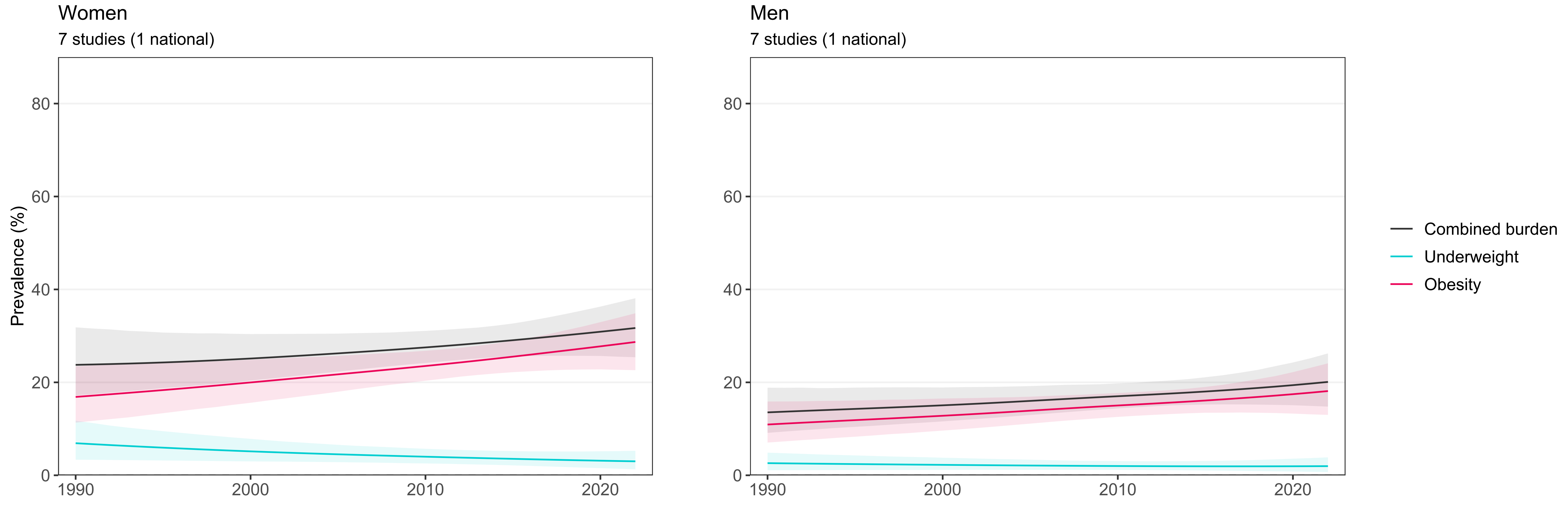

# Somalia

## School-aged children and adolescents

### Girls

No studies

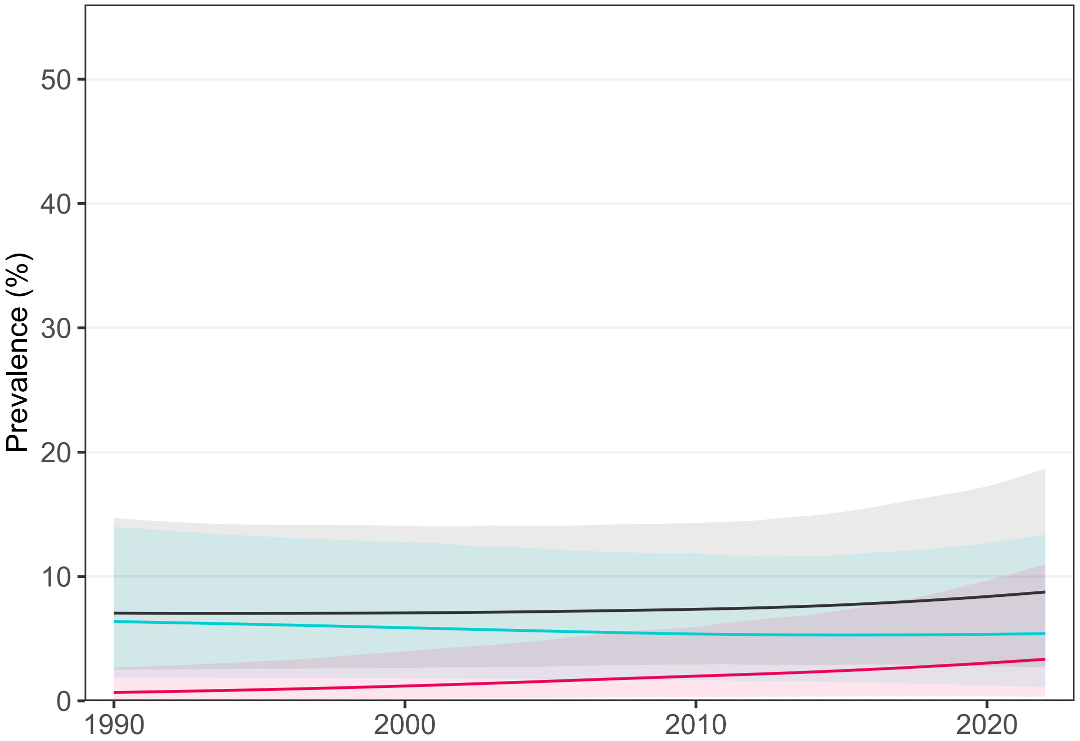


### Boys

No studies

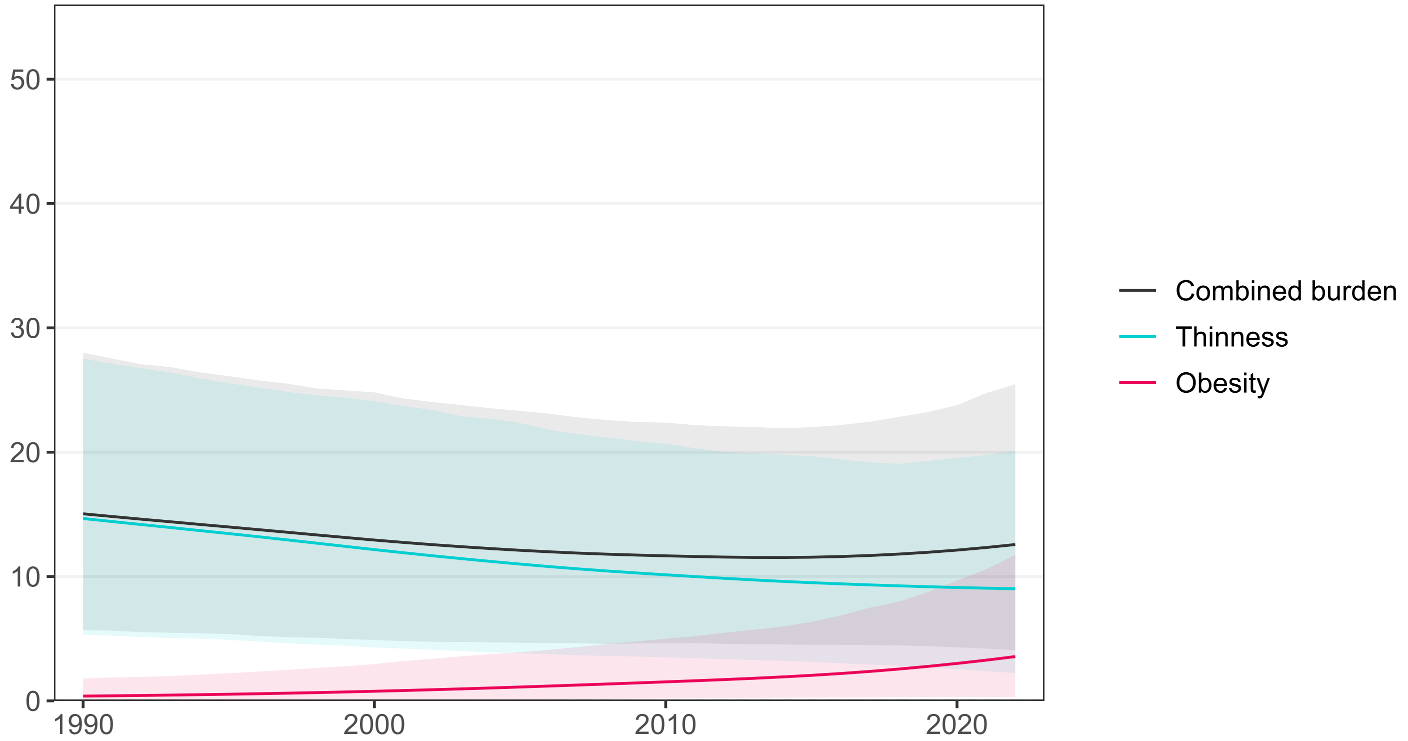


## Adults

### Women

1 study (0 national)

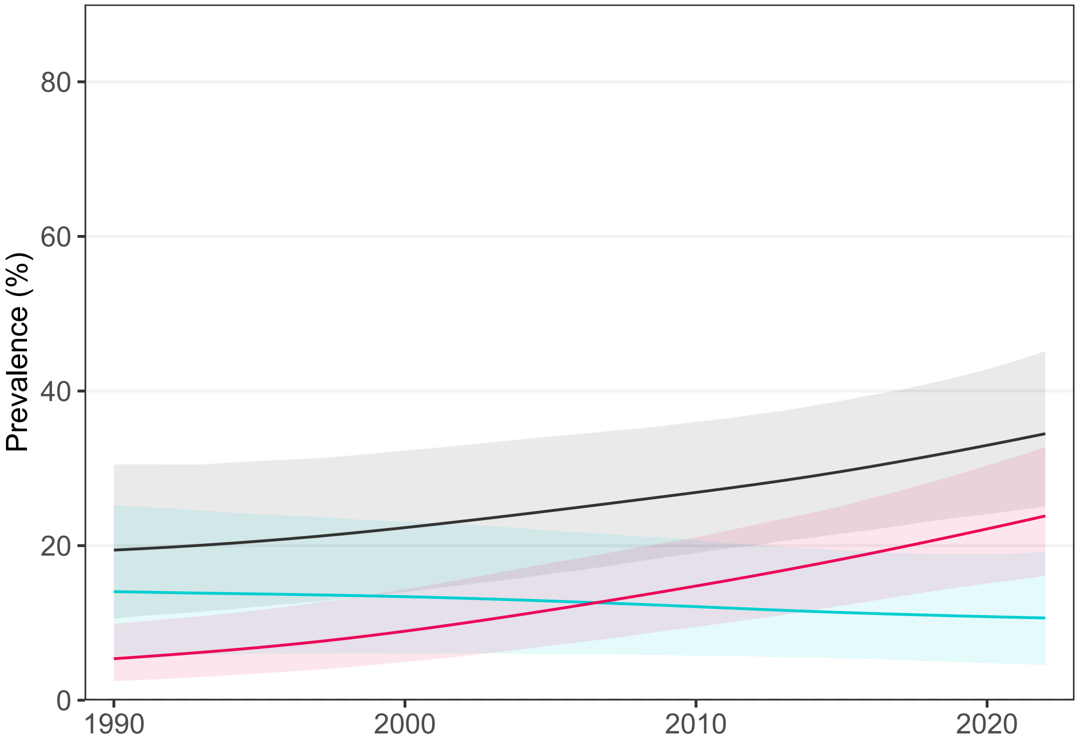


### Men

1 study (0 national)

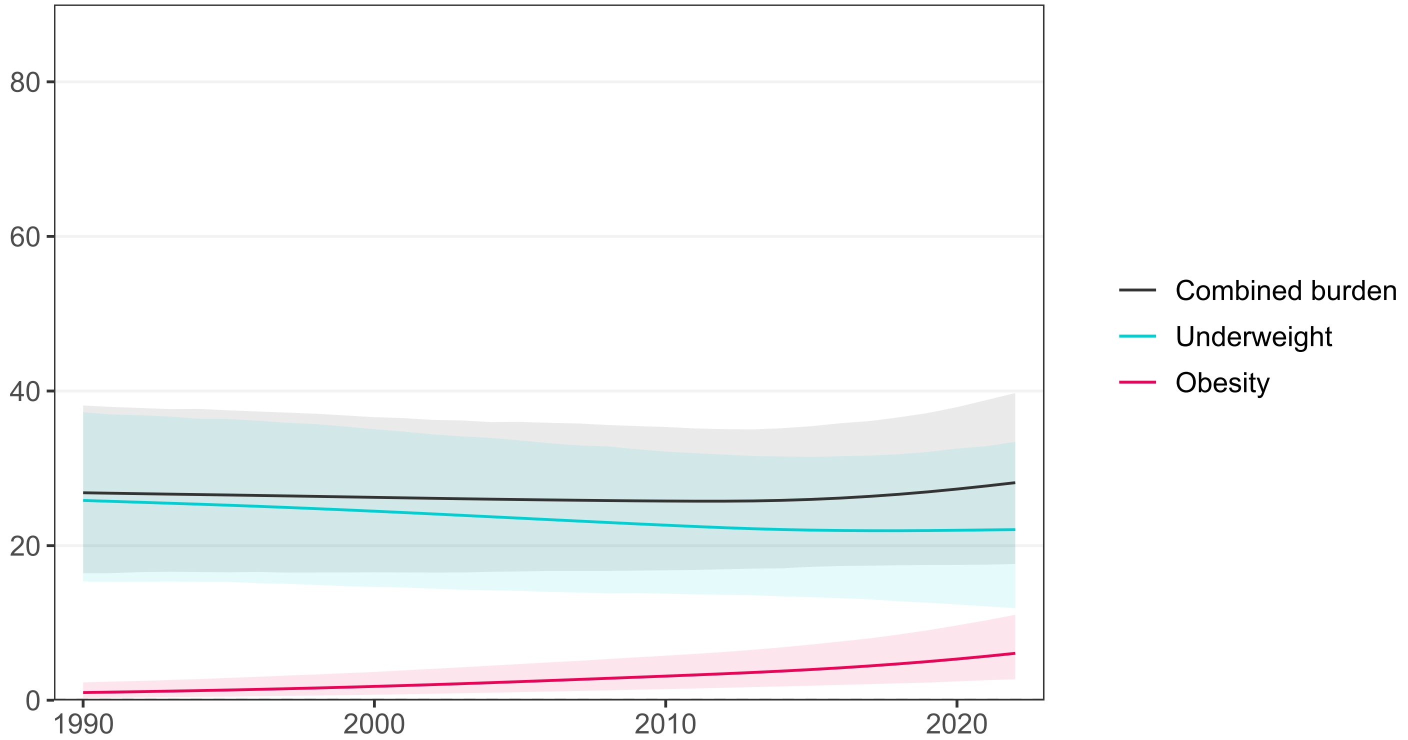

# South Africa

## School-aged children and adolescents

### Girls

25 studies (11 national)

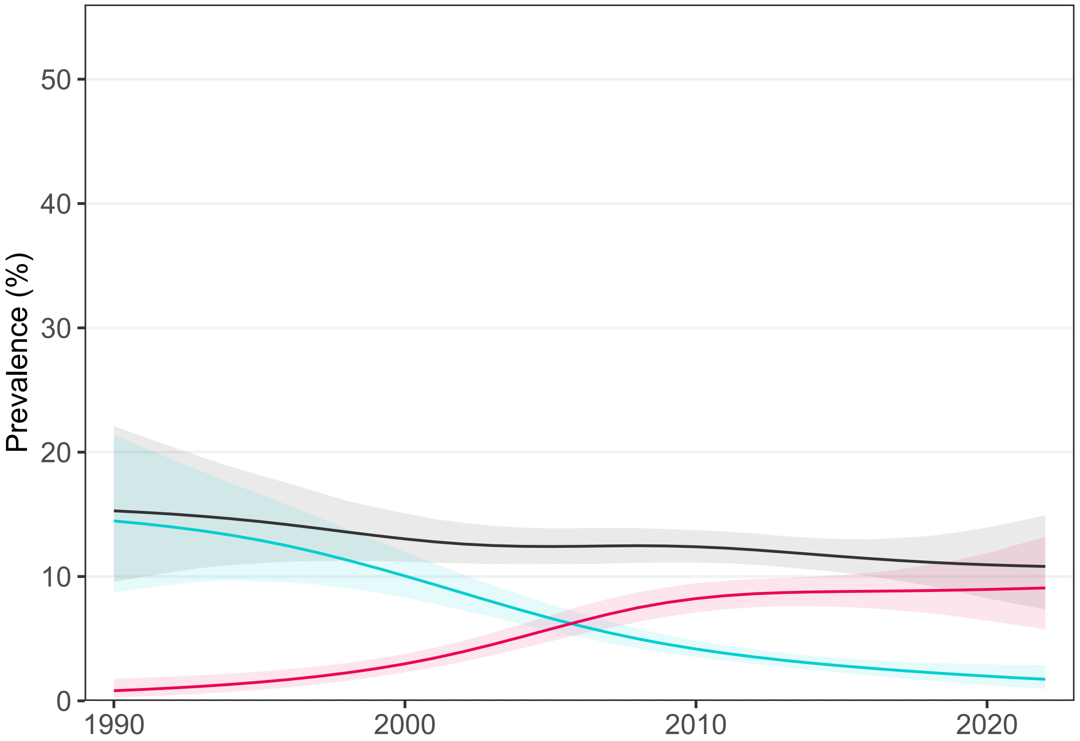


### Boys

25 studies (11 national)

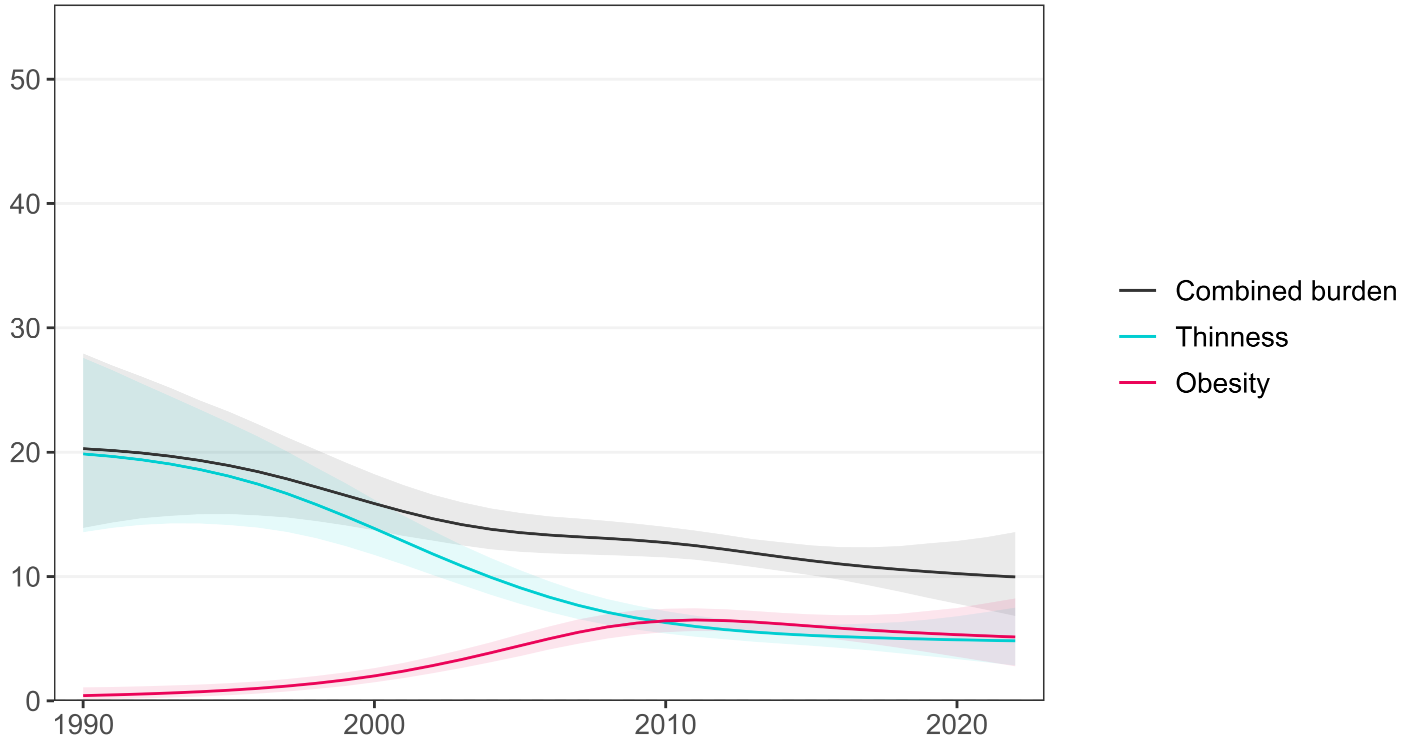


## Adults

### Women

22 studies (12 national)

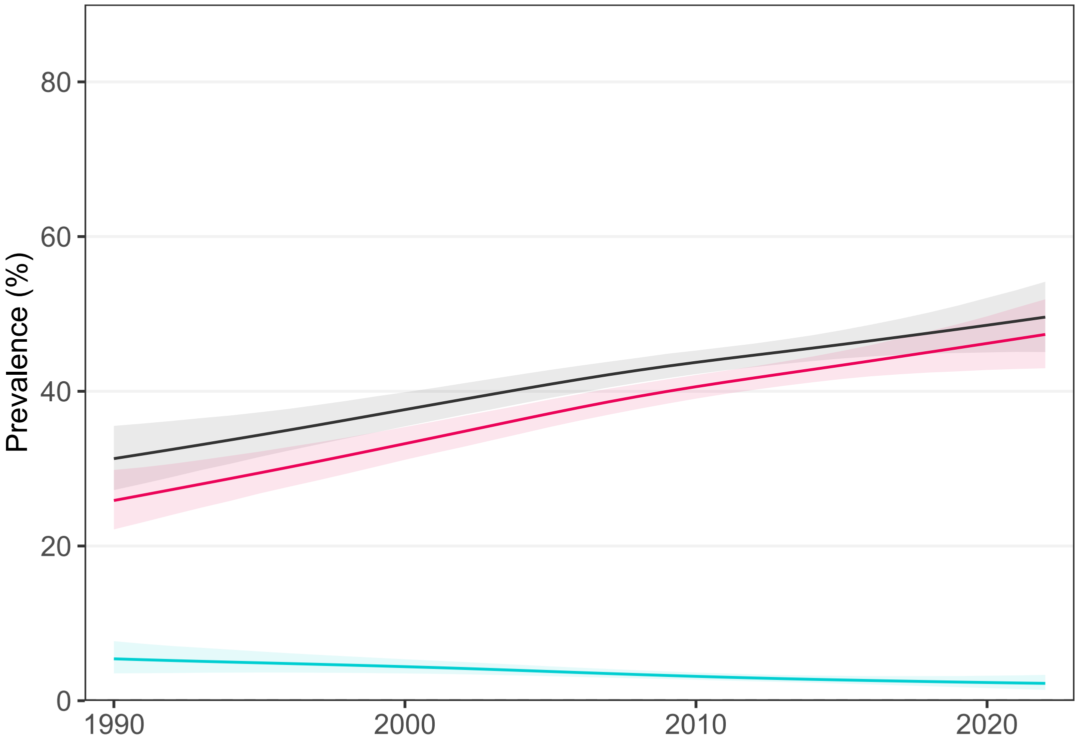


### Men

22 studies (12 national)

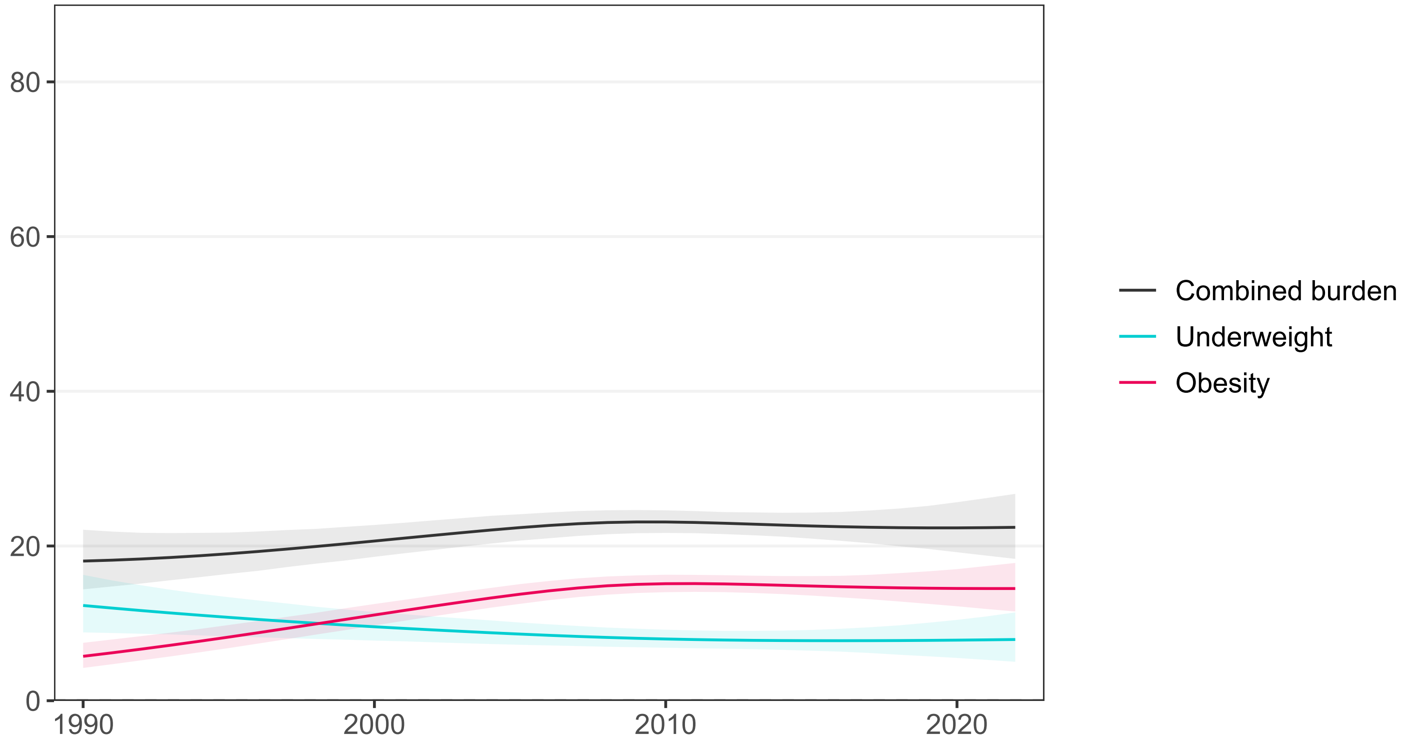

# South Korea

## School-aged children and adolescents

### Girls

29 studies (28 national)

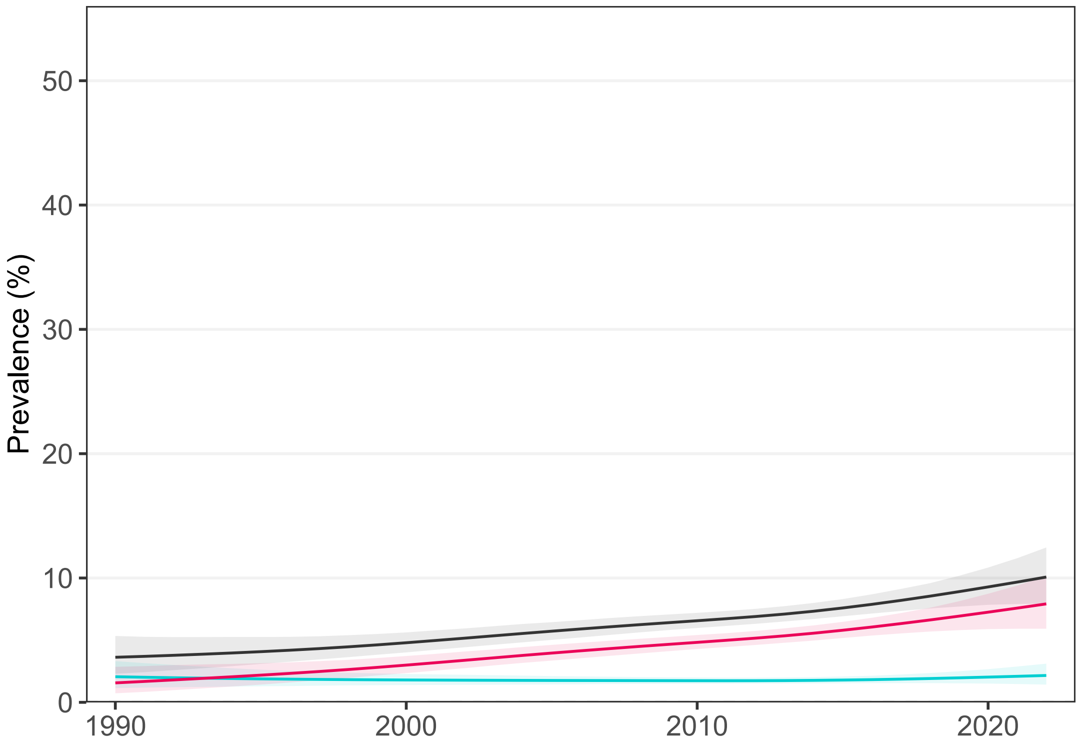


### Boys

48 studies (47 national)

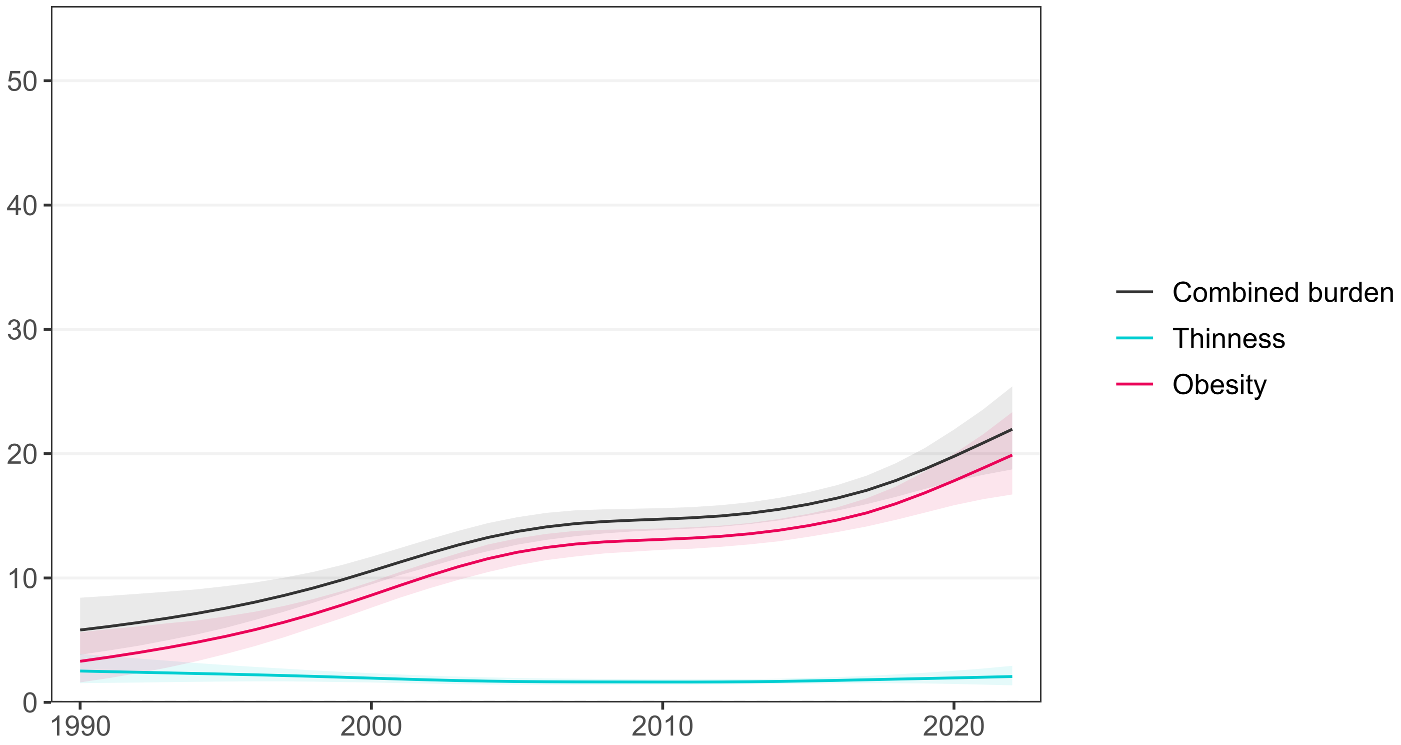


## Adults

### Women

41 studies (38 national)

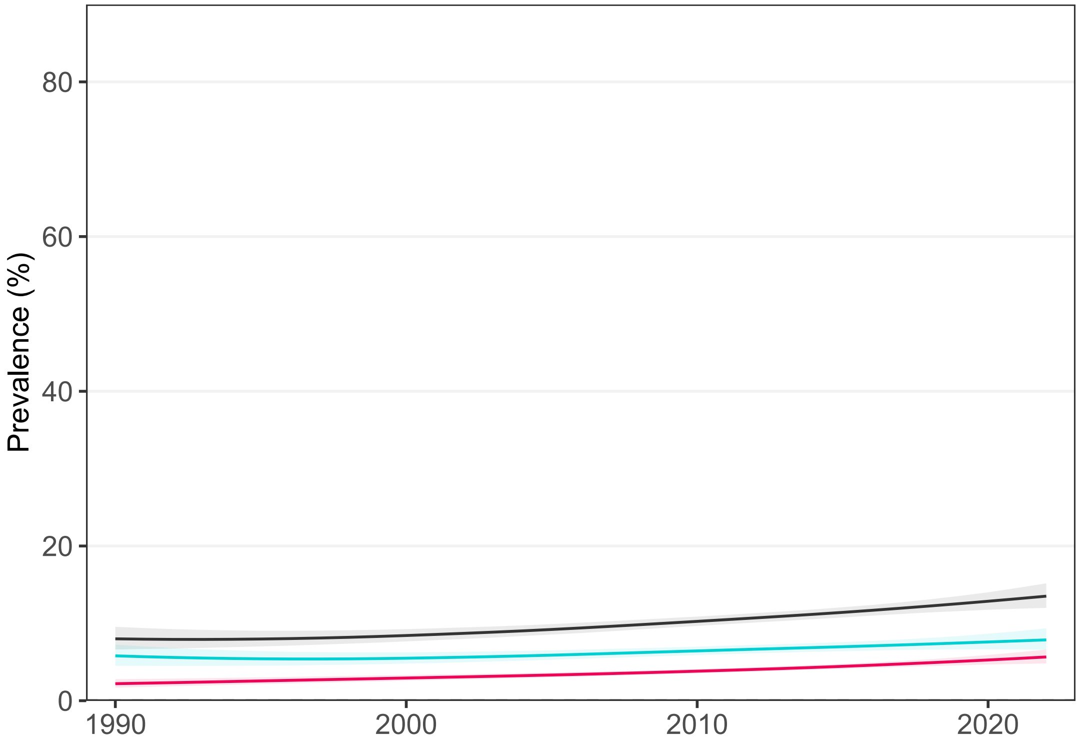


### Men

60 studies (57 national)

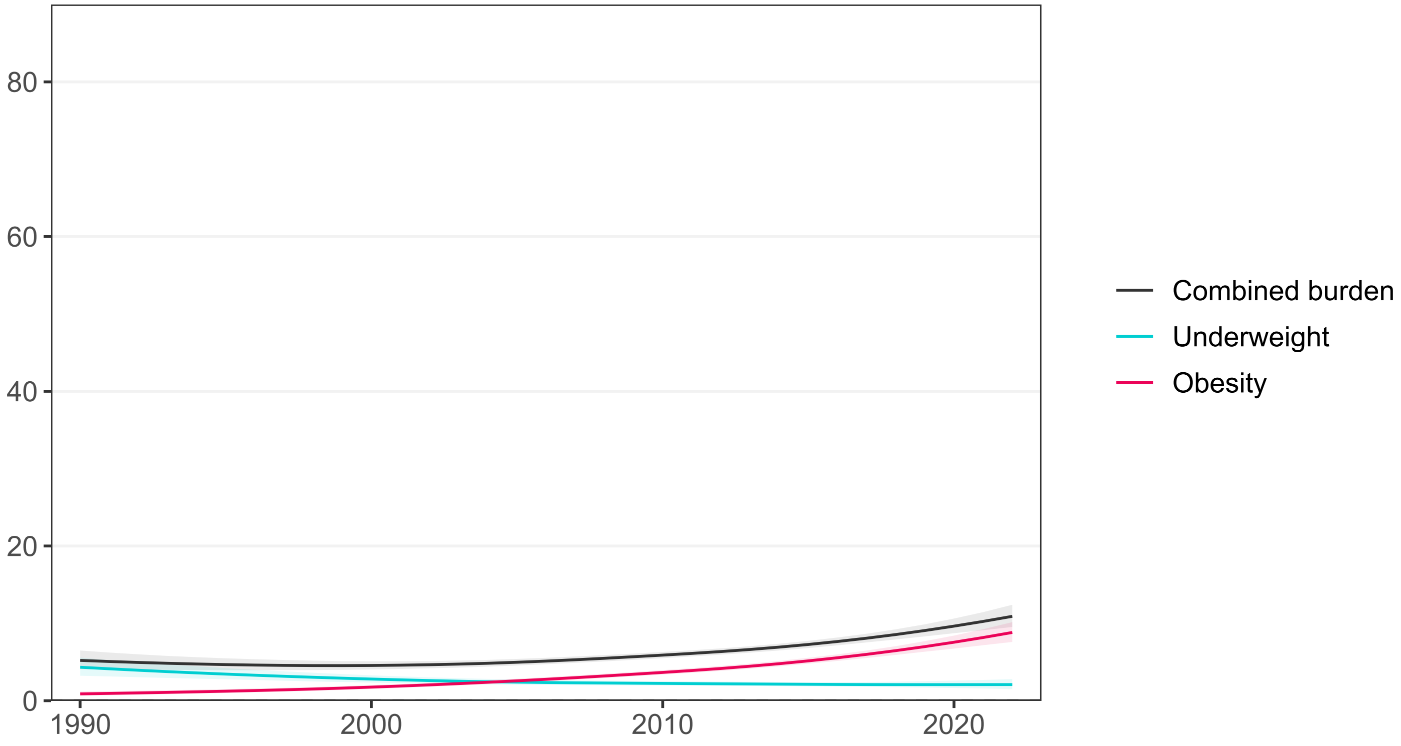

# South Sudan

## School-aged children and adolescents

### Girls
1 study (0 national)

### Boys
1 study (0 national)

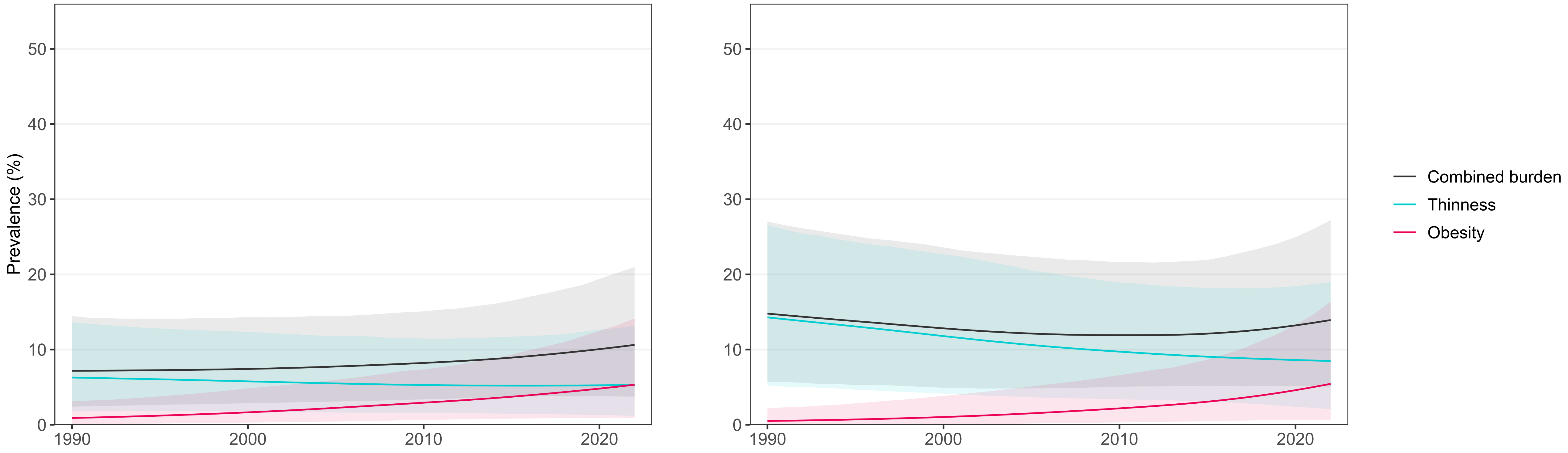


## Adults

### Women
1 study (0 national)

### Men
1 study (0 national)

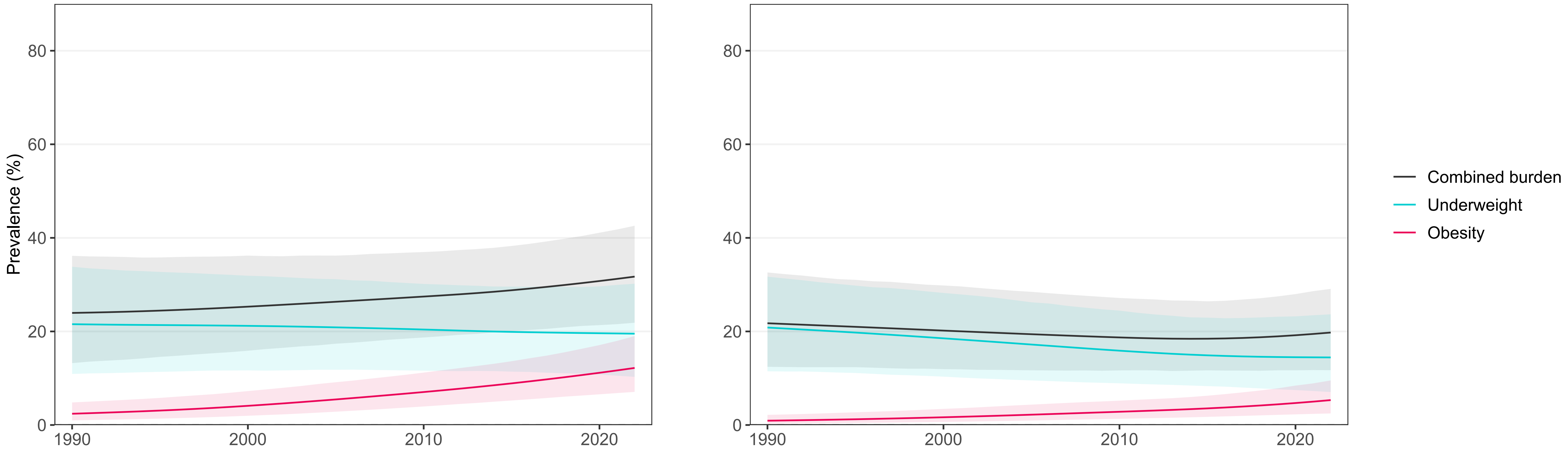

# Spain

## School-aged children and adolescents

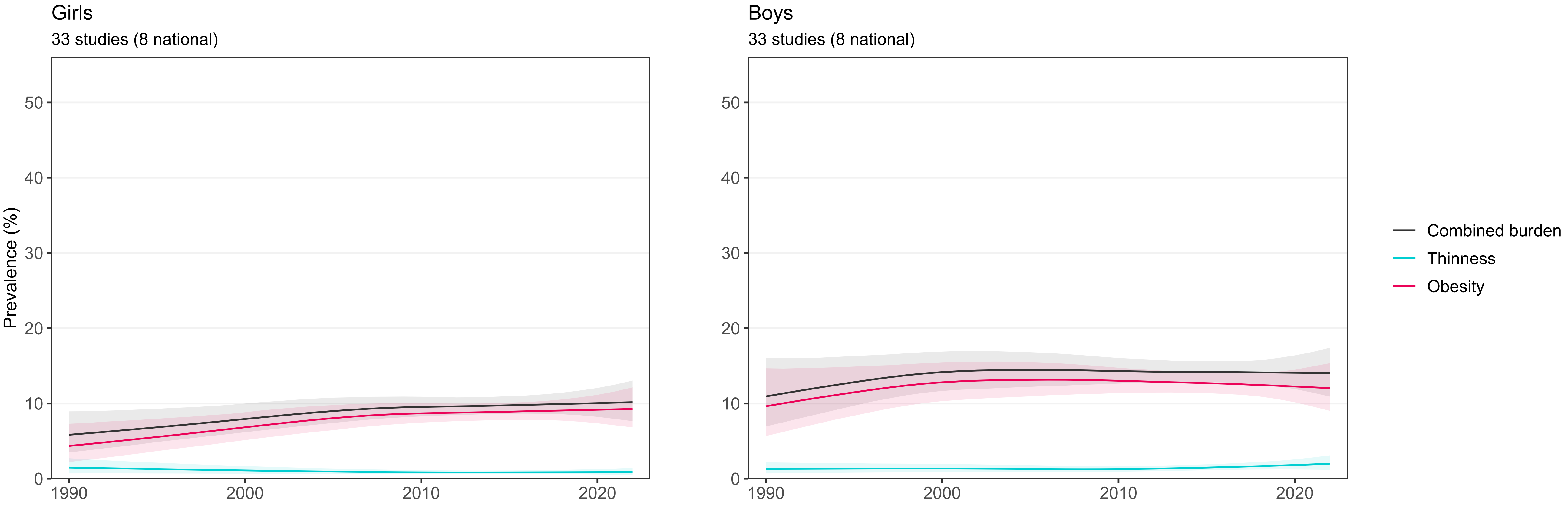


## Adults

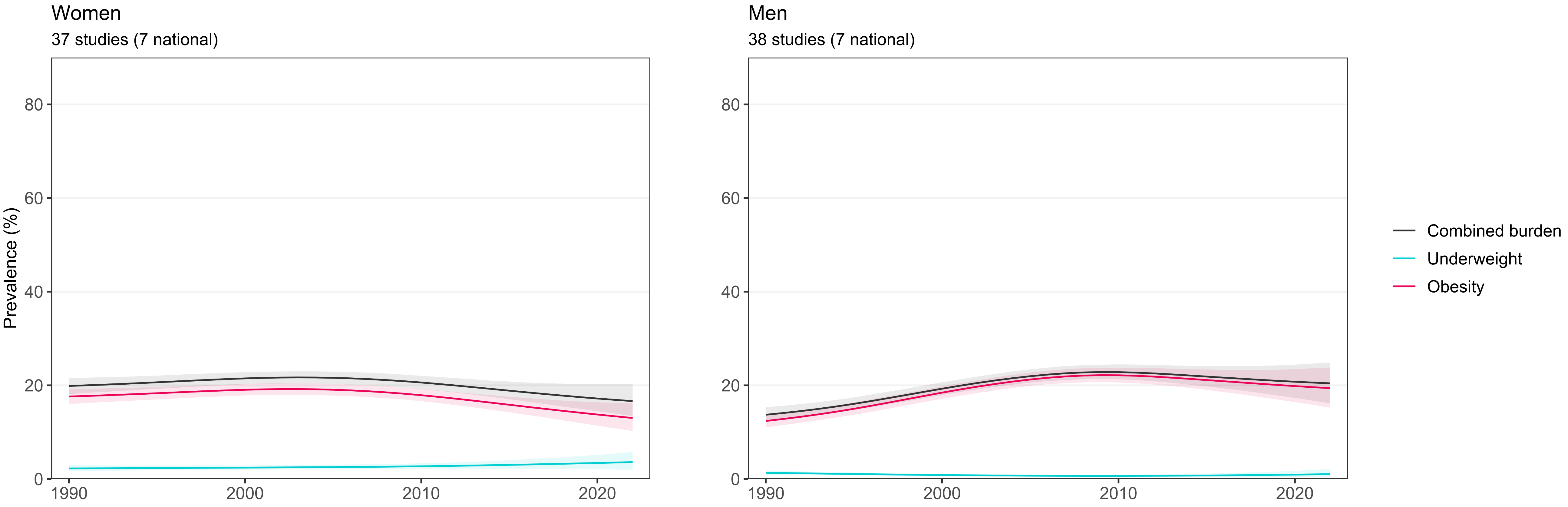

# Sri Lanka

## School-aged children and adolescents

### Girls

5 studies (5 national)

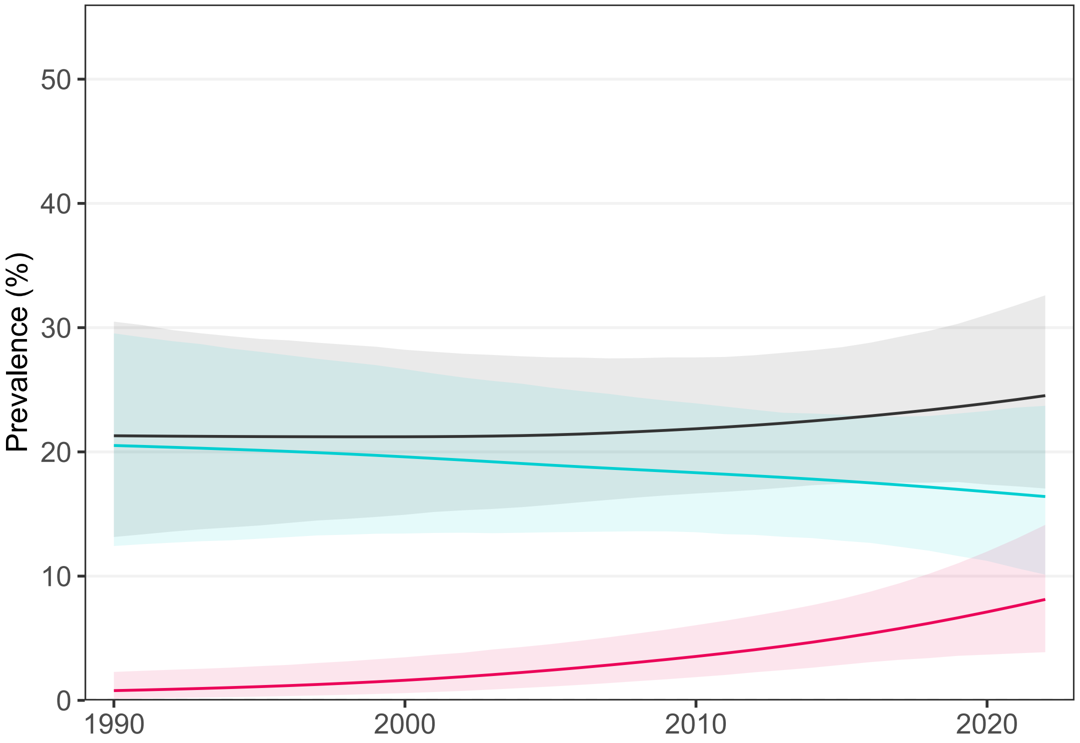


### Boys

5 studies (5 national)

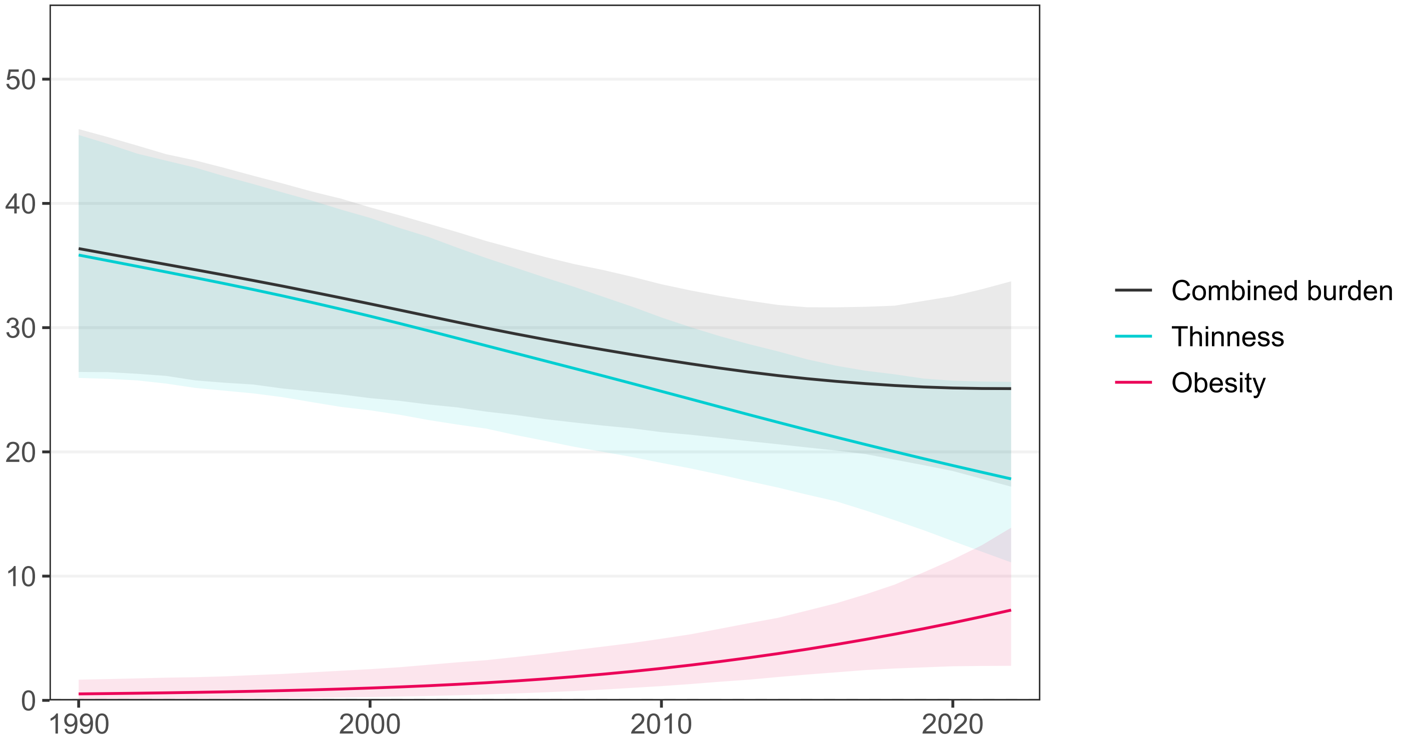


## Adults

### Women

9 studies (5 national)

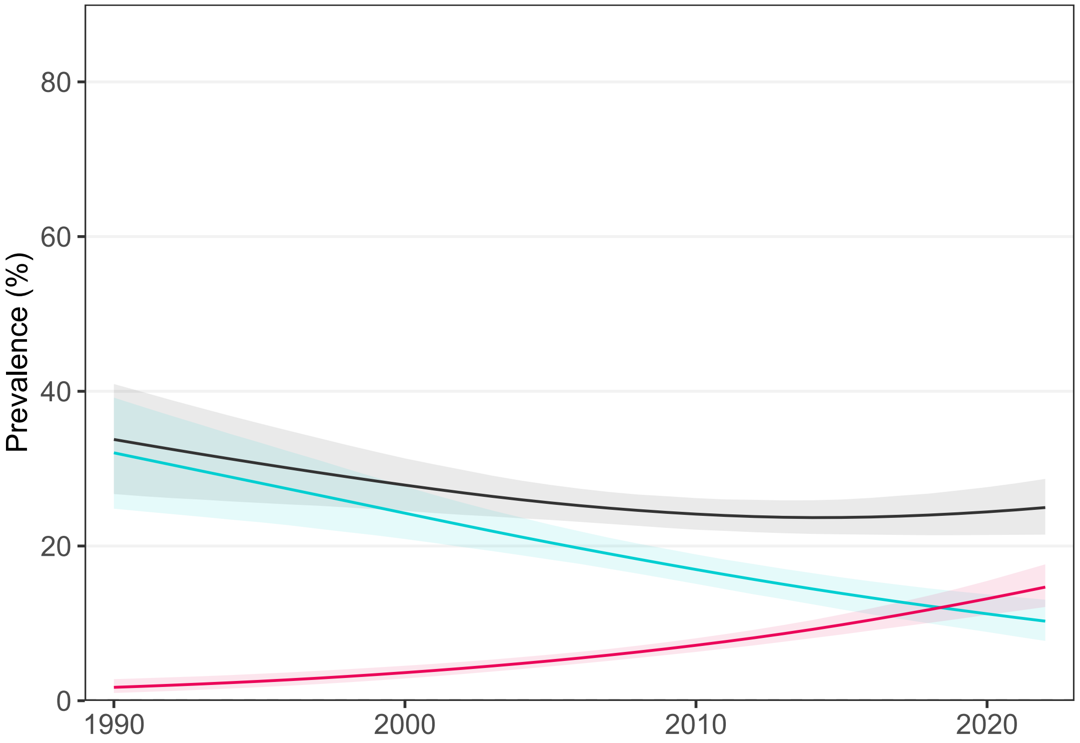


### Men

8 studies (4 national)

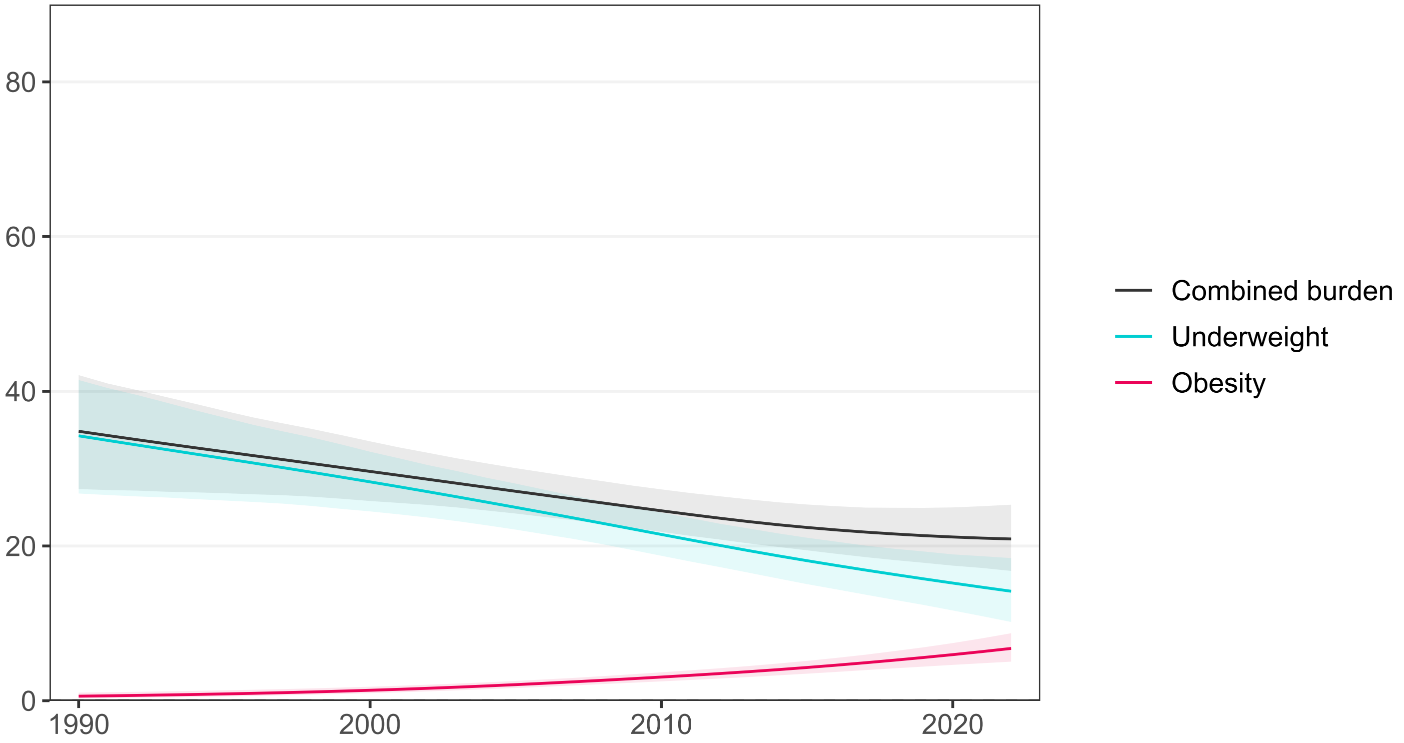

# St. Kitts & Nevis

## School-aged children and adolescents

### Girls

1 study (1 national)

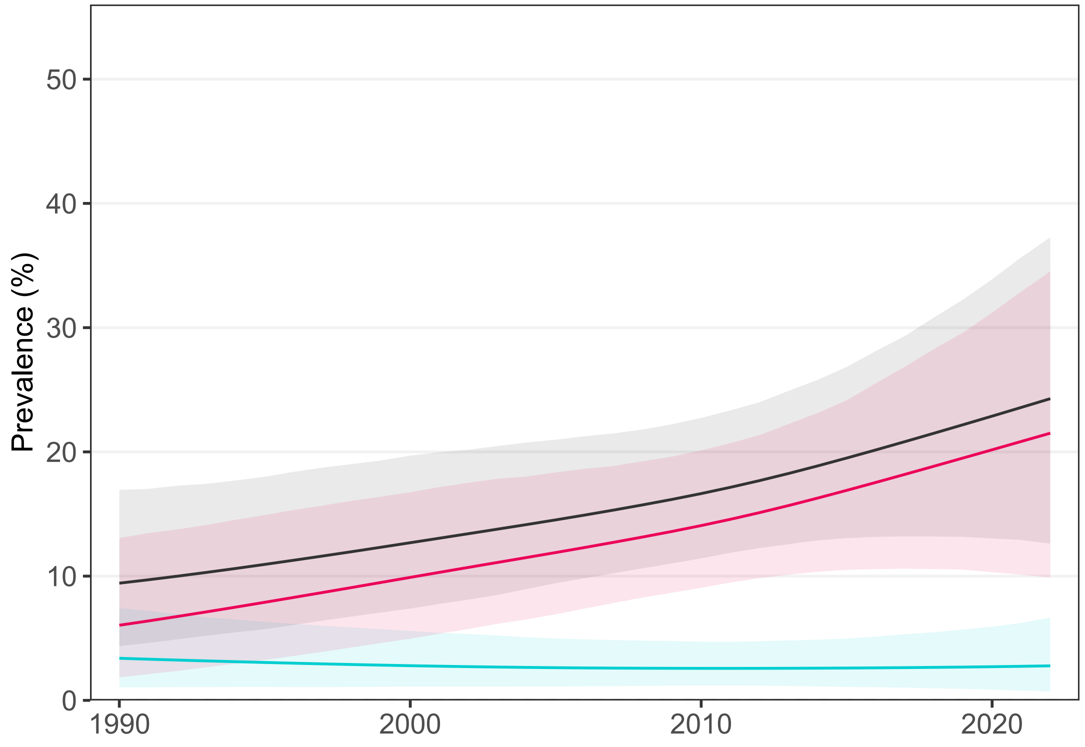


### Boys

1 study (1 national)

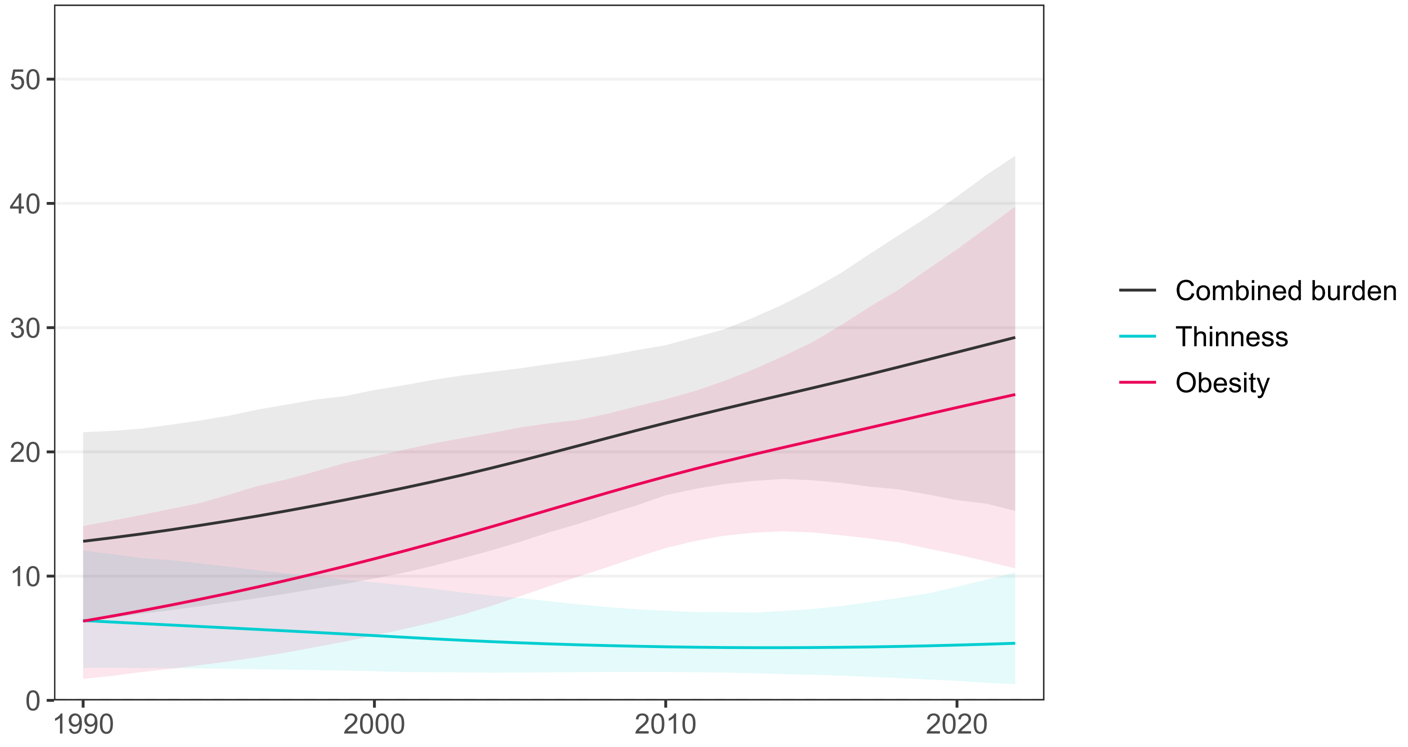


## Adults

### Women

1 study (0 national)

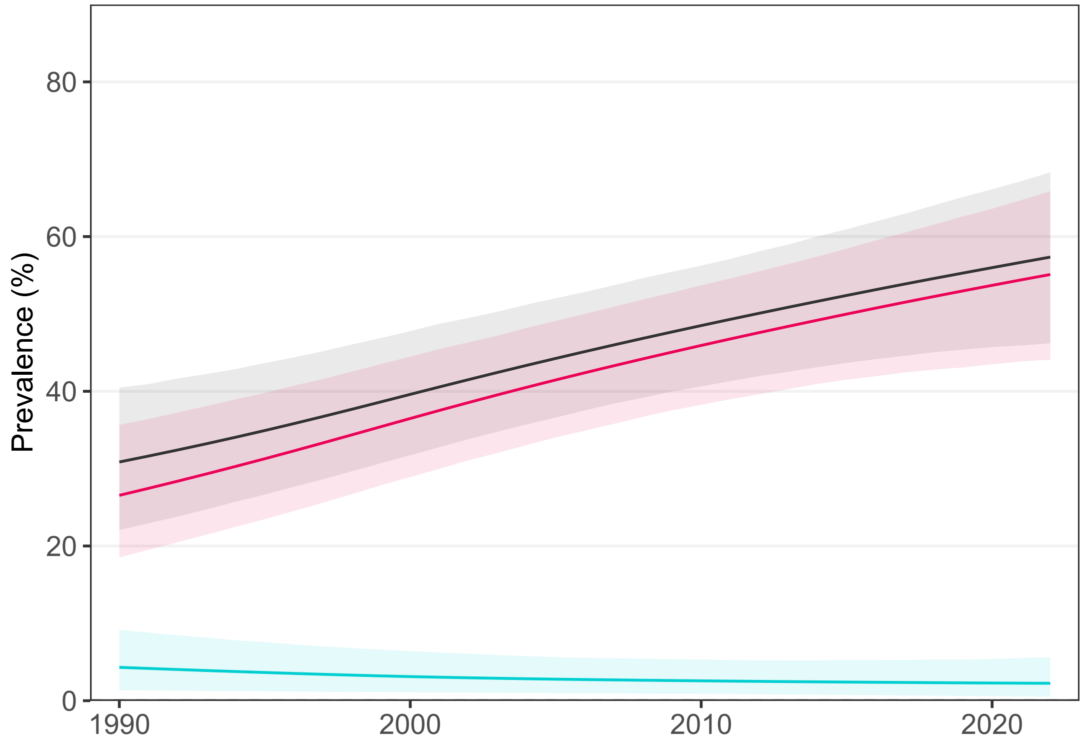


### Men

1 study (0 national)

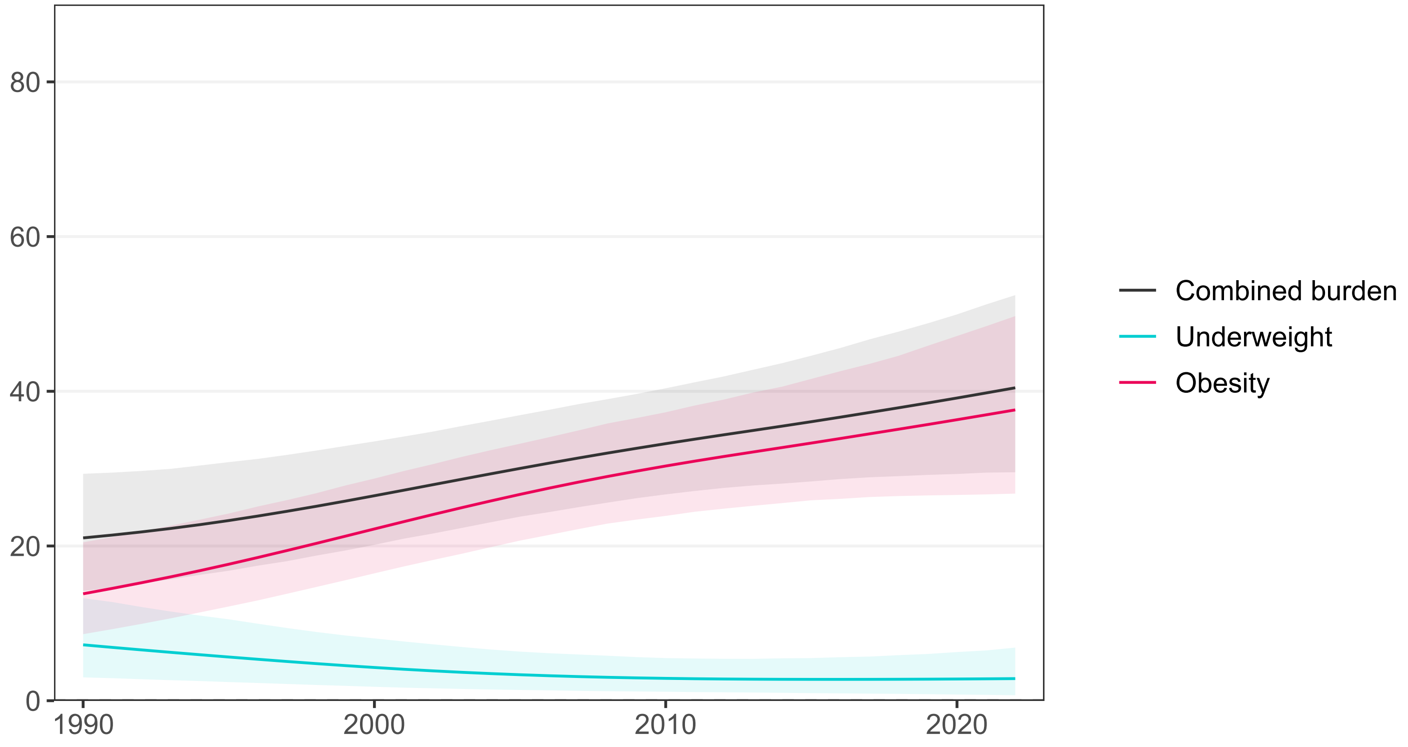

# St. Lucia

## School-aged children and adolescents

### Girls

1 study (1 national)

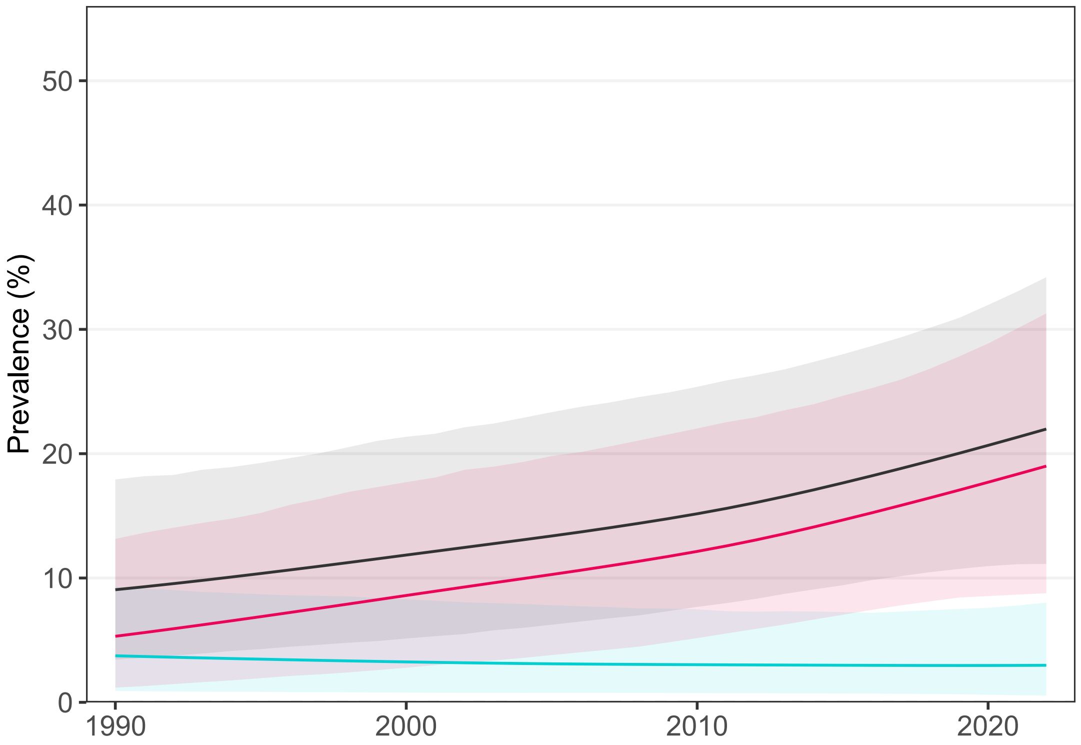


### Boys

1 study (1 national)

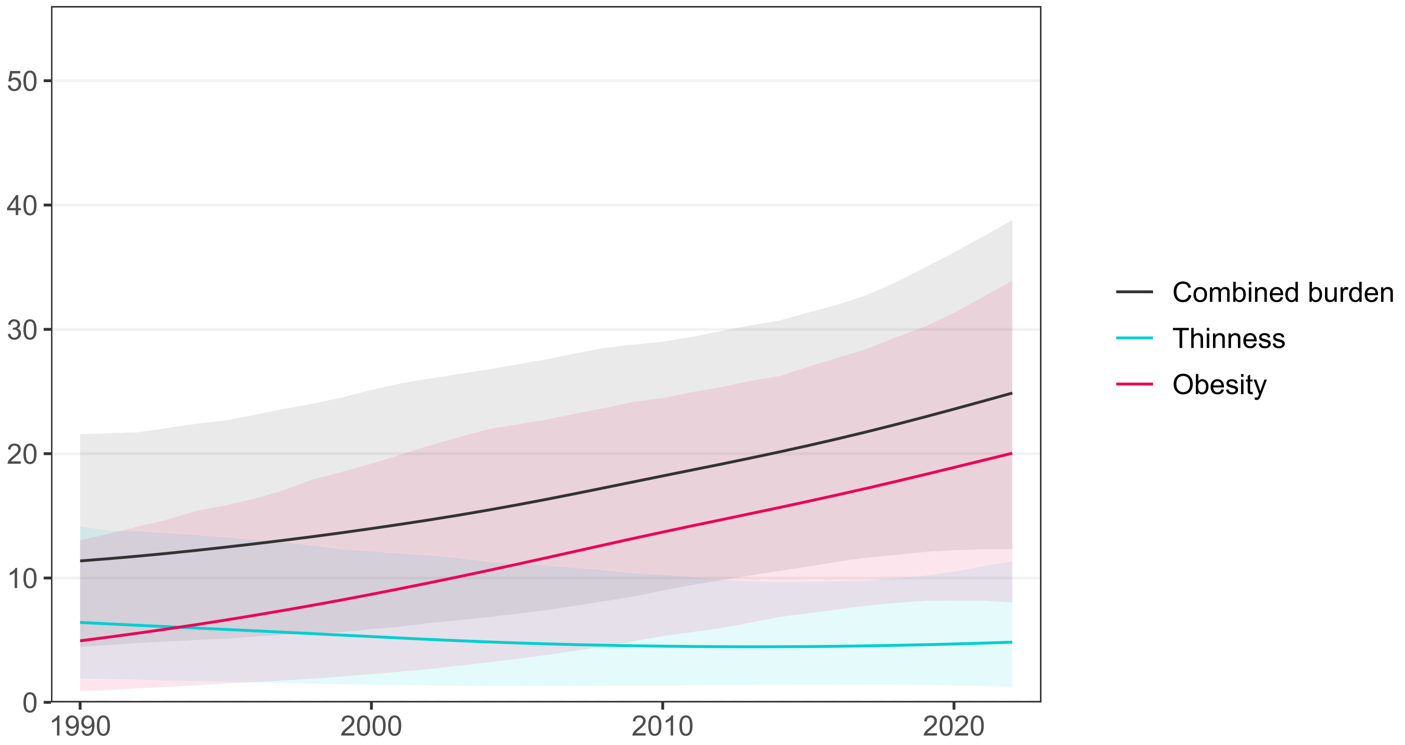


## Adults

### Women

4 studies (3 national)

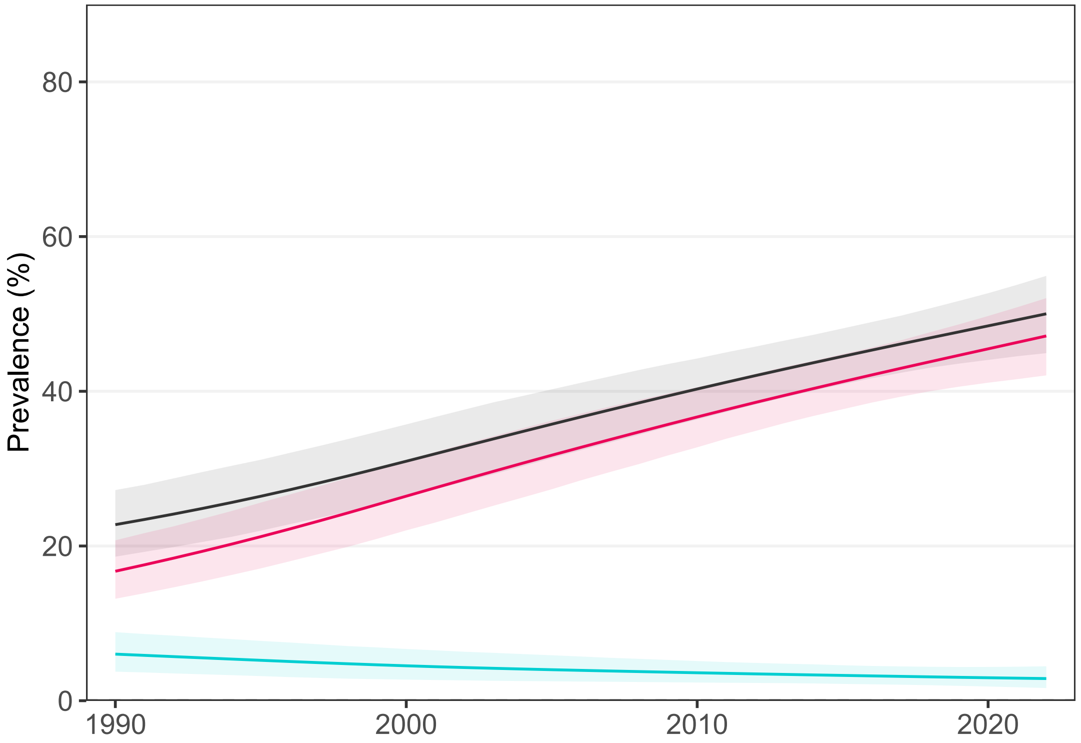


### Men

4 studies (3 national)

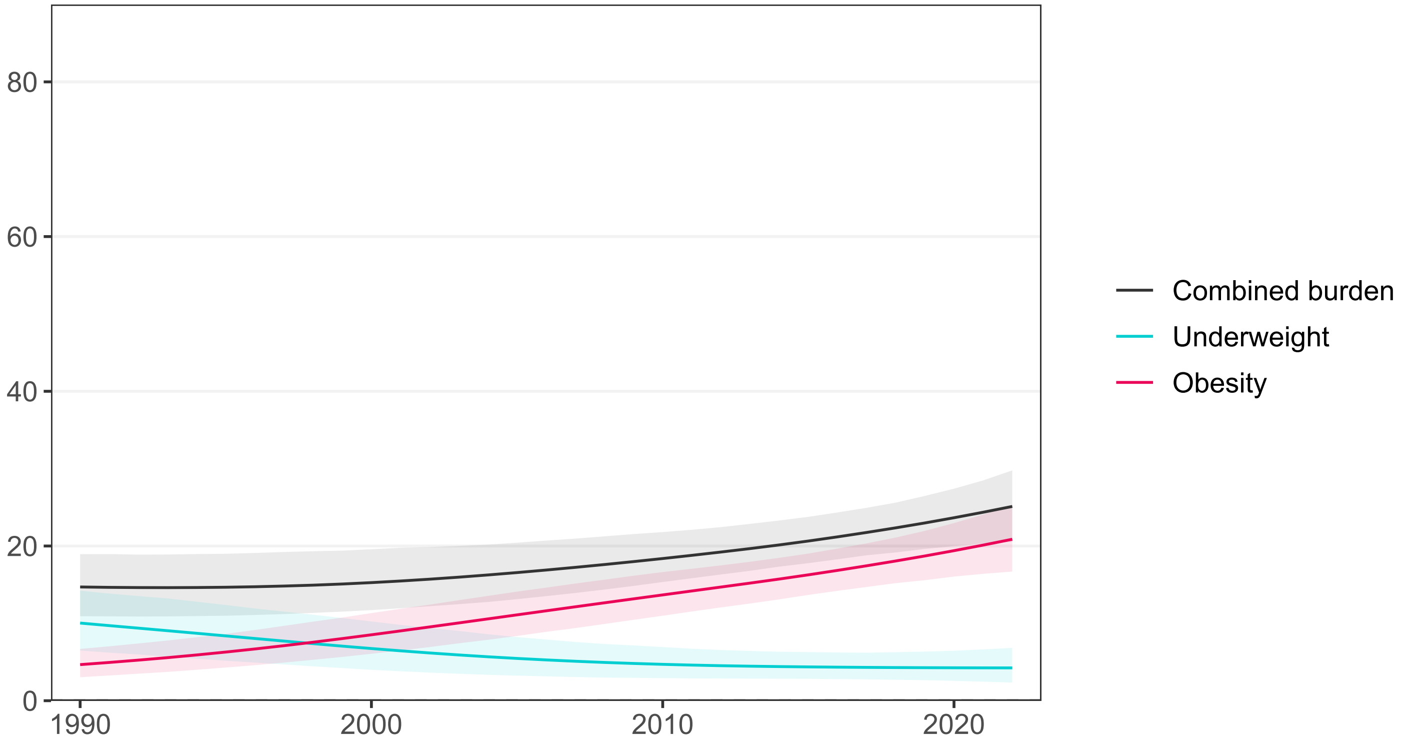

# St. Vincent & the Grenadines

## School-aged children and adolescents

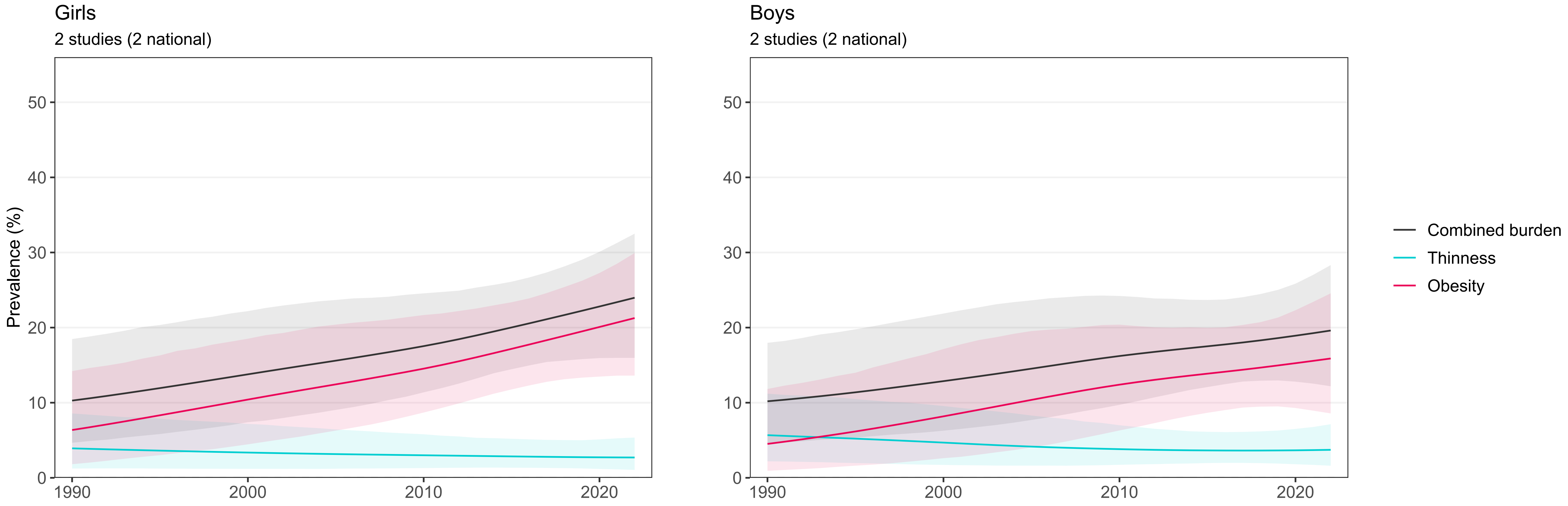


## Adults

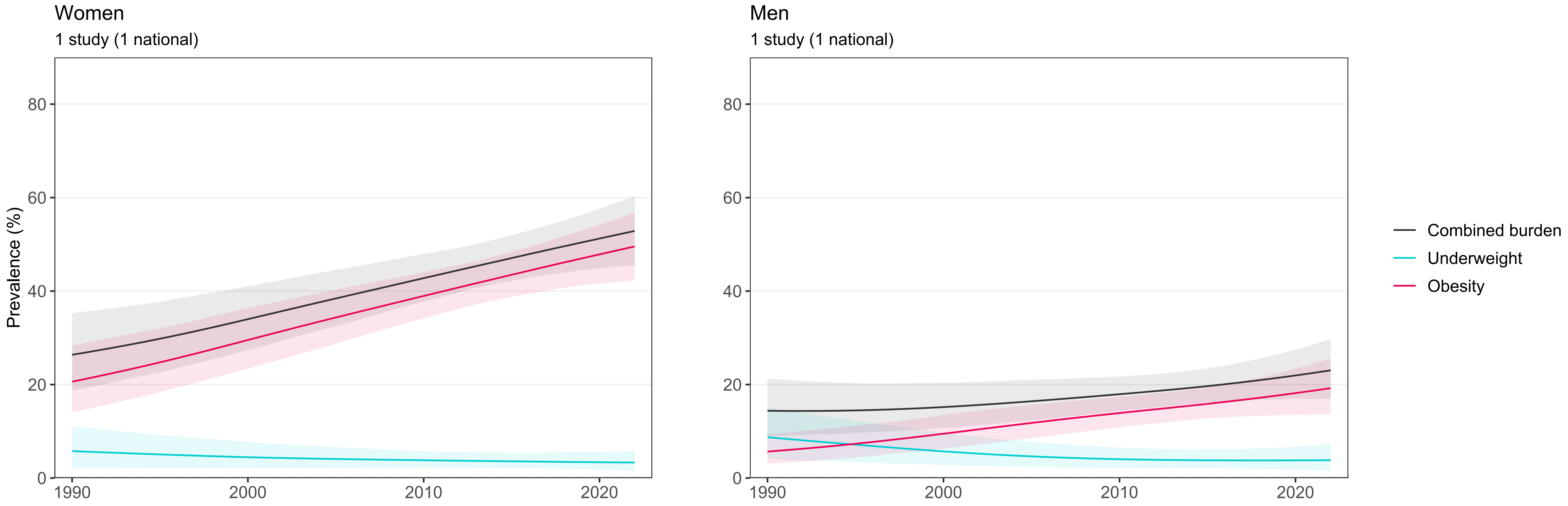

# State of Palestine

## School-aged children and adolescents

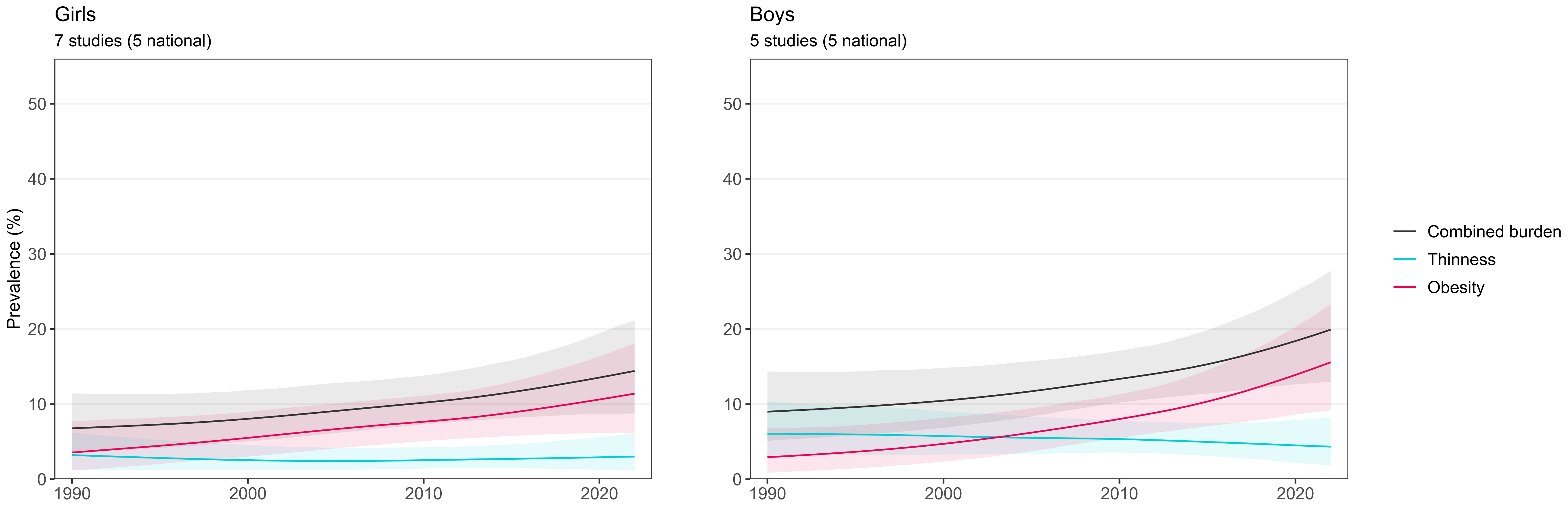


## Adults

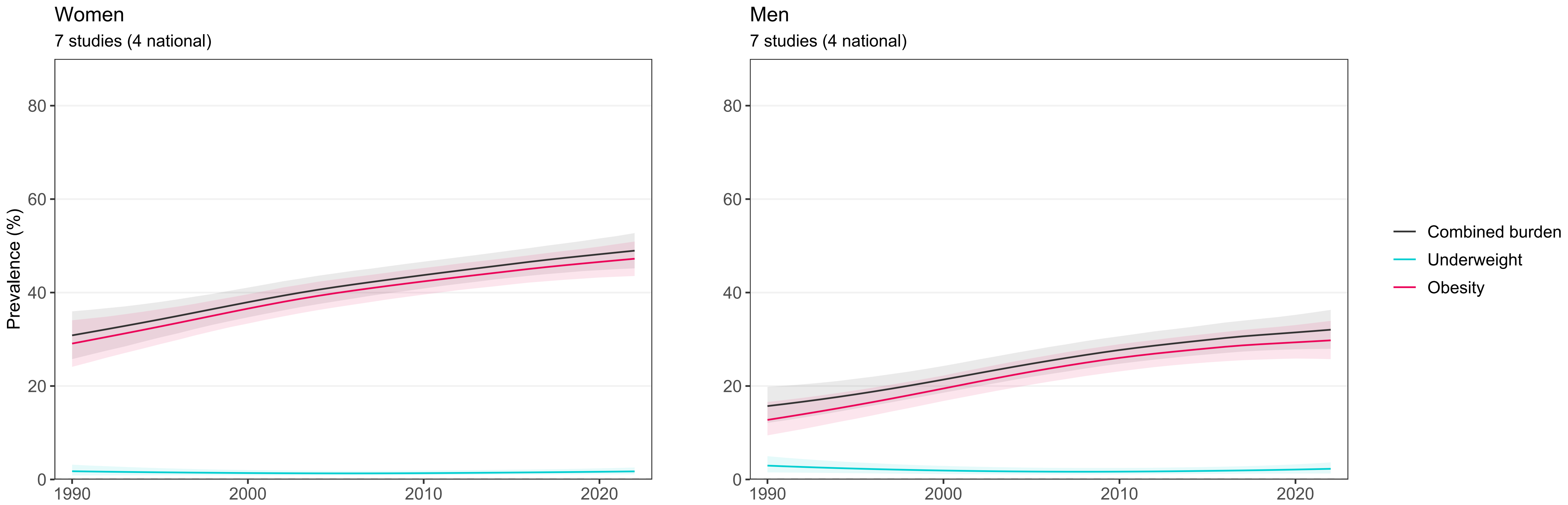

# Sudan

## School-aged children and adolescents

### Girls

1 study (1 national)

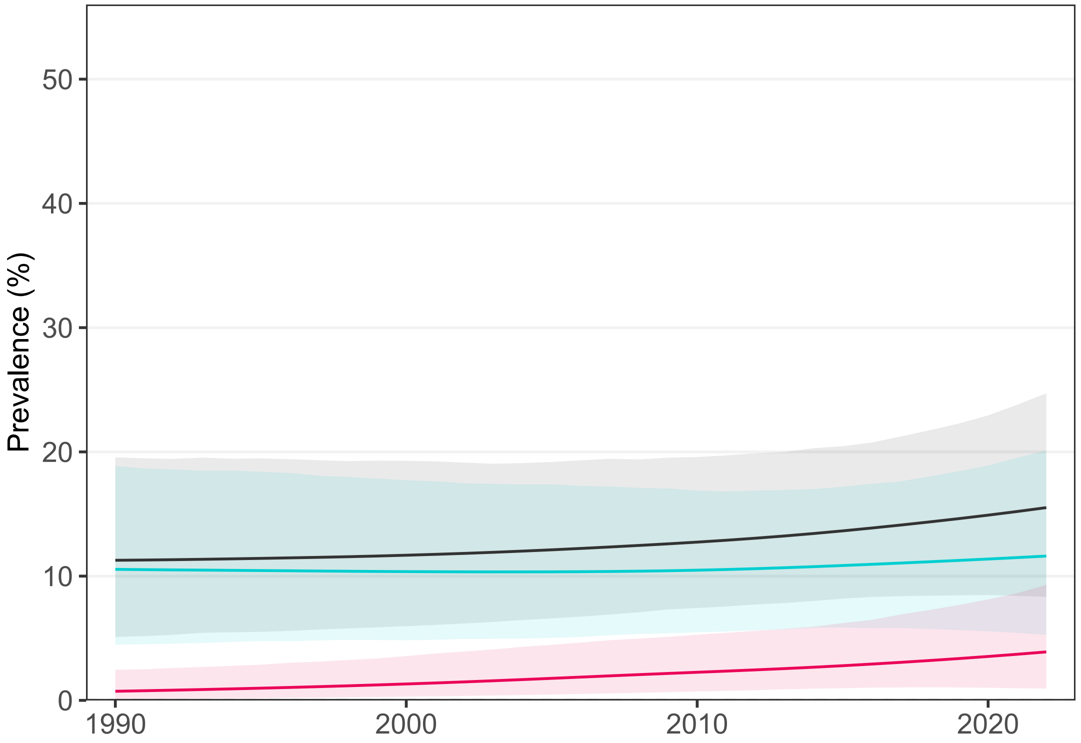


### Boys

1 study (1 national)

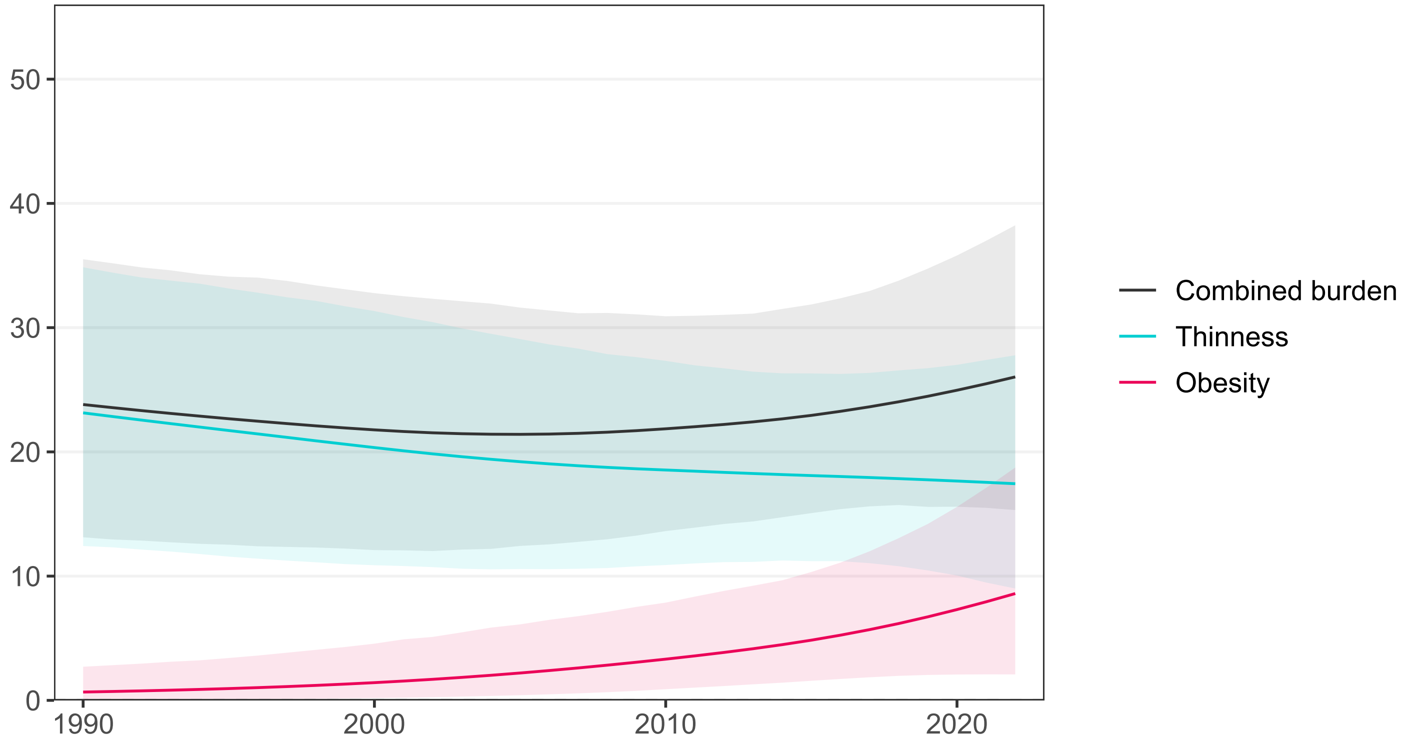


## Adults

### Women

3 studies (1 national)

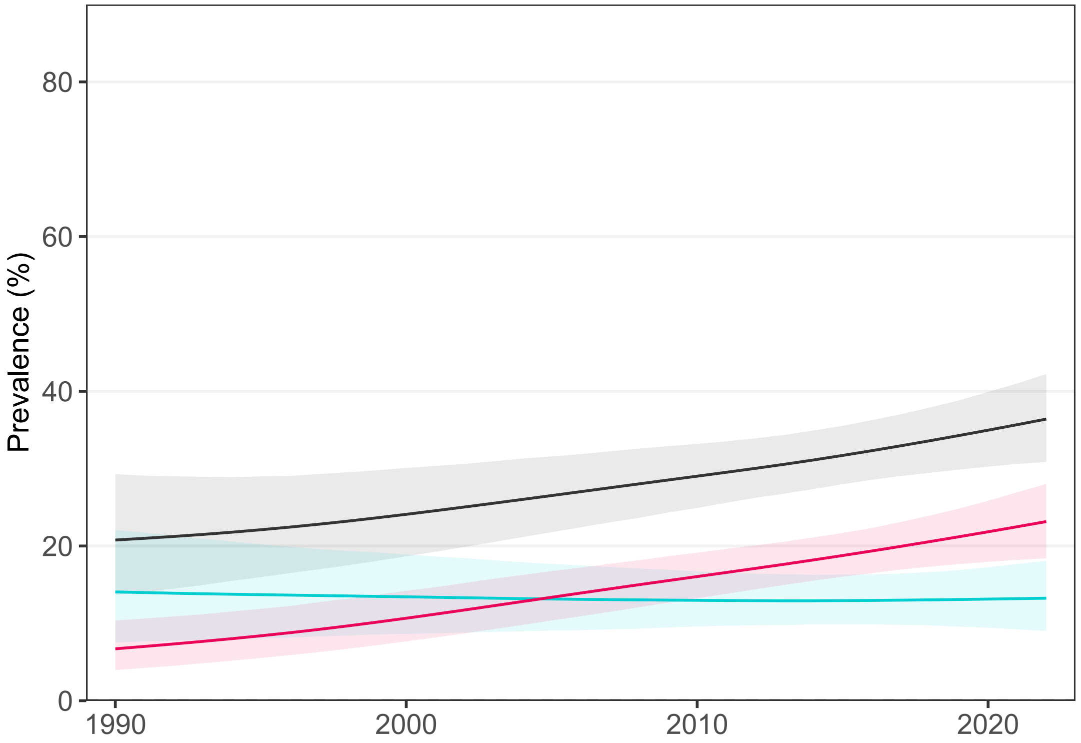


### Men

3 studies (1 national)

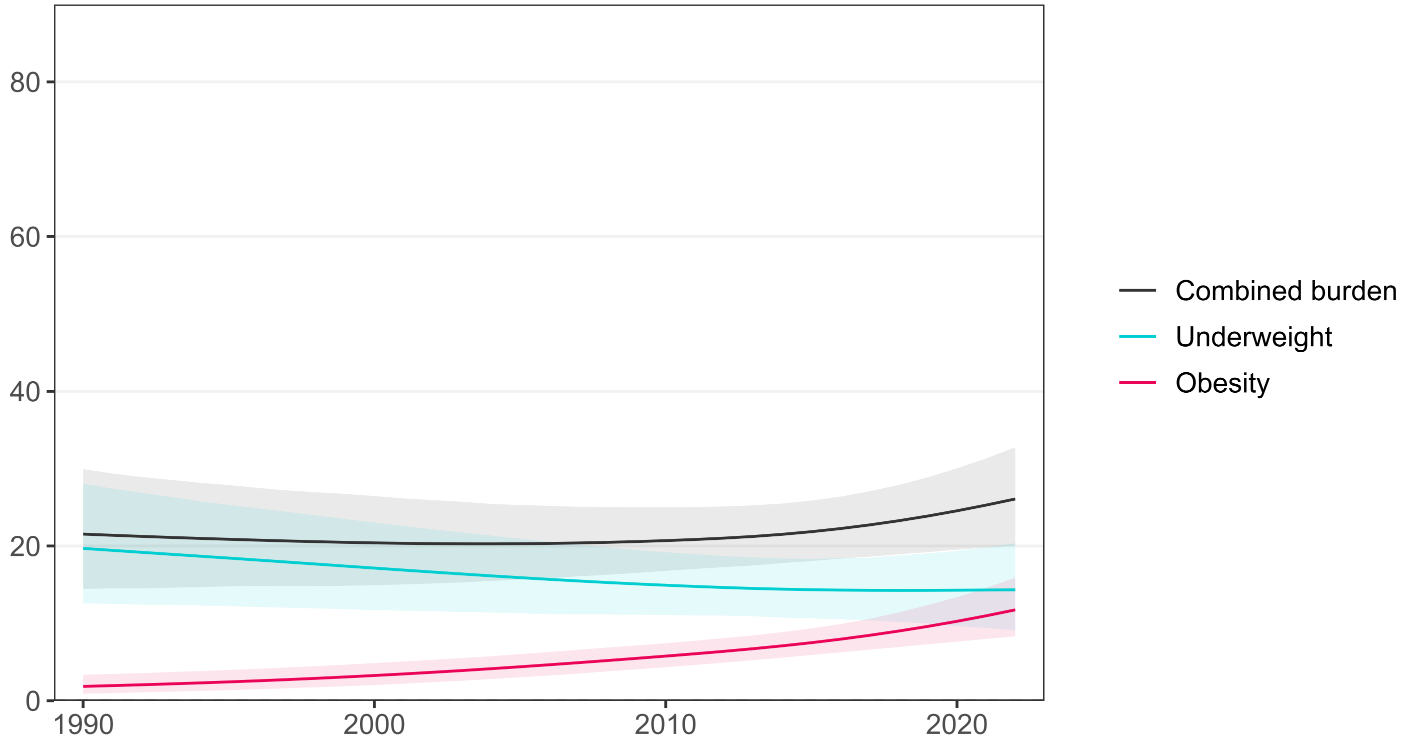

# Suriname

## School-aged children and adolescents

### Girls

1 study (0 national)

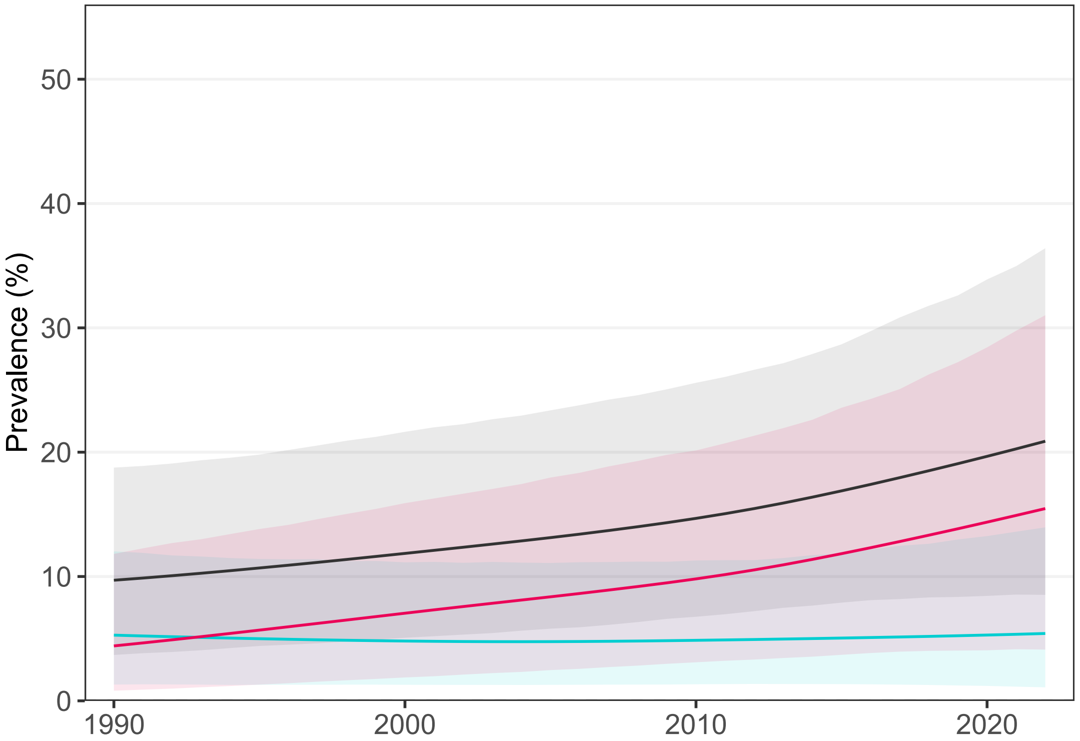


### Boys

1 study (0 national)

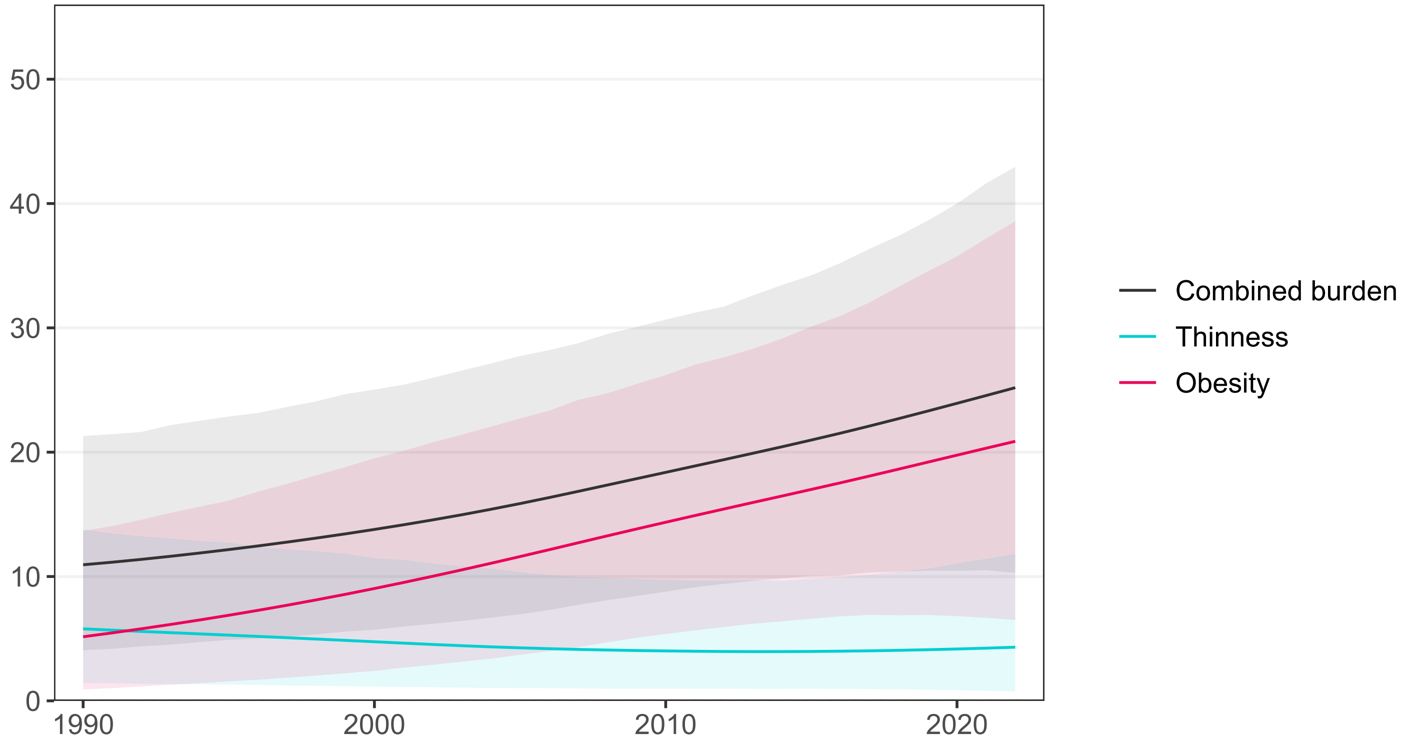


## Adults

### Women

1 study (0 national)

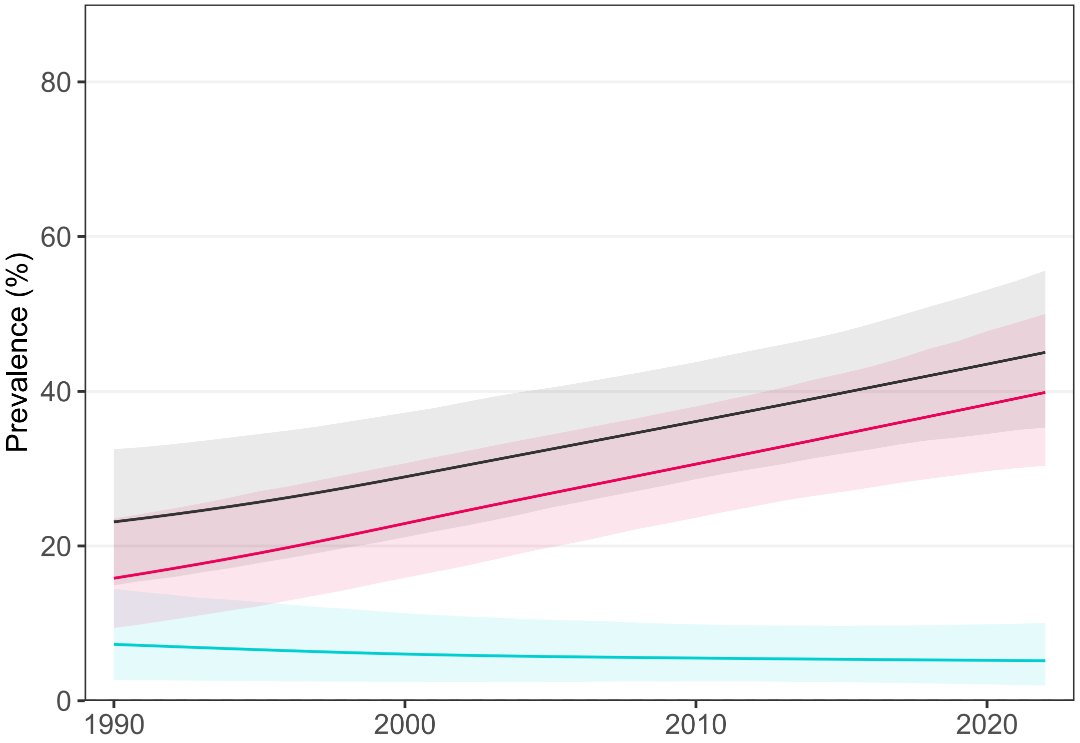


### Men

1 study (0 national)

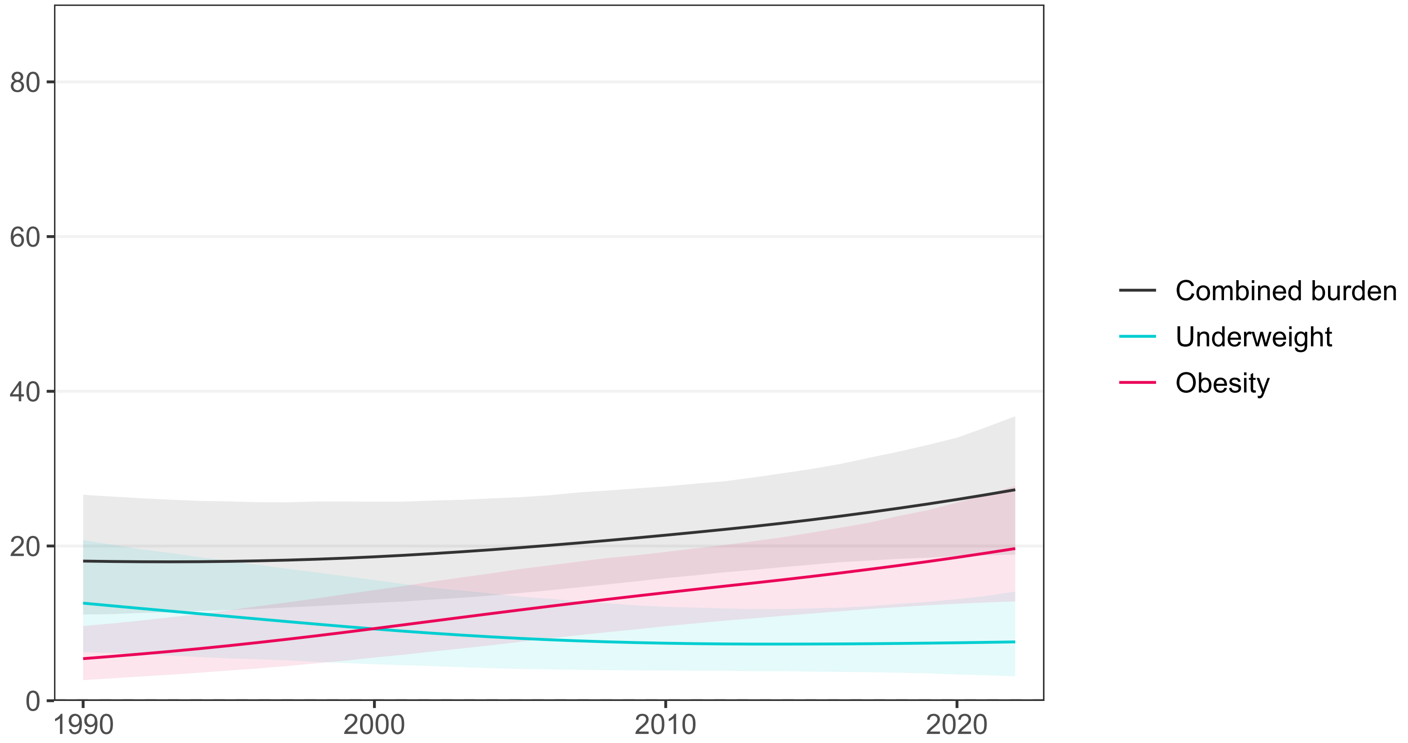

# Sweden

## School-aged children and adolescents

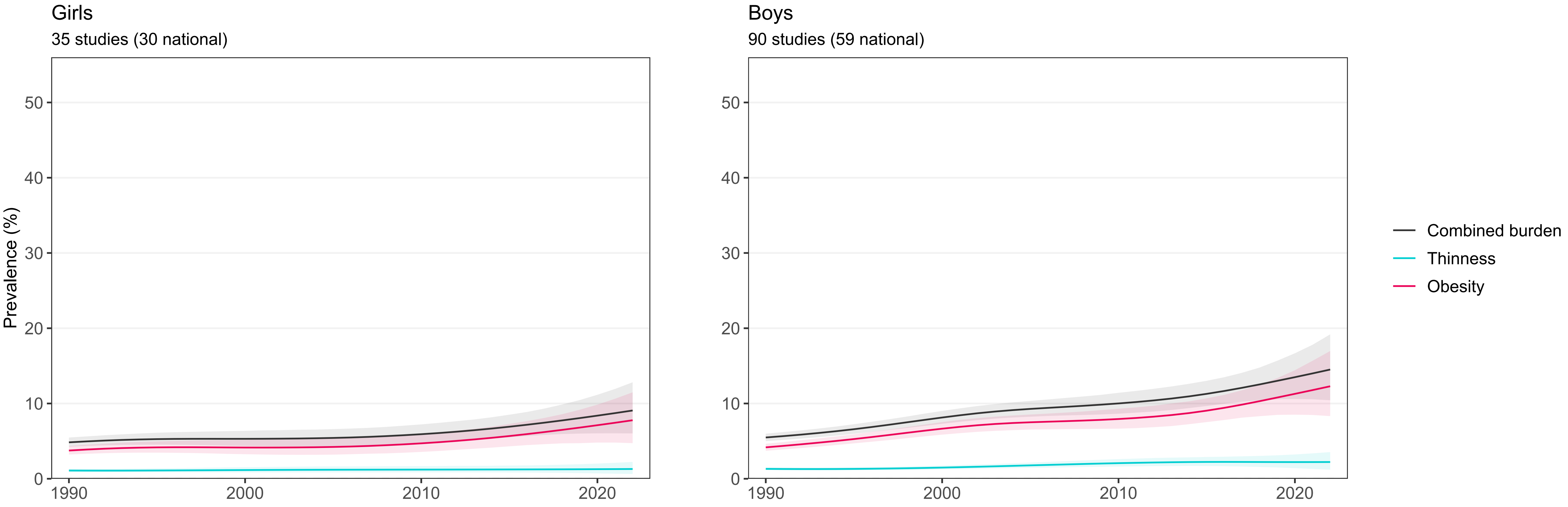


## Adults

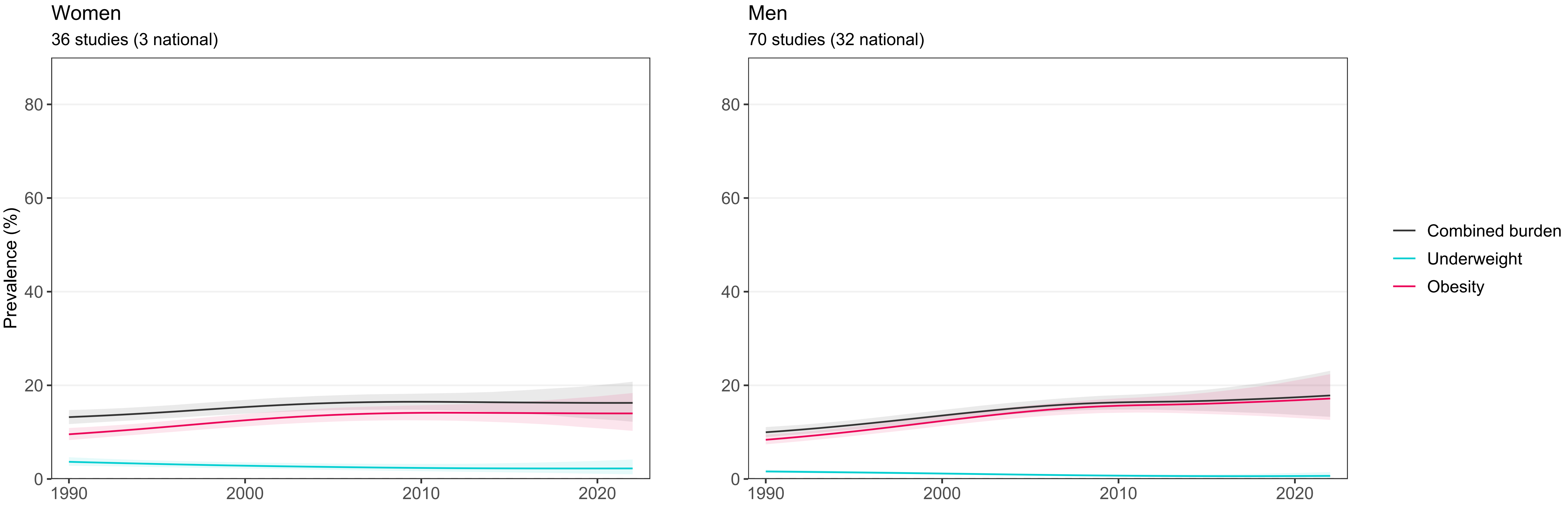

# Switzerland

## School-aged children and adolescents

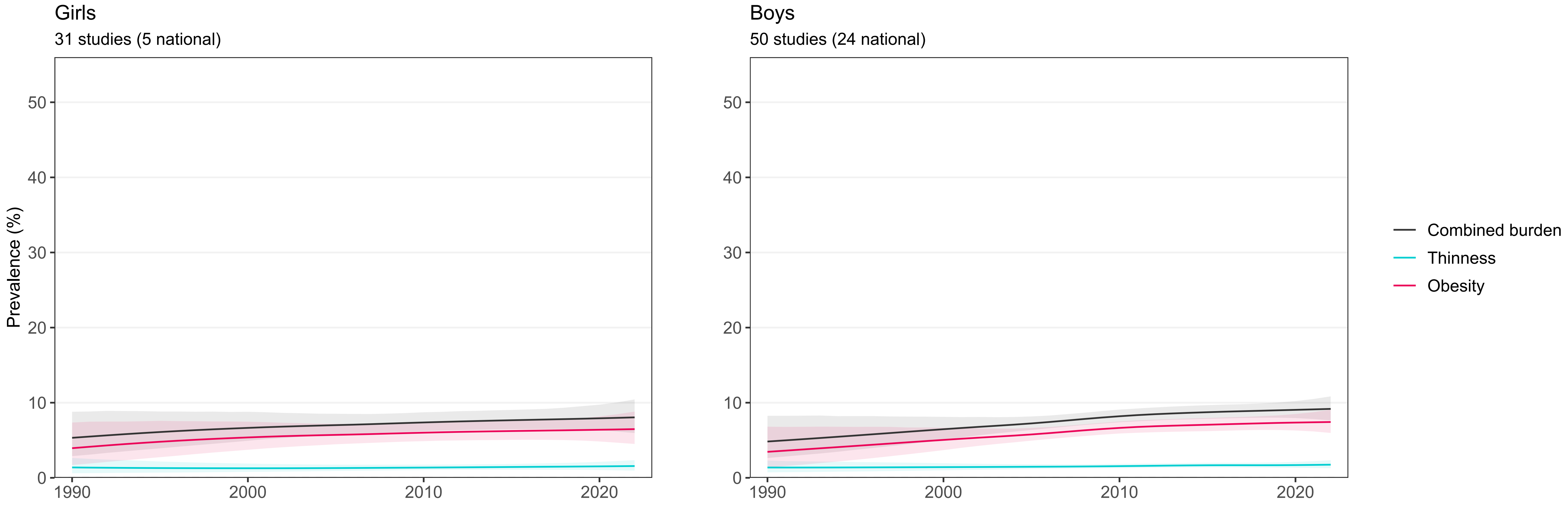


## Adults

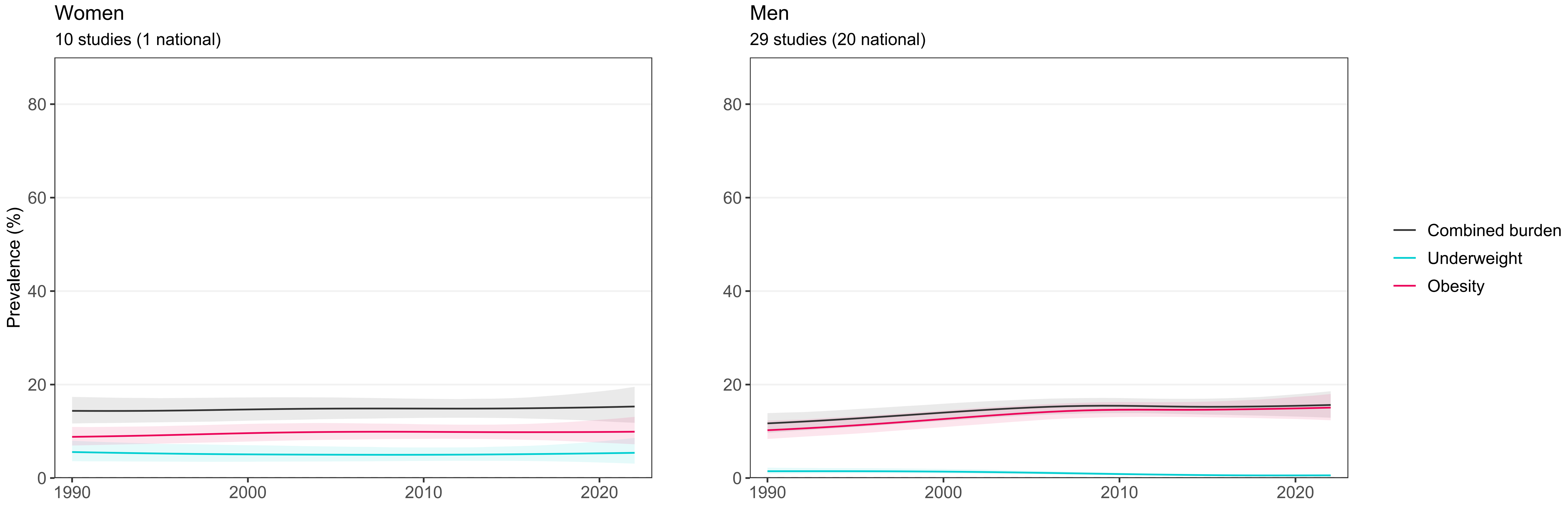

# Syrian Arab Republic

## School-aged children and adolescents

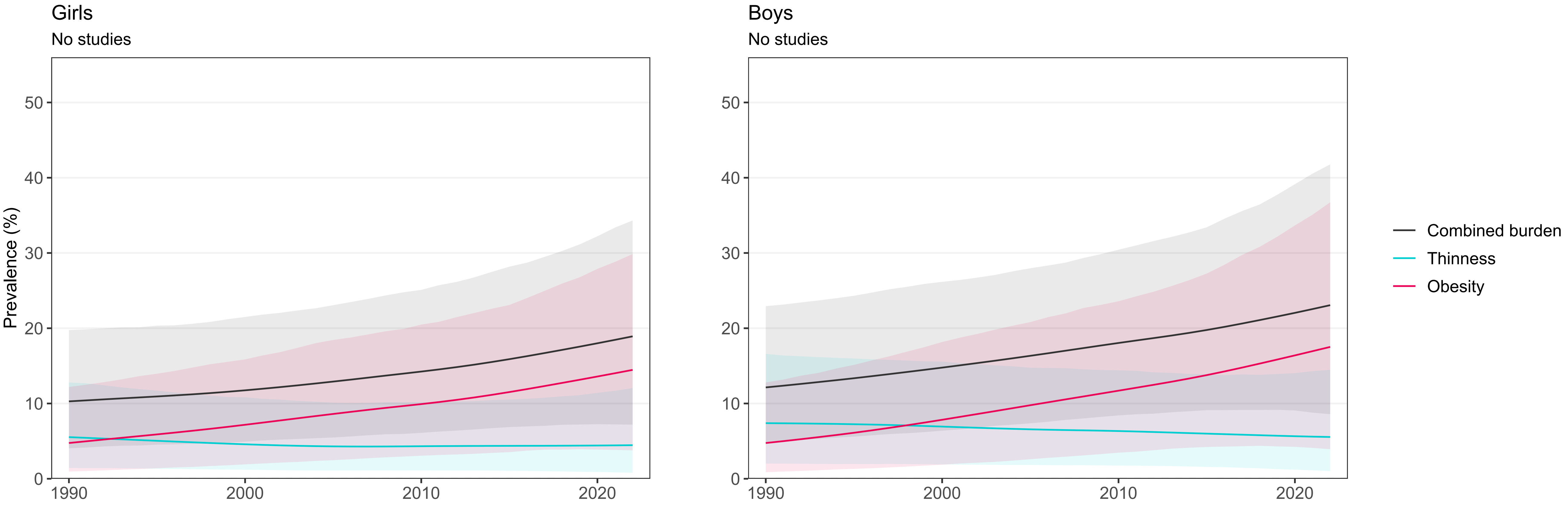


## Adults

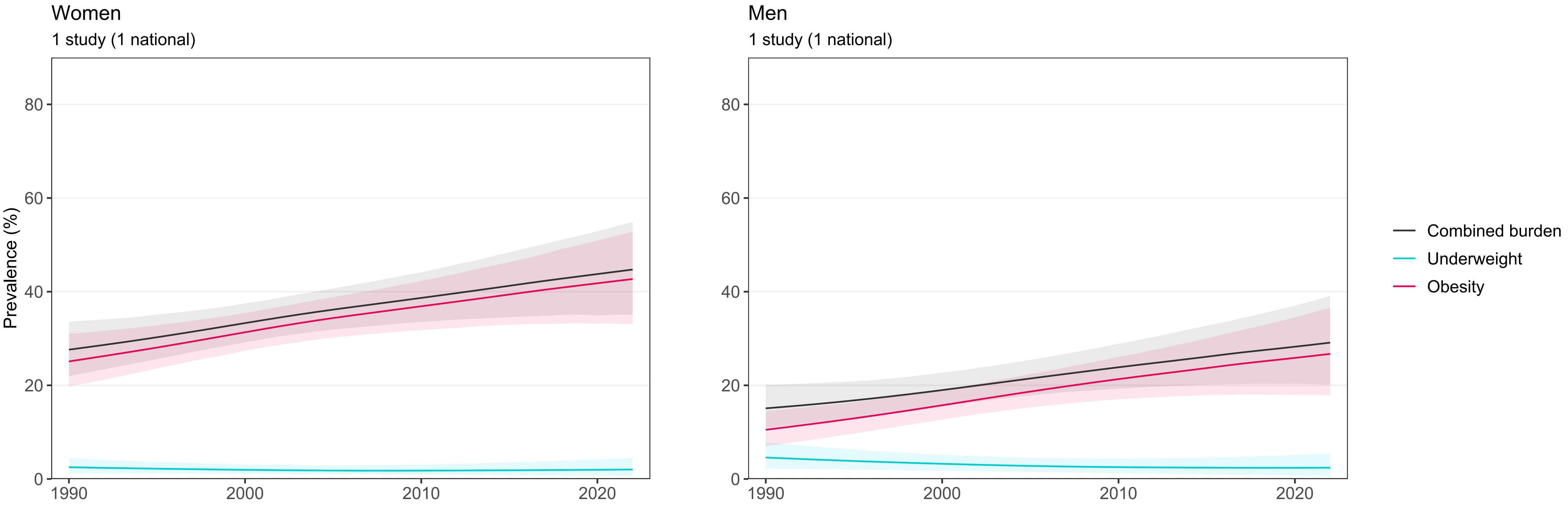

# Taiwan

## School-aged children and adolescents

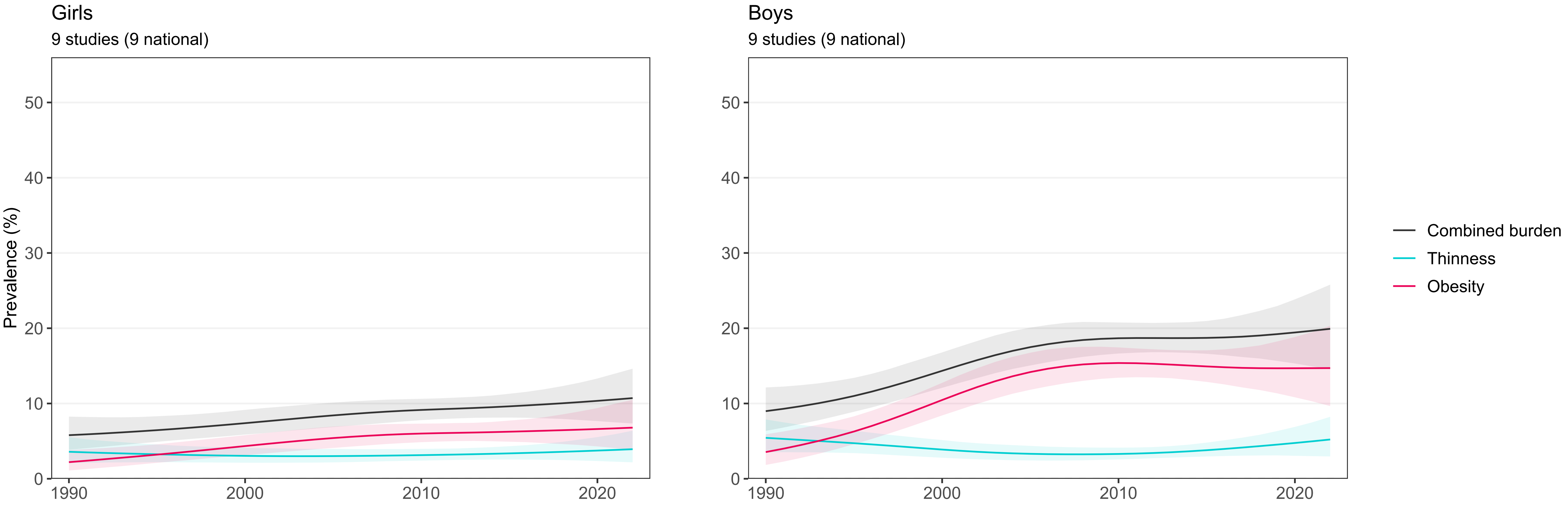


## Adults

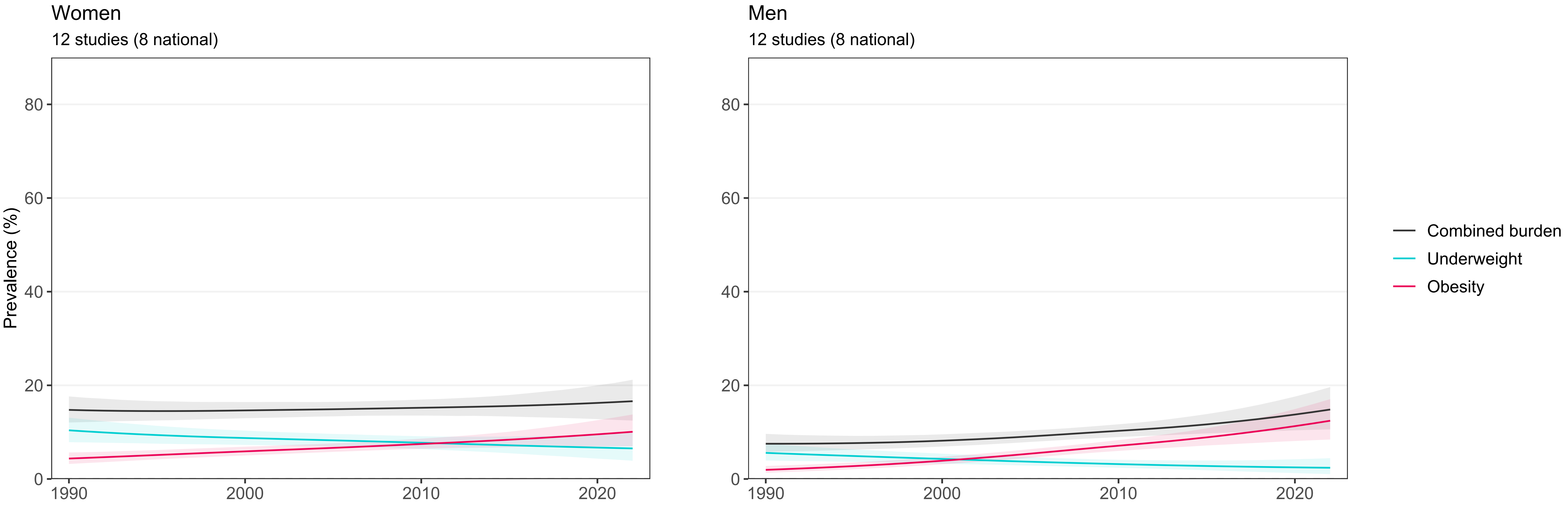

# Tajikistan

## School-aged children and adolescents

### Girls

6 studies (6 national)

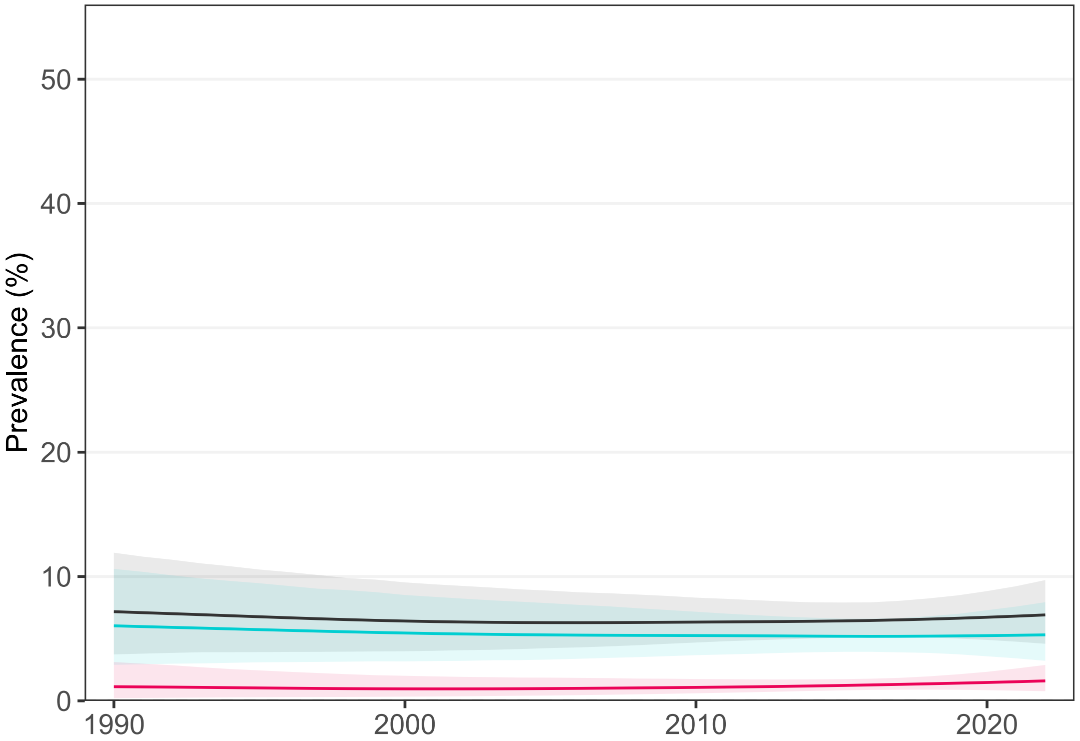


### Boys

3 studies (3 national)

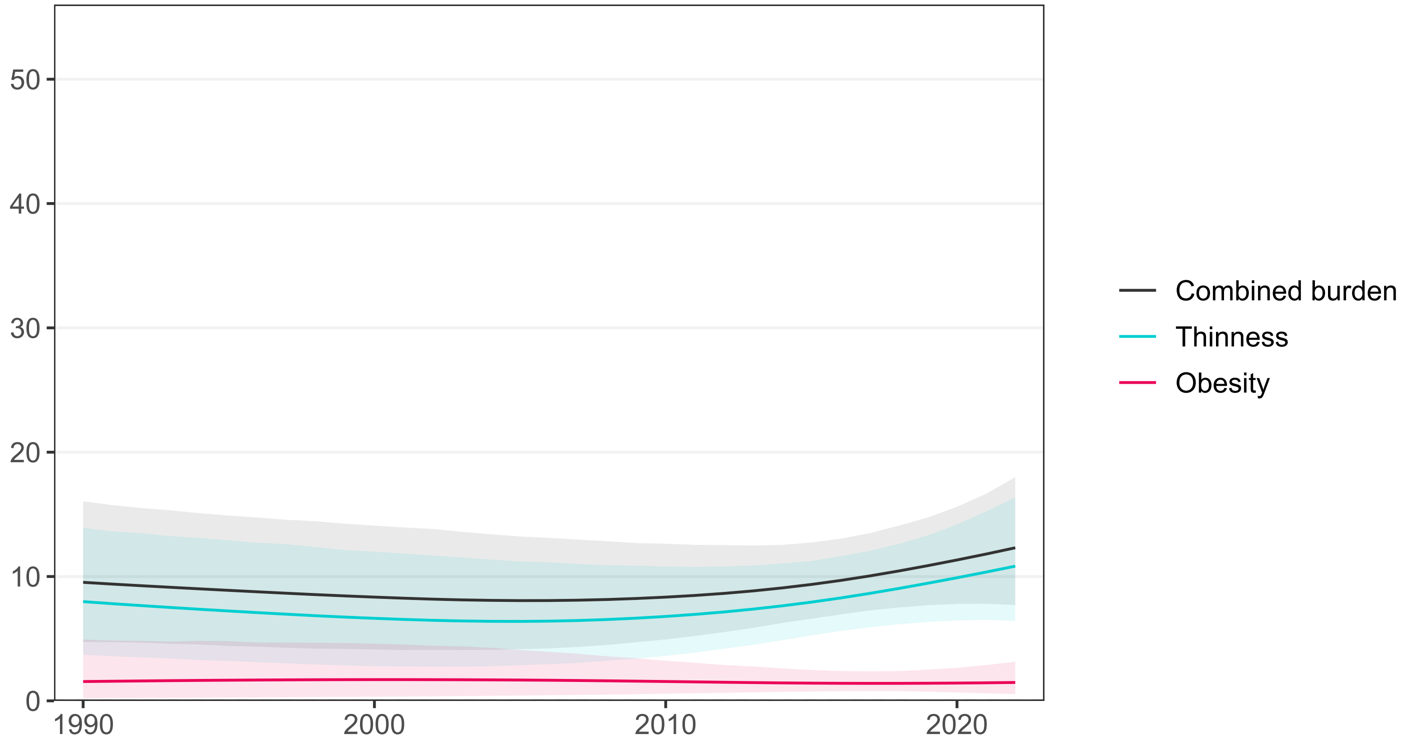


## Adults

### Women

4 studies (4 national)

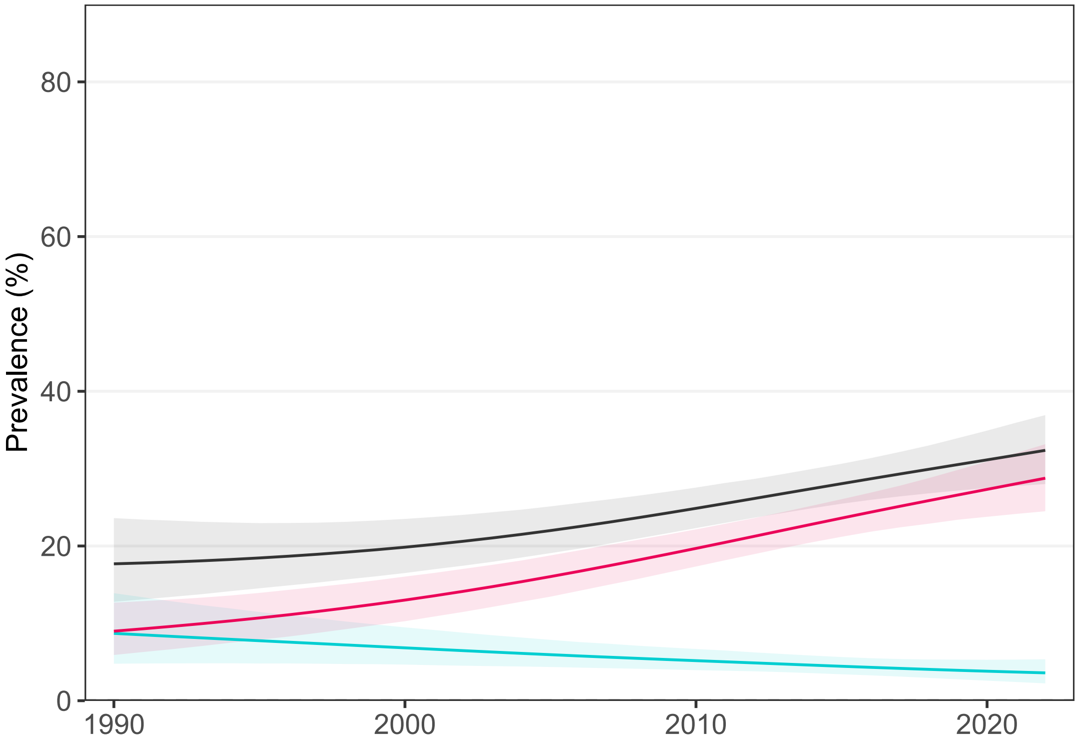


### Men

1 study (1 national)

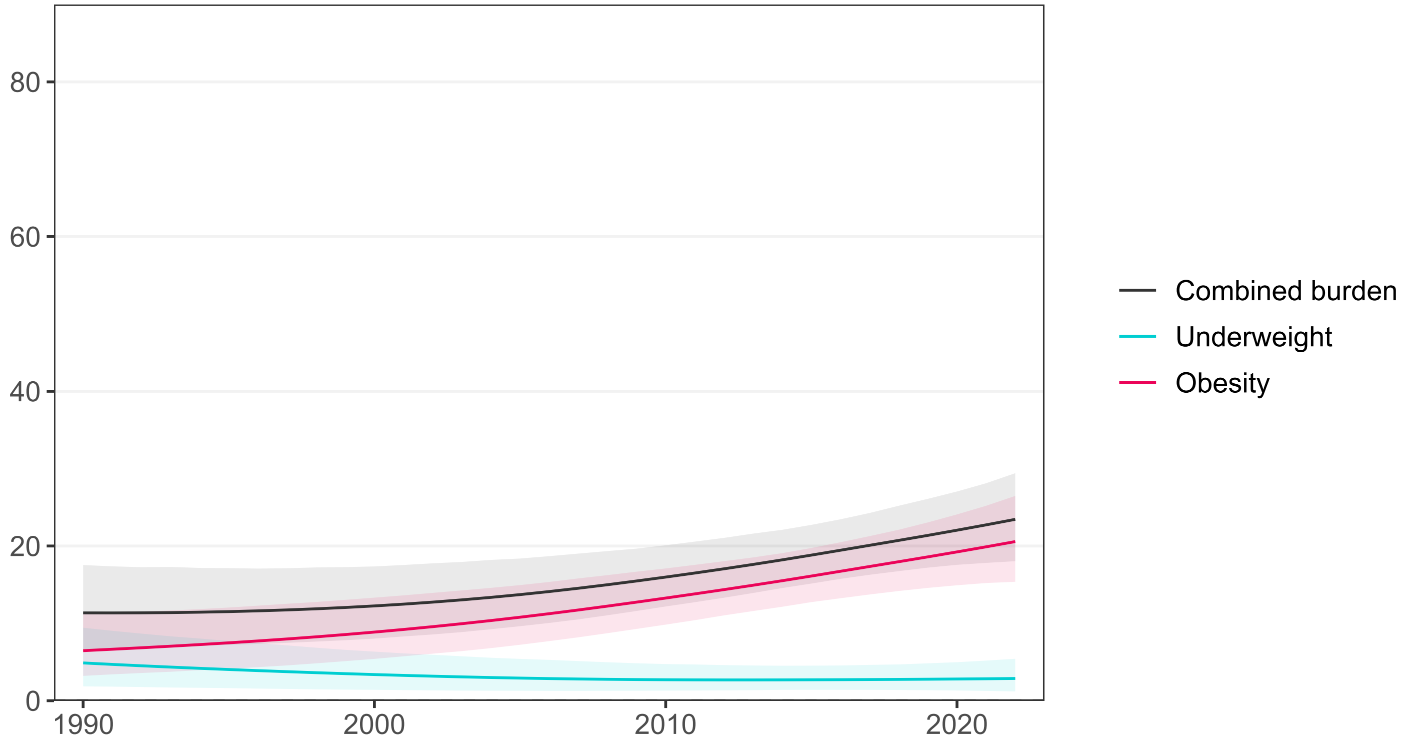

# Tanzania

## School-aged children and adolescents

### Girls

5 studies (3 national)

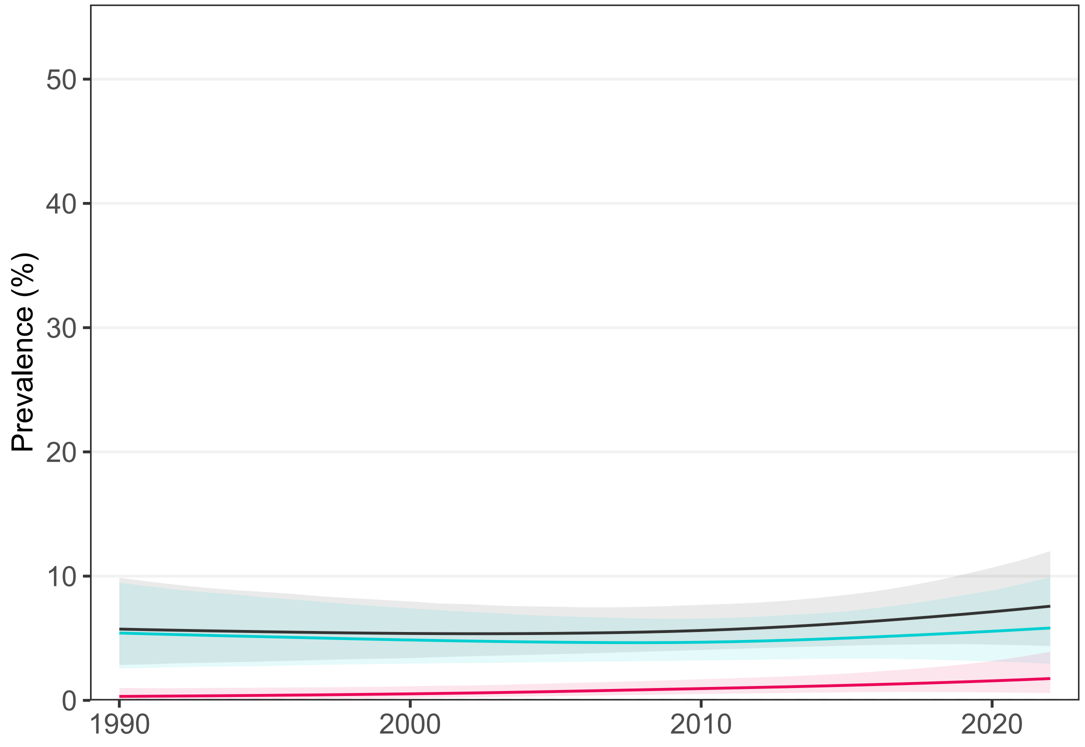


### Boys

2 studies (0 national)

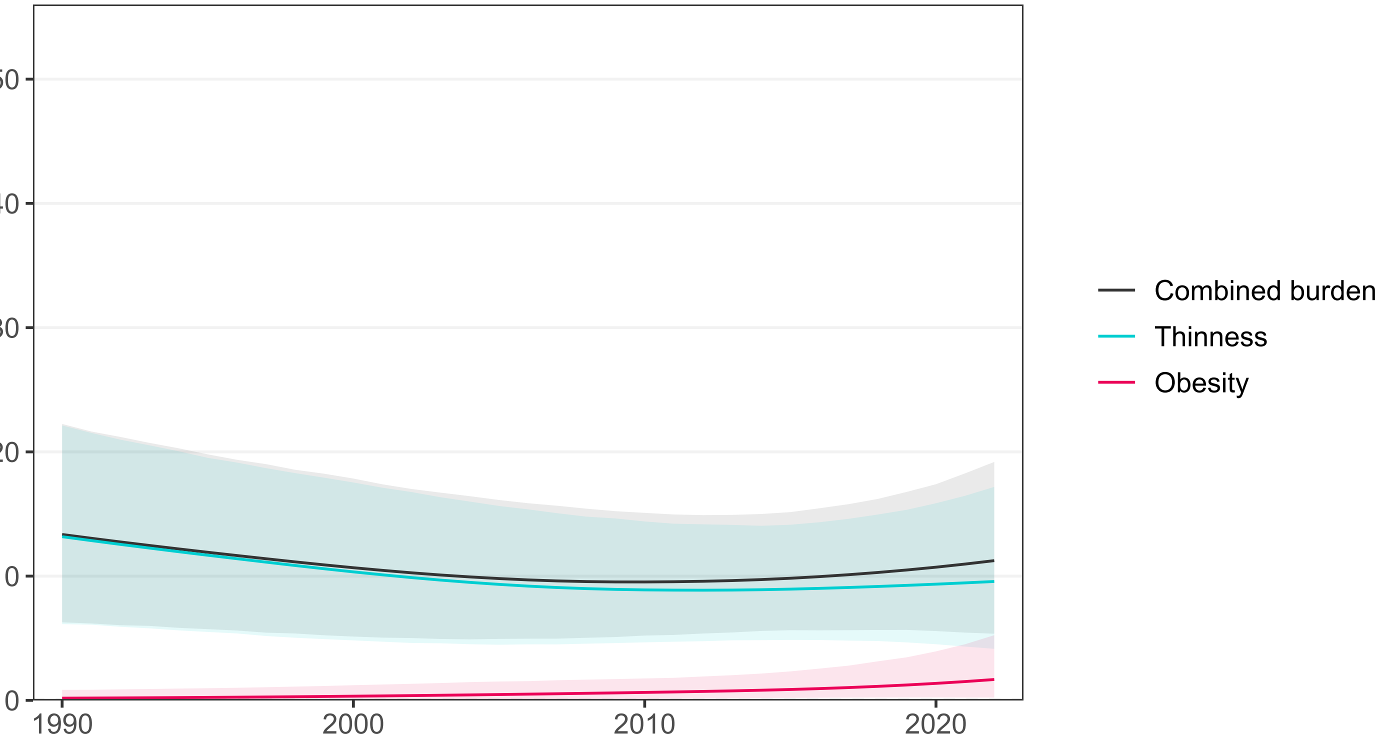


## Adults

### Women

12 studies (6 national)

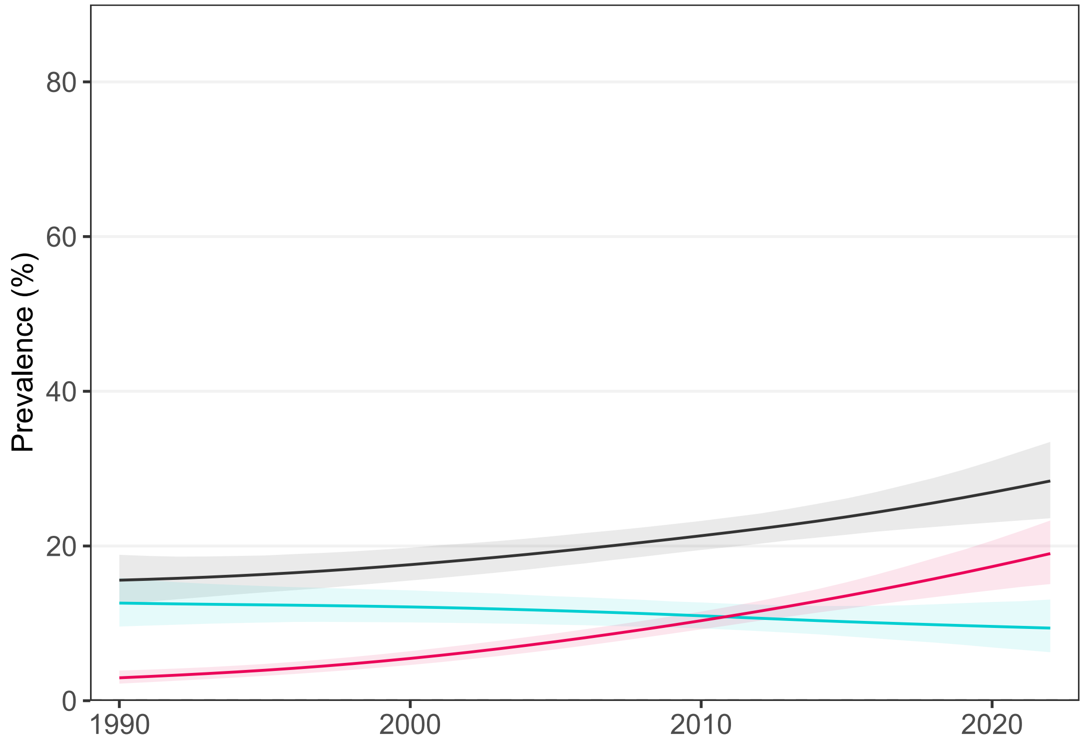


### Men

7 studies (1 national)

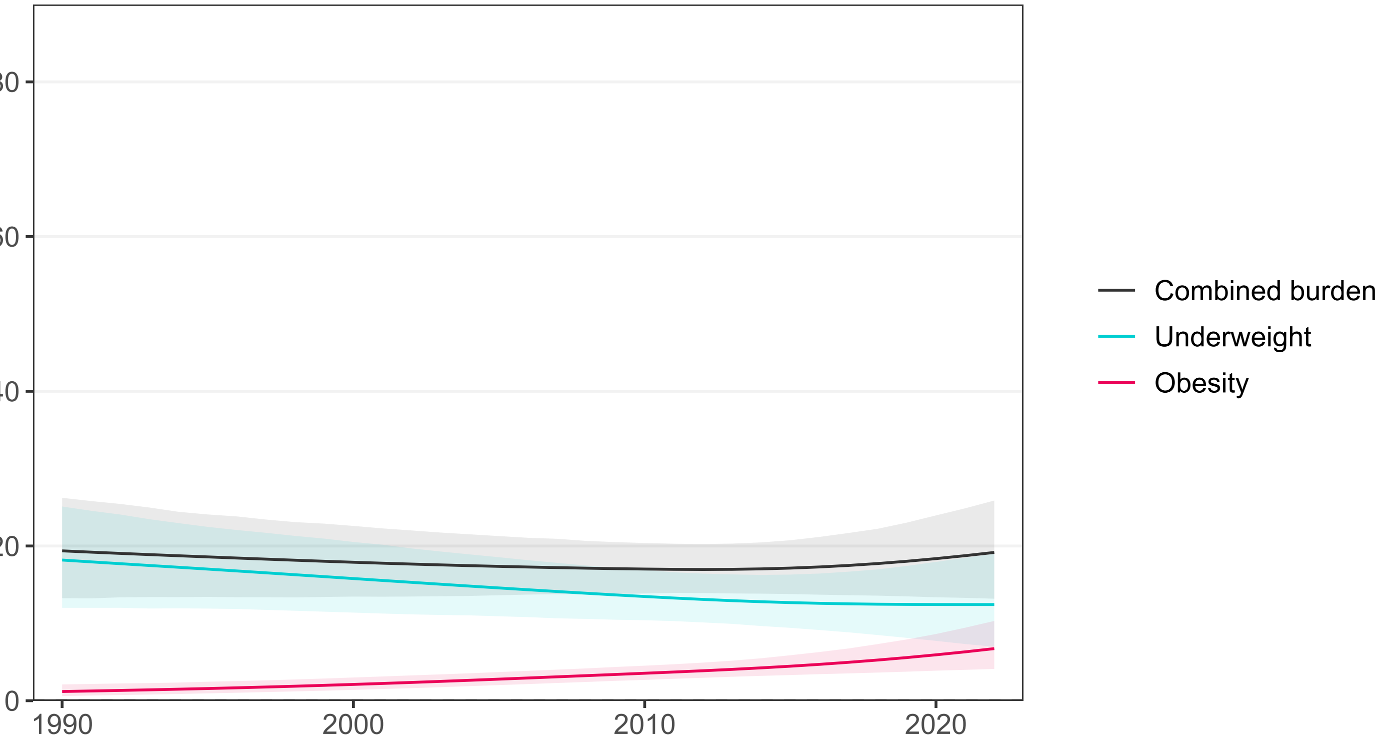

# Thailand

## School-aged children and adolescents

### Girls

14 studies (13 national)

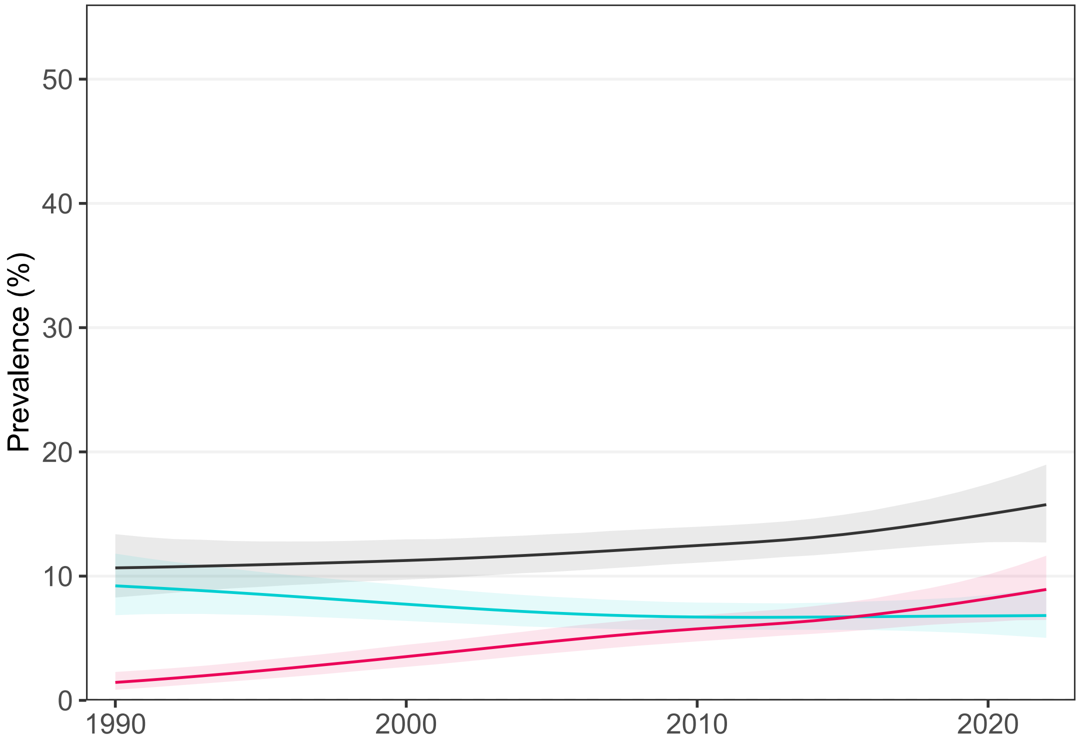


### Boys

14 studies (13 national)

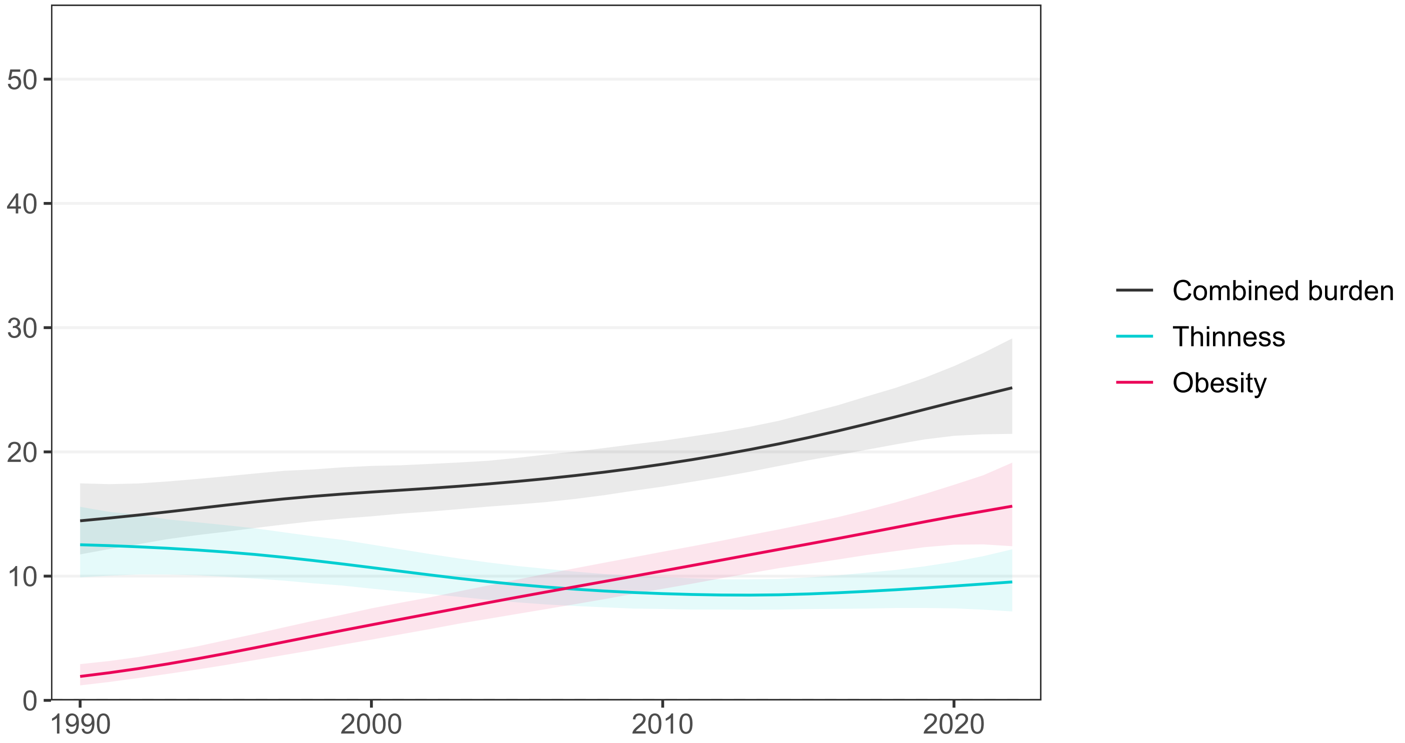


## Adults

### Women

9 studies (9 national)

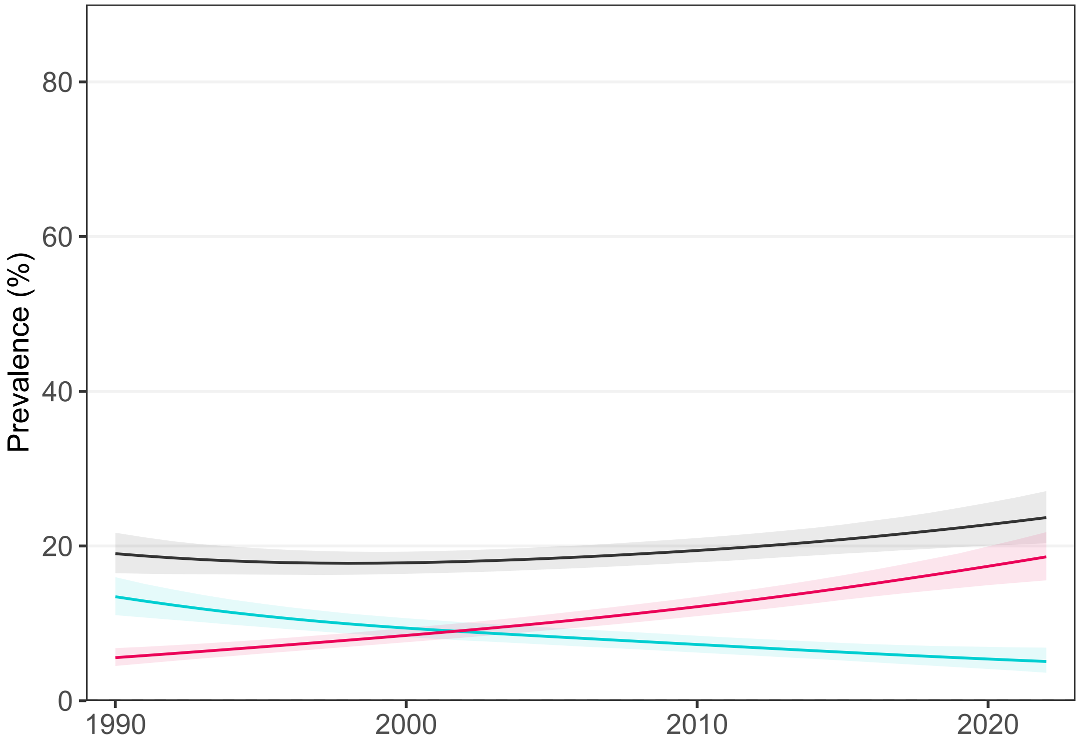


### Men

12 studies (9 national)

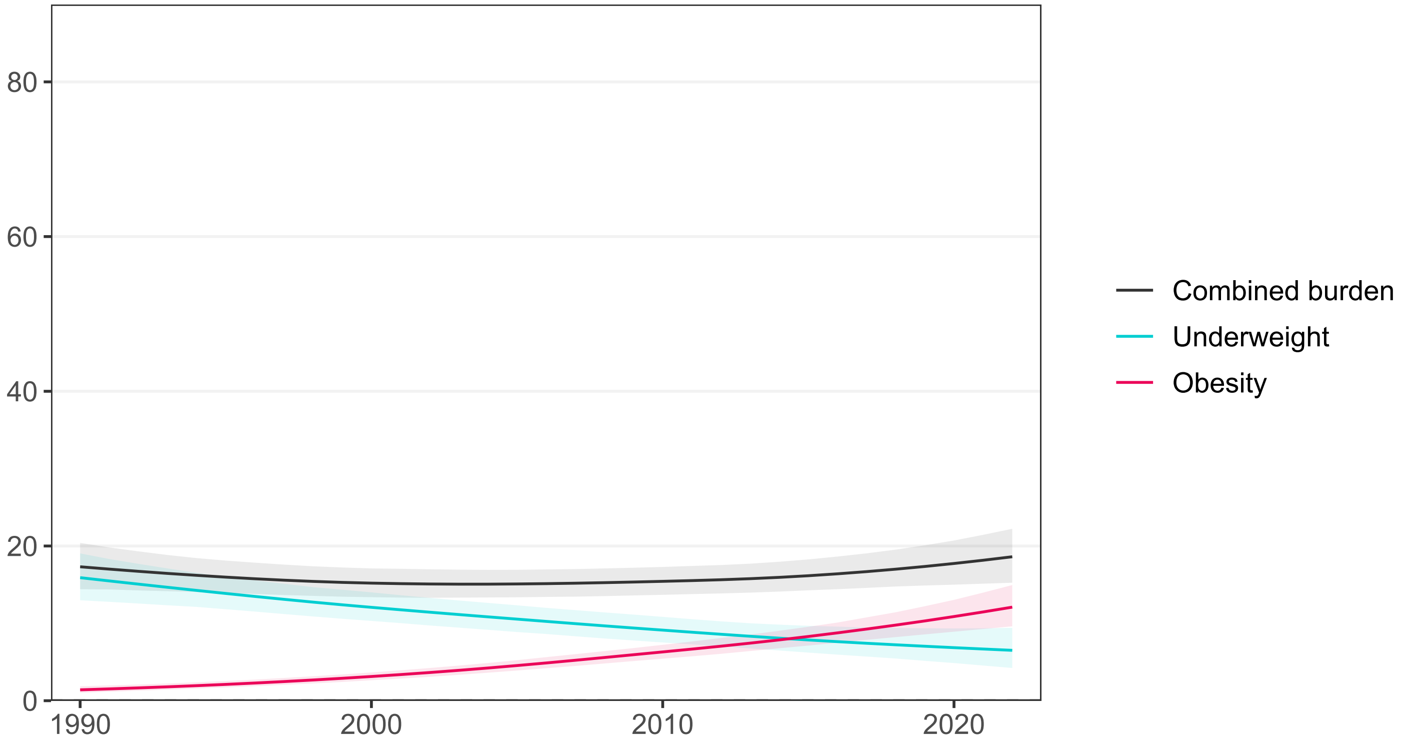

# Timor-Leste

## School-aged children and adolescents

### Girls
6 studies (3 national)

### Boys
5 studies (2 national)

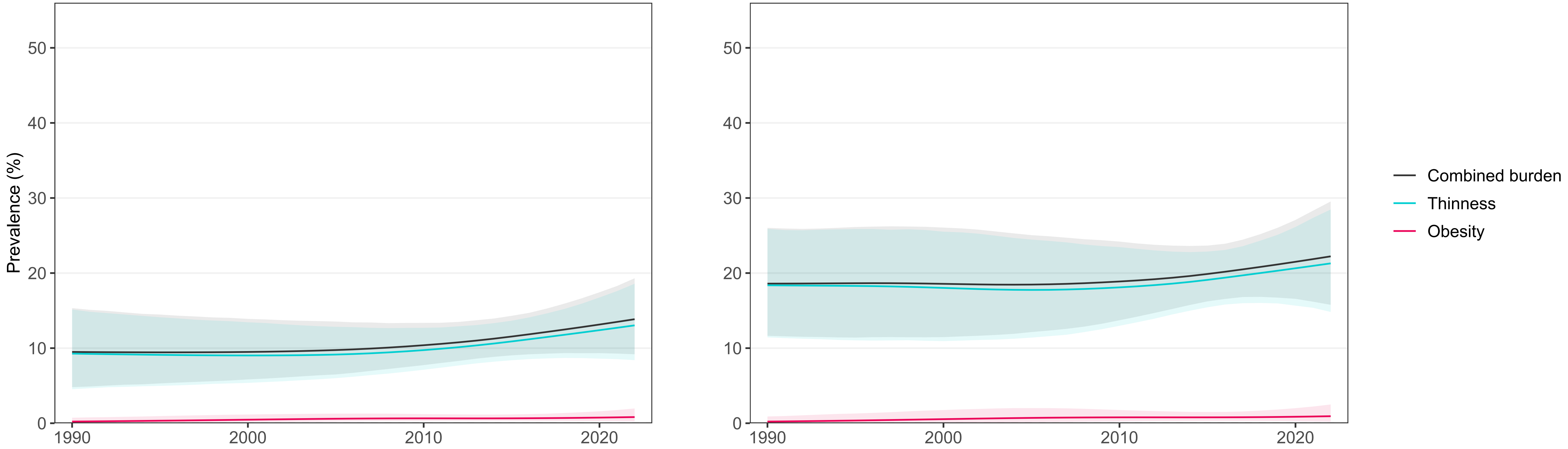


## Adults

### Women
7 studies (3 national)

### Men
5 studies (2 national)

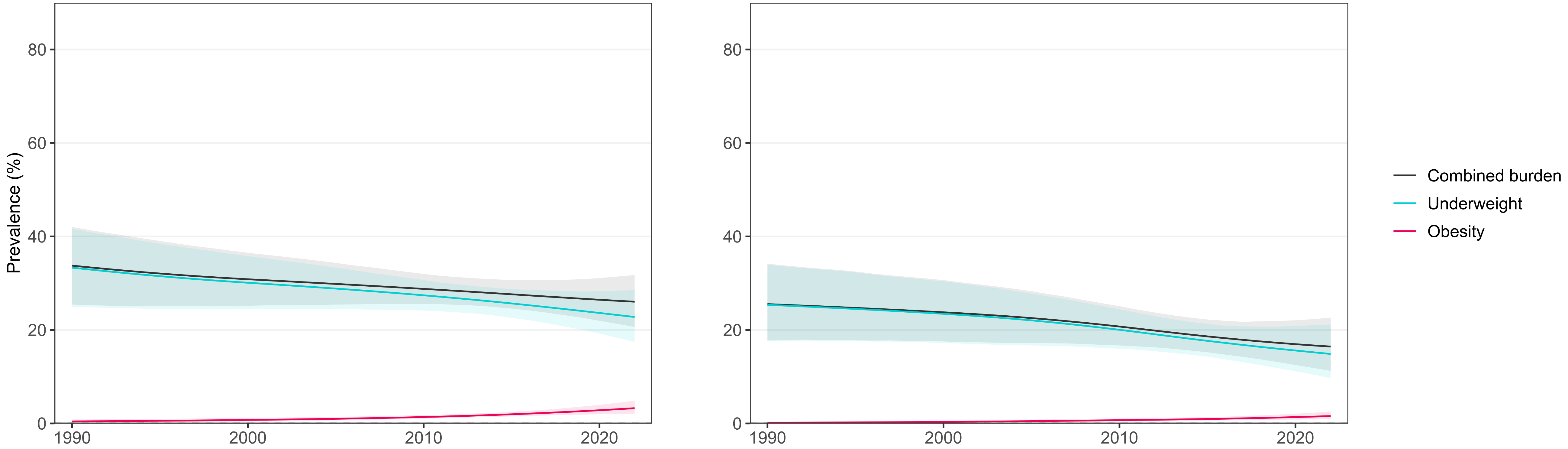

# Togo

## School-aged children and adolescents

### Girls

3 studies (3 national)

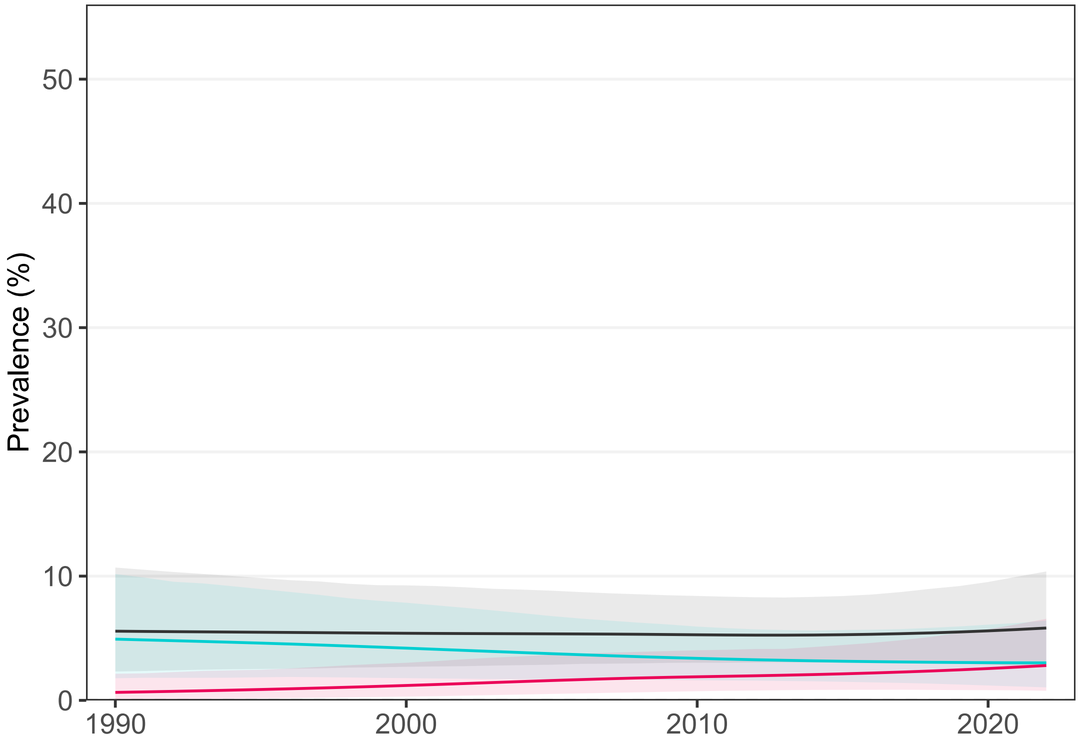


### Boys

2 studies (2 national)

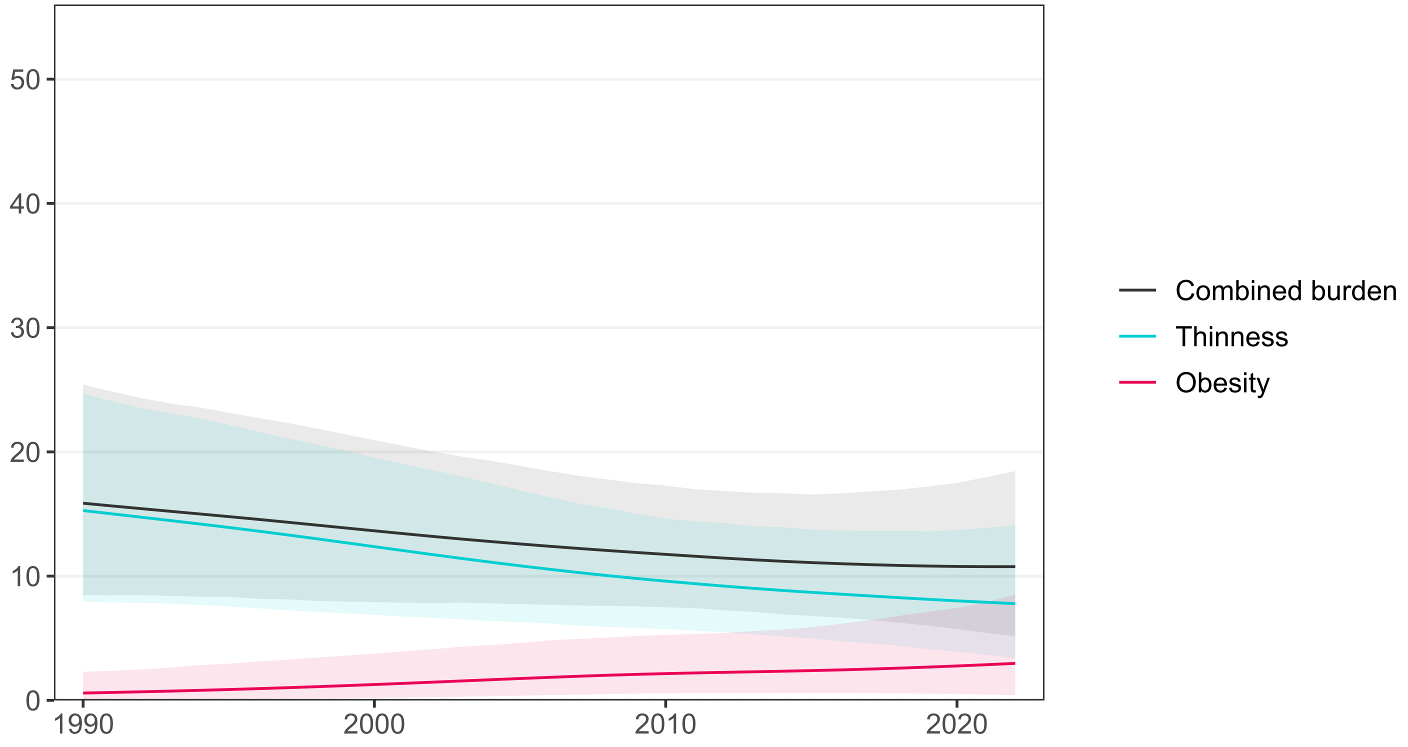


## Adults

### Women

5 studies (4 national)

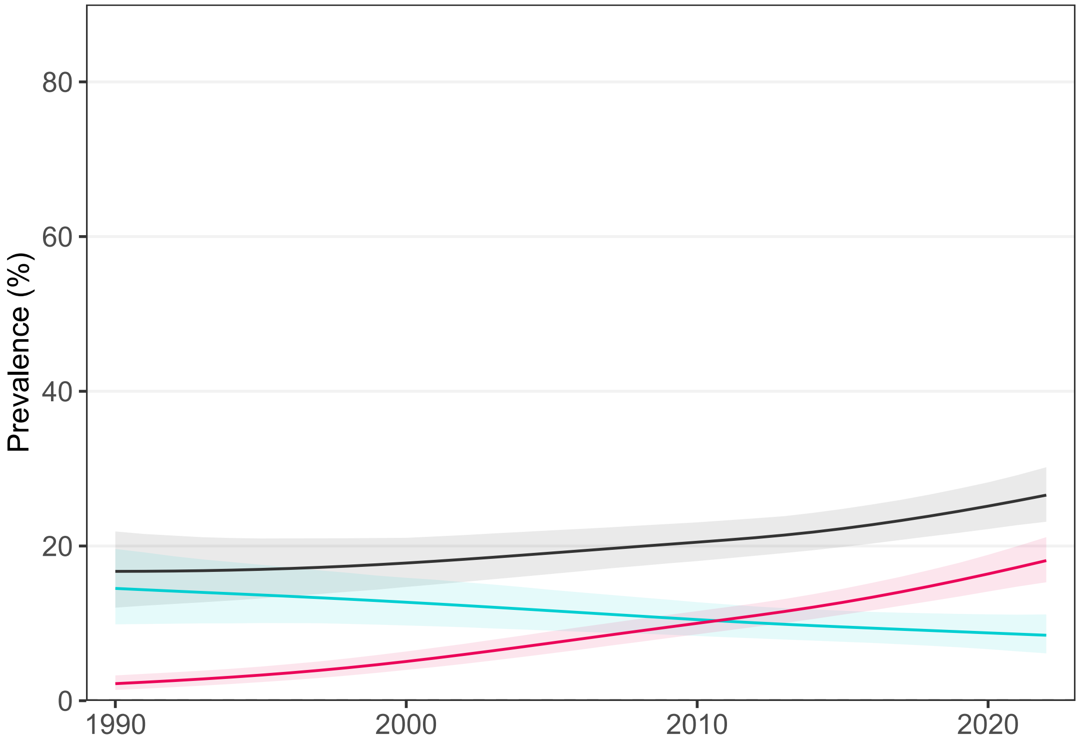


### Men

2 studies (2 national)

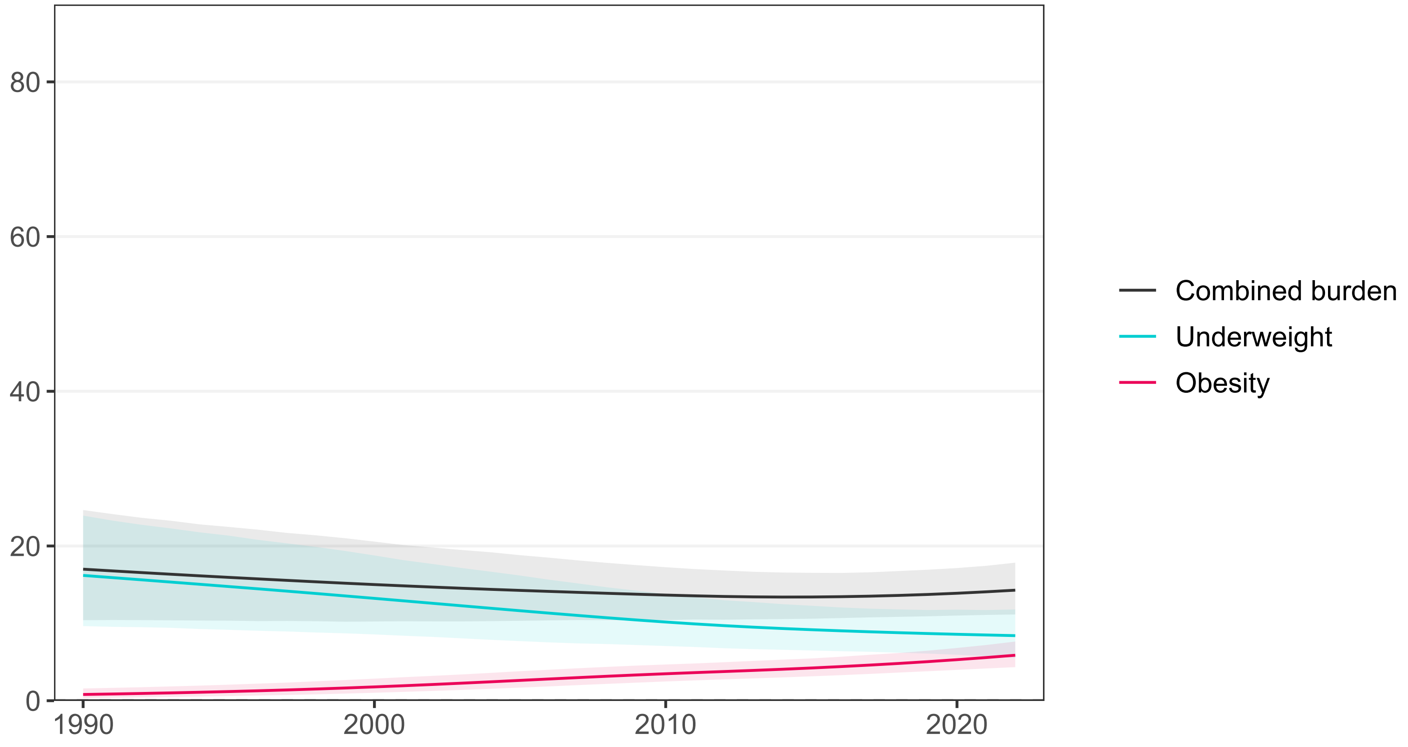

# Tokelau

## School-aged children and adolescents

### Girls

2 studies (2 national)

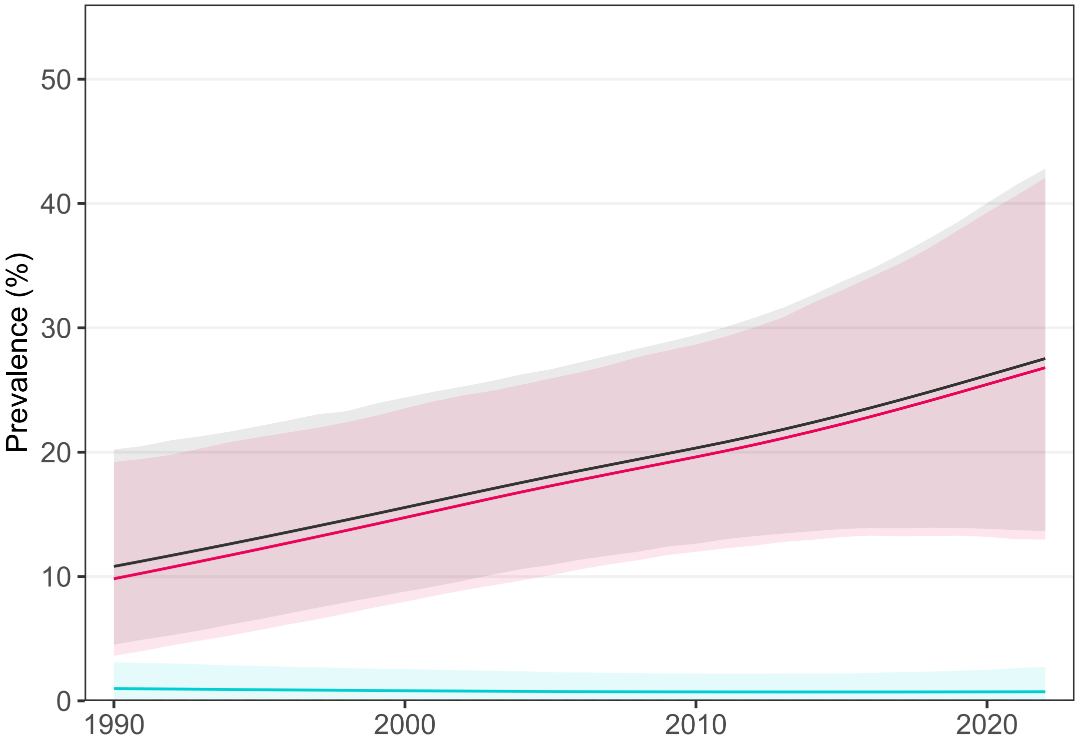


### Boys

2 studies (2 national)

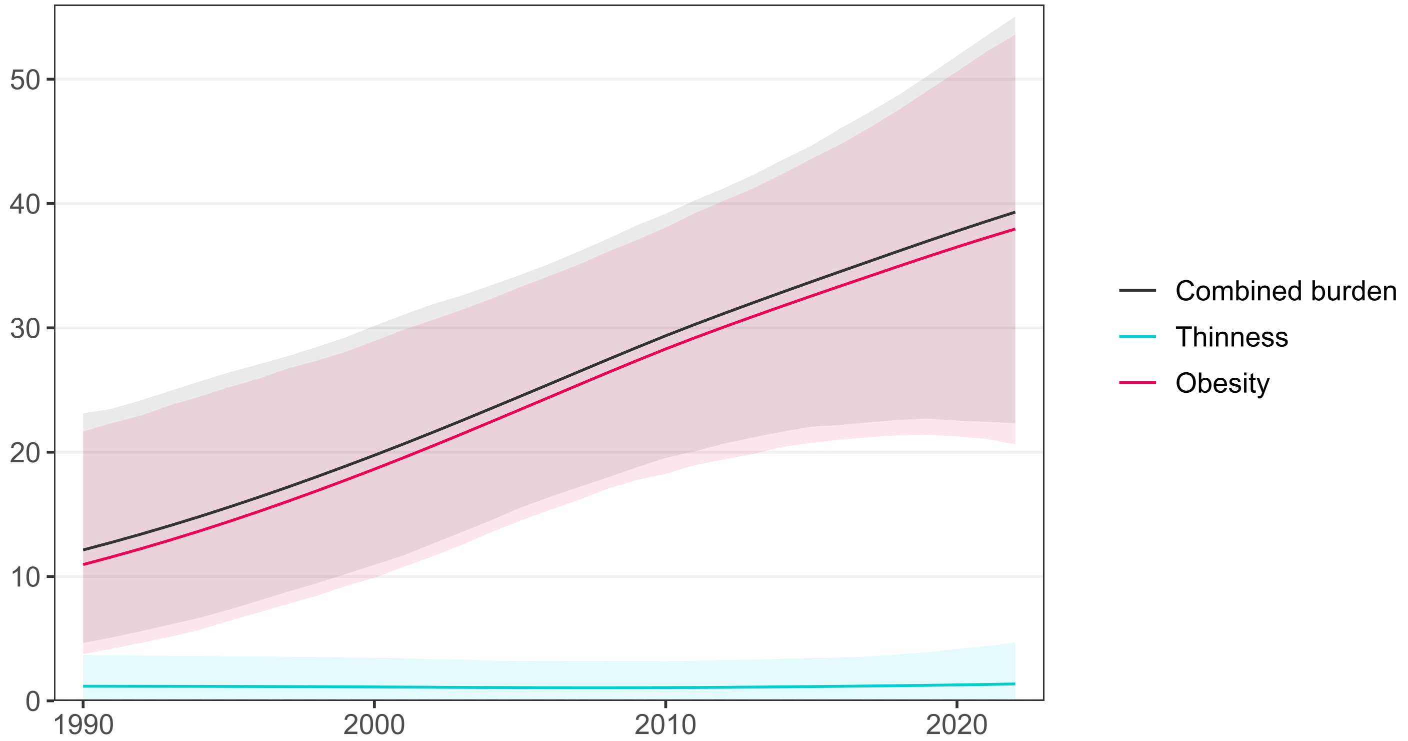


## Adults

### Women

2 studies (2 national)

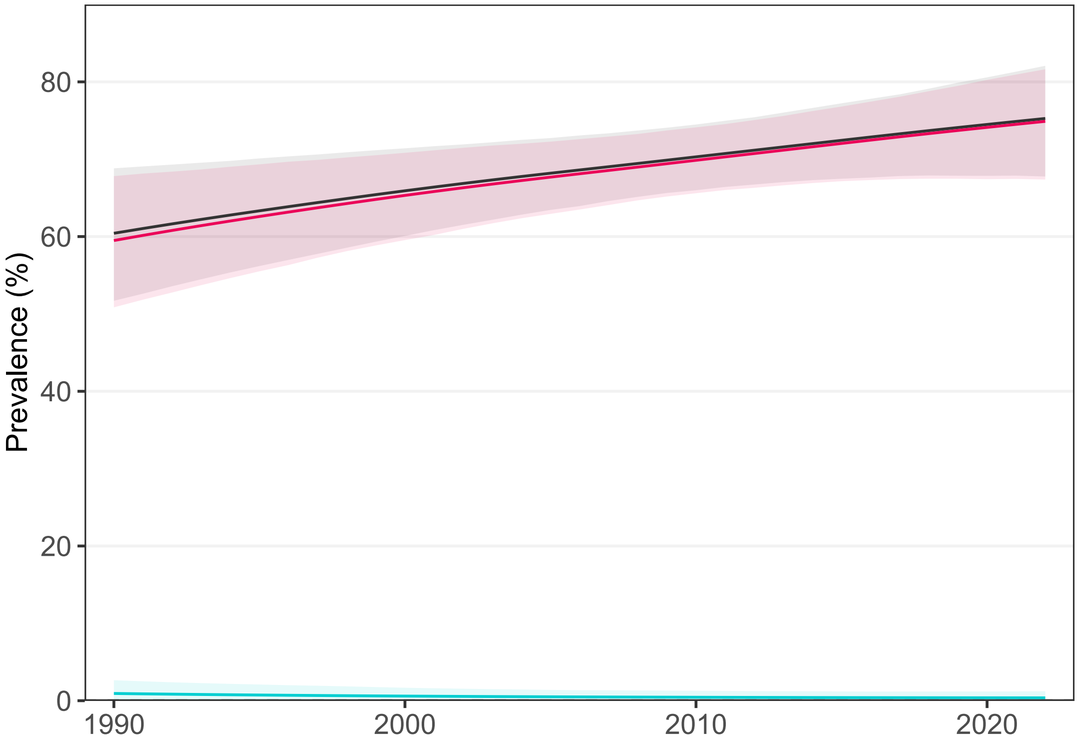


### Men

2 studies (2 national)

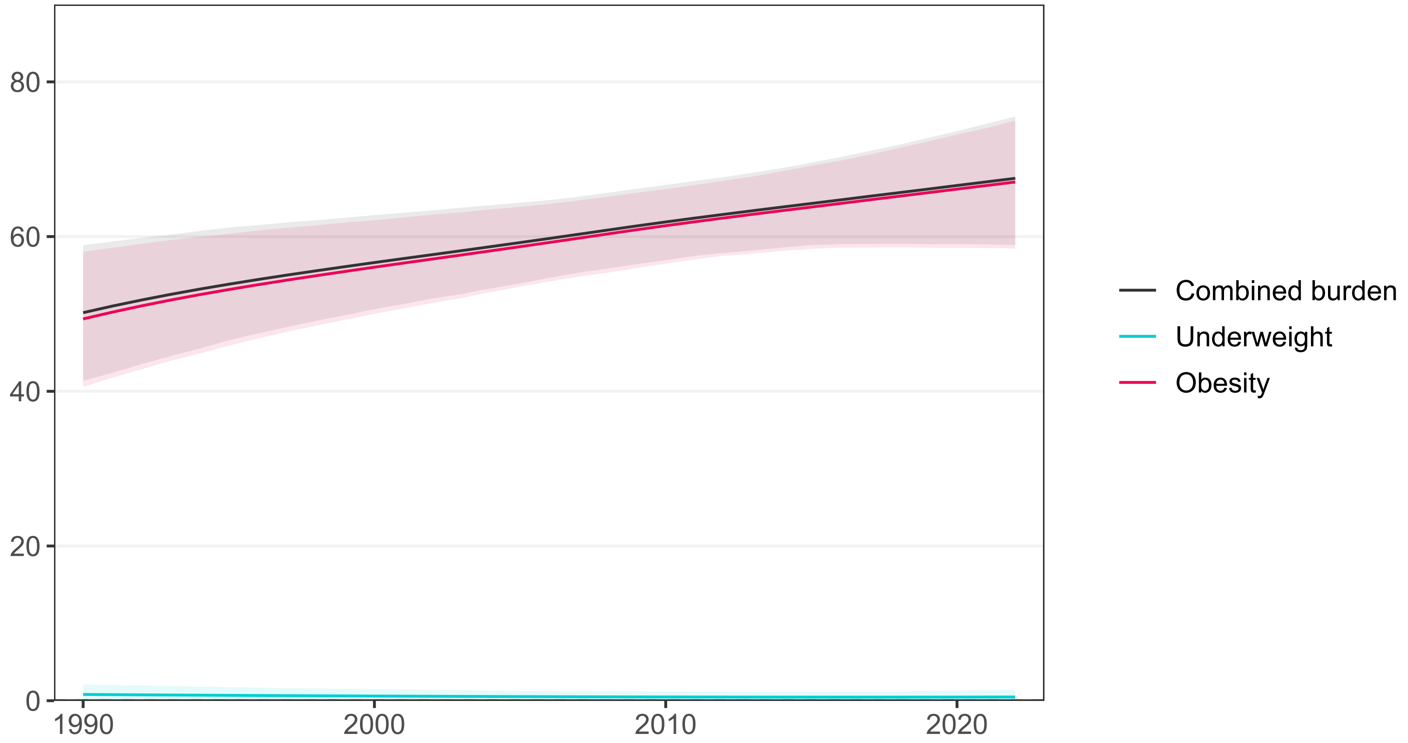

# Tonga

## School-aged children and adolescents

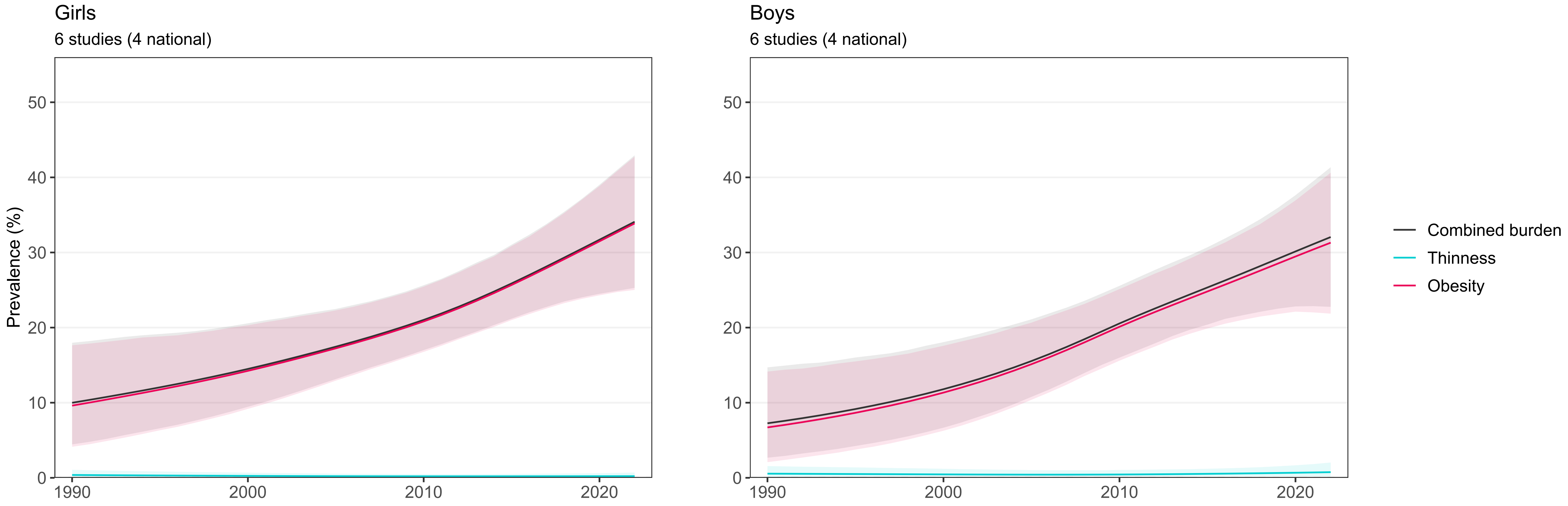


## Adults

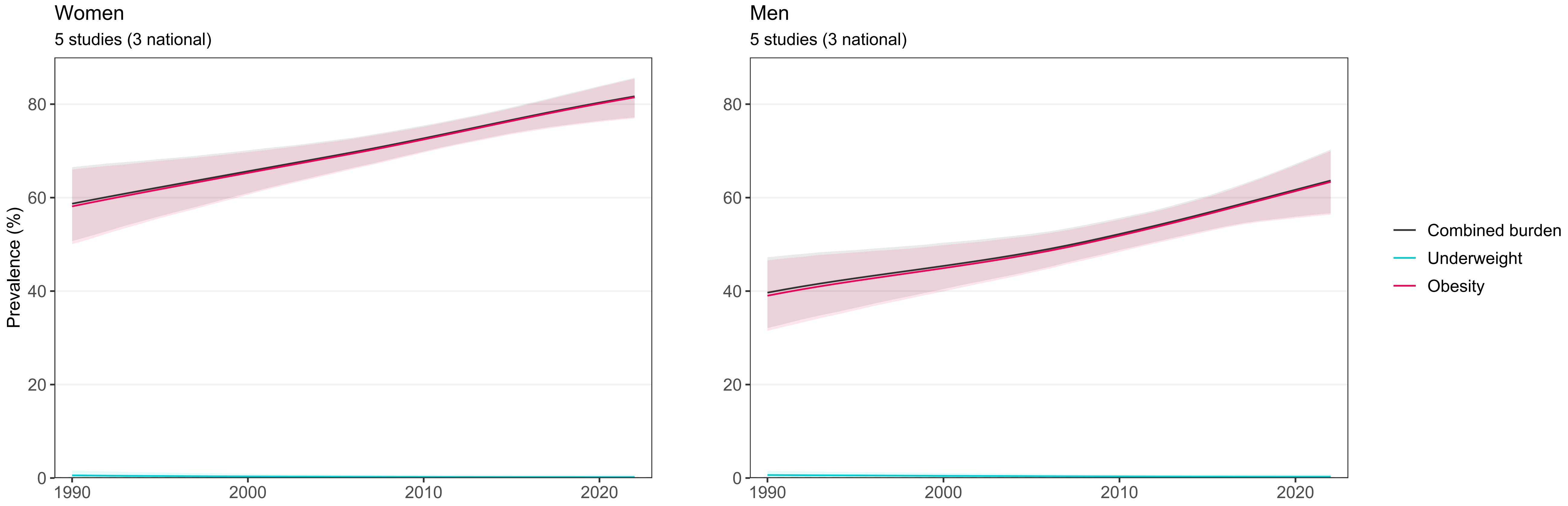

# Trinidad & Tobago

## School-aged children and adolescents

### Girls

4 studies (4 national)

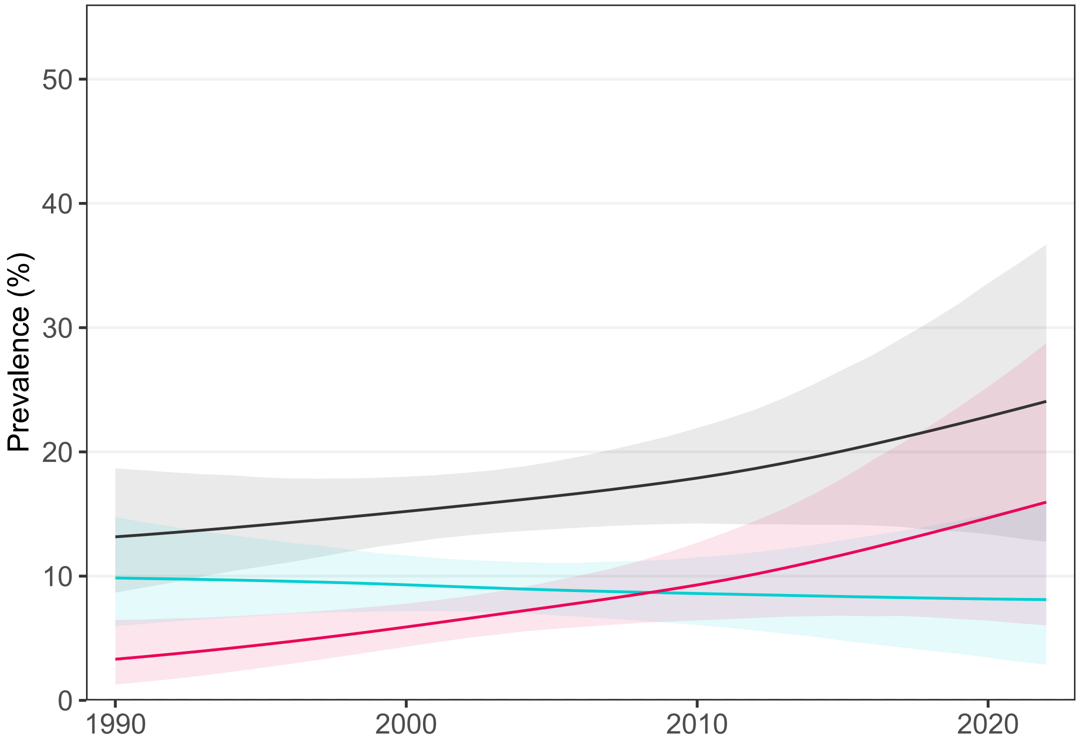


### Boys

4 studies (4 national)

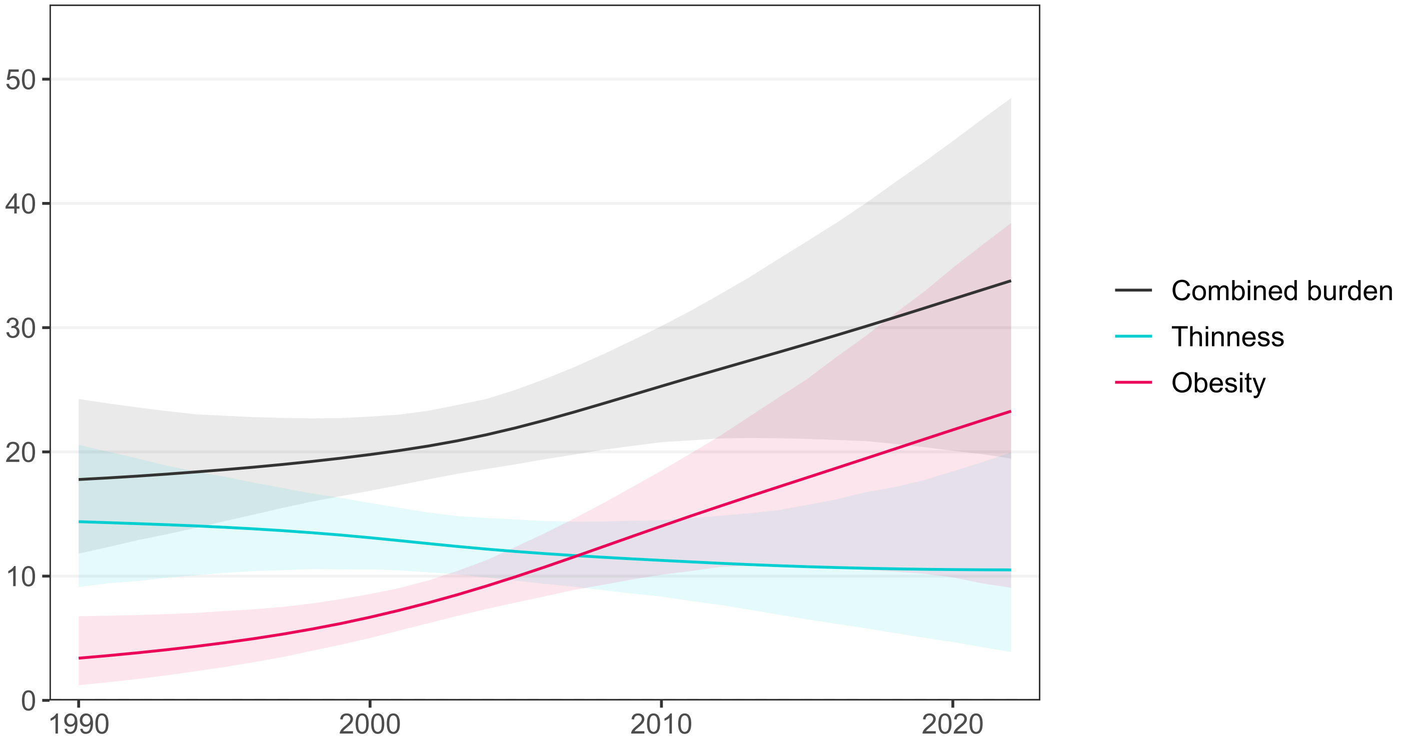


## Adults

### Women

4 studies (3 national)

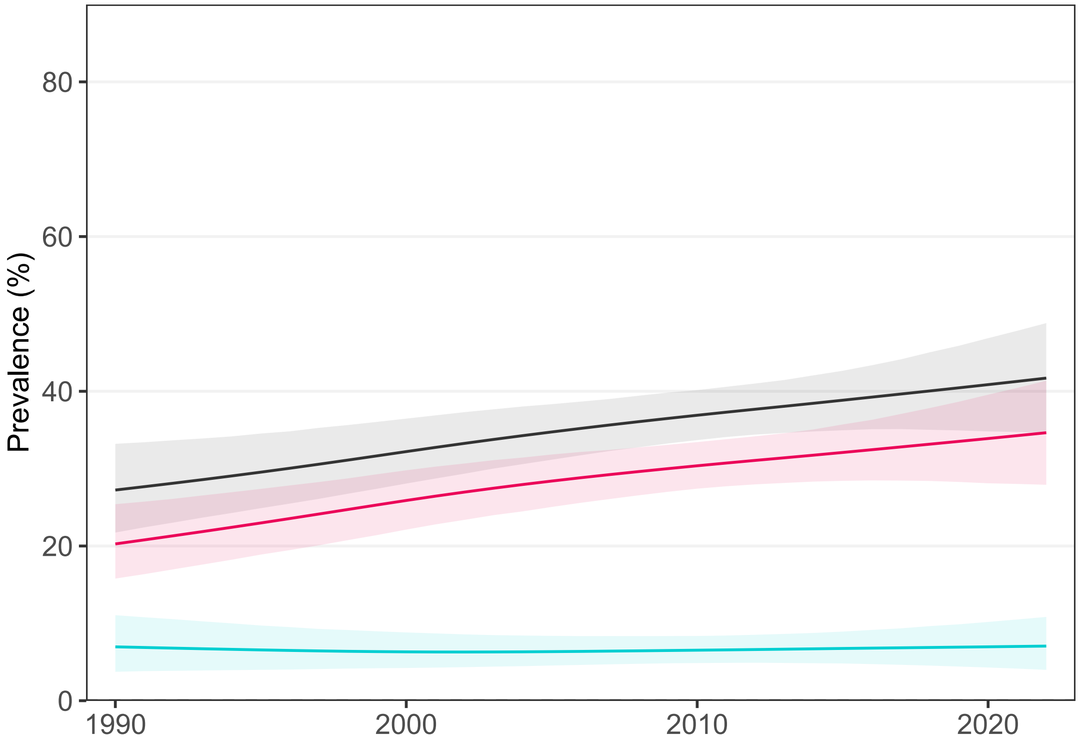


### Men

4 studies (3 national)

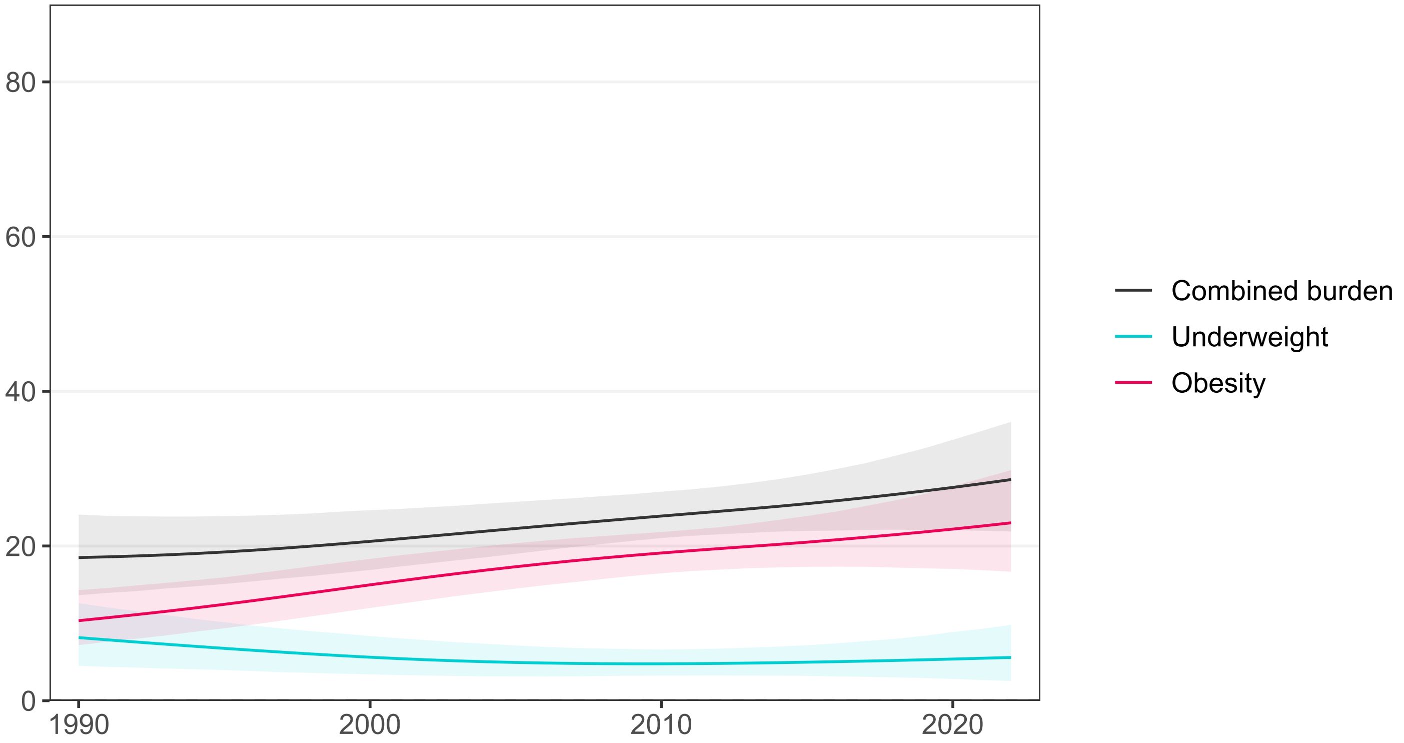

# Tunisia

## School-aged children and adolescents

### Girls

3 studies (2 national)

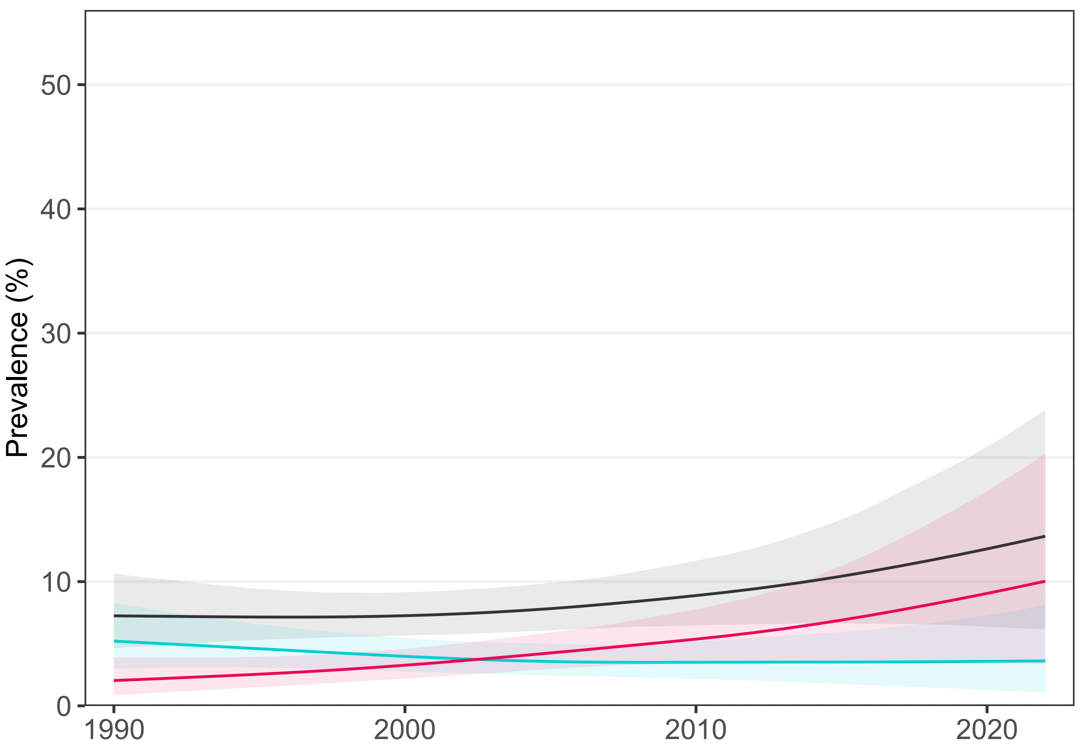


### Boys

3 studies (2 national)

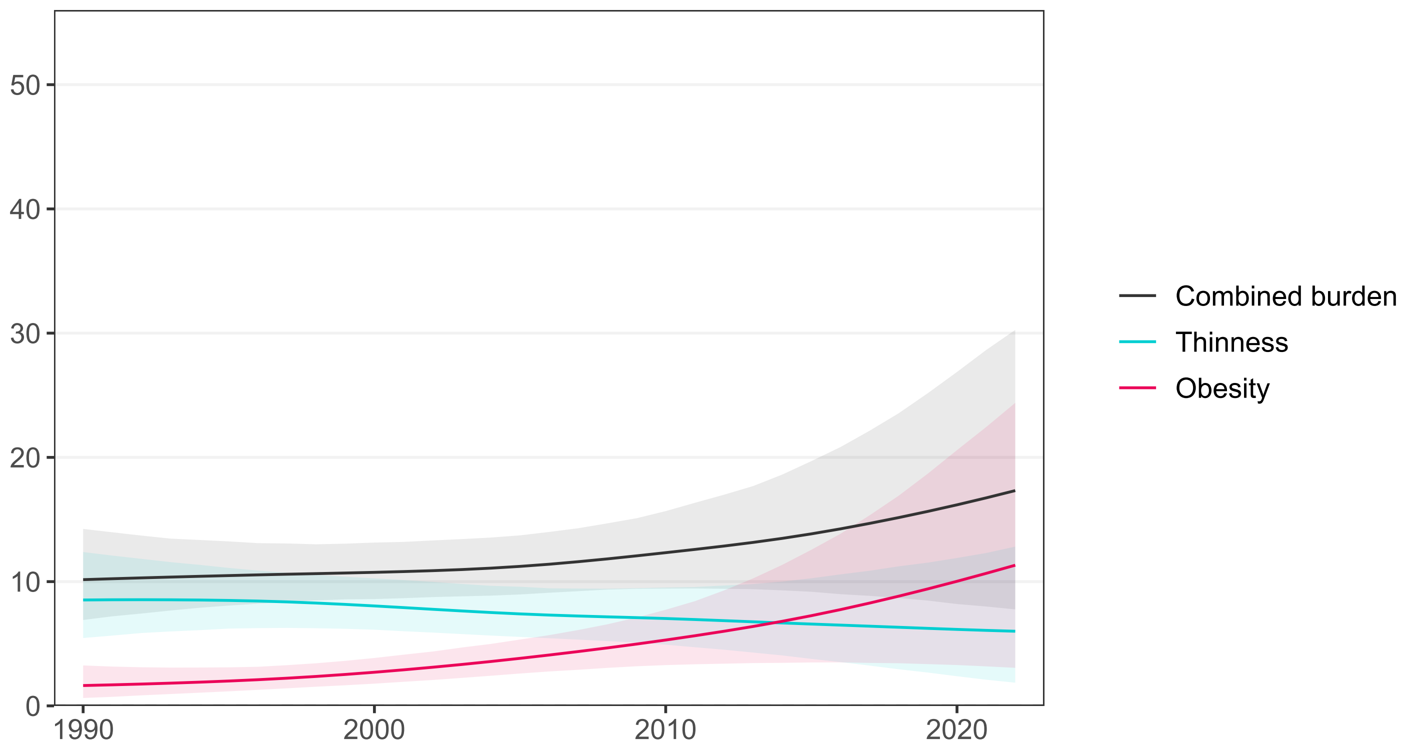


## Adults

### Women

5 studies (3 national)

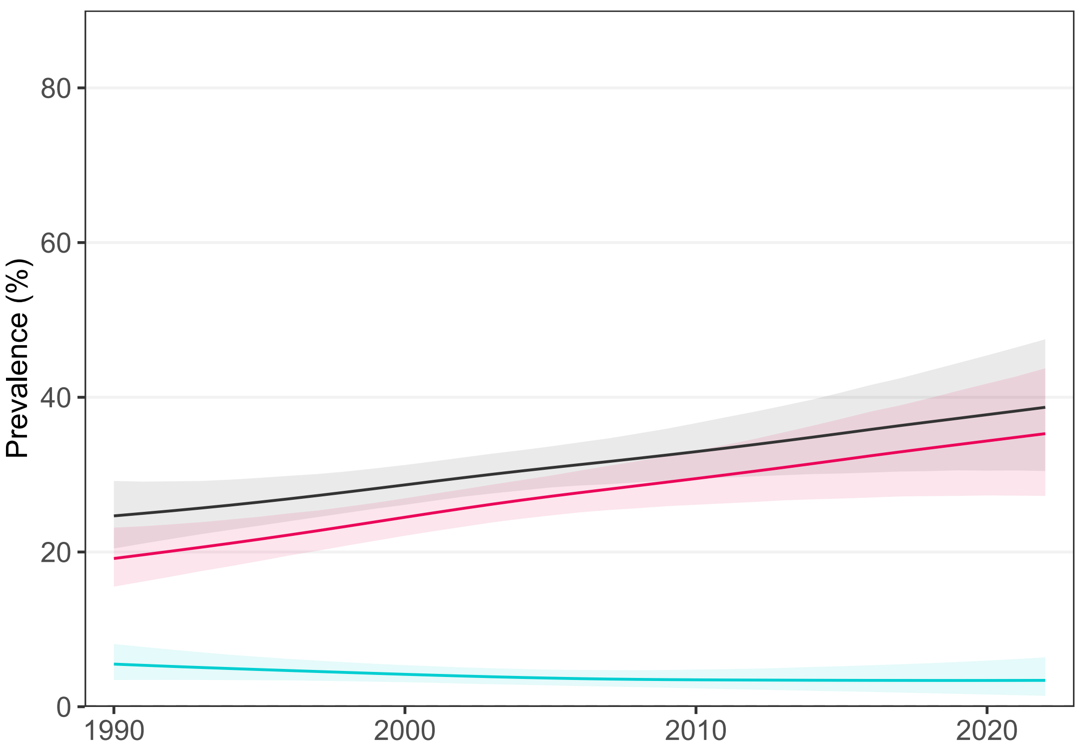


### Men

5 studies (3 national)

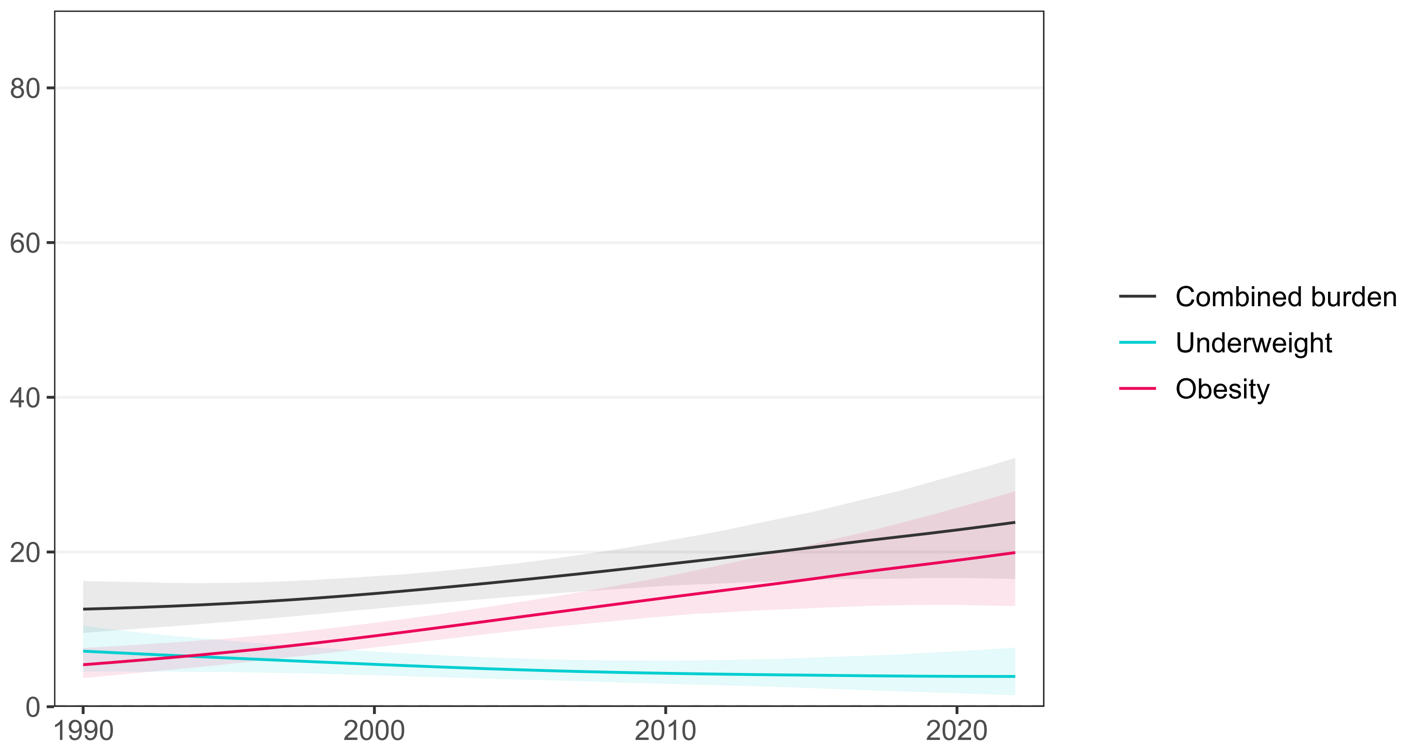

# Turkiye

## School-aged children and adolescents

### Girls
9 studies (7 national)

### Boys
7 studies (5 national)

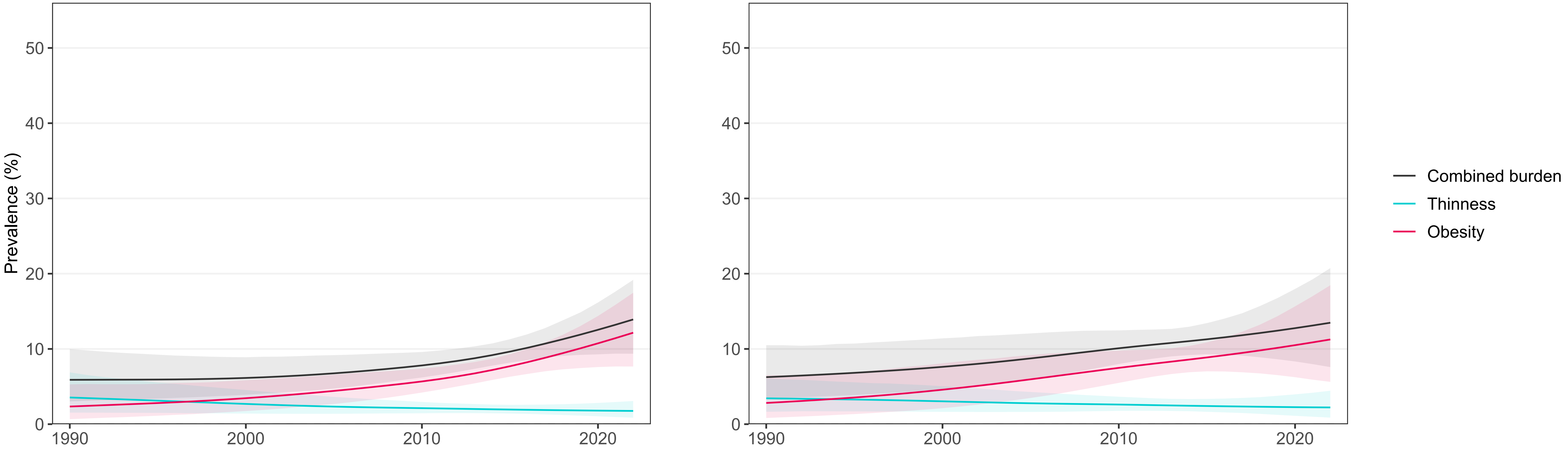


## Adults

### Women
30 studies (23 national)

### Men
23 studies (17 national)

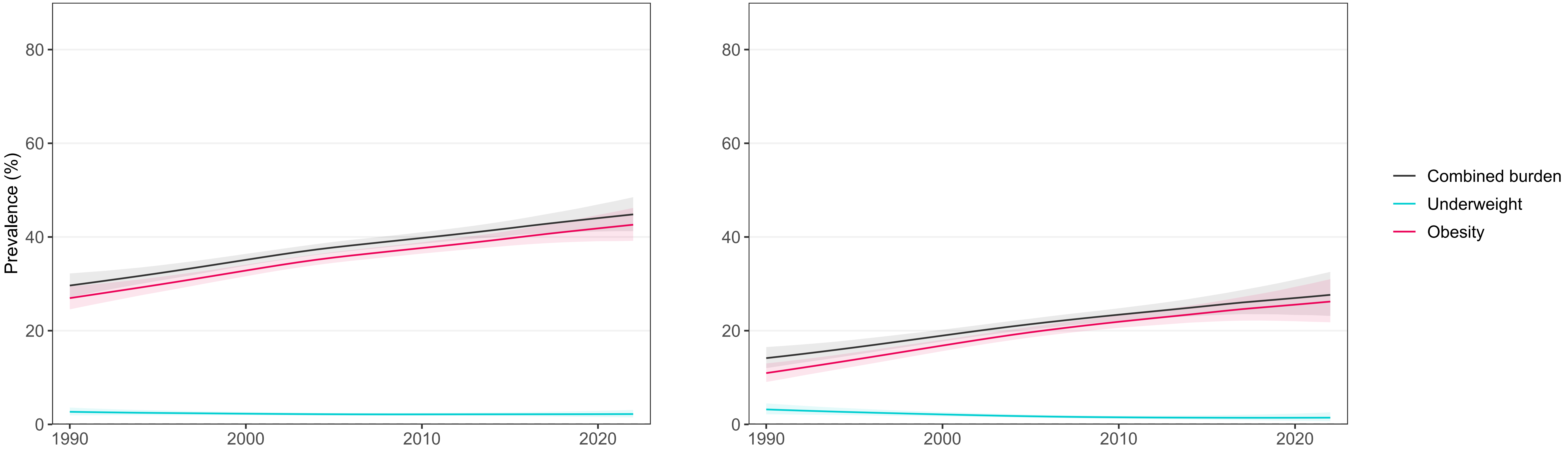

# Turkmenistan

## School-aged children and adolescents

Girls

3 studies (3 national)

Boys

3 studies (3 national)

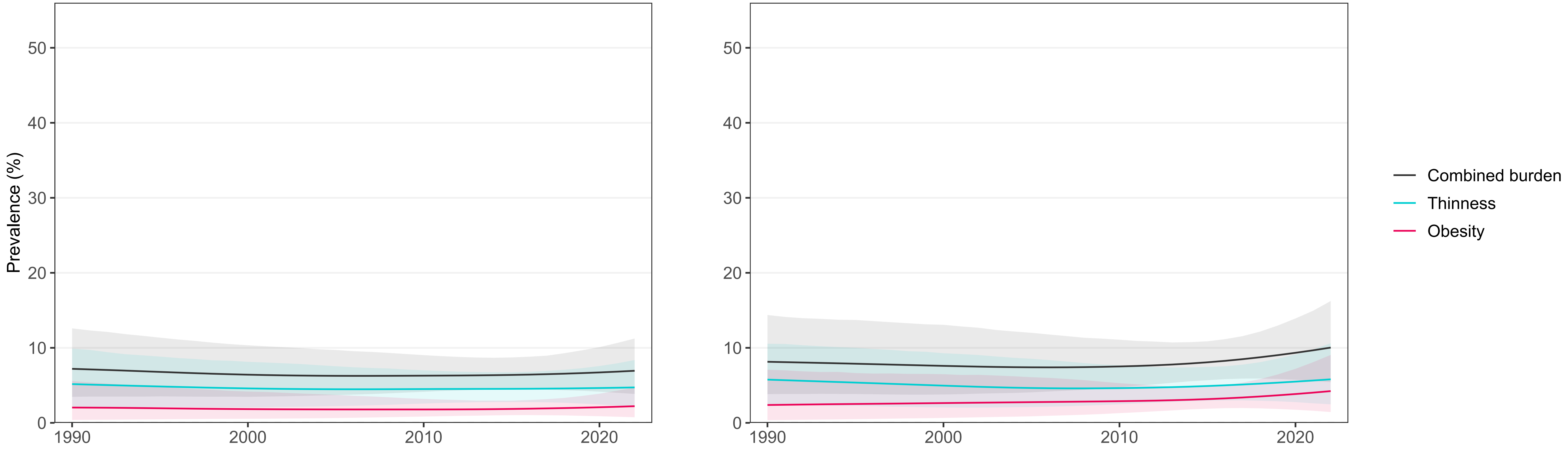


## Adults

Women

3 studies (3 national)

Men

2 studies (2 national)

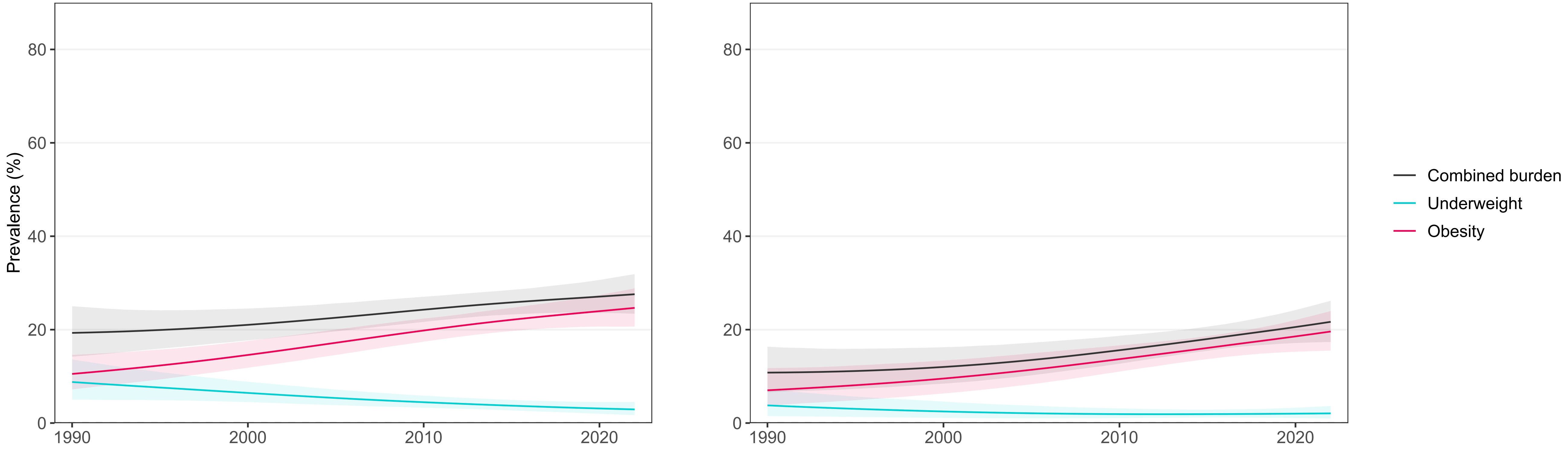

# Tuvalu

## School-aged children and adolescents

### Girls

2 studies (2 national)

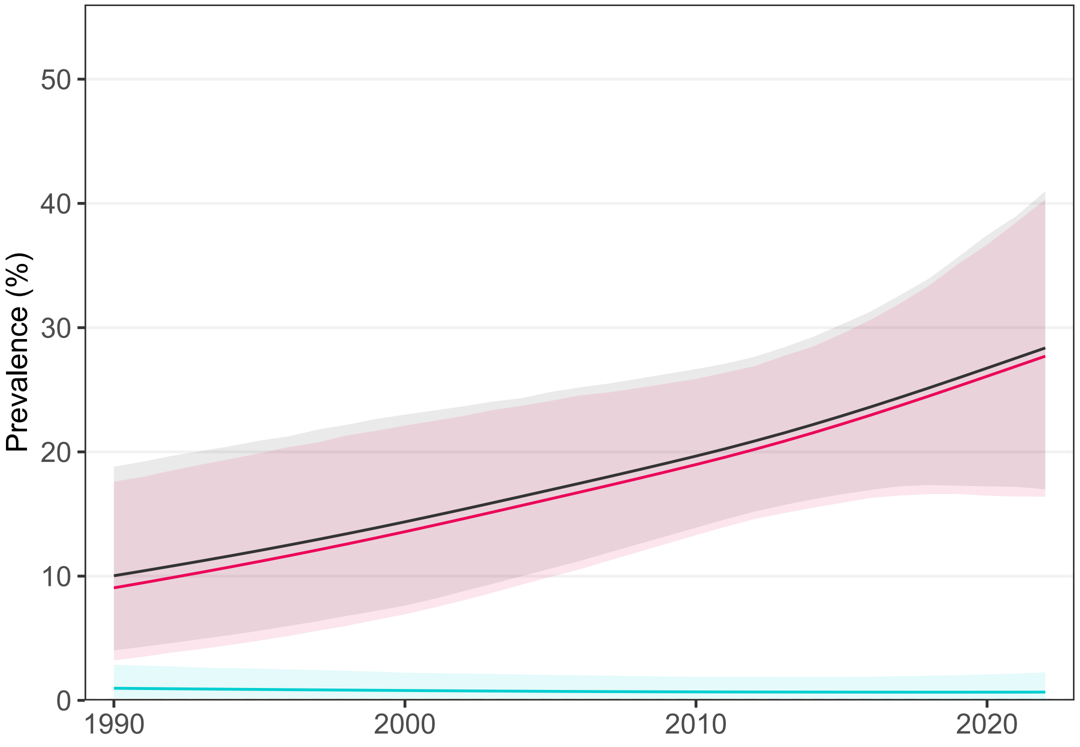


### Boys

2 studies (2 national)

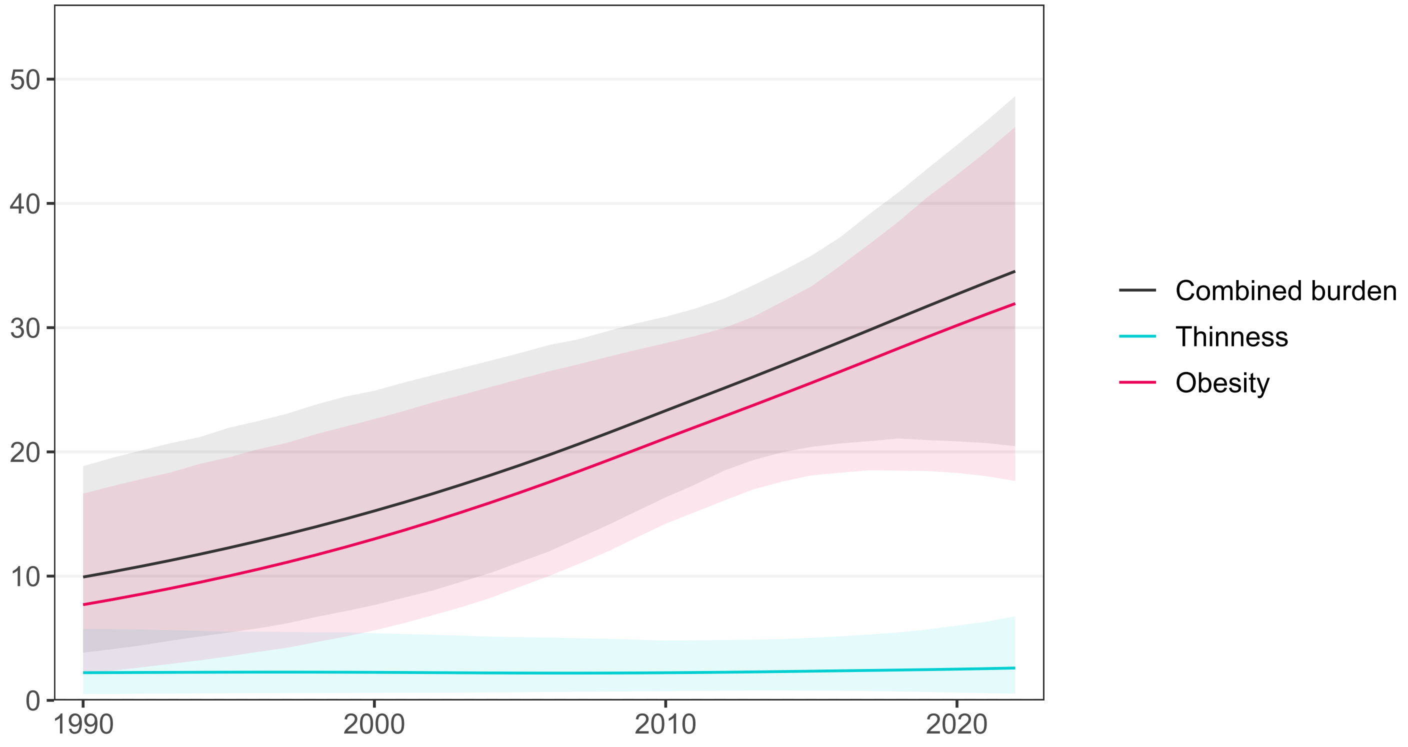


## Adults

### Women

1 study (1 national)

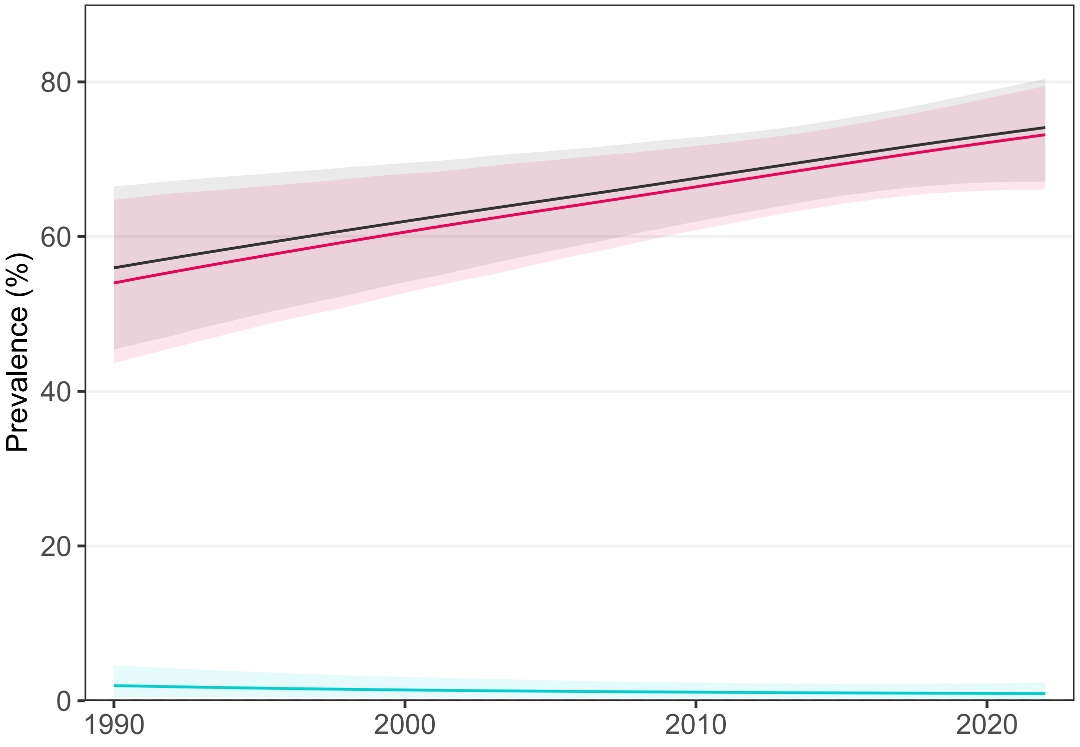


### Men

1 study (1 national)

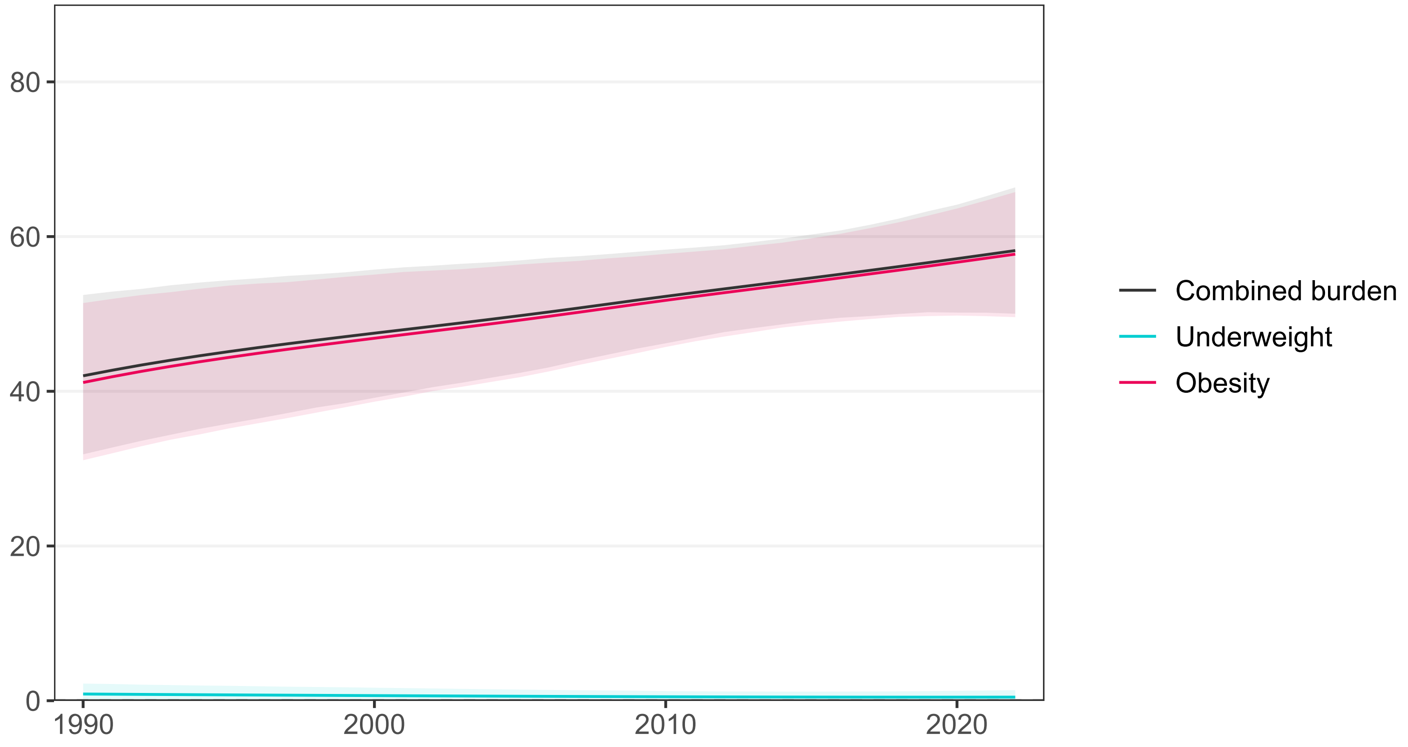

# Uganda

## School-aged children and adolescents

Girls
9 studies (6 national)

Boys
8 studies (5 national)

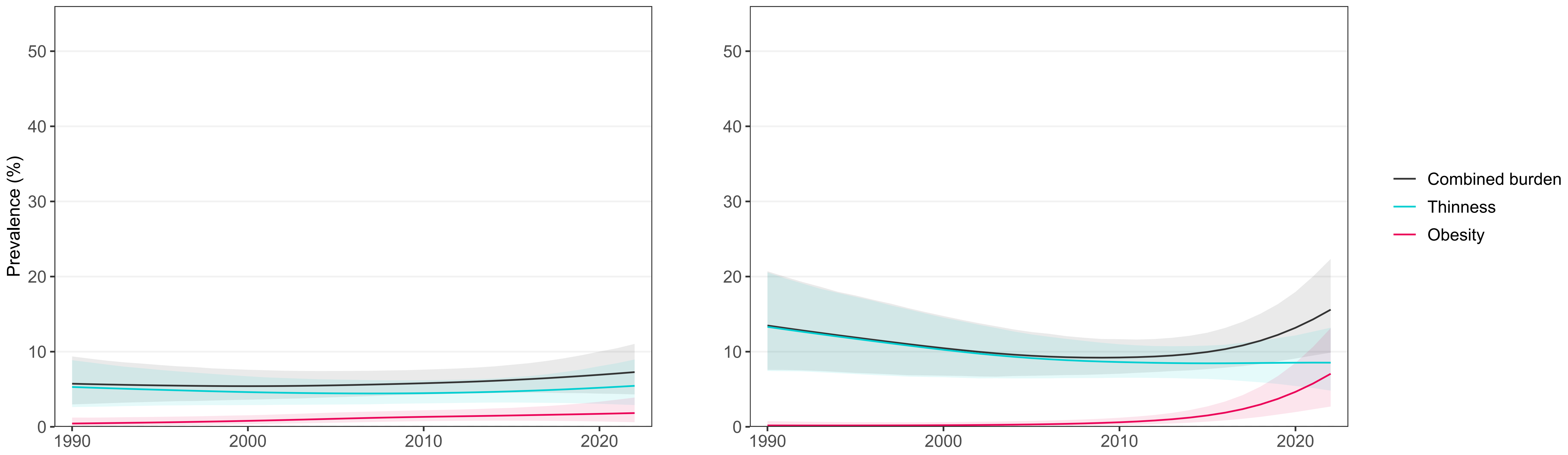


## Adults

Women
12 studies (7 national)

Men
10 studies (5 national)

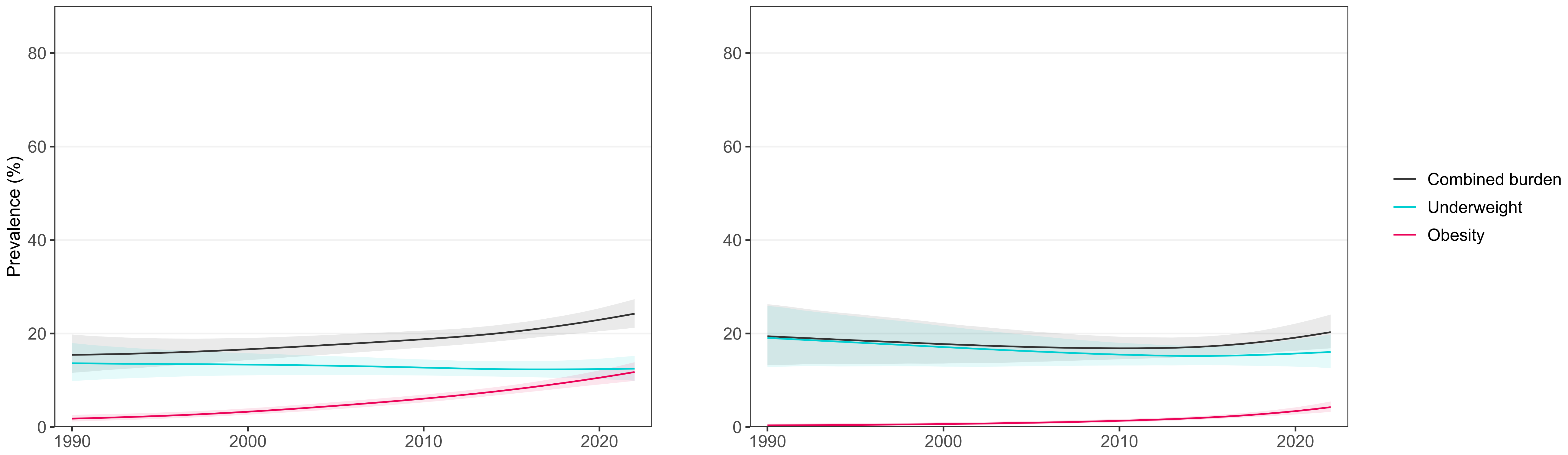

# Ukraine

## School-aged children and adolescents

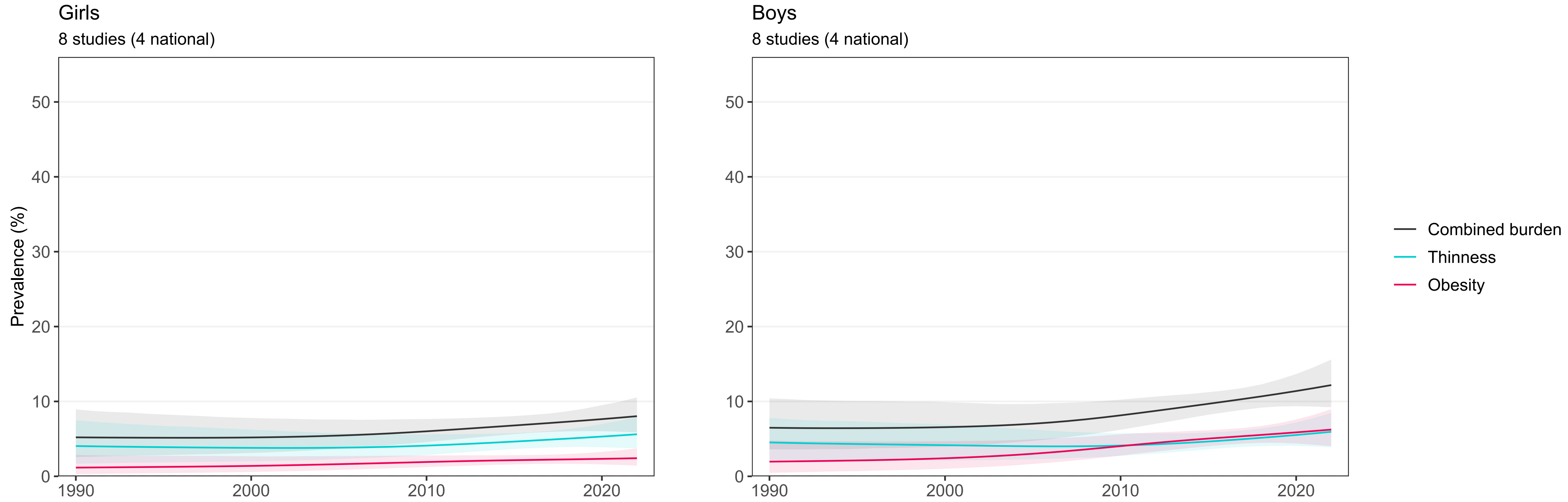


## Adults

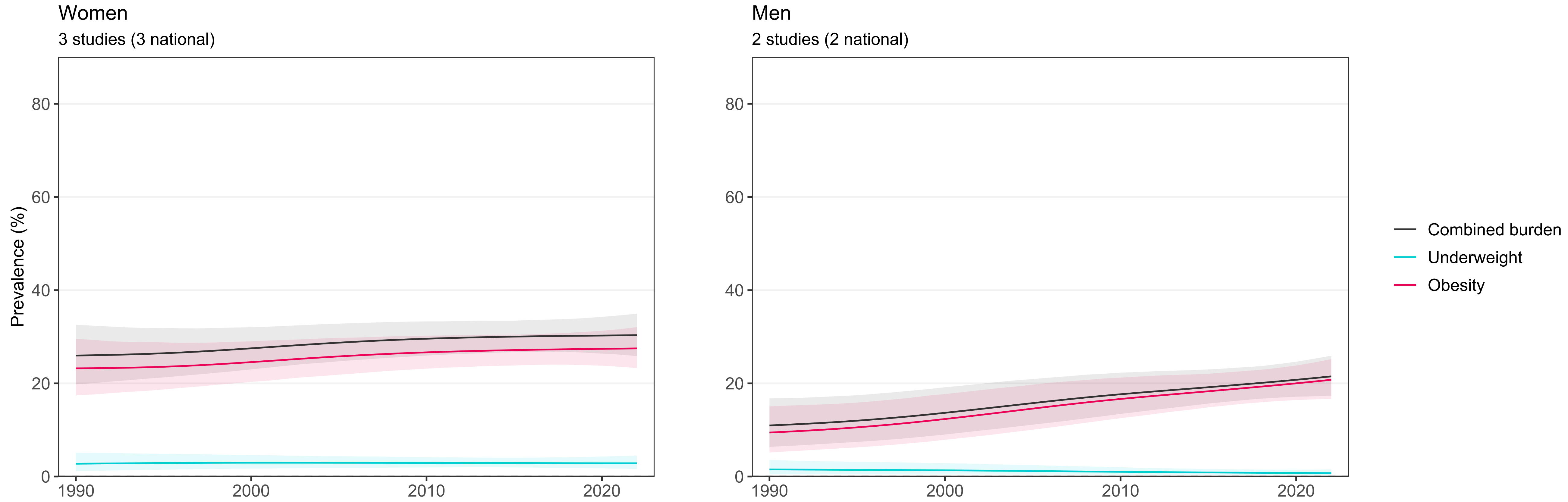

# United Arab Emirates

## School-aged children and adolescents

### Girls

5 studies (5 national)

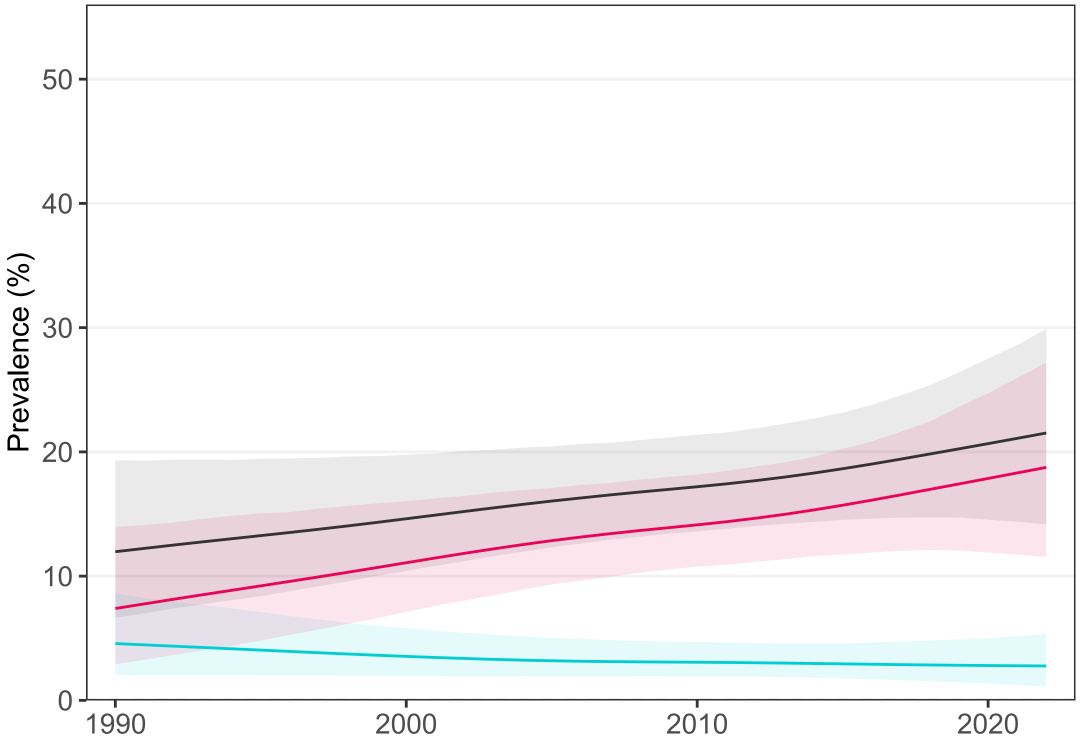


### Boys

5 studies (5 national)

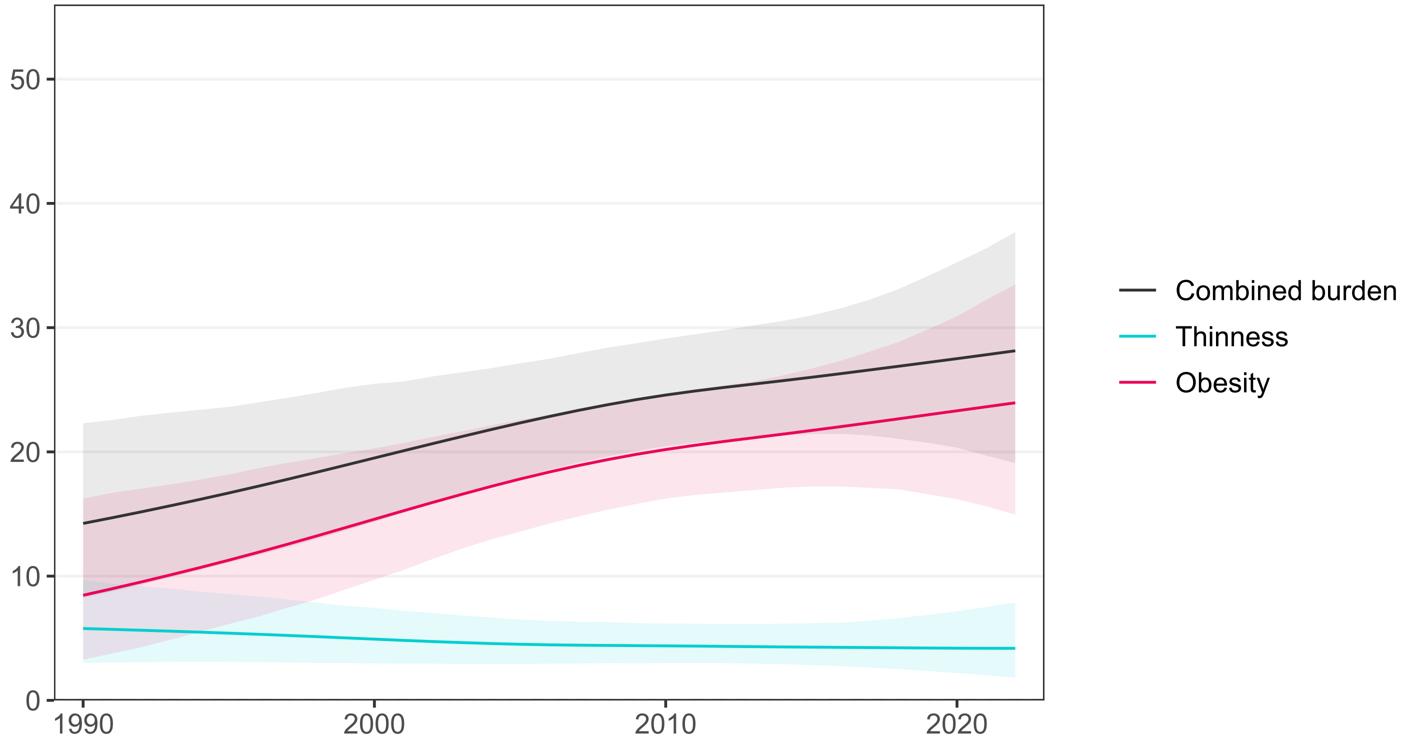


## Adults

### Women

5 studies (3 national)

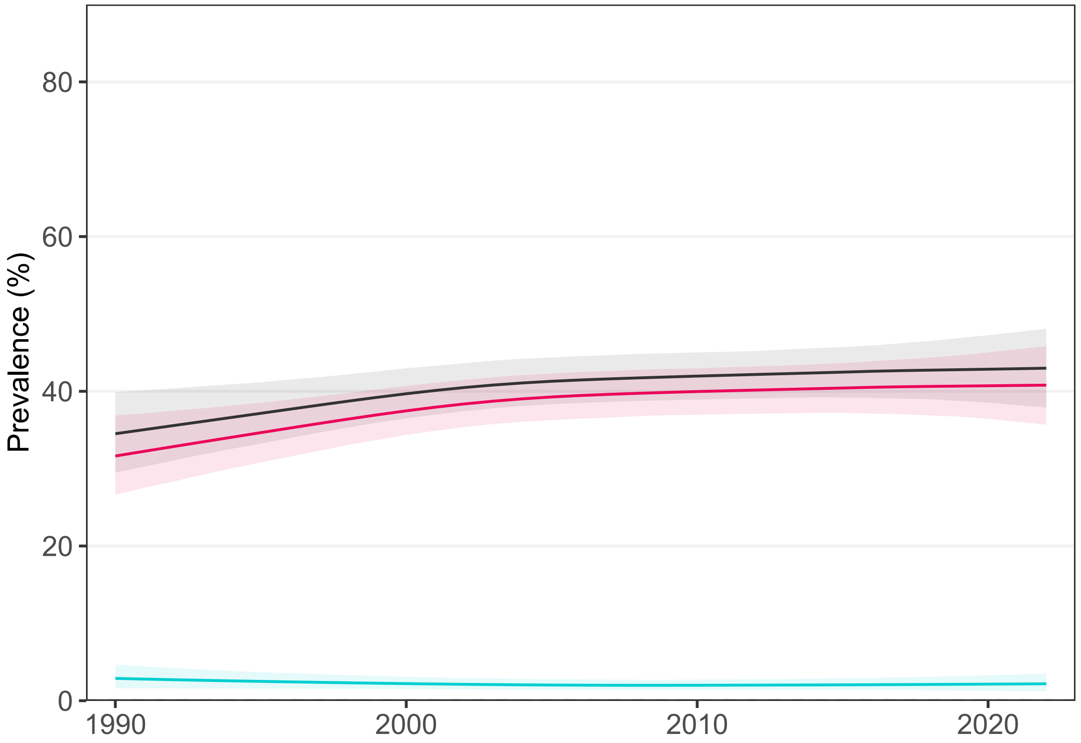


### Men

4 studies (3 national)

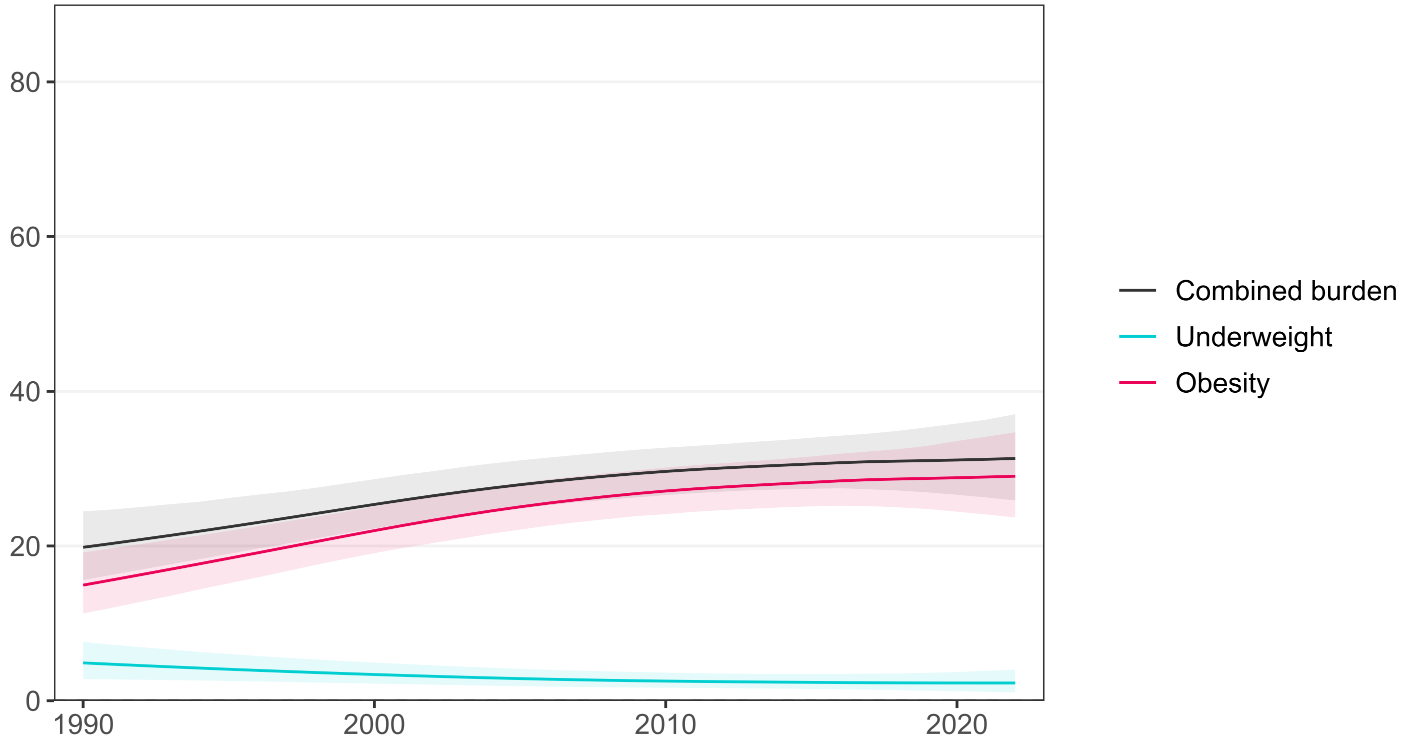

# United Kingdom

## School-aged children and adolescents

### Girls

104 studies (56 national)

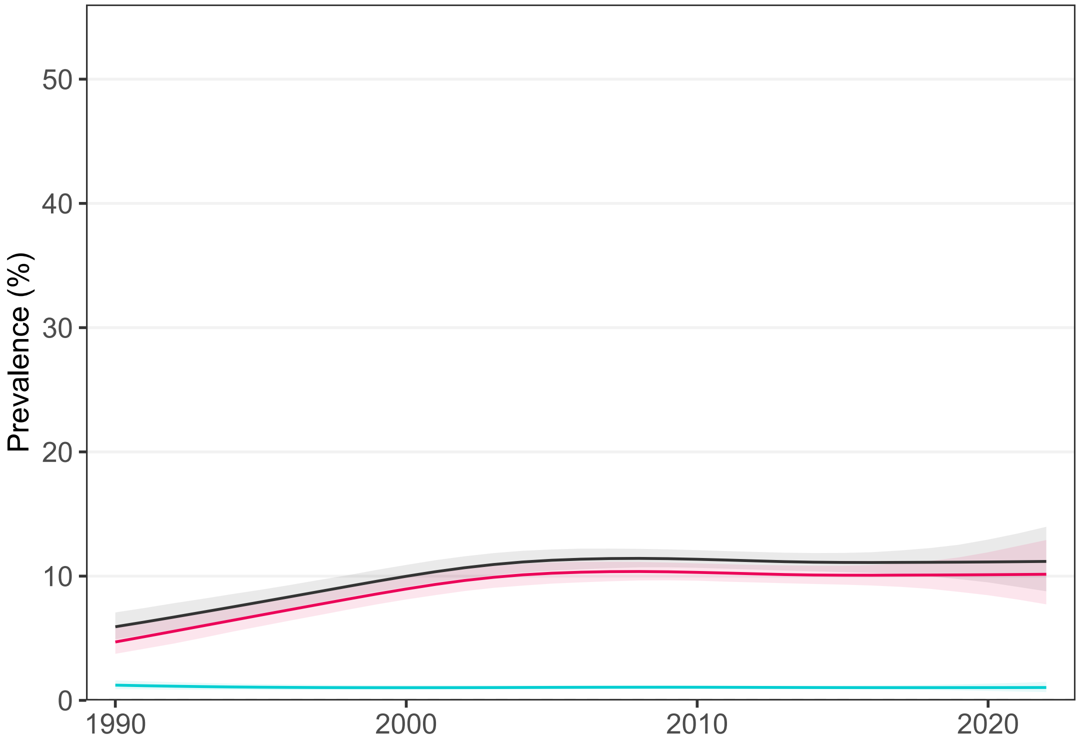


### Boys

104 studies (56 national)

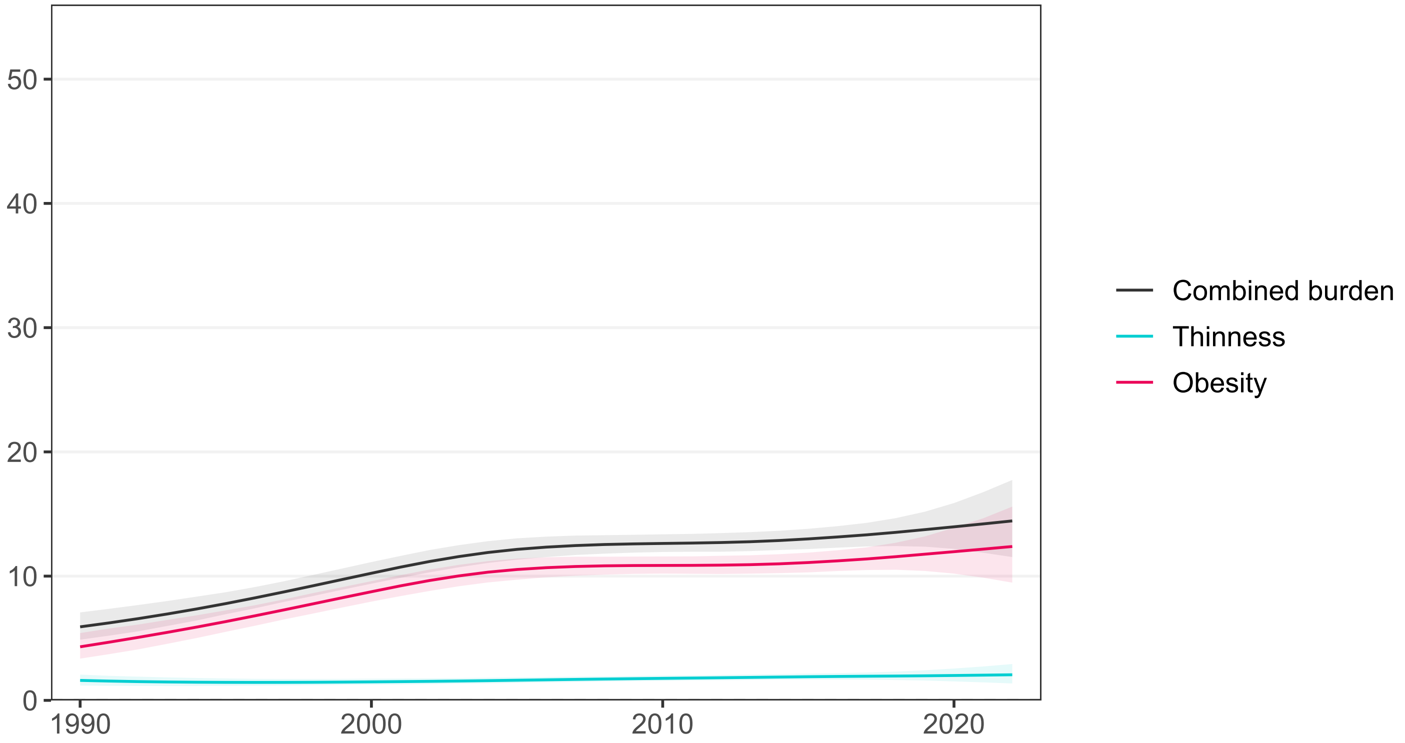


## Adults

### Women

85 studies (50 national)

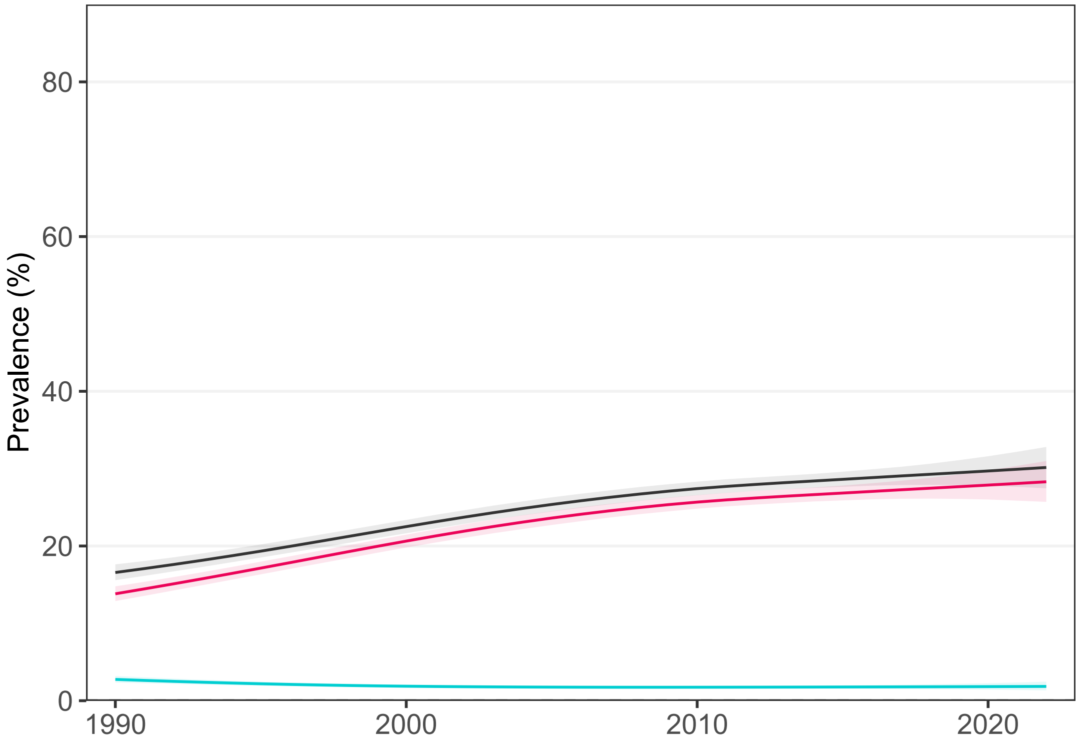


### Men

88 studies (51 national)

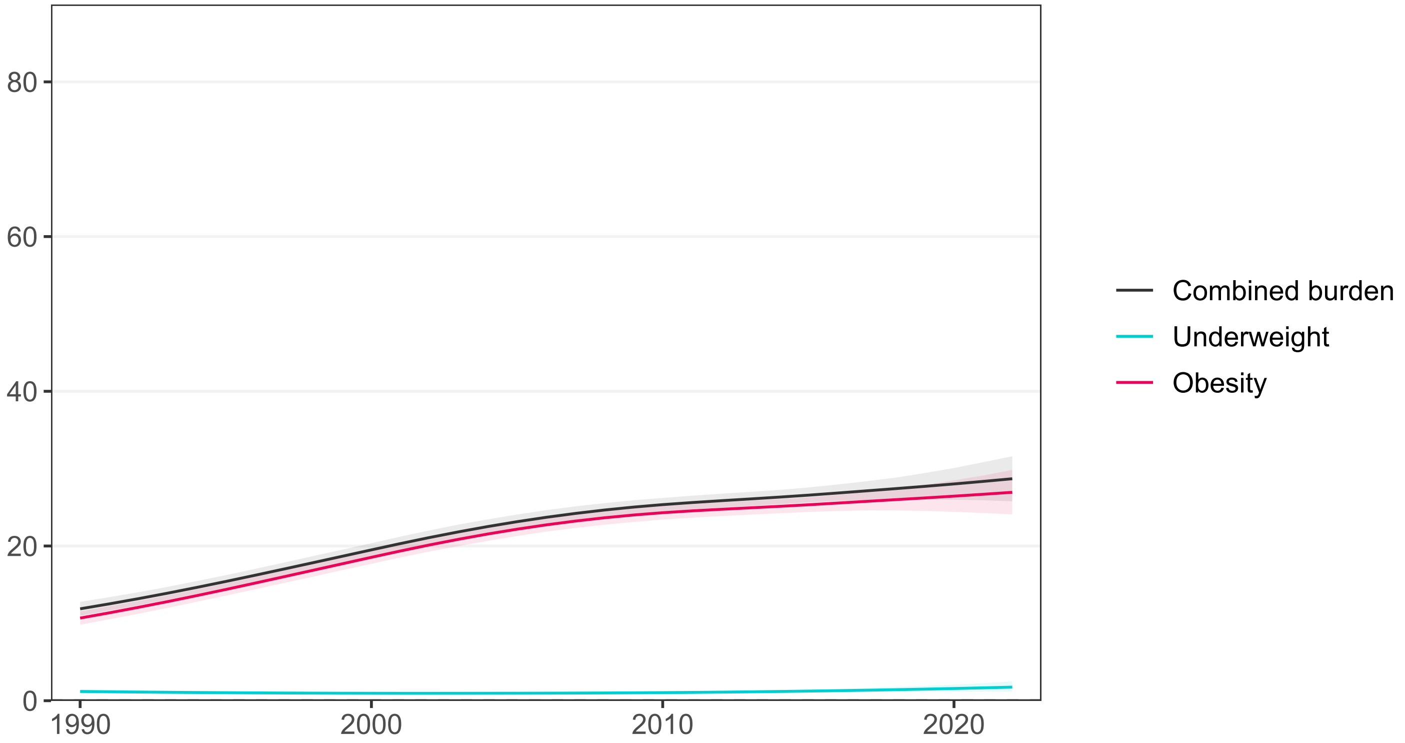

# Uruguay

## School-aged children and adolescents

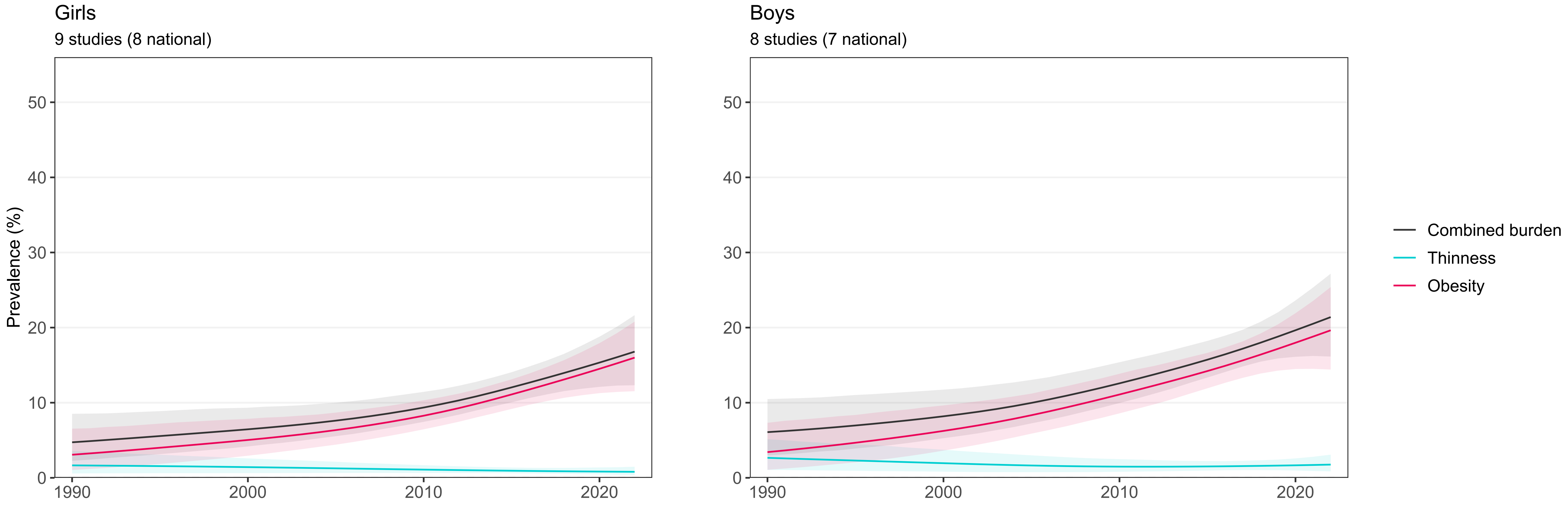


## Adults

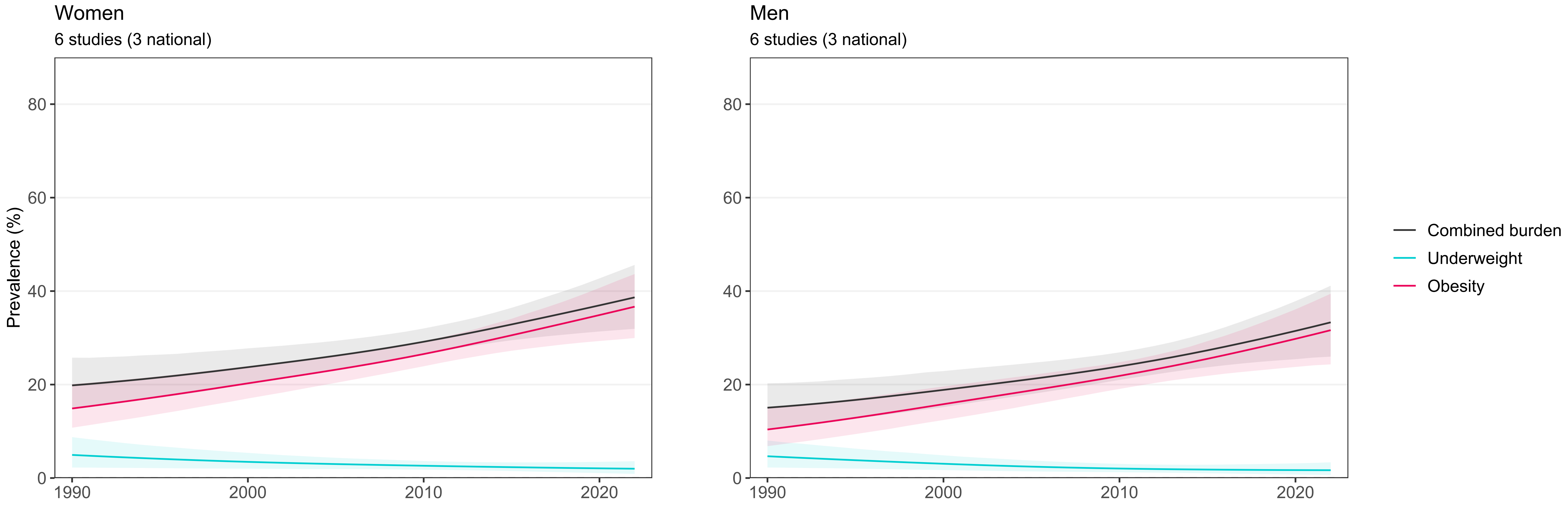

# USA

## School-aged children and adolescents

### Girls

61 studies (28 national)

### Boys

61 studies (28 national)

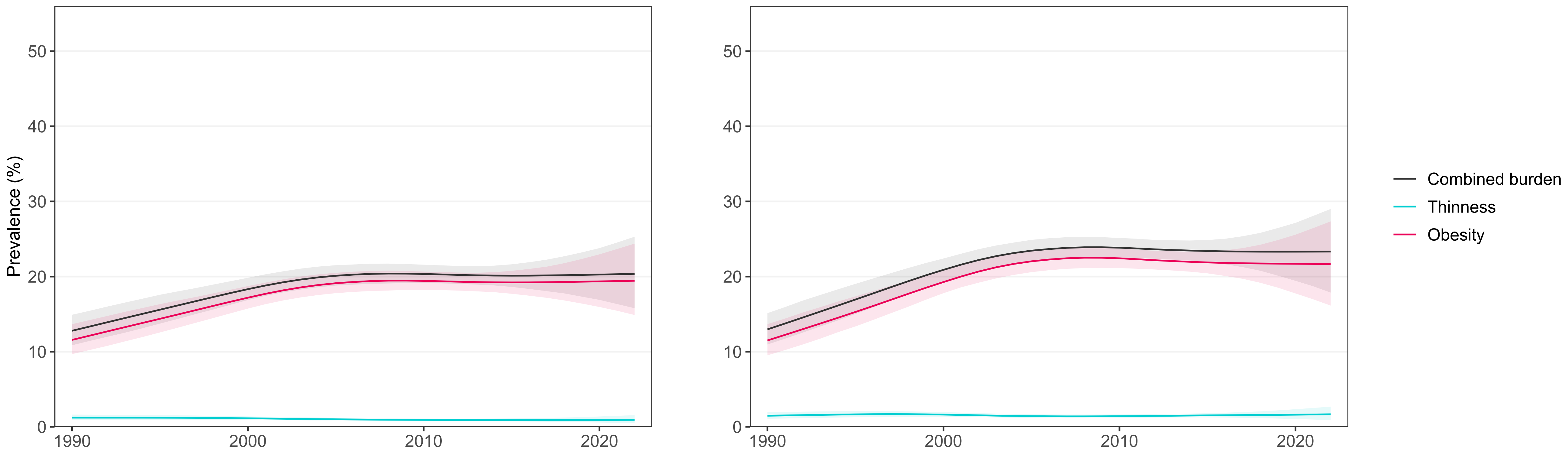


## Adults

### Women

70 studies (26 national)

### Men

64 studies (25 national)

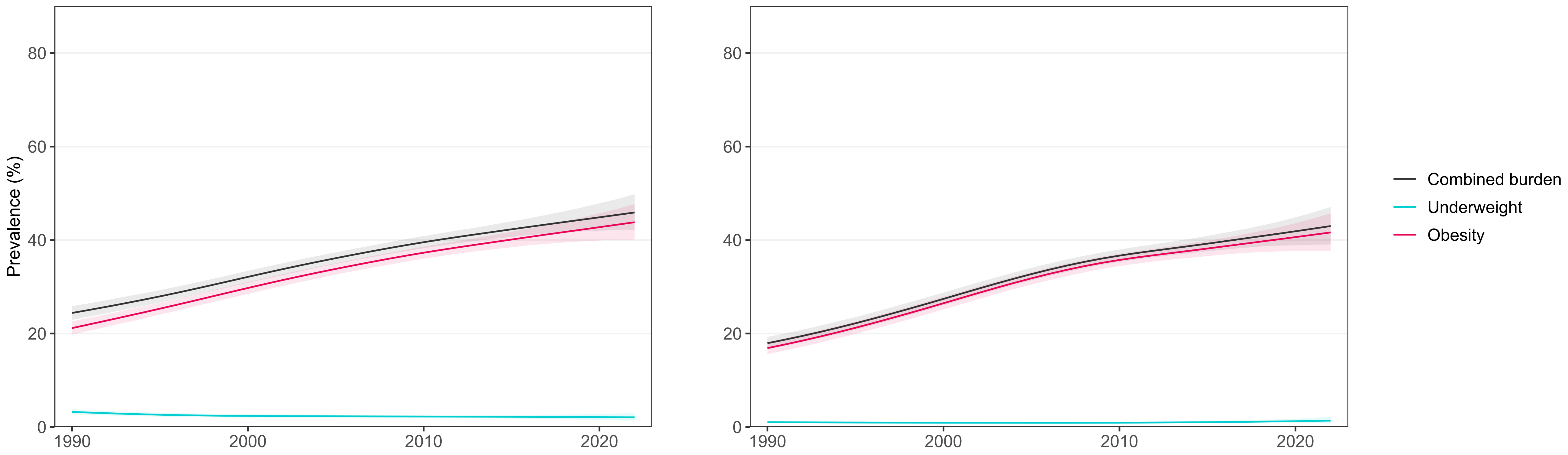

# Uzbekistan

## School-aged children and adolescents

### Girls

4 studies (4 national)

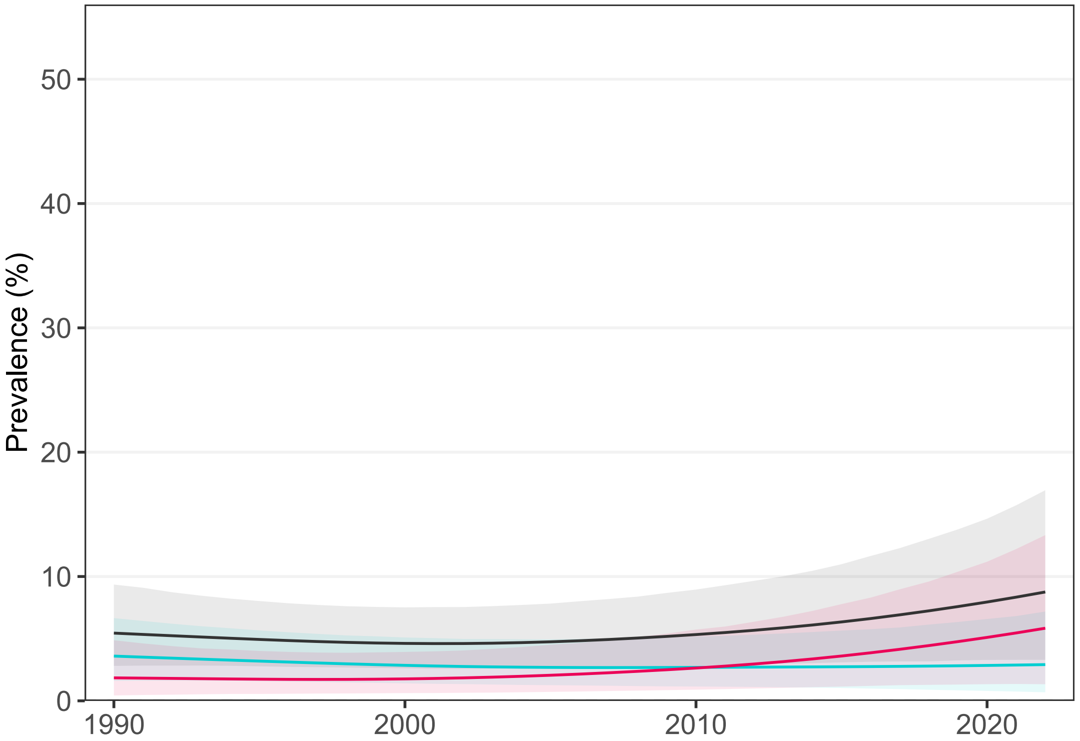


### Boys

3 studies (3 national)

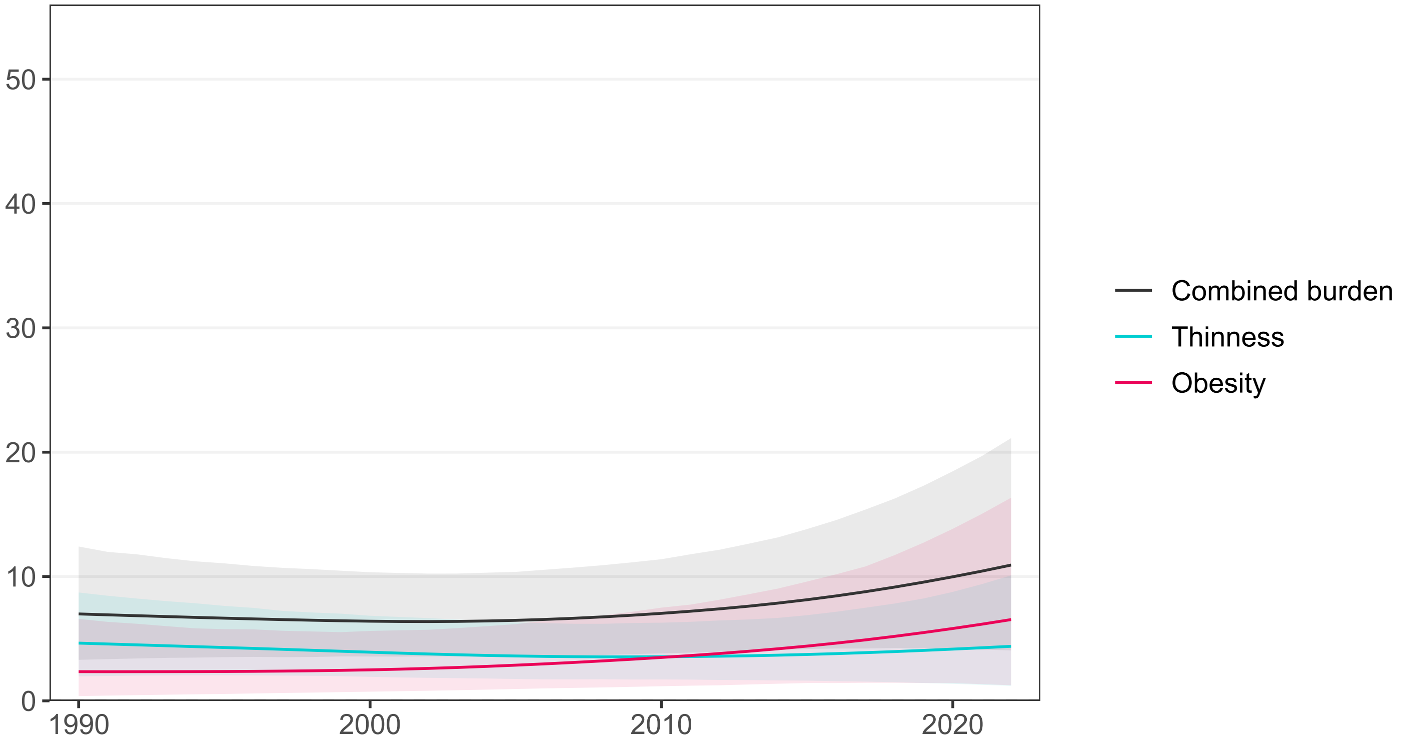


## Adults

### Women

5 studies (4 national)

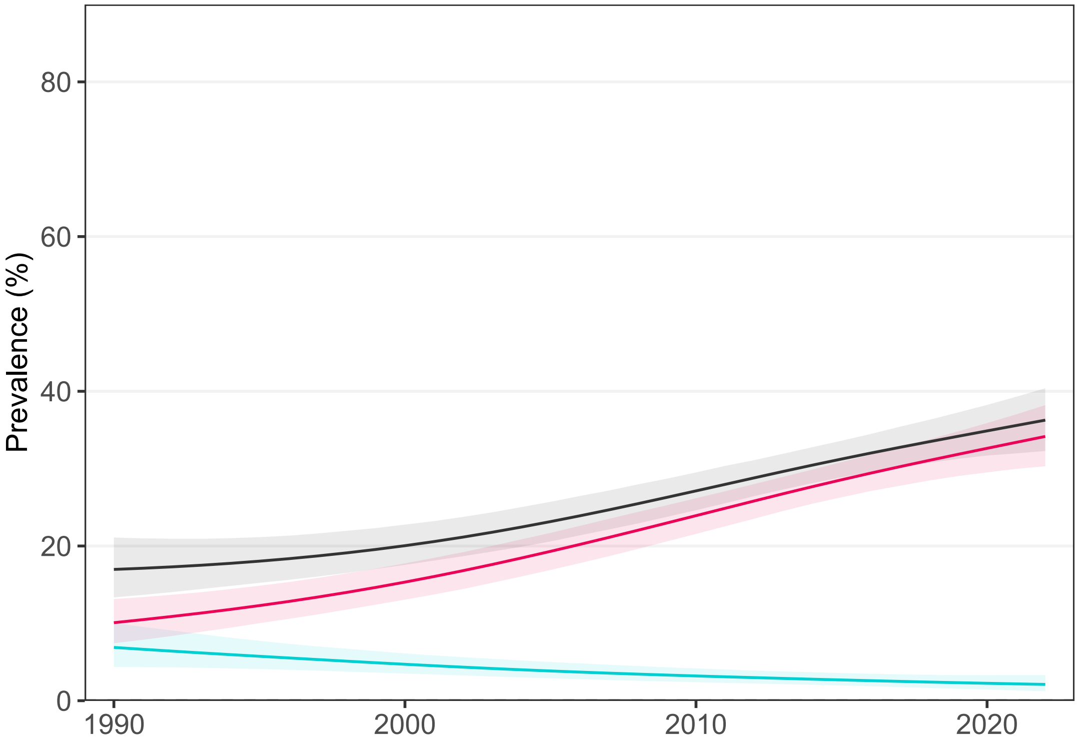


### Men

4 studies (3 national)

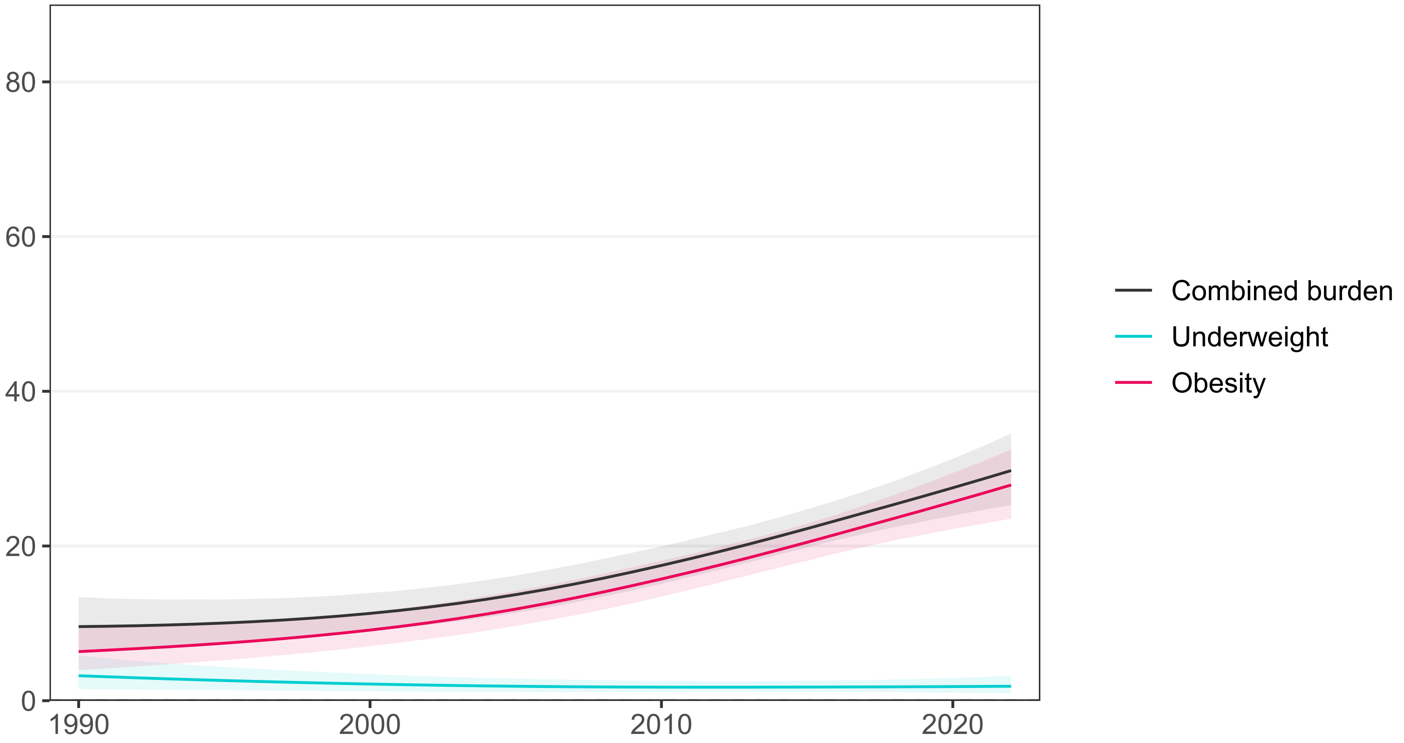

# Vanuatu

## School-aged children and adolescents

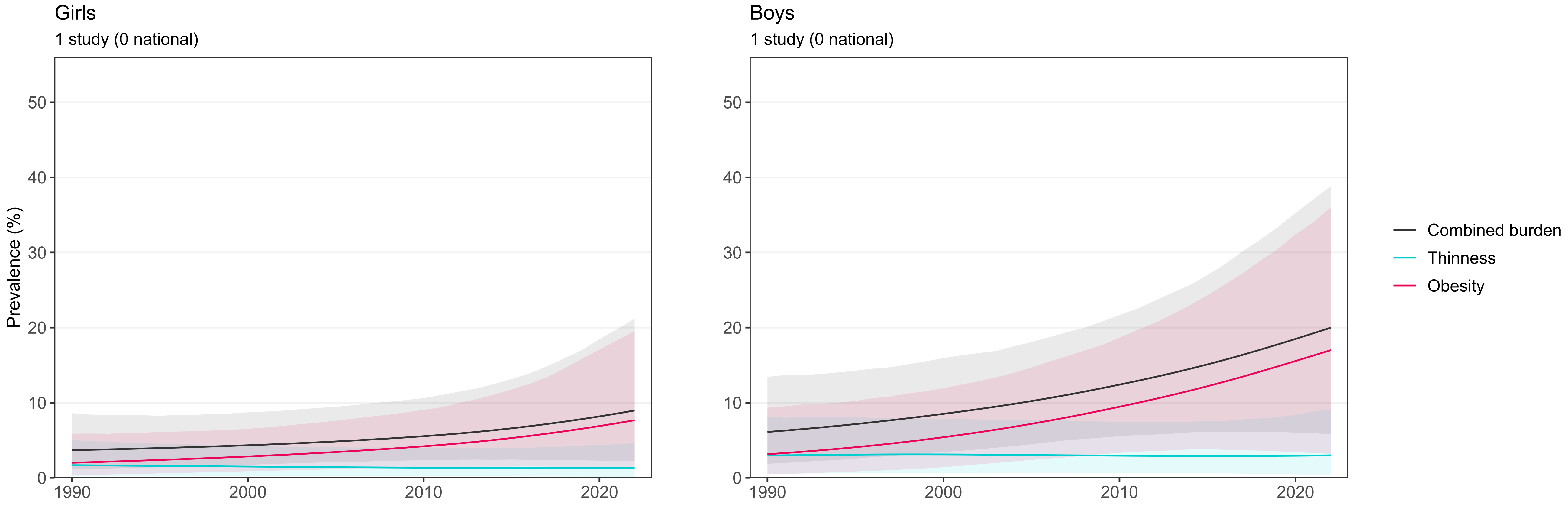


## Adults

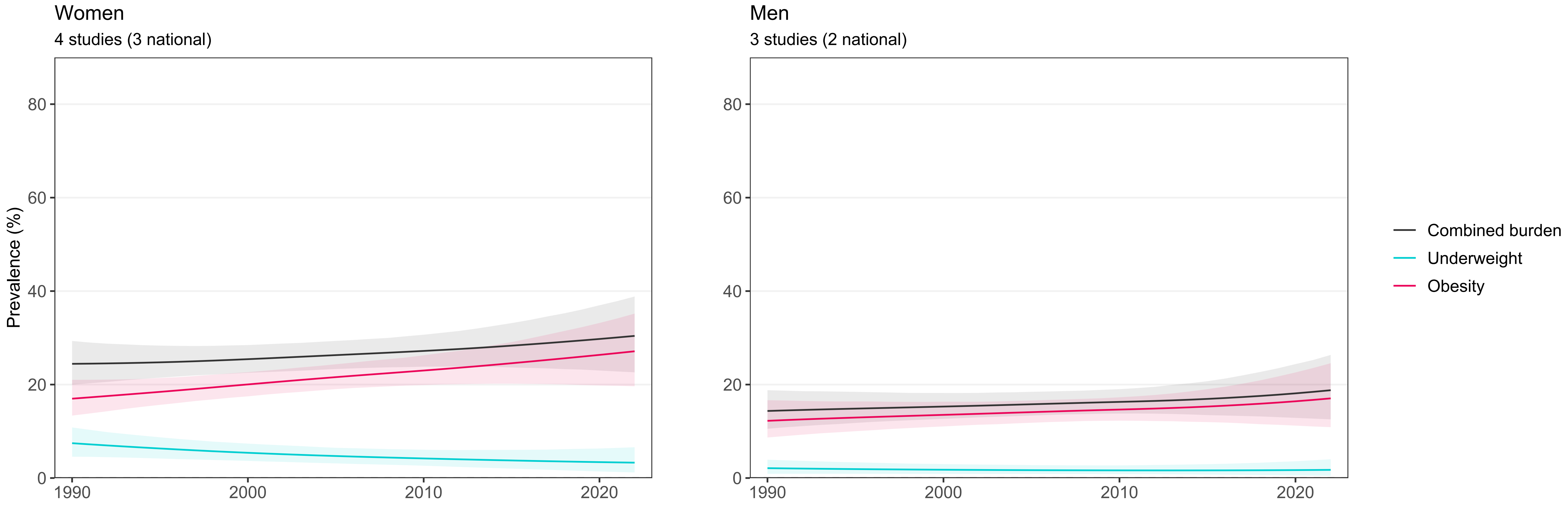

# Venezuela

## School-aged children and adolescents

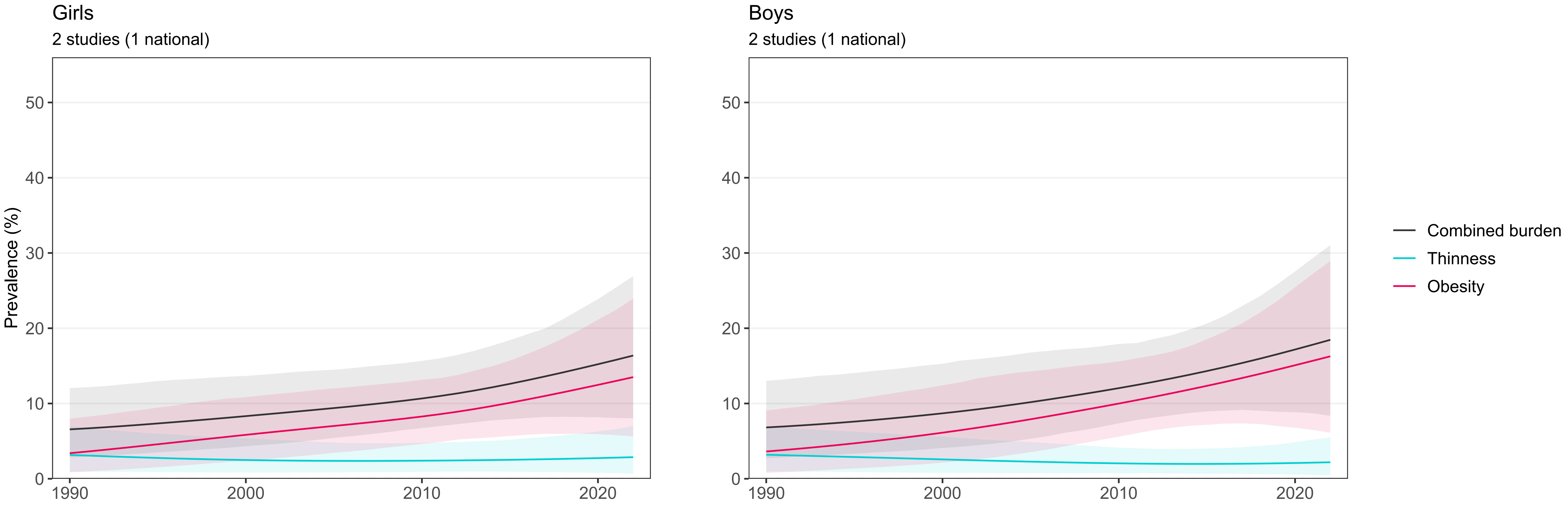


## Adults

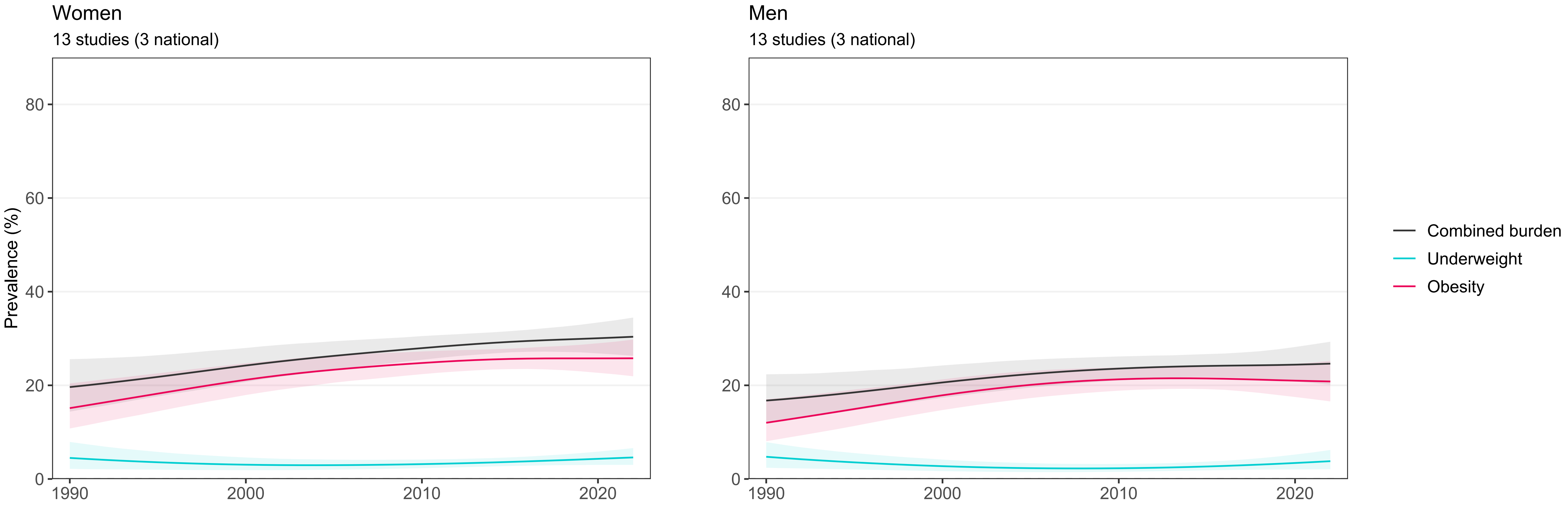

# Viet Nam

## School-aged children and adolescents

### Girls

11 studies (9 national)

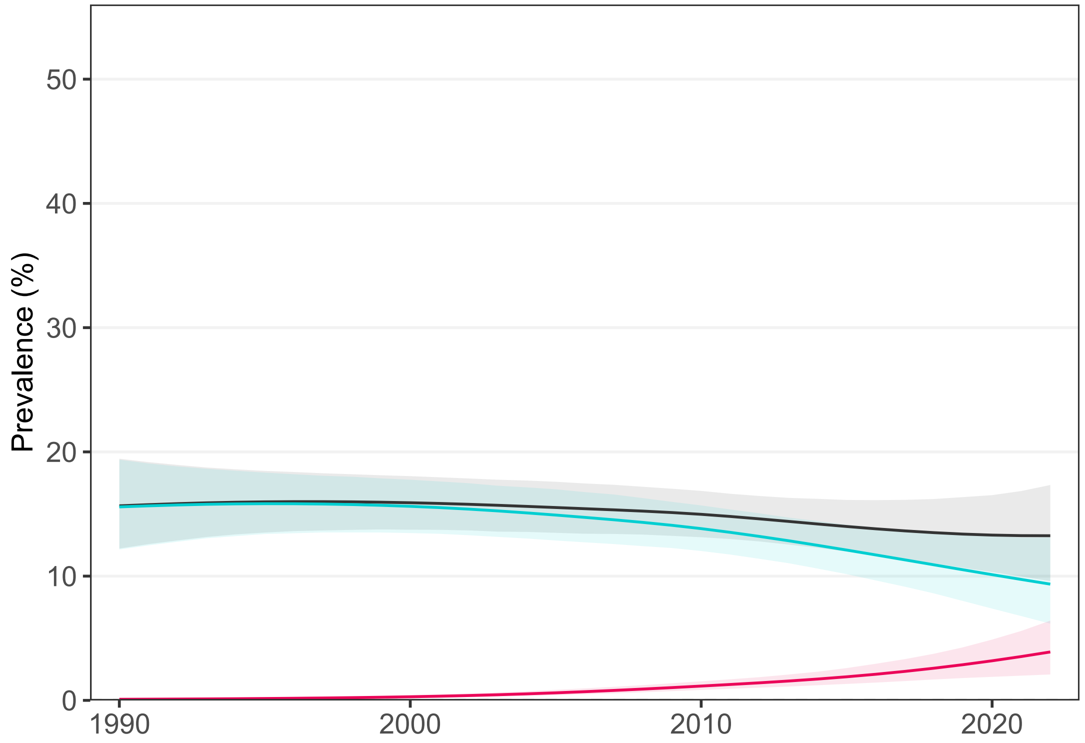


### Boys

11 studies (9 national)

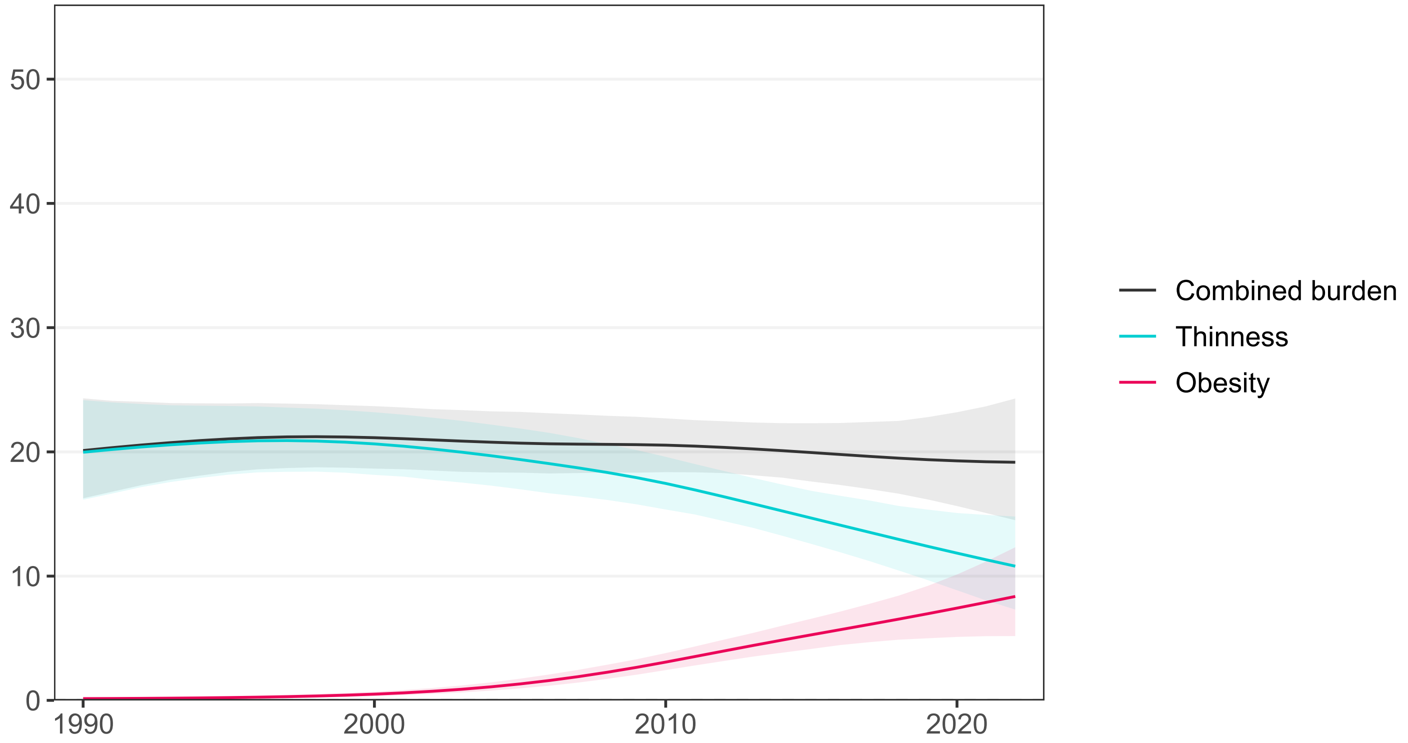


## Adults

### Women

25 studies (12 national)

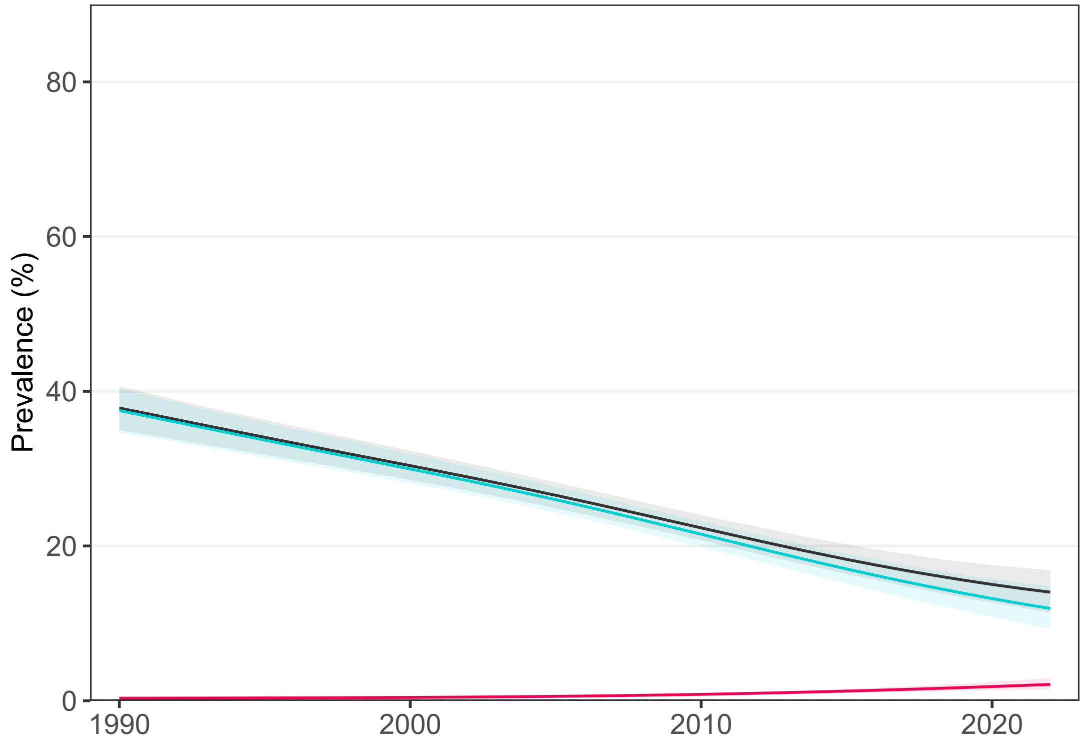


### Men

25 studies (12 national)

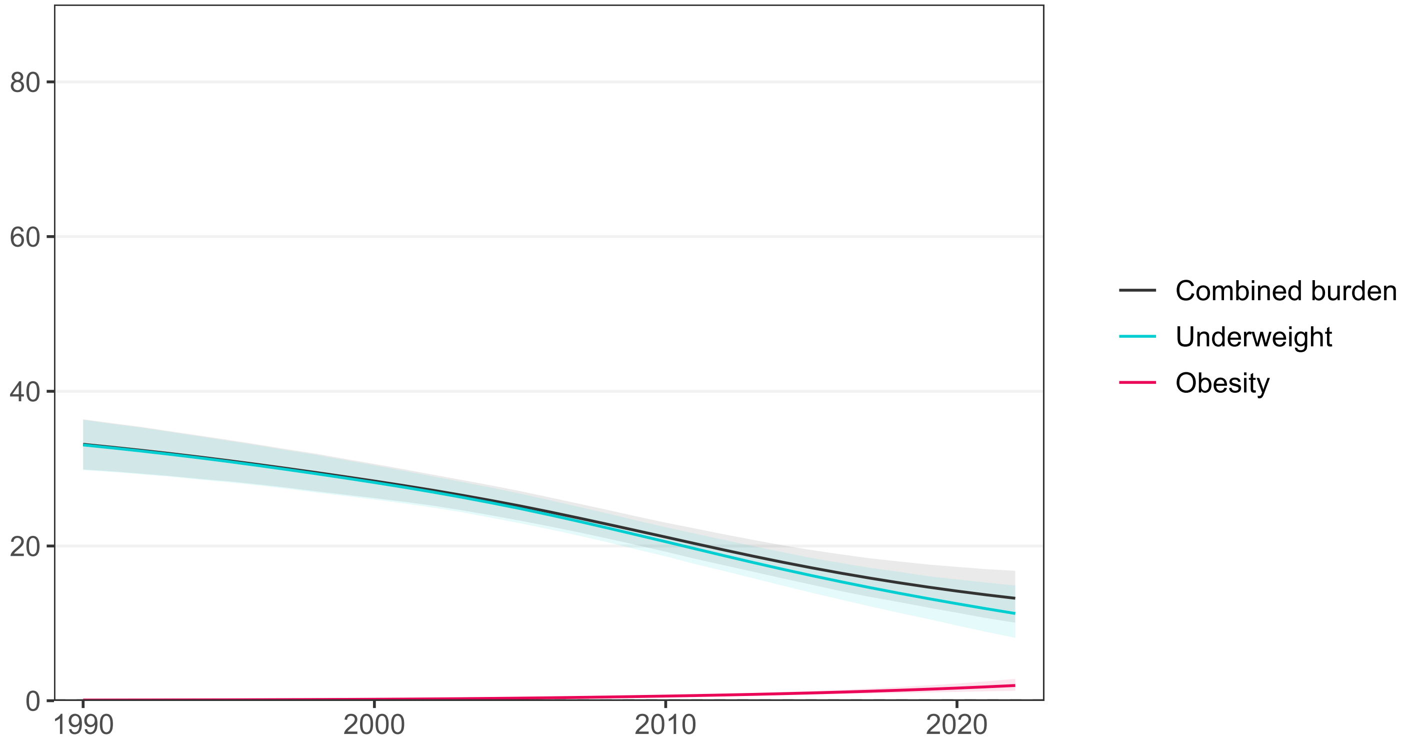

# Yemen

## School-aged children and adolescents

### Girls

4 studies (4 national)

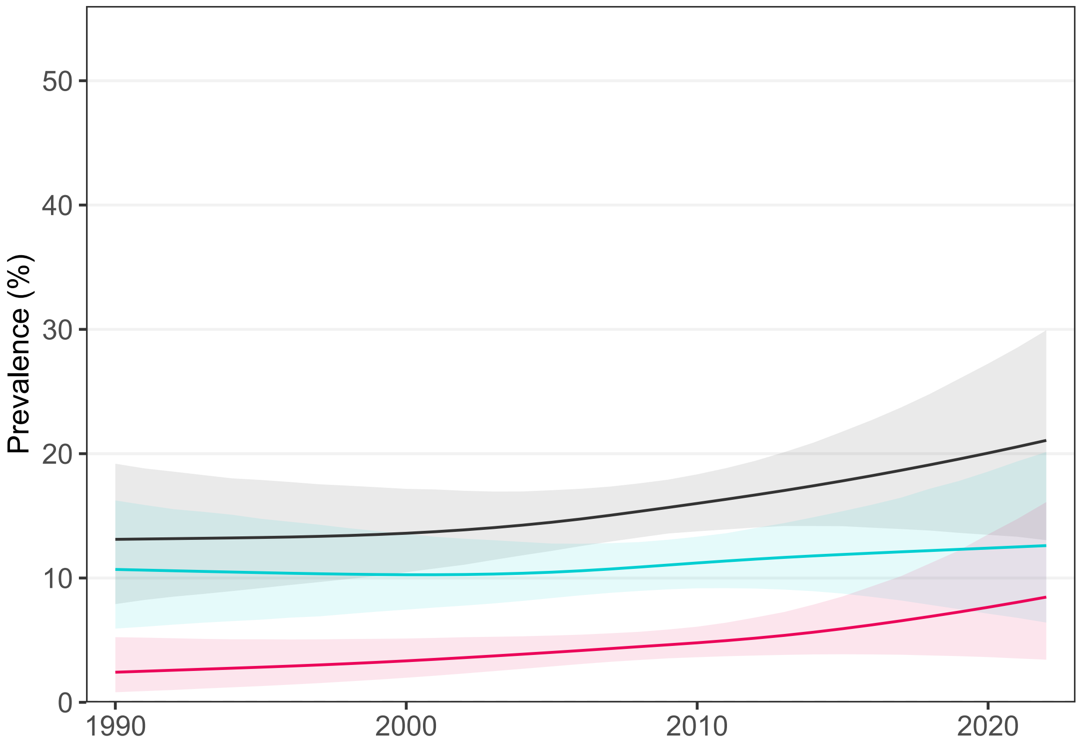


### Boys

3 studies (3 national)

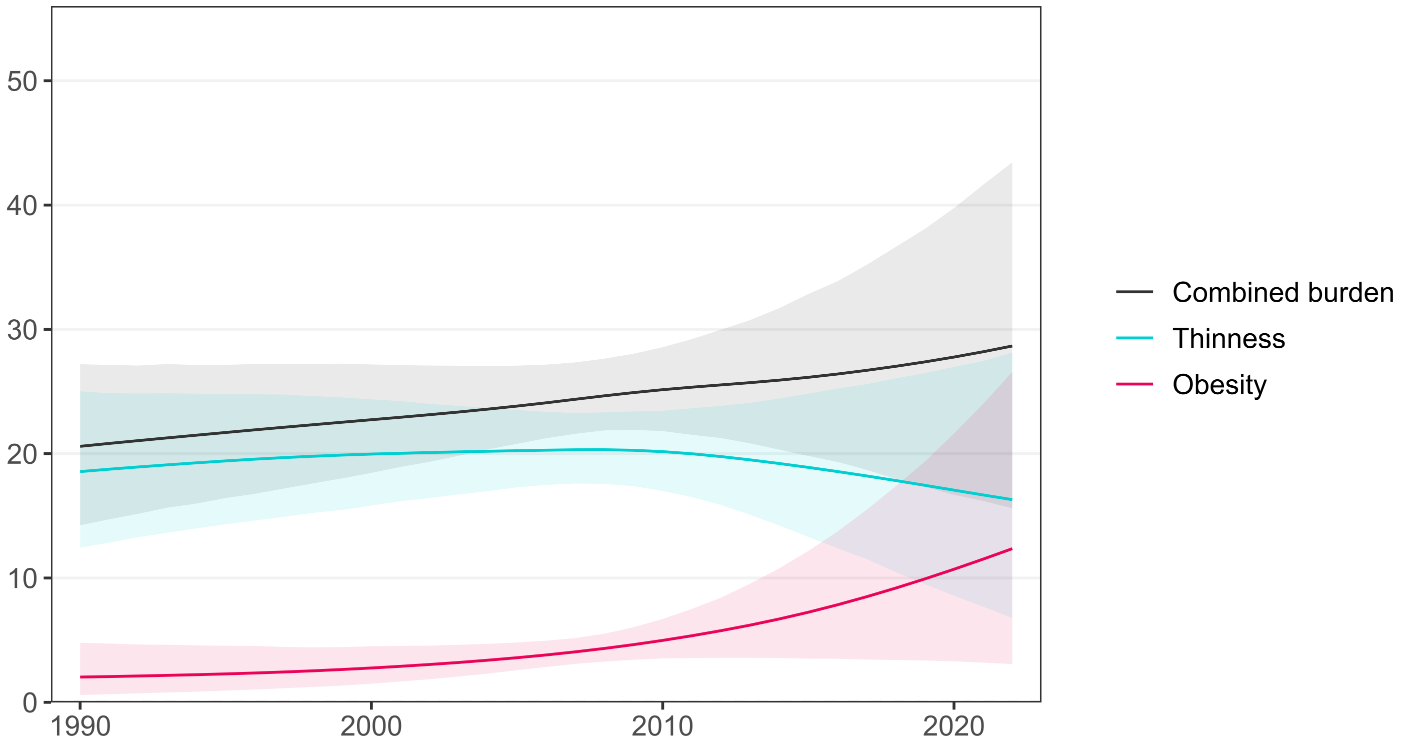


## Adults

### Women

5 studies (5 national)

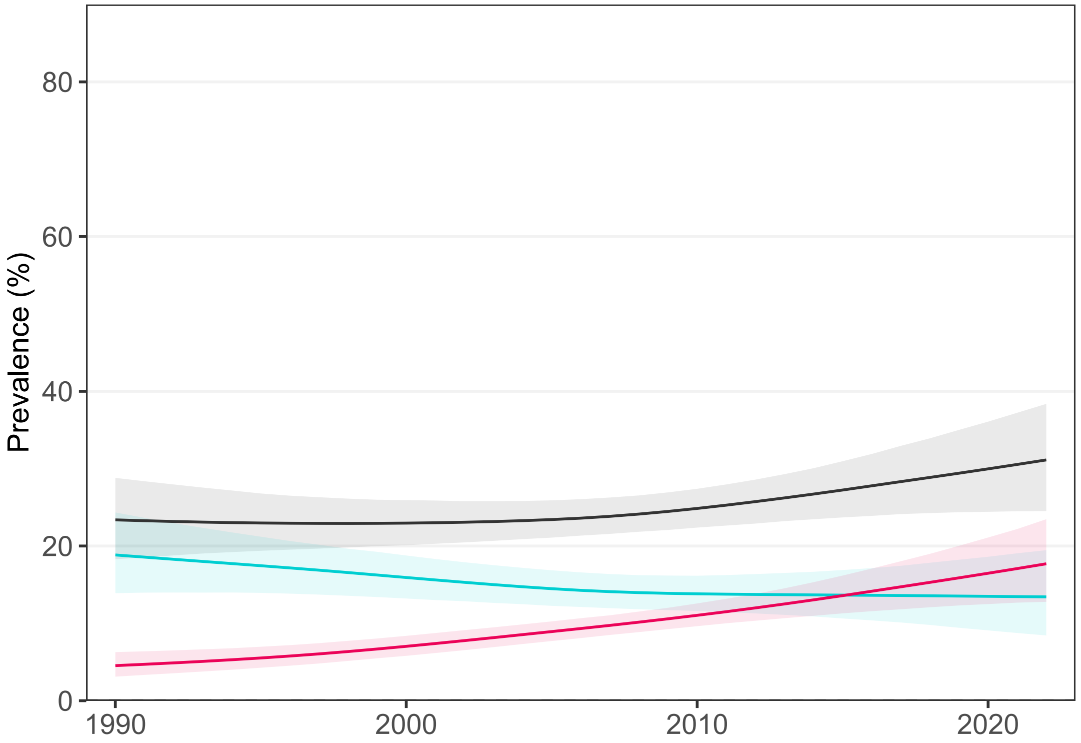


### Men

3 studies (3 national)

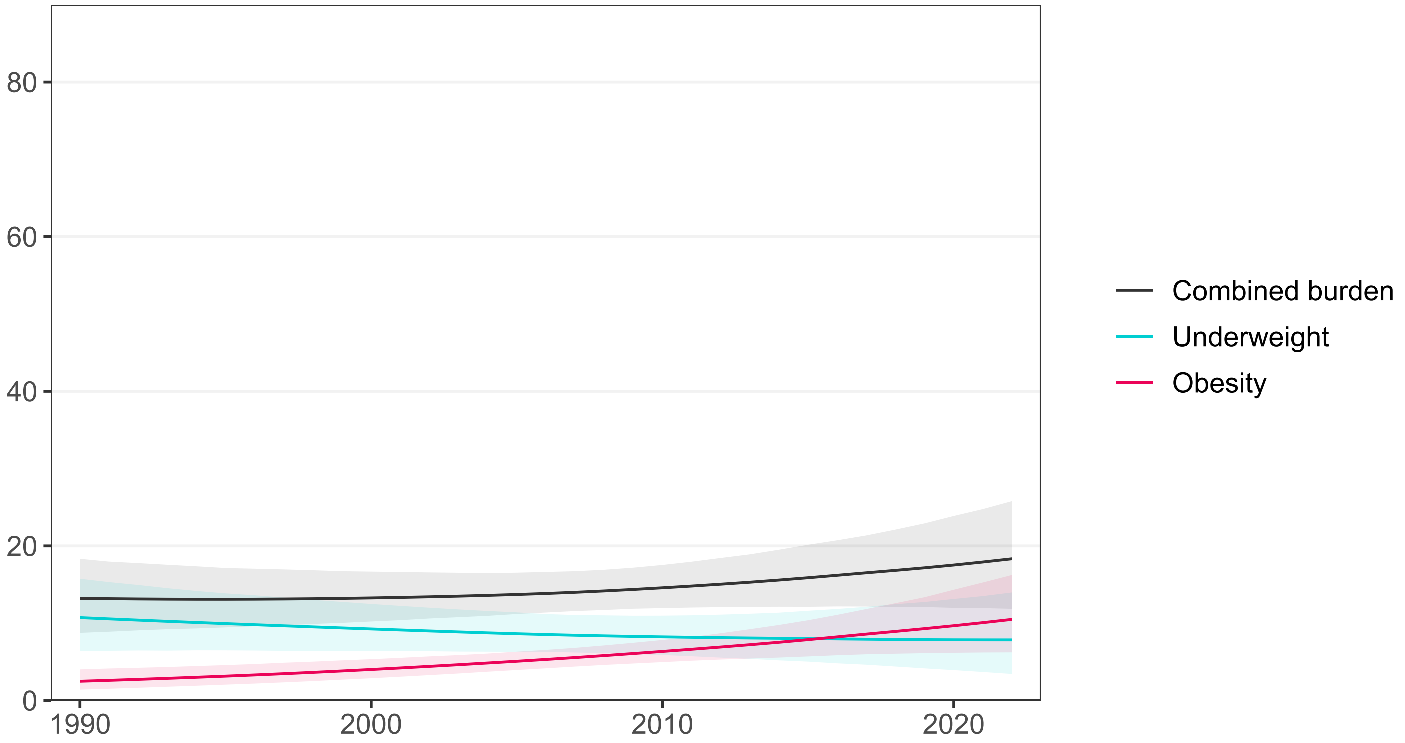

# Zambia

## School-aged children and adolescents

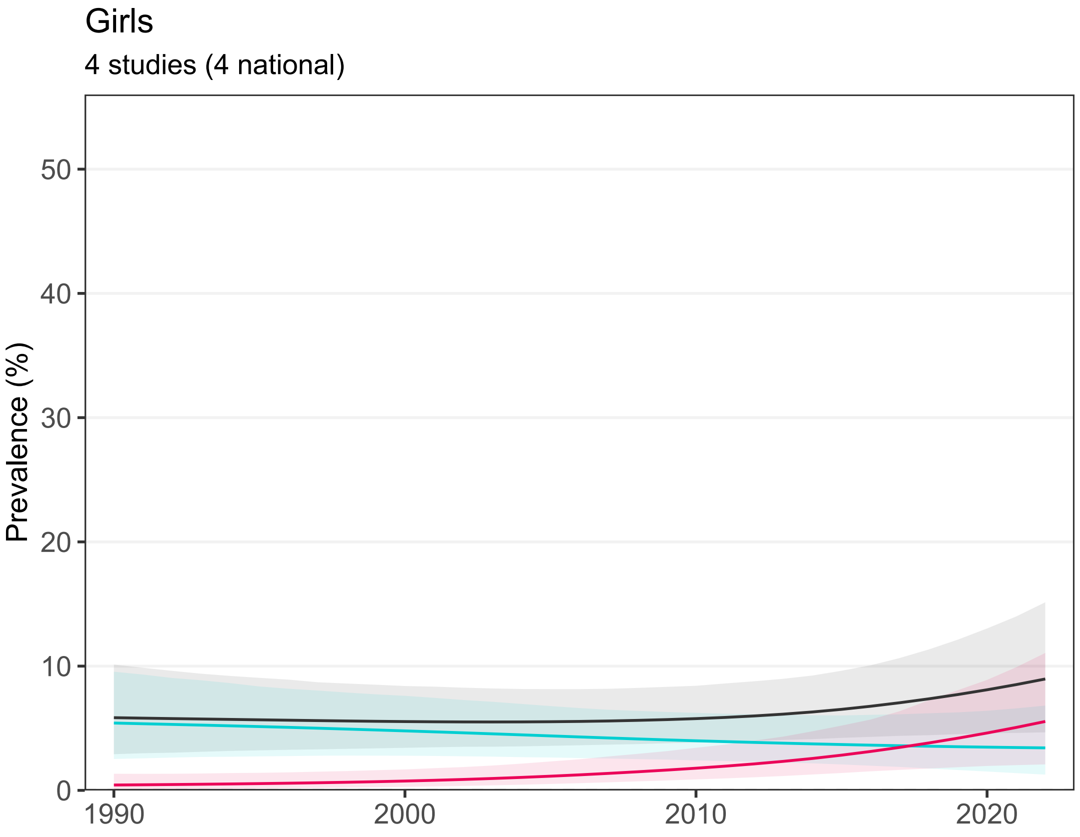


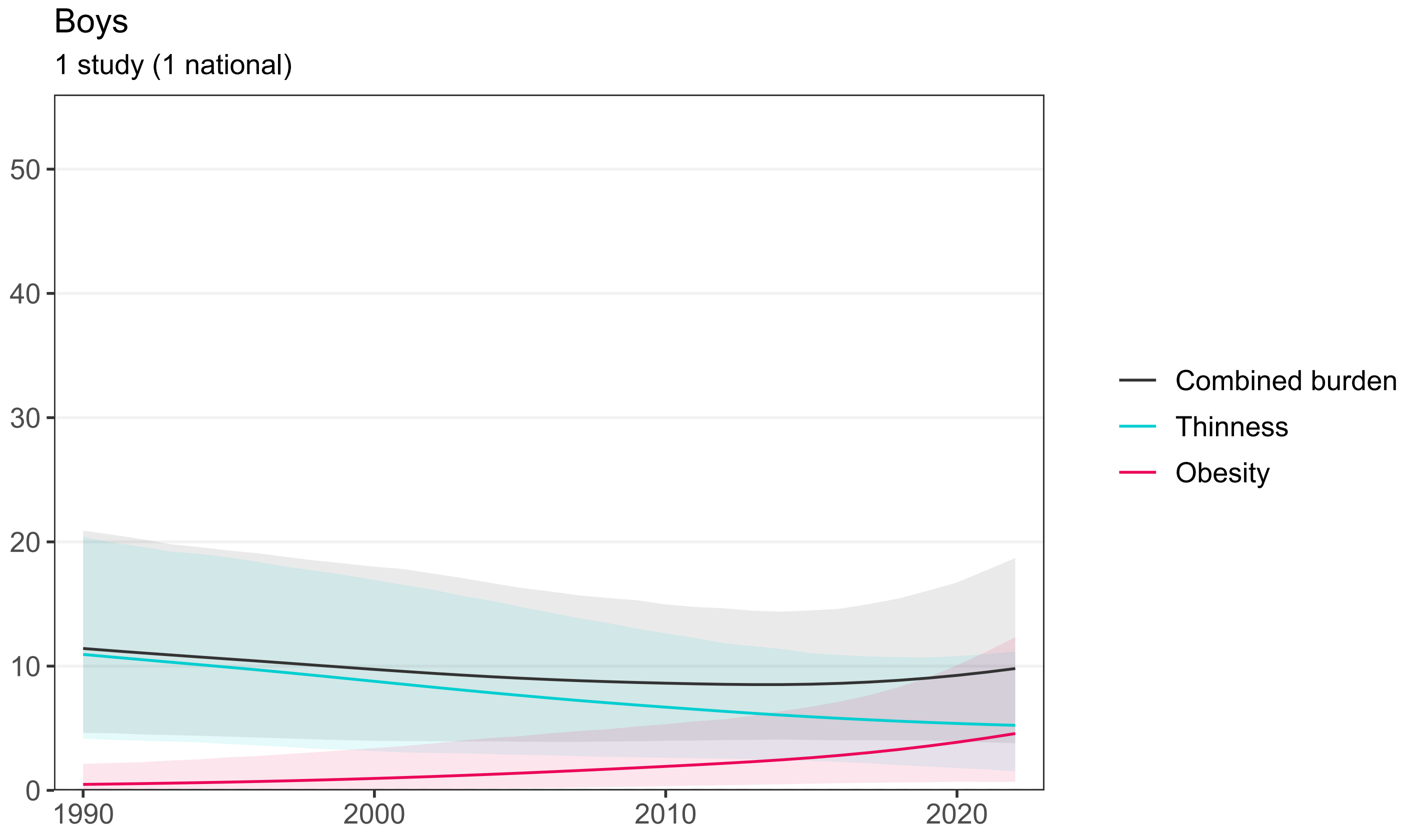


## Adults

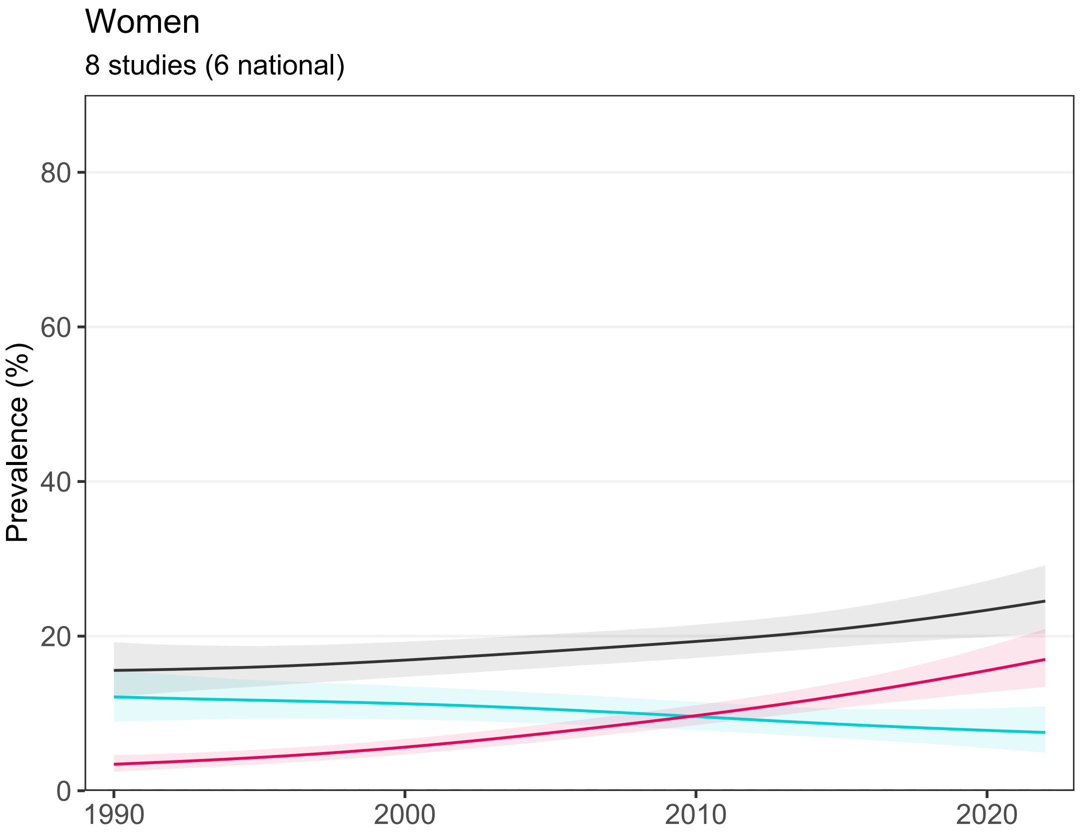


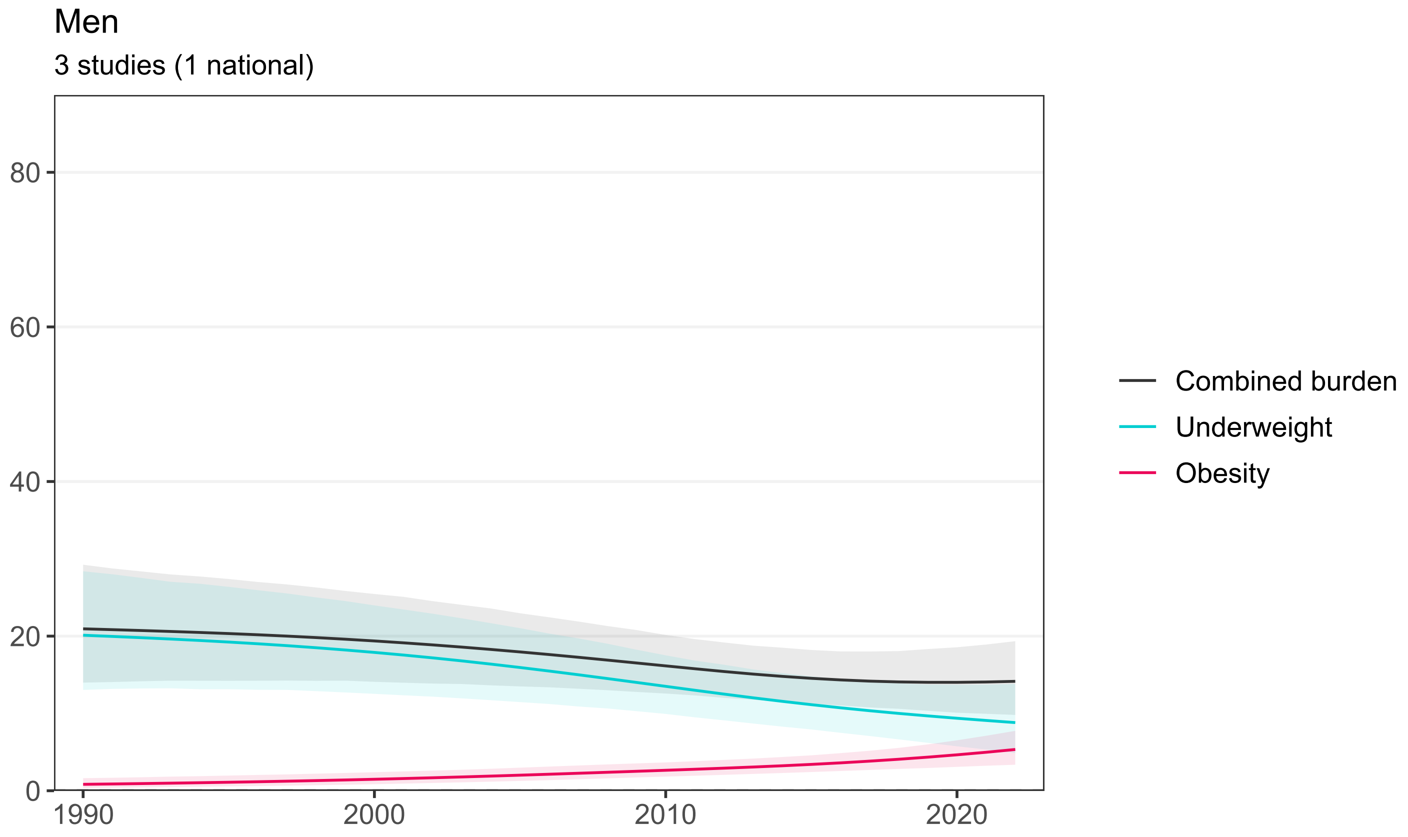

# Zimbabwe

## School-aged children and adolescents

### Girls
4 studies (4 national)

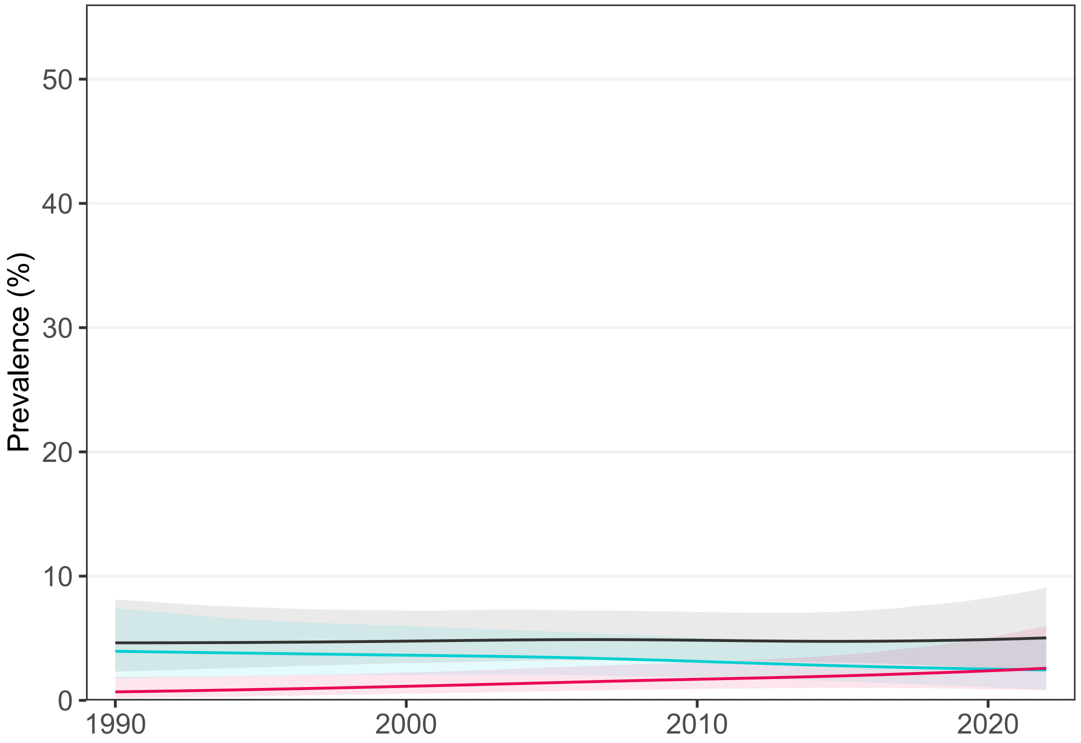


### Boys
2 studies (2 national)

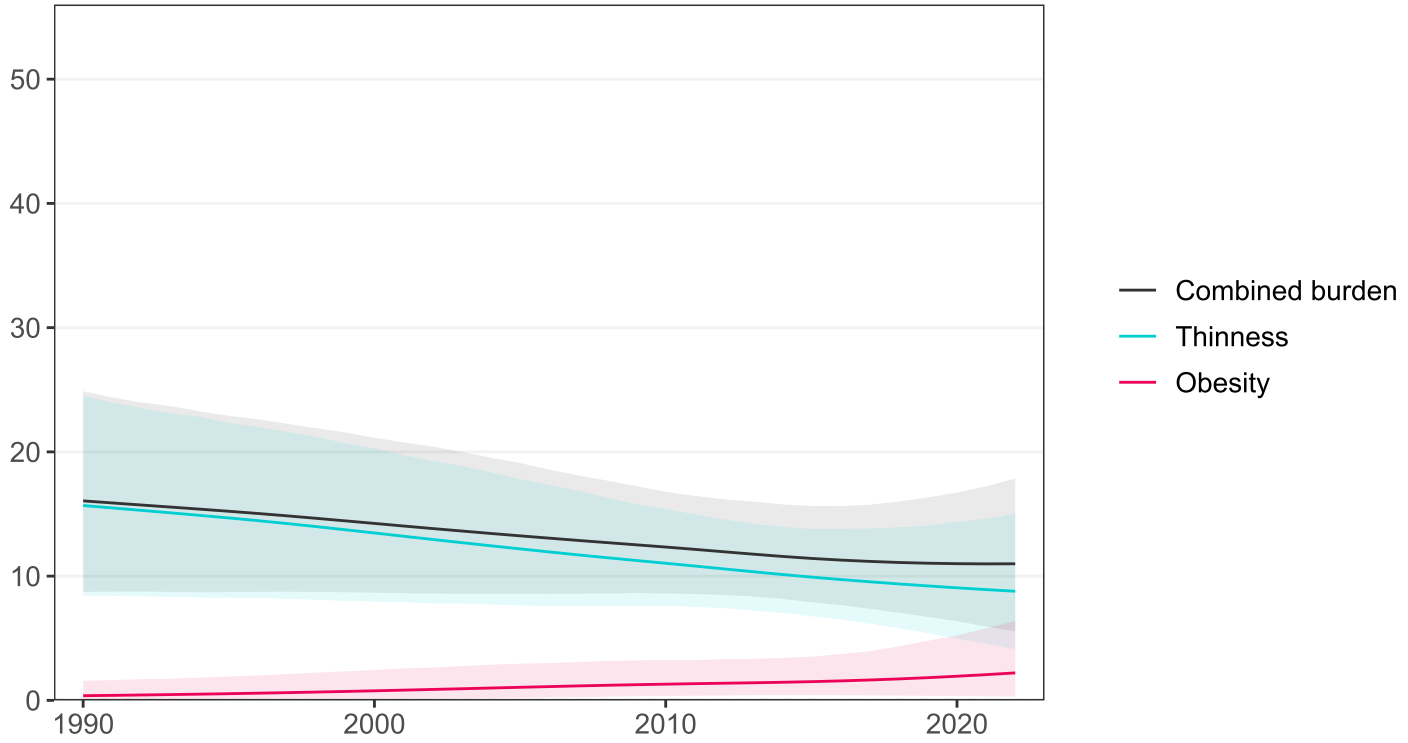


## Adults

### Women
9 studies (6 national)

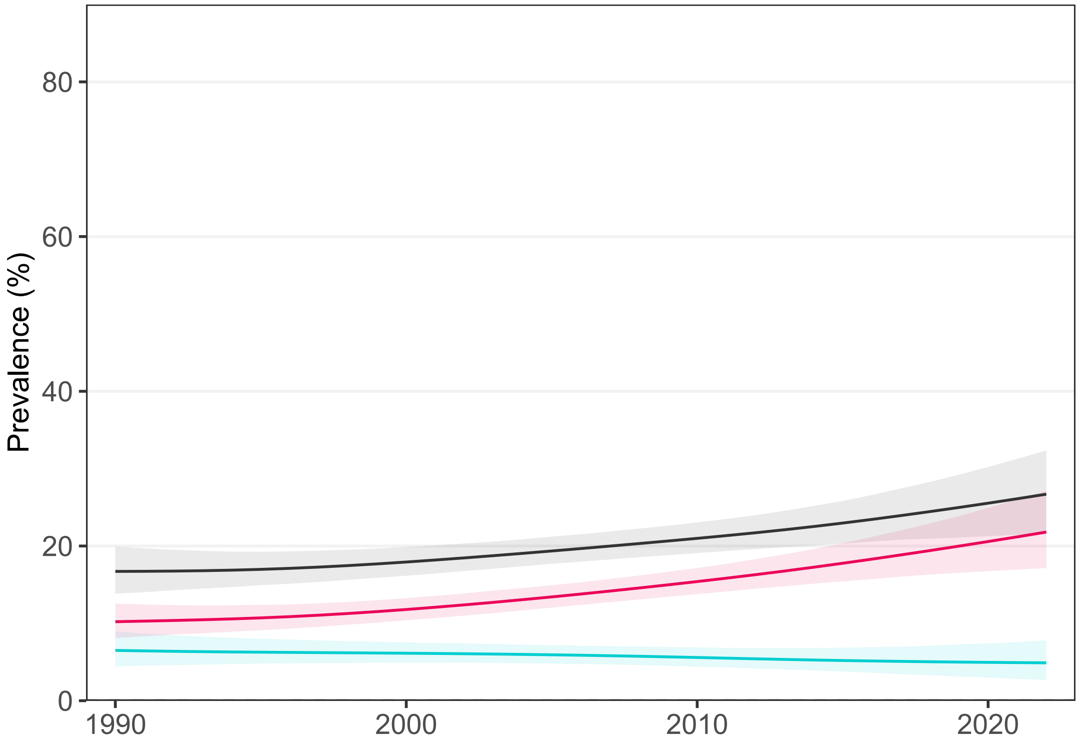


### Men
6 studies (3 national)

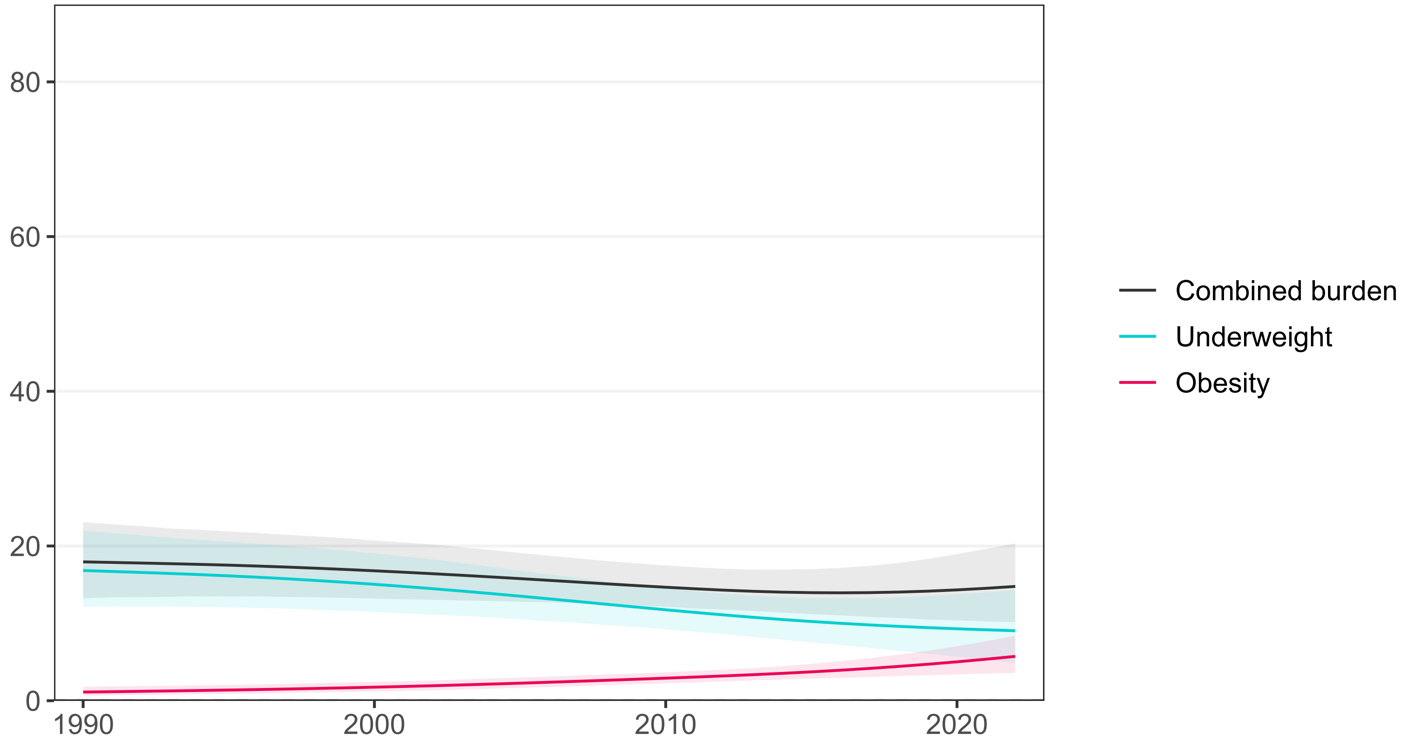

## References


1. NCD Risk Factor Collaboration (NCD-RisC). Worldwide trends in body-mass index, underweight, overweight, and obesity from 1975 to 2016: a pooled analysis of 2416 population-based measurement studies in 128.9 million children, adolescents, and adults. *Lancet* 2017; **390**(10113): 2627-42.
2. NCD Risk Factor Collaboration (NCD-RisC). Rising rural body-mass index is the main driver of the global obesity epidemic in adults. *Nature* 2019; **569**(7755): 260-4.
3. NCD Risk Factor Collaboration (NCD-RisC). Diminishing benefits of urban living for children and adolescents' growth and development. *Nature* 2023; **615**(7954): 874-83.
4. NCD Risk Factor Collaboration (NCD-RisC). Trends in adult body-mass index in 200 countries from 1975 to 2014: a pooled analysis of 1698 population-based measurement studies with 19.2 million participants. *Lancet* 2016; **387**(10026): 1377-96.
5. NCD Risk Factor Collaboration (NCD-RisC). Height and body-mass index trajectories of school-aged children and adolescents from 1985 to 2019 in 200 countries and territories: a pooled analysis of 2181 population-based studies with 65 million participants. *Lancet* 2020; **396**(10261): 1511-24.
6. Finucane MM, Stevens GA, Cowan MJ, et al. National, regional, and global trends in body-mass index since 1980: systematic analysis of health examination surveys and epidemiological studies with 960 country-years and 9.1 million participants. *Lancet* 2011; **377**(9765): 557-67.
7. Farzadfar F, Finucane MM, Danaei G, et al. National, regional, and global trends in serum total cholesterol since 1980: systematic analysis of health examination surveys and epidemiological studies with 321 country-years and 3.0 million participants. *Lancet* 2011; **377**(9765): 578-86.
8. Danaei G, Finucane MM, Lu Y, et al. National, regional, and global trends in fasting plasma glucose and diabetes prevalence since 1980: systematic analysis of health examination surveys and epidemiological studies with 370 country-years and 2.7 million participants. *Lancet* 2011; **378**(9785): 31-40.
9. Danaei G, Finucane MM, Lin JK, et al. National, regional, and global trends in systolic blood pressure since 1980: systematic analysis of health examination surveys and epidemiological studies with 786 country-years and 5.4 million participants. *Lancet* 2011; **377**(9765): 568-77.
10. Hayes AJ, Clarke PM, Lung TW. Change in bias in self-reported body mass index in Australia between 1995 and 2008 and the evaluation of correction equations. *Popul Health Metr* 2011; **9**(1): 1-9.
11. Ezzati M, Martin H, Skjold S, Hoorn SV, Murray CJ. Trends in national and state-level obesity in the USA after correction for self-report bias: analysis of health surveys. *J R Soc Med* 2006; **99**(5): 250-7.
12. Gorber SC, Tremblay M, Moher D, Gorber B. A comparison of direct vs. self-report measures for assessing height, weight and body mass index: a systematic review. *Obes Rev* 2007; **8**(4): 307-26.
13. Eurostat. Self-reported time elapsed since last visit to a medical professional by sex, age and degree of urbanisation. 2022. https://ec.europa.eu/eurostat/databrowser/view/hlth_ehis_am1u/ (accessed 17 November 2023).
14. de Onis M, Onyango AW, Borghi E, Siyam A, Nishida C, Siekmann J. Development of a WHO growth reference for school-aged children and adolescents. *Bull World Health Organ* 2007; **85**(9): 660-7.
15. Lumley T. Analysis of complex survey samples. *J Stat Softw* 2004; **9**(8): 1-19.
16. World Health Organization. Chronic non-communicable diseases risk factors survey in Iraq. 2006. https://www.who.int/publications/m/item/2006-steps-country-report-iraq (accessed 10 November 2023).

17. World Health Organization. Research report on NCD risk factors surveillance in Nepal. 2003. https://www.who.int/publications/m/item/2003-steps-country-report-nepal (accessed 10 November 2023).
18. Finucane MM, Paciorek CJ, Danaei G, Ezzati M. Bayesian estimation of population-level trends in measures of health status. *Stat Sci* 2014; **29**(1): 18-25.
19. Stevens GA, Bennett JE, Hennocq Q, et al. Trends and mortality effects of vitamin A deficiency in children in 138 low-income and middle-income countries between 1991 and 2013: a pooled analysis of population-based surveys. *Lancet Glob Health* 2015; **3**(9): e528-36.
20. NCD Risk Factor Collaboration (NCD-RisC). Worldwide trends in hypertension prevalence and progress in treatment and control from 1990 to 2019: a pooled analysis of 1201 population-representative studies with 104 million participants. *Lancet* 2021; **398**(10304): 957-80.
21. Gelman A. Prior distributions for variance parameters in hierarchical models (comment on article by Browne and Draper). *Bayesian Anal* 2006; **1**(3): 515-34.
22. Aitsi-Selmi A. Households with a stunted child and obese mother: trends and child feeding practices in a middle-income country, 1992-2008. *Matern Child Health J* 2015; **19**(6): 1284-91.
23. Breslow NE, Clayton DG. Approximate inference in generalized linear mixed models. *J Am Stat Assoc* 1993; **88**(421): 9-25.
24. Wood SN. Generalized Additive Models: An Introduction with R. Boca Raton, FL: Chapman & Hall/CRC; 2006.
25. Rue H, Held L. Gaussian Markov Random Fields: Theory and Applications. New York: CRC Press; 2005.
26. Harville DA. Matrix Algebra from a Statistician's Perspective. New York: Springer-Verlag; 2008.
27. Cameron N, Bogin B. Human Growth and Development. New York: Elsevier Science; 2012.
28. Watanabe S, Opper M. Asymptotic equivalence of Bayes cross validation and widely applicable information criterion in singular learning theory. *J Mach Learn Res* 2010; **11**(12): 3571-94.
29. Gelman A, Hwang J, Vehtari A. Understanding predictive information criteria for Bayesian models. *Stat Comput* 2014; **24**(6): 997-1016.
30. United Nations Department of Economic and Social Affairs, Population Division. World Urbanization Prospects: The 2018 Revision. New York: United Nations, 2019.
31. Smith AF, Roberts GO. Bayesian computation via the Gibbs sampler and related Markov chain Monte Carlo methods. *J R Stat Soc Series B Stat Methodol* 1993; **55**(1): 3-23.
32. The Stan Forums. Overdispersed initial values – general questions. 2018. https://discourse.mc-stan.org/t/overdispersed-initial-values-general-questions/3966 (accessed 1 June 2023).
33. Vehtari A, Gelman A, Simpson D, Carpenter B, Bürkner P-C. Rank-normalization, folding, and localization: an improved R for assessing convergence of MCMC (with discussion). *Bayesian Anal* 2021; **16**(2): 667-718.
34. Stan Development Team. RStan: the R interface to Stan. 2022. https://mc-stan.org/
35. Ahmad OB, Boschi-Pinto C, Lopez AD, Murray CJ, Lozano R, Inoue M. Age standardization of rates: a new WHO standard. GPE Discussion Paper Series 31. Geneva: World Health Organization, 2001.
36. Popkin BM, Corvalan C, Grummer-Strawn LM. Dynamics of the double burden of malnutrition and the changing nutrition reality. *Lancet* 2020; **395**(10217): 65-74.
37. Jaacks LM, Slining MM, Popkin BM. Recent underweight and overweight trends by rural-urban residence among women in low- and middle-income countries. *J Nutr* 2015; **145**(2): 352-7.
38. Jaacks LM, Slining MM, Popkin BM. Recent trends in the prevalence of under- and overweight among adolescent girls in low- and middle-income countries. *Pediatr Obes* 2015; **10**(6): 428-35.

39. Akombi BJ, Chitekwe S, Sahle BW, Renzaho AMN. Estimating the double burden of malnutrition among 595,975 children in 65 low- and middle-income countries: a meta-analysis of Demographic and Health Surveys. *Int J Environ Res Public Health* 2019; **16**(16): 2886.
40. Reyes Matos U, Mesenburg MA, Victora CG. Socioeconomic inequalities in the prevalence of underweight, overweight, and obesity among women aged 20-49 in low- and middle-income countries. *Int J Obes (Lond)* 2020; **44**(3): 609-16.
41. Hasan MM, Ahmed S, Soares Magalhaes RJ, Fatima Y, Biswas T, Mamun AA. Double burden of malnutrition among women of reproductive age in 55 low- and middle-income countries: progress achieved and opportunities for meeting the global target. *Eur J Clin Nutr* 2022; **76**(2): 277-87.
42. Seferidi P, Hone T, Duran AC, Bernabe-Ortiz A, Millett C. Global inequalities in the double burden of malnutrition and associations with globalisation: a multilevel analysis of Demographic and Health Surveys from 55 low-income and middle-income countries, 1992-2018. *Lancet Glob Health* 2022; **10**(4): e482-90.
43. Alem AZ, Yeshaw Y, Liyew AM, et al. Double burden of malnutrition and its associated factors among women in low and middle income countries: findings from 52 nationally representative data. *BMC Public Health* 2023; **23**(1): 1479.
44. Caleyachetty R, Thomas GN, Kengne AP, et al. The double burden of malnutrition among adolescents: analysis of data from the Global School-Based Student Health and Health Behavior in School-Aged Children surveys in 57 low- and middle-income countries. *Am J Clin Nutr* 2018; **108**(2): 414-24.
45. Wrottesley SV, Mates E, Brennan E, et al. Nutritional status of school-age children and adolescents in low- and middle-income countries across seven global regions: a synthesis of scoping reviews. *Public Health Nutr* 2023; **26**(1): 63-95.
46. Min J, Zhao Y, Slivka L, Wang Y. Double burden of diseases worldwide: coexistence of undernutrition and overnutrition-related non-communicable chronic diseases. *Obes Rev* 2018; **19**(1): 49-61.
47. Gutiérrez-Fisac JL, León-Muñoz LM, Regidor E, Banegas J, Rodríguez-Artalejo F. Trends in obesity and abdominal obesity in the older adult population of Spain (2000-2010). *Obes Facts* 2013; **6**(1): 1-8.
48. Jansana A, Pistillo A, Raventós B, Nurn E, Freisling H, Duarte Salles T. Impact of the COVID-19 pandemic on the incidence of adult overweight and obesity in a large population-based cohort study in Catalonia, Spain. *Obes Facts* 2023; **16**(Suppl. 1): 133.
49. Czernichow S, Renuy A, Rives-Lange C, et al. Evolution of the prevalence of obesity in the adult population in France, 2013–2016: the Constances study. *Sci Rep* 2021; **11**(1): 14152.
50. Gil J, Mora T. The determinants of misreporting weight and height: the role of social norms. *Econ Hum Biol* 2011; **9**(1): 78-91.
51. Fontbonne A, Currie A, Tounian P, et al. Prevalence of overweight and obesity in France: the 2020 Obepi-Roche Study by the “Ligue Contre l’Obesite”. *J Clin Med* 2023; **12**(3): 925.
52. NCD Risk Factor Collaboration (NCD-RisC). Repositioning of the global epicentre of non-optimal cholesterol. *Nature* 2020; **582**(7810): 73-7.
53. NCD Risk Factor Collaboration (NCD-RisC). Worldwide trends in blood pressure from 1975 to 2015: a pooled analysis of 1479 population-based measurement studies with 19.1 million participants. *Lancet* 2017; **389**(10064): 37-55.
54. NCD Risk Factor Collaboration (NCD-RisC). Worldwide trends in diabetes since 1980: a pooled analysis of 751 population-based studies with 4.4 million participants. *Lancet* 2016; **387**(10027): 1513-30.
